# Construction of Prognostic Risk Model of Patients with Skin Cutaneous Melanoma Based on TCGA-SKCM Methylation Cohort

**DOI:** 10.1155/2022/4261329

**Published:** 2022-08-25

**Authors:** Xiaoming Yu, Ping Cong, Wei Wei, Yong Zhou, Zhengqiang Bao, Huaying Hou

**Affiliations:** Department of Cancer Center, The Second Hospital of Shandong University, Jinan, Shandong 250033, China

## Abstract

Skin cutaneous melanoma (SKCM) is a common malignant skin cancer. Early diagnosis could effectively reduce SKCM patient's mortality to a large extent. We managed to construct a model to examine the prognosis of SKCM patients. The methylation-related data and clinical data of The Cancer Gene Atlas- (TCGA-) SKCM were downloaded from TCGA database. After preprocessing the methylation data, 21,861 prognosis-related methylated sites potentially associated with prognosis were obtained using the univariate Cox regression analysis and multivariate Cox regression analysis. Afterward, unsupervised clustering was used to divide the patients into 4 clusters, and weighted correlation network analysis (WGCNA) was applied to construct coexpression modules. By overlapping the CpG sites between the clusters and turquoise model, a prognostic model was established by LASSO Cox regression and multivariate Cox regression. It was found that 9 methylated sites included cg01447831, cg14845689, cg20895058, cg06506470, cg09558315, cg06373660, cg17737409, cg21577036, and cg22337438. After constructing the prognostic model, the performance of the model was validated by survival analysis and receiver operating characteristic (ROC) curve, and the independence of the model was verified by univariate and multivariate regression. It was represented that the prognostic model was reliable, and riskscore could be used as an independent prognostic factor in SKCM patients. At last, we combined clinical data and patient's riskscore to establish and testify the nomogram that could determine patient's prognosis. The results found that the reliability of the nomogram was relatively good. All in all, we constructed a prognostic model that could determine the prognosis of SKCM patients and screened 9 key methylated sites through analyzing data in TCGA-SKCM dataset. Finally, a prognostic nomogram was established combined with clinical diagnosed information and riskscore. The results are significant for improving the prognosis of SKCM patients in the future.

## 1. Introduction

Skin cutaneous melanoma (SKCM) is a malignant skin cancer formed by the canceration of melanocytes below the epidermis. Pathogenic factors for SKCM are mainly classified into external and internal factors [1]. The most common external pathogenic factor for SKCM is ultraviolet radiation, and studies found that when the underlying cells of the skin are exposed to ultraviolet radiation, DNA damage can be caused to further induce SKCM [2–5]. Moreover, environmental factors are also important external factors for SKCM. For example, di-(2-ethylhexyl) phthalate (DEHP) in cosmetics and PM2.5 in the air can increase the incidence rate of SKCM [6, 7]. Besides common environmental carcinogenic factors, genetic and epigenetic modifications are also common carcinogenic factors, and studies considered that people from families with CDKN2A gene mutation have higher incidence rate of melanoma [8, 9]. Telomerase reverse transcriptase (TERT) can also increase the risk of SKCM [10]. In addition, epigenetic modification is a crucial factor for melanoma. Studies disclosed that methylation of genes like APC, PYCARD, and COL11A1 is relevant to the incidence of melanoma, and histone H3K27me3 upregulation can promote melanoma progression [11, 12]. Additionally, current studies uncovered that immunosuppression and pigment characteristics are risk factors for melanoma [13–15].

DNA methylation is one of the most important epigenetic modifications. A considerable number of studies considered that DNA methylation modification is related to the incidence of melanoma. For example, de Unamuno Bustos et al. [16] found that aberrant methylation of genes like RARB and PTEN is associated with clinical melanoma progression. DNA methylation is correlated with the metastasis and drug resistance of melanoma as well. For example, Venza et al. [17] discovered that DNA methylation can promote melanoma metastasis by silencing E-cadherin. MGMT gene promoter methylation can promote the tolerance of temozolomide in melanoma [18]. In conclusion, DNA methylation can be widely involved in the occurrence and progression of melanoma. We believed that it had a good potential value to diagnose the prognosis of SKCM patients by detecting DNA methylation level.

The Cancer Genome Atlas (TCGA) is a database commonly applied in tumor biomarker screening, which includes abundant clinical experimental data of tumor patients, wherein it contains SKCM methylation data of 470 cases that can be used for analyzation and have significant clinical value [19]. Presently, massive studies have applied TCGA database for screening of tumor biomarkers. For example, Zhu et al. [20] obtain methylation sites related to prognosis of lung cancer via digging methylation data of lung cancer patients in TCGA. Olkhov-Mitsel et al. [21] acquired methylation biomarkers that can identify bladder cancer stage differences through digging the methylation data of patients with bladder cancer. In this study, the methylation data as well as clinical data in TCGA-SKCM database were used to screen methylation sites that resulted in poor prognosis of SKCM patients.

In this study, TCGA database was applied to screen prognosis-related methylation sites via univariate regression analysis, multivariate regression analysis, the weighted correlation network analysis (WGCNA), and unsupervised clustering analysis. After constructing a risk model by LASSO and multivariate Cox regression, we identified the independence and accuracy of the model. Lastly, a prognostic nomogram was constructed combined with relevant clinical data. The nomogram has great guiding significance for the clinical diagnosis and treatment of SKCM.

## 2. Materials and Methods

### 2.1. Data Processing

Methylation sequencing data and corresponding clinical information in TCGA-SKCM dataset from TCGA database (https://portal.gdc.cancer.gov/) were included in this study. Clinical data like patient's age, gender, and tumor stage in TCGA-SKCM dataset were first downloaded from TCGA website (Table S1). Afterward, DNA methylation (450K) data were accessed from TCGA-SKCM dataset on 20 July 2020, including DNA methylation sequencing results of 2 healthy tissue samples and 473 tumor tissue samples.

Downloaded DNA methylation data were filtrated according to the following criteria: (1) removing methylation sites that missed more than 70% of data, (2) filtrating all non-GpG methylation sites, (3) filtrating all SNP-related methylation sites, (4) filtrating all methylation sites that mapped to multiple locations, and (5) filtrating all methylation sites in the X and Y chromosomes. Afterward, 361,126 methylation sites were obtained. R package “KNN” [22] was applied to complete the missing values in the methylation expression profile, and R package “ChAMP” [23] was used to standardize the data. After obtaining standardized methylation data, tumor tissue samples with the follow-up time more than 0 d were selected (*n* = 455). The samples were randomly divided into the training set (*n* = 318) and validation set (*n* = 137) in a proportion of 7 : 3.

### 2.2. Preliminary Screening of Methylation Sites Related to the Prognosis

Data in the training set were analyzed by univariate regression to calculate methylation sites that were significantly correlated with patient's overall survival (OS) (*p* < 0.05). Thereafter, relevant clinicopathological data were combined to perform multivariate regression analysis on screened prognosis-related methylation sites, thereby screening methylation sites correlated with patient's OS (*p* < 0.05). All analyses were finished with R package “survival” [24].

### 2.3. Unsupervised Clustering Analysis

According to the methylation level of methylation sites related to patient's prognosis, unsupervised clustering analysis was performed on patients with R package “ConsensusClusterPlus” [25]. The “pam” method was selected for clustering, and the “Euclidean” method was used to calculate the sample distance. The optimal cluster number was assessed by the cumulative distribution function (CDF) and its area under curve (AUC). After clustering, OS of patients with different disease subtypes was analyzed with R package “survival” [24].

### 2.4. WGCNA

WGCNA was performed on data in the training set with the R package “WGCNA” [26]. Firstly, we evaluated prognosis-related methylation sites and screened methylation sites with the variance ranked in the top 5000 to construct a coexpression network. Thereafter, the Pearson correlation index was used to establish adjacent matrix with the soft threshold *β* = 8. Afterward, the adjacent matrix was transferred into the topological matrix (TOM). Based on TOM, average-linkage hierarchical clustering was applied to cluster methylation sites. Lastly, a dynamic tree cut algorithm was exerted to identify coexpression modules with the size of minimum module of 30.

After clustering coexpression modules, module subtype (MS) correlation was used to identify the correlation between coexpression modules and patients' subtypes. Following this, methylation sites correlated with patients' subtypes were screened by GpG-site significance (CS) and module membership (MM). CS represented the correlation between the methylation level of methylation sites and patients' subtypes, and MM represented the correlation between methylation level of methylation sites corresponding to the patients' subtypes and module eigenvalue. Methylation sites (CS > 0.6 and MM > 0.8) were screened to establish a prognostic risk model.

### 2.5. Construction and Validation of Methylation-Related Prognostic Model

After acquiring key methylation sites, LASSO Cox regression analysis was performed on data in the training set with R package “glmnet” [27] to reduce the complexity of the module and screen key methylation sites with prognostic value. Afterward, multivariate regression analysis was undertaken with R package “survminer” [28] on key methylation sites screened by LASSO Cox. Finally, a risk model was established. Riskscore in the risk model was calculated by the formula as follows:

(1) $$\begin{array}{c} \text{Riskscore}=\overset{n}{\underset{i=1}{\sum }}\left({\text{Coef}}_{i}\times x_{i}\right). \end{array}$$

In the formula, Coef_*i*_ represents the risk index of each methylation site, *x*_*i*_ represents methylation level of each methylation site, and Riskscore represents the ultimate riskscore. After screening prognosis-related methylation sites, related information of methylation sites was searched according to Ensembl database (version GRCh38.p13) (http://asia.ensembl.org).

Patients were divided into two groups according to the median of the score based on the formula: the high-risk group and the low-risk group. Survival analysis was undertaken on two groups with R package “survival,” and the accuracy of the model was identified by receiver operating characteristic (ROC) curve. Finally, the score distribution map, survival status distribution map, and methylation level heatmap of samples were drawn.

### 2.6. Assessment of Clinical Characteristics and Prognostic Independence of Riskscore

Univariate regression and multivariate regression analyses were performed on riskscore combined with clinical information including age, gender, pathological_T stage, pathological_N stage, pathological M stage, and tumor stage to analyze the correlation between the indexes and patient's OS, respectively. The indexes that were significantly correlated with patient's OS both in the results of univariate and multivariate regression analyses were considered to have the independent prognostic value.

### 2.7. Construction of a Prognostic Nomogram

Combined with clinically related indexes and riskscore, a nomogram that could predict 1-, 3-, and 5-year survival rates of patients was established with R package “rms” [29]. Correction curves of 1, 3, and 5 years were generated with R package “foreign” [30] after establishing the nomogram to identify the predictive effect of the nomogram.

## 3. Results

### 3.1. Screening of Prognosis-Related Methylation Sites

**Figure 1:**
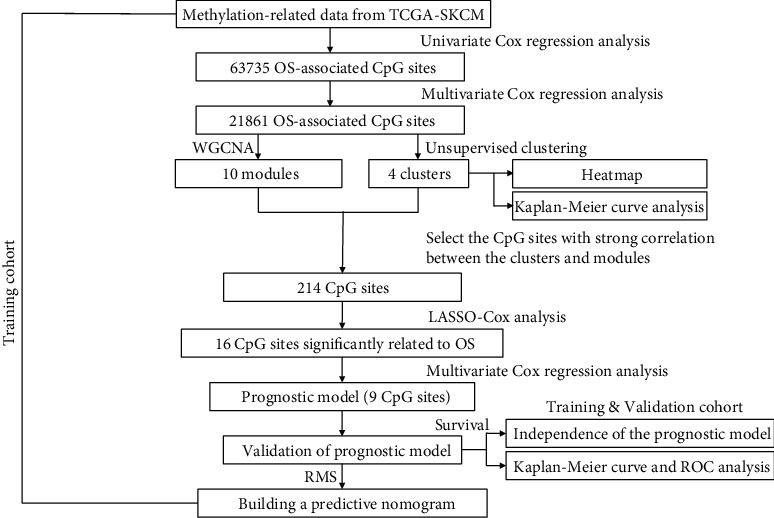
Overall flowchart of this study.

The flow chart of this study is shown in Figure 1. After downloading and preprocessing methylation-related data, we screened 63,735 prognosis-related methylation sites by univariate regression analysis and then followed by multivariate regression analysis obtaining 21,861 methylation sites which were remarkably correlated with SKCM patients' OS (Table S2).

### 3.2. Four Groups of Patients with Different Subtypes Found by Unsupervised Clustering Analysis

**Figure 2:**
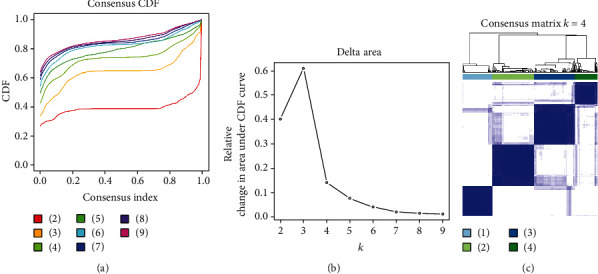
Unsupervised clustering of SKCM patients. (a) Cumulative distribution function curve of unsupervised clustering. (b) Relative change in area under cumulative distribution function (CDF) curve. (c) Clustering heatmap of 4 types of SKCM patients. Each cluster represents a subgroup of patients.

**Figure 3:**
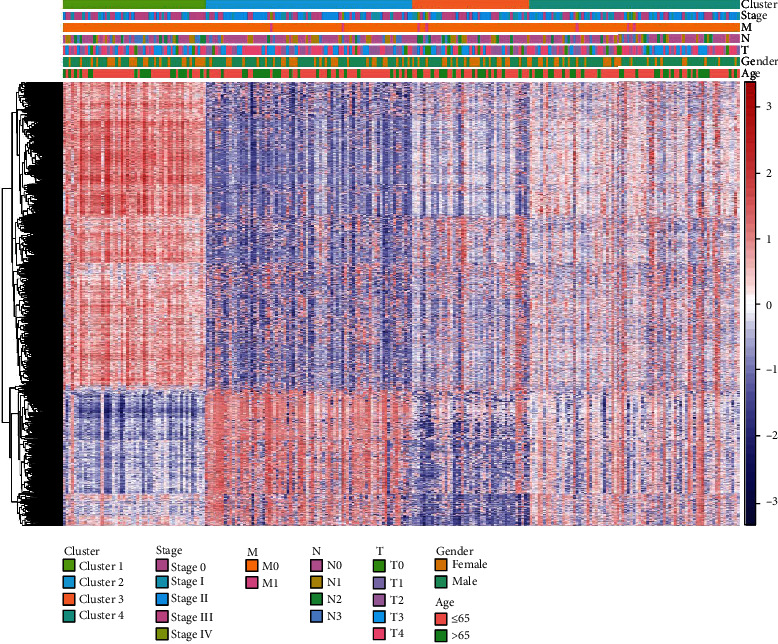
Heatmap of top 5000 variance CpG sites, with red representing high level of DNA methylation and blue representing low level of DNA methylation.

**Figure 4:**
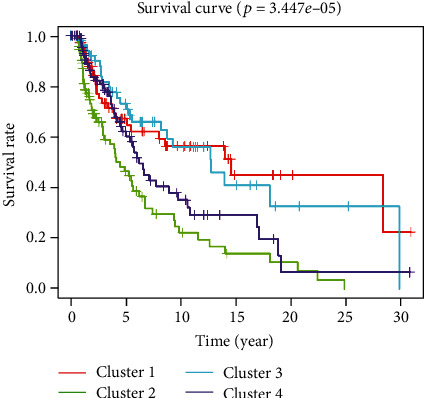
Survival curve of SKCM patients' OS, with each line representing a subgroup of SKCM patients.

After screening methylation sites that were significantly correlated with SKCM patient's OS, we clustered patients by unsupervised clustering method (Figures 2(a) and 2(b)). Clustering results showed that patients were mainly divided into 4 subtypes: cluster 1, cluster 2, cluster 3, and cluster 4 (Figure 2(c)). Heatmap of 5,000 methylation sites with the highest variance was drawn combined with patient's clinical information. The result represented that the methylation levels differed in patients with 4 subtypes (Figure 3). Finally, survival analysis was performed on patients in the 4 groups. It was shown that OS of patients with 4 different subtypes had differences; the prognosis of cluster 2 patients was the poorest, and the prognosis of cluster 1 patients was the best (Figure 4). The above results exhibited that the unsupervised clustering analysis could reliably cluster the patients into 4 subtypes, and there were differences in methylation level and OS of patients with different subtypes had differences.

### 3.3. WGCNA

**Figure 5:**
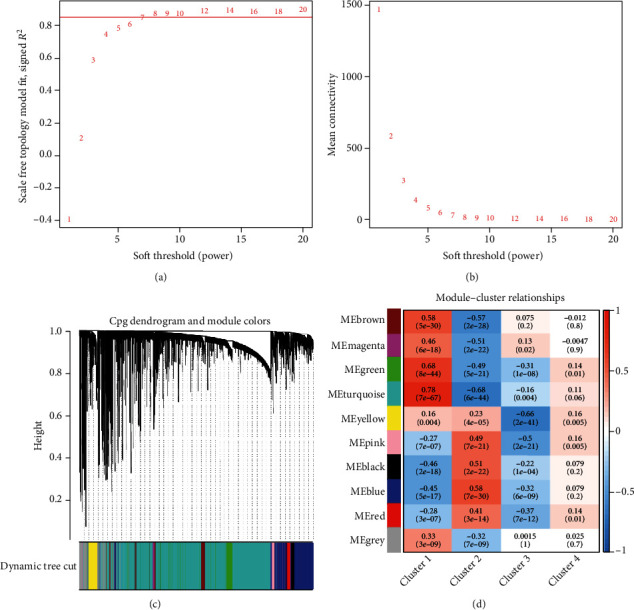
WGCNA analysis of the top 5000 variance CpG sites. (a) Analysis of the scale-free fit index for various soft-thresholding powers (*β*). (b) Analysis of the mean connectivity for various soft-thresholding powers. (c) Dendrogram of prognosis-related CpG sites clustered based on a dissimilarity measure (1-TOM). (d) Heatmap of the correlation between module and subgroups of patients.

**Figure 6:**
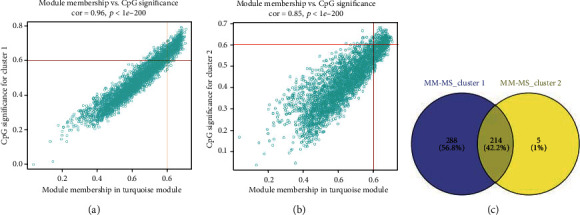
Filtering of OS-associated CpG sites. (a) Scatter plot of turquoise module member CpG sites related to cluster 1. Each dot represents a CpG site. (b) Scatter plot of turquoise module member CpG sites related to cluster 2. (c) Venn plot of important CpG sites. The blue circle represents cluster 1-associated CpG sites, and the yellow circle represents cluster 2-associated CpG sites.

After grouping patients and screening 5,000 methylation sites with highest variance, we further performed WGCNA on the methylation sites (Figures 5(a) and 5(b)) and ultimately obtained 10 different modules (Figure 5(c)). Correlation analysis was undertaken on the 10 methylation modules and 4 different patient's subtypes. Then, modules related to patient's subtypes were screened by MS. The results showed that patients with cluster 1 subtype and cluster 2 subtype were significantly correlated with most modules and represented opposite trends (Figure 5(d)). The further analysis discovered that the turquoise module had the highest correlation with cluster 1 and cluster 2. Hence, we chose the turquoise module for further analysis. Afterward, correlation analysis was performed on the 3,031 methylation sites in the turquoise module and cluster 1/cluster 2, respectively, to screen methylation sites. A total of 502 methylation sites both related to the turquoise module and cluster 1 were obtained, and 219 methylation sites both related to the turquoise module and cluster 2 were obtained (Figures 6(a) and 6(b)). At last, to further screen for methylation sites that are prominently associated with SKCM patients, Venn plot was used to intersect the sites and 214 key methylation sites were obtained (Figure 6(c)).

### 3.4. Construction and Validation of Prognosis-Related Methylation Model

**Figure 7:**
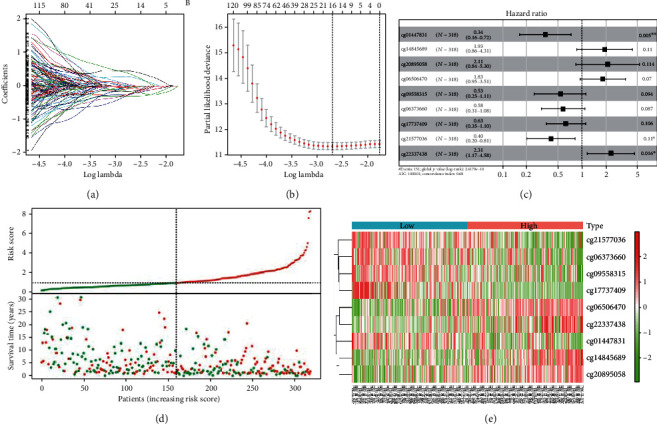
Construction of SKCM risk model. (a) LASSO coefficient profiles of key CpG sites. (b) Selection of the optimal parameter (lambda) in the LASSO model for TCGA-LUAD. (c) Key CpG sites filtered by multivariate Cox regression analysis. (d) Riskscore and survival status of patients in training cohort. (e) Heatmap of each CpG site in risk model.

To avoid module overfitting, we screened key methylation sites with LASSO Cox regression and obtained 16 key methylation sites (Figures 7(a) and 7(b)). Multivariate regression analysis was performed on the 16 key methylation sites to construct multivariate regression model, and finally, 9 prognosis-related methylation sites (cg01447831, cg14845689, cg20895058, cg06506470, cg09558315, cg06373660, cg17737409, cg21577036, and cg22337438) were screened in SKCM (Figure 7(c) and Table 1). Meanwhile, risk model was obtained: Riskscore = −1.0719∗cg01447831 + 0.6563∗cg14845689 + 0.7445∗cg20895058 + 0.6031∗cg06506470 − 0.6421∗cg09558315 − 0.5512∗cg06373660 − 0.4686∗cg17737409 − 0.9057∗cg21577036 + 0.8385∗cg22337438.

**Figure 8:**
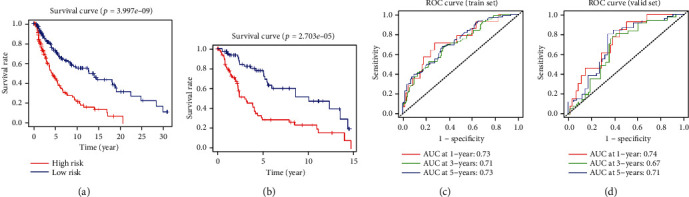
Validation of SKCM risk model. (a and b) Survival curve analysis of training cohort and validation cohort. (c and d) ROC curve analysis of training cohort and validation cohort.

After establishing the risk model, we detected the distribution and survival status of high- and low-risk patients in the training set with the score distribution map and survival status distribution map (Figure 7(d)). The result showed that patients with high risk commonly were more likely to die and the survival time of high-risk patients was relatively lower. Changes of the methylation levels of 9 methylation sites of high- and low-risk patients were analyzed with heatmap. The result was consistent with the risk index of model (Figure 7(e)). To identify the reliability of risk model, survival analysis was performed to compare the OS differences between high- and low-risk patients in the training set and the validation set. It was represented that OS in the low-risk group was significantly higher than that in the high-risk group (Figures 8(a) and 8(b)). ROC curve was further used to analyze the 1-, 3-, and 5-year survival of patients in the training set and the validation set. It was exhibited that AUC of ROC curve was 0.73, 0.71, and 0.73 in the training set, respectively, and AUC of ROC curve was 0.74, 0.67, and 0.71 in the validation set, respectively, indicating that the model was reliable (Figures 8(c) and 8(d)). The results showed that the risk model could accurately determine 1-, 3-, and 5-year survival of patients. The above findings represented that the results of the risk model were accurate and the model could be used for predicting the prognosis of SKCM patients.

### 3.5. Identifying Model's Independence and Establishing Prognostic Nomogram

**Figure 9:**
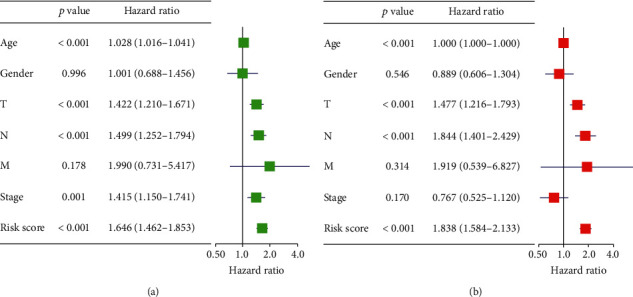
Univariate and multivariate validation of risk model's independence. (a) Univariate analysis validates independence of SKCM risk model. (b) Multivariate analysis validates independence of SKCM risk model.

**Figure 10:**
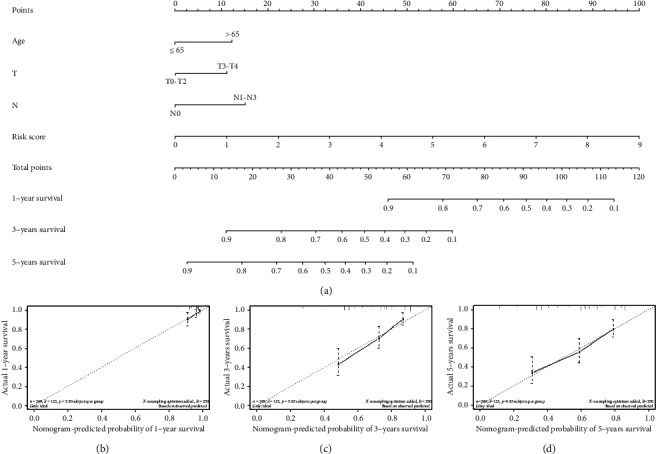
Construction and validation of prognostic nomogram. (a) Nomogram to predict 1-, 3-, and 5-year survival rate of SKCM patients. (b–d) Fitting curves used to validate prognostic nomogram.

After establishing the risk model and validating accuracy of the model, univariate regression and multivariate regression analyses were applied combining with clinical data (age, gender, T, N, M, and tumor stage) and riskscore to validate whether riskscore could independently determine patient's prognosis. It was shown that riskscore in the risk model was significantly correlated with patient's OS and could independently determine patient's prognosis in univariate and multivariate regression analyses (Figures 9(a) and 9(b)). After validating the independence of the risk model, we combined clinically related data to establish a nomogram that could be used to determine 1-, 3-, and 5-year survival rate of patients (Figure 10(a)). Afterward, fitting curve was used to validate the accuracy of the nomogram. The result showed that fitting results of 1-,3-, and 5-year were good (Figures 10(b)–10(d)). The nomogram can assist clinical doctors to diagnose patient's prognostic risk and help doctors to arrange therapeutic plans more accurately.

## 4. Discussion

Melanoma is a common modern disease, and SKCM is a melanoma that occurs in the epidermis. Despite the high incidence rate of SKCM, its mortality can be relatively low if it is diagnosed in time in the early stage. We screened 214 methylation (CpG) sites remarkably associated with OS in SKCM patients from the TCGA-SKCM dataset by unsupervised clustering and WGCNA and finally constructed a prognostic model of 9 signature CpG sites using LASSO Cox regression analysis and demonstrated that the model had a better prognostic effect.

With the development of high-throughput sequencing technology, high-throughput sequencing on tumor patients has become an important method for tumor research, from which biomarkers with prognostic values are filtered [31]. Currently, the mainstream research method is evaluating patient's risk of cancer by analyzing mRNA expression data and combining with the expression of many genes [32]. The advantage of the method is that the combination of the expression of multiple genes to assess patient's prognosis is more accurate than using each gene alone. However, it is still not comprehensive enough for the determination of patient's prognosis. Presently, researchers managed to increase the accuracy for determining patient's prognosis by screening biomarkers via analyzing miRNAs and lncRNAs, and even patient's metabolic data [33]. Besides predicting patient's prognosis based on RNA expression data, a study tried to predict patient's prognosis by the methylation level of sites through digging relevant data of gene methylation [34], which can obtain more stable prediction result and requires lower sample preserve conditions compared with using RNA expression level to predict prognosis. Hence, methylation sites that affected SKCM patient's prognosis were explored in this study by digging DNA methylation relevant data in TCGA-SKCM dataset combined with patient's clinical survival time. A total of 21,861 prognosis-related methylation sites were found through univariate and multivariate regression analyses.

Mining TCGA database by bioinformatics methods is a common study method, in which unsupervised cluster is an effective means to classify patients with cancer in the present [34–36]. For example, Wu et al. [37] found 3 subtypes with different molecular characters in lung adenocarcinoma by unsupervised clustering, and patients with each subtype are relevant to abnormal specific molecular pathways. Patients were divided into 4 groups in this study by unsupervised clustering. The OS of patients in different groups was different, wherein cluster 2 patients had the poorest prognosis, while cluster 1 patients had the best prognosis. WGCNA is also a common study method in biomarker screening, and a number of studies screened biomarkers with value for patient's prognosis by WGCNA [34]. Ten methylation modules related to patient's prognosis were obtained in this study by WCGNA. At last, 9 methylation sites and risk model relevant to OS were further screened combined with the results of WCGNA and unsupervised clustering.

The 9 prognosis-related methylation sites were screened in this study based on the previous research. Among the 9 sites, high methylation of cg01447831, cg09558315, cg06373660, cg17737409, and cg21577036 can reduce prognostic risk, while high methylation of cg14845689, cg20895058, cg06506470, and cg22337437 can increase prognostic risk. After searching regulatory genes corresponding to these methylation sites, we found that these sites were located in EN2, EVC, AL590807.1, GALNT2, AC091167.6, EMX2, AL049874.3, SDK1, and NLRP12 genes, respectively. EN2 as a transcription factor was found to be a biomarker for prostate cancer and breast cancer in relevant studies [38, 39]. Current research considered that highly expressed EN2 can promote the proliferation and drug resistance of cancer cells [40, 41]. In this study, it was found that cg01447831 high methylation could reduce the prognostic risk of patients, which may be because cg01447831 can inhibit the expression of EN2. It was discovered that methylation site cg09558315 was in the driver zone of EVC, which indicated that EVC high expression may promote the occurrence of cancers. At present, few studies were undertaken on the carcinogenic mechanism of EVC, and studies considered that EVC mutation may be an inducement for Ellis-van Creveld syndrome and EVC may be a prognostic marker of colon cancer in the early stage [42, 43]. GALNT2 gene is a Mucin O-glycosylase which is disputed for its effect in cancers. A study thought that GALNT2 may promote cancer progression via activating EGFR/PI3K/Akt/mTOR pathway [44]. Furthermore, it was also considered that GALNT2 can inhibit cancer development by reducing MET phosphorylation in some conditions [45], and it was found that cg17737409 site methylation in GLANT2 was helpful for patient's prognosis in this study. Moreover, we found that high methylation silencing of cg14845689 in EXM2 could increase patient's prognostic risk. EXM2 is thought as a tumor suppressor gene, and high expression of EXM2 can induce cell cycle stagnation thereby inhibiting the proliferation of cancer cells [46, 47]. SDK1 is a kind of immune-related protein, and studies found that SDK1 mutation causes cancers, but the effect of SDK1-related site methylation on the incidence of cancer has not been fully studied [48, 49]. Our study found that high methylation of cg06506470 site in SDK1 gene could cause the poor prognosis of patients. NLRP12 is an immune-sensor element that triggers an inflammatory response, causing the release of IL-1B and IL-18, and the cleavage and activation of Caspase-1 [50]. After screening methylation sites, we identified prognostic model and established a prognostic nomogram that could assist clinical doctors to determine patient's prognosis.

All in all, we analyzed related data in TCGA-SKCM dataset, screened 9 prognosis-related methylation sites, and established a prognostic model by univariate, multivariate, and LASSO Cox regression analyses, unsupervised clustering analysis, and WGCNA. The model was accurate for determining patient's prognosis. The model screened by experiments is reliable validated by data in the validation set, but this paper is a bioinformatics essay solely without experimental data support. Therefore, more animal and clinical experiments are needed to support the conclusion in this paper to be clinically used.

## Figures and Tables

**Table 1:** Detailed information of filtered CpG sites.

CpG site	Chrom	Position	Gene symbol
cg01447831	chr7	155,456,390-155,456,439	EN2
cg14845689	chr10	117,546,682-117,546,731	EMX2
cg20895058	chr14	60,516,569-60,516,618	AL049874.3
cg06506470	chr7	3,990,973-3,991,022	SDK1
cg09558315	chr4	5,709,574-5,709,623	EVC
cg06373660	chr13	80,840,851-80,840,900	AL590807.1
cg17737409	chr1	230,114,215-230,114,264	GALNT2
cg21577036	chr15	90,301,567-90,301,616	AC091167.6
cg22337438	chr19	53,824,135-53,824,184	NLRP12

## Data Availability

All data generated or analyzed during this study are included in this article.

## Supplementary Materials

Supplementary Materials Table S1: the clinical data was accessed from TCGA-SKCM dataset. Table S2: the result of multivariate Cox regression for the methylation sites based on SKCM patients.

## References

[1] Ward W. H., Lambreton F., Goel N., Yu J. Q., Farma J. M., Ward W. H., Farma J. M. (2017). *Cutaneous Melanoma: Etiology and Therapy*.

[2] Craig S., Earnshaw C. H., Viros A. (2018). Ultraviolet light and melanoma. *The Journal of Pathology*.

[3] You Y. H., Lee D. H., Yoon J. H., Nakajima S., Yasui A., Pfeifer G. P. (2001). Cyclobutane pyrimidine dimers are responsible for the vast majority of mutations induced by UVB irradiation in mammalian cells. *The Journal of Biological Chemistry*.

[4] Budden T., Bowden N. A. (2013). The role of altered nucleotide excision repair and UVB-induced DNA damage in melanomagenesis. *International Journal of Molecular Sciences*.

[5] Greinert R., Volkmer B., Henning S. (2012). UVA-induced DNA double-strand breaks result from the repair of clustered oxidative DNA damages. *Nucleic Acids Research*.

[6] Lee J. W., Park S., Han H. K., Gye M. C., Moon E. Y. (2018). Di-(2-ethylhexyl) phthalate enhances melanoma tumor growth via differential effect on M1-and M2-polarized macrophages in mouse model. *Environmental Pollution*.

[7] Kiss A., Wei C., Aligabi Z. (2020). 693 p38 signaling regulates human cutaneous metastatic melanoma (MM) invasion and MM-dependent disruption of keratinocyte differentiation. *Journal of Investigative Dermatology*.

[8] Begg C. B., Orlow I., Hummer A. J. (2005). Lifetime risk of melanoma in CDKN2A mutation carriers in a population-based sample. *Journal of the National Cancer Institute*.

[9] Bishop D. T., Demenais F., Goldstein A. M. (2002). Geographical variation in the penetrance of CDKN2A mutations for melanoma. *Journal of the National Cancer Institute*.

[10] Thomas N. E., Edmiston S. N., Tsai Y. S. (2019). Utility of TERT promoter mutations for cutaneous primary melanoma diagnosis. *The American Journal of Dermatopathology*.

[11] Sarkar D., Leung E. Y., Baguley B. C., Finlay G. J., Askarian-Amiri M. E. (2015). Epigenetic regulation in human melanoma: past and future. *Epigenetics*.

[12] Hoffmann F., Niebel D., Aymans P., Ferring-Schmitt S., Dietrich D., Landsberg J. (2020). H3K27me3 and EZH2 expression in melanoma: relevance for melanoma progression and response to immune checkpoint blockade. *Clinical Epigenetics*.

[13] Fredricks J. R., Bejarano P. A. (2008). Primary malignant melanoma of the esophagus with separate foci of melanoma in situ and atypical melanocytic hyperplasia in a patient positive for human immunodeficiency virus: a case report and review of the literature. *Archives of Pathology & Laboratory Medicine*.

[14] Nguyen J., Alexander T., Jiang H. (2018). Melanoma in patients with GATA2 deficiency. *Pigment Cell & Melanoma Research*.

[15] Olsen C. M., Carroll H. J., Whiteman D. C. (2010). Estimating the attributable fraction for melanoma: a meta-analysis of pigmentary characteristics and freckling. *International Journal of Cancer*.

[16] de Unamuno Bustos B., Murria Estal R., Pérez Simó G. (2018). Aberrant DNA methylation is associated with aggressive clinicopathological features and poor survival in cutaneous melanoma. *The British Journal of Dermatology*.

[17] Venza M., Visalli M., Catalano T. (2016). DNA methylation-induced E-cadherin silencing is correlated with the clinicopathological features of melanoma. *Oncology Reports*.

[18] Hassel J. C., Sucker A., Edler L. (2010). MGMT gene promoter methylation correlates with tolerance of temozolomide treatment in melanoma but not with clinical outcome. *British Journal of Cancer*.

[19] Tomczak K., Czerwinska P., Wiznerowicz M. (2015). The Cancer Genome Atlas (TCGA): an immeasurable source of knowledge. *Contemporary Oncology*.

[20] Zhu X. F., Zhu B. S., Wu F. M., Hu H. B. (2018). DNA methylation biomarkers for the occurrence of lung adenocarcinoma from TCGA data mining. *Journal of Cellular Physiology*.

[21] Olkhov-Mitsel E., Savio A. J., Kron K. J. (2017). Epigenome-wide DNA methylation profiling identifies differential methylation biomarkers in high-grade bladder cancer. *Translational Oncology*.

[22] Kuhn M. (2008). Building predictive models in R using the caret package. *Journal of Statistical Software*.

[23] Tian Y., Morris T. J., Webster A. P. (2017). ChAMP: updated methylation analysis pipeline for Illumina BeadChips. *Bioinformatics*.

[24] Zhou R. S., Zhang E. X., Sun Q. F. (2019). Integrated analysis of lncRNA-miRNA-mRNA ceRNA network in squamous cell carcinoma of tongue. *BMC Cancer*.

[25] Zheng J., Zhang T., Guo W. (2020). Integrative analysis of multi-omics identified the prognostic biomarkers in acute myelogenous leukemia. *Frontiers in Oncology*.

[26] Liang W., Sun F., Zhao Y., Shan L., Lou H. (2020). Identification of susceptibility modules and genes for cardiovascular disease in diabetic patients using WGCNA analysis. *Journal Diabetes Research*.

[27] Simon N., Friedman J., Hastie T., Tibshirani R. (2011). Regularization paths for Cox's proportional hazards model via coordinate descent. *Journal of Statistical Software*.

[28] Li S., Chen S., Wang B., Zhang L., Su Y., Zhang X. (2020). A robust 6-lncRNA prognostic signature for predicting the prognosis of patients with colorectal cancer metastasis. *Frontiers in Medicine*.

[29] Huang C., Liu Z., Xiao L. (2019). Clinical significance of serum CA125, CA19-9, CA72-4, and fibrinogen-to-lymphocyte ratio in gastric cancer with peritoneal dissemination. *Frontiers in Oncology*.

[30] Jia Z., Yan Y., Wang J. (2021). Development and validation of prognostic nomogram in ependymoma: a retrospective analysis of the SEER database. *Cancer Medicine*.

[31] Gagan J., Van Allen E. M. (2015). Next-generation sequencing to guide cancer therapy. *Genome Medicine*.

[32] Tang X. R., Li Y. Q., Liang S. B. (2018). Development and validation of a gene expression-based signature to predict distant metastasis in locoregionally advanced nasopharyngeal carcinoma: a retrospective, multicentre, cohort study. *The Lancet Oncology*.

[33] Müller S., Raulefs S., Bruns P. (2015). Next-generation sequencing reveals novel differentially regulated mRNAs, lncRNAs, miRNAs, sdRNAs and a piRNA in pancreatic cancer. *Molecular Cancer*.

[34] Wang Y., Wang Y., Wang Y., Zhang Y. (2020). Identification of prognostic signature of non-small cell lung cancer based on TCGA methylation data. *Scientific Reports*.

[35] Patel S. H., Poisson L. M., Brat D. J. (2017). T2-FLAIR mismatch, an imaging biomarker for IDH and 1p/19q status in lower-grade gliomas: a TCGA/TCIA project. *Clinical Cancer Research*.

[36] Danaher P., Warren S., Lu R. (2018). Pan-cancer adaptive immune resistance as defined by the tumor inflammation signature (TIS): results from The Cancer Genome Atlas (TCGA). *Journal for Immunotherapy of Cancer*.

[37] Wu J., Cui Y., Sun X. (2017). Unsupervised clustering of quantitative image phenotypes reveals breast cancer subtypes with distinct prognoses and molecular pathways. *Clinical Cancer Research*.

[38] Morgan R., Boxall A., Bhatt A. (2011). Engrailed-2 (EN2): a tumor specific urinary biomarker for the early diagnosis of prostate cancer. *Clinical Cancer Research*.

[39] Martin N. L., Saba-El-Leil M. K., Sadekova S., Meloche S., Sauvageau G. (2005). *EN2* is a candidate oncogene in human breast cancer. *Oncogene*.

[40] Gómez-Gómez E., Jiménez-Vacas J. M., Pedraza-Arévalo S. (2019). Oncogenic role of secreted engrailed homeobox 2 (EN2) in prostate cancer. *Journal of Clinical Medicine*.

[41] Zhao Z., Sun W., Guo Z., Zhang J., Yu H., Liu B. (2020). Mechanisms of lncRNA/microRNA interactions in angiogenesis. *Life Sciences*.

[42] Nguyen T. Q., Saitoh M., Trinh H. T. (2016). Truncation and microdeletion of EVC/EVC2 with missense mutation of EFCAB7 in Ellis-van Creveld syndrome. *Congenital Anomalies*.

[43] Zhang X., Wan S., Yu Y. (2020). Identifying potential DNA methylation markers in early-stage colorectal cancer. *Genomics*.

[44] Sun Z., Xue H., Wei Y. (2019). Mucin O-glycosylating enzyme GALNT2 facilitates the malignant character of glioma by activating the EGFR/PI3K/Akt/mTOR axis. *Clinical Science (London, England)*.

[45] Liu S. Y., Shun C. T., Hung K. Y. (2016). Mucin glycosylating enzyme GALNT2 suppresses malignancy in gastric adenocarcinoma by reducing MET phosphorylation. *Oncotarget*.

[46] Monnier A., Boniface R., Bouvet R. (2018). The expression of EMX2 lead to cell cycle arrest in glioblastoma cell line. *BMC Cancer*.

[47] Wang L., Jin J., Zhou Y. (2019). EMX2 is epigenetically silenced and suppresses epithelial‑mesenchymal transition in human esophageal adenocarcinoma. *Oncology Reports*.

[48] Zhang Y., Mao X. Y., Liu X. (2015). High frequency of the SDK1:AMACR fusion transcript in Chinese prostate cancer. *International Journal of Clinical and Experimental Medicine*.

[49] Ren S., Peng Z., Mao J. H. (2012). RNA-seq analysis of prostate cancer in the Chinese population identifies recurrent gene fusions, cancer-associated long noncoding RNAs and aberrant alternative splicings. *Cell Research*.

[50] Lukens J. R., Gurung P., Shaw P. J. (2015). The NLRP12 sensor negatively regulates autoinflammatory disease by modulating interleukin-4 production in T cells. *Immunity*.

