# Supplementary material for: Construction of Prognostic Risk Model of Patients with Skin Cutaneous Melanoma Based on TCGA-SKCM Methylation Cohort
Source: Comput Math Methods Med. 2022 Aug 25;2022:4261329. doi: 10.1155/2022/4261329 (PMC9436567; doi:10.1155/2022/4261329)
Supplement: Supplementary Materials — Table S1: the clinical data was accessed from TCGA-SKCM dataset. Table S2: the result of multivariate Cox regression for the methylation sites based on SKCM patients. [file 4261329.f1.zip › Table S1 (1).pdf]

id	fustat	futime	age	gender	T	N	M	tumor_stage	smoking	tumor_grade
TCGA-D3-A8GR	Dead	3943	19973	female	Tis	N0	M0	stage 0	None	not reported
TCGA-D3-A2JG	Dead	3453	11105	female	T3a	N1a	M0	stage iiia	None	not reported
TCGA-EB-A3XF	Alive	278	20910	male	T4b	N0	M0	stage iic	None	not reported
TCGA-ER-A199	Dead	279	31661	female	T4b	N3	M0	stage iiic	None	not reported
TCGA-WE-A8ZQ	Alive	1923	17824	male	T3a	N0	M0	stage iia	None	not reported
TCGA-EE-A2MP	Alive	7563	12474	female	T2	N0	M0	stage i	None	not reported
TCGA-EE-A182	Dead	447	30718	female	T4b	N1b	M0	stage iiic	None	not reported
TCGA-YG-AA3P	Alive	439	23219	female	T4a	N0	M0	stage iib	None	not reported
TCGA-D3-A2JA	Alive	3514	25085	male	T2a	N1a	M0	stage iiia	None	not reported
TCGA-FS-A1Z0	Dead	6164	11815	female	T1a	N0	M0	stage ia	None	not reported
TCGA-GF-A4EO	Alive	591	27114	female	T0	N3	M0	stage iiic	None	not reported
TCGA-BF-A1PX	Dead	282	20626	male	T4b	N2a	M0	stage iiib	None	not reported
TCGA-EB-A6QZ	Dead	352	28078	female	T3a	N0	M0	stage iia	None	not reported
TCGA-ER-A19M	Dead	1857	13300	male	T2a	N0	M0	stage ib	None	not reported
TCGA-FS-A1ZQ	Dead	4062	11599	male	TX	N0	M0	i/ii nos	None	not reported
TCGA-DA-A1IA	Dead	2005	11744	female	T2a	N1b	M0	stage iiib	None	not reported
TCGA-GN-A4U8	Alive	1487	18953	male	none	none	none	not reported	None	not reported
TCGA-Z2-A8RT	Alive	839	15342	female	T3b	N0	M0	stage iib	None	not reported
TCGA-DA-A95W	Alive	1136	19105	male	TX	N1b	M0	stage iiib	None	not reported
TCGA-EE-A20H	Dead	5118	20476	male	T2	N0	M0	stage i	None	not reported
TCGA-EB-A430	Alive	-2	30344	male	T4b	N0	M0	stage iic	None	not reported
TCGA-EB-A4OY	Alive	977	23773	female	T4b	N1a	M0	stage iiib	None	not reported
TCGA-BF-A5EP	Alive	335	27622	female	T4b	N3	M0	stage iiic	None	not reported
TCGA-BF-A3DL	Alive	769	30805	female	T3b	N2	M0	stage iiib	None	not reported
TCGA-ER-A1A1	Alive	3196	21209	male	TX	N3	M0	stage iiic	None	not reported
TCGA-D3-A8GP	Alive	4638	28234	male	T2	N2c	M0	stage iii	None	not reported
TCGA-EE-A3J4	Dead	3869	26540	male	T3a	N0	M0	stage ii	None	not reported
TCGA-D3-A51T	Alive	818	21823	female	T4b	N1b	M0	stage iiic	None	not reported
TCGA-D3-A1Q4	Alive	3408	19687	female	T2b	N1b	M0	stage iiic	None	not reported
TCGA-BF-AAOU	Alive	476	26750	female	T4b	N0	M0	stage iic	None	not reported
TCGA-EE-A2GJ	Dead	2270	30602	male	T1a	N0	M0	stage ia	None	not reported
TCGA-ER-A3EV	Dead	1429	20354	male	T4	N0	M0	stage iii	None	not reported
TCGA-EE-A29M	Alive	1729	12350	female	T2a	N0	M0	stage ib	None	not reported
TCGA-EB-A5VV	Alive	214	27364	female	T3b	N1	M0	stage iiib	None	not reported
TCGA-EB-A550	Dead	264	27556	female	T4b	NX	M0	stage iic	None	not reported
TCGA-EE-A2GS	Dead	2470	10421	female	T2a	N0	M0	stage ib	None	not reported
TCGA-D3-A2JE	Dead	841	27604	female	TX	N3	M0	stage iiic	None	not reported
TCGA-EE-A17Y	Dead	828	25281	male	T3b	N1a	M0	stage iiib	None	not reported
TCGA-FS-A4F2	Dead	1525	16944	female	T4b	N0	M0	stage iic	None	not reported
TCGA-D3-A3C3	Alive	None	None	female	TX	N0	M0	i/ii nos	None	not reported
TCGA-EE-A20I	Dead	412	28883	male	TX	N0	M1c	stage iv	None	not reported
TCGA-D3-A2JO	Alive	2010	18577	female	TX	N3	M0	stage iiic	None	not reported
TCGA-FR-A728	Alive	583	19936	female	T4b	N2a	M0	stage iiib	None	not reported
TCGA-GN-A26C	Dead	821	28336	male	T4b	N2b	M0	stage iiic	None	not reported
TCGA-W3-A825	Dead	1917	22097	female	T3	N0	M0	stage ii	None	not reported
TCGA-EE-A20C	Dead	4601	21660	male	Tis	N0	M0	stage 0	None	not reported
TCGA-EE-A2MF	Dead	8174	14312	female	T2	N0	M0	stage i	None	not reported
TCGA-D3-A3C1	Alive	None	None	male	TX	N0	M0	i/ii nos	None	not reported
TCGA-FS-A1YX	Dead	1478	14387	female	T2	N0	M0	stage i	None	not reported
TCGA-XV-AB01	Alive	403	20058	female	T3	NX	M0	stage ii	None	not reported
TCGA-EE-A2MK	Alive	5487	6831	female	T4a	N0	M0	stage iii	None	not reported
TCGA-EE-A180	Dead	2889	25523	male	T4a	N0	M0	stage iii	None	not reported
TCGA-BF-AAP4	Alive	335	22303	male	T4b	N0	M0	stage iic	None	not reported
TCGA-YD-A9TA	Alive	1496	27498	male	none	none	none	not reported	None	not reported
TCGA-GN-A262	Alive	4255	17239	female	none	none	none	not reported	None	not reported
TCGA-D3-A51F	Alive	1695	18776	male	T4b	N1b	M0	stage iiic	None	not reported
TCGA-FS-A4F8	Dead	5318	19137	male	T1	N0	M0	stage i	None	not reported
TCGA-EE-A2GL	Alive	2423	14701	female	T3a	N0	M0	stage iia	None	not reported
TCGA-FS-A1YW	Dead	6598	19078	male	T1b	N0	M0	stage ib	None	not reported
TCGA-D3-A2J8	Dead	1992	17626	male	T2a	N0	M0	stage ib	None	not reported
TCGA-D3-A2JB	Dead	5110	25784	female	Tis	N0	M0	stage 0	None	not reported
TCGA-FS-A1Z3	Dead	636	26435	female	TX	N0	M1	stage iv	None	not reported
TCGA-ER-A2NF	Dead	877	19499	male	T3b	N3	M0	stage iiib	None	not reported
TCGA-D3-A3CC	Alive	2644	25396	female	T4b	N0	M0	stage iic	None	not reported
TCGA-EB-A5VU	Dead	321	20610	male	T4b	N1	M0	stage iiib	None	not reported
TCGA-W3-AA1Q	Dead	2101	21033	male	TX	N1	M0	stage iii	None	not reported
TCGA-EB-A82C	Alive	17	25850	female	T4b	N0	M0	stage iic	None	not reported
TCGA-WE-AA9Y	Alive	370	13850	male	T2a	N3	M0	stage iiic	None	not reported
TCGA-EB-A42Y	Dead	721	26712	female	T4b	N0	M0	stage iic	None	not reported
TCGA-ER-A195	Dead	1078	16948	male	TX	N0	M0	not reported	None	not reported
TCGA-EB-A5SF	Dead	369	28772	female	T4b	NX	M0	stage iic	None	not reported
TCGA-XV-A9W2	Alive	417	29836	male	T1	N0	M0	stage i	None	not reported
TCGA-RP-A6K9	Alive	None	None	female	none	none	none	not reported	None	not reported
TCGA-EE-A3AE	Alive	1658	19216	female	T1a	N0	M0	stage ia	None	not reported
TCGA-DA-A1HV	Alive	2329	27553	female	T0	N2b	M0	stage iiib	None	not reported
TCGA-EE-A29X	Dead	545	21363	female	T2a	N0	M0	stage ib	None	not reported
TCGA-FR-A69P	Alive	478	12679	female	TX	N3	none	stage iiic	None	not reported
TCGA-EB-A3XD	Alive	1160	19431	female	T4b	NX	M0	stage iic	None	not reported
TCGA-FR-A7U9	Alive	571	23245	female	T3b	N3	M0	stage iiic	None	not reported
TCGA-FS-A1ZP	Dead	2273	19175	male	T3	N0	M0	stage ii	None	not reported
TCGA-ER-A2NH	Alive	1264	18220	male	T3a	N3	M0	stage iiic	None	not reported

TCGA-EB-A4OZ	Alive	620	15337	female	T4a	N3	M0	stage iiic	None	not reported
TCGA-ER-A19J	Dead	196	19733	male	TX	N3	M1	stage iv	None	not reported
TCGA-ER-A19Q	Dead	1548	13648	female	none	N0	M0	not reported	None	not reported
TCGA-HR-A2OG	Alive	7	21767	female	none	none	none	not reported	None	not reported
TCGA-D3-A8GV	Dead	5101	9344	male	TX	N0	M0	i/ii nos	None	not reported
TCGA-EE-A184	Dead	2073	26447	male	T2a	N0	M0	stage ib	None	not reported
TCGA-EB-A3Y6	Alive	126	20605	female	T4b	N0	M0	stage iic	None	not reported
TCGA-HR-A2OH	Dead	2004	16910	female	T3b	N2a	M0	stage iiib	None	not reported
TCGA-EE-A2M7	Dead	877	24371	male	T3a	N0	M0	stage ii	None	not reported
TCGA-BF-AAOX	Alive	444	30318	male	T4b	N0	M0	stage iic	None	not reported
TCGA-D3-A3MO	Dead	284	17220	male	TX	N2c	M0	stage iii	None	not reported
TCGA-BF-A1PU	Alive	387	17025	female	T4b	N0	M0	stage iic	None	not reported
TCGA-D3-A8GJ	Alive	7342	6684	male	T3	N0	M0	stage ii	None	not reported
TCGA-D3-A3MU	Alive	1209	19423	male	T3a	N2a	M0	stage iiia	None	not reported
TCGA-EB-A431	Alive	568	12484	male	T4b	N0	M0	stage iic	None	not reported
TCGA-GN-A8LN	Alive	772	24872	male	T4b	NX	M0	stage iic	None	not reported
TCGA-D3-A3C8	Alive	1409	21281	female	TX	N3	M0	stage iiic	None	not reported
TCGA-ER-A42H	Dead	426	28110	male	none	none	none	not reported	None	not reported
TCGA-EE-A2MS	Alive	4942	26466	male	T3a	N0	M0	stage ii	None	not reported
TCGA-Z2-AA3V	Alive	486	20937	female	T1a	N0	M0	stage ia	None	not reported
TCGA-DA-A3F8	Alive	1319	14327	male	T2a	N2b	M0	stage iiib	None	not reported
TCGA-EB-A4XL	Alive	777	20486	female	T4b	NX	M0	stage iic	None	not reported
TCGA-BF-AAP8	Alive	447	21414	male	T4b	N0	M0	stage iic	None	not reported
TCGA-XV-AAZW	Dead	393	22778	female	T4	N0	M0	stage ii	None	not reported
TCGA-EE-A185	Dead	151	20235	female	T4b	N3	M0	stage iiic	None	not reported
TCGA-BF-A1PZ	Alive	853	26240	female	T4a	N0	M0	stage iib	None	not reported
TCGA-EB-A3HV	Alive	39	13712	male	T4b	N0	M0	stage iic	None	not reported
TCGA-EE-A2MM	Dead	5107	23132	female	T2	N0	M0	stage i	None	not reported
TCGA-EB-A1NK	Alive	1039	17569	male	T4b	N0	M0	stage iic	None	not reported
TCGA-DA-A95X	Alive	2249	22718	male	T2a	N0	M0	stage ib	None	not reported
TCGA-GN-A269	Dead	170	25583	male	T4b	N3	M0	stage iiic	None	not reported
TCGA-D9-A6EA	Alive	766	25582	male	T4a	N3	M0	stage iiic	None	not reported
TCGA-FR-A3R1	Alive	685	25271	male	T4b	N0	M0	stage iic	None	not reported
TCGA-FS-A1ZT	Alive	1617	20284	male	T2	N1b	M0	stage iii	None	not reported
TCGA-D9-A4Z2	Dead	190	18462	male	T4b	N3	M0	stage iiic	None	not reported
TCGA-EE-A3AH	Dead	4222	10988	male	T3b	N0	M0	stage ii	None	not reported
TCGA-WE-A8JZ	Alive	731	25884	male	T4b	N1a	M0	stage iiib	None	not reported
TCGA-EB-A5FP	Dead	454	23833	female	T4b	NX	M1b	stage iv	None	not reported
TCGA-EE-A2GI	Alive	1482	14498	male	T1a	N0	M0	stage ia	None	not reported
TCGA-XV-AAZY	Alive	405	27938	female	T4	N3	M0	stage iiic	None	not reported
TCGA-W3-AA1V	Dead	1280	23314	male	T3	N0	M0	stage ii	None	not reported
TCGA-EE-A2GD	Dead	10346	21526	female	T4	N0	M0	stage iib	None	not reported
TCGA-EB-A6R0	Dead	608	21367	female	T4b	N0	M0	stage iic	None	not reported
TCGA-XV-A9W5	Alive	392	18739	male	T2	N0	M0	i/ii nos	None	not reported
TCGA-BF-AAP7	Alive	318	28073	female	T4b	N0	M0	stage iic	None	not reported
TCGA-GN-A8LK	Dead	1524	25725	male	T1b	NX	none	stage ib	None	not reported
TCGA-DA-A1I2	Dead	5370	16701	male	T4b	N2b	M0	stage iii	None	not reported
TCGA-YG-AA3O	Dead	1154	22999	male	none	none	none	not reported	None	not reported
TCGA-W3-AA1R	Dead	3379	26145	male	T3	N0	M0	stage ii	None	not reported
TCGA-ER-A3ES	Dead	7514	9366	male	none	none	none	not reported	None	not reported
TCGA-DA-A3F3	Dead	319	19330	male	T0	N2b	M0	stage iiib	None	not reported
TCGA-D3-A2JD	Dead	361	21440	male	T4b	N1b	M0	stage iiic	None	not reported
TCGA-XV-A9VZ	Alive	0	32872	female	T4	N0	M0	stage ii	None	not reported
TCGA-D3-A8GI	Dead	1780	25069	male	T1a	N0	M0	stage ia	None	not reported
TCGA-ER-A19S	Alive	1505	29603	female	none	none	none	not reported	None	not reported
TCGA-IH-A3EA	Alive	524	22577	male	T4b	N0	M0	stage iic	None	not reported
TCGA-FR-A8YC	Dead	1059	28680	male	T3b	N0	M0	stage iib	None	not reported
TCGA-FS-A1Z7	Dead	237	7293	male	T4b	N1b	M0	stage iiic	None	not reported
TCGA-D3-A51R	Alive	1941	22040	male	T3a	N0	M0	stage iia	None	not reported
TCGA-EE-A29E	Alive	1940	19961	male	T3a	N1b	M0	stage iiib	None	not reported
TCGA-DA-A95Z	Alive	396	31837	male	TX	N0	M1a	stage iv	None	not reported
TCGA-ER-A3PL	Alive	1010	11032	male	T3b	N0	M1a	stage iv	None	not reported
TCGA-ER-A2ND	Dead	710	20867	female	T1b	N3	M0	stage iiic	None	not reported
TCGA-OD-A75X	Dead	9061	17910	male	TX	NX	M0	not reported	None	not reported
TCGA-GN-A26A	Dead	988	23288	female	T3a	N1a	M0	stage iiia	None	not reported
TCGA-D3-A5GU	Alive	3808	13296	male	T1b	N0	M0	stage ib	None	not reported
TCGA-ER-A19P	Dead	4930	17511	female	none	N0	M0	not reported	None	not reported
TCGA-EE-A29N	Dead	566	28805	male	TX	N0	M0	i/ii nos	None	not reported
TCGA-GF-A6C8	Alive	62	22731	female	T3b	NX	M0	stage iib	None	not reported
TCGA-D3-A2JP	Alive	1812	13552	male	T0	N3	M0	stage iiic	None	not reported
TCGA-EB-A82B	Alive	390	21239	female	T4b	N2	M0	stage iii	None	not reported
TCGA-GN-A267	Dead	1960	14055	male	T4a	N1a	M0	stage iiia	None	not reported
TCGA-D3-A2JK	Dead	368	8952	male	T4b	N2b	M0	stage iiic	None	not reported
TCGA-FS-A1ZM	Alive	3080	27210	male	T2	N2c	M0	stage iii	None	not reported
TCGA-EE-A3JA	Dead	1618	16430	male	T2a	N0	M0	stage ib	None	not reported
TCGA-WE-A8ZR	Dead	274	18051	male	T4b	N1b	M0	stage iiic	None	not reported
TCGA-FS-A1YY	Dead	6953	20396	female	T3a	N0	M0	stage iia	None	not reported
TCGA-D9-A148	Alive	4609	14805	male	TX	N3	M1b	not reported	None	not reported
TCGA-BF-A3DJ	Alive	464	13332	female	T4b	N1	M0	stage iiib	None	not reported
TCGA-DA-A3F5	Dead	6873	16701	male	T1a	N0	M0	stage i	None	not reported
TCGA-D9-A3Z4	Dead	519	19749	male	T4b	N3	M0	stage iiic	None	not reported
TCGA-EB-A44P	Alive	741	21519	female	T4b	N0	M0	stage iic	None	not reported

TCGA-EE-A2GR	Dead	1301	28510	male	T4	N0	M0	stage ii	None	not reported
TCGA-EE-A29Q	Dead	2030	25847	female	T3b	N0	M0	stage iib	None	not reported
TCGA-EB-A97M	Alive	414	24228	male	T4b	N0	M0	stage iic	None	not reported
TCGA-D3-A51G	Alive	None	None	male	Tis	N0	M0	stage 0	None	not reported
TCGA-EE-A3J8	Dead	1044	21688	male	T4a	N1a	M0	stage iiia	None	not reported
TCGA-FS-A4F9	Alive	1035	29324	male	T4b	N3	M0	stage iiic	None	not reported
TCGA-D3-A2JN	Dead	2022	17128	female	T0	N1b	M0	stage iii	None	not reported
TCGA-GN-A266	Dead	308	16456	male	none	none	none	not reported	None	not reported
TCGA-GN-A265	Alive	2948	19712	male	none	none	none	not reported	None	not reported
TCGA-ER-A19F	Dead	802	30176	male	none	N0	M0	not reported	None	not reported
TCGA-D3-A5GT	Alive	487	15935	male	T2b	N3	M0	stage iiic	None	not reported
TCGA-ER-A2NG	Dead	1490	15903	female	T3b	N3	M0	stage iiic	None	not reported
TCGA-EB-A3XE	Alive	180	28148	female	T3a	N0	M0	stage iia	None	not reported
TCGA-FS-A1ZA	Dead	843	16705	female	T4b	N2c	M0	stage iiib	None	not reported
TCGA-EE-A181	Dead	1026	30003	female	T3	N0	M0	stage ii	None	not reported
TCGA-D3-A5GO	Alive	4195	22287	male	T4	N0	M0	stage ii	None	not reported
TCGA-EE-A183	Dead	818	17788	male	Tis	N0	M0	stage 0	None	not reported
TCGA-GF-A3OT	Alive	301	21359	female	T3	N3	M0	stage iiic	None	not reported
TCGA-DA-A1I0	Dead	620	23157	male	T4b	N3	M1a	stage iv	None	not reported
TCGA-EE-A2M5	Dead	659	17950	male	T2	N0	M0	stage i	None	not reported
TCGA-EE-A29P	Alive	1716	26899	female	T4b	N0	M0	stage iic	None	not reported
TCGA-ER-A19K	Dead	469	28916	female	T4b	N0	M0	stage iic	None	not reported
TCGA-ER-A19W	Dead	4507	17830	female	none	none	none	not reported	None	not reported
TCGA-D3-A8GQ	Dead	884	24348	male	T3	N0	M0	stage ii	None	not reported
TCGA-WE-A8ZX	Alive	1089	16736	male	TX	N1b	M0	stage iiib	None	not reported
TCGA-EE-A29A	Dead	1927	25095	male	T3a	N1a	M0	stage iiia	None	not reported
TCGA-3N-A9WC	Alive	2022	30286	male	T2b	NX	M0	stage iia	None	not reported
TCGA-FS-A4F0	Alive	2367	24717	female	T4a	N0	M0	stage iib	None	not reported
TCGA-FS-A1ZZ	Dead	822	19733	female	T3b	N0	M0	stage iib	None	not reported
TCGA-FW-A3TV	Alive	411	20972	female	T1	N2b	M0	stage iiib	None	not reported
TCGA-BF-A5EO	Alive	703	23940	male	T4b	N0	M0	stage iic	None	not reported
TCGA-EE-A3JD	Dead	832	25930	male	TX	N2b	M0	stage iii	None	not reported
TCGA-DA-A1I8	Dead	1640	23286	female	T4b	N0	M0	stage iic	None	not reported
TCGA-EB-A5UM	Alive	779	17867	female	T4b	N0	M0	stage iic	None	not reported
TCGA-EE-A3JE	Alive	1562	27463	male	T3b	N1a	M0	stage iiib	None	not reported
TCGA-FW-A3I3	Alive	531	21731	female	none	N0	M1	stage iv	None	not reported
TCGA-GN-A4U4	Alive	1197	26761	male	T2b	NX	M0	stage iia	None	not reported
TCGA-BF-A1PV	Alive	14	27124	female	T4b	N0	M0	stage iic	None	not reported
TCGA-D3-A1Q9	Dead	961	26471	male	T4b	N2a	M0	stage iiib	None	not reported
TCGA-FS-A4F4	Dead	2028	23663	male	T3a	N0	M0	stage ii	None	not reported
TCGA-EE-A17X	Dead	907	20021	male	T1a	N0	M0	stage ia	None	not reported
TCGA-EB-A44O	Alive	81	25537	male	T4a	N0	M0	stage iib	None	not reported
TCGA-GN-A9SD	Dead	1807	21851	female	T1a	NX	M0	stage ia	None	not reported
TCGA-D3-A8GM	Dead	3259	26782	male	T3b	N0	M0	stage iib	None	not reported
TCGA-EE-A17Z	Dead	263	20970	male	T4a	N0	M0	stage iib	None	not reported
TCGA-EE-A2GT	Alive	1365	28364	male	T3a	N0	M0	stage iia	None	not reported
TCGA-FS-A1ZJ	Dead	1441	27476	female	T2	N0	M0	stage i	None	not reported
TCGA-EE-A2GP	Dead	423	29501	male	T4b	N1a	M0	stage iiib	None	not reported
TCGA-D9-A3Z1	Dead	468	24192	male	T2a	N3	M0	stage iiic	None	not reported
TCGA-GN-A8LL	Dead	650	25000	female	T4b	NX	M0	stage iic	None	not reported
TCGA-EE-A3JI	Dead	4648	17835	male	T2	N0	M0	stage i	None	not reported
TCGA-FS-A1ZG	Dead	295	22252	female	T4b	N2b	M0	stage iiic	None	not reported
TCGA-D9-A4Z6	Dead	561	20072	male	T3b	N1b	M0	stage iiic	None	not reported
TCGA-EE-A29W	Alive	5932	15396	male	Tis	N0	M0	stage 0	None	not reported
TCGA-EE-A2GO	Alive	3857	24159	female	T3b	N0	M0	stage ii	None	not reported
TCGA-ER-A19N	Dead	1341	17443	male	none	none	none	not reported	None	not reported
TCGA-EE-A2GH	Alive	6699	12727	male	T2	N0	M0	stage i	None	not reported
TCGA-FS-A4F5	Dead	874	28412	female	T2a	N0	M0	stage ib	None	not reported
TCGA-YG-AA3N	Alive	306	24525	male	T4b	N0	M0	stage iic	None	not reported
TCGA-D3-A3BZ	Alive	3976	23286	male	T4a	N0	M0	stage iib	None	not reported
TCGA-D3-A3CF	Dead	746	22409	female	T4b	N3	M0	stage iiic	None	not reported
TCGA-YD-A9TB	Alive	None	None	female	none	none	none	not reported	None	not reported
TCGA-ER-A19A	Alive	2365	29167	male	TX	N0	M1	stage iv	None	not reported
TCGA-D9-A4Z5	Alive	218	24979	male	T4a	N0	M0	stage iib	None	not reported
TCGA-WE-AAA0	Alive	1229	17261	male	T1a	N0	M0	stage ia	None	not reported
TCGA-D3-A1Q1	Dead	504	29212	female	T1b	N3	M0	stage iiic	None	not reported
TCGA-GF-A769	Dead	1070	14304	male	T4b	NX	M0	stage iic	None	not reported
TCGA-EE-A29H	Alive	1966	21552	female	T1a	N0	M0	stage ia	None	not reported
TCGA-EE-A3J3	Dead	5237	15446	male	T2	N0	M0	stage ib	None	not reported
TCGA-D3-A1Q3	Dead	507	23604	male	T4b	N0	M0	stage iic	None	not reported
TCGA-EB-A4P0	Dead	326	30225	male	T4b	N0	M0	stage iic	None	not reported
TCGA-EB-A41B	Alive	291	27998	female	T4b	N0	M0	stage iic	None	not reported
TCGA-D3-A2J9	Dead	723	27428	male	T4b	N3	M0	stage iiic	None	not reported
TCGA-D3-A3C6	Dead	1766	19858	female	T2a	N0	M0	stage ib	None	not reported
TCGA-EB-A6L9	Alive	1109	20172	male	TX	N3	M0	stage iiic	None	not reported
TCGA-D3-A8GS	Dead	3564	19127	male	T1	N0	M0	stage i	None	not reported
TCGA-FS-A4FC	Dead	1655	27482	female	T3a	N0	M0	stage iia	None	not reported
TCGA-EE-A2MQ	Dead	1315	25926	female	T3a	N2a	M0	stage iiia	None	not reported
TCGA-ER-A19E	Dead	396	13297	female	T2a	N0	M0	stage ib	None	not reported
TCGA-EE-A2GC	Alive	2051	30084	male	T3b	N0	M0	stage iib	None	not reported
TCGA-BF-A9VF	Alive	440	28312	male	T4b	N0	M0	stage iic	None	not reported
TCGA-ER-A2NE	Dead	613	14259	male	Tis	N0	M0	stage 0	None	not reported

TCGA-D3-A3MV	Alive	1378	14236	female	T2b	N2a	M0	stage iiib	None	not reported
TCGA-W3-A824	Alive	6940	23131	male	T2	N0	M0	stage i	None	not reported
TCGA-D3-A1QA	Alive	2765	20223	male	T2a	N0	M0	stage ib	None	not reported
TCGA-EB-A24D	Alive	645	26439	male	T4a	N2b	M0	stage iiib	None	not reported
TCGA-EE-A20F	Alive	2785	19372	male	T1	N0	M0	stage i	None	not reported
TCGA-D3-A8GD	Alive	718	23109	female	T4b	N3	M0	stage iiic	None	not reported
TCGA-EB-A24C	Alive	632	20539	male	T4b	NX	M0	not reported	None	not reported
TCGA-FS-A1ZE	Dead	1413	14618	male	T4b	N0	M0	stage iic	None	not reported
TCGA-DA-A1I4	Dead	1093	18961	male	T3b	N2b	M0	stage iiic	None	not reported
TCGA-EE-A29T	Alive	11252	18779	female	TX	NX	M0	not reported	None	not reported
TCGA-DA-A1IB	Dead	1235	25219	female	T2b	N2b	M0	stage iiic	None	not reported
TCGA-ER-A19C	Dead	1487	28383	male	T2a	NX	M0	stage i	None	not reported
TCGA-WE-A8ZN	Alive	1794	20942	male	T4a	NX	M0	stage iib	None	not reported
TCGA-EE-A29V	Dead	787	31092	male	T3b	N1b	M0	stage iiic	None	not reported
TCGA-BF-A3DN	Alive	717	29801	female	T3b	N3	M0	stage iiic	None	not reported
TCGA-D3-A3C7	Alive	1429	20979	female	T0	N1b	M0	stage iii	None	not reported
TCGA-EE-A29G	Dead	2192	19664	male	T4a	N2a	M0	stage iiia	None	not reported
TCGA-EE-A3JH	Alive	4086	19852	male	T2	N0	M0	stage ib	None	not reported
TCGA-GN-A26D	Dead	1460	26494	female	T4b	N0	none	stage iic	None	not reported
TCGA-D3-A51K	Alive	1002	18971	male	Tis	N2b	M0	stage iiib	None	not reported
TCGA-EE-A2MR	Alive	4088	22535	male	T2	N0	M0	stage i	None	not reported
TCGA-EB-A6QY	Alive	382	26216	male	T4b	N0	M0	stage iic	None	not reported
TCGA-D3-A5GS	Alive	553	21379	male	T1b	N1b	M1c	stage iv	None	not reported
TCGA-FR-A3YO	Alive	None	None	female	T2	N0	M0	i/ii nos	None	not reported
TCGA-FS-A1ZK	Dead	728	24839	male	T4	N0	M0	stage ii	None	not reported
TCGA-EB-A42Z	Alive	441	18157	male	T4b	N1b	M0	stage iiic	None	not reported
TCGA-ER-A42L	Alive	4533	17984	male	T3	N0	M0	stage ii	None	not reported
TCGA-FS-A1ZW	Alive	1505	24087	male	T2b	N1a	M0	stage iiib	None	not reported
TCGA-D3-A2JL	Alive	5219	15710	female	TX	N0	M0	i/ii nos	None	not reported
TCGA-WE-A8K1	Alive	1492	27191	male	T3b	N3	M0	stage iiic	None	not reported
TCGA-ER-A19G	Alive	9188	17820	female	none	N0	M0	not reported	None	not reported
TCGA-WE-AAA3	Alive	651	30843	female	T4b	N2b	M0	stage iiic	None	not reported
TCGA-D3-A1QB	Alive	2912	27566	female	T0	N2c	M0	stage iii	None	not reported
TCGA-3N-A9WD	Dead	395	30163	male	T2a	N1a	M0	stage iiia	None	not reported
TCGA-FS-A4FD	Dead	2454	14426	male	T2	N3	M0	stage iiic	None	not reported
TCGA-EE-A2A0	Dead	1424	28319	female	T3a	N0	M0	stage iia	None	not reported
TCGA-GN-A268	Dead	1910	30430	female	T4a	N0	M0	stage iib	None	not reported
TCGA-FW-A5DY	Alive	587	17730	female	T3	N1	none	stage iii	None	not reported
TCGA-EE-A2A1	Alive	3527	17109	male	T2a	N0	M0	stage ib	None	not reported
TCGA-GN-A4U3	Alive	3708	11294	male	T3a	N1a	M0	stage iii	None	not reported
TCGA-EB-A3Y7	Dead	326	31622	female	T3a	N2c	M0	stage iiib	None	not reported
TCGA-EB-A551	Alive	590	28671	female	T4b	N2b	M0	stage iiic	None	not reported
TCGA-EE-A29B	Dead	2588	24618	male	T3b	N0	M0	stage iib	None	not reported
TCGA-D3-A3CB	Alive	5065	14563	male	T2	N0	M0	i/ii nos	None	not reported
TCGA-D3-A51J	Alive	4414	7194	male	T0	N1b	M0	stage iii	None	not reported
TCGA-WE-A8K6	Alive	546	29216	male	TX	N1b	M0	stage iiib	None	not reported
TCGA-YD-A89C	Alive	210	15804	female	T1a	NX	M0	stage ia	None	not reported
TCGA-D3-A8GC	Dead	2421	17668	male	TX	N3	M0	stage iiic	None	not reported
TCGA-EB-A5UL	Alive	891	26058	male	TX	N1	M0	stage iii	None	not reported
TCGA-D3-A8GK	Alive	5177	16569	male	T3a	N0	M0	stage iia	None	not reported
TCGA-EE-A2MT	Alive	2166	16768	male	T2a	N0	M0	stage ib	None	not reported
TCGA-EB-A553	Alive	226	22972	male	T4b	N0	M0	stage iic	None	not reported
TCGA-D3-A1Q5	Dead	3424	22200	male	TX	N0	M0	i/ii nos	None	not reported
TCGA-D3-A51E	Alive	5318	14305	female	T2	N0	M0	i/ii nos	None	not reported
TCGA-D3-A8GL	Dead	2711	15947	male	T2a	N1b	M0	stage iiib	None	not reported
TCGA-EE-A29C	Dead	2402	7510	male	T2a	N0	M0	stage ib	None	not reported
TCGA-D3-A5GR	Alive	5424	8637	female	T1b	N1	M0	stage iii	None	not reported
TCGA-FS-A1ZF	Dead	470	28491	female	T4b	N0	M0	stage iic	None	not reported
TCGA-EE-A29R	Alive	440	17857	female	T3b	N1b	M0	stage iiic	None	not reported
TCGA-RP-A694	Alive	21	26044	male	TX	NX	M1c	stage iv	None	not reported
TCGA-DA-A1HW	Dead	1096	13585	female	T1a	N1b	M0	stage iiib	None	not reported
TCGA-D9-A6E9	Alive	301	27537	female	T3a	N1	M0	stage iiia	None	not reported
TCGA-EE-A2MH	Dead	516	24125	male	T4a	N0	M0	stage iii	None	not reported
TCGA-WE-A8ZM	Alive	3082	25869	male	TX	N1b	M0	stage iiib	None	not reported
TCGA-EE-A2ME	Dead	3141	18841	male	T2	N0	M0	stage i	None	not reported
TCGA-ER-A19L	Dead	4000	13061	male	none	none	none	not reported	None	not reported
TCGA-DA-A1I7	Alive	2703	22940	male	T0	N2b	M0	stage iiib	None	not reported
TCGA-EE-A2MD	Dead	1438	19333	male	T3a	N0	M0	stage ii	None	not reported
TCGA-W3-AA1O	Dead	122	31351	male	TX	N2	M0	stage iii	None	not reported
TCGA-EB-A44N	Dead	205	21623	male	T4b	N0	M0	stage iic	None	not reported
TCGA-EB-A4IQ	Dead	636	15516	female	T4b	N1	M0	stage iiib	None	not reported
TCGA-ER-A194	Dead	1354	28175	male	none	N0	M0	not reported	None	not reported
TCGA-EE-A2A6	Alive	2620	15953	male	T1a	N0	M0	stage ia	None	not reported
TCGA-D3-A2JC	Alive	2639	19644	female	T0	N2b	M0	stage iii	None	not reported
TCGA-D3-A1Q6	Dead	2184	20230	male	T4	N1b	M0	stage iii	None	not reported
TCGA-D9-A149	Alive	1663	23779	female	TX	N1b	M0	not reported	None	not reported
TCGA-EB-A44Q	Alive	422	18910	female	TX	N3	M0	stage iiic	None	not reported
TCGA-FS-A1Z4	Dead	854	22932	male	T1	N0	M0	stage i	None	not reported
TCGA-D3-A5GN	Alive	4129	5684	female	T1	N0	M0	stage i	None	not reported
TCGA-BF-A5EQ	Alive	323	23353	male	T4b	N0	M0	stage iic	None	not reported
TCGA-RP-A693	Alive	10	28394	male	TX	NX	M1c	stage iv	None	not reported
TCGA-EB-A51B	Alive	931	19645	male	T4b	NX	M0	stage iic	None	not reported

TCGA-EB-A44R	Dead	315	19298	male	TX	N2b	M0	stage iiib	None	not reported
TCGA-EE-A3AD	Dead	875	18491	male	T0	N1b	M0	stage iii	None	not reported
TCGA-DA-A1HY	Alive	4407	15357	male	T2b	N1	M0	stage iii	None	not reported
TCGA-FR-A3YN	Alive	2828	16278	male	T2a	N0	M0	stage ib	None	not reported
TCGA-D9-A1X3	Alive	551	23171	male	T4b	N2b	none	not reported	None	not reported
TCGA-D3-A3MR	Alive	3151	15695	male	T0	N1b	M0	stage iii	None	not reported
TCGA-EE-A2MG	Dead	3139	8405	male	T2	N0	M0	stage i	None	not reported
TCGA-EE-A2ML	Dead	6590	13140	male	T3a	N0	M0	stage ii	None	not reported
TCGA-RP-A695	Alive	None	None	male	TX	NX	M1c	stage iv	None	not reported
TCGA-EB-A41A	Alive	0	32872	male	T4b	N0	M0	stage iic	None	not reported
TCGA-EE-A20B	Alive	4070	24445	female	T3	N0	M0	stage ii	None	not reported
TCGA-GN-A4U5	Alive	1156	22499	female	T2a	NX	M0	stage ib	None	not reported
TCGA-D3-A2J6	Dead	1321	24066	male	T3b	N0	M0	stage iib	None	not reported
TCGA-EB-A57M	Dead	472	20698	male	T4b	N1	M0	stage iiib	None	not reported
TCGA-EE-A3J5	Dead	1124	26226	male	T4a	N1	M0	stage iii	None	not reported
TCGA-LH-A9QB	Alive	11217	9058	female	none	none	none	not reported	None	not reported
TCGA-EE-A2MU	Alive	1620	26237	male	T1a	N0	M0	stage ia	None	not reported
TCGA-D9-A6EG	Dead	698	20570	male	T4a	N1	M0	stage iiia	None	not reported
TCGA-FS-A1ZS	Alive	4526	19813	male	T2	N0	M0	stage i	None	not reported
TCGA-EE-A3J7	Alive	1949	15896	male	T2	N0	M0	stage i	None	not reported
TCGA-FR-A2OS	Dead	368	18122	female	T4b	N0	M0	stage iic	None	not reported
TCGA-3N-A9WB	Dead	518	26176	male	T1a	NX	M0	stage ia	None	not reported
TCGA-BF-AAP6	Alive	325	20384	male	T4b	N2	M0	stage iii	None	not reported
TCGA-D3-A8GE	Alive	804	9517	male	TX	N0	M1b	stage iv	None	not reported
TCGA-DA-A1I1	Alive	6768	20399	male	T0	N2a	M0	stage iii	None	not reported
TCGA-D9-A1JX	Dead	216	29323	female	TX	NX	M0	not reported	None	not reported
TCGA-ER-A2NB	Dead	857	20938	male	T4b	N2	M0	stage iiib	None	not reported
TCGA-D9-A1JW	Not Repor	857	30142	male	T1a	N2a	M0	not reported	None	not reported
TCGA-FR-A8YD	Dead	1103	20782	female	T4b	N0	M0	stage iic	None	not reported
TCGA-WE-AAA4	Alive	760	20708	female	TX	N3	M0	stage iiic	None	not reported
TCGA-D3-A1Q7	Alive	4053	15531	female	T1b	N0	M0	stage ib	None	not reported
TCGA-RP-A690	Alive	6	24450	female	none	none	none	not reported	None	not reported
TCGA-BF-AAP2	Alive	405	22734	male	T3b	N0	M0	stage iib	None	not reported
TCGA-D3-A2JF	Alive	1888	27255	male	T1a	N0	M0	stage ia	None	not reported
TCGA-BF-AAP0	Alive	454	14773	female	T4	NX	M1	stage iv	None	not reported
TCGA-EE-A2GE	Alive	5286	16353	male	T2	N0	M0	stage i	None	not reported
TCGA-WE-A8ZY	Dead	1506	22676	male	T3a	NX	M0	stage iia	None	not reported
TCGA-WE-A8K5	Dead	1860	23837	male	T2a	N3	M1c	stage iv	None	not reported
TCGA-GF-A2C7	Alive	21	17545	male	T4b	N0	M0	stage iic	None	not reported
TCGA-FS-A1ZN	Dead	730	15890	male	T4b	N1a	M0	stage iiia	None	not reported
TCGA-FS-A1ZY	Dead	824	26197	male	T3b	N0	M0	stage iib	None	not reported
TCGA-WE-A8K4	Alive	614	31308	male	T4a	NX	M0	stage iib	None	not reported
TCGA-EB-A4IS	Alive	774	28315	male	T3b	NX	M0	stage iib	None	not reported
TCGA-ER-A19D	Dead	383	16968	female	T2a	N0	M0	stage ib	None	not reported
TCGA-D3-A51N	Alive	688	20559	female	T0	N3	M1c	stage iv	None	not reported
TCGA-EE-A2A2	Alive	1814	26048	male	T4b	N1b	M0	stage iiic	None	not reported
TCGA-EE-A29S	Dead	1864	29087	male	T3a	N0	M0	stage iia	None	not reported
TCGA-ER-A197	Dead	424	30454	female	T4b	N1a	M0	stage iiib	None	not reported
TCGA-DA-A95Y	Dead	430	25083	male	T4b	N0	M0	stage iic	None	not reported
TCGA-FR-A726	Dead	0	32872	male	T4b	N0	M0	stage iic	None	not reported
TCGA-GN-A4U9	Dead	673	26118	male	T2b	N3	M0	stage iiic	None	not reported
TCGA-D3-A1Q8	Dead	854	12330	male	T0	N3	M1b	stage iv	None	not reported
TCGA-ER-A193	Dead	955	22702	male	T3b	N0	M0	stage iib	None	not reported
TCGA-EE-A3JB	Alive	6138	22150	female	T3a	N1	M0	stage iii	None	not reported
TCGA-DA-A1I5	Alive	4107	10017	female	T1a	N0	M1c	stage iv	None	not reported
TCGA-GF-A6C9	Alive	480	28774	male	none	none	none	stage iiib	None	not reported
TCGA-ER-A196	Alive	1785	23533	female	T4b	N0	M0	stage iic	None	not reported
TCGA-DA-A95V	Alive	2193	30623	female	T4b	N0	none	stage iic	None	not reported
TCGA-D9-A6EC	Alive	2359	20493	male	T3a	N1	M0	stage iiia	None	not reported
TCGA-WE-A8ZT	Alive	359	9379	female	T3b	N1b	M1b	stage iv	None	not reported
TCGA-FW-A5DX	Alive	640	26056	male	T4a	N3	none	stage iiic	None	not reported
TCGA-D3-A3ML	Dead	422	25643	male	T3a	N2a	M0	stage iiia	None	not reported
TCGA-QB-A6FS	Alive	220	18134	male	T0	N3	M0	stage iiic	None	not reported
TCGA-EE-A29D	Dead	425	31790	male	T3b	N1b	M0	stage iiic	None	not reported
TCGA-EB-A85J	Alive	360	24263	female	T4a	N0	M0	stage iib	None	not reported
TCGA-W3-AA21	Dead	3195	9527	male	T2	N0	M0	stage i	None	not reported
TCGA-EB-A299	Alive	378	23322	male	T2b	N0	M0	stage iia	None	not reported
TCGA-EB-A3XB	Alive	796	23091	male	T4	NX	M0	stage ii	None	not reported
TCGA-EE-A2A5	Dead	1195	16041	male	T2a	N0	M0	stage ib	None	not reported
TCGA-FR-A7U8	Alive	847	18346	male	TX	N3	M0	stage iiic	None	not reported
TCGA-XV-AAZV	Alive	412	20545	female	T4	N0	M0	stage ii	None	not reported
TCGA-ER-A19B	Dead	2993	15437	male	TX	N0	M0	not reported	None	not reported
TCGA-DA-A3F2	Dead	1032	20365	male	T4a	N2b	M0	stage iiib	None	not reported
TCGA-ER-A19O	Dead	1032	20740	male	T3b	N1b	M0	stage iiib	None	not reported
TCGA-DA-A1IC	Dead	2071	29859	male	T3a	N2c	M0	stage iiib	None	not reported
TCGA-BF-A3DM	Alive	601	23076	male	T2b	N0	M0	stage iia	None	not reported
TCGA-EE-A3AG	Dead	1265	9161	male	T0	N2c	M0	stage iii	None	not reported
TCGA-ER-A2NC	Dead	1333	18582	male	T2a	N0	M0	stage ib	None	not reported
TCGA-EE-A2M8	Dead	601	19808	female	T3a	N1	M0	stage iii	None	not reported
TCGA-FS-A1ZR	Dead	347	13325	male	T2	N0	M0	stage ii	None	not reported
TCGA-D3-A2J7	Dead	3136	24692	male	T3b	N1b	M0	stage iiic	None	not reported
TCGA-D9-A3Z3	Alive	678	14489	female	T3a	N1b	M0	stage iiib	None	not reported

TCGA-W3-AA1W	Alive	6666	23711	male	T3	N0	M0	stage ii	None	not reported
TCGA-EE-A2MJ	Dead	2927	22050	male	T4b	N0	M0	stage iii	None	not reported
TCGA-FR-A44A	Alive	5299	10707	female	T3a	N0	M0	stage ii	None	not reported
TCGA-FS-A1ZD	Dead	1628	23344	male	T2b	N0	M0	stage iia	None	not reported
TCGA-GN-A263	Dead	467	8952	male	T4b	N3	M1c	stage iv	None	not reported
TCGA-BF-AAP1	Alive	409	31666	male	T4b	N0	M0	stage iic	None	not reported
TCGA-D3-A51H	Alive	1714	22119	male	T1b	N3	M0	stage iiic	None	not reported
TCGA-ER-A19T	Dead	270	18732	male	T4a	N3	M1a	stage iv	None	not reported
TCGA-EE-A2GK	Alive	1665	16879	female	T1	N0	M0	stage i	None	not reported
TCGA-EB-A3XC	Alive	650	27124	male	T4b	N0	M0	stage iic	None	not reported
TCGA-EE-A2GM	Alive	2296	25711	female	T4b	N0	M0	stage iic	None	not reported
TCGA-EE-A2M6	Alive	3932	22564	male	T1	N0	M0	stage i	None	not reported
TCGA-D9-A4Z3	Alive	505	26812	female	T4b	N1b	M0	stage iiic	None	not reported
TCGA-EB-A5SG	Alive	2076	20978	female	none	none	none	not reported	None	not reported
TCGA-FS-A1ZC	Dead	10870	18843	male	TX	N0	M0	i/ii nos	None	not reported
TCGA-ER-A42K	Dead	394	14628	female	T4b	N3	M0	stage iiic	None	not reported
TCGA-EE-A2GB	Alive	1803	18876	male	T2b	N1a	M0	stage iiib	None	not reported
TCGA-EB-A5UN	Alive	1792	18226	male	T4b	NX	M0	stage iic	None	not reported
TCGA-Z2-AA3S	Alive	2950	21318	male	T1a	N0	M0	stage ia	None	not reported
TCGA-GN-A264	Dead	3587	22254	male	none	none	none	not reported	None	not reported
TCGA-EE-A29L	Dead	79	28824	male	T4b	N3	M0	stage iiic	None	not reported
TCGA-EB-A5SE	Dead	401	26965	male	T3b	NX	M0	stage iib	None	not reported
TCGA-BF-A5ES	Alive	490	28092	female	T4b	N0	M0	stage iic	None	not reported
TCGA-D3-A5GL	Alive	3826	27095	male	T2a	N0	M0	stage ib	None	not reported
TCGA-EE-A2MC	Dead	1871	26872	male	T2	N0	M0	stage i	None	not reported
TCGA-FS-A1ZU	Dead	808	25856	female	T4b	N0	M0	stage iic	None	not reported
TCGA-FS-A1ZH	Dead	996	26232	female	T3b	N2c	M1c	stage iv	None	not reported
TCGA-EE-A3AF	Dead	420	17731	female	T0	N3	M0	stage iiic	None	not reported
TCGA-FS-A4FB	Dead	813	16896	female	T2	N1a	M0	stage iii	None	not reported
TCGA-FR-A729	Alive	6716	14043	female	T1	N0	M0	stage i	None	not reported
TCGA-EE-A3AC	Alive	1948	17319	male	T0	N2b	M0	stage iii	None	not reported
TCGA-FR-A7UA	Alive	1164	24032	female	T2a	N0	M0	stage ib	None	not reported
TCGA-EB-A5KH	Dead	619	20424	male	T0	N1	M0	stage iii	None	not reported
TCGA-WE-A8ZO	Alive	2145	26946	female	T3a	N1b	M0	stage iiib	None	not reported
TCGA-W3-A828	Dead	3683	24258	male	T3	N0	M0	stage ii	None	not reported
TCGA-EE-A2GN	Dead	3106	24683	male	T2b	N0	M0	stage iia	None	not reported
TCGA-HR-A5NC	Alive	0	32872	female	T4	NX	M0	not reported	None	not reported
TCGA-DA-A960	Alive	804	26708	male	T3b	N0	M0	stage iib	None	not reported
TCGA-D3-A8GB	Dead	938	17871	male	T3a	N1b	M0	stage iiib	None	not reported
TCGA-FR-A8YE	Alive	3176	15159	male	T1a	N0	M0	stage ia	None	not reported
TCGA-EE-A2GU	Alive	2884	23784	female	T1a	N0	M0	stage ia	None	not reported
TCGA-D3-A3CE	Dead	1832	27355	female	T0	N1b	M0	stage iii	None	not reported
TCGA-GN-A4U7	Dead	317	20458	female	T2b	N3	M0	stage iiic	None	not reported
TCGA-ER-A3ET	Dead	2829	23650	female	T3a	N1a	M0	stage iiia	None	not reported
TCGA-FS-A1ZB	Dead	1486	20963	male	T3a	N0	M0	stage ii	None	not reported
TCGA-BF-A5ER	Alive	327	23199	male	T4b	N0	M0	stage iic	None	not reported
TCGA-FW-A3TU	Dead	1691	26589	female	none	none	none	not reported	None	not reported
TCGA-D3-A8GO	Dead	1691	None	female	T2	N0	M0	i/ii nos	None	not reported
TCGA-EE-A2MI	Dead	6225	15880	male	T4	N0	M0	stage iib	None	not reported
TCGA-D3-A2JH	Alive	1280	24882	male	T1b	N0	M0	stage ib	None	not reported
TCGA-D3-A8GN	Alive	4897	10185	female	TX	N0	M0	i/ii nos	None	not reported
TCGA-EE-A3AA	Alive	3781	17196	male	T0	N2a	M0	stage iii	None	not reported
TCGA-FW-A3R5	Alive	1124	24868	male	TX	N2	M0	stage iii	None	not reported
TCGA-ER-A198	Dead	1544	16540	male	none	NX	M0	not reported	None	not reported
TCGA-EE-A3AB	Alive	3733	11054	male	T0	N2a	M0	stage iii	None	not reported
TCGA-EB-A85I	Alive	362	24408	male	T4b	N0	M0	stage iic	None	not reported
TCGA-QB-AA9O	Dead	549	27009	male	TX	N3	M0	stage iiic	None	not reported
TCGA-BF-A1Q0	Alive	831	29380	male	T4b	N0	M0	stage iic	None	not reported
TCGA-ER-A19H	Dead	4634	14935	male	none	N0	M0	not reported	None	not reported
TCGA-EB-A5SH	Alive	1643	21920	female	T4	N0	M0	stage iii	None	not reported
TCGA-EE-A2MN	Dead	1446	21202	male	T2	N0	M0	stage i	None	not reported
