# Supplementary material for: Construction of Prognostic Risk Model of Patients with Skin Cutaneous Melanoma Based on TCGA-SKCM Methylation Cohort
Source: Comput Math Methods Med. 2022 Aug 25;2022:4261329. doi: 10.1155/2022/4261329 (PMC9436567; doi:10.1155/2022/4261329)
Supplement: Supplementary Materials — Table S1: the clinical data was accessed from TCGA-SKCM dataset. Table S2: the result of multivariate Cox regression for the methylation sites based on SKCM patients. [file 4261329.f1.zip › Table S2 (1).pdf]

id	HR	HR.95L	HR.95H	pvalue
cg12407867	0.3399545	0.1079834	1.070248	0.0651923
cg11126767	3.3707886	1.1395217	9.971039	0.0280921
cg22825961	1.0937383	0.3205534	3.731869	0.886219
cg10602282	0.551779	0.2501148	1.217281	0.1407743
cg20233228	231.97553	0.0049223	10932479	0.3211671
cg03052794	1.9898326	0.7199654	5.499478	0.1846641
cg14825033	0.9720054	0.2103536	4.49146	0.9709956
cg05149213	0.7048865	0.2893692	1.717062	0.4413805
cg24091995	1.5111797	0.8253748	2.766821	0.1808872
cg15878555	1.55E-05	4.96E-16	484318	0.3690523
cg01675104	3.042924	0.8591507	10.77737	0.0845847
cg00990385	0.2364845	0.0911506	0.613544	0.0030343
cg06298629	9.12E-06	2.13E-18	39001329	0.4341907
cg26835302	1.0241848	0.5825433	1.800647	0.933845
cg14441603	1.9538991	0.5312058	7.186897	0.3134602
cg14626259	1.4518696	0.7137001	2.953517	0.3034546
cg06684296	0.4452132	0.154116	1.28614	0.1349048
cg10366407	0.0679747	0.0080333	0.575175	0.0136033
cg16102240	2.1508999	0.6474528	7.145495	0.2111885
cg08576104	3.72E-24	4.16E-42	3.33E-06	0.0105286
cg15085899	0.1760238	0.0253739	1.221113	0.0787777
cg02370923	0.4934367	0.2103283	1.157618	0.1044719
cg10631544	0.1144933	0.014432	0.90831	0.0402694
cg13609937	173.76144	9.51E-13	3.17E+16	0.758207
cg03482973	1.6131887	0.7122948	3.653513	0.2515664
cg08169864	0.2740775	0.068548	1.095853	0.067173
cg03466198	4.2649848	1.2137208	14.98705	0.0236953
cg18559901	1.2002338	0.5053724	2.850494	0.6791913
cg13827859	1.2136717	0.5491955	2.682103	0.6321863
cg03443944	0.1633485	0.0447289	0.596544	0.0061127
cg02272831	1.6166509	0.6586436	3.968095	0.2944069
cg24130739	0.2232009	0.057083	0.872741	0.0311138
cg08537215	1.4955795	0.6004618	3.725063	0.3873159
cg12437873	1.07E+24	11.830117	9.73E+46	0.0402116
cg03809021	6.8723513	1.3512853	34.95132	0.0201924
cg17071948	2.5574139	0.4650521	14.06373	0.2802914
cg27397943	3058.9774	11.102323	842827.5	0.0051158
cg20051314	0.6336203	0.2621947	1.531208	0.3107848
cg04848570	2.3059551	0.9963663	5.336822	0.0510022
cg08901520	2.1197744	0.7843801	5.728656	0.1385604
cg03128945	0.7184891	2.26E-05	22871.37	0.9501679
cg06878741	0.6558601	0.2638162	1.630501	0.3639843
cg27540367	11.554689	1.3005951	102.6537	0.0281065
cg19641266	3.77E-21	4.09E-39	0.00347	0.0258589
cg26894278	0.4109954	0.1590392	1.062111	0.0664213
cg24679905	1.6897907	0.6788165	4.206428	0.2595701
cg07059978	1.5290245	0.6468809	3.614137	0.3332979
cg05985317	3.0026953	0.4606755	19.57165	0.2503102
cg17093267	0.2527418	0.0780386	0.81855	0.0217964
cg25943066	1.0527626	0.3918078	2.828706	0.9187888
cg23506077	0.1266745	0.0192036	0.835594	0.0318276
cg05314679	1.6754434	0.7059013	3.976633	0.2419099
cg12035880	1.4375763	0.456193	4.530156	0.5354006
cg18516125	0.0065335	8.66E-06	4.931158	0.136745
cg06486460	0.1505661	0.0477792	0.474477	0.0012249
cg24974737	1.6763178	0.678315	4.142679	0.2630888
cg11081833	1.5942052	0.3416959	7.437872	0.552862

cg20040306	0.445574	0.2208912	0.898796	0.023946
cg20938454	0.7557635	0.2293357	2.490578	0.6453517
cg18548879	0.3242853	0.1281655	0.820509	0.0174232
cg10711303	8.6759302	0.551707	136.4343	0.1243179
cg15730180	0.0987921	0.0121743	0.801682	0.0302433
cg00369194	0.1829393	0.0539405	0.620439	0.0064106
cg20672161	3.9186698	0.8704463	17.64149	0.0752052
cg07991704	0.504573	0.2314591	1.099952	0.0853655
cg06909229	3.4184174	1.2109222	9.650147	0.020265
cg03850797	0.032765	4.95E-08	21668.84	0.6171321
cg26216855	21.857174	0.1634607	2922.636	0.216879
cg04843555	0.669435	0.3589307	1.248551	0.2069693
cg18584368	0.5322871	0.2209037	1.282593	0.1599324
cg13781819	0.6378001	0.3284138	1.238647	0.1841806
cg07866762	0.1238548	0.0427748	0.358622	0.0001179
cg11922607	1982.9705	0.153067	25689214	0.1160705
cg25154801	2.0995228	0.944924	4.664921	0.068624
cg23109891	0.417125	0.1442259	1.206395	0.1065978
cg20698942	1.9216853	0.3715289	9.93967	0.4359444
cg10816903	1.6736248	0.1737687	16.11924	0.6558633
cg00004883	0.335324	0.1484587	0.757397	0.0085795
cg20466748	1.992E+12	3.27E-07	1.21E+31	0.1994081
cg07719077	1.187388	0.1686306	8.360823	0.8630647
cg27036638	0.277698	0.0904378	0.852698	0.0251979
cg08865695	0.8530124	0.4038455	1.801754	0.6768848
cg23898701	0.5581113	0.1739391	1.790789	0.3268708
cg21578066	40.764674	2.6799903	620.0614	0.0075898
cg26393379	1.610199	0.8406976	3.084035	0.1508207
cg09250458	0.4210455	0.1176521	1.50681	0.1836126
cg21613099	0.8111903	0.3689756	1.783397	0.6026338
cg15746696	0.5617487	0.2655206	1.188464	0.131461
cg12886694	0.2182748	0.0075437	6.315718	0.3753551
cg03571073	8.280881	1.4408399	47.59237	0.0178214
cg16940399	0.4312152	0.2123152	0.875804	0.0199759
cg08162465	1.1396879	0.5323427	2.439948	0.7363723
cg14006569	1.6765847	0.6453697	4.355544	0.2887366
cg01833485	2.0886792	0.8515511	5.123099	0.1076318
cg01579784	0.1861476	0.0582965	0.594391	0.0045371
cg09301458	12.1224	1.1190405	131.3202	0.0401225
cg19380303	30.350179	1.7255384	533.8237	0.0196547
cg25576086	0.263684	0.0077544	8.966439	0.4587779
cg06779393	0.9873844	0.4710464	2.069707	0.9731789
cg12213910	2.7873363	0.2780971	27.93716	0.3833777
cg20311846	0.1470786	0.0361768	0.597955	0.0073936
cg03969996	2.2724816	0.7871175	6.560866	0.1291522
cg27469738	4.0894275	0.6292971	26.57476	0.1402302
cg05873267	0.1420793	0.0257043	0.785336	0.025288
cg15133208	0.4418381	0.1017731	1.918199	0.2755375
cg20476596	11.230867	0.227543	554.3232	0.2240617
cg00270259	9.9246813	1.3107291	75.14848	0.0262881
cg00580022	0.0355769	0.0006266	2.019812	0.1054839
cg17410625	0.3971966	0.1805553	0.873777	0.0217101
cg11202422	0.6722862	0.3483945	1.29729	0.2364465
cg10505921	0.1459005	0.0008338	25.52864	0.4651041
cg21408624	13.660743	1.2122671	153.9396	0.0343675
cg12910561	1.7917921	0.9145584	3.510458	0.089192
cg20748559	4.4585536	0.7075393	28.09554	0.1114739
cg01055386	0.5600166	0.2646324	1.185111	0.1295416

cg02380278	12580.315	0.0001757	9.01E+11	0.3063317
cg25291907	0.0020211	2.98E-52	1.37E+46	0.9138804
cg05894970	0.1983754	0.055332	0.711212	0.013025
cg09271882	1.5458801	0.4330999	5.517769	0.5022304
cg03085377	2.2767024	0.6435479	8.054372	0.2018693
cg05756136	0.0138798	0.0006326	0.304555	0.0066383
cg07409471	0.3547414	0.1008101	1.248302	0.1064264
cg06684321	1.7283271	0.4693	6.365042	0.410733
cg13202751	1.1992605	0.5136606	2.799953	0.6744696
cg02844852	0.2842305	0.0895448	0.902196	0.0327927
cg27518898	0.0830659	0.0248935	0.277178	5.19E-05
cg07274523	1.5050055	0.5405327	4.190388	0.43395
cg01549227	3.1472992	1.0524939	9.411449	0.0402174
cg18315943	0.4902765	0.2263331	1.062023	0.0707033
cg14047091	3.0646892	0.808425	11.61805	0.0995211
cg17186103	1.6227741	0.7775167	3.386931	0.1971801
cg05952475	1.7251372	0.854378	3.483351	0.1282625
cg06010020	0.2831249	0.1014205	0.790369	0.0159918
cg17495302	45.760288	1.6595001	1261.828	0.0238669
cg14983763	0.3049292	0.1297752	0.716484	0.0064325
cg11249333	0.0020348	2.10E-05	0.196946	0.0078973
cg12043952	0.2169358	0.0430779	1.092466	0.0639194
cg19365151	4.59E-09	2.00E-19	105.6172	0.1147593
cg00218893	1.8640286	0.4761992	7.296532	0.3711091
cg03772350	1.7046424	0.8503024	3.417379	0.1328417
cg17684850	32.141253	0.0368536	28031.46	0.3151421
cg26421310	0.4068408	0.1687079	0.981101	0.0452366
cg14976741	0.324861	0.0942265	1.120011	0.0749964
cg19080320	0.1025655	1.42E-05	743.2413	0.615555
cg12271047	0.5664879	0.1736339	1.84819	0.3462248
cg22385702	2.2434356	0.5957057	8.448808	0.232359
cg05134831	2.70E-09	2.85E-32	2.56E+14	0.4648463
cg12698349	5.7236941	0.6959293	47.07472	0.1046378
cg07039130	0.4105177	0.168202	1.001919	0.0504943
cg06749872	2.9583119	0.9310185	9.400038	0.0659463
cg07741162	2.2713334	1.0906924	4.729982	0.0283855
cg18597448	8.970415	1.6508516	48.74354	0.0110718
cg25212453	2.275046	0.4521703	11.44665	0.318692
cg18884805	3.95E+36	0.0004755	3.28E+76	0.0723646
cg23678906	0.6443988	0.3326442	1.24833	0.1927398
cg23999880	0.0605376	0.0021135	1.734035	0.1013405
cg06749213	0.6166487	0.3054864	1.244755	0.177325
cg07723365	0.786365	0.3411763	1.812464	0.5726773
cg04611437	0.2373258	0.1004448	0.560741	0.0010431
cg16120204	2.2933915	0.7265564	7.239142	0.1569841
cg20242427	0.3654581	0.1826174	0.731363	0.0044579
cg15743985	5.3234505	1.1007091	25.74624	0.0375913
cg10231067	1.3820785	0.6006766	3.179982	0.4465923
cg12232274	0.0076035	0.0001561	0.370442	0.0138621
cg07959741	0.6856325	0.3193013	1.472252	0.3330672
cg26024530	0.3402258	0.124658	0.928569	0.0353232
cg04884313	0.3539287	0.03957	3.165669	0.3528242
cg05679686	0.2763731	0.1121644	0.680984	0.0051895
cg03626648	0.2952729	0.0168853	5.163442	0.403412
cg07891911	0.9498042	0.4029553	2.238779	0.9062892
cg16026522	0.0025189	1.48E-10	42811.95	0.4811428
cg22340067	88.456075	6.5669071	1191.501	0.0007289
cg08778287	5.5786292	0.7133296	43.62794	0.1014113

cg08453486	5.09E-10	1.30E-29	2E+10	0.3525727
cg03615269	1.7892676	0.8177357	3.915053	0.1453083
cg20404260	0.6076069	0.1317816	2.801499	0.5228778
cg27315388	0.0001981	3.04E-07	0.129141	0.0099071
cg09053081	1.1436699	0.5867333	2.22926	0.6934221
cg03592283	0.1887806	0.0280694	1.269642	0.0864447
cg24216712	9.48E-23	1.96E-39	4.58E-06	0.0096747
cg20841648	1.0937211	0.5527766	2.164031	0.7969397
cg21205663	2.0812014	1.0749659	4.029337	0.0296734
cg04598128	1.394664	0.6413655	3.032729	0.4012917
cg03829542	0.2813684	0.1097979	0.721035	0.0082618
cg25323360	0.0290164	6.86E-05	12.27738	0.2512855
cg25729350	0.0905208	0.0038669	2.11901	0.1353905
cg16411160	0.0016066	8.66E-06	0.298008	0.0157671
cg13048591	0.5893223	0.147306	2.357683	0.4547557
cg08587397	0.1614856	0.013004	2.005349	0.1560154
cg12194517	0.3630078	0.0056636	23.26703	0.6330889
cg09481972	0.0006002	8.53E-07	0.422357	0.0265806
cg17316096	0.3029606	0.1238972	0.740817	0.008856
cg24791428	1.946285	0.9099557	4.162867	0.086032
cg19163058	2.0021084	0.9703703	4.130834	0.0603031
cg02423318	2.3356586	0.9565082	5.70335	0.0625547
cg11972810	3.2700444	0.6405551	16.69363	0.1543162
cg16228323	2.9786285	1.3980876	6.345974	0.0046792
cg00077450	0.0012979	2.80E-06	0.60265	0.033871
cg16536718	1.4404661	0.7626412	2.720732	0.2606586
cg07862930	9.0552842	0.6126694	133.8376	0.1088397
cg04055819	1.338664	0.7363642	2.433607	0.3388498
cg11254361	2.6219723	0.9355824	7.348084	0.0667547
cg22380476	0.5606158	0.0521028	6.032118	0.6330612
cg00097146	2.6322561	0.8945589	7.745463	0.078813
cg02348430	4.1136241	1.3791912	12.26944	0.0111943
cg16954204	1.747E+13	0.0237106	1.29E+28	0.0808575
cg13651857	1.017E+09	3.67E-05	2.82E+22	0.1890851
cg21792737	0.3364456	0.1311894	0.862841	0.0233917
cg13609053	8.4612859	1.3869622	51.61882	0.0206404
cg18675610	0.2463545	0.0320104	1.895963	0.1784478
cg10016690	2.136585	0.4145623	11.0116	0.3641563
cg16065270	1.7053843	0.0184002	158.0604	0.8173203
cg11023815	2490.8976	1.7534696	3538453	0.0347206
cg20265878	8.8992056	1.3494509	58.68747	0.0231245
cg03387346	1.279388	0.5637625	2.90341	0.555688
cg19458233	0.5914771	0.3198929	1.093632	0.0940224
cg19588154	4.6104699	1.7878564	11.88934	0.0015665
cg01657493	3.25E-15	3.22E-26	0.000328	0.0098655
cg22927076	0.9174483	0.3692312	2.279632	0.8528097
cg15084390	0.6873428	0.3657074	1.291853	0.244199
cg14064335	1.3034443	0.5157002	3.294486	0.5753646
cg18114036	0.5505508	0.2084556	1.454056	0.2284064
cg24138867	0.4096224	0.1112376	1.508398	0.1796163
cg06756291	3.0390871	0.6829723	13.52332	0.144467
cg07855639	0.1563606	0.0460876	0.530481	0.0029099
cg02750754	4.66E-11	1.17E-22	18.47715	0.0808242
cg09243811	1.4277427	0.3987028	5.112703	0.584292
cg01376079	0.3317877	0.1228123	0.896352	0.0295735
cg11651961	0.9588017	0.0002092	4394.259	0.9921958
cg01872947	2.9461508	0.2529304	34.31697	0.3883718
cg07298996	47.342851	0.0422547	53043.73	0.2815892

cg23760945	1.5486876	0.3373937	7.108707	0.5737294
cg20611680	1.8871429	0.717679	4.962258	0.1979375
cg17038633	0.062447	0.0094011	0.414806	0.0040943
cg16009970	1.7652118	0.7235345	4.306599	0.2117328
cg10098370	0.9400114	0.4676027	1.889684	0.8621465
cg16507827	45.480751	0.6268098	3300.042	0.0807629
cg17535691	1.8367605	1.0276281	3.282986	0.0401754
cg18930012	2.2102964	0.651187	7.502315	0.203371
cg17525357	6.6783392	0.5849232	76.24969	0.1264294
cg10197238	1.4360438	0.621457	3.318366	0.3970853
cg01778908	0.7546428	0.3604061	1.580123	0.455302
cg12397463	0.3350253	0.0911131	1.231897	0.0997555
cg11323848	0.6504749	0.2871312	1.473604	0.3026707
cg06301726	0.2034658	0.064498	0.641855	0.0065997
cg17413120	0.1685902	0.0526529	0.539811	0.0027147
cg00424286	1.466585	0.7360953	2.922001	0.2762441
cg16423910	0.2697093	0.093935	0.774398	0.0148894
cg12739647	0.0021545	2.31E-29	2.01E+23	0.8404969
cg12781405	1.3654664	0.5900045	3.160143	0.4668756
cg01894038	0.3144988	0.097706	1.012318	0.0524479
cg18333690	1.4568202	0.5640839	3.762428	0.4370193
cg08034797	1.311883	0.2323733	7.406345	0.7585441
cg17317007	3.4613647	0.9175613	13.05749	0.066809
cg15394142	1.0764494	0.5667561	2.044519	0.821918
cg24047187	0.6631605	0.3353611	1.311368	0.2377102
cg16264807	1.729934	0.3272293	9.14549	0.5188556
cg08748100	1.8375811	1.0102634	3.3424	0.0462157
cg13509517	1.8083121	0.8990451	3.637184	0.0966165
cg01716527	0.0351667	0.0003898	3.172817	0.1450264
cg04707299	0.0173889	0.0009655	0.313187	0.0060135
cg24058120	0.3815099	0.1407455	1.034135	0.0582261
cg23710218	0.0062814	9.95E-06	3.964892	0.1232595
cg01811064	164393.14	8.45E-16	3.20E+25	0.6143522
cg19739906	0.3050632	0.1440836	0.6459	0.0019217
cg07122178	1.4776496	0.6974926	3.130425	0.3080174
cg26987660	1.9701998	0.730774	5.311748	0.1802038
cg00115654	3.0780975	0.8138664	11.64157	0.097618
cg18129628	0.4219754	0.1604327	1.109894	0.0803513
cg08224785	2.2836507	0.5953023	8.760357	0.228659
cg11691561	2.1438554	1.1314881	4.06201	0.0193443
cg02898051	0.5557684	0.1161628	2.659014	0.4620473
cg18803147	1.8306571	0.8485281	3.949551	0.1232457
cg20306821	2.3878462	0.0366152	155.7225	0.6830204
cg02611507	0.5250669	0.275507	1.000683	0.050243
cg05044994	1.8058709	0.3375797	9.660443	0.4897086
cg17383727	2.078396	0.7929648	5.447568	0.1367218
cg22347212	2.277015	0.8358381	6.203112	0.1075575
cg08136809	0.1789362	0.0595119	0.538013	0.002187
cg21488396	0.6053534	0.3142932	1.165958	0.1333929
cg07715328	306792533	0.0055212	1.7E+19	0.1216013
cg19582265	1.9644189	0.0892315	43.24642	0.6686263
cg25626519	2.4474552	0.5112069	11.71744	0.2626294
cg19926250	2.8964889	0.7114112	11.79297	0.137643
cg15143809	0.1119181	0.0115945	1.080312	0.0583334
cg01866805	3.1896957	1.1016463	9.235413	0.0324815
cg21946979	0.5822622	0.0774317	4.378427	0.5993021
cg07016060	0.0077428	4.25E-07	141.1593	0.3314976
cg02816367	1.2549983	0.5480184	2.874029	0.5910784

cg26035702	7.65E-10	6.43E-17	0.009092	0.0115547
cg14104369	0.1583445	0.0368283	0.680807	0.0132629
cg26367031	1.4789889	0.3084457	7.091713	0.6246122
cg10947001	0.9790252	0.4442734	2.157433	0.9580637
cg00798431	0.5606648	0.2723243	1.154304	0.1163009
cg23362669	1.7144934	0.6810042	4.316402	0.2524481
cg14240790	0.3279953	0.07803	1.378713	0.1281082
cg09554952	0.4487298	0.1783717	1.12887	0.0886732
cg19874726	1.4071556	0.7378532	2.683578	0.2997371
cg10454162	2.0611391	0.6979278	6.087011	0.1905196
cg27234823	0.0985897	0.0122274	0.794929	0.0295951
cg12647283	5.6786047	0.7419338	43.46284	0.0944251
cg19914554	2.4789497	0.6978552	8.805826	0.1604026
cg13934625	0.8396761	0.3768568	1.870886	0.6690241
cg02290550	0.1256467	0.040596	0.388883	0.0003201
cg06268921	1.2445099	0.6312764	2.45345	0.5276238
cg13724812	2.8290285	0.6340512	12.62264	0.1729278
cg03157179	0.7930381	0.34139	1.842202	0.589731
cg26648948	0.113784	0.0220721	0.586569	0.0093902
cg14322343	0.5000898	0.2037099	1.227676	0.1304551
cg04755571	1.7735665	0.8745739	3.596652	0.1121868
cg11637721	0.3203718	0.1075777	0.954083	0.0409155
cg02059082	0.2833342	0.1140707	0.703759	0.0065917
cg02710090	0.6847118	0.3356718	1.396692	0.2977067
cg19228034	0.35904	0.179106	0.71974	0.003892
cg04373487	0.6148715	0.067776	5.578181	0.6655554
cg02644610	0.0008203	4.72E-06	0.14254	0.0069283
cg15070710	4.7793992	0.5742254	39.77995	0.1479315
cg23551979	5.0360037	1.4024353	18.08378	0.0131941
cg04963424	2.3275598	1.0581803	5.11967	0.0356785
cg05016066	1.4880681	0.1789828	12.37184	0.7129998
cg04615850	0.253813	0.1026803	0.627394	0.0029819
cg18142730	1.5062932	0.1533934	14.79151	0.7252344
cg14602530	2.0166817	0.9247255	4.398067	0.077859
cg08665810	0.3171583	0.1300673	0.773364	0.011567
cg24425984	1.6820252	0.5445223	5.195763	0.3661804
cg15727308	8.2228858	1.1430117	59.15587	0.0363732
cg02232988	0.3263195	0.1624493	0.655493	0.0016507
cg21686600	1.2268643	0.5409682	2.782411	0.6245681
cg01655958	6.7749274	0.1135071	404.3767	0.359125
cg02107304	4.13E-12	4.16E-24	4.098364	0.0629026
cg13465831	6866.3262	5.71E-08	8.26E+14	0.4973505
cg03509094	1.5950155	0.3709735	6.857834	0.530394
cg07287401	12.21669	0.8734009	170.8809	0.0629703
cg23773983	5.477E+15	2.7809725	1.08E+31	0.0437072
cg21865762	1.4891004	0.633089	3.502541	0.3615506
cg09125623	4.6766376	1.0918754	20.03062	0.0376733
cg01455456	6.6354567	0.6635281	66.35632	0.1072192
cg04869380	0.258948	0.0438642	1.528674	0.135836
cg18195628	5.82E-27	5.79E-41	5.86E-13	0.0002405
cg02692390	2.4829269	0.8789737	7.013777	0.086073
cg07152216	2.3956143	1.1079632	5.179746	0.0263814
cg26849884	0.4305003	0.1719456	1.077843	0.0718801
cg13021015	0.647832	0.3263449	1.286021	0.2146367
cg01234420	1.4459666	0.7618799	2.74429	0.2592988
cg03461967	0.2202922	0.0433792	1.118708	0.0680517
cg11979621	0.7949972	0.373661	1.691428	0.5514632
cg00032610	4.3062667	1.6547184	11.2067	0.0027714

cg27024876	0.0307388	0.0013057	0.723677	0.0307238
cg11829608	0.4818679	0.0349998	6.634235	0.5852889
cg05117823	0.2072919	0.0561159	0.765735	0.0182591
cg12686477	2.5763892	0.8600893	7.717549	0.0908928
cg02471917	0.4393545	0.0385558	5.006568	0.5076574
cg21252552	1.171028	0.349346	3.925353	0.7980837
cg12437821	1.8515426	0.9626555	3.561201	0.0649042
cg20708909	1.4160339	0.5759692	3.481353	0.4485005
cg11092157	0.5641454	0.2215315	1.436636	0.2300267
cg17173767	0.2954719	0.0844315	1.034018	0.05644
cg10462820	0.1107805	0.0167411	0.733065	0.0224873
cg14434870	2.550706	0.8893945	7.315203	0.0815238
cg08250738	1.8280491	0.1506	22.18967	0.6357673
cg15471501	1.6685404	0.8010639	3.475412	0.1714771
cg05704960	9.37E-09	4.82E-19	182.4623	0.126205
cg08858051	0.1955155	0.0077838	4.910982	0.3210331
cg05269845	0.8906954	0.3311791	2.395496	0.8186241
cg16508600	0.1943394	6.37E-09	5926828	0.8522014
cg08695337	0.9675945	0.2449561	3.82207	0.9625133
cg10448719	0.1691297	0.0345589	0.827713	0.0282827
cg09826056	4.8516046	1.3397863	17.56852	0.0161506
cg22280148	1.2597922	0.5857216	2.709609	0.554498
cg01466133	21.905754	0.0016877	284332.4	0.5229704
cg02586712	3.2976458	0.3716618	29.25904	0.2840305
cg23240171	0.9725395	0.4401516	2.148881	0.9451184
cg26346621	0.2197784	0.0513091	0.941403	0.0412192
cg24665807	5.9901786	0.400274	89.6442	0.1947272
cg14042143	0.4004607	0.2064544	0.776776	0.0067846
cg25938530	1.6935389	0.4025694	7.124421	0.4723314
cg19473799	0.5559793	0.2741571	1.127503	0.1036743
cg02780643	102.94083	0.0016554	6401253	0.4105788
cg03100801	0.4159752	0.1559682	1.109427	0.0796902
cg24126849	1.264854	0.5937851	2.694334	0.5425383
cg16636054	0.1399952	0.0306143	0.640179	0.0112442
cg15393051	8.294E+12	4575.7026	1.50E+22	0.0062404
cg00419702	0.0210539	0.0004873	0.909663	0.0445117
cg17419096	0.4421684	0.1899641	1.02921	0.058335
cg08694107	0.4332365	0.1546931	1.213331	0.1113962
cg24094746	5.23E-10	1.04E-26	26248259	0.2760348
cg05992347	2.7436257	0.5108279	14.73585	0.2392875
cg01259329	1.4951268	0.6950599	3.216131	0.3033952
cg01296532	1.6174529	0.8100241	3.229724	0.1729375
cg00055617	1.1927663	0.6834441	2.08165	0.534993
cg12627844	0.4843287	0.1408797	1.665069	0.2498536
cg08689708	0.5682534	0.2952073	1.093848	0.0907407
cg26034658	0.5272001	0.1924393	1.4443	0.2131282
cg06138439	3.5784933	1.1049407	11.58941	0.0334699
cg01899937	0.1870187	0.0693061	0.50466	0.0009322
cg15460809	0.0934047	0.0088578	0.984948	0.0485431
cg22946648	0.2484865	0.0785625	0.785942	0.0177903
cg17083429	0.4129036	0.1793911	0.950378	0.0375601
cg07497190	1.0458179	0.0164936	66.31285	0.9831181
cg12834378	0.265063	0.1039597	0.675824	0.0054281
cg04498014	0.3152527	0.1042434	0.953387	0.0409036
cg12083024	0.0003016	2.90E-09	31.4156	0.1690814
cg24904739	0.4860456	0.2236379	1.056352	0.0685234
cg22203219	2.714817	0.1741967	42.30981	0.4759908
cg18326719	13.842475	0.2625094	729.9325	0.1939897

cg07287508	0.3048398	0.092906	1.000229	0.0500442
cg14114282	0.3308297	0.1347729	0.812094	0.0157681
cg22272840	2.146718	0.9805296	4.699907	0.0560324
cg25716623	1.0814299	0.4763579	2.455067	0.8515475
cg07145598	1.3865677	0.420914	4.567608	0.5910423
cg10100318	0.3645172	0.0005607	236.9946	0.7600831
cg17285177	4.4493163	0.8636695	22.92129	0.074305
cg14644418	0.4156973	0.1727052	1.000573	0.0501496
cg27633010	0.0402669	0.006685	0.242545	0.0004546
cg04510788	0.1090599	0.0216407	0.549615	0.0072464
cg14515187	0.7289827	0.3534079	1.503689	0.3921598
cg07700266	1.01E-21	7.32E-48	138203.4	0.1153719
cg17272642	1.4057278	0.371901	5.313432	0.6156807
cg05129050	0.1327626	0.0312953	0.563212	0.00617
cg13705888	0.3950315	0.1819595	0.857607	0.0188569
cg04592201	1.4495898	0.6620021	3.174175	0.3531689
cg15133719	0.1164515	0.0104566	1.296874	0.0803651
cg15242223	2.3444202	0.5158613	10.65462	0.2700067
cg18404811	3.824287	1.0628451	13.7604	0.0400474
cg11083715	2.8575245	0.7316973	11.1596	0.1309057
cg16905506	0.4420246	0.2105699	0.92789	0.0309451
cg01073765	0.4022311	0.1975445	0.819004	0.0120622
cg00251716	0.2060835	0.0891523	0.476381	0.0002204
cg15084160	0.2071562	0.0560572	0.765534	0.0182456
cg22949256	2.7400719	0.5256838	14.28234	0.2314675
cg26909981	0.6419739	0.2961476	1.391639	0.2615377
cg21418854	0.3883317	0.1488861	1.012865	0.0531341
cg12542670	0.599329	0.2164254	1.659673	0.3245727
cg02906074	1.1349764	0.5659133	2.27627	0.7214049
cg21406144	0.6308952	0.2551603	1.559916	0.3186262
cg19498794	4.4983291	0.3000229	67.44473	0.276377
cg18557145	6.1245143	0.921647	40.69853	0.0607206
cg12065777	0.2691308	0.0543815	1.331912	0.1076853
cg20645040	0.4706134	0.1578849	1.402775	0.1761862
cg21447871	1.182807	0.5227471	2.676308	0.6869568
cg02752105	0.5455545	0.2039426	1.45938	0.2274318
cg10529401	1.6941559	0.5208907	5.510109	0.3809799
cg09874992	2.0839214	0.5445959	7.974221	0.2835452
cg10895168	0.4681965	0.1037239	2.113379	0.3237108
cg16385763	0.34348	0.1491028	0.791257	0.0120776
cg00085434	1.8741549	0.8171755	4.298289	0.1380139
cg12613129	1.3023584	0.6917559	2.451931	0.4131503
cg20474675	0.3217427	0.1163813	0.889476	0.0288369
cg06636938	0.821211	0.3472122	1.942292	0.6538127
cg10701252	7.5189648	1.4595371	38.73477	0.0158633
cg16531386	1.8513412	0.7372919	4.648721	0.1898033
cg18505752	3.1630715	0.7932096	12.61334	0.102743
cg26151910	41.220696	3.5635871	476.8077	0.0029078
cg26122129	2.1130298	0.2328289	19.17672	0.5061703
cg00502533	8.687E+12	35.243323	2.14E+24	0.0260048
cg03153775	1.8284525	0.3002886	11.13342	0.5126338
cg21064451	0.1657225	0.0336827	0.815371	0.0270329
cg07378490	3.1216219	1.0523875	9.259444	0.0401689
cg02086431	0.4342117	0.1488831	1.266362	0.1266239
cg19856499	1.2917664	0.5769408	2.892256	0.5335967
cg25560696	1.4572005	0.7451781	2.849565	0.2711724
cg19078576	0.0803099	0.0128484	0.501983	0.0069962
cg08508745	6.0303755	0.1281986	283.6647	0.3604601

cg13219301	0.5734247	0.2712135	1.212388	0.1454461
cg07211474	2.8331735	0.8999599	8.919144	0.0751055
cg21505334	0.4755517	0.2084661	1.084826	0.077318
cg26876077	3.2478314	0.8180036	12.89531	0.0940487
cg22966895	0.0618555	0.0062486	0.612318	0.0173443
cg06053959	0.3141981	0.1245616	0.792543	0.0141866
cg02120584	5.6124248	1.7406829	18.09595	0.003878
cg02707307	0.4534765	0.1935108	1.062684	0.0687532
cg11425656	4.5688464	0.1163374	179.4295	0.4172243
cg08308477	0.421812	0.1538546	1.156451	0.0934473
cg26647197	1.1464648	0.5715634	2.299625	0.7003335
cg04234016	1.7796863	0.6191996	5.115126	0.2845649
cg13939204	1.6005661	0.559281	4.580545	0.3806144
cg06333307	0.1593144	0.0061874	4.102086	0.2677269
cg07130639	1.859911	0.8471884	4.083235	0.1219511
cg01709316	63.632562	2.0775171	1949.011	0.0173713
cg14879645	0.2414923	0.0499497	1.167546	0.0771782
cg16013223	1.8511333	0.8723381	3.928173	0.1086759
cg02611848	3.9411532	0.9972569	15.57541	0.0504597
cg01294808	1.8212181	0.6358091	5.216716	0.2641892
cg25727853	2.8759573	0.4489161	18.42467	0.2649467
cg17891251	0.8948437	0.4513963	1.773929	0.7503122
cg03752628	0.1443688	0.0360909	0.577496	0.0062152
cg00867406	1.2246708	0.0752435	19.93287	0.8867706
cg17491456	0.2921895	0.0594529	1.436006	0.129894
cg25141674	1.3242751	0.4031913	4.349559	0.6434356
cg18188717	0.2013636	0.0329139	1.231923	0.0828722
cg18473335	0.4497011	0.2117434	0.955076	0.0375651
cg07507518	0.4718704	0.1880356	1.184146	0.1096187
cg05375744	1.9227524	0.0225761	163.7564	0.7731245
cg13261971	2.7298822	1.2369848	6.024534	0.0128987
cg03673688	2.018905	0.5862999	6.952034	0.265437
cg21779805	0.7992896	0.3409469	1.873793	0.6062937
cg20499941	0.315081	0.1369617	0.724845	0.0065876
cg15621656	1.216967	0.495885	2.986597	0.6681524
cg05383931	0.5317133	0.296098	0.954816	0.03445
cg14596312	0.5440918	0.2246713	1.31764	0.1774298
cg21578541	1.6783474	0.4550814	6.189772	0.4367817
cg01441865	5.263563	0.6952682	39.84807	0.1078238
cg19702383	0.5666464	0.2966946	1.082218	0.0853193
cg16587832	0.530059	0.2684569	1.046583	0.0674314
cg17934743	1.83E-06	4.20E-37	7.95E+24	0.7135577
cg15609035	1.2977	0.5697423	2.955767	0.5349445
cg04586299	0.1933521	0.054595	0.68477	0.0108694
cg09549591	1.6738784	0.6900689	4.060274	0.2545214
cg22525688	0.2559903	0.0999043	0.655938	0.0045348
cg00950086	0.3295102	0.1418167	0.765615	0.0098555
cg24059119	1.2725996	0.6149728	2.633466	0.5158991
cg05599160	1.8216839	0.7528344	4.408051	0.1834334
cg05622577	0.5562112	0.2482848	1.246033	0.1540267
cg14844588	0.9165011	0.4406566	1.906188	0.8154774
cg01937780	2.5351327	1.1289481	5.69282	0.0242076
cg01201512	3.1276927	1.2213102	8.009809	0.0174704
cg24673600	5.3130061	0.993193	28.4215	0.0509404
cg01922697	0.3100022	0.1106393	0.868601	0.0258841
cg04407929	1.83E-06	3.61E-15	930.1634	0.1964927
cg21205865	0.1641213	0.0378499	0.711648	0.0157588
cg22335340	1.7875899	0.1907579	16.75148	0.6108984

cg01358993	1.2523399	0.5982263	2.621675	0.5505478
cg19228848	2.09E-06	6.08E-12	0.717132	0.0443161
cg01158822	0.1805185	0.007952	4.097965	0.2825594
cg08534016	0.7449263	0.3530042	1.571979	0.4396254
cg15495091	11.556453	1.7654499	75.64734	0.0106826
cg07152817	0.5509797	0.2754927	1.101948	0.0919008
cg09368199	0.8800883	0.4673681	1.65727	0.6924292
cg01087594	3.39E-06	9.67E-23	1.19E+11	0.5170101
cg22806229	0.126574	0.0026458	6.055158	0.2949234
cg21109744	2.1284482	0.8870571	5.107103	0.0907247
cg09979924	1.4450101	0.6365499	3.280268	0.3788177
cg24684077	1.6101904	0.8344555	3.107072	0.155507
cg03389720	0.1313413	0.0363611	0.474422	0.0019489
cg03182917	0.4012509	0.0934121	1.723569	0.2194762
cg19286686	0.0287048	0.0008588	0.959446	0.0473578
cg23817297	0.4420882	0.1482777	1.318081	0.1430674
cg01136750	1.35E+20	8.71E-05	2.08E+44	0.1028936
cg15597069	9.9943119	0.7928296	125.9871	0.0750076
cg10062945	0.404487	0.1940972	0.842927	0.0156886
cg06771126	1.6216772	0.766881	3.429263	0.2057627
cg18597188	0.3660264	0.1465231	0.914363	0.0314268
cg04835383	0.3844977	0.13788	1.072225	0.0677465
cg27326999	3.5113606	1.0345687	11.91767	0.0439601
cg01751181	0.6955574	0.3621716	1.335831	0.2755649
cg05886546	2.0631109	0.7029491	6.055099	0.1873907
cg14201424	2.6695165	1.2530342	5.68725	0.0109433
cg00095523	3.9162498	1.3659492	11.2281	0.0110771
cg03177593	0.045084	0.006444	0.31542	0.0017934
cg11198596	1.7549144	0.7668449	4.016098	0.1830299
cg25344064	0.9238723	0.4240445	2.012855	0.8420373
cg22910449	2.0083911	0.8082884	4.990341	0.1331897
cg22979422	0.0442599	0.0037949	0.516204	0.0128618
cg02149189	2.5486964	1.0677212	6.083848	0.0350678
cg12656391	0.3790291	0.0674433	2.130132	0.2707055
cg05239940	0.5263216	0.2540806	1.090262	0.0840981
cg12033075	2.7285058	0.7854648	9.478139	0.114134
cg01856892	0.1848085	0.0633523	0.539115	0.0019947
cg16175077	2.093766	0.4676447	9.374329	0.3339443
cg02260098	0.1096568	0.0246599	0.487617	0.003692
cg15916160	2.2829366	0.9642455	5.405054	0.0604953
cg23217463	3.6621764	0.6337132	21.16342	0.1469739
cg10038185	3.7821698	0.8427059	16.97485	0.0824644
cg11724750	1.7515291	0.5532286	5.545365	0.3404883
cg24100671	0.5201255	0.2107456	1.283683	0.1561417
cg05535809	0.2527939	0.1080005	0.591708	0.001528
cg22426627	0.0067205	9.81E-05	0.460215	0.0203493
cg02068596	0.580434	0.298249	1.129605	0.1093244
cg00371355	0.042977	3.18E-05	58.14807	0.3922776
cg13406893	1.8191179	0.813168	4.069503	0.145248
cg23792364	6.49E+20	0.0099345	4.24E+43	0.0737912
cg04800347	0.2572574	0.0008046	82.25385	0.6445264
cg11994674	1.0108874	0.4713757	2.167895	0.9778069
cg00354484	0.6354921	0.2814999	1.434637	0.2751676
cg27056270	0.0051461	0.0002747	0.096405	0.0004242
cg27649253	24.364402	0.6580343	902.1172	0.0831219
cg26332926	1.2846084	0.6613079	2.495387	0.4597311
cg10141174	6.5720188	1.4398109	29.99799	0.0150777
cg08438690	1.4063177	0.6673998	2.963336	0.369914

cg11072119	0.9633735	0.4051969	2.290463	0.9327035
cg05375686	0.4579024	0.1613201	1.299743	0.1422573
cg26650480	2.6782921	0.621431	11.54311	0.1862609
cg27356513	3.2076714	1.2029395	8.553345	0.0198484
cg02772159	0.1407381	0.0340593	0.581551	0.0067532
cg08889797	1.6730545	0.857095	3.265812	0.1315315
cg01916088	0.7075597	0.4170602	1.200404	0.1996023
cg00014333	2.1235279	0.5895682	7.648599	0.2493903
cg07901253	1.3505475	0.5022642	3.631512	0.5515386
cg09171562	0.4483598	0.1408959	1.426774	0.1744044
cg03724721	0.498208	0.2575582	0.963709	0.0384733
cg06517181	1.7095225	0.7943997	3.678837	0.1702734
cg17961381	1.9829196	0.851824	4.615942	0.1122972
cg06943467	0.413042	0.2021699	0.843863	0.0152793
cg02092102	2.0764085	0.827989	5.207161	0.1193338
cg23754665	6.56E-33	7.30E-66	5.894334	0.0556021
cg25782440	0.0903222	0.0200684	0.406515	0.0017314
cg04361126	3.0611729	1.0429912	8.984524	0.0416915
cg07780630	1.243321	0.4929016	3.136218	0.6445497
cg17460713	1.2918426	0.5954768	2.802556	0.5169553
cg25403283	0.610884	0.2560083	1.457684	0.2667014
cg17573301	1.892719	0.9250535	3.872625	0.0806915
cg04872123	1.339996	0.3384057	5.306026	0.6768111
cg13345957	1.9329254	0.9110692	4.100896	0.085929
cg12410980	1.9579223	0.6235991	6.147314	0.249742
cg08695416	0.2562793	0.0948152	0.692706	0.0072821
cg23102483	1172.8885	0.0212788	64649686	0.204523
cg06879786	2.0875639	0.470509	9.262145	0.3329541
cg05385010	5.9696015	0.6196591	57.50927	0.1221336
cg12340144	0.2211637	0.0432555	1.130802	0.0699378
cg23609571	0.0005168	9.67E-07	0.276276	0.018209
cg06933777	0.5768289	0.1517452	2.192698	0.4193345
cg23139801	1.208E+18	1.39E-11	1.05E+47	0.2206864
cg25928188	0.4079679	0.0218804	7.606719	0.5480782
cg18368297	0.0602016	0.0069699	0.519985	0.0106362
cg02933962	2.3557325	0.6141362	9.036229	0.2115957
cg24584721	1.0304807	0.159035	6.677088	0.9748768
cg01909856	0.352272	0.1269338	0.97764	0.0451359
cg17588455	0.2735623	0.1126362	0.664407	0.0041961
cg03355370	0.3410973	0.1636866	0.710793	0.0040885
cg01165402	0.3637801	0.1715243	0.771529	0.0083851
cg11447849	2.4715562	0.8771536	6.964105	0.0869016
cg01628509	0.0748196	0.0164078	0.341177	0.000811
cg10179004	0.1483355	0.0512191	0.429594	0.000436
cg24434118	0.7162058	0.309662	1.656486	0.4352556
cg15899800	0.3849463	0.1738496	0.852367	0.0185816
cg10892403	0.068342	0.015069	0.309949	0.0005043
cg02081006	1.3180825	0.7142927	2.432254	0.3769387
cg13692739	3.5089878	0.7518096	16.37781	0.1102572
cg26159832	0.2399413	0.1137936	0.505932	0.0001768
cg26575057	1.5392241	0.6652886	3.561178	0.3135878
cg05716556	0.8617435	0.3159155	2.350635	0.7713376
cg09048186	0.0053808	1.04E-08	2787.461	0.4363954
cg12131620	3.2912891	0.8336286	12.9945	0.0890836
cg20630887	0.4979204	0.1841267	1.346489	0.169493
cg25221168	0.0004269	5.23E-07	0.34877	0.0233386
cg09784977	2.5325119	0.9048825	7.08779	0.0767911
cg07986257	0.8671642	0.4289964	1.752867	0.6914229

cg19128261	0.4786665	0.1901342	1.205052	0.1178162
cg01372811	3.2852625	1.3772953	7.836337	0.0073248
cg16309350	1.5486115	0.6323799	3.792337	0.3385134
cg17719360	0.5584077	0.2529248	1.232854	0.1493223
cg22450878	0.5339995	0.2730966	1.044156	0.0667028
cg04849878	5.5012862	1.0228671	29.58757	0.0469996
cg05146544	2.6918289	1.123381	6.450121	0.0263576
cg01501312	2.0275992	0.9619392	4.273824	0.0631737
cg18462381	0.2318315	0.0735625	0.730615	0.0125643
cg10323725	2.5595743	0.9987822	6.559409	0.0502974
cg27614319	1.3932119	0.7311777	2.654675	0.3133957
cg14557185	0.2337511	0.0663804	0.823129	0.023635
cg26554170	0.0390998	0.0019145	0.798538	0.0351929
cg09659223	1.393435	0.2945815	6.591252	0.6756174
cg12324629	0.2820243	0.112045	0.709873	0.0071981
cg01035261	1.1849503	0.5790899	2.424679	0.6422633
cg24332710	0.1873673	0.04648	0.755303	0.0185464
cg18759102	1.6776075	0.8716744	3.22869	0.1214253
cg16920551	1.6222018	0.7482217	3.517058	0.2204557
cg08450752	1.8934557	0.9799006	3.658712	0.0574922
cg09557991	1.3508227	0.7007722	2.603873	0.3691504
cg26569914	4.4701958	1.1912669	16.77429	0.0264623
cg03979378	1.987262	0.8279449	4.769895	0.1242164
cg11762760	2.4351371	0.9251711	6.409509	0.0714747
cg26115312	1.4015897	0.6534077	3.006475	0.3859153
cg04826413	5.9578207	0.1921368	184.7414	0.3084167
cg25254338	1.7344716	0.6678642	4.504496	0.2580721
cg20618448	0.5116385	0.0999675	2.618591	0.4211514
cg04167518	2.4416969	0.4956678	12.02798	0.2725226
cg20876760	6.5250017	1.582753	26.89974	0.0094506
cg22828884	0.7594113	0.3564573	1.617881	0.4757291
cg05163268	1.8428177	0.7908624	4.294018	0.1566769
cg06511701	0.54295	0.2719956	1.083822	0.0833221
cg15787985	0.2063583	0.0635912	0.669648	0.0085979
cg13537774	3.3413967	0.0745331	149.7984	0.534102
cg21658333	1.82E-16	1.49E-27	2.24E-05	0.0054034
cg13401079	1.1375955	0.4647563	2.784521	0.7777401
cg05024762	1.7714297	0.1723169	18.21042	0.6305616
cg16949914	1.9415544	0.9034548	4.172465	0.0891595
cg12877165	1.481173	0.5187567	4.229099	0.4630291
cg21901223	1.6724785	0.6807753	4.108822	0.2620822
cg12569835	0.2281	0.0695435	0.748159	0.01474
cg23227837	0.2933782	0.0065034	13.23471	0.5280534
cg13295050	4.0558957	0.5352165	30.73577	0.1754074
cg05638594	2.60993	0.9349074	7.285999	0.0670311
cg13696605	11.743992	0.2989815	461.304	0.1884119
cg27479845	1.55E-30	1.20E-44	2.01E-16	3.47E-05
cg08234653	0.4502147	0.1265051	1.602255	0.2179025
cg21052873	1.3737649	0.5398071	3.496119	0.5052148
cg06640718	0.686757	0.3414318	1.381345	0.2919253
cg05141059	0.4231994	0.0743616	2.408472	0.332431
cg10256336	1.0488979	0.3830673	2.872046	0.9259894
cg06381964	0.0816164	0.0032596	2.043593	0.1272616
cg13320146	1.5167737	0.6200181	3.710541	0.3614013
cg12427317	1.2489179	0.3785197	4.120779	0.715154
cg07570723	0.0140917	0.0007616	0.260738	0.0041981
cg22212691	0.1454389	0.032335	0.654166	0.0119653
cg18050903	1.8165673	0.7223739	4.568156	0.2045279

cg04253077	1.0826354	0.3971056	2.951606	0.8766954
cg03826252	1.2932024	0.3300718	5.066692	0.7120977
cg19831369	0.7127199	0.3431815	1.480178	0.3637468
cg04804094	9.8173391	0.7659272	125.8346	0.0792482
cg09935667	1.2457926	0.3762355	4.125074	0.7190265
cg22490254	1.0566679	0.4210453	2.651846	0.9065342
cg06453916	0.171769	0.0485043	0.608287	0.0063242
cg07207490	0.190373	0.0515908	0.702487	0.0127721
cg03222834	0.3183322	0.0729218	1.389644	0.1279226
cg01100525	0.0003952	3.45E-08	4.526338	0.1003162
cg15618264	2.98E-14	2.77E-33	320210.2	0.163625
cg09900585	3.60E-26	8.81E-53	14.6675	0.0609225
cg21923317	0.4922416	0.166159	1.458253	0.2008423
cg07614018	1.1729926	0.5057178	2.72071	0.7101124
cg13324311	0.3841415	0.1203484	1.226146	0.1061647
cg15384589	0.325493	0.142478	0.743593	0.007749
cg10246473	0.2321502	0.0433116	1.244327	0.0882346
cg12046837	0.1763007	0.0577495	0.538219	0.0023048
cg00443516	2.82785	0.6082228	13.14771	0.1849021
cg12147198	0.43598	0.1884388	1.008702	0.0524138
cg25009965	0.1628624	0.0472273	0.561628	0.0040612
cg00461735	0.2531821	1.30E-10	4.95E+08	0.8998531
cg04302194	2.1568933	0.9789265	4.752337	0.056505
cg08351710	0.1311028	0.0371358	0.462841	0.0015942
cg18904855	1.3792548	0.3325397	5.720651	0.6577513
cg08079763	1.9634951	0.378439	10.18741	0.4218489
cg16451306	0.3322753	0.1422632	0.776075	0.0109062
cg08882547	0.5812046	0.2757884	1.224848	0.1536591
cg10763638	2.13E-10	4.08E-18	0.011084	0.0140265
cg13250203	5.8514905	1.4951676	22.9004	0.0111566
cg27539060	1.6070011	0.8308472	3.108216	0.1587188
cg04073840	0.0022273	1.55E-05	0.319364	0.0159308
cg01334824	4.7972297	0.7749475	29.69674	0.091825
cg13887966	1.2790648	0.5182779	3.156621	0.593339
cg18333511	0.8704387	0.3922266	1.931698	0.7329809
cg23854103	0.0091392	0.0001779	0.469602	0.0194892
cg09407339	0.7385825	6.81E-06	80135.71	0.9591473
cg04045079	0.0418951	0.0064327	0.272855	0.000905
cg10157839	17448520	2.169126	1.4E+14	0.0398393
cg10818702	0.6242479	0.0109374	35.62885	0.8193703
cg07447854	1.8359456	0.3426938	9.835882	0.4780456
cg21341821	0.9701958	0.0220382	42.7113	0.9874983
cg24353213	2.9629693	0.5277563	16.63493	0.2172329
cg09371744	4983.4577	0.8173516	30384536	0.0555424
cg20189244	2.289247	0.8303347	6.311493	0.1094568
cg18056303	0.1212782	0.0182404	0.806366	0.0290635
cg06381463	0.2106315	0.0847919	0.523229	0.0007931
cg18346038	636.20938	12.787361	31653.32	0.0012021
cg10243939	1.4131877	0.6122617	3.26184	0.4177132
cg12112058	567493.88	0.0006721	4.79E+14	0.2064533
cg16505953	0.3116954	0.1252931	0.775414	0.0121768
cg19764325	0.0001551	5.70E-13	42209.53	0.3760602
cg04234238	4.398E+10	65141.178	2.97E+16	0.0003456
cg04821993	1.2804128	0.6366027	2.575322	0.4881246
cg05927190	2.220214	0.8490915	5.80544	0.1038664
cg25383057	11.938403	1.5914013	89.55973	0.0158715
cg10910512	0.3718799	0.1614759	0.856441	0.0201222
cg08920174	2.2064541	1.0355615	4.701256	0.0403153

cg24917382	0.2261702	0.0783088	0.653221	0.0060164
cg02475600	0.365331	0.1095556	1.218256	0.1012784
cg08589214	0.2194601	0.0065536	7.34905	0.3972329
cg17691988	0.3905552	0.15313	0.996104	0.0490523
cg16096631	0.3608549	0.1234244	1.055028	0.0625887
cg16467015	12.32943	0.0645215	2356.034	0.3486033
cg15244965	0.4253086	0.1674231	1.080421	0.0722802
cg13677735	0.0020907	1.65E-06	2.65376	0.0905913
cg13521229	0.2693285	0.0969823	0.747949	0.0118275
cg15882726	68.284454	0.0042203	1104852	0.3930084
cg08541862	2.8020454	0.3144433	24.96939	0.3558716
cg01825818	1.7899171	0.6924669	4.626651	0.2295534
cg00095859	1.4020069	0.5961461	3.297218	0.4386706
cg02721902	2.944024	0.9534137	9.090784	0.0605135
cg22021934	0.3818683	0.1574872	0.925938	0.0331522
cg13883603	0.1953638	0.0669923	0.569722	0.0027876
cg26833652	2.3144763	0.8375307	6.395945	0.1056407
cg00233028	0.1723754	0.0693695	0.428333	0.0001533
cg00114160	2.0657549	0.9123015	4.677558	0.0818853
cg16508068	1.8029607	0.6892228	4.716424	0.2296079
cg05963087	0.4004558	0.1582068	1.013641	0.0534372
cg07478918	2.3700796	0.711165	7.898697	0.1600222
cg07016075	1.1495131	0.460186	2.871405	0.765461
cg09185998	1.189928	0.4878771	2.902224	0.702263
cg20445197	0.7045412	0.2827898	1.755291	0.4520918
cg16967296	3.7783111	0.4442089	32.13721	0.2235939
cg23440520	2.1438417	0.9804882	4.687519	0.0560565
cg23582644	0.6132527	0.288895	1.301784	0.2029356
cg12629796	0.2280138	0.0545358	0.953324	0.0428207
cg09737197	1.5103886	0.8277921	2.755853	0.1789504
cg06972043	0.2899489	0.1397348	0.601642	0.0008867
cg26430984	1.7261485	0.4733415	6.294796	0.4082669
cg02569458	0.585874	0.2638348	1.300998	0.1890106
cg12067997	2.2470517	0.8773427	5.755153	0.0915543
cg14685146	0.4555047	0.1937347	1.070973	0.0714246
cg05815196	0.9827555	0.4316006	2.237736	0.9669509
cg18540157	1.1096214	0.5233183	2.352793	0.7861926
cg11825302	0.6919998	0.3353797	1.427826	0.3191333
cg09980771	0.2148418	0.0720038	0.641036	0.0058297
cg26009832	2.011965	0.3203393	12.63661	0.455842
cg24880022	2.3084083	0.8697087	6.12705	0.0930204
cg21156590	0.1421136	0.0452544	0.446283	0.0008323
cg13434852	33.164024	0.0001881	5846602	0.5699585
cg22117825	1.5573111	0.8160551	2.97188	0.1791244
cg21637865	1.260062	0.3927643	4.042516	0.6975254
cg06141846	2.5370301	1.2345791	5.213535	0.0112963
cg13130319	1.0979925	0.4888448	2.466197	0.8208683
cg25245161	1.1819634	0.3508961	3.981343	0.7873114
cg02067022	0.8014067	0.3500176	1.834916	0.6004162
cg06526741	1.5345611	0.4861108	4.844322	0.4653037
cg07075347	0.4996074	0.2471727	1.009851	0.0532778
cg00395632	5.2545859	0.0385474	716.2782	0.5082229
cg13962943	0.0161431	6.02E-07	433.2098	0.4277365
cg13554018	1.2029241	0.4256321	3.399712	0.7274323
cg26066597	0.0018612	2.54E-07	13.65693	0.1662657
cg15910070	0.516339	0.2660459	1.002105	0.050731
cg25889160	0.7748596	0.2748488	2.184501	0.6295588
cg18093866	0.144058	0.036723	0.565115	0.0054633

cg18822950	1.5862006	0.7730007	3.25489	0.2084213
cg02923021	1.3671014	0.5420678	3.447846	0.5076396
cg05525649	0.3622888	0.1340643	0.979032	0.0453124
cg13232821	0.3817426	0.1141284	1.276872	0.1180017
cg11756870	1.8861972	0.8377734	4.246661	0.1254026
cg09933323	0.2402809	0.1168056	0.494282	0.0001068
cg04777612	0.3505987	0.1536626	0.799931	0.012761
cg03671075	0.2776108	0.1124056	0.685622	0.0054665
cg11028091	2056.1213	10.629429	397729.2	0.0045133
cg26208507	0.3278398	0.1075316	0.99951	0.0498994
cg07958387	1.2900597	0.5227491	3.183657	0.5805423
cg21875401	0.6614296	0.2919018	1.498755	0.3219662
cg19432886	0.4154391	0.2151775	0.80208	0.0088699
cg07259732	0.0062842	1.13E-07	349.9539	0.3631887
cg01145396	1.4710439	0.4400028	4.918082	0.5308024
cg15933527	3899.0078	3.90E-10	3.9E+16	0.588239
cg14590070	1.5740727	0.8089354	3.062921	0.1816518
cg13910174	0.3930732	0.1559076	0.991013	0.0478051
cg02716128	3.0937512	1.41367	6.770531	0.0047088
cg12167518	0.5088059	0.2671073	0.969211	0.0398715
cg02652998	0.995325	0.4715871	2.100719	0.9901899
cg26993496	0.3365525	0.1011576	1.119714	0.075799
cg10485000	3536972	3.17E-12	3.94E+24	0.4769658
cg00173141	0.9219675	0.4922616	1.726773	0.7996767
cg04949153	0.6009243	0.2615783	1.380505	0.2300926
cg20978721	1.9312117	0.9475352	3.936084	0.0700445
cg07889826	501928569	252.33907	9.98E+14	0.0067814
cg16108230	2.098059	0.934079	4.712505	0.0726869
cg26760212	0.4253076	0.1479675	1.222475	0.1124975
cg19314660	2.3081042	0.906216	5.87867	0.0795141
cg21665850	1.4469162	0.4447224	4.70758	0.5393731
cg13278478	0.3163903	0.1000713	1.000315	0.0500627
cg27557317	2.47E-17	1.40E-32	0.043582	0.0327711
cg19981982	0.3030364	0.0790973	1.160989	0.081483
cg20200460	0.367297	0.1339189	1.007379	0.051693
cg13050240	1.5863172	0.7544929	3.335224	0.2236173
cg10139717	0.5866496	0.2800421	1.22895	0.157494
cg23982890	0.4833892	0.1915481	1.219877	0.1237682
cg25769590	2.1646814	0.7204457	6.504093	0.1688752
cg08408668	0.1749061	0.0451459	0.677628	0.0116313
cg15773755	2.5028032	0.1448508	43.24466	0.5280216
cg10232395	1.6664921	0.4519519	6.144892	0.4430196
cg05306395	0.0002259	8.11E-07	0.062914	0.0034674
cg16301617	2.3297463	0.7886807	6.882022	0.1259177
cg03372974	1.3425671	0.5051171	3.568452	0.5547659
cg23197405	1.0124619	0.3090846	3.3165	0.983678
cg13158604	1.4241471	0.521474	3.889351	0.4903388
cg22122342	1795.0362	4.6218785	697152.7	0.0137701
cg20172636	0.9709094	0.4008159	2.351865	0.9478548
cg09293534	0.3790884	0.1679205	0.85581	0.0195564
cg21398469	0.3332597	0.1765779	0.628969	0.000697
cg25092915	0.2965961	0.0794255	1.107568	0.0706092
cg26338428	0.5130128	0.2438767	1.079161	0.0785484
cg06716730	0.285989	0.0967771	0.845135	0.0235545
cg10479431	0.2811579	0.1091223	0.724415	0.0085992
cg07408779	8.8887788	1.6312138	48.43656	0.0115493
cg10688297	4.3356607	0.2494621	75.35394	0.3139834
cg16775752	0.67947	0.2788177	1.655847	0.3951557

cg17191462	1.1414466	0.5495097	2.371023	0.7228128
cg20043258	0.9079111	0.0063811	129.1796	0.9695344
cg10040131	2.3868459	0.291843	19.52088	0.4171501
cg25337124	1.4337119	0.6509501	3.157738	0.3711747
cg00633552	1.6197965	0.659683	3.977275	0.2926546
cg24688939	2.7181702	0.7280404	10.14841	0.1368199
cg11655130	1.2915513	0.7231279	2.306791	0.3872908
cg03199263	0.3089218	0.1535345	0.621572	0.0009914
cg26279025	0.6743226	0.3209017	1.416979	0.2983144
cg02298193	37.519937	0.4826905	2916.456	0.1026741
cg26763618	2.09E-18	2.65E-33	0.001649	0.0200135
cg19514381	2.9466117	0.9992691	8.688871	0.0501551
cg19925204	1.6439157	0.8919251	3.029917	0.1110815
cg16332159	2.5940778	0.9294537	7.239995	0.068719
cg16546658	2.4820436	0.8889772	6.929919	0.082684
cg19108055	0.042789	0.0046803	0.391189	0.0052506
cg26002659	16990.564	1.90E-08	1.52E+16	0.4878934
cg11857320	2.426425	0.995871	5.911949	0.0510739
cg21291771	2.3155241	0.7394637	7.25073	0.1493858
cg23299109	4.1124211	1.1739596	14.40595	0.0270559
cg13777717	2.9945024	1.2075464	7.425839	0.0179348
cg10683775	0.4887623	0.2093978	1.140836	0.0978641
cg26134090	9.189022	1.3786122	61.24864	0.0219224
cg22989033	0.3617734	0.128436	1.019029	0.0543184
cg02898904	1.5799136	0.6942402	3.59548	0.2756521
cg26346210	2.8305188	0.7437438	10.77231	0.1270589
cg03292675	2.633164	0.8609707	8.053181	0.089601
cg03330484	0.5327651	0.2678673	1.059624	0.0726727
cg04940089	0.0308824	0.0003516	2.71248	0.1277684
cg22633111	0.3993988	0.1913409	0.833692	0.0145092
cg03553434	0.3440565	0.1677377	0.705714	0.0036042
cg13895393	1.2439632	0.2928373	5.284315	0.7673787
cg20887073	2.9199226	0.7805968	10.92235	0.1113912
cg13035268	2.5591261	1.3143432	4.982813	0.0057103
cg26823535	3.9332043	0.6084839	25.424	0.1503688
cg13571700	0.4539458	0.1759304	1.171297	0.1024619
cg03904104	0.1216481	0.015088	0.980799	0.0479096
cg14483935	0.3919549	0.1559812	0.984918	0.0463393
cg10231049	1.2850973	0.4647909	3.553157	0.6288064
cg06795634	1.2415956	0.533477	2.889646	0.6156065
cg10110652	5.36162	0.4185193	68.68732	0.1968579
cg05838289	0.6619266	0.2759167	1.587968	0.3554085
cg01781374	0.5903279	0.3010633	1.157521	0.1249853
cg17809595	0.1951792	0.0632581	0.602214	0.0044807
cg20459495	0.4215763	0.064727	2.745788	0.3662807
cg04484967	4.9571257	1.3792427	17.81637	0.0141837
cg17410135	3.6979056	0.9760646	14.00984	0.054315
cg24457403	3.3556144	1.2316447	9.142367	0.0179139
cg04306135	0.5202985	0.093211	2.904277	0.4564502
cg15588215	2.9896789	0.8775816	10.18501	0.0799185
cg14174719	0.1366379	0.0036497	5.115479	0.2815402
cg06747009	27.528675	0.0039346	192604.4	0.4629828
cg10266211	4.2887108	1.0861721	16.93382	0.0377155
cg10059178	1.1804478	0.4700958	2.964198	0.7239789
cg09101235	0.7349546	0.1073529	5.031614	0.7537081
cg14591340	0.3239766	0.1391572	0.754261	0.0089477
cg13149276	1802692.5	2.2522747	1.44E+12	0.037798
cg11052143	0.5061713	0.2492291	1.028007	0.0596257

cg12494373	0.6741807	0.3450021	1.31744	0.248739
cg24635903	1732.7864	1.44E-29	2.08E+35	0.8431447
cg05755899	0.2112894	0.0272513	1.638202	0.1368533
cg00648919	0.0211256	1.27E-06	350.0694	0.4364764
cg10623043	39919.427	71.520394	22281207	0.0010263
cg03192598	0.7846258	0.421746	1.459736	0.4438198
cg16896911	2.0540052	0.5388029	7.830206	0.2917783
cg23750206	1.0709621	0.5189457	2.210173	0.8528664
cg13617753	1.9913226	0.7390823	5.365256	0.1731719
cg16705097	0.2402642	0.0982915	0.587303	0.0017659
cg07583394	2.968E+13	0.1568805	5.61E+27	0.0643814
cg04542080	2.8168091	0.8857879	8.957464	0.0793452
cg03225093	0.3478708	0.1044407	1.158687	0.085425
cg10230314	0.5366352	0.1645911	1.749653	0.3019612
cg11847992	0.3137368	0.1489254	0.66094	0.0022944
cg00834536	1.1536014	0.4063328	3.275138	0.7883996
cg27179622	1.8624594	0.3766984	9.208308	0.4456633
cg14617041	1.1904531	0.5788319	2.448342	0.6356011
cg20580294	2.6503141	0.990612	7.090732	0.0522366
cg01473602	0.2069099	0.074038	0.57824	0.0026591
cg25345422	0.4991768	0.2256326	1.10435	0.0863513
cg01968657	1.4320251	0.5937651	3.453716	0.4240309
cg26050906	4.4718708	0.699147	28.6029	0.1136584
cg03179450	0.1941116	0.0794942	0.473988	0.0003194
cg17187521	0.248854	0.0784493	0.789406	0.0182036
cg14529891	9.4532836	0.8615016	103.7312	0.0660643
cg03620975	0.1415355	0.0249106	0.804168	0.0273945
cg12778775	0.0660631	0.0089108	0.489778	0.0078534
cg03850730	0.8031256	0.3296944	1.95639	0.6293557
cg21245875	0.1969145	0.0532431	0.728269	0.0148862
cg06861572	1.4212135	0.6358266	3.176728	0.3916996
cg12459502	0.6009441	0.2752286	1.312123	0.2011916
cg08947151	1.5199117	0.7628511	3.028286	0.2339192
cg19779670	0.0003694	6.02E-09	22.66006	0.1599728
cg27225253	0.000126	2.91E-08	0.545741	0.0355779
cg03048372	0.3964111	0.14379	1.092856	0.0737196
cg25011368	2.0521819	0.3150131	13.36913	0.4521334
cg23582982	3.0400273	0.48814	18.93262	0.2334699
cg03036210	0.0568118	0.0144324	0.223635	4.09E-05
cg09167414	0.2833376	0.1152657	0.696479	0.0059921
cg11733958	0.8641449	0.1939643	3.849917	0.8480971
cg14143519	1.9757274	0.8290506	4.708396	0.1243317
cg02870485	0.345102	0.1120356	1.063014	0.0638101
cg08668510	17196928	0.2202281	1.34E+15	0.0723706
cg09947274	0.2884518	0.0984969	0.844742	0.0233458
cg13018793	0.1136701	0.0109738	1.177429	0.0682991
cg05859099	0.348625	0.1117809	1.0873	0.0694097
cg00082235	2.898296	0.1109724	75.69555	0.5226547
cg18118262	1.120455	0.5902384	2.12697	0.7280031
cg08751352	0.0328958	0.0003751	2.884698	0.1346961
cg16112129	1.6729224	0.8502525	3.291574	0.1361777
cg02983043	1.6936252	0.7502785	3.823069	0.2046813
cg17635080	2.0705436	0.5843276	7.336896	0.259505
cg11734777	64190222	2.88E-05	1.43E+20	0.2152594
cg00063535	0.3056796	0.1063143	0.878904	0.0278418
cg07196285	1.28E-38	7.46E-78	21.9099	0.058359
cg25414597	3.008569	0.9430816	9.597778	0.0627507
cg10989261	0.0037102	1.02E-05	1.353441	0.0629686

cg02519892	0.2862091	0.0067297	12.17235	0.5132237
cg02682955	0.0162312	0.0003606	0.73053	0.0338696
cg20007890	27.544622	0.2333123	3251.891	0.1731642
cg04038246	5.4899231	0.4808051	62.68498	0.1705054
cg23625390	0.0731374	0.0069152	0.773529	0.0297538
cg07880478	0.157131	0.0313944	0.786451	0.0243018
cg05480046	2.4853123	0.8056356	7.666962	0.1132065
cg06564900	0.0616902	0.000178	21.3785	0.3505078
cg08366845	0.6263281	0.2210092	1.774979	0.3786719
cg08928408	0.4836479	0.2430746	0.962319	0.0385099
cg21097090	1.9587643	0.2700039	14.21001	0.5060747
cg18888657	1.44E-08	1.05E-17	19.66325	0.0924848
cg26290391	0.5532204	0.2623458	1.166601	0.1199073
cg26229066	10.708056	0.6234977	183.902	0.1021894
cg06153623	0.9244795	0.2886883	2.960502	0.8947992
cg19559587	3.0753185	0.3401037	27.80794	0.3173265
cg14776321	1.6042871	0.8078476	3.185919	0.1768982
cg06032349	0.7936351	0.2833301	2.223049	0.6600745
cg12181407	1.3692173	0.6382169	2.93749	0.4197409
cg26904032	2.22E-13	2.29E-32	2156837	0.1915036
cg25145165	0.3429989	0.1386359	0.848613	0.0206061
cg25222493	1.7341642	0.6228921	4.828004	0.2919671
cg23397023	2.2767099	0.6656983	7.786423	0.1897334
cg22321572	0.23344	0.0757173	0.719707	0.0113245
cg12732155	2.3828153	0.7750435	7.325794	0.1297098
cg21249376	0.1452925	0.0427491	0.493809	0.0019989
cg24795958	1.7296148	0.8826691	3.389228	0.1104135
cg11774251	0.273859	0.0411469	1.822706	0.1805011
cg00067058	0.3441227	0.0672986	1.759626	0.2001087
cg22736807	3.4383921	1.4048081	8.415769	0.0068463
cg10761085	0.4368173	0.0556203	3.430569	0.4309012
cg13739417	1.3730582	0.6477318	2.910601	0.4082015
cg17833066	2.3832212	0.8381189	6.776775	0.1033631
cg01703733	1.4188213	0.4998679	4.027172	0.511033
cg12424867	0.5200151	0.2137149	1.265311	0.1495032
cg07320941	0.170473	0.0575984	0.504546	0.0013952
cg24966363	0.1621708	0.0191622	1.372464	0.0950362
cg26190476	0.2221243	0.0669525	0.736929	0.0139378
cg00682263	0.4515748	0.2042402	0.998431	0.0495484
cg18546236	0.1540977	0.0530893	0.447286	0.0005822
cg21389884	0.0001763	9.70E-22	3.2E+13	0.669903
cg15257489	3.8982462	0.7488397	20.29316	0.1060189
cg12645876	0.4246685	0.2129815	0.846756	0.0149994
cg07640412	0.4656475	0.1710287	1.267785	0.1347417
cg02235843	0.4998355	0.2348929	1.063614	0.0718774
cg16187883	0.0018081	5.56E-05	0.058815	0.0003783
cg02640638	2.192767	0.8495864	5.659492	0.1045863
cg23513727	0.1719942	0.0520144	0.568728	0.003916
cg06871344	0.2593112	0.0842895	0.797754	0.01857
cg06620837	2.00E-07	5.42E-14	0.741257	0.0456336
cg26334209	0.5184674	0.2192817	1.225859	0.1346174
cg05877850	2.104E+11	0.1707583	2.59E+23	0.0664273
cg24880892	2.355127	0.5192938	10.68109	0.2667973
cg12147799	0.3326652	0.1464088	0.755871	0.0085801
cg00982519	1.7925238	0.9481856	3.388727	0.0724603
cg01723148	2.3779837	0.9602214	5.889065	0.0611743
cg12202022	1.06E-29	7.99E-53	1.40E-06	0.0140449
cg21151963	1.0586777	0.3427907	3.269629	0.9210534

cg10029288	0.4452718	0.223732	0.886181	0.0212184
cg05783554	0.0119902	0.0002135	0.673279	0.0313618
cg20513976	3.3403382	1.1865187	9.403863	0.0223825
cg06602871	0.5067931	0.2261033	1.135938	0.0988515
cg06796096	0.1971546	0.0670436	0.579771	0.0031728
cg23717186	0.592972	0.2958451	1.188513	0.1407125
cg03036592	2.006732	1.0366588	3.88457	0.0387532
cg02775243	1.8191185	0.7049773	4.694041	0.2160305
cg08585782	28.574082	0.0013247	616346.8	0.5102449
cg08535938	0.287011	0.0628208	1.311275	0.1073221
cg18101784	2.30E-07	7.61E-12	0.006951	0.0036848
cg23806894	1.7924984	0.9727993	3.302891	0.0612719
cg23424003	1.285733	0.695167	2.378003	0.4230986
cg07511259	5.3285045	1.2747105	22.27405	0.0218737
cg20307184	2.0189151	0.7050932	5.780822	0.1905525
cg20664636	1.590647	0.6857512	3.689615	0.2796093
cg02124912	0.1695197	0.0589023	0.487875	0.0009995
cg24944820	2.6746624	0.7114497	10.05527	0.1453685
cg16831889	0.4726349	0.2494419	0.895534	0.0215426
cg07222250	3.0365684	0.4176531	22.07753	0.2724821
cg16888658	1.3901958	0.6374171	3.031993	0.4076376
cg09633240	0.3509682	0.1302809	0.945485	0.0383746
cg02504521	1.2260544	0.6315332	2.380254	0.5471013
cg06889422	0.9971565	0.416939	2.384812	0.9948931
cg00805193	1.5820941	0.551831	4.535848	0.3932913
cg06507987	7.2009548	1.5155255	34.21503	0.0130339
cg17209280	0.4042666	0.1663873	0.982236	0.0455504
cg07527906	1.372E+10	515.8285	3.65E+17	0.0074506
cg00445305	3.325415	0.9341869	11.83744	0.0636144
cg13698996	1361.27	2.68E-29	6.92E+34	0.8463868
cg24369728	1.6688234	0.7426423	3.750084	0.2150866
cg16436782	1.7757248	0.5427354	5.809827	0.3423895
cg24408776	2.3395079	0.2891139	18.93128	0.4256106
cg03720100	0.6156411	0.1717764	2.206438	0.456371
cg25024345	3.4333393	0.0842034	139.9921	0.5143959
cg00583291	4.4999838	1.3233801	15.30162	0.0160109
cg04750536	0.2581805	0.0920149	0.724417	0.0100993
cg03388266	0.7838747	0.3473667	1.768908	0.5575979
cg13974313	0.4387243	0.1377848	1.396954	0.1632439
cg17539151	2.1124938	0.1004561	44.42366	0.6303492
cg09256941	0.7520271	0.2708894	2.087733	0.5843549
cg01284033	0.073482	0.0011331	4.765517	0.2200286
cg06814469	0.420049	0.2001949	0.881347	0.0217902
cg17943663	0.1021424	0.0114915	0.907892	0.0406923
cg01942558	0.6568549	0.3550042	1.215361	0.1806628
cg18717067	1.4205279	0.1376334	14.6614	0.7681846
cg03614916	0.2324444	0.0718704	0.751775	0.014835
cg11481534	1.0653394	0.6074404	1.86841	0.8252363
cg23939932	5.637E+15	1.49E-05	2.13E+36	0.1335553
cg10738041	1.5688572	0.4055839	6.068567	0.5140887
cg09731996	1.1663556	0.5830685	2.333148	0.6635556
cg10550166	8.6235351	0.3353339	221.7651	0.1934456
cg18251191	3.6518965	1.3390747	9.959376	0.0113943
cg27378591	2.0677052	0.8421618	5.076702	0.1129379
cg18141888	0.7561133	0.2484392	2.301196	0.6225015
cg05050042	3.4874569	1.2197632	9.971079	0.0197744
cg25583619	0.3932823	0.1674906	0.923461	0.0321295
cg11795680	0.6342097	0.3031878	1.326643	0.2265354

cg23674788	1.8789153	0.727269	4.854219	0.1927936
cg02082462	0.0561265	0.0088436	0.356211	0.0022522
cg25655489	0.4485664	0.2046565	0.983169	0.0452459
cg25652610	2.9329616	0.4301372	19.99888	0.2719418
cg16601489	0.8886036	0.2816282	2.803754	0.8403461
cg18119977	0.4316973	0.1508083	1.235758	0.1174725
cg09736239	2.0722652	0.6775996	6.337494	0.2014035
cg02199589	3.50E-09	6.59E-29	1.86E+11	0.4007773
cg00244111	0.6052309	0.2804346	1.306203	0.2007639
cg19626656	0.7519042	0.3516598	1.60769	0.4620852
cg26737330	1.0142653	0.3750511	2.742917	0.9777375
cg24047665	0.0672353	0.0052224	0.865614	0.0383906
cg14658964	4.0281448	1.148054	14.13344	0.0295891
cg26523565	0.1748759	0.0513862	0.595133	0.0052627
cg10519882	0.2463579	0.1143094	0.530947	0.000349
cg15929437	43014347	0.0696705	2.66E+16	0.0887539
cg21745612	0.101701	0.0088497	1.168745	0.066536
cg09672233	1.1944077	0.5250809	2.716933	0.671812
cg13832669	1.1711913	0.6580722	2.084405	0.5910804
cg21069500	1.2161904	0.5164812	2.863839	0.6542158
cg24830622	3.96E-42	1.07E-67	1.47E-16	0.0015056
cg02478172	0.4223688	0.1692028	1.054329	0.0648031
cg20744437	0.0005958	3.17E-08	11.1907	0.1391534
cg07908498	0.45802	0.1683948	1.245777	0.12614
cg03393444	1.1958726	0.3772648	3.790736	0.7612132
cg27510041	88.291858	1.6328228	4774.218	0.0277507
cg20222052	2.29E+27	8815838.6	5.94E+47	0.0086198
cg12532266	0.2095235	0.0905942	0.484579	0.0002587
cg09847549	4.1093899	0.9736888	17.34341	0.0543958
cg08989214	0.5310679	0.234016	1.205187	0.130128
cg18935453	0.3183136	0.1408792	0.719223	0.0059153
cg00018229	1.4446773	0.5901259	3.53669	0.4206119
cg05094695	0.2700227	0.0044693	16.31417	0.5315264
cg25694755	0.8126355	0.399687	1.652234	0.5666089
cg23165500	0.5220621	0.2457764	1.10893	0.0908428
cg21530266	1.2425886	0.6766781	2.281774	0.4836516
cg20918218	0.5223854	0.2035766	1.340461	0.1768419
cg02851047	0.1513868	0.0479191	0.478264	0.0012967
cg15993027	4.7065881	0.9300158	23.81892	0.0611697
cg10249213	1.5492457	0.7192327	3.337115	0.2634972
cg12785276	0.0666663	0.0055148	0.805901	0.0331989
cg20468787	0.6379839	0.2987768	1.362299	0.2455684
cg12194898	1.2090227	0.6264649	2.333308	0.5715028
cg21152753	0.2776276	0.0811024	0.950368	0.0412465
cg19931491	0.32171	0.1470775	0.703692	0.004512
cg01075459	0.3626387	0.1264653	1.039865	0.0591289
cg08786077	0.3067507	0.089794	1.047909	0.0593886
cg04194054	9402.6545	0.1202437	7.35E+08	0.1115008
cg25139636	1.2288133	0.0603121	25.03615	0.8934193
cg04573851	6.7441641	0.6804685	66.84181	0.1028909
cg08227526	2.5020156	1.169346	5.35349	0.0181241
cg00071026	2.1176361	0.7313136	6.131956	0.1666252
cg02286091	2.3510591	0.8739639	6.324608	0.0904278
cg10660965	0.5026994	0.2439639	1.035836	0.0622497
cg12609063	2.182601	0.9763547	4.879115	0.0572153
cg01437135	0.9688441	0.3911833	2.399537	0.9454654
cg20387429	2.655519	0.8168536	8.632858	0.1044504
cg26915924	0.2877745	0.1321019	0.626896	0.0017158

cg20585676	3.6154553	1.1398784	11.46747	0.02909
cg10449665	4.2756865	1.6392373	11.15244	0.0029746
cg14239655	2.1529556	0.8096561	5.724922	0.1243395
cg24887381	1.647619	0.5033295	5.393382	0.4092031
cg05961294	3.808716	1.230614	11.78787	0.0203429
cg17266581	0.45323	0.2331364	0.881104	0.0196398
cg11179180	0.8483642	0.3728571	1.930288	0.6950242
cg26952362	2.8350177	0.8973661	8.956574	0.0758227
cg02344868	4.0107752	0.9196081	17.49258	0.0645395
cg23550826	2.0668444	0.7437269	5.743837	0.1638603
cg07370771	0.1394165	0.0258223	0.752721	0.0220133
cg13925011	4.8008392	1.6189651	14.23629	0.0046742
cg23627145	1.1881606	0.5929572	2.380822	0.6268436
cg04923020	2.7107133	0.9624011	7.635035	0.0591028
cg10876737	4.8854559	0.7362233	32.41907	0.1004195
cg07348263	0.3621369	0.1732369	0.757016	0.0069362
cg14788768	0.798163	0.3856039	1.652121	0.5436089
cg05068156	1.9257388	0.7225774	5.13228	0.1901032
cg02032097	29.289431	0.8285957	1035.331	0.0633681
cg03496157	0.6099034	0.0378232	9.834766	0.7274238
cg09316954	0.3507306	0.1174531	1.047329	0.0605022
cg01668653	1.4752602	0.4911873	4.430881	0.4883296
cg24361265	3.2264103	0.5392494	19.3041	0.1993699
cg01032119	1.2306096	0.5820971	2.601628	0.5869398
cg14782638	0.3220017	0.0534184	1.941	0.2163188
cg23698978	1.0515384	0.3633393	3.043252	0.9261518
cg04485956	0.5999824	0.0822253	4.37796	0.6144068
cg17191872	2.3474054	0.9702079	5.679517	0.0583748
cg06417454	861.3889	1.41E-07	5.25E+12	0.5565665
cg18311665	0.4594916	0.2213147	0.953992	0.0369489
cg03715204	1.8768172	0.889503	3.960012	0.0984116
cg05180182	0.5385863	0.2628905	1.103407	0.0908266
cg02829696	1.0807487	0.3010322	3.880042	0.905217
cg12255293	2.65E-25	1.04E-50	6.730966	0.0579496
cg12714796	0.5786478	0.0136202	24.58359	0.774885
cg02479022	0.4526863	0.2018018	1.015476	0.0545167
cg22953960	2.3902444	0.4774294	11.96673	0.2889963
cg00830252	0.3868455	0.1663834	0.899425	0.0273701
cg18918423	7.46E-06	1.73E-12	32.26756	0.1299405
cg07164606	0.5837876	0.3068568	1.110642	0.1009689
cg26845138	0.2460343	0.0854544	0.708364	0.0093491
cg18167088	1.8944088	0.0382318	93.86922	0.7483322
cg01462546	0.6067295	0.2640231	1.394274	0.2391854
cg19845715	1.3217398	0.6364524	2.744897	0.4543814
cg21457401	2.2055932	0.9885824	4.920826	0.0533697
cg19401519	1.2949993	0.3713203	4.516378	0.6850395
cg04330122	0.5389028	0.2759525	1.052414	0.0702392
cg17161388	2.639E+09	3.61E-10	1.93E+28	0.3276229
cg13982454	0.2710942	0.1276865	0.575566	0.0006788
cg03444838	4.400562	1.0825923	17.88757	0.0383704
cg06746450	3.7181056	0.0868124	159.2434	0.4933175
cg04390523	4.84E-09	3.46E-21	6762.356	0.1796313
cg16427638	0.636593	0.3160701	1.282154	0.2061492
cg09390137	0.9044955	0.3882811	2.10701	0.8160357
cg18475668	1.5432251	0.669829	3.555451	0.3082522
cg00048370	0.6920499	0.2351091	2.037067	0.5039687
cg06644998	0.249877	0.1167657	0.534734	0.0003534
cg21156386	0.4955715	0.2031061	1.209177	0.122926

cg13044475	0.0610038	0.0112215	0.331638	0.0012052
cg01419914	0.2688924	0.0965579	0.748806	0.0119522
cg06368300	1.7761351	0.5457508	5.780396	0.3400278
cg24871584	4.42E-29	3.68E-58	5.320987	0.0560003
cg05874348	0.1646596	0.0599616	0.452169	0.0004654
cg20788199	0.0134325	5.01E-05	3.60347	0.130874
cg09731079	0.4272068	0.1665998	1.095474	0.0766986
cg13794530	0.3206737	0.1314118	0.782514	0.0124623
cg14517067	1.01E+20	6.11E-11	1.66E+50	0.1944713
cg14431789	0.6092374	0.0801779	4.629335	0.6319871
cg00294261	0.4503321	0.1971493	1.028657	0.0583679
cg07682037	1.5566352	0.6869311	3.527447	0.2890304
cg24087944	1.2600837	0.5461188	2.907446	0.587871
cg13054119	1.0136415	0.3470586	2.960507	0.9802329
cg26806779	2.4669234	0.6065972	10.03254	0.2071078
cg05656360	0.4416815	0.2032536	0.959799	0.0390575
cg20289299	102.85783	2.2888507	4622.291	0.0170114
cg04613057	0.3025235	0.1291316	0.708738	0.0059132
cg27016106	0.1957717	0.0238891	1.604354	0.1286354
cg02474628	0.0020905	2.69E-06	1.626113	0.0692465
cg09307431	1.4347692	0.6476859	3.178335	0.373675
cg23753807	0.4131417	0.1732938	0.984952	0.0461335
cg02001694	0.2897913	0.1098644	0.764388	0.0123179
cg18720622	1.1690351	0.4952757	2.759358	0.7215224
cg05887749	0.0003515	7.20E-12	17152.88	0.3785691
cg14518948	0.2597763	0.0891402	0.757051	0.0135125
cg12851792	20.12684	0.0441905	9166.894	0.3364413
cg10582860	0.3388488	0.1305677	0.879379	0.0261393
cg04560225	3.1168748	0.9902601	9.810461	0.051988
cg17935811	2.2830457	1.1828536	4.406545	0.0138747
cg06852350	0.4476155	0.1736099	1.154079	0.0962294
cg12936779	25.525552	0.5660489	1151.056	0.0954903
cg03950121	4.0898099	1.1280645	14.82765	0.0320861
cg02470858	1.720285	0.4578165	6.464119	0.4218577
cg27298324	1.2611101	0.5926666	2.683463	0.547071
cg21499391	889596101	8.22E-13	9.62E+29	0.4043453
cg08242636	0.7159608	0.3269401	1.56787	0.4034525
cg22524346	0.3876636	0.0225611	6.661144	0.5137056
cg16466870	2.6094271	0.8750549	7.781352	0.0853342
cg16124719	1.6508044	0.7743014	3.519501	0.1943794
cg09162909	0.1956953	0.0883617	0.433408	5.80E-05
cg11796258	0.6108042	0.239929	1.554967	0.3011279
cg22939524	1.2033503	0.4077931	3.550948	0.7374139
cg11815008	0.4410025	0.2113494	0.920198	0.0291408
cg24162959	0.1797662	0.0659579	0.489947	0.0007947
cg12392557	0.1492704	0.0445765	0.499852	0.0020386
cg17809798	3.07E-08	3.57E-18	263.5465	0.1382512
cg04640975	0.3270025	0.0745897	1.433584	0.138254
cg12787209	0.2029905	0.0723846	0.569253	0.0024383
cg11855682	0.4549876	0.1951234	1.060938	0.0682997
cg00545918	1.6977407	0.7962563	3.619844	0.1706316
cg16385448	7.0574434	0.3513364	141.7659	0.2017421
cg09414863	0.3537144	0.1544728	0.809941	0.013946
cg09279803	1.12E-08	3.43E-22	363724.8	0.2487527
cg09179211	2.5660194	1.0757177	6.120988	0.0336272
cg22101249	0.2046892	0.070418	0.594986	0.003572
cg25096749	0.7748511	0.2495326	2.406075	0.6590421
cg13516551	231964.33	2.7013623	1.99E+10	0.0330554

cg13968061	0.7160476	0.3752827	1.366235	0.3109279
cg19043800	0.3976382	0.148923	1.061731	0.0657062
cg02524954	1.6261681	0.7355781	3.595026	0.229652
cg17282395	2.5547599	1.1039319	5.91232	0.028457
cg20320656	0.7273746	0.3253804	1.626016	0.4380185
cg20703122	1.2289231	0.5174028	2.918909	0.6404709
cg10586619	0.0039957	7.64E-05	0.208964	0.0062298
cg15027165	0.3478794	0.1320192	0.916685	0.0326847
cg23427653	1.4030931	0.7225595	2.724579	0.3171907
cg08183724	1.8066934	1.0039753	3.251217	0.0484729
cg02642561	0.0006874	5.85E-06	0.08082	0.0027516
cg21193888	0.1180048	0.0107601	1.294146	0.0803015
cg25143099	1.626316	0.6634542	3.986566	0.287749
cg18271897	0.2165665	0.0393918	1.19063	0.0785246
cg01617139	2.15E-07	2.87E-14	1.614759	0.0573499
cg06147196	0.4078101	0.1583283	1.050407	0.0631562
cg08231730	0.3155186	0.0506727	1.964609	0.2163656
cg24568150	3.8343534	0.9291296	15.8237	0.0631226
cg21849780	0.4374215	0.2205155	0.867683	0.0179765
cg01412469	1.1370657	0.4401018	2.937771	0.7908299
cg20688757	1.58E+23	1.93E-08	1.29E+54	0.1413306
cg05769153	1.2454458	0.5782303	2.682556	0.5750133
cg24245285	0.0561061	1.42E-11	2.21E+08	0.7983227
cg01715686	4.5399883	1.1753852	17.53595	0.0282108
cg04587084	1.4497827	0.6495072	3.236099	0.3646194
cg23510807	1.4056932	0.4445959	4.444426	0.5620447
cg08840441	0.9843974	0.0126249	76.75587	0.9943549
cg16477975	3.054738	0.3611662	25.83693	0.305321
cg24937136	0.9446415	0.3963829	2.251226	0.8977288
cg27328245	1.5343879	0.5873713	4.008276	0.3821854
cg15465092	242782.82	73.575076	8.01E+08	0.0027014
cg19434199	1.7210467	0.2833416	10.45382	0.5552843
cg16775460	0.9187987	0.0084146	100.3251	0.9717863
cg18103859	2.4962454	1.1705271	5.323449	0.017911
cg14005246	1.5805494	0.7496444	3.332429	0.2290463
cg18413131	1.5537533	0.8302644	2.907687	0.1681379
cg10946816	6.188217	0.6086664	62.91465	0.1234701
cg08143343	0.2725261	0.0899358	0.825817	0.0215442
cg06806214	1.4898104	0.2414737	9.191624	0.6676392
cg00061413	0.3494597	0.1512423	0.80746	0.0138762
cg03242471	3.0305445	1.3813264	6.648827	0.0056781
cg19810092	2.29E-05	1.43E-08	0.036653	0.0045336
cg11062848	0.442318	0.1819878	1.075046	0.0718192
cg21411705	1.3930304	0.0591691	32.79639	0.8370453
cg15645203	0.6444165	0.3634363	1.142628	0.13266
cg21026460	0.613764	0.2979382	1.264377	0.1855677
cg02304134	0.3074376	0.1114228	0.848281	0.0227436
cg24044478	0.500004	0.2555352	0.978354	0.0429845
cg00588198	1.1148516	0.4074102	3.050719	0.832356
cg06623219	1.2865738	0.5198288	3.184264	0.5857709
cg24354581	1.468341	0.7881844	2.735433	0.2262308
cg24960291	0.4163331	0.1834521	0.944842	0.0361128
cg13428978	2.3362999	1.0670721	5.115209	0.03381
cg26185508	0.3724375	0.1769562	0.783865	0.0092861
cg12851635	12.524349	0.7885128	198.9306	0.0732044
cg25879561	0.4083556	0.116194	1.435136	0.1625283
cg13388731	1.3250923	0.4139995	4.241236	0.6353429
cg27336481	2.1871572	0.785492	6.090013	0.1341714

cg24759654	1.5843498	0.3238266	7.75157	0.5699926
cg04669894	0.3898827	0.1823394	0.833657	0.0151336
cg17476320	0.5099678	0.2285837	1.137733	0.1000129
cg25151274	0.001505	4.18E-07	5.412894	0.1197775
cg12520549	5.4668356	1.6052628	18.61769	0.0065886
cg20790056	1.5193499	0.4556157	5.066604	0.4960657
cg14522990	2.4691309	0.331425	18.39513	0.3776973
cg16718999	0.1133765	0.0024796	5.183908	0.2643214
cg16185996	0.1964192	0.0836095	0.461437	0.0001879
cg14446604	0.1873845	0.0341004	1.029692	0.0540661
cg05401522	0.1090344	1.68E-07	70659.77	0.7454978
cg16512640	0.2390878	0.0881541	0.648444	0.0049403
cg08190044	7.35E-27	4.77E-53	1.131676	0.0504718
cg15528736	0.1586129	0.0572419	0.439504	0.0003987
cg24507266	0.3169309	0.1260628	0.796787	0.0145688
cg13745279	0.273084	0.0222809	3.347031	0.3100401
cg15229454	0.5360149	0.234485	1.225289	0.1393254
cg19724470	1.0819539	0.4937106	2.371074	0.8440047
cg05787790	1.3686154	0.5269697	3.554489	0.5193073
cg01576275	0.4316948	0.1209658	1.540604	0.1956112
cg17598574	2.2321432	0.1519818	32.7833	0.5580706
cg14209784	0.5989404	0.2510565	1.42888	0.2478974
cg05859929	3.2711036	0.6974609	15.34153	0.1328363
cg13401196	0.2410316	0.0624441	0.930371	0.038952
cg13149566	0.4731136	0.2387927	0.937367	0.0319231
cg01918043	0.2367492	0.046877	1.195687	0.081217
cg04955333	0.434194	0.1788015	1.054378	0.0653308
cg11935041	5.9045896	0.4797821	72.66669	0.1655895
cg00144550	2.0105159	0.7642814	5.288856	0.1570021
cg08241841	0.7641959	0.0959566	6.086038	0.7994719
cg27071460	2.5418094	1.1157915	5.790325	0.0263652
cg18007957	0.3757138	0.1224187	1.153099	0.0870844
cg07804434	3.0091596	0.8716181	10.38877	0.0814005
cg04275566	6.684754	1.3315005	33.56058	0.0210134
cg14596108	1.4013744	0.7667375	2.561307	0.2727609
cg10336108	0.4441991	0.2318099	0.851184	0.0144632
cg14425294	1.8227099	0.8156698	4.073059	0.143379
cg08897054	0.3863362	0.1603242	0.930962	0.0340581
cg17970936	2.970081	0.8453972	10.4346	0.0895086
cg03655701	0.2334274	0.058439	0.932398	0.0394918
cg00664609	0.0603738	0.0164317	0.221826	2.36E-05
cg00401456	1.4475854	0.1235542	16.9602	0.768305
cg16717267	0.6687507	0.2742962	1.630455	0.3762384
cg01750051	7.5995245	0.0597618	966.3821	0.4120177
cg05549773	0.2684283	0.0964123	0.747351	0.0118226
cg00403616	1.3252867	0.1437024	12.22238	0.8037807
cg18801945	1.7988195	0.3919876	8.254729	0.4500935
cg00071250	2.70794	0.853999	8.58659	0.0906621
cg20322977	2.9296237	0.8566417	10.019	0.0866537
cg08230483	1.9235221	0.8023177	4.611561	0.1425726
cg24144893	0.29246	0.0454962	1.880002	0.1953152
cg01392518	2.3941394	1.184705	4.838254	0.0150092
cg27368243	1.8721646	0.4650616	7.536637	0.3774896
cg13009098	0.3374145	0.1305574	0.872019	0.0249197
cg00975680	0.256346	0.1139873	0.576497	0.0009949
cg22760475	546.48891	10.653739	28032.42	0.0017033
cg24750887	0.7445819	0.320956	1.727346	0.4921325
cg22346081	0.258378	0.1024916	0.651363	0.0041222

cg13451886	1.9206716	0.4937339	7.471594	0.3463528
cg01932308	1.9609285	0.8950083	4.296319	0.092417
cg26738404	1.9198247	0.6089238	6.052854	0.2655957
cg13033972	0.9272537	0.3709287	2.317964	0.8716452
cg00398764	1.5814062	0.6369937	3.926013	0.3232164
cg10171347	0.8230755	0.1642234	4.125194	0.8128406
cg26632897	0.2917587	0.0900732	0.945043	0.0399539
cg15704408	0.4926716	0.2214251	1.096196	0.0827625
cg01267532	0.2555118	0.0874471	0.746581	0.0126249
cg09423126	1.1065105	0.4720844	2.593531	0.8158533
cg12571879	0.9724823	0.5112472	1.849833	0.9322184
cg00688932	0.2553581	0.0924332	0.705458	0.0084654
cg23816347	1.3331937	0.6937978	2.561849	0.3881619
cg23202468	2.2253638	0.3586281	13.80886	0.3903993
cg06657050	1.6050552	0.8076206	3.189867	0.1769391
cg19497750	0.9532358	0.4652809	1.952925	0.8958717
cg05905988	0.0314574	0.0001541	6.419888	0.2023999
cg10361281	0.3565651	0.1192005	1.066595	0.06509
cg14858784	0.2246601	0.0603531	0.836281	0.0259758
cg24705668	0.5865507	0.2990678	1.150381	0.120583
cg23768816	0.24269	0.1009641	0.58336	0.001554
cg04517323	0.248266	0.0160227	3.846794	0.3190381
cg26648203	2.1070409	0.9384544	4.73078	0.0709129
cg00205605	1.7057865	0.4284163	6.791775	0.4487309
cg19587537	0.5960758	0.3006879	1.181645	0.1383654
cg23531748	0.0602454	0.0027347	1.327194	0.074985
cg05344066	4.2137184	1.1547794	15.3756	0.0294162
cg11443683	1.1282268	0.3426761	3.714574	0.8427011
cg26158279	0.1765796	0.0439729	0.70908	0.0144993
cg11587640	3.0891067	0.9105138	10.48043	0.0703642
cg11666098	1.1130341	0.5541452	2.235596	0.7634484
cg05704955	0.2683109	0.0797231	0.90301	0.0336091
cg23385847	0.2787421	0.1236666	0.628279	0.0020642
cg16869487	3.919E+09	5.47E-16	2.81E+34	0.4493618
cg21512179	2.6553963	0.782363	9.012606	0.117274
cg03976326	3.2732905	1.5608667	6.864411	0.001699
cg17825194	0.3343662	0.126335	0.884954	0.0273784
cg01185080	0.4178993	0.2057896	0.848633	0.0157752
cg09133892	1.4740062	0.5643286	3.850052	0.4283403
cg10688790	0.6657614	0.2993464	1.480687	0.3185046
cg04094193	0.4010001	0.1976085	0.813735	0.0113793
cg01883757	0.394642	0.1904733	0.81766	0.0123636
cg10047755	3.8255232	0.3349819	43.68781	0.280239
cg09341020	0.3418234	0.0935605	1.248851	0.1044166
cg00166343	1.1082713	0.508079	2.417469	0.796142
cg07000438	1.3760437	0.5639988	3.35727	0.4830147
cg14330621	0.0494734	0.0030917	0.791668	0.0335783
cg22499809	0.2201032	0.0812071	0.596566	0.0029264
cg22256677	2.0405149	1.0483078	3.971831	0.0358349
cg26358286	2.3938058	0.8003899	7.159394	0.1183767
cg08402572	0.2001206	0.0927005	0.432018	4.18E-05
cg23612220	2.6948657	0.8084347	8.983163	0.1065734
cg13558695	2.7854154	1.0109765	7.674302	0.0475828
cg16779441	0.6184631	0.204061	1.874423	0.3956754
cg06813515	0.0548612	0.0065047	0.462705	0.0076225
cg13957377	1.5464902	0.7146336	3.346655	0.268324
cg02516234	0.0197953	0.0010371	0.377845	0.009139
cg00414384	1.5671423	0.3879482	6.330575	0.5282473

cg26070636	108.40195	1.00E-04	1.18E+08	0.5086868
cg06484415	1.3717483	0.662225	2.841471	0.3949307
cg01190637	1.6678112	0.562217	4.947546	0.3565375
cg18089852	0.4461978	0.1707157	1.166223	0.0997078
cg21951648	0.3940473	0.1809429	0.858134	0.0190143
cg10161121	2.0968957	0.7475669	5.88171	0.1593969
cg06958937	5.55E-05	2.21E-10	13.90137	0.1223433
cg10144863	0.4002873	0.1625681	0.985617	0.0464286
cg08066407	0.7786955	0.3908935	1.551233	0.4768635
cg18805894	0.1618549	0.0001486	176.3092	0.6097889
cg19817488	0.4264956	0.1962135	0.927044	0.0314604
cg23302291	1.9060447	0.6602294	5.502642	0.2330838
cg10334315	1.15E-14	4.72E-27	0.027869	0.027383
cg06801544	0.0946451	4.33E-28	2.07E+25	0.9392688
cg00575066	1.8879236	0.7031882	5.068708	0.2072581
cg10151685	0.032146	0.0020132	0.513297	0.0150263
cg14894367	0.135807	0.0419991	0.439142	0.0008551
cg23024358	0.1025847	0.0200221	0.525601	0.0063035
cg21789136	0.1432303	0.0317793	0.645544	0.0114164
cg02641539	2.6653822	0.8752201	8.117115	0.0844565
cg04434871	0.8619549	0.1589683	4.673675	0.8632554
cg16120742	1.9323407	1.0048549	3.7159	0.0483271
cg22819024	0.5315198	0.2174169	1.299408	0.1658329
cg25326319	1.8503826	0.6301496	5.433497	0.2628349
cg10457985	1.8796771	0.7534715	4.68921	0.1760314
cg05490023	2.2681097	0.5472646	9.400063	0.2589191
cg08130179	1.5187002	0.7601014	3.034398	0.2367191
cg14785303	0.7916049	0.3712952	1.687709	0.5451742
cg03501666	1.1356801	0.5766325	2.236726	0.7129329
cg01903440	0.3754703	0.1718982	0.820124	0.0139935
cg25084878	0.1584366	0.0247186	1.015518	0.0519294
cg18290233	3.0857525	1.089994	8.735707	0.0338155
cg14331206	0.3156464	0.1424515	0.699415	0.0045019
cg02458053	2.2205983	0.5654935	8.719918	0.2529839
cg13476777	2.62E+32	3.38E-11	2.03E+75	0.1384918
cg21754837	1.3322731	0.518489	3.423316	0.5512967
cg04521510	2.6135329	1.0798688	6.325356	0.0331423
cg01753241	0.8157536	0.1675968	3.970566	0.80088
cg10075733	8.4617321	0.7029362	101.8598	0.0925127
cg16065021	0.2346239	0.0732277	0.751743	0.0146754
cg26843278	0.3495122	0.1515542	0.80604	0.0136737
cg05173749	2.859829	0.2307298	35.44675	0.4132826
cg15320349	1.6264799	0.5630783	4.698168	0.3687815
cg00335908	1.3019914	0.460848	3.678397	0.6184767
cg02612397	0.3957792	0.1797158	0.871605	0.0213848
cg01027397	2.4494533	1.2206782	4.915154	0.0116978
cg07108579	0.2528594	0.0902058	0.7088	0.0089376
cg14694441	7.217915	1.281828	40.64375	0.0249914
cg08915683	8.7539952	0.3001086	255.349	0.2074524
cg07935340	1.7356469	0.5667987	5.314886	0.3342211
cg01450725	0.3621922	0.1643559	0.798165	0.0117631
cg13429424	0.4555532	0.2119457	0.97916	0.0440191
cg17750946	1.0067284	0.0439783	23.04549	0.9966504
cg24601850	1.0535632	0.5509026	2.014867	0.8746712
cg21893387	5.3040217	2.1144505	13.30494	0.0003769
cg23758354	0.4072211	0.096827	1.712633	0.2202601
cg10209670	1.9115725	0.8511993	4.292896	0.1164937
cg16318412	2.0246288	0.3782953	10.83577	0.4098381

cg03190266	1.5020928	0.7052703	3.199174	0.2915377
cg21458840	106.57512	1.81E-25	6.29E+28	0.8819886
cg04480383	1.60E+30	5156268.8	4.95E+53	0.0117353
cg11190278	0.8997238	0.2834858	2.855533	0.8576844
cg25999486	1.73E-32	1.90E-62	0.015774	0.0377227
cg20738500	2.1521227	0.8911633	5.197288	0.0884157
cg14204241	2.1440674	0.3542618	12.97635	0.4063749
cg04394254	0.5179101	0.2370797	1.131395	0.0988784
cg17112695	0.1894411	0.0633924	0.566123	0.0028959
cg16962191	1.0923492	0.429324	2.779315	0.8529283
cg10585486	0.0557241	0.0027864	1.11439	0.0588782
cg11617964	7.262981	0.3604681	146.34	0.1956503
cg08354401	1.9924986	0.8920855	4.450303	0.0926769
cg17590162	0.669521	0.0978266	4.582172	0.6826668
cg04571130	0.4436124	0.1788979	1.100024	0.0793935
cg12694058	3.2596013	0.8555724	12.41859	0.0833804
cg23841819	3.3243184	0.9111955	12.12812	0.0688912
cg16532685	0.5401602	0.2567553	1.136385	0.1045814
cg05475904	0.2342003	0.0307585	1.783241	0.1610674
cg14294859	0.4113173	0.226724	0.746202	0.0034634
cg13688769	0.2945208	0.0060414	14.3579	0.5376127
cg21206147	1.223283	0.5268803	2.840154	0.6391041
cg17126250	1.0397155	0.2374467	4.552635	0.958775
cg01934296	2.4091267	0.7971125	7.281145	0.119203
cg11056055	0.382356	0.1707373	0.856263	0.0194285
cg23020795	3.3595958	1.2167738	9.276074	0.0193561
cg15792145	3.37E-13	9.19E-28	123.3899	0.0932443
cg26484333	2.32E-24	1.97E-61	2.74E+13	0.2114806
cg15300753	0.5043939	0.2292378	1.109822	0.0889452
cg11738543	1.8514409	0.6555309	5.229094	0.2449253
cg14018275	0.0007513	4.64E-07	1.21547	0.0563649
cg01987925	0.8438473	0.3881669	1.834464	0.6682643
cg02194300	2.9857345	0.3522971	25.30424	0.3157794
cg12345778	1.7609483	0.7709231	4.022371	0.179387
cg22077389	121047341	4.73E-20	3.10E+35	0.5632556
cg17197870	0.501	0.2382598	1.053476	0.0683661
cg23207673	0.339596	0.1432034	0.805326	0.0142303
cg03708990	0.1939437	0.059066	0.636816	0.006853
cg25526475	0.7428711	0.0591924	9.32312	0.8178683
cg18968409	1.0808897	0.5409112	2.159915	0.8256999
cg10348368	0.9625967	0.0445046	20.82013	0.9806091
cg01261351	1.5048249	0.6732289	3.363638	0.3193334
cg02729383	0.3746346	0.1980353	0.708717	0.0025404
cg17160666	0.5339671	0.2692794	1.058829	0.0724461
cg09074468	0.4022172	0.033406	4.842809	0.4731307
cg17508941	4.4606544	0.907346	21.92927	0.0657244
cg02055351	0.1598203	0.053453	0.47785	0.0010328
cg09491536	1.333768	0.6314983	2.81701	0.4502523
cg09183671	0.2892355	0.1026453	0.815012	0.0189272
cg10414975	2.1618836	1.0432996	4.479769	0.0380801
cg23741520	1.9151361	0.6492024	5.649619	0.2390905
cg05185926	2.0085407	0.7350509	5.488376	0.1738958
cg02318527	1.9775092	0.7132834	5.482453	0.1900127
cg03254023	2.3303656	1.0467963	5.187832	0.0382682
cg00254426	7.53E-16	1.57E-26	3.62E-05	0.0055242
cg05963179	0.3529454	0.172722	0.721219	0.0042863
cg16848873	0.6995975	0.2710474	1.805724	0.4602471
cg04126707	0.004098	2.13E-05	0.788361	0.0405031

cg24217704	3.6975107	0.8766621	15.59505	0.0749596
cg02070232	1.6261976	0.5971564	4.42852	0.3414574
cg16868572	82283.802	1.22E-05	5.54E+14	0.3269613
cg00333583	0.5304854	0.2539598	1.108107	0.0916366
cg18112681	2.2507215	0.7031684	7.204173	0.1717219
cg04934382	0.561862	0.2928822	1.07787	0.0828525
cg09594075	0.1693672	0.0231338	1.239973	0.0804288
cg01040786	0.2251476	0.0776506	0.652815	0.0060485
cg03292213	2.879146	0.4996282	16.5913	0.2366369
cg22876901	0.4439363	0.1905396	1.034323	0.059868
cg13808088	0.0851984	0.0070326	1.032157	0.0529787
cg04983606	1.40E-16	7.85E-34	25.0582	0.0716978
cg02314348	0.5910715	0.1927241	1.812775	0.3577771
cg14501253	0.0141848	0.0005519	0.364601	0.0101975
cg18032386	0.7332157	0.0055196	97.39869	0.9009987
cg03816029	0.5611483	0.2912068	1.081319	0.0842827
cg05266497	2.451872	0.797927	7.534118	0.1173859
cg13808641	6.0985732	1.1129775	33.4172	0.0372241
cg10855276	0.3175098	0.1340764	0.751903	0.0091008
cg03120185	1.81079	0.9065658	3.616903	0.0925535
cg13694466	0.277504	0.1143103	0.673679	0.0046133
cg10995925	1.3719834	0.3328098	5.655898	0.6616668
cg20018344	1.8794825	0.6909599	5.112387	0.2164949
cg03298938	5.62E-06	3.07E-11	1.029752	0.0505569
cg13396716	12469.59	0.0004254	3.66E+11	0.2823384
cg00334691	0.41716	0.1362649	1.27709	0.1256415
cg23097843	0.6799342	0.3574635	1.293308	0.239626
cg17929068	1.7989729	0.7485873	4.323215	0.1892962
cg08234664	0.2112486	0.0360376	1.238316	0.0848766
cg04505972	2.6508325	1.1428449	6.148615	0.0231467
cg07551364	0.107448	0.0244632	0.471936	0.0031317
cg19814174	0.7757813	0.2703058	2.226503	0.6369491
cg13637893	0.6200384	0.2730854	1.407793	0.2532639
cg15439369	1.752758	0.7459146	4.118649	0.1979374
cg25321212	32.246315	1.4214471	731.5255	0.029201
cg06314111	0.1098128	0.0229194	0.526142	0.0057221
cg17641876	0.2383458	0.0881841	0.644206	0.0047019
cg23156711	1.3006684	0.2006657	8.430629	0.7827988
cg25803107	0.4371099	0.169627	1.126384	0.086613
cg15989167	1.3304463	0.5961831	2.969033	0.4857241
cg16402415	0.5013422	0.2619826	0.959392	0.0370551
cg19193851	0.2594669	0.0606347	1.110306	0.0689277
cg23150219	1.28E-12	8.36E-47	1.97E+22	0.4953621
cg07037112	1.3337305	0.6407673	2.776104	0.4413264
cg21399079	7.6881935	2.0587668	28.71055	0.0024123
cg23814154	8723.0204	3.9110846	19455239	0.0210735
cg08273672	0.2070996	0.0657755	0.652071	0.0071309
cg03670994	0.270435	0.0828097	0.883171	0.0303332
cg16619777	0.4112627	0.1050481	1.610092	0.2019632
cg07125166	3.9129669	0.1958899	78.16283	0.3718779
cg07656173	3.9642398	0.8096646	19.40952	0.0892349
cg10440447	2.3469806	0.5174733	10.64464	0.2687517
cg01903654	2.240742	0.4671937	10.74699	0.3131631
cg22747092	0.1412325	0.0320283	0.622781	0.0097238
cg02582774	1.6167695	2.39E-21	1.09E+21	0.9843371
cg14753385	1.9633017	0.7182001	5.366963	0.1885646
cg08008072	0.012654	0.0003015	0.531165	0.0219183
cg21565575	3.0889271	0.9779368	9.756735	0.0546127

cg10431651	2.0137223	0.2693353	15.05587	0.4952675
cg08866794	1.6754963	0.6218253	4.514593	0.3074768
cg01313994	0.1488517	0.0050322	4.402993	0.270362
cg06161697	0.7698169	0.0146694	40.39838	0.8969898
cg24794433	0.7144725	0.372912	1.368878	0.3108351
cg02025879	0.2075764	0.0304508	1.415002	0.108385
cg12891178	1.2864683	0.2027823	8.161466	0.7892909
cg18605031	0.1671937	0.0420605	0.664608	0.0110787
cg22856792	0.7575764	0.3061086	1.874897	0.5481857
cg16711597	0.4711767	0.2400098	0.924994	0.0287785
cg15291170	1.2820574	0.2999564	5.4797	0.7374335
cg16096432	0.210142	0.0841531	0.524754	0.0008348
cg22545957	0.3334892	0.025632	4.338917	0.4015472
cg14574951	0.6899583	0.2724065	1.747544	0.433804
cg12949023	0.2676258	0.1055806	0.678379	0.005475
cg27045723	0.3433473	0.1458664	0.808188	0.0143833
cg05448404	2.0654453	0.9189718	4.642215	0.0791807
cg27294163	1.9818185	0.7109555	5.524403	0.1909611
cg00748640	0.950964	0.201614	4.485464	0.9493432
cg00893348	0.8173629	0.3817729	1.749947	0.6035982
cg11130530	0.1827451	0.036736	0.909076	0.0378549
cg00825407	5.49E-11	4.00E-18	0.000753	0.0048374
cg08596595	1.5590724	0.0664507	36.5791	0.7826659
cg11635857	0.0186065	0.001328	0.260698	0.0030953
cg16739342	0.1425598	8.11E-05	250.6722	0.609376
cg14526718	2.5861207	0.9533869	7.015011	0.0620122
cg13980454	2.5664533	1.0108607	6.515915	0.0474024
cg05198244	0.7315775	0.3676232	1.455854	0.373355
cg12542238	5.1092997	0.0002053	127133.1	0.7521304
cg19337180	1.0668161	0.6035411	1.885699	0.8238873
cg02250353	27.445339	1.6493019	456.7064	0.0209586
cg01046434	0.5652745	0.2074252	1.540484	0.2647578
cg22572908	2.4797878	1.1079124	5.550391	0.0271569
cg01209635	1.1714912	0.6015261	2.281516	0.6416453
cg06466839	1.4805454	0.3747202	5.849736	0.5756392
cg05796838	0.2842707	0.1259794	0.641452	0.0024509
cg12080492	2.412568	0.8707232	6.684656	0.0903157
cg13227624	1.9744297	0.0161479	241.4165	0.7814616
cg03467809	0.0050558	0.0001479	0.172857	0.003346
cg06094707	1.5826811	0.5862584	4.272654	0.3648841
cg03078854	2.7567546	0.3690782	20.59102	0.3229485
cg27040709	0.3513745	0.1266052	0.975189	0.0446217
cg00089486	1.4456634	0.6439352	3.245579	0.3717322
cg07155664	245.3688	3.1715964	18982.82	0.0131309
cg14919082	0.4005372	0.2058415	0.779386	0.0070642
cg25143508	0.0402393	0.004795	0.337683	0.0030741
cg22506453	0.852654	0.377038	1.928238	0.7018197
cg23017915	0.5747634	0.2117402	1.560181	0.277061
cg19445588	0.2318579	0.0918988	0.58497	0.0019644
cg14599998	0.2891562	0.1257286	0.665014	0.0035003
cg25680629	0.3227792	0.1435726	0.72567	0.0062239
cg25295740	0.3272962	0.1356334	0.789796	0.012955
cg20972917	4.270924	1.1461371	15.91502	0.0305264
cg02939508	0.7556377	0.0635247	8.988447	0.8244814
cg08090396	2.70E+30	3.9266693	1.85E+60	0.0456099
cg23490014	0.3439269	0.0953368	1.240714	0.1030013
cg18931398	0.2826889	0.1170428	0.682768	0.0049829
cg07005444	0.0553147	0.0148123	0.206566	1.66E-05

cg10481202	0.5445782	0.2503853	1.184436	0.1252763
cg24092939	0.3847766	0.1949213	0.759553	0.0059125
cg21442182	1.710344	0.3169981	9.228058	0.5325823
cg03661160	1.16E-10	5.31E-23	255.0132	0.1146213
cg21689902	0.4155186	0.1821891	0.947673	0.0368224
cg15834388	1.490197	0.506652	4.383062	0.4686297
cg01775245	0.349379	0.1608566	0.758848	0.0078777
cg16931068	2.2845106	0.4740738	11.00881	0.3031571
cg07569288	0.9781806	0.3629315	2.636413	0.9652151
cg20818457	0.3167185	0.1368916	0.732774	0.0072217
cg09334074	0.0013883	2.96E-06	0.651112	0.0360207
cg15441473	0.3139634	0.1074788	0.917139	0.0341658
cg04276057	0.2895176	0.0777873	1.07756	0.064521
cg06744585	1.9961741	0.6362416	6.26289	0.2360691
cg21981214	3.699E+13	2.20E-07	6.23E+33	0.1885861
cg12072724	6.54E-05	2.81E-14	152241.7	0.3812847
cg04143626	0.7169171	0.291153	1.765292	0.4691598
cg23871933	1.56E-14	2.21E-32	11051.15	0.1295279
cg26113593	0.3094629	0.0948166	1.010027	0.0519639
cg16498913	1.0712151	0.420626	2.728081	0.8853138
cg06796016	2.2757788	1.81E-07	28577408	0.9214546
cg00722320	1.2584545	0.7003207	2.261403	0.4420419
cg04975846	304.32828	5.13E-05	1.81E+09	0.4723894
cg25220537	0.4966899	0.2367132	1.042193	0.064217
cg05811904	0.7863351	0.3995784	1.547438	0.4864773
cg12123728	0.353687	0.0719113	1.739566	0.2009733
cg23992583	0.8629352	0.4497134	1.655849	0.657529
cg12821724	0.2097335	0.0943167	0.466388	0.0001279
cg18558968	6.4874643	0.1485173	283.3824	0.3318798
cg16853842	0.0593903	0.0036031	0.978926	0.0482839
cg24577369	2.1871475	1.0211902	4.684352	0.0440174
cg17347810	1.4686636	0.4390466	4.912855	0.5327167
cg22324567	1.3216101	0.6847421	2.550819	0.4058861
cg19923622	0.7265265	0.330221	1.598447	0.4271285
cg05802073	0.1515052	0.0404839	0.566986	0.0050682
cg26910994	0.4497056	0.2023245	0.999558	0.0498734
cg04181546	1.2955119	0.5644203	2.973584	0.5413673
cg15246706	0.6495398	0.2461482	1.714016	0.3834451
cg04458670	0.8672549	0.3993967	1.883168	0.7188408
cg22816278	0.0001173	1.93E-07	0.071445	0.0056647
cg16165903	1.0255517	0.4353398	2.415944	0.9539778
cg09711113	3.9833505	0.1366864	116.0839	0.4217959
cg08551636	0.7080078	0.379508	1.320855	0.2777852
cg04422003	1.0889213	0.3836567	3.090652	0.8728408
cg06326667	1.4194839	0.6725014	2.996179	0.3580756
cg15837913	1.6755332	0.771993	3.636576	0.1917435
cg08362210	2.6300057	0.9621671	7.188907	0.059458
cg03117005	0.2292494	0.0740942	0.709303	0.0105887
cg06716698	4.1454507	0.5758715	29.84131	0.1579545
cg08012278	2.4957222	0.7989408	7.796109	0.1155522
cg05635712	3.70E-20	8.98E-46	1524077	0.1370512
cg08894401	0.8444126	0.0004167	1710.95	0.9652766
cg01447831	0.3903795	0.1628089	0.936043	0.0350234
cg17022577	0.4597814	0.1585469	1.333353	0.1526151
cg05049329	0.5494573	0.2958684	1.020397	0.057956
cg14448830	2.4910359	1.1996649	5.172494	0.0143537
cg09319822	0.469042	0.2024938	1.086455	0.0773149
cg14261840	1.4174232	0.7419122	2.707987	0.2908994

cg04740931	1122.1042	0.0447673	28125856	0.1741743
cg17083506	4.46E-17	5.86E-42	3.4E+08	0.1977743
cg17658634	2.5334548	1.1617661	5.524686	0.0194441
cg21221947	2.441E+10	1.06E-11	5.60E+31	0.3405333
cg06087619	1.0054386	0.3321511	3.043515	0.992342
cg04165120	99.04688	2.3948757	4096.365	0.0155283
cg08641750	1.286E+09	0.0055642	2.97E+20	0.1161591
cg04927957	0.213908	0.028617	1.598932	0.132926
cg04779161	0.2072027	0.0795956	0.539388	0.0012615
cg12555844	1.0333516	0.2665476	4.006097	0.9621509
cg21063758	0.0120457	0.0003729	0.389069	0.0126887
cg11211795	3.1762632	0.9454227	10.67104	0.061596
cg23281382	2.5929691	0.9336578	7.201234	0.0675125
cg09535924	2.8126623	0.6458093	12.24985	0.1683512
cg03221914	0.985635	0.3523757	2.756933	0.9780044
cg04115702	0.2968256	0.0978124	0.900759	0.031993
cg03710860	25.729287	0.3246639	2039.02	0.1454723
cg13557397	6670.8172	0.0733398	6.07E+08	0.1306628
cg03579624	2.0750518	0.8988513	4.79038	0.0872394
cg16872841	1.6539257	0.6131177	4.461574	0.320339
cg23625628	0.1173723	0.0065922	2.089777	0.1447659
cg01071279	5409605.7	4.14E-07	7.06E+19	0.3143343
cg09985078	4.023032	1.0312657	15.6941	0.0450388
cg25738279	0.0046581	8.03E-06	2.703174	0.0981919
cg17307989	0.7447701	0.3158827	1.755976	0.5007052
cg11378484	0.0037476	8.58E-05	0.163733	0.0037444
cg16757384	2.5973139	0.7105998	9.493445	0.1489258
cg01389428	3.5780926	0.6632241	19.3038	0.1382218
cg08358964	0.326883	0.1366609	0.78188	0.0119727
cg01895374	0.2369018	0.0870501	0.644715	0.004813
cg25111926	0.434902	0.1981082	0.954729	0.0379458
cg08792703	1.2413769	0.3728954	4.132571	0.7245621
cg00581043	3.6307888	0.7660476	17.20863	0.1043217
cg15817542	0.5130283	0.1744271	1.508929	0.2253022
cg23752348	0.3038442	0.1263717	0.730554	0.0077823
cg05135846	0.3332752	0.1142275	0.972378	0.0443007
cg09394488	2.4993999	1.0834109	5.766049	0.0317294
cg23676577	1.7798566	0.6835986	4.634136	0.237659
cg04452095	0.1471636	0.0099332	2.180268	0.1635467
cg15750501	1.3449786	0.5339087	3.388159	0.5295249
cg09716807	2.025711	0.9344419	4.391397	0.0737435
cg16015593	1.3225117	0.6730838	2.598543	0.4172722
cg10796979	1.52E-27	7.58E-43	3.05E-12	0.0005929
cg19967176	1.990003	0.1673565	23.66273	0.5859118
cg19959591	0.1929527	0.0574383	0.648187	0.0077847
cg26195178	1.7487917	0.7262614	4.21098	0.2125451
cg12114114	0.2294182	0.0569376	0.924393	0.0384024
cg10107292	0.6968654	0.1931654	2.514019	0.5811484
cg03497652	0.9471883	0.103454	8.672119	0.9616972
cg02718124	3.61E+22	1.47E-05	8.89E+49	0.1065054
cg20278381	0.0164151	0.0006488	0.41534	0.0126674
cg18726518	1.4651836	0.6440232	3.333363	0.3624075
cg13097993	2.4626327	0.9644366	6.288189	0.0595305
cg16816874	1.9067601	0.7281331	4.993228	0.1888413
cg22867729	0.7442307	0.0413994	13.37892	0.8411653
cg07611666	0.433559	0.2052191	0.915965	0.0285257
cg20574436	1.0687147	0.5137047	2.223361	0.8588769
cg17367596	0.6193813	0.2354009	1.629701	0.3317982

cg13421924	0.0041919	9.14E-11	192169.8	0.5430206
cg01634213	0.3646867	0.1546365	0.860058	0.0212027
cg04579493	0.9133175	0.4551999	1.832489	0.7985625
cg25310700	2.2477008	0.5446144	9.27658	0.2628055
cg10961323	0.2777192	0.1258314	0.612947	0.0015151
cg26074470	0.8798216	0.4538634	1.705548	0.7046009
cg06163994	0.5544929	0.2755751	1.115712	0.0983232
cg05257202	2.2767175	0.8795565	5.893246	0.0899831
cg14214797	2.1404521	0.7038814	6.508959	0.1798749
cg12212240	573810	136.66851	2.41E+09	0.0018377
cg04467832	1.2883417	0.4784869	3.468902	0.6161318
cg02855078	25646.597	32.386782	20309147	0.0028711
cg07660671	3.2596251	1.1198081	9.488372	0.0301938
cg17469934	1.5092386	0.6913219	3.294849	0.3014771
cg23051559	0.0371393	0.0051997	0.26527	0.0010276
cg18306747	0.0857638	0.004771	1.541692	0.0956553
cg08198228	0.9527425	0.4681924	1.938772	0.893758
cg27141871	1.8465654	0.6396132	5.331041	0.2568693
cg01077100	1.2264131	0.5910923	2.544593	0.5836506
cg21308656	4.6036511	1.0162608	20.85449	0.0476035
cg02670637	0.73588	0.3452112	1.568661	0.4271103
cg07839906	0.5739491	0.2772899	1.18799	0.1346914
cg22631555	1.3378928	0.7221587	2.47862	0.354817
cg05686950	0.1797253	0.0588327	0.549035	0.0025927
cg13928709	0.0308104	0.0022751	0.417253	0.0088607
cg20868668	0.5640666	0.2718198	1.170523	0.1242327
cg04138001	656.10216	0.6055232	710906	0.0688719
cg11658874	0.2538428	0.0978386	0.658597	0.0048242
cg04902851	19.291894	1.6910077	220.092	0.0171761
cg07541160	1.0868771	0.2043153	5.781759	0.9221772
cg13423759	0.2377031	0.0515872	1.095285	0.0652998
cg21380024	1.5706562	0.5703323	4.32548	0.3823746
cg03350366	0.0352108	0.0012507	0.991292	0.0494027
cg03271173	4.2120558	1.1246605	15.77491	0.0328149
cg06980932	0.7001917	0.3740135	1.310831	0.2652888
cg03874201	2.7469348	0.7148682	10.5553	0.1412229
cg19643252	4.9317969	0.7529427	32.30342	0.0961035
cg10406264	0.2504994	0.0432884	1.449578	0.1222336
cg09314135	3.3899345	1.4228128	8.076717	0.0058502
cg22601762	0.6703349	0.3048839	1.473836	0.3197145
cg26337559	5.2353593	0.9350237	29.31368	0.0596294
cg19745903	1.52E-05	4.36E-14	5295.06	0.2689217
cg17588003	1.317021	0.2144695	8.087604	0.7661818
cg27500647	4.5949039	0.6192734	34.09341	0.1358769
cg23378941	0.5333333	0.2668233	1.06604	0.0752429
cg10095539	0.2334242	0.0797697	0.683053	0.0079126
cg09246878	0.8193335	0.2313493	2.901705	0.7574415
cg02577896	0.3746883	0.1823592	0.769862	0.0075442
cg24670453	2.0384155	0.6719375	6.183816	0.2084728
cg22881265	1.2013159	0.4760238	3.031697	0.6977616
cg04445512	1.5162276	0.7363495	3.122085	0.2587008
cg06193520	0.2585534	0.1199875	0.55714	0.0005538
cg22466590	0.3099782	0.1066322	0.901102	0.0314579
cg21207028	8.22E-05	5.22E-08	0.129653	0.0122881
cg22609522	1.6638383	0.7063397	3.919301	0.2441537
cg19647370	0.3471151	0.1317757	0.914348	0.0322608
cg25353401	2.4003792	0.8401362	6.858198	0.1021001
cg04136369	0.9821885	0.4796883	2.011086	0.9607983

cg05344495	0.8673175	0.4567785	1.646837	0.663476
cg00940546	0.0760544	0.0047159	1.226543	0.0693666
cg11903880	0.5368646	0.2640722	1.091458	0.0857569
cg06923967	0.3293076	0.1617651	0.670376	0.0021941
cg02997316	0.0582137	0.0007921	4.278367	0.1946355
cg05257275	0.6703817	0.2747027	1.635993	0.3796454
cg11602901	1.2113536	0.4640882	3.16185	0.695283
cg26151087	0.2679488	0.0014635	49.05735	0.6202928
cg07720334	0.3757198	0.2058815	0.685663	0.0014251
cg19597545	0.2161085	0.0462974	1.008758	0.0513108
cg14152268	0.3252645	0.1324899	0.798529	0.0142484
cg11801651	28120130	1.97E-07	4.01E+21	0.3023079
cg27443224	1.0065448	0.3729852	2.716281	0.9897241
cg05941890	1.73E-15	4.56E-29	0.065704	0.0331201
cg21121843	1.4968296	0.6324116	3.542786	0.3588412
cg05821186	2.6687235	0.1933702	36.83135	0.4635677
cg21815337	0.4190627	0.2004206	0.876225	0.0208291
cg00147638	2.3142886	0.5690541	9.41199	0.2410733
cg02143404	0.3799108	0.1350159	1.069002	0.0667206
cg27617780	1.6025042	0.6325542	4.059763	0.3200785
cg24498500	0.5048651	0.1964322	1.297592	0.1558787
cg09596958	0.273692	0.1032965	0.725168	0.0091513
cg04460771	2.0168324	1.012241	4.018424	0.0460914
cg24136754	1.5619078	0.6602144	3.695096	0.3101343
cg26944851	1.895744	0.9559711	3.759366	0.0670916
cg11588610	0.7024901	0.3401504	1.450806	0.3399244
cg17492855	9663.6196	144.27289	647284.1	1.89E-05
cg00215182	0.4820764	0.1908012	1.21801	0.1228487
cg04494791	0.3672827	0.1039507	1.297698	0.1198704
cg25054754	1.5553614	0.7733335	3.128209	0.2153577
cg14186846	0.3449457	0.1552829	0.766263	0.0089558
cg14010318	1.5246651	0.7445613	3.122112	0.2487569
cg11099722	1.0807188	0.5080098	2.299076	0.84027
cg16217751	0.1327081	0.0165537	1.0639	0.0572182
cg24500959	0.0995485	0.0193851	0.511211	0.0057144
cg24083274	0.9337056	0.4359604	1.999737	0.8598827
cg24182468	4.2668218	1.6938945	10.74788	0.0020834
cg16182707	0.7017519	0.2782603	1.769767	0.4529919
cg01614898	0.4913563	0.1700871	1.419455	0.1892418
cg10242160	2.883111	0.8712935	9.540216	0.0828644
cg03248820	1.0258971	0.5590447	1.882613	0.934215
cg05808509	69212.49	3.32E-19	1.44E+28	0.684139
cg04661888	1.1829389	0.5677021	2.464927	0.6537857
cg23110005	2.1538361	0.5773355	8.035206	0.2533762
cg06600725	1.5196264	0.7690254	3.002845	0.2285124
cg27574000	5.09E-06	2.66E-10	0.097296	0.0153847
cg07617759	0.2807184	0.1255756	0.627533	0.0019666
cg00565882	2.5811726	0.8142868	8.181947	0.107192
cg07862575	69.846994	0.0081131	601322.9	0.3583303
cg18561589	1.8679022	0.9517445	3.665962	0.0693399
cg01231108	2.2131435	1.1678287	4.194112	0.0148657
cg18291664	1.3168201	0.1777315	9.756374	0.7876629
cg24663460	0.4433792	0.187492	1.048499	0.064009
cg13100449	1.5680254	0.7380154	3.331507	0.2420521
cg01809217	1.3281549	0.4923399	3.582881	0.5751437
cg18860310	0.2333269	0.0805386	0.675967	0.0073284
cg14063357	792.74745	5.3590338	117269	0.0088326
cg12847373	1.71E-10	1.77E-20	1.658491	0.0552584

cg12188614	0.4502388	0.2165801	0.935981	0.0325854
cg02607059	0.191454	0.0628487	0.58322	0.0036295
cg16277214	1.2462514	0.4713841	3.294855	0.6571912
cg26692085	0.2677125	0.0986988	0.726148	0.0096392
cg11813497	0.6786321	0.3135386	1.468851	0.3250971
cg18380123	2.700588	1.2609343	5.783946	0.0105694
cg10452667	0.526954	0.2359092	1.177065	0.1181976
cg06086012	0.2266111	0.0886177	0.579485	0.0019421
cg25895907	0.1029995	0.0107062	0.99091	0.049083
cg08949655	2.0664529	0.9885248	4.319798	0.0536953
cg02329835	2.1125181	0.4182057	10.67114	0.3654574
cg22450733	0.1393303	8.34E-05	232.8369	0.602701
cg19375403	2.2781565	0.8644129	6.004072	0.0958582
cg23289581	5.7573313	0.9590455	34.56235	0.0555901
cg15225091	1.8333456	0.8946938	3.756767	0.0977285
cg19583819	1.8167006	0.8561795	3.854801	0.119845
cg25233139	1.4956471	0.8297761	2.69586	0.1805055
cg16674264	0.2961566	0.1336624	0.656196	0.0027188
cg00286067	0.0230426	0.0033409	0.158925	0.0001298
cg11254053	0.4053686	0.1849079	0.888678	0.0241549
cg02249969	0.2462161	0.0919198	0.659514	0.0053038
cg24879257	0.0006504	8.28E-13	510989.5	0.4825646
cg17764549	0.136224	0.01325	1.400527	0.0936113
cg19595760	4.3486513	0.6173679	30.63128	0.1400124
cg04566359	0.3230789	0.1070333	0.97521	0.0450167
cg04362096	0.1893532	0.0618341	0.579852	0.0035639
cg15282732	0.8769251	0.3471278	2.215315	0.7811956
cg07196216	2.5594632	0.9826415	6.666574	0.0543405
cg02724608	1.69E-14	2.32E-28	1.226704	0.0514848
cg14563637	0.376074	0.139167	1.016273	0.0538379
cg23878875	0.3050873	0.1231698	0.755691	0.0103096
cg09976157	0.4480355	0.2200774	0.912115	0.0268575
cg07102001	1.6494447	0.1631328	16.67763	0.671609
cg04966159	0.2081265	0.0866236	0.500056	0.0004489
cg04887328	9.29E+26	923430.41	9.34E+47	0.0118474
cg00103209	0.3580541	0.0012639	101.4348	0.7214596
cg08430407	1.2331516	0.3204871	4.744849	0.7604947
cg25203361	0.4875819	0.2160686	1.100281	0.0836623
cg22576265	0.4294424	0.2056602	0.896726	0.02444
cg03257179	0.5113283	0.2390302	1.093823	0.0838413
cg21249093	1.9295088	0.7355444	5.061563	0.1816289
cg23233975	5.8219172	1.4968494	22.64404	0.0110217
cg15895339	0.7185981	0.3676705	1.404473	0.3337866
cg06812019	5.2013862	1.1906704	22.722	0.0283829
cg10268548	2.0389868	0.7332836	5.669658	0.172121
cg13858127	2.4934766	0.8297948	7.492726	0.1036095
cg21495622	0.1863693	0.0725218	0.478939	0.0004854
cg06144920	0.5197205	0.2696434	1.001728	0.0506059
cg15155738	1.92E+23	4.57E-07	8.05E+52	0.1234472
cg20658798	1059.6034	0.2286039	4911375	0.1058104
cg24801587	3.0054532	1.3367362	6.757316	0.0077662
cg06081609	49646.266	0.4980296	4.95E+09	0.0655838
cg21544633	0.1830847	0.0351104	0.954703	0.0439069
cg14489180	135.73582	1.6012441	11506.19	0.030175
cg01858157	1.6083047	0.7494496	3.451391	0.2225896
cg02431672	0.3358719	0.1241817	0.908426	0.0316225
cg00477365	0.0968158	0.0139898	0.670009	0.0179959
cg26490671	0.417409	0.2300796	0.757261	0.0040418

cg06567342	529.66862	0.2857752	981711.7	0.1023185
cg01909777	2.8329844	1.1884535	6.753147	0.0187979
cg01423695	2.8890264	1.1314359	7.376886	0.0265447
cg20006741	3.9068089	0.8908595	17.13307	0.0708029
cg02763019	0.0006295	2.81E-06	0.141265	0.0076178
cg06087019	2.0548784	0.926986	4.555112	0.0761805
cg03453431	0.1869959	0.0414048	0.844526	0.0292846
cg17424654	1.1808504	7.95E-13	1.76E+12	0.9907249
cg20446404	2.3930705	0.9848451	5.814911	0.0540728
cg13934406	0.3224921	0.1037829	1.002103	0.050426
cg00228799	7.492E+10	636.44091	8.82E+18	0.00827
cg05995866	1.8525315	0.9210684	3.72597	0.0837473
cg10756887	1.7708344	0.6489233	4.832396	0.2645586
cg24483767	2.6562613	0.2826171	24.96567	0.3927909
cg16550446	0.7803574	0.4124444	1.47646	0.4458843
cg13392422	0.6690014	0.3431103	1.304429	0.2380489
cg12859046	0.4472313	0.2178419	0.91817	0.0283371
cg05405872	0.2688556	0.0976944	0.739892	0.0109838
cg00347620	4.7549329	1.4423901	15.67495	0.0104126
cg07906828	0.4659373	0.218139	0.995226	0.0485727
cg25233555	3.4110943	0.8056289	14.44283	0.0956268
cg21212196	13.524573	2.0366298	89.81214	0.0070106
cg12884169	0.3761408	0.0879138	1.609325	0.1873694
cg16128096	2.5312399	0.6951992	9.216316	0.1589648
cg22839075	2.2500091	1.1316983	4.473402	0.0207327
cg00579794	40.800161	2.58E-05	64437532	0.6105471
cg18160302	0.4737774	0.2188226	1.025786	0.0580436
cg01819142	0.6191371	0.2947668	1.300454	0.2054597
cg17201651	0.2227081	0.0633194	0.783313	0.0192547
cg06823681	1.932872	0.8100246	4.612198	0.1375045
cg17641710	1.7893541	0.3977475	8.0498	0.4482362
cg07951201	2.2496157	0.7685091	6.58518	0.1390107
cg14355941	0.4739283	0.2269396	0.989726	0.0468724
cg05928537	1.9779995	0.487783	8.020948	0.3396167
cg11101117	2.2130444	1.0944714	4.474823	0.0270186
cg00840926	4.18E-09	1.25E-20	1403.65	0.1542259
cg14404318	1.2920733	0.2095066	7.968501	0.7824955
cg02711476	2.85E-07	3.17E-20	2572531	0.3221112
cg20477259	2.165595	0.6883428	6.813177	0.1863929
cg01472299	1.4320915	0.5153365	3.979703	0.4910163
cg07631919	0.0750155	0.0125909	0.446936	0.0044498
cg20247455	3.0495695	1.3326501	6.978482	0.0082939
cg25122233	0.3483142	0.1387416	0.874451	0.0247282
cg09388920	0.2630997	0.1044004	0.663038	0.0046356
cg19320294	0.5796245	0.2983894	1.125926	0.1074288
cg18094999	0.0514838	0.0051499	0.514686	0.0115565
cg25421389	0.5051551	0.1595228	1.599657	0.2455787
cg02693210	1.3926248	0.5369973	3.611571	0.495765
cg04543115	0.1264664	0.0215467	0.742284	0.0220203
cg16659407	0.0074509	0.0004412	0.125831	0.0006807
cg21970554	0.1890217	0.0257073	1.389848	0.1017213
cg08605773	0.3393568	0.0297225	3.874611	0.3843991
cg24590353	0.3309534	0.142856	0.766717	0.0098895
cg07628084	0.350307	0.1509882	0.812745	0.0145731
cg17904575	1.3214713	0.5679576	3.074677	0.5176552
cg15815156	3.53E-37	1.66E-90	7.51E+16	0.1803327
cg07579404	1.2380617	0.6268698	2.44516	0.5385567
cg10503827	0.5826938	0.2176596	1.559922	0.2823839

cg25948980	0.4186404	0.0983558	1.781896	0.2386906
cg05229035	0.301044	0.1369796	0.661613	0.0028068
cg27482619	2.4140703	0.9981954	5.838271	0.0504704
cg11351527	1.7675155	0.519401	6.014834	0.362
cg20775109	2.7888179	0.4911503	15.83529	0.2470604
cg26564730	1.53E-09	1.07E-16	0.022027	0.015791
cg24951886	2.8262142	1.1665464	6.847123	0.0213822
cg17091577	0.1077034	0.0210523	0.55101	0.0074601
cg16375048	0.6836944	0.3066441	1.524366	0.3526491
cg04671611	1.5037991	0.6519225	3.468835	0.3387056
cg27554649	1.8425561	0.6407279	5.298681	0.2567983
cg13807343	0.3936932	0.1504271	1.030362	0.0575603
cg17280740	2.7033029	1.0832127	6.746455	0.0330673
cg02633767	0.3471329	0.1415374	0.851374	0.020806
cg08307469	1.6770107	0.7363812	3.81917	0.2182371
cg11655394	2.6290266	0.0017412	3969.464	0.7957716
cg13868356	5.1900191	0.7457291	36.12076	0.096198
cg08796741	2.34E-10	1.43E-22	383.0089	0.1222462
cg03980550	0.2404156	0.0882504	0.654951	0.00531
cg22290648	1.9322937	0.9191398	4.062232	0.08229
cg20614854	4.8474951	1.1974044	19.62429	0.0269333
cg21765730	0.3891945	0.1960523	0.772612	0.0069893
cg10107186	11028.729	1.80E-05	6.77E+12	0.3672717
cg00297832	1.7234091	0.7185266	4.133652	0.2226853
cg02593507	0.3426106	0.1290613	0.909506	0.0315248
cg00119053	9.0184748	1.5762212	51.59992	0.0134634
cg17660703	0.010279	6.36E-05	1.66088	0.0776627
cg00470044	0.2855658	0.1374259	0.593395	0.0007836
cg18237323	1.2095039	0.483627	3.024852	0.6842258
cg18108513	1.3076638	0.5988417	2.855487	0.5008398
cg18147280	4.0454562	0.0471383	347.1848	0.5383926
cg11384750	64.805764	0.0392125	107103.1	0.2698875
cg24834889	0.085975	0.0141271	0.523229	0.0077463
cg08719869	0.1557089	0.0296654	0.81729	0.0279156
cg05812089	0.3058337	0.082297	1.136545	0.076917
cg01004056	1.4662298	0.6833189	3.146159	0.3258922
cg20000464	0.0172326	0.0013855	0.214342	0.0015913
cg11753750	1.8290917	0.5445456	6.143795	0.3286882
cg11639612	0.3817291	0.1145746	1.27181	0.1167906
cg08316234	0.5339636	0.2667731	1.068763	0.0763724
cg11319427	0.2467751	0.086204	0.70644	0.0091188
cg26326607	0.4724183	0.2117224	1.054112	0.06706
cg09096671	0.1854298	0.0657777	0.522734	0.001439
cg03950614	0.1219103	0.0194666	0.763465	0.0245573
cg06375949	0.9637715	0.4421013	2.101001	0.926058
cg14623306	0.1341628	0.0398555	0.451623	0.0011806
cg23063807	0.5029633	0.146029	1.732341	0.2760887
cg19435409	0.2301733	0.009623	5.505563	0.3644735
cg13374225	1.30E-10	4.76E-21	3.548982	0.0633603
cg19071452	1.0910006	0.5717881	2.081684	0.7916155
cg01807131	0.153432	0.0349538	0.6735	0.0130029
cg03482600	11.552608	1.8285968	72.98643	0.0092766
cg18149485	1.8887832	0.8368425	4.26305	0.1257416
cg00207280	0.1095808	0.0236474	0.507793	0.004711
cg22308970	2.3817707	1.0682524	5.310385	0.0338921
cg06830360	3.2328066	1.1504879	9.084006	0.0260206
cg01392313	2.4449879	0.827843	7.221134	0.105655
cg09997676	1.2531352	0.5332879	2.944653	0.6046921

cg11708963	4.90E-13	2.59E-34	9.28E+08	0.2568204
cg17500055	1.6318064	0.6727655	3.95798	0.2787172
cg11228197	1.5508679	0.430223	5.590569	0.5023893
cg19158553	0.80743	0.2248697	2.899204	0.742947
cg02737830	1863566.6	2.94E-07	1.18E+19	0.3370486
cg17739208	0.4448541	0.1584811	1.248699	0.1240008
cg20540608	0.3680897	6.25E-12	2.17E+10	0.9370408
cg18871289	0.3363702	0.0904468	1.250955	0.103983
cg26483578	0.0755349	0.009405	0.606646	0.0150916
cg01816880	6.5171137	0.9976455	42.57301	0.0502885
cg17332306	0.7986724	0.2567506	2.484425	0.6978279
cg17449088	0.2835058	0.1222275	0.657589	0.0033199
cg01826574	1.4434848	0.796896	2.614705	0.2259082
cg26931307	0.4669458	0.2083524	1.046488	0.0643711
cg02006647	0.6109374	0.2820667	1.323249	0.2114275
cg26548288	0.1191158	0.0245726	0.577414	0.0082443
cg07293736	1.0542828	0.4845089	2.294101	0.8939896
cg23298564	38079426	406.5602	3.57E+12	0.0028028
cg06838175	0.0373825	0.004482	0.311789	0.0023907
cg17085250	3.6543696	0.6492313	20.56958	0.1415671
cg07887978	1.1821813	0.5249761	2.662126	0.6861498
cg03020503	1.3495185	0.6245672	2.91594	0.4457374
cg04878685	2.2818367	1.0653924	4.887193	0.0337565
cg06246882	2.4722098	0.5743711	10.64089	0.2242137
cg13506354	0.3132155	0.1475236	0.665005	0.0025114
cg01421943	0.6749091	0.3564342	1.277942	0.227414
cg17727529	1.6233007	0.5958161	4.422682	0.343454
cg02858288	6.227614	1.041546	37.23616	0.0450082
cg19308132	1.3585639	0.7027567	2.626365	0.36223
cg07385220	0.9626615	0.0044982	206.0189	0.9889104
cg05347635	1.3630594	0.4762659	3.901037	0.5637202
cg08900101	1.8664276	1.0317494	3.376355	0.0390832
cg16719194	0.2013476	0.0605347	0.669713	0.0089548
cg07897837	357800995	20768.209	6.16E+12	7.57E-05
cg11866396	0.107697	1.65E-06	7033.01	0.6936178
cg22508782	1.8363331	0.7363167	4.579713	0.1924098
cg13692655	0.3704716	0.1034137	1.327186	0.1272118
cg02285920	1.522691	0.7478159	3.100479	0.2464647
cg12478032	0.486868	0.1983801	1.19488	0.1161176
cg23054456	0.6458319	0.2089021	1.996623	0.4477124
cg08110272	1.6304302	0.6927536	3.837299	0.2629724
cg06007395	0.0153951	0.0003239	0.731813	0.0341376
cg09247486	1.6851076	0.1755319	16.17705	0.6511258
cg17530152	0.5683898	0.1500123	2.153603	0.4058419
cg25751482	0.9664382	0.4829361	1.934009	0.9231647
cg25112002	1.2203514	0.0425797	34.9758	0.907401
cg08312412	0.2120213	0.0455572	0.986739	0.0480439
cg13504059	2.3533546	0.9209497	6.013659	0.0737876
cg15563027	1.2572255	0.4750389	3.32734	0.6448174
cg25930842	0.9938092	0.0001531	6451.262	0.9988937
cg06696963	0.1982538	0.0670891	0.585856	0.003421
cg11188429	0.5686194	0.0794694	4.068585	0.573922
cg25223263	0.0002813	4.02E-06	0.019677	0.0001616
cg17175208	0.9596772	0.4681731	1.967179	0.9105144
cg21023001	2.2730281	0.8686707	5.947774	0.0943096
cg21542223	1.7150983	0.6888642	4.270163	0.2464007
cg00155732	2.4301378	0.7471041	7.904614	0.1400791
cg00183804	6.46E-27	6.40E-59	652392	0.1087408

cg04330811	4.28E-06	2.81E-12	6.5044	0.0887289
cg27195326	2.0217004	0.7507713	5.444098	0.1636812
cg13468685	3.809387	0.7825859	18.54292	0.0976488
cg25334860	0.6009199	0.2623889	1.37622	0.2283462
cg00499290	0.6497174	0.2476358	1.704651	0.3809169
cg15590113	22.081739	2.2983012	212.1581	0.0073439
cg10052038	0.2815198	0.0336086	2.358129	0.2424529
cg01012295	1.7638702	0.5403603	5.757711	0.3471082
cg24126567	0.2627189	0.1100151	0.627379	0.0026152
cg27464846	1.4615165	0.6422317	3.32595	0.365728
cg01071185	0.3657752	0.1296424	1.032004	0.0573758
cg20203041	1.2778227	0.4229498	3.860578	0.6638655
cg07932479	2.398E+14	19.410405	2.96E+27	0.0313348
cg17945962	0.1866962	0.065926	0.528706	0.0015779
cg17402907	0.5030115	0.260612	0.970871	0.0405526
cg05516285	0.5080408	0.0838765	3.077209	0.4611972
cg01533585	1.6915285	0.2094273	13.66235	0.6218981
cg26526853	0.7382841	0.3075031	1.772546	0.4971325
cg06874426	0.957776	0.5044673	1.818423	0.895072
cg24502904	0.2523673	0.0290614	2.191542	0.2118457
cg14642636	843.98891	8.2793632	86035.27	0.0042922
cg12931642	1.0318011	0.512272	2.078219	0.9301715
cg18671854	1.4729405	0.7975951	2.720119	0.2159523
cg11635197	0.4674528	0.2027882	1.077539	0.0743099
cg20397543	3.38E-08	2.93E-14	0.038995	0.0157128
cg21398794	1.7653966	0.8227277	3.788161	0.1445503
cg06617975	0.332228	0.1130577	0.976276	0.0451107
cg25303599	0.395455	0.1727162	0.905443	0.0281649
cg25102370	0.503023	0.1589479	1.591919	0.2424135
cg15522517	0.4243528	0.0771136	2.335195	0.3245217
cg05975755	0.5057403	0.2750119	0.930044	0.0282867
cg10601943	4.1385091	1.6058872	10.66529	0.0032751
cg10078540	2.3926654	0.8577224	6.674476	0.0955645
cg21160099	1.8857847	0.8355466	4.256117	0.1266714
cg19138900	2.664E+11	5799.0335	1.22E+19	0.0034711
cg27179810	0.2639785	0.1007602	0.69159	0.0067203
cg19430967	0.3456892	0.0579877	2.060801	0.2435618
cg09971549	2.2327533	0.6412475	7.774201	0.2069855
cg14462015	0.0070099	1.23E-05	3.993935	0.1254676
cg15744108	0.6718365	0.0285103	15.83162	0.8051291
cg00448868	0.6157447	0.2447292	1.549229	0.3029739
cg01753270	0.0197581	0.0006594	0.592012	0.0236873
cg06088759	0.1797733	0.0571855	0.565151	0.0033198
cg26659665	0.5628012	0.2880513	1.099614	0.0925515
cg16278747	0.1351991	0.0501606	0.364406	7.64E-05
cg24269082	0.1005971	1.09E-06	9281.718	0.6937788
cg02870945	1.7782199	0.7392143	4.277604	0.1986996
cg02504211	1.4886866	0.2004264	11.05737	0.6973371
cg02632542	0.3389818	0.1213296	0.947079	0.0390474
cg12159259	2.1838953	0.8135354	5.862558	0.1210541
cg19580937	2.1356663	0.6518331	6.997298	0.2101484
cg20716064	6.56E-31	9.14E-52	4.71E-10	0.004561
cg25661961	0.3999225	0.1133836	1.410592	0.1541406
cg14475854	4.3901441	0.809041	23.82248	0.0864567
cg13676996	0.1660342	0.0605356	0.455391	0.0004867
cg27611624	0.3916858	0.1086924	1.411485	0.1518472
cg11882377	0.1782843	0.0377192	0.842682	0.0295587
cg02659920	1.4248738	0.663806	3.058522	0.3635906

cg27185772	4.4561522	3.36E-16	5.91E+16	0.9371197
cg26609642	2.0148701	0.6319869	6.423711	0.2363163
cg27200236	1.4964966	0.7411272	3.021751	0.2608512
cg17215863	1.4891802	0.7662659	2.89411	0.2401288
cg25754958	6.1943847	1.0681381	35.9227	0.0420055
cg18173044	0.4729008	0.2255842	0.99136	0.047374
cg17390350	3.6299947	0.8952029	14.71941	0.0710789
cg08548429	1.1967721	0.5697219	2.513969	0.6352644
cg05710204	1.329671	0.0154165	114.684	0.9002928
cg15132673	0.183139	0.0287485	1.166665	0.0723676
cg23038074	1.5011423	0.5395708	4.176335	0.4364921
cg02904285	1.0621622	0.349918	3.224151	0.9152248
cg13390284	0.3418343	0.1469325	0.795268	0.012713
cg10759602	3.7120064	1.4037358	9.815944	0.0082053
cg11784990	0.4562573	0.1551969	1.341333	0.1538051
cg25587431	0.2462987	0.1067074	0.568499	0.001026
cg23931558	2.17E-07	1.75E-14	2.696802	0.0656323
cg13861413	0.7186378	0.3261259	1.583561	0.4124275
cg13868001	0.2822897	0.090407	0.88143	0.0294644
cg08345626	5.3594951	0.932735	30.79566	0.0598485
cg11810743	2.5593621	1.0800774	6.06469	0.0327635
cg23229984	34.900309	0.6121044	1989.908	0.0850642
cg22360028	0.6310143	0.3377294	1.178988	0.1488294
cg17545652	0.5762004	0.2035699	1.630924	0.2990266
cg09000257	0.4519599	0.2139364	0.954806	0.0374195
cg17838516	1.6205407	0.7223841	3.635396	0.2415625
cg17147442	8.2110202	0.8573147	78.6419	0.0677873
cg27340506	3.2005329	1.1813848	8.670681	0.0221509
cg16401360	1.9563096	0.786611	4.865362	0.1488465
cg08113628	21.222674	0.5782392	778.9197	0.0965177
cg20693607	1.4425902	0.7015324	2.966458	0.3191389
cg04745100	6.1314609	1.332354	28.21684	0.0198911
cg16365601	7.0214499	0.2304185	213.9618	0.2635799
cg22043118	0.6797352	0.3220597	1.434641	0.3110787
cg24892948	1.873175	0.450454	7.789441	0.3880407
cg03912703	2.7907704	1.0363122	7.515496	0.0423024
cg08786674	0.2700638	0.0954377	0.76421	0.0136379
cg01799734	0.840007	0.2812332	2.508992	0.7548242
cg18087326	1.9844361	0.4654763	8.460124	0.3542651
cg24660635	0.3632687	0.1859281	0.709759	0.0030448
cg10476003	2.7415993	0.454813	16.52628	0.2711736
cg20495333	4.7118855	0.8321062	26.68153	0.0797388
cg27545615	2.7753425	0.573777	13.42425	0.2043573
cg14241045	0.2345924	0.0669569	0.821925	0.0234196
cg12849795	3.5E+14	237945.43	5.15E+23	0.0018746
cg08289323	160.22373	0.3595007	71409.17	0.1028408
cg05820435	0.424168	0.1874453	0.959845	0.039559
cg03290977	0.8724083	0.4507492	1.688514	0.6853771
cg17123655	3.5474131	1.084568	11.60291	0.0362393
cg10808810	0.9539734	0.1364397	6.670091	0.9621243
cg16005818	12.015745	1.4807577	97.50287	0.0199413
cg12536028	0.4057153	0.1644161	1.001148	0.0502918
cg15177964	0.0022208	2.95E-05	0.167406	0.0055989
cg16928994	0.025924	0.0001041	6.453327	0.1944346
cg10044101	2.0373418	0.7353048	5.644953	0.1711126
cg09652086	2.0835596	1.0303102	4.213508	0.0410456
cg25406518	0.4065258	0.1139462	1.450362	0.1654358
cg21339799	1.9683632	0.5882718	6.586163	0.2717845

cg13353550	1.9125051	0.1661485	22.0145	0.6029613
cg02992639	0.2708914	0.1013527	0.724027	0.0092207
cg08767820	0.7061152	0.170736	2.92029	0.6309342
cg03737367	1.2250086	0.3433213	4.370967	0.7545058
cg21570702	0.0835449	0.0106919	0.652808	0.0179555
cg08388802	0.3571826	0.1328854	0.960071	0.0412771
cg26996818	0.5599029	0.2562383	1.223436	0.1458625
cg15890009	1.6032004	0.716964	3.58491	0.2503151
cg03623982	1.8255679	0.7397308	4.505285	0.1915914
cg23033024	3.8317858	0.9232807	15.90262	0.0643088
cg04907257	4.0184271	1.4682628	10.99787	0.0067761
cg06607764	0.436345	0.2051529	0.928074	0.0312542
cg27325833	4.679212	0.8760791	24.99206	0.0710439
cg11364274	0.3127202	0.1463985	0.667998	0.0026832
cg12145928	0.2715673	0.0691036	1.067221	0.0619302
cg01526089	4.1987879	0.8722906	20.21095	0.0735265
cg07480567	0.3136147	0.0611932	1.607274	0.1642847
cg01040850	2.0355929	0.8088324	5.122988	0.1311923
cg19583967	0.0141551	0.0008296	0.241512	0.0032651
cg21992932	0.1477774	0.0342919	0.636833	0.0103055
cg21223227	0.2471194	0.002936	20.7995	0.5365273
cg00950343	0.5398993	0.2777442	1.049495	0.0691386
cg15108640	0.803273	0.376936	1.711823	0.5704019
cg23244600	1.8483	0.6774403	5.042825	0.230333
cg26921586	6.49E-07	8.77E-19	480292.8	0.3068852
cg26510404	0.2386992	0.1097424	0.519191	0.0003024
cg17798857	0.4388685	0.2133724	0.902673	0.0252047
cg22257574	0.2496752	0.0897975	0.694203	0.0078252
cg14259520	3.7443068	0.0045043	3112.511	0.7003167
cg15059474	1.3855127	0.4688033	4.094778	0.5553534
cg17174764	0.5030996	0.2273895	1.113109	0.0899821
cg12401918	0.1063519	0.0199946	0.565688	0.0085868
cg09495482	2.2918152	0.6482115	8.102937	0.1980515
cg21864713	5.0242135	0.6192559	40.76299	0.1307128
cg11064966	0.3906004	0.1235976	1.234398	0.1093178
cg21773665	1.91757	0.0034846	1055.236	0.8397509
cg11764177	2.1639537	0.8032124	5.829959	0.1268614
cg02231590	3.9746085	1.2391966	12.74819	0.0203069
cg16583186	0.41191	0.1690372	1.003743	0.0509687
cg09461388	1.3770002	0.438466	4.324462	0.58376
cg01475649	0.0536475	0.0002824	10.19287	0.2745159
cg00909806	2.1393455	0.7970601	5.7421	0.1311231
cg11046380	0.2577614	0.0934564	0.71093	0.0088166
cg03432464	0.3870923	0.0926024	1.618105	0.1934251
cg14325778	0.4044549	0.145552	1.123885	0.0825664
cg27225130	5.7929654	0.7581986	44.26076	0.0904254
cg17808011	30268.274	0.001687	5.43E+11	0.2259944
cg10422709	4.0157876	1.1507857	14.01351	0.0292415
cg11784564	0.2261767	0.0734242	0.696717	0.0096112
cg21158528	1.3936569	0.4283799	4.534012	0.5813016
cg19768607	1.4390177	0.247779	8.357334	0.6851088
cg11661263	887816.27	1.8965431	4.16E+11	0.0397785
cg07105221	0.2582664	0.1051808	0.634161	0.00314
cg09251680	1.6162788	0.5142257	5.080176	0.4112458
cg06223539	1.6568692	0.723829	3.79263	0.2320733
cg20954870	0.480459	0.1704675	1.354164	0.1655966
cg20525449	3.7276372	1.4749262	9.421	0.0054116
cg01643712	0.3237554	0.0213816	4.902235	0.4159883

cg14564558	9.129E+14	79873.128	1.04E+25	0.0035537
cg02231404	1.0934793	0.4478178	2.670052	0.8444561
cg24706505	1.3876846	0.1690805	11.38906	0.7603207
cg26134692	0.2132863	0.0791361	0.574846	0.0022548
cg24635754	3.163515	0.4090016	24.46892	0.2698511
cg01518459	0.2095852	0.094608	0.464294	0.0001178
cg03306082	2.3621125	0.8338166	6.69161	0.1056876
cg15244756	5.6229293	0.003674	8605.714	0.6444166
cg09425898	1.9003634	0.3167123	11.40272	0.4824922
cg07751315	8.47E-11	1.73E-20	0.413513	0.0415953
cg08153621	0.3205419	0.1079663	0.951659	0.040442
cg01177854	2.7074435	0.777753	9.424908	0.1175774
cg01172640	1.3773643	0.6450517	2.941055	0.4081132
cg16987524	2.9121393	0.6138724	13.81485	0.1784157
cg04434339	0.0152599	0.0009345	0.249199	0.0033352
cg00122683	9.82E-05	5.17E-07	0.018647	0.0005658
cg08004525	2.0303584	0.9015767	4.572384	0.0872991
cg05659262	1.6598061	0.2761746	9.975417	0.5797471
cg17181966	0.4038218	0.1407053	1.158961	0.0918506
cg02094273	0.3256392	0.1563661	0.678158	0.0027211
cg10930664	2.4459304	0.5867665	10.19584	0.2194455
cg18677906	0.1679626	0.0369621	0.763252	0.0209022
cg05352250	6.46E-10	2.58E-23	16204.69	0.1788848
cg24188163	0.4800882	0.2450675	0.940495	0.0324535
cg22905292	1.4406067	0.3335909	6.221236	0.6247678
cg13739066	1.1940322	0.2567795	5.552284	0.8210806
cg23804764	2.1325934	0.7397728	6.147773	0.1609187
cg16646597	0.0061943	0.0003138	0.122264	0.0008348
cg00376625	2.86E-06	1.10E-11	0.739654	0.0447087
cg26086649	0.12881	0.0214354	0.774049	0.0250982
cg21216100	1.1515622	0.3785103	3.503459	0.8036779
cg14530382	0.2689509	0.0877726	0.824113	0.0215301
cg07237926	1.5649297	0.6080392	4.027709	0.3531547
cg11808658	6.2174337	0.6639998	58.21761	0.1093385
cg16436377	1.7821406	0.5346452	5.940435	0.3468905
cg19413841	0.0045953	0.0001426	0.148063	0.0023812
cg23613051	0.6340816	0.3486225	1.15328	0.1355155
cg23742887	2.319E+13	9.24E-43	5.82E+68	0.6363223
cg19660531	0.4402617	0.1998403	0.969926	0.041777
cg02836919	1.2182403	0.505867	2.933794	0.6597721
cg13434706	0.2255663	0.0394794	1.288778	0.0940003
cg00348771	1.663E+09	2864.5093	9.66E+14	0.0017156
cg11176853	2.1392319	0.7985957	5.73045	0.1303782
cg12790592	0.0063161	0.0002234	0.178541	0.0029733
cg11991105	0.0005743	8.50E-06	0.038807	0.0005176
cg12822818	0.0084107	2.23E-05	3.17052	0.1144002
cg24506604	2.9063506	0.3737448	22.60064	0.3079643
cg16316162	0.4770146	0.124269	1.831051	0.2807799
cg08419918	0.1317905	0.012863	1.350283	0.0878215
cg23192346	1.3100372	0.6895859	2.488736	0.4094773
cg13674483	0.120366	0.0185089	0.782755	0.0266662
cg19768356	0.0238233	0.0002571	2.207712	0.1058261
cg07096032	0.177412	0.0331447	0.949625	0.0433469
cg12259903	1.6283306	0.5873431	4.51433	0.3486927
cg07520093	0.0271405	8.62E-09	85429.89	0.6365973
cg21336434	0.4586341	0.1784929	1.178451	0.1054607
cg03642503	1.6289546	0.3731358	7.111333	0.5163922
cg23003315	0.1683492	0.0339816	0.834023	0.0290903

cg03609639	3.1863737	1.1584114	8.764569	0.0247806
cg08258526	4.2178545	1.6573249	10.73434	0.0025279
cg25938646	0.2386264	0.0593025	0.960205	0.0436819
cg05471373	0.5179633	0.2523164	1.063292	0.073017
cg05418487	1.2431455	0.4790894	3.225725	0.6546056
cg26272069	2.0616732	0.3757874	11.31091	0.4048132
cg01800521	0.1309594	0.0264663	0.648007	0.0127115
cg07902731	1.477774	0.0138379	157.8138	0.8698299
cg15391590	0.417987	0.1808057	0.966303	0.0413372
cg18668503	2.73E-13	1.50E-25	0.497352	0.0445951
cg27486786	0.2375589	0.0645123	0.874782	0.0306869
cg15914766	2.602926	0.7006243	9.670267	0.1531084
cg22006672	0.472444	0.2281744	0.978214	0.0434579
cg06070324	0.3497875	0.1064175	1.149729	0.0836033
cg24794052	0.4100606	0.194321	0.865319	0.0193036
cg25153976	0.0959787	0.0047196	1.95182	0.1272979
cg22087209	0.8977064	0.3446054	2.33855	0.8251664
cg14959731	0.6819218	0.2956692	1.572762	0.3692376
cg00601743	309.85221	0.662756	144862.4	0.0674273
cg05353710	1.20E-47	6.08E-90	2.36E-05	0.0296751
cg01412518	3.3125703	0.9628671	11.3963	0.0574411
cg20750943	0.0678788	0.0122538	0.376007	0.002071
cg02424103	0.4106227	0.1688019	0.998869	0.0497091
cg22481632	2.8553039	1.1063611	7.368987	0.0300892
cg06804210	1.852275	0.7887191	4.349994	0.1570409
cg08922584	0.0689399	0.0068217	0.696701	0.02344
cg06858559	0.2864695	0.102101	0.803761	0.0175499
cg14097568	1.6697466	0.632126	4.410598	0.3009175
cg05915294	9.73E-20	5.99E-32	1.58E-07	0.0022756
cg26225073	0.5443333	0.1828504	1.620443	0.2745181
cg19034028	8.7462378	0.2111605	362.268	0.2536897
cg17029151	1.9607404	0.9358033	4.108238	0.074399
cg17293973	0.9932408	0.2633321	3.746323	0.992011
cg19538502	61085403	1.27E-34	2.93E+49	0.7142785
cg17905198	0.0002084	1.15E-21	3.78E+13	0.6759124
cg22812280	0.5966826	0.2759686	1.290111	0.1893514
cg12523424	0.2220924	1.28E-05	3859.616	0.7626004
cg15996644	2.4463839	1.0346984	5.784095	0.0415843
cg19713947	2.1304333	1.0918548	4.156913	0.0265799
cg20627744	6.9727044	0.6430429	75.6071	0.1102908
cg08124721	19.80956	0.060693	6465.635	0.3119326
cg10676309	2.4591261	0.3406375	17.75289	0.3723026
cg00906420	0.3432524	0.1682699	0.700198	0.0032843
cg19761848	0.1213399	0.0173023	0.85095	0.0338061
cg21162528	1.4113064	0.6479061	3.074189	0.385761
cg06258022	0.4304171	0.2116997	0.875102	0.0198871
cg23574802	0.4841366	0.1624414	1.442909	0.192952
cg06175981	2.0208696	0.8472445	4.82023	0.1126899
cg02042310	2.3847887	0.7442658	7.641379	0.1435122
cg12688234	0.3832005	0.173804	0.844875	0.0174144
cg13237147	1.0605688	0.4521762	2.48754	0.8924538
cg19373874	0.3059615	0.1189176	0.787205	0.0140416
cg26734888	0.3158142	0.1131116	0.881772	0.027797
cg14204430	0.5818891	0.288082	1.175342	0.1311559
cg09170232	0.1197386	0.008763	1.636126	0.1116262
cg24691453	1.5941988	0.7288849	3.486791	0.2428162
cg24105457	3.568639	0.5287672	24.08467	0.1915935
cg26819783	1.8827552	0.7900344	4.486852	0.1532774

cg10032110	3.4293873	1.0011133	11.74762	0.0497933
cg08516083	836.87186	2.3743771	294963.5	0.0245161
cg24830241	1.810023	0.6615158	4.95254	0.2479496
cg14647580	1.9207926	0.5074502	7.270555	0.3364914
cg01028925	0.2319227	0.0490576	1.096428	0.0652103
cg27016494	2.9698012	1.0035267	8.788724	0.0492612
cg13790810	1.2090591	0.4266165	3.426553	0.7209531
cg08436419	0.3488655	0.172728	0.704618	0.0033238
cg05950157	2.172955	0.9572701	4.932499	0.0635172
cg04820679	2.5235666	0.841443	7.568413	0.0985573
cg00849692	1.1316815	0.4421252	2.896698	0.7964308
cg09161455	0.297694	0.0027529	32.19268	0.6120995
cg26188685	1.6372894	0.5862832	4.572392	0.3467333
cg07488576	2.2778262	1.0255462	5.059248	0.0431846
cg09946349	0.8581377	0.4405668	1.671484	0.6528833
cg13937817	0.2899052	0.1272637	0.660401	0.0032014
cg16052317	0.1547727	0.0156599	1.529676	0.1104212
cg10663503	977768.54	0.0074194	1.29E+14	0.1482009
cg18041960	0.234541	0.1015426	0.541738	0.0006861
cg02731554	1.3621194	0.5920189	3.133969	0.467276
cg11792671	1.785886	0.8735759	3.650958	0.1119475
cg16025611	0.3934299	0.1468868	1.053785	0.0634897
cg14093936	1.1057241	0.4448792	2.748219	0.8287144
cg09472600	2.3206189	0.5222753	10.31118	0.2685867
cg08568298	1.9755302	0.7656134	5.097507	0.1592086
cg16477774	0.1224346	0.0237552	0.631028	0.0120639
cg13841399	7.69E-19	9.64E-36	0.061418	0.0356873
cg13986355	0.108175	0.0306332	0.381999	0.0005504
cg02283151	5.1595047	1.0790664	24.66993	0.0398519
cg22881652	2.443049	1.0758012	5.547947	0.0327964
cg07920930	0.2976677	0.088562	1.000498	0.0500941
cg09013696	0.6578493	0.3502656	1.235536	0.1928281
cg04174777	0.2857191	0.1027139	0.794785	0.0163956
cg24545728	0.1055552	0.0328391	0.339288	0.0001604
cg00140447	2.2045664	0.8639637	5.625367	0.0981235
cg18575313	1.6802648	0.8059094	3.503235	0.1662533
cg04410756	0.524564	0.2472555	1.112887	0.0927142
cg07960762	1.4163886	0.62796	3.19472	0.4015726
cg05864627	0.6280223	0.1552759	2.540072	0.5141018
cg14688879	1.1370232	0.428941	3.013985	0.7962685
cg12064276	3.2239624	1.1104924	9.359752	0.0313429
cg23406136	0.8106777	0.2671847	2.459715	0.7109178
cg14561071	0.0721151	0.0110103	0.472337	0.0061035
cg14329059	0.2074535	0.0732414	0.587604	0.0030675
cg08575399	3.777E+09	0.0004131	3.45E+22	0.1475496
cg12021220	1.7860635	0.6053705	5.269538	0.2933861
cg24402683	0.2491291	0.0959425	0.646901	0.0043091
cg13169968	1.6539208	0.8136242	3.362061	0.1644947
cg04396896	1.5774129	0.8427061	2.952668	0.1541762
cg23508264	0.2911421	0.0903885	0.937771	0.0386756
cg00481280	2.4652089	0.8886192	6.838987	0.0830712
cg10012722	2.4892229	0.8836729	7.011905	0.0843619
cg12827950	4.4727171	0.0470354	425.3226	0.519192
cg14606321	0.2033723	0.0569803	0.72587	0.0141473
cg00688820	1.5713933	0.5232631	4.718997	0.4204915
cg04742556	5.5392764	1.4395096	21.3153	0.0127806
cg19604703	2.4091885	0.9229379	6.288819	0.0724706
cg00962740	0.461591	0.1757459	1.212354	0.1166209

cg05271231	2.1012274	0.8350885	5.287052	0.1147566
cg02471319	5805.7468	20.042806	1681735	0.0027312
cg18809597	0.1184636	0.0118283	1.186443	0.0695948
cg15179976	3.9886126	0.7594954	20.94684	0.102077
cg15019001	2.2185821	0.7399046	6.652353	0.1549386
cg22495968	0.9802392	0.504877	1.903174	0.9529847
cg16001613	8.0066468	1.3486832	47.53258	0.0220721
cg15920867	0.9364845	0.2432756	3.604978	0.9239829
cg17736676	36.214371	1.14E-21	1.15E+24	0.8919982
cg10559432	1.36E-06	3.23E-12	0.572781	0.0409245
cg14828673	18792.461	0.4975809	7.1E+08	0.0672263
cg05630208	0.3791596	0.1449877	0.991546	0.0480107
cg02742418	0.5948311	0.0197549	17.91068	0.7649176
cg10546065	1.1319266	0.4267213	3.002564	0.8033844
cg10122050	0.497903	0.2664404	0.930442	0.0288186
cg20810675	0.5049813	0.1951535	1.306695	0.1589819
cg00437399	736626399	3481.2137	1.56E+14	0.0011007
cg10086104	1.702591	0.7709824	3.759899	0.1880012
cg05898591	0.8829412	0.3508489	2.221997	0.7914772
cg23594638	2.7263707	1.2004851	6.191744	0.0165487
cg17152740	0.1044833	0.000845	12.92	0.358124
cg27599211	0.3256587	0.1425364	0.744046	0.0077843
cg17425351	1.4391879	0.4960457	4.175546	0.5029055
cg25050332	4.0177075	1.1446434	14.10219	0.0299434
cg17531288	0.2121378	0.0616379	0.730109	0.0139406
cg17467925	1.4080974	0.4762439	4.163283	0.5360738
cg25306277	2.699791	1.336743	5.45271	0.0056192
cg00744739	1.4556491	0.6652126	3.185319	0.3473763
cg15035421	1.0712692	0.390812	2.936495	0.8935516
cg11558212	2.2864882	0.706902	7.39569	0.1673327
cg14537183	0.3622366	0.1329126	0.98723	0.0471347
cg10240853	0.0123707	0.0003792	0.403607	0.013503
cg16520225	5.7779499	1.0407348	32.07801	0.0448971
cg26690672	0.1221772	0.0314747	0.474262	0.0023816
cg00952516	0.4250181	0.143922	1.255127	0.1214616
cg18636698	0.5533921	0.2153282	1.422214	0.2192184
cg13746225	706.24277	3.50E-14	1.42E+19	0.7319963
cg14859088	0.5323807	0.2123431	1.33477	0.1788749
cg08647910	0.020693	0.0001238	3.458231	0.1375766
cg12177762	1.5442462	0.6004735	3.97136	0.3672424
cg15848620	0.7814433	3.18E-11	1.92E+10	0.9838825
cg12418947	1.53E-08	7.28E-14	0.003224	0.0040102
cg22303418	3.7288722	0.8216061	16.92354	0.0881282
cg04492263	4.8297602	1.6089492	14.49802	0.0049858
cg09703727	0.0784483	0.008405	0.732202	0.0255177
cg04028315	5.174E+13	115.6693	2.31E+25	0.0210517
cg25643208	1.8409046	0.8340901	4.063026	0.1308312
cg08982961	1.5630843	0.7364072	3.317774	0.2447609
cg22923895	0.2911442	0.108397	0.781986	0.0143732
cg05540133	1.2485585	0.5979121	2.607237	0.5545771
cg03967627	0.0537879	0.0127772	0.22643	6.74E-05
cg22889918	0.2849857	0.0958035	0.847744	0.0240121
cg15727188	1.5032255	0.7638752	2.958189	0.2379476
cg24275626	0.0108582	1.61E-07	733.8004	0.4253937
cg08500112	0.6693805	0.330005	1.357768	0.265969
cg23369760	0.351334	0.0009053	136.344	0.7309087
cg22213242	1.7246174	0.1679865	17.70562	0.6464698
cg27417997	0.4156138	0.2006589	0.860838	0.0181124

cg02943604	1.468667	0.4644996	4.64367	0.5128485
cg24057718	5.6401972	1.1639738	27.33036	0.0316702
cg12623544	0.7096937	0.2854503	1.764458	0.4605349
cg13610307	1.7026951	0.8828361	3.283928	0.1122608
cg14277923	0.0788168	0.0209861	0.296009	0.0001678
cg05122644	5.40E-26	2.41E-42	1.21E-09	0.0024533
cg06515771	3.3394978	0.8771762	12.7138	0.0770881
cg23630423	0.3398109	0.1300818	0.887684	0.0275844
cg24904863	0.1562848	0.0449802	0.543015	0.0034903
cg21493768	1.1043757	0.5473731	2.22818	0.7816074
cg11676109	132356.39	1.82E-06	9.62E+15	0.3553635
cg23265210	6.705601	1.7959901	25.03638	0.0046383
cg21475270	3.4108526	0.926896	12.55148	0.0649265
cg14395791	0.1250071	0.0166416	0.939017	0.043267
cg03379477	3.2353561	1.067768	9.803187	0.0379038
cg22215202	3251090.2	4.86E-10	2.18E+22	0.4199602
cg22002034	2.247199	0.9349085	5.401495	0.0703668
cg05768565	0.6970568	0.3448612	1.408938	0.3148389
cg09244707	0.351443	0.0921698	1.34005	0.1256888
cg10505785	3.0051103	1.1971557	7.543453	0.0191205
cg09044219	0.2305812	0.0015774	33.7054	0.5640303
cg06343355	3.7101724	1.2251606	11.23557	0.0203853
cg08005872	0.3108915	0.0831568	1.162305	0.0824892
cg26923754	0.2187419	0.0493212	0.970131	0.0455148
cg09785391	0.1282831	0.0171977	0.956906	0.0451849
cg04972384	0.2694054	0.0746686	0.972019	0.0451436
cg21098898	90.132357	2.3010911	3530.43	0.0161597
cg22331862	0.5557398	0.1965491	1.571346	0.267966
cg17881200	0.583324	0.2411	1.41131	0.2318105
cg15105326	2.0515837	0.4705454	8.94493	0.3388089
cg05209518	1.8798005	0.8543832	4.135908	0.116695
cg25046953	2.1112539	0.9001352	4.951915	0.0857829
cg22764436	2.5135169	0.9519541	6.63663	0.0628052
cg02247178	5.4596339	1.077768	27.65679	0.0403221
cg05241536	1.7586739	0.3457811	8.944773	0.4963133
cg27467275	2.7055022	1.0892135	6.720209	0.0320286
cg02426623	1.2858469	0.2245842	7.362058	0.7776353
cg20340302	0.1976068	0.0454914	0.858369	0.0304827
cg04986248	4.8642265	1.2782846	18.50973	0.0203384
cg05670472	1.6279089	0.6657513	3.980596	0.2854465
cg00741731	0.9307942	0.1641145	5.279107	0.9354468
cg18410271	0.1068585	0.0265039	0.430833	0.0016684
cg25508181	0.7798655	0.3822447	1.591102	0.4943473
cg03332122	0.0190141	0.0010724	0.337114	0.0069097
cg09949949	1.2326233	0.6553394	2.318433	0.516428
cg13186466	4488488	8.48E-08	2.38E+20	0.342101
cg04869854	0.9630824	0.2632924	3.522805	0.9546651
cg02927346	0.3267661	0.1429903	0.746737	0.0079888
cg15336893	0.1915906	0.0566171	0.648336	0.0078912
cg05167561	1.8449447	0.8244714	4.128489	0.1361459
cg19575244	0.0389525	0.0002687	5.647312	0.2011923
cg09460563	0.5716349	0.0001618	2019.88	0.8932736
cg02725370	1.7136554	0.9266784	3.168969	0.0859435
cg05839875	2.0813509	0.7236572	5.98629	0.1738572
cg16941122	1.3656289	0.3993629	4.669794	0.6193652
cg23189194	0.4834068	0.2337336	0.99978	0.0499306
cg00194780	0.4121463	0.0922048	1.842253	0.2459612
cg09880551	0.1202507	0.0290322	0.498075	0.0034867

cg10076951	0.903756	0.1859686	4.392004	0.9001643
cg19448269	0.0336749	8.12E-06	139.6675	0.4249618
cg17382841	0.4825223	0.2077751	1.120576	0.0900476
cg19567339	1.1701632	0.1044002	13.11571	0.8985872
cg25611796	2.8825214	1.01858	8.157365	0.0460811
cg07827420	0.1471751	0.0432685	0.500607	0.0021567
cg09290120	0.5058604	0.2360349	1.084139	0.0797313
cg03341758	12.657052	4.50E-09	3.56E+10	0.8191478
cg21963318	39.150966	0.3651411	4197.824	0.1241517
cg11155431	3.6164827	0.5183185	25.23342	0.1946493
cg08658895	0.2852396	0.1014543	0.801954	0.0173867
cg24331853	1.7592488	0.5965546	5.188052	0.3059527
cg01962146	2.0172368	0.7075505	5.751171	0.1892583
cg01907829	6.1399995	1.2514562	30.12458	0.0253273
cg24878115	0.2116772	0.0935347	0.479044	0.0001945
cg27130630	0.9656116	0.4852964	1.921312	0.9205912
cg19316407	1.7336216	0.5651694	5.317776	0.335984
cg21830341	1.1784177	0.218124	6.366415	0.84872
cg17449759	0.5947821	0.2174719	1.626719	0.3114808
cg24979288	1.8299986	0.7485247	4.473994	0.1851969
cg06807928	1.921015	0.9297859	3.968977	0.0778446
cg20135307	1.406701	0.6668207	2.967526	0.3702634
cg02181247	1.673953	0.7611685	3.681338	0.2001008
cg18766900	0.5834032	0.2466844	1.379735	0.2198156
cg05062087	4.084601	1.182082	14.11405	0.0261231
cg10205548	1.8504316	0.8311557	4.119682	0.1317912
cg00242976	0.2004435	0.027397	1.466497	0.1134476
cg01899130	1.3489608	0.7296804	2.493825	0.3396983
cg19841005	2.4774248	0.8268998	7.422464	0.1051333
cg05653646	33.502936	0.6526439	1719.846	0.0805332
cg02952451	2.092634	0.7540926	5.807134	0.1561958
cg18549575	0.8250825	0.238251	2.857327	0.7616003
cg14201291	0.4293542	0.2099186	0.878174	0.0205694
cg19357195	2.5286133	1.0164946	6.290132	0.0460266
cg04862347	1.8388907	0.9599633	3.522551	0.0662452
cg25985263	0.2560283	0.0704718	0.930166	0.0384574
cg19922137	0.0343621	0.0026839	0.439932	0.0095647
cg26543732	0.5610962	0.2635908	1.194385	0.1338378
cg04894116	1.8636659	2.32E-08	1.5E+08	0.9465588
cg07653264	0.9086761	0.2977188	2.773397	0.8664155
cg09573658	3.4406305	1.0410505	11.37115	0.0427726
cg15914463	6.31E-07	8.27E-12	0.048091	0.0128089
cg03363142	0.6295058	0.2773842	1.428623	0.2683521
cg09608652	1.2234938	0.5090393	2.94071	0.6521168
cg13715590	0.5056004	0.2357843	1.084176	0.0797207
cg10846980	1.3342628	0.445072	3.999931	0.6066836
cg04784519	0.1610839	0.0558876	0.46429	0.0007235
cg17337791	2.0559988	0.8490285	4.978786	0.1102049
cg26955383	1.6570231	0.652322	4.209157	0.2883391
cg05174890	1.52833	0.7950615	2.937877	0.2033174
cg25953692	0.9978735	0.1805492	5.515126	0.9980528
cg01550716	1.6926823	0.8378182	3.419803	0.1424293
cg11639950	0.1825463	0.0516785	0.644816	0.0082551
cg16033633	1.0414533	0.5056814	2.144878	0.9122591
cg05223459	0.2873095	0.1126644	0.732678	0.0090228
cg14519997	0.4649977	0.1994955	1.083848	0.0761495
cg14651518	1.7223707	0.6625967	4.477174	0.2646321
cg14185541	2.5585276	1.293792	5.059595	0.0069264

cg17238325	1.2513015	0.5673487	2.759776	0.5785426
cg13464240	2.9106171	0.9958039	8.50739	0.050905
cg01949993	2.2902946	0.9485625	5.529894	0.0653951
cg04578317	0.3993251	0.1239502	1.286488	0.124068
cg15188908	4.3554133	0.7867637	24.11096	0.0919347
cg25601040	18.620337	0.5401544	641.8849	0.105452
cg01055121	3.2939698	0.1074512	100.9783	0.4948506
cg25832747	0.6090984	0.3082957	1.203393	0.1535679
cg17906168	0.1850751	0.0728179	0.47039	0.0003931
cg04451579	0.7219153	0.3841282	1.356739	0.3114268
cg00970361	2.7125429	1.3343436	5.514238	0.0058366
cg08531052	0.2096314	0.0142653	3.080574	0.2545215
cg18692507	2.0655832	0.6180399	6.903493	0.2386677
cg26139559	1.4599808	0.7866257	2.709731	0.2304002
cg12121193	0.3514987	0.1638353	0.754119	0.0072626
cg04478875	1.4122544	0.4394643	4.538395	0.5622204
cg14006649	0.4465337	0.2051134	0.972108	0.0422313
cg12180613	2.530092	0.2395104	26.72688	0.4402592
cg27258450	0.0911858	0.0195968	0.424297	0.0022669
cg16476940	2.4594048	0.6447466	9.381472	0.1876916
cg04212092	0.3317636	0.1149886	0.9572	0.041263
cg12116027	0.3865465	0.1926701	0.775513	0.0074594
cg18229521	0.4822123	0.2050751	1.133871	0.0945319
cg07417857	0.0690028	0.0074882	0.635856	0.0182965
cg05310774	0.7825186	0.1573751	3.890931	0.7644193
cg27012108	1.5093299	0.5398321	4.219972	0.4326005
cg25578028	1.5654408	0.8131852	3.013588	0.1798781
cg05932560	3.9023559	0.5480799	27.78497	0.1739775
cg11732301	1.8268394	0.7352939	4.538787	0.1943721
cg05740739	1.4339937	0.6747288	3.047651	0.3487015
cg10976218	0.3432117	0.1059319	1.111981	0.0745872
cg11625933	0.4216886	0.1811795	0.981465	0.0451374
cg21528927	0.2248191	0.087242	0.57935	0.0020006
cg17328665	33.484339	0.5276683	2124.821	0.0973038
cg13425294	0.5629705	0.2420449	1.309409	0.1821963
cg16579438	3.1782171	0.9620053	10.50001	0.0579024
cg13375462	7.70E-18	1.81E-41	3280553	0.1557538
cg23475474	0.438124	0.1903072	1.008646	0.0524127
cg27651355	0.2065885	0.0704873	0.605482	0.004047
cg05897699	1.412131	0.0459174	43.42827	0.8434942
cg08575233	9.0367863	0.7261679	112.4582	0.0870394
cg18673246	28.390383	1.0382168	776.3445	0.0474589
cg08597735	0.3499573	0.1103853	1.109478	0.0745064
cg03853945	0.6294005	0.1812126	2.186079	0.466118
cg00021325	0.2900773	0.0511986	1.643499	0.1619528
cg18332814	0.2682223	0.1297929	0.554292	0.0003806
cg03279522	2.9451786	0.9113737	9.517586	0.0710908
cg18967533	0.2476185	0.0776294	0.789842	0.0183438
cg17131837	0.9895698	0.4808984	2.03629	0.9772809
cg04707408	1.8369289	0.6133786	5.501183	0.2772186
cg10172432	2.3894748	0.9567899	5.967444	0.0621292
cg00228738	4.8182644	0.8443493	27.49534	0.0768001
cg17242608	1.2175822	0.6829976	2.170588	0.504508
cg08776660	3.4705902	0.9007309	13.37247	0.0705986
cg00088428	0.3123479	0.0261144	3.735918	0.3580811
cg05734220	0.0001808	5.72E-10	57.18445	0.1822839
cg18575346	2.30E-08	7.22E-29	7.33E+12	0.4652876
cg09063683	0.2368774	0.0680288	0.82481	0.0236643

cg03665785	0.5900099	0.283935	1.226026	0.1573952
cg02679745	3.6624529	0.9028301	14.85724	0.0692334
cg20581874	0.229873	0.0730867	0.722999	0.011912
cg00701692	0.3117965	0.0732377	1.327419	0.1148521
cg04074321	3.5443002	1.1939254	10.52165	0.0226531
cg06660116	0.0079561	0.0002263	0.27967	0.0077791
cg01923516	2.0530707	0.8446621	4.990279	0.1124179
cg18525873	11.04516	0.1711364	712.8558	0.2585992
cg14453949	0.0750172	0.0016476	3.415623	0.1836985
cg08474603	1.1258038	0.5213465	2.431079	0.7628903
cg22803410	0.1909361	0.0430849	0.846157	0.0292659
cg11872743	3.2837395	0.8686525	12.41342	0.0797011
cg23059461	4.2819957	1.4114076	12.99092	0.0102138
cg18082842	1.5036427	0.508417	4.447021	0.46096
cg23164183	1.2306678	0.5834826	2.595696	0.5856873
cg08420923	0.2973304	0.1114732	0.793064	0.0153862
cg10513437	0.0334594	0.0009018	1.241404	0.0653759
cg13307058	0.1462607	0.0389128	0.549747	0.0044328
cg03921753	0.2594528	0.055465	1.213662	0.086535
cg07250916	1.5060393	0.7376708	3.074752	0.2608184
cg01603559	0.2866602	0.1002274	0.819876	0.0197862
cg25840538	0.2612733	0.0931914	0.732512	0.010718
cg13656173	0.9219432	0.4759551	1.785839	0.8096137
cg18693704	0.3618057	0.1416388	0.924205	0.0336123
cg08889949	0.034759	0.003156	0.382822	0.0060624
cg22083560	0.5258742	0.2446496	1.130366	0.0997426
cg01255417	4.3571416	1.0738603	17.67891	0.0394285
cg26219540	3.349946	0.9669919	11.6052	0.0565183
cg14471181	0.0002275	6.42E-56	8.06E+47	0.8898385
cg21027526	0.1075241	0.0220547	0.524217	0.0057976
cg17099048	0.462408	0.1934071	1.10555	0.0828584
cg22963652	3.2073798	0.8800441	11.68951	0.0773452
cg00944599	3.7561517	0.1710276	82.49354	0.401131
cg13655908	0.0160706	2.85E-05	9.049349	0.2011386
cg15819918	0.4235914	0.200789	0.893623	0.0241173
cg11854392	1.662303	0.7710374	3.58381	0.1947759
cg19735514	0.3265561	0.1020825	1.044634	0.0592462
cg19452338	0.7509492	0.3763515	1.498399	0.4164377
cg24439334	0.3492075	0.1207994	1.009491	0.0520739
cg00994032	0.0008683	1.00E-05	0.075372	0.0019672
cg09703789	0.0009728	8.85E-13	1069820	0.5137979
cg04684481	4.5666476	0.5764214	36.17886	0.1503613
cg22347696	1.9371613	0.5565107	6.743077	0.2987907
cg08629884	0.3017512	0.1305075	0.69769	0.0050829
cg01810863	0.272888	0.0882321	0.844	0.0241726
cg19781870	0.1923199	0.041289	0.895805	0.0357171
cg13081009	0.4155244	0.1897023	0.910165	0.0281452
cg09536375	0.4038253	0.1425636	1.143875	0.0878357
cg14498572	0.3856324	2.69E-07	553732.1	0.895197
cg12427286	0.4669666	0.227484	0.958563	0.037959
cg20673075	0.2882289	0.0627713	1.323469	0.109688
cg04439474	5.70E-17	1.97E-32	0.164838	0.0394736
cg24706793	2.2788099	0.2961758	17.53342	0.4288494
cg26344334	1.9513178	0.9577671	3.97554	0.0656035
cg02542218	0.1463657	0.0320451	0.668524	0.0131546
cg01161889	0.0816073	2.24E-08	296945.5	0.7451045
cg10521147	0.8185849	0.2802451	2.391054	0.7143498
cg02025407	7.183E+10	4.31E-20	1.20E+41	0.4814017

cg27486427	1.1952409	0.6006374	2.378475	0.611459
cg22345428	0.6620716	0.1717527	2.55215	0.5491671
cg20092199	0.4737554	0.1851316	1.21235	0.1191616
cg05020759	0.905925	0.4800017	1.709786	0.7604668
cg02592727	0.0056789	0.000156	0.206763	0.0048124
cg20550790	2.2243855	0.8260434	5.989868	0.113686
cg23705098	1.0826023	0.425254	2.756065	0.8677853
cg24311644	2.0262494	0.8706659	4.715571	0.1012961
cg25562958	0.346347	0.0991105	1.210328	0.0967255
cg07944396	0.5302157	0.2604843	1.079254	0.0801805
cg10362869	3.8294304	0.7407943	19.79569	0.1091566
cg08915171	0.3964005	0.1855606	0.846803	0.0168786
cg20438277	2.2275068	1.0219577	4.855178	0.0439465
cg03696393	2.8569999	0.8601299	9.489786	0.0865357
cg25162117	90.546033	0.1808074	45344.31	0.1554044
cg20445774	1.1617758	0.2135719	6.31976	0.8622429
cg05382798	0.0018336	4.22E-08	79.71706	0.2475033
cg12502031	0.677162	0.311793	1.470682	0.3245326
cg01750895	2.4360103	0.2482459	23.90431	0.4447806
cg19272744	6.565221	0.5306694	81.22218	0.1425775
cg07617814	6.0380281	0.9265615	39.3474	0.0600801
cg17922215	0.0315185	0.0012516	0.793689	0.0356988
cg06839900	1.3719015	0.4321776	4.354955	0.5916033
cg25012274	1.4809181	0.6341488	3.458365	0.3641909
cg27655706	0.2288259	0.0600283	0.872277	0.030764
cg07962303	0.0285712	0.0027049	0.301796	0.0031164
cg17525411	0.3918606	0.089826	1.709468	0.2125668
cg13318241	5.9743506	0.6593695	54.13181	0.1119256
cg11065015	0.231229	0.079424	0.673182	0.0072356
cg07788469	6.6230035	0.448921	97.71024	0.168596
cg05621218	0.4660926	0.1815307	1.196725	0.1125839
cg19605623	0.4725281	0.2117539	1.054445	0.0671731
cg11619922	0.2787351	0.0507083	1.532161	0.1417674
cg26003785	8.2468469	0.4311394	157.7459	0.1611508
cg02409177	1.8336477	0.7382702	4.554246	0.1914766
cg04299200	3.205869	0.9850909	10.43314	0.0529886
cg26402630	2.7406458	1.2693289	5.91741	0.010251
cg02577433	1.7405156	0.8923325	3.394916	0.1039979
cg01206017	0.2830209	0.0770026	1.040236	0.0573587
cg19947104	0.6875133	0.2497399	1.892668	0.4683502
cg20377955	1.5199024	0.7059608	3.272283	0.284613
cg26047355	2.9108364	0.9301725	9.109029	0.0664162
cg21936280	0.0414369	0.0048433	0.354518	0.0036514
cg03337218	2.521958	0.7857983	8.094026	0.119994
cg10851413	1.4481343	0.6205864	3.379212	0.3917485
cg00087792	1.3414397	0.3738963	4.812726	0.652235
cg00928751	2.05077	0.6183847	6.801037	0.2403233
cg13168447	2.5033181	0.8935996	7.012762	0.0808251
cg07009521	67398.009	19.52194	2.33E+08	0.0074761
cg24427850	3.2875194	1.0584049	10.21139	0.0395781
cg21421270	0.5184443	0.2568395	1.046508	0.0667857
cg06176815	1.4502696	0.6014106	3.497248	0.4078071
cg20995065	1.8675932	0.7773764	4.486764	0.1624656
cg13819787	1.3845882	0.7420444	2.583517	0.3065486
cg21022792	2.6278353	0.2613569	26.42179	0.4119553
cg24167422	0.2033706	0.0740864	0.558262	0.0019922
cg07676709	1.8084816	0.4680388	6.987895	0.3902788
cg22972318	2.3218975	0.4652808	11.587	0.3043787

cg13711950	1.4280391	0.5444881	3.745345	0.4689063
cg26449178	1.0942372	0.4631748	2.585104	0.8373274
cg26472802	1.0856237	0.4976192	2.368435	0.8364658
cg25737397	0.4169004	0.1451639	1.197309	0.104074
cg04193427	1.81E-07	2.12E-35	1.54E+21	0.6360971
cg08598654	1.7490088	0.7080153	4.320573	0.2256573
cg26549084	0.5216536	0.2604322	1.044888	0.0663467
cg07077694	1.1970965	0.6385887	2.244074	0.5747251
cg08402058	1.7122561	0.232531	12.6083	0.5975282
cg21657490	265.44205	9.86E-06	7.15E+09	0.5225548
cg02621376	1.3401957	0.5751632	3.122808	0.4974896
cg13933070	2.0403085	0.7475234	5.568868	0.163935
cg11854259	1.0642615	0.4810879	2.354357	0.8778135
cg10281378	1.5698894	0.782083	3.151267	0.2045869
cg14543730	0.5033171	0.1946746	1.30129	0.1566099
cg19730801	1171.6639	1.89E-14	7.25E+19	0.7201935
cg00765737	1.8044963	0.7232092	4.502441	0.2057567
cg24627900	0.3091456	0.0978698	0.976512	0.0454498
cg13973436	1.6111103	0.5238194	4.955289	0.4054217
cg26094789	2.1865061	0.8271097	5.780139	0.114736
cg06965373	0.5046942	0.2153718	1.182681	0.1155334
cg06560760	1.7671602	0.8330818	3.748558	0.137813
cg04606265	0.2060728	0.0004112	103.2837	0.618513
cg25568981	1.207069	0.3512362	4.148249	0.7650991
cg07389500	0.2448193	2.52E-08	2375973	0.8638789
cg03068458	110.26836	1.24E-13	9.82E+16	0.7888735
cg19681956	0.0005726	4.74E-06	0.069119	0.0022694
cg15060929	349403515	0.1908901	6.4E+17	0.0706412
cg24074913	0.0025441	6.10E-10	10602.27	0.4423972
cg15414028	1.0992857	0.4961526	2.435599	0.8155932
cg03027750	1.2323893	0.5653001	2.686685	0.5992413
cg02811232	2.5986452	1.3166501	5.128892	0.0059056
cg17563088	2.6949024	0.9699592	7.487428	0.0572416
cg07499142	2.9304885	0.7907687	10.86002	0.1076776
cg15475502	4.1266472	0.9363482	18.18684	0.0610605
cg06630455	0.2035471	0.0212098	1.953408	0.1676948
cg12996913	0.2459664	0.0330561	1.830207	0.1707815
cg00592781	1.5571398	0.739169	3.280284	0.244044
cg10335860	0.0028524	2.19E-05	0.372036	0.018382
cg09224689	1.9213847	1.0346142	3.568208	0.0386676
cg06746274	0.7947446	0.4075753	1.549699	0.5001424
cg18126247	1.7341868	0.575639	5.224462	0.3278571
cg07928883	14.341642	0.6658707	308.8928	0.0890693
cg07094915	2.0135661	0.5680833	7.137066	0.2783278
cg17012555	0.0138566	0.000888	0.216222	0.00227
cg19025461	1.1397703	0.4796196	2.708555	0.7670523
cg04323785	2.5748889	0.9335283	7.102144	0.0676865
cg10129025	2.2497423	0.7244948	6.986028	0.1607655
cg10574376	0.2697579	0.0551418	1.319675	0.1057656
cg09509503	0.7639614	0.3778154	1.544768	0.4535857
cg05984317	1.2860583	0.3644436	4.538277	0.6957655
cg08586426	1.3049298	0.7029747	2.422337	0.3990685
cg20566885	0.3092444	0.1517529	0.630183	0.0012325
cg10977115	2.2928754	0.6746096	7.793067	0.1837251
cg20066226	5.004648	0.5692602	43.99834	0.1465111
cg04130557	0.428515	0.1647874	1.114315	0.0822149
cg08825895	0.5196283	0.2581164	1.046092	0.0666925
cg13799504	0.243371	0.1044294	0.567172	0.0010617

cg01650744	1.60E+20	0.9559655	2.67E+40	0.050222
cg04907738	2.2382265	0.9315814	5.377585	0.0716247
cg17524078	1.4420727	0.6006607	3.462144	0.4126425
cg21136544	3.9507114	1.0094137	15.46256	0.0484475
cg12511137	4.7887672	1.0727665	21.37678	0.0401709
cg24724583	2.823272	0.7713302	10.33392	0.1169343
cg06263372	0.5449458	0.2199185	1.350345	0.1897856
cg23660154	2.8206114	1.1924204	6.672016	0.018246
cg16614527	2.3798923	0.0055904	1013.144	0.778927
cg19915007	4.1935377	0.8124726	21.64474	0.0869044
cg24700959	1.2665488	0.7262719	2.20874	0.4049688
cg14792680	0.0001883	4.64E-12	7643.355	0.3372432
cg26275360	1.721625	0.3806259	7.787153	0.480482
cg02724909	0.1245611	0.0315752	0.491382	0.0029329
cg12403162	0.4963158	0.2076392	1.186334	0.1151063
cg11952478	0.3972415	0.0021994	71.74801	0.7276779
cg03708590	0.5951415	0.0530811	6.672677	0.6738796
cg08923624	0.1879135	0.04619	0.764483	0.0195396
cg14288639	0.3483554	0.1399596	0.867046	0.0234146
cg06365976	0.1246383	0.034139	0.455043	0.0016235
cg08939082	1.0683758	0.552671	2.065292	0.8440861
cg06451843	3.0775514	1.2147974	7.796627	0.0177771
cg16490896	3.30E-08	5.79E-35	1.88E+19	0.5836841
cg13181928	1.4936491	0.7549247	2.955245	0.2491386
cg26162813	2.433074	0.8828535	6.705358	0.0856011
cg00226984	0.360322	0.1208516	1.074309	0.067046
cg22387286	2.50E-07	1.25E-12	0.049676	0.0145963
cg13624041	38.208271	0.9179443	1590.371	0.0554977
cg13617204	0.3241457	0.051461	2.041749	0.2302284
cg24096323	1.4058081	2.03E-06	975760.1	0.9604145
cg02108623	1.1635501	0.5793198	2.336963	0.670312
cg13092738	0.431632	0.1734524	1.074105	0.0708755
cg11178920	1.6780616	0.7610226	3.700141	0.1994718
cg13758543	2.171195	0.9874237	4.774129	0.0537952
cg08169311	0.1244948	0.02388	0.649035	0.0133963
cg11441891	3.9227373	1.0674108	14.41607	0.0395709
cg07737182	4.6403938	1.1640702	18.49824	0.0296087
cg24726370	1.5492101	0.7966635	3.01263	0.1970366
cg08369870	1299050.9	445.86941	3.78E+09	0.0005427
cg13946767	0.1889313	0.0288134	1.238836	0.0824319
cg07746882	0.2136847	0.0525441	0.869006	0.0310736
cg07823273	2.5781201	1.0091233	6.586611	0.0478226
cg01294327	0.2155902	0.0909744	0.510904	0.0004912
cg04347016	0.1052811	0.0195644	0.566545	0.0087492
cg14603886	0.4135557	0.1770569	0.965951	0.0413503
cg21686386	0.2655191	0.1003278	0.702701	0.0075739
cg15297220	2.5240467	1.223353	5.207664	0.0122276
cg10090861	0.0018128	1.48E-05	0.222738	0.0101184
cg11347411	1.3868092	0.7736787	2.485838	0.2721133
cg14957378	0.2904592	0.054703	1.542265	0.1466832
cg13648345	1.8215725	0.6676919	4.969547	0.2415421
cg21076424	0.3121835	0.0640101	1.522548	0.149873
cg26605809	0.3388123	0.1140922	1.006149	0.0513044
cg12828018	0.5329342	0.2108368	1.347103	0.1834507
cg08495770	0.2035218	0.0579863	0.714326	0.0129509
cg09905416	1.3118129	0.2926289	5.880667	0.7229083
cg13900770	0.0170116	0.0011918	0.242815	0.0026686
cg23397571	0.4048303	0.000954	171.791	0.7695784

cg21581267	0.2253943	0.0477163	1.06468	0.059993
cg10115827	8.332E+10	279299.51	2.49E+16	9.24E-05
cg24336989	1.8685118	0.8730339	3.999085	0.1073491
cg19585103	1.1253183	0.6148391	2.05963	0.7018467
cg20656587	2.8271006	1.1422067	6.997419	0.0246074
cg15944673	0.4858461	0.2009496	1.174655	0.1090254
cg03642224	1517.3292	4.70E-31	4.90E+36	0.8523967
cg15845821	0.1572192	0.0223414	1.106369	0.0631084
cg21171858	2.4494956	0.900198	6.665233	0.0794129
cg23886978	0.4127119	0.1843188	0.924111	0.0314082
cg24521756	4.2509162	1.3098203	13.79601	0.0159833
cg01648659	0.7853789	0.3391228	1.818869	0.5728705
cg09276445	4.082592	1.8011746	9.253715	0.0007534
cg27063969	0.2316709	0.0614817	0.872965	0.0307193
cg20655350	0.2514954	0.0837798	0.754955	0.0138485
cg01051524	1.8468042	0.8545728	3.9911	0.1186983
cg27121462	5.52E+29	3.677E+10	8.29E+48	0.0023671
cg13728308	1.8115431	0.3312057	9.90831	0.4931133
cg26696655	1.2383547	0.015524	98.78375	0.9237727
cg09966204	6.8456751	1.3266669	35.32406	0.0215853
cg24053721	2.18E-19	3.59E-31	1.32E-07	0.0019093
cg05264148	0.0114825	4.68E-05	2.818822	0.111636
cg14255417	1.2402532	0.5479055	2.807469	0.6054653
cg23331010	0.535328	0.2416799	1.185767	0.1235528
cg01182455	1.3096338	0.6154954	2.786602	0.4838093
cg21082141	0.7631205	0.3734526	1.559376	0.4584234
cg14193678	0.1720415	0.0230215	1.285683	0.0863289
cg12579212	1.3776625	0.7531082	2.520161	0.2984496
cg07673979	0.8520902	0.425867	1.704893	0.6510342
cg22607980	3524.1698	20.475271	606574.3	0.0018746
cg06901790	0.4813569	0.0373231	6.208064	0.5751853
cg08374732	0.0154259	0.0008892	0.267623	0.0041653
cg00287915	3.524494	0.6408143	19.3848	0.1475256
cg23709430	11.97422	1.3760122	104.2011	0.0245051
cg07996532	4.6583579	1.3793111	15.73271	0.0132181
cg21773162	3.7382376	0.9426157	14.82515	0.0606702
cg00363312	2.3510843	0.8157877	6.775779	0.1134307
cg02629603	0.2340006	0.0576938	0.949083	0.0420408
cg01905633	2.598586	0.8930553	7.561289	0.0797037
cg09622447	0.0028488	1.98E-05	0.410284	0.020816
cg04245131	0.2313559	0.0620096	0.863182	0.0293333
cg09575280	1.0739792	0.5535909	2.083545	0.8328235
cg14334161	1.7074067	0.5533316	5.268518	0.35208
cg07694806	0.3796184	0.1398901	1.030166	0.0572216
cg05773207	0.7396151	0.3361095	1.627537	0.4535181
cg05072470	1.5088704	0.7543255	3.01818	0.2448564
cg02696201	0.4848048	0.2480138	0.947672	0.0342489
cg16269943	0.5662135	0.2873881	1.115557	0.100195
cg00831735	0.4143934	0.1723573	0.996314	0.0490432
cg19106968	0.537903	0.1029096	2.811591	0.4624259
cg01881322	0.9058735	0.4480282	1.831596	0.7831618
cg13928306	0.2233614	0.0667859	0.747019	0.0149553
cg01810719	0.7404904	0.309659	1.770742	0.4994105
cg12656291	1.5528921	0.7875955	3.061818	0.2038604
cg17534029	0.1820479	0.0761288	0.435334	0.0001284
cg08088566	0.4311847	0.1830978	1.015415	0.0542344
cg15888522	1.2345237	0.3292328	4.629091	0.7547112
cg16957569	0.8663966	0.4652961	1.613259	0.6511657

cg22109370	0.2680364	0.1182772	0.607417	0.0016085
cg24352878	0.4117679	0.1070501	1.583864	0.1967346
cg23249321	0.019901	1.30E-28	3.04E+24	0.8986777
cg03086857	0.0062574	8.44E-05	0.463946	0.0209143
cg19107112	1.7020381	0.6118838	4.734451	0.308257
cg18855140	1.71E-10	7.97E-22	36.52447	0.0910916
cg07142285	2.5076426	0.0069961	898.8266	0.7593374
cg06078467	11.149003	0.5373216	231.3331	0.1191152
cg02624984	2.2974382	0.8561053	6.165389	0.0986368
cg13446689	0.184499	0.0050297	6.767794	0.3577971
cg10917602	0.4828047	0.1921452	1.213147	0.1213958
cg17391942	17.055753	2.2835595	127.3883	0.005695
cg13394990	5.4555082	0.8939395	33.29372	0.0659927
cg17159550	0.1575176	0.0522511	0.474857	0.001028
cg02576610	1.242E+19	2131342	7.24E+31	0.0033718
cg21716693	0.9507383	0.399519	2.262479	0.9090778
cg13833923	1.0764083	0.5566446	2.081498	0.8267794
cg26239984	1.3086455	0.5663554	3.023814	0.5290271
cg12448664	300.65508	2.1456416	42128.88	0.0236538
cg24536624	0.2668629	0.0257346	2.767313	0.2682952
cg27090492	0.0004131	6.60E-08	2.586374	0.0806512
cg08249780	6.17051	0.7494214	50.80612	0.0906847
cg23008177	0.9958433	0.2599177	3.815453	0.9951506
cg18877361	1.4220249	0.41594	4.86165	0.5745576
cg18731460	0.6681641	0.2984188	1.496029	0.3268508
cg01865484	0.0756648	0.0147087	0.389237	0.0020078
cg18833109	0.1143629	0.0146864	0.890545	0.0383904
cg21338747	0.298496	0.0197881	4.502695	0.3825515
cg06875985	2.1382958	0.7617575	6.002316	0.148961
cg07743799	40550946	8.94E-21	1.84E+35	0.5897765
cg16903689	0.596038	0.2837504	1.25202	0.1718007
cg11118778	0.2453461	0.1169205	0.514835	0.0002027
cg15349139	0.2623125	0.0983228	0.699815	0.0075199
cg16349667	8.1141478	0.5791187	113.6889	0.1200886
cg15192736	3.0403631	0.9086177	10.17348	0.07116
cg09761040	1.8009737	0.762446	4.25408	0.1797533
cg01787834	0.0240383	2.24E-13	2.58E+09	0.773591
cg09394001	1.9814392	0.3301976	11.89016	0.4544812
cg18132363	1.4859414	0.5224155	4.226563	0.4577408
cg20380468	2.8054559	0.6563139	11.9921	0.1639852
cg22673064	0.6183712	0.2939456	1.300863	0.2052378
cg21876964	1.0678854	0.4460702	2.556502	0.8827647
cg20313969	3.6487452	0.7059753	18.85808	0.1224653
cg19372359	3.3717089	0.2902252	39.17103	0.3313891
cg23875404	3.1512911	1.0982352	9.042358	0.0328269
cg12564698	1.3722426	0.5836362	3.22641	0.4681624
cg08864344	0.934663	0.2145407	4.071932	0.9282973
cg08768048	0.7868058	0.3737488	1.656362	0.5278357
cg24606273	1.264697	0.426385	3.751207	0.6720546
cg03136486	0.1870905	0.0193126	1.812434	0.1479805
cg03512484	1.7186336	0.6918224	4.269451	0.24345
cg10473842	0.3002931	0.0971518	0.928196	0.0366737
cg15343510	1.8552738	0.2808772	12.25461	0.521111
cg00150882	0.2230555	0.0342708	1.45178	0.1164405
cg27096572	1.7948226	0.7053759	4.56691	0.2196366
cg24090938	0.4733177	0.1603683	1.39697	0.1755582
cg02807984	1.0337499	0.448943	2.380344	0.9378273
cg16573887	0.0969557	0.0002806	33.50038	0.4339388

cg13652887	1.95E-05	5.09E-10	0.751075	0.044103
cg23598212	1.3615053	0.4697301	3.946302	0.5697999
cg09034896	1.0834769	0.3779217	3.106257	0.8813984
cg00229532	0.270139	0.0675429	1.080426	0.0642288
cg13610026	0.3047926	0.1050473	0.88435	0.0288083
cg04513214	1.7813725	0.1327167	23.91024	0.6630061
cg16774355	0.2166717	0.0319896	1.467561	0.1171288
cg07156814	4.5693422	0.7327187	28.4951	0.1037477
cg16422547	2.5162123	0.5166649	12.25422	0.2532839
cg02343503	2.463138	0.8099424	7.490717	0.1121718
cg06559864	0.6291622	0.3137635	1.261603	0.1917812
cg20954460	2.2297019	0.9011183	5.517112	0.0827906
cg26570714	0.7380526	0.3456299	1.576026	0.4326219
cg08800893	1.7437519	0.7344222	4.140221	0.2075519
cg26072437	0.026727	0.000315	2.267812	0.109913
cg15650170	3567.0699	0.0028818	4.42E+09	0.2531403
cg22914729	1.9196838	0.9499236	3.879455	0.0692412
cg21958090	0.6825387	0.3567107	1.305986	0.2486537
cg03061778	0.8277699	0.3637011	1.883973	0.6523675
cg24993576	2.79645	1.3000149	6.015418	0.0085052
cg20485084	1.8092429	0.6336221	5.166108	0.2680457
cg00554948	0.429128	0.1909784	0.96425	0.0405504
cg17222452	0.0084357	2.50E-08	2842.745	0.4621271
cg14632485	0.6152562	0.1907562	1.984419	0.4162528
cg04487600	4.0791619	0.8061653	20.64039	0.0892252
cg05619587	1.31E-05	2.52E-17	6819081	0.4140696
cg17687282	0.7834591	0.1573242	3.90155	0.7657553
cg06724409	0.6508051	0.262553	1.613188	0.3536969
cg22328895	2.7199005	1.0749105	6.882302	0.0346459
cg20381115	1.1876555	0.5277167	2.672884	0.6777461
cg02621130	2.1693395	0.7764794	6.060733	0.1395819
cg21487509	1.1389839	0.1379132	9.406526	0.9038412
cg16971668	2.7998038	1.0203653	7.682446	0.0455964
cg23605991	0.7481529	0.3021311	1.852616	0.5305512
cg27617225	9.0716907	0.6352122	129.556	0.104064
cg04606020	0.1106828	0.0250603	0.488847	0.0036803
cg13900100	1.5057268	0.7591789	2.986402	0.2414389
cg00934624	0.0469658	0.0015671	1.407528	0.077914
cg26860970	0.0219646	0.0024409	0.197649	0.0006586
cg18176723	0.2530039	0.0807345	0.792857	0.0183616
cg01382110	0.2165018	0.0316013	1.483263	0.1191305
cg00908271	4.9772633	1.3121501	18.87981	0.0183073
cg00509187	0.3422656	0.1525705	0.767814	0.0092984
cg01747518	0.4186836	0.1399974	1.252138	0.1193098
cg26168651	2.6382543	1.0525954	6.612594	0.0385178
cg08066645	2.4140802	0.6992423	8.334426	0.1632981
cg00145079	3.5880566	1.0195594	12.62717	0.0465758
cg09479650	0.4968064	0.2096342	1.177368	0.112046
cg24532083	1.0771102	0.3989905	2.907754	0.8834469
cg16795307	0.3565005	0.1346928	0.943574	0.0378091
cg24508143	1.2111891	0.6110588	2.400716	0.5830779
cg03365705	111.40242	1.1603896	10695.11	0.0429866
cg10606834	3.2412603	0.9653386	10.88299	0.057056
cg05243293	9.16E-15	2.43E-49	3.45E+20	0.4261678
cg13621378	0.3524624	0.1056351	1.176028	0.0898433
cg12798775	0.6131415	0.2655836	1.415534	0.2518368
cg12396368	0.4319983	0.1420051	1.314196	0.1392391
cg00593607	0.2602032	0.1011767	0.669183	0.0052148

cg06346335	1.7056942	0.4583963	6.346894	0.4257546
cg08838158	0.2863123	0.1157164	0.708411	0.0068145
cg20272979	2.0384721	0.7937319	5.23523	0.1388912
cg04393837	0.2451954	0.0991766	0.606199	0.002336
cg00314029	0.5765223	0.2701912	1.230159	0.154368
cg15615396	10.309034	8.97E-22	1.18E+23	0.9282714
cg04629433	0.6129637	0.3085892	1.217556	0.1621729
cg02343318	0.0045827	9.74E-05	0.215582	0.0061273
cg11299980	0.9495952	0.3458818	2.607049	0.9200499
cg26777074	1.88E-06	3.39E-25	1.04E+13	0.5493935
cg06066452	0.2529288	0.0577484	1.107787	0.0681327
cg15814508	4.6394182	1.3053955	16.48864	0.0176979
cg21562208	0.548613	0.0509104	5.911881	0.6206264
cg01129637	59.340259	3.0939251	1138.123	0.0067409
cg21534299	0.2718767	0.0980181	0.754115	0.0123446
cg15123984	0.1173035	0.0097773	1.40735	0.0909477
cg27546391	0.0054397	1.08E-07	273.6791	0.3451891
cg20632871	0.3812212	0.1363159	1.066124	0.066072
cg01237501	1.955437	0.6885792	5.553078	0.2079224
cg20418797	4.05E-15	2.12E-29	0.774522	0.0482463
cg26398921	2.8244305	1.3077769	6.099976	0.0082178
cg09847368	0.2778685	0.1352827	0.570738	0.0004883
cg03398002	0.478532	0.2380167	0.962088	0.0385994
cg03498697	1.3453215	0.6693082	2.70412	0.4049769
cg14237148	3.8007708	1.1838741	12.20219	0.0248587
cg11257728	1.5246186	0.7813662	2.974869	0.2162404
cg25399162	2.355903	1.1515872	4.819677	0.0189538
cg02348449	0.4084979	0.1748212	0.954521	0.0386916
cg20534570	0.0109959	0.000252	0.479735	0.0192191
cg14585892	2.8665953	0.549485	14.95467	0.2114735
cg01130192	0.0826599	0.0071963	0.949465	0.0453279
cg01523285	0.0027562	8.42E-05	0.090184	0.0009267
cg06218627	6.686E+14	4.82E-17	9.28E+45	0.3508045
cg16306078	0.540686	0.1610562	1.815151	0.3196621
cg26380291	0.2246703	0.0553771	0.911509	0.0366507
cg00823095	0.4781422	0.1315296	1.738164	0.262517
cg09345512	0.0164217	0.0007992	0.337425	0.0077126
cg19013682	6.3484959	0.4127759	97.63991	0.1850347
cg18062025	2.1703099	0.3097583	15.20619	0.4353341
cg23522475	0.1425515	0.0106797	1.902757	0.1406424
cg19846529	7.5018518	0.9040167	62.25303	0.0619714
cg08356445	2.3530951	0.8286513	6.682011	0.1080549
cg17788349	0.884195	0.3029878	2.580304	0.8217932
cg12648583	615565932	36.543319	1.04E+16	0.0171336
cg16860848	1.90E-11	1.98E-32	1.82E+10	0.3165861
cg22900607	0.1740484	0.0537262	0.563837	0.0035524
cg10781510	1.1810549	0.6623964	2.105825	0.5727623
cg06152215	1.9323551	0.7077376	5.275961	0.1986447
cg12856114	0.6225637	0.2163298	1.791643	0.3795526
cg13245152	1.1660068	0.5351789	2.540406	0.6990901
cg13745870	0.5153161	0.1943767	1.366165	0.1826142
cg20037575	1.9125693	0.7716613	4.74032	0.1614428
cg26525342	1.0832005	0.4703169	2.494751	0.8510664
cg00566187	0.3125139	0.1636566	0.596768	0.000425
cg04744409	1.081283	0.4538333	2.576217	0.8599598
cg18167921	0.5322041	0.2407793	1.176352	0.119089
cg26754761	5.8500085	0.7849203	43.60009	0.0847698
cg15121267	1.9269294	0.4825917	7.693992	0.3531201

cg16387593	3.7481977	1.0739228	13.08193	0.0382848
cg27629992	1.6350456	0.6766346	3.950986	0.2747379
cg17657502	1.3304059	0.622823	2.841867	0.4609842
cg19118904	0.5467755	0.319154	0.936737	0.0279572
cg22317846	0.9913138	0.4378235	2.244519	0.9833067
cg06462543	2.7272307	0.5975624	12.44688	0.1952388
cg06493781	8.0653904	0.701035	92.79212	0.0939399
cg00436610	211.39966	3.4996568	12769.77	0.0105086
cg02872914	0.3562435	0.1454141	0.872745	0.0239648
cg03161803	1.7090178	0.8716713	3.350738	0.1187271
cg11222672	3.7027428	0.4219594	32.492	0.2374747
cg17022635	24711.561	1.96E-23	3.11E+31	0.750707
cg03850256	3.1776756	0.7721116	13.07793	0.1092274
cg27436184	4.4709396	0.3785257	52.80831	0.2345169
cg25534226	0.0098966	5.77E-06	16.96275	0.2244308
cg12996100	1.4214233	0.4886365	4.134861	0.5186174
cg16699198	0.3026027	0.1325989	0.690567	0.0045191
cg26226566	1.30E+24	0.0419577	4.03E+49	0.0637283
cg16325984	0.3237499	0.1435616	0.730098	0.0065649
cg00682858	1.034917	0.085765	12.48823	0.9784514
cg20397614	17.067541	0.6397082	455.3654	0.0903924
cg13674369	1.5197596	0.4859508	4.752887	0.4718474
cg23101464	1.1518994	0.4051183	3.275271	0.7908319
cg17947242	45.238979	0.8198753	2496.191	0.0624754
cg09025625	0.128622	0.0240352	0.688309	0.0165565
cg26572992	4.3422968	0.5195998	36.28859	0.1752349
cg08524210	1.196315	0.6043896	2.367959	0.6068776
cg06706550	1.6941717	0.631755	4.543245	0.2948796
cg00683980	0.4337868	0.2138622	0.87987	0.0206323
cg04559909	3.0492089	0.9857089	9.432475	0.0529926
cg16118839	0.8734256	0.3497097	2.181444	0.7719801
cg10424581	0.1827852	0.0843268	0.396202	1.67E-05
cg19757176	0.2166981	0.0563715	0.83301	0.0260204
cg05026186	1.6662003	0.6596161	4.208848	0.2802014
cg25988321	1.6489756	0.8331723	3.263575	0.1510142
cg12177407	0.3749302	0.1762272	0.797679	0.0108713
cg08511084	10.387345	5.06E-07	2.13E+08	0.7852734
cg06967316	2.1705348	0.155718	30.25484	0.5642706
cg13062935	0.9659944	0.4369431	2.135621	0.9318863
cg08160826	2.0755786	0.8773129	4.910479	0.0965027
cg18861810	1.8484823	0.6758636	5.055587	0.2313853
cg14826972	47.90743	0.6308058	3638.397	0.0798762
cg12103475	0.0238263	0.0033226	0.170857	0.0002009
cg11236515	3.0768039	0.5237862	18.07364	0.2134567
cg00437011	1.800699	0.6969992	4.65211	0.2245311
cg17393940	0.8789117	0.4274647	1.807134	0.7256207
cg24008177	0.2017003	0.0515047	0.789889	0.0215274
cg03183345	6.79E-09	6.73E-15	0.006849	0.0076641
cg10047041	4.542726	1.9481234	10.59294	0.0004588
cg02785604	3.619274	0.6325638	20.70802	0.1483594
cg16097329	7.36097	1.5259847	35.50749	0.0129046
cg08447200	1.351185	0.4413092	4.137011	0.5980662
cg25372693	0.2261403	0.0175067	2.92114	0.2547901
cg11311190	19.377793	0.2798946	1341.572	0.170374
cg01779934	1.1303103	0.0617155	20.70146	0.934196
cg25527547	0.2947224	0.1200729	0.723405	0.0076598
cg11502555	1.4208817	0.6446849	3.131615	0.38364
cg16321846	4.3814619	1.8990067	10.10908	0.0005333

cg24790297	0.5060239	0.2100371	1.219119	0.12893
cg03382910	12547.46	11.442219	13759459	0.008232
cg12968041	0.6821814	0.2980634	1.561317	0.3652885
cg25518362	2.0204627	0.7798001	5.235021	0.1476363
cg19890431	2.5078407	0.4800837	13.10035	0.2757058
cg27179111	1.5534606	0.4382746	5.506229	0.4950712
cg19749001	2.7626649	0.8295081	9.201016	0.0978324
cg04103052	0.6050063	0.2998267	1.220814	0.1606339
cg06758672	1.8971545	0.8222775	4.377105	0.1332973
cg12205700	4.5883563	0.7050382	29.86081	0.1108827
cg01097768	1.7898275	0.7160013	4.474129	0.2130222
cg13696706	4.0852714	0.7852244	21.25436	0.0944035
cg07173823	4.3374611	0.6775989	27.76505	0.1213645
cg23664512	1.261196	0.281335	5.653812	0.7617636
cg21534578	1.7942392	0.554401	5.806797	0.3292769
cg01360333	0.2166101	0.0862301	0.544125	0.0011341
cg11536474	2.1076471	0.9482861	4.684426	0.0673012
cg24297451	0.9042506	0.4487951	1.821921	0.7782547
cg08473533	4.30E-11	3.38E-21	0.54735	0.0443537
cg20792833	3.277888	0.8493238	12.65071	0.0848976
cg00009196	2.5772607	0.8316301	7.987051	0.100903
cg00007426	2.5335946	0.9500544	6.756562	0.0632279
cg23584647	0.2898878	0.0733769	1.145251	0.0773144
cg09981407	0.9636495	0.2985047	3.110907	0.9506224
cg23343680	0.4661629	0.1970677	1.102706	0.0823158
cg19091779	1.4920907	0.1810218	12.29871	0.7100096
cg00587613	0.6145848	0.1493357	2.529298	0.5000483
cg00921266	0.7155441	0.3665148	1.396951	0.3267914
cg09524455	0.1230587	0.0086094	1.75894	0.1226276
cg02094337	5.444382	0.1843268	160.8084	0.3265896
cg02569115	0.5456048	0.213088	1.397003	0.2065874
cg26894575	1.4398108	0.3884446	5.33681	0.585535
cg06373360	7.2037893	0.4026903	128.8697	0.1796444
cg02494664	4.527E+10	1.14E-08	1.80E+29	0.2614985
cg11099706	1.977611	0.0704674	55.50007	0.6885644
cg24175899	0.0433373	2.09E-06	899.6509	0.5360159
cg10904740	0.4169279	0.1696741	1.024487	0.0564915
cg04421679	0.0612611	0.0022705	1.652941	0.0967047
cg24889694	0.9811546	0.4772703	2.017021	0.9587331
cg24317086	1.3715682	0.3669785	5.126184	0.638567
cg20281309	0.0078272	0.0002568	0.238572	0.0054033
cg13477354	105.8129	1.9434049	5761.214	0.0222683
cg02574464	4.2741021	1.3438726	13.59351	0.0138694
cg08251815	1.4724955	0.6004734	3.61089	0.3978223
cg09606832	0.1464999	0.0367008	0.584789	0.0065358
cg14618923	0.5592544	0.2632226	1.188217	0.130674
cg15478441	0.0001842	4.04E-07	0.0841	0.0059164
cg14638840	2.0545479	0.854387	4.94058	0.1077401
cg21196927	6.2075451	0.5596522	68.85279	0.1369701
cg14855874	1.8258005	0.9372383	3.556777	0.0768185
cg15575249	1.2852114	0.5802629	2.846586	0.5362698
cg08436089	0.3453775	0.1601172	0.744989	0.0067175
cg04592144	0.3622025	0.1186221	1.105954	0.074564
cg19707677	1.6136177	0.7101538	3.666476	0.2532
cg18093358	3.8297317	0.5691454	25.76994	0.1674288
cg10776926	122.18266	5.7038228	2617.298	0.002115
cg00650953	0.6075204	0.2774685	1.330172	0.2126129
cg01999399	1.4845898	0.4298647	5.12721	0.5320676

cg27635267	2.4349018	0.8707566	6.80873	0.089851
cg12675571	1.4846621	0.6388105	3.450509	0.3583883
cg20594316	7.7137655	1.1438062	52.02121	0.0359106
cg18232861	1.457592	0.6508658	3.264228	0.3596845
cg10832938	3.9986785	1.3467554	11.87256	0.0125561
cg00084432	0.3773114	0.1766482	0.805918	0.0118284
cg10559416	1.4384671	0.7515031	2.753398	0.2723964
cg24628013	0.5158661	0.2105219	1.264086	0.1477622
cg01889143	1.256774	0.6809877	2.319397	0.4647601
cg13937155	1.6500937	0.3717049	7.325191	0.5101621
cg02101571	2.3272971	0.8004499	6.766585	0.1208498
cg10607747	1.25E-20	2.10E-39	0.073738	0.0376923
cg10712623	1.1990393	0.6106526	2.354359	0.5980065
cg06430220	2.2050291	0.6190814	7.853819	0.2224329
cg05073044	0.3927228	0.1816688	0.848969	0.0174904
cg10929178	6.1593362	1.8928887	20.04208	0.002528
cg18528073	0.4889469	0.2272605	1.05196	0.0671933
cg00465883	0.4222101	0.1256591	1.418611	0.1631801
cg21462844	1.458222	0.7175231	2.963544	0.2971631
cg01032675	1.7027171	0.4486069	6.462775	0.4341774
cg21334198	2.4237015	0.368348	15.94777	0.3570607
cg25872036	0.3576449	0.106751	1.198208	0.0955487
cg03357803	3.2031944	1.0138455	10.12033	0.0473238
cg21054147	1.6581384	0.6931037	3.966828	0.2558391
cg14256028	1.8173166	0.3540514	9.328135	0.4741184
cg05202616	0.4855512	0.2292788	1.028267	0.0591399
cg16165258	0.4755706	0.2525013	0.895708	0.0213949
cg21033855	0.0045055	0.0001352	0.150123	0.0025277
cg17123539	2.6290607	0.1532738	45.09552	0.505034
cg18131870	0.0001252	3.01E-07	0.052038	0.0034915
cg17399545	0.3345198	0.1493893	0.749073	0.0077583
cg24367850	0.0960476	0.0237123	0.389045	0.0010282
cg09910876	2.1160697	0.6636091	6.747573	0.2051954
cg13571985	1.09E-05	1.27E-22	9.37E+11	0.5656969
cg23691781	4.5934127	0.922553	22.87071	0.062668
cg22622667	1.7466122	0.6889359	4.428067	0.2400188
cg21625858	1.5868312	0.6502541	3.872384	0.3103844
cg17133045	0.3777655	0.1932076	0.738619	0.004433
cg19890533	5.6913617	2.1477507	15.08164	0.0004699
cg20430077	2.4276908	1.073599	5.48965	0.0331258
cg15145341	0.8119452	0.3766892	1.75013	0.5949769
cg00933692	0.6660523	0.3415325	1.298927	0.2330635
cg04419551	3.4734585	0.506018	23.84286	0.2051951
cg21126943	0.3870532	0.099151	1.510929	0.1719379
cg05407757	1.9200196	0.9132465	4.036671	0.0853229
cg10112391	2.0090965	0.883896	4.566678	0.0958388
cg16935597	3.2952335	0.8648277	12.55575	0.0806054
cg23840812	2.8527672	1.3097612	6.213561	0.0083058
cg14816917	1.2765626	0.4662633	3.495047	0.6346752
cg10904061	680.46448	10.299863	44955.15	0.0022831
cg05678749	0.2792684	0.0918313	0.849284	0.0245862
cg05269632	0.220966	0.0622092	0.784867	0.0195672
cg10880902	1.6031072	0.58516	4.39188	0.3587125
cg16675525	0.1962101	0.0646478	0.59551	0.0040401
cg15457899	0.1029039	0.0237286	0.446263	0.0023827
cg06515235	1.4978702	0.5869296	3.822631	0.3979701
cg24640440	6.78E-09	1.77E-16	0.259543	0.0347399
cg24334045	0.304299	0.0702471	1.318174	0.1116919

cg24041822	1.8651152	0.0073029	476.3369	0.8255517
cg05087002	0.101751	0.0116701	0.887164	0.0386094
cg02915837	2.70E-07	5.04E-16	144.6304	0.1402395
cg20216139	2.4190251	0.7756382	7.544346	0.1279675
cg09633604	1.4377045	0.7127117	2.900183	0.3105757
cg22029189	1.4334274	0.6818006	3.013658	0.3422566
cg00767581	6.3863196	1.9503776	20.91138	0.0021854
cg16445268	1.8421258	0.7172015	4.731484	0.2043238
cg23750704	0.3518574	0.1725709	0.717408	0.0040576
cg00714115	1.8342037	0.7229616	4.653502	0.2015889
cg12406391	0.9208927	0.4785834	1.771986	0.8050744
cg14407437	0.1203134	0.0305439	0.473919	0.0024657
cg27087553	2.4522248	0.8718432	6.897349	0.0891238
cg03064403	2.1855635	0.5267424	9.068356	0.2814921
cg20847035	0.4228	0.1960005	0.912037	0.028185
cg23900696	15.10826	1.0059809	226.9025	0.0494979
cg02088390	2.3745698	0.7135865	7.901749	0.1585861
cg10730712	0.7432116	0.3105072	1.778907	0.5051183
cg19915762	0.3694807	0.0913057	1.495153	0.1627137
cg25076597	1.5202785	0.7742502	2.985142	0.223694
cg18395636	0.3109027	0.0945944	1.021841	0.0543077
cg01050266	2.2882301	0.7706291	6.794445	0.1360288
cg13975228	0.8741596	5.40E-08	14161333	0.9873309
cg07812849	3.3477635	1.1496884	9.748311	0.0267078
cg13859208	1.356026	0.6981288	2.633907	0.3685979
cg07085815	0.8594792	0.1590032	4.645847	0.8603815
cg03475182	1.241922	0.6437863	2.39578	0.5180894
cg26060835	1.0123947	0.4672669	2.193485	0.9750885
cg14131220	1.6819953	0.7453668	3.795592	0.2104842
cg02245918	0.7458475	0.368011	1.511608	0.4158785
cg00751271	0.5328416	0.1576032	1.801488	0.3111078
cg01919011	1.4328004	0.1556441	13.18982	0.7508395
cg18592459	1.4308702	0.7314196	2.7992	0.2953531
cg25629768	0.1737426	0.0491516	0.614151	0.0065936
cg10931252	34.630847	0.0526384	22783.66	0.2843222
cg13077175	1.40E-23	5.09E-43	0.000384	0.0212014
cg22764497	0.3227017	0.1335846	0.779554	0.0119585
cg05469346	1.3787392	0.6944015	2.737496	0.358735
cg14688905	0.3916824	0.1436822	1.067739	0.0669715
cg13375538	1.3640373	0.5992298	3.104982	0.459466
cg24390938	0.0762173	0.0006976	8.326819	0.2824127
cg17785802	0.0773694	0.0006784	8.82331	0.2896141
cg24278087	1.8324441	0.6006955	5.589939	0.2871844
cg25776635	5.00E-12	2.52E-20	0.000993	0.0076004
cg19176553	4.6734693	1.0890677	20.05506	0.0380076
cg21624359	2.9994949	1.0103124	8.905137	0.0478788
cg23055674	0.1094762	0.0181309	0.661028	0.0159006
cg05363382	1.8755486	0.6864598	5.124383	0.2200638
cg19042497	1.0040638	0.4843978	2.081232	0.9912992
cg15887459	3.2970285	0.958303	11.34338	0.0584371
cg05765647	0.5321388	0.2548394	1.111177	0.0930867
cg11750736	0.2540813	0.108454	0.595251	0.0016088
cg02495552	0.4705914	0.2547758	0.86922	0.0160553
cg24903589	6.9770265	0.4079162	119.3355	0.1799264
cg18469036	0.6838328	0.3261429	1.433811	0.3143844
cg00119073	0.0846674	0.0191453	0.374429	0.0011337
cg04567731	2.809565	0.9725601	8.116367	0.056319
cg00254258	1.5671995	0.7638405	3.215481	0.2204695

cg23389023	1.8891859	0.6636954	5.377502	0.2332994
cg20626249	1.770487	0.5726257	5.474124	0.3212446
cg04826355	6724517.5	0.0642889	7.03E+14	0.0951821
cg17142470	0.4486499	0.1976061	1.018626	0.0553841
cg18502142	3.9180586	1.167206	13.15208	0.0270911
cg08111446	1.2994135	0.6541737	2.581081	0.454468
cg13719412	3.1965853	0.6884311	14.84267	0.1379692
cg13608629	8204.9021	1.23E-11	5.47E+18	0.6048041
cg06132342	1.9445106	0.8817101	4.288395	0.0993554
cg24749559	1.3630178	0.6245425	2.974685	0.4367026
cg23501051	0.5174787	0.2102068	1.273909	0.151781
cg05697274	6.5979767	0.0251192	1733.068	0.5068144
cg00403478	1.6036377	0.7027202	3.65957	0.2619081
cg14415914	0.840547	0.2196364	3.216768	0.7997476
cg16589555	0.1317364	0.0173391	1.000886	0.0501001
cg14058476	0.4479872	0.1868636	1.074005	0.0718717
cg11990702	5.3348999	0.3268252	87.08374	0.2399655
cg14353958	2.2082515	0.3837816	12.70612	0.3749129
cg26160008	0.0046547	3.33E-08	650.6905	0.3743675
cg00109300	360997.86	246.65906	5.28E+08	0.0005793
cg06574679	0.0039745	0.0005083	0.031079	1.38E-07
cg25703338	2.6136427	0.5027745	13.58686	0.2533027
cg09269945	3.3409986	1.259047	8.865651	0.0154101
cg13403999	0.271339	0.0011471	64.18116	0.6399917
cg18023724	0.4502022	0.2204234	0.919512	0.0285055
cg05719818	1.3638443	0.6317211	2.94445	0.4293793
cg20597953	0.8391133	0.406203	1.733397	0.6355856
cg11865208	446.96278	5.0852517	39285.32	0.007538
cg25246082	2.5547978	0.3171416	20.58069	0.3782419
cg07838098	2.8099941	1.3541836	5.830869	0.0055366
cg08312191	504.53161	0.15118	1683768	0.1326998
cg18672389	2.2343733	0.6557267	7.613575	0.1986897
cg07260806	0.520784	0.2116109	1.281673	0.1556441
cg23968286	0.3386595	0.1002222	1.14436	0.0813503
cg06687772	0.3474466	0.1405525	0.85889	0.0220568
cg08393356	0.7482378	0.3020516	1.853523	0.5308825
cg09159285	0.3073875	0.0370839	2.547929	0.2743007
cg17069313	1.3297005	0.6958925	2.540771	0.3883968
cg10074727	2.6839151	1.0598037	6.79692	0.0372986
cg10142315	0.6000282	0.3103986	1.159908	0.1287987
cg22911200	0.4259435	0.1421439	1.276367	0.1274656
cg17930857	1.3901372	0.0017249	1120.361	0.9231426
cg11633770	1.2481358	0.5698413	2.733819	0.5795219
cg03927970	0.1215886	0.0232856	0.634891	0.0124652
cg11769332	0.1075038	0.0109507	1.055372	0.055656
cg01984304	2.0990565	0.9213891	4.781952	0.077552
cg17756860	1.1069607	0.3934937	3.114057	0.8473025
cg04457979	1.8261083	0.9658651	3.452523	0.0638699
cg02291020	1.6304864	0.7424543	3.580673	0.2232157
cg15919410	0.4574243	0.2209546	0.946968	0.0351406
cg11990334	1.3212728	0.710115	2.458421	0.3791873
cg03621279	2.2275391	0.979791	5.064274	0.0559733
cg04625862	1.4265064	0.5571316	3.652495	0.4589774
cg08946989	0.0007666	4.81E-28	1.22E+21	0.8008124
cg13383971	0.3948861	0.1389085	1.122574	0.0813233
cg10253847	2.2504259	1.0088621	5.019929	0.0475332
cg15217187	4.12E-25	1.24E-46	0.001369	0.0263684
cg10373607	1.6465462	0.8473436	3.199546	0.1412232

cg07636846	1.3325485	0.6943344	2.557392	0.388047
cg13612936	1.9868546	0.8953235	4.409123	0.0913931
cg06040872	2.3873221	1.0357991	5.502328	0.0410995
cg24697184	5.5327222	1.6100626	19.01231	0.006604
cg18535415	0.4653295	0.1684364	1.285539	0.1400766
cg04002608	1.1973238	0.4702247	3.048722	0.705688
cg10184289	0.7894624	0.342661	1.818856	0.5787861
cg19242583	34.638678	0.000141	8506525	0.5756088
cg03766264	8.7662279	0.4079153	188.389	0.1654284
cg06088745	1.7705809	0.8437705	3.715414	0.1308518
cg04790480	1.6994575	0.8578551	3.366718	0.1284116
cg22071805	0.0501304	0.0029505	0.851743	0.0383592
cg24102242	2.8883115	0.1665532	50.08815	0.4662246
cg08029281	1.5757941	0.7889535	3.147368	0.1976131
cg14788688	1.5457674	0.4225042	5.655321	0.5104746
cg20519373	0.1723031	0.0372503	0.796996	0.0244279
cg08923669	0.5109626	0.1922987	1.357694	0.1780843
cg12573289	4.0024402	0.7830739	20.45723	0.0956748
cg15648859	2.76E-06	6.26E-10	0.012135	0.0027842
cg17390918	2.4073985	0.752713	7.699572	0.1385876
cg14633910	0.8115517	0.2491498	2.643455	0.7289194
cg03379631	0.0650166	0.0106722	0.396091	0.003032
cg23325985	130.95368	0.0264419	648548.6	0.2614161
cg08657654	0.4436701	0.1886087	1.043659	0.0625958
cg26104752	2.3742201	1.2288017	4.587332	0.0100792
cg07704549	1.192E+09	2.18E-05	6.51E+22	0.1953464
cg02229095	0.1222702	0.0332575	0.449523	0.0015581
cg20986935	0.7034541	0.2473784	2.000367	0.5094579
cg16872028	3.73E-12	2.34E-24	5.927289	0.0663848
cg03709468	0.84047	0.4425609	1.596141	0.5953586
cg27508821	679.91921	1.84E-10	2.52E+15	0.6587135
cg17967260	1.973009	0.5928933	6.565709	0.2679468
cg24587056	8.0367308	8.89E-36	7.27E+36	0.9606519
cg20625138	0.342731	0.141613	0.829476	0.0175701
cg24934413	0.2076799	0.0369028	1.16877	0.0745786
cg20643094	2.37E-20	4.99E-36	0.000112	0.0141348
cg16269776	0.0834763	0.0172324	0.404372	0.0020375
cg07565021	0.5738999	0.2095049	1.572093	0.2801227
cg12842389	1.031E+13	8.89E-09	1.20E+34	0.22595
cg02164407	1091.8117	20.1051	59291.07	0.0005983
cg00811382	0.0423373	7.76E-08	23111.4	0.638961
cg11137605	0.6139431	0.3005699	1.254038	0.1806476
cg24644551	1.501962	0.7617308	2.961532	0.2402829
cg10479234	2.2808036	0.8589681	6.05618	0.0979552
cg26714230	1.5100149	0.6892339	3.308231	0.30306
cg05025159	1.9316986	0.4330331	8.617031	0.388152
cg20216030	0.2098692	0.0048347	9.110112	0.4170562
cg03458463	1.0339174	0.5005607	2.135576	0.9281885
cg16677112	0.2323419	0.028174	1.916047	0.1751362
cg03667544	1.8910192	0.5226637	6.841786	0.331516
cg21207418	0.4278116	0.1790901	1.021959	0.055995
cg04329125	2.1854825	0.5261298	9.078242	0.2818957
cg18733548	0.4361619	0.2189855	0.868721	0.0182603
cg13700250	5.1210421	0.6091098	43.05476	0.1326861
cg00237391	0.8813451	0.3917337	1.982901	0.76014
cg14286320	0.4122527	0.1720213	0.987972	0.0469114
cg04911679	7.996824	0.1921273	332.848	0.2744589
cg11549143	1.8629621	0.5910219	5.872249	0.2881661

cg16567290	4.8338625	0.894402	26.12497	0.0672013
cg25021970	0.3695932	0.1373943	0.994213	0.0486713
cg26840561	2.5038179	0.4727251	13.26163	0.2805524
cg16260696	1.7143423	0.6166102	4.766332	0.3015185
cg08599448	1.1454551	0.5574068	2.353877	0.7117238
cg06504636	1.480556	0.2134529	10.26946	0.6912791
cg08320225	0.9538144	0.4998452	1.820087	0.885952
cg16336989	0.7091438	0.3494712	1.438988	0.3411233
cg24211478	11154.436	7.40E-14	1.68E+21	0.6442312
cg27462975	2.1468447	0.7822386	5.89199	0.1380265
cg16583279	66.018425	1.18E-33	3.71E+36	0.9182535
cg14125353	0.0518646	0.0080944	0.332323	0.0017938
cg13809236	11.835348	0.1082746	1293.706	0.3021859
cg11752275	1.7839334	0.4204351	7.569346	0.4324867
cg05263495	0.1932604	0.0392459	0.951681	0.0432951
cg08745960	2.549438	1.1448057	5.6775	0.0219623
cg05833635	1.3024942	0.3189721	5.31862	0.7127513
cg24119471	1.216E+09	6.13E-05	2.41E+22	0.1805483
cg00880682	1.7752079	0.384977	8.185848	0.4617747
cg10700435	0.3322693	0.1390596	0.793925	0.013167
cg00163546	3.9928579	1.3683741	11.65099	0.0112779
cg22144450	0.9777204	0.0766514	12.47122	0.9861609
cg10862587	0.0047292	6.42E-05	0.348587	0.0146748
cg20042662	4.5701445	1.5779271	13.23649	0.0051007
cg01815783	0.2899148	0.1246821	0.67412	0.0040284
cg05241277	4.0759465	1.2010668	13.83215	0.0242062
cg08252303	1.5841677	0.4854473	5.16964	0.4458336
cg05471602	0.6290254	0.3389387	1.167388	0.141724
cg02268584	1.2818855	0.4088015	4.019629	0.6701942
cg10203211	1.7181314	0.8426015	3.503406	0.136526
cg06025456	0.2674908	0.0732726	0.976508	0.0459399
cg03361504	16.464868	1.5726902	172.3746	0.0193949
cg15174552	2.3081777	1.0840793	4.914479	0.030057
cg15435170	0.5074092	0.1454398	1.770245	0.2872612
cg09511963	3.4344039	0.9205943	12.81252	0.0662399
cg06395414	0.1222351	0.0394976	0.378287	0.0002658
cg00255631	2.4295611	0.8999298	6.559141	0.079794
cg04238758	0.4686465	0.1958082	1.121657	0.0887314
cg14188346	0.2227075	0.084005	0.590424	0.0025345
cg14143326	1.3002414	0.6344876	2.664556	0.4732452
cg12559474	0.618173	0.3217149	1.187815	0.1488955
cg04185382	0.2178832	0.0938109	0.506051	0.0003939
cg16259714	3.6077126	1.4214761	9.15639	0.0069327
cg01514075	0.3385491	0.1548956	0.739953	0.00663
cg03887528	3.5007721	1.240023	9.883208	0.0179702
cg26603656	0.9741419	0.4742392	2.000999	0.9431338
cg11281005	6.2293996	1.5597598	24.8791	0.0096213
cg23936747	2.1200425	0.0067103	669.7999	0.7980355
cg13276704	0.5642948	0.245476	1.297189	0.1778888
cg10517193	1.6887194	0.6127538	4.654028	0.3110499
cg23078268	1.8385542	0.8601916	3.929684	0.1160976
cg09438075	0.488239	0.2145588	1.111012	0.0874464
cg16760812	5.8291888	1.5077962	22.53583	0.0106135
cg12963168	1.203204	0.6076951	2.38228	0.5955612
cg08986727	1.6259885	9.33E-06	283454.8	0.9370759
cg08422803	3.3609796	1.0732808	10.52491	0.0373983
cg23923934	2.7993199	1.1642317	6.730784	0.0214666
cg24391314	2.8836915	1.0233895	8.125623	0.0451016

cg03013118	0.1008692	0.0215606	0.471908	0.0035695
cg09163035	1.160042	0.4228305	3.182593	0.7731133
cg07577997	1.6496047	0.654539	4.157423	0.2885492
cg27639142	5.3027323	0.6180604	45.49551	0.1282095
cg07042729	2.3635077	0.9305301	6.003211	0.0705174
cg04663203	1.0703081	0.4958934	2.310092	0.8625729
cg09247619	3.2673663	1.1272542	9.470519	0.0292149
cg17295834	0.6496094	0.2697998	1.564094	0.3359372
cg07832738	1.9602783	0.8088078	4.751056	0.1361775
cg13754259	2.1704324	0.8607298	5.473003	0.1005586
cg18658151	0.3648262	0.2109547	0.630933	0.0003087
cg04105726	1.0288796	0.4437039	2.38581	0.9471028
cg17598999	4.6009705	1.1980515	17.66947	0.0262045
cg05126444	1.7616704	0.8109522	3.826961	0.1525515
cg06168875	0.1636727	0.0296562	0.903311	0.0378349
cg08239804	0.3285293	0.1311452	0.822992	0.0175137
cg01815671	1.4197303	0.7326607	2.751116	0.2991121
cg26122063	7.2107314	1.4274322	36.4253	0.0168206
cg18391058	2.460397	0.8894547	6.805915	0.0828643
cg02640809	1.6266702	0.4869708	5.433706	0.4291487
cg08825190	315.76366	7.01E-10	1.42E+14	0.6742236
cg11559446	0.4034496	0.1889381	0.861507	0.019022
cg09256201	1.5817722	0.5692097	4.395574	0.3792159
cg02329576	0.2146944	0.0376287	1.224962	0.083346
cg14880079	0.9888354	0.3132568	3.121386	0.9847268
cg00684594	0.3849851	0.1856325	0.798425	0.0103224
cg08230140	3.1859143	0.659973	15.37949	0.1491317
cg23281972	0.3831662	0.1802296	0.814607	0.0126738
cg19845250	33396629	4.70E-07	2.38E+21	0.2870798
cg00759427	5.2099431	0.9361892	28.99361	0.0594738
cg02293044	0.2575603	0.104216	0.636537	0.0032984
cg16532529	8.50E-05	8.01E-14	90205.57	0.376732
cg11414913	0.1691904	0.0584832	0.489463	0.0010449
cg18874332	0.480292	0.2329036	0.990455	0.0470404
cg02465761	0.2367405	0.054998	1.019057	0.0530374
cg09299082	0.3291658	0.1381765	0.784143	0.0121068
cg16527057	6.7273192	0.4821612	93.86243	0.1563369
cg07976064	1.4527242	0.8386075	2.516562	0.1828252
cg24852446	1.7148794	0.404396	7.272108	0.4643505
cg00151919	8.7288425	0.1975084	385.7693	0.2623447
cg26288449	0.1248958	0.0342943	0.454855	0.0016074
cg15005368	1.3598433	0.6821398	2.710843	0.3825375
cg07271261	0.4096788	0.1437494	1.167564	0.0949114
cg09355008	0.7759571	0.3854902	1.561932	0.4772985
cg27530015	1.40E-06	1.73E-12	1.138306	0.0522213
cg00922301	0.0011563	1.30E-10	10262.55	0.407412
cg21625817	2.2900018	0.6999656	7.491951	0.1706595
cg09588074	0.4915714	0.1888539	1.27952	0.14568
cg01168231	2.6290813	0.9351973	7.391027	0.066814
cg13321077	0.3205362	0.1232819	0.833402	0.0196076
cg15575538	1.6453448	0.7108796	3.808183	0.2448431
cg25467493	2.171E+15	908.41533	5.19E+27	0.0151667
cg03062454	2.4918149	0.5875682	10.56752	0.2155009
cg03602212	0.4870676	0.1983203	1.196221	0.1166149
cg04868376	1.3524398	0.3344518	5.46893	0.6719146
cg05697909	1.6645158	0.5923957	4.676963	0.3337149
cg23827572	1.338135	0.7647717	2.341359	0.3075181
cg06954677	0.7790521	0.4047365	1.499549	0.4548868

cg24879087	1.4343866	0.5835856	3.525558	0.4317495
cg25179853	0.3258275	0.0751396	1.412884	0.1340833
cg17284531	1.7863284	0.7963128	4.007181	0.1593004
cg25480117	3.00E-05	8.34E-10	1.078418	0.0516717
cg07716287	1.6018752	0.0849582	30.20313	0.7531745
cg19230755	0.2131921	0.0860659	0.528094	0.0008391
cg09791366	2.8186479	0.6284007	12.64285	0.175971
cg17558772	6.1561332	1.3856099	27.35112	0.0169129
cg12511887	124.91643	8.29E-06	1.88E+09	0.5669859
cg08847417	1.20175	0.1257109	11.48829	0.8732328
cg23873669	0.0778603	0.0127562	0.475238	0.0056743
cg07909402	0.2716695	0.0831524	0.887579	0.0309749
cg25955817	0.2407719	0.0672538	0.861976	0.0286537
cg19225308	0.1568533	0.0435041	0.565532	0.0046393
cg11249835	0.4486964	0.240697	0.83644	0.0116684
cg16739530	1.1660881	0.2159286	6.297272	0.8582725
cg12193277	0.2654156	0.0829292	0.849465	0.0254279
cg03114899	5.0623888	1.252501	20.46129	0.0228518
cg16681104	69.012869	2.2525058	2114.435	0.0153071
cg03043127	1.5942839	0.7262164	3.499978	0.244999
cg27618305	1.0180597	0.4560567	2.272625	0.9651556
cg16277944	0.3290181	0.1410948	0.767236	0.0100729
cg10274830	385400.76	56.926316	2.61E+09	0.004262
cg26425555	2.4104281	1.0537265	5.51392	0.0371674
cg14191688	3.6142113	0.956254	13.6601	0.0582217
cg10214640	2.1420172	0.9120454	5.030712	0.0803561
cg11439390	2.7630796	1.1869936	6.431887	0.0183928
cg09605317	0.3062928	0.1022683	0.917345	0.0345058
cg14710465	0.4262961	0.2159263	0.841622	0.0140182
cg09015246	0.8984907	0.4425514	1.824162	0.7670391
cg08462988	1.6411231	0.7980728	3.374736	0.1780573
cg16173229	9.91E-05	2.43E-08	0.403474	0.0297055
cg23948236	2.7713104	1.3273037	5.786288	0.0066513
cg20491914	0.2452273	0.0896109	0.671084	0.0062095
cg01581222	1.1234661	0.3324905	3.796127	0.8513437
cg21216543	1.153E+17	17894.898	7.43E+29	0.0090362
cg12243738	0.3461316	0.1223922	0.978878	0.0454774
cg11983038	0.4801542	0.2311168	0.997539	0.049233
cg06119275	20.328029	0.0082571	50045.2	0.4496454
cg06686679	1.524091	0.3657564	6.350822	0.5627856
cg15677957	1.4236925	0.7450872	2.720354	0.2849452
cg26110898	0.0915879	0.0107773	0.778331	0.0285605
cg15666177	232.69039	1.36E-05	3.99E+09	0.5213999
cg18147080	0.0825183	0.0105373	0.646207	0.017512
cg14645721	367.27929	0.0442911	3045626	0.1995248
cg26344859	10.387281	2.2650805	47.63433	0.0025938
cg26853536	0.3706036	0.0676113	2.03142	0.2528306
cg13098960	0.2355781	0.0754522	0.735525	0.0128194
cg03542374	0.5723189	0.2510827	1.304546	0.1843314
cg07802909	0.6027313	0.2556122	1.421235	0.2473621
cg16996632	0.7742272	0.3441873	1.741574	0.5361403
cg11298446	0.0275219	0.0024517	0.308952	0.0035917
cg16374343	0.7751608	0.2613748	2.298899	0.6461106
cg01603396	3.5162333	1.3048362	9.47544	0.0129175
cg12337098	0.0004097	2.51E-08	6.675604	0.1149539
cg20959960	1.68E-59	1.31E-105	2.16E-13	0.0124771
cg24731625	1.0741691	0.348854	3.307514	0.9007705
cg01269537	0.2700841	0.0880656	0.828308	0.0220553

cg06593258	0.1925826	0.0136193	2.723205	0.2229399
cg03719509	0.0972509	0.0225021	0.420306	0.0018047
cg15543551	0.3058312	0.0803711	1.163761	0.0822918
cg04007987	0.3374017	0.1680196	0.67754	0.0022556
cg05942111	0.2310089	0.0951443	0.560886	0.0012055
cg23043230	57.226106	0.3377071	9697.242	0.1222451
cg19636519	0.3317178	0.0877206	1.2544	0.1039538
cg12808290	0.8951643	0.4290726	1.86756	0.7678643
cg22758471	0.311878	0.1392181	0.698672	0.004636
cg03233918	0.3204063	0.0822348	1.248379	0.1009524
cg27501458	0.0340808	0.0034659	0.335124	0.0037628
cg13966609	6.9706645	0.8253899	58.86935	0.0744753
cg16145216	2.313657	0.6885329	7.774514	0.1749485
cg03176203	0.2792264	0.1178487	0.661589	0.0037484
cg16344215	0.1532154	1.97E-05	1193.243	0.6815622
cg17440085	0.5172091	0.257728	1.037936	0.0635688
cg02132463	0.0058179	0.0003727	0.090828	0.0002418
cg00697095	0.2892699	0.0985127	0.849404	0.0240112
cg24451081	2.2940679	0.5201538	10.11768	0.272786
cg23471362	0.3016409	0.1014302	0.897043	0.0311338
cg02620013	0.3767439	0.1381783	1.027194	0.0564513
cg27523417	0.4362377	0.1656208	1.14903	0.0931853
cg25245118	0.4647709	0.1970718	1.096108	0.0800608
cg17323982	0.9541499	0.1064005	8.55637	0.9665502
cg11753018	1.789952	0.6312767	5.075315	0.2735754
cg04413644	0.0112343	0.0001114	1.132709	0.0565176
cg04909834	1.2233606	0.0275041	54.41419	0.9170753
cg25241102	0.2358894	0.0651582	0.85398	0.0277761
cg05992830	1.2039735	0.4279014	3.387584	0.7250685
cg05065989	1.5853225	0.727783	3.453292	0.2460378
cg06102690	0.4366495	0.0787501	2.421112	0.343043
cg18089519	0.3818535	0.1446514	1.008024	0.0519162
cg07112473	1.7964541	0.9093018	3.549149	0.091742
cg11318307	8948791.4	1.12E-22	7.14E+35	0.637334
cg07112337	1.66064	0.8802073	3.13304	0.117348
cg00612625	2.61962	1.0547806	6.506006	0.0379985
cg12766383	6.814705	0.000127	365722.6	0.7298118
cg11448366	0.3680301	0.1424907	0.950561	0.0389521
cg00947032	4.6689485	0.0123465	1765.611	0.6108588
cg12307237	1.5092117	0.7090431	3.212386	0.2855795
cg19411922	0.5546071	0.2866504	1.073046	0.080015
cg11832543	3.5113575	0.3077512	40.06363	0.3119231
cg26094752	2.542E+13	8.57E-07	7.54E+32	0.1772397
cg11531997	0.6637566	0.082251	5.356442	0.7004725
cg22149555	1.7198552	0.4308878	6.864669	0.4425964
cg22535103	1.0350232	0.397084	2.69785	0.9438553
cg18028470	1.0906491	0.4102403	2.899558	0.8619158
cg15786205	2.5059902	0.8544066	7.350115	0.0942567
cg08284733	0.8544225	0.0885941	8.240252	0.8917742
cg21647257	0.025263	0.0027334	0.23349	0.0011869
cg01372551	0.0112968	0.000223	0.572149	0.0251696
cg16820615	0.3318359	0.12654	0.870199	0.0249216
cg13733630	3.1156791	1.1543172	8.409696	0.0248815
cg18478394	19.975572	1.5232047	261.9631	0.0225821
cg08606951	0.3020133	0.1090776	0.836212	0.0212108
cg09159223	0.2545559	0.0832749	0.77813	0.0163952
cg25247976	1.2661569	0.3545556	4.521585	0.7163288
cg05438727	0.0844194	0.021369	0.333504	0.000421

cg27316130	0.8192741	0.3310501	2.027518	0.6663541
cg01040749	0.0636057	0.003514	1.151301	0.0622363
cg00551679	1.7748775	0.8361529	3.767481	0.135177
cg11462509	3.05E-15	3.10E-29	0.30021	0.0420363
cg04669815	1.8414139	0.9491869	3.572326	0.0709612
cg25369062	6.1423413	1.4237428	26.49942	0.014949
cg01450807	0.3379243	0.1140219	1.001499	0.0503167
cg16627193	1.7295356	0.8223812	3.637356	0.1486268
cg16476235	1.6467795	0.5494849	4.935318	0.3730687
cg11380607	5.65E-24	1.19E-47	2.692275	0.0543094
cg13518390	0.0045318	2.05E-05	1.002716	0.0501152
cg16812893	1.3330194	0.4392421	4.04547	0.6118146
cg08458852	0.0873527	0.0020556	3.711991	0.20254
cg26780705	0.347648	0.1606688	0.752225	0.0072974
cg22621652	0.285733	0.115061	0.709565	0.0069492
cg05597270	1.15E-05	4.13E-29	3.22E+18	0.6797466
cg22011366	0.3350115	0.1086661	1.032821	0.0569433
cg14887955	13.046566	1.1098501	153.3656	0.0410664
cg08529345	2.2192052	0.8886131	5.5422	0.0878089
cg11539780	0.2906571	0.0771943	1.094401	0.0677584
cg11893763	0.4308345	0.1354358	1.370526	0.1538318
cg08598406	7.7378269	0.1348508	444.0015	0.3220398
cg10943921	0.1462581	0.0520603	0.410898	0.0002648
cg23399173	0.1365206	0.0090334	2.063216	0.1506555
cg01070209	0.1381733	0.0176303	1.0829	0.059545
cg06622151	3.9602248	1.2147523	12.91076	0.0224533
cg18447645	2.6000389	1.1681055	5.787322	0.0192539
cg17376813	1.254027	0.3393797	4.6337	0.7342721
cg20912272	3.3441069	0.7392203	15.12817	0.116975
cg17643564	2.7602351	0.8831607	8.626853	0.0807646
cg08441475	6.50E-09	2.32E-24	18222555	0.2989237
cg06588782	0.4298144	0.1861916	0.992206	0.0478954
cg14841203	1.8480045	0.8771966	3.893221	0.1062408
cg08625564	0.1246287	0.0380638	0.408059	0.0005792
cg00559473	0.001509	1.01E-12	2262857	0.5467586
cg25079915	1.5806482	0.4231012	5.905085	0.4959692
cg16617817	0.8452917	0.0066475	107.4863	0.9457974
cg02585598	1.3140725	0.6770452	2.550474	0.4195219
cg22083053	2.3476294	1.0805718	5.100415	0.0311067
cg14950237	4.2979978	1.7647981	10.46736	0.0013239
cg07981328	2.5028835	0.8943702	7.004288	0.0805775
cg09050775	1.3410014	0.6377919	2.819548	0.439026
cg11416290	1.6119498	0.80692	3.220124	0.176272
cg05338009	0.8003997	0.1862469	3.439733	0.7647198
cg14192130	1.4307878	0.5855162	3.496323	0.4319803
cg12303318	0.555963	0.2592534	1.19225	0.1315019
cg05587400	4.5502514	0.8474225	24.43266	0.0772443
cg09548893	0.5197895	0.2596273	1.04065	0.0646801
cg06049972	0.0009323	4.42E-08	19.65699	0.1695548
cg23352483	0.5029472	0.2031099	1.245414	0.1373923
cg04400131	0.4818884	0.2558178	0.907741	0.0238487
cg17461600	0.9255867	0.4805325	1.782836	0.8171595
cg00603435	1.3825145	0.7407344	2.580339	0.3089898
cg20976833	1.1078399	0.1651081	7.43337	0.9160217
cg03823352	0.4805938	0.2005696	1.151572	0.1002938
cg26639864	1.7328843	0.5932011	5.062175	0.3148096
cg10389032	0.4466308	0.0015025	132.765	0.7814604
cg07849438	1.47E-06	1.67E-12	1.299129	0.0545432

cg01744412	0.6703535	0.3474082	1.293504	0.2330344
cg26675395	1.9971797	0.6108983	6.529282	0.2523996
cg00500989	0.0036488	3.56E-07	37.40063	0.2335246
cg13456688	3.4842356	0.8826795	13.75346	0.0747775
cg05382305	0.9939214	0.299731	3.295888	0.9920463
cg23893629	1.1840719	0.513635	2.729616	0.6917392
cg08888354	0.1916831	0.0559036	0.657245	0.0086005
cg18561976	0.7078306	0.333885	1.500589	0.3674131
cg07053727	0.2194459	0.0823502	0.584777	0.0024225
cg04965987	1.8759527	0.8045482	4.37413	0.145259
cg26855280	1.4428413	0.5270564	3.949845	0.475529
cg15797102	0.6508546	0.3323585	1.274563	0.2104023
cg10886095	0.2541311	0.0776404	0.831818	0.0235535
cg19612114	0.4263809	0.1782378	1.019989	0.0554302
cg23348700	1.5060134	0.4320459	5.249619	0.5204157
cg04031507	3.6380363	1.0275776	12.88011	0.0452698
cg22400068	0.0139213	8.39E-06	23.10498	0.2585166
cg17362927	0.8429041	0.3985018	1.782896	0.6547826
cg14135522	0.5898824	0.1332468	2.611403	0.4868166
cg01616876	2.4208627	0.8410517	6.968152	0.1012019
cg26788180	0.5429955	0.1619201	1.820924	0.3225936
cg08264481	0.481381	0.2192074	1.057116	0.0685201
cg10601234	0.6140162	0.2737884	1.377034	0.2365774
cg19256094	3.1500651	0.9210839	10.77308	0.0674094
cg23111106	0.819459	0.3404394	1.972489	0.6568473
cg23205231	0.9853929	0.4232493	2.294154	0.9727754
cg16864063	2.7072354	0.8180405	8.959365	0.1028817
cg03271761	2.3668699	0.9572553	5.852225	0.062128
cg02858997	2.0270179	0.8177421	5.024569	0.1271251
cg03767258	8.2922546	1.3401529	51.30869	0.0229165
cg08984405	4.8E+10	0.0341942	6.74E+22	0.084813
cg05387963	0.9090186	0.3938955	2.097802	0.8230981
cg11527913	2.4421974	0.5979814	9.974103	0.213598
cg06698399	0.2495307	0.1051287	0.592279	0.0016462
cg13633964	0.4621966	0.2115292	1.009911	0.0529616
cg14997321	3.225178	0.6295151	16.52347	0.1600914
cg25328202	6.21E-08	6.66E-22	5784485	0.3119248
cg10683929	1.8133755	0.7111122	4.624208	0.212705
cg14947466	0.2082422	0.0837866	0.517563	0.0007306
cg02983911	1.2218299	0.6645378	2.246476	0.5190713
cg08186671	0.34067	0.0822395	1.411196	0.1375493
cg14157549	1.5292405	0.7006316	3.337812	0.2861481
cg11631523	1.8386239	0.8819615	3.832976	0.1041951
cg14478663	0.2036438	0.0760194	0.545529	0.0015491
cg17306261	1.0231502	0.561781	1.863424	0.9403587
cg07783843	1.4171195	0.4809866	4.175226	0.5271497
cg26885951	0.225577	0.0963436	0.528161	0.0006022
cg13982590	2.3185837	0.4926776	10.91146	0.2872524
cg18502617	1.1510493	0.5853808	2.263338	0.6834474
cg24849091	0.1253605	0.0264618	0.593884	0.0088829
cg07566169	1.3416093	0.6260238	2.875155	0.4498668
cg08026502	4.0606674	0.7117262	23.16764	0.1147443
cg17505819	0.3647581	0.150165	0.886015	0.025932
cg22075918	0.367773	0.1242503	1.088585	0.070815
cg01088546	0.2652906	0.1241278	0.566989	0.0006166
cg19546057	0.4308001	0.1562957	1.18742	0.1035484
cg26404223	38.160286	0.2807438	5186.961	0.1461958
cg00089384	0.4458382	0.2160843	0.91988	0.0288191

cg12640656	0.0314402	0.0020132	0.490997	0.0136161
cg14069412	1.8727109	0.6363793	5.510937	0.2545953
cg18811550	4.1112993	1.0157869	16.64009	0.0474879
cg22562383	4.8477676	1.0727515	21.90708	0.0402454
cg02117924	0.3296408	0.1006365	1.079758	0.0667726
cg22346461	1.7672752	0.7048878	4.430863	0.2246543
cg21037892	463192.02	0.0016314	1.32E+14	0.188959
cg24759279	0.4534293	0.1781451	1.154105	0.0970595
cg19848082	0.5424967	0.1958029	1.503056	0.2395043
cg18792146	0.2926129	0.1159064	0.73872	0.009298
cg18179002	0.3224091	0.0513554	2.024086	0.2271748
cg10866199	0.2160128	0.0322756	1.44572	0.1141233
cg19553961	0.6492665	0.3206983	1.314466	0.2300726
cg03840511	0.4180799	0.1911101	0.914608	0.029003
cg23917744	0.5634539	0.1064134	2.983462	0.4999376
cg01523759	0.0006507	2.15E-05	0.019725	2.49E-05
cg16243243	1.8782562	0.6941934	5.081936	0.2145227
cg13038544	8.2137014	0.274468	245.8024	0.2246075
cg01142735	0.9252692	0.2464176	3.474278	0.9083973
cg23384406	3.2829943	1.1997131	8.983857	0.0206418
cg14360405	1.7675808	0.3467172	9.011211	0.4930917
cg19830245	2008.5446	0.0005576	7.23E+09	0.3234754
cg00037056	0.1085329	0.0067323	1.749694	0.1174504
cg02811368	3.010424	1.1214075	8.081498	0.0287141
cg18147865	1.7587714	0.6196796	4.991736	0.2887663
cg24020549	0.520234	0.1549289	1.746888	0.2903485
cg10871120	0.250858	0.0988234	0.63679	0.0036198
cg23851027	0.256957	0.0995321	0.663373	0.0049833
cg12567635	0.6757404	0.3934012	1.160711	0.1556019
cg13303069	1.3964335	0.5987776	3.256679	0.4395866
cg24278948	1.5184214	0.4300037	5.361822	0.5164301
cg21805118	3.1090162	0.6858521	14.09339	0.1413035
cg09540085	1.9307793	0.540666	6.89503	0.3110293
cg01978236	0.2462824	0.0956399	0.634202	0.0036894
cg20091187	0.6899215	0.3700748	1.286204	0.2428202
cg24425149	1.3394736	0.4382168	4.094296	0.6081595
cg19084794	2.8263377	0.2692686	29.66624	0.3864023
cg00693583	0.5908021	0.1660052	2.102627	0.4164863
cg05925971	5282.1119	1.08E-19	2.58E+26	0.7477635
cg22941315	1.059673	0.5666646	1.981608	0.8559868
cg14154186	1.9426764	0.7057523	5.347473	0.1986507
cg05845141	0.2204103	0.0609035	0.797667	0.0211974
cg26440467	0.16671	0.0311422	0.89243	0.0363564
cg08461546	0.4142409	0.0878016	1.954356	0.2655263
cg10521450	0.1848427	0.0670649	0.509459	0.0010996
cg16391955	2.0986794	0.752545	5.852747	0.1565803
cg27023360	1.1307871	0.516848	2.473995	0.7583084
cg26407324	0.0945334	0.0014799	6.038657	0.266076
cg17282145	0.5865594	0.2891235	1.189982	0.1393935
cg20563854	1.370553	0.6858889	2.738659	0.3721466
cg09718582	2.502185	1.0388821	6.026603	0.0408532
cg06550462	0.0507613	0.0034118	0.755223	0.0304822
cg26081025	0.0011948	1.33E-05	0.107027	0.0033427
cg19025113	1.8254135	0.7253849	4.593609	0.2012094
cg09502865	0.2956358	0.0871119	1.003313	0.0506234
cg11554650	3.7137594	1.1640596	11.8482	0.0266496
cg25507001	0.4082546	0.1329185	1.25394	0.1176477
cg16796354	1.0874868	0.4916037	2.405652	0.8359774

cg13779907	0.0740953	0.0115893	0.473723	0.0059731
cg27252395	2.540547	0.9101724	7.091381	0.0750347
cg04664169	0.3949697	0.1773382	0.879681	0.0229814
cg08574423	2.2499938	0.84033	6.024386	0.106576
cg24689895	1.5196622	0.7249206	3.185691	0.267803
cg16889990	4.2114916	0.79663	22.26462	0.0905794
cg13776285	2.1704761	0.8134072	5.791646	0.1217323
cg25814649	1.1880807	0.4385732	3.218473	0.7346535
cg18597411	0.3114149	0.1158657	0.836997	0.0207393
cg11014582	0.1911944	0.0263516	1.387215	0.1017839
cg01324543	0.4240221	0.2076191	0.865984	0.0185276
cg17088014	3.9838375	1.4952733	10.61409	0.0056989
cg25215194	0.1376648	0.039016	0.485739	0.0020532
cg16196175	2.6013213	1.1265685	6.006623	0.0251503
cg00762605	0.3975321	0.1886251	0.837809	0.0152996
cg24830367	0.7472241	0.1236182	4.516679	0.7509154
cg14335275	0.0670445	0.0010395	4.323987	0.2036531
cg27625897	0.0004062	3.34E-11	4941.019	0.3481775
cg26982601	2.6354579	0.4592631	15.12344	0.2770058
cg01957599	10.763116	0.5075296	228.2521	0.1273184
cg16205846	0.2752991	0.062676	1.209228	0.0875714
cg10951873	0.8681727	0.3677641	2.049476	0.7470222
cg07831322	4.8625577	0.8882079	26.62042	0.0682587
cg20337547	22.552053	0.0005543	917472.8	0.5650291
cg15699050	2.3158856	1.1123847	4.821467	0.0247913
cg22539450	1.2704143	0.575435	2.804752	0.5536339
cg03397616	0.8211079	0.3256973	2.070076	0.6761119
cg16163174	0.3769381	0.1605417	0.885018	0.0250615
cg01172735	0.0021224	6.77E-06	0.665007	0.0358091
cg26712743	0.9956175	0.3716251	2.66735	0.9930304
cg02032558	2.2114992	0.7592914	6.441175	0.1456402
cg20956815	1.1605234	0.603115	2.233097	0.6557443
cg02741548	0.1922807	0.0649469	0.569263	0.0029074
cg27275851	1.092107	0.5512172	2.163753	0.8006014
cg14557954	0.3293451	0.1348375	0.804437	0.0147866
cg00223552	0.9629348	0.0006485	1429.767	0.9919124
cg18121601	0.2772181	0.1019189	0.75403	0.0119723
cg08352439	1.6273997	0.8116731	3.262927	0.1700409
cg07963497	1.5464244	0.7385133	3.238165	0.2476357
cg09568217	2.1054744	0.4214252	10.51912	0.3643331
cg24924243	6.19E-16	3.39E-30	0.113087	0.0366129
cg01208873	14.758606	2.57465	84.60041	0.0025152
cg07157506	93625793	0.0402449	2.18E+17	0.0953153
cg22552457	0.1055387	0.0282278	0.394589	0.0008318
cg18773129	0.534777	0.309935	0.922731	0.0245182
cg15036894	0.2667953	0.1040296	0.684225	0.0059657
cg09379340	0.6553385	0.2884626	1.488819	0.3127901
cg07957294	3.2742044	0.9341802	11.47575	0.0638019
cg01145686	0.4192446	0.1626103	1.080904	0.0720239
cg27571769	3.5276397	0.7371691	16.88112	0.1145183
cg26837178	2.1217498	0.8297223	5.425697	0.1163447
cg14008686	0.5960214	0.294849	1.204825	0.1495667
cg15171962	0.4712447	0.22405	0.99117	0.047329
cg13984756	0.438174	0.2139929	0.89721	0.0240333
cg26284735	1.7288402	0.7206498	4.147491	0.220126
cg15696309	1.2130003	0.4112823	3.577518	0.7263993
cg11354105	0.4859551	0.240382	0.982405	0.0444954
cg10920427	7.859E+13	5.30E-20	1.17E+47	0.4116335

cg14989316	2.6180169	0.7194164	9.527184	0.1442105
cg02605634	0.0252491	0.0009508	0.670526	0.0278884
cg07109115	1.7777617	0.8126993	3.888814	0.1496808
cg18170229	1.2363307	0.5395527	2.832927	0.6160387
cg05661060	0.4003642	0.1787652	0.896659	0.0260736
cg17479060	1.7130436	0.5922884	4.954543	0.3205292
cg19864048	2.1658842	0.6728973	6.971427	0.1950628
cg15701579	5.2984185	0.6195809	45.31005	0.1278148
cg21208996	4.89E-07	1.65E-15	144.8905	0.1442834
cg16438688	2.0773771	0.7679234	5.619695	0.1498973
cg07544102	0.2603217	0.0942122	0.719306	0.0094505
cg15436123	0.320564	0.1446011	0.710653	0.0050961
cg22141459	0.0774216	0.0188981	0.317179	0.0003767
cg11918740	40162.561	1.09E-11	1.48E+20	0.5621228
cg12750378	1.44E-06	5.89E-13	3.528898	0.07314
cg10234998	0.5445469	0.197751	1.499519	0.2395774
cg02717503	0.2026138	0.0262703	1.562694	0.1256043
cg04595807	0.2516427	0.0734393	0.862264	0.0281057
cg14911189	3.7097512	0.7682026	17.91487	0.1027346
cg20009378	2.5234129	0.8285126	7.685596	0.1033336
cg21417130	21.990615	1.7747747	272.478	0.0160979
cg09347306	11.765042	0.053315	2596.196	0.3706334
cg00040566	1.6884911	0.6693614	4.259287	0.2671609
cg20330021	5.17E-09	1.62E-22	165189.7	0.2291028
cg14461006	5.1383075	0.2042455	129.267	0.3199038
cg19074393	0.53031	0.2074807	1.355445	0.1852485
cg18649198	2.7829679	1.075362	7.202143	0.0348817
cg27454064	3.2589944	1.0251381	10.3606	0.0452808
cg10946681	0.8133005	0.4054345	1.631478	0.5606814
cg12373657	3.413857	1.0510657	11.08819	0.0410696
cg22053108	0.9608897	0.0001489	6198.795	0.9928877
cg27151122	1.6769994	0.5362394	5.244536	0.3741479
cg00993308	0.3535173	0.1484818	0.841682	0.0188048
cg23885965	2.1710015	0.9391274	5.018752	0.0698206
cg01543603	1.9471536	0.5819491	6.515016	0.2795165
cg11940495	0.1046047	0.0079819	1.370868	0.085491
cg14926149	2.14E-10	1.67E-26	2735914	0.2393168
cg26648306	19.587245	1.1173082	343.379	0.041763
cg11496569	0.3586024	0.1102303	1.166609	0.0883948
cg14582478	0.8398965	0.4007035	1.760469	0.6440216
cg25346709	0.98074	0.5084392	1.891772	0.9537325
cg12134349	0.3147855	0.0769307	1.288041	0.1078661
cg16225703	1.8486603	0.7105191	4.809927	0.2078642
cg07970752	0.5077197	0.2765697	0.932059	0.0287444
cg02307239	3.5940763	1.0467981	12.3399	0.0420896
cg14189391	0.3883803	0.1735484	0.869148	0.0213808
cg07604117	0.2672289	0.1005825	0.709977	0.0081207
cg20611272	4.3101648	1.3074952	14.20848	0.0163729
cg26118435	0.4017521	0.1106224	1.45906	0.1657969
cg16234557	2.598339	1.3647984	4.946786	0.0036528
cg03892041	3.8889135	1.1668885	12.96066	0.0270181
cg11563844	0.169328	0.0515054	0.556679	0.0034488
cg05521538	0.3013711	0.1039958	0.873348	0.0271454
cg19784816	1.6789696	0.4294543	6.564001	0.4563308
cg00765128	2.2938509	0.9160433	5.743999	0.0762745
cg14634534	1.2899248	0.6087897	2.733138	0.5063493
cg08045063	20.479679	0.1924489	2179.37	0.2048155
cg06930757	2.0267485	0.8188114	5.016674	0.1265936

cg00701856	0.3427015	0.1301014	0.902714	0.0302288
cg06329735	2.0677234	1.1288435	3.787487	0.0186517
cg06774703	4.2052642	0.389423	45.4114	0.2367579
cg06901890	2.6719251	0.9300594	7.676052	0.0679556
cg08470863	2.5875092	1.2262992	5.459682	0.0125802
cg06689961	1.75E+10	3.54E-05	8.66E+24	0.1718648
cg23520574	0.6038946	0.2735066	1.333381	0.2120261
cg17465423	0.458825	0.1841	1.143511	0.0944967
cg26781726	0.0484975	0.0039347	0.597761	0.0182012
cg16363104	0.3231037	0.1437707	0.726129	0.0062461
cg08702225	1.12E-05	2.54E-10	0.492531	0.0366378
cg03801429	0.1237191	0.0344689	0.444065	0.0013508
cg15881486	2.2948799	0.3975914	13.24595	0.3530202
cg21573345	0.5536068	0.287175	1.067226	0.0774493
cg05716671	2.1587429	1.0298152	4.52525	0.0415737
cg14638729	680.52226	2.8620447	161811.1	0.0194574
cg21441657	1.6133756	0.5259953	4.948676	0.4028914
cg24740404	0.6186748	0.2782878	1.375406	0.2387996
cg21906852	0.028808	8.39E-06	98.91598	0.3931418
cg08114317	0.8650737	0.3761421	1.989547	0.7330338
cg21243750	0.6736495	0.31646	1.434	0.3054421
cg08786026	1.2614764	0.7061665	2.253467	0.4326364
cg20028827	1.6023039	0.7408269	3.465557	0.2309997
cg08220696	0.4683432	0.2051885	1.068994	0.0716221
cg00238848	0.5279187	0.1708251	1.631483	0.2671385
cg03978242	4.5935942	1.3087225	16.12344	0.0173153
cg24033871	3.2932817	0.9867281	10.99158	0.0525943
cg10481534	108.69929	0.0020787	5684125	0.3976552
cg16257091	1.4814195	0.710571	3.088508	0.294441
cg07786657	3.6592603	1.0420652	12.84966	0.0429434
cg19456540	1.2333264	0.6427456	2.366557	0.5282436
cg07721872	0.8492998	0.035995	20.03919	0.9193289
cg27534520	0.2107013	0.0457861	0.969619	0.0455459
cg19093939	1.7078164	0.4899154	5.953348	0.4008799
cg21155870	1.3929402	0.5731756	3.385145	0.4644686
cg27657603	1.3103529	0.4205096	4.0832	0.641138
cg25704227	0.1437327	0.0466073	0.443259	0.0007357
cg14284824	0.3893141	0.1314641	1.152904	0.0885504
cg23779644	9.1375961	1.119482	74.58419	0.038892
cg02343254	0.1509242	0.0363472	0.626682	0.0092326
cg13941441	136.92118	3.0719706	6102.731	0.0111082
cg15977893	3.5836917	1.162561	11.04703	0.0262696
cg15408080	1.2146247	0.6146047	2.400426	0.5758724
cg22563376	0.3592907	0.1364238	0.946241	0.0382835
cg21151950	3.5896656	0.9625924	13.38645	0.0570157
cg23684249	0.3020069	0.0989903	0.921385	0.0353929
cg25228625	0.1936873	0.0515681	0.72748	0.0150491
cg08323480	2.2951562	0.6467514	8.144926	0.198581
cg19779166	0.6230611	0.2904877	1.336391	0.2242993
cg19936022	5.7296202	0.1224666	268.0612	0.3736257
cg10030658	0.2759992	0.0472678	1.611574	0.1527429
cg09468777	1.328543	0.5481603	3.219909	0.5293808
cg14918359	0.1863965	0.0474543	0.732149	0.0161014
cg05804139	2.9696449	0.6988861	12.61835	0.1403221
cg00961866	2.0400065	0.9825411	4.235575	0.055785
cg06766659	3.41E-05	9.62E-08	0.012063	0.0005922
cg15860839	20281.821	20.3822	20181936	0.0048635
cg17008978	2.7184574	0.9429908	7.836779	0.0641261

cg11485154	0.0795725	0.0063851	0.991645	0.0492429
cg03646096	1.4946882	0.5348398	4.177126	0.4433744
cg02687288	1.3412101	0.2584441	6.960285	0.7267652
cg10942056	0.6118096	0.3212663	1.165111	0.1349177
cg06688396	0.0041097	2.74E-05	0.616396	0.0316156
cg11111139	0.4112285	0.1297382	1.303463	0.13112
cg24741225	113.72895	4.03E-05	3.21E+08	0.5322218
cg22349506	1.4201349	0.7138622	2.825172	0.3175612
cg23318703	1.4904049	0.4949053	4.488347	0.4780477
cg01710351	1.5834156	0.7430713	3.374111	0.2337986
cg11838439	1.1364625	0.3883187	3.325998	0.8153909
cg02571857	16.009396	2.1898176	117.0421	0.0062913
cg22704780	0.1221703	0.0428753	0.348116	8.32E-05
cg04964031	2.8666843	0.9185948	8.946142	0.0697189
cg14206172	0.1652628	0.041268	0.661816	0.0109888
cg16853712	4.1441671	0.1830847	93.80426	0.3717261
cg01647204	0.3637028	0.1499016	0.882444	0.0253188
cg25509732	7.4146555	0.1695377	324.2766	0.2986545
cg01607369	0.1293147	0.0376919	0.443658	0.0011458
cg02852025	1.6683934	0.8667317	3.211532	0.1255441
cg12865837	2.6804311	1.1205993	6.41149	0.0267012
cg27631724	0.1538044	0.0320947	0.737062	0.0192035
cg20482698	0.0529733	0.005018	0.559217	0.0145525
cg01225850	0.4944619	0.1951822	1.252638	0.1375405
cg03548857	1.3795021	0.5867707	3.243219	0.4607348
cg05004855	1.1541009	0.4152793	3.207357	0.7834511
cg16279290	1.7350218	0.6409841	4.696373	0.2781138
cg17740322	0.2873154	0.1214824	0.679524	0.0045161
cg26999725	1.6091817	0.7437348	3.481705	0.2270098
cg22854448	1.3360411	0.2962093	6.026164	0.706218
cg14550760	0.1783917	0.0604354	0.526572	0.0018004
cg09782637	0.0058903	2.35E-05	1.47891	0.0685811
cg09292226	1.7803566	0.6356819	4.986251	0.2723164
cg26472118	1.8872953	0.8076365	4.410256	0.1424758
cg15228606	8.2427686	0.4549009	149.3583	0.1535613
cg00151810	0.0225559	3.62E-12	1.4E+08	0.7417486
cg17024828	1.2791312	0.6889784	2.374786	0.4354865
cg11820270	0.408796	0.1815502	0.920485	0.0307702
cg07160746	0.8881797	0.4275305	1.845162	0.7505786
cg13835688	132.38573	2.7572077	6356.424	0.0133828
cg05228573	4.0745812	0.3763586	44.11274	0.2477284
cg07028929	1.6551103	0.7702002	3.556725	0.1967113
cg22609784	2.1144178	0.8312747	5.378201	0.1159501
cg11699517	0.9640416	0.0406394	22.86887	0.9819152
cg26090619	1.6561003	0.343572	7.982806	0.5295872
cg01101058	0.8293713	0.2453923	2.803091	0.763337
cg18155853	4.7089375	0.7671045	28.90622	0.0942109
cg00413380	2.4124814	0.7634054	7.623821	0.1335872
cg06679990	0.0989739	0.0259991	0.376777	0.0006961
cg09535072	8.4466985	0.5246314	135.994	0.1323262
cg14210830	0.4770633	0.1917089	1.187161	0.1115818
cg13094252	1.6722458	0.8250805	3.389252	0.1537197
cg24596472	0.000498	6.91E-07	0.358963	0.0235051
cg03170343	0.2587857	0.0693248	0.966033	0.0442848
cg07531549	1.6883565	0.5565896	5.121454	0.3549258
cg08132711	0.3339678	0.1368559	0.814977	0.0159763
cg00516481	0.3013479	0.1168638	0.777063	0.0130699
cg26784011	2.9721121	1.0017358	8.818144	0.0496358

cg22627406	1.3898308	0.4936442	3.913	0.5330917
cg10694914	3.9679154	1.2388272	12.70908	0.0203108
cg13625875	2.4160038	1.0558867	5.528126	0.036732
cg01098927	2.799E+10	0.0027006	2.90E+23	0.1156778
cg03398865	3.0226398	0.9670662	9.447493	0.0571229
cg10433327	0.4907904	0.2360429	1.020472	0.0566888
cg25202280	0.0695919	0.0070326	0.688659	0.0226717
cg22283925	2.5905114	1.1638734	5.765876	0.0197162
cg18871253	0.4851544	0.2301319	1.022782	0.0573328
cg03895159	0.2188753	0.0920507	0.520435	0.0005865
cg14359680	3.7366368	1.0989955	12.70474	0.0347598
cg23524195	1.2422971	0.7033946	2.194077	0.4546977
cg04780481	0.2220343	0.0008258	59.70048	0.5980175
cg26087408	3.5861158	0.9171686	14.02166	0.0664051
cg01284192	25.484026	0.7525748	862.9515	0.0715781
cg06162279	4.27E+38	2.94E-09	6.20E+85	0.10841
cg10111816	1.3515692	0.6484119	2.817251	0.4214468
cg07622996	0.4352413	0.1109857	1.706842	0.2328202
cg07289335	0.3141369	0.131707	0.749254	0.0090313
cg05507832	45.080251	0.1657813	12258.49	0.1829864
cg19229182	2.3654104	1.0094531	5.54277	0.0475218
cg11357369	0.4653124	0.2104153	1.028992	0.0588406
cg19189355	2.0570082	0.7556762	5.599333	0.1580501
cg12920963	0.3090383	0.1122441	0.850866	0.0230561
cg27209072	4.3754574	1.1762818	16.27554	0.027651
cg09838876	3406.9279	2.36E-13	4.92E+19	0.6683326
cg11149409	5.36E-12	1.61E-23	1.787086	0.0552282
cg09047573	0.4130074	0.1624795	1.049826	0.0631961
cg00839675	284921549	0.0022677	3.58E+19	0.1354387
cg11835347	0.3094272	0.053265	1.797527	0.1913078
cg05379509	3.6837788	0.2137211	63.49503	0.369364
cg07787632	1.9026971	0.7400359	4.892	0.1818377
cg16705138	0.2444655	0.0637655	0.937237	0.0399274
cg17919186	1.2371432	0.6120907	2.500484	0.5533639
cg13933311	3.98E-09	1.21E-31	1.31E+14	0.4646819
cg14682080	5.7770473	0.4086106	81.67747	0.1943764
cg10281770	0.276617	0.1155145	0.662402	0.0039213
cg15075988	0.3008492	0.1044101	0.866873	0.0261115
cg06358191	0.4764405	0.2332311	0.973264	0.0419193
cg08278440	0.0718487	2.90E-05	177.7137	0.5089137
cg11864327	0.3475738	0.124883	0.967366	0.0430223
cg10345337	1035.9931	21.566511	49766.13	0.0004405
cg24824842	0.508526	0.2307347	1.120762	0.0935034
cg08092513	10.457968	0.6750398	162.0187	0.093173
cg19497511	0.3166911	0.1192669	0.840915	0.0210154
cg12506582	0.343726	0.1413092	0.836092	0.0185384
cg06492521	0.3824006	0.1983509	0.73723	0.0041021
cg17789713	1.0326196	0.1731825	6.157107	0.9718923
cg02522041	1.5529318	0.3693374	6.529523	0.5480636
cg18760837	0.9703614	0.4334376	2.172403	0.9416712
cg02606423	2.3515096	0.6460047	8.559686	0.1945912
cg27360282	0.5590492	0.1076113	2.904306	0.4891138
cg02801114	1.249187	0.568509	2.744843	0.5796204
cg04399988	0.5151427	0.1974774	1.34381	0.1751302
cg26316423	3.9690826	1.3423909	11.73549	0.0126912
cg23829577	1.2953856	0.6514177	2.575957	0.4605634
cg04384112	0.3853259	0.1459777	1.017115	0.0541423
cg11068238	1.4511925	0.4603881	4.574314	0.5249529

cg27285720	2.1651627	1.2232498	3.832357	0.0080094
cg09938227	1.0961658	0.4867325	2.468665	0.8245745
cg13050884	0.0046208	9.29E-05	0.229815	0.0069822
cg15700776	0.0491146	0.0069262	0.348278	0.0025671
cg26344867	0.3919059	0.1774589	0.865497	0.0204869
cg02306612	0.2218999	0.0355344	1.385686	0.1071946
cg15204874	2.8288323	0.71065	11.26052	0.1401205
cg21896938	1.261E+11	94454.275	1.68E+17	0.0003825
cg24135977	1.4990677	0.7812512	2.876416	0.2233957
cg05349624	0.1431815	0.0503306	0.407326	0.0002688
cg00648301	0.1180033	0.0113517	1.226664	0.0736239
cg03602528	3.75E-19	2.49E-54	5.65E+16	0.3046062
cg06425430	1355.4844	9.22E-07	1.99E+12	0.503089
cg02791891	1.1220153	0.4399204	2.861696	0.8095566
cg01548249	1.2741406	0.1622431	10.00618	0.8177788
cg10813475	5.326E+17	0.0001289	2.20E+39	0.1079947
cg11481582	1.374127	0.6334364	2.980923	0.4211853
cg22888055	1.1648777	0.5385946	2.519409	0.6981929
cg21574752	0.6011751	2.75E-05	13153.98	0.9205008
cg24975834	1.1912344	0.604406	2.347825	0.6132151
cg01350330	0.1530879	0.038063	0.615714	0.0082192
cg23074992	1.7685646	0.7362921	4.248071	0.202216
cg19048251	1.8878022	0.9363876	3.805899	0.0756943
cg19623519	3.6429805	0.3970051	33.42856	0.2529895
cg19375537	2.0178973	0.9449861	4.308962	0.0697123
cg26655340	0.3142458	0.1036079	0.953117	0.0408762
cg02343604	1.8612959	0.6754443	5.129101	0.2296479
cg19082708	0.9367434	0.2877845	3.049115	0.9135829
cg01253818	2.2740106	0.7651817	6.758035	0.1393143
cg05169499	2.7164375	0.6053526	12.18964	0.1920103
cg04548483	0.1322462	0.0171556	1.019439	0.0521992
cg19736994	0.1453593	0.0121808	1.734643	0.1273722
cg14511782	3.2387018	1.0270906	10.21253	0.0449008
cg15745106	2.68E-09	1.89E-16	0.037909	0.0187984
cg01170124	1.6167476	0.5596665	4.670411	0.3747531
cg03445516	3.3222393	1.200949	9.19046	0.0207405
cg25863289	0.4266955	0.2135399	0.852623	0.0158923
cg20791007	0.3034254	0.1055101	0.872589	0.0269084
cg19138250	82.1639	1.0480027	6441.688	0.0475877
cg13866214	3.3584085	0.6150784	18.33735	0.1618717
cg13133883	0.1124241	0.0109374	1.155591	0.0660148
cg02321112	2.2094809	0.8504385	5.74034	0.1036525
cg15694605	1.1231148	0.5092138	2.477126	0.7735815
cg01250960	0.2928229	0.0737865	1.162072	0.0807441
cg13772514	3.3531889	1.2066728	9.318082	0.0203282
cg07676093	0.2095273	0.0617269	0.711225	0.0121947
cg04668521	0.3688234	0.1621527	0.838905	0.0173638
cg01923037	1.8304216	0.7328061	4.572074	0.1955391
cg08230177	1.7497006	0.9516848	3.216876	0.0717693
cg13650866	3.66E-13	7.37E-35	1.82E+09	0.2612153
cg09247944	0.0858937	0.005443	1.355445	0.0811773
cg26987928	2.0759764	0.4779563	9.016887	0.3296722
cg22001483	1.704277	0.7093387	4.094744	0.2332282
cg19475870	0.8294674	0.383397	1.794527	0.634885
cg21876656	6.0857081	1.4009167	26.43686	0.0159604
cg08791953	1.8529829	0.6579803	5.218311	0.2429708
cg12686920	0.3439435	0.1284701	0.920814	0.0336573
cg02200389	75.876244	0.4324547	13312.85	0.1005881

cg25542041	1.8301714	0.9165171	3.654626	0.0867292
cg04387597	2.663419	0.8977486	7.901768	0.07747
cg18764164	0.7149785	0.3479631	1.469105	0.3611908
cg04837231	2.0259073	0.0876917	46.80374	0.6594313
cg06666194	2.6991223	0.8304541	8.772624	0.0987297
cg01020840	0.0705863	0.0118537	0.420327	0.0035904
cg04751839	3.440721	1.0188659	11.61935	0.0465838
cg21770330	0.2118905	0.0416192	1.078772	0.0616713
cg14901671	5.59E-05	2.13E-07	0.014647	0.000568
cg02897008	1.9713398	0.9395706	4.136124	0.0726372
cg01568319	0.0684514	0.0039877	1.175001	0.0644894
cg10211456	1.1903918	0.3521617	4.023813	0.7791234
cg02462195	1.6052913	0.750622	3.4331	0.2223312
cg00004429	0.0943337	0.0351382	0.253253	2.79E-06
cg25340466	2.4126965	0.9401833	6.191457	0.0669981
cg09527615	0.3616647	0.1367459	0.956528	0.0404104
cg11888571	16298.261	0.0119036	2.23E+10	0.1785146
cg00290758	0.0872427	0.0269171	0.282767	4.80E-05
cg06891458	0.7162397	0.249414	2.056818	0.5352074
cg01765152	1.1388161	0.4952973	2.618432	0.7596021
cg16531903	0.5819861	0.1852239	1.82864	0.3540894
cg13667782	0.4116777	0.1754076	0.966198	0.0414537
cg02948476	1.3213461	0.7574757	2.304966	0.3263243
cg01446692	0.7925554	0.3518349	1.785337	0.5747225
cg00701890	0.5189094	0.1569596	1.715518	0.2822377
cg01479122	5.26E-07	4.11E-11	0.006744	0.0027363
cg13799772	0.5516367	0.2413969	1.260592	0.1583167
cg24044052	2.0549959	0.8931634	4.728147	0.0902267
cg17796323	1.7997245	0.7116545	4.551378	0.2144683
cg10519766	0.0106394	1.99E-29	5.69E+24	0.8849606
cg21833076	0.2674713	0.0581646	1.229974	0.0902537
cg15881332	1.6110836	0.7272983	3.568811	0.2398876
cg14871333	0.0146424	0.0004503	0.476085	0.0174185
cg14611112	2.22621	0.6385481	7.761375	0.2091178
cg27405400	1.57833	0.701791	3.549669	0.2697613
cg13153049	243.31427	1.5183887	38989.91	0.0339045
cg05352541	2.09E+28	4.94E-17	8.81E+72	0.2135759
cg10517014	0.6200646	0.2960447	1.298723	0.2051462
cg23639541	4.3154778	1.8383971	10.13021	0.0007836
cg24898863	0.1374692	0.0290069	0.651492	0.0124283
cg03732535	0.0252535	0.0019129	0.333383	0.0052007
cg11776014	0.5314999	0.1858057	1.520363	0.2385237
cg02431562	1.3881248	0.3776171	5.102763	0.6214829
cg04997558	1.1879348	0.0113873	123.9262	0.9421019
cg08774009	2.0927068	0.4305436	10.17184	0.3599972
cg04383154	1.5471092	0.1483023	16.13965	0.7152961
cg16308841	1.5844629	0.1037246	24.20374	0.7407354
cg01837574	0.2053437	0.0571399	0.737944	0.015284
cg02697989	2.3275166	0.6574925	8.239385	0.190255
cg02756845	0.2669426	0.057795	1.23295	0.0906978
cg18546622	2.653106	1.1890433	5.919861	0.0171812
cg07359991	0.4689056	0.1449653	1.516725	0.2060565
cg16520046	1.3640525	0.6252672	2.97575	0.4353439
cg10632917	6.70E-06	8.14E-15	5515.245	0.2553597
cg03848545	0.2752386	0.0974389	0.777475	0.0148899
cg24166295	0.5033329	0.2384937	1.062267	0.0716313
cg20941258	4.9061412	1.1196316	21.49834	0.0348701
cg15844365	5.64E-08	5.06E-31	6.3E+15	0.5376292

cg03600687	0.0666171	0.0116786	0.379998	0.0022951
cg07265985	40.164323	0.0245037	65833.97	0.3281387
cg26207568	0.3824082	0.1703661	0.858363	0.0197962
cg24908197	0.3725274	0.1482931	0.935827	0.0356331
cg05634915	0.3728607	0.1851391	0.750922	0.0057465
cg10106639	0.2798928	0.0934491	0.838317	0.0229023
cg14149988	0.2077168	0.0654978	0.658744	0.0076119
cg16530491	20.989071	0.5574839	790.2311	0.1001096
cg21907579	2.8023683	1.4361257	5.468371	0.0025183
cg13495118	0.2333707	0.097273	0.559887	0.0011179
cg16711096	0.3380683	0.1355276	0.843298	0.0200499
cg24535130	0.9700439	0.4565893	2.060901	0.936949
cg13496596	0.1664915	0.0409784	0.676439	0.0121936
cg18227471	1.4829161	0.6069141	3.623314	0.3873621
cg00481644	2.8576429	0.7934956	10.29133	0.1082428
cg11942450	20.995665	1.362908	323.4393	0.0291191
cg07954706	1.4649371	0.5055589	4.244888	0.481813
cg01434160	1.9476987	0.8219033	4.615543	0.129921
cg06620723	2.1190219	0.5054903	8.882967	0.3044313
cg04352505	1.0990591	0.5917014	2.041453	0.7649596
cg11780546	3.1701966	1.5011855	6.694806	0.0024853
cg13882486	0.2066186	0.0555863	0.768018	0.0185737
cg07455504	1.1200175	0.3480065	3.604644	0.8492663
cg12749132	2.2597247	0.9693104	5.268029	0.0590547
cg25503999	1.5441015	0.6681572	3.568396	0.309394
cg17812850	0.0128221	1.13E-30	1.45E+26	0.8948379
cg03885684	3.32E-06	4.90E-42	2.25E+30	0.7644329
cg13458651	0.0638498	0.0064919	0.627978	0.018331
cg18594669	0.9960152	0.4238163	2.340746	0.9926927
cg21783672	0.2611228	0.0955376	0.7137	0.008859
cg25305703	0.3366037	0.1407325	0.805088	0.0143954
cg15237829	0.6084529	0.1984757	1.865291	0.3847101
cg19474239	0.2696175	0.0706131	1.029463	0.0551763
cg13728287	4.025004	0.6958028	23.2834	0.119954
cg07176692	1.1954627	0.3210821	4.450983	0.7901001
cg22813711	1.5606655	0.4692309	5.190785	0.467881
cg15177917	0.0344262	0.0052475	0.225852	0.0004477
cg07194839	1.1015104	0.5239899	2.315551	0.7986839
cg19390582	0.2564215	0.092173	0.713354	0.0091336
cg23075139	1.9726569	0.6852127	5.679076	0.2079322
cg17592534	0.14419	0.0009173	22.66561	0.4529442
cg18770763	1.0500275	0.433768	2.541815	0.9138165
cg21377489	1.0878546	0.5033602	2.351055	0.8304225
cg26383057	4.6220759	0.8721645	24.4949	0.0719856
cg13491234	6.1233501	1.6809121	22.30659	0.0060083
cg07062658	0.0731998	0.006028	0.888883	0.040128
cg02578070	2.3640759	1.1719363	4.768907	0.0162567
cg25531986	1.5535115	0.4498151	5.365311	0.4860493
cg20322611	0.0434062	0.0047553	0.396207	0.0054268
cg24955895	1.1772155	0.3361101	4.123162	0.7986384
cg02166394	1.5165012	0.8549412	2.689982	0.1544437
cg03722643	19.68081	1.7521956	221.0565	0.0157593
cg14143580	0.2479056	0.0740378	0.830079	0.0236969
cg21073748	0.5536357	0.0794544	3.857716	0.5505571
cg11315979	0.0011099	1.36E-05	0.09039	0.00244
cg00511334	0.2165202	0.0660787	0.709473	0.0115112
cg03100044	0.1886034	0.0714354	0.497949	0.0007583
cg24696028	2.13E-05	2.08E-09	0.219177	0.0224883

cg12259522	0.0445578	0.0052622	0.377298	0.0043139
cg00820740	3.0132207	0.5186921	17.5046	0.2191806
cg01634423	0.2818978	0.1240495	0.640602	0.0025002
cg08308286	3.553E+12	1.2656213	9.97E+24	0.0481467
cg10914667	1.6665393	0.6125497	4.534087	0.3172256
cg17281638	99.504579	1.4043438	7050.383	0.0343302
cg16276850	0.1972045	0.0825708	0.470985	0.0002571
cg07215697	8.4500893	0.2477859	288.1682	0.2359491
cg27485402	1.0701699	0.2892581	3.959313	0.9190735
cg14598764	0.1860911	0.0051979	6.662227	0.3569915
cg19446990	0.2752042	0.0839933	0.901707	0.0331025
cg14716734	5.8809096	1.4594078	23.69804	0.012717
cg06532856	3.011516	0.9198421	9.85955	0.0684725
cg21197336	0.1580854	0.050695	0.492968	0.0014784
cg11942594	0.7841632	0.391041	1.5725	0.4934202
cg20605886	1.671667	0.7187793	3.887801	0.2327985
cg15209921	2.1396659	0.8710262	5.256065	0.0971498
cg05442477	2.5480575	0.8849575	7.336621	0.0830139
cg14840351	1.3243702	0.5569207	3.149383	0.5250179
cg02888518	0.0640422	0.005285	0.77605	0.0308379
cg08483570	0.7389236	0.4056297	1.346076	0.3227845
cg17971328	0.4554297	0.186678	1.111091	0.0839056
cg14039622	2.6198537	0.9455285	7.259044	0.0639907
cg01753263	0.1871882	0.0599916	0.584072	0.0038997
cg20417500	1.3113158	0.5133175	3.349874	0.5711297
cg03160788	3.112763	0.3817386	25.38201	0.2889022
cg08877967	2.70E-06	1.91E-11	0.382436	0.0341073
cg08058160	0.5581444	0.2675582	1.164327	0.1200862
cg08278108	1.5760829	0.7027501	3.534738	0.2696071
cg02270010	0.9351568	0.2264519	3.861828	0.9261796
cg16467775	0.3073044	0.0998729	0.945561	0.0396307
cg08787837	1.8112407	0.9302266	3.52666	0.0805989
cg11290182	0.385306	0.1741258	0.852606	0.0186001
cg06874891	0.4921814	0.2054572	1.179041	0.1117329
cg19262563	0.5096893	0.2267627	1.145617	0.1028948
cg08590069	1.8998665	0.8606625	4.193854	0.1121617
cg05620821	0.3054402	0.0406479	2.295166	0.2490851
cg21088108	0.0461452	0.0026998	0.788726	0.0336839
cg20616186	0.2957845	0.0994713	0.879535	0.0284644
cg16686279	0.3889144	0.1467149	1.030941	0.0576038
cg20114732	1.2710741	0.668974	2.415086	0.4639111
cg08718490	1.3980238	0.7385481	2.646369	0.3034278
cg24207161	1.5243425	0.7842358	2.96291	0.2137902
cg00259834	1.646274	0.8037945	3.37178	0.1729264
cg17873456	2.2887646	0.8604687	6.087896	0.0971383
cg25569575	685943347	0.0048532	9.69E+19	0.1203705
cg22057240	0.2782974	0.0598472	1.294121	0.1028569
cg00429513	2307.1966	0.001421	3.75E+09	0.2885295
cg15721424	3.37E+30	19.105078	5.94E+59	0.040774
cg25839877	0.1472195	0.045884	0.472356	0.0012779
cg12747076	6.4058699	0.1903741	215.5502	0.3005314
cg19635644	1.4792032	0.2049816	10.67433	0.6978237
cg11599539	1.4369205	0.8153818	2.532238	0.2098589
cg12581730	118.98962	5.7274001	2472.069	0.0020186
cg06447341	0.1701257	0.0591381	0.48941	0.0010185
cg12595742	0.0760052	0.0102591	0.563089	0.0116675
cg14290127	0.011843	0.0001813	0.77355	0.0374907
cg09409486	0.7141381	0.3596216	1.418139	0.3361057

cg17798563	6.9824596	0.8789096	55.47185	0.0660775
cg19464087	0.4746797	0.2116597	1.064543	0.0705779
cg08446824	0.3502782	0.1692567	0.724904	0.0046997
cg23992426	0.2514023	0.0090505	6.983383	0.415611
cg03485669	0.441515	0.1654576	1.17816	0.102561
cg22108374	0.2735549	0.1124086	0.665717	0.0042812
cg07274516	5.9857632	0.3529154	101.5239	0.215394
cg14831174	0.0448335	0.0028101	0.7153	0.0280163
cg18896032	2.9516826	0.6054813	14.38926	0.1805096
cg03290871	0.3698942	0.1456015	0.9397	0.0365542
cg15352671	0.221016	0.0584745	0.835373	0.0260738
cg13052034	1.746651	0.967844	3.15215	0.0641037
cg06808571	0.6390026	0.2076915	1.966013	0.4347851
cg18173058	2.0641466	1.0414592	4.091088	0.0378616
cg25147866	0.0013865	3.90E-16	4.93E+09	0.6553631
cg06678594	0.3050724	0.1311044	0.709886	0.0058664
cg09794680	2.2172393	0.5927626	8.293623	0.2368078
cg20457962	731694264	3.90E-10	1.37E+27	0.3417291
cg00652908	2.2355954	0.8781954	5.691088	0.0915039
cg26015163	1.2662029	0.5795378	2.766463	0.5539192
cg15645986	1.4763279	0.0536133	40.65302	0.8178689
cg16878245	0.4146154	0.1959772	0.877173	0.0212939
cg27247736	2.0430977	0.8888232	4.696376	0.0924848
cg04369588	0.1567064	0.0616284	0.398467	9.93E-05
cg21999939	1.3650744	0.7716273	2.414933	0.284964
cg04399085	0.2922746	0.0930409	0.918138	0.0351866
cg00512872	0.4313244	0.1852858	1.004075	0.0511128
cg26933107	0.4557652	0.1761243	1.179405	0.105273
cg21606490	2.295552	0.9870416	5.33874	0.0536471
cg23032536	0.4453924	0.0011804	168.0634	0.7893304
cg17543539	1.2784314	0.5891249	2.774262	0.5343337
cg24046616	0.6503195	0.2528238	1.672768	0.3720412
cg12434312	0.8310005	0.3586002	1.925715	0.6659354
cg05217331	0.9191707	0.2786515	3.032012	0.8899172
cg06383233	1.9147619	0.7396534	4.956799	0.1807189
cg01974660	5.50E-07	7.74E-15	39.10933	0.1181708
cg17551295	3.6980085	0.7958451	17.18333	0.0951943
cg21665078	103.45854	0.4218241	25374.72	0.0984329
cg09388080	0.3102891	0.1489086	0.646566	0.0017833
cg24786986	0.3826302	0.1881045	0.778322	0.0080083
cg15332871	0.2017915	0.0613726	0.663485	0.0084013
cg11990813	0.0060409	0.0002314	0.157679	0.0021417
cg14283140	0.6515756	0.1193675	3.55667	0.6208221
cg00623826	2.49E-06	2.86E-15	2169.4	0.2192477
cg10768564	0.1265625	0.0079947	2.003597	0.1424269
cg26529645	2.7718598	0.6106247	12.58254	0.1865405
cg23204746	0.9444117	0.14989	5.950455	0.9514388
cg09537568	0.3405154	0.0125971	9.20452	0.5218993
cg14499928	1.8203458	0.706986	4.687022	0.2144618
cg01719100	0.3868214	0.1678495	0.891458	0.025768
cg05255351	1.4593328	0.3504708	6.076547	0.603519
cg03826463	2.415496	1.0648413	5.479334	0.0348326
cg13299728	0.1928952	0.0540977	0.687803	0.0111831
cg13739115	0.3296275	0.177832	0.610994	0.000424
cg16720005	2.8603949	1.4727179	5.555619	0.0019165
cg10167266	1.90462	0.8888205	4.081339	0.0975459
cg04511411	0.818608	0.2797674	2.395272	0.7148295
cg03534655	1.6670747	0.7494263	3.708354	0.2102586

cg05305025	3.85E-09	1.22E-19	121.1819	0.1161816
cg16133703	6.024419	1.1315239	32.07499	0.0353097
cg24452282	3.312826	1.0838916	10.12538	0.0356159
cg06786804	1.7546941	0.4536478	6.787097	0.4152403
cg07587746	0.9328037	0.4910521	1.771956	0.8317331
cg11377047	0.4975065	0.1936527	1.278127	0.1469978
cg01355392	0.9611032	0.449222	2.056265	0.9185678
cg24401026	0.3035678	0.1322931	0.696585	0.0049057
cg19477921	2.0016792	0.2039192	19.64856	0.551493
cg19073171	0.3610839	0.08676	1.502784	0.1614803
cg11863380	0.6212991	0.3412368	1.131216	0.1195417
cg22697684	5.7735539	1.1114962	29.99014	0.0370046
cg21754388	1.4170173	0.6547897	3.066539	0.3762001
cg23010507	0.8593322	0.4469021	1.652379	0.6495015
cg08259810	1.2100713	0.6829527	2.144032	0.5135279
cg17827767	3.0253937	0.7453817	12.27962	0.1214218
cg08075719	1.3897468	0.4793399	4.029283	0.5445151
cg09883659	0.6315099	0.2683002	1.486413	0.2926058
cg19645616	0.3863893	0.1503825	0.99278	0.0482664
cg18755984	2.4075657	0.0860394	67.36882	0.6052324
cg03407594	0.1666651	0.0524955	0.529135	0.002367
cg05850327	2.1971068	0.9345463	5.165371	0.071113
cg03234557	2.3097759	0.9313207	5.728494	0.0708511
cg19280572	1.5572897	0.7215457	3.36105	0.2591101
cg25147447	2.3210533	1.0817724	4.980057	0.0306367
cg00536080	6.0274657	1.8611907	19.51995	0.0027347
cg00135293	2.107747	1.0313307	4.307636	0.0408985
cg03388575	0.7764981	0.2535538	2.377994	0.6577781
cg07168392	1.14E+26	6.745E+09	1.93E+42	0.0016492
cg21572599	0.2942556	0.1417403	0.61088	0.0010293
cg11902180	0.2883203	0.1274339	0.652327	0.0028313
cg20971488	4.5457504	0.9400475	21.9817	0.0596899
cg09141835	6.0926584	1.4878796	24.94858	0.0119911
cg23929609	0.8200612	0.40531	1.659225	0.5811415
cg23777956	1.1649637	0.5747299	2.361354	0.6718841
cg06319102	2.62E-08	4.34E-18	158.2429	0.1287026
cg05929882	1.3966844	0.6854385	2.845955	0.357593
cg19212779	0.2627771	0.1097149	0.629375	0.0027087
cg10402987	2.4965824	1.1863111	5.254038	0.0159527
cg08753736	0.2088559	0.0844157	0.516738	0.000703
cg00837831	0.4194358	0.0618469	2.844545	0.3736836
cg10886225	0.4226156	0.1837127	0.972191	0.0427326
cg16309727	0.6119664	0.2828466	1.324049	0.2123526
cg16929199	2.396349	1.0054049	5.711618	0.048595
cg24492119	0.3525959	0.0437305	2.842957	0.3276544
cg13072940	0.036401	0.0033273	0.398229	0.0066425
cg01761966	1.651641	0.6684324	4.081068	0.2769581
cg26899496	1982.6962	1.0593636	3710798	0.0482721
cg11881472	0.4326801	0.0671956	2.786078	0.3779675
cg01881464	0.2396873	0.0475603	1.20794	0.0834471
cg07963725	1.151E+10	0.0179702	7.38E+21	0.0948776
cg05412696	0.3885316	0.1878919	0.803424	0.0107588
cg06899522	0.2924707	0.1435349	0.595946	0.0007112
cg14214182	1.6654265	0.7693913	3.604987	0.1954569
cg01600222	2.0449887	1.1178485	3.741096	0.0202609
cg12048225	1.588488	0.5771085	4.372305	0.3703422
cg09359064	1.1685972	0.5713464	2.390178	0.6695579
cg00718452	0.0120411	0.000113	1.283471	0.0635677

cg20054939	286.58006	4.60E-06	1.78E+10	0.5366394
cg25763292	3.45304	0.155384	76.73562	0.4334898
cg07116712	2.0960519	0.5720951	7.679551	0.2639774
cg03554394	0.6699522	0.3431738	1.307897	0.2405799
cg19206437	1.6422731	0.3895582	6.923384	0.4991921
cg22011319	2.75E-07	1.43E-16	528.3684	0.1660314
cg05651657	0.3314336	0.1263202	0.869601	0.0248415
cg03361102	3.3533148	1.2948581	8.684133	0.0126951
cg16764274	2.1603255	0.8151659	5.725222	0.1213843
cg07950786	5.0468731	0.8586781	29.66295	0.0732358
cg16541931	0.0061705	0.0001284	0.296425	0.0100104
cg06697536	0.1484695	0.0142325	1.548789	0.1108693
cg23636833	0.2039521	0.052647	0.790102	0.0213959
cg13788685	0.4377816	0.1454605	1.317559	0.1417265
cg20921890	2.5907517	0.7677743	8.742145	0.1250051
cg19093405	0.6706974	0.3343719	1.345313	0.2607044
cg03745372	1.3323252	0.5921444	2.997732	0.4880084
cg00502190	4.0599638	1.2555569	13.12828	0.019282
cg22260869	2.8858942	1.2826275	6.493222	0.0104201
cg08193082	1.6348829	0.8422091	3.173609	0.1463537
cg10021122	0.1592626	0.0423415	0.599047	0.0065666
cg18219418	0.4861544	0.2179582	1.084365	0.0780555
cg03155112	1.3518423	0.3811658	4.794442	0.6406976
cg21086326	0.1358712	0.0173942	1.061327	0.0570128
cg24312520	1.8853019	0.6426272	5.530988	0.2482092
cg19592898	2.8632554	0.3510167	23.35567	0.3259352
cg11869007	1.6806085	0.6414403	4.403286	0.2907819
cg14666564	1.1462348	0.583736	2.250768	0.6917942
cg05483131	1.43E-10	1.48E-17	0.001385	0.0057462
cg02600394	2.0969403	0.6172332	7.123983	0.2353481
cg26540630	5.8192565	2.0895267	16.20642	0.0007512
cg07095252	0.3052709	0.1258837	0.740289	0.0086571
cg15632675	3.3757674	0.5980847	19.05383	0.1682556
cg23032045	1.243583	0.4752668	3.253959	0.6568975
cg27002247	0.1562048	0.0356908	0.683648	0.0137059
cg20697417	1.5709709	0.4557488	5.415153	0.4743664
cg26874229	2.9320908	1.0218705	8.413157	0.0454796
cg14909842	12.439238	1.4541493	106.409	0.0213432
cg06728497	0.4158774	0.0887287	1.949246	0.2656438
cg11995741	5.67E+27	0.9855788	3.26E+55	0.0500521
cg24834590	0.3836888	0.1651136	0.891611	0.0259717
cg13346655	0.0027044	7.94E-06	0.921082	0.0468562
cg05756754	1.3808913	0.3829105	4.979913	0.6219163
cg13598256	1.5976568	0.798599	3.196231	0.1854022
cg23139173	8.9050514	0.2863081	276.9742	0.2124643
cg23414759	4.5984726	0.2185077	96.77438	0.326335
cg15454857	587083.68	0.0019265	1.79E+14	0.1826333
cg04001071	0.7948724	0.4112718	1.536264	0.4946942
cg07483304	2.4542438	0.7656886	7.86653	0.130858
cg05385282	1.2655468	0.4810717	3.329252	0.6332114
cg15792134	1.5622313	0.7527743	3.242096	0.231075
cg22456162	1.9377346	0.7954511	4.72036	0.145336
cg22222281	0.5818712	0.3170163	1.068002	0.0805269
cg07439409	3.99E-07	1.08E-15	147.7242	0.1432679
cg01176028	4.4265042	1.7822547	10.99391	0.0013508
cg26967385	0.2726605	0.1086355	0.684341	0.0056433
cg13781869	1.0993066	0.3077241	3.927137	0.8841217
cg02687026	1.8103111	0.6757182	4.849989	0.23785

cg21833776	0.4701152	0.2253519	0.980725	0.0442366
cg08710911	0.2103067	0.0785315	0.5632	0.0019204
cg04409509	0.3951364	0.1445573	1.080076	0.0703238
cg08209711	1.869617	0.2267342	15.41659	0.5610253
cg17799563	0.2977882	0.1151195	0.770311	0.0124852
cg22676401	1.3755588	0.7613388	2.485309	0.2907433
cg12091414	0.2766372	0.0903389	0.847123	0.0244158
cg11809014	0.4638304	0.2300659	0.935117	0.0317547
cg09217215	1.0968019	0.6010187	2.001559	0.7633664
cg01955745	0.3124811	0.1020437	0.956888	0.0416358
cg26796679	0.1094789	0.0109114	1.098449	0.0600873
cg12869623	0.0126588	0.00019	0.84339	0.0414036
cg04278110	0.3185822	0.1388474	0.73098	0.0069443
cg06144990	2.1407484	0.7180654	6.382154	0.1720289
cg01970322	2.0835679	0.5975814	7.264709	0.2493252
cg10428805	2.64E-17	6.34E-29	1.10E-05	0.0051662
cg11798758	0.1789417	0.0143681	2.228559	0.1811554
cg20466291	198.51898	3.705251	10636.2	0.0091936
cg08484560	3.7732871	1.1506362	12.37376	0.0284136
cg23333072	0.1106249	0.0193021	0.634018	0.0134547
cg04293930	0.1302314	0.0267075	0.635035	0.0116795
cg15880846	6.4475854	0.564565	73.63432	0.1336476
cg11369686	1.59E-05	1.35E-15	187860.2	0.3505057
cg03133868	2.4600645	0.7828767	7.730358	0.1233295
cg04923496	0.4446592	0.0010741	184.0849	0.7920837
cg19739482	3.5909513	0.0493236	261.4356	0.5589703
cg07793203	3.03E-17	3.79E-29	2.41E-05	0.0065224
cg09620016	7.97E-11	8.47E-20	0.07489	0.0273968
cg27019731	1.1042456	0.5615861	2.171276	0.7737738
cg15505276	0.2026067	0.0679296	0.604294	0.0041918
cg01804429	0.2599106	0.1015646	0.665128	0.0049461
cg13639157	0.4185953	0.0994935	1.76114	0.2348599
cg08461576	0.3618592	0.1514129	0.864802	0.0222115
cg05103718	6.234882	1.2221339	31.8081	0.0277194
cg04817379	0.0052667	9.07E-07	30.57327	0.2354301
cg17186388	3.5122948	0.7455674	16.54608	0.1121354
cg04266908	1.9591443	0.4859925	7.897747	0.3444044
cg04506569	2.1220625	0.9737926	4.624341	0.058339
cg00536532	1.7272397	0.4053683	7.359621	0.459907
cg23442209	1.6316396	0.7942733	3.351803	0.1825657
cg17226917	27.87632	0.3983749	1950.648	0.1247012
cg14325930	1.2230375	0.6044394	2.474724	0.5755461
cg16038120	1.4447263	0.5741139	3.635575	0.4345693
cg24824917	0.0055026	0.0001795	0.168668	0.0028904
cg16516490	0.4844867	0.2577433	0.910702	0.0244203
cg10184649	0.0009154	3.99E-16	2.1E+09	0.6299609
cg09700085	0.3163028	0.0955072	1.047539	0.0595718
cg25880365	4.6742697	0.3963143	55.12998	0.2206406
cg23783076	179.47692	0.1813235	177649.2	0.1402724
cg01432329	0.5540978	0.2817607	1.089664	0.087062
cg10778915	0.3259488	0.1246232	0.852511	0.0222978
cg23243408	1.8439456	0.3611818	9.413917	0.4619436
cg07495027	0.4113881	0.167053	1.013093	0.0533996
cg20426959	1.7754818	0.7108957	4.434316	0.2189675
cg13300521	3.1420695	0.9386276	10.51812	0.0632799
cg16648140	2.7124498	0.6739609	10.91663	0.1601531
cg00208504	3.319409	1.4043307	7.84607	0.0062641
cg16011679	304.93612	0.3981997	233516.1	0.0913723

cg20084504	1.7260365	0.8034274	3.708116	0.1618158
cg03164070	0.4105452	0.2057662	0.819121	0.0115335
cg03960220	2.1502345	0.6828448	6.77095	0.1908311
cg12078775	2.7458747	0.8732743	8.633974	0.0839643
cg14551952	2.0643164	0.8997435	4.736241	0.0871502
cg04801716	8.02E+20	0.0335479	1.92E+43	0.0671248
cg13501202	1.9515552	0.512212	7.43553	0.3272361
cg12682931	0.2757615	0.0688346	1.104742	0.0688676
cg05202389	0.0420303	0.0007824	2.257916	0.1189319
cg14646244	1.2958115	0.5742845	2.92386	0.53254
cg25388738	0.6405698	0.3280431	1.25084	0.1920762
cg08711175	1.1813786	0.6828995	2.04372	0.5511394
cg06380725	0.2917294	0.1260848	0.674991	0.0039981
cg15617609	0.3929487	0.1770399	0.872169	0.0216654
cg10393369	3.3747453	1.373054	8.29458	0.0080268
cg08506654	2.9742767	1.0442791	8.471223	0.0412413
cg26396492	0.1348036	0.0369446	0.491872	0.0024107
cg18222853	0.3078735	0.1118419	0.847501	0.022594
cg23421560	2.1305396	0.8083306	5.615523	0.126105
cg04875514	7.1018227	1.6822669	29.98091	0.0076345
cg23848889	4.0227962	1.5312375	10.5685	0.0047349
cg27657685	0.1350011	0.0107065	1.702263	0.1214823
cg14089714	1.2087237	0.591052	2.471886	0.6035272
cg13535231	0.3763993	0.0286822	4.939531	0.456934
cg01973456	0.4052086	0.1631885	1.006162	0.0515676
cg15986671	2.7164586	0.6518506	11.3203	0.1699684
cg07587250	0.4588757	0.2199207	0.957467	0.0379139
cg11467506	2.1534307	0.7848689	5.908329	0.1363399
cg20820622	0.1945796	0.0548498	0.690271	0.0112862
cg16867086	0.4597907	0.2333913	0.905807	0.0247088
cg03043078	0.4952885	0.2251628	1.089482	0.0806571
cg25014229	0.2560143	0.0970136	0.67561	0.0059232
cg11523350	0.3208102	0.1261002	0.816169	0.0170172
cg26357596	2.109373	1.0024017	4.438794	0.0492659
cg07757252	3.9438908	1.1211166	13.87391	0.0325082
cg13601740	0.1397531	0.0388175	0.503148	0.0026048
cg21848524	3.1589137	1.0958397	9.106018	0.0332216
cg04556519	0.8256534	0.3705502	1.839706	0.639307
cg17972213	3.6611083	1.1214796	11.95181	0.0315637
cg10580269	0.0761627	0.0070923	0.81789	0.0335082
cg03116557	1.6264686	0.2054125	12.87848	0.6449817
cg22671342	0.43273	0.1945548	0.962481	0.0400026
cg18209835	0.3348103	0.1247346	0.898692	0.0298561
cg10129518	0.0001353	3.34E-07	0.054852	0.0036436
cg26506007	3.4871591	0.8422287	14.43821	0.0848703
cg24975642	0.1402819	0.0437043	0.450276	0.0009637
cg21230427	2.1305519	0.9863943	4.601863	0.0542171
cg00988708	0.9598819	0.4606405	2.0002	0.9129608
cg13223209	0.5346492	0.2389343	1.196353	0.1275841
cg24035107	0.350764	0.1710322	0.71937	0.004253
cg00189101	0.0398137	0.0011972	1.324063	0.0713941
cg16791601	0.3017177	0.0188229	4.836308	0.3972717
cg05239609	5.35E-09	1.19E-16	0.241159	0.0341627
cg13199429	0.511894	0.1561032	1.678604	0.2690985
cg20053465	0.0674611	0.0131305	0.346598	0.0012427
cg25575349	0.6013106	0.2243189	1.611877	0.312
cg25735922	0.0040164	0.0001317	0.122445	0.0015537
cg27074995	0.2341674	0.0845772	0.648335	0.0052063

cg26507637	6.9937632	0.872421	56.0655	0.0670334
cg25966809	1.9396731	0.8201415	4.587418	0.1314264
cg04091822	6.1569971	1.4475176	26.18871	0.0138678
cg08600461	13.492388	1.2563059	144.9046	0.0316861
cg05770626	0.1416031	0.0180496	1.110908	0.0629012
cg23819145	1.6108616	0.3675417	7.060084	0.5271429
cg19544065	0.3573179	0.1089531	1.171844	0.0894556
cg05494776	2.6777773	0.7989357	8.975054	0.1104448
cg05102394	0.323679	0.1503196	0.696969	0.0039453
cg08273740	2.0428637	0.9516441	4.385349	0.0668326
cg17805875	17.528099	0.1167705	2631.096	0.2626931
cg09607915	1.6138656	0.4135167	6.298565	0.4908705
cg24451981	6.16E+20	568539.66	6.68E+35	0.0067252
cg10837858	0.0029092	4.27E-05	0.198006	0.006687
cg01465361	2.02E+23	1.93E-06	2.11E+52	0.1154768
cg07437464	0.1196765	0.0304529	0.470315	0.0023638
cg23757825	4.7927829	1.1213176	20.48551	0.0344758
cg01797381	1.929117	0.3225159	11.53894	0.471532
cg13160386	1.2057781	0.3480904	4.176791	0.7678432
cg08036514	0.3552755	0.1192157	1.058759	0.063243
cg14617594	28180491	0.0003737	2.13E+18	0.1794753
cg10040748	0.0867353	0.0077949	0.965113	0.0467177
cg09233429	0.1497312	0.0523798	0.428017	0.0003949
cg01913077	0.4384086	0.0194154	9.899472	0.6041127
cg21307155	2.1062729	0.693913	6.393288	0.1885299
cg18080370	0.0432448	0.000235	7.957591	0.2378247
cg23340350	1.4533632	0.6221666	3.395014	0.3877495
cg16075769	5.42E-18	7.80E-33	0.003771	0.0226072
cg07233908	2.24E-09	4.28E-27	1.17E+09	0.3386948
cg08681473	3.1976829	0.8357119	12.23529	0.0895399
cg21546532	0.6463782	0.3436107	1.215925	0.1758823
cg02298327	3.9764041	0.8968776	17.62982	0.0692593
cg14233838	1.1534774	0.6582707	2.02122	0.6178462
cg08573478	10.84701	2.09E-07	5.64E+08	0.7925692
cg02838178	0.0695341	0.0107687	0.448986	0.0050878
cg10093265	1.9112332	0.8726249	4.186005	0.1053729
cg25746394	2.5100771	0.5425103	11.61358	0.2389923
cg22961241	0.3915611	0.1215785	1.261079	0.1161278
cg09989134	3.67E-20	7.07E-41	19.01172	0.0659228
cg01037823	1.6034678	0.5794783	4.436938	0.3632167
cg06794612	1.6270323	0.7886667	3.356594	0.1877013
cg07216647	5.7063972	0.2569215	126.7429	0.2709361
cg23002907	3.47E-07	1.64E-12	0.073251	0.0174171
cg13365524	2.3334809	1.0411416	5.229964	0.0396018
cg07120889	0.8931192	0.301796	2.643051	0.8382014
cg15957004	0.3320128	0.0791786	1.3922	0.1316702
cg04888874	35.387591	0.9376705	1335.524	0.0542015
cg06003958	0.6540337	0.2658414	1.60908	0.3552815
cg02103924	0.4645115	0.2024086	1.066016	0.0704309
cg23793336	0.5080961	0.2520755	1.024144	0.058324
cg23165310	1.9616711	0.7931747	4.851584	0.1447231
cg04944853	0.5001113	0.2448654	1.021424	0.0571991
cg12623536	2.086724	0.6597432	6.60017	0.2105501
cg03077331	0.3273126	0.0896614	1.194868	0.0909355
cg26552650	0.4222311	0.1916844	0.930066	0.0323627
cg07033624	2.8861746	1.2840221	6.48743	0.0103196
cg26430365	2.1837986	0.2795333	17.0605	0.4564586
cg07479786	0.1894601	0.0661673	0.54249	0.0019391

cg26239297	0.8879134	0.1745001	4.517994	0.8861208
cg10114555	0.0822124	0.0165406	0.408625	0.002259
cg12067764	2.3778917	0.6903193	8.190948	0.1698526
cg26828842	2.3840824	0.4539615	12.52055	0.3045606
cg09743615	0.1323381	0.0312452	0.560513	0.0060328
cg00618323	1.510124	0.3247897	7.021387	0.599096
cg20211778	0.6258738	0.2788029	1.405	0.2560433
cg09759588	0.4536624	0.2009755	1.024053	0.0570738
cg14305028	2.2304423	0.3085855	16.12154	0.42667
cg20146868	539.70658	31.275058	9313.594	1.50E-05
cg02178032	1.7644987	0.5747205	5.417339	0.3210958
cg23884175	0.118893	0.0350638	0.403138	0.0006304
cg25065131	0.5906927	0.2111413	1.652533	0.3158671
cg17479840	1.7484278	0.3173091	9.634139	0.5210899
cg22339902	0.2530083	0.0768694	0.832752	0.023755
cg26413174	3.0302992	1.0558628	8.696881	0.0393014
cg24512005	0.2150173	0.0473175	0.977068	0.046591
cg05584070	0.9874805	0.1171723	8.322086	0.9907571
cg14778235	1.5499926	0.8675575	2.769243	0.1388385
cg03249917	1.24337	0.4882445	3.166383	0.647869
cg07496861	2783.3156	0.3910973	19807975	0.0796833
cg19092105	1.262E+11	2.08E-13	7.66E+34	0.3602799
cg17301053	2.3554283	0.676565	8.20031	0.1782822
cg10950615	0.4879106	0.2281358	1.043487	0.0642829
cg25511730	8.3790113	0.3286637	213.616	0.1982591
cg24216893	0.2482167	0.0853659	0.721735	0.0105043
cg18609783	1.460657	0.7477576	2.853222	0.2673925
cg03105379	6.1154873	0.7672042	48.74737	0.0873116
cg13890969	0.1722497	0.0357953	0.828878	0.0282286
cg27573591	0.4472018	0.1640192	1.219305	0.1158317
cg13200556	2.518173	0.3464567	18.30299	0.3614736
cg05231970	2.8605535	0.7258137	11.27392	0.1330984
cg22912834	1.8988339	0.6989535	5.158526	0.2085553
cg09969545	13.149535	1.8162039	95.20421	0.0107482
cg17960727	0.0058875	2.75E-05	1.261646	0.0607799
cg10518475	0.7064741	0.3089347	1.61557	0.4103181
cg04621944	0.6346609	0.3171629	1.269993	0.1989168
cg11378052	2.0220742	0.5369086	7.615419	0.2980023
cg16304527	0.0040967	5.26E-06	3.189542	0.1055561
cg14944923	0.5146622	0.2113802	1.253084	0.1434545
cg13659268	1.7683212	0.0008472	3690.8	0.8837893
cg21145248	0.5153734	0.1594398	1.665894	0.2681362
cg23688411	8.55E-07	1.68E-16	4342.997	0.2204424
cg03617054	0.2089234	0.059738	0.730674	0.0142385
cg00163372	0.6942262	0.3969934	1.214	0.2005832
cg11699257	0.1297616	0.0125561	1.341031	0.0865824
cg01104717	0.5789716	0.1957737	1.712223	0.3232244
cg14499053	2.1410998	1.0029509	4.57082	0.0491165
cg22797031	1.6369115	0.5799602	4.620109	0.3519133
cg26191586	1.2544842	0.2286811	6.88177	0.7940425
cg23287485	2.0602516	0.0523416	81.09495	0.6996945
cg16435779	1.8685001	0.8914357	3.916483	0.0978017
cg14986362	2.4880309	1.1587955	5.342011	0.0193872
cg25090302	0.4144962	0.1831549	0.938043	0.0345621
cg11754402	0.0502434	0.0022991	1.097998	0.0573601
cg15658306	1.7197716	0.5441897	5.434896	0.3557234
cg00358611	0.0069494	5.98E-05	0.80817	0.0405862
cg13313916	2.05E-11	4.40E-20	0.009597	0.0156854

cg11310639	0.5812826	0.2144728	1.575442	0.2862168
cg10234477	0.2632654	0.1121592	0.617949	0.0021719
cg25512439	0.5769864	0.2114517	1.574418	0.2829339
cg14691529	2.9953246	1.2480822	7.188605	0.0140452
cg14252617	2.9336721	0.6909809	12.45538	0.1445926
cg07095997	1.6498658	0.6218076	4.377652	0.3145789
cg14789828	0.2181862	0.0799293	0.595591	0.0029648
cg02608453	0.4232634	0.2115164	0.846988	0.0151332
cg19138960	4.2096404	1.4627084	12.11525	0.0076973
cg18763911	0.2566163	0.1049359	0.627544	0.0028712
cg06816106	0.2977822	0.08792	1.008579	0.0516259
cg07589899	2.5719671	0.8178734	8.088067	0.1060868
cg04863758	0.1654982	0.0532741	0.514127	0.0018689
cg25251204	2.5946461	1.129653	5.959519	0.0246202
cg02926397	2.3112142	0.8329229	6.413212	0.107642
cg14021871	0.8593294	0.3101081	2.381257	0.7706462
cg20238128	1.045197	0.5922405	1.844583	0.8787736
cg24909396	48.740674	0.0216034	109966.6	0.3238721
cg03243506	12.035748	0.5519942	262.4289	0.113629
cg12290660	1.6425827	0.8370425	3.223346	0.1490739
cg27131891	1.4500811	0.7729706	2.72033	0.2469782
cg06626184	2.7725436	1.165192	6.597194	0.0211313
cg09311052	443.05393	9.54E-05	2.06E+09	0.4365592
cg22495636	0.2498367	0.0940424	0.663726	0.0053994
cg13914531	3.374475	1.0254991	11.10394	0.0453495
cg05922993	0.9408594	0.5149387	1.719071	0.8428648
cg24675735	4.1577841	0.8096744	21.35076	0.0878127
cg15446115	4.1259766	1.0222317	16.65345	0.0464974
cg24690236	2.0417899	0.5501153	7.57824	0.2860568
cg27430977	0.2535273	0.0583814	1.100969	0.0670142
cg04092033	0.6340428	0.0836205	4.807555	0.6593396
cg00250811	4.94E-17	2.27E-30	0.001076	0.0165704
cg14158558	0.4366321	0.179848	1.060048	0.0670847
cg00292071	0.2994895	0.1008427	0.889444	0.0299374
cg11518184	0.1315492	0.0139191	1.243274	0.0767343
cg16139770	1.3060011	0.2848702	5.987425	0.7311202
cg10355997	1.15E+26	64.943309	2.03E+50	0.0351631
cg01965939	0.103246	0.0136924	0.778516	0.027605
cg06717565	1.9555747	0.8183546	4.673124	0.1313102
cg19769827	1.5858733	0.8348135	3.012642	0.1589835
cg00252594	0.4031657	0.1429792	1.136827	0.0858874
cg26791148	0.4595757	0.2043622	1.033508	0.0600734
cg20071744	0.6899358	0.3148606	1.511816	0.35376
cg23301378	3.1749851	1.0037877	10.04249	0.0492526
cg22676075	1.0927546	0.5358668	2.228376	0.8072475
cg14836602	1.3107832	0.6461017	2.659261	0.4533846
cg09892671	0.8076456	0.4058464	1.607237	0.5428825
cg00825309	498.66523	1.86E-09	1.33E+14	0.6435694
cg15818800	6.7736282	0.4690023	97.82903	0.1602582
cg09615521	62.857406	0.0230503	171409.9	0.3049318
cg06130354	1.4575903	0.5699148	3.727872	0.4316251
cg23678154	1.376104	0.317393	5.966302	0.6696887
cg06776898	1.815177	0.8635539	3.815474	0.1157363
cg26791489	9.3934258	1.4855098	59.39809	0.0172867
cg20414015	0.7557993	0.1235023	4.62528	0.7619487
cg12508392	0.4769802	0.2184052	1.041688	0.0632424
cg10221596	0.3317239	0.146816	0.749515	0.007972
cg12468255	0.4290751	0.239579	0.768454	0.0044304

cg13412834	1.1627275	0.5885915	2.296899	0.6642485
cg10884953	0.1173481	0.0291628	0.472196	0.0025588
cg05389183	0.7645015	0.1328974	4.397848	0.7635592
cg13381015	1.9686492	0.8210637	4.720193	0.1289906
cg24867180	0.8319492	2.24E-11	3.09E+10	0.988179
cg15446945	3.6148405	0.7408411	17.63816	0.1120525
cg12433559	2.5835877	0.9670908	6.902067	0.0583287
cg19801141	9.795E+12	0.4614926	2.08E+26	0.0560595
cg00858483	0.4490915	0.2112036	0.954923	0.0375441
cg15337006	4.4344905	0.8283148	23.74062	0.0818729
cg01283887	0.3223993	0.1103494	0.941929	0.0385151
cg19554948	1.9696992	0.6996718	5.54505	0.1992602
cg24700219	0.266339	0.1117179	0.63496	0.0028394
cg24088438	1.224927	0.5133502	2.922851	0.6475083
cg04467549	6.8268696	0.3864641	120.5963	0.1898361
cg26821634	1.655448	0.4172335	6.568284	0.4734607
cg11174847	3.7599435	0.6880894	20.54555	0.126385
cg00142482	0.8046927	0.4070393	1.59083	0.5320473
cg16170237	0.05877	0.0069374	0.497866	0.0093305
cg14613594	0.3481299	0.1361933	0.889871	0.0275499
cg03833601	0.9464003	0.4112342	2.178013	0.8969286
cg15705428	1.8762875	0.4643681	7.581173	0.3770821
cg15713103	0.908385	0.3823725	2.158009	0.8277016
cg24930443	1.769E+09	7.19E-30	4.35E+47	0.6368419
cg01234945	0.331777	0.1529085	0.719882	0.0052454
cg07224114	2.9373033	0.6207173	13.89965	0.1742578
cg04109768	0.2020367	0.0528819	0.771886	0.0193581
cg03626734	0.9561044	0.4620685	1.978355	0.903698
cg09724427	712561366	12.447188	4.08E+16	0.0253106
cg18445760	20.687348	1.5019226	284.9457	0.0235791
cg17129400	0.061852	0.0051927	0.736744	0.0276895
cg02827340	2.6195596	0.8492539	8.080142	0.0938068
cg22903735	5.08E-06	5.22E-12	4.943637	0.0831303
cg11554391	0.0873904	0.0169213	0.451329	0.0036179
cg19255241	0.8933142	0.3885584	2.053771	0.7905412
cg10743291	0.4680827	0.2229935	0.982546	0.0448023
cg23462788	0.8285985	0.4023873	1.706256	0.6099274
cg06727067	0.6388382	0.3340411	1.221749	0.1755634
cg13470644	94.188976	2.45E-44	3.61E+47	0.9323604
cg04426653	1.3808378	0.6362706	2.996701	0.4143473
cg19785918	2.0237228	0.2314577	17.69418	0.5239899
cg23190164	0.6680044	0.3456269	1.291074	0.2301121
cg03988540	3.9239308	1.1673859	13.1895	0.027093
cg04614923	1.2281126	0.5356313	2.815856	0.6274341
cg21857668	0.9446594	0.1862069	4.79242	0.9452207
cg01708273	1.5141601	0.5182491	4.423897	0.4482193
cg16444001	3.0171429	0.4947936	18.39788	0.2312371
cg09045305	4.1228717	1.3894619	12.23356	0.0106896
cg14765206	0.5722116	0.2630346	1.244803	0.1592025
cg00275103	1.8818646	0.8021921	4.414671	0.1461316
cg17130745	0.9192165	0.4626362	1.8264	0.809974
cg16608407	1.7603569	0.5327152	5.817098	0.3537689
cg00557536	0.9052907	0.4169548	1.965564	0.8013954
cg07586435	0.2625703	0.1180853	0.583842	0.0010388
cg26343512	2.19E-14	2.80E-30	170.8253	0.0920602
cg16209310	0.9563568	0.5029131	1.818641	0.8917563
cg11242708	0.0291239	0.0003836	2.211088	0.1094282
cg08542715	2.5951494	0.7038302	9.568786	0.1520243

cg08545365	3.4169282	1.0393519	11.23334	0.0430188
cg25203007	0.2545639	0.0736409	0.879983	0.0306188
cg01786362	3.1046419	0.5419907	17.78407	0.2033151
cg01993685	0.0006982	2.55E-08	19.14168	0.1633768
cg10632214	0.0987873	0.0189021	0.516287	0.0060789
cg07835283	0.0839348	0.0199646	0.352876	0.0007207
cg25330361	2.4438695	0.0173367	344.5012	0.7233971
cg23764129	0.6914881	0.2381178	2.008064	0.4976252
cg06477056	2.0385889	0.4645332	8.946281	0.345224
cg05115468	74.744114	0.0011233	4973653	0.4464385
cg06689372	0.214291	0.0689656	0.665848	0.0077436
cg04273431	0.5353209	0.2391208	1.198426	0.1285749
cg06206831	1.0301844	0.2491889	4.258937	0.9672428
cg09178025	3.5967095	0.8500898	15.21759	0.0819858
cg16615829	0.5420482	0.273141	1.075695	0.0798943
cg00846400	3.17E-24	5.82E-51	1723.611	0.0849419
cg19483330	1.6737618	0.3603845	7.773582	0.5109302
cg20872981	0.2371151	0.0511039	1.10018	0.0660576
cg00499139	0.5648189	0.2729497	1.168788	0.1236561
cg00042837	1.6876474	0.7585966	3.754504	0.1995774
cg00920938	0.2008967	0.0675263	0.597685	0.0039115
cg18128887	2.1390847	0.8513822	5.374418	0.1057339
cg01776592	1.4945286	0.293303	7.615386	0.6286436
cg24971209	0.1231611	0.0237098	0.639762	0.0127276
cg12637448	1.2057946	0.5274047	2.756783	0.6573659
cg22531801	2.1300006	0.8513734	5.328922	0.1060808
cg12500876	0.5404325	0.1903805	1.534124	0.2476708
cg08343899	2.3285134	0.9424837	5.752857	0.0670112
cg11990980	0.0390264	0.0018052	0.843712	0.0386086
cg26315261	0.4364132	0.2037419	0.934793	0.0328867
cg22125370	1.38892	0.6338965	3.043239	0.411711
cg05980782	0.2232104	0.0493487	1.009609	0.0514695
cg18661379	1.0059436	0.4441918	2.278121	0.9886632
cg17372996	1.4198183	0.7855063	2.56635	0.2458044
cg10157098	3.7641119	0.973442	14.55509	0.0547373
cg09576124	1.5353313	0.5837053	4.038412	0.3848965
cg08035082	5.3413123	1.0705279	26.65005	0.0410457
cg16213567	0.6271487	0.2569814	1.530521	0.3053743
cg12881854	1.5611696	0.6803268	3.58247	0.2932263
cg07277549	0.2608662	0.1156408	0.58847	0.0012062
cg06404334	0.683954	0.218566	2.140283	0.5139954
cg16961070	0.0376638	0.0003386	4.189782	0.1725631
cg19356825	0.2426835	0.0605708	0.972338	0.0455459
cg19778201	9.729E+18	137943.99	6.86E+32	0.0072013
cg08641278	1.6415226	0.5867572	4.592354	0.3450464
cg19354255	0.024519	1.96E-09	307370.5	0.6565401
cg00353006	0.0384594	0.0015188	0.973883	0.0481532
cg10774636	1.706E+17	20434.838	1.42E+30	0.0089551
cg26149678	1.1532277	0.4305569	3.08887	0.7767111
cg21272996	0.3779123	0.1168633	1.222092	0.1041563
cg07713411	0.0547839	0.0080791	0.371487	0.0029402
cg02651190	4.4630633	1.0861767	18.33858	0.0380224
cg19039673	2.0879513	0.3692112	11.80771	0.4049547
cg08076266	1.3452986	0.5797365	3.121812	0.4898086
cg18876157	0.602343	0.2702468	1.34254	0.2151084
cg26829071	1.4388117	0.6043283	3.425587	0.411063
cg23505823	0.4222182	0.2111212	0.844388	0.0147574
cg16688483	1.9570764	0.8339389	4.59284	0.1228962

cg20809036	0.3756894	0.1583052	0.891585	0.0264045
cg07799277	0.2070835	0.0607461	0.705948	0.0118543
cg17771031	1.0754749	0.5636008	2.052244	0.8253241
cg00792185	0.4904954	0.2333716	1.030913	0.0601584
cg23416002	0.3936794	0.162213	0.955432	0.0393271
cg08569180	0.3759353	0.1429824	0.988425	0.0473039
cg08802053	2.7947555	0.8300224	9.410178	0.0970763
cg02763409	3.1288013	1.2909211	7.583266	0.01156
cg11740416	0.4854071	0.1992059	1.182797	0.1117169
cg07211707	1.6065052	0.5970238	4.322874	0.3479046
cg05684195	0.8134354	0.1226263	5.395885	0.8306313
cg05793013	2.5957457	0.9116379	7.390978	0.0739891
cg00293660	0.5871888	0.2735443	1.260456	0.171923
cg19634527	0.0036277	7.47E-05	0.17623	0.004566
cg23117727	1.5374213	0.7251076	3.259743	0.2619963
cg15663484	0.0138378	0.0002813	0.680792	0.0312872
cg09041678	3.256804	0.995016	10.6599	0.0509732
cg08036796	0.1383489	0.0423598	0.451853	0.0010549
cg20053158	0.0039266	0.0001306	0.118042	0.0014202
cg15035476	1.5146218	0.4790728	4.788581	0.479618
cg26919953	3.9082857	0.7948988	19.2159	0.0934482
cg15244049	1.2539781	0.6135276	2.562983	0.534914
cg25038060	27813604	755.41673	1.02E+12	0.0013963
cg01329057	0.6953111	0.3062192	1.578795	0.3851056
cg18094907	2.3777308	1.0137305	5.577029	0.0464461
cg21602614	1.3122773	0.5862038	2.937667	0.5086285
cg03517226	8.4336466	1.3516481	52.62198	0.0224581
cg15352829	1.8371736	0.2291236	14.73094	0.5668793
cg14052114	207.15973	0.9508541	45133.27	0.0521833
cg15728909	0.4036816	0.1827768	0.891573	0.024842
cg08404749	1.0349152	0.3038108	3.525383	0.9562339
cg08827060	0.16202	0.0265446	0.988921	0.0486056
cg26283874	0.5640217	0.2978836	1.067936	0.0787187
cg12738347	1.0209359	0.5295037	1.968466	0.9506783
cg04481603	0.2713617	0.085224	0.864042	0.0272955
cg07748583	0.1767605	0.0486755	0.64189	0.0084448
cg19895185	0.2402944	0.0997556	0.578828	0.0014784
cg07837534	1.4293251	0.5572681	3.666045	0.4573122
cg06293172	0.4622175	0.2080855	1.026717	0.0580643
cg02064106	0.488764	0.118045	2.023721	0.3233848
cg25119986	0.6443681	0.3124859	1.328733	0.23396
cg08125574	0.2402923	0.0997349	0.578938	0.001482
cg09368875	0.0353652	0.0030746	0.406789	0.0073247
cg15957055	0.0371122	0.0022723	0.606129	0.0208173
cg20504993	1.1011928	0.3478627	3.485931	0.869769
cg11958644	1.4624642	0.5704454	3.749353	0.4287385
cg16005592	2.8811071	0.6938971	11.96255	0.1451571
cg06769372	3.385514	0.9358331	12.2476	0.0630447
cg19190699	4.5214764	0.9123933	22.40673	0.0646473
cg21029108	0.2273497	0.079227	0.652402	0.0058865
cg08193551	5.5100979	0.7896897	38.44697	0.0851148
cg00340958	0.5579602	0.2410883	1.291309	0.1729392
cg05064489	0.2861775	0.1166621	0.702007	0.0062805
cg11721591	0.4411872	0.1750259	1.112099	0.0827901
cg27651070	0.2041123	0.0682934	0.610042	0.0044452
cg20996351	3.2011941	0.9794593	10.46255	0.0541524
cg15130211	2.8525702	1.2492617	6.513572	0.0128371
cg04510153	0.1059575	0.0090691	1.237938	0.0734898

cg01992487	0.3621779	0.1308127	1.002753	0.0506217
cg01392184	1.514E+12	2241.1855	1.02E+21	0.0068573
cg04224340	0.3950837	0.1095269	1.425139	0.1559759
cg05700339	1.3660421	0.7083968	2.634217	0.3518623
cg21582373	4.2832646	1.1646069	15.75326	0.0285753
cg06728252	0.435163	0.1928695	0.981839	0.0450582
cg23560320	1.7143984	0.7157218	4.10657	0.2264651
cg06721712	3.7413521	0.0140204	998.3835	0.6434374
cg21062760	2.5225057	0.6908169	9.210885	0.1614496
cg26719831	0.1828117	0.0416129	0.803119	0.0244292
cg10261191	1.5436493	0.7719315	3.086871	0.2195
cg24053748	0.5005241	0.2355645	1.063507	0.0718854
cg26593267	0.1654096	0.0274642	0.996218	0.0495185
cg20577663	0.2667501	0.0571255	1.245602	0.0928316
cg03595855	1.5965771	0.8402318	3.033756	0.153156
cg18345924	0.3124838	0.1231599	0.79284	0.0143405
cg07010948	4.5609912	1.6215117	12.82916	0.0040273
cg12570309	0.4080109	0.0817923	2.035312	0.2742681
cg21967790	0.6582862	0.302632	1.431906	0.2916446
cg19322788	1.8806628	0.8468837	4.176361	0.1207372
cg02769951	0.7350095	0.3389187	1.594008	0.4356938
cg13231680	0.5806938	0.2798348	1.205016	0.1444908
cg27548631	5.42E-15	1.74E-51	1.68E+22	0.4435471
cg01308968	2.7881042	0.4168929	18.64634	0.2902551
cg12573935	0.3448305	0.0971555	1.223895	0.0994841
cg17128312	0.2689217	0.0834278	0.866844	0.02786
cg19682048	1.8552263	0.7508455	4.583985	0.1805483
cg01308202	0.8927227	0.3531386	2.256774	0.8104678
cg07707687	0.095735	0.0037079	2.471785	0.157241
cg19697042	0.0652252	0.0059463	0.715451	0.0254847
cg00587042	0.3493182	0.179978	0.677989	0.00188
cg13741668	2.2806606	1.1215376	4.637752	0.0228041
cg03285577	1.3205049	0.5756271	3.029276	0.5116577
cg25457027	6.078639	0.8648556	42.72373	0.0696735
cg10487998	0.2240612	0.0547292	0.917306	0.0375269
cg23322851	0.5013682	0.2258349	1.11307	0.0897517
cg08198280	6.164405	1.1807254	32.18351	0.0310069
cg09106624	0.0350426	0.0002473	4.965929	0.1848741
cg21797001	2.4725581	1.2045898	5.075208	0.013614
cg07084930	0.1956739	0.0521047	0.734834	0.015677
cg06473276	0.2825062	0.0978621	0.815533	0.0194416
cg02227605	0.203667	0.0097372	4.259956	0.3050081
cg22562853	1.067264	0.1554664	7.32668	0.9471934
cg11958234	0.5166152	0.2146072	1.243627	0.1406105
cg14458815	3.5756902	1.0356725	12.34518	0.0438621
cg16051651	6.61E-06	6.92E-10	0.063074	0.0107415
cg23844090	0.1994259	0.0547265	0.726718	0.0145332
cg26840757	0.0079315	6.10E-06	10.31117	0.1861101
cg16649052	1.0380801	0.3759558	2.866322	0.942506
cg02587405	1.6015995	0.0920281	27.87325	0.746577
cg23202177	2.5842949	1.1478193	5.818495	0.0218537
cg19811761	0.1492749	0.0394597	0.564702	0.0050822
cg13751548	1.4939054	0.7027687	3.175658	0.2968446
cg13060531	0.2102634	0.0613979	0.720068	0.0130334
cg04728402	0.3717822	0.1770249	0.780806	0.0089614
cg25604616	0.3840544	0.1666714	0.884961	0.0246459
cg12924825	1.0890711	0.4584031	2.587408	0.8467551
cg14826215	0.7566272	0.314085	1.822706	0.5341385

cg02921627	0.4643126	0.0446542	4.827907	0.5207719
cg23416994	1.2595505	0.6024776	2.633239	0.5396894
cg13761843	0.1036419	0.0267108	0.402146	0.0010501
cg05404091	2.0924072	0.7059456	6.201848	0.1829176
cg24670442	33.911554	0.7876291	1460.07	0.0664161
cg12143125	2.692E+12	4290.2803	1.69E+21	0.0056189
cg00845891	9.794E+18	1.26E-14	7.63E+51	0.2577827
cg20589096	0.5536146	0.2610576	1.174029	0.1231589
cg14195216	0.8981533	0.4031518	2.000932	0.7926868
cg06035600	2.711518	0.2273531	32.33881	0.4302677
cg11251877	3.2195493	0.6227964	16.64348	0.1630164
cg06676006	1.0056399	0.0120934	83.62489	0.9980105
cg00054741	0.5032026	0.2501921	1.012073	0.0540667
cg05952643	279.96822	0.2912373	269135.2	0.1078496
cg14227911	2.8984018	1.2894172	6.51514	0.0100224
cg26536354	0.0168637	0.0022112	0.128611	8.20E-05
cg17696194	0.2532795	0.0997045	0.643406	0.0038888
cg04220005	0.0631643	0.0025665	1.554548	0.0910254
cg20701183	1.6539785	0.7544658	3.625936	0.2089539
cg24935135	0.250511	0.0821026	0.764358	0.0150117
cg11468000	0.781319	0.2937274	2.07832	0.6210406
cg25840926	0.2103156	0.0613623	0.720844	0.0131092
cg11241541	0.9639793	0.4852726	1.914916	0.9165672
cg27365342	2.3700534	1.0156143	5.530794	0.0459558
cg23818062	0.1097374	0.0074183	1.623321	0.1079419
cg07082452	2.86986	0.6062059	13.5863	0.18385
cg08548070	1.0143984	0.3857498	2.667543	0.976881
cg07815491	0.2029695	0.0147506	2.792874	0.233201
cg00487187	1.7899978	0.6922257	4.628681	0.2297103
cg06012483	0.5657659	0.1788154	1.790064	0.332447
cg24065807	1.8568087	0.4763082	7.238462	0.3726563
cg24417237	8.08E-13	7.48E-27	87.32402	0.0912457
cg02586212	0.7200997	0.4333122	1.196697	0.2051299
cg10091408	1.7442953	0.7909922	3.846518	0.1679378
cg27315341	0.3664046	0.1525947	0.879797	0.0246713
cg14633800	4.9570644	1.2264393	20.03563	0.0246787
cg01761256	1.2177622	0.4842476	3.062369	0.6754133
cg12834596	4.2153623	0.9407999	18.88742	0.060079
cg11691189	0.2274752	0.0854289	0.605707	0.0030434
cg13703070	11896.751	1.7264629	81978409	0.0374279
cg12583076	0.4677988	0.2394342	0.91397	0.0262014
cg18712727	6.235E+14	1.28E-19	3.04E+48	0.3893655
cg16296417	1.0124821	0.4400491	2.329558	0.9767228
cg13632630	0.1494001	0.0513503	0.434669	0.0004848
cg01777861	3.7533967	1.4498561	9.716817	0.0064228
cg16712637	0.1501142	0.0252536	0.89232	0.0370473
cg04941246	2.8005792	1.3519874	5.80127	0.0055781
cg11398794	0.8206882	0.3964896	1.698731	0.5944537
cg06679087	0.0458273	0.0044868	0.46807	0.0093153
cg16323491	1.28E-05	3.90E-09	0.041726	0.0063479
cg12988946	1.267179	0.1168235	13.74503	0.8456397
cg07479615	0.3688955	0.1484871	0.91647	0.0317273
cg02240252	0.8692636	0.1644776	4.594054	0.8689891
cg00292029	1.1377564	0.505696	2.559818	0.7550821
cg00841692	0.4805689	0.2109152	1.094973	0.0811545
cg21083936	0.4639437	0.2066968	1.04135	0.0626396
cg08967456	1.959536	0.2748605	13.96993	0.5020567
cg22595391	2.3564314	0.8603574	6.454026	0.0954386

cg07464977	0.270553	0.1004114	0.72899	0.0097378
cg11415047	0.4144863	0.1999784	0.859087	0.0178647
cg25371919	2.1697688	0.8274486	5.689655	0.1152845
cg24671666	3.5956511	0.3975038	32.52474	0.2547367
cg13475860	0.2857453	0.0971294	0.840635	0.0228891
cg12530103	164.82071	0.1817685	149453.1	0.1417681
cg27279968	0.002566	1.67E-06	3.93898	0.111001
cg09781827	1.776746	0.8643272	3.652351	0.1179622
cg13877670	1.4803313	0.8579269	2.554275	0.1587191
cg02023912	0.1573317	0.0448849	0.551482	0.0038528
cg02466588	0.9116082	0.4039855	2.057078	0.8236307
cg21210531	0.3092262	0.1468663	0.651074	0.0020041
cg02799466	2.5631745	1.1505634	5.710127	0.0212705
cg15425541	2.7393904	1.0914498	6.875497	0.0318457
cg26391674	1.9419912	0.700888	5.380788	0.2017971
cg09495769	1.6339129	0.66007	4.044528	0.2883782
cg18833788	1.8728413	1.0043393	3.49238	0.0484292
cg08118942	0.2914517	0.1308529	0.649157	0.0025488
cg15885043	1.9393663	0.5667448	6.636394	0.2913006
cg25307468	3.4594554	1.0410722	11.49568	0.0427997
cg08743199	0.1903835	0.0661513	0.547924	0.002102
cg01220257	0.3425458	0.1606804	0.730255	0.0055389
cg12535280	0.1196966	0.0044317	3.2329	0.2068582
cg08452338	0.6239529	0.2623523	1.483948	0.2859499
cg03056526	2.0300017	0.8883548	4.638807	0.0931129
cg12401343	3.935E+18	5.41E-17	2.86E+53	0.2958232
cg21920167	0.5454947	0.2721699	1.093304	0.0875443
cg14494607	0.5008622	0.2256857	1.111559	0.0891434
cg03166753	1.0710111	0.5543842	2.069079	0.8382045
cg19645256	1.7267487	0.5855824	5.091787	0.3221576
cg22044408	20.347207	1.0079802	410.7311	0.0493971
cg05340882	0.0024023	1.16E-05	0.498022	0.0266843
cg09837648	0.2576048	0.1010115	0.656958	0.0045179
cg05158606	0.2439223	0.0465761	1.277437	0.0948952
cg00356645	1.4018752	0.5828274	3.371932	0.4506234
cg09435266	0.667472	0.1484344	3.001454	0.5981645
cg14399060	2.8526674	1.1383322	7.148802	0.0253269
cg05661533	2.861098	0.4040777	20.25819	0.292521
cg02102889	0.5978945	0.2765806	1.292491	0.1909892
cg13672514	0.2534544	0.0749849	0.856694	0.0271829
cg18750167	1.2904047	0.6808887	2.445545	0.4344333
cg26478215	11.266919	0.5143916	246.7837	0.1240862
cg14947547	0.0310918	0.0018928	0.510728	0.0150787
cg12377256	0.4497873	0.2306979	0.876942	0.0190044
cg01808641	4.073561	0.6119611	27.11594	0.1464449
cg27097386	0.4222839	0.1914631	0.931374	0.0326689
cg14358282	1.733474	0.848989	3.539424	0.130923
cg22375757	0.3480001	0.1475538	0.820745	0.0158995
cg00516030	0.3910752	0.1770889	0.863633	0.0201974
cg02455203	1.6432827	0.6524954	4.13854	0.2918918
cg11350520	0.0539792	7.42E-10	3924973	0.7519521
cg23618156	0.5785822	0.242603	1.379857	0.2172432
cg12026992	0.4164531	0.200364	0.86559	0.0189435
cg06935361	2.8540636	0.4513465	18.04751	0.2650489
cg05083852	0.436642	0.153363	1.24317	0.1206071
cg24449885	412.95356	2.0979072	81286.08	0.0254252
cg01782132	3.40E-07	1.79E-12	0.064657	0.0163251
cg08492875	1.4939293	0.122015	18.29139	0.7534689

cg09722785	1.41E+10	0.3494361	5.69E+20	0.0607133
cg07086381	1.1441099	0.174711	7.492301	0.8883366
cg19449948	1.5060766	0.5369021	4.22473	0.4364806
cg16072535	0.262107	0.0666512	1.030741	0.0552856
cg01345354	3.4598514	0.7008582	17.07988	0.1275988
cg01414464	1.6806786	0.7808264	3.617553	0.1843662
cg23418066	6.3511147	1.0045937	40.15221	0.0494333
cg07058998	0.310915	0.1361207	0.710165	0.0055694
cg14265220	92.528189	0.0558134	153394.4	0.2313014
cg25636820	1.4848089	0.501489	4.396223	0.475382
cg00412851	0.2969513	0.1471854	0.599109	0.0006975
cg04094210	0.1101006	0.0222718	0.544283	0.0068099
cg18186763	6.2385343	1.746853	22.27967	0.0048196
cg03374231	3.7345219	1.2040351	11.58326	0.0225208
cg04833514	0.2470776	0.0825638	0.739396	0.0124256
cg02231066	0.0018998	1.59E-05	0.227502	0.0102768
cg23902610	2.2921097	1.0828404	4.851838	0.0301601
cg12006375	1.9604911	0.5217114	7.367149	0.3189208
cg04380561	0.255484	0.0003309	197.2706	0.687508
cg21029045	1.3257376	0.6919695	2.539968	0.3953301
cg24538029	0.2940144	0.0640204	1.350265	0.1155185
cg06820975	1.0177598	0.4644354	2.23031	0.9649212
cg20284891	9.1294686	0.494486	168.5532	0.1371268
cg13312174	0.2909791	0.0757508	1.117729	0.072197
cg09407650	0.4455389	0.2039981	0.973072	0.0425141
cg01371799	1.5330216	0.6288053	3.737493	0.3474061
cg01252713	1.0981643	0.5131393	2.35017	0.8093854
cg13870539	0.9211208	0.3203119	2.648867	0.8788273
cg01562349	1.8648384	0.8811456	3.946706	0.1032771
cg17050756	1.0957693	0.3515506	3.415469	0.8747146
cg13861904	1.4955844	0.6672081	3.352437	0.3283769
cg20464143	0.3740283	0.1645283	0.850292	0.018925
cg14889079	0.016608	0.0003591	0.768147	0.0361885
cg23047335	0.0003652	3.90E-08	3.420379	0.0898102
cg13096260	1.0518427	0.3266441	3.387091	0.932491
cg09077672	487.19968	4.24E-06	5.6E+10	0.5133929
cg17384865	2.0856437	0.5987077	7.265498	0.2483474
cg22338356	0.2761263	0.1297676	0.587556	0.000837
cg00981651	0.0848109	0.0194521	0.369774	0.0010227
cg16155724	27.797125	0.0783859	9857.389	0.2670072
cg11297382	1.165E+18	2.12E-18	6.40E+53	0.3218028
cg03071124	0.7955164	0.3898008	1.623512	0.5296537
cg20257866	0.0952983	0.0250969	0.361868	0.0005542
cg22095490	3.755523	1.2885082	10.94596	0.0153339
cg04686778	0.8114678	0.3788823	1.737954	0.5908434
cg05825420	0.3162057	0.0615867	1.623501	0.1677709
cg09872737	0.0248871	0.0024034	0.2577	0.0019553
cg26907697	0.1643318	0.054223	0.498035	0.001412
cg22923409	1.2532215	0.6551451	2.397277	0.4951981
cg04672750	0.4218525	0.2068188	0.860461	0.0176349
cg23661344	10.153186	1.4496073	71.11387	0.0196048
cg17382918	3.0353622	0.5958015	15.46392	0.1813563
cg20417071	2.000455	0.7100172	5.63623	0.1895311
cg19628299	0.9437276	0.4500142	1.979097	0.8781733
cg23749482	4.4257876	0.8533365	22.95413	0.0765414
cg14935206	1.1702933	0.466114	2.938308	0.7377735
cg01928078	0.1067642	0.0281939	0.404293	0.0009912
cg18564464	2.3529101	0.8522795	6.49574	0.0986443

cg14109388	4.0707149	1.4830812	11.17317	0.00643
cg16186435	3.4232495	1.4085056	8.319908	0.006609
cg15046388	0.9245484	0.4625039	1.848178	0.8243244
cg13005202	0.8528079	0.4213718	1.725985	0.6580283
cg23898059	0.7804183	0.069285	8.790543	0.8409625
cg13515584	2.4313498	0.8473216	6.976645	0.0985511
cg04791718	0.3317495	0.1559844	0.705569	0.00416
cg04089246	2.8015174	0.4121552	19.04258	0.292105
cg18587063	1.1501551	0.6034243	2.19225	0.6707753
cg06045576	0.149862	0.0255469	0.879114	0.0354923
cg12678695	0.1983093	0.0665302	0.591109	0.0036906
cg00350519	17865.095	0.0006129	5.21E+11	0.2642327
cg03127334	0.8108545	0.3631562	1.810474	0.6089366
cg13862920	1.7378482	0.457284	6.604466	0.4171916
cg11525980	0.3795434	0.1656669	0.869535	0.0219934
cg07581492	0.2944865	0.0834061	1.03976	0.0575136
cg21516443	3.3627475	0.8880496	12.7336	0.0742296
cg06581175	2.6828396	1.1796009	6.101749	0.018575
cg21387009	0.9381812	0.1709252	5.149526	0.9414456
cg26956157	1.5795957	0.8103562	3.079044	0.1794424
cg04731861	2.3866261	0.8559702	6.654419	0.0963722
cg23096689	1.1207969	0.5768954	2.177493	0.7364564
cg10192198	1.9704216	0.9909797	3.917902	0.0530978
cg25922969	0.342032	0.1601446	0.730502	0.0055876
cg03263730	2.3298912	0.7308788	7.427213	0.1527321
cg12881453	2.4986827	0.3698307	16.88182	0.3474811
cg11472725	2.444987	0.3630832	16.46444	0.3582045
cg01498231	1.7902235	0.8201897	3.907511	0.1436754
cg03521214	3.756293	0.6199023	22.76123	0.1499398
cg12615235	1.2077944	0.5245874	2.780789	0.657247
cg20777315	4.1155026	0.822323	20.59697	0.0850919
cg17554126	1.5848455	0.7368972	3.408529	0.2385714
cg03490289	2.9307667	0.1575658	54.51304	0.4709371
cg27434487	0.4097497	0.1591056	1.055242	0.0645215
cg02359181	0.001587	2.02E-05	0.124899	0.003805
cg09500565	0.1414263	0.0351724	0.568668	0.005869
cg18385570	0.591596	0.2730764	1.281641	0.1832371
cg08852989	3.65E-13	7.45E-25	0.178505	0.0370299
cg16061012	0.1728811	0.0230963	1.294055	0.0874572
cg12290311	0.4834274	0.2472236	0.945306	0.0336407
cg27106950	0.3205243	0.1282974	0.800763	0.0148676
cg20710266	2.0149891	0.8271034	4.908916	0.1230408
cg08525429	0.2888547	0.1137408	0.733572	0.0090139
cg18186394	1.4698448	0.6475969	3.336094	0.3570494
cg08381422	7.57E-16	2.70E-32	21.24906	0.0715769
cg05940672	1.8621531	0.8304601	4.175533	0.1312847
cg06536868	0.7444511	0.3899678	1.421162	0.3710274
cg15370140	0.2435307	0.0956712	0.619906	0.0030459
cg00538212	3.005419	1.0033819	9.002099	0.0492991
cg09452568	1.9872816	0.9880938	3.996876	0.0540586
cg03635863	0.6002827	0.2574412	1.399696	0.2374006
cg21719626	0.6642766	0.1106145	3.989198	0.6547046
cg19140335	0.3109916	0.1290625	0.749371	0.0092424
cg17287034	0.326287	0.1535496	0.693347	0.0035884
cg03905847	0.1539822	0.0262974	0.90163	0.0380051
cg18315960	0.3427401	0.1087657	1.080035	0.0674762
cg21158476	0.5582024	0.200868	1.551217	0.2635475
cg20595457	1.4786213	0.6328803	3.454557	0.3663443

cg21394442	1945.8775	17.008889	222615.3	0.0017376
cg03607907	0.497203	0.1127044	2.193445	0.3561499
cg14236758	8.6332131	1.0574273	70.48463	0.044211
cg22095604	0.3635612	0.1203265	1.098484	0.0728983
cg01868379	1.6942896	0.5709708	5.027608	0.3420558
cg25984344	0.3278161	0.1238931	0.867388	0.0246698
cg07311694	1.8260382	0.455	7.328386	0.3957156
cg03309770	2.24727	0.6141748	8.222776	0.2211699
cg24722073	2.1961543	1.0673833	4.518615	0.0325892
cg24977027	0.5990099	0.2625493	1.366649	0.2233241
cg11148364	0.2831784	0.0991885	0.808461	0.0184128
cg13799287	4.305E+18	0.0123839	1.50E+39	0.0754053
cg19451167	2.6507905	0.959891	7.3203	0.0599747
cg07039378	0.5505447	0.2646825	1.145144	0.1102071
cg09063663	0.657759	0.223068	1.939529	0.4476824
cg15753394	2.4168887	1.1000961	5.309855	0.0279834
cg02035961	0.7260805	0.2811114	1.875388	0.508515
cg04166294	4.1780071	1.2256789	14.24169	0.0223014
cg14415616	0.9580744	0.2827607	3.246231	0.9451575
cg18377014	1.9799401	0.3935726	9.960458	0.4072832
cg00254133	0.0833354	0.0104807	0.662625	0.0188236
cg10423113	2.74E-06	2.12E-14	354.6272	0.1789806
cg21338523	0.2315508	0.0367672	1.45825	0.119192
cg16051656	0.6664218	0.3234952	1.372874	0.2710883
cg19065177	0.9348474	0.467079	1.871074	0.8490737
cg21823426	1.5868788	0.8160209	3.085931	0.1735751
cg19788317	1.7842687	0.5900129	5.395839	0.3051283
cg23058435	1.9445181	0.9219271	4.101356	0.0807276
cg17516809	0.5642645	0.2823072	1.12783	0.1053377
cg12069042	0.2945191	0.1242832	0.697934	0.0054875
cg27512502	3.2274075	0.1795844	58.00148	0.4266414
cg07281318	0.6628565	0.2887522	1.521647	0.3321241
cg22000330	0.1213764	0.0165437	0.890502	0.0380777
cg22957339	0.1692775	0.062838	0.456011	0.0004431
cg07782630	5.638E+13	1.65E-07	1.93E+34	0.1893417
cg04420991	0.1136671	0.0155724	0.829686	0.032028
cg23685155	1.9521105	0.8295994	4.593464	0.1255013
cg09127044	2.868738	0.9089524	9.054003	0.0723084
cg11747897	2.992345	0.6417249	13.95322	0.1629342
cg09064148	0.8839497	0.47092	1.659235	0.7010229
cg03899643	1.8850221	0.7625749	4.659619	0.1697724
cg23008352	1.6299372	0.8208521	3.236509	0.1627443
cg00996053	0.0144487	1.79E-05	11.6868	0.2148592
cg18125735	4.71E-13	2.57E-48	8.63E+22	0.4932437
cg04021497	1.8269706	0.6248028	5.3422	0.2709611
cg15690696	0.9603587	0.3552401	2.596241	0.9364641
cg13617301	1.6315988	0.3126289	8.515254	0.5614301
cg18213443	0.2703791	0.0866782	0.843405	0.0242352
cg01667953	0.3021048	0.1172505	0.778396	0.0131844
cg26897283	0.067667	0.0160934	0.284516	0.0002375
cg00591949	0.2006147	0.0405947	0.991417	0.0487768
cg08106708	0.3191899	0.0865069	1.177735	0.0864605
cg24468765	15.970988	0.7113039	358.5984	0.0809193
cg18252633	0.4701197	0.1861507	1.187278	0.1103119
cg17183432	1.3513582	0.631597	2.891352	0.4378044
cg08578734	2.1396669	1.2537342	3.651631	0.0052853
cg12887711	1.75E-06	6.54E-13	4.672797	0.0791284
cg16870442	73.278846	1.73E-13	3.11E+16	0.8026787

cg05733285	0.3242903	0.1330517	0.790401	0.0132332
cg04013166	0.2001575	0.077138	0.519368	0.0009443
cg18945299	138.02976	1.3292434	14333.13	0.0375154
cg25335557	2.8280211	1.0904399	7.334383	0.0325143
cg15423035	2.0093125	0.6405102	6.303314	0.2316003
cg26672688	1.2424539	0.46372	3.328931	0.6659463
cg25591336	0.4466085	0.1597163	1.248834	0.1244355
cg24448269	2.1248062	0.689139	6.551366	0.1895549
cg17430781	5.9479084	2.1987376	16.08997	0.0004452
cg16853982	0.309793	0.1199175	0.800315	0.0155222
cg23083984	4.5363945	1.3042845	15.7779	0.0174219
cg24880131	2.9056439	1.2082647	6.987514	0.0171937
cg06840243	0.2936293	0.1524232	0.56565	0.0002491
cg26382251	43.3534	0.4498777	4177.84	0.1058246
cg06824727	3.36E-16	1.74E-30	0.064642	0.0337397
cg26297493	0.4424159	0.1702462	1.149698	0.0941959
cg01076485	2.0086134	0.0186029	216.8767	0.7703101
cg10096873	2.4921159	0.8662666	7.169434	0.0903255
cg10954248	760850.77	3.56E-17	1.63E+28	0.605703
cg09234936	0.3014703	0.1125788	0.807296	0.0170371
cg13482172	0.4600337	0.1966085	1.076408	0.0734213
cg21237861	4.9585411	1.2814722	19.18663	0.0203839
cg23967474	0.0077489	0.0004266	0.140762	0.0010187
cg08097657	4.5180045	1.1204995	18.2172	0.0340147
cg03898044	0.6804662	0.3338168	1.387091	0.2893846
cg16138602	1.0432642	0.2848635	3.820778	0.9490102
cg06088575	1.2090567	0.3675909	3.976752	0.754654
cg13962212	0.9710438	0.4151413	2.271337	0.9459657
cg07567260	5.0436572	0.8725998	29.15251	0.0706503
cg07813851	2.1824662	0.9213054	5.170011	0.0761144
cg00661719	3.8178399	1.2968496	11.23947	0.0150238
cg21096966	0.3608146	0.1193202	1.091074	0.0709842
cg02945866	0.4573598	0.2264673	0.923656	0.0291528
cg18664899	0.0891922	0.0044054	1.805795	0.1152859
cg13057898	5.6333106	0.5997903	52.90881	0.1303639
cg20410537	0.6352349	0.0060016	67.23613	0.8487075
cg13995427	1.5305152	0.7100435	3.299061	0.2774301
cg11429960	30.078576	0.2145667	4216.5	0.1771217
cg05393058	0.1462081	0.0364733	0.586095	0.0066445
cg18256236	2.77E-16	1.80E-44	4.26E+12	0.2793403
cg17235897	1.6566282	0.8206475	3.344209	0.1589982
cg00099295	1.6802054	0.9167364	3.079501	0.093206
cg01616797	2.4026566	0.9009496	6.407416	0.0798524
cg11068337	0.1161499	0.0006729	20.04781	0.4126877
cg24604214	30.075578	0.2024048	4468.967	0.1822329
cg18337735	1.0845069	0.4616198	2.547887	0.8523227
cg15829728	1.6677791	0.7122534	3.905193	0.2386803
cg00860090	1.8052697	0.5242528	6.216464	0.3491006
cg10545738	0.1070545	0.0060503	1.894228	0.1274586
cg21452942	0.1923832	0.0490633	0.754358	0.0180636
cg06219660	0.2825578	0.0923241	0.864768	0.0267912
cg11736929	0.2573551	0.0656021	1.009597	0.0516225
cg15511801	2.3193351	0.7360961	7.307899	0.1507996
cg15331578	0.4936667	0.2620828	0.929885	0.0288905
cg06398114	0.3264124	0.089677	1.188098	0.0894147
cg17031787	0.1122137	0.0373026	0.337562	9.92E-05
cg26782539	5.0806348	0.964236	26.77026	0.0552364
cg07570394	1.4688684	0.5119119	4.214737	0.47466

cg20104243	1.3548998	0.2783382	6.595406	0.7068135
cg18261944	1.6123423	0.5351433	4.857854	0.3959409
cg26453588	0.2092944	0.0780869	0.560967	0.001876
cg18183435	1.0912617	0.2936472	4.055384	0.8962529
cg14450427	0.2472167	0.0818288	0.746878	0.0132368
cg16283183	0.4246777	0.2044104	0.882299	0.0216975
cg11197418	0.510176	0.004615	56.39828	0.7792277
cg10230885	2.394799	0.7456175	7.691695	0.1424044
cg24690709	2.7625702	0.9720861	7.850944	0.0565415
cg03371404	105.11009	0.1081503	102155.4	0.1847543
cg08780063	0.3327101	0.1459463	0.758471	0.0088576
cg00238349	0.0480602	0.0035297	0.654383	0.0227111
cg06325540	1.7968399	0.7985327	4.043208	0.1567004
cg13008315	3.65E-05	2.98E-10	4.461746	0.0873127
cg22891413	1.5815979	0.5617124	4.453261	0.385414
cg12193817	0.6844662	0.2318612	2.020579	0.492446
cg06114087	1.1309517	0.4070366	3.142351	0.8134168
cg21741562	1.1667494	0.6092701	2.234319	0.6417648
cg26338947	0.5247234	0.2309955	1.191948	0.1234353
cg03599575	47.426619	1.5402004	1460.384	0.0273166
cg10384245	2.0451758	1.0494863	3.985516	0.0355659
cg13952988	0.0050616	1.09E-31	2.36E+26	0.8752823
cg06530441	0.5015721	0.2171566	1.158494	0.1062001
cg10736208	0.5619799	0.24538	1.287071	0.1728654
cg14205016	0.4306133	0.1671382	1.109428	0.0810018
cg17387122	0.277013	0.1010045	0.75973	0.0126385
cg19275653	2.1752223	0.572434	8.265743	0.2538929
cg09258878	1.3968818	0.0035006	557.4058	0.9128983
cg13918433	0.2312506	0.0964794	0.554283	0.0010272
cg01564818	0.1694449	0.0338804	0.847439	0.0306551
cg24754223	1.4470597	0.5376815	3.894465	0.4644299
cg22981461	0.1719889	0.0162382	1.821638	0.1437685
cg24800754	0.8586785	0.2447428	3.012668	0.8119508
cg23418219	0.1118947	0.0191035	0.6554	0.0151643
cg21145416	1.4780351	0.5713666	3.823444	0.4204057
cg15245625	2.3708673	0.9657071	5.820618	0.0595899
cg04618842	6258.8598	1.4118914	27745283	0.0413029
cg01723892	0.5485496	0.24617	1.222353	0.1418768
cg25049733	0.3543271	0.1335604	0.940007	0.0371377
cg01891925	0.3567098	0.1504617	0.845676	0.0192554
cg21093170	0.3661222	0.1784683	0.751089	0.0061308
cg08618878	1.3985765	0.3750334	5.215578	0.6174057
cg05672223	0.1904354	0.0575534	0.630122	0.0065989
cg04132809	10.233107	2.0137666	52.0003	0.0050482
cg14509902	0.4114313	0.1706055	0.992205	0.0479979
cg05206657	2.9789177	0.7311945	12.13624	0.1277306
cg13373589	0.3340574	0.1639631	0.680606	0.0025308
cg04704193	1.534318	0.6983951	3.370773	0.2864058
cg05446414	0.1130002	0.0369304	0.34576	0.0001328
cg21881034	0.592755	0.0102817	34.17334	0.8004127
cg17277820	0.5150067	0.2598564	1.020687	0.0572632
cg24535312	2.76E-21	3.57E-35	2.14E-07	0.003719
cg11209289	1.680579	0.5166822	5.466311	0.3883176
cg06066180	0.6112014	0.2525697	1.479066	0.2748812
cg21521989	0.4788483	0.193889	1.182613	0.1104094
cg27231441	1.4757898	0.4714465	4.61973	0.503842
cg04498418	1.7091905	0.9215484	3.170026	0.0889922
cg02421172	0.4510545	0.1420132	1.432614	0.1769307

cg00585116	1.1606533	0.3174299	4.243822	0.8218034
cg18235100	0.9869918	0.5339286	1.8245	0.9666827
cg08723357	7.28E-08	2.22E-19	23883.46	0.2244243
cg23443327	0.4204883	0.1053068	1.679002	0.2200496
cg21699894	1.729713	0.8864952	3.374984	0.1081207
cg00947686	1.5917129	0.6887412	3.678523	0.276809
cg24712290	0.1336089	0.041534	0.429801	0.0007342
cg06940925	0.0714187	0.0089599	0.56927	0.0127053
cg23050873	0.4485715	0.1093054	1.840864	0.2657665
cg00128054	0.4904768	0.2502685	0.961238	0.0379751
cg12537337	2.2165162	0.9230452	5.322539	0.0749448
cg01430604	1.1168435	0.4724306	2.640259	0.8012431
cg06218079	0.3372641	0.1377713	0.825622	0.0173379
cg02454025	2.5731928	0.3695332	17.91807	0.3398064
cg26202762	0.2664699	0.094838	0.74871	0.0121068
cg09238312	3.4105065	0.3278676	35.47638	0.3045486
cg10118717	0.4232677	0.1540647	1.16286	0.0954445
cg13356792	0.2703126	0.0698333	1.046333	0.0581751
cg04828157	0.0046412	1.10E-09	19618.54	0.4900659
cg08269461	0.0220267	2.25E-13	2.15E+09	0.7676004
cg23996349	0.0096379	0.0009861	0.094198	6.58E-05
cg02353184	1.5751691	0.7058624	3.515073	0.267246
cg10611165	0.3118624	0.0985264	0.987127	0.047479
cg06099446	9.4698709	0.9585677	93.55464	0.0543854
cg22942200	1.6897492	0.6974116	4.094071	0.2453112
cg09368716	0.5629392	0.094964	3.337058	0.5268694
cg07534099	0.1545901	0.0124443	1.920407	0.1464052
cg23918953	0.3323799	0.1776912	0.621733	0.0005661
cg10341792	0.6546246	0.3153765	1.358799	0.2554932
cg23683046	0.5152685	0.20345	1.304997	0.1619607
cg16119134	265.15366	0.0205291	3424717	0.2479295
cg14368473	2.3482684	0.8013213	6.88159	0.1196613
cg18638914	1.456628	0.7648994	2.773914	0.252431
cg16401465	0.2432273	0.0459703	1.286907	0.0962695
cg04287289	6.8333082	1.4453949	32.30543	0.0153188
cg07162085	0.3938238	0.1533497	1.011395	0.0528165
cg25206536	0.3542209	0.1465736	0.856037	0.0211537
cg15735417	1.2349003	0.6322636	2.411935	0.5367528
cg11817892	0.1687339	0.0732345	0.388767	2.93E-05
cg06916001	0.729884	0.3901441	1.365472	0.3244992
cg17085123	1.7963565	0.7322176	4.407019	0.2008005
cg02041435	7.237E+16	0.0424502	1.23E+35	0.0699157
cg17044159	2.8189009	0.9282666	8.560259	0.0674562
cg21002575	0.719804	0.3589325	1.443496	0.3544178
cg18337525	1.7109703	0.6481565	4.516532	0.2781829
cg04960665	0.0039685	1.45E-05	1.089797	0.0536131
cg11592082	8.8796507	0.6177184	127.6442	0.1083294
cg09350141	0.0114051	0.000553	0.235239	0.003766
cg09584658	69.225091	7.98E-07	6E+09	0.649567
cg26569315	0.1951238	0.0599939	0.634619	0.0066145
cg18594551	2.0656029	0.5029191	8.483901	0.3142193
cg03353926	2.3142929	1.0472592	5.114256	0.0380697
cg23539780	194783.21	187.38705	2.02E+08	0.0005892
cg13501527	3.4808985	1.193865	10.1491	0.0223414
cg04403423	0.6532978	0.2582224	1.652831	0.3686884
cg17187705	0.5047455	0.2260527	1.12703	0.0952793
cg14374432	0.6702593	0.3555797	1.263423	0.2160814
cg07157117	4.3909087	0.9572337	20.14145	0.056946

cg04536922	0.5211772	0.2081009	1.305259	0.1641562
cg10222734	0.8566103	0.4245542	1.728357	0.6656292
cg21483999	0.3764064	0.1627787	0.870395	0.0223416
cg03764027	0.758252	0.30066	1.91228	0.5576369
cg25775494	2.3976996	0.8401562	6.842732	0.102165
cg23669276	36.882938	1.8057377	753.3493	0.0190828
cg03319184	1.0113612	0.1845655	5.541942	0.9896145
cg11759875	0.4828999	0.211399	1.103091	0.0841376
cg02535060	1.3724957	0.7219057	2.609405	0.3340924
cg08887425	63.16434	0.7508698	5313.483	0.0667636
cg10068996	1.1898602	0.4502122	3.144666	0.7259088
cg20981086	1.6637618	0.776001	3.567139	0.190788
cg07012823	0.3507377	0.1424164	0.863783	0.0227026
cg10616216	0.4233053	0.2001855	0.895107	0.0244494
cg17516330	0.2688616	0.1090172	0.663075	0.0043437
cg10588471	1.8403458	0.8067996	4.197911	0.1471369
cg00713366	1142095.6	0.0011896	1.1E+15	0.1862323
cg09287190	2.4433896	0.7030454	8.491846	0.159838
cg00007036	33766.601	9.58E-09	1.19E+17	0.4793291
cg17066238	2.80E-07	4.89E-12	0.016073	0.0069575
cg01706515	0.4154279	0.1417313	1.217659	0.1093675
cg01112784	5.0769875	0.2172038	118.6711	0.3123076
cg07814977	0.0551176	0.0079311	0.383043	0.0033884
cg20960181	0.6252488	0.3323593	1.176246	0.1452548
cg13011999	0.8038008	0.408979	1.579778	0.5263924
cg17033854	0.774488	0.2837573	2.11389	0.6178933
cg06773306	0.3397455	0.1114591	1.035599	0.0576369
cg06691218	1.2452104	0.6401613	2.422123	0.5182603
cg24105729	0.5158738	0.2540565	1.047506	0.0670205
cg14696458	4.3144791	1.5149303	12.28752	0.0061848
cg01504212	0.2880677	0.1433112	0.579041	0.0004762
cg11356029	3.0803102	1.44E-15	6.58E+15	0.9501899
cg25716149	1.3496039	0.4560351	3.994058	0.5881037
cg00766914	0.0015498	2.54E-05	0.094393	0.0020306
cg20253410	0.3862605	0.1466471	1.017389	0.05422
cg16914989	0.0133222	0.0005854	0.303197	0.0067599
cg08578641	0.5026498	0.2148169	1.17615	0.1127626
cg01966129	0.4821132	0.1515649	1.533555	0.2165591
cg17641649	2.2684482	0.9291695	5.538125	0.0720749
cg24496563	0.5097945	0.2300603	1.129662	0.0969865
cg25605243	3.5317506	1.4938844	8.34955	0.0040495
cg05941106	1.1820188	0.547676	2.551086	0.6700767
cg22942971	3.56E-05	8.07E-29	1.57E+19	0.7123027
cg24947255	1.7393653	0.9054413	3.341345	0.0965626
cg05894801	0.5280543	0.1720949	1.620277	0.2642923
cg12198090	0.4972454	0.2439364	1.013596	0.0545053
cg02447227	0.5808952	0.2872742	1.174624	0.1305434
cg07670736	0.2158225	0.0844487	0.55157	0.0013611
cg20741169	1.5440967	0.6943346	3.433841	0.2867096
cg01059398	3.645138	0.9337859	14.2292	0.0626917
cg10407598	0.3434088	0.1506303	0.782908	0.0110209
cg07257718	0.1181505	0.0041465	3.366597	0.211413
cg00586094	0.4282228	0.1286336	1.425559	0.1669281
cg16650073	0.1977892	0.0759137	0.515329	0.0009104
cg06119452	1.6359917	0.6903383	3.87704	0.2634904
cg18391323	2.3822411	0.773638	7.335566	0.1303542
cg02159896	77006.468	10.644728	5.57E+08	0.0130799
cg23482872	1.199359	0.6410359	2.243965	0.5695269

cg19933664	0.2993047	0.0001618	553.6372	0.7533059
cg07624483	4.86E-42	3.75E-79	6.31E-05	0.0291275
cg06136573	0.608289	0.2671582	1.385005	0.2363639
cg16985778	0.3464243	0.101619	1.180978	0.0902403
cg13932501	0.4719315	0.229508	0.970421	0.0411915
cg14352032	1.7767197	0.6021594	5.242355	0.297806
cg26674479	3.6666169	1.1856435	11.33906	0.0240965
cg14699004	0.0074206	0.000128	0.430072	0.0179165
cg13911392	0.1626945	0.0291786	0.907156	0.0383494
cg14599823	0.7191072	0.3280657	1.576255	0.4102179
cg04956471	2.1963246	0.7669418	6.28971	0.1427393
cg08570574	0.5147723	0.2627494	1.008529	0.0529643
cg00337968	2.3159844	0.4194786	12.78679	0.3353462
cg02721602	3.429641	0.4605886	25.53784	0.2289188
cg13572071	2.7984244	0.0029532	2651.784	0.7685508
cg07474269	0.5409543	0.2386612	1.226138	0.1411135
cg23478293	0.8736274	0.4237249	1.801227	0.7143988
cg08309069	1.8198075	0.5778272	5.731297	0.3063517
cg00631221	0.4173598	0.1859393	0.936807	0.0341577
cg14561541	1.9380996	0.7491849	5.013756	0.1724112
cg22354618	0.318793	0.1339079	0.758947	0.0097883
cg26899598	0.1214252	0.0368485	0.400127	0.0005293
cg24276070	7.63E-08	1.58E-17	368.3171	0.1497085
cg05645927	0.6531982	0.3487917	1.223274	0.1833873
cg03826247	1.0770776	0.5245605	2.211559	0.8396971
cg06202492	0.6081699	0.3199772	1.155928	0.1290828
cg17829533	0.2712257	0.0918498	0.80091	0.0181856
cg08028026	1.4998665	0.4963047	4.532698	0.4725027
cg17980283	5.2688095	1.3350763	20.79308	0.0176657
cg24515368	0.2106411	0.0928277	0.477979	0.0001948
cg23414431	0.009381	0.0002181	0.403518	0.014981
cg17962384	0.6505878	0.2139186	1.978624	0.448753
cg08817204	0.5048333	0.2385192	1.068495	0.0739732
cg20402284	1.9334467	0.6545234	5.711356	0.232865
cg19771469	4.7726657	0.7876164	28.9206	0.0890856
cg24642372	0.943213	0.0273966	32.47303	0.9741697
cg20811564	0.5219046	0.2547371	1.069276	0.0755799
cg13806292	0.2126273	0.0743351	0.608197	0.0038855
cg10068491	0.2094688	0.0373692	1.174152	0.0755001
cg17508905	0.5135821	0.2483946	1.061885	0.0721854
cg25402422	0.7809919	0.2787585	2.188089	0.6381598
cg08719296	1.8213969	0.7186617	4.616201	0.2063374
cg19761149	0.8572108	0.3853621	1.906805	0.7056507
cg15087907	0.2838574	0.1248972	0.645131	0.0026441
cg23976388	2.6163584	0.6826592	10.02745	0.1606013
cg15112475	0.3944608	0.0957439	1.625162	0.1978388
cg24795046	3.7551047	0.7609795	18.52982	0.1042521
cg13964192	2.1517989	0.8454117	5.476904	0.1079108
cg00561891	4.0927186	1.3206771	12.68315	0.0146083
cg17601595	7.81E-07	4.55E-33	1.34E+20	0.6481852
cg23606751	48308555	651.59007	3.58E+12	0.0019851
cg16959787	1.2536388	0.6448181	2.437292	0.5051526
cg14178506	0.1726748	0.0294109	1.013793	0.0517996
cg20419110	0.0585743	0.0117769	0.291329	0.0005267
cg13568659	1.8119588	0.544165	6.033455	0.3327949
cg14813947	7.91E-18	7.78E-33	0.008049	0.0255174
cg04156995	0.0012356	1.63E-07	9.388817	0.1419011
cg04945608	1.5258006	0.5185185	4.489844	0.4429161

cg02714192	2.1699525	0.7014829	6.712485	0.1787584
cg16933922	0.0608673	0.005003	0.740525	0.0281209
cg03607179	0.1263381	0.0344622	0.463155	0.0018011
cg14857877	3.4064693	0.2434005	47.67464	0.362614
cg25354348	0.2477861	0.07084	0.866712	0.0289714
cg00516639	0.0003298	1.38E-07	0.785474	0.0432974
cg27105990	0.5466243	0.2821033	1.05918	0.0735167
cg05846166	0.0629622	0.0012575	3.1525	0.1660797
cg14092010	0.6410244	0.254586	1.614041	0.3452497
cg11705747	0.1744085	0.0580033	0.524424	0.0018767
cg01982833	0.548291	0.2203764	1.364135	0.1962735
cg09460345	0.4235953	0.1800641	0.996495	0.0490671
cg10678801	0.5489782	0.2518949	1.19644	0.1313638
cg27317813	1.0178773	0.4548744	2.277715	0.9656076
cg27366766	3.4278135	0.7697535	15.2645	0.1059707
cg23243474	1.5719463	0.512824	4.818447	0.4286879
cg02002960	1.8970858	0.7419609	4.850572	0.1812726
cg27070902	0.647933	0.2873745	1.460871	0.2954687
cg05401764	1.7583627	0.8655505	3.572107	0.1185982
cg06073141	113.52627	2.375691	5425.038	0.0164597
cg03946168	1.6891108	0.8194287	3.48181	0.1555029
cg21774457	0.1014685	0.0184792	0.55716	0.0084614
cg26333454	0.3816007	0.164275	0.886435	0.025072
cg25425140	1.8765338	0.8411376	4.186448	0.1241946
cg01311537	1.0270591	0.4544506	2.321155	0.948827
cg05131526	0.0996579	0.0262808	0.377906	0.0006967
cg22764289	0.3045791	0.1492807	0.621436	0.0010851
cg00141162	1.471857	0.510019	4.247612	0.4747286
cg23198793	0.2995806	0.123239	0.728248	0.0078214
cg27034568	3.2865155	1.1841141	9.121742	0.0223464
cg23762579	2.3139575	0.7925359	6.756034	0.1248726
cg27385272	0.0749718	0.0073635	0.763331	0.0286652
cg04219048	0.556728	0.2359499	1.31361	0.1811651
cg03510528	0.4827256	0.1774706	1.313029	0.1537134
cg24870966	0.1825168	0.0631383	0.52761	0.0016864
cg18445438	0.5238646	0.1963686	1.397546	0.1965697
cg05988147	2.7292666	0.8996212	8.280036	0.0762037
cg20203357	2.2586896	1.0822683	4.713876	0.0299632
cg21187265	0.8804328	0.2125276	3.647346	0.8606096
cg11211259	0.0111489	0.0005137	0.241971	0.0041879
cg03319315	2.3779769	0.9042259	6.253718	0.0791067
cg22877230	0.4611844	0.2317649	0.917702	0.0274822
cg20744943	3.2029008	0.9307048	11.02237	0.0648812
cg23359385	3.1596908	0.5516107	18.09908	0.1963873
cg01424901	2.5542918	0.7796337	8.368555	0.1214228
cg00768195	0.6205732	0.2796365	1.377185	0.2407651
cg12385643	1.6818855	0.6940517	4.075689	0.2496218
cg10331424	0.2481946	0.101122	0.609171	0.0023508
cg16411361	0.5013884	0.2356362	1.066858	0.0731364
cg00530127	6.9909801	1.2124029	40.31152	0.029598
cg12099657	0.0004714	5.20E-07	0.427037	0.0274618
cg09963856	2.6804751	1.0492989	6.847378	0.0393479
cg01355757	0.4282	0.1977739	0.927095	0.0313948
cg12845923	1.905856	0.7402817	4.906628	0.1813261
cg10643916	0.063964	0.01233	0.331826	0.001063
cg20609092	0.2681596	0.005495	13.08646	0.5069891
cg20386580	0.4646396	0.1699436	1.270363	0.1352691
cg26620242	0.4289152	0.1744219	1.054731	0.0651987

cg13163521	3.4510853	0.7703794	15.4599	0.1054478
cg08686960	1.5044258	0.3332764	6.79105	0.5953493
cg16385335	0.1568494	0.0410361	0.599515	0.0067722
cg17593342	1.9434336	0.6798141	5.555834	0.2150374
cg09255586	0.2211962	0.0844271	0.579527	0.0021398
cg25557835	0.0289068	0.0027965	0.298804	0.0029432
cg12219531	1291.7461	24.121089	69176.32	0.0004199
cg05583831	2.0508167	0.825506	5.094874	0.121875
cg05387565	1.6898236	0.7106758	4.018012	0.235178
cg02708956	0.3792038	0.1430677	1.005087	0.0512046
cg26110834	0.5101341	0.1959123	1.328333	0.1680546
cg06955214	0.2667176	0.0890763	0.798622	0.0181847
cg21730858	0.3209053	0.1574636	0.653994	0.0017539
cg12047860	2.1241922	0.4469772	10.09491	0.3434463
cg24337025	0.2128526	0.0780921	0.580164	0.0024932
cg15597770	0.3607476	0.1366463	0.952377	0.0395443
cg05718253	0.887224	0.106612	7.383471	0.9118683
cg05342515	1.6224356	0.6676168	3.942827	0.285454
cg11644052	0.1629139	0.0521595	0.508842	0.0017924
cg01763666	9.2635651	0.3208631	267.4463	0.1944814
cg00011578	5.41E-23	1.47E-34	1.99E-11	0.0001612
cg22655196	0.3726965	0.1346805	1.031349	0.0573642
cg02373104	0.6868101	0.3260539	1.446718	0.3229566
cg05369318	3.56E-09	1.13E-19	112.3405	0.1147563
cg07139162	0.8737601	0.1817016	4.201707	0.8662517
cg04550439	3377.8717	0.004364	2.61E+09	0.2402175
cg24103837	2.68E-10	4.65E-22	154.3709	0.1106615
cg00632811	0.3218787	0.0419152	2.4718	0.2757602
cg03525467	1.8673903	0.6107694	5.709432	0.2733865
cg14356225	0.3805495	0.1232297	1.175187	0.0930805
cg04786592	0.3615752	0.1619274	0.807378	0.0130649
cg09830308	1.2359518	0.2941057	5.193972	0.7724234
cg06309968	0.1664609	0.0526218	0.526573	0.002277
cg12584458	1.9933121	1.0287431	3.862279	0.0409603
cg26665055	331102.07	1.62E-32	6.78E+42	0.7718449
cg04855107	0.3787617	0.1452313	0.987807	0.0471391
cg24650262	1.7217119	0.070239	42.20295	0.7392377
cg04079399	1.3038286	0.1703349	9.980155	0.798348
cg16373998	0.4223724	0.1666781	1.070317	0.0692596
cg04129548	0.4754355	0.2523066	0.89589	0.0214454
cg04622125	1.8439329	0.0964671	35.2461	0.6843893
cg00814573	3.5879056	0.7086463	18.16571	0.1226375
cg04247135	0.1629071	0.0487711	0.544149	0.003189
cg18280251	1.4567174	0.4077486	5.204249	0.5625493
cg12160233	1.6111816	0.4681181	5.545409	0.4494446
cg23562261	3.9084718	1.5509126	9.849783	0.003846
cg06194738	12.95211	2.4873774	67.44339	0.0023474
cg11453837	3.3831263	1.1224614	10.19683	0.030373
cg26307218	1.8977422	0.885763	4.065902	0.0993655
cg08802149	0.6270804	0.2926732	1.34358	0.2300092
cg00375444	2.2381934	0.5857096	8.55289	0.2388397
cg16615291	5.7621469	1.6174147	20.52803	0.0068978
cg07986943	2.8367109	0.9506615	8.464557	0.0615876
cg18653282	0.1949852	0.0534704	0.711033	0.0132641
cg23434070	0.7632781	0.2869704	2.030152	0.5883519
cg24029926	7323.4206	19.683458	2724749	0.0032123
cg03992638	1.3535129	0.6759169	2.710388	0.3928816
cg08401872	0.3231627	0.1094525	0.95415	0.0408618

cg20921894	1202.7607	0.6677469	2166440	0.0636856
cg17971015	0.6718837	0.2922457	1.544686	0.3491446
cg13824500	0.4751902	0.2054958	1.098834	0.0819286
cg02234281	0.5339695	0.2137454	1.333939	0.1792273
cg07659054	1.8726201	0.5606806	6.254374	0.3079253
cg13102889	4.9802393	0.8841047	28.05413	0.068713
cg08202399	0.1238739	0.0418163	0.366956	0.0001637
cg20167762	1.8559614	0.7919409	4.349558	0.1546957
cg01382414	1.1777024	0.4287189	3.23518	0.7510576
cg12965095	0.2345983	0.05364	1.026032	0.0541258
cg09065876	1.8169231	0.8432699	3.914772	0.1273331
cg26467809	0.2350748	0.0969545	0.56996	0.0013549
cg13147664	3.428201	0.6298022	18.66072	0.1541151
cg03511041	0.3406578	0.0727118	1.595996	0.1717309
cg15144453	1.1010326	0.4890637	2.478762	0.8161811
cg01037673	1.12E-08	6.89E-38	1.84E+21	0.5938055
cg22719308	0.1359336	0.0091139	2.027445	0.1477975
cg16387834	3.3468616	0.4894805	22.88443	0.2180962
cg14876077	0.4574319	0.1799421	1.16284	0.1003753
cg14086599	0.4447426	0.0100789	19.62481	0.6749645
cg04496791	0.9109577	0.3788797	2.190257	0.834953
cg22530111	0.5095602	0.2437812	1.065101	0.0730848
cg24952158	2.293985	0.4801335	10.96022	0.2981028
cg20123355	0.4430113	0.2085225	0.941189	0.0342078
cg11365440	0.4366123	0.2151765	0.885925	0.0217065
cg19122901	1.6214382	0.8018132	3.278896	0.1785623
cg18243072	1.346988	0.6342893	2.860488	0.4382239
cg10815203	0.0760286	0.0029657	1.949069	0.119527
cg10895452	1.9481814	1.0073724	3.767634	0.0475025
cg10932874	1.4356404	0.6428763	3.206003	0.377687
cg01552272	0.1124061	0.0175709	0.719094	0.0209868
cg19824242	0.4031989	0.1735278	0.936849	0.0347196
cg10445449	1.1864681	0.6458554	2.1796	0.5816112
cg18455739	0.0926608	0.0064059	1.340327	0.0809703
cg13667739	2.2953616	0.7691459	6.850046	0.1363682
cg05592483	0.3865883	0.1520596	0.982842	0.0458994
cg07091016	0.3850048	0.1607933	0.921858	0.0321449
cg02658657	1.39E+20	4.14E-12	4.67E+51	0.2104703
cg11034122	0.3537515	0.1493238	0.838045	0.0182025
cg18763536	0.253273	0.0600919	1.067486	0.0613465
cg23828467	1.1370392	0.4858339	2.661111	0.7672121
cg25310676	0.4489326	0.1846924	1.091222	0.0771745
cg21179654	1.2766041	0.4644774	3.508713	0.6359271
cg13671409	0.1665684	0.04908	0.565301	0.0040421
cg07222243	0.893341	0.1632644	4.888134	0.8965149
cg17452615	0.424106	0.1114699	1.613583	0.2083306
cg09645699	902.13495	0.0002011	4.05E+09	0.383888
cg20225999	0.1491509	0.0506979	0.438795	0.000548
cg05065572	1.9365227	0.9847645	3.808139	0.055433
cg10222608	0.3983356	0.1698024	0.934446	0.0343605
cg11168614	0.8175108	0.403112	1.657911	0.5764758
cg22330625	0.3572922	0.1344647	0.949377	0.0390033
cg19803984	0.0982672	0.0161793	0.596837	0.0117119
cg06608166	0.0356822	0.0004045	3.147579	0.1447602
cg08177833	0.2441146	0.0535057	1.11375	0.0686294
cg14311134	1.5656673	0.6553341	3.740556	0.3130214
cg26433554	0.6325567	0.2402466	1.665489	0.3538179
cg05685483	2.8604953	1.0675968	7.664348	0.0366142

cg11641840	3.1236583	1.1306553	8.629722	0.0280345
cg05616819	1.9785036	0.9840874	3.977773	0.0554992
cg15824062	0.1271158	0.0243701	0.663042	0.0143827
cg19014730	1.6779937	0.4134488	6.810185	0.4689425
cg11630152	1.5991508	0.7346752	3.480835	0.2368026
cg05802386	1.6092333	0.6493396	3.988101	0.3042094
cg14367014	0.2409496	0.0949897	0.611189	0.0027295
cg19668951	0.1800344	0.0357644	0.906274	0.0375888
cg21583694	1.8742172	0.6944256	5.058411	0.2149442
cg07304343	0.0113317	1.78E-05	7.205622	0.1737262
cg05831242	0.1474814	0.0359215	0.605508	0.0079035
cg21817284	1.41E-06	1.62E-11	0.123365	0.0203141
cg10327067	0.189018	0.0280945	1.271701	0.0867423
cg05188868	0.9981637	0.5080797	1.960974	0.9957436
cg12734710	0.2141872	0.0984524	0.465973	0.0001021
cg07085177	0.2524336	0.0847888	0.751546	0.0133951
cg19283264	0.0109253	0.0003261	0.365996	0.0117029
cg26565578	6234070.8	1630.5626	2.38E+10	0.0002012
cg18317135	0.1477855	0.040715	0.536425	0.0036506
cg18985499	0.3174715	0.0006584	153.0712	0.7158691
cg05045781	0.0012739	2.40E-05	0.067578	0.0010026
cg24054898	2.8486282	0.7191687	11.28342	0.1360746
cg08284873	1.2667857	0.5363884	2.991761	0.5896524
cg07410188	0.6736314	0.2357477	1.924851	0.4608122
cg02245810	0.4702222	0.2222271	0.994968	0.0484778
cg18025976	4.49E-30	3.35E-50	6.02E-10	0.0042651
cg11486637	0.2090506	0.0410866	1.06366	0.059347
cg19495650	0.2317754	0.1036999	0.518032	0.0003669
cg25342894	4.5904724	1.6915991	12.45711	0.0027715
cg23521468	2.7267667	0.7134185	10.42201	0.142556
cg27255656	0.3746603	0.1142921	1.228171	0.1050872
cg04503593	0.5491166	0.2589973	1.164217	0.1179571
cg16171290	2.8390573	0.922649	8.735983	0.0688231
cg02192746	0.284218	0.1052968	0.767164	0.0130228
cg09069694	0.2779363	0.1204386	0.641394	0.0026923
cg27111250	0.5763816	0.2640514	1.258148	0.1665448
cg02989828	2.5988435	0.7066809	9.557336	0.1505929
cg17592853	7.1127566	1.1980935	42.22651	0.0308627
cg11238995	1.3365069	0.7357845	2.427682	0.3408602
cg04774139	0.4016793	0.201733	0.799801	0.0094397
cg26945413	0.0956949	4.76E-06	1922.934	0.6425155
cg00310215	0.1726729	0.0366315	0.813942	0.0264054
cg15989318	0.3742735	0.1595962	0.877719	0.0238283
cg06582250	0.8060584	0.3524557	1.843438	0.6094769
cg25165880	0.2337565	0.0557708	0.979762	0.0468198
cg11391618	0.6500108	0.3497627	1.208002	0.1730917
cg05313771	1.79E-16	1.12E-27	2.85E-05	0.0058674
cg13628643	40561.95	0.0150681	1.09E+11	0.1601364
cg26944151	0.0815915	0.0063613	1.046516	0.0542245
cg26361553	6.1769143	0.4690093	81.35078	0.166257
cg25616055	2.1815015	0.6981776	6.816244	0.1796347
cg10018272	2.3276099	2.59E-09	2.09E+09	0.935988
cg07176842	2.0916863	0.8880926	4.926458	0.0913282
cg14945182	12.121314	0.2178473	674.4462	0.2236981
cg11706999	0.1848686	0.0700179	0.48811	0.0006548
cg26705829	18.077833	3.4110564	95.80845	0.0006688
cg18860644	0.3100515	0.0032937	29.1867	0.6135492
cg23239407	1.0533843	0.2129731	5.210134	0.9491576

cg03366574	16.384206	0.0045787	58628.5	0.5029902
cg25455811	0.2714772	0.0940197	0.783877	0.0159504
cg25274157	0.3179198	0.0854341	1.183052	0.0874066
cg23418671	7.4887914	0.9726383	57.65966	0.0531962
cg15174248	1.4473969	0.4191571	4.998025	0.5586818
cg04014805	0.5498106	0.2722143	1.110492	0.0953619
cg10002850	1.8875392	0.7710898	4.620479	0.1642739
cg01473336	0.039298	0.0042853	0.360382	0.0042013
cg24444408	270601838	0.0485746	1.51E+18	0.0899251
cg23882131	1.5753734	0.2085094	11.90259	0.6595816
cg10560393	1.859252	0.9989166	3.460567	0.0504011
cg11176481	1.8169072	0.8227257	4.012457	0.1396133
cg04126957	0.1420687	0.0527499	0.382626	0.0001132
cg25003357	0.3875383	0.1975881	0.760096	0.0058141
cg01702513	0.417866	0.1468488	1.18906	0.1019608
cg14143752	1.5628936	0.5280717	4.625577	0.4199027
cg23044504	2.423E+15	0.070571	8.32E+31	0.0682287
cg07002423	0.0439835	0.0031841	0.607561	0.0197043
cg19750282	0.1827427	0.0391829	0.852283	0.0305097
cg06855803	1.0749586	0.2579716	4.479314	0.9209275
cg25947184	1.107515	0.4460777	2.749721	0.8257981
cg02672229	2.0446963	0.5502786	7.597574	0.285511
cg05870029	0.2396526	0.0602968	0.952511	0.0424512
cg12667125	0.2169713	0.058799	0.800635	0.0218051
cg22155724	0.2630983	0.1079244	0.641382	0.0033159
cg17167253	2.1052956	1.1076168	4.001627	0.0230935
cg00481884	1.9140905	0.6103579	6.002613	0.2655634
cg17642941	2.9577773	0.7142905	12.24774	0.1346926
cg16947025	0.4789193	0.1435424	1.597881	0.2310789
cg22582999	0.406206	0.205046	0.804713	0.009798
cg07189401	0.1688024	0.0288516	0.987616	0.0484057
cg10373887	261374.78	1.1990417	5.7E+10	0.0467114
cg12002708	0.9210963	0.4151947	2.043423	0.8397854
cg08104023	3.9233479	1.0599676	14.52182	0.04064
cg19699088	0.1603319	0.024237	1.060622	0.0575765
cg13348530	0.3614281	0.1372252	0.951941	0.0394327
cg14835138	1.8195121	0.7209781	4.591851	0.2050426
cg06412435	1.5441992	0.5666687	4.208016	0.3956013
cg02257712	5.39E-11	2.23E-34	1.3E+13	0.3894057
cg18491230	2.417E+10	0.0004514	1.29E+24	0.1382449
cg26848442	2.6451073	0.2431753	28.7718	0.4244073
cg15244129	0.4427879	0.1672585	1.172204	0.1009859
cg04708264	8.906084	1.1957099	66.33577	0.0328077
cg00246768	0.4778851	0.2063552	1.106704	0.0848279
cg18034719	0.1567912	2.40E-05	1024.21	0.6793152
cg15238694	0.1885691	0.0391461	0.908348	0.0375436
cg02863169	2.2376131	0.9091837	5.507041	0.0796418
cg08715231	1.271955	0.4429524	3.652468	0.654901
cg17661462	1.3240444	0.6814501	2.572593	0.4075277
cg13633756	0.4018616	0.0025488	63.36015	0.7240224
cg08619102	0.7524451	0.3585506	1.579062	0.4520179
cg06183267	2.962651	1.4212059	6.175953	0.0037575
cg04986336	0.6847106	0.2740355	1.710831	0.41756
cg02434051	0.2337885	0.0587685	0.93004	0.039121
cg00679763	1.5452581	0.7694631	3.103232	0.2212117
cg03759454	1.8806613	0.9257304	3.820645	0.0807124
cg08949143	3.6036083	1.1237281	11.55617	0.0310705
cg01492538	0.2607058	0.1252631	0.542598	0.0003246

cg17369527	0.0004413	1.57E-06	0.124247	0.0072604
cg08961196	0.1675487	0.0445399	0.630278	0.0082219
cg01135546	1.2305461	0.6031861	2.510409	0.5684804
cg00332937	2.951828	1.1563159	7.535388	0.0235922
cg10082398	0.5667551	0.281876	1.139549	0.1110718
cg22466209	0.9651529	2.07E-05	44970.4	0.99484
cg21049958	0.2617382	0.0768133	0.891862	0.0321191
cg01637125	3.7730591	1.1235903	12.67008	0.0316735
cg13548265	0.0240859	0.0018414	0.315047	0.0045049
cg09628707	2.8739214	1.0873415	7.59598	0.0332691
cg16822666	0.2001526	0.0889354	0.450451	0.0001015
cg26187724	1.4991192	0.4720983	4.760361	0.4922166
cg25277041	1.6271175	0.6370711	4.155755	0.3088971
cg19585100	3.6763415	1.009133	13.39317	0.0484105
cg19476788	1.1428605	0.3849185	3.393264	0.8099452
cg04085542	0.3560057	0.1555351	0.814864	0.0145035
cg02305353	0.5344523	0.2420099	1.180279	0.1211616
cg23525596	1.919941	0.6063619	6.079165	0.267329
cg19445684	0.2931621	0.1109476	0.774636	0.0133215
cg24408001	2.6502878	1.1485462	6.11558	0.0223365
cg02147194	1.8906289	0.6485687	5.511332	0.2433047
cg26687497	0.5814224	0.3063832	1.103363	0.097109
cg21266502	2.3498768	0.8928744	6.184432	0.0835477
cg14350176	11006757	1.00E-18	1.21E+32	0.5815271
cg00083046	1.647605	0.7475248	3.631455	0.2155988
cg06398735	0.0002869	1.08E-10	758.8555	0.2796895
cg18131582	0.5152853	0.2345272	1.132146	0.0987539
cg23867441	0.8837407	0.3868143	2.019051	0.7693808
cg22850505	0.316366	0.117617	0.850961	0.0226286
cg05473871	0.0120679	0.0004437	0.328227	0.0087673
cg23062876	2.212E+13	3.2862716	1.49E+26	0.0414591
cg01820192	2.7858467	0.9570794	8.108985	0.0601778
cg27610545	0.8065175	0.4141295	1.570694	0.527198
cg16810626	1.2848439	0.5833873	2.829722	0.5338209
cg17479131	2.5435195	0.6320284	10.23608	0.1888104
cg07313437	0.303556	0.1381653	0.666928	0.0029914
cg19029285	0.0645845	0.011031	0.378131	0.0023775
cg17015506	1.5629012	0.0863567	28.28571	0.7624745
cg10539861	0.7632489	0.2275034	2.560617	0.6617685
cg02371052	0.263866	0.0849058	0.82003	0.0212829
cg02516330	0.7381577	0.2823883	1.929531	0.5357386
cg01212326	3.534624	1.432993	8.718512	0.0061258
cg09225462	2.2262316	0.9004988	5.503735	0.0830933
cg07469555	0.5517801	0.2722454	1.118334	0.0990094
cg16529799	0.4646897	0.2140792	1.008675	0.0526087
cg02617919	6066.298	7.6668165	4799902	0.0105222
cg08007899	1.2671783	0.5118223	3.137301	0.6086966
cg25856663	0.0731278	0.0174072	0.30721	0.0003548
cg15794034	1.7194468	0.7323023	4.037263	0.2132964
cg24135793	22.713158	1.1055264	466.6442	0.0428658
cg04929703	2.4823676	0.9173951	6.717007	0.0734208
cg18743034	0.4461274	0.1362635	1.460623	0.1822471
cg12873610	0.5270763	0.1946877	1.426949	0.2075661
cg27255672	0.7958222	0.3885199	1.630117	0.5324553
cg27540038	1.1197192	0.535635	2.340719	0.7637483
cg08892499	2.9480132	0.3400766	25.55536	0.3265248
cg06119296	1.7831687	0.0711314	44.70164	0.7249272
cg02351713	2.7487131	1.2736757	5.931984	0.0099854

cg01788444	0.3418952	0.1387414	0.84252	0.0196821
cg04938456	0.0059818	1.28E-05	2.80332	0.1027966
cg16822035	2.6926385	1.0612039	6.832148	0.0370687
cg02367696	3.0783841	1.1712172	8.091111	0.0225777
cg01477379	0.6506895	0.267688	1.58168	0.3430048
cg09236658	2.4512163	1.1216614	5.356751	0.0245887
cg07889938	0.4661347	0.1883499	1.153606	0.0987588
cg22131172	0.3089796	0.1136754	0.839833	0.0213293
cg09481121	5.6626869	0.7623818	42.06032	0.0901176
cg26870057	3.1198301	1.1030485	8.824036	0.0319646
cg06350257	1.0628602	0.0512312	22.05046	0.9685686
cg05285677	3.7535849	0.7580099	18.58736	0.1051197
cg15429134	2.5175556	0.5358328	11.82848	0.2421667
cg00533811	32.794679	0.0001852	5805862	0.5713269
cg11728664	1.4972908	0.5043332	4.445235	0.4671977
cg19010396	21.321219	0.0968359	4694.481	0.2662745
cg26313188	0.0892526	0.0218344	0.364839	0.0007695
cg05250119	3.5508115	1.1995551	10.51078	0.0221041
cg17055721	2.01E-12	9.00E-24	0.448366	0.0433701
cg03790804	0.1717342	0.0368316	0.800743	0.0249063
cg19778647	0.4460545	0.210398	0.945658	0.0352306
cg12197470	0.7218991	0.2909079	1.79142	0.482226
cg12214908	1.0840248	0.4810121	2.442994	0.8456961
cg18450931	0.441224	0.1895606	1.027	0.0576753
cg17907457	0.3465194	0.165544	0.72534	0.004924
cg04661382	2.5649148	1.0518649	6.254404	0.038345
cg15094819	0.1820832	0.0506361	0.654755	0.0090932
cg20453222	439200.78	0.2080527	9.27E+11	0.0803489
cg14777768	2.0420495	0.6901418	6.042188	0.1970774
cg05240948	0.9693409	0.2967394	3.166488	0.9588817
cg05665095	6.4683529	0.5335166	78.42228	0.1425222
cg27227742	0.8191787	0.3143714	2.134589	0.6831448
cg15648896	3.4400787	1.0508671	11.26131	0.0411549
cg18208171	0.312239	0.1281359	0.760857	0.0104254
cg19561453	0.6970043	0.2143022	2.266962	0.5486005
cg14605877	3.4478792	0.8257032	14.39727	0.0896336
cg18126978	3.643645	0.6756257	19.65015	0.1326101
cg13575499	0.054129	0.0014691	1.994384	0.1130068
cg05056325	0.1293503	0.0264965	0.63146	0.011463
cg16557736	0.0350241	0.0017004	0.721418	0.0298917
cg18623980	1.5631874	0.8239377	2.965704	0.1715474
cg10785537	1.8651873	0.2218648	15.68038	0.5660657
cg01581024	0.4252305	0.1879216	0.962215	0.0401297
cg03449108	2.1960267	1.1267517	4.280032	0.0208619
cg02969038	1.3586993	0.4604978	4.008844	0.578713
cg07717632	2.0273078	1.0448588	3.933524	0.0366428
cg21684012	2.3978968	1.0671051	5.388325	0.0342435
cg22981158	2.6880616	0.5840792	12.37105	0.2042369
cg09244071	0.9319724	0.3832596	2.266277	0.876509
cg01040523	1.1954099	0.636767	2.244157	0.5786006
cg00213052	1.4157242	0.5730815	3.497365	0.451201
cg09450024	0.7651884	0.3794394	1.543101	0.4545601
cg14113970	0.4271277	0.2013075	0.906266	0.026664
cg26111265	0.0354511	0.0044292	0.283752	0.0016498
cg06250904	8.59E-19	8.75E-36	0.084275	0.0371709
cg05233373	0.3927348	0.1895599	0.813678	0.0119115
cg26366091	0.9470941	0.4524403	1.982554	0.885331
cg10779656	1.0841086	0.5245055	2.240761	0.8274265

cg03286783	101894.16	1.6036683	6.47E+09	0.0409867
cg10317827	25.062549	0.8695025	722.4031	0.0603231
cg22358291	4.3426207	1.0635622	17.73131	0.0407745
cg06529685	14.406784	3.0002362	69.17969	0.0008609
cg19956914	0.1873873	0.0472567	0.743048	0.0171949
cg24666096	0.3566875	0.1307115	0.973334	0.0441427
cg09862440	0.1716859	0.0529007	0.557196	0.0033501
cg25699034	0.2151492	0.0631487	0.733018	0.0140278
cg03142975	0.2257163	0.0559184	0.91111	0.0365532
cg14023589	0.8706186	0.4050713	1.871218	0.7226577
cg01339648	3.0932194	0.8157571	11.72899	0.0968114
cg27406618	0.9474823	0.4175266	2.150097	0.8973347
cg01377268	0.3463475	0.154602	0.775906	0.0099806
cg26180843	1.3189424	0.4758351	3.655908	0.5945931
cg14704921	0.2363129	0.046616	1.197953	0.0815296
cg04876028	0.4753053	0.2070574	1.091075	0.0793664
cg13584784	1.267186	0.0236574	67.8756	0.9071877
cg21894914	0.2432848	0.0746368	0.793006	0.0190442
cg18878907	1.7626795	0.8023419	3.872463	0.1580795
cg08159291	0.2247159	0.0447937	1.12733	0.0696299
cg19378036	1.2558276	0.6504083	2.424789	0.4974059
cg14427527	0.284794	0.1247549	0.650136	0.0028603
cg17740822	0.1809217	0.0277297	1.180418	0.0739971
cg17417496	3.0305301	0.7155542	12.83496	0.1321967
cg00215851	0.4599778	0.2448167	0.864237	0.0158039
cg00605358	24.211064	0.6066719	966.2152	0.0902152
cg17101681	1.933754	0.9209344	4.060446	0.0814479
cg14510314	45304.931	14.786886	1.39E+08	0.0088534
cg08091707	1.881084	0.714616	4.951578	0.2007119
cg07029980	0.090083	0.0046419	1.748206	0.111656
cg26680009	4.24E-55	2.43E-93	7.40E-17	0.0053247
cg13819166	6.676291	0.4973163	89.62678	0.1519148
cg10841956	1735.861	0.1154927	26090082	0.1284898
cg06501716	0.8583017	0.242688	3.03551	0.8125902
cg10482632	2.179356	0.5313414	8.938871	0.2793292
cg04380118	0.4815345	0.1632736	1.420165	0.1854035
cg24507921	0.3354475	0.1299559	0.865871	0.0239684
cg07366082	3.9283302	0.3465761	44.52638	0.2693635
cg24475517	0.4574308	0.2194368	0.953545	0.0368984
cg09734932	2.1048688	1.0555547	4.197293	0.034558
cg07772537	1.95E-06	4.05E-15	937.9247	0.197385
cg21248130	17.903521	2.96E-07	1.08E+09	0.7523023
cg19296371	2.7759498	1.045499	7.370545	0.0404357
cg02500075	0.4252985	0.1939431	0.932639	0.0328402
cg07213487	0.2328589	0.1018901	0.532174	0.0005488
cg25354466	1.6935715	0.7678727	3.735234	0.1917338
cg03564793	0.4260151	0.2059307	0.881311	0.0214133
cg18267131	0.8589341	0.3455978	2.134758	0.7433921
cg06719332	2.511884	0.834432	7.561505	0.1014113
cg16647921	1.3482444	0.8015883	2.267701	0.2600313
cg00221658	0.1618286	0.0474148	0.552328	0.0036408
cg18810310	0.0960953	0.0219805	0.420114	0.0018571
cg16887070	0.1193923	0.0361607	0.3942	0.0004876
cg02140384	1.6571102	0.4839814	5.673801	0.4212192
cg13484553	1.2468574	0.6592757	2.358123	0.4974025
cg16317730	2.56E-24	1.55E-42	4.23E-06	0.0111466
cg14725733	0.3103063	0.1356651	0.709763	0.0055699
cg01291593	0.5998709	0.2818353	1.276792	0.1848517

cg12751456	0.5922088	0.3039973	1.153666	0.1236032
cg05129610	2603.9494	5.0124615	1352739	0.0136928
cg00836641	0.3741076	0.1730664	0.808687	0.0124243
cg14962159	2.4587697	0.8909351	6.785622	0.0823877
cg14221039	29.162352	3.1586572	269.2419	0.0029381
cg13691257	0.7814993	0.3894567	1.568188	0.4878018
cg15046675	2.9656757	1.0699917	8.219907	0.0366154
cg24310126	0.5940169	0.2701249	1.306271	0.1951663
cg04809316	2.5389496	0.9083584	7.09661	0.0756198
cg18468844	3.0950732	0.0819525	116.8906	0.5420042
cg02160692	6.30E-07	7.57E-12	0.052419	0.013509
cg12726927	0.0488384	0.0010125	2.355704	0.1268363
cg16082503	0.7349548	0.1252015	4.314315	0.7330896
cg20162240	0.3082495	0.1363093	0.697075	0.0047024
cg26997090	2.8303217	0.3233641	24.77307	0.3472358
cg25605289	0.1487878	0.0107384	2.061561	0.1554483
cg23246095	1.6512224	0.5833405	4.674003	0.3448157
cg10536349	0.4519132	0.2357077	0.866436	0.016772
cg17208953	0.3299478	0.1407904	0.773246	0.0107176
cg03677492	1.6711867	0.8478416	3.294088	0.1380153
cg04972669	0.5726408	0.3019826	1.085882	0.0877111
cg05223158	1.126426	0.3466526	3.660251	0.8430491
cg02125191	1320780.7	2.12E-06	8.22E+17	0.3090804
cg11077392	0.89396	0.2765779	2.889473	0.851448
cg18196463	40.889733	1.1347059	1473.483	0.0424518
cg27405791	1.5888129	0.854665	2.953586	0.1433218
cg06304097	0.0627081	0.0081664	0.48152	0.0077533
cg20377232	2.89501	0.465991	17.9855	0.2540307
cg20334252	1.2641008	0.5587907	2.859659	0.5736525
cg16410796	0.4752529	0.1566715	1.441649	0.1888786
cg11178985	0.0001998	1.14E-07	0.351147	0.02545
cg13452923	1.823412	0.6099022	5.451417	0.282348
cg21375511	0.1685324	0.0260934	1.088521	0.0613664
cg01348086	1.9508031	0.6635832	5.734975	0.2245278
cg06717068	0.5831705	0.2190316	1.552688	0.280435
cg24768116	0.4058484	0.1966696	0.837511	0.0146997
cg12819747	0.4468449	0.0013888	143.7698	0.784507
cg17357797	3.33E-08	2.71E-22	4079342	0.2981869
cg06166809	0.5243805	0.2187742	1.256889	0.147802
cg15138846	2.2292441	0.1875234	26.50085	0.5256186
cg16666329	2.7578096	1.0925163	6.961465	0.0317728
cg03612791	2.0978461	0.6602005	6.666094	0.2090944
cg10896604	4.3485356	0.8306638	22.76464	0.0818071
cg05647733	0.1233249	0.0383639	0.396441	0.0004432
cg18474885	0.0342293	0.0024437	0.479459	0.0122176
cg16021217	1.2310934	0.4808197	3.152098	0.6647133
cg11878254	1.3093061	0.3602106	4.759111	0.6823324
cg26253974	0.0348391	0.0019143	0.634049	0.0233442
cg14573833	0.2965456	0.1439388	0.610949	0.0009805
cg08454824	0.4655543	0.1712316	1.265775	0.1341
cg07544451	0.2325065	0.0466893	1.15785	0.0749077
cg18842363	0.6441266	0.1936447	2.142579	0.4731847
cg10876207	1.2509837	0.5489898	2.850618	0.5941064
cg02990598	2.0512366	0.4928415	8.537372	0.3234193
cg14438325	0.2647806	0.0826165	0.848605	0.0253375
cg01026458	0.2309446	0.0739821	0.720923	0.0116239
cg02701826	0.5163359	0.2578746	1.033847	0.0620419
cg08296191	0.4653358	0.2315136	0.935312	0.0317363

cg07117059	0.7815778	0.413968	1.475631	0.4472405
cg27572053	0.2862494	0.0963363	0.850549	0.0243668
cg02751453	0.1638391	0.027953	0.960299	0.0449775
cg22232704	1.2005097	0.5404095	2.66691	0.6536163
cg13982695	3.0176943	0.8181478	11.1306	0.0972037
cg00467326	1.14E-05	2.80E-10	0.467594	0.0357042
cg01787574	2.4887597	1.1069985	5.595242	0.0273915
cg14018414	0.4248702	0.1924442	0.938011	0.0341467
cg10023862	6.6612107	0.1241165	357.5005	0.3507297
cg23258940	0.4856235	0.1626743	1.449708	0.1955084
cg11796455	2.3005826	0.926947	5.709798	0.0724305
cg06811361	0.7563214	0.2653105	2.156048	0.6012936
cg08465708	0.7891121	0.3946512	1.577844	0.5028905
cg25375420	0.0920839	0.0133823	0.633633	0.0153663
cg11606261	0.8558514	0.3805942	1.924574	0.7065605
cg17055371	3.405E+09	8.42E-14	1.38E+32	0.4085638
cg17737681	1.878825	0.6831028	5.167573	0.221828
cg26870745	1.0387442	0.5727866	1.883755	0.9003955
cg23413051	2.1226831	0.8287432	5.436887	0.1167613
cg11576217	141475356	0.1181203	1.69E+17	0.0784613
cg12596182	0.6279837	0.2519035	1.565534	0.3181654
cg04725637	2.9850934	0.998578	8.923472	0.0502985
cg21368479	0.3402647	0.1324096	0.874408	0.0251779
cg21669272	2.04E-49	2.64E-78	1.57E-20	0.0009544
cg05331498	0.2193603	0.0833793	0.577109	0.0021135
cg07096238	1.4826554	0.590849	3.720523	0.4014711
cg07539244	2.032756	0.7871379	5.249521	0.1427848
cg01941586	0.632632	0.3292414	1.215592	0.16942
cg12904904	0.5998381	0.2397795	1.500569	0.2746269
cg08869244	0.1700108	0.0393969	0.733653	0.017543
cg23493787	0.3926217	0.1120627	1.375585	0.1438835
cg25355482	1.8624095	0.7547131	4.595878	0.1772273
cg08493294	2.8597877	0.9878894	8.278645	0.0526847
cg25215834	0.2741633	0.0542726	1.384962	0.1173778
cg02669193	1.3199577	0.5817994	2.994655	0.5065988
cg12435551	1.7834222	0.8049927	3.951085	0.1540188
cg02770351	0.0010464	7.66E-06	0.14292	0.0062294
cg17850880	239132404	5.90E-14	9.69E+29	0.4472514
cg13660174	1.5401098	0.6013847	3.944128	0.3680744
cg13328209	0.5597748	0.2336881	1.340881	0.1929738
cg13231131	0.319447	0.126621	0.80592	0.0156507
cg25465483	0.7596221	0.3428137	1.683205	0.4982328
cg13800349	0.3832357	0.2132803	0.688623	0.0013383
cg03268893	1.2224081	0.6794548	2.199236	0.5027245
cg04246763	1.6071901	0.7091779	3.642331	0.2556624
cg03997139	0.1799637	0.0105064	3.082589	0.2367104
cg24277817	2.2879411	0.854656	6.12489	0.0994842
cg18260397	0.2528058	0.0147593	4.330205	0.3427384
cg18531916	0.2139963	0.0635243	0.720896	0.0128434
cg26731327	0.0823128	0.0075996	0.891549	0.0399368
cg21650866	0.3431353	0.1207357	0.975203	0.0447411
cg02317738	6.0611592	0.4224497	86.96338	0.1848713
cg16334072	2.04E-06	5.78E-10	0.007224	0.0016744
cg03106245	12.947008	1.1786243	142.2209	0.0362267
cg16330965	0.2455712	0.0776722	0.776406	0.0168079
cg18751133	0.3927157	0.1838242	0.838984	0.0158107
cg19611441	1.099787	0.5186938	2.33188	0.8040947
cg13474897	0.7175601	0.3777936	1.362894	0.3105682

cg14766621	0.5931081	0.290176	1.212289	0.1520949
cg02640612	0.1490657	0.0194801	1.140683	0.0667743
cg01896517	2.4470656	0.998988	5.994196	0.0502595
cg20471691	0.5391367	0.209925	1.38463	0.1992363
cg06083089	1765552	0.0006064	5.14E+15	0.195771
cg24062767	0.003358	8.39E-05	0.134352	0.0024749
cg17415355	5.9923954	6.14E-06	5851458	0.7991481
cg20234767	0.4664156	0.2005624	1.084667	0.0765245
cg21205855	0.2580686	0.0842998	0.79003	0.0176524
cg07707227	0.4026532	0.0056852	28.51785	0.6755744
cg03632704	1.4918181	0.6079243	3.660853	0.3824904
cg08295639	0.3836738	0.1735831	0.848041	0.0179195
cg19605773	0.4936286	0.1704305	1.429728	0.1932189
cg03393241	0.5486168	0.2398442	1.2549	0.1549925
cg02927821	0.5232987	0.2390413	1.145582	0.1052371
cg22748722	5.4134013	0.362323	80.88064	0.2209069
cg07217935	1.440536	0.6574383	3.156408	0.3617512
cg12747657	0.1264941	0.038634	0.414163	0.000634
cg16863829	0.3536823	0.1518596	0.823729	0.0159741
cg10269127	1.160547	0.3132899	4.299115	0.8236547
cg13096351	0.3654743	0.1087937	1.22775	0.1035067
cg25420477	1.9453708	0.093181	40.61417	0.6677613
cg05951364	0.4545277	0.1722243	1.199572	0.1112804
cg11896795	3.54E-22	2.30E-38	5.43E-06	0.0093901
cg05121006	2.115456	1.1317391	3.954228	0.018888
cg17953816	0.2138144	0.0634949	0.720005	0.0127654
cg23530330	1.8204061	0.4063872	8.154486	0.4336192
cg09373597	3.0833308	1.1513607	8.257125	0.0250651
cg12598235	2.6398671	0.8093685	8.610291	0.107545
cg19323245	0.2556751	0.0819138	0.798031	0.0188521
cg23020977	0.0118654	1.60E-09	87887.25	0.5827155
cg11011938	0.1911602	0.0128374	2.846547	0.2298315
cg16940935	0.5889177	0.2972435	1.166801	0.129077
cg16008138	3.9792478	1.100113	14.39344	0.035255
cg03847348	0.4701852	0.1717671	1.287057	0.1418918
cg13594903	0.0005331	9.12E-07	0.311779	0.0204227
cg19463193	0.5678418	0.2768375	1.164742	0.1226087
cg10599571	0.1853112	0.0569295	0.603206	0.0051193
cg19194367	0.8563817	0.4077738	1.798521	0.6821524
cg11015833	2425.5067	0.4358921	13496649	0.0765187
cg03882612	0.2161006	0.0714438	0.653654	0.0066705
cg03776878	6.373662	0.9257811	43.88032	0.0598876
cg05991442	1.2882071	0.0140064	118.4803	0.9125849
cg02876466	2.870336	0.2153118	38.26464	0.4249275
cg27635653	0.2142117	0.0575066	0.797936	0.0216539
cg15097019	1.642E+14	503184.81	5.36E+22	0.0010656
cg00754777	9.738E+13	216.00581	4.39E+25	0.018644
cg16081992	0.322201	0.0545684	1.902448	0.2112658
cg16202600	0.9984719	0.2680688	3.718994	0.9981814
cg18259930	1.3099957	0.5530565	3.102918	0.5393891
cg16669395	0.3852786	0.1656547	0.896079	0.0267767
cg08885800	1.0065342	0.3822743	2.65022	0.9894798
cg14620184	0.517196	0.2555051	1.046913	0.066871
cg16785912	1.5298998	0.8302021	2.819305	0.1727819
cg15505198	1.7171796	0.6805483	4.332838	0.2522196
cg14235350	25.642278	0.6507996	1010.336	0.0834878
cg19519310	1.4069531	0.5510806	3.592065	0.4752596
cg13276981	2.8737056	0.0559641	147.5621	0.5993797

cg23968552	2.1654936	0.9827149	4.771844	0.0552748
cg00609424	1.10E-19	6.97E-34	1.74E-05	0.0088702
cg15317817	7.65E-07	3.53E-20	16595033	0.3687242
cg09175485	0.3118795	0.1153874	0.842976	0.0216376
cg26374686	0.9125803	0.2914899	2.857056	0.8751645
cg25696974	0.1492975	0.0445388	0.500457	0.0020587
cg00495512	2.294598	0.6950209	7.575571	0.1728996
cg19006628	0.1213776	0.0088996	1.655417	0.1136795
cg02848448	0.1454273	0.0441726	0.478783	0.001517
cg01625761	0.0339616	0.002622	0.439891	0.0096426
cg17653203	3.3986347	1.0479998	11.02168	0.041543
cg23159844	1.93E-05	3.01E-09	0.124348	0.0152686
cg26778345	0.2339896	0.0675958	0.809978	0.0218704
cg09600096	1.2922275	0.7304124	2.286177	0.3784615
cg05850280	0.0113857	0.0002141	0.605464	0.0272824
cg23137128	8.66E+34	2.45E-10	3.06E+79	0.124261
cg02844611	3.6612101	1.0313579	12.9969	0.0446719
cg25579648	0.8174661	0.3810061	1.753911	0.604838
cg09791621	1.0101868	0.512036	1.99298	0.9766775
cg17377463	9.6055539	0.3530185	261.365	0.1795265
cg22262152	1.3755525	0.525515	3.600554	0.5160317
cg25460340	0.5049546	0.2217599	1.149798	0.1036332
cg22178798	1.6067459	0.5581797	4.625092	0.3793589
cg01405761	0.0120974	0.0003122	0.468783	0.0179819
cg13130338	1.6957842	0.6887804	4.175038	0.2505917
cg07636326	0.3287722	0.1288437	0.838933	0.019943
cg25091458	0.0016178	9.29E-15	2.82E+08	0.6265081
cg13702421	3.6806653	1.0395345	13.03208	0.0433761
cg07042144	2.0055035	0.8899398	4.519457	0.0932126
cg27086773	0.959947	0.4084932	2.255847	0.9252913
cg18356403	1.5755806	0.3252168	7.633229	0.572272
cg17176029	1.9355556	0.6554127	5.716055	0.231978
cg11333968	1.8417997	0.3429837	9.890342	0.4763565
cg18406285	2.9296267	0.6799085	12.62333	0.1492199
cg09072291	0.9626297	0.2509402	3.692736	0.9557218
cg07566286	7.4431831	1.6736803	33.10129	0.008379
cg08642528	0.0031476	1.43E-06	6.920787	0.1423032
cg20882852	7393391.5	1.19E-06	4.58E+19	0.2926007
cg06609004	0.1645346	0.0546665	0.495215	0.0013273
cg13574337	6.1611669	1.6021831	23.69266	0.0081478
cg01022567	0.0180278	0.0018485	0.175815	0.0005484
cg15195321	1.0655464	0.4100236	2.769082	0.8963345
cg20541456	1.1003008	0.5136564	2.356949	0.8057417
cg01642564	28.122818	0.38087	2076.543	0.1284687
cg17063452	0.3153545	0.1294999	0.767942	0.0110402
cg25485875	0.1343871	0.0280148	0.644655	0.0121157
cg07824824	2.1079735	0.6618987	6.713342	0.207031
cg14077369	0.1117788	0.0383215	0.326045	6.02E-05
cg03687532	0.2008798	0.079121	0.510012	0.0007346
cg24877198	8.5452958	0.9395334	77.72165	0.0568331
cg07617147	0.7995894	0.0654616	9.76669	0.8609545
cg07483413	4.6367364	0.5421038	39.65906	0.161266
cg21834679	2.190733	0.7225413	6.642265	0.1658295
cg24150244	3.7566801	1.009676	13.9774	0.0483444
cg00424354	0.3823418	0.1912272	0.764459	0.0065331
cg11082362	4.86E-07	8.72E-28	2.71E+14	0.5508897
cg09975219	14.809359	1.6619925	131.9604	0.0157269
cg17126555	0.199288	0.0566799	0.700703	0.0119237

cg23155805	0.4545629	0.156746	1.318231	0.1466808
cg24775616	0.8033619	0.2562434	2.518662	0.7072505
cg14468720	99.697061	1.5326215	6485.296	0.0307415
cg06708560	3.74E-06	9.72E-14	144.0542	0.1608471
cg21074413	2.7759098	0.9370361	8.223456	0.065388
cg24501177	0.3994979	0.1870073	0.853435	0.0178271
cg02743632	2.4542601	0.7037544	8.558941	0.1589177
cg12598837	0.6013946	0.2531855	1.4285	0.2493111
cg12872489	7.9357665	0.0278623	2260.269	0.4725615
cg18580265	5.5065853	1.6065873	18.87385	0.0066412
cg06017697	0.1322128	0.0350039	0.499379	0.0028445
cg15747712	0.6498583	0.310953	1.358134	0.2517866
cg06193004	1.644368	0.6595176	4.099885	0.2859788
cg05951178	0.699028	0.3180875	1.536181	0.3727575
cg23660356	0.4463646	0.2040182	0.976586	0.0434587
cg18327385	0.5063843	0.2124514	1.206982	0.1246703
cg00265277	0.3288339	0.1017663	1.06255	0.0630871
cg10997718	2.1199563	0.8884008	5.058769	0.0903988
cg01906922	6.42E-22	3.35E-43	1.231454	0.0509813
cg05561555	4.0899527	1.100732	15.1969	0.0354414
cg24447625	2.4777165	0.9578299	6.409362	0.0613293
cg18739887	0.2956644	0.1039456	0.840992	0.0223333
cg16944958	6.4167346	1.2659539	32.52447	0.024785
cg11032188	0.281838	0.1143618	0.694573	0.0059246
cg03918377	5.07E-09	1.81E-17	1.418937	0.0542665
cg23562479	0.6231466	0.2257285	1.72026	0.3612911
cg12691994	0.7602269	0.2581539	2.238761	0.6188554
cg01624637	0.2139934	0.0389096	1.176911	0.0762819
cg27600804	0.3814453	0.1314302	1.107055	0.0762483
cg02514000	0.4443452	0.2058463	0.959175	0.0388159
cg17346345	1.2330583	0.5211706	2.917342	0.6335052
cg23247595	0.0046971	2.34E-06	9.415823	0.166997
cg05184456	0.2210432	0.0439242	1.112373	0.0671316
cg19130189	0.6390931	0.2685165	1.521098	0.3115698
cg13427728	1.3740136	0.726398	2.599007	0.3285551
cg18236211	1.2587769	0.497102	3.187513	0.6273302
cg18677603	1.6431522	0.6925472	3.898578	0.2599248
cg06049177	0.4301605	0.1900877	0.973435	0.0429105
cg15643703	3.3910576	0.0559051	205.6927	0.559887
cg26309261	0.1184316	0.0157188	0.89231	0.0384011
cg12373898	0.1110082	0.0110744	1.112731	0.061604
cg03584094	2.3473476	0.5871567	9.384276	0.2274849
cg17634650	0.500469	0.1543242	1.623007	0.2488361
cg00135433	0.3314631	0.1262786	0.870043	0.0249159
cg09907068	0.6929675	0.3552768	1.351633	0.2819273
cg00732810	0.3646684	0.1271353	1.045996	0.0606114
cg19460713	0.0058135	7.89E-07	42.84097	0.2572326
cg00274203	0.2801229	0.1067256	0.735239	0.0097476
cg26084700	1.5772647	0.3914777	6.354803	0.5215718
cg13700939	0.1157151	0.0325019	0.411975	0.0008725
cg11835209	0.2690797	0.089312	0.810685	0.0196511
cg23792952	0.1256286	0.019225	0.820941	0.0303131
cg04355222	0.2176312	0.057516	0.823482	0.0247034
cg05037544	1.779255	0.9168148	3.452986	0.088524
cg15333426	1.4092092	0.5799681	3.424103	0.4488804
cg15595495	3.0510748	0.5207336	17.87681	0.2162344
cg23083424	0.2308006	0.0626818	0.849831	0.0274799
cg18709589	16.505902	0.022708	11997.72	0.4042668

cg10125399	3.1319516	1.4295241	6.861809	0.0043316
cg02919936	1.7955223	0.9941313	3.242932	0.052325
cg04592560	0.6123639	0.3070917	1.2211	0.163707
cg19391892	1.75E+19	0.000103	2.97E+42	0.104473
cg10676084	0.0601898	0.0120235	0.30131	0.0006268
cg12668482	0.2878995	0.1175685	0.705003	0.0064312
cg16034168	0.3692428	0.1524241	0.894479	0.027315
cg26433208	0.2278927	2.70E-07	192444.9	0.8317923
cg00036299	2.1028004	0.9057567	4.881851	0.0836978
cg10242279	0.950187	0.3980278	2.268322	0.9083709
cg25854527	0.6728697	0.3564107	1.270314	0.2217059
cg21084508	0.3585374	0.0879812	1.461098	0.15244
cg14216196	0.0241738	1.41E-06	413.7288	0.4541717
cg24466377	0.1945202	0.0596499	0.634336	0.0066336
cg24935042	1.0011444	0.4269425	2.3476	0.9979014
cg24535956	0.3819508	0.1349877	1.080738	0.0697314
cg20821314	1.1890588	0.5704271	2.478601	0.6440445
cg03383184	0.8945516	0.3046968	2.626291	0.8393005
cg11539055	0.5028576	0.2707674	0.933886	0.0295157
cg15699226	0.0789559	0.0067241	0.927125	0.0433655
cg09560452	253.53678	0.1490041	431403.6	0.1447327
cg20495019	0.7440575	5.72E-16	9.69E+14	0.9867164
cg05952786	0.2427733	0.0892919	0.660069	0.0055376
cg20784733	5.17E-07	2.02E-13	1.324219	0.0545222
cg25116615	0.7820703	0.4497473	1.35995	0.3838609
cg05253165	2.4582912	1.013596	5.962134	0.0466087
cg06320981	4.1379643	1.2174832	14.06405	0.0228926
cg18179305	92.826814	2.3526557	3662.592	0.0156825
cg10471544	0.1059636	0.0197317	0.569047	0.0088611
cg04164058	1.9935076	0.9799393	4.055427	0.0569055
cg22008490	1.6572858	0.5595793	4.908323	0.3618017
cg05432003	1.3571521	0.5090537	3.618207	0.5415982
cg15838288	0.830695	0.1615742	4.270818	0.8242765
cg00548268	0.2579522	0.0815977	0.815456	0.0210346
cg09251400	1.6942725	0.2658725	10.79675	0.5768495
cg15051239	3.169084	0.5392234	18.62511	0.2017912
cg14370507	0.2922478	0.1332784	0.64083	0.002135
cg02383154	1.4489139	0.6761724	3.104758	0.3402706
cg05483406	0.1047421	0.0253578	0.432644	0.0018227
cg09234599	0.0296452	0.002075	0.42353	0.0095099
cg27545611	2.4623976	0.6859328	8.839644	0.1670093
cg13487726	2.7572536	0.0565854	134.3536	0.6089915
cg15756227	2.3329012	1.0177997	5.347248	0.045322
cg25843873	0.4004777	0.1848819	0.867485	0.0203174
cg27453745	1.5230807	0.7863992	2.949869	0.2122165
cg16535717	2.778253	0.5698542	13.54503	0.2061599
cg26507725	1.624737	0.68352	3.862023	0.2719209
cg22936253	0.4176742	0.1359402	1.283297	0.127401
cg27299725	251688.07	3.95E-28	1.61E+38	0.7469354
cg13626676	1.1298163	0.0519404	24.57596	0.9380848
cg11797228	7435275.4	1582.0632	3.49E+10	0.0002449
cg00231728	1.4965933	0.6819043	3.284613	0.3147419
cg20160695	5.1998378	0.8856184	30.53043	0.0679308
cg27230882	0.3316763	0.1468203	0.749278	0.0079506
cg05593832	0.6945216	0.3684062	1.309317	0.2598028
cg08160063	0.4369763	0.1948693	0.979879	0.0445056
cg22868943	1.903108	0.6770915	5.349085	0.2223106
cg00501711	0.33273	0.1518378	0.729128	0.0059742

cg05816000	0.7846234	0.403324	1.526401	0.4749941
cg10687087	0.6257914	0.2518971	1.554662	0.3126987
cg03356492	3.2535068	1.4644956	7.227954	0.0037707
cg05962382	3.1292456	1.1186608	8.753483	0.029734
cg01414687	0.1923698	0.0758029	0.488189	0.0005223
cg09664812	80.49852	1.916759	3380.713	0.0213831
cg13432616	100005575	6.65E-07	1.50E+22	0.2687337
cg24621599	1.5008951	0.6285318	3.584045	0.3605401
cg15054260	507.1892	2.04E-07	1.26E+12	0.5724997
cg10811945	5.6951901	1.3427855	24.15515	0.0182855
cg15925478	2.1130392	0.9396569	4.751665	0.0703845
cg20168849	1.1745435	0.6130177	2.250428	0.6277295
cg23920016	1.1951354	0.391336	3.649929	0.7543257
cg25688583	0.5266196	0.2384409	1.16309	0.1126818
cg02106466	0.160809	0.0227275	1.137809	0.0671543
cg02801485	71.98993	0.0003376	15349148	0.4945349
cg07121644	1.2932764	0.5017501	3.33346	0.5944725
cg17959580	1.6239505	0.5983226	4.407681	0.341223
cg26267678	0.0462614	0.0049188	0.435089	0.007194
cg10567706	1.4653143	0.6694068	3.207535	0.339149
cg02627216	0.2917641	0.1047464	0.812689	0.0184337
cg23029021	1.7126947	0.8224642	3.566506	0.1505136
cg08488569	0.1517245	0.0563225	0.408723	0.0001918
cg21101086	0.4639746	0.224161	0.960348	0.0385486
cg11195360	0.8651812	0.4426043	1.691214	0.6719541
cg20853148	1.498E+11	9.24E-06	2.43E+27	0.17661
cg15835620	1.0192702	0.483125	2.150399	0.9600357
cg05641535	0.3142574	0.10956	0.901403	0.0313157
cg12581035	1985.2931	0.0331366	1.19E+08	0.1760799
cg12459759	0.3941747	0.1817426	0.854911	0.0184326
cg21528204	2.5575259	1.0270815	6.368471	0.0436569
cg13952159	2.1527214	0.9685257	4.784808	0.0599056
cg22846395	0.0002026	1.10E-10	372.8087	0.2479022
cg20672708	0.4080572	0.1270778	1.310305	0.1320896
cg10196532	0.4115507	0.2053956	0.824623	0.0122881
cg23539494	1.1541801	0.3729775	3.571614	0.8035234
cg25821245	0.2592144	0.0727537	0.923555	0.0372843
cg04282594	0.2671833	0.0522144	1.367189	0.1130825
cg24151204	0.2199989	0.074372	0.650776	0.0062133
cg03116740	0.2172221	0.0644332	0.732315	0.0138008
cg25619459	0.556439	0.2978659	1.039476	0.0659838
cg10108402	2.04E-12	5.66E-31	7369761	0.2169649
cg20656566	3.1787236	0.5388191	18.75265	0.2015687
cg10510775	0.2801178	0.0356197	2.202882	0.2265124
cg11468148	0.2770793	0.1046945	0.733305	0.0097481
cg19722371	6.0207027	0.0621183	583.5457	0.4417387
cg09651522	4.4060867	1.5240157	12.73845	0.0061843
cg06019853	1.9036425	0.9667962	3.748313	0.0625634
cg18115658	3.1113239	0.5587999	17.32344	0.1950947
cg06486622	2.1052257	0.4868998	9.102438	0.3189918
cg16253634	0.1989855	0.052565	0.753263	0.0174474
cg22579265	2.1551593	0.9167013	5.066766	0.0783143
cg00087884	2.0190039	0.6243491	6.529003	0.2406634
cg19556599	0.0055115	8.21E-14	3.7E+08	0.6826136
cg04726869	2.8641291	1.1437727	7.172085	0.0246535
cg17101285	0.2002849	0.0664966	0.603249	0.0042577
cg04308167	2.1599985	0.8650015	5.393741	0.0990736
cg21192370	0.7866063	0.3403346	1.818062	0.5744396

cg27519392	4.5438125	0.9887227	20.88172	0.0517282
cg12261451	2.0257243	0.752971	5.449824	0.1620969
cg00929860	9.4262984	0.1080361	822.4575	0.3251272
cg06971659	0.7446426	0.2862551	1.937057	0.5455234
cg26287741	0.2816281	0.1137608	0.697203	0.0061476
cg17469434	0.8165311	0.2931771	2.274131	0.6981303
cg00266389	5.6232961	1.4780694	21.39376	0.0113055
cg08311343	0.3180505	0.1318911	0.766967	0.0107502
cg15299888	0.3723186	0.1625748	0.85266	0.0194398
cg01692482	2.9591021	0.9828964	8.908655	0.0536955
cg12991125	1.3580033	0.7038256	2.620213	0.3614674
cg03476195	1.4385962	0.5561419	3.721279	0.4532699
cg13301731	0.231976	0.0567668	0.947963	0.0419143
cg01988136	3.3683693	1.2937982	8.76946	0.0128614
cg24650940	0.1626725	0.0608204	0.43509	0.000297
cg09406547	1.8217945	0.6576279	5.046829	0.2485907
cg00564996	3.1838682	0.1653313	61.31336	0.4428568
cg26986541	2.8130239	1.4033497	5.638725	0.0035564
cg27353361	1.4741939	0.6999281	3.104958	0.3071574
cg15883451	514268880	0.0009979	2.65E+20	0.1449024
cg23648082	5.511136	1.1028419	27.54032	0.0375975
cg06019726	0.1596022	0.05068	0.502622	0.0017168
cg05226729	1.0793896	0.4405218	2.644777	0.8673096
cg24527560	0.33067	0.1146351	0.953832	0.0406174
cg26860507	1032856.4	2.54E-08	4.19E+19	0.3864021
cg20335179	0.4733794	0.197102	1.136914	0.0943424
cg12868738	1.145804	0.3745546	3.505142	0.81143
cg18403478	0.3735326	0.1656957	0.842065	0.0175753
cg22977246	0.2968456	0.1090295	0.808197	0.0174696
cg19510698	1.8412814	0.831731	4.076219	0.1321794
cg04036777	2.665029	0.7629373	9.309257	0.1245451
cg12566152	3.3928792	1.1877482	9.691978	0.0225336
cg02617791	2.1329576	0.225946	20.13537	0.5083932
cg03796224	4.9718861	0.5433358	45.49608	0.15564
cg26297530	0.910535	0.3601843	2.301805	0.8429901
cg05338969	1.2012312	0.4148372	3.478368	0.7353728
cg15857470	0.5874822	0.1865671	1.849926	0.3634191
cg05643494	22250.762	11.154336	44386006	0.0098204
cg08178124	1.32E-14	2.90E-45	5.99E+16	0.3748754
cg06879406	1.08E-06	4.05E-27	2.86E+14	0.5668663
cg03016385	0.3822823	0.1288597	1.134099	0.0830681
cg06970884	0.0591293	0.00563	0.621012	0.0184221
cg25394505	1.8806676	0.5006083	7.065226	0.3496174
cg14827643	0.5864906	0.1140893	3.01493	0.5229505
cg03272408	0.1570823	0.0481262	0.512711	0.0021635
cg22754179	1737044.3	61.736955	4.89E+10	0.0059828
cg11525552	0.1087135	0.0038917	3.036886	0.1915092
cg25712316	5.877E+11	7720.7115	4.47E+19	0.0034253
cg03218003	9.59E-46	9.15E-82	1.01E-09	0.0143034
cg08615694	0.2438401	0.0838685	0.708943	0.0095514
cg11123720	1.6027019	0.5893593	4.358383	0.3554248
cg27020668	2.5722821	0.998769	6.62479	0.050299
cg21278889	0.1123619	0.000651	19.3947	0.4055313
cg17239236	0.0916842	0.0139085	0.604376	0.0130167
cg17619020	0.2209138	0.0369982	1.319062	0.0976765
cg15867728	1.4102484	0.6378588	3.117932	0.3957631
cg19777470	2.1619842	0.9060311	5.158957	0.0822855
cg24683222	1.7425179	0.8537659	3.556442	0.1271021

cg08229199	1.3951904	0.654733	2.973054	0.3882694
cg21488132	0.3013879	0.0945845	0.960354	0.0425216
cg24704123	2.26E-05	4.51E-08	0.011327	0.0007448
cg20889476	1938.1481	1.03E-10	3.64E+16	0.6273811
cg08650639	0.1768489	0.0415345	0.753001	0.0190909
cg25918726	3.15E-09	3.27E-16	0.030223	0.0170089
cg03453585	1.3097514	0.6239595	2.749295	0.4756981
cg14685990	3.07E-22	8.22E-39	1.14E-05	0.0109468
cg09850925	1.9156456	0.6970255	5.264797	0.2075841
cg18434712	0.0224011	0.0005313	0.944553	0.046608
cg10937802	3.2335472	0.9914364	10.54614	0.0516902
cg04688590	0.451212	0.1326771	1.534495	0.2025552
cg04329105	1.2999544	0.5867807	2.87992	0.5180304
cg19995899	2.1608189	0.3977296	11.73948	0.3722523
cg11596897	1.7757881	0.6475584	4.869713	0.2645546
cg19129839	1.0287346	0.3612872	2.929235	0.9576824
cg25461389	0.1867435	0.0413379	0.843611	0.0291831
cg12166899	2.3813754	0.3695157	15.34698	0.3613895
cg15526081	1.3517456	0.694688	2.630269	0.3748682
cg00208967	1.1533059	0.6091923	2.183407	0.661387
cg07686441	0.240382	0.090111	0.641248	0.0044057
cg15286618	265.61628	3.9536063	17844.98	0.0093139
cg05037327	0.4600458	0.2190752	0.966071	0.040252
cg13613748	2.81E-11	1.78E-20	0.044341	0.024556
cg16471362	0.239075	0.0760058	0.752006	0.0143882
cg00120147	27.313827	0.7054326	1057.571	0.076243
cg24745316	3.3101389	1.1673028	9.386613	0.0243945
cg03945021	0.9901765	0.422946	2.318143	0.9818527
cg01302019	0.2018288	0.0394195	1.033369	0.0547858
cg01226614	0.401122	0.1691957	0.950963	0.0380674
cg20556675	0.050196	0.0060695	0.415131	0.0055103
cg21122075	3.8927937	1.2304955	12.31524	0.0207258
cg24819342	9.7298652	2.38E-05	3984592	0.7300383
cg24901679	0.0206659	7.88E-05	5.418266	0.1721694
cg22760037	11.144878	0.209057	594.1361	0.2346557
cg10419011	1.1625393	0.1681602	8.036965	0.8786566
cg04443870	0.0766728	0.0129911	0.45252	0.0045772
cg23358564	0.1736016	0.0372576	0.808895	0.0257422
cg10277175	0.2707188	0.1107469	0.661767	0.0041671
cg12119858	0.322956	0.1268373	0.822318	0.0177778
cg12580783	1.4654858	0.565068	3.800691	0.4318568
cg10112407	0.2627968	0.063446	1.088518	0.0653301
cg16874617	7.8376932	0.1957585	313.8022	0.2740981
cg11011640	0.3368979	0.1323417	0.85763	0.0224826
cg23688713	2.523214	0.425517	14.96205	0.3081473
cg06640254	0.4357231	0.1446749	1.312285	0.13972
cg26110883	1.3405994	0.4826667	3.723495	0.5738562
cg02478828	4.284482	0.0078054	2351.81	0.6512053
cg18550003	1.7299023	0.4452997	6.720333	0.4286246
cg06722503	1.0316779	0.2695196	3.949098	0.9636792
cg06301252	0.6272893	0.2567415	1.532638	0.3062341
cg24708056	1.3353021	0.4616227	3.86253	0.5936396
cg21643748	0.3860858	0.141766	1.051467	0.0626334
cg05548393	0.4854964	0.2381321	0.989815	0.0467973
cg18798774	1.3850437	0.2708555	7.082545	0.6956392
cg01042100	0.7605737	0.2963659	1.951886	0.5692573
cg24201034	3.6201109	0.7638668	17.1564	0.1050944
cg25914931	4.94E+37	20697933	1.18E+68	0.0150162

cg09867208	2.7825715	0.6638555	11.66324	0.1616207
cg26735846	0.1954285	0.0417111	0.915638	0.038283
cg04658856	0.4198767	0.1859995	0.947832	0.0367136
cg05424422	1.9512029	0.7639389	4.983635	0.1623672
cg16248756	1.3276142	0.5759799	3.060106	0.5059719
cg07906726	4.7458597	1.446384	15.57206	0.0102067
cg05044743	1.0982315	0.4710473	2.560492	0.8282444
cg00243574	2.40E-09	3.17E-14	0.000183	0.0005374
cg18633990	2.528512	0.4488872	14.24272	0.2929002
cg15989436	2.5146046	0.5811382	10.88078	0.2172923
cg18593945	0.7798244	0.3064339	1.984526	0.6017942
cg07187607	0.1135379	0.0203929	0.632125	0.0130079
cg17859634	7.2047894	1.4555451	35.66292	0.0155214
cg13649441	0.3739292	0.1623529	0.861229	0.0208367
cg11492886	0.3491897	0.1754355	0.695033	0.002737
cg01898628	0.4466076	0.2036375	0.979478	0.04425
cg04717018	0.273262	0.0383113	1.94909	0.1955944
cg23152772	1.1934831	0.5318536	2.678184	0.6679901
cg08578245	0.3583464	0.1444741	0.888825	0.0268117
cg22499719	1.303E+10	1.66E-14	1.02E+34	0.4067376
cg26538556	1.0693992	0.5649755	2.024185	0.836711
cg26429140	0.5480705	0.2489927	1.206386	0.1352121
cg04996728	2.5211256	0.532786	11.92988	0.2436072
cg20348196	1.614707	0.8613622	3.026925	0.1350495
cg19997502	1.1721698	0.665824	2.063581	0.5819792
cg09125402	5.225E+12	3.03E-08	9.00E+32	0.2180239
cg02707176	4.1285178	1.1233402	15.1732	0.032753
cg08957605	5.9274235	0.6405573	54.84966	0.1169733
cg13395086	2.4335575	0.7215715	8.207367	0.1516145
cg20856545	0.8931613	0.4035009	1.977039	0.7804742
cg13375984	0.0065382	2.00E-05	2.13569	0.0885573
cg08608532	0.698148	0.0483011	10.09108	0.7920324
cg00381131	0.4754419	0.26442	0.854871	0.0129993
cg25584862	0.1186348	0.0322375	0.436578	0.0013427
cg00163859	4.2187373	0.6376789	27.9102	0.1353707
cg12291247	0.3997656	0.2121322	0.753363	0.0045691
cg10820084	3.2566462	0.6769556	15.66683	0.1407052
cg14151096	7.2591389	1.17E-13	4.51E+14	0.9026398
cg03648690	4.1418011	0.6393813	26.82987	0.1360173
cg23894854	1.3672276	0.5466425	3.419623	0.5036731
cg20502376	3.2369472	1.3542082	7.737235	0.008243
cg25344964	1.4406022	0.3757108	5.523756	0.5944681
cg26816688	0.5604325	0.2522456	1.245154	0.1551284
cg12387859	0.1944464	0.0074275	5.090475	0.325582
cg15542741	0.0342318	0.0002876	4.074051	0.1663822
cg22923006	0.2469665	0.1202749	0.507109	0.0001391
cg27483469	1.18019	0.1402499	9.931192	0.8788325
cg01188235	0.4505195	0.1800081	1.127548	0.0884754
cg24525176	2.0778812	0.6849083	6.303895	0.1965035
cg22173241	1.572E+14	2.09E-12	1.18E+40	0.2822393
cg24622589	0.2436553	0.0867631	0.684253	0.0073585
cg13677120	3844.3258	5.17E-19	2.86E+25	0.7480239
cg08481112	0.1743831	0.0275691	1.103027	0.0634862
cg02660089	12.571675	0.1552653	1017.916	0.2588361
cg13423554	0.1647666	0.0602184	0.450826	0.000446
cg02692052	1.6997383	0.6773469	4.265333	0.2584495
cg23682824	71267.357	2.8672549	1.77E+09	0.030468
cg06060054	0.0855743	0.0156231	0.468728	0.0046079

cg16510485	0.484682	0.0676245	3.47384	0.4710651
cg02872868	2.5975009	0.8892569	7.587246	0.0809224
cg25385940	0.1985852	0.0581943	0.677662	0.0098432
cg20336016	0.7360441	0.3528659	1.535317	0.4139278
cg05938889	4.4328376	0.9215495	21.32284	0.0631661
cg20275507	0.7257598	0.3941086	1.336503	0.3035262
cg18740359	0.4999827	0.2089936	1.196126	0.1193381
cg06829791	0.1153008	0.0221584	0.599965	0.0102562
cg14147196	2.9660467	1.3292427	6.61838	0.0079316
cg17053963	1.9383045	0.8089487	4.644329	0.137699
cg21728792	2.5354342	0.8208092	7.831816	0.1059201
cg11699334	0.1957133	0.0875873	0.437321	7.00E-05
cg03532040	0.160464	0.04802	0.536207	0.0029543
cg00804995	8.3766395	0.769047	91.24032	0.0810832
cg08314370	0.7444135	0.0941802	5.883951	0.7796141
cg08383399	0.070009	0.0092584	0.529386	0.0099906
cg11576274	2.4857724	1.0237052	6.03598	0.044249
cg20486877	3.0532782	0.1261136	73.9215	0.4923953
cg26424079	1.5657135	0.3921302	6.251645	0.5256291
cg01891252	1.8411769	0.7196154	4.710756	0.2028442
cg24593272	1.6974577	0.82956	3.473362	0.1474908
cg04914221	0.482033	0.2184168	1.063818	0.0707948
cg07328888	0.2857221	0.0034099	23.94153	0.5792681
cg00065088	0.7256925	0.22202	2.371992	0.5956958
cg12955789	0.7745185	0.2653268	2.260906	0.6401591
cg19389468	0.2105995	0.0807271	0.549409	0.0014518
cg00773653	1.1831864	0.5386327	2.599044	0.6752504
cg10339481	2.3046214	0.7435949	7.142706	0.1479971
cg14174928	0.2755713	0.0632247	1.201106	0.0861616
cg14021133	1.6067903	0.7863276	3.283333	0.1933689
cg05347215	0.4839831	0.1917556	1.221553	0.1244632
cg11542224	0.2201028	0.0303689	1.595227	0.134177
cg13278353	2.9712825	0.8756801	10.0819	0.0806381
cg21184415	4.420243	1.5479477	12.62223	0.0055011
cg24633978	0.105842	0.0149645	0.748608	0.0244457
cg04654530	3.3698247	1.1764456	9.652566	0.02366
cg14269109	0.9397854	0.4339185	2.035397	0.8748449
cg19773466	2.0171488	0.3759707	10.82236	0.4129849
cg22036248	1.9104395	0.7790557	4.684876	0.1572364
cg00245075	0.2487032	0.0900696	0.686728	0.007249
cg24524470	0.3264035	0.1027867	1.036509	0.057546
cg12352601	0.0100004	2.14E-05	4.674369	0.1420263
cg27490380	0.0073407	0.000164	0.328521	0.0112791
cg08400124	2.8256723	0.8670357	9.208875	0.0848401
cg06839377	0.2175547	0.0747133	0.633489	0.005156
cg15742245	0.5278001	0.2612201	1.06643	0.0749549
cg02680314	2.2910489	0.8982024	5.843788	0.0826977
cg24196612	2.4450151	0.6881789	8.686839	0.166907
cg19872814	0.0008023	4.25E-16	1.52E+09	0.6211347
cg09953406	2.4055832	0.8514693	6.796288	0.0976154
cg25212025	1.6948087	0.5508248	5.214683	0.3575624
cg09818637	3.9295969	0.861686	17.92037	0.0771141
cg17155018	1.8612655	0.2512416	13.78876	0.543167
cg21937462	0.4035463	0.1815627	0.896933	0.0259545
cg11179816	0.2486617	0.0784542	0.788137	0.0180556
cg18194850	0.5224586	0.165118	1.653139	0.2693124
cg01572891	2.0427408	0.9129344	4.570744	0.0821604
cg15646919	0.1127843	0.025008	0.50865	0.0045175

cg11118198	0.6803062	0.2340167	1.977708	0.4792599
cg25103726	1.7057475	0.8655893	3.36138	0.1228536
cg20545410	2.0443167	0.4356618	9.592832	0.3646395
cg13027458	1.8658691	0.8949268	3.890226	0.0961466
cg26823666	2.2029736	0.8910362	5.446572	0.0872372
cg00709387	0.0999537	0.0002332	42.84698	0.4564032
cg00872677	0.6631811	0.0699841	6.284417	0.7203739
cg23111300	1.3589075	0.2512628	7.349394	0.7217614
cg22796355	0.406522	0.1689004	0.978448	0.0445813
cg14795708	3.21E-16	1.77E-27	5.80E-05	0.0069848
cg00270625	0.5567819	0.2999556	1.033507	0.0635204
cg04187814	0.2883906	0.096129	0.865182	0.0265333
cg16178415	0.0004078	3.99E-07	0.417043	0.0272926
cg06804564	1.8060701	0.938953	3.473964	0.0765221
cg03151469	3.4267884	1.0626876	11.05017	0.039232
cg11820558	4.25E-29	1.23E-82	1.47E+25	0.2989657
cg16513459	0.537448	0.2109492	1.369288	0.1931584
cg14958663	3.5269723	1.1016129	11.29211	0.0337569
cg05825053	0.4234711	0.2065027	0.868404	0.0190253
cg26459819	0.4817744	0.2360434	0.983322	0.0448383
cg01751992	0.1890789	0.0451527	0.791776	0.0226376
cg01809371	5.3578934	0.7199213	39.87522	0.1011963
cg03313451	3.9333719	0.6257329	24.72527	0.1442594
cg26648488	0.0826807	0.0004435	15.4149	0.3500386
cg15072680	7.6013787	1.3697648	42.18313	0.0203506
cg05464016	0.0288708	0.002916	0.285844	0.002441
cg03573179	3.2128982	0.5430135	19.01005	0.1981734
cg23084416	0.2369567	0.0931713	0.602637	0.0024999
cg22530668	2.9493976	0.1176694	73.92698	0.5105048
cg02539798	0.5473273	0.1241355	2.413227	0.4259213
cg19577080	1.4867001	0.2508319	8.811785	0.66228
cg26625897	2.6052534	0.5442119	12.47188	0.230738
cg09192732	0.0466502	0.0003199	6.803011	0.2279246
cg00458681	1.154501	0.6095062	2.186807	0.6593433
cg09371059	0.3060412	0.1216627	0.769843	0.011879
cg25312054	0.6194937	0.2754042	1.393488	0.2469716
cg00799539	1.6410446	0.9320793	2.889269	0.0861153
cg25266895	1.7213815	0.7623756	3.886738	0.1911992
cg04470060	0.4516429	0.200229	1.01874	0.0554635
cg26178529	9.551E+14	0.0022048	4.14E+32	0.0959663
cg13104880	1.4241093	0.5869127	3.455518	0.434378
cg09680055	1.2418813	0.561872	2.744876	0.5924155
cg25149218	1.6876198	0.7204834	3.952986	0.2281813
cg06621373	1.2096486	0.5152586	2.839836	0.662029
cg22153799	2.4886882	0.9339926	6.631283	0.068243
cg01802593	1.2630587	0.2372126	6.725265	0.784313
cg25355213	1.0436625	0.4413252	2.468093	0.9224749
cg02909446	1.7807477	0.4412261	7.186934	0.4175995
cg14505616	5.5332827	1.0808249	28.32764	0.0400486
cg07721400	0.6100131	0.249729	1.490079	0.2780492
cg23971381	0.485183	0.1554846	1.513993	0.2128998
cg16063587	2.5864173	0.8439189	7.926774	0.0963146
cg06137032	408.47621	0.4119298	405051.6	0.087634
cg02109484	5.086708	1.1355506	22.78595	0.0334937
cg03942471	2.6802747	0.8908617	8.063959	0.0793744
cg02564302	3.1029283	0.6474827	14.87015	0.1566873
cg14985891	0.693243	0.3467417	1.386005	0.2999732
cg19538115	0.2798749	0.1134038	0.690717	0.0057315

cg23393100	0.4658691	0.2240729	0.968587	0.0408123
cg06224587	0.2481929	0.0942274	0.653734	0.0048
cg13601936	0.2875519	0.1334144	0.619769	0.0014678
cg20906291	6.0448842	0.9268522	39.42443	0.0600307
cg14629571	0.7033301	0.3455695	1.431472	0.3317278
cg09033641	0.273882	0.1008933	0.743472	0.0110302
cg05952379	0.2343872	0.094978	0.578422	0.0016452
cg22240998	0.1929737	0.0534603	0.69657	0.0120023
cg13878066	5.570199	0.0678664	457.1791	0.4450481
cg25068347	1.781324	0.4935689	6.428921	0.377947
cg01712159	0.3179582	0.1261868	0.801173	0.0150946
cg03608224	0.2003837	0.064648	0.621111	0.0053516
cg00864083	0.0117122	0.0003697	0.371067	0.0116613
cg13569101	2.2940248	1.1904732	4.420553	0.0131046
cg08680007	0.6650907	0.3462032	1.277705	0.2208429
cg02721176	1.82761	0.9323644	3.58246	0.0790842
cg18736448	2.4498235	1.0996524	5.457757	0.0283507
cg08227227	0.3805971	0.0637987	2.270489	0.289098
cg05905124	0.8751987	0.0864325	8.862093	0.9101446
cg16880396	1.5549037	0.4294673	5.629591	0.5013146
cg03333757	0.1788204	0.0422322	0.757165	0.0194006
cg26734287	1.4413678	0.7898866	2.630176	0.2335157
cg14337027	1.7028607	0.7279346	3.98351	0.2195851
cg23184653	0.767151	0.3890895	1.512559	0.4441032
cg16560272	3.0761351	1.1526676	8.209311	0.0248546
cg26647524	0.3104904	0.0923707	1.043668	0.0586423
cg18841796	0.2837639	0.1018655	0.790474	0.0159621
cg23883738	114.54841	1.1346715	11564	0.0440489
cg15941057	1.11E-08	1.04E-38	1.19E+22	0.6037012
cg11183632	0.0837532	0.0054457	1.288088	0.0753358
cg18982286	0.2724231	0.0956149	0.77618	0.0149224
cg16851385	0.5465614	0.1284295	2.326017	0.4136137
cg22325059	32.460997	0.0341977	30812.52	0.3197793
cg01567783	1.9179937	0.7349238	5.005553	0.1832924
cg05556020	1.8557792	0.6445702	5.342966	0.2518002
cg02541582	0.0133777	0.0003576	0.500388	0.0195619
cg09787089	0.1315277	0.0496306	0.348566	4.51E-05
cg13765206	0.0596739	0.0096847	0.367689	0.0023783
cg13512948	1.6486463	0.2838654	9.575082	0.5775223
cg03110787	0.218867	0.0780909	0.613423	0.0038602
cg06082821	0.2218261	0.0120361	4.088262	0.3111305
cg01442620	0.3893333	0.1766184	0.858237	0.0193339
cg21992250	1.3219233	0.4570407	3.823469	0.6065296
cg01281426	1.4633045	0.1862691	11.49552	0.7173605
cg09075215	1.6775243	0.8194095	3.434287	0.1570305
cg18204158	1.5734485	0.6782357	3.650266	0.2911113
cg03149567	0.1738873	0.0599785	0.504127	0.0012768
cg25087423	5.5321965	0.7575704	40.39915	0.0917435
cg11338178	0.3188518	0.1066618	0.953167	0.0407748
cg24932241	1.8804294	0.7389854	4.784959	0.185101
cg09681167	2.2362624	1.0995407	4.548144	0.0262868
cg04965934	0.9720127	0.5219319	1.810215	0.9287071
cg09448677	0.1176045	0.0066086	2.092846	0.1450658
cg15138339	0.1946344	0.0557413	0.679614	0.0103066
cg06151625	27213324	178.75572	4.14E+12	0.0049274
cg07121050	5.39E-09	3.78E-19	76.7543	0.1104685
cg06127256	0.3630342	0.14222	0.92669	0.0340735
cg04660829	1.9588237	0.1851636	20.72216	0.5764026

cg14147105	0.9991009	0.0002063	4839.142	0.9998342
cg16019612	0.1633541	0.0498637	0.53515	0.0027659
cg03548963	1.3709686	0.3978437	4.724356	0.6171911
cg06820822	1.0894021	0.5260214	2.256176	0.8176862
cg06934092	0.292073	0.0824961	1.034069	0.0563878
cg01156249	1.7012506	0.4617218	6.268393	0.4245427
cg03109018	2.4023656	0.5166864	11.16995	0.2636487
cg18407136	0.6422206	0.3080764	1.338783	0.2374005
cg25805368	4.8671271	0.415879	56.9611	0.2073438
cg02142483	0.3068026	0.1347118	0.698735	0.0048987
cg07355157	0.3154725	0.1060739	0.938241	0.0380237
cg19550904	2.4552891	0.6376386	9.454328	0.1916179
cg08646805	0.5235856	0.2499341	1.096856	0.086356
cg14747903	0.4230078	0.1916988	0.93342	0.0331233
cg11874426	0.11436	0.0258981	0.504987	0.0042151
cg08597832	0.3637371	0.1517794	0.87169	0.0233348
cg13656408	1.4259015	0.5619501	3.618106	0.4551692
cg14971718	0.4468214	0.2084657	0.957708	0.0383537
cg17343167	1.6620443	0.5870539	4.705516	0.3386551
cg03681481	1.0499167	0.5380571	2.048714	0.8864371
cg06058681	2.0517145	0.6944558	6.061627	0.1935107
cg00880079	0.0004167	1.03E-06	0.168891	0.0110701
cg06847006	1.7926483	0.7190583	4.469162	0.2104468
cg02293775	0.1699584	0.0464951	0.621266	0.0073687
cg03002479	22.50493	0.428457	1182.083	0.1234123
cg08234168	0.9650784	0.4671908	1.993567	0.9234949
cg05509696	0.0602557	5.74E-05	63.2855	0.4286917
cg06659380	1.8116052	0.5182735	6.332397	0.352051
cg16317553	0.101929	0.0004368	23.78714	0.4117578
cg18801459	2.864433	0.7147961	11.47877	0.1373078
cg05103581	4.436809	0.5990932	32.85845	0.144716
cg03458048	0.0545085	0.0068511	0.433678	0.0059686
cg03570994	0.4720632	0.1883439	1.183175	0.1093376
cg01223793	0.163202	0.0301544	0.883284	0.0353774
cg14943749	0.0068189	0.0001219	0.381492	0.0151284
cg03323793	3.1226291	0.826631	11.79585	0.0931153
cg03031183	0.9925708	0.4584628	2.148913	0.9849038
cg12149299	0.3551354	0.1416376	0.890449	0.027289
cg02685484	4.3055814	0.7001153	26.47854	0.1151916
cg15908367	2.2427525	0.8159918	6.164203	0.1174045
cg14765414	2.3002103	0.945396	5.596562	0.0663297
cg27271486	2.377863	1.103803	5.122502	0.0269535
cg11362183	0.4643521	0.2132983	1.010898	0.0532786
cg01360413	1.2410857	0.528396	2.915037	0.620065
cg13534999	2.9023054	0.1686947	49.93268	0.4629493
cg11841741	1.7954121	0.8197252	3.932421	0.1434617
cg08218971	0.0500424	0.0092399	0.271025	0.0005115
cg06185204	120.46553	2.1206124	6843.28	0.0200893
cg11657808	0.1062753	0.0126614	0.892034	0.0389022
cg27642027	2.2712036	0.9350366	5.516753	0.0700445
cg02233835	1.0383496	0.3661169	2.944879	0.9435921
cg04568799	2.355504	1.1311567	4.905066	0.0220639
cg04850999	1.8528215	0.5037973	6.814144	0.3533268
cg24004483	3.2157224	0.7956939	12.99604	0.1011649
cg19620017	6.2787842	0.6211107	63.47198	0.1195936
cg19108736	7.8646146	1.5877968	38.95471	0.0115264
cg01942023	3.4285233	0.625252	18.80006	0.1558691
cg09140851	0.3403768	0.1446321	0.801042	0.0135872

cg09697245	0.3646505	0.1542503	0.862041	0.0215533
cg23349242	0.096437	0.0253614	0.366703	0.000599
cg09326832	0.282096	0.1271278	0.62597	0.0018589
cg00989765	0.2396528	0.049551	1.159076	0.0756676
cg02676175	0.2463981	0.08257	0.735279	0.0120312
cg00813993	1.7405281	0.5214726	5.809391	0.3674887
cg26461905	0.8464713	0.3115879	2.299555	0.7437566
cg13641043	4.849174	0.8064502	29.15802	0.0845382
cg19610529	3.1345472	1.2096282	8.122649	0.0186875
cg14529080	3.377E+14	2319.2649	4.92E+25	0.0107467
cg10720080	2.8375375	0.7889282	10.20577	0.1102776
cg27391267	0.091291	0.021792	0.382436	0.0010564
cg18055610	2.2883616	1.0475401	4.998949	0.0378513
cg26148904	1.2034786	0.5026306	2.881561	0.6775769
cg03673190	0.4744559	0.2188332	1.028676	0.0589779
cg15093769	0.7601146	0.3672726	1.573148	0.4598499
cg03017946	1.1347589	0.5190102	2.481026	0.7514326
cg14521746	0.0331246	0.0028561	0.384168	0.0064294
cg18833140	0.2517257	0.1024446	0.618537	0.0026359
cg12954234	0.2176759	0.0985621	0.48074	0.0001621
cg00082140	1.1571395	0.4172804	3.208806	0.7791214
cg26314330	2.1233717	0.8378299	5.381412	0.112503
cg03625512	0.1102508	0.0040793	2.979754	0.1899028
cg07266910	0.3961966	0.1749311	0.897334	0.026441
cg04972770	1.5609958	0.5774069	4.220088	0.3801512
cg02758499	0.8621733	0.1944806	3.822194	0.8452449
cg11770080	1.3069002	0.6484355	2.634014	0.4541471
cg12377874	0.6353903	0.2929906	1.377931	0.2508571
cg00108873	0.3216137	0.1042445	0.992238	0.0484363
cg00454719	2.4309829	0.9514441	6.211272	0.0634582
cg14210311	0.1889524	0.0002321	153.8542	0.6260674
cg02709068	0.1029927	0.0315918	0.335768	0.0001633
cg14994639	0.7683302	0.3909898	1.509838	0.4445063
cg07588439	0.3917997	0.1476681	1.039541	0.0598262
cg25274735	2.3264249	1.1328473	4.777566	0.0214651
cg16907514	0.4316327	0.1169184	1.593478	0.2073828
cg10734044	0.2996638	0.1415531	0.63438	0.0016366
cg04176169	0.2897627	0.1006051	0.834575	0.0217328
cg08608663	1.2408032	0.5065383	3.03944	0.6369202
cg10305797	1.916947	0.8487236	4.329661	0.1174901
cg04310460	0.1849646	0.0655828	0.52166	0.0014224
cg09096787	0.45187	0.1295459	1.576171	0.2127007
cg02250548	0.1451096	0.036136	0.582709	0.0065011
cg11200222	1.2796186	0.5664064	2.890899	0.5532185
cg15930643	0.4451318	0.1719566	1.152281	0.09534
cg00409658	2.0567521	0.4332053	9.764954	0.364211
cg15985157	1.410942	0.5421658	3.671861	0.480523
cg08477158	0.3992311	0.181178	0.879718	0.0227334
cg24008901	0.7651198	0.3782108	1.547836	0.4564309
cg10655041	0.4363425	0.1784686	1.066825	0.0690413
cg01196388	201.97528	0.1524456	267597.2	0.1478519
cg26372517	0.1955843	0.0824152	0.464152	0.000215
cg05947740	0.9686075	0.2366596	3.964346	0.9646169
cg01988891	1.3610493	0.7312036	2.533433	0.3308518
cg15701412	0.2569344	0.1137321	0.580446	0.0010825
cg01143764	1.9023655	0.4575837	7.908924	0.3763769
cg06518251	0.0622085	0.0077758	0.497687	0.0088539
cg17369830	0.0101309	2.03E-06	50.46818	0.2904202

cg21011133	0.9070984	0.4768143	1.725677	0.766351
cg13224852	0.3518271	0.0767865	1.612033	0.1785894
cg04589649	0.9373796	0.3221031	2.727948	0.905552
cg15829654	2.1854362	1.014191	4.709302	0.0459406
cg09321817	2.1814334	1.1580298	4.109265	0.0157756
cg07924154	1.7156529	0.6763793	4.351796	0.2556899
cg17765892	1.7695724	0.7493012	4.179076	0.1930161
cg15284327	2.01E-21	1.03E-59	3.92E+17	0.2894149
cg09059319	1.7863892	0.7180381	4.444313	0.2121514
cg05599536	0.4350631	0.057454	3.29446	0.4203979
cg03265268	1.1808397	0.5355357	2.603715	0.6803185
cg00639984	0.042663	0.0048654	0.374094	0.0044055
cg01717649	0.2348098	0.0835919	0.659581	0.0059654
cg12991306	1.031126	0.4574962	2.324	0.9410687
cg13283153	2.2899327	1.0136103	5.173381	0.0463191
cg04185542	0.7515677	0.3230163	1.748686	0.5074232
cg16711291	0.4602132	0.2220226	0.95394	0.03691
cg25840057	4.9079157	0.5869315	41.03995	0.1420504
cg20170533	2.0469618	0.9082322	4.613416	0.0840236
cg07935784	1.8363841	0.9145824	3.687264	0.0874661
cg15948851	2.659163	0.987898	7.157771	0.0528844
cg06776548	2.2907363	0.0606955	86.45572	0.6545544
cg13894747	6.8701294	0.2002797	235.6638	0.2853176
cg02194684	2.0776028	0.697243	6.190716	0.1893145
cg10958087	0.4839941	0.2237947	1.04672	0.0651919
cg06819546	1.9158936	0.7825563	4.690587	0.1546646
cg18978493	2.3130401	0.7717676	6.932339	0.1342999
cg03373115	0.1319786	0.0194583	0.895161	0.0381394
cg08843248	4.7595124	1.0629122	21.31216	0.0413771
cg15521745	1.6962533	0.3921153	7.337829	0.4794814
cg05888037	1.5069556	0.551895	4.114759	0.4236108
cg17610800	2.8135722	1.2231126	6.472166	0.0149411
cg19114576	5.7937807	1.1868811	28.28244	0.0298735
cg21450547	0.6767627	0.1339295	3.419768	0.6366649
cg00373467	0.0001657	3.77E-07	0.072872	0.0050575
cg18677834	0.0634493	0.0092948	0.433126	0.0048966
cg20986665	1.2451416	0.5913705	2.621669	0.563841
cg06697439	103.81931	1.3734268	7847.851	0.0354005
cg04688889	1.9613244	0.2017867	19.06366	0.5615424
cg03854595	1.1248998	0.3091494	4.093165	0.8582568
cg20899213	4.449545	1.0584866	18.70449	0.0415954
cg06821107	0.0069007	2.10E-06	22.65679	0.2283634
cg03735370	1.8068132	0.9310471	3.506347	0.0803325
cg22283754	3.9722362	0.110696	142.5405	0.4501968
cg22543892	1.3055663	0.4910389	3.471219	0.5930467
cg25059792	3.37E-14	2.47E-24	0.00046	0.0091761
cg20122645	1.4478432	0.0369612	56.71489	0.8432419
cg16639766	41531.603	5.21E-15	3.31E+23	0.6320108
cg08997713	0.0105378	3.86E-06	28.7774	0.2594192
cg01446515	2.1619885	0.8364205	5.58833	0.1115406
cg09565111	1.1083954	0.2419516	5.077628	0.8945625
cg15871371	0.623706	0.2129874	1.826442	0.3891589
cg00345314	0.3721764	0.1121127	1.2355	0.1064147
cg07206208	0.0071906	8.32E-05	0.621626	0.0300897
cg09446583	3.9351522	0.9121702	16.97646	0.0662522
cg03714676	0.5989898	0.2512154	1.428212	0.2476741
cg00868766	0.1520184	0.0369113	0.626084	0.0090977
cg17790159	2.7168918	0.6617591	11.15436	0.1654325

cg23370946	2.2633501	0.9394843	5.452729	0.0686349
cg24342283	0.2153657	0.0715512	0.648241	0.0063141
cg16448058	1.4614335	0.7553237	2.827646	0.2598738
cg05740106	1.7320122	0.7363205	4.074131	0.2081735
cg25463470	0.0504443	0.0021268	1.196468	0.0644681
cg16692258	1.7603679	0.5433739	5.703063	0.345713
cg12311002	1.8300391	0.7239275	4.626214	0.2015307
cg13640091	0.057244	0.0025745	1.272811	0.0706797
cg13598109	0.5241922	0.2682125	1.024477	0.0588608
cg11469778	2.2945185	1.0236829	5.143014	0.043716
cg24687395	5.344939	1.4295377	19.98434	0.0127366
cg15027050	0.7815629	0.4147242	1.472884	0.4458846
cg09120987	0.6181033	0.3001509	1.272865	0.1917774
cg20141578	1.9212046	0.6494371	5.683425	0.2380247
cg14283454	0.1395963	0.0299426	0.650816	0.0121825
cg02433278	0.0764588	0.0082479	0.708777	0.0236398
cg09484032	2.6581686	1.0011054	7.058059	0.0497414
cg19815340	2.0482725	0.7366465	5.695296	0.1693885
cg10085741	5.3097536	0.930566	30.29714	0.060248
cg13270707	18.74043	0.1114287	3151.826	0.2623835
cg26963090	0.198251	0.072904	0.539112	0.0015222
cg02965712	8.4975393	1.3094044	55.14582	0.0249307
cg25442239	94.201551	0.3313545	26780.78	0.1148429
cg23840823	0.0270266	5.09E-05	14.3621	0.2594205
cg11762968	2.0872916	0.8715836	4.998701	0.0986364
cg23125328	2.938E+16	0.2122413	4.07E+33	0.0597014
cg06728793	7.4179463	1.6576227	33.19569	0.0087679
cg22399133	2.6276202	1.0586127	6.522109	0.0372729
cg00189989	2.4893813	0.5610699	11.045	0.2302381
cg10369169	4.388629	0.1100106	175.0747	0.4316339
cg08511818	2.263859	0.6548995	7.825717	0.1966632
cg17153794	2.4101513	0.6199179	9.370321	0.2041685
cg14425199	0.3964074	0.1801516	0.872259	0.0214699
cg08073507	58.316227	1.5913814	2137	0.0269101
cg14103147	0.2501552	0.078195	0.800277	0.0195185
cg04101755	1.8418096	0.6501859	5.217374	0.2502971
cg08643472	2.444E+11	0.0025766	2.32E+25	0.1102829
cg04142955	0.107686	0.0268436	0.431994	0.0016656
cg13151707	0.1851047	0.0303983	1.127162	0.0672357
cg08508325	0.1061918	0.0113067	0.997344	0.0497286
cg25884883	0.2625268	0.1214356	0.567546	0.000674
cg00072689	0.4111527	0.0983121	1.719489	0.2234202
cg10958126	0.6614632	0.2660577	1.644506	0.3737635
cg11857548	0.6229453	0.3006536	1.290724	0.2028897
cg09776463	3.2595691	0.7524217	14.12079	0.114182
cg19421368	2.0875438	0.7399067	5.889714	0.164302
cg06309882	2.1732949	0.755505	6.251727	0.1498974
cg04652536	3.8018092	0.7788531	18.55774	0.0987418
cg20754979	0.0396047	0.0041974	0.373693	0.0048098
cg04043919	0.289555	0.0202084	4.148878	0.3615258
cg19824990	1.6665326	0.3316906	8.373258	0.5351854
cg23845646	0.4393376	0.206725	0.933692	0.0324896
cg10256242	1.6758067	0.7628593	3.681319	0.1985019
cg12778228	0.407146	0.0985209	1.682565	0.2145192
cg01509464	1.7647325	0.8197473	3.799074	0.1465289
cg07791476	0.1029085	0.0064081	1.652615	0.1084247
cg07910075	0.3355374	0.1358773	0.828581	0.0179008
cg15992935	0.213151	0.0340231	1.335366	0.0987269

cg06526388	0.3139542	0.092923	1.060741	0.0621766
cg15923688	6.8573211	0.9460951	49.70203	0.0567628
cg23830590	0.0456479	0.0005088	4.09524	0.1784778
cg12659494	1.2895648	0.622104	2.67315	0.4941271
cg04494136	2.1201124	0.2444921	18.38455	0.4953256
cg08712808	0.7956133	0.4089827	1.547744	0.5006712
cg06980565	4.1807809	1.1418038	15.30817	0.0307557
cg03895435	1.5957399	0.7162658	3.555085	0.2528446
cg11744006	1.6849266	0.7967341	3.563269	0.1721562
cg06548892	0.4922618	0.2442976	0.991912	0.0474029
cg19460792	0.4258844	0.1977978	0.916984	0.0291504
cg08894020	2.1445294	0.6579751	6.989635	0.2056627
cg23649708	1.2524691	0.6300692	2.489693	0.5207411
cg13453904	2.1384684	1.1682025	3.914602	0.0137424
cg12025938	0.6732237	0.006282	72.14698	0.86823
cg23920079	0.446691	0.2014224	0.990619	0.0473498
cg11646240	0.142768	0.0265466	0.767809	0.0233424
cg15211734	14.251575	0.2124258	956.1333	0.2156897
cg18835815	5.7277721	0.6600806	49.70207	0.1133838
cg24612991	4.82E-06	5.66E-11	0.409951	0.0345212
cg23510415	0.1586629	0.0428619	0.587327	0.005835
cg05886626	1.7659251	0.9421215	3.310073	0.076067
cg03998835	2.0705718	1.1553837	3.710687	0.0144772
cg08279189	0.3622397	0.1725834	0.760314	0.0072672
cg00475235	0.6835505	0.2662872	1.754652	0.4289551
cg23200506	0.8933509	0.1941398	4.110831	0.8848617
cg16344442	0.1717655	0.052558	0.56135	0.0035498
cg02423817	4.1374504	1.1418013	14.99254	0.0306308
cg24976089	1.548055	0.5508822	4.350248	0.4071306
cg07437908	0.6492151	0.2811462	1.49915	0.311678
cg13339881	1.8800773	0.6985302	5.060183	0.2113961
cg03470754	0.1244304	0.0332559	0.46557	0.0019647
cg08513626	0.3269196	0.1213509	0.880722	0.0270248
cg00798876	0.1229067	0.0170672	0.885095	0.0374211
cg03721657	0.3100574	0.1421941	0.676087	0.003239
cg21241410	1.8225004	0.6151686	5.399346	0.2787362
cg11198912	182259.42	0.463153	7.17E+10	0.0653488
cg22752052	1.2563103	0.6180086	2.553873	0.5284358
cg16991768	1.8857941	0.3596202	9.888819	0.4530696
cg15802263	0.1284181	0.0378063	0.436203	0.0010028
cg03593358	0.0414212	0.000307	5.588888	0.2032559
cg07242214	2.199838	0.3204461	15.10172	0.4224896
cg22388608	0.8084284	0.3565311	1.833098	0.6106591
cg08299859	0.2035422	0.0279985	1.479702	0.1157614
cg06832898	1.3802947	0.7044636	2.704488	0.3476511
cg17940256	0.3568496	0.1699663	0.749217	0.0064708
cg03147051	2.1676477	0.6450641	7.284077	0.2109226
cg17945233	0.7063194	0.3133772	1.59197	0.4017222
cg04565201	0.3479575	0.1660202	0.729275	0.0051712
cg26343183	0.1783978	0.0318373	0.999638	0.0499519
cg19872463	1.8174051	0.6395242	5.164717	0.2622537
cg11801842	0.42733	0.163138	1.119365	0.0835491
cg00590620	2.7474711	1.2451786	6.062261	0.0123137
cg08937273	0.0248996	0.000686	0.903717	0.0438826
cg00206058	39454045	0.000441	3.53E+18	0.1740084
cg01792616	3.9784263	0.5794868	27.31361	0.1600588
cg15813090	0.0415457	0.0014264	1.210041	0.0644384
cg21373145	0.3652831	0.1691743	0.788723	0.0103386

cg14241894	0.6076126	0.323056	1.142815	0.1221572
cg19632760	0.1456165	0.0401723	0.52783	0.0033629
cg19657875	0.0002353	2.32E-07	0.238898	0.0180158
cg10683055	0.1418822	0.0474865	0.423922	0.000471
cg15687000	0.5030119	0.0763298	3.314837	0.4750665
cg03945400	0.208285	0.0601298	0.721483	0.0133254
cg21081729	0.5694456	0.2698562	1.201633	0.1394406
cg19016664	0.5245989	0.2354286	1.168949	0.1145429
cg12942165	0.3176003	5.05E-10	2E+08	0.9116465
cg24959663	1.90776	0.6696647	5.434881	0.2265574
cg10055231	1.9196018	0.4208551	8.755677	0.3996695
cg12559925	0.5423231	0.2545062	1.155627	0.1129129
cg20255723	2.9513564	1.2861699	6.772437	0.0106546
cg22193702	1.4445058	0.6020558	3.465787	0.4101548
cg19414016	0.5170408	0.2695396	0.991807	0.0471759
cg07911361	1.5554583	0.6391583	3.78537	0.3302776
cg03535793	1.1811673	0.5843646	2.387476	0.6428433
cg14343548	1.8957502	0.7901429	4.548378	0.1520136
cg01983682	4.7841119	1.5213686	15.04417	0.0074107
cg13269555	1.1477784	0.547548	2.405991	0.7151217
cg11595059	0.5422973	0.2496149	1.17816	0.1221517
cg15177211	0.1545706	0.0501443	0.476466	0.0011512
cg06225154	1.6395457	0.6268389	4.288359	0.3135209
cg25286482	2.163909	1.1698545	4.002637	0.0138977
cg04505435	0.192633	0.0136044	2.72761	0.2232499
cg22282877	3.98E-21	6.84E-33	2.32E-09	0.0006778
cg17309077	2.2927135	0.9683067	5.428585	0.0591967
cg05569109	3.2973332	0.8523285	12.75612	0.0839016
cg02069715	0.3402909	0.1121199	1.032804	0.0570429
cg04273417	0.000814	4.38E-09	151.1519	0.2504612
cg16885600	0.2484265	0.0872193	0.707593	0.0091171
cg08099293	2.2317822	1.1155744	4.464832	0.0232626
cg25185947	0.416908	0.1461782	1.189044	0.1018075
cg04131890	2.1210151	0.8340097	5.394068	0.1143753
cg02946204	2.492187	1.2587663	4.934193	0.0087844
cg23484234	0.2218869	0.047432	1.037986	0.0557989
cg06285293	0.3127489	0.1048245	0.933102	0.0371498
cg07029781	4.65E-22	1.68E-34	1.29E-09	0.0007784
cg24592576	1.9277407	0.9154818	4.059266	0.0840705
cg08144858	0.0332792	0.0022541	0.491335	0.0132375
cg03523129	0.5376412	0.1663937	1.737194	0.2997148
cg03473518	1.9376179	0.8643658	4.343489	0.1082621
cg13532359	0.3038488	0.0651082	1.41801	0.1296193
cg15241074	0.3326697	0.0920216	1.202643	0.0932403
cg04902929	0.2822867	0.0807037	0.987387	0.0477224
cg23176873	11.800529	0.5945972	234.1963	0.1054561
cg04029421	0.2033035	0.0757673	0.545517	0.0015597
cg21480725	1.2366614	0.5046083	3.030729	0.6423262
cg01162920	0.0878721	0.006443	1.198437	0.0681249
cg10495164	1.3470957	0.2251759	8.058886	0.7440791
cg20948431	2.8791795	0.454739	18.22952	0.2614066
cg15835825	0.0313757	0.0030266	0.325266	0.003717
cg03221483	0.2768363	0.0942953	0.812748	0.0194246
cg09777775	2.5822341	0.9460475	7.048201	0.0640682
cg02272547	0.1349377	0.0177624	1.025095	0.0528668
cg04173182	0.4871902	0.2105912	1.127085	0.0928795
cg05682742	2.165379	0.9324009	5.028809	0.0723113
cg00419321	1.8720721	0.9926067	3.530758	0.0527404

cg01266850	1.8526594	0.7498107	4.577618	0.1815242
cg20668321	0.4276187	0.1813995	1.008039	0.0521778
cg14884445	0.3599524	0.1436695	0.901832	0.0292233
cg14007549	0.9222379	0.4018302	2.116622	0.8485393
cg16114380	0.4530862	0.1913876	1.072625	0.0717794
cg27431150	2.860567	1.1755464	6.960885	0.0205354
cg08092930	0.0002182	9.84E-08	0.483898	0.0319824
cg02491878	1.6797948	0.6947793	4.061305	0.2495285
cg02853324	0.9140814	0.3963629	2.10803	0.8331064
cg04730866	0.5921887	0.2898512	1.209888	0.150635
cg04949346	0.4504447	0.0171664	11.81963	0.6323562
cg13697968	1.3262437	0.4009403	4.386993	0.643656
cg18873763	831294006	12.821876	5.39E+16	0.0252245
cg23677272	2.192021	0.8773798	5.476483	0.0929673
cg13800828	1.5615075	0.0001013	24075.03	0.9278285
cg14633268	4.3253492	0.7134261	26.22366	0.1112227
cg14174099	0.0111671	0.0003735	0.333902	0.009523
cg21130221	1.9701124	0.886645	4.377561	0.0959889
cg11472521	1.3384852	0.5907623	3.032595	0.484776
cg14789039	2.8658219	1.0088729	8.140703	0.0480928
cg12167057	2.3466089	1.2370508	4.451372	0.0090227
cg00485312	1.1774524	0.461181	3.006183	0.7326692
cg17152524	1.4892379	0.5406978	4.101791	0.4410356
cg03000305	0.160921	0.0165498	1.564703	0.1154446
cg19778253	0.4871227	0.2378781	0.997522	0.0492121
cg16170767	1.674089	0.5933718	4.723133	0.3302131
cg24932585	5.4899781	1.3382969	22.52106	0.0180504
cg21500300	1.5369025	0.47876	4.933723	0.4701652
cg07298473	0.1710842	0.0478128	0.612176	0.0066393
cg20006618	1.2185264	0.210477	7.054484	0.8254068
cg18879389	0.3107038	0.0996356	0.9689	0.0439664
cg14334441	1.3506642	0.6478772	2.815802	0.4225777
cg22118017	5.3340282	0.0269886	1054.219	0.5348107
cg15590671	0.3522823	0.172008	0.721494	0.0043388
cg17330252	0.2902564	0.1164915	0.723218	0.0079158
cg17106450	0.0069787	2.58E-19	1.89E+14	0.7970436
cg17661220	1.9342977	0.4260944	8.780936	0.3926983
cg11536196	1.4281302	0.7470256	2.730236	0.2811047
cg09623377	0.1121289	0.0302547	0.415569	0.0010613
cg24630195	1.7616936	0.7524388	4.124674	0.1920123
cg02473254	1.2365335	0.5060321	3.021577	0.6414014
cg06442073	1.8152756	0.9312959	3.538323	0.0799565
cg11528995	0.0005332	3.09E-07	0.920757	0.0475159
cg02936012	0.1498703	0.0491965	0.456559	0.0008394
cg03830181	1.7094694	0.5170372	5.651983	0.3795053
cg04348709	0.4329716	0.1581761	1.185163	0.103248
cg01699524	3.66E-05	4.95E-13	2711.66	0.2692197
cg02195151	0.2442362	0.0776703	0.768007	0.0158857
cg21625464	1.5622291	0.5941144	4.107895	0.3657858
cg16061668	2.172672	0.2927434	16.12505	0.4480026
cg27645858	0.4338427	0.1955033	0.962743	0.0400413
cg27319530	2.0452962	0.5384986	7.768333	0.2933052
cg13602461	0.9361086	0.4543579	1.928654	0.8579217
cg04431133	0.2525363	0.0847507	0.752497	0.0134956
cg12950829	0.2840389	0.1151905	0.700388	0.0062697
cg06357748	5.928438	0.4063261	86.49796	0.1931157
cg23548640	1.5392206	0.5725997	4.13762	0.3926498
cg12436612	3.7404526	0.3686358	37.95341	0.2644858

cg25520146	0.5409742	0.2232384	1.310944	0.1736901
cg03150210	6.73E-10	6.07E-23	7460.596	0.1681748
cg13798842	0.0851111	0.0293094	0.247152	5.90E-06
cg19490598	0.4217791	0.1905504	0.933599	0.0332172
cg21449397	0.483644	0.2208167	1.059302	0.0693788
cg03691170	2.1694069	0.8644779	5.444125	0.0989954
cg19378892	0.9298302	0.4964026	1.7417	0.8202685
cg03229780	1.8865987	0.9230553	3.855949	0.0817823
cg06017249	0.4267342	0.1888037	0.964505	0.0406749
cg12486287	0.1040683	0.0298774	0.362489	0.0003799
cg14034968	1.6152139	0.819599	3.183162	0.1659874
cg10585872	41.116356	0.3152348	5362.843	0.1348014
cg18302225	0.1352581	0.0407963	0.448442	0.0010703
cg25739875	3.1894143	1.0136273	10.03561	0.0473555
cg00066449	778.7196	1.6518287	367110.8	0.0340262
cg10048215	0.2056012	0.099248	0.425922	2.07E-05
cg24312039	0.0003024	9.75E-10	93.79272	0.2090839
cg21039380	2.4407197	1.1499073	5.180516	0.0201403
cg11826961	0.3989814	0.1477726	1.077237	0.0698085
cg13448704	0.1751563	0.0548382	0.559459	0.0032802
cg25807071	1.44E-13	1.72E-31	120302.1	0.1601992
cg05251000	0.0169425	0.0006963	0.412224	0.0122747
cg02998591	10.160506	0.2030071	508.5332	0.2455199
cg10993442	2.4622896	0.4348688	13.94184	0.3083776
cg01077274	2.7375814	0.392932	19.0729	0.3092425
cg03173975	2.73E-07	3.59E-13	0.207984	0.0287259
cg19638477	1.441896	0.7981389	2.60489	0.2252204
cg27294813	1.5303751	0.739956	3.165118	0.2511025
cg03087855	0.0047289	6.68E-05	0.334745	0.0137583
cg11990630	1.4098305	0.5449966	3.647036	0.4787671
cg07804711	3.3613512	0.0378198	298.7506	0.5964368
cg23697796	0.3134819	0.0924333	1.063154	0.062648
cg00944421	0.4195111	0.044003	3.999489	0.4502078
cg10134799	0.4223783	0.1385335	1.2878	0.129705
cg02034689	1.877543	0.6347683	5.553471	0.2548926
cg21515956	1.3712642	0.6521264	2.883438	0.4050737
cg09945482	0.3512971	0.1546881	0.797797	0.0124274
cg16521451	0.6570682	0.2860669	1.509223	0.3222478
cg14906905	197.5523	5.850142	6671.105	0.0032434
cg11002258	0.3666344	0.1561026	0.861106	0.0212665
cg22502856	1.4228723	0.0819693	24.69908	0.808632
cg22372849	1.4351917	0.2727602	7.551597	0.6697675
cg22266001	1.5367379	0.7624343	3.097399	0.2295631
cg24706992	3.6515842	0.4537098	29.38898	0.2235192
cg24672173	1.8485515	0.4173549	8.187619	0.418424
cg09975804	0.4305652	0.1414188	1.310904	0.1379685
cg22198132	0.0543426	0.0030563	0.966237	0.0473279
cg17885542	1.5135154	0.7548391	3.034725	0.2429709
cg00318320	0.1537694	0.0474364	0.498458	0.0018068
cg12776966	1.1323108	0.4515294	2.839522	0.7910835
cg09463917	2.4024374	0.6486732	8.897709	0.1895042
cg14854112	2.1443315	0.2845246	16.16084	0.4591527
cg01775613	399078094	3.61E-18	4.41E+34	0.517443
cg24194053	0.7855174	0.328097	1.880656	0.5878374
cg21992400	0.5114397	0.2537504	1.030818	0.0607811
cg13207180	0.5708006	0.0709633	4.591292	0.5981098
cg14949292	0.2720007	0.1241164	0.596089	0.0011444
cg05186320	1.4511092	0.6763261	3.113465	0.3391174

cg15032239	0.0008204	1.96E-05	0.034278	0.0001905
cg00735744	2.30E-12	1.72E-22	0.030631	0.0242582
cg18383585	0.4863024	0.1249485	1.892701	0.2984426
cg00889627	1.1711699	0.4829326	2.840229	0.7266589
cg01477255	0.1053954	0.0020513	5.415107	0.2629243
cg10143823	0.7605348	0.4054424	1.426622	0.3937177
cg03756485	0.5238412	0.1087677	2.522897	0.4201563
cg16611852	1.10E-20	1.18E-45	103625.9	0.1172737
cg21494132	4.5783305	0.1103498	189.9515	0.4234911
cg11481351	7.4638602	0.6003674	92.79186	0.1180094
cg10650821	1.3882079	0.4327841	4.452847	0.5812291
cg06918917	0.7283638	0.2655912	1.997483	0.5380432
cg01031400	2.3803271	0.8609804	6.58082	0.0946286
cg08659212	3.7531259	0.9935087	14.17799	0.0511332
cg02285725	0.7321064	0.3566611	1.50277	0.395398
cg00379619	0.2402529	0.1094799	0.527233	0.0003762
cg06520675	2.2637294	1.0156653	5.045432	0.0457193
cg13929988	0.3038085	0.0954028	0.967472	0.0438083
cg14526579	0.5010612	0.1922704	1.305777	0.1573549
cg26361807	1.723E+19	1.0785552	2.75E+38	0.0496095
cg04646186	1.6074919	0.7913625	3.265292	0.189251
cg14328230	13953.096	1.01E-15	1.93E+23	0.671291
cg17036624	0.4304705	0.1986606	0.932771	0.0326499
cg24349832	0.1661104	0.0175847	1.569133	0.1171729
cg07716927	1.5972361	0.7372463	3.460395	0.2351652
cg02875089	0.3210315	0.1085387	0.949535	0.0400188
cg18449021	0.8470847	0.4246455	1.689768	0.6376222
cg22944062	1.3043775	0.3793952	4.484507	0.6732116
cg10987774	2.4827419	0.6323124	9.748358	0.1925346
cg11868900	4.3493104	1.6522603	11.44886	0.0029126
cg11883418	0.3416458	0.1566963	0.744892	0.006923
cg06936155	3.2235334	0.9243141	11.24203	0.0662869
cg04432599	0.3208761	0.1125017	0.915199	0.0335303
cg15420468	1.1650869	0.4576001	2.966406	0.7486307
cg04701182	1.08E+24	1.81E-07	6.45E+54	0.1258693
cg13430450	0.2026163	0.085401	0.480713	0.0002927
cg00693344	0.2637454	0.0818048	0.850337	0.0256548
cg20125559	1.71E-08	3.65E-14	0.008005	0.0072607
cg27141120	11.99496	0.8508813	169.0942	0.0657172
cg24506574	2.64E-13	5.48E-25	0.127336	0.0348481
cg00278517	2.4480818	1.1327928	5.290557	0.0227811
cg17634797	0.286058	0.130458	0.627245	0.0017824
cg24630209	20.814983	0.6127898	707.0345	0.0914698
cg06444161	347.79869	0.0648508	1865266	0.1816882
cg10171609	1.4648927	0.6049087	3.547495	0.3975363
cg00927495	1.7245939	0.8134499	3.656309	0.155187
cg18149207	2.040959	0.4801917	8.674689	0.3338765
cg10142566	12.207131	0.7072743	210.6878	0.0851332
cg27321913	1.012714	0.4643515	2.208649	0.9746663
cg01692110	3.4680291	1.0631935	11.31236	0.039251
cg01065697	0.4465667	0.1687202	1.181968	0.1045202
cg12072290	39.371828	0.6024428	2573.092	0.0850085
cg16321474	1.677595	0.8530685	3.299061	0.1337699
cg24173049	1.3393695	0.690072	2.599599	0.387811
cg00059089	0.5825903	0.2703946	1.255245	0.1677385
cg04559323	4.4737026	0.9602174	20.84321	0.0563579
cg14209854	2.3005287	0.3293795	16.06789	0.4008426
cg22309568	0.3665587	0.140474	0.956513	0.0402847

cg19621008	3.8094315	1.2843651	11.29879	0.0159034
cg10122865	2.4251678	0.9860041	5.964923	0.0536975
cg16985320	1.44E-08	2.94E-18	70.22028	0.1126397
cg11221635	15657.462	3.5418695	69216582	0.0241174
cg04636564	1.5251073	0.7375348	3.153685	0.2548521
cg23587449	2.6239307	1.0484355	6.566939	0.0393013
cg21519787	0.4534667	0.2364635	0.869615	0.0172896
cg09862303	0.807093	0.3653753	1.782822	0.5960943
cg11291978	0.0057131	1.86E-05	1.75774	0.0772277
cg11991942	0.3077487	0.147957	0.640113	0.0016113
cg00187120	0.1523621	0.0376032	0.617347	0.0083987
cg00966522	4.2945163	1.1940915	15.44511	0.025642
cg02838877	0.4280466	0.1996231	0.917849	0.029241
cg25994988	0.1106391	0.0289434	0.42293	0.0012919
cg00692611	1.564126	0.8730173	2.802339	0.1327041
cg02613713	85.67138	0.0952161	77083.48	0.1997121
cg13315706	1.5831236	0.624275	4.014706	0.3332478
cg13574150	1.4905972	0.6327016	3.511735	0.3612474
cg00856375	0.1883794	0.0050068	7.087719	0.3671136
cg23088110	2.8202749	0.4738544	16.78564	0.2545779
cg27525647	5.47E-21	6.17E-51	4.86E+09	0.1848249
cg08861270	206.22435	9.2106815	4617.3	0.0007797
cg09382002	2.7162826	0.8146215	9.057201	0.1038902
cg17338254	2.1633841	0.5292752	8.842717	0.2827125
cg07788369	3.3031711	1.2801277	8.523321	0.0134891
cg04598300	1.2664097	0.4909617	3.266637	0.6251768
cg23726178	9.8545385	0.6960073	139.5272	0.0906524
cg19767622	3.8259106	1.4402555	10.16319	0.0071057
cg11284582	6.1861488	1.5665721	24.42814	0.0093072
cg00626856	1.1121561	0.5395376	2.292502	0.773323
cg22705228	35.804897	0.0008495	1509116	0.5101817
cg13364107	1.21E+20	14.639512	9.97E+38	0.0374591
cg16503259	0.6464234	0.3185104	1.31193	0.2269868
cg19956836	1.3019895	0.4979556	3.404273	0.590484
cg10118167	1.1291816	0.4118953	3.095571	0.8133395
cg08232654	0.4949756	0.1743886	1.404913	0.186424
cg22790835	0.2637622	0.0881556	0.789179	0.0171542
cg22640209	1.1665505	0.4541212	2.996645	0.7489417
cg10885151	1.7071153	0.6824638	4.270179	0.2529308
cg13051700	1.0964214	0.5508045	2.182516	0.7932655
cg04734712	1.1243855	0.5558488	2.274436	0.7443019
cg13899420	2.1358958	1.0856701	4.20206	0.0279462
cg07444131	2.92E-21	2.59E-41	0.33054	0.0447546
cg10124993	0.4319203	0.2017282	0.924784	0.0306744
cg18873914	0.0959665	0.0157703	0.58398	0.0109669
cg04103993	0.1094144	0.026133	0.458099	0.0024577
cg04176384	1.03E-11	2.60E-29	4084560	0.2210864
cg25972787	0.2790836	0.0670872	1.160992	0.0793065
cg26926805	4.61E-05	3.56E-11	59.70321	0.1643841
cg27199872	1.4522402	0.5217324	4.042305	0.475016
cg06996940	2.7780329	1.215687	6.348235	0.0153862
cg15462925	1.59E-17	2.44E-50	1.04E+16	0.3157305
cg13397818	1.9132874	0.545423	6.711614	0.3109316
cg22267234	1.3286073	0.0037413	471.8092	0.9244494
cg02756056	1.4967845	0.7143534	3.136212	0.2852188
cg06775570	0.0630001	0.0118368	0.335312	0.0011915
cg16477492	0.4011649	0.1964056	0.819392	0.0121895
cg14308479	2.2473936	1.0672162	4.732666	0.0330749

cg17586345	0.3156709	0.1064293	0.936284	0.0376497
cg25967612	2.8285509	0.8392182	9.533516	0.0935001
cg00000321	1.5198726	0.6567653	3.517257	0.328135
cg02153855	0.5143083	0.0960247	2.754637	0.4374161
cg06686742	0.2395666	0.0848387	0.676486	0.0069778
cg06013378	0.37394	0.1633333	0.856109	0.0199345
cg21057587	1.089589	0.39278	3.022568	0.8690863
cg10557907	0.4818211	0.1949209	1.191004	0.1137871
cg01541398	1.2850512	0.5904268	2.796886	0.5273494
cg21825944	3.0435981	0.9583316	9.666267	0.0590558
cg00338529	0.514947	0.2403837	1.103113	0.0877315
cg25593309	0.1843826	0.0424799	0.800308	0.0239844
cg26940178	1.3306464	0.6905868	2.563935	0.3932972
cg08644678	0.7557436	0.0893849	6.389765	0.7970837
cg00034755	2.7713399	0.70054	10.96344	0.1462973
cg12905836	2.2509529	0.5308068	9.545448	0.271018
cg03962821	0.2080875	0.0656829	0.659234	0.0076261
cg03072102	1.179E+14	1.15E-06	1.21E+34	0.1681629
cg20430047	2.6856617	1.2448694	5.794004	0.0117927
cg01862418	1.1987198	0.5708254	2.517283	0.6320642
cg13227473	15.231425	9.13E-14	2.54E+15	0.8705243
cg22085702	1.7380458	0.5712291	5.288251	0.3302364
cg06027843	0.0206056	0.0003955	1.073586	0.0542593
cg20969194	0.0038901	6.29E-05	0.240435	0.0083556
cg13643240	2.1088951	0.1657339	26.83481	0.5653121
cg08863953	2.7430523	0.83768	8.982351	0.0954534
cg23860321	21585.413	0.0199836	2.33E+10	0.1591483
cg19699140	1.0821447	0.5323563	2.199724	0.8273383
cg11364273	0.7014589	0.3791799	1.297655	0.2585666
cg10754705	0.04291	0.0033258	0.553631	0.0158176
cg07343445	3.1372431	1.1064673	8.895241	0.0315367
cg02311932	2.1861795	1.0263579	4.656641	0.0426215
cg03038262	3.4851248	1.4559676	8.342283	0.0050545
cg01613010	1.317864	0.5870702	2.958361	0.5034914
cg23972735	0.525682	0.2005636	1.377825	0.1908631
cg23918490	0.1622172	0.0623771	0.42186	0.0001915
cg17518825	1.39306	0.143045	13.56647	0.7752923
cg15174682	2.5777741	0.3907366	17.00613	0.3252511
cg07103093	1.6456098	0.8262097	3.277657	0.1565077
cg14482116	0.6471453	0.2750167	1.522806	0.3188928
cg07429038	1.4616894	0.5547131	3.851606	0.4425636
cg10855395	1.5538701	0.6570692	3.67467	0.31555
cg13877974	1.8626211	0.6897785	5.029669	0.2197457
cg00729699	1.6389591	0.4692048	5.724978	0.4388169
cg07209009	3.4592917	1.1700253	10.22773	0.0248408
cg25189764	13.027204	0.7712261	220.0497	0.0751004
cg02022733	2.3639698	0.8813938	6.340359	0.0874224
cg06374249	2.5515003	0.7993909	8.143893	0.1136862
cg24116317	0.3313359	0.1684159	0.651859	0.0013771
cg11735605	968.38365	0.011701	80144457	0.2340198
cg10628699	0.6153152	0.1941767	1.949837	0.4092379
cg07828606	4.1483567	0.9088067	18.93567	0.0662796
cg09587503	6.07E-08	3.83E-15	0.961694	0.0494627
cg16461423	3.0976906	1.1317581	8.478567	0.0277435
cg11199011	6.308819	0.3864876	102.9818	0.1960958
cg15657704	1.8183254	0.7461272	4.431292	0.1883121
cg15653855	0.1694994	0.0466429	0.615958	0.0070174
cg23629913	0.3147665	0.0016994	58.30059	0.6643687

cg15326513	1.6655854	0.6352529	4.36704	0.2995645
cg23184070	0.0451278	0.0093359	0.218138	0.0001162
cg11905061	2.9212459	1.1100406	7.687717	0.029899
cg04787888	2.0999147	0.982906	4.486331	0.0554353
cg05127450	1.1210537	0.4946847	2.54053	0.7842699
cg24772001	3.1235739	0.9188472	10.61843	0.0680922
cg08478685	3.4954225	0.9993334	12.22613	0.0501221
cg10476085	0.1160419	0.0224402	0.600071	0.0101945
cg04882739	0.5432707	0.2511967	1.174948	0.1210664
cg02345908	1.7035858	0.8598841	3.375111	0.1267091
cg25723866	112.86212	0.6606862	19279.74	0.0715553
cg02375313	0.3277729	0.1385911	0.775195	0.0110927
cg13952002	0.4609999	0.1258469	1.688726	0.2424151
cg14873488	0.0542715	0.0073729	0.39949	0.0042247
cg21120413	2.7983756	0.7828902	10.00256	0.1133408
cg27225658	0.226833	0.0898508	0.572652	0.0016904
cg09310186	1.4037428	0.3797595	5.188795	0.6111488
cg27048140	0.2640654	0.0696889	1.000597	0.0501027
cg04484334	0.3073043	0.1277053	0.739483	0.0084486
cg03993926	9.27E-29	5.47E-61	15727.87	0.0882406
cg15392489	8.82E-12	1.18E-21	0.0661	0.028226
cg22786811	2.5937108	0.6801219	9.891367	0.1628554
cg20820767	0.3473872	0.1430281	0.843735	0.0195302
cg24749672	4.1655935	1.3255476	13.09057	0.0145913
cg11565042	14.264856	2.1650265	93.98782	0.005728
cg12162996	0.3184146	0.1120341	0.904973	0.0317678
cg26551092	0.7395162	0.360435	1.517289	0.4105399
cg24932525	3.8033648	1.0948856	13.21196	0.0354969
cg10338787	1.0305682	0.4810619	2.207763	0.9382569
cg13441730	0.6332723	0.1148512	3.491769	0.5999474
cg21840153	0.0831745	0.0131034	0.527953	0.0083547
cg26645315	5.78E+22	3859374.9	8.67E+38	0.005815
cg13698224	1.8884855	0.8075799	4.41613	0.1424091
cg26112706	0.5503845	0.2303855	1.314853	0.1789747
cg13949137	0.0875736	0.0213629	0.358994	0.0007166
cg06004033	0.1541247	0.04822	0.492626	0.0016095
cg18106507	3.4172999	0.3827569	30.51007	0.2712572
cg26972389	0.3113842	0.1376379	0.704458	0.0050944
cg01525538	2.499974	0.8155899	7.663005	0.108872
cg25626472	0.4394502	0.2011328	0.960144	0.0392116
cg09480047	1.5331479	0.67122	3.501895	0.3105869
cg14968732	1.6322029	0.6514693	4.08935	0.2957914
cg09881017	7.5160099	0.2269717	248.8875	0.2586742
cg10367023	3.3797332	1.1490867	9.940587	0.0269367
cg08448711	0.2158811	0.0653562	0.713087	0.0119155
cg00234625	1.3371855	0.4966719	3.600093	0.5652742
cg13883553	0.6867169	0.1392943	3.385494	0.6442721
cg05333368	1.7451893	0.4138491	7.359411	0.4482094
cg06438312	1.5158274	0.4418899	5.199786	0.5083606
cg09059250	0.6435861	0.3204444	1.292589	0.2154825
cg27482328	0.2042421	0.0656892	0.635033	0.00606
cg11381564	1.9030123	0.5954392	6.08199	0.2777462
cg03057712	0.0502208	0.0038466	0.655672	0.0224916
cg01231009	0.3677182	0.134826	1.002898	0.0506644
cg21859053	1.940411	1.0012349	3.760551	0.0495742
cg06358792	0.2937981	0.0678014	1.27309	0.1015823
cg01489926	2.577363	0.8513388	7.802769	0.0938968
cg19303748	0.2253374	0.0449004	1.130878	0.0702142

cg14611683	0.3539704	0.1125006	1.113728	0.0757679
cg17224732	440.22066	0.0066147	29297306	0.28269
cg15057440	14455.463	20.861448	10016582	0.0041013
cg27313492	0.17934	0.0521845	0.61633	0.0063652
cg15995714	14.120151	0.4329262	460.5372	0.1364607
cg04972745	2.3452084	1.0730936	5.125371	0.0326131
cg21091197	2.6040446	0.6837457	9.917501	0.1606896
cg04959837	1.2744411	0.3085028	5.264782	0.7375728
cg13300756	0.0761029	0.0165262	0.350454	0.0009475
cg14023350	1.8509341	0.3367693	10.17301	0.4788486
cg16511841	63.784548	0.4796606	8481.973	0.0958112
cg13850871	2.7489359	0.4983605	15.16302	0.2457928
cg06826155	1.558495	0.6561782	3.701596	0.3147256
cg07348922	0.0025407	1.48E-05	0.434959	0.0227721
cg08623277	1.5606677	0.6603706	3.688359	0.3104173
cg02852557	1.6563094	0.7206539	3.806766	0.2346714
cg26688315	0.6288698	0.2496256	1.584282	0.3251616
cg19090761	0.5378727	0.2573652	1.124111	0.0991704
cg10185013	0.2955168	0.048951	1.784032	0.1838778
cg19377014	0.1320792	0.0242441	0.719552	0.0192586
cg07061298	0.658604	0.2936443	1.477159	0.310888
cg24409649	8.432E+10	8.83E-06	8.05E+26	0.1802176
cg01100912	1.1808692	0.4992978	2.792826	0.705032
cg08733172	3.891561	0.7534586	20.09964	0.104794
cg04553793	0.5999098	0.2936719	1.225489	0.1609055
cg16612699	0.7220392	0.3465947	1.504179	0.3844496
cg07233761	0.1540716	0.0231363	1.026009	0.0531843
cg03595348	0.2808082	0.0857874	0.919171	0.0357928
cg03353765	1.8944935	0.7960823	4.508461	0.1486197
cg02114341	0.3094744	0.0735667	1.301872	0.1095818
cg18819515	0.4425125	0.1818807	1.076625	0.0723022
cg23154270	0.4721004	0.1736391	1.283575	0.1413546
cg21587469	1.3681251	0.5716015	3.2746	0.4814938
cg06623625	0.7495111	0.3375735	1.664132	0.4786363
cg08697665	0.3192609	0.1119487	0.910484	0.0327322
cg17475304	3.9581	0.4695545	33.36473	0.2059044
cg21616088	4.08E-05	4.16E-08	0.040025	0.0040319
cg02234613	1.7332976	0.0234624	128.0482	0.8021496
cg12256803	0.8958829	0.2711963	2.959503	0.8568929
cg17801295	2.4689466	0.7984098	7.634797	0.116624
cg11783050	1.1843104	0.5321192	2.635859	0.6785731
cg17441062	1.1514265	0.5672246	2.337316	0.6962885
cg12732998	1.5785461	0.7854853	3.172317	0.1998678
cg20971158	1.1981542	0.4875129	2.944688	0.6935534
cg12194055	0.6239061	0.3013445	1.29174	0.2038956
cg25749165	0.0597185	0.0031044	1.148774	0.0617582
cg26435178	2.1000046	0.7934425	5.558083	0.1351638
cg01813033	0.0041943	0.0001783	0.098684	0.0006809
cg01581781	1.2214751	0.5078198	2.938053	0.655054
cg07239938	0.5570512	0.1704055	1.820986	0.3329608
cg18766586	0.9198356	0.2526259	3.349211	0.8991516
cg03134083	0.9873451	0.5022174	1.941092	0.9705441
cg10944379	0.0283542	0.0004577	1.756433	0.0905687
cg15545878	0.2633805	0.0655895	1.057628	0.0599758
cg13985437	0.1590485	0.035958	0.703498	0.0153693
cg03173570	0.282313	0.1067221	0.746805	0.0108286
cg10057126	1.1630573	0.5904283	2.291053	0.6623375
cg15895543	2180.6774	1.6910906	2812004	0.0354013

cg14234426	1.4591753	0.6788232	3.136594	0.3331508
cg02013841	0.4646907	0.2126252	1.015578	0.0547048
cg02467858	0.5009108	0.2094678	1.197853	0.1201544
cg01003599	0.504783	0.2476949	1.028708	0.0598303
cg10937205	1.9032714	0.9449642	3.833417	0.0716233
cg20631044	0.5286434	0.1457106	1.917938	0.3323054
cg12658012	0.3369828	0.121802	0.932311	0.036175
cg21341928	1.9546004	0.9536593	4.006108	0.067194
cg24376434	1.5125755	0.5662847	4.040167	0.4090708
cg10257110	0.540514	0.219283	1.332321	0.181348
cg04421971	1.9725068	0.9913024	3.92492	0.0529805
cg06156640	0.3866339	0.1860578	0.803437	0.0108832
cg02485642	0.218279	0.0867528	0.549212	0.0012254
cg24353217	1.3508062	0.5181248	3.521695	0.5385229
cg04460816	0.3553068	0.1347358	0.936967	0.0364775
cg10846615	1.7688606	0.9843156	3.178724	0.0565073
cg08194377	0.5857293	0.2842195	1.207091	0.1471089
cg01479473	0.3746837	0.1555662	0.902432	0.028606
cg16052975	2.3714116	0.803405	6.999699	0.1179141
cg08070725	0.301184	0.102231	0.887321	0.029494
cg00832924	0.3129661	0.133391	0.734291	0.0075902
cg08897132	0.0001142	8.92E-41	1.46E+32	0.8305459
cg06492708	0.0084215	2.38E-05	2.982865	0.110702
cg23020514	0.5382969	0.2572029	1.126595	0.100253
cg00047079	13.665242	0.7035296	265.4314	0.0840542
cg02574073	0.6942936	0.3214466	1.499607	0.3530747
cg18595258	0.2935292	0.1227353	0.701993	0.0058637
cg09682183	1.0802739	0.5261161	2.218126	0.8333924
cg09535750	5.521086	0.0114702	2657.531	0.5877026
cg24116779	2.856892	0.7713784	10.58084	0.1160914
cg00326127	2.0270134	0.8791403	4.673638	0.0973686
cg12640109	0.3962046	0.1014067	1.548004	0.1830182
cg07010222	3.3586197	1.1472769	9.832261	0.0270594
cg10970045	9.874777	0.9566715	101.9276	0.0545087
cg19717640	0.5313344	0.2381131	1.18564	0.1225507
cg03182620	0.2776006	0.1285672	0.599392	0.0011014
cg19508437	1.1523786	0.5284302	2.51306	0.7214423
cg01471572	0.4905102	0.1747755	1.376625	0.1760927
cg09603795	0.5483417	0.2550962	1.178687	0.1238281
cg18832247	1.7225695	0.8554245	3.468741	0.12783
cg06139614	0.5306571	0.1039956	2.707778	0.44605
cg01529552	4.5465346	0.1036064	199.5144	0.4325144
cg25188032	0.5029584	0.2165295	1.16828	0.1099855
cg16435860	4.27E-12	1.12E-25	162.2911	0.1008058
cg19022525	0.3998782	0.179424	0.891199	0.0249826
cg23532713	1.37E-12	2.40E-25	7.844522	0.0683746
cg08122114	1.6042085	0.6730264	3.82375	0.2862104
cg27527345	0.7209286	0.3805869	1.365623	0.3154171
cg16955101	10344.32	26.049108	4107817	0.0024644
cg04150100	0.239702	0.1066168	0.538912	0.0005492
cg19620724	2.813535	1.2157999	6.510923	0.0156741
cg13894250	2.0830964	0.1565227	27.72308	0.5784289
cg03011594	4.9097901	0.9669949	24.92882	0.0549238
cg03999941	0.3549168	0.1231373	1.022971	0.055122
cg13699934	0.599671	0.2491435	1.443366	0.2538345
cg25371329	0.069779	0.0014735	3.30445	0.1761555
cg24113274	0.6525314	0.0173914	24.48324	0.8174536
cg01816768	1.0617649	0.4574685	2.464311	0.8890462

cg24149237	1.0815234	0.3425514	3.41465	0.8937167
cg17478228	0.2366669	0.0927435	0.603937	0.0025697
cg11033588	0.9084841	0.3948273	2.090391	0.8214059
cg06702499	0.4037708	0.0670204	2.432557	0.3222791
cg19710184	3.5577328	0.9241728	13.69599	0.0649929
cg21470090	10.121412	1.5983726	64.09205	0.0139716
cg06368760	0.6476177	0.1020917	4.108158	0.6448567
cg26344024	0.46166	0.2238318	0.952188	0.0363844
cg08550353	0.1594354	0.0494215	0.514344	0.0021225
cg27448426	0.3242574	0.1414456	0.743345	0.0077987
cg04832767	2.0608658	1.0346505	4.10493	0.0397002
cg08462122	0.2617543	0.0789633	0.867686	0.028374
cg12078872	0.2892564	0.088777	0.942466	0.0395625
cg11909137	1.0343049	0.462232	2.314393	0.9345829
cg20647118	1.1013391	0.5286425	2.294458	0.796591
cg10578112	6892756.9	1.64E-06	2.9E+19	0.2883623
cg25869295	0.9933902	0.4844253	2.037103	0.9855599
cg13164814	2.8334332	0.2967985	27.04981	0.3655996
cg00657415	2.1612863	0.2362953	19.76831	0.4949452
cg14851846	2.09E-08	5.91E-17	7.368629	0.0782243
cg09887220	3.1214056	1.3155951	7.405906	0.0098177
cg17088180	1.29E-09	6.42E-17	0.025759	0.017009
cg25258033	2.3496438	0.7905434	6.983584	0.1242766
cg04654167	0.1554864	0.0552664	0.437445	0.000421
cg23934075	0.0371295	0.0009235	1.492876	0.0805748
cg05979597	1.4156736	0.5745212	3.488351	0.4499714
cg00289094	6.6013166	0.8145918	53.49598	0.0770827
cg18104015	1.2683947	0.4519495	3.559746	0.6515831
cg01890726	5.2267852	1.1836916	23.07973	0.0290715
cg14014854	0.0016196	1.95E-07	13.43754	0.1628179
cg11594260	2.1423867	0.9871025	4.649792	0.0539634
cg14839440	1.44E-11	5.86E-20	0.003549	0.0113351
cg08233594	0.2244618	0.0139247	3.618257	0.2921928
cg16791210	0.0485474	0.0006277	3.754766	0.1726898
cg09888283	0.4118331	0.1875061	0.904538	0.0271127
cg23537141	146.48596	0.1081446	198420.8	0.1752848
cg09659803	0.3731098	0.0998327	1.394443	0.1427407
cg24588381	1.4314964	0.6775566	3.02437	0.3472345
cg21132104	12479.421	11.697412	13313709	0.0080184
cg06640593	0.2379556	0.0737115	0.768168	0.0163476
cg10843276	0.1440654	0.0366636	0.566088	0.0055218
cg18308184	1.4942276	0.6625369	3.36995	0.33312
cg18850300	0.7911685	0.397988	1.57278	0.5040083
cg20474425	1.0883389	0.4366544	2.712629	0.8558391
cg09925057	2.9372817	0.1438954	59.95759	0.4838188
cg09170051	5.397E+13	1.70E-19	1.72E+46	0.4076297
cg23676961	54285.341	7.81E-09	3.77E+17	0.4699176
cg11183001	0.0557071	0.0082255	0.377278	0.0030891
cg12883279	0.4063107	0.1531592	1.077888	0.0704059
cg19142341	2.5637025	0.4221557	15.56907	0.3063364
cg07424295	0.0205739	0.0022968	0.184295	0.000517
cg16409306	0.3827003	0.1319054	1.110338	0.0771653
cg17196713	0.3420957	0.1315184	0.889834	0.0278583
cg14456395	7.28E-05	1.80E-08	0.294177	0.0245273
cg09854007	11.180718	1.1538987	108.3357	0.0372054
cg06710742	1.1181563	0.4404805	2.838431	0.8142321
cg23276829	5.0971348	0.8033007	32.34254	0.0840545
cg26469387	2.1026564	1.0004398	4.41922	0.0498645

cg25288897	2.4758633	1.0643494	5.759292	0.0353132
cg12491094	1.57E-09	1.31E-17	0.187535	0.0326504
cg20257171	4.441E+19	17.609999	1.12E+38	0.0363805
cg01874562	0.0839116	0.0143285	0.491407	0.0059995
cg02397934	2.6751717	0.996435	7.182148	0.0508343
cg04653913	1.4552642	0.6169634	3.432609	0.3914874
cg19966212	2.233313	1.2456377	4.004123	0.0069898
cg04411454	2.9387893	0.9232287	9.354651	0.0680384
cg15847272	0.2297655	0.0696472	0.757994	0.0157378
cg12061285	51.990306	0.2596661	10409.49	0.1439386
cg06031301	1.7494479	0.7952658	3.848484	0.1643897
cg23728359	0.8289038	0.4288254	1.602241	0.5768056
cg13449368	0.345269	0.1604256	0.74309	0.006543
cg12891692	12.486465	0.6729674	231.6781	0.09023
cg01212887	0.045025	0.0029646	0.683813	0.0254971
cg26444282	0.2803722	0.115328	0.681608	0.0050215
cg23865698	2.6761656	1.0660375	6.718208	0.0360702
cg13482309	0.5293116	0.2229902	1.256426	0.1491882
cg20131682	565146139	4.48E-16	7.13E+32	0.476616
cg22022988	3.497158	0.8402956	14.55454	0.0852872
cg10661163	0.0710172	0.0190755	0.264393	8.03E-05
cg25616869	0.3164004	0.1387784	0.72136	0.0062053
cg26709300	2.1152441	0.6391974	6.999805	0.2198286
cg17015844	1.9799639	0.8668765	4.522278	0.1050267
cg08993172	2.0469762	1.1305494	3.706261	0.0180267
cg17086398	2.3051199	0.9743616	5.453394	0.057322
cg05184519	0.5102961	0.2564934	1.015239	0.0552542
cg12442108	3.2930976	0.6301654	17.20896	0.1577621
cg25382128	2.7549237	0.9060702	8.376398	0.0740811
cg17315014	0.7181236	0.3522763	1.463912	0.3621971
cg00814909	1.6244519	0.0569781	46.31329	0.7765377
cg26577017	5.992962	2.0449794	17.56281	0.0010984
cg14663264	3.3957928	1.0628128	10.8499	0.0391363
cg11141380	0.2594523	0.0846054	0.795641	0.0182841
cg07440775	2.1412415	0.8428781	5.439595	0.1094609
cg07402310	0.9893543	0.3198393	3.060356	0.9851791
cg08633745	0.5018473	0.2649824	0.950443	0.034349
cg15690598	0.5189742	0.2092003	1.287447	0.1570921
cg27537972	0.7713011	0.3695478	1.60982	0.4891219
cg03047813	2.2972808	1.1121695	4.745229	0.0246269
cg18040225	0.4403636	0.2160534	0.897556	0.02398
cg00744431	0.1497977	0.0507708	0.441973	0.0005838
cg14503063	0.6225053	0.282857	1.369995	0.2388925
cg08912058	3113.9129	0.382754	25333383	0.0799605
cg18031270	0.0002427	2.56E-07	0.229876	0.0172926
cg04832557	1.6883806	0.0505708	56.36905	0.7698093
cg21142349	0.1755161	0.0513271	0.600187	0.005541
cg20402783	1.8872163	0.8416609	4.231616	0.123181
cg09506504	2.0372149	0.5528897	7.50646	0.2848937
cg07200280	1.1753905	0.6285214	2.198084	0.6128765
cg10238972	0.7399256	0.3957559	1.383403	0.3454618
cg10284440	3.4992155	0.3984196	30.7327	0.2585383
cg14250984	0.2910847	0.095743	0.884976	0.0296039
cg16154857	3.0796642	1.0694726	8.868232	0.0371211
cg00532474	0.4699291	0.2517707	0.877121	0.0177048
cg19449067	0.337068	0.1666532	0.681744	0.0024783
cg23395449	2.0256517	1.038007	3.953022	0.0385164
cg17799760	5.6011138	0.1856641	168.9743	0.3215666

cg18373623	1.1375067	0.6083137	2.127063	0.6866188
cg04504312	2.44E-08	7.15E-16	0.834143	0.0476525
cg04984852	1.0108095	0.4551055	2.245053	0.9789323
cg03945800	0.2455038	0.0950251	0.634276	0.0037309
cg22598744	0.578043	0.2881444	1.159605	0.1228118
cg27126575	3.16265	1.0025889	9.976527	0.0494866
cg27434368	2.9355674	0.576557	14.94658	0.1946918
cg03356760	9022478.4	1.40E-09	5.83E+22	0.3885523
cg27603082	1.2979563	0.0368961	45.66035	0.885847
cg25956985	0.636205	0.2464653	1.642246	0.3499496
cg22487912	1.4373153	0.5455773	3.786586	0.4629406
cg03523533	0.5156312	0.0359434	7.397054	0.6259628
cg16791173	3.7207403	0.7407274	18.68961	0.1105961
cg00409684	1.7105434	0.8003788	3.655717	0.1659523
cg26107597	2.428746	0.5706909	10.33626	0.2297952
cg18332146	1003.5847	1.0986122	916776.9	0.0469221
cg04886857	0.3016444	0.0752923	1.208482	0.0905429
cg21547371	0.2231803	0.073777	0.675135	0.0079182
cg25103161	2.5697194	0.6888311	9.58647	0.1600108
cg14085952	2.985461	0.8270406	10.77695	0.0949172
cg13215970	1.3623097	0.6975177	2.660704	0.3653312
cg01530154	1.9565133	0.7929458	4.827498	0.145255
cg19014792	1.2372923	0.5203418	2.94209	0.6299534
cg01948850	0.3072152	0.1321752	0.714061	0.0060955
cg21051046	1.5388768	0.6330132	3.741062	0.3415708
cg03597174	1.7401209	0.4636687	6.530569	0.4116781
cg11822932	2.8889857	0.4961689	16.82136	0.2378923
cg22257099	0.0001546	6.60E-09	3.622379	0.0874075
cg02657539	7.067587	0.6687638	74.69122	0.1040495
cg19632953	4.9660631	0.8335414	29.58675	0.0784058
cg10636760	0.5023588	0.2013087	1.253619	0.1400749
cg10775844	1.2554315	0.417296	3.776955	0.6856319
cg10786087	40.453343	0.06417	25502.15	0.2605894
cg13798112	0.0789393	0.0052791	1.180399	0.0657993
cg00966098	0.0178805	0.0008087	0.395315	0.0108499
cg23497016	1.6134974	0.6249664	4.165622	0.3228559
cg13932362	1.3195546	0.7061183	2.46591	0.3847335
cg00355447	0.0839935	0.0077779	0.907041	0.0413174
cg07814707	0.7451397	0.2220336	2.500672	0.6339122
cg06379095	0.8615928	0.1239128	5.990845	0.8803171
cg06038342	0.2695326	0.1147584	0.63305	0.0026173
cg13218710	3.5053453	0.9547989	12.86914	0.0587236
cg11272515	1.6045153	0.7792846	3.303632	0.199429
cg18517369	1.3573042	0.6047557	3.046312	0.4589015
cg13474848	2.8904457	0.9414874	8.873912	0.0636518
cg13807056	0.4560871	0.177616	1.171152	0.1027603
cg24794881	1.0846225	0.4049092	2.905357	0.8716342
cg05987043	1.04E-05	5.20E-10	0.209122	0.0232333
cg00784153	1.3145681	0.5619148	3.075358	0.5282167
cg03160145	1.3329917	0.70548	2.518664	0.3759733
cg20303561	1.8814476	0.3489024	10.14566	0.462231
cg10888348	0.3170119	0.1376845	0.729905	0.0069363
cg20088969	0.0019779	4.76E-06	0.82106	0.0429637
cg10778249	0.351837	0.0940752	1.315855	0.1206349
cg03562531	0.1493657	0.0531294	0.41992	0.0003119
cg20713174	1.3988179	0.5149935	3.799449	0.5103289
cg09607452	0.3360789	0.1505167	0.750409	0.0078008
cg12649539	1.6322072	0.5449229	4.888949	0.3814064

cg03544320	0.3273409	0.121573	0.881381	0.0271179
cg10953003	0.3366405	0.0799105	1.418172	0.1378573
cg09706021	0.3473377	0.1739828	0.693422	0.0027184
cg26738010	0.3983422	0.13799	1.149913	0.0888093
cg04093381	0.2885605	0.1184374	0.703048	0.0062301
cg18151134	0.3167223	0.1435198	0.698949	0.0044154
cg02891153	0.1842173	0.0147863	2.295102	0.1887
cg01452444	9.36E-05	2.06E-20	4.25E+11	0.614045
cg19784545	3.561082	0.3929974	32.26817	0.2587169
cg07516483	2.7612581	1.2277565	6.210146	0.0140432
cg23855986	59985651	10.898964	3.3E+14	0.023722
cg14052235	0.4685801	0.0356388	6.160903	0.5641392
cg01792601	3.022592	1.1943772	7.649227	0.0195479
cg00344801	0.2539286	0.0746862	0.863342	0.0281414
cg16014770	0.2064057	0.043975	0.968808	0.0454861
cg19911116	0.5790626	0.257485	1.302264	0.186414
cg27619006	3.6482804	0.0120876	1101.127	0.656849
cg24374505	0.6712035	0.3370711	1.336555	0.256594
cg00463957	0.3286698	0.1326647	0.814262	0.0162228
cg18786420	1.2540216	0.4230191	3.717492	0.6830859
cg17791799	0.2544135	0.057229	1.131004	0.0721397
cg16549043	0.3462685	0.1447463	0.828359	0.0171672
cg11588903	0.2509694	0.1031635	0.610542	0.0023056
cg01090609	0.0013015	3.76E-07	4.502052	0.1100229
cg03461399	0.1312548	0.0053515	3.21926	0.213566
cg20064693	3.3611506	0.2345137	48.17345	0.3721797
cg16897216	0.608083	0.304353	1.214921	0.1589333
cg20131670	3.7532695	1.0972445	12.83855	0.0350429
cg22891561	0.7847215	0.3724046	1.653545	0.5238099
cg02273827	0.5999249	0.238093	1.511636	0.2785213
cg06899799	0.3047264	0.0746925	1.243207	0.0976194
cg00729291	4.0866881	1.4542931	11.48394	0.0075758
cg14054990	1.1314899	0.5283588	2.423106	0.750522
cg27552857	0.1696322	0.0590566	0.487246	0.0009824
cg06930722	0.6778226	0.3448497	1.3323	0.2593859
cg18313416	0.6454222	0.3308244	1.259187	0.1991146
cg21387735	2.5317229	0.8767066	7.311021	0.0860203
cg16439798	3.96E-15	3.03E-31	51.62511	0.0798348
cg10637512	0.1649633	0.0291016	0.935098	0.0417733
cg20539307	0.4616965	0.161063	1.323481	0.1503324
cg10564626	0.3296619	0.1664574	0.652882	0.0014582
cg26865290	5.5219931	0.2631374	115.8802	0.271208
cg07278975	35352.103	0.482899	2.59E+09	0.0668639
cg19139554	5.96E-07	8.13E-44	4.37E+30	0.7407
cg24431193	0.1730352	0.0115816	2.585231	0.2035435
cg26238041	0.1488323	0.0282035	0.7854	0.0247939
cg11590700	0.0171594	3.33E-05	8.844036	0.2020062
cg11007120	5.1459612	1.6721596	15.83636	0.0042851
cg20847625	1.4343361	0.5722847	3.594924	0.4416414
cg12425378	0.5020557	0.2366302	1.065206	0.0725944
cg00450571	13.687646	0.0056589	33107.56	0.5103946
cg04576021	4.9807609	0.4319244	57.43594	0.1980862
cg23229261	2.5859555	1.0988747	6.085467	0.0295629
cg05232694	0.7230488	0.2107839	2.480263	0.6061205
cg16171182	0.2553688	0.1062264	0.613908	0.0022869
cg25901381	1.730722	0.5180613	5.781938	0.372755
cg16601359	0.2566925	0.1007714	0.653867	0.0043648
cg00045114	1.6438383	0.5197694	5.198853	0.3975136

cg14555996	0.3215144	0.1353063	0.763981	0.0101814
cg00017489	0.4539398	0.2115248	0.974171	0.0426492
cg00204501	4.82E-05	1.63E-11	142.5928	0.1910366
cg23871796	17742307	4.9900324	6.31E+13	0.0300956
cg11656177	5.5809912	1.1950786	26.06311	0.0287714
cg18034329	10.728121	1.68E-07	6.86E+08	0.795826
cg17931529	3.2907924	0.1435704	75.4286	0.456043
cg07100508	0.0001865	3.37E-12	10321.7	0.3451838
cg05084827	0.2433623	0.094688	0.625477	0.0033435
cg25966908	1.8318642	0.8610749	3.897137	0.1160372
cg04493000	2.1614722	0.9881833	4.727829	0.0535825
cg02078518	0.7877658	0.2368868	2.619711	0.6971971
cg27467552	0.0202475	0.0005921	0.692392	0.0304685
cg13868216	0.2014505	0.0632985	0.641127	0.0066767
cg03557445	0.4626942	0.1745353	1.226606	0.1212977
cg04221521	2.0891063	1.0502075	4.155717	0.0357666
cg21016699	0.4517617	0.2041447	0.999726	0.0499209
cg25006077	0.5292604	0.2571826	1.089174	0.0839912
cg08730743	0.3395103	0.1295938	0.889451	0.0279225
cg05437648	0.1661843	0.0614972	0.449081	0.0004027
cg10170495	0.2408289	0.0706506	0.820921	0.0228856
cg24052359	2.172767	1.0148067	4.652035	0.0457379
cg04588336	1.3130176	0.3450179	4.996887	0.6896205
cg14141912	0.7211912	0.3370454	1.543165	0.3997011
cg15000813	0.3487333	0.1357762	0.895701	0.0286094
cg06163425	1.1643797	0.6076174	2.231306	0.6465089
cg01557547	0.7135426	0.2662399	1.912347	0.5022126
cg20813387	1.72E-09	2.61E-16	0.011307	0.0117518
cg09084320	1.3349344	0.4007341	4.446964	0.6379815
cg19374752	0.1903493	0.04773	0.759122	0.018751
cg08938669	1.10E-37	3.25E-67	3.74E-08	0.0141696
cg12137450	0.2173117	0.0661955	0.713408	0.0118437
cg26267852	2.0581663	0.9065477	4.672725	0.0844488
cg22104478	29590265	1.7843398	4.91E+14	0.0425364
cg14074117	1.2977696	0.6218812	2.708244	0.4874133
cg20677901	0.6020066	0.284257	1.274945	0.1849991
cg23112609	0.3197837	0.1047471	0.976272	0.0452702
cg11813441	0.205332	0.0723279	0.582918	0.0029418
cg12158124	0.041784	0.0047007	0.371413	0.0043929
cg10678486	1501823.8	3.90E-05	5.78E+16	0.2527623
cg07361491	6.30E-21	1.08E-33	3.66E-08	0.001923
cg04775890	0.1270134	0.0279547	0.57709	0.0075447
cg08293102	0.2855235	0.1100339	0.740896	0.0099838
cg25072962	1.682753	0.7461602	3.794973	0.2097456
cg26160189	2.9329657	0.5172632	16.63039	0.2242211
cg20076718	0.1878063	0.0233801	1.508602	0.1156804
cg17889402	2.0678298	0.8113628	5.270047	0.1280032
cg01011367	2.5650028	0.6515774	10.0974	0.1778891
cg26195356	2.1954296	1.0478986	4.599597	0.0371645
cg11062672	1.2940311	0.4658645	3.594428	0.6209446
cg22472290	0.2796895	0.1106088	0.707233	0.0071065
cg16546442	3.0155752	1.1081356	8.206301	0.0306963
cg01091117	2.402617	0.8766845	6.584545	0.0883612
cg07723559	9.2213822	0.5765626	147.4842	0.1162674
cg07824081	0.0473937	0.0075603	0.297101	0.0011304
cg26668276	2.8201823	1.0721513	7.418196	0.0356279
cg02528389	0.1665455	5.11E-08	542551.1	0.8147768
cg18557891	0.1209932	0.019454	0.75251	0.0235197

cg10739344	1.4007751	1.40E-11	1.4E+11	0.9791953
cg02534363	0.4056511	0.1749155	0.940756	0.0355305
cg23989110	8.277815	0.9848607	69.57555	0.0516645
cg26701242	1.5085892	0.8171175	2.785207	0.1887296
cg07602200	3.02E-07	1.48E-14	6.185759	0.0804985
cg27510066	0.3688464	0.1108816	1.226964	0.103861
cg22942897	1.6471336	0.6253741	4.338282	0.3125114
cg12078031	4.3427521	1.2915262	14.60249	0.0176236
cg00769161	0.3860562	0.1904734	0.782468	0.0082782
cg15995125	0.0684432	0.0150282	0.311713	0.0005264
cg12379775	0.2140235	0.06028	0.759888	0.0170931
cg10458581	1.18E-37	8.10E-73	0.017272	0.03957
cg02981942	2.0040759	0.9466179	4.24281	0.0692772
cg17928671	1746.9724	1.5890381	1920604	0.0366545
cg13957837	2.7406885	0.001195	6285.659	0.7984326
cg13709271	0.7659397	0.3950401	1.485074	0.4299194
cg04201527	2.401531	0.8844811	6.520604	0.0855969
cg23310253	1.3515141	0.1824623	10.01078	0.768119
cg12421834	1.4398446	0.6487571	3.195576	0.3701483
cg03609102	1.6025909	0.5967968	4.303471	0.3493876
cg06254214	13.082913	0.2884519	593.3836	0.1864428
cg19868364	1.3357405	0.6340192	2.814114	0.4464058
cg00933835	0.1906598	0.0705454	0.515287	0.0010869
cg12086773	715.93264	6.95E-12	7.37E+16	0.6896637
cg01909024	1.21E-06	7.77E-13	1.881086	0.0610539
cg26566236	2.8140176	0.0437153	181.1425	0.6263247
cg13558810	0.1894807	0.068884	0.521209	0.0012725
cg21334513	2.746456	0.7988316	9.442566	0.1088269
cg00213295	0.4663051	0.1933495	1.124598	0.0894076
cg16615388	0.4689863	0.1998111	1.100781	0.0819664
cg14899522	1.4357734	0.6493506	3.174626	0.3716257
cg22954906	0.6218173	0.17138	2.256138	0.4699562
cg21173406	1.5435789	0.7585893	3.140877	0.2310429
cg03949996	0.1348266	0.0316668	0.574046	0.0067105
cg07694319	2.2909098	0.7488353	7.008574	0.146227
cg09842331	1.5057449	0.5842991	3.880321	0.3967634
cg00820311	2.1805847	0.5763537	8.250054	0.2508399
cg05638739	0.4751795	0.1555467	1.451626	0.1915937
cg17936460	19.589425	3.68E-18	1.04E+20	0.8924314
cg02270689	0.3563544	0.1810398	0.70144	0.0028238
cg15771128	6.63E-06	2.81E-10	0.156417	0.0202821
cg26471497	2.2689811	0.7024919	7.32859	0.1707936
cg08847944	2.160008	1.0119854	4.610377	0.046507
cg07005654	0.2679612	0.0826224	0.869052	0.0282521
cg13086402	1.5833434	0.0932268	26.89117	0.7504794
cg14218223	0.4145073	0.1969882	0.872216	0.0203326
cg27466709	2.6775645	0.7860551	9.120673	0.1152552
cg02743029	2.2129521	1.1605564	4.219663	0.0158598
cg06651563	0.5288382	0.1912688	1.462182	0.2195353
cg08617982	2.3032303	0.9213062	5.757988	0.0743196
cg02425108	1.7693413	0.315206	9.931817	0.5168044
cg00167670	1.2348481	0.5681674	2.683804	0.5943105
cg17855963	0.6194188	0.1080148	3.552101	0.5909142
cg24138650	2.5730533	0.3279512	20.18777	0.3685432
cg22602105	0.3011402	0.105582	0.85891	0.0248079
cg13976657	4.4799036	0.9442603	21.25424	0.0590575
cg09148852	0.2397164	0.068921	0.833765	0.024715
cg16797656	4.9216871	0.5238383	46.24137	0.163233

cg13303573	2.1904946	0.0005289	9071.744	0.8536025
cg18985738	0.84218	0.4005648	1.770668	0.6505353
cg14519032	0.250487	0.0852412	0.736073	0.0118315
cg12332558	0.5681727	0.3084643	1.04654	0.0696777
cg11154384	22.565887	1.1965474	425.5738	0.0375521
cg03746765	0.3389844	0.1497051	0.767579	0.0094783
cg24689119	1.0947489	0.4135133	2.898275	0.8553954
cg10434728	0.5677612	0.1790872	1.799977	0.3362833
cg14245836	2.18E-19	7.13E-36	0.006686	0.0265192
cg08779207	2.168545	0.482749	9.741268	0.3125639
cg03248158	1.0062305	0.442783	2.286673	0.9881679
cg23882019	1.4344943	0.6481603	3.174792	0.3733733
cg20224311	0.0003261	1.33E-07	0.801959	0.0438663
cg22613010	0.0009005	9.68E-12	83810.64	0.4538236
cg03325693	0.3053025	0.0956689	0.974294	0.0450753
cg21162779	1.8506645	0.6264629	5.467138	0.2653786
cg04384031	0.0876747	0.0151344	0.507907	0.0066111
cg10726559	1.4044183	0.670428	2.941988	0.3680243
cg26159990	0.4221391	0.1636577	1.088867	0.074446
cg17832277	182529621	0.0004113	8.1E+19	0.1644695
cg08555653	1.8587617	0.8061535	4.285778	0.1458326
cg20418394	0.2714229	0.0821829	0.89642	0.0324076
cg06993429	0.2071759	0.0679885	0.63131	0.0056221
cg00720581	2.29654	0.9414498	5.6021	0.0676465
cg18843739	1.6803447	0.780234	3.618861	0.184855
cg25162948	1.0823912	0.5763248	2.032831	0.8055195
cg13763287	0.0911754	0.0188232	0.441633	0.0029274
cg11396313	0.2803445	0.081829	0.960454	0.0429509
cg07282889	1.1342785	0.4781162	2.690952	0.7749896
cg11773456	0.3890446	0.1660054	0.911752	0.0298122
cg07054564	1.3504022	0.6297758	2.895612	0.4401912
cg11076487	29.289824	0.9072729	945.5742	0.0567708
cg14534144	0.2612933	0.1263204	0.540484	0.0002956
cg18869127	0.1938599	0.0636249	0.590676	0.0038998
cg13745346	2.0700494	0.7696821	5.56737	0.1494813
cg11595635	0.5865137	0.2869469	1.198822	0.143521
cg03479209	1.7346495	0.6755863	4.453922	0.2522764
cg00997699	3.2316227	0.7397917	14.11666	0.1189221
cg01916724	0.3942576	0.048762	3.18771	0.3827619
cg21198550	1.8670814	1.0043363	3.470942	0.0484226
cg21417675	4.396E+09	828.40911	2.33E+16	0.0049465
cg27175491	0.7441016	0.3644787	1.519121	0.4169608
cg20497554	2.76E-12	5.32E-28	14290.72	0.149377
cg00096418	1.0517521	0.5309407	2.083439	0.8849671
cg25946389	0.3878721	0.0980956	1.533654	0.1769338
cg23589237	1.5107168	0.162748	14.0233	0.7166596
cg01022670	0.3944714	0.1805219	0.861988	0.0196829
cg02236134	49.321615	1.73E-09	1.41E+12	0.7509648
cg13166942	0.3978905	0.1481602	1.068552	0.0674871
cg05620288	0.3022263	0.0816695	1.118419	0.0730805
cg13214190	1.4308257	0.6801605	3.009969	0.3450816
cg22876643	1.5402977	0.2098989	11.30314	0.6709876
cg25022311	0.6753702	0.3198053	1.426258	0.3034498
cg20056542	1.4337425	0.8307777	2.474329	0.1956398
cg13299824	8.6710328	1.6664272	45.11857	0.0102631
cg07504428	0.3370757	0.1184413	0.959294	0.0415658
cg18957462	1.67E-07	3.85E-20	722166.5	0.2931372
cg26254678	5.1249744	0.2834883	92.6506	0.2685355

cg01220746	1.74E-12	1.33E-25	22.7923	0.0789024
cg09635036	0.4005832	0.1741838	0.921251	0.0313187
cg10243307	0.9146675	0.3774732	2.21636	0.8434197
cg16595607	3.5219123	1.0142882	12.22913	0.0474455
cg18867001	7.5052998	1.2696632	44.36572	0.0261943
cg25408329	1.6322795	0.1519101	17.5389	0.6858835
cg22946562	0.679486	0.3233248	1.42798	0.3078346
cg03655684	1.3230018	0.2818222	6.210773	0.7227666
cg04269907	0.1065314	0.012863	0.882295	0.0378881
cg02939078	0.3140174	0.1625976	0.606448	0.000562
cg24134020	0.4763989	0.000198	1146.195	0.8519239
cg05514971	0.1398163	0.0286654	0.681958	0.0149571
cg02048412	0.0741023	0.0138336	0.396942	0.0023739
cg21869609	0.2340018	0.0983841	0.556562	0.0010181
cg04267164	0.0001484	6.50E-12	3388.801	0.3078475
cg24000300	0.5981994	0.2879025	1.24293	0.1684754
cg23568643	0.3137214	0.1266687	0.776996	0.0122364
cg10672884	4894.6836	1.54E-06	1.56E+13	0.446648
cg02727104	3.3581348	1.2013577	9.386937	0.0209018
cg01517500	1.7178417	0.5521269	5.34475	0.3501485
cg09288218	0.2507352	0.0803497	0.782432	0.0171945
cg13066481	0.1399704	0.0245206	0.798991	0.0269349
cg15999311	1.6375045	0.6616321	4.052737	0.286138
cg18667659	0.3812938	0.0971373	1.496696	0.166981
cg14604681	3.6511813	1.0248203	13.00826	0.04574
cg19175226	17.150206	0.6206877	473.8769	0.0932847
cg04656009	2.0531918	0.9164086	4.600128	0.080486
cg24857560	0.6413519	0.3150286	1.305698	0.2207341
cg04351541	1.4612552	0.6373998	3.349965	0.3702296
cg17845761	0.3428764	0.1340193	0.877219	0.0255302
cg17537177	0.1238719	0.0130663	1.174338	0.0687703
cg21545862	3.62278	1.5098691	8.692498	0.0039435
cg26219182	2.51E-08	3.60E-15	0.174826	0.0294882
cg04242070	0.3323861	0.0912138	1.211226	0.0950179
cg02799298	0.6768183	0.2958969	1.548117	0.3551296
cg07037251	1.9503002	0.7896395	4.816971	0.1476181
cg24802745	0.1675689	0.0406818	0.690219	0.0133881
cg24754277	0.664619	0.2992897	1.47589	0.3155401
cg12394628	0.001019	5.95E-11	17446.29	0.4175648
cg15575967	2.5171747	0.6748757	9.388646	0.1692928
cg17759921	0.237757	0.0369516	1.529796	0.1304383
cg04552852	0.1581418	0.0407924	0.613076	0.0076379
cg03996465	4.003E+13	1.82E-21	8.82E+47	0.4375755
cg00324725	0.273545	0.0690654	1.08342	0.0649108
cg00549973	1.4514889	0.316491	6.656807	0.6316016
cg01564135	2.2183621	1.0255232	4.798654	0.0429717
cg05783185	0.6227761	0.2608759	1.486723	0.2861096
cg11815984	0.4423084	0.1904102	1.027449	0.0578288
cg20627046	0.2858462	0.0854283	0.956451	0.0421314
cg10941484	1.08E-31	7.14E-51	1.65E-12	0.0015562
cg18148375	0.5624061	0.1735255	1.82279	0.3374162
cg15871509	0.0015627	1.43E-06	1.709466	0.0703295
cg16275483	1.5498387	0.5110028	4.700561	0.4389399
cg00222684	1.1948604	0.4372158	3.265416	0.7285376
cg04246726	23.197079	0.0011935	450868.4	0.5326118
cg18571045	1.9244614	0.2580291	14.35323	0.5231073
cg12077460	2.4578615	0.3944115	15.3167	0.3353763
cg00205703	0.3702744	0.0525412	2.609443	0.3186513

cg20778547	0.4376676	0.1759263	1.088825	0.0755752
cg17371911	200927069	4.3250085	9.33E+15	0.0337921
cg06455686	0.3558209	0.1362523	0.929221	0.0348714
cg03446165	0.7714175	0.135764	4.38323	0.7696856
cg03222672	54.855265	0.0198857	151319.6	0.3218141
cg05068452	1.4405382	0.5727474	3.623151	0.437945
cg10631289	0.1709696	0.0508244	0.575129	0.0043216
cg17669365	2.7581484	0.1919514	39.63182	0.4555867
cg20269607	4.4454262	0.9718017	20.33523	0.0544684
cg07841500	1.53E-10	1.58E-20	1.485491	0.054075
cg03968436	0.2993482	0.0730305	1.227012	0.0937905
cg16749578	0.1205829	0.0237416	0.612437	0.0107322
cg18077307	9.7273297	0.8140186	116.2393	0.0722742
cg04438525	1.5405882	0.7031196	3.375545	0.2802089
cg07317017	0.3623086	0.1378678	0.952126	0.0394484
cg13526040	0.1018089	0.0105853	0.979192	0.0479095
cg22454744	0.4238316	0.0787431	2.281256	0.3175045
cg17828456	1.2701514	0.5890629	2.738731	0.5418622
cg04687517	1.5596093	0.6172787	3.940491	0.3473175
cg16392084	0.5667412	0.1627242	1.973865	0.3724394
cg06438404	1.98E-16	9.32E-28	4.20E-05	0.0065818
cg14633892	0.2684859	0.1255967	0.573938	0.0006929
cg01013171	1.0407301	0.399945	2.70817	0.9347916
cg00689612	2.1729022	0.4987952	9.465817	0.3013274
cg13783434	1.1541105	0.5286529	2.519557	0.7189905
cg16137928	0.6800017	0.267845	1.72638	0.4171921
cg27415032	16.451968	0.5065668	534.317	0.1147986
cg03974488	0.5066184	0.2424135	1.058779	0.0705918
cg27436579	5.97E-13	2.18E-20	1.64E-05	0.0012765
cg20155988	5.8527312	4.60E-26	7.45E+26	0.9540565
cg09984096	2.5616163	1.1462817	5.72449	0.0218636
cg03873049	0.1934531	0.0758127	0.493639	0.0005882
cg12651286	0.4909943	0.2246211	1.073253	0.0746216
cg15543443	7821950.3	6.87E-11	8.90E+23	0.4282869
cg26195829	2.2256349	0.9290035	5.332004	0.0726921
cg14497545	0.208732	0.0720882	0.604386	0.0038738
cg25829961	3.9406719	0.2784186	55.77536	0.3104534
cg09046427	0.5105516	0.1878628	1.387518	0.1875363
cg14046302	0.2878859	0.1382148	0.599634	0.0008808
cg15866393	0.4735919	0.1730886	1.295806	0.1455658
cg01894192	0.2631922	0.0894989	0.773977	0.0152868
cg07330634	0.6432183	0.297966	1.388513	0.2610394
cg23022574	3.2723606	1.0962638	9.768036	0.0336132
cg18635791	0.8234053	0.4045438	1.675953	0.5920507
cg02084553	0.2952465	0.1315541	0.662621	0.0030986
cg05987030	0.0001703	5.46E-10	53.16538	0.1788194
cg18713028	2.8347237	1.1162278	7.198941	0.0284375
cg13599258	1.8292782	0.8080685	4.141058	0.14741
cg01806181	0.3843624	0.136489	1.082392	0.0702826
cg26948274	0.37409	0.1002254	1.396287	0.1434111
cg14887116	0.742319	0.3953738	1.393713	0.3538761
cg24603028	5.1347912	0.0988297	266.7829	0.4169578
cg18180276	0.6231541	0.3443281	1.127765	0.1181251
cg14374347	400.5341	6.6234776	24221.05	0.0041928
cg05921581	1.7748243	0.6707779	4.696042	0.247839
cg25388447	0.9267683	0.4249788	2.021041	0.8483823
cg21141234	1.6227425	0.6625694	3.974366	0.2894694
cg19758873	2.1027655	0.2345419	18.85217	0.5065884

cg09363342	0.1808181	0.062177	0.52584	0.001689
cg07025242	2.2240556	0.7154386	6.913834	0.1671858
cg11193281	0.237062	0.0735796	0.763777	0.015891
cg11466131	3.2006633	0.707499	14.47952	0.1308778
cg14255103	4.3095297	0.6458137	28.75759	0.1314362
cg04339790	1.3198014	0.488956	3.562439	0.5838931
cg23431450	0.6875664	0.0453743	10.41885	0.7870813
cg08443351	436628.81	1.7254129	1.1E+11	0.0407667
cg16762841	1.1157797	0.6270284	1.985499	0.7094654
cg03708254	0.0917264	0.0187321	0.44916	0.003204
cg03846076	1.5974584	0.7410401	3.443637	0.2319967
cg13132965	4.857381	1.2058706	19.56607	0.0261954
cg02675920	0.4022098	0.1920613	0.842297	0.0157335
cg24128316	0.2054987	0.0790228	0.534399	0.0011744
cg23627083	0.6106085	0.2860034	1.303631	0.2023923
cg27662481	0.8503628	0.4245398	1.703296	0.6474264
cg03854238	0.4957124	0.1897395	1.295095	0.152081
cg04486305	153.13035	5.2382812	4476.45	0.0034828
cg10925518	2.909921	0.8709723	9.722054	0.0826515
cg17077610	2.397862	0.6664923	8.626869	0.1806182
cg19519911	1.5957593	0.5952867	4.277683	0.3529222
cg05996512	1.0359682	0.3588429	2.990808	0.9479151
cg16138910	4.1235902	0.8873379	19.16293	0.0706894
cg19754308	216.76813	12.808106	3668.647	0.0001939
cg03336706	0.5684754	0.1993622	1.620991	0.290764
cg11628880	2.5724741	0.5455079	12.13112	0.2324463
cg10386863	270949.06	2.1072952	3.48E+10	0.0371465
cg15845118	4.2231349	1.2821948	13.90964	0.0178516
cg10824972	0.4074131	0.1975258	0.840323	0.0150593
cg14158424	1.0770094	0.5634716	2.058576	0.8224054
cg03001048	2.4616226	0.4972845	12.18535	0.269641
cg18362330	1.5863524	0.7716075	3.261391	0.2095285
cg10061384	3.1499714	0.9768414	10.15755	0.0547644
cg04037952	5.0949455	0.9002926	28.83337	0.0655934
cg01466330	0.8733014	0.3482423	2.190013	0.772728
cg16418163	131464.54	5.73E-24	3.02E+33	0.7235267
cg14387312	0.3507489	0.1453348	0.846493	0.0197688
cg16179938	1.9327254	1.0445092	3.576251	0.035847
cg20099005	1.1210682	0.4482849	2.803561	0.8069469
cg13054419	0.2885361	0.1203863	0.691549	0.0053207
cg22758493	0.4072618	0.1736389	0.955214	0.0388936
cg09404289	0.6134064	0.301178	1.249319	0.1781017
cg17631390	2.6347486	0.5942099	11.68257	0.2023286
cg00647658	0.1001505	0.0256707	0.390723	0.0009231
cg00073837	3.3127086	0.9391844	11.68465	0.0625461
cg00852603	0.6641871	0.33579	1.313751	0.2396671
cg04348247	0.6641806	0.2303576	1.915004	0.4488143
cg13209441	1.5130165	0.7419079	3.085584	0.2547384
cg04546041	0.1195003	0.0185962	0.767914	0.0252093
cg13751927	1.7838662	0.4017215	7.921355	0.4466928
cg27627209	0.8549256	0.4000623	1.82696	0.6858145
cg27652887	2.883E+17	28523.829	2.91E+30	0.0085028
cg00076797	2.9268466	0.7783623	11.00571	0.112019
cg03900378	0.4485212	0.2072077	0.970868	0.0418507
cg23551132	15991.434	75.965241	3366355	0.0003904
cg19051802	2.1833642	0.8191872	5.81928	0.1184737
cg22125112	1.5119346	0.6352438	3.598534	0.3501111
cg10227368	0.8374719	0.3061976	2.290544	0.7297134

cg00822840	1.7231762	0.706616	4.202192	0.2315245
cg17426146	1.1070367	0.4296974	2.852078	0.8332003
cg10132694	1.3167406	0.639067	2.713027	0.4556548
cg25517015	0.5728925	0.2736541	1.199346	0.1394744
cg22867816	4.6890093	1.0343018	21.25763	0.0451025
cg11824921	0.5112993	0.1886136	1.386045	0.187382
cg23394510	0.4519354	0.230021	0.887943	0.0211741
cg09302895	0.5910595	0.1477046	2.365202	0.4573485
cg15684962	1.7402422	0.8360367	3.62238	0.1385571
cg07950000	0.2064777	0.0398489	1.069867	0.0601757
cg27099455	0.0006071	1.70E-07	2.166046	0.0759366
cg07814974	3.48E-06	8.44E-11	0.143819	0.0204721
cg06351796	0.5046082	0.2441094	1.043096	0.0648801
cg01885814	6.5560428	0.019116	2248.462	0.5278214
cg19237879	0.368436	0.1537952	0.882636	0.0250883
cg18113826	3.9475007	0.7349523	21.20241	0.1093954
cg05714552	9.743323	1.5985277	59.38736	0.0135637
cg20608990	0.7911701	0.3824547	1.636665	0.5276531
cg24592892	0.372163	0.1667857	0.830439	0.0157923
cg18741175	0.298191	0.0945743	0.94019	0.0389017
cg10673833	2.4218817	0.6277937	9.343056	0.1990999
cg27105183	3.5101565	1.4634499	8.419283	0.0049072
cg08385610	0.3679057	0.1738559	0.778545	0.008936
cg09468264	0.323944	0.1378352	0.761342	0.0097271
cg10530336	0.3573211	0.1301849	0.980746	0.0457489
cg05924485	0.5058516	0.2036562	1.25646	0.142063
cg25536137	1.632333	0.6269096	4.250232	0.3155744
cg02589899	396.12066	0.1510641	1038709	0.1363908
cg13673164	0.0948845	0.0142158	0.633313	0.0150327
cg10003262	0.2643073	0.0401985	1.737835	0.1661068
cg15081344	1.435614	0.5405361	3.812858	0.468114
cg13622267	2.068E+09	3.30E-09	1.30E+27	0.3049483
cg00096536	3.6274	1.4136135	9.308082	0.0073645
cg07142523	0.3504241	0.0638146	1.924278	0.2275398
cg09610767	2.0738282	0.9316319	4.616376	0.0740166
cg09810089	3.7838074	0.8502625	16.83856	0.0806354
cg24717935	2.2902521	1.0270185	5.107264	0.0428554
cg00334257	2.92E+34	2.05E-07	4.17E+75	0.100708
cg13244804	27814.566	38.986009	19844301	0.0022675
cg23369234	0.3756228	0.1499253	0.941085	0.0366589
cg16512755	0.0005477	6.76E-10	443.5269	0.2792912
cg10444281	0.0146779	0.0012682	0.169882	0.0007281
cg01427567	0.1545754	0.0332651	0.718278	0.017212
cg12624641	0.4095183	0.1232177	1.361048	0.1451382
cg12586150	1.6906511	0.4750421	6.016943	0.4175162
cg06089960	0.6355292	0.2652078	1.522947	0.3093468
cg02928840	4.2480316	1.0214692	17.66649	0.0466818
cg04089800	0.3433164	0.1629359	0.72339	0.004931
cg13370852	0.2732128	0.0880298	0.847954	0.0247438
cg22689630	1.5141121	0.8136283	2.817669	0.1905041
cg14675200	2.5441528	0.6501826	9.955223	0.1797577
cg21988461	0.305823	0.0995545	0.939462	0.0385441
cg03760839	5.2963024	1.4820026	18.92764	0.0103069
cg00639447	0.4969119	0.2248339	1.098239	0.0839217
cg25881591	2.1271714	0.8445622	5.357638	0.1092629
cg25003307	0.2262577	0.0786004	0.651301	0.0058724
cg05652719	0.6668407	0.3054533	1.455792	0.3090569
cg14398353	0.6347552	0.2966129	1.358384	0.2416385

cg03045079	2.88E-09	1.58E-18	5.252623	0.070683
cg14083421	2.9472718	1.1426342	7.602093	0.0253671
cg10492511	0.3316824	0.1294295	0.849986	0.0215341
cg19272547	4.1702816	0.3832401	45.37951	0.241005
cg22465516	2.3416627	0.6652808	8.24221	0.1851006
cg19053046	0.2489732	0.1051695	0.589407	0.0015655
cg03811629	1.6338568	0.6969369	3.830315	0.2587399
cg03257822	1.2371486	0.6691415	2.287314	0.4973378
cg07421415	1.879E+17	1.77E-07	2.00E+41	0.1588027
cg10958362	0.991799	0.5644218	1.742784	0.9771588
cg07288869	9.261209	7.68E-15	1.12E+16	0.9000267
cg00458454	1.3753008	0.2003689	9.43985	0.745751
cg00073090	0.4332839	0.1246017	1.50668	0.1884037
cg16646909	0.0001521	4.73E-07	0.048914	0.0028415
cg23352157	0.2026802	0.069846	0.58814	0.0033195
cg22807241	0.6248062	0.2985952	1.307398	0.2118656
cg14186992	0.1806316	0.0517426	0.630579	0.0072991
cg09639890	0.542503	0.2674393	1.100472	0.0901387
cg17893426	1.9774764	0.6331102	6.176512	0.2406627
cg16509173	1.4992825	0.4969699	4.523106	0.4722355
cg02305377	1.3147909	0.7129543	2.424665	0.3807879
cg23484358	0.4982124	0.1784329	1.391087	0.1835501
cg15463989	0.9525375	0.4415386	2.054923	0.9013506
cg20316549	1.772374	0.9002225	3.489481	0.0977526
cg26405475	1.5222865	0.623346	3.717608	0.3563066
cg05510665	1.6598906	0.8575725	3.212833	0.1325924
cg08937407	1.0607027	0.1390792	8.089564	0.9546628
cg23485436	0.0741306	0.0186189	0.295149	0.0002234
cg13491724	0.3753309	0.1378664	1.02181	0.055144
cg11097541	4.5169421	1.1374941	17.93659	0.0321079
cg24848973	4.2234017	0.7206706	24.75073	0.1102952
cg26474666	3.3543413	0.8344496	13.48387	0.088195
cg23587050	3.27E-18	3.70E-29	2.89E-07	0.0017426
cg27167562	0.1297007	0.0334612	0.502739	0.0031288
cg26389053	0.1687918	0.0540283	0.527329	0.0022061
cg10042298	0.4629697	0.245705	0.872351	0.0171977
cg18874502	2.27E-19	2.04E-64	2.53E+26	0.4172608
cg15637095	0.3337345	0.1097655	1.014697	0.0530826
cg01129128	0.7855612	0.0075355	81.89335	0.9189138
cg26515805	0.0001005	7.40E-11	136.5368	0.2013966
cg06760566	0.4246062	0.216261	0.83367	0.0128304
cg01048346	0.0063532	1.88E-05	2.142602	0.0884971
cg27245185	1.971109	0.1412339	27.50947	0.6138579
cg00137016	0.1036855	0.0143359	0.749912	0.0247647
cg03304437	1.1358261	0.5941677	2.171274	0.7000553
cg15554284	25.037795	0.8474004	739.7815	0.0623053
cg24349886	0.1798085	0.0552753	0.58491	0.0043572
cg20038996	1.3372671	0.7057589	2.533844	0.3727833
cg24442454	0.2815474	0.109135	0.726338	0.0087617
cg27421308	2.208E+14	6.92E-08	7.05E+35	0.1910901
cg10976732	3.349653	1.3285538	8.445405	0.010405
cg03399598	16257.081	0.3207728	8.24E+08	0.079387
cg08506743	1.4716188	0.6392486	3.387824	0.3637866
cg01317029	0.1101338	0.0267273	0.453822	0.0022618
cg10821830	1.7985966	0.7811711	4.141154	0.1677209
cg01968402	2.204484	1.0702108	4.540928	0.0320325
cg05991092	0.3742484	0.1451191	0.965151	0.0420174
cg01759597	2.0499994	1.0553755	3.981993	0.0340848

cg11348106	0.4915491	0.2363278	1.022396	0.0573429
cg17255397	1.361947	0.734133	2.526653	0.3272122
cg08786003	1.0736583	0.5888258	1.957696	0.81662
cg22492099	1.1242622	0.5653639	2.235667	0.7384137
cg10852861	2.2973314	1.1076382	4.764851	0.0254423
cg02341119	1.5063683	0.4112423	5.517782	0.5362368
cg24767540	1.3459636	0.6300724	2.875254	0.442965
cg16401490	2.7687816	0.7243217	10.5839	0.1366049
cg05488632	3.0132901	1.1669032	7.781209	0.0226756
cg02616368	0.7503765	0.220314	2.555739	0.64603
cg26093711	7.44E+26	49.096626	1.13E+52	0.0364752
cg17156558	0.0103361	3.03E-07	352.3544	0.3905517
cg07797073	1.5157961	0.6157209	3.731623	0.3655169
cg05175964	3.096179	0.6124146	15.65332	0.1716549
cg01094192	0.1586477	0.0606243	0.415165	0.0001761
cg01464515	0.4262553	0.0834247	2.177935	0.3055302
cg18674914	4.22E-05	1.78E-14	100063	0.3603804
cg27637086	10.280054	1.2655518	83.50469	0.0292334
cg05280806	0.2025665	0.076146	0.538875	0.0013815
cg14067788	3.2906008	0.9323102	11.61422	0.0641641
cg06535942	25.167802	4.77E-38	1.33E+40	0.9434729
cg04759187	827.13261	0.6669687	1025758	0.0645271
cg05801989	32.776194	4.04E-15	2.66E+17	0.8518871
cg11380092	7.2029992	0.9886901	52.47671	0.0513267
cg27370991	3.0088215	0.509936	17.75322	0.223862
cg24724506	0.074252	0.0192004	0.287148	0.0001645
cg17485838	0.4352396	0.2057612	0.920648	0.0295358
cg10489124	1.0558817	0.4509274	2.47243	0.9003168
cg23008646	1.4673316	0.7302047	2.948573	0.281527
cg24091438	1.2103428	0.4781167	3.063958	0.6870621
cg11642681	0.4895739	0.1404794	1.706176	0.2621838
cg10311358	2.83E-09	7.07E-14	0.000113	0.0002724
cg08923160	1.3407457	0.6296024	2.855134	0.4470696
cg03559568	0.0353975	7.89E-06	158.7449	0.436098
cg01931883	35.197872	0.5793491	2138.417	0.0892325
cg22706186	0.3577821	0.1515064	0.844902	0.0190591
cg19907725	0.5305343	0.1569517	1.793333	0.3077059
cg07220782	0.0607377	0.0079714	0.46279	0.0068592
cg06298729	0.2582668	0.1086728	0.613785	0.0021758
cg23519803	0.0107589	1.40E-05	8.267715	0.1812688
cg11270917	1.417254	0.5458853	3.679544	0.4737529
cg10392572	0.420874	0.1022635	1.732143	0.2305628
cg17427702	1.6887608	0.8102108	3.519964	0.1620141
cg12046314	0.1802654	0.0364051	0.892612	0.0358037
cg04877966	7.04E-20	3.86E-37	0.012816	0.0296425
cg18436758	0.0062085	4.83E-05	0.798769	0.0403031
cg22191606	1.4831211	0.6514009	3.376796	0.3477756
cg22127570	0.3048692	0.094765	0.980797	0.0463176
cg16651877	2.0428943	0.760451	5.488081	0.1565304
cg25043050	0.3444304	0.1285665	0.922731	0.0340138
cg01382953	3.7531263	0.9023372	15.61052	0.0689645
cg10743062	0.1560816	0.0244886	0.994809	0.0493597
cg19641804	0.1563793	0.0568129	0.430439	0.0003285
cg03679755	2.3814463	1.0981541	5.164381	0.0280178
cg22428147	0.4168674	0.1559751	1.114142	0.0810764
cg07914962	1.1276548	0.1200569	10.59169	0.9162774
cg02066681	3.8910254	0.6996904	21.63825	0.1206562
cg01781529	0.2333962	0.0879217	0.619572	0.0034887

cg00399175	1.3125193	0.6773922	2.543146	0.4203495
cg25486757	0.1454597	0.0040216	5.261273	0.2923253
cg13453750	0.397541	0.1356322	1.165202	0.0927057
cg17548735	0.1402194	0.0298552	0.658561	0.0128025
cg04612276	1.9962093	0.4328521	9.206035	0.3754492
cg19981263	0.9046992	0.4076724	2.007692	0.8054883
cg20457275	6.9427969	1.5947359	30.22596	0.0098284
cg11022236	1.045E+12	0.8254941	1.32E+24	0.0515976
cg03046445	0.0761481	0.0137897	0.420498	0.0031405
cg08370082	0.0644191	0.0012558	3.304488	0.1722501
cg22627876	2.1806821	0.5354722	8.880713	0.2765183
cg10583180	2.9231354	1.2130924	7.043751	0.0168274
cg04232649	4.41E-21	3.51E-36	5.53E-06	0.0082303
cg13376246	2.6240423	0.7149785	9.630497	0.1458844
cg04849824	1.8917781	0.0432636	82.72131	0.7408432
cg12151296	1.8854791	0.3797882	9.360563	0.4379072
cg10959907	0.2993178	0.1139339	0.786343	0.014377
cg07747220	3.8460988	0.698427	21.1797	0.1217176
cg06013895	0.48259	0.2267338	1.027165	0.0587018
cg04157243	4.3943902	1.1841851	16.30713	0.0269217
cg19159011	1.641859	0.7014631	3.842969	0.2531452
cg00983520	0.1788753	0.0500779	0.638932	0.0080588
cg07362168	1.7122783	0.995422	2.945381	0.0519698
cg21462934	2.9844133	0.6546749	13.6048	0.1577562
cg04583904	0.2186028	0.0637371	0.749754	0.0156073
cg24427895	1.9215787	0.938954	3.93253	0.073845
cg07100542	1.2771338	0.5855081	2.785736	0.5387168
cg00982680	0.0010785	4.74E-28	2.45E+21	0.8112881
cg00026703	0.1989738	0.0302235	1.309927	0.0931151
cg03211192	0.8541723	0.1895768	3.848627	0.8373958
cg17872838	2.2329153	0.1668055	29.89057	0.5439138
cg19142399	0.380803	0.122146	1.187193	0.0960743
cg00549433	0.2858444	0.0648667	1.259613	0.0979345
cg07579831	2.6270499	1.1348756	6.081188	0.0241075
cg09852079	30.672259	1.1974126	785.6836	0.0385609
cg11506907	20945.432	7.53E-06	5.83E+13	0.3698515
cg14398957	0.2739291	0.0983517	0.762947	0.0132242
cg25721982	2.1577521	0.7733194	6.020661	0.1418438
cg22066894	2.4918437	0.9599459	6.46837	0.0606591
cg24020398	9.78E-07	7.82E-13	1.223716	0.0533867
cg08160970	0.4903517	0.2076089	1.158163	0.1041378
cg04084354	0.8925071	0.3977984	2.002444	0.7826849
cg26960719	2.1902362	0.1392432	34.45149	0.577083
cg23467659	0.6226235	0.2594844	1.493963	0.2886786
cg09454230	1.5585253	0.271793	8.936951	0.618492
cg03346271	2.1236344	0.8732829	5.164218	0.0966908
cg17650272	0.2016589	0.0338991	1.199628	0.0784233
cg26737948	0.0072399	2.73E-10	192002.3	0.5720254
cg09773458	0.5654951	0.2912774	1.09787	0.0921595
cg10062919	0.0928497	0.0163127	0.528489	0.0073906
cg15145908	0.9013869	0.2928942	2.774033	0.8563546
cg16348254	6.0797471	1.1964395	30.89444	0.0295401
cg07481491	0.3317056	0.1212661	0.907332	0.0316044
cg22030072	2.5199184	1.1325945	5.606586	0.0235054
cg27057275	0.4659545	0.2354475	0.922132	0.0283266
cg03804126	0.5293736	0.2663974	1.051949	0.0694599
cg21877956	0.6645215	0.0830334	5.318208	0.7001371
cg23384340	26812.208	0.049101	1.46E+10	0.1303273

cg21052814	1.2197586	0.4814545	3.090242	0.6753327
cg06206218	0.1267607	6.10E-06	2633.484	0.6838589
cg12720444	0.0028501	3.04E-05	0.267598	0.0114451
cg13073141	4.5108776	0.6800043	29.92336	0.1186452
cg04780159	0.1093999	0.0146189	0.81869	0.0311797
cg22911315	0.5167706	0.2677968	0.997218	0.049037
cg13683218	1.1089633	0.5678831	2.165586	0.7619775
cg10428982	1.6852704	0.7757131	3.661323	0.1873648
cg03969301	1.2402152	0.44841	3.430195	0.6783161
cg23597650	1.9138402	0.4017477	9.117126	0.4150775
cg01499816	7.2890829	1.0700485	49.65264	0.0424456
cg06613286	0.1174566	0.01509	0.914249	0.0407964
cg13970437	0.3534747	0.16496	0.757422	0.0074844
cg17153666	2.2720432	0.6110333	8.44828	0.2206526
cg19513940	1.893515	1.0157103	3.529942	0.0445354
cg17259183	0.4347564	0.1039538	1.818242	0.2538693
cg10131297	0.602479	0.2677488	1.355677	0.2207429
cg22315746	0.125519	0.0050624	3.112145	0.2051925
cg25460984	1.1506651	0.3411834	3.880699	0.8209973
cg12028936	0.4661961	0.2208703	0.984011	0.0452576
cg07746960	4.1379616	1.1430546	14.9798	0.0304904
cg13210239	0.1692308	0.0475504	0.602289	0.0060925
cg09118625	5.3330252	0.7660827	37.12544	0.0908731
cg20237264	1.3223528	0.0881659	19.83325	0.8397339
cg04108328	0.4769922	0.2090207	1.088512	0.0786638
cg25592526	1.5166288	0.7472638	3.078113	0.2488055
cg18487309	0.146518	0.0409565	0.524154	0.0031445
cg05693982	0.1858408	0.0683742	0.505114	0.0009713
cg26889677	2.283378	0.7322483	7.120283	0.1547642
cg21282282	0.5827575	0.2476875	1.371108	0.216101
cg02059317	3.2242095	1.10568	9.401931	0.0320375
cg06983746	3.3080827	0.6358057	17.21188	0.1550908
cg02245467	0.3410819	0.1483181	0.784374	0.0113552
cg04365721	2.0109072	0.8903236	4.541886	0.0928588
cg20357228	1.3905478	0.1605637	12.04272	0.7646835
cg16028012	1.0863818	0.4440066	2.658126	0.8559858
cg17048233	0.531437	0.2375649	1.188834	0.12383
cg13313487	16147.973	0.3342991	7.8E+08	0.0782649
cg24650913	0.5863554	0.2290681	1.500919	0.2656308
cg15728692	0.1470693	0.0451646	0.478901	0.0014612
cg25246431	0.0827516	0.0150217	0.455863	0.004206
cg07609844	1.5756333	0.3260823	7.613477	0.5716038
cg20660269	2.2270517	0.8401294	5.903565	0.1074543
cg26027052	0.3259442	0.1200492	0.884967	0.0278238
cg14334425	0.2760626	0.0248259	3.0698	0.2949516
cg08738430	0.0836106	0.0004215	16.58481	0.3578741
cg18131365	7497.9267	0.554153	1.01E+08	0.0660127
cg08618463	0.3811512	0.1897191	0.765744	0.0067323
cg20640499	2.8934839	1.2894755	6.492755	0.0099809
cg17540765	0.3077329	0.0107975	8.770499	0.4904908
cg03705036	0.6476979	0.3053791	1.373744	0.2575476
cg23906067	0.1172826	0.029631	0.464217	0.0022639
cg02516101	0.2801912	0.0778586	1.00833	0.0515026
cg25031670	0.5649722	0.2528652	1.262307	0.1639068
cg19178324	1.4630817	0.4567723	4.686379	0.5217144
cg21174750	0.3054722	0.1367472	0.682378	0.0038288
cg11684450	0.663586	0.3331901	1.321607	0.2433418
cg21197233	1.4174221	0.5501271	3.652038	0.4700479

cg13636907	0.4137255	0.2114853	0.809365	0.0099455
cg19275832	2.00E-05	3.52E-16	1138676	0.3918489
cg16823277	0.215569	0.0881318	0.527279	0.0007726
cg14242024	518195.21	1.89E-05	1.42E+16	0.2832177
cg26062581	3.3531036	0.5814096	19.33801	0.1759415
cg19003629	7.02E-13	3.68E-23	0.013421	0.0205107
cg09245698	0.2452932	0.1050909	0.57254	0.0011562
cg08371532	0.2902854	0.1166057	0.722654	0.0078608
cg18127619	0.1534903	0.0185486	1.270138	0.0821786
cg21715751	1.5094415	0.5857294	3.889874	0.3939443
cg16807961	1.309599	0.5635539	3.043275	0.5306992
cg00018184	0.215703	0.066427	0.700435	0.0106962
cg09628488	1.1543641	0.1780398	7.4846	0.8803606
cg26749844	0.0157521	0.000257	0.965558	0.0480807
cg23031196	2.0661265	1.0275594	4.154386	0.0417253
cg08011255	0.5935397	0.2267625	1.553561	0.2879716
cg07041999	1.3686842	0.7467526	2.50859	0.3099681
cg20233073	2.2687732	0.8491816	6.06152	0.1022775
cg21317795	1.00E+25	8.66E-14	1.16E+63	0.1979723
cg13637733	2.3514289	1.11E-08	5E+08	0.9303543
cg15519065	71.452132	4.7879584	1066.301	0.0019642
cg21750887	0.5838368	0.2917842	1.168211	0.1283516
cg00028318	3.1263865	0.1335817	73.17091	0.4785805
cg10061342	1.706921	0.0620441	46.95985	0.751874
cg08821431	0.2951537	0.1039207	0.83829	0.0219544
cg12793681	0.0004473	1.63E-06	0.12257	0.0070834
cg25625514	1.1465033	0.4078221	3.223145	0.795451
cg15256539	2.75E+26	3.09E-11	2.44E+63	0.1607818
cg06885583	0.3444323	0.1368031	0.867185	0.0236697
cg11512009	0.7157288	0.2650702	1.932574	0.5092956
cg11301254	0.1024096	0.018339	0.57188	0.0094104
cg08754149	1.3347186	0.5503018	3.237267	0.523027
cg22239963	3.7070455	0.5529304	24.85338	0.1771357
cg27494254	2.5717813	0.6710043	9.856955	0.1682197
cg12121643	2.9458243	0.7719555	11.24143	0.1138403
cg15797314	1.0516854	0.3581062	3.088587	0.926951
cg08079908	1.6091628	0.6597328	3.92493	0.2956995
cg20383637	0.3458551	0.1161203	1.030102	0.056559
cg12421138	1.8332465	0.8901597	3.775494	0.1001146
cg15006175	2.7092506	0.7748924	9.472332	0.1186118
cg19202020	660511735	4.58E-05	9.52E+21	0.1889522
cg13419986	0.3542237	0.1144467	1.096357	0.0718008
cg26272623	2.2317208	0.9246161	5.386644	0.0741594
cg18651470	1.8206114	0.9622528	3.444652	0.0655201
cg21527584	2.1374844	0.628048	7.274666	0.2241316
cg00632486	0.3237888	0.1287469	0.814304	0.0165512
cg17470231	0.513947	0.1990396	1.32708	0.1690421
cg17526483	4.0268029	1.2475685	12.9974	0.019809
cg15972506	1.9473282	0.6957308	5.45051	0.2044012
cg14378348	0.2913246	0.0962751	0.881536	0.0290239
cg21849844	0.0570542	0.0067746	0.480497	0.0084353
cg05294112	1.7974175	0.8510461	3.796163	0.1242592
cg11433659	0.3931807	0.1762454	0.877135	0.022597
cg03436397	2.0462672	0.9034989	4.634438	0.0860407
cg01717524	1.2635945	0.2874521	5.554564	0.7567923
cg01020346	0.4576974	0.1278139	1.639	0.2298216
cg17028658	0.1756836	0.0758043	0.407163	5.01E-05
cg19472956	4.2022688	0.9912842	17.81433	0.0514048

cg04678916	0.3931593	0.193245	0.799887	0.0099916
cg12444761	0.0034707	1.14E-05	1.052749	0.0520967
cg09989886	0.2641008	1.52E-07	457957.9	0.8558592
cg03601828	0.0074316	0.0001079	0.511904	0.0232043
cg09926027	2.0198828	0.8329783	4.897999	0.1198028
cg15190445	1.9424415	0.9059258	4.164887	0.0879912
cg27494383	0.4523932	0.1806523	1.132892	0.0903488
cg00992153	0.0999561	0.0130701	0.764432	0.0265032
cg15468976	0.5122001	0.2379759	1.102419	0.0871454
cg06579154	1.2484203	0.5913582	2.635549	0.5605691
cg19010166	1.3383932	0.5587387	3.205964	0.5131317
cg13487026	5.2748309	1.1979882	23.22547	0.0278908
cg12042060	0.5258711	0.2301079	1.201786	0.1274877
cg00418150	4.7784941	1.4760503	15.46967	0.0090651
cg15096829	0.1806842	0.0693362	0.470848	0.000463
cg03198372	0.059339	0.0106794	0.329712	0.0012465
cg08862742	0.186817	0.0733588	0.475752	0.0004356
cg18765753	0.1787386	0.0056179	5.686781	0.329381
cg12956598	4.6041792	0.2428355	87.2956	0.3090823
cg08956463	2.3318193	0.6619659	8.21399	0.1875598
cg14120088	8.75E-09	5.88E-18	13.02672	0.0851134
cg27662505	0.4325094	0.2182764	0.857007	0.0162954
cg26716103	0.185938	0.0459903	0.751743	0.018259
cg25772221	2.1022458	0.8976966	4.923086	0.0870106
cg03784054	0.0457271	0.0015783	1.32486	0.0724656
cg08306303	9.6890156	0.6924649	135.5694	0.0916084
cg05500919	0.7668643	0.3840196	1.531382	0.451905
cg00483825	1.599671	0.6910731	3.70286	0.2726067
cg19725418	0.0008498	5.23E-06	0.138082	0.0064836
cg21235532	6.5308145	1.4291378	29.84425	0.0154969
cg01008894	1.1323919	0.0800807	16.01273	0.9267059
cg14423177	1.1961706	0.2715781	5.268555	0.8128161
cg07706352	1.9637453	0.6702174	5.753798	0.2185472
cg01574513	1.5176158	0.7184756	3.205617	0.2742328
cg03547640	0.0053123	1.97E-09	14330.19	0.4881431
cg06843240	9.11E-06	1.79E-15	46269.27	0.3087406
cg17333042	0.9779823	0.394143	2.426656	0.9617032
cg06595595	2.2116858	0.7833874	6.244107	0.1338875
cg06626599	2.2893533	0.7455534	7.029864	0.147899
cg15107349	11239705	55.986792	2.26E+12	0.0091583
cg24257776	0.3357821	0.1528651	0.737576	0.0065657
cg06167664	1.2678728	0.6513853	2.46782	0.4848816
cg22808692	6.56E-26	1.00E-56	428709	0.1092139
cg15204740	0.2645376	0.0756769	0.924723	0.0372943
cg07810733	0.8392322	0.3245599	2.170048	0.7176568
cg04987734	1.2409125	0.3130596	4.918756	0.7587056
cg10642449	4675.7401	0.000136	1.61E+11	0.3398743
cg00017475	10.746066	1.0572204	109.2279	0.0447503
cg16924658	0.0024527	2.64E-06	2.277731	0.0847312
cg25191611	1.7589207	0.7159095	4.321499	0.2182213
cg20741105	1.3408972	0.6785443	2.649798	0.3986302
cg20587808	1.3257534	0.7016563	2.504962	0.3850757
cg21432763	3.1691873	1.2317327	8.154162	0.0167471
cg09487733	191098821	0.0007122	5.13E+19	0.1555488
cg27023929	0.2541371	0.0765743	0.843437	0.0252109
cg03376308	0.4446013	0.1940718	1.018542	0.0552984
cg24345747	1.6072911	0.5879134	4.394158	0.3550668
cg08985530	0.7189508	0.3380088	1.529221	0.3915046

cg21996247	0.44164	0.1981803	0.984184	0.0456129
cg24038105	1.268846	0.4306997	3.738034	0.6657902
cg26192612	0.1798057	0.0447063	0.723166	0.0156746
cg22633280	0.0933421	0.0203786	0.427545	0.0022557
cg07857119	5.061043	1.0513178	24.36386	0.0431373
cg03661409	0.2118741	0.0942357	0.476365	0.0001741
cg06976003	76910.927	2.05E-10	2.89E+19	0.5111628
cg16723982	0.6495986	0.2200382	1.91775	0.4347724
cg04784699	0.3892006	0.1613588	0.938759	0.0356724
cg03269060	0.4813825	0.2041829	1.13491	0.0947702
cg01580568	0.1631095	0.0032629	8.153771	0.3635891
cg11436143	6.8038432	0.2992461	154.6964	0.2289685
cg24598973	15.973274	1.3736283	185.7456	0.0268587
cg16015474	0.3041165	0.0887036	1.04265	0.0582879
cg23678364	2.009229	0.6565821	6.148509	0.2214337
cg09556940	2.3105927	0.9162169	5.827046	0.0759708
cg12668043	1.4692522	0.6382382	3.382283	0.3657726
cg20435238	0.5108543	0.2596842	1.00496	0.0516989
cg26198107	1.3343361	0.465392	3.825706	0.591469
cg02567958	3.2121405	0.8920081	11.56699	0.0742383
cg06905453	0.5980087	0.3137676	1.139743	0.1181788
cg21860285	0.5362259	0.2354281	1.221342	0.1378429
cg00647403	16115.174	6.241441	41608797	0.0156573
cg20140034	8.2524763	2.237591	30.43602	0.0015271
cg08843517	0.399456	0.1333923	1.196209	0.1010436
cg00088079	5.6495867	0.3550612	89.89388	0.2200031
cg16657244	0.1724491	0.0437577	0.679622	0.0120076
cg06057566	2.87E-07	1.55E-14	5.300506	0.0776323
cg16575444	0.2731906	0.0068217	10.94052	0.4906919
cg07426533	1.5312262	0.6092876	3.848189	0.3648382
cg26072123	1.81E-09	1.10E-19	29.71864	0.0934721
cg17787387	0.3401216	0.165496	0.699006	0.0033432
cg15007959	0.4107341	0.1808691	0.932733	0.0334721
cg25027603	1.3856874	0.338234	5.676927	0.6502903
cg23552977	0.9310481	0.3535258	2.452015	0.8850232
cg26922780	6.7777095	1.540254	29.82453	0.0113628
cg09350895	0.2419854	0.0449976	1.301336	0.0983117
cg05044291	0.9374185	0.3488848	2.51875	0.898029
cg13379763	3.9795323	0.2646805	59.83319	0.3179115
cg13321259	1.1281902	0.4421818	2.878484	0.8007398
cg04109941	3.2876596	1.3462963	8.028475	0.008982
cg06264108	16.668156	1.3077453	212.4477	0.0302672
cg21123519	0.2483797	0.1084707	0.568748	0.0009842
cg16856213	27.509771	1.3331773	567.6571	0.0318601
cg07546293	0.0868225	0.0161353	0.467184	0.0044229
cg23476877	0.2041684	0.0750709	0.555272	0.0018556
cg18062778	83.551101	0.951172	7339.142	0.0526182
cg19056418	3.8286925	1.2932626	11.33481	0.0153352
cg06965300	2.277471	0.7155409	7.248886	0.1635183
cg02473395	2.37E+24	26037722	2.16E+41	0.0048481
cg01024247	0.1420856	0.0505107	0.399684	0.0002174
cg03450734	0.2333349	0.0818924	0.664838	0.0064481
cg07035145	1.3164047	0.6349913	2.729047	0.4598772
cg17074431	0.2099755	0.0597971	0.737322	0.0148721
cg10529796	0.3323789	0.1394459	0.792248	0.0129387
cg12113316	0.1248023	0.0222343	0.700523	0.0180617
cg19375210	0.0505405	0.0031631	0.807533	0.0347589
cg12497190	0.0464285	0.0013915	1.549128	0.0862742

cg16188984	2.0589969	0.9252031	4.582202	0.0768121
cg16925499	0.0128553	0.0001115	1.482489	0.0722679
cg21416022	0.1897454	0.0334836	1.075252	0.0603834
cg14065841	5.97E-39	1.91E-61	1.87E-16	0.0008674
cg00231274	1.112474	0.4273888	2.89572	0.8271389
cg03879320	0.2435101	0.1061771	0.558475	0.0008514
cg26169156	0.10808	0.022268	0.524578	0.0057728
cg10503563	5.957E+15	2.42E-12	1.46E+43	0.2589852
cg02483204	0.4391193	0.189416	1.018001	0.0550633
cg05520811	2.8918674	0.8378101	9.981853	0.0929581
cg11206526	13.860534	1.7602906	109.1379	0.012523
cg09640621	0.4306202	0.1595605	1.162153	0.0962531
cg25526759	0.0123938	0.0004221	0.363949	0.0108935
cg24673769	5.2882172	0.0988926	282.784	0.4120245
cg26672794	0.4548439	0.1996019	1.036478	0.0608334
cg25334575	1.3594244	0.6172001	2.994223	0.445957
cg00495775	0.3273031	0.0978588	1.094714	0.0698221
cg08983279	0.338158	0.1397	0.818546	0.0162217
cg26511972	6.798719	1.2781709	36.16307	0.02459
cg16693012	4.9214604	0.1747784	138.5799	0.3493983
cg01255458	15.995588	0.001391	183941.8	0.5611505
cg07759857	0.4832927	0.0203017	11.50505	0.6530084
cg23908698	4.72E-17	1.63E-33	1.364277	0.0519073
cg12616941	0.4792102	0.2325427	0.987528	0.0461538
cg04344021	4.1439709	1.2493994	13.7446	0.0201284
cg00093095	1.5054178	0.6103147	3.713302	0.3745219
cg18866212	0.2495922	0.1086877	0.573168	0.0010674
cg24042578	0.3139954	0.1116096	0.883375	0.0281683
cg25353384	0.234424	0.0711007	0.772913	0.0171656
cg09456329	2.66E-20	4.30E-34	1.64E-06	0.0054002
cg24858658	7040317.2	229.34261	2.16E+11	0.0027805
cg05170921	1.9387795	0.0701951	53.5488	0.695783
cg27252325	9.4866679	0.6549271	137.4151	0.0990155
cg08893853	0.3356602	0.1536725	0.733168	0.00617
cg00040569	8.83E-35	6.63E-63	1.18E-06	0.0176343
cg08122156	1.505849	0.6906021	3.283484	0.3033775
cg13473086	0.3358073	0.1569151	0.718647	0.0049378
cg02145310	1.1340525	0.2286448	5.624773	0.8776365
cg01765545	0.6943117	0.358772	1.343663	0.2787891
cg06962067	5.5701162	0.4146837	74.81894	0.1950402
cg04172640	0.4592061	0.1760557	1.197747	0.1115945
cg05276108	0.4974699	0.2085713	1.186531	0.1154127
cg23552779	1.43E+26	5.0404285	4.07E+51	0.0440037
cg27418894	0.2577674	0.064901	1.023775	0.0540328
cg02898499	0.3448619	0.1237639	0.960941	0.0417336
cg00286986	8350892.5	0.006483	1.08E+16	0.1364404
cg15832311	0.0012896	1.84E-06	0.901798	0.0464926
cg23648263	5.8234255	0.8054323	42.10445	0.0808823
cg20448594	2.7482535	0.5230639	14.43972	0.232339
cg13623638	3.023285	0.695857	13.13524	0.1399043
cg14769207	0.1953269	0.0441261	0.864627	0.0314285
cg08414643	4.455533	1.5994469	12.41165	0.0042569
cg17062109	2.4063526	0.8875025	6.524526	0.084445
cg23716141	1.8085079	0.562445	5.815148	0.3200868
cg09975995	0.4696243	0.2196884	1.003908	0.051188
cg19820919	0.1787733	0.0619097	0.516234	0.0014625
cg13975093	0.0285864	0.0004106	1.990374	0.1005861
cg07983363	2.2171621	1.0543369	4.662464	0.0357745

cg05196046	0.1270964	0.0153265	1.053955	0.0559693
cg07402411	1.7555203	0.6147609	5.01309	0.2931715
cg00235933	0.1409132	0.0187176	1.060846	0.0570908
cg21822834	0.0925983	0.0262878	0.326176	0.0002124
cg20696451	0.5286934	0.2305279	1.212507	0.1323332
cg26655764	2.2750411	0.5340965	9.69078	0.2662562
cg14144728	1.5895641	0.5940422	4.253425	0.3560664
cg23536372	6.9498627	1.1823714	40.85061	0.0319257
cg21840976	4.1988283	1.5168167	11.62313	0.0057462
cg15055149	2.2399892	0.6237872	8.043691	0.2163043
cg20148210	0.6212734	0.1753553	2.201134	0.4608155
cg26383138	0.3704221	0.1434347	0.956621	0.0402106
cg17976191	1.5213854	0.7824624	2.958115	0.2161299
cg21803535	0.1243966	0.0071841	2.153998	0.1519818
cg12255774	19.488197	0.6999379	542.605	0.080159
cg09776041	1.627001	0.5993171	4.416914	0.3394619
cg26200347	1.6983758	0.7210497	4.000391	0.2256041
cg05166248	2.4882136	0.9684392	6.392974	0.0583108
cg14330675	1.5631003	0.7506152	3.25504	0.2326803
cg11905100	0.2914195	0.0645332	1.315994	0.1089403
cg20777266	3.6581718	0.1095887	122.1131	0.4686771
cg08597205	0.593747	0.0309795	11.37964	0.7293552
cg05629195	0.350849	0.1571514	0.783289	0.0105874
cg22335246	19807234	22.7915	1.72E+13	0.0160381
cg06207120	3.2115093	0.6750679	15.27815	0.1426002
cg17692230	0.5434171	0.23522	1.255429	0.153431
cg09793883	2.3537362	0.8021454	6.906571	0.1191017
cg10281768	2.6693927	0.2190999	32.52242	0.4414584
cg21287322	2.8764718	0.3442785	24.03313	0.3293193
cg02300576	0.3708579	0.1384897	0.993111	0.0484136
cg24035112	0.7476682	0.4029463	1.387301	0.3565204
cg24459498	1.6917072	0.0327152	87.47846	0.7939729
cg10947826	1.7112697	0.8235705	3.55579	0.1499331
cg19529732	2.6000724	0.7389753	9.148312	0.1365678
cg14549524	2.0435673	0.9164629	4.556832	0.080679
cg00333843	1.766468	0.3670816	8.500586	0.4778361
cg04167205	0.3569855	0.0185968	6.852736	0.4944326
cg12461287	0.1701148	0.0363426	0.796285	0.0244979
cg14990430	0.0021225	8.76E-06	0.514274	0.0279943
cg07222863	1.6030077	0.8114125	3.166865	0.1743413
cg25608871	254.02702	0.0033533	19243417	0.3340467
cg26804411	3.5646217	0.8694788	14.61396	0.0774501
cg09282258	2.2671003	0.8678699	5.922251	0.0947804
cg02856952	11011841	1.65E-10	7.36E+23	0.4120384
cg21220965	0.1886532	0.0441115	0.80682	0.0244823
cg18986048	0.5652	0.1413764	2.259578	0.4196646
cg15830544	0.2729015	0.0714559	1.042255	0.0575078
cg22273555	0.1236148	0.0289084	0.528588	0.0048034
cg07688613	3.08E-05	4.36E-13	2172.434	0.2598809
cg13752239	6.3965817	0.0448242	912.8172	0.4634374
cg11566561	2.1075955	0.2370236	18.74058	0.5036748
cg07402396	5.4360434	0.1740337	169.7979	0.33495
cg23001650	0.2461102	0.0677816	0.893608	0.0330941
cg01291336	0.3777755	0.1169487	1.220316	0.1037055
cg06643271	2.4977781	1.0593924	5.889126	0.0364559
cg14031452	19.98128	1.7027806	234.4703	0.0171443
cg25028639	0.8297497	0.3224255	2.135329	0.6987735
cg12428416	2.8122281	0.4725551	16.73588	0.2558596

cg11340260	0.1340597	0.0457492	0.392837	0.000249
cg27185793	0.228165	0.0534136	0.974645	0.0460833
cg14135596	0.4276013	0.1925356	0.949658	0.0369022
cg09243445	2.6369198	0.8640173	8.047693	0.0885277
cg09183124	0.4998119	0.2373941	1.052309	0.0678897
cg22342100	0.1870058	0.0545927	0.640583	0.007609
cg17756521	3.9464905	0.6667657	23.35871	0.1302282
cg26495109	0.1749036	0.0349095	0.876303	0.0339587
cg17786129	0.3737686	0.1658169	0.842514	0.017634
cg03807298	2.0121518	1.0286922	3.935827	0.0410917
cg02736016	3.9443153	0.8515378	18.27003	0.0793465
cg03750478	2.5182711	1.0416589	6.088067	0.0403075
cg19392142	0.0474358	0.0031321	0.718423	0.0279164
cg23181159	0.3237893	0.1551899	0.675556	0.0026538
cg15840985	1.4738182	0.3536275	6.142452	0.5943257
cg22176097	1.77E-06	5.27E-18	596945.5	0.3281318
cg06627151	1.2495085	0.1365981	11.42967	0.84364
cg02664162	3.08E-09	1.14E-16	0.083225	0.0247857
cg02316066	2.7477324	1.0957978	6.889988	0.0311612
cg08423533	1.476803	0.5967397	3.654771	0.3990666
cg18720486	1.7158068	0.4054594	7.260883	0.463257
cg11258381	4.2746361	1.2983895	14.07321	0.0168724
cg00766322	3.3060187	1.0228808	10.68527	0.0457426
cg22702351	3.5341707	1.1337085	11.01726	0.0295333
cg02608145	1.5819293	0.5660909	4.420669	0.3817127
cg08125436	0.4261884	0.1608299	1.12937	0.0862932
cg18154567	0.5593777	0.2762967	1.13249	0.1064756
cg14450231	0.3497109	0.0686863	1.780526	0.2057891
cg27391982	0.6964596	0.3044404	1.593271	0.391571
cg26700716	1.827806	0.7373644	4.530833	0.192862
cg09169673	0.4939382	0.2021376	1.206975	0.1217917
cg11596009	0.1282369	0.0391504	0.420039	0.0006917
cg02005771	1.8502631	0.8173953	4.188272	0.1398825
cg04914982	604.6787	0.0005467	6.69E+08	0.3670383
cg12640482	0.2171881	0.0648154	0.727769	0.0133228
cg25581090	0.3124988	0.1395506	0.699786	0.0046861
cg21825027	0.0981981	0.0154551	0.623926	0.0138943
cg09985739	0.4351736	0.1487486	1.273128	0.1287431
cg16573726	0.6098559	0.2945758	1.262576	0.1828657
cg09973676	2.3606131	0.7926645	7.030079	0.1229164
cg27614178	0.2496691	0.0583451	1.068378	0.061374
cg06342072	0.3275729	0.1082713	0.991066	0.0481717
cg13236257	2.9350043	0.7795314	11.05055	0.1114384
cg24367777	0.5074647	0.2152383	1.196444	0.1211157
cg14136186	0.1886834	0.0733434	0.485408	0.0005419
cg22325958	0.4181051	0.1747683	1.000249	0.0500655
cg22707455	2.2820902	0.7572723	6.87723	0.1426556
cg00150848	7.7648418	0.2361082	255.3607	0.2501295
cg15004938	2.8001191	0.1340783	58.47827	0.5066463
cg17124387	0.3448499	0.1619782	0.734182	0.0057549
cg03619256	3.9779268	0.7132588	22.18536	0.115346
cg15130855	0.0151606	0.0004618	0.497694	0.0186887
cg26252045	7.493681	0.5741081	97.81304	0.1243948
cg15663074	0.2489209	0.0660744	0.937756	0.0398857
cg00215017	1.6673652	0.7579378	3.667988	0.2037438
cg06801163	3.7321077	0.5086659	27.38267	0.1952568
cg19429051	3.3291734	0.9146444	12.11771	0.0680597
cg10091752	2.911751	1.0408057	8.145895	0.0417341

cg27327334	0.1109007	0.0049812	2.469099	0.1648165
cg10370858	3.4785292	1.1872654	10.19163	0.0230299
cg00982799	0.3323441	0.063548	1.738098	0.1918712
cg08417719	0.5803966	0.272488	1.236239	0.1584691
cg11799704	0.1513421	0.0105079	2.179739	0.1653138
cg27004182	2.7100293	1.0971416	6.693994	0.0307024
cg17163404	1.9865219	0.6799034	5.804162	0.209583
cg06491116	1.1341981	0.5873727	2.190101	0.7076016
cg21806985	0.4001316	0.1681036	0.95242	0.0384393
cg18427465	0.1654377	0.0503948	0.543104	0.0030122
cg22890545	2.2106124	0.4016164	12.16785	0.3619728
cg19430622	3.307915	0.9987489	10.95601	0.05024
cg03691429	0.2321879	0.0353469	1.525202	0.1284033
cg17392201	0.5163733	0.2216818	1.202812	0.1255369
cg27630192	0.1575002	0.0261787	0.947577	0.0435104
cg14434755	2.3478782	1.0117025	5.448768	0.0469168
cg01330991	459.65892	1.3262692	159308.8	0.0399178
cg20150659	3.3457829	0.9292076	12.04711	0.0646551
cg23861715	0.2228689	0.016497	3.010878	0.258412
cg12708994	1.8282588	0.6405322	5.218364	0.2595201
cg12741994	1.7980745	0.8479029	3.813021	0.1260715
cg19313408	2.6939527	0.3635685	19.96152	0.3321386
cg18255166	0.3650091	0.1058355	1.258856	0.1105952
cg16988986	0.3101153	0.1055352	0.911274	0.033262
cg01209642	2.1556506	0.6703614	6.931828	0.1974451
cg23590273	2.1730169	0.9696645	4.869728	0.0594109
cg19777991	44.835639	0.3879612	5181.535	0.1165876
cg16077741	24879146	0.1549462	3.99E+15	0.0773062
cg16109731	0.5701818	0.2231831	1.456684	0.2404208
cg05947761	1.8437834	0.6020468	5.646632	0.2839932
cg04874562	0.6583796	0.2094127	2.069902	0.4745018
cg14111974	6.1939749	1.3254456	28.94523	0.0204427
cg25577821	3.9568182	1.5540322	10.0747	0.0039204
cg07464605	1.2214817	0.1465756	10.17916	0.8532779
cg10503133	2.8759114	0.6410682	12.90169	0.1677751
cg11047382	81.667839	0.0605014	110239.3	0.2312319
cg16908123	0.4470545	0.1475245	1.354743	0.154669
cg11944933	2.0338263	0.8142447	5.080106	0.1285145
cg21583668	0.1876557	0.0525683	0.669884	0.0099643
cg21533897	1.8203508	0.7043137	4.704831	0.2162947
cg13579295	0.2071011	0.07032	0.609938	0.0042758
cg09046939	1.1761716	0.4268145	3.241173	0.7537155
cg26682641	0.7181571	0.356865	1.445223	0.3534815
cg03672752	0.1168273	0.0052893	2.58042	0.173938
cg16019273	0.2958678	0.1339119	0.653697	0.0026037
cg06388946	1.40E-12	1.82E-21	0.001075	0.0089299
cg21512241	4.7285635	0.8306028	26.91938	0.0799797
cg10672201	2.0236003	1.0032127	4.081845	0.0489619
cg12070036	0.1034246	5.09E-10	21034765	0.8161853
cg27657537	0.0172327	5.45E-05	5.451194	0.1667884
cg22947154	0.2891017	0.0257126	3.25054	0.3148227
cg19369262	3.5714505	1.5954075	7.994985	0.0019608
cg26390965	250.15017	5.8908657	10622.4	0.0038872
cg07710266	2.2188187	0.4757031	10.34922	0.3104134
cg09555124	0.3231064	0.1538621	0.678515	0.0028399
cg26320006	1.5110235	0.254953	8.955344	0.6493549
cg03356172	1.1795597	0.6048382	2.300385	0.6279715
cg12604162	0.3409852	0.1155664	1.006096	0.0513008

cg06498809	2.993E+11	1.05E-20	8.56E+42	0.4745732
cg11274337	0.328891	0.1546162	0.699598	0.0038814
cg06555971	1.57E-05	3.27E-13	751.8186	0.2201871
cg21620979	0.4522076	0.1188076	1.721201	0.2445421
cg16468280	0.0210476	0.0001025	4.324008	0.1552994
cg13297150	4.75E+27	7091803.8	3.18E+48	0.0091951
cg12895304	1.1109078	0.1156936	10.66711	0.927386
cg20240860	0.0365525	0.0010499	1.272593	0.0677181
cg16372632	2.3280045	0.7355005	7.368595	0.150605
cg12464048	1.4912737	0.7089866	3.136727	0.2921536
cg06551828	2.4682566	0.1110118	54.87968	0.568039
cg11297227	0.056105	0.0129031	0.243954	0.0001224
cg01200186	0.4461918	0.2247727	0.885726	0.021064
cg05306029	0.599038	0.3043811	1.178938	0.1379616
cg16393207	0.2495148	0.1174747	0.529966	0.0003039
cg21001667	1.3213083	0.3620775	4.821773	0.6731363
cg06282863	0.4515919	0.1474653	1.382937	0.1638634
cg01821452	0.3040133	0.1487465	0.621353	0.0010958
cg01536956	0.1845831	0.0514587	0.662102	0.0095235
cg19900050	0.2666268	0.0692388	1.026734	0.0546545
cg09026253	2.0004367	0.8316901	4.811584	0.1215257
cg00779206	0.3104749	0.1357298	0.710196	0.0055958
cg23224142	2.6252607	1.0426	6.610391	0.0405105
cg05695429	2.4223558	0.7453066	7.873012	0.1412471
cg18261413	0.4176912	0.020881	8.355251	0.5679062
cg19803081	3410.2694	0.2718561	42779758	0.0911326
cg10053622	0.297424	0.0867677	1.019516	0.0537043
cg17316862	0.3271025	0.1267132	0.844396	0.020915
cg14094409	4.9806303	0.9665052	25.66637	0.0549541
cg09237846	0.5815593	0.2761185	1.224877	0.1537984
cg18288168	0.0015009	6.47E-06	0.347932	0.0192877
cg23963317	1.365987	0.7347464	2.539544	0.3242577
cg05560697	0.0030609	8.19E-09	1144.696	0.3765754
cg22494858	3.2973915	1.167632	9.31183	0.0242874
cg27009556	2.7381924	0.9451305	7.932976	0.0634556
cg13603214	7.6809623	0.1476414	399.598	0.3119337
cg16681830	1.63E-09	1.44E-15	0.001854	0.0044508
cg07430820	0.2215495	0.0711179	0.69018	0.0093348
cg02849956	0.7892584	0.2779729	2.240969	0.6566941
cg06873452	0.1782872	0.002517	12.62844	0.4276061
cg19187110	0.3946102	0.1602769	0.971551	0.0430993
cg26463200	1.8582975	0.6912136	4.995952	0.2194238
cg22243956	2.5768023	0.3398671	19.53678	0.3597665
cg12668403	0.3500803	0.1499839	0.817129	0.0152265
cg08030922	0.5511914	0.2532746	1.199536	0.133253
cg00597055	4311.4061	27.583414	673891.3	0.0011665
cg20227714	0.4045649	0.210682	0.776871	0.0065598
cg24825767	0.7005407	0.2436162	2.014469	0.5089949
cg04994761	2.3308787	0.9641683	5.634903	0.0602519
cg06943912	1.7339926	0.4933864	6.094069	0.3907144
cg09214616	2.5532488	1.0551193	6.178523	0.0376209
cg23217622	2.1380419	0.9563679	4.779775	0.0641297
cg14254080	1.9644894	0.7746493	4.981891	0.1549794
cg13447284	0.6220377	0.2029327	1.906696	0.4061366
cg13649209	0.1326528	0.020882	0.842677	0.03224
cg03420907	0.1471686	0.0132168	1.638714	0.1191625
cg15225991	0.2165674	0.0544006	0.86215	0.029977
cg11838876	0.3349624	0.0767257	1.462349	0.1457945

cg03203320	7.23E-15	1.80E-31	290.9256	0.0950883
cg13154880	2.1199064	0.7939377	5.660397	0.133752
cg20165946	2.9748695	0.508515	17.40332	0.2264232
cg13624163	1.622125	0.6879781	3.824671	0.2690032
cg04142080	0.2558326	0.0949516	0.689302	0.0070235
cg08873353	6.95E-13	8.07E-27	59.872	0.0872657
cg01176694	2.2919933	0.7941947	6.614541	0.1250693
cg22926670	0.4749317	0.1685049	1.338597	0.1590225
cg12545570	2.57E-23	1.28E-39	5.18E-07	0.0066157
cg14085177	1.7099099	0.7061993	4.140179	0.2344514
cg18736141	1.2452738	0.6043391	2.565955	0.5520668
cg02434121	1.2404613	0.5898266	2.608808	0.5699595
cg18955698	501.60375	14.787016	17015.36	0.0005439
cg13235059	1.1365716	0.4189673	3.083283	0.8014928
cg17140476	0.2829197	0.022633	3.53658	0.3272038
cg07050194	0.3486896	0.1711592	0.710359	0.003709
cg27028938	0.5548599	0.2535842	1.214072	0.1403699
cg12885484	0.6731968	0.3237493	1.39983	0.2893932
cg02711268	0.154467	0.0340145	0.701468	0.0155532
cg02382320	0.2286053	0.0591228	0.883929	0.032454
cg18813035	1.9277279	0.6426481	5.782534	0.2415756
cg20280170	0.6693107	0.320113	1.399434	0.2860042
cg18422587	1.3047309	0.5958908	2.85677	0.5058977
cg18593232	9.6627167	1.1817901	79.00565	0.0343649
cg25234117	0.1658061	0.0463209	0.593505	0.0057482
cg22115706	0.2355092	0.0834099	0.664964	0.0063256
cg22584718	0.471144	0.163189	1.360242	0.1641572
cg21163972	33.980494	0.303779	3801.033	0.1429408
cg00033666	2.4623375	0.7812521	7.760754	0.1239273
cg00471190	3.3919138	0.6805913	16.90454	0.1361147
cg09763439	0.5864335	0.2816454	1.221054	0.1537968
cg09478597	2.316E+15	1.37E-06	3.93E+36	0.1560365
cg05191313	0.298094	0.0946099	0.939225	0.0387301
cg08204867	0.3298033	0.1253416	0.867791	0.0246243
cg22673543	1.7716094	0.5008582	6.266444	0.3749446
cg09882629	0.0140265	0.0005642	0.348699	0.0092524
cg26295435	0.4123597	0.1412475	1.203848	0.1051094
cg05852452	5.7564019	1.2943523	25.60057	0.0215146
cg23251057	1.1938419	0.4358772	3.269862	0.7303573
cg17274072	1.035E+15	56.538047	1.89E+28	0.0264916
cg16134717	1.1630581	0.5207788	2.597464	0.7125239
cg24039962	0.4653	0.2015253	1.074327	0.0731274
cg01998606	0.1004737	0.0139931	0.721423	0.0223356
cg15720343	78.388826	0.0010089	6090476	0.4477475
cg15598244	0.0513905	0.0035283	0.748503	0.0298614
cg25403152	0.0955932	0.0062384	1.464814	0.0918255
cg00964355	2.1397413	0.5183921	8.832103	0.2929659
cg22247041	0.8566729	0.2482071	2.956758	0.8066436
cg13683361	0.6529992	0.32204	1.324084	0.2373515
cg23010077	1.5257724	0.0909412	25.59876	0.7690306
cg14024243	2.3917922	1.0262081	5.574571	0.0433951
cg06152856	0.3354309	0.1119111	1.005386	0.0511317
cg24617504	4.4154333	1.3789105	14.13874	0.0123825
cg07166409	0.1933142	0.0024325	15.36305	0.4616218
cg14366490	2.3262664	0.7600211	7.120217	0.1390902
cg14769991	9.8589536	1.7498632	55.54661	0.0094783
cg24759108	2.3634105	0.5350914	10.43879	0.2564259
cg01622304	1.7599639	0.747984	4.141096	0.1953744

cg11001247	1.283265	0.5290948	3.112427	0.5811335
cg20035679	0.3921712	0.1481246	1.038303	0.0595245
cg00673202	0.7494812	0.0840729	6.68137	0.7961329
cg08969549	0.4208176	0.1890534	0.936706	0.0339958
cg03048083	3.1658584	1.0607463	9.448687	0.0388597
cg26099902	0.2418833	0.1096154	0.533753	0.0004403
cg14180511	1.036276	0.1744359	6.156231	0.9687343
cg08610987	0.3486403	0.1151336	1.055731	0.0623182
cg04590721	2.4183493	0.734965	7.957405	0.1461616
cg25386234	3.575306	1.4846196	8.61016	0.0044946
cg10486488	4.2679849	0.7606997	23.94597	0.099121
cg09155852	4.56E-16	1.45E-27	0.000144	0.008925
cg13389508	3.8159275	1.0283131	14.16038	0.0453174
cg22353096	2.5885946	1.1452234	5.851105	0.0222626
cg01858698	1.3148212	0.6817524	2.535752	0.414062
cg00730561	0.2641864	0.124714	0.559636	0.0005097
cg20232748	14.928902	0.0131326	16970.97	0.4514251
cg04130108	1.4816371	0.6999908	3.136111	0.304123
cg21044968	0.4759122	0.225099	1.00619	0.0519185
cg17078980	2.9908212	0.565394	15.82085	0.197389
cg14410395	1.9504233	0.9227122	4.122793	0.0802338
cg24894158	0.8578942	0.054616	13.47559	0.9131421
cg07722220	0.021554	0.0008187	0.56748	0.021478
cg10681065	2.3181637	0.7119307	7.548324	0.1627549
cg12603560	0.4115838	0.1679399	1.008701	0.0522558
cg06912304	1.470487	0.5104285	4.236307	0.4750709
cg15558882	0.0745637	0.0076207	0.729555	0.0256857
cg18571792	0.2589093	0.0897135	0.747201	0.0124586
cg13964030	1.81409	0.7185839	4.579733	0.2074779
cg20101110	8.1413265	0.0119132	5563.688	0.5289048
cg01131038	1.3382591	0.5737049	3.121705	0.5001692
cg13483882	1.2200209	0.6334213	2.34986	0.5520892
cg17122213	4.3519832	1.0524224	17.99635	0.0423047
cg03871460	1.2646245	0.6167043	2.593261	0.5216831
cg24694879	1.8890159	0.8815353	4.047916	0.1019012
cg23850212	0.0210869	0.0012642	0.351736	0.0071954
cg18334915	1.0009591	0.3739623	2.679199	0.9984773
cg23298492	5.56E-09	6.80E-29	4.55E+11	0.4165199
cg24630419	5.119506	1.887296	13.88724	0.0013393
cg06648455	1.3504952	0.4938256	3.693282	0.5582957
cg23202316	1314.365	6.931208	249243	0.0072874
cg12297440	0.2661385	0.0993121	0.713203	0.0084887
cg00610310	0.1377101	0.0334737	0.566536	0.0060078
cg13432945	0.4751929	0.2032401	1.111042	0.0859843
cg22548735	2.2717705	0.5154228	10.01302	0.2782631
cg03986322	0.2166188	0.056422	0.831656	0.0258454
cg22528270	2.8345578	1.1081881	7.25032	0.0296791
cg07953201	2.1226307	0.8533581	5.279801	0.1054722
cg14528056	0.2767503	0.133662	0.573018	0.0005411
cg23740940	0.2651188	0.0477152	1.473072	0.129199
cg00511228	1141308.5	1.375043	9.47E+11	0.0448814
cg11754259	88240.456	41.106661	1.89E+08	0.0036215
cg07423943	0.2296976	0.0558014	0.945513	0.0415928
cg01471710	4.8172783	0.0408117	568.6155	0.5183594
cg11389756	0.3254002	0.1504331	0.70387	0.004344
cg04388548	2.1330873	0.8018789	5.67425	0.1291051
cg26037142	0.389155	0.1402259	1.079983	0.0699528
cg27262821	0.5777189	0.2677013	1.24676	0.1621109

cg05279622	0.2168198	0.0686404	0.684886	0.0091888
cg19016062	3.0001248	0.2628016	34.24922	0.3765248
cg00945209	6.7898228	1.2051754	38.2531	0.0298901
cg04684267	0.0875582	0.008938	0.857732	0.0364589
cg09797433	0.0543118	0.0158333	0.186302	3.62E-06
cg14966074	0.3295164	0.1202085	0.903272	0.0309517
cg24019564	2.2681636	0.6106823	8.424292	0.2212161
cg13200550	0.9523723	0.4970829	1.824671	0.8830524
cg01619825	2.7466318	0.7919237	9.526153	0.1113154
cg05213661	0.7491453	0.2415912	2.323009	0.6169267
cg14564965	0.2197876	0.0834005	0.579212	0.0021803
cg21386414	0.2525472	0.1031878	0.618097	0.0025826
cg17503287	647.12505	4.8937717	85572.2	0.0094003
cg00050692	2.2905137	0.8829483	5.941971	0.0883795
cg24925986	1.8106972	0.5005279	6.550332	0.3654647
cg17614703	3.8026501	0.2655141	54.46095	0.3253514
cg09890891	1.3522047	0.4228586	4.32404	0.610931
cg02921122	0.3298336	0.1138242	0.955774	0.0410235
cg13393721	0.1982009	0.0648701	0.605573	0.0045092
cg22833375	5.50E-06	3.79E-11	0.798159	0.0458096
cg11099899	40.658472	3.2063067	515.5812	0.00425
cg12424921	2.4402247	0.3646908	16.32807	0.3576463
cg12103037	1.9649306	0.9176742	4.207323	0.0820696
cg12422351	0.2716915	0.083374	0.885364	0.0306203
cg25924840	1.16E-06	5.66E-13	2.378243	0.0653103
cg24222175	1.8778194	0.8381984	4.206887	0.1257471
cg22663389	0.3535618	0.1766337	0.707713	0.003321
cg19513374	3.8957146	0.6097895	24.88825	0.1506618
cg22183049	1.7570526	0.8212294	3.759283	0.1463802
cg21929761	0.7140883	0.1434174	3.55551	0.6809544
cg16051195	2.4682732	0.8379643	7.270444	0.1011641
cg13397939	0.3926119	0.1687292	0.913559	0.0300235
cg04728310	0.2458685	0.0949773	0.636482	0.0038408
cg08651573	1.3528809	0.5400879	3.388868	0.5188603
cg15000222	0.2776865	0.1293108	0.596313	0.001017
cg27582240	0.2287754	0.0641389	0.816013	0.023006
cg08918658	1.8804847	0.8385301	4.217169	0.125376
cg19758859	0.4204986	0.2146194	0.823873	0.0115849
cg10180415	1.1468865	0.6284302	2.09307	0.6552256
cg17771150	3.2504835	1.2149484	8.696372	0.0188879
cg11835219	2.2569151	0.6322563	8.056331	0.209915
cg10100437	3.2164639	0.6338789	16.32116	0.1585946
cg05879505	1.2651384	0.5540424	2.888904	0.5766714
cg22160769	0.2978337	0.1484661	0.597476	0.0006497
cg03189453	0.8441306	0.3514024	2.027751	0.7047169
cg01595397	0.4256573	0.1218772	1.486612	0.1807101
cg12328660	0.2867595	0.1081774	0.76015	0.0120281
cg18170491	8048239.5	2.13E-06	3.05E+19	0.2818981
cg26516759	0.2235985	0.0455947	1.096538	0.064839
cg00578304	0.3885072	0.1380394	1.093441	0.0733309
cg15812020	2.7865544	0.618255	12.55936	0.1821969
cg25262866	77.103605	1.45E-09	4.1E+12	0.7302292
cg06691963	0.6846111	0.3031342	1.546155	0.3619918
cg11126068	0.0103624	7.48E-14	1.44E+09	0.7270131
cg23454205	0.3077707	0.0920037	1.029554	0.0557878
cg07210466	0.3222832	0.0582163	1.784148	0.1946706
cg12486493	0.5665527	0.0616205	5.209014	0.6157
cg22806527	3.0271002	0.6620994	13.83982	0.153219

cg02217815	1.9456521	0.3983751	9.502506	0.410758
cg05923785	1.1741411	0.6476188	2.128733	0.5969257
cg04747036	0.3358534	0.1287719	0.875948	0.0256969
cg01278041	0.3263883	0.0252002	4.227322	0.3915466
cg11393119	36.650119	5.83E-13	2.3E+15	0.8241822
cg11815057	1.4465053	0.3644361	5.741411	0.5996938
cg14278073	7.0817814	1.4731508	34.04378	0.0145434
cg06479433	0.0476391	4.21E-05	53.95206	0.3961984
cg05827312	1.0346314	0.4621402	2.316315	0.9340143
cg09160359	0.2521354	0.0673281	0.944216	0.0408383
cg27064287	1.4091986	0.6732166	2.94978	0.3627628
cg04640971	2.2169082	1.0777778	4.560014	0.0305015
cg14595252	1.0822914	0.494348	2.369494	0.8432019
cg22919728	0.0999073	0.0238442	0.418613	0.0016258
cg03625287	0.1984094	0.0414601	0.949499	0.0428841
cg24856732	863.58413	0.5721186	1303537	0.070228
cg07429284	0.0943425	0.0257189	0.346069	0.0003707
cg00221709	2.399425	0.5721415	10.06262	0.2314696
cg23564432	0.4115425	0.1505041	1.125333	0.0836488
cg11573318	0.5754893	0.2202374	1.503777	0.2595453
cg17815035	0.2687325	0.1035987	0.697085	0.0068935
cg27427318	1.7859979	0.4839695	6.590887	0.3839798
cg23836542	0.3314728	0.0963572	1.14028	0.0798234
cg13736279	2.2221126	0.7262847	6.79869	0.1616822
cg27094076	1.2243004	0.6885262	2.176986	0.4907485
cg16215071	5.0482387	1.5123303	16.85129	0.0084743
cg03090982	7.22E-08	1.27E-13	0.041001	0.0149963
cg24700760	2.66E-12	2.58E-24	2.736414	0.0589409
cg14740251	0.4762783	0.1919783	1.181598	0.1095946
cg17852876	0.5636938	0.1750491	1.81521	0.3366807
cg27258394	0.5039525	0.2264078	1.121729	0.0932327
cg20342184	1.5761476	0.7676637	3.236106	0.2151238
cg04111344	0.5239455	0.2568282	1.068881	0.0755933
cg10961055	1.13E-08	2.25E-27	5.67E+10	0.4048989
cg00713939	0.2883395	0.0348619	2.384829	0.2486289
cg08109345	8.8380031	1.1810186	66.13808	0.0338391
cg24377826	0.4864025	0.2409324	0.981966	0.0443533
cg22264409	0.8160142	0.3105789	2.143994	0.679947
cg01432609	0.2217546	0.1001972	0.490783	0.0002024
cg08371685	18523.017	4.03E-10	8.51E+17	0.540381
cg00090782	0.2302303	0.0544114	0.974172	0.0459863
cg27023727	0.683456	0.2855782	1.635672	0.3926554
cg26351481	0.5133589	0.2525716	1.043416	0.0653987
cg08626445	1.3534757	0.65774	2.785138	0.4110287
cg10501602	2.2389629	0.7229607	6.933925	0.1622623
cg14482745	6.7482934	0.4533348	100.4544	0.1658187
cg15035145	0.0536022	0.0009018	3.185971	0.1603227
cg09861057	1.7368057	0.6783966	4.446505	0.2497445
cg26532621	1.2743428	0.5125672	3.168267	0.6018679
cg08455073	0.49252	0.1915732	1.266232	0.1415572
cg14422338	0.0039445	7.71E-07	20.17152	0.2039252
cg00257893	2.6518875	0.8104417	8.677376	0.1068599
cg26418691	49840318	4.06E-07	6.11E+21	0.2842312
cg03934689	0.0716459	0.0075941	0.675939	0.0213359
cg08288561	0.9984294	0.4041262	2.466708	0.9972823
cg12050271	0.6776366	0.3152069	1.456793	0.3190037
cg09367855	0.1614658	0.0526926	0.49478	0.0014153
cg05713242	1.6341241	0.7720964	3.458586	0.199203

cg14605021	2.2400811	0.9699	5.173691	0.0589717
cg11071207	2.9310516	1.0526356	8.161479	0.0395765
cg02757456	0.1445317	0.0426273	0.490047	0.0019035
cg22088670	0.9912898	4.61E-12	2.13E+11	0.9994757
cg12542080	0.7680823	0.3566941	1.653939	0.50016
cg20576726	3.0004568	1.0506091	8.569068	0.0401529
cg10649156	0.2373897	0.0719297	0.783457	0.0182478
cg14839935	1.6481041	0.7989192	3.399902	0.1762708
cg03139377	0.1456043	0.04262	0.497433	0.0021123
cg01324548	3.1496592	0.5253677	18.88268	0.2092737
cg11018018	1.0249882	0.526282	1.99627	0.9421496
cg08621473	0.0040357	3.58E-05	0.45501	0.0222198
cg18844900	2.3946467	1.1084302	5.173382	0.02629
cg23038277	3.3943826	0.159469	72.25124	0.4334583
cg09746044	1.9241612	0.1050153	35.25578	0.6591414
cg03002136	0.0646505	0.0075037	0.55702	0.0126846
cg25537119	1.7249866	0.3578595	8.314936	0.4968737
cg12652442	0.1886299	0.0775101	0.459053	0.0002371
cg23863670	0.5020824	0.2260255	1.115302	0.0906496
cg09352815	0.4304666	0.1693735	1.094041	0.0765427
cg03122624	2.0670967	0.9404897	4.54326	0.0707214
cg26339348	376.56171	11.063303	12817.03	0.0009825
cg19471856	0.5056079	0.2528449	1.011052	0.0537461
cg04295991	0.2492643	0.0400554	1.551167	0.136401
cg20528837	0.0867101	0.0141127	0.532757	0.0082965
cg07478467	0.3621588	0.1700916	0.771108	0.008437
cg14180029	0.4090441	0.101033	1.656064	0.2102291
cg14019523	35.74554	1.669967	765.131	0.0221356
cg07648207	1.68E-09	2.60E-15	0.001092	0.0030889
cg25666403	2.4301419	0.7203124	8.19865	0.1523772
cg15411034	2.0441138	0.7131363	5.859191	0.1832832
cg04759648	2.2902703	0.873319	6.006211	0.0920659
cg12575511	0.1623635	0.017983	1.465937	0.10539
cg02918577	1.5733619	0.8112667	3.051361	0.1798998
cg18877506	1.1818243	0.582143	2.399254	0.643789
cg05942181	2.9028992	0.0477481	176.485	0.6110897
cg22343980	1.06E-35	1.70E-74	6553.591	0.0772044
cg22775789	0.2336849	0.0855571	0.638271	0.0045714
cg14883961	0.3431098	0.1148177	1.025315	0.0554665
cg22912331	0.1955987	0.0592526	0.64569	0.0074093
cg25079544	0.1749061	0.0433601	0.705537	0.0142808
cg22820316	2.4745617	1.2565558	4.873206	0.0087813
cg08935418	0.1319695	0.0320384	0.543596	0.005049
cg10122479	372.28019	5.777465	23988.47	0.0053493
cg04322429	3.7203695	0.4597531	30.10561	0.2181151
cg26517584	0.1386712	0.0448939	0.428336	0.0005961
cg05133205	0.237114	0.0600364	0.936482	0.0400141
cg03627771	1.2541936	0.5198072	3.026125	0.6142617
cg19935065	0.4710528	0.2014406	1.101519	0.0824094
cg19178742	0.2073244	0.042625	1.008409	0.0512252
cg03570636	1.2892796	0.5910893	2.812167	0.5231094
cg22134823	3.4415193	0.8976287	13.19483	0.0714733
cg11968804	3.1693111	0.8354012	12.0236	0.0899609
cg04946709	4.2528812	1.1076822	16.32869	0.0349482
cg24704151	0.0016746	1.72E-05	0.162814	0.0061958
cg23114964	1.7876002	0.7933388	4.027931	0.1610855
cg06103654	1.8476869	0.9831395	3.472495	0.0565022
cg08343644	0.1428915	0.0529494	0.385613	0.0001224

cg11828180	1.4222113	0.7869944	2.570139	0.243377
cg07950084	1.0952083	0.4857874	2.469149	0.8264424
cg07209550	2.2556911	0.7973762	6.381106	0.1252281
cg10868567	1.8225182	0.7366906	4.508776	0.1940328
cg02152351	0.4159254	0.1989432	0.869565	0.0197322
cg00476955	6.4238309	1.5801894	26.11434	0.0093391
cg22008551	0.5110253	0.1993663	1.309885	0.1621476
cg24617091	0.1078438	0.0025886	4.492853	0.2418504
cg04174651	3.364925	1.1911197	9.505947	0.0220191
cg01678049	1.59E-09	1.04E-20	243.2138	0.1231069
cg23270757	1.8663965	0.7665189	4.544488	0.1693349
cg24953228	1.5306463	0.774567	3.024759	0.2206089
cg24657347	7.3486039	0.7413723	72.84056	0.0883325
cg03134230	0.4776514	0.220887	1.032885	0.0604172
cg25483907	0.6094986	0.3216914	1.154798	0.128878
cg11947782	4.2616054	0.6585666	27.57698	0.1281206
cg26964651	7.8838764	0.5355196	116.0658	0.13237
cg23446939	0.4305254	0.2279296	0.813199	0.0093979
cg15323253	0.3725816	0.1799726	0.771324	0.0078295
cg14888255	1.2365666	0.4929668	3.101825	0.6508825
cg02388150	1.1068848	0.5684804	2.155208	0.7651709
cg15851882	2.3726853	0.5917962	9.512794	0.2226446
cg16538343	3.2795884	0.8409438	12.79003	0.0871759
cg01877352	0.3950127	0.1470739	1.06093	0.0653829
cg03950246	0.3468831	0.1683026	0.71495	0.0041138
cg20432960	1.6150899	0.8551457	3.050375	0.139506
cg08854834	0.5906413	0.2590016	1.34693	0.2106163
cg06094150	1.4992662	0.3247938	6.920697	0.6038034
cg21546868	1.2130599	0.4191888	3.510386	0.7216426
cg24492058	0.064492	0.0151065	0.275326	0.0002142
cg14119581	1.8649186	0.7216499	4.819403	0.1982544
cg10773601	0.2946059	0.1281653	0.677193	0.0040037
cg13686059	1.6631648	0.6680341	4.140683	0.2743408
cg01468309	0.0563543	0.0034776	0.913221	0.0429865
cg09232478	3.5479179	1.5422629	8.161852	0.0028898
cg23573900	0.5265917	0.2107997	1.315461	0.1697596
cg26098637	4.9033991	1.2032867	19.98138	0.0265453
cg09562503	2.6387422	0.7564458	9.204837	0.127983
cg18049676	0.3843761	0.1583818	0.932841	0.0345453
cg03358592	0.0209479	0.0002027	2.164655	0.1023399
cg04568592	0.0285346	0.0022104	0.368354	0.0064261
cg18003970	0.1584975	0.0380599	0.66005	0.0113824
cg23980740	2.1677134	0.9975322	4.710606	0.0507338
cg08302868	23.095353	1.5437834	345.5118	0.0229328
cg17279125	0.4200131	0.1884085	0.936322	0.0339366
cg08283130	0.2390495	0.0907791	0.629492	0.003769
cg08315277	1.7379258	0.0599518	50.3802	0.7476522
cg26389888	1.6994117	0.5662534	5.10019	0.3442953
cg02054724	1.2488807	0.5256159	2.967382	0.6147327
cg12438819	0.374097	0.1305218	1.072224	0.0672256
cg01565824	0.033263	0.0012094	0.914853	0.0441577
cg18892517	2.1356652	1.0404505	4.383742	0.0386363
cg19547746	0.017649	0.0001101	2.829973	0.1191388
cg26472225	0.3942829	0.1095428	1.419162	0.1543741
cg13189207	0.3181773	0.1443122	0.701512	0.0045282
cg23943360	1.2142823	0.6546576	2.252294	0.5379256
cg02766070	0.2704456	0.1225865	0.596646	0.0011987
cg13399952	1.1100449	0.5249705	2.347179	0.784653

cg03677141	0.3148409	0.0256451	3.865254	0.3663906
cg16520539	84360961	9.03E-08	7.88E+22	0.2994053
cg17280398	0.5537451	0.0400161	7.662752	0.6592832
cg14763096	0.3976405	0.132226	1.195816	0.1006668
cg13446404	8.2338555	0.7635694	88.78875	0.0822754
cg09007290	1.7566649	0.5454012	5.657985	0.3451142
cg13586115	8.4318697	0.3404603	208.8244	0.1929225
cg18586212	0.4529415	0.2076723	0.987883	0.0465244
cg18857655	1.5073444	0.6101455	3.723845	0.3738533
cg06613515	3.4483669	0.9062794	13.12094	0.0694272
cg01287054	1.7651198	0.7966158	3.911105	0.1615713
cg26198197	0.9467743	0.4680978	1.914945	0.8790379
cg26052186	1.0629027	0.2925072	3.862339	0.9261686
cg13953900	2.7914887	0.5123589	15.20889	0.235292
cg06214831	1.6083676	0.5182549	4.991456	0.4108296
cg04425005	0.3524896	0.1191367	1.042911	0.0595584
cg24685006	3.1717113	0.9109029	11.04372	0.0697763
cg25687358	1.377616	0.7384196	2.570118	0.313996
cg20119891	0.8289032	0.3591278	1.913192	0.6601414
cg04388244	0.5336444	0.1927966	1.477081	0.2266507
cg21281951	1.3411015	0.7124242	2.524554	0.3631637
cg16318253	0.4654689	0.2272919	0.953229	0.0365339
cg10028929	0.8295326	0.3122988	2.203417	0.7076871
cg01943873	0.0230032	0.0006294	0.840756	0.0399338
cg03689799	0.862677	0.4134903	1.799828	0.693816
cg00246969	0.4940422	0.2380086	1.025499	0.0584393
cg26645834	12.49636	1.9021457	82.09625	0.008553
cg15036475	0.4190718	0.179351	0.979203	0.0445904
cg16253249	1.4835369	0.2356708	9.338797	0.6743376
cg02594677	3.4392826	0.5604765	21.10466	0.1820437
cg00321638	2.4991071	0.9282152	6.728544	0.069901
cg04095556	0.0005378	1.63E-06	0.17723	0.0109304
cg17660682	8.26E-06	8.84E-29	7.72E+17	0.6644968
cg10733063	0.3409566	0.1659628	0.700467	0.0033995
cg02418793	24.515324	0.7511704	800.0863	0.0720079
cg24031597	671.76095	0.0036831	1.23E+08	0.2922189
cg04993082	1.5395659	0.6519331	3.635746	0.3250236
cg09965419	0.3192734	0.1003895	1.0154	0.0531038
cg09631059	2.7885062	0.671202	11.58484	0.1581574
cg18316498	7.2909656	1.6097896	33.02182	0.0099454
cg27029018	0.8266223	0.4350986	1.570459	0.5609041
cg00346326	1.4016432	0.5894057	3.333194	0.4449153
cg08875705	1.7928001	0.2372131	13.54956	0.5715929
cg11390468	5.6037539	0.8753495	35.87374	0.0688477
cg14804384	5.4348154	0.4286038	68.91497	0.1914756
cg03501387	0.9987937	0.377036	2.645872	0.9980624
cg24437859	4.9327315	1.1296398	21.53947	0.0338333
cg10306450	0.0483016	0.0082557	0.282598	0.0007737
cg01763719	1.8942674	0.9275806	3.868396	0.0794981
cg09313940	1.18E-06	3.05E-53	4.56E+40	0.8030491
cg20575856	0.4889907	0.1829899	1.306695	0.1537075
cg11940149	1.0455859	0.4060272	2.692553	0.9264074
cg04919425	0.6136008	0.3017166	1.247879	0.1774858
cg24581749	0.2662334	0.0785304	0.902584	0.0336281
cg07178825	4.8390317	1.496458	15.64777	0.0084596
cg21221899	0.13004	0.0295008	0.573219	0.0070342
cg11561737	1.5936973	0.8346708	3.042961	0.157855
cg14203417	2.8605492	0.5049666	16.20452	0.2349179

cg14534848	1.135059	0.569072	2.263965	0.7191272
cg04078410	1.8319394	0.8002196	4.193851	0.1519822
cg11523020	2.8870135	1.1728302	7.10661	0.0210643
cg27053157	0.2614956	0.0005595	122.2122	0.6688861
cg07018580	1.4352385	0.5892505	3.495813	0.4263136
cg05739816	0.6377913	0.1797171	2.263434	0.4864735
cg02931762	2.8715189	0.9837159	8.382116	0.0536161
cg03987847	2.0874722	0.0781741	55.7415	0.660567
cg26590603	0.5295932	0.2121589	1.321976	0.1732248
cg13442966	0.4257221	0.1904405	0.951685	0.0374688
cg06999858	0.3717327	0.1519217	0.909581	0.0301932
cg22070991	2.0539693	0.9752482	4.325863	0.0582234
cg10160276	1.7973207	0.9408027	3.433623	0.0758647
cg19123345	2.5797817	0.322972	20.60635	0.3713658
cg15010140	0.372894	0.1415598	0.98227	0.0459161
cg20061364	2.3028236	0.4740528	11.18651	0.3009693
cg09651136	0.517363	0.2127964	1.257843	0.1459804
cg25722212	1.1229544	0.5349062	2.357472	0.7592499
cg22508957	0.0121015	1.93E-05	7.582735	0.1791316
cg23146560	0.2411289	0.0732489	0.793775	0.0192897
cg00419568	0.0036058	7.94E-05	0.163714	0.0038582
cg05057910	3.4746188	1.3107546	9.210706	0.0122798
cg17213699	1.9277656	0.6528203	5.692654	0.2348113
cg01993847	2.7775805	0.327343	23.56841	0.3490838
cg24364827	0.0321532	0.0040191	0.257228	0.0011964
cg03981727	2.3585514	0.6620447	8.4024	0.1855971
cg05694870	367300526	2.67E-06	5.05E+22	0.2350895
cg18389931	2.2653532	0.7357309	6.975138	0.154122
cg18888205	1.3809008	0.6079465	3.136604	0.440693
cg01139503	0.4754643	0.1689574	1.338008	0.1590221
cg07899076	0.4648608	0.1702738	1.269106	0.1349423
cg13290785	2.3483445	0.9243139	5.966287	0.0727292
cg11109621	0.6221415	0.3016233	1.283257	0.1988651
cg23695990	3.0528041	1.0020517	9.300531	0.04958
cg00442174	0.0054307	6.90E-05	0.42761	0.0192155
cg10161888	1.4440516	0.7530734	2.769033	0.2686348
cg25382900	1.1157481	0.4700299	2.648542	0.8038899
cg18832655	78.604739	0.4622494	13366.6	0.0958137
cg05955332	0.3784337	0.1363728	1.050151	0.0620421
cg17246606	0.2033184	0.0667669	0.619144	0.0050508
cg26396452	0.3560371	0.1580137	0.802224	0.0127154
cg15072619	0.6052639	0.2927826	1.25125	0.1754033
cg11758861	1.6429703	0.8095054	3.334569	0.1691942
cg16284178	0.487888	0.1893455	1.257144	0.1372542
cg17544177	0.4317463	0.2173903	0.857466	0.0164302
cg08983961	0.1886382	0.0536393	0.6634	0.0093343
cg18170545	0.4559876	0.2077032	1.001067	0.0503114
cg06924878	4.7133234	0.8645199	25.69683	0.0731774
cg13475638	0.173302	0.0008553	35.11427	0.5177735
cg09331545	1.9472986	0.6914225	5.484305	0.207133
cg04272820	0.2243588	0.0734149	0.685649	0.0087393
cg13519035	0.0780889	0.0133141	0.458001	0.0047261
cg17201227	0.4790778	0.1865799	1.23012	0.126141
cg19272468	0.4137195	0.1213792	1.410158	0.1583567
cg16349093	0.5249619	0.2317972	1.188906	0.122323
cg03095364	0.0521814	0.0067306	0.404555	0.0047133
cg08537660	0.5385568	0.2582453	1.123131	0.0988801
cg21221767	2.1520898	0.8810215	5.256955	0.0925736

cg18566555	0.4236253	0.1752655	1.023923	0.0564609
cg05444541	0.4630517	0.1829902	1.17174	0.1040826
cg24064279	0.5625927	0.1273166	2.486012	0.4480186
cg20685431	431.16664	0.1187247	1565847	0.1469286
cg25748136	0.7287379	0.3505509	1.514927	0.3967122
cg12105691	0.5880688	0.3061441	1.129615	0.1109282
cg02136620	0.5472976	0.1889223	1.585491	0.2667019
cg22588546	0.5852042	0.2971976	1.152311	0.1211721
cg06284285	0.8799684	0.3448943	2.245164	0.78903
cg18848959	0.4052408	0.2203642	0.745221	0.0036599
cg07314983	0.6389457	0.3012262	1.355299	0.2429939
cg27558095	1.7085544	0.8680376	3.36294	0.1210565
cg16928046	2.2613145	0.8037841	6.361837	0.122084
cg13361185	2.8784245	0.9926675	8.346529	0.0516049
cg09804649	0.4041757	0.1616827	1.010362	0.052634
cg14292823	0.1870886	0.0571426	0.61254	0.0056067
cg11795262	0.3682938	0.1489069	0.910907	0.0306235
cg15959205	0.592295	0.2512883	1.396059	0.2312066
cg21241814	1.6935406	0.5959185	4.812872	0.3228658
cg03576748	0.1582035	0.0401261	0.623743	0.0084303
cg00864012	0.3069535	0.1015768	0.927579	0.0363311
cg17495087	3.7881239	1.0953955	13.10018	0.0353876
cg11900861	2.1848863	0.8372552	5.70164	0.1102633
cg14641472	1.2115538	0.5640321	2.602445	0.62275
cg03526142	1.4209053	0.7191326	2.80751	0.3119959
cg06087101	0.5064528	0.1832032	1.400054	0.1897447
cg21499610	0.6384976	0.3319719	1.228054	0.1788268
cg20018425	0.9405028	0.3020586	2.92839	0.9157001
cg27590407	1.1845119	0.4737789	2.961441	0.7172172
cg01554437	2.3567385	1.0290903	5.39721	0.0425815
cg00280239	0.1636121	0.0125067	2.140359	0.1676177
cg26782218	35.47845	0.4343287	2898.083	0.1121224
cg08758727	0.8547037	0.3381691	2.160217	0.7399855
cg06400428	0.5813446	0.0851251	3.970174	0.580024
cg21029769	1.3996241	0.6905427	2.836823	0.350966
cg20804831	1.8940573	0.6302406	5.692196	0.2552556
cg00416208	1.0096593	0.5246966	1.94286	0.9770363
cg15393490	5.0551948	1.1235488	22.74489	0.0347053
cg13229078	0.3092372	0.1516522	0.630572	0.0012448
cg27491280	1.2195684	0.4503029	3.302993	0.6961821
cg22711792	1.3224545	0.632256	2.766104	0.4578985
cg00360761	4.1360624	1.1158827	15.33048	0.0336702
cg08480461	1.1758252	0.4194749	3.295942	0.7580871
cg13347071	0.0502507	0.0034155	0.739317	0.0292477
cg17621241	1.9719496	0.7159267	5.431541	0.1890073
cg12371704	6.1680671	0.2417093	157.4	0.2709846
cg09294139	1123960.9	3.73E-33	3.39E+44	0.7579322
cg26460506	0.6204631	0.2983251	1.290453	0.201436
cg04137171	0.2696225	0.0930825	0.780988	0.015713
cg20599967	1.5587514	0.6764154	3.592032	0.2973546
cg09136346	1.6300395	0.8519649	3.118707	0.1399451
cg00681462	2.4592861	0.842293	7.180504	0.0997583
cg23617848	3.1285559	1.0388108	9.42218	0.0425955
cg12208770	0.4681837	0.0634318	3.455614	0.4568073
cg07169660	0.3314329	0.1019956	1.076986	0.0662663
cg06649257	0.2631003	0.1043432	0.663405	0.0046602
cg07300060	3.0733096	0.5805831	16.26853	0.186673
cg16776065	0.2274814	0.0821624	0.629823	0.0043755

cg07412281	0.3086462	0.1023577	0.930682	0.0368402
cg08113897	4.1529189	0.7365328	23.41611	0.1066499
cg27129048	1.3220328	0.5729739	3.05035	0.5128312
cg10609868	0.49822	0.2488916	0.997314	0.0491189
cg02726501	1.2836729	0.4392489	3.75144	0.6481005
cg11520003	0.2061684	0.0754626	0.563265	0.0020746
cg14074184	0.3470629	0.1280039	0.941007	0.0375766
cg04840761	0.7491193	0.0579875	9.677598	0.8248846
cg04437605	1.2233261	0.1807339	8.280276	0.8363243
cg27175287	2.5544987	0.7809364	8.355948	0.1208923
cg02734782	11778.491	2.66E-17	5.21E+24	0.6991421
cg01831743	0.406514	0.0559072	2.955858	0.3738607
cg06014401	2.9555371	1.3828745	6.316697	0.0051662
cg26218809	5.5394291	0.4636963	66.17538	0.1761534
cg01559502	0.2051511	0.087032	0.483581	0.0002939
cg19563452	1.0529929	0.0422332	26.2541	0.9748966
cg00307530	3.2138309	0.2442935	42.27991	0.3745522
cg19676502	1.6113358	0.7533308	3.446564	0.2187756
cg08241368	9.952595	1.2353882	80.18059	0.0308863
cg06482904	507.09883	1.088312	236282.6	0.0469268
cg10550869	0.0377992	1.12E-05	127.879	0.4295406
cg02127980	6.5617613	1.1965026	35.98547	0.0302672
cg20975182	8.5405382	1.4325459	50.9169	0.0185442
cg25314315	2.2010647	0.8096978	5.983326	0.1220462
cg26036288	4.1100733	0.0968697	174.3858	0.4598021
cg15745401	1.4592043	0.5650622	3.768217	0.4349822
cg15389589	15.610396	0.1215255	2005.213	0.2673385
cg14368247	1.377322	0.5138943	3.691452	0.5244816
cg15443767	0.3999864	0.1972707	0.811013	0.0110604
cg06282802	1.6116928	0.6385275	4.068038	0.312326
cg08416875	1.7388207	0.754792	4.005736	0.1938517
cg04362586	1.8145883	0.9415122	3.497279	0.0750869
cg17036427	2.6083996	0.8769895	7.758073	0.0847187
cg06459070	0.8340935	0.3833449	1.814846	0.6474123
cg19654437	0.2250487	0.0850835	0.595262	0.0026536
cg03320947	38336.164	127.17456	11556253	0.0002905
cg11052780	0.4069435	0.1835719	0.902115	0.0268571
cg17791977	1.9749301	0.7522663	5.184798	0.1669982
cg07340922	0.8176536	0.3904345	1.712342	0.5934803
cg22079098	4.0686308	0.3534557	46.83403	0.2602919
cg18764192	9.2976559	0.3273126	264.1097	0.1915941
cg20934105	1.6543822	0.676111	4.048123	0.2701692
cg22402701	1.880587	0.9567866	3.696339	0.0669753
cg01947936	0.5099124	0.1674236	1.553011	0.2359042
cg08493356	3.25E+15	2017.1125	5.24E+27	0.0127539
cg18693985	0.6397762	0.3136227	1.305115	0.2194908
cg13906377	1.6176569	0.7521927	3.478914	0.2182871
cg14162552	0.4129513	0.1514299	1.126124	0.084006
cg02748539	0.1249547	0.0230992	0.67594	0.0157493
cg23619910	1.6297139	0.7308605	3.634028	0.2326037
cg21188037	2.0480014	0.7202505	5.823405	0.1787873
cg05576619	0.3925824	0.1467038	1.050559	0.0626376
cg10211725	1.7281857	0.5242292	5.697176	0.3687304
cg08041140	12.592998	0.222312	713.3381	0.2187358
cg22321558	3.34E-08	6.64E-14	0.016795	0.010168
cg07912501	635240536	8.43E-05	4.79E+21	0.1802885
cg15108641	1.3308736	0.4939527	3.585818	0.571918
cg16102052	0.1609078	0.0455659	0.568217	0.0045388

cg17036164	4.5712815	0.4678413	44.66604	0.1912814
cg19108747	4.9076561	1.322873	18.20665	0.0173936
cg07341609	2.8627366	1.0291463	7.963164	0.0439042
cg04676463	3.91E-30	1.33E-56	0.001151	0.0294365
cg12835736	2.7141007	1.079242	6.825478	0.0338346
cg22533992	0.2818213	0.1179261	0.6735	0.004383
cg26115667	1.0134227	0.399222	2.572568	0.9776201
cg24659054	0.2722707	0.0727698	1.01871	0.0533066
cg09651496	2.9732367	0.208891	42.31937	0.4212709
cg21790305	0.0005538	2.22E-06	0.138235	0.0077543
cg19645221	1.9498936	0.9125011	4.166664	0.0847766
cg15853299	71.204479	0.5553724	9129.149	0.0849833
cg00844308	2.4517117	0.9263712	6.488641	0.0709264
cg25695568	0.2650769	0.1090209	0.644517	0.0034011
cg07197642	0.0039458	5.71E-05	0.272662	0.0104281
cg16774946	4.4615541	1.1599961	17.15994	0.0295623
cg01815912	0.1972885	0.0698612	0.557144	0.002182
cg03160174	1.2703228	0.3592501	4.491913	0.7104094
cg00941241	0.0003495	1.42E-06	0.086049	0.0046101
cg22708508	0.627389	0.3051427	1.289944	0.2049195
cg19884556	0.3062812	0.1259354	0.744891	0.0090681
cg26766064	0.6691164	0.2477102	1.807421	0.4280679
cg20066627	1.3812177	0.7062899	2.701104	0.3452728
cg22154024	0.0401264	0.0015509	1.038162	0.0526964
cg00137918	0.6649482	0.0362937	12.18274	0.7833063
cg05760424	39.991062	0.2147773	7446.247	0.1666088
cg10453977	0.8645311	0.0335955	22.24746	0.9299985
cg06591185	1.3040368	0.5071785	3.352886	0.5816622
cg04721719	0.1734133	0.0259528	1.158725	0.0706149
cg22764044	0.4135644	0.0879872	1.943869	0.2634862
cg17886678	0.000105	1.55E-08	0.709271	0.0417187
cg02149376	4.7490595	0.6063029	37.19851	0.1379418
cg00781208	0.4595617	0.2124069	0.994304	0.0483281
cg21965995	0.2055283	0.0580027	0.728275	0.0142381
cg23372684	1.8158513	0.8012025	4.115459	0.1529961
cg23740652	0.2788243	0.1104956	0.703585	0.0068427
cg21305834	2.0286763	0.7472208	5.50778	0.1650937
cg17084937	2.2998422	1.0549538	5.01375	0.0362152
cg14707974	0.0740108	0.0126944	0.431498	0.0037997
cg24160158	8.259E+13	178.69524	3.82E+25	0.019368
cg09419102	3.0530491	0.6291318	14.81583	0.1660696
cg02631196	7.4685022	1.5206173	36.6815	0.0132831
cg20771595	0.2845959	0.0533557	1.518017	0.141215
cg11653233	1.177423	0.4358793	3.180525	0.7473459
cg04525683	0.6611921	0.1616421	2.704586	0.5648694
cg17403397	0.6126624	0.1880337	1.996213	0.4162389
cg12734838	2.8097374	0.5733548	13.76918	0.2026634
cg14582400	1.6848178	0.821028	3.457387	0.1549386
cg05509777	1.6296504	0.6722527	3.950539	0.2797132
cg22206745	37.385162	0.6346461	2202.251	0.0816269
cg16930728	0.6464898	0.3012085	1.387574	0.262978
cg15952725	3191.039	1.4847932	6858012	0.0393093
cg27657983	1.779585	0.5510943	5.746608	0.3351934
cg06850442	2.1520709	0.7617598	6.079881	0.1480621
cg03460239	0.7328943	0.3486504	1.540609	0.4123218
cg08081780	0.3538257	0.1770587	0.707068	0.0032687
cg17303119	0.800181	0.3778164	1.694711	0.5604232
cg11270806	0.286843	0.105791	0.77775	0.0141336

cg12873476	0.2876201	0.087955	0.940542	0.039268
cg16359901	0.038989	0.0011749	1.293888	0.0694058
cg26876664	1.740851	0.7666048	3.953226	0.1852342
cg27449041	0.1198117	0.0241369	0.594727	0.0094409
cg00778880	0.5320043	0.2566035	1.10298	0.089794
cg27200267	4.8346394	1.3406736	17.43432	0.0160422
cg06051243	1328.1446	18.340381	96179.47	0.0009969
cg00918522	1.2908202	0.6284035	2.651508	0.4870211
cg07464201	0.0791748	0.0164597	0.380849	0.0015534
cg26534677	0.2398979	0.0460486	1.249787	0.0900401
cg23082635	0.4285413	0.2040903	0.899835	0.0251677
cg25561913	1.8427066	0.0001189	28565.79	0.9011875
cg00392377	2.4039535	0.9727765	5.94072	0.0574108
cg11661187	0.5714652	0.2876146	1.135451	0.1101911
cg19404835	0.4056312	0.1913923	0.859683	0.0185485
cg25754195	0.2894133	0.104955	0.798057	0.0165824
cg01871127	1.3734062	0.6365546	2.96321	0.4186793
cg08224569	0.8629435	0.3554056	2.095272	0.7446638
cg25611443	0.5377325	0.2376026	1.216974	0.1365546
cg16754455	0.6198761	0.3067516	1.25263	0.1827259
cg20745568	13.374875	1.9098943	93.66344	0.0090134
cg01314252	2.1695015	0.6607862	7.122934	0.2016436
cg23693749	0.2805197	0.0786636	1.000353	0.0500635
cg14788655	0.7127003	0.3118448	1.628829	0.4219016
cg26244646	0.6554674	0.3661851	1.17328	0.1550263
cg05714155	2.3685506	0.5873206	9.551907	0.2255273
cg12595401	5.8559838	0.6229778	55.04618	0.1221013
cg11836171	33.297534	0.0778879	14234.9	0.2567333
cg26577454	1.310318	0.7270246	2.361589	0.3685172
cg06242416	0.4863058	0.2496194	0.947416	0.0341145
cg00445405	3.1438085	0.9683554	10.20651	0.0565926
cg04976685	2.52E-06	2.68E-12	2.379944	0.0662945
cg04369651	0.4913309	0.2124499	1.136296	0.0966602
cg18394275	0.0872735	0.002414	3.155154	0.1827775
cg09875213	1.4957312	0.5221059	4.284977	0.4534057
cg08749576	6.1211217	1.186781	31.57122	0.0304217
cg23131974	0.5577778	0.2451545	1.269062	0.1639623
cg15209710	0.8247082	0.4006697	1.697517	0.6007954
cg08264376	3.7728365	1.2263082	11.60744	0.0205715
cg01037703	0.345615	0.1205029	0.99126	0.0481216
cg06968241	0.5935291	0.2761206	1.275808	0.181515
cg13975344	1.6746422	0.8106771	3.459363	0.1636382
cg20379919	1.6005377	0.5514981	4.645022	0.3869209
cg10298855	0.9106748	2.17E-20	3.82E+19	0.9967614
cg01028017	0.9823707	0.4864526	1.983856	0.9604403
cg24489370	0.560407	0.1627995	1.929097	0.358526
cg10166697	6.97E-26	3.43E-49	0.014158	0.034392
cg07613752	3.3533509	0.9939689	11.31319	0.0511507
cg13847853	3.4504409	1.0036951	11.86171	0.0493196
cg12169852	920.77149	2.2111637	383427.1	0.0265676
cg25816696	2.3146237	0.9166684	5.844516	0.0757574
cg25498385	0.439261	0.2002066	0.963756	0.0401647
cg14951497	1.9323344	0.9289273	4.0196	0.0779537
cg00875013	7.21E-06	2.55E-10	0.20404	0.0235808
cg12039422	0.4244458	0.1543076	1.167501	0.0969177
cg01763692	1.6294324	0.7310683	3.63174	0.2325027
cg24704476	0.0767841	0.0174439	0.337987	0.0006874
cg05437294	0.2677494	0.1022596	0.701056	0.0072927

cg24060954	167.39969	0.5620399	49858.84	0.0781162
cg06974428	1.464085	0.460944	4.650337	0.5179376
cg00008971	0.4124413	0.1697444	1.002141	0.0505545
cg21048927	0.0152532	5.97E-12	38963214	0.7050681
cg18144373	1.8107311	0.5513673	5.946575	0.3277559
cg26503078	2.4071766	0.8026575	7.219142	0.116959
cg23911372	2.8278937	1.0123105	7.899733	0.0473331
cg20219891	0.5387506	0.216173	1.342685	0.1843417
cg08716736	0.4656465	0.2392676	0.90621	0.0244579
cg09678615	1.1922626	0.6161379	2.307097	0.6015927
cg05301794	0.697403	0.3168727	1.534909	0.3705685
cg26898087	0.1584099	0.0166402	1.508019	0.1090108
cg08558500	69.315457	0.0049911	962634.8	0.3837899
cg26313421	0.4421324	0.1766082	1.106863	0.0813132
cg18617091	2.2501342	0.8716363	5.808734	0.0937304
cg01807408	1.1510123	0.4733089	2.79908	0.756414
cg05745631	6.44796	1.3851878	30.01484	0.0175387
cg12827601	0.4169813	0.2050944	0.847772	0.0156869
cg11317254	0.405931	0.101673	1.620686	0.201821
cg16164923	0.2540449	0.1052557	0.613162	0.0023039
cg26917256	0.2595249	0.096709	0.696452	0.0074014
cg23417523	0.2898822	0.0327501	2.565842	0.2657056
cg07212702	0.24887	0.1232779	0.502412	0.0001043
cg20169988	1.5990079	0.7767583	3.291663	0.2025974
cg14539363	1.7390732	0.7616348	3.9709	0.1889852
cg17062665	1.4355007	0.0426738	48.28874	0.8402757
cg12703269	1.7798888	0.3681819	8.604453	0.4732875
cg03466951	3.1017997	1.0379135	9.269714	0.0427048
cg07492757	3.2320784	1.0471318	9.976137	0.0413451
cg16180082	1.2538904	0.6125581	2.566681	0.5359021
cg05491608	6.0978981	1.0825397	34.34919	0.0403754
cg17792658	0.2310684	0.0492672	1.083734	0.0631713
cg08480609	45.784275	0.0147123	142479.9	0.3514203
cg25982965	0.4031827	0.1360078	1.195198	0.1013477
cg18741166	7.2907704	0.9012796	58.97763	0.0625303
cg21959618	0.3410202	0.1655449	0.702497	0.0035272
cg25686793	1.6198253	0.570518	4.599038	0.364992
cg26585452	1.9796024	0.5047251	7.764278	0.3273943
cg24764168	3.7960459	1.2743886	11.30735	0.0166043
cg17351085	0.1888101	0.027948	1.275555	0.0872165
cg18443486	0.1305416	0.0447018	0.381217	0.0001963
cg15990724	1.0033735	0.3516894	2.862635	0.9949763
cg14446942	26.157495	0.8345235	819.8865	0.0633042
cg17430268	4.1109977	0.3435708	49.19016	0.2642858
cg01359999	3.2864576	0.3051875	35.39071	0.3264885
cg00912580	0.1826253	0.050155	0.664978	0.0099159
cg17514495	1.6857279	0.5447511	5.216471	0.3649128
cg23223330	0.6804571	0.2732684	1.694385	0.4081832
cg10914555	0.4279827	0.1745472	1.049396	0.0636534
cg09170127	1.04E-18	8.78E-36	0.122195	0.0389441
cg10075378	0.009902	4.03E-05	2.43491	0.1003577
cg03124318	1.6342848	0.5443731	4.906354	0.381161
cg15325875	2.284584	1.0050861	5.192912	0.0486012
cg02050181	1.108E+10	3.00E-07	4.09E+26	0.2347131
cg16938805	1.7256881	0.7839082	3.798914	0.1753404
cg25851789	2.5048247	1.1168252	5.617841	0.0258759
cg27108147	1.6545844	0.6352542	4.309534	0.3025486
cg07861456	1.6524839	0.3967879	6.882021	0.490162

cg07063068	0.3564585	0.153453	0.828023	0.0164483
cg18445426	24.885207	2.53906	243.8987	0.0057785
cg15742737	1.2992109	0.3947293	4.276219	0.6667254
cg01739210	3.530228	0.8271946	15.06599	0.0884341
cg17139666	2.4198742	0.9858629	5.939762	0.0537449
cg22205341	0.6466659	0.3126568	1.337494	0.2397206
cg20946037	0.2842268	0.1145502	0.705235	0.0066647
cg00890049	1.8308529	0.9741369	3.441018	0.0603031
cg14234152	1.5465915	0.760671	3.14452	0.2284364
cg13762257	23.098314	1.5037515	354.8007	0.0242804
cg16585682	0.3288891	0.0106052	10.19951	0.5256714
cg06263943	1.2375007	0.4288168	3.571241	0.6935202
cg01489419	0.5566303	0.2478685	1.250007	0.155799
cg26216343	7.3768361	0.6253943	87.01344	0.1124745
cg15088646	0.611567	0.3217534	1.162425	0.1334467
cg24874389	0.4205343	0.1907999	0.926883	0.0316925
cg09347430	0.4921505	0.1976634	1.225377	0.1276908
cg16140321	52.762851	2.1184052	1314.158	0.0156245
cg26704870	0.209032	0.0583866	0.748363	0.0161541
cg05091238	2.3321438	0.9507515	5.720627	0.0643639
cg00131893	17.147428	0.4783219	614.7205	0.1196749
cg02762440	0.6063307	0.1426559	2.577088	0.4979605
cg21830797	7.617E+10	3.13E-11	1.85E+32	0.3186178
cg19396360	2.7475509	1.0455916	7.21987	0.0403246
cg13241740	1.09E-08	2.50E-17	4.720706	0.0707573
cg24110635	0.5257453	0.2428315	1.138272	0.1028162
cg08969950	1.5993726	0.7735147	3.306974	0.2051332
cg25407736	0.5193517	0.2534799	1.064093	0.073419
cg00159614	0.0996973	0.0142532	0.697356	0.0201702
cg26969179	4.3010396	1.385552	13.35132	0.0115962
cg12188860	0.3271545	0.114099	0.938046	0.0376205
cg04051152	0.5607918	0.1988586	1.581463	0.2741906
cg06528575	0.369242	0.1226055	1.112019	0.0765266
cg07270153	1.4532908	0.7378457	2.86246	0.2797382
cg25011577	0.7364934	0.0399339	13.58303	0.8370478
cg15580075	0.2295115	0.0624123	0.843993	0.0267429
cg26282887	3.563288	0.764522	16.60779	0.1056503
cg05644321	559.10648	8.0028385	39061.15	0.0035017
cg14579957	1.9884481	0.7033243	5.621767	0.1948866
cg24956866	0.1929945	0.0436412	0.853479	0.030095
cg14153919	3.9487595	0.9677107	16.11298	0.0555924
cg08208480	1.1171243	0.4896858	2.548505	0.7923896
cg05937818	2.0052065	0.8169718	4.921655	0.1288375
cg14153677	0.8381281	0.4245251	1.654693	0.6108788
cg05152629	849369.89	11.336124	6.36E+10	0.0171285
cg25952997	0.9850689	0.4991372	1.944076	0.9654057
cg16118803	0.1262027	0.0309486	0.514631	0.0038981
cg24252805	0.1470349	0.035906	0.602107	0.0076924
cg19304407	0.1498509	0.0125999	1.782181	0.1329557
cg13649253	0.0412361	0.0039352	0.432104	0.0078145
cg20430847	1.4003795	0.4483193	4.374254	0.5622765
cg00794881	0.1529847	0.0228714	1.0233	0.0528419
cg05005791	3.3332695	0.9743048	11.40371	0.0550497
cg12897901	1.952E+11	1.05E-10	3.61E+32	0.2981011
cg18088360	9.8693941	1.5145901	64.31109	0.0166619
cg10169194	4.40E-06	4.48E-11	0.432798	0.0354872
cg14644001	0.7933728	0.448488	1.403472	0.4264295
cg18126504	3.7379155	0.3431061	40.72214	0.2792176

cg16223436	14.299849	0.4921418	415.5015	0.1217358
cg07483245	1.83E-05	2.90E-15	115394.4	0.343361
cg16208084	1.3585427	0.3212407	5.745344	0.6770573
cg07790342	2.7126132	1.0781834	6.824693	0.0340164
cg24471576	0.1764483	0.0148585	2.095361	0.1694286
cg03031672	0.3931163	0.1400949	1.103113	0.0761382
cg16685257	2.256339	0.7806736	6.521376	0.1329092
cg09490523	0.5373259	0.1975773	1.461297	0.2236596
cg15076145	1.461325	0.7835824	2.725266	0.2328725
cg11979420	0.2679979	0.0762905	0.941439	0.039966
cg08989212	0.8947295	0.3055665	2.619858	0.8391929
cg10258419	1.5058251	0.7844308	2.890643	0.218603
cg26038465	3.9854492	0.5550591	28.61642	0.1692314
cg04432875	0.4210751	0.2115102	0.838278	0.0138121
cg07336350	2.4807663	0.7369984	8.350359	0.1423296
cg18303608	0.5901217	0.2703427	1.288156	0.1854299
cg10877050	0.8073198	0.0201171	32.3986	0.9095391
cg13755873	3.3858542	1.3006512	8.814053	0.0124732
cg05758804	0.2381058	0.0464706	1.220006	0.0851739
cg14824732	1.4536127	0.6787018	3.113282	0.3357563
cg02529500	0.4756641	0.0081147	27.88242	0.7205445
cg06158040	0.0144385	0.0006689	0.311649	0.0068549
cg01187496	0.3159458	0.1354971	0.736708	0.007645
cg17119907	0.2569805	0.0396909	1.66383	0.1539428
cg12777753	0.6748471	0.2769239	1.644562	0.3868537
cg20441701	0.1127274	0.0194268	0.654121	0.01497
cg15963326	0.2601154	0.0390608	1.732172	0.1639054
cg14581287	0.5113802	0.246065	1.062767	0.072358
cg16705744	2.823936	1.0582556	7.535623	0.0381693
cg20566643	9.25E-06	4.13E-26	2.07E+15	0.6277891
cg27544219	0.0806457	0.0078992	0.823344	0.0336746
cg12867037	230.91845	0.1212845	439655.1	0.1578222
cg22284390	0.193031	0.0185577	2.007842	0.1686354
cg17554182	4.4028729	0.8149466	23.78719	0.0850323
cg27462969	24.213469	1.10E-12	5.34E+14	0.8388988
cg11382993	1.821E+10	286.68639	1.16E+18	0.0099598
cg16321461	0.3507773	0.0459306	2.678928	0.3125153
cg17039722	1.660585	0.7034619	3.91996	0.2471419
cg16429975	0.6870778	0.290963	1.622461	0.3919526
cg13505794	1480218.4	2.9812035	7.35E+11	0.033737
cg26677194	0.3517783	0.1427289	0.867014	0.0232065
cg11623339	1.9482001	0.6911001	5.491945	0.2072251
cg10943786	1.4614353	0.7196741	2.967723	0.2938135
cg10256045	0.5242679	0.2019072	1.361303	0.1847045
cg05843574	166.94631	3.0836811	9038.247	0.0119735
cg22298088	1295.2618	0.0136722	1.23E+08	0.2202823
cg16168311	3.27E-08	2.38E-13	0.004497	0.0043
cg16372825	0.5772822	0.2228595	1.495358	0.2578883
cg21107579	0.4249604	0.2004932	0.900735	0.0255671
cg14509437	0.602432	0.2924776	1.240862	0.169254
cg18138202	0.2539214	0.0937038	0.688084	0.0070394
cg02265690	3.5589797	1.1568669	10.94883	0.0268213
cg24563570	0.3582033	0.0717028	1.789464	0.2109607
cg25927375	1.8918411	0.1129394	31.69011	0.6575089
cg26638011	1076322.7	2.06E-12	5.63E+23	0.5046166
cg14473924	0.1793326	0.0354765	0.90652	0.0376472
cg12037947	3.7172945	1.1832683	11.67806	0.0245704
cg06848762	3.11E-10	1.10E-17	0.008765	0.0123778

cg11348249	1.7404182	0.7192728	4.211275	0.2190411
cg21692450	2.8593602	1.3036627	6.271515	0.0087493
cg25483596	0.006334	5.34E-05	0.751585	0.0377877
cg10382148	0.4891614	0.2371666	1.008906	0.0528728
cg15181396	81753276	16140.205	4.14E+11	2.84E-05
cg12140977	1.4466626	0.4736436	4.418581	0.5168666
cg03706951	1.6645497	0.7624785	3.633841	0.2008304
cg20795913	0.1269322	0.037271	0.432287	0.0009623
cg20546098	0.1873073	0.07718	0.454574	0.0002132
cg13395868	0.1780284	0.0028335	11.18559	0.4139572
cg06259557	3.63E-08	9.36E-16	1.406689	0.0546451
cg13582028	0.3494022	0.1079966	1.130424	0.0792046
cg19579500	0.5527671	0.3163018	0.966012	0.0374002
cg04757411	0.8132755	0.3727661	1.774349	0.6035693
cg06454760	0.4487641	0.201104	1.001418	0.0504058
cg00532802	0.0835506	0.0135163	0.516467	0.0075645
cg10585870	702.35949	2.14E-12	2.3E+17	0.7007121
cg05051976	1.9252345	0.3815421	9.714598	0.4276574
cg05079049	5.7051278	1.3674454	23.8024	0.0168777
cg03537386	0.5663326	0.2628786	1.220079	0.1465052
cg04702872	0.1359109	0.0341397	0.541064	0.0046353
cg03531951	0.0756196	0.0168115	0.340143	0.0007637
cg21333861	0.0122699	0.0002401	0.626965	0.0283379
cg07329360	3.0297867	1.1195229	8.199571	0.0290926
cg01706991	1.1437424	0.4886292	2.677177	0.7569252
cg11229513	2.4240591	0.9687191	6.065807	0.0584838
cg04411086	2.2583382	0.7218592	7.065216	0.161548
cg02695163	0.0012087	2.61E-06	0.559689	0.0319294
cg02471507	1.8800638	0.9232738	3.828377	0.0818686
cg24873410	1.6200831	0.757759	3.463726	0.2133236
cg07080019	0.2623672	0.0365596	1.882861	0.1833037
cg10424462	0.4476819	0.0674513	2.971313	0.4052711
cg14116465	11293693	4.46E-11	2.86E+24	0.4270388
cg20608306	0.1137278	0.0273314	0.473228	0.0028039
cg01260308	0.3879363	0.1841768	0.81712	0.0127259
cg26307871	0.3782283	0.1731415	0.826241	0.0147393
cg23545250	0.172913	0.0589818	0.506918	0.0013837
cg16987900	1.391234	0.6909457	2.80128	0.3551376
cg20321265	1.3983004	0.611903	3.195349	0.4265617
cg01100322	0.3810131	0.1792707	0.809786	0.0121263
cg00415978	1.5571809	0.8965124	2.704717	0.1159127
cg20481720	2.2408595	0.6986513	7.18735	0.1748133
cg25037610	0.0462167	0.0020591	1.037319	0.0527599
cg26653990	1.6391517	0.8164587	3.29082	0.1646154
cg06495924	43.978761	3.48E-22	5.55E+24	0.8891213
cg24937747	0.2595257	0.0845665	0.796457	0.0183857
cg15016771	1.1698421	0.611188	2.239132	0.6357996
cg13457549	0.9994219	0.403685	2.474316	0.9990024
cg25141490	0.3376267	0.1318636	0.864468	0.0235999
cg14307223	0.267827	0.077988	0.919773	0.0363658
cg25942990	3.0013686	1.1568896	7.78658	0.0238472
cg13191352	0.3294533	0.1521902	0.713183	0.0048356
cg10636490	0.4000412	0.1864372	0.858374	0.0186723
cg10045909	3.1561411	1.0334807	9.638522	0.0436142
cg09817024	1.8356858	0.9190853	3.666409	0.0852667
cg02309355	0.1818666	0.0625267	0.528982	0.0017543
cg08166554	0.1001417	0.0107045	0.936839	0.0436793
cg12253175	0.274994	0.103926	0.727649	0.0093128

cg07289618	0.1756549	0.0572964	0.538509	0.0023436
cg01346718	0.2645882	0.0725211	0.965332	0.0440732
cg04172533	2.7637631	0.9358046	8.162373	0.0657854
cg25510614	2.5691789	0.6963379	9.479135	0.1565966
cg06849477	1.7378505	0.6181839	4.885479	0.2946653
cg17947364	0.6856327	0.3375575	1.392629	0.2965303
cg21075261	2.2254601	0.9267958	5.343866	0.0734749
cg07295678	0.123419	0.0125036	1.218227	0.0732958
cg12034943	1.2338424	0.5306043	2.869119	0.625513
cg20717228	3427.0377	1.78E-10	6.6E+16	0.6019896
cg00971695	2.8919269	0.7792947	10.73181	0.1124582
cg02414889	0.3893725	0.1632189	0.928881	0.0334806
cg06382559	1.6314843	0.6834752	3.894422	0.2701708
cg00011225	2.9466983	1.0089967	8.605609	0.0481159
cg02739870	1.6944976	0.6153476	4.666179	0.3075203
cg16064518	829578134	1.02E-21	6.73E+38	0.5589131
cg05673137	0.3456791	0.1258653	0.949381	0.0393282
cg21551549	0.1281613	0.0291957	0.562594	0.0064874
cg05614013	0.0121235	0.0003242	0.453335	0.0169347
cg08120831	4.3473285	1.0666004	17.71916	0.0403739
cg01699581	3.9324519	1.0588227	14.60507	0.040821
cg20621200	0.1407169	0.0251614	0.786969	0.0255671
cg10812027	0.3672622	0.1365302	0.987924	0.0472528
cg15638292	2.4124698	0.3918699	14.8519	0.3422684
cg13953838	0.5515593	0.2600778	1.169718	0.1208389
cg01968793	0.1559239	0.042531	0.571637	0.0050522
cg07372034	0.0252604	0.0006684	0.954671	0.0471449
cg15344028	0.6708077	0.3199228	1.406536	0.2905396
cg27583655	1.0899871	0.3643923	3.26042	0.8775049
cg03839626	2.127E+09	0.0553862	8.17E+19	0.0841188
cg03886520	2.4769511	0.2237476	27.42057	0.4596567
cg16533838	1.4581497	0.5343645	3.978933	0.4614846
cg20431135	1.035979	0.4637693	2.314195	0.9313091
cg15759889	10914.986	10.28004	11589149	0.0089114
cg20726664	3.2382207	1.1616829	9.026623	0.0246723
cg27232130	10.718039	0.2324903	494.1125	0.2249212
cg08112313	0.4146068	0.1011587	1.699299	0.221226
cg16489610	0.9691761	0.4552596	2.063224	0.9352709
cg00151914	0.2701014	0.0987795	0.738562	0.0107586
cg14927712	0.68124	0.2435874	1.905222	0.4644674
cg05686118	0.3401751	0.1177752	0.982542	0.0463154
cg21034034	0.5102426	0.2276349	1.143706	0.1022779
cg26922706	3.725498	0.0679479	204.2644	0.5197323
cg12164973	852.29781	5.2738906	137737.3	0.0092996
cg16739441	2.4338392	1.1323208	5.231356	0.0227109
cg15504461	1.9629533	0.7967805	4.835944	0.1426139
cg11642722	5.50E-05	2.05E-17	1.47E+08	0.5017155
cg23357832	0.3555249	0.1319933	0.957609	0.0407912
cg23489080	97.268197	1.7905534	5283.898	0.0247196
cg07349815	0.3978273	0.1851651	0.854732	0.0181647
cg02461341	1.9242759	0.5690591	6.506947	0.2923402
cg16686951	1.1369676	0.5462143	2.366645	0.7314609
cg13297120	0.8213859	0.3209948	2.101825	0.6814751
cg26312839	8524576.7	0.0010232	7.1E+16	0.1709233
cg08392484	0.004761	1.17E-06	19.31426	0.2071379
cg03723730	0.1659773	0.0273526	1.00716	0.0509135
cg08126211	2.902465	1.0051082	8.381489	0.0489094
cg19674091	1.9362016	0.123145	30.44279	0.63833

cg06694734	1.6213675	0.7945295	3.308666	0.1841956
cg02988947	0.6190078	0.2942014	1.302409	0.2063065
cg16375290	1.4158367	0.7061338	2.83883	0.3272549
cg02167021	1.2088636	0.5486552	2.663514	0.6379178
cg25338972	0.4377579	0.2259784	0.84801	0.0143397
cg27470554	2.4175349	0.7681832	7.608179	0.1312712
cg13254553	5.5881038	0.6735033	46.36489	0.1109737
cg08711188	4.9338184	0.9371275	25.97572	0.059654
cg18315103	0.2236322	0.0758396	0.659436	0.0066352
cg06532767	3.2394671	0.0867699	120.9423	0.5245064
cg20686554	0.4484771	0.1813187	1.109271	0.0826484
cg00005619	0.3434095	0.1426579	0.826664	0.0170944
cg14625154	4.7865406	1.229044	18.64129	0.0239909
cg05002602	0.4425718	0.1842825	1.062878	0.0682204
cg11204099	0.1538693	0.0007049	33.58514	0.4957911
cg09316688	4.65E-20	1.86E-34	1.16E-05	0.008489
cg12485727	2.2001139	0.7074423	6.842256	0.1731665
cg02981620	7.13E-05	1.21E-12	4188.91	0.2954616
cg11115673	0.952257	0.4760571	1.9048	0.8900043
cg14618634	2.4376343	0.7351401	8.082896	0.1451522
cg05197660	0.0018466	3.22E-08	105.9457	0.2602102
cg23649129	14.571118	0.1932354	1098.751	0.2244971
cg20090551	1.56E-05	5.49E-14	4436.668	0.2651053
cg08219241	1.0807914	0.5525728	2.113948	0.8204347
cg16462183	0.4125944	0.1408065	1.208994	0.1065354
cg06177278	0.867065	0.3909061	1.923228	0.7256364
cg16287284	0.4860273	0.2445003	0.966144	0.0395691
cg17279445	0.8149843	0.4229841	1.57027	0.5409297
cg10884288	1.6943435	0.8465823	3.391046	0.1363556
cg19890879	1.7244404	0.7843982	3.791053	0.1751749
cg06344553	0.7618227	0.3663589	1.584168	0.4664278
cg17478979	0.4084223	0.1997779	0.834971	0.014116
cg06938705	7.1369796	1.254662	40.59777	0.0267092
cg27464065	0.2461545	0.1073448	0.564462	0.0009311
cg15843262	1.0743856	0.4989294	2.313563	0.854535
cg15067870	0.2921546	0.0448762	1.901996	0.1979741
cg01499736	0.0016555	4.85E-07	5.645417	0.1228489
cg18618936	1.8555856	1.03E-06	3351395	0.9329743
cg23892856	1.4834489	0.4400298	5.001071	0.5247599
cg08830300	1.1511326	0.3043057	4.354523	0.8357453
cg00037681	1.6941807	0.9759218	2.941064	0.0610191
cg03185622	0.3164115	0.126188	0.79339	0.0141507
cg12480416	0.0575269	0.0087452	0.378417	0.0029679
cg17751438	232.76086	2.6062814	20787.32	0.0174106
cg01502373	7.066336	2.0887133	23.90615	0.0016642
cg01234044	1.5302223	0.6515656	3.593775	0.3287773
cg00904578	0.6781494	0.3400969	1.352222	0.2700242
cg02746725	1.5327176	0.7230547	3.249026	0.2652647
cg15310387	4.95E-05	5.60E-09	0.436606	0.0324572
cg20247911	0.1350503	0.0379353	0.480781	0.001999
cg13452162	5.4622785	1.4549632	20.50669	0.0118854
cg20056997	1.7072727	0.7728754	3.771345	0.1858958
cg06550082	2.9016071	0.78038	10.78875	0.1118639
cg06705986	0.421585	0.1239078	1.434405	0.1668086
cg00933182	3.5513984	0.8153297	15.46912	0.0914054
cg05716821	0.0439759	0.0045782	0.42241	0.0067984
cg00134728	3.3958364	0.659983	17.47273	0.1435297
cg16164619	1.1786413	0.4607909	3.014807	0.7315922

cg26605164	0.3072655	0.1264662	0.74654	0.0091788
cg00239353	1.5196206	0.4429665	5.213142	0.5058389
cg12955872	3.0609694	0.7151479	13.10153	0.1315465
cg18543610	3.4426649	0.9488628	12.49068	0.0600899
cg22187354	0.5917303	0.1258352	2.782566	0.5064927
cg25283626	0.7325354	0.2649513	2.025308	0.5486065
cg02385908	1.5359373	0.4202657	5.613362	0.516343
cg22984587	2.0625427	0.6266587	6.788515	0.2336319
cg00231528	2.0942695	0.9161431	4.787423	0.079715
cg16626067	2.1817689	0.8987459	5.296397	0.0847
cg02030652	0.3411553	0.0746779	1.558519	0.1652985
cg18035255	1.664199	0.72256	3.83298	0.2314747
cg24405068	0.0639421	0.0045433	0.899914	0.0415372
cg22836787	0.2145492	0.0465565	0.98872	0.048323
cg16107105	0.2082512	0.07707	0.562716	0.001977
cg05244581	0.0073461	0.0001217	0.443459	0.0188421
cg02939879	1.8577825	0.6682479	5.164784	0.2351172
cg07195577	0.0134948	0.0004971	0.366381	0.0105862
cg13023623	2.5149896	1.2608636	5.01654	0.0088463
cg10318368	0.0247868	0.0010211	0.601681	0.023077
cg04691264	17.767028	1.002997	314.724	0.049762
cg16163756	0.8241097	0.3809529	1.782784	0.6231616
cg08905358	0.7300241	3.57E-11	1.49E+10	0.9792735
cg26999154	0.5670451	0.291658	1.102456	0.0944416
cg10671668	0.2101503	0.0617368	0.715345	0.0125616
cg22588850	3.9536006	0.8159499	19.15676	0.0877604
cg19449738	0.0593357	0.0010718	3.285012	0.167832
cg08348461	0.8319782	0.0489307	14.1463	0.8987473
cg09910207	0.4488772	0.2085316	0.966236	0.0405822
cg02576381	1309.0965	1.9360188	885184.4	0.0308763
cg03944089	0.0963519	0.0117654	0.78907	0.0292006
cg07283011	0.4050743	0.1808944	0.907077	0.0280149
cg24926370	17114.513	0.6032646	4.86E+08	0.0624127
cg16815882	3.4565553	0.5738818	20.81923	0.1758001
cg12854186	1.1267831	0.3787163	3.352484	0.8301016
cg25587223	1.4750321	0.7457608	2.91745	0.2640127
cg17979173	11.647932	1.6020239	84.68932	0.015285
cg01919208	0.1072108	0.0201635	0.570047	0.008813
cg16720578	1.5024978	0.8012074	2.817622	0.2044093
cg19853760	0.27716	0.0702332	1.093752	0.0669486
cg17940268	2.1938328	0.1375843	34.98148	0.5781644
cg16884822	0.481879	0.1954695	1.187947	0.1127732
cg06197360	0.0465836	0.0002502	8.674317	0.250196
cg15169077	0.9335553	0.4060848	2.146166	0.8713981
cg17071855	1.2489795	0.5189067	3.006224	0.6198245
cg08868213	1.9040418	0.3174524	11.42022	0.4810764
cg09363068	0.4139711	0.2078016	0.824691	0.0121385
cg08313420	5.1797115	1.5138757	17.72233	0.0087752
cg15706936	1.7294235	0.7875874	3.797554	0.1722618
cg00585901	0.4349603	0.2105828	0.898413	0.0244861
cg12303685	0.1347825	0.0474158	0.383128	0.00017
cg04139630	2822.4182	0.4420473	18020797	0.0755104
cg23611525	108.96602	9.21E-25	1.29E+28	0.8782822
cg22522598	0.4905264	0.2297963	1.047085	0.0656155
cg04746240	3.880977	0.893072	16.86536	0.0704358
cg08530610	3.5200123	0.3347635	37.01266	0.2944789
cg13664931	12.588145	1.4038888	112.8732	0.0236301
cg12076709	1.1412385	0.3920874	3.321773	0.8084974

cg11395698	1.4249842	0.6126272	3.314544	0.410913
cg18067127	0.0797577	0.0069199	0.919277	0.0426169
cg19868125	3.3916265	1.5390475	7.474188	0.0024498
cg10675659	4.71E-11	3.54E-20	0.062746	0.0265376
cg18526607	2.5852457	0.9897048	6.753019	0.0525208
cg13959344	0.056017	5.90E-05	53.20454	0.4100004
cg24668914	0.9682244	0.0958573	9.779735	0.9781667
cg14595786	1.736818	0.7905806	3.815597	0.1692007
cg19894980	1.4512502	0.4677054	4.503106	0.5191677
cg01572884	2.5999089	0.4142248	16.3185	0.3079506
cg11683364	0.6600396	0.1821326	2.391951	0.5271146
cg21242327	0.0307084	0.002396	0.393569	0.0074397
cg02595575	0.6917484	0.3163721	1.512509	0.3558436
cg18247179	3.2132915	0.48451	21.31069	0.2265542
cg06531476	1.7996125	0.6276958	5.159514	0.2742298
cg25426203	0.4140504	0.191342	0.895975	0.025165
cg07678517	0.3214258	0.1020884	1.012011	0.0524331
cg23543481	0.238783	0.0771385	0.739155	0.0129833
cg08085639	1.5432703	0.4067639	5.855198	0.5236153
cg18119485	0.328267	0.1678439	0.64202	0.0011349
cg11087660	0.0541826	0.0008933	3.286473	0.1639506
cg11879741	0.0730594	0.0010335	5.164864	0.2284857
cg22472360	4.0373058	0.3738512	43.5998	0.2503368
cg07514107	0.8123692	0.4285218	1.540047	0.5242799
cg08979183	8.72E-07	2.52E-10	0.003018	0.0007918
cg16552153	11.419513	0.3639075	358.3473	0.1660357
cg02610106	0.1782987	0.0444992	0.714405	0.0148977
cg14585353	8.1833879	1.4327343	46.74128	0.0180583
cg15563782	8.38E-13	2.44E-30	287802.2	0.1770762
cg19390934	2.3243403	0.3608277	14.97268	0.3748451
cg16922167	2.3335896	0.2540429	21.43591	0.4538949
cg06138931	0.000271	1.06E-07	0.695648	0.0403088
cg09930046	1.8232735	0.6295993	5.280067	0.2682365
cg20634074	0.1526716	0.0432583	0.538825	0.003489
cg23731826	0.2243223	0.0860321	0.584904	0.0022373
cg08691235	0.5523029	0.0648539	4.703469	0.5869801
cg00620628	5.3165921	1.00406	28.15186	0.0494457
cg19582491	0.461879	0.1891259	1.12799	0.0899628
cg03820828	1.8716934	0.904793	3.871865	0.0909905
cg14821257	0.8574028	0.3541694	2.075672	0.733064
cg22845037	1.0545493	0.5364624	2.072977	0.8775909
cg24134018	2.7996938	1.4098215	5.559772	0.0032694
cg08059719	2.472614	0.8528608	7.168603	0.095534
cg17204562	0.188792	0.0423447	0.841721	0.0288237
cg09333584	1.8300514	0.6384013	5.246055	0.2607022
cg02720618	0.5617706	0.2904295	1.086619	0.0866808
cg01082602	0.2193246	0.0600911	0.800506	0.0216295
cg17673691	0.6765416	0.3538268	1.293595	0.2373759
cg15430661	0.3597427	0.1357289	0.95348	0.0398066
cg10391633	0.3519776	0.1116634	1.109479	0.0746507
cg02628374	1.297E+09	8.42E-17	2.00E+34	0.4782546
cg11584519	0.3501442	0.1456001	0.842039	0.0190787
cg14305278	1.8840718	0.9816532	3.61607	0.0568717
cg13066703	3.1942148	1.2056397	8.462734	0.0194832
cg13657209	0.0566231	1.80E-08	178078.4	0.7068044
cg05789637	4.1699307	0.1447001	120.168	0.405025
cg05218976	0.0112577	0.0003847	0.329448	0.0092006
cg07817055	0.5780634	0.3028873	1.10324	0.096509

cg05014291	1435.4274	2.79E-05	7.38E+10	0.4223056
cg11977693	0.0015516	2.96E-05	0.081257	0.0013607
cg07195926	1.1718859	0.6372014	2.155232	0.6098864
cg07891179	4.8284471	1.0932981	21.32438	0.0377401
cg11964364	1.0469481	0.2759659	3.971869	0.9462313
cg13926338	0.0128873	0.0008495	0.195508	0.0017108
cg26312410	7.0984851	1.2750554	39.51867	0.0252631
cg26800525	0.4553569	0.2019963	1.026503	0.0578426
cg15730857	0.1775913	0.0687976	0.458427	0.0003543
cg00973823	1.0369384	0.4684005	2.29556	0.9287178
cg21801165	3.0922129	0.8201976	11.6579	0.0954684
cg27226238	2.0804416	1.024751	4.223696	0.0425974
cg16300565	1.3982142	0.522947	3.738434	0.504125
cg06708720	0.2429484	0.065837	0.896516	0.0336747
cg03780648	1.7236535	0.5384219	5.517943	0.3590925
cg09199031	114430.19	0.0054266	2.41E+12	0.1758303
cg06763568	0.3272609	0.121483	0.881603	0.0271612
cg00893368	0.4870087	0.2295066	1.033424	0.060887
cg27455017	2.8288401	0.9539324	8.388788	0.0608027
cg04752591	9.01E-18	1.30E-38	6227.752	0.1089104
cg22668767	3.2353144	0.9019104	11.60565	0.0716155
cg00335124	0.1802623	0.0644879	0.503886	0.0010876
cg25880958	0.251788	0.0770119	0.823213	0.0224993
cg07953890	0.0283774	0.001801	0.447127	0.0113372
cg10225488	1.8088298	0.7562685	4.326328	0.1828316
cg20752903	1.2793744	0.6667777	2.454789	0.4587015
cg14164044	1.5497754	0.831533	2.888404	0.1678342
cg10972932	0.8414664	0.3660747	1.934211	0.6843979
cg01409163	0.3292432	0.1129407	0.959805	0.0418392
cg09708044	0.2584726	0.0905516	0.73779	0.0114645
cg24813176	1.1525657	0.5938172	2.237065	0.6747464
cg01405004	1.7013399	0.7939328	3.645847	0.1717625
cg12887581	0.2683468	0.0820614	0.877513	0.0295469
cg00714564	4.8350811	0.6367531	36.7144	0.1276145
cg12114888	0.5615905	0.1579575	1.996637	0.3726423
cg13936972	2.8359198	0.1175367	68.42492	0.5210208
cg03314840	5.5614716	0.7404389	41.77248	0.0953437
cg21070161	1.7288038	0.7858344	3.803298	0.1735629
cg27563126	0.3167814	0.1066209	0.941189	0.0385407
cg03522707	5.14E-06	2.16E-10	0.122429	0.017864
cg17304531	1.254463	0.0315459	49.88536	0.9039721
cg13622546	0.3169084	0.1165796	0.86148	0.0243102
cg22959827	0.4311195	0.2162449	0.859507	0.0168472
cg04903600	0.1809129	0.052188	0.627146	0.0070269
cg06252564	3.3056666	0.9827719	11.11899	0.0533737
cg13090238	0.8583274	0.3875851	1.900811	0.7064643
cg21781988	2.0462278	1.0207073	4.102105	0.0436202
cg25484252	3.7017558	1.3574307	10.0948	0.010558
cg16269431	0.3156969	0.0971039	1.02637	0.0552768
cg08380397	0.2867044	0.1135157	0.724124	0.0082221
cg19290410	1.8807223	1.0042118	3.522281	0.048485
cg17245290	0.5449768	0.0673586	4.409232	0.5693216
cg13441112	1.0191915	0.4623334	2.246758	0.9624067
cg16919569	1.959847	0.5021283	7.64944	0.3328228
cg26034629	1.4537244	0.5714789	3.697975	0.4322284
cg04239886	1.5356786	0.346271	6.810587	0.5724394
cg14066773	0.9602688	0.2547282	3.62	0.9522517
cg22540040	0.1909288	0.0685869	0.531498	0.0015245

cg13880412	0.0367605	0.0062566	0.215985	0.0002559
cg00545916	3.0634937	0.7258349	12.92993	0.1275527
cg16555181	0.0029764	0.0001351	0.065575	0.0002271
cg25204955	0.4354846	0.1177949	1.609974	0.2127229
cg05920653	0.3958625	0.0432772	3.621011	0.4118933
cg15483381	0.1313683	0.0491072	0.351427	5.28E-05
cg14947010	2.218097	0.9018479	5.455415	0.0827456
cg20670084	2.4915454	0.4814127	12.89496	0.2764185
cg18856004	0.3862166	0.1306937	1.141319	0.085276
cg14181409	0.6297536	0.2844306	1.394328	0.2541694
cg13103143	0.9889719	0.342268	2.857601	0.9836574
cg14829948	2.1110346	0.9975116	4.467584	0.0507663
cg02911077	1.4265654	0.6223148	3.270192	0.4012669
cg03774288	0.5571082	0.3062677	1.013393	0.0553167
cg22775642	0.4303635	0.1548192	1.196316	0.1060222
cg01565013	1.9802762	0.8694643	4.510241	0.1037599
cg19874091	0.0279516	0.0023215	0.336542	0.0048357
cg00325866	1.7230962	0.7321783	4.055106	0.2127357
cg14605948	2.633083	0.9243624	7.500442	0.0698776
cg23498518	0.4555235	0.2069209	1.002807	0.0508194
cg01247188	13.050151	0.1019363	1670.714	0.2994462
cg01057248	2911.2682	0.4680703	18107284	0.0735121
cg06823672	3.05E-13	3.84E-21	2.42E-05	0.0019007
cg02253535	0.3770212	0.1483642	0.958081	0.0403686
cg11755819	8.9059973	0.5184673	152.9832	0.1317573
cg10992465	0.3074027	0.124756	0.75745	0.0103555
cg17277615	0.3622373	0.1808166	0.725685	0.0041775
cg18766864	0.1108665	0.0206006	0.596651	0.010426
cg09465288	0.2411232	0.0827246	0.702819	0.0091589
cg06902765	0.8563626	0.4208252	1.742664	0.6688244
cg23702046	1.381092	0.1790478	10.6531	0.7567472
cg07450902	0.6714112	0.3117292	1.446105	0.3088388
cg12744812	2.5488514	1.0024459	6.480792	0.0494033
cg24674635	1.4627611	0.7224786	2.961569	0.2906254
cg11219485	3.2306237	1.1115895	9.389194	0.0312157
cg04382470	2.8649744	1.4294506	5.742121	0.0030056
cg10502118	1.3495758	0.645489	2.821667	0.4256393
cg18821972	0.5069016	0.0950461	2.703417	0.4263075
cg00149213	1.5964775	0.7487222	3.40412	0.2259374
cg14794428	0.0299637	0.0022464	0.399672	0.007959
cg25444002	1.7618967	0.8280546	3.748883	0.1415054
cg04621069	0.7531268	0.3292151	1.722885	0.5018934
cg21258952	1.0820549	0.0610735	19.17106	0.9571177
cg14018735	0.1681823	0.0522449	0.541398	0.0028022
cg15321908	1.5479536	0.2691564	8.902484	0.6244696
cg05972047	0.3352145	0.0003568	314.8944	0.7543189
cg14909614	3.3783244	0.3154141	36.18441	0.3143051
cg27182133	0.6113308	0.2404619	1.554198	0.3012712
cg23035330	0.7753024	0.1251093	4.804549	0.784497
cg06110575	1.698353	0.5190758	5.556805	0.3811523
cg10009657	1.0613244	0.4959121	2.27139	0.878151
cg23989012	2.7442367	0.9702398	7.761828	0.0570391
cg09314702	0.440692	0.2017686	0.962536	0.0398055
cg18086187	0.3945659	0.1930226	0.80655	0.0107935
cg17431382	0.0006523	6.11E-07	0.696543	0.0392455
cg16849046	0.9283045	0.2301114	3.744922	0.9167406
cg16171723	0.8378216	0.3890835	1.804099	0.6511499
cg26104640	1.4575447	0.649378	3.271494	0.361068

cg25114752	0.2506864	0.0852885	0.736837	0.0118991
cg09449449	0.486486	0.2195498	1.077972	0.0758969
cg22591002	0.7413103	0.1692068	3.247748	0.6912673
cg12611832	0.8268395	0.3992759	1.712259	0.6086874
cg05967001	0.006324	0.0002726	0.146707	0.0015971
cg26435254	2.6028644	1.0091338	6.713582	0.0478417
cg05281206	1.4884448	0.7620014	2.907433	0.2443048
cg15105791	1.4178225	0.5515527	3.644658	0.4686045
cg14485643	0.2461812	0.1201274	0.504508	0.0001287
cg01660934	0.5499672	0.0564633	5.356826	0.6066827
cg11661236	2.131876	0.6410881	7.089346	0.2169133
cg23059701	0.3564458	0.184561	0.68841	0.0021279
cg09867343	0.1803442	0.030264	1.074679	0.0599884
cg09109724	1.5039996	0.4702365	4.810377	0.4914448
cg00618596	0.6741873	0.2918787	1.557251	0.3560053
cg11635029	0.5101555	0.2587231	1.005935	0.0520305
cg01039429	0.1248819	0.0085184	1.830793	0.1288786
cg11803771	5.00376	0.9904604	25.27876	0.0513713
cg16038636	0.2721032	0.0825459	0.896957	0.0324641
cg06060754	0.3740427	0.1249729	1.119507	0.078724
cg19870978	0.1726144	0.0242189	1.230269	0.0795763
cg18875631	0.6661176	0.3067265	1.446607	0.3045035
cg00443946	1.0640037	0.4121145	2.747062	0.8979931
cg06165982	2.7072722	1.0500151	6.980207	0.039307
cg06199563	0.617188	0.0066231	57.51375	0.8347736
cg20482337	3.728E+12	1335.1776	1.04E+22	0.0090942
cg21234162	0.1721574	0.014623	2.02682	0.1619857
cg05230392	0.6161728	0.2215305	1.713845	0.3535315
cg04407660	0.413715	0.19234	0.889883	0.0239139
cg15239628	0.5836946	0.3075166	1.107906	0.0996471
cg19003304	2.5216181	1.099953	5.780754	0.0288868
cg15309421	2.0418184	0.97027	4.296765	0.0600456
cg20060598	1.2432516	0.4874995	3.170617	0.6485145
cg20164479	0.8710212	0.4099288	1.850755	0.7195185
cg23939182	5.6259212	0.8475834	37.34262	0.073659
cg22795586	2.1838653	0.5821695	8.192232	0.2468824
cg01076838	0.1964014	0.0012741	30.27544	0.5266019
cg16696727	0.2874724	0.1098747	0.752133	0.0110717
cg20266316	0.169316	0.0446936	0.641431	0.0089648
cg19518104	1.9285837	1.0703462	3.474983	0.0287968
cg04465120	0.0860697	0.0001116	66.39484	0.4696482
cg16661473	2.7115961	0.9614954	7.647206	0.0593304
cg22094551	4.7173462	0.5073263	43.86399	0.1727264
cg15687973	0.19338	0.0475227	0.786905	0.0217538
cg19477190	5.6897552	0.313803	103.1644	0.2395838
cg01098474	0.3837933	0.1694161	0.869441	0.0217167
cg02879471	1.11E-27	3.12E-52	0.003937	0.0314015
cg07843568	0.4239664	0.1636931	1.098076	0.077182
cg13790576	1.6374738	0.6742415	3.976796	0.2760187
cg11121623	0.522252	0.2018169	1.351458	0.1805379
cg11975608	0.4283021	0.1867811	0.982127	0.0452249
cg24156658	4693.0441	38.313821	574849	0.0005686
cg23486701	1.6099786	0.3718773	6.970125	0.5241656
cg18509719	0.2771377	0.112367	0.683522	0.0053352
cg17054708	2.8435243	1.3219392	6.116492	0.0074919
cg21007342	1.4663561	0.6856164	3.136156	0.3237062
cg26548251	0.4511339	0.167748	1.213259	0.1147987
cg03221054	0.5410077	0.2469237	1.185343	0.1247634

cg07960138	0.9086397	0.4364905	1.89151	0.7978636
cg09811393	4.0842283	0.7848333	21.25409	0.0945118
cg07179000	2.8458045	0.1363506	59.39546	0.4999018
cg05014452	12.923786	0.0159251	10488.12	0.4540204
cg16854630	0.8568168	0.440807	1.665434	0.6485954
cg25753024	0.2455027	0.0336528	1.790982	0.1659943
cg16411857	1.7113839	0.8863527	3.304367	0.1094688
cg02156783	8.5443237	1.5378252	47.47319	0.014213
cg24396741	3.64E-11	2.40E-40	5.51E+18	0.483195
cg02791145	0.3646251	0.1563499	0.850346	0.0195333
cg06705122	0.3841529	0.129544	1.139176	0.0845248
cg11012061	1.6762399	0.8086863	3.474499	0.1648381
cg22248471	2.6786764	1.1107422	6.459921	0.0282492
cg03356087	2804.8157	3.38E-10	2.33E+16	0.6009255
cg24281668	0.1156991	0.009482	1.41175	0.0910673
cg02550537	0.3350638	0.138102	0.812933	0.0156085
cg04223044	0.2292521	0.0839575	0.62599	0.004054
cg05138776	1.5557067	0.8265209	2.928206	0.1708368
cg21910416	0.4302763	0.2036559	0.909071	0.0271217
cg10501085	2.3260861	0.9159585	5.90712	0.0758399
cg15237494	0.0834123	0.0121231	0.573915	0.0115948
cg13323091	0.7038027	0.3523189	1.405937	0.3197715
cg01295034	0.2200638	0.093043	0.520491	0.0005676
cg11163777	4.24E-07	1.81E-12	0.099247	0.0200075
cg11273848	0.5512891	0.2731799	1.112526	0.0964521
cg09518219	0.6349426	0.1815253	2.220914	0.4770917
cg15806044	1.7124404	0.7355786	3.986592	0.212152
cg02863842	4.30E-09	6.90E-18	2.676778	0.0622283
cg11844087	0.3068204	0.0974023	0.966494	0.0435727
cg12886634	86296.206	2.8453609	2.62E+09	0.0308841
cg01986648	0.5167994	0.2155246	1.239216	0.1390579
cg03960595	0.4541225	0.2222033	0.928102	0.0304213
cg14239324	0.6282383	0.2650899	1.488866	0.2910258
cg17877600	2.5314941	0.4428523	14.47088	0.2963796
cg13646859	123.09824	6.2211646	2435.746	0.0015766
cg14375111	1.3608406	0.3666271	5.051147	0.645202
cg21725716	0.1976354	0.0512802	0.761693	0.0185015
cg04506601	0.9227585	0.3753159	2.26871	0.8609685
cg19149898	1.293E+15	4.13E-12	4.04E+41	0.263624
cg12780873	1.6490429	0.6873103	3.956499	0.2626261
cg04645545	2.1304422	0.9916573	4.576968	0.0525633
cg01569052	0.1144795	0.0326971	0.400817	0.0006991
cg12516954	0.3927203	0.1224805	1.259215	0.1158937
cg06262280	0.3700688	0.1929483	0.709781	0.0027751
cg12112434	0.5415376	0.0838602	3.497047	0.5192621
cg24761542	0.2657821	0.0635313	1.111896	0.0695692
cg04891577	10.627289	0.254529	443.7188	0.2144962
cg18183042	0.3138968	0.1079569	0.91269	0.0333597
cg04243822	0.3571682	0.1438447	0.886853	0.0265049
cg20916523	1.3693863	0.7495973	2.501635	0.3065446
cg21130958	0.2651146	0.1100106	0.6389	0.0030939
cg07104495	0.1028452	0.0165151	0.640452	0.0147907
cg03253737	1.5552078	0.7755537	3.118638	0.2135108
cg26926665	1.6253864	0.6604956	3.999847	0.290408
cg10762626	0.4536858	0.1978041	1.040579	0.0620346
cg24211826	3.3229146	0.0225125	490.4731	0.6374713
cg06327727	1.5866011	0.6294012	3.999521	0.327824
cg00214855	1.12E-16	4.17E-33	2.987335	0.0570068

cg03932201	0.1119493	0.0321262	0.390106	0.0005863
cg25311656	3.55E-08	1.82E-17	69.13932	0.1159898
cg00333679	1.677018	0.7138145	3.939945	0.2354772
cg19720672	982.47816	8.36E-13	1.15E+18	0.6971493
cg02479177	2.4084308	0.5258715	11.03034	0.2575722
cg25202370	0.2993693	0.1052613	0.851424	0.0237238
cg15185986	0.1429328	0.0450449	0.453543	0.00096
cg02261771	1.3840323	0.5240685	3.655143	0.5118727
cg06436504	0.4270735	0.1935675	0.942264	0.0350953
cg21694161	1.2422993	0.0582897	26.47652	0.8894503
cg03735592	3.1096985	0.3509713	27.55275	0.3080717
cg07623567	2.6737067	1.0510933	6.801211	0.0389634
cg14289852	1.719582	0.9185224	3.21926	0.0902038
cg08282428	6.7824185	1.5883227	28.96212	0.0097477
cg05010058	0.1766105	0.0133134	2.342842	0.188679
cg24075113	0.233172	0.0438301	1.240454	0.0877675
cg17639749	0.1982982	0.0535428	0.734406	0.0154325
cg12855547	0.3707489	0.1297004	1.059787	0.0640827
cg25341268	0.5861966	0.2777125	1.237346	0.1611447
cg27184903	0.3317514	0.108992	1.009789	0.052039
cg00268744	0.6337837	0.1897487	2.116915	0.4586003
cg21873674	1.2722767	0.586027	2.762139	0.5426273
cg02725259	2.3666338	0.7469998	7.497934	0.1431412
cg03531211	5.5682511	1.3200302	23.48842	0.0193859
cg03517776	1.3022997	0.5764682	2.942026	0.5252806
cg08604523	0.3597189	0.1437981	0.899857	0.0288508
cg10187703	26.248944	1.9605477	351.436	0.0135659
cg26649688	1.1780298	0.1564315	8.871325	0.8736262
cg26104986	0.1638584	0.0515265	0.521083	0.0021819
cg22761205	3.2690614	0.6833278	15.63929	0.138029
cg04653776	0.002159	7.65E-07	6.092967	0.1299823
cg20790998	5.4145219	0.0922727	317.7217	0.4162272
cg19838107	34.963027	0.2497211	4895.113	0.1586303
cg26002615	0.5122129	0.2138675	1.22675	0.1332678
cg24313303	0.1070956	0.0254535	0.450605	0.0023088
cg00979931	0.1833629	0.0662778	0.507289	0.0010865
cg00619207	1.4664079	0.5962569	3.606419	0.4044131
cg19673233	1.3541739	0.5183637	3.537645	0.5360277
cg24189904	0.2270556	0.0562261	0.916909	0.0373633
cg21175326	0.3562311	0.1375673	0.922462	0.0334849
cg15375488	0.3540432	0.1192612	1.051026	0.0614393
cg12655375	0.3535021	0.132372	0.944035	0.0379977
cg26770917	1.568751	0.8321169	2.957492	0.1639617
cg10502957	0.3962618	0.1951174	0.804764	0.0104415
cg07565472	3.2083201	1.0006224	10.28692	0.0498778
cg00137385	9.1003745	0.0884937	935.8504	0.3502075
cg07926895	2.2037729	1.172981	4.140404	0.0140557
cg21198455	0.9963731	0.0008044	1234.152	0.9992021
cg24095374	3.5213701	1.1473945	10.80714	0.0277869
cg22817421	1.84E+46	1.201E+09	2.82E+83	0.0147472
cg15907392	0.2319524	0.0933418	0.576397	0.0016536
cg26269613	1.0713775	0.5182602	2.214813	0.852388
cg07644807	1.7257138	0.6463868	4.607285	0.2761357
cg10749822	0.7844545	0.2634981	2.335383	0.6627283
cg14017444	1.4832373	0.6160967	3.570857	0.3791542
cg16325482	0.1875257	0.0567082	0.62012	0.0060874
cg12017631	0.1911729	0.0749246	0.487785	0.000536
cg12878710	2937.8082	5.2796501	1634714	0.0132925

cg03196381	5.00E-05	1.63E-08	0.153508	0.0156302
cg10216717	0.4628785	0.1636535	1.309208	0.1464806
cg09237515	0.2796698	0.110117	0.710292	0.0073777
cg21095280	2.8633561	0.666356	12.30395	0.1572886
cg08790676	1.0879314	0.5798562	2.041187	0.7929322
cg16479474	0.2479482	0.1081837	0.568277	0.0009825
cg11969213	0.2956886	0.1115351	0.783895	0.0143083
cg08390979	0.3928658	0.154133	1.001366	0.0503352
cg03864338	6.771E+13	1.22E-14	3.75E+41	0.3285337
cg20517764	24.618017	0.563714	1075.096	0.0964146
cg07200060	4.0260548	0.6402145	25.31826	0.1376469
cg26585100	2.2414879	0.8735926	5.751271	0.0931778
cg26162295	1.3225447	0.5149572	3.39664	0.561307
cg22982173	6.4931046	1.12E-29	3.78E+30	0.9573343
cg07695566	0.1492193	0.0552489	0.40302	0.000175
cg14898116	11969.223	1.7969473	79725372	0.0365783
cg07155223	2.6421563	1.3295041	5.250823	0.0055586
cg01518016	3.8857738	1.0714538	14.09229	0.0389266
cg19999035	0.3321726	0.1413607	0.780547	0.0114598
cg01975495	3.9771976	0.6443719	24.54809	0.1370935
cg22040627	1.7729812	0.6178443	5.087791	0.287007
cg13415831	1.923466	0.843542	4.385936	0.119853
cg14095283	2.0370997	0.7389498	5.615774	0.1690564
cg21195376	1.318176	0.5102624	3.405283	0.5683469
cg04216240	3.6699716	0.3757773	35.84221	0.2634815
cg11936311	1.7529436	0.8002181	3.839967	0.160642
cg02259760	0.3580463	0.1413069	0.907225	0.0303709
cg11152829	0.7073162	0.322871	1.549523	0.3868031
cg04726013	1.1081573	0.328165	3.742059	0.8686266
cg22785170	1.8336752	0.4489043	7.49016	0.398417
cg26173417	0.6442195	0.2523512	1.644608	0.3578031
cg24373253	1.3948252	0.5020618	3.875096	0.5232786
cg18424233	0.5992834	0.2730347	1.315366	0.2017609
cg23595342	7.6318382	0.0819676	710.5847	0.3796266
cg00256485	2.6592346	0.26047	27.14911	0.4093245
cg08064672	0.3164021	0.0875174	1.14389	0.079269
cg07364009	0.0002878	2.40E-07	0.344688	0.0241658
cg11003309	1.4533973	0.64631	3.268345	0.3658288
cg20843286	6.59E-05	1.94E-13	22359.34	0.336722
cg23179678	0.0867337	0.001472	5.110715	0.2397647
cg19882826	15105.33	6.71E-07	3.4E+14	0.4288143
cg14161438	4982.9718	25.607004	969656.9	0.0015465
cg09547756	0.7252048	0.2915597	1.803823	0.4895014
cg07721547	0.4101909	0.1434737	1.172735	0.0963782
cg12012886	0.1526231	0.0524016	0.444525	0.0005681
cg25760227	0.8658482	0.2387061	3.140653	0.8265605
cg17265120	0.375763	0.1492437	0.946089	0.0377467
cg01349368	0.2408051	0.0712965	0.813323	0.0218656
cg13830300	1.6839322	0.8872764	3.195878	0.1109098
cg15479387	4.8300985	0.212897	109.5828	0.3227872
cg05225012	0.4132582	0.1785161	0.956677	0.0390767
cg09061632	3.2572274	0.702509	15.10234	0.1313472
cg06331715	0.2054109	0.0588346	0.717156	0.0130964
cg16814808	1.1210125	0.4350182	2.888774	0.8130281
cg02705759	0.2734905	0.100443	0.744671	0.0111865
cg24152683	3.13E-13	1.99E-21	4.94E-05	0.0027948
cg09704544	5.9092307	0.6257185	55.80626	0.1209724
cg14688962	1.9288325	0.9950601	3.738864	0.0517389

cg02625481	2.6252994	0.7215118	9.552438	0.1430158
cg25440811	1.1789744	0.5388743	2.579415	0.6802122
cg26400325	0.000232	5.14E-11	1046.657	0.2843872
cg07063463	8.8090279	2.242898	34.59764	0.0018254
cg01496136	3.0351389	0.6792229	13.56266	0.1460696
cg10724965	0.2289133	0.0725962	0.721819	0.0118595
cg04919489	3.2894605	0.1318883	82.04332	0.4681096
cg22736037	0.6847548	0.340471	1.377178	0.2881218
cg09478002	0.3121485	0.0989664	0.984543	0.0469729
cg13899314	2.4152253	0.2321416	25.12825	0.4605825
cg26990406	2.0311669	0.8729109	4.7263	0.1000686
cg18566177	0.3587035	0.1577786	0.815498	0.0144177
cg17445145	0.3474708	0.1302167	0.927192	0.034779
cg07365452	0.2971356	0.0911402	0.968723	0.04415
cg24453664	0.2108395	0.0596215	0.745591	0.0157128
cg13557773	2.4435256	0.360384	16.56793	0.3602522
cg04172166	0.3705584	0.1614125	0.850699	0.0192161
cg21050234	2.2606874	0.7061034	7.237903	0.1694925
cg17825384	1.762E+13	1677.6882	1.85E+23	0.0095794
cg07740705	0.0014051	1.65E-05	0.119364	0.0037576
cg00691240	6.94E-08	2.15E-12	0.002243	0.0018622
cg05852008	0.1103285	0.0194521	0.62576	0.0127966
cg03137447	1.6658293	0.8878367	3.12556	0.1119628
cg13229972	0.1142197	0.032606	0.400114	0.0006936
cg14883605	0.4269263	0.2071788	0.879753	0.0210407
cg01607569	1.322091	0.4048086	4.317904	0.6438099
cg05295286	11.310668	0.0070877	18049.75	0.5191548
cg24858279	1.7485602	0.0226535	134.9665	0.8010477
cg24306340	0.5621736	0.2920741	1.082051	0.0847216
cg03161309	0.3808129	0.1781705	0.813931	0.0127309
cg23239574	1.7723375	0.6966457	4.509007	0.2296602
cg08917060	10.992806	6.10E-11	1.98E+12	0.8561455
cg23480730	2.1158824	0.8814534	5.079064	0.0934388
cg12558090	0.3481149	0.1101666	1.100007	0.0722418
cg09532691	0.2771373	0.1058858	0.725357	0.0089477
cg17918184	0.5989659	0.3120103	1.149835	0.1234703
cg05038582	1.8054001	0.1965743	16.58136	0.6015515
cg20935165	2.3501421	0.9618534	5.742214	0.0608428
cg05516617	2.9901148	0.6814062	13.12108	0.1466151
cg24000528	4.1547249	0.6555397	26.3321	0.1306029
cg15913680	0.011235	0.0003783	0.333667	0.0094769
cg18145961	1.11E-06	4.84E-11	0.025646	0.0074764
cg06629170	2.6460415	0.2566317	27.28242	0.413692
cg08483876	0.0229699	1.57E-05	33.64311	0.3102797
cg21582785	0.3698734	0.1492998	0.916319	0.031653
cg13497926	1.8087496	0.9209878	3.552246	0.08526
cg10531637	1.3787062	0.3762165	5.052492	0.6279239
cg18395917	2.0973857	0.73713	5.967776	0.1650434
cg05858607	4.43E-09	2.84E-23	690930.3	0.2486801
cg03096285	0.3371558	0.1590875	0.714538	0.004553
cg24494009	2.0966049	0.8455413	5.198744	0.1100775
cg14320087	0.2520087	0.0458793	1.38425	0.1127752
cg02630694	0.0270094	0.0013467	0.541701	0.0182418
cg27000496	0.3880151	0.0618253	2.435181	0.3123857
cg03294028	5098.0013	1.38E-29	1.88E+36	0.8234398
cg02466830	3.0886575	1.185567	8.046618	0.0209772
cg07213685	0.1881283	0.0510169	0.693735	0.0121017
cg07881061	5.7228785	0.7917941	41.36345	0.0838753

cg06365057	1.8216829	0.4683747	7.0852	0.3867861
cg10319905	0.5143153	0.1931323	1.369632	0.1833387
cg26562691	0.8374578	0.3491636	2.008616	0.6910645
cg08552553	0.7266008	0.3627667	1.455339	0.3674969
cg11865578	0.6070768	0.0066032	55.81309	0.8287018
cg01430327	5.6048988	0.6573803	47.78801	0.114951
cg10615591	0.1604471	0.0661502	0.389164	5.17E-05
cg11761821	0.0100304	0.0005034	0.199843	0.0025715
cg15854125	1.0356285	0.4131649	2.59588	0.9404774
cg20374595	1.9681987	0.8179907	4.735758	0.1306621
cg04985273	1440.2731	0.9226668	2248251	0.052561
cg01375719	20.146686	0.9478465	428.2223	0.054152
cg16908552	0.5646838	0.3065151	1.040301	0.0667692
cg12609140	0.1934859	0.059055	0.633931	0.0066723
cg15821816	573.48403	3.6038485	91259.09	0.0140654
cg10413513	0.5094155	0.2090133	1.241568	0.1378288
cg05219445	1.8541324	0.5683377	6.04888	0.3061231
cg13896192	13.415949	1.6964291	106.098	0.013859
cg20817150	2.51E-06	6.54E-11	0.096426	0.0166546
cg11227541	118.7732	3.6147684	3902.622	0.0073364
cg26563932	0.1112929	0.013767	0.899696	0.0394855
cg04792863	2.4242805	0.861676	6.820587	0.0933707
cg02674384	2.3414113	1.0430328	5.256025	0.0391995
cg04778192	30285.916	17.706357	51802678	0.0065956
cg04743106	0.8920457	0.223123	3.566398	0.8716448
cg02488702	1.3685756	0.6777608	2.763511	0.3815051
cg27251709	5.06E-10	2.15E-55	1.19E+36	0.6879915
cg12000995	0.5443622	0.3075196	0.963614	0.0368727
cg21670438	1.0869867	0.2240761	5.272941	0.9175489
cg03277137	4.7789628	1.2013984	19.00992	0.02639
cg15731180	1.697694	0.9410556	3.062693	0.0787216
cg19672997	0.3575958	0.0139251	9.183059	0.5346107
cg25407064	2.848E+12	0.0008667	9.36E+27	0.1156782
cg24113818	0.4731533	0.2483832	0.901325	0.0228506
cg20622131	1.8676691	0.6379679	5.467654	0.2543517
cg17797940	0.1719063	0.0450206	0.656406	0.0100012
cg10593047	0.3265176	0.1380628	0.772212	0.010817
cg10207510	0.9240928	0.4410065	1.93636	0.8343251
cg04785900	0.3166897	0.1240538	0.808459	0.0161891
cg08644598	47.792839	1.7395558	1313.068	0.0221686
cg02980023	4.3407197	0.5406529	34.85018	0.1671819
cg12166018	0.1255764	0.0225056	0.70069	0.0180068
cg08077682	15.806102	0.1810056	1380.25	0.2261045
cg03302259	1.588445	0.6402573	3.940849	0.3181936
cg03324138	3.8688131	0.8252264	18.13771	0.086111
cg05488681	1.0181821	0.4324877	2.397051	0.9670992
cg14217534	6.089E+12	0.006546	5.66E+27	0.0941324
cg09232555	1.3811587	0.7836603	2.434217	0.2640619
cg23007391	1280.1024	0.0658541	24883208	0.1555949
cg14788563	1.9375338	0.2438214	15.39667	0.5316887
cg10528559	1.0463644	0.1281912	8.540977	0.9662528
cg13917589	0.7479348	0.0061275	91.29472	0.9056857
cg13737776	0.0064112	0.0001397	0.294161	0.0096876
cg27523644	0.4081542	0.1847758	0.901578	0.0266777
cg02807948	0.1668092	0.0372316	0.747357	0.0192552
cg00747944	3.9744489	1.2520732	12.61607	0.0192111
cg19584660	0.5103756	0.1821855	1.429769	0.2006363
cg08495813	1.3136639	0.6705201	2.573693	0.4265591

cg19449919	0.5237259	0.259752	1.055964	0.0706431
cg09868780	4.1635365	1.3465197	12.87396	0.0132662
cg16415819	0.4390948	0.2000847	0.963613	0.0401316
cg18065177	2.4907689	0.5790131	10.71466	0.2202281
cg25352188	0.7738467	5.77E-15	1.04E+14	0.9876755
cg27033231	0.2095043	0.0566938	0.774195	0.0190918
cg25360180	3.0033759	0.9854228	9.153702	0.0530962
cg11824121	1.8117051	0.4997615	6.567684	0.3657938
cg00714464	0.7691877	0.3464898	1.707553	0.518961
cg01410163	1.6907998	0.7248801	3.94383	0.224217
cg27518324	0.2506448	0.1056979	0.594362	0.0016841
cg07172334	1.0766968	0.497651	2.329496	0.8511334
cg04465154	2.229479	0.7707987	6.448606	0.1389903
cg15683166	0.6087145	0.2506139	1.478503	0.2729268
cg05141432	1.1919815	0.2379827	5.970266	0.8308318
cg11828983	1.7994757	0.8152468	3.971941	0.1458584
cg20150591	0.4266212	0.1988435	0.915321	0.0287325
cg16085531	4.2824414	1.1458197	16.0054	0.0305933
cg22587479	2.0410178	0.7165916	5.813289	0.1815664
cg10091265	2.9233565	0.9997421	8.548217	0.0500551
cg02587648	1.2911864	0.6676061	2.497224	0.4476341
cg26136365	0.0642128	0.0141486	0.291426	0.0003742
cg21541638	2.1414803	0.8140824	5.63326	0.1227978
cg14266859	4.6036976	0.9227448	22.96846	0.0626147
cg20956548	11.636692	0.8822449	153.4864	0.062214
cg22717014	1.6853423	0.8089274	3.51129	0.1633903
cg20707126	1.686535	0.7911304	3.595362	0.1759511
cg11158239	9.94E-10	2.99E-19	3.29924	0.063847
cg10271186	1.4483441	0.5976494	3.509919	0.4121062
cg00815399	2.3731177	0.9960476	5.654035	0.0510543
cg04303901	0.514464	0.2399274	1.103139	0.0876836
cg05989795	1.3596343	0.6153489	3.004158	0.4475428
cg14453174	0.7686876	0.3430629	1.722369	0.5227566
cg11128216	1.3864522	0.6617835	2.90465	0.3865262
cg00262054	1.2476259	0.1923132	8.093933	0.8166141
cg20464360	4.7431911	0.5715866	39.36037	0.1493354
cg04236786	2.6450299	0.5694086	12.28675	0.2144986
cg14804187	2.2755906	0.2398886	21.58632	0.4738023
cg23504719	1.5411724	0.6670503	3.56077	0.3113742
cg04101060	1.5231403	0.2091592	11.09182	0.6778664
cg21388543	0.3707933	0.0414485	3.317073	0.3748547
cg20504236	0.1978644	0.0655474	0.597283	0.0040501
cg03352975	32.050708	2.9859855	344.0231	0.004192
cg07034850	1.5378486	0.7663619	3.085981	0.2258435
cg24742349	0.012022	0.0001571	0.920243	0.0457689
cg21467717	3.1183715	0.780402	12.46055	0.1075832
cg15409133	2.0883549	0.8685636	5.021194	0.0999398
cg05178005	105822.81	3.0569094	3.66E+09	0.0300449
cg18245160	2.3872976	1.0203737	5.585394	0.044806
cg02454483	8.31E+27	9.78E-08	7.06E+62	0.1171978
cg05737535	0.4662494	0.2337967	0.929818	0.0302671
cg07179634	0.3144488	0.0985299	1.003534	0.0507006
cg02473123	2.3829925	0.7146336	7.946244	0.1576038
cg16819777	3.20E-16	1.11E-27	9.29E-05	0.0080627
cg09805943	0.0004507	7.71E-07	0.263398	0.0177684
cg13541970	3.4037379	1.0762797	10.76433	0.0370602
cg21244177	3.0912376	0.6846832	13.95645	0.1422593
cg01906170	2.8843372	0.8220891	10.11983	0.0981151

cg08667740	3.1483096	0.4545798	21.80443	0.2454333
cg27597505	0.2098861	0.0883135	0.498816	0.0004083
cg26578476	1.1092613	0.3694251	3.330744	0.8533508
cg04936970	0.0143454	0.0002331	0.882875	0.0434634
cg06014958	2.8091446	0.9798424	8.053635	0.054597
cg26305986	2209.4499	0.3504908	13928095	0.0845107
cg09641325	2.597896	0.6195872	10.89284	0.1917529
cg01242872	0.2831436	0.0966666	0.829348	0.0213794
cg26309457	2.1472212	0.9489641	4.858518	0.0666203
cg04694035	1.792307	0.786588	4.083922	0.1649328
cg15962969	0.4543347	0.2454409	0.841017	0.0120368
cg03218479	1.3548643	0.4520366	4.06086	0.5876342
cg25182501	0.5137124	0.1823061	1.447568	0.2076048
cg20011983	0.2594919	0.0367073	1.834406	0.1763957
cg07959138	1.3210257	0.2474666	7.051897	0.7445793
cg16179969	0.0280662	0.0024984	0.315289	0.0037889
cg21248554	0.4000596	0.1853904	0.863301	0.0195679
cg19308397	0.3225532	0.087442	1.189824	0.0893218
cg08167706	2.8495222	1.1756947	6.906365	0.0204326
cg14105018	0.6108816	0.2305427	1.618686	0.3215475
cg00588262	1.6235171	0.5192575	5.076109	0.4047409
cg09423283	1.1658668	0.4139604	3.283515	0.771443
cg03756929	0.532649	0.2667224	1.063709	0.0742698
cg19711800	0.2320236	0.0403384	1.334583	0.1017076
cg07992918	962.21257	7.4422216	124405.5	0.0056215
cg14947634	0.3881917	0.2065073	0.729721	0.0032988
cg13736585	0.0039821	0.000104	0.152528	0.002969
cg07766803	0.9852851	0.5004696	1.939752	0.965787
cg08975850	2.5226206	0.9603669	6.626233	0.060396
cg24527008	2.363279	1.1095105	5.033831	0.0257918
cg15975832	1.0099182	0.5155888	1.978194	0.9770468
cg27032056	2.5966363	1.0964987	6.149136	0.0300521
cg06654127	0.7663179	0.326818	1.796851	0.5404463
cg03831405	1.1884062	0.5502061	2.566873	0.6604241
cg14206626	0.6756875	0.2771884	1.647088	0.3885126
cg17061862	0.40517	0.1557876	1.05376	0.0639417
cg02922676	3.9736605	0.8290235	19.04648	0.0844438
cg21144340	2.4449107	0.8811328	6.783981	0.0859916
cg24279744	0.1588327	0.0449436	0.561322	0.0042837
cg19567689	2.8104255	0.9829294	8.035665	0.0538749
cg02148034	1.9237931	0.8619847	4.293556	0.1101813
cg02127209	1.8259443	0.7598391	4.387867	0.1783065
cg00503852	1.06E-07	7.92E-33	1.41E+18	0.5863722
cg07352544	0.3070423	0.0685307	1.375661	0.1227947
cg23122901	1.1227551	0.3708317	3.399329	0.8376864
cg21792155	1.2924869	0.7423984	2.25017	0.3644167
cg04161398	0.0036613	3.36E-05	0.399197	0.0190991
cg20307125	0.472754	0.2100236	1.064149	0.0703315
cg09802688	1.7851694	0.6628187	4.807996	0.2516256
cg21043746	1.4772588	0.4557453	4.788406	0.5155007
cg24476826	0.3413876	0.1284498	0.907323	0.0311638
cg26958236	0.5244968	0.0132615	20.744	0.7309054
cg16773899	8.63E-07	2.77E-11	0.02684	0.0081594
cg14689537	1.2196281	0.6858678	2.168775	0.4990117
cg24964215	0.0953455	0.0137185	0.662665	0.0175039
cg16954727	7.7615146	1.0367882	58.10358	0.0460277
cg13825375	0.029264	0.0005444	1.573191	0.0823726
cg19379572	0.0606072	0.0083712	0.438791	0.0055113

cg15477209	26.823496	1.3930249	516.5018	0.029286
cg22378252	0.1430745	0.0278766	0.73432	0.019805
cg22077197	0.4971867	0.2280841	1.083787	0.0788177
cg03385727	0.4965447	0.1986065	1.241433	0.1342905
cg05413957	1.8677216	0.3279115	10.63819	0.4815557
cg17922283	0.4770851	0.1719209	1.323924	0.1552789
cg20907456	0.1246538	0.0359518	0.432206	0.0010297
cg07949777	4.7117108	0.401505	55.29251	0.2173221
cg20660350	0.8194019	0.2489224	2.697305	0.7431677
cg00753630	0.0778829	0.0027239	2.226836	0.1356966
cg11808871	0.4125979	0.1416558	1.201765	0.1045866
cg03550384	3.6050668	0.4256569	30.53282	0.2394338
cg22782986	1.7639681	0.971143	3.204043	0.0623488
cg22897701	0.1059871	0.0157276	0.714238	0.0211281
cg15285733	0.2352432	0.018975	2.916442	0.2598918
cg20298425	0.0262782	9.55E-05	7.230395	0.2041889
cg25017774	1.6757608	0.8943079	3.140053	0.1071097
cg07834841	0.3093142	0.1090458	0.877386	0.027393
cg14938677	2.844424	0.483423	16.73637	0.24764
cg06611115	0.4457354	0.2256783	0.880369	0.0199717
cg07648709	0.6223822	0.275154	1.407792	0.2548368
cg08202754	1.7681536	1.0154527	3.078792	0.0439931
cg25808926	4.31E-09	1.40E-21	13325.46	0.1892809
cg07669182	5.0284216	0.9146468	27.64458	0.0632593
cg08370146	2.3561828	1.1244386	4.937217	0.0231649
cg13262424	1.4117998	0.7206005	2.765997	0.3148788
cg16579981	0.6813679	0.3675914	1.262984	0.2230508
cg08020574	8.484E+13	0.0023905	3.01E+30	0.0990437
cg22970435	1.6760762	0.6746373	4.164062	0.2660084
cg03401488	0.6902642	0.0080992	58.82898	0.8701758
cg11093142	1.3504267	0.5580729	3.267767	0.5052097
cg22074858	1.6597873	0.5937721	4.639649	0.3339984
cg08482080	1.21E-06	4.93E-10	0.002945	0.0006168
cg16333440	6.8422499	0.2506613	186.7715	0.2543465
cg19042287	3.4297362	0.5016136	23.4505	0.2089128
cg25725418	0.2776655	0.1044199	0.738347	0.0102323
cg07872854	0.9338704	0.2189964	3.982321	0.926331
cg19738980	1.9654659	0.4501082	8.582505	0.3689121
cg20495819	1.3178542	0.6985184	2.486319	0.394117
cg01371207	1.915E+09	0.0011524	3.18E+21	0.1365666
cg18892169	1.0605733	0.4813575	2.336758	0.88399
cg23741159	2.216205	0.0002023	24274.49	0.8668286
cg11518945	0.2897822	0.0763303	1.100137	0.0687964
cg27152133	0.067211	1.99E-07	22753.01	0.677693
cg14317513	1.7772675	0.7123326	4.434277	0.2176517
cg17938867	0.4230086	0.1807676	0.989869	0.0473185
cg15999590	0.0980611	0.0226495	0.424556	0.0018979
cg25130385	0.9676925	0.4476859	2.091709	0.9334505
cg04784635	2.4403386	0.7631637	7.803375	0.13252
cg05749642	0.3803054	0.1914831	0.755326	0.0057541
cg15703773	2.3938019	1.1122981	5.151755	0.025607
cg25796145	6.0203531	0.7913595	45.80049	0.0829288
cg21771250	5.3481954	1.5714749	18.2015	0.0072894
cg13918640	0.615477	0.2362337	1.603547	0.3205009
cg03406535	1.4733814	0.6165519	3.520957	0.3832453
cg09550809	3.3912125	0.9719894	11.83174	0.0554411
cg23172400	0.4921297	0.214931	1.126834	0.093455
cg10294853	2.9726338	0.3567563	24.76915	0.3138695

cg06588841	1.9081149	0.9883135	3.683955	0.0542366
cg23141745	0.0010298	1.24E-05	0.085762	0.0022993
cg23869158	0.5219043	0.250578	1.087023	0.0823758
cg13607226	0.4938324	0.1996055	1.221762	0.126862
cg11411904	1.61E-08	1.11E-20	23408.36	0.2091876
cg01963702	0.9608607	0.2009235	4.595049	0.9601184
cg19378416	0.2476825	0.1044985	0.587058	0.0015262
cg02935097	64.759493	2.1053424	1991.976	0.01704
cg19025497	0.0003491	7.13E-08	1.710166	0.06633
cg09160231	1.2252391	0.5914738	2.538085	0.5845925
cg24317585	1.4147675	0.5629388	3.555568	0.4605568
cg05366812	0.0008276	9.26E-10	739.3902	0.3100545
cg00584238	2.2930373	1.1630265	4.52098	0.016575
cg00367962	3.4870752	1.5546592	7.821453	0.0024409
cg27632288	0.3918737	0.179425	0.855873	0.018751
cg01515802	1.5257532	0.7196996	3.234576	0.2704569
cg19814934	1.9401056	0.8131723	4.628797	0.1352247
cg09477124	5.9028946	0.7117428	48.95612	0.0999857
cg14121142	2.2810741	0.7489788	6.947191	0.146702
cg00213301	1.2524089	0.5918874	2.650045	0.5561596
cg20000602	0.5602977	0.3026122	1.037412	0.065314
cg21640187	2.2367392	0.8543313	5.856045	0.1011388
cg08223235	0.6115527	0.3018525	1.239005	0.172233
cg02937491	0.7420912	0.3845889	1.431917	0.3737685
cg25677261	1.4676477	0.7552181	2.852143	0.2577295
cg04336379	0.3888506	0.1588626	0.951796	0.0386272
cg09244244	0.144042	0.0317166	0.65417	0.0120859
cg08220966	1.110417	0.2587302	4.765682	0.8879336
cg04871173	0.3614879	0.1150402	1.135894	0.0815362
cg09485853	0.2830878	0.099941	0.80186	0.0175181
cg06383163	0.229467	0.0739955	0.711598	0.0107973
cg26019498	1.9598201	0.8138477	4.719427	0.1334627
cg23254497	0.0001963	7.84E-07	0.04915	0.0024525
cg09085259	0.2999234	0.1490352	0.603576	0.0007383
cg12352896	3.0934598	0.4237046	22.5853	0.2655542
cg00527307	0.3536487	0.171317	0.730035	0.0049408
cg20557071	0.1137827	0.0402077	0.321991	4.22E-05
cg10369363	0.319428	0.1166817	0.874467	0.0263492
cg00061678	0.5418026	0.2653445	1.106298	0.0924502
cg05477174	5.499697	0.9159611	33.02178	0.0623245
cg11999002	3.2206365	1.0922562	9.496398	0.0340134
cg05698228	1.0949644	0.5053528	2.372495	0.818121
cg02895582	0.0475294	0.007227	0.312584	0.0015242
cg18524739	0.2052106	0.0064804	6.498259	0.3689982
cg03871754	0.1590039	0.0365212	0.692263	0.0142858
cg14934253	2.1555767	0.9903631	4.691724	0.0529215
cg18688230	245.9563	0.1242568	486850.8	0.1551744
cg24947379	0.5532462	0.2872114	1.0657	0.0767727
cg27494226	1.4192846	0.7272308	2.769917	0.3047242
cg06599908	0.2498597	0.0685735	0.910408	0.0355319
cg22844586	0.7849148	0.2804308	2.196945	0.6446724
cg26032317	0.2673284	0.1070682	0.667467	0.0047147
cg18295744	1.1340678	0.3153792	4.077979	0.8472127
cg07085664	2.8190186	0.551135	14.41909	0.2133029
cg12354377	0.9077368	0.3969534	2.075775	0.8185757
cg11317019	3112801.4	0.0224199	4.32E+14	0.1180645
cg24020152	0.6501349	0.2347442	1.800579	0.4074247
cg21817287	0.7504326	0.3713573	1.516461	0.4237698

cg12666279	0.3981817	0.1481295	1.070339	0.0679665
cg22374165	1.9850968	0.1488205	26.47894	0.6039438
cg02059509	0.5194134	0.2588136	1.042412	0.0653154
cg19604907	1.5390022	0.6330109	3.741686	0.3415258
cg24121503	1.0282936	0.4178252	2.530694	0.9515819
cg15518950	1.3678129	0.5581678	3.351881	0.4934039
cg08927844	0.4184415	0.1958778	0.893891	0.0244739
cg05059480	0.2076287	0.0647813	0.665465	0.0081619
cg17507671	2.0899993	0.9544976	4.576331	0.0652559
cg19820058	3.9816658	0.6952655	22.80231	0.1207182
cg12778007	0.2524085	0.1017203	0.626326	0.0029877
cg01354088	0.1408116	0.0501912	0.395048	0.0001957
cg13100965	1.3554996	0.4124176	4.455143	0.6163546
cg11346837	2.0065635	0.8669923	4.643982	0.1038206
cg02083836	2.15308	0.8208806	5.647293	0.1190486
cg03771739	0.0043539	1.40E-06	13.56457	0.1852863
cg00111948	1.75E-11	2.38E-22	1.285639	0.0523454
cg00415263	3.3936993	0.2719788	42.34593	0.342683
cg11771198	1.1229723	0.0597739	21.09728	0.9382273
cg14001992	0.4047581	0.2261344	0.724477	0.0023262
cg11252801	0.8083866	0.3303485	1.978181	0.6413006
cg27305255	0.0005047	2.52E-07	1.011606	0.0503487
cg03878133	0.1933332	0.058361	0.640457	0.007165
cg07941108	0.970866	0.389744	2.418461	0.949374
cg05284631	1.5929827	0.7153735	3.547229	0.2543183
cg07826387	0.6938994	0.3200333	1.50452	0.3547192
cg07501506	0.3632217	0.1698445	0.776769	0.0090195
cg07230786	0.3599567	0.1332669	0.97225	0.0438549
cg15260508	0.4709243	0.2001955	1.107766	0.0844441
cg14329889	4.4435913	1.2791807	15.43606	0.0188997
cg14800299	0.5029951	0.0554852	4.559846	0.5412264
cg27609113	4.6915746	0.9065341	24.28025	0.0653333
cg18173726	0.7479734	0.4264195	1.312004	0.3111445
cg20696173	2.6227375	0.7492145	9.181285	0.1314754
cg12971338	3.88E-10	8.54E-24	17600.72	0.1768013
cg16215084	0.2104237	0.0541046	0.818381	0.0245003
cg04541607	1.651064	0.565642	4.819325	0.3589168
cg01966878	0.4394658	0.168639	1.145228	0.0924768
cg12209165	1.4978256	0.4517866	4.965799	0.5088232
cg17141577	2.4758455	1.0074015	6.084775	0.0481505
cg10831196	0.4762212	0.2259827	1.003558	0.0511015
cg04021098	8.6214998	0.5784384	128.5016	0.1180926
cg10952190	0.25264	0.0198213	3.220122	0.2893991
cg22215815	1.4551022	0.5822659	3.63635	0.4221864
cg22544416	9.7291291	1.0978968	86.21571	0.0409667
cg04388983	0.5609955	0.2635851	1.193982	0.1336359
cg00089915	1.6912508	0.8294329	3.448536	0.1483147
cg14455590	0.5524833	0.2552471	1.195852	0.1320706
cg11547104	0.5709256	0.2721306	1.197793	0.1381878
cg25390243	1.5497827	0.3657499	6.566854	0.5520488
cg11414507	1.8945497	0.7156577	5.015413	0.1982945
cg18381051	1.4447683	0.7758239	2.690502	0.2461121
cg20295992	1.4151052	0.7830647	2.557289	0.2501427
cg06840246	2.9117405	0.2555217	33.18009	0.3892993
cg04502490	0.1514451	0.0588643	0.389635	9.05E-05
cg12253753	0.5715617	0.2634536	1.240001	0.1568947
cg00089814	0.4277958	0.1379523	1.326612	0.1414256
cg15188353	2.282238	0.8250044	6.313433	0.1119644

cg17663418	29.568117	1.2967781	674.189	0.0337652
cg04498110	0.543635	0.2639308	1.11976	0.0982998
cg10869131	1.6962009	0.581128	4.950884	0.3336369
cg06892679	1.3106497	0.1985674	8.650978	0.7787404
cg05230642	0.2021259	0.057238	0.713772	0.0129996
cg00319912	0.2459779	0.0734778	0.823447	0.0229015
cg13953753	5.2249379	0.9588264	28.47228	0.0559583
cg01429449	4.3316559	0.8899007	21.08465	0.0694464
cg07674277	2.3078907	0.7375403	7.221788	0.1507429
cg03477080	1.383024	0.6510604	2.937908	0.3989132
cg21611708	0.3724899	0.1660653	0.835507	0.0165753
cg03598112	1.5282309	0.7717357	3.026282	0.2237388
cg01619796	0.2808309	0.121376	0.649766	0.0030041
cg08049745	2.5444786	0.7214194	8.97449	0.1464415
cg25662822	0.4613447	0.2296697	0.926717	0.0297185
cg18653560	2.2185793	0.8078786	6.092616	0.1220937
cg09414156	57628.888	5.48E-23	6.06E+31	0.7298681
cg08548066	0.6747209	0.3798625	1.198456	0.1794862
cg08619378	0.6846818	0.2755971	1.700994	0.4145849
cg21188227	13.29392	1.2388244	142.6581	0.032611
cg08553630	0.7535548	0.3716402	1.527943	0.432718
cg27303654	0.0001769	4.73E-07	0.066147	0.0042559
cg07285248	10.807559	0.2980922	391.8362	0.1938475
cg02449694	10.838384	1.4325084	82.0034	0.0209949
cg15798088	1817312.7	55.817831	5.92E+10	0.0065552
cg18272456	1.3826012	0.2963745	6.449901	0.6801288
cg20035362	0.5217511	0.2449076	1.111538	0.0918097
cg27297263	1.9650078	1.096711	3.520759	0.0231942
cg14266251	1.6183245	0.7184743	3.645188	0.2452623
cg13439181	0.6650351	0.3352234	1.319334	0.2431779
cg21576590	0.0001508	3.43E-18	6.64E+09	0.5830124
cg22843950	0.3127948	0.1260426	0.776251	0.0122063
cg00979347	1.58E-12	3.39E-24	0.737199	0.0474563
cg01415909	1.8912723	0.8759277	4.083569	0.1046648
cg16034411	0.3829987	0.1409286	1.040868	0.059912
cg16547341	0.227892	0.0598924	0.867135	0.0300785
cg25994968	2.0974701	0.6965509	6.31595	0.1878332
cg19624630	0.4424904	0.1911032	1.024565	0.0569998
cg09379146	1.0805098	0.2053944	5.684194	0.9271659
cg10600783	8.24E-14	9.22E-25	0.007369	0.0191995
cg24404823	0.3233927	0.1554296	0.672863	0.0025288
cg09661503	2.9722806	0.5109758	17.28938	0.2252946
cg24998096	0.3052941	0.1107664	0.841451	0.0218086
cg18315963	0.0060466	0.0001395	0.26209	0.007901
cg25856018	1.3248505	0.628502	2.792718	0.4597008
cg25005674	0.7234866	0.2964412	1.765722	0.477077
cg26789888	0.5633865	0.272553	1.16456	0.1214392
cg01511828	1.8566414	1.0209764	3.376295	0.04256
cg16960593	0.4872788	0.2018052	1.176583	0.109951
cg24278841	3.6548255	0.9215636	14.49466	0.065218
cg11828966	5.582015	1.935038	16.10247	0.0014666
cg07948085	0.9241969	0.3837497	2.225774	0.8604623
cg27009246	0.7929347	0.4053316	1.551188	0.4979817
cg09913956	1.9282229	0.4759888	7.8112	0.3576225
cg09969806	2.0009914	0.9244875	4.331012	0.0782946
cg04580170	5.6200112	0.2484692	127.1165	0.2779662
cg02821150	2.1350136	0.9709926	4.694457	0.0591954
cg11342764	2.7111224	1.0317794	7.123795	0.0430284

cg20471413	0.1624088	0.0486758	0.541884	0.0031106
cg22902964	3.1139587	1.0563586	9.1794	0.0394592
cg16715667	4.7681198	0.6166439	36.86888	0.1344718
cg01765406	2.9331918	1.3209859	6.513025	0.0081949
cg22807707	15.204643	1.2316608	187.6987	0.0337993
cg10438034	0.1593375	0.0366831	0.692101	0.0142427
cg12841020	0.1769891	0.0507819	0.616857	0.0065606
cg00707688	0.2148737	0.0565939	0.815825	0.0238833
cg04498032	88505.176	9.75E-05	8.03E+13	0.2790831
cg02482218	0.0195245	0.0009663	0.394507	0.0102753
cg11217654	0.6052728	0.223811	1.636895	0.3226055
cg26481896	0.4149496	0.0038225	45.04408	0.7130202
cg25190638	2.0283684	0.5087264	8.087409	0.3162368
cg03814610	1.2439132	0.7590361	2.038533	0.3864794
cg10104336	0.4829732	0.1981124	1.177428	0.1094373
cg13810385	0.2841528	0.1235257	0.653652	0.0030735
cg22762215	2.1598535	0.7231269	6.451105	0.1678005
cg19727026	0.7143969	0.2179801	2.341329	0.578685
cg06770735	1.7253465	0.5698455	5.223908	0.3345562
cg11200794	1.645862	0.6083828	4.452561	0.326457
cg16201762	2.5072313	0.7317607	8.590525	0.1434893
cg24634746	0.3672178	0.1565955	0.861129	0.0212346
cg14449051	0.0107717	6.04E-06	19.20539	0.2355252
cg05303559	8.3377167	1.4919211	46.59598	0.0157071
cg05057794	0.2825319	0.1005192	0.794119	0.0165229
cg25135457	0.2701772	0.1002792	0.727925	0.0096551
cg21750428	1.7070728	0.4139222	7.040206	0.4594391
cg08855504	0.8565616	0.3254275	2.254566	0.7538551
cg17564183	0.1721724	0.0157344	1.883986	0.1495519
cg19561908	21.079457	0.1393384	3188.952	0.2339079
cg13633529	21532795	3.38E-05	1.37E+19	0.2233763
cg12680719	0.6636421	0.3048874	1.444536	0.3015187
cg03580272	0.1608219	0.0406114	0.636859	0.0092535
cg00687447	0.2017458	0.0365648	1.113129	0.0662138
cg04581728	0.7812684	0.4058652	1.503899	0.4600726
cg26403608	0.1663091	0.0352752	0.784083	0.0233653
cg12829325	0.0443982	0.0050874	0.38747	0.0048366
cg18396992	0.7170051	0.3625894	1.417847	0.3389141
cg25461186	0.4644805	0.1441915	1.49622	0.1988502
cg12727795	1.2397924	0.6011338	2.556977	0.5605823
cg16512661	3.0559098	1.0594878	8.814245	0.0387453
cg06446548	2.9289011	1.0775735	7.960907	0.0351689
cg19179099	0.4287444	0.1393553	1.319087	0.1396806
cg04074066	0.3770699	0.1371732	1.036512	0.0586972
cg05986007	0.6475797	0.0486801	8.614594	0.7421007
cg03844971	0.0036377	2.65E-05	0.499582	0.0253329
cg02985708	72448.04	1.70E-09	3.08E+18	0.4846016
cg16989443	52.734566	0.4007382	6939.528	0.1112339
cg14265704	4.1279573	0.7043997	24.19085	0.1160548
cg14480531	0.305433	0.1233166	0.756502	0.0103776
cg13933427	0.0539981	0.0055112	0.529069	0.0121858
cg12078157	1.2450085	0.5896998	2.628534	0.5654516
cg20796298	1.4733612	0.4711675	4.607264	0.5052551
cg26162564	0.9758391	0.396119	2.40398	0.9575975
cg16090347	1.4756325	0.6520263	3.339576	0.350465
cg07848042	9.96E-22	3.71E-37	2.68E-06	0.0076353
cg03246584	1.3876036	0.5374406	3.582617	0.4984751
cg05891054	0.1807997	0.0718873	0.454719	0.0002783

cg18338319	9381.3119	0.7264646	1.21E+08	0.0582519
cg09052244	1.6860694	0.6042686	4.70458	0.3183734
cg15730030	0.1469347	0.0313757	0.688106	0.0149125
cg01522430	1.0276958	0.2535006	4.166296	0.9694851
cg03165700	82.503042	0.5012107	13580.62	0.0901328
cg10708189	0.1624352	0.0427428	0.617302	0.0076271
cg10047026	0.00051	4.23E-08	6.148842	0.1138419
cg08442823	2.2836131	0.6225652	8.376454	0.2130261
cg13333265	2.2669625	0.8141356	6.312362	0.1172521
cg03790740	0.4368436	0.2280801	0.83669	0.0125001
cg16912129	0.5332974	0.2382595	1.193682	0.1261912
cg18204321	0.2220694	0.0089435	5.514017	0.3585185
cg25028406	0.0073524	5.91E-05	0.914413	0.0458989
cg22561845	1.7921504	0.8331214	3.855144	0.135489
cg23969506	0.2621776	0.1068405	0.643362	0.0034676
cg20257634	2.761512	1.0606662	7.189772	0.0374703
cg07675184	1.4487401	0.6102319	3.439427	0.4007303
cg03776087	3.7487119	0.7875241	17.84433	0.0969317
cg13754355	0.3301249	0.1352089	0.80603	0.0149567
cg16745596	1.1682309	0.5114306	2.668522	0.7121734
cg18241048	4.994E+14	6.45E-29	3.87E+57	0.5017786
cg13114921	2.4461218	0.9271242	6.453841	0.070747
cg10975889	5.0811317	0.9239762	27.94217	0.0616159
cg23154272	0.2507003	0.0776912	0.80898	0.0206342
cg24875808	2.0921508	0.8235937	5.314629	0.1206758
cg18436735	2.000642	0.1473186	27.16947	0.6023458
cg12152420	1.2687119	0.5404525	2.9783	0.5846261
cg07627452	0.212103	0.0671095	0.670362	0.0082627
cg05219493	4.8972655	0.5004283	47.92537	0.1722216
cg00153693	1.0850053	0.3568134	3.299305	0.885673
cg20821885	0.4014797	0.1870901	0.861542	0.0191546
cg16748643	1.266592	0.5807984	2.762155	0.5524545
cg01815167	0.4879382	0.1860479	1.27969	0.1446623
cg03046886	0.9016568	0.3213511	2.529896	0.8440905
cg10058779	0.1182506	0.0266672	0.524361	0.0049616
cg12607555	7.4028538	0.0277603	1974.124	0.4824333
cg04505205	1.7086663	0.7944765	3.674798	0.1703394
cg01686463	6.9581894	1.143363	42.34561	0.0352597
cg13588086	2.2200805	0.8913284	5.529676	0.0867333
cg13832290	5.7969093	1.0280505	32.68726	0.0464466
cg18420965	0.4041464	0.0576552	2.832949	0.3618363
cg13668129	0.1911877	0.0227052	1.609886	0.128022
cg08243524	1.9410503	0.7050978	5.343481	0.1992576
cg24682415	0.3358198	0.1675325	0.673153	0.0021017
cg24778160	0.2442674	0.0699992	0.852389	0.0270752
cg26297819	0.0082385	0.0002027	0.334782	0.0111203
cg02707152	0.1622497	0.0458953	0.573587	0.0047621
cg24192909	0.25314	0.1126979	0.568599	0.0008767
cg19344626	0.3518599	0.1170184	1.057999	0.0629434
cg20246331	1.12E-06	5.83E-15	216.4953	0.1592904
cg14417329	1.6574777	0.6154772	4.463581	0.3174525
cg15946224	0.3291043	0.1376582	0.786802	0.012449
cg12762816	2.9107178	0.9590444	8.834083	0.0592762
cg13701124	6.60E-15	2.85E-27	0.015259	0.0245817
cg24414282	1.0861335	0.2908207	4.056403	0.9021872
cg05221264	272.53436	4.8963885	15169.34	0.0062459
cg13725590	2.1434045	0.6708279	6.848528	0.1983226
cg25960893	1.9915056	0.8314211	4.77026	0.1221723

cg05513455	2.3536705	0.8466066	6.543493	0.1008446
cg25866552	2.4929041	1.0282195	6.044011	0.0432227
cg12909559	0.6726812	0.1918034	2.359187	0.5357209
cg03868944	0.2680447	0.1233558	0.582445	0.0008841
cg10329345	0.3129873	0.1460177	0.670885	0.0028259
cg04033718	0.1631444	0.0505974	0.526037	0.0024022
cg24663455	0.6836219	0.3132192	1.492051	0.3395171
cg18729787	0.170085	0.0595722	0.48561	0.0009347
cg08565197	0.2813976	0.0871422	0.908683	0.0339999
cg04377609	0.2155648	0.0647593	0.717553	0.0123876
cg06646650	1.39E-22	1.42E-39	1.37E-05	0.011701
cg05048259	2.1092978	0.9390632	4.737847	0.0706554
cg08274633	2.2237212	0.8431073	5.865132	0.1062947
cg06439603	1.1224505	0.4378277	2.877605	0.8099538
cg20330566	5.1102144	0.0648329	402.7936	0.4641132
cg09805254	0.7637497	0.3549774	1.643242	0.490546
cg06807029	0.4152882	0.2010125	0.857978	0.0176101
cg09050331	2.0761854	0.8027222	5.36991	0.131879
cg06226703	1.4723672	0.489704	4.426889	0.4909461
cg02237208	1.5866195	0.4336432	5.805145	0.4855015
cg09928766	0.4084886	0.1122459	1.486584	0.174338
cg04439241	1.9258097	0.7697285	4.818248	0.1613286
cg23649083	2.9664209	0.8924716	9.859868	0.0760084
cg05234387	1.2561898	0.29138	5.415653	0.7596544
cg01726982	1.8376466	0.4734507	7.132622	0.3791949
cg06159404	0.9463966	0.4582669	1.954465	0.8816351
cg23829990	0.5475702	0.2445385	1.226118	0.1431058
cg11689625	0.217746	0.0494332	0.959139	0.0438929
cg03680517	0.4205115	0.1680325	1.052355	0.0641797
cg06820405	0.4798816	0.1865101	1.234712	0.127833
cg01821993	0.4800569	0.2262357	1.018648	0.0558982
cg20117153	3.1929147	1.3383903	7.617139	0.0088708
cg07397579	0.1277402	0.0300912	0.542269	0.005277
cg04622200	0.1028038	0.0187308	0.564237	0.0088258
cg23325963	2.5325528	1.2538727	5.115211	0.0095775
cg17788761	0.0637104	0.0179069	0.226673	2.12E-05
cg08076091	0.305701	0.1434445	0.651493	0.0021415
cg07530194	0.2335776	0.0591366	0.922585	0.0379929
cg11897433	1.521062	0.6193809	3.735391	0.3602207
cg26015743	0.7109509	0.235391	2.147283	0.5452357
cg11721566	1.2491262	0.2059762	7.575226	0.8088694
cg03965648	4.6336766	1.3237448	16.21986	0.0164529
cg00470051	2.185343	0.9723399	4.911579	0.0584808
cg22202088	9.4703506	1.4803897	60.58374	0.0175834
cg16492584	3.96E-06	6.41E-10	0.024434	0.0052143
cg14086364	1.7382135	0.7271197	4.155281	0.2137496
cg26271591	0.2823925	0.0665181	1.198855	0.08651
cg23220533	1.5219267	0.5616864	4.123762	0.4089221
cg26052357	5.0783634	0.3709871	69.51664	0.2235249
cg02379082	2.3062262	1.041886	5.104858	0.0392861
cg16061928	135878.42	47.29994	3.9E+08	0.0036238
cg00901675	0.3219378	0.1123467	0.922537	0.0348523
cg12593336	2445821.5	1.02E-05	5.86E+17	0.2711973
cg20955507	0.172704	0.0390523	0.763762	0.0205988
cg14760020	0.496915	0.2174629	1.135479	0.0971909
cg15991104	0.0978241	0.0026107	3.665575	0.2086263
cg25262481	2.4412431	0.6643648	8.970475	0.178909
cg00686404	0.1666308	0.0622412	0.4461	0.0003617

cg05811846	0.5057581	0.0437506	5.846571	0.5851396
cg10575441	5.9637712	0.4430398	80.27849	0.17823
cg21126844	5.0267883	0.1075315	234.9878	0.4104072
cg06416929	0.2750428	0.1078212	0.701611	0.0068994
cg14125707	1.6927589	0.558066	5.134577	0.3525191
cg00833080	0.1706798	0.0259838	1.121144	0.0656367
cg16553338	2.83E+36	12993868	6.17E+65	0.0148835
cg13305186	0.2973324	0.0721205	1.225817	0.0932999
cg08312371	0.9804979	0.044961	21.38242	0.9900079
cg12518844	0.3693678	0.0746438	1.827783	0.2221829
cg03803210	4.9287418	0.437979	55.46498	0.1965284
cg19764066	0.2904117	0.0931664	0.905251	0.0330422
cg14058851	0.5041721	0.233242	1.08981	0.081633
cg15350840	2.4595251	0.8516555	7.102947	0.09627
cg23740473	1.9856851	0.9293629	4.242633	0.0765856
cg04184019	1.4760294	0.7891154	2.760893	0.2229732
cg12309360	1.7981753	0.8191523	3.947293	0.1435517
cg22506490	0.5923856	0.2974224	1.179873	0.1363711
cg17782974	2.0626945	0.9842656	4.322724	0.0551172
cg18160402	0.1241185	0.0288344	0.534271	0.0050839
cg22909042	2.53E-06	7.94E-12	0.805002	0.0462049
cg03762802	0.4921069	0.2440718	0.992205	0.0474974
cg20639805	2.1923608	0.8763053	5.484899	0.0933958
cg10616404	11210.077	0.0496969	2.53E+09	0.1381651
cg13944870	0.155353	0.0482158	0.500553	0.0018131
cg06654076	0.8929675	0.0128748	61.93411	0.9582589
cg04887172	1.8908394	0.6734237	5.309099	0.2265272
cg10088332	2.0055137	0.611341	6.579119	0.2509287
cg00074818	4.0642756	0.5413561	30.51289	0.1727823
cg06159394	1.5716579	0.4896425	5.044718	0.4473364
cg08397344	2.6532377	0.9642469	7.300693	0.0588295
cg02474109	0.1155389	0.0072701	1.83619	0.1261818
cg03790848	0.0022387	4.36E-12	1148256	0.5509658
cg09080173	0.038613	0.0068137	0.218818	0.0002361
cg01407332	0.0422301	0.006017	0.296391	0.0014567
cg19972822	3.5704319	0.818766	15.56975	0.090296
cg03342032	0.5580447	0.2122228	1.467391	0.236993
cg20615879	1.7568988	0.9138712	3.377603	0.0910492
cg10307632	18.619301	0.5218273	664.3546	0.1088598
cg13628185	0.0261582	1.51E-30	4.53E+26	0.9125451
cg11076814	0.411665	0.1951442	0.868425	0.0197864
cg14607876	0.2221453	0.0563134	0.876319	0.031673
cg23877497	0.553313	0.2547009	1.202019	0.1348821
cg14076239	0.3501977	0.1399398	0.876366	0.0249647
cg08787664	1.3350213	0.4310874	4.134386	0.6163711
cg24366425	73.671254	3.0627796	1772.068	0.0080543
cg01650776	0.2337216	0.087278	0.625883	0.0038238
cg16100244	1.7561263	0.7100803	4.343142	0.2228917
cg22895083	1.5740193	0.6171614	4.014406	0.3422974
cg00804628	0.3209088	0.0547524	1.880874	0.2077531
cg14205169	0.1946728	0.0191239	1.981677	0.1668936
cg01501551	1.4427231	0.5179366	4.018735	0.4831437
cg23606925	0.2286812	0.0480776	1.087724	0.0636984
cg18734842	0.8422451	0.2282653	3.107685	0.796608
cg24131595	0.1357406	0.0201	0.916691	0.0404403
cg07647771	1.7328551	0.3628221	8.276195	0.4907425
cg18832152	0.3278717	0.0935319	1.149339	0.0814254
cg17008412	0.3555193	0.1756311	0.719656	0.0040491

cg12253235	8.4867649	0.5773739	124.7462	0.1188943
cg11374169	1.4317633	0.2630335	7.793479	0.6780225
cg06020727	4.9984558	1.1716679	21.32393	0.0297045
cg05708512	2.8875555	0.7085122	11.76829	0.1390693
cg19119538	0.4613267	0.0359015	5.927956	0.5526046
cg13180315	1.268305	0.6534879	2.461557	0.4823577
cg24960118	1.5458365	0.4360301	5.480379	0.4999741
cg11402505	1.1773001	0.6265687	2.212104	0.612002
cg21317965	2.6827981	1.1854069	6.071675	0.0178792
cg24615749	0.1354169	0.0081632	2.246401	0.1629543
cg20984991	7.42E-09	1.98E-16	0.278669	0.0354176
cg18965086	0.2494535	0.0247482	2.514409	0.2388686
cg25570676	0.2773667	0.0644649	1.193398	0.0849813
cg27337540	0.29845	0.1159095	0.768465	0.01222
cg23727813	0.1765017	0.062369	0.499492	0.0010837
cg09842892	5.4489395	0.806652	36.80762	0.0819445
cg04164925	0.0893761	0.020375	0.392054	0.0013686
cg26523099	0.3919824	0.134933	1.138715	0.08521
cg01140585	0.5299503	0.1814814	1.547527	0.2455053
cg27233847	0.305918	0.130322	0.718113	0.0065177
cg05291508	1.3864382	0.6392471	3.006992	0.4081422
cg04790874	1.4046816	0.3839754	5.138689	0.6075943
cg22633036	0.9760977	0.4022291	2.368717	0.9573455
cg27236629	1.4885814	0.6736025	3.289588	0.3254453
cg13962355	3.05E-12	8.83E-30	1054513	0.1981386
cg01499815	1.3878238	0.6066872	3.174708	0.4375856
cg09611685	0.8895195	0.4434622	1.784244	0.7416628
cg27022853	0.5444257	0.2484665	1.192915	0.1287095
cg01008680	2.967052	0.2430057	36.22712	0.3942833
cg10306079	5144671.8	7.15E-33	3.70E+45	0.7349705
cg12215294	1.1888327	0.5548134	2.547385	0.6564276
cg02872476	0.7108762	0.3131893	1.613545	0.4145114
cg14737507	0.4105993	0.2180058	0.773336	0.0058563
cg03660010	0.4173129	0.2143844	0.812326	0.0101232
cg04321126	0.2658848	0.0859482	0.822527	0.0215026
cg14335435	18.415239	0.3529225	960.8937	0.1487988
cg12357606	0.4911739	0.226108	1.066976	0.0724659
cg08200420	3.3400897	0.5579503	19.99497	0.1865377
cg07305776	0.1525151	0.0177525	1.310287	0.0865858
cg16693872	0.3556066	0.0748223	1.690084	0.1935691
cg04685253	2.8793793	0.6317838	13.12288	0.1717563
cg20070536	3067.6117	4.391777	2142696	0.0162692
cg03300078	0.9335695	0.4528638	1.924534	0.8522595
cg05634428	2.0163275	0.8507879	4.778602	0.1111792
cg23260105	1.2245979	0.4136154	3.625687	0.7144713
cg07603511	2.3259919	0.8689569	6.226129	0.0928872
cg05616969	5.2406166	1.5189611	18.08082	0.0087531
cg01390419	2.8902738	0.4854323	17.20875	0.2436171
cg05550420	1.1008674	0.5176186	2.341316	0.8028992
cg05052463	0.6248619	0.2924043	1.335317	0.2248887
cg26868799	4.917E+13	6.24E-08	3.87E+34	0.199068
cg18992875	0.6475415	0.3211929	1.305477	0.2244421
cg19655177	1.6563256	0.5099311	5.379971	0.4011879
cg20239912	1.6190003	0.5769586	4.543068	0.3600711
cg11281396	0.602417	0.1761962	2.059671	0.4190886
cg03221837	5.095017	1.2548675	20.6868	0.0227552
cg16555755	7.1721904	0.116464	441.6844	0.3486659
cg26430059	0.0398935	0.0025849	0.615694	0.0210362

cg08130572	1.0686609	0.4770736	2.393836	0.8717915
cg04892724	2.4032092	1.2304202	4.693855	0.0102569
cg21987785	3.007506	1.0650258	8.492838	0.0376259
cg24060527	9.6320596	0.3739583	248.0933	0.1717677
cg25353142	1.7523249	0.6469355	4.746443	0.269878
cg17900697	3379.9568	0.1821944	62702852	0.1051433
cg11585952	0.3848474	0.124581	1.188845	0.0970416
cg19680631	1.2112429	0.3973541	3.692196	0.7361104
cg03409120	6590.0154	4.0454751	10735032	0.0197878
cg25047379	4.3287726	1.1290686	16.59622	0.0325975
cg19775367	5.0354023	1.2344106	20.54039	0.0242243
cg01127249	4.9645295	0.2182425	112.932	0.3148356
cg18545771	3.2683397	0.5863604	18.21754	0.1766967
cg00134210	0.7618781	0.360643	1.609509	0.4760133
cg05777400	14.07576	0.3443821	575.3117	0.162452
cg17045389	0.1214286	0.0221743	0.664956	0.0150869
cg01518113	1.3406734	0.4304044	4.176084	0.6130492
cg23280166	90428.764	0.002367	3.45E+12	0.200124
cg07261196	0.090679	0.0069189	1.188441	0.0674808
cg09492887	1.8901501	0.8937698	3.997301	0.0957
cg08151370	0.8113085	0.3591438	1.832752	0.6150207
cg18560571	0.5926231	0.294797	1.191336	0.141955
cg06931356	0.6342577	0.2935989	1.370178	0.2466348
cg01396587	0.1678498	0.0392245	0.718265	0.0161236
cg05140648	1.5336348	0.6561031	3.584857	0.3235733
cg13203804	0.0014992	3.77E-06	0.596327	0.0332348
cg15021629	0.0685225	0.0069964	0.671108	0.0213046
cg25780389	2.7327323	1.2087222	6.178281	0.0157168
cg14420490	0.363977	0.1311766	1.009931	0.05226
cg17851795	5.0518219	0.4481792	56.94353	0.1899973
cg09667226	0.1222492	0.0068474	2.182559	0.1529452
cg06611744	0.0430559	0.0020212	0.917168	0.0438662
cg08200625	1.452615	0.3509215	6.012999	0.606457
cg12577320	4.7462458	0.768517	29.3121	0.0936356
cg17589079	13.322781	1.0797788	164.3823	0.0434005
cg04330884	1.8536415	0.486819	7.058037	0.3656249
cg26710371	0.8000299	0.1101985	5.808134	0.8254156
cg26633899	0.4320366	0.2051652	0.909782	0.0271879
cg06782204	0.3134291	0.0959818	1.023505	0.0546704
cg19004565	0.1781556	0.0439216	0.722639	0.01575
cg06715885	0.4753268	0.0053337	42.35997	0.7454352
cg24553184	2.1244423	0.536673	8.409693	0.2830962
cg17622968	1.86E-22	1.09E-41	0.003179	0.0267944
cg06002638	0.1692533	0.0596994	0.479848	0.0008347
cg21940640	0.2695169	0.0723838	1.003531	0.0506174
cg12369353	3.3147135	1.1727251	9.369054	0.0237895
cg10858898	0.0481487	0.008532	0.271719	0.0005909
cg14988396	1874.5694	3.01E-12	1.17E+18	0.6645822
cg17432647	0.4303954	0.1616396	1.146008	0.0915619
cg19476053	2.8184541	0.9543402	8.323744	0.0607406
cg27550152	0.008443	1.53E-05	4.650689	0.1381657
cg22823644	2.2567865	1.1114364	4.582435	0.0243017
cg04320629	1.5168331	0.7999342	2.876215	0.2018892
cg02806927	5.962E+10	3.71E-20	9.57E+40	0.484434
cg01072942	0.2818154	0.087467	0.907999	0.0338679
cg13470919	172.74393	3.0616412	9746.558	0.0122877
cg08218061	0.0005717	3.22E-09	101.4582	0.2259587
cg22122329	13.084118	1.749347	97.86174	0.0122555

cg02390329	1.9066373	0.6839319	5.315245	0.2173114
cg13476336	3.624E+13	4.04E-11	3.25E+37	0.2672245
cg26970242	0.0244801	2.64E-05	22.72588	0.2872941
cg15296871	2.0567644	0.6928526	6.105598	0.1939467
cg01977209	2.9123005	0.7701012	11.01348	0.1152457
cg06358608	0.0762645	0.004725	1.230952	0.0697487
cg23173573	2.05E-15	4.70E-35	89071.9	0.1426549
cg08749305	2.0420397	1.0143405	4.110973	0.0455167
cg04830812	0.4031545	0.122522	1.326566	0.1349338
cg02569108	6.6901873	0.6829867	65.53365	0.1025786
cg07620968	16.052634	0.1281683	2010.537	0.2600156
cg14419102	1.0458258	0.4539078	2.409634	0.9162051
cg26241863	3.587748	0.4033466	31.91284	0.2519205
cg25734939	2.0285786	0.77159	5.333313	0.151515
cg00045515	2.499897	0.6421631	9.731929	0.1864125
cg27043582	4252.5043	6.04E-09	3E+15	0.5483183
cg04846961	4.3119033	1.1547149	16.10139	0.029708
cg11978890	4.6086426	1.8045056	11.77031	0.0014039
cg20704972	2.3968422	0.5392194	10.65402	0.2507645
cg00233943	3.3991983	0.8116279	14.23626	0.0940617
cg26733444	4.3407714	1.0315916	18.26527	0.0452431
cg23530239	2.772971	1.346791	5.7094	0.0056409
cg11582403	2.1642522	0.6812106	6.875976	0.1905083
cg07895203	0.3257971	0.1604115	0.661697	0.0019204
cg08578703	2.7916822	1.0492999	7.427323	0.0397484
cg18486906	0.1536617	0.0371853	0.634979	0.0096721
cg23715673	0.0116522	0.0001517	0.894878	0.0444197
cg07135732	0.3179203	0.0934573	1.081491	0.0665726
cg16869622	0.7759297	0.0201781	29.83759	0.8916257
cg17750252	4.0954818	0.6671972	25.13945	0.1277922
cg07120972	0.4104067	0.1792365	0.939729	0.0351146
cg10852875	0.2447369	0.0661047	0.90608	0.0350616
cg21332500	1.4737017	0.3159117	6.874694	0.6216569
cg02770313	1.4799908	0.4840361	4.525226	0.4917652
cg01355932	0.4143466	0.1409874	1.217719	0.1091919
cg16327891	1.2453276	0.6721968	2.307124	0.4855579
cg06334495	0.6318549	0.2406935	1.65871	0.3511736
cg17516660	0.7076758	0.3841914	1.30353	0.267241
cg09067459	1.8080919	0.829465	3.941331	0.136307
cg08124446	1.0550653	0.5334654	2.086663	0.8775671
cg18866529	1.6380298	0.731658	3.667207	0.2300876
cg15462411	0.0205495	0.0003074	1.373755	0.0700071
cg19274341	1.0924566	0.5701041	2.09341	0.78986
cg09304624	0.4645395	0.2256029	0.956534	0.0374748
cg03363565	0.4229962	0.189133	0.946031	0.0361659
cg17677987	0.2488129	0.0246627	2.510185	0.2381806
cg01006983	7.3349048	1.0286897	52.30035	0.0467911
cg15017982	0.0115244	0.0008186	0.162247	0.0009404
cg08351331	1.9178303	0.4761482	7.72464	0.3596188
cg05321594	1.8128857	0.7867383	4.177443	0.1624732
cg08012219	0.1900783	0.0634853	0.569105	0.003003
cg21223341	2.2517063	0.2213565	22.90504	0.4928254
cg21164697	0.8802919	0.0732881	10.57353	0.9199249
cg15660684	3.3462178	0.7190864	15.57139	0.1236565
cg23314805	0.0059698	1.15E-05	3.095307	0.108342
cg11201894	1.4760617	0.4577028	4.760203	0.5145495
cg25059899	0.6761564	0.3642088	1.25529	0.2150892
cg25577866	2.25E-25	3.13E-49	0.161793	0.0428723

cg23679992	2.8182485	0.6581863	12.06729	0.1626253
cg25575138	0.1858921	0.0668093	0.517231	0.0012701
cg04879696	3.25E-30	3.70E-51	2.86E-09	0.0057904
cg18147048	0.3499385	0.1688658	0.725173	0.0047379
cg06651508	1.6036535	0.4389047	5.859369	0.4749935
cg19269039	1.5059705	0.6403683	3.541629	0.3480324
cg20762310	0.1514723	1.07E-20	2.15E+18	0.9331505
cg06062132	9761146.8	1.73E-06	5.49E+19	0.2826365
cg01627669	1.2935254	0.5453828	3.067952	0.559163
cg04523291	3.9704525	1.5486534	10.17948	0.0040983
cg09839170	1.4777594	0.7354594	2.969264	0.2726749
cg11427039	0.3850098	0.002253	65.79201	0.7159393
cg22588640	1.6255037	0.7492576	3.526507	0.2189088
cg01432552	0.5041621	0.245774	1.0342	0.06173
cg12940558	5.858E+09	4.95E-07	6.93E+25	0.2336258
cg10818566	2.0287941	0.3066708	13.42157	0.4630377
cg11846559	4.2511144	0.868316	20.81267	0.0741426
cg19368203	0.9461692	8.48E-05	10552.53	0.9907151
cg22903457	0.3851464	0.1748996	0.848131	0.0178394
cg26481302	8.6595642	0.2734107	274.2689	0.220795
cg00430080	2.9394818	1.1294331	7.650346	0.0271491
cg12297329	0.8035851	0.4071532	1.58601	0.5284476
cg06520450	0.8910473	0.4879738	1.627065	0.7072947
cg13110334	1.3618959	0.5450572	3.402873	0.5085538
cg27361011	1.4335895	0.6304261	3.259984	0.39018
cg03084838	0.1719674	0.062995	0.469447	0.0005908
cg11498254	5.0756579	0.9776567	26.35107	0.053227
cg18189288	6.2286055	0.7139819	54.33684	0.0979001
cg08290076	1.0939052	0.6348039	1.885036	0.7465001
cg12118798	3.1624619	1.4038082	7.12431	0.0054608
cg14167389	0.1911106	0.0340502	1.072628	0.0600669
cg01795955	0.5707763	0.2718276	1.1985	0.138457
cg13169065	4.3171675	0.7397677	25.19431	0.104149
cg24937306	0.3914018	0.0260869	5.872509	0.4972433
cg22001782	4.49E-05	1.89E-10	10.67419	0.1129569
cg16261730	1.1956147	0.6039124	2.367056	0.6081603
cg25679083	1.5611684	0.7124487	3.420943	0.2657592
cg23417096	0.259765	0.0825436	0.817481	0.0211949
cg24580153	57.599476	0.0181908	182383	0.3243015
cg19716930	0.2594836	0.1007784	0.668117	0.0051783
cg13514218	0.095073	0.0213514	0.423338	0.0020151
cg05101821	0.3077175	0.1185895	0.798469	0.0154109
cg09619624	2.6622858	0.8598583	8.242946	0.0894854
cg25927215	0.4609969	0.1888967	1.12505	0.0889202
cg21819722	0.4693481	0.1145644	1.922828	0.2931253
cg17160094	1.423029	0.2653862	7.630432	0.6805331
cg05343105	0.4660901	0.1443323	1.505138	0.2018394
cg18538270	26.879306	0.1797444	4019.58	0.1976638
cg12883767	3.4944781	0.509653	23.96018	0.2027446
cg01286935	0.9201718	0.0603487	14.0304	0.9522744
cg20266910	1.0964927	0.585943	2.0519	0.7732609
cg19219068	1.0095714	0.2179333	4.676817	0.9902834
cg15991405	1.0353668	0.5057994	2.119387	0.9242429
cg03104936	1.9783072	0.8515856	4.595779	0.1126493
cg07800034	0.1657986	0.0356197	0.771741	0.0220106
cg20150640	0.3166869	0.130758	0.766994	0.0108421
cg19691177	0.4278023	0.1310506	1.396521	0.1595269
cg05310260	1.7729399	0.7402963	4.246024	0.1987502

cg04707332	4.6400372	1.6437447	13.09811	0.0037484
cg02386311	1.29824	0.3449259	4.886345	0.6995239
cg05274944	0.5789345	0.2745323	1.220859	0.1510707
cg04765483	0.4431765	0.1787773	1.098604	0.0789288
cg13513157	2.0766902	0.7792956	5.534026	0.1439291
cg12438037	0.2216002	0.077347	0.634887	0.0050174
cg03819692	0.5937527	0.2425424	1.453528	0.2537805
cg04300159	0.5443644	0.2473187	1.198181	0.1308415
cg12931620	2.1078708	0.9126964	4.868124	0.080801
cg22419414	0.3916812	0.1396406	1.098636	0.0748798
cg15279616	2.5291338	0.710693	9.000395	0.1519553
cg02197228	0.2144794	0.0568962	0.808515	0.0229713
cg01877450	0.1246635	0.033581	0.462791	0.0018629
cg25189873	11.766319	0.6542766	211.602	0.0944836
cg05236474	1.2631809	2.25E-28	7.10E+27	0.994282
cg08556541	0.4341314	0.2047084	0.920676	0.0295972
cg03264133	4.0054352	1.283718	12.49769	0.0168408
cg11397854	0.0580141	0.0029242	1.150962	0.0617999
cg07974485	0.3408819	0.1385141	0.838907	0.0191676
cg01111041	0.1205541	0.0139721	1.040164	0.0543358
cg26721908	0.0479627	0.0090789	0.253381	0.0003482
cg23462129	3.8929533	0.9615143	15.76168	0.0567855
cg02355558	0.3557536	0.127589	0.99194	0.0482192
cg23027329	0.4430992	0.2291728	0.85672	0.0155343
cg06142537	0.3642135	0.0983064	1.349368	0.1306503
cg01677601	3.37E-08	3.56E-15	0.318202	0.0357557
cg21609684	1.9066438	0.7494656	4.85051	0.175542
cg08425678	1.8907252	0.7101696	5.033786	0.2023366
cg20707323	0.1521328	0.0330168	0.700988	0.0157033
cg09352844	4.3062392	0.7340726	25.26139	0.1057736
cg12386061	0.9742718	0.4606945	2.06038	0.9456183
cg14555824	0.0596943	0.0042716	0.834213	0.0361996
cg00659559	0.0769509	0.0072342	0.81854	0.0335075
cg26734350	0.1124311	0.0281231	0.44948	0.0019949
cg18440818	9.5998602	0.9261208	99.50896	0.058008
cg19556284	0.9222576	0.418559	2.032113	0.840867
cg10783469	0.1070316	0.0311996	0.367177	0.0003809
cg14225031	1.2987478	0.4877133	3.458479	0.6009069
cg07637741	0.2681656	0.1191993	0.603299	0.001465
cg24709745	3.4074109	9.67E-05	120077.5	0.8184824
cg20512711	2.3136758	0.9737741	5.497267	0.0574626
cg00729269	1484.0844	0.0067794	3.25E+08	0.2444338
cg24710886	0.003282	5.14E-05	0.209544	0.0069987
cg05934592	0.1734821	0.0309454	0.972554	0.0464144
cg24360241	1.2882328	0.4127004	4.021182	0.6627718
cg15630950	2.3158332	0.5365061	9.996314	0.2603963
cg16930811	1.5776324	0.3446341	7.221933	0.5569149
cg10785394	2.1330535	0.7463118	6.096537	0.1574054
cg20509092	1.2442133	0.579024	2.67358	0.5755627
cg11251554	0.1999673	0.0613279	0.652019	0.0076036
cg17884843	2.0538724	0.8867542	4.757115	0.093054
cg04685228	1.2861498	0.6573707	2.51636	0.4624046
cg06960356	2.0457329	0.8563197	4.88722	0.1072076
cg21837069	1.1153742	0.5521043	2.253306	0.7608754
cg24496475	0.2251611	0.0840184	0.60341	0.0030333
cg00673290	2.5119152	1.1393874	5.537816	0.0224021
cg23990814	4.1732312	0.1867607	93.25229	0.3673968
cg02558288	25.530955	1.1237229	580.0626	0.0420359

cg05566582	0.3415348	0.0614057	1.899597	0.2197935
cg15006881	1.8346154	0.9520992	3.53515	0.0697872
cg17793286	0.3554195	0.133659	0.945115	0.0381639
cg04484550	0.6455139	0.3030639	1.374918	0.2565326
cg02090014	0.3324823	0.0645309	1.713048	0.1880218
cg13835073	258.83592	0.1035784	646814.7	0.1639433
cg12720459	4.17E-12	2.58E-18	6.73E-06	0.0003272
cg15195412	0.1581826	0.0359224	0.69655	0.0147655
cg19036773	0.4581743	0.1709424	1.228037	0.1207567
cg24949747	5.3189625	0.089836	314.9222	0.4221781
cg14775296	2.286209	0.8256283	6.330635	0.1115561
cg13270625	4.7531636	0.6540641	34.54182	0.1234575
cg16576935	0.7012761	0.1600394	3.07292	0.637831
cg06495233	0.8422046	0.3187571	2.225232	0.7290196
cg11337261	1.13E-39	1.55E-76	0.008258	0.0383848
cg02483058	2.2403153	0.6391662	7.852438	0.2074859
cg18924222	0.9299623	0.4421086	1.956148	0.8482199
cg02482603	2.1108976	0.4665139	9.551459	0.3320393
cg11283427	1.2648013	0.6087254	2.627987	0.5289594
cg09513276	2.2560005	1.0061097	5.058631	0.0482967
cg24760467	0.5709478	0.2524839	1.291098	0.1782205
cg06303744	1.9134879	0.9067408	4.038018	0.0885606
cg04992974	3.0823207	1.4432309	6.582939	0.0036419
cg09255124	1222.739	8.1553881	183325.5	0.0054198
cg24678095	4.5909103	1.1061641	19.05364	0.0358241
cg05216730	2.0840025	0.7956955	5.458202	0.1349814
cg16585380	1.2772032	0.4857619	3.358123	0.6198494
cg14392772	2.2731874	0.8569214	6.030169	0.0989928
cg20348765	1.0014213	0.4784031	2.096234	0.9969934
cg15236881	0.683545	0.3314431	1.409695	0.302918
cg11540692	2.5178285	1.0754563	5.894671	0.0333724
cg07368443	0.1831075	0.0454135	0.73829	0.0170101
cg03054370	7.4254759	9.17E-29	6.01E+29	0.9529248
cg08877463	5.3852321	1.8687473	15.51881	0.0018217
cg01857186	0.5780439	0.2545477	1.31266	0.1902554
cg17389949	0.5766965	0.2328343	1.428393	0.234253
cg07913887	0.3087374	0.0877866	1.0858	0.0670005
cg25368560	2.2909212	0.9190565	5.710552	0.0752668
cg25456221	0.2894503	0.0459978	1.821426	0.1864883
cg05280750	0.1789607	0.0586544	0.546028	0.0025019
cg22526555	0.736702	0.2547294	2.130613	0.5727847
cg07109046	1.3647457	0.2764605	6.73706	0.702664
cg07564233	1.5153348	0.4010493	5.725579	0.5399922
cg06042849	0.6936747	0.2985004	1.612007	0.39525
cg00945108	1.2814442	0.1679005	9.780195	0.8109873
cg01248010	0.0004939	7.94E-07	0.307053	0.0203553
cg06448055	1.2923788	0.6531638	2.557158	0.4613336
cg14038617	0.7406908	0.3499798	1.567584	0.4326055
cg22821013	3.1960261	1.105867	9.236719	0.0318884
cg09981841	0.2553852	0.0890288	0.73259	0.0111265
cg02052915	3.9123992	1.0702574	14.30204	0.0391479
cg24346905	2.45431	1.087826	5.537317	0.0305611
cg09877744	4.803634	1.3517716	17.07012	0.0152712
cg25759064	0.292695	0.1130719	0.757663	0.0113463
cg25363289	0.3416101	0.1031338	1.131515	0.0787884
cg06891538	2.7069249	0.9648014	7.594767	0.058506
cg21760363	0.6586349	0.2902918	1.494358	0.3178006
cg25694218	4111704.9	1.21E-11	1.40E+24	0.4596856

cg26808436	5.352128	1.5465602	18.52193	0.0080885
cg21933664	6.7878626	1.5287123	30.1398	0.0118026
cg21227325	0.720309	0.354676	1.462871	0.3640877
cg05262335	0.5938089	0.277993	1.268409	0.1783167
cg23762517	0.2308113	0.0796492	0.668856	0.0069164
cg05902884	0.2228465	0.0897045	0.553601	0.0012225
cg04152021	0.2471372	0.1029791	0.593099	0.0017508
cg02525785	69950.408	0.0003357	1.46E+13	0.2536787
cg27531571	0.4120316	0.1971427	0.861153	0.0184032
cg18157012	1.4240463	0.6060593	3.346055	0.4173448
cg21279806	1.4635873	0.6981019	3.068446	0.3132414
cg03545972	0.356485	0.1442952	0.880705	0.0254008
cg24312985	0.1666757	0.0439516	0.632076	0.0084263
cg05877788	0.1482644	0.0432828	0.507877	0.0023778
cg05951558	1.0162125	0.4802858	2.150153	0.9664519
cg25499067	2.6177125	1.2253472	5.592226	0.0129662
cg03016934	0.5841821	0.300072	1.137289	0.1137692
cg04547425	531536.85	0.3220657	8.77E+11	0.0710976
cg02420027	0.4493345	0.1743197	1.158225	0.0977389
cg19015711	6.5321786	0.2418771	176.4092	0.2644307
cg01626707	0.413096	0.1280622	1.332542	0.1390023
cg04173397	1.6892975	0.6959261	4.100617	0.2465461
cg12114049	1346.6466	0.0042571	4.26E+08	0.2648064
cg25483003	2.0179818	0.9286478	4.38514	0.0762246
cg21498475	0.2330113	0.0824389	0.6586	0.0060001
cg20687616	0.8756014	0.4462412	1.718079	0.699292
cg14241879	0.7426048	0.1381308	3.992318	0.7287579
cg26662674	1.2435103	0.4303194	3.593418	0.687294
cg01908020	0.533293	0.1838595	1.546841	0.24723
cg23867673	0.9688628	0.4184644	2.24319	0.9411309
cg24688826	0.9045458	0.3789985	2.158855	0.8211739
cg18597220	0.5655699	0.2727069	1.172942	0.1256819
cg14007559	4.0179858	0.1632731	98.87859	0.3947638
cg22429852	0.5109581	0.2587339	1.00906	0.0531149
cg04065405	3.4271414	0.6890227	17.04631	0.132355
cg14439622	2.1068458	0.5820453	7.62621	0.2562169
cg25256099	0.4609555	0.2242601	0.947471	0.0351394
cg01113811	0.3839196	0.116088	1.269677	0.1167142
cg21376090	0.5810556	0.2552028	1.32297	0.19592
cg17748645	0.6580436	0.3192051	1.356561	0.2568897
cg25930451	1.55E-08	2.79E-24	85476427	0.330825
cg02952078	0.0220292	0.0013956	0.347715	0.0067203
cg15033653	0.0725165	0.0147259	0.357102	0.0012556
cg00200734	2.3215828	1.1834687	4.554195	0.0142874
cg07129392	1.5804676	0.654072	3.818964	0.3092294
cg02707264	1.5871376	0.776599	3.243638	0.2052727
cg13165422	2.60E-07	7.82E-20	863173.1	0.3026323
cg04761768	0.4716729	0.1715701	1.296702	0.1452807
cg00613113	0.2386677	0.088058	0.646872	0.0048589
cg02532014	11943.572	14.345373	9943897	0.0062142
cg07820548	1.3324314	0.4087848	4.343051	0.6340189
cg06927305	0.1839194	0.0580958	0.582251	0.003979
cg25430838	0.5462254	0.220892	1.350715	0.1904884
cg01406456	1.4638957	0.3796939	5.643996	0.5799204
cg24923509	1.5057432	0.813177	2.788154	0.1928977
cg17381847	1.07E-05	9.04E-18	12780086	0.4199702
cg21582611	2.1834104	0.7616347	6.259275	0.1461586
cg08057786	0.2914372	0.1330402	0.638421	0.002059

cg21507807	2.7420124	0.4044509	18.58973	0.3016225
cg14562851	4.3099577	0.5306692	35.00436	0.1716068
cg12785983	0.0093093	6.71E-07	129.1733	0.3365352
cg14323928	0.3633478	0.1587612	0.831574	0.0165496
cg08669954	0.4916613	0.2180896	1.108402	0.0869311
cg05571310	1.2463354	0.4118998	3.771189	0.6966717
cg19655195	0.1439377	0.0099771	2.076561	0.1546241
cg02717339	0.2833352	0.1167611	0.687548	0.0052999
cg15621178	2.0503849	0.3618209	11.61923	0.4171934
cg15153700	2.1358129	0.8587267	5.312163	0.102607
cg23628563	2.0546591	0.5251181	8.03938	0.3008747
cg09323975	1.0551076	0.56582	1.967502	0.8660109
cg09889848	0.795409	0.3048827	2.075144	0.639893
cg06134790	0.2952747	0.0657213	1.32662	0.1115482
cg00777079	0.2876614	0.1275137	0.648943	0.0026848
cg00362569	2.5060375	0.571683	10.9855	0.2230763
cg04340918	1.3492071	0.5998425	3.03473	0.4689412
cg08787401	1.2135439	0.5192055	2.836427	0.655013
cg12037538	10827.379	29.359383	3993004	0.002065
cg01557738	2.9255072	1.2753518	6.71077	0.0112724
cg08428878	1.0858417	0.4552416	2.589948	0.8526908
cg13943217	1.0525146	0.502801	2.203232	0.8919858
cg01052805	5.62E-06	1.16E-17	2733584	0.3786112
cg07891531	1.2672035	0.6841474	2.347162	0.4514509
cg18964630	1.2278491	0.5690014	2.649578	0.6009276
cg00667862	2.1278173	0.8393873	5.393942	0.1115984
cg17953385	3.2806051	1.3074535	8.231551	0.01137
cg00830817	1.9895616	1.1043429	3.584353	0.021997
cg05943554	2544.3332	1.2340484	5245849	0.0440121
cg24477899	6.02E-23	1.49E-59	2.43E+14	0.234147
cg00616243	2.1286207	0.995092	4.553374	0.0515009
cg13019143	4.0013665	1.3252857	12.08112	0.0139134
cg12141659	1.3684428	0.4977655	3.762085	0.5432409
cg09491542	5.3635081	0.4545588	63.28602	0.1822546
cg21706038	0.0941288	0.0072257	1.226207	0.0711901
cg18725544	2.1157907	0.809285	5.531512	0.1264105
cg13479148	12.866991	0.7053979	234.7036	0.0846367
cg13348574	1.145849	0.5106834	2.571006	0.7412587
cg12872311	0.1289488	0.0250589	0.663547	0.0142585
cg19592472	3.808379	0.8287229	17.50133	0.0857015
cg26305504	5.2549552	0.5661999	48.77174	0.1444053
cg20730595	6.0964495	0.2349001	158.2234	0.2765702
cg02663821	0.3350109	0.1170953	0.95847	0.0414447
cg09661370	1.8241876	0.4732532	7.031459	0.3825424
cg24343524	0.0231944	0.0003789	1.419781	0.0729739
cg12705762	3.0037666	1.2419784	7.264711	0.0146511
cg02878289	0.2949274	0.1264161	0.688063	0.0047286
cg03506799	4.31E-10	7.87E-21	23.63059	0.0873978
cg19938436	0.4502138	0.194603	1.041569	0.0622105
cg23490555	1.3298238	0.4410589	4.009513	0.6126993
cg27616811	49.499718	0.6140812	3990.062	0.081467
cg11623855	1.5018791	0.3901407	5.781609	0.5542702
cg13717394	1.7722318	0.6760082	4.646106	0.2445427
cg08196138	0.1823358	0.0410979	0.808955	0.0251645
cg13458659	7.8017372	0.0321612	1892.564	0.4634149
cg22788657	2.4514056	0.9329725	6.441122	0.0688805
cg26877861	0.3609765	0.1512841	0.86132	0.0216518
cg17067226	6.5570956	1.1525335	37.30521	0.0340058

cg04071948	0.3144472	0.1518513	0.651144	0.0018385
cg20092334	0.3296189	0.0886498	1.225593	0.0976489
cg19816734	2.48E-13	9.02E-24	0.006811	0.0179431
cg25006073	0.1330918	0.0315251	0.561883	0.0060614
cg10502136	4.2993462	1.0690297	17.2908	0.0399785
cg22920586	33.430997	0.8613262	1297.571	0.0601087
cg13858742	0.879713	0.352797	2.193598	0.7833836
cg02283366	2.0962905	0.9143323	4.806167	0.0803939
cg02420024	2.1499497	1.0790349	4.28372	0.0295381
cg06399569	0.6774473	0.3192512	1.437535	0.3103482
cg16951074	0.6734491	0.36375	1.246828	0.2083926
cg22436429	4.3079397	1.2147551	15.27744	0.023749
cg26131670	1.6737863	0.3896833	7.189328	0.4885246
cg22156700	2.0030581	0.8893661	4.51135	0.0935555
cg06237216	0.0364044	0.0021324	0.621492	0.0221079
cg05904135	0.2938127	0.0624454	1.382422	0.1211133
cg01108370	1.9559547	0.7041855	5.432885	0.1980572
cg20899354	1.3188698	0.684737	2.540271	0.4079134
cg20427486	1.0979528	0.5596091	2.154183	0.78581
cg11552868	0.3787582	0.146426	0.979729	0.0452633
cg05904366	1.3954159	0.6134719	3.174042	0.4268233
cg24312792	0.1968472	0.04627	0.83745	0.0278006
cg10003267	2.47E-09	1.08E-16	0.056828	0.0219301
cg00421815	0.5979133	0.2111412	1.693181	0.3328433
cg13634319	1.8153806	0.747639	4.408018	0.1877001
cg02096633	6.13E-07	1.17E-13	3.212983	0.0699687
cg07299645	0.6812627	0.3478051	1.334422	0.2631792
cg13857210	3.3941316	1.0609193	10.85863	0.0394338
cg00682386	0.2225875	0.0579059	0.855615	0.0287468
cg14141399	0.2323801	0.0836303	0.645705	0.0051285
cg10664429	0.0764866	0.0048441	1.207706	0.067863
cg13766043	5.5200147	0.1349031	225.87	0.3669825
cg15467085	0.3898039	0.1807951	0.840438	0.0162421
cg15139182	3.5795044	1.2921746	9.915728	0.0141656
cg06572465	1.0830446	0.550172	2.132034	0.8174272
cg15733729	2.1731139	0.9153334	5.159239	0.0785054
cg15686334	2.0608394	0.9808387	4.330028	0.0562762
cg18932278	0.391586	0.1758161	0.872159	0.0217471
cg17282904	0.127847	0.0306728	0.532879	0.0047393
cg19830849	1539.6419	0.0038559	6.15E+08	0.2647138
cg22822867	2.9496093	0.8661414	10.04477	0.083611
cg04546950	1.8448656	0.9452554	3.600645	0.0726617
cg23428192	0.4585048	0.161768	1.299556	0.1423714
cg08653574	27.544723	1.3141455	577.3423	0.0326841
cg03925294	2.7876389	0.9889518	7.857745	0.0525072
cg09101894	0.297647	0.1242886	0.712807	0.0065328
cg06718369	0.3619946	0.1520452	0.86185	0.0216824
cg01768201	1.5820112	0.7486706	3.342938	0.2294933
cg24085707	4.9325999	1.1730089	20.74199	0.0294272
cg04901044	1.8041263	0.4879488	6.670519	0.3764521
cg23397578	0.1146612	0.0094509	1.391111	0.0889918
cg16573755	1.6257974	0.65815	4.016132	0.2921943
cg02888166	1.6758229	0.5025684	5.58806	0.4007676
cg17327404	4.1654785	0.139254	124.6012	0.4105502
cg25956483	0.3004678	0.0943759	0.956609	0.0418466
cg00426498	0.0154822	0.0002155	1.112109	0.0559738
cg01573859	0.6234957	0.1114414	3.488353	0.5907528
cg08324115	0.1141364	0.0274261	0.474989	0.0028519

cg25067686	0.4480015	0.1577841	1.272025	0.1315378
cg15573861	0.0229366	1.66E-06	316.6676	0.4376625
cg14148981	0.6753684	0.2980803	1.5302	0.3469304
cg24345184	1.7711454	0.6896378	4.548701	0.2349054
cg27618145	0.2271661	0.1008615	0.511637	0.0003467
cg06618490	0.5204964	0.2257475	1.200086	0.125516
cg12831729	5285306.3	7.91E-09	3.53E+21	0.3740956
cg13303464	0.397488	0.162941	0.969656	0.0425922
cg20928986	0.3976138	0.1283615	1.231653	0.1098708
cg22399984	2.6899835	0.8918895	8.113125	0.0789462
cg16260700	0.3471506	0.1350234	0.892538	0.0280973
cg13521908	7.57E-05	2.15E-09	2.672008	0.0757323
cg01052502	5.118E+14	4.54E-35	5.77E+63	0.5567147
cg14211598	1.2901988	0.4780536	3.482064	0.6149652
cg17373759	42.107038	0.3023952	5863.197	0.1375236
cg14596967	0.5191339	0.2288793	1.177477	0.1166536
cg14689355	1.78E-14	1.88E-26	0.016877	0.0244442
cg19211851	0.3745312	0.1794917	0.781505	0.0088735
cg09094473	4.5345886	0.6408201	32.08778	0.1299689
cg06455470	2.0380561	0.7549005	5.502278	0.1599945
cg01804679	0.0657345	0.010511	0.411096	0.0036101
cg07742017	1.3554586	0.4711346	3.899667	0.5726927
cg03483464	0.0039781	8.85E-05	0.178765	0.0044169
cg20716058	2.5306742	0.9876783	6.484208	0.0530963
cg14395744	3.4830009	0.5900729	20.55898	0.1683213
cg09291824	1.2240234	0.4873892	3.073997	0.6670106
cg21792144	411716.98	1.94E-07	8.75E+17	0.3720297
cg16697433	2.4777303	1.1012288	5.574815	0.0283056
cg26587228	0.302487	0.0798433	1.145975	0.0784982
cg01181415	2.2072494	1.0459737	4.657813	0.0377158
cg08644498	0.0255621	0.0019078	0.342496	0.0056194
cg16575530	0.4272898	0.2041285	0.89442	0.02407
cg03298704	0.3913147	0.1815512	0.843438	0.0166426
cg11700800	2.3319394	0.9926562	5.478172	0.0520103
cg14604700	0.6069443	0.197169	1.868353	0.3840871
cg19117545	5.48457	0.1463322	205.5631	0.357309
cg01407748	0.2181364	0.0099966	4.759969	0.3330305
cg23119026	0.9353362	0.306315	2.856059	0.9065647
cg26307728	0.0841489	0.0194606	0.363866	0.0009222
cg23195199	0.797388	0.2518472	2.524657	0.7002096
cg06082141	0.3113401	0.0085805	11.29682	0.5242507
cg21495956	0.873579	0.4013318	1.90152	0.733424
cg16698281	0.2594907	0.0975757	0.690084	0.0068658
cg26035118	1.7739909	0.5622997	5.596738	0.3281439
cg03180404	0.633004	0.3247599	1.233816	0.1792992
cg10995511	2.3212549	0.5030593	10.71091	0.2804295
cg12105382	0.3635318	0.1291012	1.023657	0.0554035
cg10596537	3.9135998	0.9268089	16.52581	0.063376
cg19216285	0.0044546	1.25E-05	1.588731	0.0709855
cg08210322	1.1521196	0.5747408	2.309527	0.6898327
cg16290301	1.76E-15	5.99E-28	0.005188	0.0203872
cg18081760	1.9759408	0.643394	6.068353	0.2341893
cg07147475	1.5098521	0.6230218	3.659027	0.3616266
cg17553677	0.3916867	0.1732093	0.88574	0.0243598
cg10017105	0.5207743	0.1281403	2.116477	0.3617847
cg12181459	1.4004806	0.4766034	4.115258	0.5402432
cg08106706	1.2368116	0.6396513	2.391464	0.5275422
cg17790273	0.4595733	0.2060956	1.024804	0.0574222

cg25782847	1.773936	0.8426909	3.734286	0.1312233
cg23746050	2.5614844	0.6199961	10.58265	0.1937704
cg06278461	1.2588926	0.052416	30.23525	0.8871145
cg03127898	0.4840112	0.2415459	0.969865	0.0407319
cg16312514	1.2687564	0.5507437	2.922853	0.5761241
cg03277051	0.2166707	0.0426421	1.100935	0.06518
cg11800620	3.2167677	1.4763903	7.008712	0.0032771
cg25861327	7.032E+09	3.15E-19	1.57E+38	0.4960023
cg22632523	2.0368557	0.8269279	5.017101	0.1219175
cg10573932	0.0956778	0.0157835	0.579987	0.0106965
cg14494421	1.7233176	0.8185559	3.628125	0.1518982
cg21826978	6.0728188	1.0383772	35.51612	0.045311
cg11946503	1.6926198	0.7301754	3.923663	0.2198731
cg09225840	0.2886588	0.1207327	0.690152	0.005209
cg12746652	2.2686766	0.4567207	11.26924	0.3164912
cg19730379	0.3187892	0.1558568	0.65205	0.0017408
cg20569804	0.551536	0.2253411	1.349917	0.1925867
cg22339338	1.8312068	0.7006271	4.786167	0.2171415
cg13145504	2.0714049	0.7903372	5.428972	0.1385176
cg23170439	1.6536371	0.7866825	3.476009	0.1845182
cg02926266	1.9948677	0.7583392	5.247648	0.1616916
cg26969937	0.3603653	0.1477626	0.878864	0.0248425
cg25011252	2.7651118	0.2049429	37.3072	0.4436238
cg07567724	2.2759851	0.5834262	8.878772	0.2363609
cg21852842	0.0389259	0.0001994	7.600296	0.2277119
cg09642378	0.5598558	0.261132	1.200307	0.1360262
cg18754695	1.4438323	0.3177612	6.560435	0.6343809
cg15122603	0.781053	0.0302139	20.19085	0.8816186
cg04322596	0.4301629	0.2175103	0.850719	0.0153233
cg21545013	0.230613	0.090586	0.587092	0.0020908
cg14374799	4.6373044	0.4597333	46.77623	0.1932703
cg05889234	2.1949162	0.9840301	4.895843	0.054778
cg08845333	0.0044782	0.0001604	0.125019	0.0014522
cg20128773	4.1579781	0.548725	31.50719	0.1678532
cg18049182	3.4541959	0.9638172	12.37939	0.0569915
cg09088576	2.7687569	0.8864855	8.647648	0.0796696
cg16480209	3.8142657	1.2318705	11.81019	0.0202548
cg05057352	0.3650555	0.1357275	0.981861	0.0459093
cg01257697	0.265705	0.1014459	0.695929	0.0069785
cg13421594	3.08E-06	2.63E-09	0.003597	0.0004293
cg16681130	0.8793403	0.0862591	8.964153	0.9135644
cg26863534	0.5125134	0.2460011	1.067759	0.0742785
cg02832357	0.130932	0.0281581	0.60882	0.0095193
cg26571739	1.4907284	0.2983992	7.447309	0.6266274
cg05279291	0.377142	0.151348	0.939795	0.0363263
cg25489732	0.3800624	0.1278061	1.130207	0.0818881
cg00142239	0.5653193	0.295191	1.082641	0.0853505
cg17839314	1.847E+15	9.8269988	3.47E+29	0.0360614
cg25805709	0.0533189	0.0054062	0.525859	0.0120608
cg08908089	1.8360657	0.9198138	3.665021	0.0848964
cg02853154	0.4072005	0.1992778	0.832066	0.0137321
cg00423030	0.4520144	0.1368099	1.493437	0.1928465
cg24343361	1.880684	0.6344399	5.574953	0.2545917
cg16379311	3.18E-08	1.70E-16	5.957992	0.0756798
cg20052079	0.1289066	0.0380685	0.4365	0.0009946
cg05418719	1.2702689	0.5389528	2.993923	0.5844542
cg15854179	1.86E-10	5.81E-54	5.96E+33	0.6611255
cg25653759	3.0454298	0.3938148	23.55077	0.2859427

cg16403932	0.3985764	0.1978758	0.802843	0.0100358
cg22289810	0.0004008	4.57E-07	0.351856	0.0236954
cg18862005	0.2684788	0.0621955	1.158941	0.0780216
cg06477423	6.11E+25	137.36181	2.72E+49	0.0325857
cg13712476	66.351323	0.7439006	5918.127	0.0671238
cg05346286	0.5849027	0.1886044	1.813909	0.3530213
cg09039751	3.3954448	0.6243339	18.46615	0.1571345
cg25787377	3.479739	0.7190619	16.83942	0.1211407
cg11930908	1.4404557	0.4873279	4.257734	0.5092454
cg05793240	0.1626974	0.0655226	0.403989	9.11E-05
cg11977716	1.8487366	0.7512693	4.549403	0.1810622
cg09174233	0.0014116	8.42E-06	0.236514	0.012014
cg08325813	1.4438991	0.6304627	3.306849	0.3849186
cg02088785	1.6043267	0.733078	3.511037	0.236835
cg01866427	0.0042102	1.76E-05	1.005995	0.0502506
cg07387309	0.1641074	0.0025754	10.45712	0.3938848
cg14662522	1.5503687	0.8093993	2.969663	0.1860716
cg14861920	0.583494	0.2563748	1.327998	0.1991759
cg04223389	5.56E-27	9.12E-47	3.39E-07	0.0092993
cg25138553	0.3081845	0.1122605	0.846047	0.0223465
cg17943391	4.3961253	1.1483143	16.82982	0.0306277
cg00730794	2.39E+64	5.08E+27	1.13E+101	0.0005803
cg01130273	1.5976006	0.6483157	3.936859	0.3086071
cg13791671	1.8227939	0.7971505	4.168068	0.154818
cg22650458	2.9307823	0.667172	12.87447	0.154446
cg12124018	0.2966368	0.1236188	0.711813	0.0065055
cg14142087	2.5828515	0.5962874	11.18776	0.2045536
cg03915012	0.0293699	0.0041104	0.209856	0.0004379
cg10858828	0.3820986	0.0904134	1.614797	0.1907712
cg25117600	0.4734742	0.2206331	1.016066	0.0549775
cg00497905	0.2029063	0.0760634	0.541272	0.0014419
cg26104690	0.354401	0.1646243	0.76295	0.008012
cg17082225	0.324016	0.1137985	0.922563	0.0347778
cg13331200	0.1987318	0.0312034	1.265705	0.0871695
cg05044414	0.2677895	0.1011701	0.708818	0.0079796
cg24124145	0.067212	0.0096021	0.470463	0.0065387
cg15814717	2.9651851	0.9652069	9.109262	0.0576799
cg23460809	0.4202402	0.025376	6.959415	0.544966
cg13757081	0.4194781	0.2065336	0.851977	0.0162571
cg27579805	0.1937362	0.087396	0.429467	5.32E-05
cg25593948	1.5464752	0.8054828	2.969133	0.1901961
cg00499707	0.3170142	0.1374712	0.731048	0.0070423
cg01684528	3.28E-05	1.91E-22	5.63E+12	0.610077
cg02536045	3.02E-05	1.53E-13	5950.177	0.2855023
cg03637696	1.8585628	0.7149686	4.831339	0.2035125
cg05130355	0.3318901	0.1062294	1.036917	0.0577496
cg24489074	0.3187631	0.1345398	0.755241	0.009382
cg09909381	4.0171158	0.2250099	71.71781	0.34434
cg08025954	2.0547299	0.7640372	5.525798	0.1536528
cg16411445	2.4355825	1.0637806	5.576396	0.035182
cg21232161	1.9872859	0.2435167	16.2178	0.521409
cg25560471	0.4567511	0.2144285	0.972919	0.0422423
cg01288904	0.3824056	0.1841839	0.793957	0.0099095
cg08228724	6.124E+18	7.3380857	5.11E+36	0.039915
cg07141055	1.5228346	0.7091442	3.270174	0.2807851
cg06110816	0.1338348	0.0131378	1.363379	0.089465
cg04144394	0.809992	0.3530639	1.858267	0.6189101
cg13811936	1.4300082	0.5670214	3.606431	0.4485408

cg24530460	2.4985761	0.9206075	6.781264	0.0722436
cg15583058	0.0289948	0.0016795	0.500553	0.0148458
cg04955511	1.9077055	0.4947851	7.355396	0.3482135
cg19090128	0.4957968	0.219992	1.117379	0.0905962
cg08596637	2.945E+16	7.94E-60	1.09E+92	0.669277
cg08027792	1.254162	0.549157	2.864249	0.5909389
cg20050877	0.3514672	0.1365103	0.904908	0.0302311
cg03881961	29801567	2.47E-05	3.59E+19	0.2252948
cg13368983	1.9552681	0.6192728	6.173488	0.2530168
cg14957089	1.6413821	0.5465464	4.92938	0.3771256
cg12123019	1.3140747	0.5393132	3.201835	0.5477765
cg03309967	0.582326	0.2004186	1.691976	0.3204151
cg17419731	0.2364591	0.0946232	0.5909	0.0020298
cg04361266	0.4724613	0.2275981	0.980763	0.0442105
cg02266086	0.3145939	0.1491376	0.663611	0.0023917
cg03954144	3.657828	0.6814081	19.63538	0.1303891
cg27164797	0.8427174	0.4548386	1.561373	0.5865338
cg24032252	0.2968249	0.1183625	0.744366	0.0096166
cg16068833	3.6076476	1.0785113	12.06767	0.0372834
cg26768556	0.3094917	0.1265379	0.756968	0.0101661
cg19517476	2.8105265	0.7291003	10.83398	0.1333462
cg17959327	1.7484167	0.8727177	3.502806	0.115038
cg26557756	0.4330571	0.2117083	0.885834	0.0219078
cg09432775	1.3878745	0.0505822	38.08052	0.8461972
cg25404995	0.3478662	0.1393574	0.868349	0.023672
cg01923312	2.2295569	0.6656246	7.468059	0.1935945
cg02378074	8.38E-05	7.57E-08	0.092926	0.0086849
cg13006424	15.444373	1.5819303	150.7833	0.018549
cg24702091	2.0210968	0.6080187	6.718268	0.2509192
cg08248285	0.2676802	0.0632181	1.133421	0.0734733
cg17212685	1.155761	0.5163271	2.587087	0.7247544
cg09136052	0.8811687	0.3852998	2.015206	0.7643806
cg05372669	0.5429365	0.1373865	2.145626	0.3836945
cg09878267	2.3500176	0.39817	13.86991	0.345528
cg05962079	2.0129013	0.4575568	8.855231	0.3546774
cg00996827	0.178446	0.0522023	0.609991	0.0059929
cg03695162	0.367691	0.0018941	71.37724	0.7097389
cg21624854	2.64E-09	4.19E-18	1.662543	0.0560319
cg09145019	4.4637978	0.3468222	57.45161	0.2511251
cg16567815	0.8604937	0.3150049	2.350597	0.7694905
cg16653538	1.19E-14	9.72E-27	0.014554	0.0239566
cg04537900	0.023584	0.000106	5.249194	0.1742285
cg03434029	0.0720509	0.0078095	0.664743	0.0203323
cg22822824	1.2384365	0.5121693	2.994566	0.6349996
cg00920254	0.4949921	0.1790917	1.36811	0.1751917
cg00327697	0.001802	5.45E-06	0.596295	0.0327919
cg16188379	0.2789297	0.0770925	1.009201	0.0516541
cg17248495	71756.59	0.2050578	2.51E+10	0.0860355
cg09351623	5.846E+12	1.79E-15	1.91E+40	0.3631256
cg03301026	447.49098	0.8170654	245082.2	0.057806
cg26245549	0.8844384	0.0282223	27.71683	0.9442978
cg04812644	0.4116761	0.1956765	0.866109	0.0193482
cg19727499	0.2139085	0.0928276	0.492923	0.0002937
cg01544903	2.8008277	0.9940733	7.891406	0.0513293
cg03431741	0.6587753	0.3097617	1.401028	0.2783227
cg15234271	0.8696206	0.3966094	1.906762	0.7272787
cg13554818	0.0836321	0.0243047	0.287776	8.30E-05
cg08402963	0.3698252	0.1859937	0.735351	0.0045601

cg15948088	3.1479706	1.2951146	7.651615	0.0113857
cg21581129	8.16E+16	2.60E-13	2.56E+46	0.2611352
cg16142349	3.4471806	0.9130533	13.01463	0.0678851
cg27475522	0.6283536	0.3063292	1.288902	0.2049391
cg04439600	5.38221	1.0967167	26.41355	0.0381062
cg18201070	5.7146722	1.49559	21.83585	0.0108191
cg14993964	0.0005069	1.66E-07	1.546071	0.0638078
cg23808004	2.6109369	1.3153869	5.182499	0.0060758
cg07849944	1.7994428	0.9091971	3.561378	0.0916685
cg08508431	2.1406491	1.0408269	4.402633	0.0385716
cg11826685	0.0044006	1.66E-06	11.63253	0.1771369
cg12126857	1.2609728	0.647314	2.456385	0.4955039
cg27492874	1.978E+10	3.80E-05	1.03E+25	0.1702874
cg09500421	3.7849476	0.6548816	21.87545	0.1370025
cg01454148	0.4326135	0.1973067	0.948546	0.0364526
cg20566897	2.1641388	0.8799059	5.322725	0.0926985
cg12771178	0.9133919	0.3720936	2.242137	0.8432666
cg25351550	0.4726971	0.1905164	1.172826	0.1060667
cg07506689	0.1615472	0.0481897	0.541557	0.00314
cg25471923	2.3814703	0.9183749	6.175475	0.0742907
cg02512108	0.1928286	0.0491007	0.757278	0.018358
cg12697325	1.6982139	0.596344	4.836018	0.3212864
cg10592766	1.6565557	0.7267379	3.77602	0.2298767
cg19653700	0.7622828	0.3194223	1.819144	0.5407749
cg07115975	4.03E-08	1.23E-13	0.013277	0.0086219
cg20564283	1.3863713	0.6089964	3.156054	0.4363598
cg19725489	0.1515915	0.0128804	1.78411	0.1336803
cg16266809	2.1112158	0.6050115	7.367186	0.2412363
cg25095518	3.1380544	1.2515486	7.86816	0.0147528
cg01259637	18.21998	0.0657342	5050.152	0.3118202
cg20048521	1.4073119	0.5378223	3.682493	0.4863019
cg06742628	0.2398887	0.0960983	0.598831	0.0022238
cg18162120	2.65E-09	1.80E-19	38.96908	0.0982621
cg21009226	4.34E-34	4.47E-55	4.20E-13	0.0018343
cg09775972	0.5806483	0.2646036	1.27418	0.1751969
cg16258229	0.1890766	0.0310327	1.152011	0.0708425
cg06110221	0.0152861	0.0015355	0.152176	0.0003629
cg22438280	0.4496234	0.2170221	0.931524	0.0314898
cg11420192	0.2314719	0.0695364	0.770521	0.0170873
cg09660810	0.2912988	0.1228125	0.690931	0.0051269
cg09399371	1.0668431	0.4659446	2.442681	0.8783285
cg20136061	1.704538	0.8605294	3.376352	0.1262053
cg06902898	1.51E-07	3.10E-11	0.000741	0.0002913
cg03467725	0.1307712	0.0169811	1.007066	0.0507955
cg12215739	2.9967037	0.7128348	12.59792	0.1341459
cg26038547	8.4346109	0.1654369	430.029	0.2877678
cg17057514	37164.46	2.5984262	5.32E+08	0.0311174
cg03759993	1.7948506	0.3385599	9.51527	0.491884
cg18395354	0.552899	0.2536527	1.205181	0.1360849
cg14810638	0.4566604	0.1084824	1.922328	0.2851574
cg09108191	46.20045	0.9054882	2357.272	0.0560715
cg11960711	0.0002388	4.93E-07	0.115699	0.0082028
cg20214973	1.4966276	0.4173647	5.366755	0.5360108
cg17047566	165.42788	3.27E-10	8.37E+13	0.7102401
cg19038804	0.5368655	0.0274816	10.48791	0.681683
cg01410553	0.0321309	4.84E-08	21320.34	0.6152086
cg18739675	2.777681	0.8329631	9.262729	0.096405
cg06611426	0.2884192	0.0927094	0.897273	0.031781

cg07568588	1.7677973	0.475316	6.574799	0.3952508
cg18812944	0.1193082	0.0215689	0.659953	0.0148436
cg19205850	0.5104264	0.1351747	1.927396	0.3211814
cg09862193	0.4759769	0.2314395	0.978891	0.0435958
cg26021627	1.5397754	0.8605051	2.755252	0.1459702
cg04485988	0.6410952	0.106434	3.861577	0.6274931
cg14825735	1.344361	0.6993784	2.584161	0.3747894
cg16193203	0.5707584	0.2461254	1.323573	0.1913027
cg07274590	3.732892	1.1433414	12.18751	0.0291203
cg23710498	0.1365204	0.0322788	0.577401	0.0068011
cg20770006	0.1887453	0.0579289	0.614975	0.005663
cg07087018	2.836E+11	0.0160832	5.00E+24	0.0901561
cg07985164	0.8436519	0.0749396	9.497631	0.8905284
cg22379213	5.023763	0.9430234	26.76306	0.0585935
cg00027400	0.0105475	0.0006432	0.172962	0.0014254
cg06737561	0.2660045	0.0744289	0.950684	0.041571
cg00219321	0.3881589	0.1976316	0.762365	0.0059997
cg16281276	1.2422677	0.5783884	2.668154	0.5780691
cg08895056	1.6144892	0.6822813	3.820382	0.2757093
cg14039246	0.3950825	0.1863072	0.837811	0.0154619
cg08258582	3.8514098	1.1440564	12.96558	0.0294608
cg24536824	0.2816349	0.1072925	0.739271	0.0100678
cg01085830	706.05019	0.1047732	4757962	0.1447299
cg10761315	0.0903213	0.0165021	0.494358	0.0055671
cg00009292	1.2076136	0.6284297	2.320595	0.5713507
cg16226669	0.0464501	0.0077445	0.278599	0.0007845
cg13813391	6.6551006	0.6012563	73.66304	0.1222938
cg05574141	0.6211791	0.2685108	1.43705	0.2658597
cg12158889	0.3628863	0.1117551	1.178348	0.0916304
cg17410650	2.269423	1.0404067	4.950257	0.0394454
cg04168675	2.003207	0.4453664	9.010196	0.3651416
cg23492582	1.117E+13	387858.51	3.22E+20	0.0006071
cg04134048	1.9032533	0.8263332	4.383671	0.1305739
cg14114267	0.3016102	0.1127475	0.806836	0.0169639
cg07537523	1.5393308	0.783414	3.024632	0.2106924
cg11835068	0.1191083	0.0170637	0.831403	0.0318563
cg10503083	1.4943948	0.7917994	2.820431	0.2151208
cg02912184	0.1105403	0.0002594	47.11123	0.4759029
cg12150931	0.8088912	0.2876889	2.274349	0.6876064
cg04864453	1.4718459	0.536997	4.034157	0.4524487
cg04751149	2.3934089	0.7841789	7.304974	0.1252937
cg15262505	0.2755882	0.0991965	0.765641	0.0134289
cg09132923	0.3244123	0.0051855	20.29584	0.5937259
cg05348973	1.2556367	0.6191355	2.546492	0.5280331
cg26811705	0.1161046	0.0366655	0.367656	0.0002509
cg17675386	0.3987516	0.1861646	0.854098	0.0179927
cg02370770	2.790244	0.7306612	10.65536	0.1333682
cg16601494	0.5512193	0.3065132	0.991288	0.0466804
cg21584461	74.420107	0.4967122	11150.02	0.0917596
cg02165670	0.8002351	0.3968257	1.613747	0.533472
cg12504148	0.2444936	0.1015259	0.588787	0.0016825
cg20367223	376280.24	0.0373291	3.79E+12	0.118679
cg06637812	3.0391809	1.0580509	8.729845	0.0389438
cg08598296	3.89E-11	1.55E-17	9.75E-05	0.0014299
cg06085713	0.2259737	1.48E-05	3454.033	0.7622196
cg13922021	1.9153877	0.7340368	4.997992	0.184139
cg21609706	0.7713099	0.3535299	1.682797	0.5141578
cg01651886	0.1830243	0.005534	6.053152	0.3414582

cg18355146	0.3937872	0.1401079	1.106778	0.0771369
cg27128984	0.5264252	0.1952061	1.419646	0.2049135
cg07897296	6.3801961	1.2739784	31.95258	0.0241616
cg12544392	0.386331	0.1291107	1.155997	0.0889931
cg01596963	0.3837001	0.1475577	0.99775	0.0494625
cg18681998	6.968154	0.8030774	60.46138	0.0782334
cg20528338	0.4559818	0.0385729	5.390294	0.5331741
cg18562935	4.6929227	1.7206703	12.79939	0.0025267
cg00775197	0.4840557	0.1716444	1.365089	0.1701816
cg02100997	1.9784565	0.7088852	5.521755	0.1925929
cg10068417	3.3053711	0.8851732	12.34276	0.075319
cg05914034	0.5475652	0.0680103	4.408563	0.5714396
cg18446916	16.008737	0.6044021	424.0218	0.09716
cg15648345	0.3696378	0.1689936	0.808505	0.0126924
cg00087568	0.0550295	0.0024467	1.237688	0.0678941
cg11902424	0.3710908	0.1502446	0.916562	0.0316483
cg09324669	0.0545384	0.0022103	1.345718	0.0753338
cg07757358	0.7441066	0.046532	11.89921	0.8344619
cg06902698	0.2946063	0.1000109	0.867834	0.0266141
cg20375608	1.0597521	0.0863439	13.00699	0.9638176
cg12332674	0.3508054	0.1093474	1.125444	0.0781937
cg18339718	0.3555439	0.1730908	0.730319	0.0048675
cg15577373	0.1464387	0.0183933	1.165874	0.0695278
cg00970057	1.70829	0.528817	5.518459	0.3707576
cg08125291	0.3902145	0.1705032	0.893047	0.0258978
cg14235768	3.0403443	0.8363247	11.05276	0.0913063
cg03522247	0.4541871	0.2019679	1.02138	0.0562877
cg04439077	0.4811698	0.2054406	1.126965	0.0920473
cg05783223	3.2991228	1.1217551	9.702841	0.0301046
cg03421807	8.60E-10	1.74E-28	4.26E+09	0.3419028
cg26032026	0.1748981	0.0597549	0.511913	0.0014627
cg23958600	0.5947843	0.2950654	1.198949	0.1463202
cg13313047	0.3643257	0.1205697	1.100883	0.0735162
cg16964249	1.07E-14	1.54E-28	0.74108	0.0478846
cg07156824	1.8143771	0.1438434	22.88575	0.6450525
cg26807432	0.4834324	0.2534971	0.921931	0.0273315
cg03149958	0.1461904	0.0394971	0.541093	0.0039796
cg24524396	8.69E-06	1.04E-09	0.07236	0.0114017
cg13548361	0.0030852	8.70E-05	0.109436	0.0014982
cg22776856	0.631356	0.2167114	1.83936	0.3992619
cg19500479	1.5775573	0.8165565	3.047783	0.1748443
cg09985192	1.019384	0.2228044	4.663929	0.9802585
cg02062480	0.6124837	0.1976731	1.897761	0.3955378
cg27313021	0.1507367	0.0441445	0.514709	0.0025283
cg03217915	0.1805033	0.020923	1.557206	0.1194383
cg03046001	0.6991543	0.3140169	1.556657	0.3808484
cg23102014	1.147208	0.5285461	2.490012	0.7283448
cg13802583	0.4677798	0.18324	1.19416	0.1120879
cg20335425	0.3825203	0.1000886	1.461922	0.1600758
cg03141594	1.055E+09	0.0002013	5.53E+21	0.164403
cg18277507	0.7115655	0.2231812	2.268675	0.5651463
cg22493877	0.0329459	0.0069293	0.156645	1.78E-05
cg00574819	0.3803937	0.1903646	0.760117	0.0062092
cg09939229	163.79956	6.51E-05	4.12E+08	0.4977281
cg23720306	0.0799036	0.0296062	0.21565	6.09E-07
cg22954052	2.710355	0.4410478	16.65585	0.2817877
cg12786198	0.299731	0.1109998	0.809358	0.0174401
cg17773636	0.0020546	2.68E-14	1.57E+08	0.6284552

cg23173466	1.3199052	0.5971056	2.917658	0.4928263
cg08580836	0.2281999	0.0630366	0.826111	0.0243863
cg00625021	4.88E-16	7.47E-29	0.003191	0.0191958
cg12743970	0.3447205	0.1391739	0.85384	0.0213683
cg04124361	11.564847	0.1776096	753.0318	0.2506003
cg24577193	3408.8177	33.060036	351482.9	0.0005838
cg27100547	0.0084551	1.71E-07	418.9105	0.3868525
cg13453607	0.3971836	0.1331658	1.18465	0.0977103
cg24433189	0.1151267	0.0247345	0.535856	0.0058672
cg26938314	1.7007547	0.663189	4.361602	0.2690542
cg09565226	0.2268866	0.0861907	0.597252	0.0026674
cg27541454	1.2187097	0.6015847	2.468901	0.5829246
cg10717312	1.8219071	0.5214445	6.365674	0.3473092
cg23998391	0.1171695	0.0257272	0.533626	0.0055728
cg01297762	0.5508113	0.2530534	1.198929	0.1328957
cg03792788	2.5278696	1.1874243	5.381501	0.0161473
cg02212846	2.868259	1.1151873	7.377155	0.0288042
cg00822007	3.3779496	1.6542912	6.897542	0.0008319
cg04304802	0.3036369	0.0982947	0.937948	0.0383321
cg00794400	0.1138759	0.0234106	0.553925	0.0071055
cg02152417	0.3535518	0.1330982	0.939148	0.036986
cg27305303	1.8588309	0.6925811	4.98895	0.2184228
cg02943290	0.5376786	0.2633822	1.097638	0.0883604
cg14831539	0.9513365	0.3404775	2.658152	0.9241885
cg14667838	0.1937819	0.0622577	0.603161	0.0046163
cg07784084	0.4406785	0.187534	1.035532	0.0601265
cg24152098	1.5959849	0.6562088	3.881642	0.302569
cg08303483	0.767638	0.3606496	1.633908	0.4926506
cg20576510	5.249844	0.0742596	371.1419	0.4453429
cg03798359	0.0137002	0.0002906	0.645854	0.0290843
cg11183445	6.17E-10	3.99E-17	0.009526	0.012039
cg16709691	4.9220936	0.4724011	51.28482	0.1825932
cg25576789	0.2957003	0.099237	0.881109	0.0287296
cg00848007	1.8711047	0.8931121	3.920037	0.0968371
cg17064981	0.654245	0.3499793	1.223034	0.1837806
cg01416168	0.0773304	0.0103886	0.575633	0.0124473
cg16891298	0.7296191	0.003338	159.4795	0.9086919
cg06127746	0.2790576	0.0246378	3.160716	0.3026958
cg22869239	4.42E-08	3.88E-22	5040366	0.3051624
cg06297857	0.0031392	4.29E-07	22.94823	0.2041809
cg24010952	0.4270267	0.1078431	1.690899	0.2255587
cg22057050	0.124932	0.0119946	1.301248	0.0819086
cg11557618	5.3330841	1.2084467	23.53582	0.0271103
cg22653012	0.9784414	0.407627	2.348587	0.9610909
cg19404426	0.53916	0.2635579	1.102959	0.0907199
cg12135573	0.0067913	0.0001549	0.297763	0.0096533
cg08865099	3.9339977	1.3615436	11.36676	0.0114046
cg08173649	1.1411844	0.3094585	4.208325	0.8427715
cg05407489	0.2538077	0.0814914	0.790493	0.018003
cg08318283	1.605539	0.696092	3.703182	0.2668443
cg03890505	0.7684434	0.3851445	1.533204	0.4548515
cg05026785	2.5601285	1.0076144	6.504728	0.0481652
cg00584022	4.93E-10	2.36E-20	10.29651	0.0771215
cg05028089	2875513.9	3021.6016	2.74E+09	2.14E-05
cg17120143	5326.7955	1.49E-08	1.9E+15	0.5272234
cg01923252	2.2317173	1.0517086	4.735687	0.0365009
cg19746874	45.17508	1.48E-06	1.38E+09	0.6647951
cg05770740	0.1958265	0.0572326	0.670038	0.0093777

cg27046201	0.825524	0.4426123	1.539699	0.5465801
cg25550425	1.3802269	0.6128845	3.108296	0.4365739
cg03466124	0.5910954	0.1680554	2.079039	0.4125765
cg26630171	0.378143	0.1670391	0.85604	0.0196567
cg11187916	1.586005	0.4766825	5.276913	0.4520637
cg25799955	0.8866119	0.3099426	2.536213	0.8224247
cg17755321	2.4068373	0.0222676	260.1475	0.713169
cg05694052	0.5175559	0.2469469	1.084703	0.0810547
cg13700315	0.1648853	0.035788	0.759673	0.0207436
cg08271153	1.2305274	0.4823728	3.139061	0.6641738
cg02003272	4.9258876	0.5270726	46.0361	0.1620122
cg10906607	1.3840353	0.5255546	3.644824	0.5106373
cg10627206	0.0094491	3.85E-05	2.317648	0.0968024
cg09692822	0.0841023	0.0026965	2.623095	0.1583838
cg17984956	0.1229336	0.0322259	0.468961	0.0021515
cg08396863	1.210749	0.5805201	2.525172	0.6101123
cg06922938	0.5694284	0.1661158	1.951943	0.3703073
cg02288819	7.2096374	0.6609631	78.64111	0.1051605
cg27425996	1.2743269	0.6193381	2.622007	0.5102093
cg11137517	1.29E-17	5.96E-31	0.000281	0.0130704
cg23518678	2.3089456	0.8056002	6.617711	0.1193299
cg20908131	0.6303748	0.3288577	1.208342	0.1645528
cg07896448	0.5543604	0.2707032	1.135249	0.1067214
cg05087842	1.1693221	0.5585437	2.447999	0.6781772
cg27575873	2.457941	0.8524906	7.086851	0.0959984
cg22933028	8.0776201	0.3937068	165.7273	0.1753367
cg03795574	1.3881594	0.3501855	5.502759	0.6406864
cg15358701	0.0251617	0.0004773	1.326442	0.0687107
cg04566233	0.0051286	0.0003385	0.077697	0.0001433
cg15889797	2.006977	0.2803893	14.36559	0.4878626
cg12044531	1.0553116	0.1043022	10.67746	0.9636343
cg11570508	5.983577	0.8151103	43.92436	0.0785832
cg09798888	2.2642316	1.0932234	4.689568	0.0278146
cg03171419	0.2012682	0.0813801	0.497774	0.0005206
cg22234712	4.6841597	0.9769966	22.45796	0.0534998
cg13096128	0.4477754	0.1238752	1.618587	0.2203954
cg10196737	0.2660867	0.0710864	0.996001	0.0493086
cg02805665	3.2336622	1.2524372	8.348978	0.0153051
cg09938213	1.3056534	0.4458476	3.823573	0.6266166
cg01432898	0.705919	0.3567069	1.397006	0.3173243
cg22720139	1.0879528	0.510081	2.320497	0.8273374
cg17066452	1.4196217	0.4185465	4.815058	0.573921
cg20054412	2.1210667	0.4189246	10.73922	0.3635612
cg01244651	0.5774272	0.2296712	1.451737	0.243008
cg11671265	0.6025101	0.2676677	1.356228	0.2209918
cg09625274	0.6845401	0.3283336	1.427192	0.3119884
cg00063909	0.2280276	0.0983269	0.528814	0.0005722
cg27326876	2.1907801	0.3134888	15.31001	0.4291795
cg11630206	3.2273849	0.9692824	10.74611	0.0562456
cg27544294	5.1851558	1.7101648	15.7212	0.0036362
cg07414060	0.2877808	0.1197854	0.691384	0.005349
cg16449012	0.1774446	0.0654888	0.480793	0.0006741
cg10769251	5.44E-24	1.34E-62	2.22E+15	0.2376115
cg10581650	2.061716	0.5345748	7.951502	0.2934469
cg05372679	2.4780559	1.0224681	6.005822	0.0445204
cg24873967	0.0226895	7.68E-10	670520	0.6662056
cg02078882	0.5128132	0.2128586	1.235456	0.1365784
cg18568930	0.3770883	0.1573979	0.903415	0.0286825

cg15678380	0.3360361	0.1393983	0.810055	0.0151322
cg08631783	0.5126942	0.1893673	1.388071	0.1886192
cg00877739	0.4689398	0.1673828	1.313782	0.1496566
cg13210403	0.6036231	0.2375857	1.533598	0.2886408
cg08510178	1.8319047	0.7638	4.393657	0.1750119
cg25737215	1.0417922	0.4343621	2.498678	0.9269138
cg14435109	2.1633968	0.9604476	4.873025	0.0625243
cg21611810	1.8391581	0.9746075	3.470631	0.0600291
cg21188409	0.2523357	0.1199832	0.530685	0.000283
cg16052388	0.2218833	0.0796632	0.618004	0.0039667
cg08854008	3.1192652	1.1520477	8.445671	0.0251899
cg01411921	1.6847495	0.890942	3.18582	0.1085573
cg23715407	0.3915207	0.1317081	1.16385	0.0916053
cg14399183	0.2248599	0.0666158	0.759009	0.0162073
cg13659813	0.3353604	0.1127243	0.997714	0.049521
cg08905239	0.9254077	0.3734148	2.293373	0.8670421
cg15248935	0.3158938	0.085438	1.167968	0.0841248
cg20862496	1.6728997	0.4166329	6.717169	0.4681487
cg08496030	152.23597	6.7247225	3446.356	0.0015923
cg18107425	3.5654197	0.8472822	15.00352	0.0829301
cg12390081	0.0158045	0.000287	0.870321	0.042573
cg18806561	2.0447288	0.3754442	11.13592	0.4081692
cg12656020	1.9785516	0.9205959	4.252318	0.08046
cg19862272	0.4858631	0.169074	1.396211	0.1801634
cg21005410	2.8548241	1.285369	6.340608	0.0099782
cg19917639	0.6438816	0.3070744	1.350108	0.2438768
cg02596486	6.2738136	1.897267	20.74602	0.002617
cg26145504	0.325907	0.099766	1.064645	0.0634179
cg21144120	2.4315586	1.0132808	5.834984	0.0466455
cg06707910	0.1679661	0.0381029	0.740433	0.0184226
cg22018051	0.2391706	0.0655578	0.872552	0.0302789
cg19496364	0.128043	0.0385633	0.425146	0.0007883
cg08118599	4.2014763	1.3751048	12.83713	0.0117714
cg07569151	0.8938007	0.2484374	3.215617	0.8635359
cg24130287	0.419113	0.1491535	1.177684	0.0990039
cg19597652	7.35E+35	40.878565	1.32E+70	0.0401533
cg22283115	2.1047659	0.8778708	5.046346	0.0953125
cg19407656	0.524721	0.2002811	1.374728	0.1894106
cg01899260	279.0872	3.87E-16	2.01E+20	0.7883734
cg20692059	1.49E-19	4.20E-39	5.295855	0.0591083
cg06035970	1.7244297	0.5464302	5.441972	0.3527409
cg02692952	9.64E-14	3.73E-40	2.49E+13	0.3341215
cg16684184	4.634764	0.784908	27.36758	0.0905212
cg08660971	0.3748659	0.1784278	0.78757	0.0095857
cg05311589	0.2029857	0.0014678	28.07232	0.5260603
cg08813349	2.0498385	0.9674822	4.343065	0.0609763
cg13212080	0.1827018	0.0470173	0.70995	0.0141037
cg03554199	0.1405202	0.0085048	2.321736	0.170267
cg07136133	2.620975	0.8963347	7.664001	0.0783987
cg18006568	0.4710695	0.2093512	1.059972	0.06888
cg19563307	0.2805879	0.1122637	0.701291	0.0065446
cg01091514	1.8143399	0.5526597	5.95634	0.3259939
cg05875433	69.032421	1.133772	4203.204	0.0433989
cg08926642	5.5834499	0.2726925	114.3226	0.264236
cg05955224	887.64073	0.1305621	6034721	0.1316113
cg20786074	1.7491528	0.7537555	4.059056	0.1929856
cg11567608	0.3091949	0.0936968	1.020328	0.0539887
cg08973382	0.8367296	0.2691996	2.600734	0.7580251

cg03505225	1.8753241	0.6106853	5.758843	0.2720168
cg01003666	1.9910444	0.7310183	5.422925	0.1779527
cg26118221	0.1305926	0.0336278	0.507152	0.0032738
cg19238380	0.2463995	0.0706722	0.859074	0.0279243
cg01324802	2.5132522	0.7587568	8.324719	0.1315117
cg09264065	3.127562	0.6361233	15.37696	0.1605391
cg11650763	2.364237	0.7928897	7.049677	0.1226769
cg07764786	0.5412223	0.2652753	1.104217	0.0915129
cg20972453	2.048907	0.7551703	5.559037	0.1589697
cg19325139	0.2138878	0.0230419	1.985425	0.1748842
cg19266329	0.877446	0.3750528	2.052809	0.763046
cg01005486	1.9928601	0.813739	4.880547	0.131315
cg06511276	1.9895514	0.6821974	5.802301	0.20779
cg16932827	0.2226214	0.0890065	0.556816	0.0013192
cg14655122	6.8719982	1.3445284	35.12336	0.0205786
cg23251798	0.6522541	0.3127055	1.360499	0.2546053
cg25531478	78.525333	1.2790495	4820.946	0.0377902
cg23931420	0.4197679	0.1723932	1.022111	0.0559022
cg04945668	1.7211218	0.0274655	107.8537	0.7970296
cg16201674	0.1415633	0.034533	0.58032	0.0066087
cg01229506	0.6318455	0.2767133	1.442752	0.2757839
cg20346388	0.207388	0.0771086	0.557782	0.0018304
cg23799313	0.3574675	0.0987545	1.293946	0.1170355
cg22551372	1.7482196	0.5786329	5.281883	0.3220841
cg14563196	1.228096	0.6458341	2.335305	0.5309192
cg25421917	0.0422703	0.0039278	0.454906	0.009062
cg01680999	2.044E+09	0.0428529	9.75E+19	0.0874748
cg01263077	0.015237	0.0011399	0.203677	0.0015626
cg23491424	1.7842421	0.6603218	4.821164	0.2536071
cg21042919	0.2451573	0.0812322	0.73988	0.0126125
cg18500830	1.6603383	0.6868477	4.013588	0.2602308
cg00556627	0.5378782	0.2722513	1.062669	0.0742612
cg21453309	0.2364184	0.0849218	0.658178	0.0057684
cg24567591	0.0150138	0.0008822	0.255522	0.0036903
cg02402141	0.0455916	0.0005275	3.940501	0.1747024
cg03728296	3.294031	1.0562371	10.27292	0.039952
cg15608397	0.0748785	0.0066752	0.839948	0.0356084
cg11027707	0.2727205	0.0998363	0.744984	0.0112724
cg18229196	2.0005973	0.8794246	4.551146	0.0982138
cg09792008	1.3423751	0.446597	4.034892	0.6000193
cg12720921	1.9379458	0.7741891	4.851055	0.1575772
cg06235437	3.5473952	1.64E-05	768860.8	0.839925
cg08199953	1.4894279	0.7770035	2.855065	0.2301447
cg19010441	0.8012608	0.3931343	1.633078	0.5419313
cg12968413	72199930	5.55E-05	9.39E+19	0.2035786
cg27141474	5.1957299	1.0600414	25.46656	0.0421678
cg24064224	35.290977	2.2587033	551.4018	0.011056
cg08715720	0.5195596	0.2611594	1.03363	0.0620814
cg25356261	0.0564625	2.61E-21	1.22E+18	0.8993123
cg11002404	0.0092041	0.0001829	0.463187	0.0190313
cg23334660	0.4787904	0.2400371	0.95502	0.0365635
cg16483466	0.5724888	0.2594544	1.263202	0.1671805
cg21359747	2.0850566	1.2099198	3.593181	0.0081401
cg24846807	0.5029184	0.2458441	1.02881	0.0598109
cg00588614	0.0893471	0.0097171	0.821534	0.0328735
cg09355027	0.1679569	0.0482018	0.585238	0.0050926
cg02501166	0.4013295	0.1793472	0.898065	0.0263116
cg16421340	1.3183738	0.5317496	3.268662	0.5507522

cg25225693	3.799346	0.6263988	23.04447	0.1466797
cg11323117	0.5499874	0.133001	2.274314	0.4091058
cg22455725	0.5381722	0.2631441	1.100649	0.089648
cg07976816	1.7193031	5.89E-07	5016877	0.9431193
cg09656848	0.4397937	0.1790189	1.080436	0.0732513
cg14008679	0.1549078	0.0066151	3.627495	0.2464151
cg20390613	1.633305	0.7378771	3.615352	0.2262195
cg25589945	5.5698651	0.548177	56.59376	0.1465637
cg02217022	0.218505	0.0673778	0.708608	0.0112833
cg05859323	3.37E-05	9.24E-19	1.23E+09	0.5180671
cg18067096	0.4535624	0.179091	1.148683	0.0953967
cg22961727	1.2508531	0.3701499	4.227026	0.7186456
cg27147000	1.2749177	0.4380375	3.710676	0.6558928
cg25472720	248.073	0.191034	322142.7	0.1317043
cg16708623	0.349384	0.1638403	0.74505	0.0064952
cg14276379	1.7757402	0.8480479	3.718249	0.1277945
cg13567169	0.0532835	0.0099627	0.284976	0.0006096
cg23205936	1.7164783	0.7623955	3.864527	0.1919653
cg14794041	1.7678863	0.7198457	4.341794	0.2139014
cg19410791	3.7988601	1.1283304	12.78999	0.0311697
cg20096409	1.8342691	0.6447633	5.218261	0.2554382
cg06915192	1.491369	0.5466696	4.068603	0.4350539
cg12492653	0.5178036	0.2380861	1.12615	0.0968604
cg22019177	1.7058257	0.808254	3.600157	0.1611056
cg16330359	2.3590299	1.0449537	5.325616	0.0388468
cg04499690	11602815	9.1465441	1.47E+13	0.0232894
cg25104437	6.177575	1.7050903	22.38147	0.0055642
cg10319893	0.8393791	0.3066171	2.297841	0.7332776
cg15651980	0.9404898	0.3577995	2.472113	0.9009748
cg10930290	1.6030251	0.7054494	3.642627	0.2598268
cg13849232	0.3035075	0.104114	0.884768	0.0289446
cg26145959	1.88579	0.821949	4.326551	0.1343455
cg09540961	0.3222243	0.0409805	2.533605	0.2817539
cg13816321	2.1288092	1.062128	4.266744	0.0331821
cg25278353	0.8716689	0.3702261	2.052277	0.753241
cg05673214	0.2889871	0.1214466	0.687657	0.005007
cg25950112	1.9448056	1.0524034	3.593935	0.0337552
cg18347921	1.7377468	0.8664223	3.485326	0.1196662
cg04089240	1.727553	0.7608461	3.922527	0.1913185
cg05487207	1.2042762	0.5340132	2.715815	0.6541567
cg04237918	0.3720564	0.1386024	0.998727	0.0497053
cg17739555	3.5104145	0.9328259	13.21041	0.0632934
cg16181383	1.1232241	0.4567608	2.762129	0.800178
cg15459165	3.0430115	0.7848469	11.79838	0.1074934
cg07084019	0.5489995	0.2450615	1.229897	0.1450793
cg01026503	1.5797555	0.7401541	3.371767	0.2371641
cg15941159	0.592669	0.2179792	1.611422	0.3053385
cg21113776	1.4815037	0.5949629	3.689059	0.3984331
cg10214581	0.5589289	0.2077442	1.50378	0.2493109
cg09900253	0.0124127	0.0002596	0.593616	0.026131
cg07580867	338.65489	0.2587959	443156.6	0.1116526
cg07312676	17.361027	3.38E-05	8913162	0.6705075
cg16462254	1.6758472	0.7256633	3.870202	0.2266409
cg14316431	1.8355737	0.9279598	3.630902	0.0809615
cg05386230	0.095493	0.0205787	0.443123	0.0027056
cg07211472	8.9822831	2.0237812	39.86667	0.003888
cg23110109	0.1483954	0.0244438	0.900889	0.0381358
cg16236851	1.9205017	0.8007063	4.606342	0.1437353

cg14236443	0.348408	0.106616	1.138555	0.0809522
cg12431891	0.6969029	0.3771845	1.287629	0.2489633
cg11796565	1.0806333	0.3596287	3.24715	0.8901269
cg25966893	1.695288	0.8097731	3.549144	0.1614417
cg10232198	0.525435	0.220304	1.253186	0.1467614
cg09310065	0.2167388	0.0930045	0.505091	0.0003967
cg15700197	0.8191123	0.4140912	1.620283	0.56643
cg18578263	1.1728605	0.4690753	2.932582	0.7331015
cg05347898	1.6946207	0.7237508	3.967857	0.224313
cg05048168	0.0321596	0.0025272	0.409248	0.0080875
cg01998213	3.5251643	0.9959505	12.47731	0.05074
cg09584650	1.5375612	0.6729139	3.51322	0.3075508
cg23707540	1.5290188	0.6649446	3.51593	0.317558
cg13470367	0.2201239	0.0641696	0.755101	0.0161015
cg14644941	0.4129154	0.2084491	0.817941	0.0112063
cg18576635	801648664	4.27E-33	1.51E+50	0.6724263
cg16876876	0.2172815	3.92E-07	120358.4	0.8210131
cg11837417	2.3953604	0.1725004	33.26224	0.5151972
cg14899716	0.5354747	0.2753078	1.0415	0.0657437
cg20677570	2.8682805	0.6525972	12.6066	0.16303
cg18698699	0.0006899	2.79E-06	0.170301	0.0096038
cg26089877	2.0657731	0.8725846	4.89055	0.0989447
cg24576133	30.190241	0.0510832	17842.47	0.2953273
cg23719132	3.6441671	1.0375905	12.79884	0.0436395
cg15718289	0.0001304	5.89E-10	28.8878	0.1543377
cg04573316	0.4177551	0.1504666	1.159854	0.0938689
cg07448856	4.97E-36	9.42E-82	2.63E+10	0.1301975
cg16811443	3.5771541	0.8735777	14.64785	0.076386
cg15245120	0.4947986	0.0260733	9.389887	0.6393949
cg10278699	0.0481701	0.0083857	0.276703	0.0006729
cg04201285	0.3604264	0.1194707	1.087356	0.0700927
cg04342594	4.4614373	1.3658046	14.5734	0.0132813
cg00736104	0.7839602	0.2313273	2.656814	0.6959037
cg26434376	4.8569862	0.0343946	685.8731	0.5314884
cg03454920	2.8063873	1.2286467	6.41015	0.0143424
cg06407441	3.534768	0.0042658	2928.998	0.7126658
cg03597607	0.0282501	7.86E-05	10.15095	0.2348293
cg14410476	4.3358519	1.8632699	10.08958	0.0006637
cg14818812	2.0275438	0.705793	5.82456	0.1892476
cg16736964	0.6513382	0.3179016	1.334505	0.2414055
cg23834919	0.6730205	0.2674294	1.693743	0.4003907
cg19355087	0.2488688	0.046494	1.332123	0.104179
cg20784813	0.1954782	0.078041	0.489637	0.0004936
cg07074666	2.3225278	0.8138533	6.627897	0.1152606
cg25771096	1.1600953	0.5249044	2.563936	0.7136071
cg08505560	3.0931113	0.0154155	620.6323	0.6763479
cg05490864	4.073376	1.2764516	12.99884	0.0176808
cg10957180	0.0185123	0.0008263	0.41473	0.0119108
cg00807366	0.482886	0.1794749	1.299229	0.1494189
cg07711387	0.9910592	0.5181124	1.895725	0.9783482
cg25093532	3.70E-18	3.45E-32	0.000396	0.0148835
cg02063817	3.4471041	0.9412981	12.62355	0.0616759
cg03880987	0.2405489	0.072139	0.802115	0.0204049
cg08048948	0.0262376	0.0001766	3.898897	0.1536619
cg03711457	0.0965079	0.0210595	0.442261	0.0026091
cg05135644	2.1112624	0.9405516	4.739165	0.0700787
cg04434896	2.170696	0.9597846	4.909353	0.0626898
cg02864126	1.628268	0.6917725	3.832556	0.2643208

cg23716866	0.4289603	0.1893559	0.971752	0.0424946
cg12431299	1.8106309	0.7606292	4.310095	0.1797133
cg00767042	4.3157815	0.5662254	32.89497	0.1582136
cg12952190	0.0022896	8.71E-06	0.602213	0.0324887
cg01434679	2.3805629	0.3291121	17.2193	0.3902705
cg03932027	0.7156039	0.3640541	1.406629	0.3318178
cg01265662	0.3188265	0.1508677	0.673771	0.0027509
cg09308536	2.3498388	0.7578241	7.286311	0.1389568
cg13920312	0.749716	0.2549316	2.204804	0.6006975
cg16597406	0.4394832	0.1208718	1.597937	0.21192
cg05539509	1.5815767	0.5667818	4.413312	0.3812753
cg09352372	1.4744822	0.7241463	3.002291	0.2844776
cg07235805	0.2381856	0.0651205	0.871191	0.0301302
cg11553045	3.2429067	1.0479539	10.03522	0.0412277
cg14060496	0.1865335	0.0413356	0.841761	0.0289611
cg06080459	6.8508558	1.6901242	27.76969	0.007041
cg09302903	1.05354	0.5235205	2.120159	0.8837851
cg00927777	2.2422653	0.7486111	6.716108	0.1491125
cg23044391	0.000119	3.06E-08	0.462934	0.0321478
cg25820479	1.1792459	0.5419787	2.565822	0.6776459
cg26811252	0.1268704	0.0154678	1.040621	0.0544953
cg23900712	0.382022	0.1575325	0.926417	0.0332485
cg12476490	585519.05	321.71881	1.07E+09	0.0005254
cg10488050	1.0973401	0.3715251	3.241114	0.8665028
cg08922729	0.1180365	0.0307651	0.452871	0.0018417
cg26235434	94.428695	0.0131779	676646.5	0.3153219
cg27386563	0.0299618	0.0045761	0.196173	0.0002534
cg06776741	8.6007876	0.6190632	119.4927	0.1089834
cg07310916	0.1836856	0.0502601	0.671315	0.0103879
cg18045395	0.1093914	0.019383	0.61737	0.012204
cg17458693	2.1089537	0.7728932	5.754593	0.1451264
cg24944395	0.8185261	0.2948344	2.272411	0.7007
cg15604182	1.4859907	0.2488797	8.872433	0.6639612
cg05451210	0.332781	0.1096311	1.010144	0.0521192
cg06939970	2.3455285	0.4033179	13.64061	0.342581
cg16523839	2.1643919	0.9978736	4.694575	0.0506332
cg18223379	0.4257708	0.1722819	1.052234	0.0643613
cg01717881	2.5946522	0.8210075	8.19995	0.1043692
cg15150017	3.3906437	1.0604574	10.84104	0.0394993
cg05337806	1.9108203	0.6573793	5.554227	0.2342753
cg26974035	0.8396936	0.2695141	2.616135	0.7631594
cg03880407	3.7195435	1.3469079	10.27168	0.0112579
cg19117070	0.7733406	0.3548867	1.685202	0.5177824
cg18106312	1.7947613	1.0045927	3.206442	0.0482182
cg24794906	6.368871	1.1975329	33.87174	0.0299024
cg10395448	1.952574	0.9430864	4.042626	0.0715219
cg11094248	2.3078736	0.6848011	7.77785	0.1772847
cg02181506	0.0408274	0.0049168	0.339019	0.0030608
cg18705773	2.1972887	1.1843081	4.076708	0.0125465
cg01895992	0.90919	0.002838	291.2684	0.9741999
cg07460010	2.1320996	0.8721038	5.212509	0.0969281
cg17152101	0.0097648	5.03E-05	1.896045	0.0850748
cg20355301	0.3889768	0.1978833	0.764607	0.0061755
cg19744326	16.180735	0.9787922	267.489	0.0517765
cg26209990	5.63291	1.3206889	24.02509	0.0195006
cg16118212	0.2146801	0.0411321	1.120476	0.0679959
cg10195894	1.4367772	0.6348807	3.251522	0.3844684
cg09484009	24.402458	0.0568757	10469.84	0.3016144

cg13476077	1.3960848	0.7676854	2.538869	0.2741595
cg26059632	0.3944676	0.1082181	1.43788	0.1586499
cg19406706	0.5937443	0.3143028	1.121633	0.1082123
cg07139196	2.6829795	0.498148	14.45028	0.250636
cg15125666	0.3369622	0.1461386	0.776958	0.0107092
cg22209929	0.1706003	0.0566179	0.514051	0.0016757
cg14201467	0.9031574	0.4468604	1.825387	0.776626
cg14343178	0.317073	0.119056	0.844437	0.0215454
cg12984877	0.3785877	0.1496627	0.957677	0.0402386
cg22496437	0.1225189	0.0254515	0.589783	0.0088321
cg14311481	0.3685643	0.1245237	1.090874	0.0714101
cg04198075	1.2371262	0.2868275	5.335894	0.7753882
cg00903998	1.3385991	0.7219195	2.48206	0.3546152
cg12501546	1.567518	0.7290739	3.370183	0.2497694
cg16536855	0.4149176	0.2027835	0.848968	0.0160312
cg07520810	0.2129471	0.0341987	1.325969	0.0973995
cg24738627	1.4513747	0.7731284	2.72463	0.246362
cg22163674	0.0314164	0.0013391	0.737068	0.0315977
cg18320766	0.1434865	0.036584	0.56277	0.005362
cg11736352	0.0022015	2.13E-05	0.228026	0.009756
cg10500084	1.4332903	0.5448661	3.770323	0.4657149
cg22129183	1.0111057	0.4244406	2.408664	0.9801046
cg02688422	0.386653	0.1204989	1.24068	0.1101719
cg25248415	1.5340138	0.7865539	2.991783	0.2093009
cg01246520	1.5144496	0.6516608	3.519558	0.3347119
cg23251858	0.4224002	0.1885081	0.946495	0.0363002
cg15073853	0.00936	0.000664	0.131934	0.0005395
cg19852827	2.4216024	0.7844842	7.475177	0.1240745
cg05810129	1.5103972	0.7716958	2.956216	0.2287597
cg21102746	2.2494116	0.219393	23.06296	0.4948354
cg07187105	0.1868715	0.0376847	0.926661	0.0400533
cg02395846	1.7460524	0.8539118	3.570274	0.1267052
cg19051238	0.6296158	0.323252	1.226337	0.1737885
cg18078696	6.02E+25	2.75E-08	1.32E+59	0.1296488
cg06524039	0.2433667	0.0712019	0.831823	0.0242211
cg02175308	0.1191954	0.0245107	0.579646	0.0083954
cg21150288	0.6431101	0.3224781	1.282539	0.2100564
cg03919781	0.000675	2.02E-07	2.251926	0.0777592
cg12212453	1.3267229	0.6355067	2.769748	0.4515613
cg20387392	0.6620247	0.3412822	1.284206	0.2224489
cg09866757	0.8982042	0.322613	2.500739	0.8371838
cg25755905	3.5704224	0.9967466	12.78953	0.050588
cg17810781	2.3933107	0.4873564	11.75307	0.2824811
cg01679225	0.6181229	0.2954313	1.293282	0.2015404
cg27283993	385.66004	3.1463323	47272.08	0.0152178
cg16710348	0.5125782	0.1696945	1.548291	0.2360584
cg22070232	0.4219731	0.1619228	1.099667	0.077471
cg02490942	5.3208788	1.3082894	21.64028	0.0195234
cg11674664	8.03E-05	8.40E-10	7.673783	0.1070321
cg06727093	0.3002475	0.0894671	1.007616	0.0514531
cg23622235	1.7259599	0.454671	6.551852	0.4226066
cg14625175	1.26476	0.5083907	3.146434	0.6134739
cg08200419	0.4544424	0.1758326	1.174514	0.1035374
cg25984249	5.5289341	0.0009393	32544.71	0.6994195
cg12838061	3.8816257	1.1496338	13.10593	0.0289203
cg00774005	2.7353623	0.7516464	9.954424	0.1268123
cg21413173	0.3881508	0.1856083	0.811715	0.0119317
cg25969802	0.5391173	0.2662279	1.091724	0.0861276

cg18235443	3.29E-36	1.70E-62	6.38E-10	0.0081557
cg14769021	0.2070502	0.0334595	1.281243	0.0903682
cg08575330	1.3984442	0.7523415	2.599413	0.2890173
cg19093689	6.1657674	0.7539239	50.4251	0.0897872
cg23963476	0.1086918	0.0290229	0.407055	0.0009874
cg21110337	2.4280745	0.7399155	7.967864	0.1434277
cg07473634	2.1510798	0.7531791	6.143484	0.152553
cg18065397	1.5877512	0.8220618	3.066623	0.1686503
cg05076914	0.1813095	0.0451952	0.727359	0.0159926
cg03740333	0.3867212	0.0612666	2.441024	0.3121905
cg24425807	3.1806041	0.7533384	13.42855	0.1153644
cg15692862	0.0010632	1.61E-12	701465.9	0.5087496
cg04779640	268.58435	3.1393116	22978.78	0.0137425
cg18629151	3.8329381	1.3271818	11.06963	0.0130259
cg07313705	1.519151	0.7772078	2.969373	0.2213812
cg09362335	0.5341487	0.2366026	1.205882	0.1312079
cg00495811	0.6242898	0.2643845	1.474132	0.2824958
cg04286194	2.2829496	0.7876877	6.616657	0.1284111
cg20575163	443.75783	6.37E-05	3.09E+09	0.4483396
cg25023684	1.5124306	0.6525685	3.505297	0.3347029
cg01139553	2.6184582	0.0950666	72.12125	0.5693635
cg25570929	0.5599059	0.2589859	1.21047	0.1403747
cg17080335	0.5187871	0.2423377	1.110599	0.0910565
cg16127683	0.2731327	0.1003987	0.743052	0.0110352
cg22610676	4.4909286	1.3230982	15.24334	0.0159969
cg07914866	0.9042887	0.4771615	1.713755	0.7577455
cg13311440	2.9269357	1.1644069	7.357353	0.0223938
cg16006841	0.4582519	0.1789066	1.173768	0.1039298
cg10838157	5.616629	1.8272125	17.26483	0.0025947
cg11459133	1.5156453	0.7359551	3.12136	0.2592405
cg02417408	1.4984696	0.5561128	4.03769	0.4238783
cg07783477	0.2184169	0.0724399	0.658559	0.0068974
cg13686739	0.444728	0.1473063	1.342665	0.1506324
cg11891393	2.3294305	1.2129395	4.473633	0.0110927
cg06834507	2.1017129	0.4955962	8.912895	0.3136326
cg15657888	0.6232655	0.1606476	2.418087	0.4943024
cg21330896	0.9752064	0.4464004	2.130436	0.94979
cg25595793	0.2113839	0.0557124	0.802032	0.0223589
cg02932669	0.9897903	0.4692019	2.087982	0.9785035
cg13745632	0.2672428	0.068316	1.045416	0.0579411
cg19990022	0.2484164	0.1026419	0.601223	0.0020137
cg18147676	0.8867994	0.0599429	13.11938	0.9303572
cg22600868	0.0239952	0.0020857	0.276062	0.0027653
cg00268518	4.45E-19	4.44E-58	4.47E+20	0.3564118
cg11435167	0.3225273	0.1633921	0.636652	0.0011088
cg16880567	0.9928769	0.4618084	2.134661	0.9853963
cg11270005	0.0009634	7.25E-06	0.128029	0.0053709
cg15061330	0.4141806	0.1584731	1.08249	0.0721363
cg12082129	9.33E-05	2.45E-11	355.6556	0.2300411
cg14294708	0.1633873	0.0297682	0.896776	0.0370352
cg10653000	1.9665006	0.8942733	4.324321	0.0925636
cg17386710	5.4762138	1.438037	20.85407	0.0126863
cg11187204	0.4237162	0.2032708	0.883233	0.021947
cg03653026	1.567159	0.7185515	3.41797	0.258807
cg05992904	1.4175804	0.3196785	6.286109	0.6460886
cg14531564	18.151045	2.7961236	117.8276	0.0023864
cg01457883	1.894401	0.4871874	7.366272	0.3564741
cg26444116	1.695933	0.7087681	4.058011	0.2353597

cg19116959	0.3928154	0.1725635	0.894186	0.0259841
cg27292264	0.6548141	0.252714	1.696707	0.3834198
cg00539925	2.6168578	0.4712589	14.53117	0.2714132
cg08747807	1.4123871	0.3911765	5.099584	0.5981204
cg17972600	27.244937	0.1744027	4256.165	0.1997238
cg00926318	0.2390232	0.0576086	0.991729	0.0486776
cg21156483	8.51E-05	1.05E-20	6.89E+11	0.6160634
cg23852354	0.2483058	0.0432951	1.424081	0.1179926
cg06308537	0.6154387	0.0030101	125.8314	0.8580763
cg18770029	0.3544295	0.1630376	0.770499	0.0088443
cg11912215	0.1437849	0.0225996	0.914802	0.0399481
cg26568226	2.7653613	0.1585664	48.22725	0.485569
cg17272620	2.4700269	1.1304601	5.396947	0.0233622
cg10046451	0.0073982	7.01E-05	0.780528	0.0389979
cg07549381	1.5214218	0.6159378	3.758049	0.3630453
cg01281501	1.4895506	0.5303133	4.183868	0.449517
cg17982866	1.6986481	0.7813215	3.692981	0.181165
cg16312552	2.7076436	0.896429	8.178377	0.0773783
cg13193782	0.2788788	0.0870926	0.892996	0.031511
cg14942906	1.7408574	0.3429974	8.835591	0.5035623
cg23571812	1.2786603	0.4233489	3.861997	0.6629402
cg16543823	0.6107124	0.2115855	1.762737	0.361869
cg24937727	2.206868	0.8723746	5.58277	0.0945974
cg15317221	1.286E+09	69.230599	2.39E+16	0.0140422
cg21370522	0.9631093	0.4853275	1.911244	0.9143951
cg11293821	3.032E+14	1.77E-05	5.20E+33	0.1400278
cg04187708	0.5436243	0.1857056	1.591376	0.2660591
cg03906434	3.4443052	0.8383148	14.15129	0.0862814
cg17199800	2.3731728	0.9297921	6.057213	0.0706531
cg16155702	1.176074	0.3545534	3.901105	0.7909357
cg26996656	0.8491833	0.4155102	1.735486	0.653952
cg26862022	6.3207914	1.334279	29.94307	0.0201601
cg08376141	0.7022078	0.3204949	1.538545	0.3770259
cg04572706	0.6365054	0.3095026	1.309001	0.2194379
cg04642300	0.2847022	0.1008189	0.80397	0.0176962
cg01542019	0.3496823	0.1450545	0.842978	0.0192608
cg26326372	0.5937302	0.192045	1.835588	0.3653155
cg20362308	0.5277138	0.1060735	2.625367	0.4348913
cg13434352	0.0066442	0.000389	0.113481	0.0005344
cg02023548	1.6566899	0.5819965	4.715873	0.3442414
cg09854734	2.0788423	0.8087693	5.343409	0.1286806
cg20067334	0.0350618	0.0034061	0.360924	0.004853
cg20992181	0.2020794	0.0734181	0.556213	0.0019648
cg09270951	1.5503043	0.0114132	210.5845	0.8611041
cg02483101	0.6644959	0.2443126	1.807335	0.4233489
cg11306783	109837.23	0.0294804	4.09E+11	0.1327155
cg21601405	1.015567	0.4342781	2.374921	0.9715704
cg21026830	1.7443717	0.944397	3.221985	0.0755306
cg19474865	0.8486512	0.4147345	1.736554	0.653275
cg05844798	9.783818	1.3072905	73.22251	0.0263584
cg14264125	11.10801	1.3368755	92.29572	0.0258325
cg14430542	1.298879	0.6135191	2.749852	0.494394
cg11063170	0.5638447	0.1784187	1.781881	0.3290719
cg11377277	2.9338925	0.4393421	19.59231	0.2665698
cg02584432	0.0176172	0.0018335	0.169277	0.0004678
cg01551699	0.6343571	0.293228	1.372341	0.2476686
cg12580770	0.6044952	0.2480136	1.473365	0.2681324
cg02335804	1.9725234	0.9436117	4.123358	0.0709673

cg15736783	0.4115745	0.1276057	1.327476	0.1373208
cg06285439	1.3936817	0.4058113	4.786334	0.5979758
cg11868041	2.116936	0.7324321	6.118544	0.1660704
cg15780361	0.4914979	0.235332	1.026508	0.0587131
cg14636113	1.3802057	0.4993294	3.815052	0.5344832
cg20667684	0.5454639	0.1715563	1.734305	0.3044149
cg06191203	0.0131471	0.0003532	0.489307	0.01891
cg16579101	10743.099	0.0121653	9.49E+09	0.1839243
cg07924575	0.6082083	0.2284005	1.6196	0.3197122
cg25310427	0.3872724	0.1885313	0.795517	0.0097998
cg07127456	1.685137	0.6759848	4.200814	0.2628268
cg05867154	1.4348685	0.7950805	2.589483	0.2306474
cg19194924	0.1552914	0.0414814	0.581356	0.0056873
cg19082304	0.5916013	0.2958614	1.18296	0.1376163
cg07435894	0.0029018	9.81E-07	8.585994	0.1519431
cg12904135	0.6973646	0.3376303	1.440384	0.3300803
cg20514520	6.2916695	0.8885654	44.54946	0.0655248
cg24148085	0.606973	0.3120417	1.180664	0.1413616
cg26276014	0.3831637	0.0487755	3.010004	0.3616834
cg01487494	79.629009	0.2037428	31121.49	0.1505708
cg01035923	0.4420237	0.1998932	0.977447	0.0437683
cg09428349	1.2610535	0.4925025	3.22893	0.6287253
cg14765172	1363.7623	51.608365	36037.71	1.56E-05
cg13693136	3.3421864	0.9662931	11.55986	0.0566752
cg23203684	2.34E-07	4.05E-21	13467032	0.3449007
cg20249566	0.334449	0.1115761	1.00251	0.0505255
cg06615840	1.4568226	0.5219482	4.066174	0.4724784
cg07658383	0.1094174	0.0088396	1.354374	0.08477
cg17278072	2.7399037	0.860903	8.719998	0.0879337
cg21890646	2.8132688	1.1091567	7.135585	0.0293968
cg16978571	1.3578415	0.4982753	3.70023	0.5498057
cg06510261	1.3932384	0.6018316	3.225343	0.4387319
cg02805028	0.8025759	0.2809975	2.292291	0.6812709
cg16752400	0.5713608	0.2247529	1.452498	0.2396684
cg01477971	0.3200014	0.0813561	1.258676	0.1029514
cg01856525	1.7862244	0.8734821	3.652734	0.1119792
cg05658215	0.4048545	0.1380227	1.187538	0.099577
cg25453841	1.7312183	0.868191	3.45214	0.1190977
cg08433673	8.95E-07	2.35E-14	34.03274	0.1178488
cg10724928	2.3692094	0.2546606	22.0417	0.4484641
cg11243391	35.571509	0.00188	673066.9	0.4772018
cg22855237	0.2000058	0.0654307	0.611369	0.0047564
cg01362541	2.3330669	1.0860954	5.011716	0.0298803
cg17136548	9.2045206	1.5901043	53.28154	0.0132245
cg27471156	10.735704	0.005155	22357.78	0.5426514
cg01624128	2.6037844	0.9557002	7.093954	0.0612955
cg11818031	2180.8775	3.13402	1517612	0.0213333
cg02881170	0.1596965	0.0352099	0.724314	0.0174037
cg01311805	1.2020907	0.2676774	5.398372	0.8101924
cg17970299	0.2478834	0.0994522	0.617846	0.0027595
cg02210967	3.0123033	0.8551344	10.61116	0.0860934
cg21122474	0.2003993	0.0531235	0.755972	0.0176473
cg07925311	0.3463176	0.1221487	0.981884	0.046114
cg08500017	1.2208268	0.0150878	98.78283	0.9290718
cg08015883	1.9788404	0.9756139	4.013687	0.0585521
cg12466599	2.0736567	0.8374996	5.134393	0.1148858
cg06103642	2.4469347	1.1863614	5.046935	0.0154089
cg04299274	3.21315	0.8040479	12.84045	0.0986558

cg09760081	109.96937	0.0496089	243772.1	0.2317727
cg03449398	0.3906808	0.175028	0.87204	0.0217801
cg19147218	1.8505121	0.8188851	4.181777	0.1389784
cg20988802	111.93202	0.1605075	78057.25	0.1578555
cg00510447	0.1012095	0.0001588	64.48785	0.4868828
cg18496502	2.63E-12	1.28E-18	5.41E-06	0.0003245
cg09321086	0.4251516	0.1719139	1.051421	0.0641084
cg25625968	0.4106313	0.1865421	0.903914	0.0270429
cg23231670	0.8248186	0.3293756	2.065502	0.6809213
cg10102897	1.1897738	0.4855426	2.915422	0.7039503
cg02612971	0.8352597	0.3548961	1.965811	0.6801852
cg23088461	1.60E-28	3.05E-42	8.38E-15	7.16E-05
cg21434530	1.0022121	0.5066135	1.982634	0.9949349
cg16290866	1.8477504	0.9190643	3.714845	0.0848707
cg27103585	1.6894488	0.8611248	3.314545	0.1272278
cg10546210	0.6987659	0.3387348	1.441463	0.331941
cg07883600	0.2444572	0.0866532	0.689638	0.0077633
cg25759457	0.4332326	1.01E-14	1.86E+13	0.9583475
cg05467568	0.5131388	0.2477735	1.06271	0.0724593
cg20015401	1.6772456	0.6036339	4.660362	0.3212756
cg14392929	2.6681756	0.3676482	19.36406	0.3318113
cg12259537	0.3681671	0.1339332	1.012049	0.0527761
cg06697175	0.310795	0.12205	0.791426	0.0142672
cg25446890	0.4516498	0.1917866	1.063617	0.068937
cg14519621	0.2589128	0.0328128	2.042975	0.1998022
cg08168847	1.58E-28	3.82E-53	0.000649	0.0268448
cg26039954	0.6204226	0.206838	1.860993	0.394363
cg22904296	4.3082017	0.2302594	80.60735	0.3284226
cg17322444	3.698E+10	0.3568212	3.83E+21	0.0600628
cg13466988	0.1494269	0.0392937	0.568243	0.0052822
cg09444979	0.3267518	0.005697	18.74096	0.5882227
cg10775039	0.7801821	0.3711921	1.639809	0.512486
cg23571170	0.575811	0.3015928	1.099358	0.0943515
cg23995446	0.4172127	0.0669402	2.600328	0.3490957
cg16635128	0.4805774	0.223853	1.031725	0.0601296
cg17252884	1.3314313	0.6478062	2.736481	0.4361083
cg25274503	0.7947978	0.0148435	42.55759	0.9099627
cg08554860	0.2728297	0.064114	1.160996	0.0787591
cg17315639	0.554149	0.2361495	1.300368	0.1749561
cg08378356	4.046E+19	1.9046432	8.60E+38	0.0467741
cg24996605	2.0130489	0.6764072	5.991015	0.2086234
cg06416764	0.2906988	0.1129711	0.748031	0.0104077
cg15235185	177.15767	4.75E-07	6.61E+10	0.6071868
cg12792931	0.2107976	0.0562269	0.790291	0.0209422
cg22560979	1.5473681	0.7550839	3.17097	0.2330457
cg06464468	0.635438	0.2834761	1.424393	0.2708886
cg13550670	0.5443358	0.2029223	1.460172	0.2270306
cg06331271	0.1144701	3.37E-23	3.89E+20	0.9317168
cg14918214	11.657917	0.7423061	183.0876	0.0804837
cg07918726	0.0372567	0.0047345	0.29318	0.001774
cg07742842	0.4304367	0.2144265	0.864053	0.0177421
cg10926487	0.4125286	0.2116365	0.804114	0.0093176
cg00088916	0.4381872	0.1984483	0.967547	0.0411917
cg14182621	1.6549657	0.7802615	3.510248	0.1891211
cg09977064	39.765439	0.0351756	44954.13	0.3045328
cg18954401	1.3032907	0.374515	4.535376	0.677163
cg07380095	1.0470008	0.5142499	2.13167	0.8992446
cg09874482	2.7782154	0.3353789	23.01421	0.3435271

cg08128274	0.4823356	0.2373702	0.980104	0.0438506
cg00150520	0.1427979	0.0361893	0.563461	0.0054516
cg01632474	1.7810725	0.5729795	5.536357	0.3185064
cg13814761	0.5368655	0.1357446	2.123285	0.3752698
cg18645642	0.3650136	0.1913069	0.696446	0.0022322
cg02908963	0.3581502	0.1566661	0.818758	0.0149341
cg14499385	1.7418113	0.5707198	5.31593	0.3296731
cg16579049	0.0482493	0.0047459	0.490525	0.0104089
cg01370384	0.0011926	1.07E-05	0.133346	0.0051551
cg14582154	1.214974	0.4700876	3.140185	0.6877408
cg24276445	5.1828536	0.50543	53.14677	0.1659249
cg12164777	0.3825744	0.1301618	1.124471	0.0806892
cg13147090	2.7287843	0.7572578	9.833195	0.1248234
cg04091712	0.1108999	0.0273759	0.449255	0.002063
cg24049493	0.9741351	0.4027875	2.35593	0.9536231
cg02939356	8.5452752	0.9372071	77.91419	0.0571116
cg17182270	2.3402089	0.4380631	12.5018	0.3199727
cg21918313	0.7003795	0.3117956	1.573247	0.3884067
cg06633814	0.1793095	0.0445859	0.721122	0.0155029
cg19934476	2.4685018	1.2142247	5.01843	0.0125543
cg16617297	0.2758225	0.1359401	0.559644	0.0003599
cg22969914	1.3713695	0.5925108	3.174042	0.4607684
cg07727358	0.2449636	0.0943247	0.636177	0.0038671
cg00473929	0.2716913	0.0812988	0.907961	0.0342759
cg21671806	1.240444	0.6281556	2.449555	0.5348308
cg14022881	0.4868097	0.0221319	10.70779	0.6480379
cg12044210	12.908463	1.7703235	94.12314	0.0116215
cg15691862	0.8109676	0.3219203	2.042954	0.6566956
cg05663064	2.4829788	0.4295792	14.35168	0.3096217
cg09819651	2.69E-11	1.77E-23	40.86132	0.0889956
cg03454705	15397.01	0.0039383	6.02E+10	0.2131307
cg21618017	0.1932744	0.0325107	1.149007	0.0707247
cg17611936	1.438187	0.5847156	3.537415	0.4287444
cg26860935	0.1819468	0.0388254	0.852656	0.030601
cg18576800	4.8652199	0.5528641	42.81407	0.1539106
cg09218250	2.77828	0.7841879	9.8431	0.1133573
cg05592292	0.1457948	0.0464155	0.457954	0.0009761
cg23216745	0.2238936	0.0919395	0.545232	0.000982
cg17595735	0.6211049	0.2493409	1.547164	0.3064257
cg11726858	0.0972346	3.47E-06	2725.742	0.6555685
cg12283584	51.168117	0.1944564	13464.08	0.1663518
cg05970307	0.9605033	0.1753414	5.261544	0.9629592
cg08716396	2.44E-05	2.11E-18	2.83E+08	0.4889195
cg22172724	2.80556	0.4683218	16.80718	0.2587174
cg11857445	4.65E-16	8.12E-27	2.66E-05	0.0052134
cg19143990	1.1300404	0.4778694	2.67226	0.780704
cg06607825	0.0829221	0.0119192	0.576891	0.0118763
cg03008584	0.1995281	0.0344076	1.157056	0.0722886
cg26818165	1.3001611	0.5406642	3.12656	0.5576576
cg13611006	0.2183921	0.0812748	0.586838	0.0025542
cg23606023	3.2390409	0.9379055	11.18597	0.0630858
cg14186824	11.408678	1.9233921	67.67104	0.0073607
cg00301792	3.3454351	1.3907829	8.04722	0.0070061
cg01161811	2.4956153	1.0290614	6.05221	0.0430377
cg24935598	2.4561154	0.8171033	7.382791	0.1095435
cg19180698	0.2717513	0.1028738	0.717858	0.0085687
cg06422694	0.1341374	0.025687	0.700466	0.0172135
cg14161359	1.4484496	0.6775308	3.096547	0.339211

cg04569608	0.0191326	0.0002957	1.23801	0.0629407
cg07976328	3.1812041	1.0914381	9.272225	0.0339833
cg22145318	13.032279	0.5588405	303.9155	0.1100815
cg12230289	1.8513265	0.9054546	3.785292	0.0914504
cg08025592	7.388047	0.3493958	156.2218	0.1989533
cg04411342	0.0694923	0.0163952	0.294549	0.000296
cg16185419	0.9469452	0.3611067	2.483214	0.9117528
cg17623116	0.5645493	0.2658647	1.19879	0.1367363
cg03368690	2.7767561	0.5331529	14.46184	0.2251416
cg25169784	1.81E-22	7.08E-39	4.62E-06	0.009396
cg24851181	1.9871635	0.9131057	4.324602	0.0834799
cg01715901	1.4259839	0.8093033	2.512569	0.2194973
cg24210813	2.2752665	0.6683759	7.745399	0.1883992
cg17921439	2.9499232	0.3890647	22.36658	0.295271
cg27305525	4.285E+09	0.1297108	1.42E+20	0.0727034
cg24796546	2.3332696	1.2681338	4.293039	0.0064583
cg26551026	0.5575588	0.2849945	1.090799	0.0879822
cg04730665	0.0838227	0.001514	4.640763	0.2260881
cg07484910	1.4759903	0.7012868	3.1065	0.3051733
cg03700024	0.1931947	0.0446882	0.835214	0.0277336
cg20976456	1.3244297	0.6774419	2.589321	0.4113871
cg20045040	0.1870718	0.0676128	0.517592	0.0012453
cg07630649	1.066E+16	0.5643484	2.02E+32	0.0536011
cg24537688	0.4124709	0.157469	1.080417	0.0714619
cg23413809	2.5672081	1.146249	5.749673	0.0219202
cg26494337	0.3524952	0.1245946	0.997257	0.049398
cg03354616	0.6766007	0.3216925	1.423063	0.3030624
cg18470780	1.7613275	0.2527663	12.27329	0.5676654
cg01539269	0.554634	0.0499388	6.159915	0.631319
cg02493211	0.0929869	0.0198724	0.435105	0.0025536
cg23419907	0.1858274	0.0492137	0.701672	0.0130429
cg08976646	0.1186086	0.038137	0.36888	0.0002308
cg17288102	38.931449	0.4851095	3124.362	0.1017041
cg01434611	0.6485187	0.2792565	1.506058	0.3137452
cg21787078	0.1268166	0.0288913	0.556654	0.0062158
cg00332950	0.0565365	0.0091392	0.349742	0.0020024
cg05432213	8.485392	2.3794552	30.25982	0.0009799
cg22679316	3.5661724	0.999782	12.72036	0.0500393
cg04548204	2.7263358	0.8564767	8.678469	0.0895614
cg20782215	0.5454506	0.2495657	1.192136	0.1286569
cg16403326	1.8746464	0.5086977	6.908423	0.3450124
cg26066912	1.4115865	0.395824	5.033996	0.5951675
cg00479912	0.2925273	0.094434	0.90616	0.0331073
cg26434983	2.1969311	0.9347862	5.163219	0.0710305
cg11658159	0.0783996	0.0063283	0.971273	0.0474051
cg16409012	0.392515	0.16462	0.935901	0.0349111
cg26562921	0.3129399	0.1071131	0.91428	0.0336871
cg06761421	3.0662641	0.5415212	17.36216	0.2053008
cg18503793	3.56E-10	3.51E-17	0.003606	0.008207
cg05476287	1.4035373	0.0732702	26.88567	0.8219569
cg06330722	0.1391196	0.0189147	1.023236	0.0526963
cg23608868	0.9212255	0.3974484	2.135262	0.8482892
cg13871900	0.2547622	0.0689692	0.941055	0.040258
cg23256882	0.9350477	0.4611168	1.89608	0.8522954
cg02404377	0.4661282	0.1621612	1.339873	0.156521
cg27622679	4.0995528	1.2500477	13.44455	0.0198981
cg00003287	1.3317284	0.5673353	3.126018	0.5105185
cg17586094	0.1901639	0.0450893	0.802014	0.0237954

cg08888918	0.6235113	0.3046536	1.276093	0.1960939
cg10521153	2.1452184	0.7538132	6.10491	0.152619
cg09778596	2.6287272	0.8978423	7.696459	0.0778403
cg13151498	261.98455	1.09E-05	6.3E+09	0.5207921
cg09026038	0.9880112	0.4651332	2.098681	0.9749677
cg11210138	3.9845838	0.6503948	24.41118	0.1349621
cg25080348	2.3309279	0.1786479	30.41303	0.5184475
cg10542123	0.5538844	0.2703782	1.134662	0.1063791
cg19682013	2.3196429	0.8919414	6.03262	0.0844446
cg25921007	2.3232401	0.4777569	11.29747	0.2962019
cg07218744	0.219195	0.0682334	0.704148	0.0108014
cg17296589	0.3162185	0.1396284	0.716145	0.0057717
cg02108645	1.608985	0.6165588	4.198842	0.3311447
cg11466837	2.4344669	0.9085337	6.52329	0.076857
cg17144548	0.9125327	0.4373858	1.903847	0.8072744
cg03750567	0.1402002	0.0487141	0.4035	0.0002698
cg11569478	0.448	0.2045995	0.98096	0.0446392
cg16331727	0.1336556	0.0291411	0.613012	0.0096062
cg04534404	0.6714207	0.3022351	1.491573	0.3279878
cg24541425	1.0987966	0.4507885	2.678316	0.8358101
cg01442064	0.0382641	0.0010784	1.357739	0.0731295
cg26314722	1.1662571	0.4655191	2.921804	0.7427422
cg06989253	0.2400323	0.0972125	0.592676	0.001973
cg16184112	0.1077857	9.94E-07	11684.02	0.7064778
cg10800464	1.3998853	0.6162237	3.180142	0.421677
cg16041434	0.6427706	0.3014319	1.370638	0.2526493
cg17467898	0.3985346	0.142594	1.113861	0.0793736
cg05461182	2.5519495	0.8492273	7.668673	0.0951481
cg21001273	1.912575	0.8387286	4.361295	0.1231206
cg02563503	1.0368098	0.4942349	2.175028	0.9238164
cg20055581	2.0629983	0.9673026	4.399825	0.060939
cg07017214	0.3041228	0.1145406	0.807493	0.0168883
cg00562504	8.6912903	0.3175279	237.8957	0.2003435
cg08393805	1.5180845	0.6309058	3.652813	0.3514278
cg07381973	2.6760971	0.7960201	8.996627	0.1115656
cg19574297	0.3395572	0.1126914	1.02314	0.0549447
cg23002316	0.7504567	0.3274155	1.720094	0.4975539
cg07520687	21.865972	1.3293326	359.6697	0.0308334
cg09233658	0.4793205	0.1788529	1.284565	0.1437186
cg25734490	0.0407296	0.0013909	1.192681	0.0632119
cg13204687	0.0017291	6.93E-10	4311.861	0.3973735
cg06545143	1.7852558	0.6585026	4.839978	0.2547282
cg07277828	2.7348784	0.570559	13.10918	0.2083169
cg09321109	32.773996	0.1599224	6716.601	0.1987997
cg06855751	0.7062607	0.3443511	1.448534	0.3426703
cg16166160	1.3056616	0.6563143	2.597463	0.4472594
cg02794779	0.062364	0.004939	0.787464	0.0319816
cg12749164	2.0183413	0.6367541	6.397606	0.2328239
cg04346127	7.31E-14	2.03E-32	263750.4	0.165324
cg16941568	2.1154551	0.7288122	6.140333	0.1681648
cg08768385	2.1658494	0.650928	7.206486	0.2076837
cg02166450	3.4015492	1.4337407	8.070174	0.0054809
cg05586466	0.1996422	0.0557468	0.714965	0.0133068
cg10690003	6.882802	1.4078653	33.64879	0.0171982
cg00109551	4.5112852	0.7263263	28.02004	0.1059187
cg09077096	1.8637931	0.8888884	3.907943	0.0993178
cg21859216	0.2435669	0.0668273	0.887733	0.0323196
cg05798012	0.0549072	0.0061974	0.486461	0.0091238

cg09108394	1.7288175	0.8379422	3.566845	0.1384768
cg16675872	2.0186034	0.7713792	5.282434	0.1524018
cg08224682	0.5647737	0.0344499	9.258921	0.6888881
cg15059511	4.75E-08	9.76E-21	230596.5	0.2578648
cg04308589	0.5184447	0.2124954	1.264898	0.1488588
cg26454662	0.219826	0.0756336	0.638915	0.0053874
cg10144604	0.167705	0.0273406	1.02869	0.0536813
cg17403699	2.2133546	1.0281541	4.76479	0.0422613
cg06634862	3.4223773	0.8173529	14.33	0.0921963
cg04093671	0.0184234	0.0007328	0.463162	0.0151904
cg14110086	0.3207388	0.0698623	1.472517	0.1436524
cg02096296	0.4665065	0.2216295	0.981947	0.0446496
cg06451739	0.0126634	0.0004043	0.396654	0.0129134
cg02428792	1.3393078	0.7060375	2.540581	0.3711257
cg15482792	1.2038134	0.4773867	3.035624	0.6942654
cg16044480	3.2519542	0.7486881	14.12498	0.1155531
cg24959965	0.2888188	0.0701399	1.189284	0.0854504
cg06851000	0.0713792	0.0173072	0.294386	0.0002607
cg03128025	2.130411	0.9140158	4.965615	0.0798209
cg11870755	1.7806845	0.2448569	12.94976	0.568688
cg14645264	0.0892559	0.0153964	0.517434	0.0070433
cg09331106	0.3255937	0.0774665	1.368479	0.1255857
cg21138542	2.3388039	0.7726452	7.07958	0.1327045
cg08488945	1.661924	0.7089875	3.895684	0.2425213
cg04464465	6.69E-21	4.10E-35	1.09E-06	0.0053992
cg04824022	0.3690093	0.0838356	1.624224	0.1873398
cg02527496	0.1115802	0.0134542	0.925369	0.0421713
cg14565651	0.5441762	0.2372778	1.248021	0.1507751
cg06703856	0.2489919	0.1152011	0.538163	0.0004069
cg20140110	0.0352646	0.0008285	1.501096	0.0805119
cg21226059	0.3902342	0.0917273	1.660169	0.2027411
cg08186362	0.0566574	0.0067783	0.473578	0.0080514
cg07780074	0.3152079	0.1242983	0.799335	0.0150277
cg19619028	0.4358461	0.2056365	0.923775	0.0302472
cg14642696	1.4848972	0.7225015	3.051786	0.2820933
cg00655552	2.0779094	1.1484052	3.759742	0.0156351
cg09498572	1.6137623	0.7867998	3.3099	0.1916416
cg25390440	0.451203	0.1729387	1.177205	0.1038363
cg20302637	0.5990295	0.2959977	1.212294	0.1542367
cg02588309	0.0824259	0.0130404	0.521	0.0079773
cg01944110	0.7912695	0.2791598	2.242828	0.6596283
cg08748184	0.1928989	0.0212746	1.74903	0.1434808
cg19711268	0.6270239	0.0976384	4.026684	0.6227671
cg12730381	60900.146	3.77E-12	9.83E+20	0.5628739
cg27080171	0.5195291	0.2283388	1.182062	0.1184773
cg13993764	0.0470273	0.0045431	0.486796	0.0103563
cg00267746	2.7150606	0.8689866	8.482932	0.085729
cg08069147	137.71884	0.8358797	22690.44	0.058607
cg18151030	0.500962	0.2456666	1.021559	0.0572637
cg02137183	1.4110455	0.5242811	3.797675	0.4954583
cg03322338	1.2713509	0.4812426	3.358666	0.6281231
cg03041841	0.1114788	0.0081444	1.525903	0.1002972
cg10530793	3.4581733	0.6010468	19.89689	0.1646065
cg13141065	0.2449449	0.0020746	28.92066	0.5633596
cg11337945	0.903289	0.4750044	1.717733	0.75643
cg03135127	27273.098	5.1406952	1.45E+08	0.0195901
cg09978395	0.9178102	0.2103227	4.005157	0.9091657
cg06046369	1.24E-06	3.81E-11	0.04016	0.0102715

cg09737078	0.3282794	0.1431967	0.752583	0.0085017
cg16649771	0.0191124	0.0001228	2.974113	0.1243613
cg06667761	1.9113367	0.768292	4.754973	0.1635845
cg08034990	8.0165397	0.8951429	71.7929	0.062754
cg22048590	1.4859738	0.3089096	7.148104	0.6211624
cg13694680	0.7646103	0.3195247	1.829683	0.5465879
cg20133923	0.0098139	0.0001649	0.584031	0.0265599
cg00401745	2.2556691	1.1658781	4.36413	0.0157029
cg13205578	0.8984498	0.3940875	2.048307	0.7989698
cg13788625	0.280607	0.1052453	0.74816	0.0110903
cg15536845	1.7326633	0.7735159	3.881138	0.1816016
cg12569217	0.0808136	2.61E-05	250.218	0.5396093
cg14786713	0.1901453	0.0241676	1.496021	0.1147422
cg02566775	0.6393799	0.2665207	1.533865	0.3164487
cg05422096	0.5732403	0.2741086	1.198811	0.1393416
cg11592503	0.3062898	0.094043	0.997559	0.0495277
cg22429169	0.174411	0.0508383	0.598352	0.0054949
cg14375890	2.0499121	0.673914	6.235424	0.2059979
cg01914231	1.9246956	0.8177103	4.530276	0.1338267
cg26400835	0.4809326	0.234578	0.98601	0.0456704
cg09399716	0.1672568	0.0701567	0.398748	5.48E-05
cg14138611	0.5657261	0.2829221	1.131216	0.1071293
cg05819594	0.1679157	0.0448812	0.628229	0.0080378
cg14708940	1.2854921	0.4649924	3.5538	0.6283435
cg07797367	1.7033673	0.5383275	5.389767	0.3648101
cg02629976	1.8242033	0.4618193	7.205671	0.391068
cg26040816	0.0231166	0.0016391	0.326014	0.0052698
cg02407342	1.6159299	0.6498015	4.018503	0.3018374
cg26757673	5.4206018	1.3533266	21.71163	0.0169717
cg00972761	1.6724964	0.7434034	3.762754	0.2137873
cg03232620	2.4198076	0.970195	6.035352	0.0580824
cg22828707	0.6455392	0.3095262	1.346319	0.2431991
cg25390334	14.378416	0.656271	315.0205	0.0905422
cg02699834	1.7539373	0.7330051	4.196828	0.2068747
cg22610655	1.5177356	0.8384619	2.747318	0.1681917
cg24863152	1.7440122	0.5410615	5.621502	0.3516518
cg06424138	2.6153356	0.5254066	13.01845	0.2403817
cg05884115	0.0076392	1.19E-12	48895373	0.6722123
cg06355908	0.8997373	0.4140661	1.955068	0.7896058
cg14848077	0.0534876	0.0021725	1.316863	0.0732031
cg13751386	0.1296371	0.0285229	0.589204	0.0081752
cg19424261	1.7109458	0.6782249	4.316173	0.2553127
cg27289662	3.2197892	1.4625287	7.088437	0.0036824
cg04014686	0.5220341	0.242374	1.124376	0.0968138
cg06496470	0.3263078	0.1098105	0.969641	0.0438579
cg05425936	0.7567124	0.3544254	1.615611	0.4713029
cg08169827	2.4081307	0.9000714	6.442926	0.0800684
cg14398929	0.6005612	0.2821338	1.278378	0.1858955
cg03058346	3.226303	0.8757667	11.88562	0.0783101
cg11376341	0.0224138	0.0012299	0.40847	0.0103323
cg15059558	3.0758437	1.464558	6.459843	0.0029995
cg22678436	0.3603701	0.1473067	0.881607	0.0253498
cg15478005	5178.0913	33.28902	805449.7	0.0008963
cg14532497	2.0967131	0.5637347	7.79836	0.2692797
cg07921759	2.2806893	0.66619	7.807898	0.1891576
cg20874785	0.5220605	0.1963706	1.387923	0.1926188
cg26953288	0.167159	0.0482308	0.579342	0.0047917
cg25521338	0.0950325	0.0010818	8.348335	0.3026975

cg27216788	0.2680084	0.0655538	1.095719	0.0668425
cg22700854	0.2225741	0.0722204	0.685945	0.0088867
cg16556397	2.8150057	0.9792231	8.092392	0.0547325
cg16646298	1.3653888	0.4651751	4.007709	0.5707931
cg23375552	0.3274905	0.0907244	1.182152	0.0882951
cg04691908	1.7233122	0.8624256	3.443549	0.123337
cg15928392	1.0214699	0.4172885	2.50043	0.9629054
cg19787532	1.9215226	1.024629	3.603498	0.0417701
cg27107150	0.4341747	0.1646096	1.145181	0.0917934
cg06443114	2.4450027	0.8705744	6.866775	0.089716
cg14164908	1.7607887	0.6642293	4.667631	0.2553569
cg02317313	0.2705802	0.0917302	0.798141	0.0178608
cg11480029	0.8467886	0.4243249	1.689863	0.6371118
cg06453088	1.33398	0.6438978	2.763642	0.4380959
cg10880485	3.2612647	1.3986857	7.604173	0.0062045
cg05234035	2.0549506	0.8577285	4.923261	0.1061594
cg26816636	0.1881737	0.0753862	0.469706	0.0003448
cg02605237	0.0709571	0.0039419	1.277285	0.0728115
cg15513743	4.2953848	1.4424582	12.79089	0.008845
cg03560652	2.05835	0.5272837	8.035152	0.2988488
cg26219843	3.0935176	0.6090064	15.71388	0.1732301
cg02522367	3.1814495	0.6974319	14.5127	0.1350183
cg01589461	0.2364342	0.0724153	0.771951	0.016908
cg25670330	1.5515522	0.8554616	2.814053	0.1481675
cg25279922	1.6567141	0.6553298	4.188275	0.2860359
cg06640206	0.6724346	0.3689836	1.225443	0.1949672
cg04569804	445.53804	0.1986855	999087.1	0.1212773
cg00182937	0.3385463	7.92E-05	1447.703	0.7995717
cg00693994	0.4971524	0.2219539	1.113567	0.0894082
cg07732421	2640941	0.0254055	2.75E+14	0.1164154
cg18349298	0.3658355	0.1617194	0.827579	0.0157633
cg17393458	0.1150965	0.0256756	0.515945	0.0047353
cg23912186	3.659271	0.8001348	16.73501	0.094427
cg07078329	0.4524848	0.2185645	0.93676	0.0326857
cg24367316	3291221.8	0.8192869	1.32E+13	0.0530796
cg22982767	1.4222745	0.7638341	2.648304	0.2667448
cg22006060	0.7059225	0.3428577	1.453451	0.3445972
cg14178899	1.3831775	0.3406502	5.616259	0.6500356
cg17733447	1.7443964	0.8187779	3.716415	0.1493462
cg26217572	0.4748813	0.1753241	1.286259	0.1429761
cg11273702	0.509777	0.2356444	1.102817	0.0870104
cg07584669	3.0015241	1.2170953	7.402171	0.0170068
cg01569346	0.1964366	0.0542626	0.711122	0.0131627
cg04243581	0.5056364	0.2476148	1.032524	0.061193
cg00055771	0.4196283	0.1896252	0.92861	0.0321356
cg13464117	0.3371517	0.120815	0.940871	0.0378603
cg18127012	0.9454684	0.2869225	3.115512	0.9265664
cg05497652	0.0589731	0.0114555	0.303596	0.0007097
cg19141913	0.2013426	0.0393414	1.030436	0.054358
cg20911989	9.0289984	1.2552777	64.94405	0.0288296
cg12070159	1.5447388	0.5934353	4.021025	0.3729874
cg04610718	1.7761688	0.8832297	3.571863	0.1070464
cg27558387	0.2790361	0.0448151	1.737387	0.1713237
cg14264079	152.09098	3.1586294	7323.324	0.0110283
cg11077161	0.1410834	0.0131667	1.511732	0.1055667
cg21856334	0.2791475	0.1079356	0.721943	0.0084883
cg07973479	2.6541222	0.7786679	9.046687	0.1187321
cg24690054	1.2948491	0.6687209	2.507226	0.4434208

cg00741609	2.0325033	0.9944358	4.154184	0.0518152
cg22801992	1.6790481	0.8624013	3.269015	0.1273874
cg00147244	1.424874	0.5963689	3.404379	0.4255709
cg11146208	0.6499042	0.3213453	1.314397	0.2304496
cg13337716	2.48866	0.9422746	6.572849	0.0657724
cg21871091	1.4124748	0.6342835	3.145415	0.3978659
cg10296205	0.2733969	0.1394615	0.53596	0.0001594
cg00197728	2.910102	1.2450573	6.80185	0.0136649
cg06705017	2.5131786	0.8327411	7.58467	0.1020088
cg01084215	0.3890589	0.1694484	0.893292	0.0260108
cg05864261	1.5159903	0.442709	5.19128	0.5076502
cg17336577	1.24E-05	2.96E-15	52112.13	0.3176691
cg08547097	8.9508657	0.427262	187.5149	0.1579214
cg25193885	2.122531	0.7107772	6.338327	0.1775504
cg00525823	1.1215217	0.5221105	2.40909	0.768758
cg23415712	227.72775	1.99E-16	2.60E+20	0.7980503
cg15497006	1.5296003	0.5204062	4.495867	0.4397498
cg10925082	1.4333139	0.7454503	2.755903	0.2804765
cg13681800	2.564943	0.828868	7.93725	0.1021948
cg03727192	1.7955441	0.6958265	4.633308	0.2262192
cg16661579	0.3263506	3.81E-07	279727	0.8723669
cg01740650	6.5757341	0.6066799	71.27363	0.1213933
cg16725050	0.966777	0.466616	2.003056	0.9275665
cg18153624	6.89E-07	2.87E-13	1.656795	0.0584094
cg15379170	0.1033386	0.026042	0.410062	0.0012483
cg23730408	3.9701219	1.1974117	13.16328	0.024161
cg19694878	1.4261584	0.5406014	3.762343	0.4732308
cg10665379	0.0273279	0.0032792	0.227745	0.000876
cg17269733	1.2126586	0.4203318	3.498524	0.721332
cg26579616	2.0912145	0.8784621	4.97822	0.0954872
cg02557174	0.6811292	0.3247275	1.428697	0.30962
cg20869844	3.1304704	0.996797	9.831335	0.0506457
cg08932256	0.5606331	0.2650146	1.186008	0.1300949
cg13706365	0.0050016	0.0002964	0.084386	0.000238
cg17633463	0.2745595	0.0992481	0.75954	0.0127836
cg13692869	0.1021472	7.16E-13	1.46E+10	0.8617965
cg01305291	0.5844212	0.1734023	1.969686	0.3862351
cg13908336	3.4847832	1.1979861	10.13677	0.0219318
cg02016985	0.2377281	0.1009744	0.559692	0.0010075
cg12093241	0.2772853	0.1380788	0.556835	0.0003112
cg12229387	1.2679105	0.4524439	3.553141	0.6516407
cg01418618	1.8208989	0.573565	5.780815	0.3092305
cg23685580	0.0019144	2.50E-10	14652.65	0.4390178
cg27156584	0.714306	0.357112	1.428776	0.3415126
cg18606375	2.8310843	1.3118222	6.109851	0.0080132
cg21242356	1.4819424	0.4783532	4.59107	0.4953614
cg15029935	0.4945321	0.2059042	1.187747	0.1152358
cg03427058	5.4112263	1.3159503	22.25112	0.019255
cg20004910	0.1178301	0.0228884	0.606593	0.0105307
cg15963095	0.5874337	0.2386915	1.445709	0.2469547
cg15252951	0.0007805	3.92E-11	15528.97	0.4039973
cg08737246	0.6024619	0.2495904	1.454224	0.2597147
cg04109898	1.8959971	0.8409143	4.274877	0.1230094
cg24953213	7.1231696	1.4415029	35.19906	0.0160149
cg26443646	0.3630296	0.1528122	0.862435	0.021722
cg03377610	3.7703913	1.4171548	10.03126	0.0078535
cg23837220	0.2805929	0.0912347	0.862965	0.0266177
cg14396035	0.0573131	0.0068738	0.477874	0.0082328

cg14128735	3.5286633	1.2889794	9.659941	0.0141273
cg01718733	6.2305094	0.9212531	42.13744	0.0606741
cg06969739	7.2798171	0.6975068	75.97881	0.0971328
cg10867751	0.4110121	0.0721796	2.340424	0.3164202
cg21177821	2.6780073	0.7854677	9.130513	0.1154649
cg13192836	0.6257676	0.3210448	1.219721	0.1686155
cg20651084	1.802E+15	2.42E-16	1.34E+46	0.3327717
cg14582763	0.3862122	0.1246331	1.196792	0.0992187
cg04755345	0.5517228	0.2707904	1.12411	0.1014676
cg18017926	1.4386399	0.6889628	3.004059	0.3329564
cg16898193	1.6628991	0.7717726	3.582964	0.1941158
cg09326135	0.4075655	0.1852513	0.896672	0.0256765
cg01373166	2.6420189	0.7874683	8.864185	0.1156974
cg25996119	0.9573759	0.5232428	1.751708	0.8876226
cg18646365	1.7732601	0.7737937	4.063681	0.1757845
cg08453194	0.1402774	0.0137195	1.434287	0.0977427
cg12614148	0.4277148	0.1757439	1.040946	0.0612713
cg12079975	0.3890945	0.168745	0.897179	0.0267938
cg03311274	0.1137517	0.0160223	0.807588	0.0297308
cg02192965	1.0477501	0.3468455	3.165041	0.9340929
cg10625666	0.4347205	0.1954051	0.967129	0.0411626
cg21846949	0.443841	0.2152	0.915404	0.0278579
cg22737478	0.234591	0.0872646	0.630645	0.0040573
cg10473059	0.4643907	0.2280427	0.945694	0.0345289
cg18009127	0.05405	0.0030298	0.964217	0.0471723
cg21555696	0.2801371	0.0918085	0.854788	0.0253762
cg26547506	0.1989045	0.0546995	0.72328	0.0142143
cg00668150	0.2905565	0.0879904	0.959458	0.0425736
cg06950683	1.5846953	0.777835	3.228524	0.2047973
cg21178689	0.1254976	0.044469	0.354171	8.82E-05
cg25712987	4.2560624	0.9705218	18.66425	0.0548209
cg21513826	1.512654	0.5083487	4.501088	0.4569516
cg20697464	2.0644864	0.527796	8.075287	0.2975716
cg08902766	0.1026646	0.0063157	1.668851	0.1096014
cg11584277	1.5541493	0.7191655	3.358587	0.2620836
cg06855485	0.3816879	0.1416357	1.028594	0.056881
cg00035980	3.7042641	0.6904034	19.87472	0.1265779
cg02279147	0.3409119	0.1433281	0.810873	0.014926
cg24519157	6.021091	0.8819271	41.10718	0.0669866
cg18259535	4.411473	0.4191455	46.43041	0.2164956
cg09745430	1.0281325	0.5014445	2.108023	0.9396308
cg27333952	0.7909688	0.1649064	3.79386	0.7694167
cg15688324	0.5749874	0.2650222	1.247483	0.1613937
cg07537181	2.1113399	0.8292434	5.375691	0.1170488
cg16254746	1.4436739	0.576637	3.614396	0.432926
cg25004981	2029.4259	1.9824437	2077521	0.0312813
cg23497683	2.5516145	1.2367543	5.264374	0.0112442
cg16227623	2.0251234	0.8300507	4.940812	0.120989
cg04391205	0.3590204	0.1562891	0.824726	0.0157741
cg09558425	0.5194341	0.1869878	1.442938	0.2089189
cg04931256	1.6428048	0.6431082	4.196506	0.2995429
cg23528791	0.126496	0.0284458	0.562518	0.0066147
cg27651218	1.972913	0.70705	5.505107	0.1943359
cg14989351	0.7736816	0.4085497	1.465142	0.4309332
cg16691714	1.4322052	0.4260313	4.814697	0.5614566
cg02442436	0.1500423	0.0232464	0.968438	0.0461879
cg18976046	1.9580973	0.7767886	4.935892	0.1542995
cg19730691	5.7874182	1.4290253	23.4385	0.0138855

cg07648498	2.863E+19	2.97E-19	2.76E+57	0.3153892
cg15142890	0.95639	0.3120734	2.930983	0.9378001
cg00554437	2.7380967	0.3771105	19.88058	0.3193361
cg08105834	1.7543345	0.9951337	3.09274	0.052004
cg18094261	2.4510809	0.4597616	13.0672	0.2937429
cg04689058	1.3081097	0.4946672	3.459196	0.5882836
cg08206156	3.985524	0.7417374	21.41513	0.1070244
cg09211372	3.4844426	1.0972376	11.06537	0.0342291
cg04093149	0.3000303	0.0908573	0.990765	0.048247
cg04865531	0.5679562	0.2687552	1.200253	0.1383843
cg15456821	0.2918179	0.0768502	1.108099	0.0704226
cg14372466	5.13E-12	1.96E-23	1.346718	0.0526507
cg08811309	1.4059403	0.8049968	2.455498	0.231099
cg17301379	0.9012353	0.3035329	2.675904	0.8514395
cg18970250	0.0502149	0.0015084	1.671688	0.0943951
cg12495975	2.0381631	0.962324	4.316747	0.0629331
cg23923495	0.0171354	3.77E-05	7.792351	0.1927772
cg21712331	0.241452	0.1008226	0.578235	0.001426
cg11905007	0.0367266	0.0004654	2.898493	0.1382074
cg22253393	0.7035255	0.3259296	1.518574	0.3703771
cg04745805	1.2213242	0.532172	2.802915	0.6371288
cg00667938	3.52E-05	3.89E-08	0.031803	0.0031495
cg09869291	0.6570675	0.2980226	1.448674	0.2978224
cg09754110	2.3348496	0.6781642	8.038647	0.1788591
cg17973038	0.148283	0.0511698	0.429704	0.0004382
cg06436854	0.1914233	0.0500283	0.732443	0.0157461
cg12216208	0.2153144	0.0742677	0.624232	0.0046889
cg01380194	0.1521955	0.0429716	0.539042	0.0035262
cg01661307	0.0019444	1.14E-05	0.330326	0.0171843
cg21517865	2.42E-22	1.07E-46	550.4576	0.0819613
cg17541483	0.3701849	0.1428067	0.959597	0.0408719
cg19622474	3.2886447	0.8792255	12.30081	0.076939
cg19597450	15592.296	0.0021348	1.14E+11	0.2311776
cg00835193	0.301745	0.1275376	0.713907	0.0063921
cg15123727	1.9322484	0.8959376	4.167236	0.0930075
cg22878627	1.4065622	0.8006929	2.470881	0.2353313
cg19300124	0.2859146	0.0380437	2.148772	0.2237254
cg12131828	1.7567278	0.8927088	3.456998	0.1028138
cg04233686	0.270603	0.0568179	1.288782	0.1007176
cg23092449	1.7389863	0.8122921	3.722889	0.1542537
cg14252395	6.1531353	0.3089674	122.5407	0.2338734
cg06900257	0.098393	0.0272041	0.355872	0.0004076
cg20045155	0.4220846	0.186041	0.957614	0.0390574
cg23698269	0.8715067	0.443626	1.712082	0.6897451
cg01860763	0.5680962	0.2645515	1.219926	0.1470146
cg12617332	2.8108713	0.8517809	9.275856	0.0897713
cg14213394	2.9725115	0.7349256	12.02275	0.1265157
cg02571534	0.1910045	0.0555744	0.656467	0.0085852
cg24632582	0.0578985	0.0117294	0.285797	0.0004697
cg13488811	0.2818725	0.1321766	0.601106	0.0010483
cg15972148	0.470212	0.1924467	1.148886	0.0978302
cg25461801	4.2326398	1.0665218	16.79782	0.0402146
cg09427605	0.3270702	0.0389033	2.749763	0.3035728
cg17775765	2.0247874	0.5749966	7.130067	0.2720444
cg24358846	5.3413027	1.123896	25.38448	0.0351317
cg05336997	1.1979576	0.4015732	3.573701	0.7460223
cg16332631	0.355911	0.04833	2.620994	0.310533
cg12491805	3.1704028	1.0379313	9.684122	0.0428354

cg11333576	0.7777262	0.3619217	1.67124	0.5195148
cg05950755	0.3911852	0.1874479	0.816365	0.0124017
cg23054119	0.6655279	0.3287032	1.347499	0.2579287
cg10256249	0.0603109	0.0072062	0.504762	0.0095791
cg27097846	0.257321	0.0905257	0.731439	0.0108746
cg26164488	1.9599722	0.8764192	4.383166	0.101269
cg01149239	0.4959215	0.2134995	1.151938	0.1028859
cg16827257	3.3954246	0.8705277	13.24359	0.078357
cg12054554	3.8917365	0.1715726	88.27522	0.3935564
cg07434438	0.3218568	0.1244163	0.832622	0.0194034
cg25518365	0.3150601	0.1278147	0.776616	0.012101
cg01413054	1.3167348	0.7038851	2.463173	0.3891907
cg02408532	3.0312431	0.9736623	9.436983	0.0556329
cg01255894	1.1670095	0.5459549	2.494549	0.6902813
cg22550679	0.7932439	0.2197672	2.863194	0.7235755
cg22628281	1.7878591	0.9368715	3.411823	0.0780373
cg19617373	0.5482813	0.1108938	2.710813	0.4611267
cg25416230	0.0320207	1.15E-05	89.33473	0.3952362
cg14855367	0.409076	0.1228604	1.362059	0.1452617
cg05421651	0.5581081	0.2155344	1.445174	0.2295939
cg03233793	0.1827159	0.0522248	0.639258	0.007809
cg13462909	0.6899193	0.3687239	1.290908	0.2455747
cg15220055	0.405067	0.1710629	0.959175	0.0399045
cg12057576	1.7387021	0.6434962	4.697907	0.2754047
cg14595083	1.549E+12	2.82E-06	8.51E+29	0.1780403
cg03006953	1.6677013	0.568849	4.88922	0.3513512
cg22993667	0.3525164	0.1357587	0.915358	0.0322241
cg01532600	40.387646	1.45E-12	1.13E+15	0.8148816
cg18202741	1.2396428	0.3819902	4.022916	0.7205886
cg23750514	2.8861936	1.1258753	7.398789	0.0273274
cg03685886	1.5274668	0.7893161	2.95592	0.208539
cg06442148	2.1414846	0.6464746	7.093792	0.212718
cg04238038	1.5479344	0.6773628	3.537396	0.3001301
cg04074033	0.4026868	0.1682961	0.96352	0.0410079
cg01761662	0.4028176	0.0111856	14.50634	0.6189997
cg07696006	3.6030169	0.8398552	15.45711	0.0845135
cg13106758	0.3956255	0.1021824	1.531766	0.179411
cg09442403	0.3180599	0.1029143	0.982974	0.0466137
cg26715559	3.4903654	1.1450249	10.63964	0.0279414
cg18795169	5.3454511	1.111063	25.71758	0.0364956
cg09183138	0.3090089	0.1489999	0.64085	0.0016018
cg17997279	1.6549687	0.8277066	3.309049	0.1541394
cg02311794	0.2186657	0.0475708	1.005128	0.0507733
cg27107076	0.1407706	0.036136	0.548382	0.004715
cg15861156	0.5549779	0.2057289	1.497118	0.2448482
cg24386135	0.4103431	0.1926061	0.874227	0.0209835
cg19327213	3.5541746	0.7194616	17.55779	0.1197141
cg27598761	0.1400277	0.0573367	0.341976	1.59E-05
cg03406609	1.5775118	0.4273489	5.823213	0.493907
cg01157004	0.6302526	0.121361	3.273031	0.5828424
cg22538780	1.7587553	0.8220355	3.762879	0.1456813
cg21459486	0.5339403	0.240664	1.184607	0.1227602
cg25318211	0.4876471	0.2298565	1.034557	0.0612851
cg19242448	2.498E+15	0.1012056	6.17E+31	0.0656185
cg10455971	2.1281324	0.8776884	5.160086	0.0946696
cg04075986	1.7097809	0.76267	3.833048	0.1928494
cg16328456	0.3750519	0.1897372	0.741362	0.0047914
cg03515869	0.0001393	1.72E-10	112.8814	0.2008718

cg06451900	3.4743852	0.9741451	12.39174	0.0549098
cg18481241	3.1069911	0.7876729	12.25559	0.1054277
cg26675485	0.4671515	0.2095703	1.041324	0.0627504
cg01140244	1.443723	0.5497125	3.791684	0.4560293
cg19237691	1.4608093	0.6852424	3.114174	0.3264513
cg01135464	0.373155	0.1437155	0.968891	0.0428796
cg09795027	2.0326326	0.6900148	5.987691	0.1981513
cg17386213	1.6192903	0.7223754	3.629832	0.2418729
cg25814293	0.4128031	0.1773922	0.96062	0.0400536
cg18346182	0.0160702	0.0003351	0.770664	0.0364487
cg08453008	0.3738091	0.1728833	0.808252	0.0123828
cg14565014	1.8759167	0.3498422	10.059	0.4628216
cg23322933	2.4661381	0.985301	6.172567	0.0538141
cg21907389	0.4505765	0.1929423	1.052228	0.0654288
cg06515745	1.6333367	0.6864964	3.886093	0.2672565
cg04416111	1.5030827	0.7307524	3.091687	0.2680819
cg02674305	133.97936	4.1656	4309.216	0.0056799
cg04011474	1.0856309	0.3636845	3.240706	0.8829375
cg13917578	2.335828	0.6579511	8.29255	0.1893936
cg27458485	1.4385995	0.699281	2.959566	0.3231091
cg20367304	3.2745314	0.6579954	16.29579	0.1474067
cg16366686	0.1245341	0.0160952	0.963564	0.0459864
cg11021940	1.9410962	0.5032035	7.487735	0.3355889
cg03915752	1.2313849	0.6633912	2.285693	0.5095483
cg22489883	1.5098997	0.4852348	4.698338	0.4768184
cg26964875	0.9367319	0.4282849	2.048792	0.8699808
cg22967542	1.7034348	0.7620414	3.807785	0.194349
cg18869404	3.2388056	1.1693537	8.970649	0.0237619
cg02016178	4.5701839	1.4755183	14.15542	0.0084294
cg16016640	1.5412263	0.6627838	3.583941	0.3150488
cg12250496	0.0128139	0.0005227	0.314124	0.0075993
cg19130824	0.4476497	0.1970301	1.017054	0.0549114
cg12072972	0.4269667	0.1987639	0.917171	0.029139
cg03206445	0.2681729	0.0804096	0.89438	0.0322257
cg24988789	0.0750534	0.000273	20.63595	0.3661806
cg03676624	0.424166	0.2002332	0.898536	0.0251354
cg07505695	5633785.9	27.000399	1.18E+12	0.0128696
cg15375772	0.4911375	0.1813969	1.329769	0.1617708
cg06495586	1.3372309	0.5618891	3.182455	0.5112444
cg04891836	1.1236388	0.4264198	2.960848	0.8135796
cg01348584	2.1235657	1.0238082	4.404664	0.0430552
cg19062258	0.7843776	0.0746466	8.242149	0.8396253
cg22223402	0.7244481	0.3490362	1.503641	0.3869397
cg09748975	5.2725909	1.0359865	26.83453	0.0452257
cg17314244	5.1314762	1.3417562	19.62506	0.0168712
cg03526702	0.1335119	0.035789	0.49807	0.002721
cg20585659	4.588079	0.7751816	27.15553	0.0931009
cg08743392	0.0077301	2.12E-05	2.815898	0.1061106
cg22698744	0.4403868	0.2303111	0.842081	0.0131508
cg20253172	2.660322	0.7919275	8.936819	0.1135064
cg00217306	0.7466564	0.2351145	2.371167	0.6202244
cg04195863	0.5568935	0.1655579	1.873244	0.3442418
cg10634136	1.8249016	0.6882646	4.838642	0.2266377
cg08826840	2.2985878	0.902685	5.853101	0.0809369
cg18969411	32518.595	45.613282	23183138	0.0019371
cg06511274	0.2953308	0.1391621	0.626753	0.0014885
cg22273355	20.286699	1.7971925	228.9961	0.0149323
cg19500393	1.57E-05	4.23E-08	0.005852	0.0002499

cg21813473	4.781E+14	5.4683547	4.18E+28	0.0390467
cg09636079	18.109388	0.7651998	428.5808	0.0727831
cg21806750	1.7425186	0.313457	9.686724	0.5257573
cg03585389	0.505311	0.2368223	1.078189	0.0775183
cg22043936	3.3073169	0.6006155	18.21189	0.1693639
cg25402112	0.0023359	5.93E-13	9208510	0.5909196
cg02737621	0.0359306	0.0041003	0.314858	0.0026691
cg14256968	0.3845115	0.1804926	0.819142	0.0132501
cg11503875	0.5347112	0.1698708	1.683138	0.284605
cg27511957	3.206769	0.1203554	85.4417	0.4865803
cg13525835	0.4656924	0.1816283	1.194029	0.1116486
cg22461649	2.2419953	0.7175884	7.004772	0.1648262
cg11200414	0.3609214	0.1443411	0.902475	0.0293011
cg20325547	0.3306116	0.1433767	0.762356	0.0094174
cg02799448	1.9383961	0.7240718	5.189236	0.187723
cg19084127	0.2965023	0.1125443	0.781147	0.0139054
cg10496762	0.400733	0.0720669	2.228304	0.2961843
cg24010336	0.5739807	0.2353514	1.399838	0.2222764
cg27041868	0.1616005	0.0273476	0.954916	0.0443403
cg27131958	7.28E-08	1.51E-13	0.03507	0.0138238
cg00315239	0.2539506	0.1070197	0.602608	0.0018788
cg23638640	6.6638274	1.2798072	34.69788	0.0242577
cg15193596	4.11E-14	5.59E-26	0.030255	0.0270437
cg04021706	6.9496864	1.0816378	44.65278	0.0410873
cg04037044	2.3185632	0.348902	15.40758	0.3841493
cg24847541	0.0847118	0.0124285	0.577389	0.0117072
cg10644206	1.6539983	0.6550461	4.176363	0.2869766
cg13354523	0.2052042	0.0760163	0.553944	0.0017733
cg21172377	1.2908626	0.603597	2.76066	0.5103565
cg26992600	2.2889531	0.976679	5.364409	0.0566943
cg14414464	0.0837439	0.0101413	0.691533	0.0213129
cg03112145	0.2985909	0.0805471	1.106887	0.0705982
cg24671886	0.6255032	0.3100394	1.26195	0.1901088
cg09871057	1.586616	0.4872599	5.16634	0.4434665
cg02486161	0.4096291	0.1709149	0.981752	0.0453644
cg16087267	47.888969	0.8900626	2576.62	0.0570812
cg08141956	0.5077313	0.2193102	1.175464	0.1135314
cg25713684	1.827E+09	2672.0104	1.25E+15	0.0018642
cg11348713	142.58377	1.5964165	12734.85	0.0304604
cg09916174	0.1730809	0.04286	0.69895	0.0137817
cg22938061	1.334527	0.3473431	5.127387	0.6743377
cg10210397	0.6699858	0.3606496	1.244646	0.2050126
cg15241633	2.1702874	1.2223389	3.853389	0.0081599
cg15491385	2.8027313	0.2289809	34.30549	0.4199826
cg08869700	2.0572637	0.6273963	6.74587	0.2338202
cg19258868	0.0196598	0.0003223	1.199335	0.0610265
cg12406822	0.1871068	0.0574564	0.609314	0.0053959
cg22473973	0.0168048	0.0010227	0.276125	0.0042225
cg10837685	7.55E-07	1.38E-14	41.42231	0.1210456
cg20078054	12.504762	0.9537162	163.9577	0.0543706
cg04918504	1.4752673	0.8583293	2.535639	0.1593889
cg00966357	0.2048172	0.0490308	0.855586	0.0297213
cg04269351	0.3366884	0.0723143	1.567588	0.1653985
cg09651654	0.4199919	0.136321	1.293955	0.1307669
cg05628390	2.7098353	0.5654834	12.98572	0.2124297
cg18641937	1.1516268	0.2440992	5.433218	0.8584407
cg08447387	0.3821606	0.111893	1.305236	0.1248078
cg11749672	0.5881395	0.1132611	3.054077	0.5276814

cg17513592	2.6563232	0.6946345	10.15794	0.1534252
cg02013018	0.0087355	0.0001778	0.429252	0.0170529
cg23839074	1.1041976	0.499934	2.438827	0.8063275
cg09786593	0.157233	0.0421944	0.585913	0.0058428
cg22431093	0.4141634	0.16282	1.053503	0.0642349
cg22090404	1.246516	0.094262	16.48386	0.8671616
cg22272713	0.254054	0.103343	0.624556	0.0028299
cg08185798	0.5795937	0.2954775	1.136902	0.1125792
cg22631938	3.1754375	1.0699407	9.424264	0.037364
cg22364906	3.3839217	0.5609084	20.41497	0.1837117
cg00761242	0.2901752	0.1149854	0.732281	0.0088006
cg25300805	1437.5646	24.491523	84379.89	0.0004665
cg01160766	1.631E+15	67.80794	3.92E+28	0.0258675
cg19005210	2.7061621	0.4976243	14.71655	0.2492334
cg16831573	0.174692	0.0394057	0.77444	0.0216528
cg05129572	0.1742336	0.0469271	0.646904	0.0090349
cg25212262	1.50E+34	2.9560646	7.58E+67	0.0468851
cg06889607	0.0404217	0.0014688	1.112394	0.0578297
cg03212131	1.8407267	0.6033332	5.615926	0.283666
cg07346187	0.237736	0.0908249	0.622278	0.0034312
cg26886708	1.0977228	0.5583639	2.158082	0.7869013
cg20437276	0.0120467	3.28E-05	4.421832	0.1424859
cg00670339	10001.334	0.0651547	1.54E+09	0.1306042
cg10187559	0.3460191	0.1633554	0.732937	0.0055835
cg03699601	1.7263877	0.5017688	5.939817	0.386432
cg23076476	0.0698741	0.0096731	0.504738	0.0083478
cg09406615	0.5012814	0.1631566	1.540134	0.2278713
cg24160331	0.9731896	0.3859461	2.453965	0.9540746
cg03136781	0.0148485	0.0005892	0.374169	0.0105558
cg24035352	3.72E-15	9.79E-38	1.41E+08	0.2103926
cg10851763	1.1948029	0.6086199	2.345559	0.6050544
cg06258971	0.0238555	0.0007059	0.806161	0.0375319
cg08883223	1.7715016	0.9682396	3.241158	0.0635608
cg18285544	7.93E-10	1.17E-20	53.56678	0.0995457
cg00013618	0.5894353	0.2265522	1.533571	0.2785928
cg02339243	0.416678	0.166368	1.043594	0.0616405
cg11012965	1.5577999	0.64117	3.784863	0.3277418
cg06021865	2.25E-09	2.74E-18	1.846454	0.0572489
cg10230466	2.74E+26	10482946	7.15E+45	0.0076164
cg12467226	1.5440936	0.4866699	4.89906	0.46084
cg03450829	0.3214569	0.1145155	0.902363	0.0311575
cg00598204	0.629468	0.276155	1.43481	0.2708434
cg21774281	40.614265	0.4902197	3364.856	0.100253
cg18208185	0.5300917	0.2361238	1.190042	0.1239796
cg05284742	0.2316604	0.0666753	0.804893	0.0213616
cg09588284	3.306493	0.4184847	26.12496	0.2568116
cg00329656	11.444871	1.6412437	79.80842	0.0138945
cg07014438	2.0799053	0.8866576	4.879004	0.0922916
cg01566552	0.5233304	0.2379901	1.150782	0.1072579
cg26465743	25012.078	5.0267436	1.24E+08	0.0197131
cg08115682	1.5937185	0.738434	3.439628	0.235059
cg14121987	0.5045043	0.1940692	1.311515	0.1604317
cg13153708	0.5316481	0.2704051	1.045283	0.0670154
cg15418980	0.2307304	0.0808038	0.658836	0.0061543
cg21139496	0.3360625	0.1046224	1.079482	0.0670249
cg06242730	1.2289062	0.2737751	5.516244	0.787892
cg16847760	0.4195353	0.1516415	1.160698	0.0943376
cg18946802	467.90306	4.3635961	50172.67	0.0099478

cg25122752	0.3550928	0.1345661	0.937018	0.0364954
cg16268769	1.2681904	0.3875404	4.150037	0.6944702
cg18446578	0.5561972	0.1782428	1.735584	0.3123176
cg04203702	0.3314339	0.144894	0.758129	0.0088999
cg18456933	0.4573457	0.2158803	0.968894	0.0411054
cg08762819	1.4953493	0.7638589	2.927333	0.2403966
cg02914652	0.1654487	0.0480699	0.569447	0.0043326
cg07331701	1.527073	0.5039849	4.627028	0.4541599
cg01093311	0.4417678	0.2033261	0.959831	0.0390636
cg12799314	4.4901929	0.9271692	21.74558	0.0620397
cg16043635	3.7281805	1.2273707	11.32448	0.0202665
cg05314142	0.3774624	0.1771599	0.804233	0.011587
cg19500979	0.403039	0.1564734	1.038134	0.0597767
cg01933946	2.93E-28	1.94E-48	4.43E-08	0.0074907
cg05951776	0.1968826	0.0610871	0.634549	0.0064946
cg14484973	1.289643	0.5958498	2.791272	0.5184892
cg18696159	0.8200602	0.3957653	1.699236	0.593566
cg11841562	0.6677532	0.3154769	1.413398	0.2911624
cg13325231	1.7718638	0.3274488	9.587764	0.5066799
cg05295006	0.1125375	0.0082148	1.541695	0.1018801
cg17915125	294.40683	0.0056878	15238721	0.3046432
cg21037057	0.9208794	0.4048752	2.094519	0.8441441
cg25975823	0.3949204	0.0657224	2.373043	0.3098924
cg07935012	0.0063689	0.0003652	0.111085	0.0005273
cg13116174	10128.345	7.53E-11	1.36E+18	0.5784496
cg23736843	1.9269257	1.0314996	3.599655	0.0396636
cg06097717	0.8172788	0.4209159	1.586884	0.5511778
cg07142201	2.5727669	1.2146122	5.449583	0.0135994
cg05532966	0.2288467	0.0871944	0.600622	0.0027402
cg07570498	0.2519344	0.0813491	0.78023	0.0168371
cg08432053	0.562665	0.136268	2.323304	0.4267121
cg14189614	0.4432423	0.2147663	0.914779	0.0277426
cg02279153	15.453759	0.0176531	13528.43	0.4283148
cg25119077	0.2589454	0.1062364	0.631165	0.0029557
cg20896728	0.1036546	0.0180118	0.596512	0.0111298
cg00845765	2.047669	0.8944861	4.68755	0.08987
cg01590541	0.3257601	0.1135831	0.934291	0.0369426
cg25341032	0.4152973	0.1823313	0.945926	0.0364093
cg26468693	2.4941732	0.5952347	10.45117	0.2112034
cg14657277	1.8688414	0.7752532	4.505068	0.1636462
cg19488260	0.3574745	0.1221787	1.045911	0.0603791
cg00151370	1.265251	0.4630212	3.457423	0.6464409
cg15352367	0.5750008	0.2825511	1.170146	0.1268798
cg12952446	0.1676362	0.054847	0.512369	0.0017299
cg12723116	2.8686067	1.1210913	7.340084	0.0279197
cg14910288	3.5234387	0.5867388	21.15868	0.1685081
cg04165857	0.3521352	0.1675974	0.739864	0.0058634
cg14107561	0.6211402	0.0713021	5.410993	0.6663422
cg24237636	0.6919742	0.2744277	1.744825	0.4352125
cg22756211	0.1868901	0.0400018	0.873159	0.032973
cg02376553	9.25E-05	2.93E-08	0.291741	0.023842
cg10656128	2.9722976	0.8894426	9.932685	0.0767878
cg26509250	0.8807849	0.1035738	7.490137	0.9074674
cg08325103	6.4206112	1.6367221	25.18708	0.0076652
cg11213707	3.1737131	1.061711	9.487003	0.0387204
cg17397004	0.3064468	0.1250521	0.750964	0.0097033
cg20744464	0.357806	0.1335372	0.958723	0.0409743
cg08491013	2.5914655	0.8835909	7.600456	0.0828247

cg09892131	1363.8912	1.08E-09	1.73E+15	0.6116872
cg08638184	2.302462	0.9761725	5.430732	0.0567955
cg02288964	0.3795734	0.1626799	0.885641	0.0250327
cg12124911	1.9357115	0.8357827	4.483198	0.1232356
cg12719836	2.2400506	0.3219124	15.58755	0.4151818
cg21542922	3.0235171	1.0740488	8.511397	0.0361495
cg19206078	0.2660377	0.1127703	0.627612	0.0024968
cg13894719	2.0198278	0.7648656	5.333884	0.1559188
cg08321272	130857.42	157.84374	1.08E+08	0.00059
cg11130432	0.8749478	0.2548754	3.00356	0.8318824
cg24232444	2.3213328	0.5709393	9.438106	0.2392836
cg19580344	0.2577032	0.1259317	0.527357	0.0002061
cg01697794	0.3436076	0.1477065	0.79933	0.0131407
cg11911653	0.2462127	0.0730749	0.829569	0.0237315
cg26083576	356713462	0.0120892	1.05E+19	0.1093792
cg00980592	0.012693	0.0005628	0.286248	0.0060175
cg16216305	3.832E+09	1.46E-25	1.00E+44	0.5852543
cg25913233	0.0044263	4.58E-06	4.278782	0.1222314
cg12291109	5.5761647	0.3864276	80.46427	0.2070123
cg10331073	0.2990962	0.1179983	0.758134	0.0109761
cg23729763	0.4939189	0.2111985	1.155102	0.1036691
cg09992653	109.89321	0.6393474	18888.82	0.0735143
cg03785652	5.5729121	1.2764886	24.3303	0.0223362
cg17695831	0.4108829	0.2048594	0.8241	0.0122529
cg23811268	0.0974844	0.0096755	0.982194	0.0482446
cg24362123	1.0086684	0.4172732	2.43824	0.9847089
cg18190433	0.2562109	0.0948002	0.692446	0.0072642
cg16767867	6.2597621	0.5528379	70.87905	0.1385283
cg01631333	0.2745773	0.0822064	0.917115	0.035678
cg21723903	1.1257179	0.588668	2.152726	0.7203381
cg17975258	2.3007716	0.6865186	7.710716	0.1768875
cg11823253	0.2508582	0.0820573	0.766901	0.0152893
cg19863224	1.704748	0.4204451	6.912117	0.4551565
cg12554634	0.1351334	0.0397122	0.459835	0.0013583
cg16028793	195461915	2.55E-20	1.50E+36	0.5600573
cg22507756	3.0940967	1.3969856	6.852923	0.0053695
cg18277815	0.0853906	0.0107203	0.68016	0.0201251
cg12984086	0.2490134	0.0872619	0.710592	0.0093614
cg14381255	3.4395276	1.0557745	11.20538	0.0403613
cg04127825	7.335E+11	0.0006896	7.80E+26	0.1217147
cg14067101	1.9269092	0.904817	4.103569	0.0890124
cg23959772	0.5074437	0.2602516	0.989424	0.0464618
cg18834990	1.242E+11	159.2811	9.69E+19	0.0144707
cg04031424	0.1299622	0.0009439	17.89395	0.4167625
cg26544870	0.0259861	0.0015948	0.423411	0.0103614
cg02276944	0.2674618	0.0903922	0.791394	0.0171879
cg09085041	1.8419577	0.6847279	4.954972	0.2263441
cg25321720	0.9413076	0.4321525	2.050341	0.8789659
cg00861207	2.2414779	0.8200255	6.126911	0.1156687
cg08713711	0.0209042	0.0016085	0.271668	0.0031177
cg06950666	4.02E-05	3.04E-08	0.053157	0.0057764
cg27447006	2.0805409	0.7020876	6.165399	0.1862286
cg15696506	0.3826062	0.1473488	0.993476	0.048449
cg04947157	1.881154	0.7028165	5.035084	0.2084229
cg00028993	0.5536745	0.2285748	1.34116	0.1903054
cg24384437	0.3674303	0.1510175	0.893969	0.0273116
cg02725900	2.24E-05	7.52E-17	6686481	0.4271069
cg05294243	1.7198531	0.7550261	3.917606	0.1967191

cg08647039	1.11187	0.2198855	5.622267	0.8979574
cg13911707	0.398288	0.2158649	0.734873	0.0032223
cg07170231	0.9870847	0.3626422	2.68677	0.9797006
cg11533712	0.1749774	0.0247905	1.235032	0.0804218
cg01208855	2.3642786	0.7108965	7.863048	0.1604906
cg09125999	1.8406102	0.7390385	4.584126	0.1900512
cg24250684	0.4813898	0.2130145	1.087889	0.0788394
cg12371924	1.6909756	0.5067917	5.642157	0.3928551
cg13563661	4.42E-05	6.66E-08	0.029397	0.0024978
cg06764387	2.6700081	1.2909544	5.522227	0.0080791
cg17354880	0.611962	0.2921264	1.281971	0.1930543
cg15441325	156711.08	185.68643	1.32E+08	0.0005023
cg27586068	2252856.3	5.40E-07	9.4E+18	0.3238526
cg17241776	2.0113505	0.7313125	5.531877	0.1758104
cg12689427	1.52E-16	1.28E-76	1.82E+44	0.6058369
cg26366833	1.506251	0.512435	4.427473	0.4565044
cg04140803	8.551E+12	2.12E-30	3.44E+55	0.5519023
cg23076086	4097515.9	0.0022124	7.59E+15	0.1619797
cg18259504	0.21827	0.0412747	1.154262	0.0732713
cg12449722	0.2420662	0.0819662	0.71488	0.0102451
cg05261702	0.1117581	0.0196763	0.634769	0.0134052
cg05591270	0.3347661	0.1266225	0.885059	0.0273759
cg08104146	0.3426544	0.1360083	0.863271	0.0230968
cg12897067	0.377125	0.1459738	0.974307	0.0440396
cg02613156	0.0119356	3.53E-07	404.0411	0.4053214
cg19406789	0.1734614	0.0435625	0.690706	0.0129606
cg14176836	2.0544064	0.9357464	4.510394	0.072742
cg04990605	1.3886913	0.4158039	4.637916	0.5935567
cg16421871	0.0891803	0.0213829	0.371939	0.0009087
cg24765658	1.4098292	0.4961781	4.005856	0.5191632
cg18143863	3699.7054	1.5013215	9117181	0.0392131
cg00389081	1.20E-08	2.40E-12	6.01E-05	2.71E-05
cg11464895	0.2000815	0.0637304	0.628156	0.0058419
cg19032799	0.2200821	0.0279311	1.734128	0.1506403
cg15974430	9.19E-14	1.11E-23	0.000764	0.0099999
cg21625271	0.3529175	0.1558309	0.799269	0.0125189
cg00495303	0.397522	0.1477777	1.069334	0.0676716
cg20749576	1.3787534	0.5539503	3.431645	0.4899754
cg10654010	0.0732071	0.016268	0.329437	0.0006571
cg15323840	0.4294718	0.1755221	1.050842	0.064121
cg07580975	3.2714272	0.669205	15.99246	0.1432297
cg03382501	1.7310166	0.4305251	6.959916	0.4395853
cg05345154	0.6785342	0.3557126	1.294328	0.2391994
cg27661846	2.0125022	0.9146544	4.428082	0.082168
cg17996619	0.1514148	0.0355759	0.644437	0.0106326
cg06008435	4.3160625	1.2279704	15.17007	0.0225971
cg21771679	0.3894148	0.1553412	0.976198	0.0442897
cg04358214	0.2040551	0.0630915	0.659969	0.0079579
cg19683417	0.2658096	0.1168087	0.604876	0.0015868
cg08872425	0.2672251	0.0876264	0.814928	0.0203568
cg02643782	1.4666713	0.6723835	3.199253	0.3358098
cg17737621	2.9052557	0.907352	9.302355	0.0724596
cg24253904	1.1878464	0.4899705	2.879722	0.7032061
cg02030219	1.633264	0.5206714	5.123292	0.4003112
cg14873515	4.096564	0.734599	22.84489	0.1077886
cg12513352	4.3686363	1.3079507	14.59152	0.0165631
cg08885633	0.5104653	0.2667894	0.976706	0.042239
cg16679650	1.7424511	0.7125078	4.261196	0.223586

cg24419324	2.9668224	1.0030995	8.774838	0.0493498
cg18795433	0.1741358	0.0458703	0.661065	0.0102264
cg19499884	0.4666413	0.1981791	1.098774	0.0810918
cg02994956	0.2399772	0.0784236	0.734333	0.0123807
cg10409981	2.4392818	0.7661586	7.766141	0.1312591
cg21225319	2.5266282	0.9275882	6.882203	0.069841
cg02547724	1.7378803	0.6723541	4.49202	0.2540142
cg02262923	3.5922877	0.7770787	16.60647	0.1016128
cg24115264	1.7266718	0.7373369	4.043464	0.208356
cg03407228	0.9750696	0.3337575	2.848657	0.963187
cg05745142	11.03629	0.0547053	2226.469	0.3751855
cg09813610	2.1298466	0.6018177	7.537576	0.2410072
cg11961237	0.1540922	0.0210467	1.128179	0.0655881
cg23330051	0.1034907	0.0268295	0.3992	0.0009906
cg04985611	0.9249438	0.4602735	1.858723	0.8265628
cg11656175	1.977227	0.3756997	10.40572	0.4210746
cg01376826	1.3148791	0.537158	3.218619	0.5489492
cg12253437	1.7645418	0.91743	3.393837	0.0888073
cg00747975	1.6207048	0.5923367	4.434444	0.3470944
cg25424742	1.6005343	0.2693615	9.510304	0.6049483
cg17382048	3.49647	1.0752697	11.36952	0.0374716
cg07143303	0.2672551	0.1116047	0.639984	0.0030595
cg07486085	0.5868152	0.2816032	1.222828	0.1547491
cg05328670	0.2168489	0.0420374	1.118609	0.0678409
cg24431872	1.6110049	0.7868816	3.298256	0.1921088
cg03920999	1.6649572	0.3311455	8.371193	0.5361181
cg04483989	0.7687547	0.3815453	1.548922	0.4618707
cg07832178	3.5154286	1.128025	10.95564	0.0301828
cg15699099	0.33145	0.1172105	0.937281	0.0373342
cg09005322	0.3277208	0.1525179	0.704186	0.0042545
cg00810292	1.0602526	0.5511035	2.03979	0.8608846
cg11637968	0.5670261	0.2784199	1.154798	0.1179658
cg27358811	9.07E-37	8.59E-65	9.58E-09	0.01171
cg15879949	1.0588382	0.5050358	2.219918	0.8796875
cg02626307	0.3897081	0.1816627	0.836013	0.0155244
cg18893528	2.3759454	0.0538822	104.7677	0.65418
cg25756470	1.7315283	0.6804132	4.406426	0.2493244
cg12371563	0.2678695	0.1176424	0.609934	0.0017034
cg01757206	2.57E-05	7.15E-11	9.253088	0.1054296
cg19003066	0.5427892	0.2495852	1.180439	0.1232008
cg01367627	1.0842112	0.3809057	3.086102	0.8795882
cg06209689	2.7739361	1.1220858	6.857516	0.0271462
cg00059309	0.2288369	0.0767376	0.682408	0.0081586
cg26935685	0.6757759	0.1720122	2.654888	0.5745567
cg05272790	0.7929798	0.3739458	1.681573	0.5453048
cg18024859	3.1698397	0.0032284	3112.386	0.7427548
cg03497399	2.636993	0.8337352	8.340457	0.0988509
cg17519696	3.2736197	1.3987727	7.66142	0.0062662
cg02331196	0.3898044	0.158588	0.958127	0.0400547
cg02749887	0.3359878	0.0922093	1.224256	0.098277
cg19414980	0.5430253	0.2815467	1.047345	0.0684646
cg18250736	0.5951071	0.2866566	1.235459	0.1637346
cg22027879	0.3422897	0.1589895	0.736918	0.0061393
cg15724159	0.9067178	0.2952823	2.784241	0.8641658
cg16787352	0.2344189	0.0909282	0.604347	0.0026803
cg11284973	0.2803661	0.1201977	0.653965	0.0032529
cg22830947	1.8881524	0.745899	4.779627	0.1798231
cg15256053	0.3300472	0.1184912	0.919319	0.033929

cg14844624	0.6657722	0.319419	1.387684	0.2776462
cg04912542	1.7232475	0.6529642	4.547848	0.2717161
cg24503957	0.4286291	0.2027275	0.906255	0.0265796
cg10415509	2.2249227	0.4086025	12.11515	0.3550282
cg10091458	1.9277551	0.4823596	7.704293	0.3531219
cg07642463	0.1750052	0.0630977	0.485388	0.000812
cg10585621	0.3352395	0.1265369	0.888164	0.0279105
cg02678141	0.0005787	6.79E-08	4.935944	0.1064738
cg20793665	0.2080444	0.09113	0.474953	0.0001932
cg04297067	2.0030296	0.9241138	4.341595	0.0784061
cg15416661	0.112604	0.0140872	0.900082	0.0394727
cg05455409	1.2850937	0.6775749	2.437319	0.4424411
cg05957567	12.633065	2.25095	70.90088	0.0039535
cg27569863	1.8077198	0.9026167	3.62042	0.0947559
cg25082361	0.3650875	0.0564448	2.361403	0.2901184
cg05081167	2.4251272	0.8046831	7.308768	0.1155129
cg23858731	1.529929	0.3821033	6.125785	0.5480033
cg16469223	5.8702797	1.4690411	23.4576	0.0122755
cg11225405	0.3408864	0.1429928	0.812653	0.015183
cg13875008	0.0565599	0.0093554	0.341944	0.0017549
cg06589421	0.5417956	0.2547525	1.152265	0.1114208
cg16049699	0.4738133	0.2086286	1.07607	0.074297
cg11260715	1.2599632	0.5074391	3.128469	0.6184827
cg23714541	0.0626188	1.69E-05	231.4563	0.5085902
cg17338212	2.4706515	0.9621343	6.344352	0.0601434
cg16885861	0.5841174	0.2869818	1.188901	0.1381349
cg07654588	0.3889542	0.1773184	0.853185	0.018466
cg04224064	2.4922918	1.0477764	5.928286	0.0388734
cg01592801	0.1550245	0.0367459	0.654022	0.0111463
cg17141783	2.0610915	0.9277203	4.579072	0.0757732
cg21897034	0.2916633	0.1266433	0.671709	0.0037931
cg05293216	0.4103766	0.2203928	0.764131	0.0049833
cg07021644	1.2132664	0.5479071	2.686615	0.6336361
cg03137135	3.3770229	1.1384962	10.01697	0.0282513
cg23645373	2.4990258	0.4322421	14.44822	0.3062795
cg05315865	0.3447298	0.0063539	18.70327	0.6012097
cg18224988	0.1240241	0.019487	0.789347	0.0270722
cg01876548	0.2605262	0.124048	0.547158	0.0003812
cg08821193	57.74183	0.1745922	19096.61	0.170589
cg27372422	4.1201044	0.4828798	35.15422	0.1955191
cg17079034	0.077087	0.0148239	0.400866	0.0023138
cg26640901	0.2457208	0.0966908	0.624451	0.0031829
cg17536532	0.0765622	0.0088517	0.662218	0.0195753
cg03020863	0.0925213	0.0203704	0.420228	0.0020508
cg13902498	6.22E-13	4.99E-20	7.75E-06	0.0007469
cg24516259	1.6874776	0.7671385	3.711951	0.193296
cg25188594	1.2293586	0.5322771	2.839353	0.6287509
cg17826428	1.7823304	0.8091875	3.925792	0.1514448
cg08899626	0.6770231	0.267224	1.715266	0.4108703
cg26976450	0.0020353	1.54E-05	0.268615	0.0128603
cg19992808	1.8792422	0.8593383	4.109617	0.114051
cg12686016	1.4667971	0.5290725	4.066539	0.4615409
cg02892367	1.3813315	0.4316855	4.420062	0.5861855
cg07257628	0.2183589	0.0627304	0.760087	0.0168013
cg09354264	0.3078282	0.1040043	0.911099	0.0333262
cg06648277	0.0806639	0.0102036	0.637683	0.0170111
cg16932560	0.1732407	0.0370482	0.810089	0.0259084
cg04075546	0.6114485	0.2654013	1.408695	0.2479897

cg04898035	4.0806015	0.1428171	116.5919	0.4109943
cg24587744	1.9256786	0.8573932	4.325014	0.1124506
cg06473288	2.0339702	1.0055809	4.114074	0.0482172
cg05729000	1.7742747	0.5658457	5.563443	0.3254224
cg25281849	0.455122	0.220236	0.940518	0.0335408
cg00020991	0.6820477	0.2256848	2.061234	0.4976855
cg19544707	4.7142078	1.1350324	19.57984	0.0328176
cg10759972	1.3194486	0.4476633	3.88896	0.6152097
cg26946235	0.4741373	0.2217694	1.013693	0.054244
cg27621129	2.2656405	0.9182564	5.59008	0.0759153
cg24662698	0.2155549	0.0567362	0.818947	0.0242435
cg23517954	0.0629876	0.0045299	0.875832	0.0395245
cg24119674	0.385367	0.1236047	1.201473	0.1002602
cg27061489	0.0006078	6.83E-07	0.540637	0.0325598
cg01076129	0.1811775	0.0537018	0.611251	0.0058988
cg06767762	11149.205	58.547249	2123153	0.0005023
cg23676439	2.4096829	1.1134249	5.215055	0.0255676
cg16781880	0.3634147	0.1419003	0.930726	0.0348941
cg26717935	0.187737	0.0667371	0.528119	0.0015255
cg24871414	2.7829883	1.0717305	7.226652	0.0355315
cg04638265	1.9661749	0.8606753	4.49164	0.1087133
cg15761531	0.0673175	0.0123461	0.367053	0.0018199
cg24001468	2.1888458	0.021919	218.5799	0.7387527
cg01960748	0.2571792	0.0082292	8.037405	0.4393742
cg20682517	11.930774	0.2660404	535.0442	0.201392
cg18728493	2.5487014	0.1948871	33.3315	0.4756903
cg09277532	1.0688263	0.5260093	2.171805	0.8540114
cg24135841	0.4979266	0.2103901	1.178435	0.1126431
cg01288645	0.2896865	0.1327308	0.632244	0.0018625
cg12032489	0.045915	0.0037479	0.562496	0.0159508
cg18789758	0.2528486	0.0660522	0.967907	0.0446868
cg10857774	0.1831568	0.0543454	0.617281	0.0061776
cg01705005	7.5913185	0.4979695	115.7262	0.1447453
cg11891330	16850.879	0.0280812	1.01E+10	0.1516671
cg09997532	1.3476986	0.4927015	3.686393	0.561094
cg13389958	2.1582003	0.9915516	4.697515	0.0525521
cg09580922	0.3559083	0.153956	0.822772	0.0156828
cg01156892	1.8154491	0.9546633	3.452375	0.0689907
cg02745822	0.3338956	0.1346017	0.828268	0.0179597
cg06154903	1.2901518	0.5665262	2.938067	0.5440411
cg26505691	1.2095407	0.5068692	2.886324	0.6681365
cg05744888	0.1273266	0.0401746	0.40354	0.000462
cg08292104	0.4873956	0.1905121	1.246926	0.1337401
cg06854353	1.463E+12	14.021371	1.53E+23	0.0304678
cg01620611	0.4161145	0.1609282	1.075953	0.070462
cg06264679	0.1732355	0.0346828	0.865284	0.0326559
cg22226904	0.3044676	0.1034311	0.896254	0.0308655
cg04584009	0.2346377	0.0877543	0.627375	0.0038641
cg01254034	2.4697764	1.1062119	5.514129	0.027364
cg11779239	2.0011349	0.132784	30.1583	0.6162233
cg11631592	0.36305	0.1755347	0.750879	0.0062818
cg24209738	0.1849987	0.0199332	1.716964	0.137695
cg17826344	1.5273578	0.7354469	3.171979	0.2560055
cg07764113	0.463089	0.1565321	1.370015	0.1641998
cg07091500	0.3912729	0.1286308	1.190185	0.0982873
cg25721832	0.5466113	0.2980342	1.002515	0.0509565
cg08443680	1212366.3	5.57E-06	2.64E+17	0.2929495
cg12849224	0.5928449	0.2913826	1.206198	0.1491168

cg00581320	0.9771793	0.3939044	2.42414	0.9602822
cg20784098	12.445036	0.3860733	401.1646	0.1547737
cg27253814	5.17E-09	2.80E-32	9.54E+14	0.4851311
cg00849610	3.7827723	1.4514047	9.858978	0.0064852
cg26400275	0.402944	0.1953146	0.831294	0.0138923
cg03656020	0.1302091	0.0295541	0.573673	0.007051
cg18699025	0.2380777	0.1013166	0.559444	0.0009933
cg15605918	220.87882	4.9401202	9875.762	0.0053723
cg09821936	0.3512881	0.1278599	0.965145	0.0424823
cg13574488	2.6342114	1.1126728	6.236397	0.0276109
cg25383605	0.208024	0.0870309	0.497226	0.0004132
cg06413398	1.2507758	0.5014265	3.119979	0.6313678
cg21153898	2.7095624	1.0263591	7.153177	0.044168
cg20452739	0.6448058	0.1137638	3.654716	0.6200689
cg25825300	2.16E-23	2.82E-41	1.66E-05	0.0129937
cg01247987	0.2612483	0.0889952	0.766903	0.0145662
cg01985595	0.2495519	0.0577633	1.078127	0.0629977
cg04338175	0.2441771	0.0003742	159.3186	0.6698301
cg01417823	0.2321658	0.1037802	0.519376	0.0003784
cg23213158	4.2215168	0.4852291	36.7274	0.1919581
cg13761421	0.6395209	0.327827	1.24757	0.1897969
cg11343579	1.47E-28	8.07E-65	2.69E+08	0.1325027
cg22788243	0.6412059	0.286905	1.433035	0.2787703
cg14971286	0.0040177	4.16E-05	0.38785	0.0179729
cg07533249	2.6353879	0.8958352	7.752842	0.0783806
cg21062780	3.1249939	0.8868578	11.01145	0.0762093
cg27219748	2.0519274	0.8395172	5.015271	0.1149489
cg17981470	3.5370925	0.581579	21.51217	0.1702114
cg24798956	0.2396563	0.0558195	1.028943	0.054658
cg00520247	2.6399971	0.5938592	11.73609	0.2021838
cg24105147	0.9858102	0.4759063	2.042045	0.9693183
cg11188440	0.398779	0.166393	0.955717	0.0392527
cg18767278	2.1100515	0.476748	9.338932	0.3251653
cg07683820	0.0615748	0.0002725	13.91515	0.3134934
cg12689803	0.3935101	0.1080705	1.432863	0.1572227
cg08840230	1.665213	0.4917946	5.638399	0.4125065
cg04848349	0.1102115	0.0312264	0.388984	0.0006094
cg02187503	0.2044419	0.0596431	0.700777	0.0115478
cg14741236	1.57889	0.6379047	3.907941	0.32329
cg08841511	0.0572756	0.0034202	0.959143	0.0467039
cg07416364	0.5247423	0.2451356	1.123274	0.096793
cg06009497	8.3888648	0.596059	118.0639	0.1149206
cg06927813	140692.01	6.070801	3.26E+09	0.0207968
cg18520925	0.6057028	0.2812546	1.304426	0.200209
cg09330834	6.4861659	0.3365284	125.0128	0.2155202
cg14326413	0.234642	0.0746113	0.737916	0.0131434
cg20855160	0.4417259	0.1922832	1.014763	0.0541756
cg09801334	33.437149	0.9940083	1124.782	0.0503929
cg14239618	1.6292877	0.611202	4.343209	0.3291639
cg02087289	3.1253157	0.8594626	11.36477	0.0836238
cg14759342	0.3526914	0.0983799	1.264397	0.1096354
cg08314660	1.8677558	0.3511073	9.935743	0.4638044
cg20464368	0.0409959	0.000135	12.45032	0.273392
cg07965326	0.0054444	1.29E-05	2.290216	0.090808
cg24414344	2.6945192	0.528584	13.73563	0.2329587
cg19682367	0.6006738	0.2278825	1.583312	0.3026704
cg06218688	1.694056	0.7414514	3.870551	0.2111634
cg23026554	0.3443038	0.10404	1.139418	0.0807745

cg12668309	0.4963145	0.242626	1.015258	0.0550485
cg14543291	1.4975177	0.5889229	3.8079	0.3964148
cg02773045	0.2698177	0.0037955	19.18104	0.5470675
cg02388319	2.1276198	0.5548977	8.157839	0.2708761
cg09336671	0.4867656	0.2541215	0.932392	0.0299271
cg14697425	0.3575698	0.1439405	0.888257	0.0267466
cg11583106	1.8975747	0.719121	5.007209	0.1956882
cg27025137	0.5174273	0.250874	1.067193	0.074441
cg14648936	0.1070204	0.0006205	18.45964	0.3950846
cg00512726	0.0043276	9.86E-05	0.189967	0.0047913
cg17321385	1.7656664	0.5550892	5.616355	0.3355671
cg10142436	0.3571115	0.1065535	1.19685	0.0951667
cg20685215	79.42666	0.003718	1696758	0.3897428
cg17591915	9.3745021	1.3482215	65.18312	0.0237006
cg20583945	3.5372805	1.1387268	10.98802	0.0289177
cg04246526	0.2568667	0.080168	0.823028	0.0221496
cg07422940	1.9932331	0.6194916	6.413288	0.2473373
cg05063374	2.8827974	0.724238	11.47485	0.1330454
cg02408697	5.5048251	0.7177754	42.21808	0.1008097
cg09293816	0.3300932	0.145314	0.749835	0.008104
cg07135614	2.6559938	1.0000879	7.053683	0.0499794
cg25286536	1.3565935	0.6683769	2.753455	0.3984382
cg20102585	0.672947	0.2743806	1.650473	0.3868647
cg08657020	723168902	8.6064761	6.08E+16	0.028439
cg17171215	1.9932807	0.9306454	4.269261	0.0758972
cg25105657	0.3055957	0.1058949	0.8819	0.0283518
cg02587316	0.669191	0.2546527	1.758538	0.415154
cg15594471	0.1549353	0.0377352	0.636142	0.0096632
cg10689512	1.2653027	0.4284955	3.736308	0.6701517
cg04087039	0.12882	0.0237986	0.697293	0.0173876
cg22171993	0.3202927	0.1385306	0.74054	0.007759
cg00632853	0.3625575	0.1361793	0.965256	0.042281
cg18350249	0.5382374	0.1795419	1.613548	0.2687885
cg10131879	12.06247	1.9308354	75.35763	0.0077259
cg04163147	0.497897	0.2279457	1.087546	0.0802167
cg13078563	0.7718773	0.3020378	1.972582	0.5885905
cg18190219	2.0941053	0.2448274	17.91171	0.4997084
cg08653403	0.0437841	0.0001342	14.28075	0.2893744
cg19882663	1.5538527	0.2303565	10.4814	0.6508831
cg00423969	0.3395711	0.1071823	1.075817	0.0663942
cg01961211	2.5697218	0.9033851	7.309695	0.0768157
cg19755459	0.4457646	0.1876708	1.058801	0.067173
cg15602548	1.6378659	0.7293484	3.678084	0.2319508
cg21674010	0.3959743	0.1461856	1.072579	0.0684322
cg17587385	0.4083994	0.1479199	1.12757	0.0839435
cg23597271	3.9944199	0.6345975	25.14254	0.1400886
cg19091146	248.5649	2.3515695	26273.73	0.0203648
cg10001534	0.0001206	2.32E-13	62770.26	0.3782429
cg16590794	7.7174237	1.2752628	46.70302	0.026103
cg09059904	2.3290164	1.0302246	5.26518	0.0422028
cg15383705	0.6449286	0.0888634	4.680589	0.6644842
cg09557747	2.8364029	0.8188908	9.824486	0.1000224
cg02100150	0.4327051	0.1552723	1.205841	0.1091539
cg01293250	3135.0908	3.40E-09	2.89E+15	0.566821
cg00037930	4.3652467	1.4290343	13.33445	0.0096939
cg02480320	0.4367019	0.1584388	1.203673	0.109243
cg04876548	8.0875993	0.1221147	535.6378	0.3285348
cg05501617	1.6700468	0.7404992	3.766455	0.2164797

cg13411507	0.5750481	0.2867025	1.153392	0.1192094
cg09259534	0.4225931	0.1791946	0.996598	0.0490969
cg15836912	2.5712207	0.2638696	25.05471	0.4162154
cg16897103	1.8557694	0.4308414	7.993382	0.4066226
cg18502630	0.5053554	0.1922614	1.328317	0.1663091
cg27351783	4.653771	0.4490082	48.23427	0.1974567
cg07180219	0.2163905	0.022688	2.063858	0.1834336
cg10705085	1.0783445	0.4525481	2.56951	0.8648063
cg20798740	0.7222905	0.2941866	1.773376	0.4777734
cg22693435	1.5555557	0.4966655	4.871998	0.4481419
cg15156975	0.0432179	0.0009362	1.995171	0.1081211
cg04719766	1.6383767	0.7242845	3.70611	0.2358442
cg11414505	1.6817734	0.2505922	11.28671	0.5925176
cg27119412	0.8283016	0.19733	3.476834	0.7968842
cg05500783	0.7207245	0.3772971	1.37675	0.321319
cg26273417	1.5123597	0.6704915	3.411277	0.3188807
cg07938459	1.6026716	0.617112	4.162221	0.3327183
cg27638035	3.6672155	1.62733	8.264132	0.0017209
cg07248195	0.348164	0.1128612	1.074046	0.0664048
cg06514003	0.6275533	0.287142	1.371528	0.2428086
cg02068015	2.1602869	0.867034	5.382534	0.0981992
cg03267026	0.302045	0.121781	0.749142	0.0097897
cg12127048	0.4311251	0.2080137	0.893542	0.0236554
cg05778528	3.9969003	0.4399928	36.3079	0.2184337
cg20454486	0.5993031	0.2956585	1.214794	0.1555421
cg22476960	0.8483138	0.3774574	1.906536	0.6905158
cg01542693	1.5085278	0.5939005	3.831713	0.3873482
cg09131511	0.490861	0.240163	1.003254	0.0510503
cg00843019	1.8356325	0.6753248	4.98952	0.2338422
cg00662122	0.0001672	5.63E-10	49.62146	0.1761649
cg24533757	1.11E-11	1.50E-23	8.129404	0.0703502
cg04564980	0.9193883	0.4319152	1.957039	0.827394
cg15056751	0.2687904	0.0680071	1.062363	0.0609733
cg00971676	0.5254051	0.0788865	3.499336	0.5058956
cg11250081	2.2299879	1.0634012	4.676359	0.033782
cg13315047	0.147178	0.0235492	0.919835	0.0404294
cg21232625	0.1706983	0.0476669	0.611281	0.0066037
cg22151713	0.2963418	0.1444127	0.608107	0.0009126
cg04147479	0.8378024	0.4024366	1.744157	0.636178
cg12939283	1.6559792	0.7908272	3.467593	0.181021
cg27480573	1.3756214	0.5800493	3.262368	0.4691832
cg03527422	1.584E+18	0.0010269	2.44E+39	0.0922737
cg24724567	0.440624	0.1531689	1.267552	0.128462
cg22872195	0.7842209	0.4081832	1.506682	0.4656463
cg24681612	0.8180034	0.3791137	1.764984	0.6086589
cg05989861	3.7443369	1.142339	12.27312	0.0292819
cg14495514	1.6865209	0.8834339	3.219655	0.1131285
cg15016232	0.3632935	0.1685728	0.782938	0.0097498
cg14345917	2.1942206	0.8405583	5.727865	0.108455
cg12690996	2.148817	0.5421624	8.516662	0.2763009
cg01744729	6.7148627	1.1301724	39.89602	0.0362103
cg06052984	18.357353	0.7418277	454.2731	0.0754785
cg21962791	0.2547814	0.0621741	1.044061	0.0574269
cg10903903	1.5858124	0.9023894	2.786824	0.1089534
cg27129984	1.3853235	0.4725331	4.061348	0.55256
cg17249452	2.0665096	0.7764494	5.499987	0.1461268
cg01326135	8182156.3	127.96721	5.23E+11	0.0048126
cg24867665	1.408897	0.5540105	3.582948	0.4716194

cg01496759	2.0633649	0.5166569	8.240429	0.3052459
cg10071824	0.0232648	0.0009469	0.571633	0.0213162
cg07280182	2.3292759	1.2000131	4.521222	0.0124617
cg13342158	1.845E+14	9.74E-08	3.49E+35	0.1888099
cg13380624	1.0163283	0.4420852	2.33648	0.9695811
cg02138264	1.9945491	0.7336348	5.422625	0.1760635
cg00470924	152.08008	5.3771694	4301.213	0.0032148
cg05878101	0.1054143	0.0217484	0.510941	0.005209
cg07440387	53447.945	402.77747	7092459	1.27E-05
cg19878326	0.5075534	0.2552588	1.009213	0.0531361
cg25457672	3418.5307	0.0003209	3.64E+10	0.3243312
cg24674680	0.2528545	0.05992	1.067014	0.0612527
cg14750551	6.6125088	1.2115093	36.09157	0.0291428
cg01915888	0.0059563	1.03E-14	3.43E+09	0.7107755
cg16404245	0.009023	2.58E-09	31545.47	0.5402586
cg18830697	0.6371658	0.3086453	1.315362	0.2229339
cg15006866	0.1652207	0.0134925	2.023192	0.1589406
cg19482025	0.1628831	0.0534239	0.496611	0.0014198
cg05163329	2.3547334	1.3260268	4.181491	0.0034656
cg18760360	0.743919	0.3192804	1.733322	0.4930555
cg02668694	0.6985391	0.3496794	1.395441	0.3095477
cg22705225	0.9877678	0.3080489	3.167306	0.9834828
cg17222164	3.0093238	1.0132228	8.937847	0.0472987
cg11829371	0.1709809	0.0284039	1.029241	0.0537937
cg20516262	1.9519047	0.8964027	4.250246	0.0920837
cg05000748	3.8916581	1.1550911	13.11152	0.0283354
cg24136314	0.1854604	0.0307081	1.120081	0.066303
cg21008399	0.538164	0.118236	2.449511	0.4229491
cg21805936	0.638008	0.3351704	1.214469	0.1712056
cg07013325	0.7964473	0.2837679	2.235377	0.6655648
cg09433131	0.9417414	0.4508702	1.967034	0.8730972
cg21907496	7.9786251	1.1002275	57.85936	0.0399317
cg14062589	1.0796794	0.451447	2.582158	0.8631836
cg23553912	1.2335766	0.5744641	2.648923	0.5903308
cg05135521	1.6925939	0.4233703	6.766828	0.4566836
cg02325250	3.2144686	0.9431397	10.95576	0.0619865
cg24940706	1.3559031	0.2817615	6.524926	0.7040851
cg11248166	0.490504	0.2488979	0.966638	0.0395905
cg05928342	0.2383963	0.0597281	0.951526	0.0423236
cg08846870	0.2889722	0.0977736	0.854064	0.0247506
cg07128624	0.5566127	0.2567113	1.206872	0.1378703
cg07534448	0.3972314	0.1520858	1.037525	0.0594622
cg14377523	6.8143468	0.4959076	93.63704	0.1511825
cg09269454	21.721976	0.974406	484.2379	0.0519444
cg15584435	879435868	0.0206245	3.75E+19	0.099114
cg21519058	0.3826046	0.1395187	1.049223	0.0619561
cg23889684	1.328288	0.708574	2.49	0.3759084
cg14068788	0.1411028	0.0314322	0.633426	0.0105903
cg04879750	2.1821692	0.9765511	4.876204	0.057155
cg08931515	0.2649515	0.0897186	0.782439	0.0162158
cg14020762	0.6354372	0.1530673	2.637927	0.532394
cg16787483	1.6868161	0.6052342	4.701236	0.3174189
cg24463290	1.4331829	0.1216698	16.88186	0.7748742
cg09609051	1.387945	0.5413847	3.558267	0.4949332
cg26304297	2.5424584	0.8117389	7.963268	0.1091773
cg08936817	0.5108946	0.2285613	1.141984	0.1017446
cg24968629	85.187348	0.0537851	134923.6	0.2370315
cg14339848	0.2575689	0.0518935	1.278421	0.0970208

cg23622878	0.2382624	0.0880532	0.644712	0.0047393
cg14744022	1.6307481	0.0791055	33.61763	0.751431
cg08649201	1.7457529	0.7375561	4.132097	0.2049817
cg16506910	0.3474799	0.0892664	1.352606	0.1274102
cg19917744	5.4382336	0.2981382	99.1969	0.253005
cg22435132	0.3542205	0.1664063	0.754011	0.0070927
cg04563438	0.0221352	0.0012167	0.402687	0.0100384
cg07777703	0.0003385	2.68E-06	0.042781	0.0012104
cg10588362	0.3874072	0.1649776	0.909726	0.0294666
cg00484358	3.2395294	0.8392212	12.50511	0.0880788
cg21430465	0.739609	0.3994763	1.369346	0.3371675
cg04550823	1.7136207	0.7168641	4.096308	0.2257661
cg13291283	1.1400556	0.6162418	2.109118	0.6762375
cg14609289	0.278218	0.0374839	2.065025	0.2109599
cg04666975	2.0873988	1.0398275	4.190343	0.0384704
cg10773266	1.6838027	0.6150316	4.609831	0.3105769
cg13296485	0.0087994	2.04E-06	37.976	0.2677247
cg07743702	0.1247341	0.029847	0.521278	0.0043334
cg01814898	0.2564315	0.0979468	0.671355	0.0055815
cg25141995	0.2696655	0.1116757	0.651166	0.0035716
cg08835113	2.1254916	0.9849389	4.586796	0.0546946
cg06634914	1.6759046	0.8512648	3.299392	0.1351678
cg09580393	0.130728	0.0171424	0.996931	0.0496543
cg17952719	0.2383029	0.0820948	0.69174	0.0083449
cg11348701	0.510009	0.2367402	1.098712	0.0855138
cg07696985	0.3567746	0.1219992	1.043352	0.0597748
cg26978976	1.2096256	0.4776303	3.063444	0.6881178
cg11706226	1.3529338	0.5312318	3.445633	0.5262447
cg24170090	6.25E-09	2.95E-14	0.001322	0.0025329
cg18351939	1.5237171	0.7807583	2.973665	0.2170137
cg06976485	0.1665445	0.0226557	1.224288	0.0782141
cg07235218	0.1026468	0.0223186	0.472089	0.0034547
cg20149625	0.006758	0.0002752	0.165974	0.0022166
cg20761844	4.1538669	0.4046076	42.64529	0.2307379
cg12405421	2.3950896	0.6753305	8.494292	0.1763055
cg10924779	0.6291285	0.2929044	1.351303	0.2347959
cg17891820	0.3417206	0.1345545	0.867849	0.023944
cg23696618	0.8860838	0.4545971	1.727122	0.7224566
cg24730915	4.329053	0.8820779	21.24608	0.0710163
cg07949060	0.5853086	0.2249579	1.52289	0.272272
cg13321967	0.2789104	0.1008701	0.7712	0.0138691
cg17662683	0.2654553	0.0727676	0.968378	0.0445766
cg14950169	1.0644645	0.5491434	2.063367	0.8532326
cg27297576	1.4747218	0.7994588	2.720346	0.2136814
cg05539369	0.4488195	0.1622814	1.241294	0.1227074
cg08744177	1.2997325	0.6674922	2.530823	0.4406735
cg10911865	0.2463329	0.0828615	0.732305	0.0117209
cg13209683	0.2071861	0.0711135	0.603628	0.0039117
cg06068935	0.1753737	0.0158115	1.94516	0.1561893
cg12991064	7.6313072	0.0402521	1446.802	0.4475888
cg16482344	0.2446665	0.0867969	0.689676	0.0077532
cg21040360	0.8816344	0.064408	12.06806	0.9248186
cg10150530	1.9413973	0.9666394	3.8991	0.0622375
cg24379915	0.4865074	0.1610061	1.470065	0.2015892
cg07245678	1.9350149	0.7335408	5.104396	0.1822583
cg11152364	0.7319319	0.3608277	1.48471	0.3871643
cg18179931	2.36985	0.9496594	5.913898	0.0644202
cg17369406	0.3665106	0.1662102	0.808193	0.0128544

cg21914290	2.2321208	0.8524175	5.84498	0.1020805
cg21575706	0.4540516	0.1896562	1.087035	0.0762946
cg19433091	1.1019173	0.5445829	2.229636	0.7872417
cg21131024	1.5337937	0.8876786	2.650197	0.1252843
cg05419696	3.071E+10	0.0102408	9.21E+22	0.0994732
cg01321106	0.6227773	0.1930862	2.008697	0.4280125
cg05222986	470919.85	17.755206	1.25E+10	0.011954
cg03666350	0.5184862	0.2582545	1.040942	0.0647291
cg21110456	2.1160472	0.8794375	5.0915	0.0942917
cg02171718	0.72083	0.3033379	1.712928	0.4585387
cg24345062	1.8466334	0.9480713	3.596834	0.0713576
cg15828364	1.9222905	0.6216175	5.944493	0.2565554
cg12192749	0.372949	0.1065123	1.305867	0.1229314
cg12899423	2.7940608	0.9097835	8.580916	0.0726842
cg15028507	0.2946755	0.1010072	0.859677	0.0253031
cg06698844	0.4516831	0.1863853	1.094601	0.0784386
cg24137448	1.9673964	0.6547175	5.911937	0.2280233
cg24444188	0.9433431	0.4038637	2.203457	0.8928096
cg09495859	10378.123	8.279088	13009336	0.0110629
cg10594245	1.7172308	0.6721417	4.387292	0.2585486
cg20992429	0.4232477	0.1718176	1.042609	0.0615882
cg26270899	2.6324922	0.9556222	7.251836	0.0611837
cg12578567	12759208	2.40E-09	6.78E+22	0.3758047
cg02306995	2.6635478	1.1489073	6.174987	0.0223995
cg09365240	0.0957321	0.0133936	0.684257	0.0193836
cg25110757	0.2005049	0.0096586	4.162305	0.2990767
cg19958750	0.1861215	0.0723096	0.479068	0.0004911
cg22202689	0.5873416	0.2287682	1.507946	0.2686587
cg22506841	2.8647129	1.2734569	6.444333	0.0109477
cg10434075	4.52E-28	3.28E-52	0.000623	0.0264035
cg05023214	0.5830706	0.127443	2.667635	0.4868689
cg09077934	1.7120164	0.7364521	3.979892	0.211586
cg22631616	0.4042441	0.1835955	0.890073	0.0245036
cg00067768	0.4377332	0.1991777	0.962007	0.0397463
cg21990700	1.3446846	0.6931554	2.608617	0.3810536
cg03688640	3.64E-10	1.29E-21	102.1576	0.1060982
cg21052415	2.8219171	0.7991352	9.964792	0.1070426
cg19985870	1.1371916	0.4500154	2.87369	0.7857691
cg22869025	2.1694647	0.463401	10.1566	0.3254312
cg12810084	2.156581	0.6581022	7.06705	0.2044173
cg18099096	0.6958545	0.1797999	2.69307	0.5994645
cg25064052	1.3206716	0.432324	4.034413	0.6254321
cg21197594	3.0853217	0.6110499	15.57845	0.1726508
cg22157629	3.0719915	0.0533325	176.9488	0.5873592
cg26333564	0.1328709	0.0348237	0.506974	0.0031346
cg09706586	3.0657661	0.6921724	13.57887	0.1401003
cg15965190	0.5152283	0.2671899	0.993526	0.0477767
cg16736279	3.7846372	1.540307	9.299106	0.0037104
cg14868574	0.01805	0.0005926	0.54979	0.0212699
cg11853697	1.0865566	0.4167092	2.833163	0.8651915
cg04523524	1.3932303	0.7176425	2.704815	0.3272108
cg21868798	0.5022251	0.1800706	1.400728	0.1881673
cg16470529	6.70E-05	6.03E-18	7.44E+08	0.530601
cg25988214	0.5948387	0.3236375	1.093301	0.0943809
cg01770362	0.2698824	0.1101344	0.661342	0.0041812
cg15645605	2.30E-11	6.18E-27	85845.34	0.1805853
cg12864721	0.305659	0.1393161	0.670615	0.00311
cg23510258	1.9822103	0.6209076	6.328088	0.2479759

cg22232327	1.5282448	0.7973264	2.929204	0.2013693
cg23206630	2.1605035	0.7812481	5.974767	0.1377279
cg21454485	1.7182153	0.8010947	3.685287	0.1644314
cg01004363	0.4503334	0.1432005	1.416197	0.1723474
cg06143615	3.1267853	0.7352907	13.29649	0.1226825
cg00853742	0.3390611	0.1503363	0.764702	0.0091484
cg06297571	0.4983469	0.27622	0.899101	0.02071
cg02294764	2.1201089	1.1240257	3.998896	0.020282
cg21609640	0.385049	0.1470223	1.008437	0.0520329
cg03770907	0.2482829	0.0706922	0.872012	0.0297325
cg07516252	2.0720192	0.7097469	6.049006	0.1826104
cg19175742	0.5049255	0.1568378	1.625563	0.2519973
cg17274681	2.75E-17	3.88E-29	1.95E-05	0.0061656
cg00194886	0.3104907	0.0001449	665.4035	0.7650347
cg14994947	1.1303361	0.2599726	4.914593	0.8702158
cg10559742	4.2479559	0.9395424	19.20629	0.0602508
cg14561985	0.2552001	0.101513	0.641564	0.0036887
cg22355517	0.0978671	0.0117201	0.817227	0.031844
cg12282552	3.7400749	0.5665818	24.68869	0.1707065
cg25567674	0.1185055	0.0210546	0.667006	0.0155494
cg24131452	0.30509	0.1474268	0.631363	0.0013776
cg01829241	0.2449425	0.093885	0.639046	0.0040382
cg12743248	1.1201213	0.5159976	2.431546	0.7742295
cg02387701	0.1697442	0.0487528	0.591004	0.0053323
cg16323293	0.220982	0.1007518	0.484687	0.000165
cg02638589	1.394141	0.3839689	5.061944	0.6135205
cg26388450	1.371E+12	1.48E-15	1.27E+39	0.3776962
cg23306526	3.9987375	1.6964013	9.425778	0.0015349
cg15905329	0.4178134	0.1481543	1.178286	0.09898
cg27140058	2.2876994	1.0124057	5.169438	0.0466343
cg05342835	0.382702	0.1593141	0.919321	0.0317064
cg21016445	0.2395153	0.0757297	0.757531	0.014989
cg16524818	5.2868933	0.6282055	44.49379	0.1254703
cg04339692	3.1968809	0.6062242	16.85853	0.1706964
cg00036258	5.3042519	0.0513113	548.3212	0.4807863
cg23773809	2.6239635	0.3116541	22.09239	0.3748378
cg00912772	0.9109729	0.3577974	2.31939	0.8449617
cg17306637	0.2958386	0.1461576	0.598809	0.0007108
cg12883980	4.7663841	1.1792285	19.26549	0.0284297
cg06996739	2.7673238	0.5499246	13.92569	0.2169621
cg21563049	0.5231025	0.2090839	1.308739	0.1660824
cg06836406	0.352587	0.1133269	1.096982	0.0718409
cg13487666	0.1960123	0.0507981	0.756343	0.0180152
cg04450459	0.11839	0.0228177	0.614269	0.0110826
cg03717931	3.0134535	1.0292713	8.822652	0.0441564
cg09613130	0.9200891	0.4597072	1.841529	0.814015
cg03994721	2.6097154	0.8637261	7.885155	0.0890767
cg02324291	0.5241406	0.2494871	1.101153	0.0880899
cg24710655	1.3745777	0.3371257	5.604627	0.6572803
cg18279326	3.3284308	1.3965858	7.932525	0.0066515
cg16565063	422.10589	0.0593046	3004378	0.1816329
cg05922771	9.1420684	1.6055325	52.05588	0.012651
cg03328299	0.524391	0.1996177	1.377563	0.1902136
cg11876705	1.7135453	0.7820281	3.754644	0.1784154
cg14446504	0.0024451	2.69E-07	22.25159	0.1960306
cg11910652	0.4955144	0.2037801	1.2049	0.1214266
cg07964097	2.4928648	0.8251169	7.531509	0.1054031
cg11165254	0.528992	0.2521835	1.109638	0.0920421

cg03498173	44.231856	0.9517849	2055.567	0.0530229
cg16674484	1.5402566	0.3664164	6.474575	0.5554692
cg12608633	0.2460751	0.1059705	0.571413	0.0011065
cg23908019	4.8045129	2.025405	11.3969	0.0003689
cg22537343	0.4499452	0.2012604	1.005914	0.051703
cg23277693	1.5595204	0.4790536	5.076893	0.4605718
cg11770816	1.2983861	0.6512099	2.58873	0.4582839
cg26180191	5.2308872	2.3358266	11.71413	5.76E-05
cg01658421	1.5361129	0.6988783	3.376329	0.2853839
cg15884223	0.1550828	0.0592508	0.405913	0.0001467
cg07099073	0.4084374	0.1951518	0.854827	0.017491
cg26130090	4.2191664	0.2814752	63.2431	0.2973123
cg00438775	0.1074661	1.55E-11	7.44E+08	0.8470001
cg17321883	0.2470056	0.0728912	0.837025	0.0247256
cg06912966	1.9405517	1.0471407	3.596213	0.0351774
cg24267038	2.5978699	0.4767403	14.1564	0.2697577
cg14453403	1.6504659	0.7700115	3.537659	0.1977121
cg20703523	1565958.9	1.76E-15	1.39E+27	0.5622049
cg10703826	2.1949292	1.13245	4.25424	0.0198934
cg17786845	0.5210653	0.2513291	1.080293	0.0797134
cg09206294	0.3841913	0.1545404	0.955109	0.0395122
cg21901160	0.4560445	0.2264436	0.918447	0.0279402
cg09203880	12.790565	0.0132493	12347.67	0.46731
cg20281912	0.6571954	0.2369444	1.822815	0.4199625
cg16455376	0.8345108	0.1370038	5.08313	0.8444212
cg20215257	0.268851	0.1013886	0.712909	0.0082885
cg18857871	3.2559339	0.3057769	34.66941	0.3279998
cg05201956	5.52E-44	9.33E-84	0.000327	0.0330295
cg04310824	1.4186952	0.5201514	3.869443	0.4945002
cg02876066	38.874825	0.5939965	2544.21	0.0861988
cg18282393	2.6162612	0.3387615	20.20544	0.3564697
cg15282973	0.3519527	0.1000706	1.237833	0.1036425
cg02341645	0.1575869	0.0466881	0.531905	0.0029101
cg07546062	0.9040965	0.4541358	1.799881	0.7741223
cg15439466	15481.982	3.33E-29	7.19E+36	0.8015181
cg06068373	0.1980647	0.0814077	0.481891	0.000358
cg24255201	2.1643243	0.9611886	4.873445	0.0622683
cg25419914	7.2983049	1.7546768	30.35616	0.0062733
cg07076845	1.7496603	0.5616132	5.450925	0.3346085
cg25404375	0.0870431	0.0081337	0.93149	0.043524
cg22748407	0.0958791	0.0140786	0.652964	0.0166012
cg02743713	2.5341047	0.889044	7.223137	0.081877
cg01271812	2.9400686	0.7234666	11.94803	0.1316875
cg06531916	0.4720125	0.2296831	0.970014	0.0410717
cg20674521	2.1484118	0.7669022	6.018595	0.1456668
cg17326769	2.4428369	1.1647013	5.12359	0.018108
cg25762974	2.5512305	0.5882962	11.06378	0.2108569
cg04341594	1.85E+22	2.16E-18	1.58E+62	0.274443
cg08703522	0.9242898	0.1169944	7.302156	0.9404881
cg19954000	0.330994	0.1306603	0.838487	0.0197317
cg17047890	5.6235731	0.3683176	85.86224	0.2143207
cg16078597	0.1138392	0.0001763	73.50565	0.5103929
cg20801532	2.0461631	0.7016872	5.966737	0.1897973
cg04598683	5.66E-12	5.97E-25	53.69792	0.0893793
cg00529422	3.7932773	0.0140368	1025.086	0.6407286
cg05579549	3.2681282	1.1597118	9.209755	0.0250733
cg10334489	2.158944	0.4948802	9.41852	0.3058308
cg18461678	3.8951797	0.9662631	15.70217	0.055913

cg09327855	3.0301174	0.3802347	24.14722	0.2951664
cg18997983	5.7259803	1.1754183	27.89377	0.0307706
cg12159992	1.6807544	0.5183983	5.449354	0.3869281
cg18165914	0.410197	0.1629139	1.032825	0.0585695
cg06996175	0.121748	0.0314528	0.471264	0.0022928
cg08815652	3.13E-13	4.69E-25	0.209085	0.0382074
cg21770800	1.6784038	0.8410126	3.34958	0.1418771
cg05431947	1.0372699	0.4059028	2.650706	0.9390684
cg26394687	2.2265513	0.9218978	5.377527	0.0752057
cg00832476	1.6149557	0.5465647	4.771772	0.385887
cg09926747	0.2755388	0.1038752	0.730893	0.0096032
cg08528984	0.2425152	0.0702156	0.837615	0.0250806
cg12573002	1.05129	0.3888236	2.842447	0.9214869
cg17399362	2.1995337	1.1917563	4.059512	0.0117011
cg01789576	0.1195958	0.0335716	0.426049	0.001052
cg27490198	0.4894568	4.08E-05	5866.059	0.88147
cg24641276	2.9054668	1.3329551	6.3331	0.0072992
cg03639249	1.879231	0.5092856	6.934241	0.3436168
cg15596647	0.2430939	0.0804566	0.734491	0.0121784
cg23616524	0.1197527	0.0391358	0.366434	0.0001997
cg09509952	0.8496146	0.383446	1.88252	0.6880584
cg14462758	0.5748293	0.2131916	1.549914	0.2739202
cg02502849	428732171	0.1423581	1.29E+18	0.0742766
cg15396434	0.9930194	0.4469467	2.206276	0.9862784
cg26678970	1.387836	0.7435787	2.590296	0.303294
cg08839858	2.1658967	0.8376614	5.600245	0.1108261
cg08910550	1.8139003	0.9170334	3.587911	0.0870647
cg00734813	0.0825423	0.0010317	6.603932	0.2645602
cg22694635	1.884635	0.678551	5.234462	0.2240163
cg06259833	1.0926868	0.5355424	2.229449	0.8075236
cg05842113	0.093855	2.18E-05	404.7797	0.5795245
cg09555736	0.481426	0.2408466	0.962318	0.0385779
cg02503299	0.1537028	0.0059058	4.000206	0.2600658
cg24048517	0.1934635	0.0608215	0.615376	0.0053969
cg17043823	253781.82	4.05E-11	1.59E+21	0.5025175
cg01797036	2.1097983	0.7897474	5.636295	0.1364468
cg04757991	0.5023131	0.2086247	1.209437	0.1245846
cg20222376	0.09211	0.0026308	3.224986	0.1886687
cg04565473	2.1428106	0.7287322	6.300857	0.1660784
cg14192259	0.912217	0.3812853	2.18246	0.8364539
cg14453641	34.751189	0.5269789	2291.639	0.0968689
cg26023748	2.546109	1.0607878	6.111185	0.0364328
cg13470920	1.57657	0.6247275	3.978652	0.3350946
cg18588794	18.243881	0.1354964	2456.444	0.2456886
cg21565823	2.3834883	0.4628185	12.27483	0.2989607
cg14341449	0.1070815	2.26E-14	5.07E+11	0.8807405
cg12904298	0.1842674	0.039437	0.860981	0.0315349
cg14327296	1.3145164	0.5371097	3.217133	0.549269
cg27155504	0.1434237	0.0337863	0.608836	0.0084719
cg27044455	0.4265791	0.1580394	1.15142	0.0926357
cg15964523	7.2931981	0.770325	69.04974	0.0831945
cg14442746	1.9713533	0.2291136	16.96204	0.5365226
cg21156263	0.3914646	0.1886977	0.812116	0.0117718
cg07624742	0.098226	0.0285425	0.338034	0.0002332
cg03432814	0.3045971	0.1341445	0.691638	0.0044953
cg01262290	0.7149038	0.3115533	1.640449	0.4283881
cg21618273	2.8898312	0.6541958	12.76548	0.1614842
cg23184638	0.9201838	0.4265537	1.985068	0.832066

cg01157280	0.2499662	0.1076169	0.580607	0.0012624
cg07616394	1.3797226	0.6978523	2.727847	0.3546836
cg01416043	1.6777483	0.7661986	3.673773	0.1956677
cg24036280	1.375647	0.6102029	3.101271	0.441916
cg13669036	2.3608118	0.5152261	10.81745	0.2686934
cg25630676	4.15E-57	4.47E-91	3.86E-23	0.0011409
cg12453631	2.9628514	0.8469354	10.365	0.0891403
cg10823693	1.2133741	0.5894805	2.497584	0.5995259
cg26664107	2.7636625	1.3044166	5.855361	0.007961
cg02085294	0.2738895	0.1236245	0.606801	0.0014187
cg05944137	3.1137362	1.4556516	6.66049	0.0034142
cg24255327	100.16396	1.7214988	5827.956	0.0262861
cg24698488	0.4998724	0.1772423	1.409779	0.1899383
cg26131194	0.0378162	0.0006825	2.095216	0.1098509
cg13504245	1.1071226	0.6306842	1.943477	0.7230012
cg13286614	3.3958334	1.2003134	9.607228	0.0212189
cg06716182	2.2356589	0.9554509	5.231217	0.0636111
cg14911689	2.0839342	0.7435648	5.840488	0.1625799
cg23924887	0.0023851	2.81E-07	20.25554	0.1908028
cg05016953	0.3663844	0.1360771	0.986482	0.0469338
cg25270899	0.1947422	0.0317564	1.194234	0.0770389
cg00080060	5.4407212	2.3113793	12.80683	0.0001052
cg07217954	0.1892071	0.0504176	0.710055	0.0136089
cg24797574	1.3832969	0.3625297	5.278216	0.6348574
cg21404906	87.707699	0.004701	1636392	0.3725578
cg17197065	0.5334006	0.2276222	1.249949	0.1480421
cg19830983	0.3350358	0.1678081	0.668913	0.0019365
cg24203758	0.7817409	0.3614665	1.690665	0.5315377
cg16971889	1.0838719	0.3586222	3.275811	0.8865092
cg25050447	0.1788908	0.0636744	0.502587	0.0010934
cg02029242	0.6644212	0.2292391	1.925742	0.4514474
cg23468927	1.811564	0.402666	8.150089	0.4386865
cg09528501	0.3896253	0.1620421	0.936842	0.0352293
cg00917154	1.0624593	0.5524927	2.043139	0.8558985
cg10366018	14204.168	0.0106112	1.9E+10	0.184049
cg01090161	2.0904764	0.7939484	5.504251	0.135479
cg12696488	0.7235943	0.3496433	1.497494	0.3833019
cg02083559	0.0489122	0.0018011	1.32832	0.0732255
cg00990690	1.8254189	0.6123795	5.441322	0.2801686
cg20603222	0.4052972	0.1873655	0.876713	0.021779
cg05266781	0.026079	0.0008524	0.797897	0.0366788
cg05307752	1.7216951	0.6179973	4.796516	0.2986561
cg13883366	0.1360292	0.0264468	0.699665	0.0169686
cg08935238	1.7019593	0.5949977	4.868364	0.3213369
cg10211873	3.2892299	0.646016	16.74731	0.1516281
cg24050519	10.132313	1.238927	82.86507	0.0307896
cg10511758	12.90859	0.5253641	317.1738	0.1173676
cg17765751	7.18E-05	4.09E-09	1.261174	0.0556927
cg10933428	2.1593271	0.9311408	5.007507	0.0728576
cg04915494	3.91E-06	1.92E-10	0.079924	0.0139347
cg00159243	1.293004	0.5186137	3.223709	0.5814277
cg09338645	0.0083792	0.000138	0.508746	0.0224575
cg01933695	0.0189048	0.0020017	0.178546	0.0005325
cg12673499	3.5319318	0.8688488	14.35755	0.0778182
cg13213755	0.6448182	0.2831068	1.46867	0.2961237
cg25369248	1.6072087	0.5444592	4.744377	0.3902556
cg10045137	0.8421478	0.2356581	3.009499	0.7914786
cg00043004	0.32253	0.1120693	0.928225	0.0358998

cg20959480	27.045905	0.6451609	1133.796	0.0836239
cg13817545	1.9274234	0.6201085	5.990825	0.2567612
cg05298922	1.420551	0.7654953	2.636156	0.2657829
cg03738253	46.751034	0.9121604	2396.135	0.0555958
cg14604871	2.473496	0.0059961	1020.354	0.7681922
cg21177626	2.3559137	0.8817344	6.294786	0.0874592
cg10257049	0.2575566	0.0744034	0.891564	0.0322636
cg02937055	0.4331084	0.1975532	0.949531	0.0366836
cg21477033	2.31E-06	2.85E-10	0.018689	0.0047031
cg26446913	0.367095	0.1340577	1.005229	0.051198
cg06741399	2.2310506	0.7895465	6.304362	0.1299963
cg26870720	5.4310251	8.97E-28	3.29E+28	0.9586527
cg22383924	1.8020208	0.6368442	5.099016	0.2671293
cg18070458	0.7974073	0.3215484	1.977489	0.6251557
cg10092374	0.498068	0.1037449	2.391171	0.3838568
cg13441683	6.40E+36	8.77E-12	4.67E+84	0.1317642
cg04745820	0.1964524	0.0640524	0.602531	0.0044278
cg10505630	7.0749566	1.7171446	29.15014	0.0067613
cg12661316	2.2011375	1.1681091	4.147734	0.0146612
cg02514888	1.7030942	0.6207792	4.672402	0.3011194
cg21692516	0.178917	0.0312745	1.023559	0.0531382
cg26839512	1.3256136	0.623216	2.819651	0.4641703
cg16339797	2.3568082	0.790626	7.025502	0.1239523
cg16725948	0.4339608	0.1854376	1.015554	0.0543064
cg20707970	7.9678673	2.022963	31.38313	0.0030042
cg21769117	1.4591329	0.4961216	4.291425	0.4924111
cg19896129	0.3875175	0.1607536	0.934161	0.0347146
cg25400746	0.1681039	0.0458362	0.61652	0.0071571
cg16050468	0.4705715	0.200274	1.105673	0.0837217
cg11199137	1.0001274	0.4721659	2.118439	0.9997345
cg07613945	1.2624682	0.5830265	2.733711	0.5543429
cg26841068	0.1099779	0.0220496	0.548542	0.0070949
cg14437534	2.0603351	0.8317946	5.1034	0.1182873
cg04277470	1.72E+30	2.471E+09	1.20E+51	0.0044658
cg03196265	0.7077716	0.1822641	2.748432	0.6175434
cg00562430	0.3057185	0.0779637	1.198812	0.089156
cg02183025	2.0165278	0.8696059	4.676124	0.1021767
cg01522446	3811826	1478.9136	9.82E+09	0.000156
cg04767841	1.6706971	0.3304524	8.446689	0.5347692
cg09502464	0.3415591	0.173013	0.674299	0.0019644
cg05959170	1.8314021	0.7716301	4.346686	0.1700363
cg27183791	2.3559178	0.7322847	7.579496	0.150622
cg18124488	0.5682454	0.2430237	1.328689	0.1921674
cg22197050	2.2280034	0.8374284	5.92767	0.1085826
cg18385671	0.3023902	0.1326294	0.689438	0.0044503
cg06476549	2.9186296	0.7511369	11.34067	0.1219289
cg04348643	0.2093113	0.0879046	0.498395	0.0004106
cg15874302	1.142345	0.396969	3.287289	0.805081
cg07038400	0.3305945	0.1509288	0.724134	0.0056608
cg10906826	1.8037542	0.786987	4.134159	0.1633462
cg21024311	0.3762524	0.1797479	0.78758	0.0094996
cg12024104	0.5006625	0.2086743	1.201216	0.1212912
cg12179661	0.0705706	0.0164596	0.302572	0.0003577
cg14239983	0.1129234	0.0334536	0.381175	0.0004417
cg08345719	0.1223507	0.0358741	0.417285	0.0007903
cg05506011	0.2210945	0.0813124	0.601173	0.003106
cg07471614	0.4153143	0.1592745	1.082948	0.0723353
cg01011898	0.0732731	0.0108628	0.494249	0.0072845

cg20235658	3.0132413	1.3647701	6.652859	0.0063424
cg02326806	1.7562454	0.6694554	4.607324	0.2524273
cg23301925	0.0472827	0.0029418	0.75996	0.0312648
cg15747733	0.2365083	0.0624171	0.896167	0.0339
cg23754320	2.1319921	1.0443256	4.352465	0.0376107
cg14368080	8.4809172	0.8981763	80.07999	0.0620108
cg01673307	2.0097991	1.0450535	3.865154	0.0364351
cg13698613	0.2009704	0.0268194	1.505965	0.1184007
cg22315164	0.3087578	0.1089554	0.874957	0.027014
cg22090592	0.2606719	0.0874223	0.777259	0.0158645
cg23251761	0.2139375	0.0835059	0.548096	0.0013149
cg14619064	2.6966125	0.8692113	8.365882	0.0859229
cg05800339	4.6123332	0.6500857	32.72433	0.1262184
cg16755067	0.4752215	0.1115595	2.02435	0.3143344
cg12536880	0.6077593	0.2791067	1.323406	0.2097617
cg25457331	0.1190886	0.0323642	0.438203	0.0013685
cg16658473	0.5124847	0.1923656	1.365319	0.1811842
cg22041590	0.5074703	0.2237891	1.150754	0.1044141
cg03500646	2.4553481	0.9303621	6.479987	0.0696495
cg03962451	0.4309109	0.1841373	1.008401	0.0522972
cg14879760	2.0931577	1.1352189	3.85944	0.0179703
cg21456301	0.3671051	0.1634393	0.824564	0.0152165
cg11688553	0.3020746	0.1313871	0.694506	0.0048292
cg03612413	1.434367	0.7123457	2.888217	0.3124333
cg02872373	3.952223	1.1973057	13.04601	0.0241016
cg26918754	2.1477773	0.7857931	5.870435	0.1362048
cg01337207	0.2284054	0.0841359	0.620057	0.0037561
cg16747785	1.6667048	0.1673554	16.59884	0.6631191
cg11277156	0.3514211	0.1398766	0.882898	0.026085
cg08023442	0.6676836	0.3392823	1.313954	0.2422145
cg04788418	1.5250383	0.7536812	3.085843	0.2405646
cg00368973	0.1890689	0.0387103	0.92345	0.0395536
cg18645081	1.8063428	0.7844237	4.159581	0.164703
cg26984624	1.6271325	0.6388042	4.144557	0.3074891
cg25527090	3.5176981	1.3095627	9.449108	0.0125988
cg13849525	2.168805	0.9352587	5.02932	0.0712317
cg07595906	2.4950248	0.8484334	7.337228	0.0966512
cg15242771	8.314E+18	1.36E-09	5.07E+46	0.1820055
cg09508934	1.6626073	0.8038135	3.438936	0.1703699
cg23912509	0.3872204	0.1371945	1.092898	0.0731069
cg07123481	2.1327436	0.370956	12.26182	0.3960324
cg11813073	3.230898	1.1815643	8.834646	0.0223106
cg10654803	0.6840607	0.2551127	1.834244	0.4505364
cg15068180	0.6054613	0.2993139	1.224746	0.162731
cg00085123	2.7276944	0.8016081	9.281738	0.108266
cg09365529	0.4416322	0.2099826	0.928834	0.031194
cg13636189	0.5349845	0.2872799	0.99627	0.048639
cg27372452	1.2171685	0.4031291	3.674999	0.727407
cg24690479	0.4917469	0.1824602	1.325303	0.160561
cg08864310	0.5430907	0.2379163	1.239711	0.1471434
cg11854777	1.6707076	0.7340935	3.802327	0.2212413
cg20784391	0.5514667	0.2661189	1.14278	0.1093861
cg22280820	1.7513604	0.6498121	4.720231	0.267946
cg24883899	2.0290599	0.0800875	51.40735	0.6678784
cg16929393	0.3773727	0.1739253	0.818801	0.0136707
cg18628449	3.096915	1.1094137	8.645001	0.0309121
cg22901100	0.0022394	0.0001128	0.044438	6.27E-05
cg02025192	87.385783	2.5031009	3050.726	0.013658

cg08012844	0.5521344	0.2415903	1.261857	0.1589993
cg03308092	2.6810241	0.5825896	12.33783	0.2054189
cg08426951	0.1460977	0.0003304	64.604	0.5360078
cg06379754	0.0884183	0.0070361	1.111099	0.0603287
cg11308697	1.806435	0.167809	19.44596	0.6257262
cg05867134	0.085597	0.0201782	0.363107	0.000856
cg15450160	0.1840665	0.0260622	1.299985	0.0897118
cg03925642	0.3298103	0.1272678	0.854693	0.0224218
cg10087973	0.3469282	0.1449619	0.830282	0.0174211
cg15393816	1.3666134	0.4724542	3.953044	0.5643809
cg24125901	1.3152828	0.6201753	2.789483	0.4749452
cg10875499	0.421852	0.2044593	0.870389	0.0195117
cg25258843	2.1874416	0.9015145	5.307625	0.0835023
cg04219726	0.1765867	0.0587393	0.530868	0.0020182
cg06175447	2.0395634	0.4702162	8.846609	0.3410734
cg20073553	1.4279416	0.568889	3.584209	0.4480507
cg12786605	1.1515949	0.5581348	2.376076	0.7025012
cg00928816	0.410154	0.1218326	1.380799	0.1501542
cg00735591	3.712E+10	0.0001333	1.03E+25	0.1515236
cg23527974	0.6463137	0.0527427	7.91998	0.7328127
cg16274098	7.50E-06	2.09E-11	2.688964	0.0705484
cg22706007	0.0540508	0.0011221	2.603634	0.1399627
cg22031736	0.0287448	2.54E-28	3.26E+24	0.907687
cg11413800	2.43E-06	1.03E-13	57.12469	0.1354834
cg14734340	0.3465049	0.1451488	0.82719	0.0169724
cg16590190	2.3164769	1.1791503	4.55079	0.014757
cg22580629	0.0628285	0.008329	0.473935	0.00727
cg02686793	0.2221996	0.1107273	0.445894	2.31E-05
cg13698168	0.1281974	0.010028	1.638867	0.114108
cg23242509	1.4380619	0.544748	3.796291	0.4632419
cg01395047	1.6672315	0.409709	6.784476	0.4753221
cg26146900	0.4229623	0.2022509	0.884531	0.0222588
cg16473288	0.6410246	0.334763	1.227473	0.1797219
cg17476271	0.2384334	0.0663936	0.856264	0.0279595
cg10698889	2.0991867	0.8233616	5.351943	0.1204376
cg10297473	0.7903372	0.3575151	1.747151	0.5610078
cg07201996	0.2748025	0.1005216	0.751245	0.0118227
cg15357934	0.2003135	0.0315224	1.272922	0.0883451
cg04343928	1.9362926	0.7964596	4.707368	0.1448792
cg08318726	1.6984242	0.8369433	3.446643	0.1423764
cg18181134	5.8628008	0.5633548	61.01383	0.1389204
cg14101494	0.0170463	0.0009182	0.316475	0.0062977
cg14377596	1.7391105	0.6594138	4.586658	0.2633992
cg02359132	0.0100074	1.12E-05	8.909334	0.1839173
cg21813538	0.1848698	0.0568953	0.600697	0.0049907
cg16273075	0.0372792	8.83E-05	15.7455	0.2862706
cg25943276	2.8099276	0.8353827	9.451588	0.0950493
cg17898069	0.3376931	0.1398938	0.815165	0.0157576
cg04569897	0.3233802	0.0393979	2.654325	0.2932199
cg08701843	1.2127598	0.5918153	2.485212	0.5982195
cg25399818	0.1627186	0.0337033	0.785602	0.023799
cg25279553	0.3754647	0.1563657	0.901564	0.0283924
cg10164367	1.7165635	0.7560471	3.897363	0.1965231
cg04075191	0.1146251	0.0268817	0.488769	0.0034175
cg19061690	0.0433134	0.0005195	3.611137	0.1642207
cg15007813	0.5871392	0.2523631	1.366018	0.2164596
cg27250607	4.5390783	1.0234105	20.13193	0.0465456
cg22831425	1.1029759	0.2441121	4.983595	0.8986433

cg19917300	0.3577933	0.1125754	1.137158	0.0814903
cg04391718	2.1574889	0.6141592	7.579075	0.2303344
cg07886914	2.3425243	0.7069478	7.762129	0.1637395
cg05690644	1.6670639	0.9653997	2.878706	0.0667097
cg20517614	1.2231643	0.6060808	2.468534	0.5739311
cg07396581	0.5267457	0.23453	1.183051	0.1204748
cg03920172	656766467	70.881889	6.09E+15	0.0131173
cg24207009	0.6026824	0.2217477	1.638015	0.3209021
cg02234314	0.6558642	0.3405872	1.262989	0.2070866
cg07265015	4.998902	0.3411314	73.25335	0.2400721
cg11328455	0.2816363	0.1110398	0.714329	0.0076215
cg09467020	18.127058	0.0159699	20575.65	0.4195024
cg25996586	0.61932	0.2690528	1.425583	0.2600024
cg11263296	0.9707012	0.4238973	2.222852	0.9439192
cg01379846	0.1969791	0.0384919	1.008024	0.0511321
cg17133056	1.5408848	0.5973822	3.974551	0.3711584
cg22306928	5.8527752	1.123126	30.49967	0.0359205
cg05050657	1.5906643	0.7856721	3.220444	0.1971508
cg06033579	2.7011917	0.2654446	27.4876	0.4012064
cg26237595	0.8466499	0.4498887	1.593319	0.605842
cg08711867	0.0137266	0.0007438	0.253331	0.0039384
cg27651003	2.362E+13	158862.47	3.51E+21	0.0013397
cg13454941	0.4397607	0.2034263	0.950661	0.0367437
cg21346966	0.0318315	0.0039681	0.255347	0.0011746
cg01870976	0.2323809	0.0994839	0.54281	0.0007476
cg23679769	0.0025189	4.98E-05	0.127502	0.0028024
cg17104824	4.7831488	0.9536816	23.98968	0.0571293
cg02633149	8.5424583	0.5814623	125.5001	0.1176999
cg09171565	2.2210747	1.0747375	4.590119	0.0311955
cg06544715	25.801674	0.0464413	14334.8	0.313441
cg25978327	0.3151068	0.155021	0.640508	0.0014184
cg12913090	7.33E-16	1.40E-33	384.6298	0.0941224
cg09335647	0.3830588	0.1226154	1.196701	0.098738
cg07596529	1.1300635	0.4699008	2.717687	0.784772
cg27049993	6.4637057	0.3399404	122.9024	0.2142657
cg07821739	1.1095724	0.3744863	3.287572	0.8511756
cg11545871	2.1566397	0.0037263	1248.189	0.8128031
cg26451499	0.9310231	0.3267274	2.652989	0.8935825
cg08555594	1.1831964	0.062884	22.26247	0.9105479
cg00296501	4051.9437	1.91E-05	8.59E+11	0.3957635
cg26460366	0.4034316	0.1504274	1.081964	0.071317
cg22304592	0.0001134	2.89E-09	4.453604	0.0923358
cg27246147	1.4497046	0.705947	2.977056	0.311776
cg06242449	1.8374456	0.51016	6.617936	0.3520936
cg03507641	0.6720429	0.2993668	1.508657	0.3354097
cg22328786	0.0002203	3.54E-07	0.137137	0.0103112
cg27366007	0.3461799	0.0917322	1.306417	0.1174646
cg09419005	1.9324267	0.5883913	6.346581	0.2775636
cg11149849	1.7522398	0.5615452	5.467671	0.334015
cg09614389	2.7782006	0.6459348	11.94919	0.1698199
cg03218584	4.0661837	1.5566563	10.62139	0.0041924
cg19849557	1.5103912	0.0450085	50.68552	0.818053
cg16243019	3.2116667	1.0134279	10.17813	0.0474089
cg26751588	6.0234515	0.6064168	59.83009	0.1252872
cg05152479	0.5227058	0.2691625	1.01508	0.055395
cg27077773	3.9073639	0.6911809	22.089	0.1230618
cg12633102	2.5963752	0.8921714	7.555907	0.0800117
cg07484727	0.7508167	0.162726	3.464263	0.7133576

cg06119630	0.2970642	0.0969859	0.909896	0.033562
cg15142694	1.2147101	0.3282709	4.494826	0.7707749
cg21123366	0.2752452	0.0408619	1.854049	0.1849721
cg26569144	0.2668037	0.0622118	1.144223	0.0753051
cg19937480	0.8974012	0.3340251	2.410983	0.8300127
cg25488284	0.615439	0.2315853	1.635532	0.3303465
cg09255034	0.1361888	0.0092485	2.00544	0.1462598
cg16205760	7.4750811	0.7358961	75.93033	0.0890008
cg23704023	0.0517851	0.0020064	1.336547	0.0742515
cg27312916	5.7302834	0.4777221	68.73483	0.168451
cg01804664	1.8765504	0.8728852	4.034255	0.1069997
cg04615654	0.0037325	4.95E-06	2.816761	0.0981986
cg03066618	0.1402485	0.0072366	2.71806	0.194006
cg11690886	0.3278872	0.1403291	0.766128	0.0100179
cg09567173	5.086052	1.3613565	19.00158	0.0155764
cg21296087	0.0006704	5.70E-14	7879346	0.536776
cg00645755	2.0903105	0.6169034	7.082791	0.2363464
cg24886563	3.8321863	0.830606	17.68065	0.0850593
cg25103895	0.2406118	0.0303968	1.904612	0.1771462
cg06981439	0.1682827	0.0486553	0.582034	0.0048804
cg15545222	1.54E-24	1.37E-57	1.72E+09	0.157872
cg21083247	0.3334011	0.1548808	0.717689	0.0049855
cg07696485	0.8304378	0.3764482	1.83193	0.6453108
cg23972767	1.8119353	0.8890357	3.692888	0.1017985
cg17932096	5.8513176	1.1422656	29.97369	0.0340443
cg09764761	0.2908034	0.1240769	0.681566	0.0044814
cg21483092	0.6998371	0.2964948	1.651874	0.4153459
cg00502099	2.0374063	0.8740574	4.749144	0.0993086
cg08998375	0.3211466	0.0034828	29.61232	0.6226556
cg12525675	0.5156926	0.2647493	1.004493	0.0515604
cg03869495	6.220569	1.541383	25.10439	0.0102347
cg04813697	1.218518	0.5738715	2.587315	0.6069514
cg09811510	0.884798	0.401902	1.947906	0.7611379
cg11245806	0.1261843	0.0202722	0.785434	0.0264971
cg01767525	2.9518435	1.2989073	6.708239	0.0097558
cg04705952	0.2550611	0.1008682	0.644962	0.0038951
cg07343703	0.2585843	0.1246545	0.536409	0.0002801
cg13450708	0.1470576	0.0204219	1.058957	0.0570284
cg12804063	0.5667893	0.2368415	1.356393	0.2022104
cg18464216	0.4353187	0.044375	4.270478	0.4753064
cg01942106	2.584086	0.8546762	7.812901	0.0926105
cg14440640	1.482577	0.6032403	3.643713	0.390729
cg04522045	1.5598464	0.6644169	3.662039	0.3072432
cg01056889	0.3462001	0.1630097	0.73526	0.0057765
cg12516959	0.6564385	0.311888	1.381623	0.2676047
cg06553114	1.4063313	0.2419368	8.17473	0.7041592
cg19916794	0.2615273	0.0643808	1.062374	0.0607431
cg14845689	3.7422609	1.5693307	8.923879	0.0029173
cg13906689	0.6744335	0.074481	6.107067	0.7260561
cg20752472	0.3873481	0.0740869	2.025169	0.2610898
cg27207470	0.3753546	0.1643179	0.85743	0.0200765
cg18318560	0.0225384	0.0026142	0.194313	0.0005596
cg19611822	0.5151192	0.246439	1.076728	0.0778264
cg27387193	0.2763845	0.0953175	0.80141	0.0179066
cg05682128	0.6165369	0.3036889	1.251668	0.1806875
cg07073120	0.0090152	7.15E-07	113.7018	0.3283639
cg17024944	0.574929	0.2138637	1.545579	0.27263
cg14642374	5360.8023	1.90E-12	1.51E+19	0.6361695

cg12808347	1.160224	0.2467445	5.45552	0.8507503
cg24703168	0.8767012	0.1699457	4.522651	0.8750902
cg13599092	2.6301989	0.9878606	7.002958	0.0529267
cg20148881	1.2501045	0.49271	3.171767	0.6384178
cg17200810	0.4680483	0.2055433	1.065806	0.0705794
cg23271558	0.1767108	0.0135967	2.296638	0.1853161
cg20706526	0.2939251	0.0394408	2.190422	0.2321546
cg20111643	0.0127149	0.000594	0.272179	0.005231
cg27527922	0.2621828	0.0685128	1.003314	0.0505676
cg15084405	0.2009819	0.095277	0.423961	2.52E-05
cg05790451	3.8512872	1.2167794	12.18989	0.0218063
cg00451513	6.46E-16	6.96E-26	6.00E-06	0.0028195
cg19495043	4.404E+19	1.37E-08	1.41E+47	0.1615976
cg18184053	0.3655843	0.1439876	0.928218	0.0342896
cg01636575	0.3349382	0.126808	0.884673	0.0272975
cg12819431	0.5806708	0.3129359	1.077468	0.0848163
cg09276451	0.1974208	0.0801385	0.486345	0.0004203
cg01636582	0.0480676	0.0064286	0.359409	0.0031078
cg13481271	1.68E-06	1.84E-09	0.001534	0.0001319
cg11299459	0.2637886	0.0377065	1.845422	0.1793878
cg11465939	1.638314	0.7714236	3.479376	0.1989186
cg17538280	1.5436564	0.6556112	3.634586	0.3203805
cg14440571	2.4676654	1.0460099	5.821525	0.0391425
cg08993681	2.342866	0.8000723	6.860656	0.1204059
cg17890298	0.5036958	0.2046332	1.239825	0.1356451
cg02135003	0.0303986	0.0005488	1.683894	0.0880928
cg01604539	0.5053776	0.2441884	1.04594	0.0659252
cg07208853	2.7394275	1.4143429	5.305972	0.0028104
cg05230816	5.5788442	1.8927444	16.44359	0.0018281
cg24376339	0.341111	0.1529258	0.760871	0.0085981
cg12710228	0.5587503	0.2674523	1.167318	0.1215258
cg14192037	1.7365282	0.5300842	5.688776	0.361993
cg25653816	0.3425647	0.1575869	0.744672	0.0068487
cg18559739	1.1782953	0.4998087	2.777823	0.707687
cg05998503	0.0767415	0.0165983	0.354811	0.001015
cg02714994	0.4603641	0.1244843	1.702505	0.2450151
cg20909645	6.5318273	0.3121287	136.6897	0.2264561
cg02853948	1.6295555	0.7187837	3.694368	0.2422876
cg06951647	1.2961478	0.4953989	3.391205	0.5970782
cg08079209	0.5405177	0.2654452	1.100639	0.0899482
cg23398047	5.4118985	0.4435301	66.0353	0.1858364
cg08433492	3.5066641	1.0418591	11.80264	0.0427457
cg25526061	1.7054909	0.7611288	3.82156	0.1946714
cg27248148	0.4365433	0.2179722	0.874286	0.0193303
cg14315444	1.2176274	0.5993604	2.473664	0.5861115
cg24896096	1.3716758	0.159353	11.80709	0.7735439
cg27493484	1.66E-21	8.01E-36	3.46E-07	0.0044496
cg26127778	1.1391573	0.4789769	2.709273	0.7681916
cg04531704	1.5879019	0.6480752	3.890648	0.3118594
cg08296288	3.8035238	0.5515389	26.22987	0.1751034
cg01609214	0.4498799	0.2318973	0.872766	0.0181541
cg23763043	6.56E-07	2.62E-15	164.7908	0.1491128
cg06851151	0.9761839	0.3499329	2.723194	0.9632701
cg00055981	0.6072079	0.0854657	4.314026	0.618003
cg20147645	0.2206725	0.0655097	0.743346	0.0147437
cg07729082	2.7715431	0.1875263	40.96198	0.4581748
cg16155207	0.4083556	0.1787222	0.933037	0.0336395
cg23627335	1.8016744	0.9162411	3.542769	0.0879312

cg04183425	1.4063273	0.5313193	3.72235	0.4923392
cg01301885	0.4431184	0.214666	0.914695	0.0277294
cg07311313	1.0907966	0.5372433	2.214708	0.8099296
cg17119387	0.7439206	0.0219714	25.18808	0.8692486
cg05969360	4.6224271	0.4927428	43.36305	0.1801428
cg00343414	4.1665635	1.0005066	17.35146	0.0499187
cg01590844	13107.015	4.97E-31	3.46E+38	0.814634
cg21538208	3.0869418	1.0446578	9.121848	0.0414505
cg10900202	2.3977041	1.0496272	5.477168	0.0379975
cg25572367	0.0507465	0.0069604	0.369979	0.0032722
cg02466801	1.4710371	0.4912659	4.404845	0.4903468
cg24315340	0.197675	0.0882691	0.442685	8.11E-05
cg07568841	1.3836961	0.5172291	3.701677	0.5177315
cg00357551	0.3647658	0.1389481	0.957582	0.0405615
cg05095290	0.0338254	3.77E-06	303.5378	0.4658599
cg12280150	0.3919802	0.1887887	0.813865	0.0119878
cg00867694	0.0242884	0.0014488	0.407185	0.0097494
cg25420398	2.89E-39	1.67E-60	4.98E-18	0.0003754
cg00960395	1.6132273	0.7566283	3.439605	0.2157101
cg13074458	0.5391211	0.2704265	1.07479	0.0792467
cg19759502	0.0932167	0.0133826	0.649305	0.0165732
cg26365925	2.309714	0.7017769	7.601816	0.1684182
cg08270734	0.13261	0.0122517	1.435345	0.0964006
cg00397714	0.8567132	0.408604	1.796256	0.6822358
cg01882535	0.6361828	0.2831103	1.429579	0.2735894
cg16597045	3.961498	1.1953435	13.12883	0.0243323
cg11466369	0.0292314	0.000277	3.08519	0.1372714
cg11236526	0.1085644	0.0074449	1.583123	0.1043827
cg02827112	1.66E-06	4.84E-12	0.570218	0.0407228
cg17233896	0.5098649	0.2221834	1.170034	0.1119627
cg11579646	1.882038	0.7546922	4.693393	0.1750017
cg16709110	0.7509677	0.2164603	2.60534	0.6518185
cg12456539	6.0307211	1.0699707	33.99121	0.0416882
cg12244275	0.2963494	0.1101721	0.797143	0.0159942
cg13889085	0.4409834	0.15696	1.238955	0.1203207
cg05972518	1.8502508	0.4571902	7.487973	0.3883132
cg09855140	2.7317132	0.6011105	12.41412	0.1932509
cg02483593	1.4861897	0.6698472	3.297408	0.3298279
cg18307978	3.1676945	0.7967053	12.59473	0.1015794
cg01907761	0.7448979	0.3711451	1.49503	0.4073489
cg21784396	6.8995178	1.2126648	39.25516	0.0294559
cg04205193	1.63E-11	6.82E-29	3876069	0.2236565
cg16965936	0.0005497	9.13E-06	0.0331	0.0003306
cg12288074	55.423791	1.4301305	2147.913	0.0314214
cg12659370	0.0064913	1.65E-07	255.6846	0.3507895
cg12670403	4.7737804	0.7955489	28.64561	0.0873059
cg00398781	2.7898689	0.8682514	8.964418	0.0849337
cg01699819	2.5882857	0.2679465	25.00209	0.4111644
cg08747889	0.2450452	0.065364	0.918658	0.0369969
cg21029504	0.7207806	0.2959363	1.755529	0.4709754
cg11175473	1.603384	0.6574416	3.91037	0.2993026
cg21751873	0.1908582	0.0164153	2.219085	0.1857813
cg25921502	0.621328	0.2419329	1.595684	0.3227067
cg06932483	5692.106	8.99E-05	3.6E+11	0.3454571
cg01960016	0.3959384	0.0951587	1.647429	0.2027772
cg05101846	0.4175557	0.1845914	0.944533	0.0359952
cg22891707	0.3433305	0.1336605	0.881905	0.0263472
cg24601705	1.3541938	0.4324475	4.240609	0.6026404

cg04580179	2.0250714	0.4255427	9.636905	0.3753396
cg22685566	0.8260928	0.3847031	1.773911	0.6241595
cg19425603	0.219669	0.0890675	0.541774	0.0009994
cg25720969	0.8549454	0.4179428	1.74888	0.6677925
cg08161546	0.6396376	0.2951648	1.386128	0.2574364
cg21025494	0.4225132	0.1457	1.22524	0.1127375
cg08863459	5.46E-09	1.52E-15	0.01963	0.0134979
cg04897439	0.3451682	0.1215049	0.980545	0.0458421
cg23785771	3.5558327	0.9295064	13.60286	0.0638571
cg03475285	1.6454631	0.6932764	3.905439	0.2587731
cg00479101	9.21E-08	1.94E-12	0.004375	0.0031924
cg04042828	1.8745622	0.8304142	4.231603	0.1303735
cg09837314	0.6200242	0.3145512	1.222154	0.1674177
cg01442781	5.9454196	0.9001762	39.26789	0.0642015
cg03342498	0.0154587	0.000245	0.975303	0.0486336
cg17106011	0.0162236	0.0002395	1.098793	0.0553447
cg14590011	1.6334404	0.5351835	4.985444	0.3887454
cg13525448	1.3015756	0.6861251	2.469082	0.4197565
cg06914505	0.3002855	0.0553541	1.628993	0.163202
cg00044665	0.8598814	0.3084976	2.396764	0.772858
cg15056105	0.2707943	0.044612	1.643717	0.1556524
cg19982668	0.242703	0.0623452	0.944817	0.0411693
cg21164131	0.2331719	0.0566597	0.959574	0.0436816
cg10832304	0.0848313	0.0058116	1.238284	0.0712773
cg17137500	1.1999336	0.3448666	4.175065	0.7744898
cg16374999	3.8616524	0.9652008	15.45001	0.0561466
cg04531756	0.7466625	0.2367456	2.354869	0.6181334
cg06212297	2.3906953	0.8686684	6.579524	0.0915289
cg00599163	8.7525926	0.6544353	117.0595	0.1011028
cg12850379	0.3343586	0.1667183	0.670566	0.0020321
cg18042724	1.0053489	0.2315979	4.364144	0.9943175
cg26215849	22.247081	0.0028141	175875.8	0.4981283
cg23238134	10.578412	2.3309564	48.00725	0.0022388
cg23220025	3.10E-10	6.89E-23	1393.012	0.1407628
cg16290431	0.3598826	0.1592089	0.813494	0.0140483
cg18506020	0.5364376	0.1599919	1.798624	0.3129906
cg27186851	2.2834604	0.4717021	11.05399	0.3048251
cg01060282	0.5810575	0.2778508	1.215141	0.1492193
cg10824926	0.6386297	0.3234844	1.260796	0.1962946
cg13060704	0.1804079	0.0464105	0.701286	0.0134281
cg16636104	0.4953333	0.2257564	1.086813	0.07972
cg20130571	0.1650434	0.040518	0.672277	0.0119335
cg23339629	0.0287704	0.0019615	0.421984	0.009608
cg24720038	1.5340217	0.6571954	3.580705	0.322483
cg26108594	0.0042334	0.0001468	0.122062	0.0014412
cg04529078	0.672178	0.3360723	1.344423	0.2613759
cg14185201	1.4893914	0.6518463	3.403082	0.3447087
cg16322302	3.57E-16	3.16E-27	4.03E-05	0.0061597
cg11636151	7.0349954	0.5974315	82.83989	0.1210084
cg00273449	3.6790333	0.9964789	13.58311	0.0506219
cg25294504	0.8308747	0.4266649	1.618021	0.5858535
cg03818826	225863.08	157.2268	3.24E+08	0.000889
cg09335713	2.5479771	0.9776898	6.640335	0.0556462
cg25446076	1.4434298	0.6799016	3.064399	0.3393104
cg25816610	1.1238866	0.5710262	2.212019	0.7353125
cg09110394	1.7906672	0.5139385	6.239052	0.3603125
cg20835659	0.4609087	0.0620061	3.42606	0.4491741
cg12213111	1.5016376	0.6290562	3.584601	0.359768

cg21633143	0.0914194	0.0133802	0.624615	0.0146889
cg04589674	0.367679	0.1928755	0.700907	0.0023691
cg03221715	0.6687714	0.0006144	727.9975	0.9102172
cg13378563	2.7963561	0.7306035	10.70294	0.1331976
cg20039983	0.2327289	0.0855384	0.633197	0.0043064
cg22134923	0.3471307	0.1404041	0.858236	0.0219643
cg07442409	3.2827057	1.4093276	7.646311	0.0058641
cg27364244	0.1902749	0.0273542	1.323545	0.0935997
cg14166459	0.4498215	0.1744302	1.160002	0.0983537
cg14149304	0.2611718	0.0677136	1.007341	0.0512543
cg08255481	1.758556	0.2766546	11.17827	0.5496964
cg04136484	0.1701039	0.0421122	0.687101	0.0128892
cg07281125	6.260916	1.2378372	31.66739	0.0265581
cg03303006	2.1892839	0.7228389	6.63075	0.1657769
cg04308089	1.2198206	0.7129684	2.086996	0.4683248
cg15641641	0.5204157	0.2047742	1.322591	0.1699252
cg04223844	1321447.6	9.59E-08	1.82E+19	0.3611983
cg23216724	2.2636732	0.7754534	6.608026	0.1349922
cg20080492	1.4378431	0.5149401	4.014822	0.4882221
cg19663284	0.7165852	0.2485315	2.066114	0.5373495
cg04178787	2.1823433	0.6652295	7.159368	0.1979273
cg15516835	0.4108696	0.1717734	0.98277	0.0456058
cg00854751	8.475E+09	34.355836	2.09E+18	0.0204117
cg09050461	0.8192795	0.2736708	2.452651	0.7216181
cg25828963	0.4080759	0.1618946	1.028607	0.0574111
cg20652464	1.1431772	0.4478884	2.917812	0.7795601
cg26015087	3.2083452	0.9421667	10.92533	0.062227
cg02805871	0.9053976	0.3973358	2.063103	0.8130411
cg07534554	2.5494793	1.0310145	6.304319	0.0427559
cg16457916	4.327729	0.72128	25.96667	0.1090305
cg24199203	0.4402739	0.2251092	0.861098	0.0165341
cg06068897	2.130897	0.7027603	6.461267	0.1813149
cg08874974	0.6841566	0.3284583	1.425053	0.310654
cg01611721	1.02E-14	7.08E-30	14.66971	0.0704283
cg22511684	1.2753251	0.7205758	2.257159	0.4037581
cg19393699	0.0116085	0.000293	0.459907	0.0176094
cg12688965	0.3663694	0.1765132	0.760433	0.0070385
cg13191172	4.247E+10	12.247915	1.47E+20	0.0289987
cg01138652	0.3086453	0.1315353	0.724231	0.0069051
cg25970447	5.1788362	0.3320482	80.77243	0.2406466
cg01915076	0.4093979	0.1672855	1.00192	0.050493
cg19192120	0.2117081	0.0419492	1.068442	0.0601342
cg15026243	0.1435197	0.0001277	161.2736	0.5880511
cg02637247	1.2810227	0.6601076	2.485987	0.4640967
cg11885433	0.0380169	0.008357	0.172943	2.33E-05
cg16190225	0.4101275	0.1711878	0.982573	0.0455649
cg02366772	2.7212419	0.876827	8.445403	0.0831874
cg04517749	2.1678141	0.5907472	7.95504	0.2434399
cg12030710	0.0935825	5.27E-05	166.0623	0.5348538
cg15499736	2.6104454	1.1602916	5.873028	0.0203772
cg22169990	0.32874	0.1321296	0.817909	0.0167484
cg19457237	0.4311538	0.1996323	0.93118	0.0322368
cg09005221	1.4815413	0.5086338	4.315413	0.4711396
cg16017414	0.474405	0.2438052	0.923115	0.0281271
cg02365303	0.8331584	0.3874841	1.791436	0.6402721
cg08625192	16.373448	0.0282864	9477.705	0.389018
cg01020801	0.4359622	0.1879478	1.011255	0.0531262
cg03755566	0.8857528	0.3397572	2.309173	0.8040194

cg02445972	0.9215086	0.3635694	2.33567	0.8632292
cg15614301	29655887	4.76E-05	1.85E+19	0.2143448
cg14558191	2.6404506	0.3320077	20.99945	0.3587425
cg06042156	0.3434318	0.08941	1.319152	0.119576
cg02675652	0.2592009	0.1009184	0.665737	0.0050264
cg23903708	0.463394	0.1746711	1.229362	0.1223101
cg02164574	2.4810232	1.0579879	5.818097	0.0366546
cg15490070	2.1597161	0.4751524	9.816585	0.3189016
cg01928807	1.7761263	0.9323016	3.383695	0.080672
cg23031807	5.34E+22	22.26275	1.28E+44	0.0372056
cg27566947	2.0175856	0.4412441	9.225396	0.3654484
cg09127448	1.4113288	0.427835	4.655648	0.5715529
cg22784595	0.3680837	0.1692014	0.800736	0.0117235
cg05242371	0.3353878	0.137459	0.818316	0.0163705
cg12903224	0.0966593	0.0058021	1.610277	0.1035199
cg11744817	2.6559055	1.1595362	6.083324	0.0208871
cg11657203	1.9041845	0.9059353	4.002404	0.0892596
cg24292016	0.0181968	0.0007925	0.417812	0.0122177
cg05394010	1.6571975	0.0113511	241.9416	0.8425292
cg16228077	0.3281879	0.1343294	0.801815	0.0145017
cg14125530	0.6623895	0.3444472	1.273809	0.2169843
cg12809031	0.6244459	0.2406566	1.620287	0.3330724
cg08626831	6.0473015	0.1060236	344.9219	0.3830649
cg18703117	1.3968457	0.6057051	3.221333	0.433069
cg07526904	0.3221278	0.094108	1.102631	0.0711768
cg21192313	1.2659698	0.6691896	2.394956	0.4684248
cg20962746	28.963296	1.2126028	691.795	0.0376148
cg00910127	0.1556548	0.0319866	0.757456	0.0212197
cg18252616	1.4518368	0.736896	2.860418	0.2812315
cg07160574	2.2999436	1.011712	5.228504	0.046839
cg18887186	1.7336768	0.7831978	3.837645	0.1747142
cg01250175	4.124E+11	3.68E-06	4.62E+28	0.1817945
cg16150112	0.040374	0.0033203	0.490941	0.0117979
cg27452868	0.787906	0.312952	1.983677	0.6128528
cg15179805	0.1727929	0.0158864	1.879432	0.1493601
cg01839639	1.1272922	0.3135869	4.052426	0.8543738
cg22177707	2.44E-45	1.66E-71	3.58E-19	0.0008331
cg25773262	8.8944371	1.4605727	54.16438	0.0177423
cg26804848	0.2932328	0.0872128	0.985927	0.0473818
cg27141850	2.2840284	0.9000495	5.796109	0.0821525
cg22091609	2.3140647	0.640281	8.363352	0.2005977
cg20924425	0.1207156	0.0304859	0.478	0.0026018
cg27223805	0.4679903	0.1784105	1.22759	0.1227785
cg00598394	1.315486	0.3969116	4.359922	0.6537803
cg16268449	1.74E-12	9.96E-25	3.027833	0.0597098
cg13676763	0.2588612	0.1079335	0.620837	0.0024618
cg04086097	2.0570217	0.6237157	6.784082	0.2361639
cg12065670	0.644175	0.2476225	1.675782	0.3672837
cg07068998	9.54E-10	6.00E-17	0.015175	0.0140891
cg06833823	0.5624686	0.1862235	1.698877	0.3075966
cg18664667	0.4255333	0.1487749	1.217131	0.1110494
cg15579084	0.4116759	0.1881262	0.900869	0.0263347
cg05269440	2.5809477	1.0982089	6.065596	0.0296417
cg09120267	0.3349178	0.1155623	0.970645	0.043921
cg00080972	0.308972	0.0789927	1.208513	0.091449
cg01750375	0.0296071	0.0010052	0.872017	0.0414192
cg03770593	206.18956	0.7067148	60157.41	0.0657541
cg11169641	1.9238726	0.9675282	3.825507	0.0620645

cg22221320	1.6190158	0.8522098	3.075783	0.1411455
cg14951955	0.5326289	0.2111798	1.343374	0.1820136
cg08096038	0.0268987	0.0001521	4.758097	0.1709206
cg04121561	3.61187	0.4498228	29.00166	0.2269331
cg05304507	0.606156	0.2863694	1.283046	0.1907
cg00788012	24.723471	1.1837286	516.3768	0.0385703
cg21858823	731273.8	11.733599	4.56E+10	0.0165242
cg19035395	0.5657648	0.2371283	1.34986	0.1992173
cg22220710	4.2799574	0.5866838	31.22301	0.1515703
cg11245384	2.6866767	0.8611663	8.381925	0.0886643
cg09851951	2.3486971	0.8079836	6.827339	0.1168016
cg01306801	0.284512	0.0033853	23.9115	0.5782401
cg07373589	12.954778	1.0933055	153.5035	0.042287
cg11505661	1.3232483	0.5331325	3.284336	0.5459283
cg09455126	0.3908487	0.1357428	1.125383	0.0816754
cg16636522	1.8469938	0.9200457	3.707844	0.0844198
cg21209395	1.9850366	0.5189825	7.592492	0.3164808
cg14091154	2.3932067	0.8656909	6.616031	0.0925745
cg02286857	0.4592771	0.1995727	1.056935	0.0672874
cg17317439	0.1514928	0.033735	0.680305	0.0137922
cg10188797	0.3224279	0.1247593	0.833283	0.0194682
cg02129122	3.7477418	0.4849046	28.96563	0.2054259
cg03337256	0.3304763	0.1374845	0.794378	0.0133459
cg00910338	0.6998376	0.3121174	1.569194	0.3863158
cg14453145	1.2301416	0.5598091	2.70315	0.6060984
cg08248330	610.55182	1.1734469	317674	0.044422
cg25028189	2.6042835	0.7196354	9.424623	0.1446775
cg03188064	1.8176441	0.621997	5.31165	0.2747754
cg16664472	3.2733375	0.9401806	11.39647	0.0624549
cg15359321	0.6372582	0.3075322	1.320506	0.2254789
cg01485975	0.6718528	0.3343591	1.350004	0.2639698
cg16151960	0.6902455	0.2652209	1.796385	0.4474757
cg20618076	0.0029003	5.52E-07	15.2526	0.181339
cg12985245	5.0355248	1.3946495	18.18128	0.0135955
cg12226453	99.55479	0.0514331	192699.8	0.2334711
cg09696044	1.1808315	0.4425482	3.15076	0.7399278
cg09916783	1.2014885	0.5480924	2.633816	0.6466763
cg10055227	1.72E-07	5.96E-14	0.497535	0.0401758
cg03683087	2.9452815	1.1204576	7.742089	0.0284794
cg00697658	3.2405928	0.8900691	11.79846	0.0745328
cg26797166	1.3257333	0.4213008	4.171767	0.6297499
cg06617528	4.4604148	0.4089029	48.65531	0.2200303
cg13488556	1.3749776	0.7320159	2.582681	0.3221436
cg00260911	6.2335759	1.3761109	28.23716	0.0175883
cg23980176	0.9406714	0.4740622	1.866554	0.8611318
cg15858747	0.6990288	0.3470323	1.408057	0.316263
cg21923959	2.7910956	1.128495	6.903189	0.0263099
cg23288962	1.4569788	0.638472	3.324793	0.371274
cg08157914	0.3013766	0.1209289	0.751085	0.0100435
cg00821051	2.8380254	1.2048701	6.68486	0.0170176
cg02799090	0.4052908	0.1506382	1.090432	0.073692
cg11672479	0.2395636	0.037478	1.531319	0.1311102
cg25846336	2.8981392	1.2347406	6.802409	0.0145114
cg14529224	1.523E+16	8.57E-09	2.71E+40	0.1908905
cg10318647	0.4804428	0.2086484	1.106289	0.0849614
cg03168614	0.3128384	0.0401197	2.439397	0.2674456
cg21080246	1.02E-30	2.65E-87	3.91E+26	0.2988675
cg25673784	1.9599314	0.66428	5.782698	0.2228555

cg00555339	9.4753409	0.6660351	134.8008	0.0969234
cg06281297	1.810427	0.8654596	3.787174	0.1149686
cg07946277	2.2215872	0.8174552	6.037578	0.1176231
cg12150784	1.0816706	0.5516749	2.120835	0.8192338
cg23676480	1.2663966	0.6846667	2.342396	0.4516438
cg17480278	1.986656	0.925215	4.265822	0.0783052
cg00784882	0.143222	0.0521222	0.393547	0.0001644
cg13596833	1.2648858	0.6711468	2.383884	0.4673997
cg10168149	2.4666119	1.033479	5.887081	0.0419356
cg11755803	0.5234422	0.2534295	1.081136	0.0802625
cg07012062	0.5902884	0.0145903	23.88169	0.7800765
cg19901005	1.8071055	0.2703796	12.07795	0.5415226
cg24566261	0.1031915	0.0208267	0.511289	0.0054104
cg08185241	0.4146184	0.1670933	1.028817	0.0576047
cg20754708	1.7089147	0.2930603	9.965148	0.551412
cg13581527	2.7239293	1.3383668	5.543914	0.0057131
cg01031983	0.1271051	0.0355698	0.454198	0.0015005
cg20873136	2.0143934	0.5591023	7.257671	0.2842207
cg07756501	2.9004111	0.8381075	10.03736	0.0927354
cg22545356	3.7124923	0.6063588	22.7301	0.1559502
cg14033514	0.3413281	0.1333973	0.873367	0.0249338
cg06358131	1.3242016	0.3613821	4.852232	0.6717016
cg12343363	2.243296	0.6951005	7.239783	0.176517
cg07381371	0.8242893	0.2475528	2.744678	0.7528762
cg08491376	3.4733597	0.9021117	13.37332	0.0702655
cg03384920	1.7285008	0.7602378	3.929974	0.1916028
cg16290275	0.1562028	0.0081968	2.976683	0.2169798
cg02713760	1.6065566	0.6736693	3.831292	0.2850034
cg18647570	0.8925308	0.1804916	4.413565	0.8891228
cg22882543	45.969975	1.3281861	1591.071	0.034267
cg18303028	1.2644086	0.6147073	2.600797	0.5237606
cg02041677	3.2859218	0.7102086	15.20297	0.1279765
cg05937445	0.1845768	0.0614558	0.554359	0.0026008
cg03556669	0.1801287	0.0557719	0.581769	0.0041632
cg14659129	0.9405341	0.216535	4.085272	0.9347945
cg13483431	0.0197305	0.0003808	1.022293	0.0512934
cg02073545	0.0050924	6.26E-05	0.414307	0.0186442
cg06829584	2.7120302	0.6738796	10.91457	0.1602077
cg10031532	3.5350227	0.6821883	18.31809	0.1324952
cg06139893	1.7251994	0.3518124	8.459943	0.5014347
cg17593721	0.028534	6.85E-05	11.89242	0.2478654
cg16178743	0.0199143	0.00123	0.322434	0.0058393
cg14881601	0.2764737	0.1051803	0.726731	0.0091257
cg26079864	0.5032398	0.2067097	1.22515	0.130368
cg23204345	0.0898244	0.0078449	1.028489	0.0526988
cg17614582	0.3983123	0.1722225	0.921208	0.0314122
cg09337049	1.4928561	0.6113263	3.645548	0.3790641
cg01231125	0.3066415	0.1254839	0.749331	0.009515
cg02443473	2.3896385	0.7003576	8.153509	0.1641701
cg14383815	0.314653	0.105471	0.938708	0.0381368
cg13892386	0.8583201	0.384431	1.916373	0.7092954
cg06687489	1.600251	0.6987614	3.664775	0.2660929
cg16474725	1.4075487	0.7190494	2.755295	0.3185094
cg24272002	0.4963585	0.1650242	1.492944	0.2125089
cg25098174	2.0565499	0.5807724	7.282366	0.2637143
cg19347588	0.613739	0.1488239	2.531016	0.4994597
cg21495713	0.0828969	0.0091233	0.753228	0.0269904
cg05328547	1.3255857	0.6929324	2.535857	0.3944268

cg19889855	0.3254009	0.1471429	0.719612	0.0055619
cg26880239	0.1432203	0.0271364	0.755888	0.0220386
cg00040575	0.287468	0.0625443	1.32127	0.1091632
cg21818252	0.004219	1.19E-05	1.495052	0.0678967
cg25249713	1.2128941	0.5681594	2.58926	0.6179016
cg16924776	0.3302577	0.1307244	0.834352	0.0191316
cg16655193	1.1588431	0.376642	3.5655	0.797107
cg08634133	0.2401339	0.0578071	0.997529	0.0496033
cg21243021	0.0502038	0.0005661	4.452271	0.1910948
cg09872841	0.0333143	0.0001098	10.10715	0.243357
cg00221327	2137.2232	26.12569	174836.5	0.0006449
cg24030037	3.0958467	0.9598687	9.984977	0.0585696
cg09754845	0.0247385	0.0016067	0.380888	0.008004
cg20667949	3.3227582	0.4235953	26.06432	0.2532007
cg08323832	4.0268042	0.0001085	149389.5	0.7952576
cg19754709	0.3807264	0.1655019	0.875836	0.0230947
cg13397568	2.1933207	0.945075	5.090237	0.0674816
cg15947910	0.4330402	0.2314487	0.810218	0.0088349
cg00239685	9.69E-08	3.22E-14	0.291084	0.0338265
cg04905434	0.0617305	0.0114807	0.33192	0.0011745
cg15117516	0.1540219	0.0362269	0.654837	0.0112994
cg18267049	2.2763992	0.9349716	5.542407	0.0700075
cg01434025	5.3912664	1.4844077	19.58071	0.0104599
cg09866565	0.7836057	0.2970649	2.067016	0.6221962
cg25401594	0.5858583	0.2953428	1.162141	0.126021
cg13236637	6.03E+26	118606.56	3.06E+48	0.0156004
cg06728077	1.777831	0.9162923	3.449427	0.0888565
cg26593380	0.0529914	0.0011692	2.401656	0.1311219
cg26463328	3.9879411	1.1927985	13.33308	0.0246876
cg17067638	0.3180838	0.1120094	0.903293	0.0314797
cg03099780	0.3295897	0.1356752	0.800658	0.0142502
cg08077337	1.3517073	0.6683488	2.733771	0.4016668
cg01227294	2.442145	1.147082	5.199342	0.0205649
cg09888330	1.4673219	0.4486468	4.79895	0.5259361
cg04623023	0.5387895	0.2922874	0.993181	0.0474912
cg27365324	2.9179223	0.5585662	15.24308	0.2042489
cg00635560	0.1483462	0.0440306	0.499803	0.0020766
cg13319175	0.3168275	0.1107219	0.906592	0.0321309
cg11776930	1.0754181	0.1138665	10.15684	0.9493958
cg08882472	0.8621701	0.3807123	1.952491	0.7221425
cg24222435	0.1982129	0.038914	1.009619	0.0513625
cg09190051	0.2738382	0.1257062	0.596529	0.0011122
cg14334488	1.0302936	0.3709882	2.86129	0.9543336
cg14728024	0.0264532	0.0027829	0.251459	0.0015697
cg12368583	0.6792546	0.2736626	1.68597	0.4043772
cg00983697	1.4625742	0.7859923	2.721558	0.2301601
cg27371539	0.4951454	0.211493	1.15923	0.105334
cg09365147	0.232579	0.0899758	0.601195	0.0026116
cg16556008	7.6239959	0.052667	1103.638	0.4235684
cg13647382	1.6809198	0.717308	3.939021	0.2319776
cg01459453	0.628937	0.2994532	1.320947	0.2206549
cg24940248	254.25848	1.85E-06	3.49E+10	0.5623925
cg19880947	1.7730694	0.2392271	13.14138	0.5752122
cg13262831	0.68901	0.342168	1.387432	0.2969261
cg18943289	0.6834943	0.2970631	1.57261	0.3707495
cg18073788	0.8091277	0.3826743	1.710822	0.5793071
cg11114211	0.1501333	0.029606	0.761332	0.0220704
cg11781718	0.1800101	0.0587599	0.551458	0.0026826

cg17015340	3.9899376	1.1098893	14.34341	0.0340339
cg25465406	0.9546644	0.5066613	1.798803	0.8858657
cg01977079	1.4906921	0.678654	3.274368	0.3200163
cg09500328	8.47E-08	6.40E-18	1120.717	0.1708557
cg03062587	0.225565	0.069789	0.729048	0.0128487
cg24265280	1.11E-09	1.69E-17	0.073523	0.0248261
cg02212836	2.9275323	1.2524227	6.843094	0.0131558
cg01267373	1.8609925	0.7878524	4.395866	0.1566992
cg14071650	1.8244518	0.747103	4.455376	0.1868544
cg03124998	1.3092778	0.561655	3.052066	0.532593
cg07202610	6.3704509	0.6321283	64.20001	0.1162155
cg01433520	2.07E-06	3.50E-10	0.01226	0.0031461
cg27240158	0.535368	0.2134218	1.342969	0.1830143
cg03203274	0.1442586	0.0456425	0.455946	0.0009751
cg22820108	0.1452028	0.0309397	0.681451	0.0144386
cg27519599	0.8145753	0.4369124	1.518686	0.5187469
cg03075709	1.23266	0.6779576	2.241218	0.4928685
cg25102842	0.2084374	0.0833028	0.521545	0.000805
cg15124540	2.1288848	0.9356674	4.843763	0.0716348
cg22315453	0.4122358	0.192202	0.884166	0.0228349
cg26399662	1.7210118	0.1622675	18.25308	0.6522681
cg10714639	0.7639805	0.3815804	1.529602	0.44722
cg16886672	0.4742703	0.2024769	1.110904	0.0858371
cg07283415	0.0061815	7.88E-07	48.46116	0.2662577
cg07755203	2.140242	0.6692438	6.844496	0.1995364
cg11219400	0.9300515	0.4892349	1.768059	0.8249015
cg09062595	4.826195	0.0719667	323.652	0.4632126
cg04359880	0.0491989	1.23E-08	196918.5	0.6977901
cg01576340	12276.781	1.68E-19	8.97E+26	0.7259379
cg06317358	0.5293722	0.13373	2.095528	0.364888
cg11137578	2.8868377	0.8203626	10.15872	0.0986349
cg26889990	0.2050685	0.0504047	0.834309	0.0268988
cg20995188	3.6061994	0.7377268	17.62803	0.1131352
cg00155063	1.5422026	0.8124826	2.92731	0.1852108
cg09739017	0.9025216	0.3424967	2.378258	0.8356469
cg26269196	0.1150817	0.0008518	15.54845	0.3877205
cg07543823	0.0794516	0.0155329	0.406399	0.0023563
cg04983977	0.8519577	0.1871353	3.878648	0.83587
cg27427491	0.3270964	0.1551283	0.6897	0.0033248
cg19895786	1.0169277	0.3630201	2.848718	0.9745205
cg17152981	0.1369685	0.031653	0.592688	0.0078183
cg17546649	0.2695442	0.075716	0.95956	0.0430027
cg07454556	0.3225931	0.1482773	0.701835	0.0043348
cg22851781	2.6174681	0.8180802	8.374654	0.104895
cg23252902	0.9896042	0.4998648	1.959163	0.9760752
cg19285508	54.904295	0.0006547	4604270	0.4886228
cg12673726	1.9330217	0.9186207	4.06759	0.082503
cg19216791	0.1321086	0.0402541	0.433563	0.0008431
cg19989581	5.6690287	2.2048948	14.5757	0.000317
cg01044465	367.4348	3.58E-08	3.77E+12	0.6155368
cg16134399	0.4997594	0.2035265	1.22716	0.1301914
cg21640432	2.5064844	0.8205428	7.656474	0.1067864
cg22085751	1.7635931	0.7954632	3.909999	0.1625182
cg24365518	4.1690063	0.4106762	42.32194	0.2272959
cg07812715	0.1971498	0.0626835	0.620069	0.0054789
cg22603569	0.3552809	0.1283612	0.983354	0.0463402
cg24420742	1.0127907	0.1853882	5.532957	0.9882953
cg03753331	0.205554	0.049834	0.847864	0.0286524

cg05692746	2.0639773	0.7751054	5.496029	0.1470183
cg25846061	0.1113942	0.0127482	0.973363	0.047214
cg21561124	2.4058763	0.4724831	12.25068	0.2904455
cg03797139	0.2562894	0.1169495	0.561647	0.0006711
cg05234135	9.50E+24	3.45E-11	2.62E+60	0.1671731
cg18219212	2.369E+19	4.04E-23	1.39E+61	0.3632689
cg09297626	10.000715	0.2893634	345.6357	0.2026946
cg23555592	3.583E+13	76.184329	1.69E+25	0.0228487
cg05856677	2.5994151	0.9314079	7.254564	0.0681116
cg08056069	1.3448365	0.4863839	3.718431	0.5680266
cg23920047	1.5891482	0.6088512	4.147798	0.3440004
cg15980707	2.7297354	0.9804834	7.599777	0.054576
cg01930746	1.1357292	0.5604707	2.301424	0.7239325
cg27202708	0.1732061	0.04421	0.678588	0.0118529
cg25769732	0.3986084	0.1398527	1.136115	0.0852215
cg17937726	2.8287016	1.0258537	7.799896	0.0445067
cg03758633	0.9154058	0.4306728	1.945718	0.8182849
cg26158023	0.000708	2.14E-06	0.233786	0.0142418
cg25642315	0.1940767	0.0671612	0.560827	0.0024604
cg24027965	1.5216279	0.6009671	3.852709	0.375812
cg10525720	3.4075848	0.8853467	13.11535	0.0746066
cg13777759	0.5247759	0.2352019	1.170865	0.1153216
cg08793877	2.2030567	0.9031234	5.374081	0.0825627
cg13615030	0.0392401	0.0003288	4.682372	0.1844433
cg02773640	1.11E-06	1.25E-10	0.009873	0.0031219
cg19431274	2.1487986	0.9917038	4.655962	0.0525199
cg03898385	1.8234729	0.8893442	3.73877	0.1010365
cg07501233	1.7132082	0.8229704	3.56645	0.1501109
cg04060571	0.0539983	0.0122283	0.238448	0.0001172
cg17501982	6.1392358	0.6705776	56.2056	0.1082188
cg19838963	0.39331	0.156546	0.988162	0.047111
cg04422256	2.2479875	0.9132641	5.533392	0.0779772
cg12937434	2.1832883	0.9507457	5.013694	0.0656384
cg06797009	1.527E+11	0.6035862	3.86E+22	0.0545708
cg04950627	0.6957186	9.98E-05	4851.392	0.9359579
cg26406014	3.3333443	1.0216434	10.87579	0.0459935
cg24949488	1.5938959	0.7074649	3.590997	0.2606313
cg23250574	1.4475957	0.82782	2.531388	0.1945373
cg13841742	1.7854119	0.4554084	6.999641	0.4056548
cg09527731	1.8378235	9.31E-07	3627173	0.9344175
cg19908207	2.4197249	0.8471049	6.911858	0.0989205
cg11671925	9.097144	0.8836499	93.65477	0.0634553
cg27178677	0.3325704	0.1226547	0.901743	0.0305268
cg00689360	0.5092623	0.2281733	1.136628	0.0994912
cg05522145	0.4303842	0.1535264	1.206506	0.1089304
cg20038187	0.2917571	0.116612	0.729961	0.0084714
cg11118171	0.0007768	5.66E-06	0.106702	0.0043592
cg14473102	0.3176386	0.0636647	1.584776	0.1619666
cg26799474	1.1835827	0.6396804	2.18995	0.5913681
cg01344452	1.896392	0.7819429	4.599188	0.1568373
cg17400476	2.043435	0.9024444	4.627018	0.0865659
cg15623519	0.1117899	0.0216228	0.577953	0.0089477
cg21510348	2.2500286	0.9344981	5.417484	0.0704738
cg22796593	0.1761597	0.0259999	1.193551	0.0752864
cg07840472	5.63E-77	7.02E-113	4.51E-41	3.15E-05
cg01959980	0.0420903	0.0022737	0.779182	0.033376
cg05259946	0.0618653	0.000495	7.731964	0.2586193
cg02204428	0.866372	0.4564187	1.644543	0.6609076

cg17720153	0.2022846	0.0575843	0.710594	0.0126695
cg13461390	2.4814928	1.1948317	5.153702	0.0147962
cg10001186	0.3045785	0.0713095	1.300921	0.1085303
cg11987853	0.319178	0.149616	0.680907	0.0031353
cg01225525	0.4399445	0.2105504	0.919263	0.0289724
cg10773526	0.547274	0.1975886	1.51582	0.2461636
cg24161652	1.9916209	0.9819149	4.03961	0.0562126
cg07047601	0.0258985	8.13E-05	8.247763	0.2140712
cg01693830	0.7171225	0.0550027	9.349801	0.7996558
cg24123634	0.558122	0.278995	1.116508	0.0992606
cg10513302	1.6223381	0.5636233	4.669752	0.3697076
cg21330553	0.5439659	0.2328361	1.270847	0.1596204
cg07383092	1.2636907	0.6028043	2.649142	0.5354537
cg01207912	3.564778	0.2147856	59.1643	0.3751673
cg02844051	1.8701197	0.1653421	21.15219	0.6129964
cg06342317	1.5365536	0.7832385	3.014404	0.2115372
cg17819119	0.4401048	0.154113	1.256819	0.125274
cg18307783	3.0614499	1.0372476	9.03591	0.0427454
cg18812058	0.7168833	0.3873737	1.326682	0.2892155
cg02471993	0.8051053	0.0162425	39.90737	0.9133202
cg25934581	9.4527685	0.0081369	10981.4	0.5327478
cg00452199	0.0401398	0.0009386	1.716607	0.0933514
cg24735129	1.5480346	0.7648469	3.133191	0.2244623
cg24617612	4.4898388	1.135084	17.75961	0.0323095
cg08982904	1.4713843	0.3841293	5.63605	0.5730051
cg02307823	0.2091975	0.0774126	0.565329	0.0020395
cg10385395	7.8412108	2.0818243	29.53399	0.0023372
cg27451920	1.4412069	0.5650332	3.676027	0.4442586
cg07431961	9.01E-09	2.04E-16	0.398666	0.0391747
cg01216497	0.2575045	0.1129494	0.587064	0.0012522
cg11228717	0.0056316	3.66E-05	0.867301	0.0438667
cg13287553	1.2329881	0.3037024	5.005754	0.7695441
cg03848675	1.6470758	0.7867746	3.448076	0.1855777
cg07090424	1.7061524	0.7872256	3.697741	0.1758193
cg07065756	1.8843784	0.376921	9.420759	0.440322
cg02897366	0.8046666	0.3270799	1.979603	0.636096
cg15166332	0.180313	0.0368717	0.881782	0.034403
cg04097131	0.0131581	0.0003338	0.518699	0.0208814
cg06572115	3.0038441	0.9315109	9.686499	0.0655923
cg26282283	0.295236	0.106266	0.820246	0.0192819
cg16171407	2.1982474	0.9190515	5.257912	0.0766859
cg04470754	0.5713515	0.2726021	1.197506	0.1381879
cg26990380	0.3460565	0.0420441	2.848322	0.3237958
cg24669528	1.7826928	0.4200487	7.565773	0.4331113
cg09155024	0.3576196	0.1449886	0.882082	0.0255916
cg24174911	2.0876284	0.3182501	13.69424	0.4431119
cg07673838	3.2272974	1.2310001	8.460965	0.0171914
cg22096787	0.7702865	0.3157152	1.879356	0.5662917
cg16473141	1.7453138	0.536568	5.677045	0.354729
cg06882877	0.2431075	0.097698	0.604938	0.002361
cg17472664	0.2298615	0.0561395	0.941161	0.0409259
cg08871711	1.8858065	0.4219531	8.428107	0.4063031
cg02276831	6.1669324	0.8720092	43.61313	0.0683424
cg14827528	0.0966384	0.0097494	0.957906	0.0458566
cg13323825	1.5347604	0.7130265	3.303509	0.2734257
cg02756915	1.2397113	0.4823078	3.186521	0.6555152
cg06747745	360730137	4.32E-23	3.01E+39	0.5875417
cg07930539	2.7415713	0.8711951	8.627474	0.0846676

cg22078179	0.4119059	0.176797	0.959668	0.039844
cg20909159	1.362E+18	1709189.7	1.08E+30	0.0028227
cg12531953	0.167188	0.0532114	0.525297	0.0021977
cg08874645	0.4078483	0.1367147	1.216696	0.1077808
cg21155316	0.3538117	0.1116664	1.121042	0.0774329
cg27248980	1.7710525	0.7509212	4.177038	0.1916797
cg01423883	1.9866106	0.9019887	4.375467	0.0883981
cg18771126	1.23E-08	1.86E-13	0.000809	0.0012929
cg20558328	0.2419466	0.0089933	6.509102	0.398226
cg26501007	3.6595644	0.965027	13.87776	0.0564405
cg22876812	2.6219515	1.0233727	6.717621	0.0446332
cg18888671	6.3556713	0.9591991	42.1128	0.0552648
cg16083429	0.4013114	0.1855119	0.868143	0.0203881
cg07580591	1.6457538	0.9002306	3.00868	0.1055529
cg07132492	3.36721	1.185291	9.565671	0.0226633
cg25043279	1.8103959	0.6761117	4.847621	0.237558
cg04736751	0.4478728	0.2224468	0.901744	0.0244729
cg06804846	0.3045956	0.0558548	1.661064	0.1695646
cg26055841	3.4831838	0.064086	189.317	0.540422
cg16548154	0.1346499	0.035352	0.512858	0.0032968
cg22735222	1.4832708	0.8494839	2.589916	0.1656417
cg02051616	1.6472652	0.6760479	4.013743	0.2720266
cg06911354	2.276535	1.0125513	5.118369	0.0465757
cg03666441	0.0448811	0.0025301	0.796145	0.0344011
cg00995893	0.315995	0.1401237	0.712605	0.005493
cg09481404	1.5036871	0.6984588	3.237235	0.2971071
cg18714398	0.2911365	0.065006	1.303886	0.1067253
cg14062083	1.6628283	0.5991291	4.615029	0.3288791
cg08359717	0.7545611	0.160345	3.550858	0.7215566
cg20377305	3.2171292	1.1880669	8.711564	0.0215037
cg04074010	0.6017758	0.3215526	1.126205	0.112225
cg07627445	0.1948422	0.0553135	0.686333	0.0109016
cg03380819	2.4229526	0.5126977	11.45061	0.2640551
cg02152399	0.1772968	0.0419274	0.749729	0.0186977
cg22755414	0.4703129	0.0692312	3.195005	0.4402991
cg11901715	0.2031974	0.0649624	0.635586	0.0061646
cg25835179	0.7152896	0.3261946	1.568509	0.4029407
cg14651896	0.2973011	0.1102995	0.801345	0.0164973
cg09985615	0.6912509	0.3474726	1.375152	0.2927075
cg11713064	0.2861526	0.0890632	0.919385	0.0356318
cg10045864	0.0358116	0.0005054	2.537399	0.1256155
cg19837174	2.7092527	1.0423539	7.041802	0.040847
cg08745288	3.4832474	1.2002907	10.10839	0.0216863
cg19201144	0.3760849	0.1960079	0.721603	0.0032684
cg24734735	2.5962349	0.8905897	7.568508	0.0805153
cg13995516	0.4754956	0.1351303	1.673171	0.2468213
cg12645284	30.183341	1.3101195	695.3824	0.0332773
cg04735719	0.0967894	1.99E-07	47051.24	0.7266844
cg06905823	1.2917268	0.6490658	2.570707	0.4659903
cg15607672	1.915	1.0013756	3.662187	0.0495162
cg15280456	4.557563	1.0997187	18.8879	0.0365278
cg26890309	0.0404922	0.0055482	0.295522	0.0015669
cg25623174	0.919148	0.0026433	319.617	0.9774712
cg14363242	4.0975377	0.8994017	18.66776	0.0683146
cg14204784	1.2370516	0.7147274	2.141091	0.4472326
cg14823389	1.0514164	0.5258096	2.102427	0.8872287
cg21364077	1.2009635	0.4986061	2.892691	0.6830574
cg01574788	4.8580706	0.5889537	40.0725	0.1420465

cg06610329	0.0015864	1.44E-06	1.742153	0.071143
cg01715143	1.342311	0.7128054	2.527757	0.3619697
cg26805224	8.5172725	0.068036	1066.259	0.3846974
cg24836748	6.8969699	0.6966684	68.27953	0.0987485
cg20277670	2.2243865	0.4130756	11.97818	0.3520019
cg03371609	1.7584494	0.6760372	4.573926	0.2471676
cg10298567	0.892021	0.420503	1.892261	0.7658562
cg07540593	9.90E-08	4.85E-14	0.201954	0.0295726
cg19457506	0.3216797	0.0745662	1.38773	0.1283478
cg09531892	0.1349093	0.0027839	6.53774	0.3116867
cg11269599	0.1505045	0.045745	0.49517	0.0018289
cg04923366	419.9132	2.3255172	75822.74	0.0227088
cg01646461	3.2923241	1.3042646	8.310736	0.011661
cg00134787	0.7737777	0.3754225	1.594822	0.4870324
cg03116409	0.7036522	0.3630171	1.363921	0.2979449
cg03007524	3.341703	0.1972852	56.60323	0.4033289
cg20563910	0.1476861	0.0085614	2.547629	0.1880546
cg03214640	5.0279777	1.016513	24.86988	0.0476986
cg10974463	2.302922	0.9000606	5.892325	0.0818069
cg04303330	0.3310013	0.1275799	0.858771	0.0230282
cg13473196	0.1907221	0.0297492	1.222718	0.0804892
cg19348484	0.3579765	0.1107804	1.156767	0.0860495
cg02025270	1.6285166	0.6689432	3.964561	0.2826971
cg12334759	0.976547	0.3873598	2.461908	0.9598801
cg21194765	5.3226965	1.1293909	25.08529	0.0345327
cg00393279	193287.7	9.56E-15	3.91E+24	0.5914941
cg10056739	5.2284818	0.8951089	30.54044	0.0662233
cg13587180	0.6588836	0.3316171	1.309123	0.2336459
cg09195389	2.0877547	0.9551547	4.563365	0.0650428
cg24000797	0.5109649	0.2516177	1.037626	0.0632015
cg23309159	0.4130729	0.213367	0.799698	0.0087126
cg17741572	0.5636701	0.1772597	1.792421	0.3314124
cg05898545	0.4087884	0.187989	0.888924	0.0240056
cg09351254	7405.2794	9.511498	5765460	0.0087133
cg16747828	0.2236622	0.1112527	0.44965	2.63E-05
cg04895854	0.5319164	0.2180916	1.297322	0.1652179
cg03617487	0.9730167	0.3814717	2.481866	0.9543409
cg01670789	0.7473966	0.2450856	2.279211	0.6087844
cg21279955	0.3159642	0.119774	0.833514	0.0199167
cg24857117	1.6764391	0.0031863	882.0352	0.8716028
cg02349416	2.4088953	1.2410315	4.675769	0.0093736
cg24303123	0.411439	0.193379	0.87539	0.0211416
cg12420104	1.3792269	0.6340376	3.000243	0.4174479
cg14567963	1.747461	0.947009	3.224489	0.0741366
cg17202717	1.718023	0.8487413	3.477624	0.1325454
cg05971751	640905.96	42.069412	9.76E+09	0.0065102
cg26509691	1.6164653	0.6993843	3.736086	0.2612292
cg00080706	76.475644	1.3500343	4332.13	0.0352319
cg01243072	19.67885	1.2370938	313.0378	0.0347994
cg17944572	0.0106927	0.0006726	0.169996	0.0013023
cg22131726	0.5368525	0.2346099	1.228467	0.1408123
cg26744079	2.8127964	0.8924266	8.865517	0.0774538
cg03251852	4.0036821	0.3300435	48.56775	0.275973
cg17229371	2.8626445	0.3904155	20.98978	0.300818
cg07997634	0.4167542	0.1833929	0.94706	0.0366324
cg19321696	0.6798031	0.2964109	1.559093	0.3621236
cg03494429	0.3593949	0.1658127	0.778979	0.0095197
cg06122871	2.0361885	0.9442259	4.390966	0.0697405

cg25693317	0.3807363	0.2020889	0.717309	0.0028075
cg01126560	0.091372	0.021214	0.393553	0.0013199
cg04755857	32.56342	1.2796179	828.6664	0.0349212
cg21581845	4.5948704	1.3419285	15.7332	0.0151699
cg00320288	0.803304	0.381552	1.691244	0.5642056
cg15823982	0.0001114	3.44E-07	0.036085	0.002027
cg08889843	0.1582941	0.0407905	0.614285	0.0077149
cg07896558	3.999183	0.8274786	19.32795	0.0846403
cg05380821	3.4523777	1.1306267	10.54186	0.0295908
cg05187003	1.1574492	0.2301801	5.820177	0.8591638
cg02588532	3.4101199	1.2503149	9.300791	0.0165593
cg02057160	0.022842	0.0003622	1.440385	0.0738766
cg02192472	0.0475971	3.49E-07	6483.505	0.6136807
cg13519863	2.3938807	0.6520448	8.788759	0.188342
cg26138978	8.7974225	1.5516937	49.87752	0.0140397
cg12081946	1.3961245	0.6309516	3.089245	0.4102273
cg04867749	3.1008515	1.5639781	6.147963	0.0011926
cg14720455	2.0296853	0.4330399	9.513264	0.3691218
cg07361154	3.0665006	0.8974746	10.47765	0.0738702
cg06689049	0.9708051	0.384273	2.452586	0.9500361
cg22897522	0.0900903	0.0300249	0.270317	1.76E-05
cg20138861	2.5438309	0.6041976	10.7102	0.2030199
cg26536164	1.423425	0.3670691	5.519775	0.6096337
cg04048802	0.4174145	0.1708808	1.019628	0.055199
cg13404674	2.369296	1.2048971	4.658957	0.0124113
cg19106489	0.3738745	0.1382721	1.010921	0.0525547
cg13361558	0.0537641	0.0131261	0.220216	4.84E-05
cg00094898	3.5203177	1.074362	11.53488	0.037671
cg02025435	0.6548727	0.3487592	1.229669	0.1878957
cg16268473	0.7312614	0.3171202	1.686248	0.4628115
cg14001429	0.3633599	0.1413637	0.933977	0.0355735
cg10284406	0.3357857	0.1215868	0.927338	0.0352468
cg05508408	0.4210324	3.34E-07	530600.5	0.9039282
cg16122370	0.3795465	0.1381398	1.042824	0.0602926
cg18912530	0.4644369	0.199098	1.083395	0.0759606
cg15866977	1.4320641	0.8088229	2.535546	0.2179333
cg13189020	0.0886408	0.0246241	0.319085	0.000209
cg11357070	0.0195306	6.08E-08	6274.826	0.5429526
cg20336460	2.9615168	0.5365758	16.34547	0.212881
cg01686177	0.5043381	0.1810147	1.405173	0.1904283
cg22655561	0.3932931	0.1375587	1.124462	0.0816653
cg07253191	0.4059833	0.1557482	1.058262	0.0651659
cg15912010	0.5406167	0.2843842	1.027717	0.06058
cg06195379	1.8747043	0.9474514	3.709442	0.0710852
cg26945643	0.2580718	0.0798716	0.833852	0.0235979
cg10552275	0.3412982	0.1240328	0.939143	0.0373844
cg23791600	2.4244214	1.1747074	5.003646	0.0165969
cg09883255	0.0900389	0.013424	0.60392	0.0131633
cg03369465	11.443178	1.1200235	116.9139	0.0398253
cg20171453	3.7900925	0.8232265	17.44939	0.0872158
cg20255231	1.3757346	0.5997494	3.155728	0.4514206
cg10113069	0.0252478	0.0023979	0.265834	0.0021912
cg25103905	0.0159553	0.0005405	0.470951	0.0165766
cg04003327	0.5831641	0.3108233	1.094128	0.0930031
cg12688670	0.0269651	0.000155	4.690318	0.1698215
cg04508839	64856.703	0.4895586	8.59E+09	0.0655821
cg25921358	30.838593	1.5980963	595.0948	0.0231837
cg07256442	0.0001116	2.04E-19	6.1E+10	0.5991613

cg17582724	0.8798394	0.2672531	2.89657	0.8332207
cg11217218	0.1408115	0.0251632	0.787973	0.0256693
cg15835988	6.2727147	1.3440733	29.27441	0.0194816
cg26576206	0.248162	0.0719711	0.855682	0.0273317
cg09160776	3.3419902	0.9446551	11.82325	0.0612557
cg11359684	0.91721	0.4588662	1.833376	0.8067961
cg23716690	0.4563201	0.213477	0.975412	0.0429501
cg12192112	0.4929345	0.1716804	1.41533	0.1886846
cg03529015	0.2638991	0.0877466	0.79368	0.0177269
cg04566159	1.4157943	0.6116494	3.277161	0.4168187
cg03310469	0.0320257	0.0011353	0.903404	0.0434267
cg06667961	2.3637713	0.6590608	8.477844	0.1867895
cg23419756	2.2147156	0.7873113	6.23002	0.1318624
cg14550153	0.113615	0.0377958	0.341529	0.0001075
cg01461211	2.3542369	0.7864965	7.046988	0.1258618
cg19859729	2.7380963	0.4867862	15.40136	0.2530345
cg06885782	0.2103723	0.0824941	0.536481	0.0010996
cg19917530	1.0806826	0.5210864	2.241231	0.8348465
cg06915334	5.78E-10	5.95E-21	56.15491	0.0993713
cg02855363	0.2598611	0.0592661	1.139399	0.0739504
cg01656216	0.5054544	0.2560091	0.99795	0.0493128
cg06553513	0.6201525	0.2684959	1.432384	0.2632917
cg02246002	0.3480544	0.1315095	0.921165	0.0335592
cg11400759	0.5357054	0.2571692	1.11592	0.0955079
cg22514229	2.0293305	0.1885719	21.83879	0.5593606
cg02562519	1.89E-05	3.21E-11	11.14792	0.1086572
cg13390022	0.120601	0.003641	3.994679	0.236235
cg04444104	1.166472	0.5113793	2.660759	0.714375
cg13876776	1.54E-08	6.41E-22	370948.1	0.2525345
cg09455513	0.3179505	0.1305318	0.774466	0.0116481
cg04439561	0.0006778	1.55E-06	0.295584	0.0186226
cg16916782	0.3435953	0.16162	0.730465	0.0055008
cg02735684	0.3939637	0.1117039	1.389453	0.1474772
cg04265961	2.6102779	1.3091295	5.204642	0.0064303
cg14251216	0.7986713	0.375996	1.696497	0.5586467
cg09067351	0.0390031	0.0032826	0.463426	0.0101985
cg02462933	3.1574967	0.7910245	12.60364	0.1035189
cg23969554	0.3398663	0.1336587	0.864209	0.0234235
cg21811896	1.4217784	0.595652	3.393682	0.4279036
cg19003958	2.0168768	0.9454291	4.302588	0.0695547
cg02817601	2.0227658	0.5091346	8.036345	0.3168831
cg24843346	2.1001407	0.915555	4.817396	0.0798276
cg10282807	0.1310067	0.0361663	0.474552	0.0019681
cg07470708	2.2803924	0.7000572	7.428235	0.1712685
cg08328324	283684.9	75.829453	1.06E+09	0.0027793
cg04781648	2.48E-07	2.25E-12	0.027185	0.0102065
cg06933808	0.0402402	0.0010571	1.531815	0.083577
cg04438074	0.5984739	0.2877128	1.244891	0.1695071
cg09164726	3.3513632	1.2746838	8.811311	0.0142047
cg03385495	7.3164367	0.2920445	183.2948	0.2258987
cg04247717	3.7092863	0.7844689	17.539	0.0981845
cg03194442	0.8776535	0.4229148	1.82135	0.7260781
cg02030628	9.34E-20	2.43E-31	3.58E-08	0.0012831
cg11342331	0.3394395	0.1236363	0.93192	0.0360116
cg18664514	1.2855689	0.5964998	2.770642	0.5214076
cg10621809	2.9063117	0.5784295	14.60273	0.1952119
cg05570980	0.1602918	0.0443102	0.579854	0.0052595
cg08634041	0.4756436	0.2305106	0.98146	0.0443677

cg24160823	0.3375404	0.1145115	0.994953	0.0489372
cg00252733	1.7961035	0.0253908	127.0535	0.7875457
cg20559657	1.0091909	0.4572683	2.227284	0.9819284
cg24767237	1.1003185	0.3841495	3.15164	0.8586794
cg23728085	0.4510305	0.162317	1.253279	0.1267626
cg23113318	0.2745252	0.033569	2.245048	0.2279398
cg22344631	2.4162816	0.8902778	6.557972	0.0833055
cg26690304	0.6239448	0.1575716	2.470668	0.5017189
cg07746628	143.76451	0.3622246	57059.17	0.1036654
cg18581950	0.2015852	0.0468843	0.866741	0.0313858
cg11786365	1.2236033	0.4133181	3.622404	0.7155431
cg19462635	0.3331776	0.1492777	0.74363	0.0072948
cg01021547	3.5565099	1.565036	8.082091	0.0024502
cg00731232	0.2897948	0.125136	0.671118	0.0038431
cg09960202	0.1791142	0.0577965	0.555084	0.0028829
cg22647546	3.8099906	0.8488437	17.10094	0.080803
cg27213509	2.2319635	0.6873614	7.247513	0.1815189
cg03041617	0.3265921	0.1443622	0.738853	0.0072188
cg12116192	0.3862297	0.1332795	1.119252	0.0796997
cg09921707	2.845E+14	4.81E-05	1.68E+33	0.1312561
cg27546012	1.4897908	0.4485868	4.947709	0.5150876
cg02886033	2.9512979	1.0514526	8.28393	0.0398554
cg21202522	0.0607229	0.0126146	0.292302	0.0004758
cg14065382	3402.3659	0.111784	1.04E+08	0.1225996
cg13502215	1.5822829	0.754293	3.31916	0.2247567
cg27263151	0.2214031	0.0745123	0.657869	0.0066555
cg05494467	0.1640474	0.0513425	0.524158	0.0022895
cg12446446	1.4742786	0.4900909	4.434886	0.4896936
cg19499709	11.650227	1.3343704	101.7167	0.0263591
cg02501827	0.438572	0.1646189	1.168428	0.0992263
cg01829672	0.3340956	0.1262382	0.884201	0.0272577
cg13002712	2.4132135	0.5913947	9.847229	0.2195012
cg02755938	0.0464375	0.0048792	0.441966	0.0075796
cg12432526	0.4897436	0.1998096	1.200387	0.118602
cg16303918	2.0626289	1.0513817	4.046521	0.0352309
cg26447747	1.8260367	0.6323922	5.272693	0.2657204
cg03645571	1.2813239	0.5007558	3.278626	0.6050628
cg06234741	0.4109093	0.1835874	0.919706	0.0304964
cg24384195	1.2445157	0.571848	2.708446	0.5814028
cg01556706	0.3208935	0.1458197	0.706164	0.0047356
cg26833936	2.5439097	0.6918492	9.353884	0.1598873
cg26266308	0.1998423	0.0728603	0.548131	0.0017607
cg02312175	1.1121736	0.5256298	2.353235	0.7809895
cg18103730	1.3772241	0.7314018	2.593302	0.3215629
cg16126816	0.2927878	0.0642651	1.333924	0.1123843
cg06814048	480.85428	0.0718157	3219643	0.169441
cg26518580	1.6746418	0.6199841	4.523383	0.309152
cg00866953	1.2243862	0.5323725	2.815926	0.633786
cg01005582	0.2861724	0.0650977	1.258026	0.0976967
cg01350699	0.7331047	0.0115469	46.54418	0.8834504
cg22903773	0.0121041	0.0006286	0.233059	0.0034435
cg22617002	1.7270044	0.5818078	5.12634	0.3249772
cg14115756	0.2734464	0.1385138	0.539823	0.0001865
cg19338277	2.77E-05	1.31E-15	587255.2	0.3870139
cg05036032	0.5511968	0.2773139	1.095574	0.0892192
cg05194300	0.174441	0.0529431	0.574762	0.0041012
cg03246478	0.1825941	0.0674168	0.494544	0.0008227
cg00461905	1.9139182	0.6290667	5.823043	0.2528397

cg02461806	2.901538	1.25E-16	6.72E+16	0.9558137
cg23361356	6.0146384	0.9689779	37.33406	0.0540877
cg02202664	1.6016632	0.6361488	4.032586	0.3173833
cg27354586	3.2749047	1.0398082	10.3144	0.0426979
cg24616795	0.3429305	0.002745	42.84234	0.6639332
cg12411858	0.2293816	0.0525417	1.001413	0.0502199
cg22129135	0.3617319	0.1172619	1.115878	0.0768604
cg00787022	1.754797	0.8606795	3.577769	0.1218191
cg08754294	0.3407759	0.1365166	0.850653	0.0210813
cg27004170	72295.13	1.1458314	4.56E+09	0.0472442
cg15706657	2.655793	0.9364581	7.531823	0.0662801
cg24078566	2.56E-11	8.49E-24	77.1064	0.0961964
cg06878009	0.5723141	0.319461	1.0253	0.0606589
cg24317406	1.6680692	0.6819869	4.079924	0.2621864
cg02015607	2.9302326	0.5479074	15.67101	0.2088679
cg17871403	1.3161179	0.614437	2.819112	0.4797063
cg12414681	10.354642	1.160893	92.35873	0.0362965
cg24840099	2.509965	0.9175883	6.865742	0.0730616
cg08199133	3.1145177	1.0232169	9.480122	0.0454596
cg07730301	0.2011257	0.0592732	0.682459	0.0100865
cg07800524	2.5124888	0.5760992	10.95749	0.22018
cg05178136	0.3601895	0.1011074	1.283155	0.1151811
cg08518568	2.18E-10	4.86E-18	0.009752	0.0133203
cg06312846	0.5847902	0.2679212	1.276419	0.1779348
cg19220586	0.8273142	0.3202956	2.136929	0.6953953
cg23907921	1.54E-08	1.74E-28	1.36E+12	0.442683
cg00959431	1.8513341	0.7843603	4.369724	0.1598309
cg25749107	0.3129058	0.12304	0.795758	0.0146998
cg25616216	2.9445489	0.1351075	64.17387	0.4921672
cg03700279	0.0104743	1.56E-05	7.046594	0.16999
cg13950250	7.36E-05	3.98E-13	13626.69	0.3271791
cg16492851	1.4294837	0.1346541	15.17535	0.7668865
cg08626436	0.2814687	0.1135749	0.697554	0.0061852
cg13492364	1.6536424	0.8319097	3.287055	0.1513031
cg25423146	8.21E-06	4.79E-11	1.407177	0.0568563
cg24461627	2.0303719	0.4715679	8.741922	0.3417059
cg24364282	3.1525707	0.0749288	132.6419	0.547294
cg01772743	3.8477254	0.8658256	17.09928	0.0766193
cg19416904	1.06E+09	8.10E-08	1.39E+25	0.2723995
cg26814565	0.0004518	3.94E-07	0.517537	0.0320932
cg25403442	0.2561799	0.0857339	0.765487	0.0147496
cg15952963	2.19E-10	6.48E-17	0.00074	0.0037345
cg24370881	0.5617425	0.0545947	5.779954	0.6277533
cg03260790	13543048	2.3504162	7.8E+13	0.0386811
cg03892566	2.3412083	0.4825139	11.35979	0.2911368
cg14180030	0.2693143	0.0296872	2.443146	0.2436125
cg15285975	1.6052521	0.5388516	4.782085	0.3954489
cg01865114	3.2640277	0.9374493	11.36475	0.0631
cg16652874	0.448744	0.1854776	1.08569	0.0754728
cg10117400	0.3144413	0.1491711	0.662818	0.0023589
cg03117611	0.1119504	0.0194566	0.644147	0.0141828
cg23958684	0.3383166	0.0602683	1.899142	0.2182219
cg23523755	1.2961159	0.5412936	3.103521	0.5604298
cg02328440	0.1224623	0.0245211	0.611597	0.0104924
cg19963178	2.1843623	0.8614429	5.538891	0.0998053
cg16579158	1.6196996	0.6543202	4.009393	0.2970505
cg04863713	0.0444527	0.0047874	0.41276	0.0061768
cg09681977	0.3518685	0.1520569	0.814244	0.0146868

cg21230450	6.6538344	0.4082387	108.45	0.1832407
cg03673965	3.6657807	1.584915	8.478656	0.002394
cg23909075	3.3615363	1.1987449	9.426464	0.0211928
cg11724151	2.7525756	0.8149651	9.296929	0.1029997
cg01306563	0.1499229	0.0315496	0.712431	0.0170159
cg01275685	0.7731348	0.385338	1.551203	0.4689268
cg08331981	0.309595	0.1110304	0.863269	0.0250273
cg16016716	0.9157006	0.1147716	7.305881	0.9337612
cg01505111	0.4006047	0.1943717	0.825656	0.0131692
cg14825384	375652269	560.70448	2.52E+14	0.0039182
cg10503007	5.2672309	1.0982762	25.26115	0.0377871
cg21764708	4.1804037	0.7383219	23.66959	0.1058757
cg13169484	0.7369811	0.1858807	2.921987	0.6641034
cg20494108	11521266	40.090405	3.31E+12	0.0112266
cg13299707	5.0910832	0.8694817	29.80986	0.0710961
cg24421504	0.9984713	0.020789	47.95544	0.9993821
cg19345940	0.0136777	0.0005032	0.371796	0.0108609
cg12409149	0.3209043	0.1270979	0.810239	0.0161609
cg11753286	1.47E-13	8.77E-25	0.024661	0.0250427
cg19728718	4.0995552	0.7251806	23.1754	0.1104039
cg07342322	0.03196	0.0019546	0.522573	0.0157274
cg09672383	4.0606871	0.9370525	17.59686	0.0610597
cg17437088	0.0838853	0.0221486	0.317706	0.0002647
cg04823720	0.0043249	0.0001017	0.183835	0.0044373
cg10132010	1.0834502	0.5752447	2.040635	0.8040356
cg20811659	0.0172585	0.0013202	0.225608	0.0019663
cg07434707	1.001E+16	14.626683	6.86E+30	0.0345242
cg07725033	0.6464796	0.3039966	1.374804	0.2571662
cg23261443	2.1379451	0.6004114	7.612795	0.2409296
cg10083218	155.98976	0.0029848	8152226	0.3622812
cg13184539	343620.91	41.867024	2.82E+09	0.0055698
cg26620747	0.4595486	0.0770416	2.741181	0.3934977
cg05477405	1.9110999	0.7632872	4.784965	0.1666281
cg16013543	1.2270956	0.3965531	3.79713	0.722523
cg05898333	1.7493854	0.2834468	10.79691	0.5469909
cg12857957	1.12E-21	7.13E-41	0.017712	0.0324514
cg14584255	0.8844103	0.3929743	1.990414	0.7666256
cg00576435	6.6682037	0.1946242	228.4657	0.2926784
cg12865888	0.1057399	0.0037028	3.019546	0.1889241
cg08446111	0.5564219	0.2072326	1.493999	0.2447026
cg11387591	3.0931452	0.6265449	15.27033	0.1657258
cg12904880	1.1702562	0.2325079	5.890119	0.8487755
cg22430565	2.9439616	0.7026332	12.3349	0.1396345
cg19064846	0.2308982	0.0799836	0.666561	0.0067311
cg21013206	0.5208409	0.2093534	1.295777	0.1606882
cg26727231	2.3368819	0.6977219	7.826925	0.168717
cg05597850	51.848778	0.4092853	6568.269	0.1099698
cg05498866	1.4038307	0.3594675	5.482389	0.6255452
cg06866616	1.5688074	0.2668901	9.221611	0.618274
cg17400113	2.0450746	0.5687028	7.354158	0.2732395
cg04212127	3.1224252	0.9399738	10.37214	0.0630413
cg13957745	0.5399774	0.2778922	1.04924	0.0690416
cg27018587	126836.77	8.05E-16	2.00E+25	0.6204449
cg16787199	1.2530932	0.222889	7.044954	0.7978779
cg09577144	1.1758059	0.3317412	4.167464	0.8019242
cg15204286	0.4199741	0.1996796	0.883306	0.0221916
cg26128123	1.7776093	0.6964554	4.53711	0.2288644
cg27265637	12547.227	2.7058397	58182647	0.0284466

cg09253762	0.0636217	0.005305	0.762999	0.0297524
cg07791578	1.6643493	0.7255911	3.817658	0.2290981
cg21180572	0.2509948	0.0741394	0.849729	0.0263051
cg24644902	2.0515608	1.0196719	4.127702	0.0439489
cg23820244	0.7273159	0.3641957	1.452484	0.3669374
cg17409313	0.1802054	0.0558742	0.581198	0.0041275
cg19507267	0.4614744	0.1983118	1.073858	0.0727171
cg00177237	5.237648	1.6920938	16.21243	0.0040747
cg11377827	4.7138667	1.1358518	19.56289	0.0327284
cg27607136	3.2423062	0.5859495	17.94105	0.1777884
cg01002891	31.702456	0.2419041	4154.728	0.1646963
cg01569601	4.4658708	0.4638023	43.00108	0.1952973
cg22818556	2.4603326	0.828852	7.303157	0.1048435
cg19375336	0.5511664	0.222907	1.36283	0.1971378
cg00862588	1.3638479	0.3931789	4.730877	0.6248542
cg23245481	0.3221498	0.1226743	0.845984	0.0214768
cg06609369	6.04E-07	1.16E-14	31.4392	0.1141935
cg19576099	1.6513411	0.6515315	4.185411	0.2904793
cg05275012	0.1400241	0.0026869	7.297036	0.329736
cg16735290	0.9826249	0.2695646	3.581894	0.9788103
cg05902801	3.3958471	0.9141236	12.61512	0.0678706
cg16401173	2.5339071	0.2641426	24.30765	0.4202653
cg10289055	1.6751391	0.5678666	4.941462	0.3499371
cg25094149	113.75144	4.6460489	2785.031	0.0037156
cg09032423	0.4332669	0.1137672	1.650038	0.220224
cg23746359	0.1758159	0.0387205	0.798318	0.0243386
cg04287216	0.4208865	0.1559532	1.135888	0.0875574
cg14180004	6.7982495	0.063706	725.4608	0.4211743
cg22721951	1.6156382	0.5239804	4.98165	0.4037088
cg02819828	3.1294845	0.9494854	10.31472	0.0608225
cg10176070	11.794853	1.4019549	99.23183	0.0231537
cg23279117	2.807658	1.0347569	7.618159	0.0426572
cg16622652	0.001419	2.11E-05	0.09527	0.0022481
cg02901753	6.4585988	0.6608541	63.12059	0.108752
cg05481452	0.3382423	0.0641075	1.784626	0.2014583
cg21165051	4.5632825	0.4129452	50.42691	0.2155564
cg10724947	1.7384176	0.8577932	3.523105	0.1249447
cg12269743	3.1200123	0.4551797	21.38601	0.246634
cg19454081	1.1582868	0.461311	2.908295	0.7544082
cg13759229	0.2054123	0.0571314	0.738547	0.0153442
cg03836195	0.3704133	0.0944567	1.452581	0.1543096
cg16119239	3.8280725	0.4834592	30.31102	0.2035412
cg04313565	0.3785366	0.1568593	0.913494	0.0306752
cg26477856	2.5711407	0.7899436	8.368654	0.1167968
cg16071091	1.591825	0.6144967	4.123549	0.3384367
cg12056618	25733981	5.5041205	1.2E+14	0.0294344
cg16241884	1812.7451	2.00E-09	1.64E+15	0.5932769
cg02924110	0.0364806	0.0041328	0.322017	0.0028847
cg05215481	0.2663475	0.096192	0.737493	0.0108979
cg26489057	0.8899881	0.3924444	2.018321	0.7802642
cg04675502	1.4351767	0.6930547	2.971962	0.3306685
cg04344190	0.4507215	0.2261812	0.898173	0.0234985
cg11427028	0.9100017	0.4676008	1.770962	0.7813113
cg03490073	0.0501715	0.0038976	0.645824	0.0217128
cg18194483	2.7251362	0.7331062	10.13	0.1345193
cg17551192	1.3725783	0.4218568	4.465903	0.5988067
cg08226379	2.1384675	0.9423703	4.852703	0.0690652
cg16576895	0.5784964	0.2745226	1.219055	0.1501108

cg23845168	0.3495288	0.1658865	0.73647	0.0057028
cg00952322	4.51E-05	2.51E-08	0.08092	0.008854
cg03533294	0.0248059	0.0040408	0.15228	6.53E-05
cg23646343	1.972912	0.5622199	6.923237	0.2887385
cg01521258	0.4909505	0.2392874	1.007293	0.0523617
cg25145687	1.3838735	0.6782821	2.823465	0.3718672
cg23348081	0.202739	0.0779515	0.527291	0.0010667
cg08977887	0.2607206	0.0872861	0.778764	0.0160475
cg17342807	3.7087877	0.6533292	21.05387	0.1390126
cg00996875	0.0004549	5.63E-06	0.036752	0.0005941
cg14040899	1.5782843	0.6862053	3.630081	0.2829012
cg10071690	0.7897786	0.2143445	2.910036	0.7228327
cg26092988	3.659E+18	281598923	4.75E+28	0.0003214
cg11235411	0.5005993	0.2504359	1.000654	0.0502166
cg16407323	1.183496	0.5268988	2.658315	0.6832383
cg06457713	0.0070353	3.80E-05	1.300845	0.0627151
cg08473553	0.1336986	0.0249374	0.716808	0.0188453
cg10410146	0.2609304	0.0916253	0.743077	0.0118662
cg20034792	2.174096	1.0117099	4.671985	0.0466138
cg17836145	1.4722876	0.678484	3.194815	0.327768
cg15265222	1.9033235	0.5218846	6.941458	0.3296088
cg17875584	0.3516343	0.173421	0.712986	0.0037559
cg16018972	2.2212712	0.9246868	5.33591	0.0742855
cg07507251	0.4179338	0.1241818	1.406557	0.1588337
cg03900314	1.2295126	0.2096972	7.208973	0.8189
cg15448144	190.95094	0.042026	867611.1	0.2215859
cg13152082	0.0130408	1.79E-16	9.48E+11	0.7898628
cg22331349	0.1840553	0.0632409	0.535672	0.0019013
cg11005998	0.6324978	0.3355923	1.192082	0.1565974
cg19160624	1.515521	0.6204246	3.701987	0.3615586
cg23170981	0.630226	0.3089799	1.285472	0.2042792
cg01983248	0.3801593	0.1761176	0.820594	0.013754
cg11177552	1.5162019	0.4842818	4.746964	0.4747572
cg18599081	2.4599114	0.8763049	6.905318	0.0874078
cg02028216	0.4440167	0.1744887	1.129877	0.0884328
cg12691230	3.221619	0.4441561	23.36753	0.2471945
cg14514581	0.346399	0.1412506	0.849499	0.0205398
cg19230867	0.4152567	0.1966637	0.876817	0.0211838
cg22504024	1.9400803	0.7043749	5.343619	0.1998299
cg07194551	1.4527064	0.5242896	4.025172	0.4726581
cg03894284	2.0412582	0.3351642	12.43192	0.4388726
cg17517296	0.2538268	0.0972998	0.66216	0.0050688
cg07815177	0.0032374	4.61E-05	0.227511	0.008233
cg19351026	1.8426455	0.8933011	3.800894	0.0980202
cg02688118	0.2553194	0.0959299	0.679538	0.0062664
cg25285433	0.6405145	0.2687318	1.526648	0.314769
cg04529897	5.223663	1.6326194	16.71342	0.0053355
cg04752768	19.80421	0.6047682	648.5241	0.0934573
cg08668772	0.0988489	0.0065729	1.486566	0.09427
cg23181580	1.7711253	0.6600463	4.752522	0.2563619
cg09755784	0.560697	0.2325556	1.351854	0.1975564
cg25435332	20.538209	0.5358121	787.25	0.1042559
cg25422226	0.4391903	0.2066217	0.933533	0.0324568
cg10812186	0.173738	0.0533064	0.566253	0.0036914
cg02359511	0.5583916	0.2383831	1.307983	0.1796816
cg01941755	1.9902779	0.9143601	4.332217	0.0828545
cg22643193	1.6747243	0.5098939	5.500559	0.3954038
cg17431888	0.1925965	0.0560462	0.661837	0.0089152

cg22952895	1.8653869	0.8078775	4.307173	0.1442151
cg08357627	0.7497136	0.3330523	1.687634	0.4865318
cg10606888	0.1628227	0.0315097	0.841366	0.0303039
cg07786995	1.1495023	0.4411498	2.995254	0.7755357
cg25311470	0.0635424	0.007723	0.522804	0.0103739
cg15896624	0.5232002	0.2549435	1.073722	0.0773887
cg21180599	0.0417167	0.0034837	0.499545	0.0121463
cg00420808	73.450541	2.1473564	2512.383	0.0171253
cg14250336	0.7042214	0.3748142	1.32313	0.2758081
cg03965207	0.036476	1.10E-05	121.2984	0.4235569
cg07271394	3.7518596	1.014445	13.87601	0.0475402
cg08132132	1.6661026	0.7856723	3.53315	0.1831802
cg25308508	1.5850387	0.6772329	3.709725	0.2883926
cg16932094	0.6536534	0.3114272	1.371951	0.2610205
cg18297736	2.4018734	1.2391651	4.65555	0.0094584
cg13569342	0.3270131	0.1568334	0.681854	0.0028696
cg10283371	0.2402797	0.0787545	0.733093	0.0122276
cg04496920	0.1185933	0.0341863	0.411404	0.000781
cg00706994	1.3488447	0.5625714	3.234047	0.5024127
cg21560819	1.868E+15	2.22E-17	1.57E+47	0.3484878
cg26837630	55.213344	0.7533186	4046.778	0.0671479
cg21982455	1.9292499	0.9621892	3.868267	0.0641161
cg27175851	0.4641514	0.2167338	0.994014	0.048221
cg17181598	0.8015955	0.2172072	2.95826	0.7399248
cg13206530	1.130136	0.5316378	2.402402	0.7505207
cg10825315	1.2229623	0.5648212	2.647983	0.6095912
cg12989217	0.2965526	0.0982964	0.894676	0.0309664
cg20329047	0.2076398	0.0362738	1.188579	0.0774141
cg14378231	0.6158411	0.3291254	1.152327	0.1294086
cg26650973	2.1218216	0.6619333	6.801481	0.2056015
cg15610488	4.6334014	1.2008798	17.87723	0.0260356
cg11854729	0.3226638	0.1045264	0.996035	0.0491979
cg17344770	0.4674845	0.2371973	0.92135	0.028049
cg24080793	2.4700841	0.8894145	6.859923	0.0827241
cg00683158	0.018874	0.0005081	0.70107	0.031356
cg23206160	0.2131541	0.0322526	1.408713	0.1086469
cg12700271	1.7429746	0.6787039	4.476121	0.248269
cg24670445	0.2838944	0.1329654	0.606143	0.0011395
cg22180580	0.4607158	0.2327449	0.911982	0.0261201
cg24430189	1.7328093	0.6485193	4.629975	0.2729357
cg05986933	2.238769	0.700048	7.159633	0.1742271
cg03207574	1.2766471	0.646685	2.520281	0.4815405
cg06822340	0.5270326	0.2129955	1.304081	0.1658688
cg05527785	1.3965313	0.771964	2.526413	0.2694832
cg19025430	0.1010633	0.0226842	0.450259	0.0026409
cg13236679	2590198	1.68E-05	4E+17	0.2612441
cg13910439	2.3159315	0.5392191	9.946863	0.2587399
cg26654770	2.5881332	0.9594509	6.981528	0.060353
cg23375968	0.1759897	0.0465269	0.665687	0.0104832
cg13212831	0.5524203	0.2392831	1.275344	0.1644661
cg02084979	1.7844309	0.1089128	29.23619	0.6848179
cg10353108	0.7569357	0.4098397	1.39799	0.3736594
cg08680239	3.9742605	0.0024559	6431.468	0.7143634
cg07089056	0.8634427	0.3999693	1.863976	0.7084341
cg03199745	5.1232129	0.8305679	31.60164	0.0784114
cg17750075	0.0126094	0.0002443	0.65083	0.029749
cg08191854	1.620214	0.7527311	3.487426	0.2172974
cg15011721	1.12E-15	4.84E-48	2.59E+17	0.365254

cg06529600	0.5414435	0.2064152	1.420249	0.2124252
cg17378342	10.110835	0.5774055	177.0489	0.113203
cg17518962	0.0234156	0.0007906	0.69347	0.029878
cg23839556	37.018361	1.3998598	978.9259	0.0306747
cg04352288	3.7311652	1.2287356	11.33002	0.0201556
cg18539461	1.4769433	0.6422423	3.396478	0.3587077
cg10843796	2.571473	1.0337874	6.396357	0.0422109
cg02598807	0.263143	0.1008279	0.686757	0.0063772
cg25285053	1.8743006	0.9808366	3.581639	0.0572494
cg01972288	0.7312509	0.3183281	1.679801	0.4607405
cg19893751	2.2146701	1.1150088	4.398856	0.023154
cg14014604	51.349651	1.71E-27	1.54E+30	0.9062845
cg02295216	2.0299335	0.6476537	6.362397	0.2244857
cg17809748	2.0871067	0.8458862	5.149646	0.1103224
cg15633893	0.872112	0.3790079	2.006764	0.7475853
cg05155864	0.0087235	2.34E-05	3.246971	0.1164118
cg13616581	1.4075641	0.5638536	3.513743	0.4639115
cg16656826	0.403026	0.1557082	1.043168	0.0610871
cg18946226	2.1250906	0.8323624	5.425534	0.1149615
cg12152867	5.30E-06	4.19E-10	0.067045	0.0117124
cg24914185	0.1955731	0.0498213	0.76772	0.0193449
cg17855595	5.3239801	0.8969947	31.5997	0.0657197
cg03564455	0.5936466	0.3039103	1.159606	0.1268877
cg17818724	0.6917845	0.0004627	1034.251	0.9212982
cg27543361	0.7776896	0.2719208	2.224182	0.6390994
cg08867825	1.6056556	0.7906254	3.260874	0.1901866
cg25869889	0.2940276	0.1069419	0.808404	0.0176853
cg19962750	3.5981395	0.874272	14.80844	0.0760924
cg19748546	0.4564114	0.0936433	2.224519	0.3317525
cg18345635	1.8377096	0.6223252	5.426707	0.2706955
cg09730719	0.0709643	0.0170381	0.295569	0.0002787
cg03689403	0.72017	0.3760564	1.379168	0.3220669
cg00320035	0.4959354	0.0312899	7.86042	0.6188685
cg01257171	1.2898855	0.6268816	2.654097	0.4892836
cg03457195	3.2210638	1.0552451	9.832078	0.0399363
cg21843616	1.5645959	0.8400549	2.914048	0.1583342
cg02417576	1237779.1	0.0446511	3.43E+13	0.1086225
cg07050616	1.9418931	0.8998816	4.190495	0.0908084
cg09307264	0.1590298	0.0351409	0.719688	0.0169866
cg04992127	0.2080188	0.0302414	1.430882	0.1105308
cg23378365	1.6289817	0.6907551	3.841566	0.2649563
cg13416517	0.6047009	0.2736339	1.336322	0.2137397
cg14853974	0.1305122	0.0346149	0.492084	0.002637
cg00002593	0.6982423	0.3460908	1.408712	0.3158422
cg25043986	1.4889443	0.7582522	2.92377	0.2476076
cg26724450	3.87E-06	3.91E-10	0.038387	0.0079453
cg14381040	2.1670971	0.8073159	5.817189	0.1247563
cg25244420	10.121047	1.7155512	59.71002	0.0105889
cg12637911	0.4207429	0.0304781	5.808252	0.5180209
cg08339979	2.3684433	0.9766478	5.74365	0.056432
cg22111705	0.019377	3.60E-06	104.377	0.3683098
cg22863637	0.3983883	0.1829745	0.867407	0.0204341
cg08549810	0.3994709	0.1531145	1.042207	0.0607281
cg02697649	3.8406788	1.0826869	13.62426	0.0372568
cg21250061	0.4284462	0.2098555	0.874727	0.0199385
cg10332864	1.3208528	0.6075628	2.871558	0.482473
cg25567938	2.4522094	0.7771878	7.737294	0.1260165
cg10215102	0.5335329	0.184703	1.541163	0.2457335

cg27665181	0.0280513	4.16E-06	188.9703	0.4268647
cg17574739	2.5141101	1.1445629	5.522414	0.0216603
cg20975414	1.7593811	0.5740002	5.392719	0.3228647
cg09084370	1.3647555	0.5331523	3.493481	0.5166891
cg25085158	0.2820656	0.1206574	0.659396	0.0034879
cg08463932	1.3348953	0.1247578	14.28324	0.8112184
cg23064601	2.4516131	0.9032838	6.653952	0.0783582
cg21676440	0.4968892	0.2501322	0.987073	0.0458124
cg10344369	2.2346633	0.9929934	5.028956	0.0520193
cg06812586	236967.35	7.14E-21	7.87E+30	0.6797797
cg14928057	0.6072505	0.1500873	2.456925	0.4842623
cg03039556	0.8998815	0.2155989	3.755988	0.8849438
cg21279874	1.6206251	0.6542648	4.014316	0.296828
cg12041340	1.282655	0.5841431	2.81644	0.5350549
cg18162670	4.5420375	1.1275422	18.29653	0.0332691
cg15903956	3.5027899	1.202293	10.20511	0.0215824
cg17917325	0.0973258	0.0231127	0.409832	0.0014931
cg21727223	0.4499965	0.1312775	1.542509	0.2039355
cg00455386	1.5834026	0.5398768	4.643955	0.402515
cg11469908	1.0016388	0.487315	2.058792	0.9964458
cg04976330	0.4026702	0.1665899	0.973308	0.0433788
cg17110202	0.0156239	0.0002122	1.150404	0.0579487
cg03875496	2.0464952	0.8812215	4.752656	0.0957473
cg19113686	0.3676494	0.1650594	0.818894	0.0143268
cg27004669	0.3155076	0.0884552	1.125372	0.0754164
cg06994787	1.0534284	0.4284356	2.590147	0.9097187
cg14283978	0.3023593	0.106247	0.860459	0.0249864
cg13536447	2.6765775	0.8812043	8.129859	0.0824117
cg27276395	2.1614111	0.0283074	165.0343	0.7275028
cg00852924	0.1845697	0.0530418	0.642248	0.0079089
cg01730857	0.4152149	0.1646455	1.047119	0.0625459
cg27571329	0.3029492	0.1478538	0.620736	0.001103
cg08199013	1.0305406	0.1804617	5.884984	0.9730036
cg22367556	2.1760165	0.7347771	6.444196	0.1604386
cg24830036	5.4919302	1.1137473	27.08092	0.0364118
cg00913953	5.7116656	0.7814502	41.7469	0.0859839
cg20668221	1.22507	0.3785381	3.964717	0.7347799
cg01051047	0.1951075	0.0374255	1.017137	0.0524047
cg01648131	152.40594	9.62E-07	2.41E+10	0.6018077
cg23986143	8.3477898	0.9691046	71.9072	0.0534336
cg12425292	24.924418	1.4999288	414.1708	0.0249163
cg25399352	1.8367709	0.9475096	3.560626	0.0718113
cg00036110	1.3803768	0.3955594	4.817078	0.6131921
cg23729283	0.4702444	0.2548412	0.867716	0.0157818
cg25534601	2.2102101	0.6733926	7.254355	0.1909172
cg02597698	2.9751568	1.0520859	8.413342	0.0398117
cg08144172	1.27	0.6048932	2.666421	0.5276536
cg02076378	0.7692946	0.3720656	1.590618	0.4791437
cg06908450	1.6900265	0.4989677	5.724197	0.3992032
cg06669056	4.3643391	1.4176002	13.43641	0.0102229
cg02733351	0.7125382	0.3751183	1.353468	0.3005041
cg26078436	0.6421087	0.2972279	1.387163	0.2596446
cg17177995	1.9665585	0.6070746	6.370473	0.2594436
cg24082072	0.9841059	0.1598199	6.059723	0.9862166
cg10542127	0.1674376	0.0339581	0.825586	0.0281342
cg04889688	0.1070689	0.0172496	0.664579	0.016457
cg17742947	5.6429797	1.1398295	27.93683	0.0339779
cg03754153	0.0613244	0.0053912	0.697553	0.0244296

cg20928238	0.2984542	0.0879127	1.013221	0.0525131
cg21098005	0.0693841	0.0107454	0.44802	0.0050522
cg24788333	0.3928132	0.1750443	0.881504	0.0234639
cg00488514	0.3707902	0.1334855	1.029964	0.0569984
cg02819231	3.89E-12	5.95E-27	2543.008	0.1311814
cg27508021	0.0510713	0.0022474	1.160567	0.0619682
cg20639948	1.729139	0.8419535	3.551172	0.1358454
cg22783747	0.0313354	0.0009554	1.027791	0.0518266
cg01521274	0.2212815	0.0760628	0.643751	0.0056342
cg13065504	0.2293656	0.010433	5.042505	0.3503795
cg00266592	0.5723347	0.2688852	1.218241	0.1476744
cg03046247	2.6147378	1.05549	6.477422	0.0378344
cg25908973	0.3641108	0.1712275	0.774272	0.0086759
cg10901368	2.4174455	0.7798856	7.493461	0.1261998
cg22817258	0.7488661	0.188351	2.977422	0.6813233
cg23499126	1.513923	0.5288079	4.334206	0.43967
cg03006588	1.6955881	0.7401997	3.884113	0.2118121
cg24738036	5.4061099	0.9818927	29.76499	0.0525052
cg02863594	1.2028301	0.522446	2.769282	0.6642507
cg13289579	0.1803625	0.0571423	0.569292	0.0034937
cg21400170	0.0433749	0.0047335	0.397457	0.0054978
cg08798701	104.8099	4.141207	2652.636	0.0047739
cg26163234	0.1278254	0.0184889	0.88374	0.0370468
cg00681513	2.56E-14	1.32E-24	0.000495	0.0096053
cg21463518	7.8560294	1.6853517	36.61977	0.0086755
cg01666436	2.1693473	0.6465609	7.278615	0.2098832
cg06044945	1.7243086	0.7480166	3.974832	0.2010342
cg14415885	4.3450468	1.434204	13.1637	0.0093875
cg04981619	0.1829437	0.0516267	0.648277	0.0085021
cg23483563	0.5191031	0.194726	1.383832	0.1899928
cg23994025	0.6091596	0.2017533	1.839253	0.379313
cg19889066	1.2272547	0.6232787	2.416502	0.5535971
cg01701585	0.0285523	0.0004701	1.7343	0.0896631
cg26785617	0.0065995	2.07E-06	20.99576	0.2224127
cg11503274	2.4871013	0.7703365	8.029832	0.1276024
cg24637417	2.4029157	0.3647942	15.82811	0.3620337
cg08263106	0.7134676	0.3253656	1.564505	0.3993663
cg19673440	0.3788483	0.1440024	0.996692	0.0492204
cg09247362	0.7308839	0.3732394	1.43123	0.3605524
cg14562076	5.2031088	1.5325142	17.66531	0.0081814
cg14567414	2.7957617	2.33E-07	33516796	0.9016108
cg15996950	4.8817594	1.5964475	14.92788	0.005432
cg08870914	0.3817092	0.1815949	0.802346	0.0110547
cg07310984	1.41E-05	4.12E-16	481779.6	0.3667312
cg14298020	1.8358626	0.4938226	6.825105	0.3645152
cg17727795	0.5013971	0.2276616	1.104266	0.0865736
cg27190138	1.6566949	0.6396254	4.291008	0.2984996
cg22817352	1.5141874	0.7846919	2.921865	0.2160789
cg16295676	0.2766473	0.0836302	0.915145	0.0352708
cg12465251	0.0053761	9.41E-05	0.307304	0.0113557
cg07148167	1.8203594	0.8657338	3.82763	0.1141653
cg03711944	0.4465952	0.120377	1.656856	0.2281599
cg06444452	0.9260952	0.3763201	2.27905	0.8672889
cg09377704	0.0315152	0.0045196	0.219755	0.0004845
cg06871560	0.5973896	0.2462168	1.449431	0.2546161
cg03379100	1.3587685	0.4296106	4.2975	0.6017768
cg11493223	1.6153868	0.6817894	3.827391	0.2758638
cg19059495	2.4254351	0.7945132	7.404201	0.1197088

cg16209860	0.1425657	0.0416194	0.488354	0.0019295
cg22597058	0.7186337	0.1622765	3.182435	0.6634272
cg22541254	3.4421717	1.4935009	7.933404	0.0037135
cg27057650	2.3506623	1.0731729	5.148856	0.0326393
cg15220969	3.9898243	0.8669847	18.36099	0.0756181
cg00352780	0.35639	0.1354227	0.937907	0.0366348
cg23261640	2.6096905	0.1166059	58.40601	0.5452632
cg17717282	1.1352791	0.4513835	2.855351	0.7874506
cg16459103	1.3369544	0.6204863	2.88072	0.4584277
cg13567299	0.3043688	0.1248583	0.741964	0.0088852
cg02303701	2.0572173	0.7080784	5.976942	0.1849712
cg00145118	0.2317463	0.0725044	0.740733	0.0136564
cg18014109	2.4507578	0.5837648	10.28876	0.2207185
cg03892815	0.567623	0.0330408	9.751471	0.6963093
cg02575193	2.7995802	0.1620145	48.37622	0.4788909
cg15576918	2.7848178	0.872844	8.88499	0.0835921
cg15270687	1.174521	0.4823235	2.860113	0.7231533
cg12712481	0.1982169	0.0761048	0.516261	0.0009209
cg01460481	8.0392754	1.1290114	57.24473	0.037423
cg11824564	0.4792833	0.2612334	0.879338	0.0175374
cg11333835	132813.05	82.852266	2.13E+08	0.0017297
cg19182035	188.82968	0.0170643	2089548	0.2699731
cg25358853	2.277626	0.9145023	5.672572	0.0770615
cg16850690	1.2948744	0.4090977	4.098531	0.6602471
cg07591921	0.5658574	0.280314	1.142271	0.1121036
cg23445604	15.298471	0.4564221	512.778	0.1279452
cg14546523	1.1631959	0.2315736	5.84274	0.8543493
cg22950598	0.2215999	0.0844518	0.581474	0.0022021
cg03232056	1.6341372	0.6943417	3.845951	0.2607508
cg10257521	0.0802254	0.0046734	1.377183	0.0819776
cg27538003	0.1425216	0.0327731	0.619789	0.0093813
cg16668394	73862.815	103.78381	52568078	0.0008218
cg24747764	1.9541725	0.8670266	4.404467	0.1061304
cg08878802	1.5557746	0.0905993	26.71583	0.7606204
cg12598524	0.7870825	0.1234775	5.017098	0.8000039
cg02387803	1.8336365	0.7469224	4.501435	0.1857804
cg12837552	1.0317125	0.3349803	3.177592	0.9566198
cg16253834	1.2854369	0.2157346	7.659172	0.7827469
cg14629665	1.1871896	0.4180979	3.371026	0.7472643
cg27021368	0.2857219	0.1388582	0.587916	0.0006671
cg17860747	5.53E-17	5.79E-27	5.28E-07	0.0014096
cg01993124	3.0168806	1.0962655	8.30234	0.0325239
cg00629585	3.8240451	1.4744713	9.91767	0.005806
cg07177661	0.3233989	0.1319236	0.792784	0.013605
cg26020069	0.4736351	0.1099844	2.039655	0.3157823
cg12056772	0.3118191	0.1296681	0.749846	0.0092406
cg04087742	1.5816053	0.7312004	3.421053	0.2441661
cg27117749	0.2336883	0.0843576	0.647366	0.0051673
cg08432509	0.3043176	0.1362019	0.679941	0.0037268
cg21366688	0.3166097	0.1268072	0.790505	0.013758
cg17490806	61.707191	3.3411699	1139.654	0.0055926
cg09014154	125.7693	0.0609198	259651.4	0.2144493
cg12686407	0.6821327	0.1766081	2.634675	0.5790056
cg14237025	1.6647434	0.6993906	3.962551	0.2493671
cg25890048	0.8815423	0.2675912	2.904119	0.8357945
cg18674487	0.4413136	0.2150108	0.905804	0.0257724
cg18711535	1.677769	0.6180525	4.554481	0.3098261
cg00303876	0.3872164	0.1805216	0.830574	0.0148203

cg26406407	0.3223576	0.1626418	0.638916	0.001181
cg11289006	1.579081	0.5145984	4.84552	0.4245242
cg20724257	1.7316628	0.8424242	3.559556	0.135294
cg25413977	4.0006962	0.5347386	29.93158	0.1769162
cg24988684	2.5469591	1.1181048	5.801782	0.0260319
cg04718702	0.3496927	0.1251219	0.977327	0.0451022
cg17767659	7.56E-05	6.00E-10	9.516476	0.1132076
cg02256455	2.0168176	0.3110985	13.07481	0.4619753
cg20780811	0.0110147	0.0004147	0.29254	0.0070476
cg10137597	5.2531756	1.0049006	27.46128	0.0493267
cg27331495	0.0898892	0.0125611	0.643261	0.0164232
cg06204948	1.29709	0.6163058	2.729883	0.4932594
cg24152630	0.0078431	0.000187	0.32896	0.0109844
cg05261824	1.2264755	0.279272	5.386297	0.7868518
cg02052536	0.4162034	0.1564706	1.107079	0.0790601
cg02782292	0.2652025	0.1134263	0.620071	0.0021926
cg22060041	0.5823856	0.2727105	1.243711	0.1625459
cg03754044	1.236971	0.4862273	3.146877	0.6553135
cg16885557	0.3574881	0.0910732	1.403241	0.1403793
cg06018119	0.4933883	0.2037107	1.194989	0.117519
cg27416412	0.2173175	0.1073722	0.439843	2.20E-05
cg08152198	0.4196864	0.1813915	0.971031	0.042494
cg03436722	3.02052	1.1774062	7.748848	0.0214641
cg07194321	0.3205813	0.0736258	1.395876	0.1296152
cg03925135	3.574E+15	0.0038542	3.31E+33	0.0897678
cg05099145	0.6040704	0.3262123	1.1186	0.1088371
cg04415689	13.902873	0.4919094	392.938	0.1226282
cg06108254	1.1082996	0.400509	3.066917	0.8430423
cg24939819	0.08627	0.0242022	0.307513	0.0001579
cg25570278	0.0119314	0.0001844	0.771988	0.0373787
cg18251187	0.5015389	0.2614857	0.96197	0.0378346
cg27657926	0.0172102	0.0001289	2.297933	0.1037846
cg19038540	1.4954283	0.8117835	2.754806	0.1967047
cg11795128	0.7476205	0.3787164	1.47587	0.4019114
cg02053171	2.058E+16	1.1802931	3.59E+32	0.0489931
cg07775601	6.9668275	0.1930189	251.4608	0.288725
cg12690313	42.399827	0.0060737	295990.5	0.4066673
cg07203258	886636.6	0.0228966	3.43E+13	0.124466
cg05270325	1.0829417	6.85E-05	17124.41	0.9871127
cg02417480	0.1477746	0.0258923	0.843391	0.0314266
cg01331461	0.1958504	0.0504335	0.760554	0.0185038
cg01494348	0.0659627	0.0034886	1.247223	0.0698833
cg10678427	1.1679418	0.634623	2.149446	0.6178983
cg12728623	0.4412802	0.2138448	0.910605	0.0268753
cg17283266	0.3262167	0.1404325	0.757783	0.0091889
cg26021007	0.9972457	0.5477249	1.815691	0.9928021
cg08176353	0.3601958	0.1508641	0.859986	0.0214667
cg24361808	0.4150208	0.1191244	1.445903	0.167295
cg23093404	1.1757837	0.2789536	4.955903	0.8253923
cg21756367	27852.725	0.6765153	1.15E+09	0.0590424
cg00950265	3.3294593	1.3434581	8.251317	0.0093884
cg24169773	0.0061457	0.0005987	0.063087	1.82E-05
cg07672814	3.5533613	0.8947596	14.11147	0.071557
cg18975055	1.6816748	0.1392576	20.30791	0.6825806
cg03062717	0.3352856	0.1495493	0.751701	0.0079815
cg16743754	0.5256408	0.050558	5.464977	0.5903411
cg04167842	16.166005	0.5915638	441.7777	0.0991672
cg19139320	0.5824655	0.2101	1.614784	0.2988606

cg04840203	0.878003	0.1535796	5.019477	0.8837128
cg08986653	17.939648	0.6678926	481.8603	0.0855132
cg17212791	1.5831611	0.8378245	2.991556	0.1570732
cg16427420	0.1794005	0.057366	0.561039	0.003142
cg07362130	0.3851408	0.089967	1.648754	0.1984348
cg15440688	1.5893728	0.7400045	3.413636	0.2348459
cg16861964	1.37762	0.6666454	2.846846	0.38702
cg15733932	0.3464617	0.133062	0.902104	0.0299332
cg22709362	0.4329306	0.167528	1.118792	0.0839451
cg01499197	2.6271649	1.2581569	5.485799	0.0101317
cg00763945	0.5779009	0.2776712	1.202752	0.1425645
cg13909826	22.066856	0.8402783	579.5058	0.0635108
cg10872212	0.0399207	0.0008349	1.908895	0.1026142
cg23899628	2.714682	0.4359548	16.90427	0.2845079
cg09491901	1.434075	0.5784134	3.555538	0.4364438
cg06499892	0.0279209	0.0014	0.556833	0.0191096
cg00662446	3.9614877	0.8863093	17.70644	0.0715481
cg04126266	1.3125372	0.3885122	4.434233	0.6614948
cg16382382	4.67E+25	3.38E-25	6.44E+75	0.3156678
cg17204394	1.0603523	0.6054471	1.857052	0.837605
cg08995871	2.5691451	0.1899765	34.74381	0.4776505
cg18267489	2.2142003	0.9704861	5.051781	0.0589215
cg14832352	1.4257189	0.4768999	4.262266	0.5255784
cg19875535	0.4097592	0.103244	1.62627	0.2046049
cg03846129	0.0016063	2.87E-24	8.98E+17	0.7918114
cg14853282	0.2850434	0.1289852	0.629915	0.00192
cg27429799	8.561405	4.70E-32	1.56E+33	0.9548186
cg26886462	0.1442572	0.0311939	0.667123	0.013211
cg16312002	2.0514309	0.7905644	5.323246	0.1396979
cg00152117	3.3739949	0.5752421	19.78965	0.1778745
cg12916580	0.343984	0.1457072	0.812074	0.0148947
cg17716358	0.4054122	0.1600364	1.02701	0.0569408
cg06395652	0.7763164	0.3610489	1.669212	0.5168338
cg26233209	0.1808785	0.030469	1.073781	0.0598863
cg21887591	0.3551261	0.1532243	0.823071	0.0157795
cg00563090	0.6525314	0.0049928	85.28208	0.8636684
cg05459280	0.6691465	0.2756286	1.624494	0.3746562
cg13208159	6.1549673	1.7330134	21.85997	0.0049496
cg16433632	27.425318	0.3397051	2214.121	0.1393929
cg17099072	0.4367623	0.1391928	1.370482	0.1556695
cg06498267	0.0529367	0.007403	0.378534	0.0034133
cg13971504	2.3840856	0.9382932	6.057663	0.0678362
cg03934572	1.40E-08	1.11E-21	176960.7	0.2400436
cg20546002	0.3019882	0.0487365	1.871224	0.1982165
cg15696462	4.07747	1.2809654	12.97909	0.0173545
cg12747584	1.0506182	0.5374218	2.053878	0.8852058
cg18308339	0.4430034	0.1957491	1.00257	0.0507242
cg21386140	1.5204939	0.7440534	3.107172	0.2504814
cg20526432	0.9923476	0.1813838	5.429117	0.9929314
cg06148974	0.9776884	0.4294108	2.226014	0.9571335
cg05969306	0.292285	0.0892771	0.956915	0.0420782
cg05006304	0.6272417	0.2374897	1.656628	0.3465643
cg27329533	1191.6626	1.70E-39	8.35E+44	0.885436
cg25032793	0.433943	0.1407878	1.33752	0.1460568
cg15870857	0.2877425	0.1019938	0.811772	0.0185704
cg27650175	0.455513	0.176873	1.173113	0.1032775
cg18198301	0.8046782	0.3810377	1.699325	0.5688364
cg14731462	0.9135947	0.4721271	1.767861	0.7884646

cg15081402	2.3610042	0.5240634	10.63677	0.2633024
cg04260507	4.4672089	0.216011	92.38399	0.3328223
cg09879186	2.174435	0.0236417	199.993	0.7363355
cg27152208	1.865555	0.7807727	4.457501	0.1605834
cg02676865	0.3080882	0.0821872	1.154904	0.0807505
cg27384695	3.1502498	1.0113068	9.813119	0.0477757
cg24292612	0.3088892	0.0912793	1.045281	0.0589237
cg12631713	0.4061689	0.1525515	1.081426	0.071343
cg08363345	3.0255494	0.6401292	14.30016	0.1623996
cg07312366	3.508E+09	0.0026551	4.64E+21	0.1227248
cg21052164	0.8445592	0.4191545	1.701712	0.6364728
cg16624069	0.2774324	0.125625	0.612687	0.0015144
cg04551619	0.005177	8.95E-05	0.299596	0.0110192
cg12298688	1.9615625	0.632811	6.080373	0.2431202
cg00875272	1.3347129	0.6616681	2.692375	0.4199987
cg09443479	0.7216064	0.3321358	1.56778	0.4098538
cg08478006	6.4269198	1.1937084	34.6025	0.0303024
cg19576806	0.0003327	1.36E-09	81.30943	0.2058222
cg12037340	0.1520901	0.0003699	62.54264	0.5397184
cg02928664	0.3655529	0.1549646	0.862319	0.021547
cg08550065	6.86E-05	8.35E-15	563044.2	0.4104297
cg25329685	0.1805416	0.0320171	1.018058	0.052418
cg25565203	0.5162771	0.1745193	1.527292	0.2322137
cg04646113	3.57E-05	1.88E-19	6.78E+09	0.5415554
cg13708016	5.2371103	1.0879159	25.21089	0.0389176
cg13460670	2.0772428	0.4301914	10.03027	0.3628361
cg20462883	0.0274026	0.0030986	0.242336	0.0012185
cg14632140	2.3857387	1.1088276	5.133124	0.026134
cg26362197	0.5092996	0.248775	1.042653	0.064935
cg10489672	17.371038	0.8829467	341.7567	0.0603721
cg13578841	0.6959503	0.3743974	1.293671	0.2518171
cg14185025	0.2819104	0.0828674	0.959044	0.0426719
cg02402436	2.5691365	0.8623805	7.65377	0.0902402
cg26517262	0.3762559	0.1215138	1.16504	0.0900624
cg16191009	0.3104657	0.0929168	1.037369	0.0573861
cg09547170	10245502	135.64042	7.74E+11	0.0048516
cg26705724	1.3470693	0.4971166	3.650241	0.5580282
cg01587064	1.3416783	0.4664576	3.859087	0.5855728
cg14388093	1.2334012	0.5845214	2.602605	0.5819089
cg26632162	0.1674259	0.0484513	0.578549	0.004729
cg06340367	2.0919294	0.7421463	5.896639	0.1627276
cg05124117	0.7272652	0.3789671	1.395674	0.3382843
cg15331996	2.5566934	0.7101572	9.204555	0.1509236
cg05115106	0.6276064	8.83E-05	4458.663	0.9180002
cg05130081	0.3865625	0.1509571	0.989888	0.0475745
cg24382918	0.4064903	0.1942506	0.850625	0.0168765
cg02376496	0.1407833	4.30E-05	461.0729	0.6349735
cg19520234	2.8006881	0.8017742	9.78312	0.1065763
cg15454811	0.5960643	0.2721202	1.305646	0.1959004
cg04111064	1.5688254	0.8721092	2.822139	0.1327899
cg21579726	3.3261364	0.3347478	33.04931	0.3049703
cg16775167	1.7212818	0.5583427	5.306438	0.3444474
cg07478501	0.6990168	0.2403136	2.033279	0.5109845
cg13285174	3.0088751	0.4717461	19.19111	0.2439263
cg00895196	0.3629231	0.1165578	1.130025	0.0802867
cg25588787	3.0260674	1.0628972	8.615211	0.0380578
cg13572172	17.488054	1.8397098	166.2393	0.0127549
cg23367351	0.7495551	0.1905134	2.949047	0.6799812

cg00772991	0.5243499	0.2012611	1.3661	0.1863574
cg10146423	0.6209786	0.283688	1.359291	0.2332598
cg17423597	0.5029043	0.2279344	1.109585	0.0886777
cg17839611	2.4987888	0.9909012	6.30128	0.0523074
cg13639582	104.2496	4.31E-15	2.52E+18	0.8092268
cg23740474	1.9623713	0.8658359	4.447611	0.1063367
cg23439277	0.9372239	0.4339832	2.024015	0.8689088
cg21395147	0.018756	2.27E-22	1.55E+18	0.8650639
cg13390740	5.7312347	1.2818488	25.62475	0.0223174
cg01068006	31722.675	36.981418	27211723	0.002633
cg05371749	9.51E-09	7.96E-16	0.113523	0.0263067
cg00514241	0.4057992	0.1634548	1.007452	0.0519002
cg17078393	6.7121125	1.1476155	39.25745	0.0346219
cg14592065	0.1134258	0.0306411	0.419875	0.0011161
cg02683197	2.1545652	0.8574341	5.414004	0.1025145
cg10016835	4.128E+19	64410.348	2.64E+34	0.0094172
cg16377679	4.2858782	1.2559407	14.62549	0.0201336
cg02764346	2.1891309	1.0595533	4.522938	0.0343272
cg20758834	2.5297241	0.4745183	13.48632	0.2770639
cg05315321	0.3197691	0.0262875	3.889775	0.371108
cg26642965	0.382692	0.0320137	4.574709	0.4479819
cg18006769	0.1855319	0.0544015	0.632741	0.0071203
cg20136100	1.2444379	0.6052867	2.558499	0.552053
cg25024515	0.4491509	0.2147554	0.939378	0.0334966
cg02386159	1.2804659	0.7347055	2.231633	0.3830652
cg01288611	0.3428582	0.1500368	0.783486	0.0111286
cg08092966	0.3607283	0.1562059	0.833034	0.0169512
cg11698445	0.3951733	0.1646369	0.948523	0.037685
cg06495038	1.3964759	0.4845376	4.024754	0.536343
cg24467349	2.5305602	1.1149498	5.743519	0.0264081
cg08520191	0.0690926	0.0012809	3.726999	0.1890563
cg16122427	2.5568154	0.9286189	7.039815	0.0692704
cg03009363	2.857401	0.9715055	8.404214	0.0564631
cg25943481	0.3894777	0.1620525	0.936073	0.0350637
cg12192120	0.4825016	0.2245761	1.036654	0.061803
cg16759783	0.3612566	0.1001241	1.303446	0.1199037
cg05839570	11.45308	1.6075261	81.59931	0.0149416
cg25490527	68.061445	2.8009025	1653.881	0.0095232
cg22345519	1.7857987	0.6732915	4.736547	0.243967
cg21580394	14.902576	1.4021415	158.3911	0.0250746
cg05179172	2.9011169	0.9175421	9.172854	0.0697636
cg06798115	0.1545252	0.0426443	0.559936	0.0044716
cg04194745	0.9787391	0.176166	5.437656	0.9804043
cg05278307	0.2553943	0.0689094	0.946551	0.0411368
cg17449465	0.335129	0.1210852	0.927541	0.0353105
cg19128113	2.1282564	0.9454802	4.790661	0.0680708
cg12634080	1.8297812	0.8663958	3.864399	0.113197
cg18536644	0.1985834	0.0447291	0.881648	0.0335373
cg16922763	1.9556535	0.6905885	5.538147	0.2066258
cg00010946	2.2961576	0.4164113	12.66138	0.3399614
cg27178155	0.3216499	0.1127438	0.917644	0.0339515
cg12105361	8188565.7	119.40863	5.62E+11	0.005083
cg22731359	0.1536687	0.012936	1.825452	0.1379862
cg15306012	0.2941665	0.0961871	0.899642	0.0319214
cg06155620	0.2515655	0.097911	0.646355	0.0041519
cg23132774	2.1265014	0.2285979	19.78149	0.5073071
cg05958206	0.9050935	0.2816878	2.908164	0.8670243
cg11708454	4.1431135	1.2937721	13.26771	0.0166796

cg07234865	1.5880845	0.7304855	3.452516	0.2430664
cg15416329	0.8208198	0.3822202	1.762715	0.6126189
cg27500720	0.0116785	0.0009682	0.140866	0.0004606
cg22025263	0.3277329	0.1552366	0.691904	0.0034335
cg12308664	1.5220693	0.5759007	4.022733	0.3969193
cg08183303	0.1486288	0.0417363	0.529287	0.0032634
cg06966113	2.3622047	1.1122075	5.017059	0.0253073
cg01649266	4.2290325	0.1056501	169.2825	0.4436786
cg07181952	0.184202	0.0504017	0.6732	0.0105154
cg16632785	0.2517912	0.0813528	0.779307	0.0167326
cg10415524	211.36685	0.3992844	111890	0.0943167
cg03907847	1.5138325	0.60904	3.762789	0.3720939
cg20258698	1.0653662	0.3596667	3.155714	0.9090114
cg26276650	0.196989	0.0721493	0.537838	0.0015234
cg07230639	0.1868391	0.0403107	0.865996	0.0320461
cg14181528	0.0812509	0.0201825	0.3271	0.0004115
cg10977770	3.1520766	1.4030886	7.081225	0.0054345
cg05864564	2.2413132	1.0004215	5.021368	0.0498804
cg08188268	1.9145587	0.7497233	4.889184	0.1745332
cg01795082	3.7457626	1.0778704	13.01709	0.0377136
cg04381249	0.168746	0.0470889	0.604712	0.006288
cg17127769	1.3858443	0.7494101	2.562768	0.2981998
cg04248279	2.7817516	0.8042467	9.621602	0.1061189
cg08356262	4.468825	1.0616217	18.81122	0.0412006
cg06233985	0.6847338	0.3607853	1.299555	0.2466714
cg08942875	2.1113592	0.8208747	5.430595	0.1210316
cg19389884	1.5382986	0.7733167	3.060017	0.219686
cg01468220	0.287508	0.1224464	0.675078	0.0042072
cg13827597	6.0512949	0.6640159	55.14653	0.1103118
cg04839616	3.1165749	0.596218	16.29109	0.1779456
cg08901242	0.4330931	0.1201921	1.560582	0.2007318
cg25199322	0.7326481	0.3696833	1.451981	0.3727222
cg23640929	4.9142978	1.2545394	19.25035	0.0222845
cg16445596	1.236529	0.5222287	2.927844	0.629268
cg22023046	0.0495234	5.01E-47	4.89E+43	0.9546617
cg21200667	0.9282284	0.3043032	2.831413	0.895864
cg00971332	0.4915539	0.217243	1.112235	0.088261
cg27466999	1.339695	0.2048454	8.761646	0.7602025
cg24434987	2.2871068	0.8063169	6.487347	0.1198858
cg05062323	1.140259	0.5304329	2.451188	0.7367631
cg25214310	0.3266178	0.0983144	1.085081	0.0677501
cg04794420	0.0009522	3.56E-06	0.254538	0.0146933
cg14657725	2.1465342	0.6590115	6.991697	0.2048628
cg22363301	0.7335628	0.3593403	1.497506	0.3947937
cg18448565	65.219623	0.4386678	9696.63	0.1016156
cg07313701	2.1744558	0.595816	7.935769	0.2395931
cg07818978	0.4201133	0.1890052	0.933811	0.0333369
cg25991569	2.2247535	0.9930321	4.984258	0.0520193
cg20952076	0.1755921	0.0364756	0.845292	0.0300386
cg02131862	1.7364735	0.5567868	5.41561	0.3416386
cg21266698	0.6417128	0.3191894	1.290128	0.2131238
cg23880668	1.4345274	0.719948	2.858358	0.3049681
cg12796743	10.453201	1.2968864	84.25518	0.0275164
cg13771219	4.7563979	0.7461061	30.32186	0.0989295
cg23709117	0.1580656	0.0100753	2.479807	0.1890554
cg22841779	0.1410809	0.0197632	1.007113	0.0508319
cg01066588	0.3428783	0.0680853	1.726738	0.194385
cg16006724	1.3592764	0.5567247	3.318754	0.5003266

cg09881503	0.2554516	0.0197824	3.298672	0.2957622
cg02626388	0.1723215	0.0379629	0.782203	0.0227133
cg11107212	0.6838239	0.2546834	1.836064	0.4507374
cg02375178	5.3628997	0.7194631	39.97522	0.1012734
cg01830463	0.3700155	0.1626337	0.841839	0.0177663
cg22039287	0.4855659	0.2502529	0.942144	0.0326641
cg12938014	2.1709405	0.7778749	6.058792	0.1387982
cg17472786	3.5677315	1.1244541	11.3199	0.0308444
cg24403305	0.0447367	0.0056954	0.351403	0.0031323
cg16701896	1.6320209	0.7207023	3.69569	0.2401692
cg19008720	1.7668179	0.8068773	3.868798	0.1546338
cg12739584	1.7200645	0.8006103	3.695458	0.164522
cg12848131	0.9842103	0.0166879	58.0462	0.9938955
cg00578885	0.3761072	0.0182254	7.761493	0.5266289
cg01469847	0.1117537	0.0356008	0.350804	0.0001735
cg07694975	0.1418159	0.0340541	0.590583	0.0072852
cg15582138	0.3301075	0.1220399	0.892913	0.0290312
cg12374682	0.4728815	0.1840181	1.21519	0.1198926
cg11328436	3.2831227	1.2688471	8.49503	0.0142517
cg24494491	1.9677747	1.0679686	3.625703	0.0299423
cg23149825	0.5789454	0.2757166	1.21566	0.1487376
cg25274975	1.5119491	0.2942628	7.768531	0.6205609
cg21787848	0.9315295	0.4282831	2.026106	0.858014
cg04506109	1.3806838	0.3526174	5.406109	0.6432222
cg05941904	0.7837433	0.3427891	1.791928	0.5635864
cg13826247	2.1968465	0.75682	6.376859	0.1477558
cg15629311	3.0932944	0.7692478	12.43874	0.1117291
cg20268039	1.9495483	0.8057202	4.717194	0.1386574
cg25002426	31.824984	1.8218732	555.9276	0.0177402
cg15156059	0.3442653	0.1561859	0.758831	0.0081851
cg25482900	0.2238953	0.0711163	0.704889	0.0105391
cg00166868	0.1142862	0.0107018	1.220477	0.0726418
cg13348062	24.343991	0.7768782	762.8351	0.069322
cg19008371	0.1626605	0.0273233	0.968346	0.0460097
cg05139070	4.52E-13	2.36E-22	0.000866	0.0091446
cg15887497	0.0206935	0.0004322	0.990696	0.049449
cg05003685	3.492E+13	643.79895	1.89E+24	0.0134055
cg04203824	2.0494413	0.1263856	33.23329	0.613689
cg00344577	0.172454	0.054851	0.542203	0.0026359
cg24604013	0.2702136	0.0832553	0.877006	0.0293723
cg00480115	0.4341969	0.2139336	0.881241	0.0208865
cg10918735	2.0777665	0.9625517	4.485072	0.0624983
cg02925831	2.7156689	0.6041829	12.20633	0.192625
cg03193847	0.4617026	0.2090332	1.019787	0.0559401
cg01829129	8014909.4	9.99E-06	6.43E+18	0.2556791
cg01226742	0.54333	0.2269428	1.300801	0.1708237
cg23123250	0.4047279	0.1905196	0.859778	0.0186243
cg27549944	1.3485004	0.5509855	3.300365	0.5126371
cg01666652	2.2478134	0.5544477	9.11297	0.2567399
cg21967668	2.2462735	0.6322644	7.980434	0.2108691
cg18652721	1.0442902	0.5623716	1.939184	0.8908437
cg15401332	0.291751	0.0920756	0.924442	0.0363063
cg22746058	0.1340832	0.0302597	0.594133	0.008158
cg18254819	0.0973575	0.0246854	0.383971	0.0008773
cg05901196	1.754914	0.8401389	3.665731	0.1345273
cg11926456	2.1704729	0.6765503	6.963197	0.1925848
cg24968721	2.8556621	0.6568337	12.41533	0.1616931
cg19433807	1.5582137	0.4921239	4.933778	0.4506989

cg07808036	0.2485143	0.0616726	1.001406	0.0502315
cg09577115	2.235758	1.0865809	4.600314	0.0288509
cg13169525	6.13E+09	0.0002872	1.31E+23	0.1501034
cg16924565	0.2988102	0.0856541	1.04242	0.0581195
cg10892335	1.9680335	0.5415593	7.151859	0.3037687
cg09254210	2.5609109	0.7812991	8.394051	0.1205403
cg00167491	1.8259363	0.6743572	4.944032	0.2361302
cg13821008	1.1188526	0.5079676	2.464392	0.7804381
cg14329275	0.2538716	0.1170649	0.550556	0.0005184
cg15732768	2.981533	1.0216138	8.701467	0.0455981
cg02882813	1.6828501	0.7340618	3.857965	0.2188467
cg08702805	2.484983	0.6336551	9.74527	0.1916974
cg04787211	0.0465905	0.0018781	1.155793	0.0612631
cg22774088	1.6084947	0.6989458	3.701654	0.2637019
cg12757310	0.0071389	7.33E-06	6.950168	0.1592114
cg13939721	2.5848804	0.7341678	9.100926	0.1391988
cg10642330	3.2713886	0.7387641	14.48633	0.1184883
cg08508337	0.1440083	0.0100214	2.06942	0.1541202
cg00876273	1.948466	0.8567834	4.431132	0.1115566
cg07713946	0.5415046	0.0605367	4.843795	0.5832141
cg24375218	0.6922219	0.262086	1.828297	0.457891
cg18494672	1.8432748	5.33E-05	63733.93	0.9086918
cg00269659	0.4368136	0.1844956	1.034204	0.059635
cg02078370	0.3878933	0.1168975	1.287121	0.1217402
cg20110707	1.7337363	0.8353096	3.598476	0.139685
cg24822602	0.3829638	0.1686838	0.869445	0.0217682
cg06180869	2.2640089	0.6780629	7.559382	0.184054
cg22186503	1.9421283	0.9063001	4.161825	0.0878292
cg23087707	0.4807913	0.2385037	0.969211	0.0406191
cg05392844	0.8077694	0.4481219	1.456058	0.47763
cg18583306	1.994241	0.8133318	4.88976	0.1314413
cg16226866	0.5290127	0.1910522	1.464806	0.2204374
cg19164987	0.4850801	0.2172907	1.082894	0.0774621
cg25926189	2.0631136	0.7915901	5.377073	0.138399
cg07621749	0.5391605	0.228102	1.274404	0.159282
cg05274924	0.8964014	0.432016	1.859967	0.769012
cg09269103	1430.4268	0.0123857	1.65E+08	0.2218443
cg08187246	2.1946369	0.6307085	7.636541	0.2166491
cg18655898	0.0874463	0.0082149	0.930855	0.0434516
cg09988853	6.43255	0.8203142	50.44128	0.0764837
cg12912869	0.1541673	0.0273132	0.870187	0.034223
cg14518209	0.2606133	0.0084654	8.023157	0.4418592
cg00401753	3.2283775	0.9905387	10.52197	0.0518721
cg01825806	5.2315004	1.1619859	23.55329	0.03112
cg18324707	0.5005924	0.1907947	1.313415	0.159723
cg02058628	0.2911298	0.0973836	0.870337	0.0272085
cg23384093	1.9396561	1.0791725	3.486251	0.0267827
cg02002258	0.231276	0.0887008	0.603023	0.0027498
cg23610213	2.3065141	0.6173884	8.616953	0.2139382
cg14375632	0.3354049	0.1546298	0.727521	0.005689
cg04654907	2768562.5	0.2512691	3.05E+13	0.0729712
cg22636278	989.05928	1.88E-34	5.20E+39	0.8729851
cg02928928	0.3215029	0.1201821	0.860063	0.0238067
cg17433678	4.0123352	0.8965864	17.9557	0.069188
cg14524955	2.4411054	1.0142719	5.875146	0.0464163
cg14390047	9.0897285	0.437661	188.7835	0.1538477
cg23036852	3.0132586	0.9895026	9.176052	0.052211
cg23449588	2.3579843	0.4717886	11.78513	0.2960708

cg01156550	2.113254	0.7912829	5.6438	0.1354678
cg00332048	0.2001957	0.0650802	0.61583	0.0050231
cg26530341	1.204033	0.3476499	4.169987	0.7695562
cg11038285	2.3183098	1.73E-19	3.11E+19	0.970152
cg13305415	1.4579757	0.7032114	3.022837	0.3108134
cg22049038	0.3191749	0.1334655	0.763288	0.010253
cg10820587	2.0999669	0.1070893	41.1793	0.6251108
cg26034934	2.2893979	0.9286645	5.643957	0.0719863
cg07071978	1.4126254	0.6121288	3.259952	0.4181486
cg10432093	0.234599	0.0813077	0.676894	0.0073233
cg05288172	1.2708623	0.6711703	2.40638	0.4618148
cg02532928	2.6133745	0.656107	10.40947	0.1730974
cg20287514	0.2055046	0.0781129	0.540655	0.0013458
cg15348609	0.1826108	0.0638963	0.521888	0.0015049
cg12396523	2.2979695	1.1372234	4.643471	0.0204358
cg03155027	1.5265579	0.4749437	4.906642	0.4776419
cg12524061	1.695767	0.539753	5.32767	0.3658815
cg25275452	0.0206867	0.0007064	0.605827	0.0243968
cg16202598	0.540344	1.64E-17	1.78E+16	0.9746947
cg19977428	3.0074923	0.8367128	10.81017	0.0916314
cg07176385	0.6861971	0.3433343	1.371452	0.2864622
cg16638540	0.2162666	0.0628343	0.744358	0.0151773
cg22761077	0.1408047	0.0380947	0.520439	0.0032917
cg00459068	1.6324071	0.6508495	4.094269	0.2962341
cg23077498	0.339752	0.062302	1.852773	0.2122531
cg19272832	1550.3328	10.094202	238110.1	0.0042355
cg00725356	3.00E-05	2.10E-08	0.042853	0.0049565
cg12535090	2.9355364	0.1939576	44.42916	0.4372559
cg17497320	0.6705882	0.00765	58.78282	0.8610185
cg27250032	0.2405437	0.085382	0.677676	0.007013
cg02227217	0.1054528	0.0155675	0.714328	0.0211878
cg10585371	0.8807468	0.1421515	5.456958	0.8914576
cg04923875	0.923428	0.4194158	2.033112	0.843176
cg23051411	1.419597	0.5999439	3.359074	0.4252699
cg00080418	1.6646915	0.4968636	5.577382	0.4087216
cg06586505	2.6327625	1.1289307	6.139826	0.0250478
cg05238069	1.2814195	0.4873393	3.36939	0.6151626
cg00870633	0.7218679	0.2257253	2.308529	0.5826793
cg12439199	1.4359814	0.577656	3.569673	0.4360878
cg14670092	1.1425523	0.4316209	3.024473	0.7884596
cg22012156	2.2073843	0.2455356	19.84456	0.4797765
cg00083265	0.037282	1.65E-10	8408768	0.7374917
cg21222350	0.2759314	0.0934348	0.81488	0.0197805
cg10262032	0.1439095	0.0231116	0.896083	0.0377506
cg19614504	4.1942431	1.2718822	13.83121	0.0185225
cg18169971	1.8553338	0.0028584	1204.242	0.8516052
cg11831419	0.3343339	0.1656866	0.674642	0.0022227
cg17322774	0.2429843	0.0552592	1.068443	0.0611584
cg02394698	1.9395686	0.5488693	6.853956	0.3036883
cg14174232	4.7201572	0.6190238	35.99197	0.1343333
cg05100518	3.6667926	1.1274546	11.92542	0.0308246
cg14646039	0.329046	0.1140926	0.948977	0.0396985
cg04498198	0.2230147	0.0859478	0.578671	0.0020397
cg02589353	0.6333912	0.2417675	1.659381	0.3527168
cg13089906	5.4221986	0.018623	1578.709	0.5592456
cg15940128	0.4058245	0.1626511	1.012557	0.05321
cg21134096	0.5702363	0.2274796	1.429444	0.2309297
cg01302716	3.10E-20	8.38E-32	1.15E-08	0.000949

cg22488750	1.2782534	0.5365405	3.04531	0.5793984
cg13443733	1.5584423	0.5226658	4.646836	0.4260425
cg03340408	1.1645923	0.3101387	4.373124	0.821425
cg09096612	2.28E-07	2.63E-19	198057.8	0.2755592
cg15890707	0.0464841	0.0022411	0.964147	0.0473044
cg01003303	4.2828596	0.3653763	50.20273	0.2467567
cg12400956	1.8173708	1.0265657	3.217365	0.0403709
cg21970261	0.3816953	0.1782052	0.817548	0.0132002
cg01324372	1.2457712	0.7380564	2.102747	0.4106392
cg04119977	1.0145272	0.4621017	2.227357	0.9713251
cg23359895	9248056.5	15565.673	5.49E+09	8.56E-07
cg01335785	0.0004116	9.22E-06	0.018373	5.77E-05
cg24580199	48.471484	0.9045362	2597.447	0.0560597
cg21916596	1.2567406	0.4763864	3.31537	0.6442794
cg14335069	1.0180734	0.3334389	3.108436	0.9749091
cg12440062	0.4408147	0.1499018	1.296299	0.1366423
cg08474953	4.8447503	1.6945072	13.85158	0.0032406
cg12486533	1.8338275	0.6363734	5.284513	0.2614466
cg01019876	0.7190688	0.0518987	9.962863	0.8057583
cg22609984	0.3888469	0.1656885	0.912568	0.0299938
cg17524651	0.9407876	0.4393833	2.014372	0.8751405
cg04453138	1.4065099	0.6034198	3.278431	0.42951
cg02964163	7.61E-05	7.03E-08	0.082477	0.0078177
cg01754267	0.2613254	0.1238201	0.551534	0.0004293
cg11406765	0.0042982	1.19E-05	1.557434	0.0698932
cg03689601	0.1413809	0.0087573	2.282505	0.1680626
cg01281582	0.0290255	0.0003975	2.119238	0.1059039
cg08336604	0.5968408	0.302229	1.178639	0.1371332
cg10555106	0.1323911	0.0421985	0.415356	0.0005281
cg00735667	0.2117443	0.074477	0.602007	0.0035925
cg04436207	1.3312256	0.4826558	3.671688	0.5804667
cg18934187	0.4192163	0.0830547	2.115982	0.2925556
cg26018431	2.9259245	0.3445132	24.84966	0.325292
cg04908668	13.088542	0.7026878	243.7924	0.0847982
cg10685336	6.04E-07	3.40E-16	1073.23	0.1875793
cg06172732	8.3856346	0.5776601	121.7305	0.1192509
cg08071130	0.2204862	0.0762012	0.637971	0.0052854
cg26279745	1.8034702	0.6991047	4.652386	0.2226004
cg10477621	2.237939	0.8442999	5.931981	0.1053031
cg06706875	1.1319166	0.500902	2.557856	0.7657808
cg18988435	5.6769384	1.4455944	22.29369	0.0128466
cg22697386	0.4488913	0.2277242	0.884857	0.0207086
cg20696698	1.7523999	0.3943866	7.786536	0.4609823
cg04772575	0.3510337	0.1039755	1.185132	0.0917275
cg16861076	0.112507	0.012412	1.019809	0.0520737
cg13540210	8.5601129	0.9612358	76.23054	0.0542886
cg02635407	0.3299817	0.108559	1.00303	0.0506268
cg05211947	0.6114921	0.2546204	1.468549	0.271196
cg08572565	0.369169	0.1834853	0.742761	0.0052115
cg18741958	0.4470752	0.1845168	1.083241	0.0746054
cg14841540	0.7212942	0.3396602	1.531723	0.3951774
cg02091185	0.2671403	0.1289848	0.553274	0.0003804
cg07493324	0.4499801	0.2032153	0.996392	0.0489673
cg14643415	0.7042208	0.3254901	1.523631	0.3731729
cg26284685	0.5254524	0.2758654	1.000851	0.0503033
cg04493143	0.3423517	0.1635051	0.716826	0.0044699
cg13550608	1.6120967	0.0899456	28.89363	0.7457122
cg12527478	1.4063064	0.5985816	3.303973	0.4339875

cg02659232	5777.3472	1.50E-11	2.23E+18	0.6132165
cg02277965	1.4724219	0.5106099	4.245954	0.4739672
cg15204594	0.1464262	0.0455694	0.470505	0.0012558
cg04369341	1.5710251	0.7371857	3.34803	0.2419488
cg02511315	0.22424	0.0954937	0.526564	0.0005979
cg01475735	265902539	3507.9123	2.02E+13	0.0007147
cg09053680	0.3120372	0.11523	0.844982	0.0219425
cg27162882	2.2582317	0.7122396	7.159965	0.1664853
cg15974867	2.3853807	0.32867	17.31232	0.3899725
cg22841772	1.0126823	0.5154385	1.989617	0.970824
cg17318990	1.0676025	0.3641194	3.130223	0.9051244
cg22491305	0.5151482	0.2561015	1.036221	0.0628599
cg07505478	3.9405313	0.398356	38.97967	0.240877
cg03780927	0.1459916	0.0382343	0.557446	0.0048801
cg24215727	1.77E-07	7.46E-14	0.418344	0.0378632
cg16544661	2.99E-09	8.19E-27	1.09E+09	0.3414765
cg11205981	4.6424109	1.1710497	18.40398	0.0289139
cg11902863	2.9321792	1.0682104	8.048671	0.0367941
cg10320160	1.1240543	0.4622174	2.733558	0.7964699
cg22494081	5789.8358	0.1248724	2.68E+08	0.1140041
cg03074244	3.0596114	0.982796	9.525092	0.0536051
cg12659952	0.2117105	0.0376223	1.191349	0.0781823
cg16753846	1.6699333	0.8357842	3.336599	0.146498
cg08425187	3.5192392	0.817116	15.15702	0.0912451
cg12966915	444706.05	1.88E-28	1.05E+39	0.740123
cg05044843	8.2587944	1.3351646	51.0856	0.023155
cg20399462	1.5495591	0.7634458	3.145126	0.2252684
cg14914422	4.24417	1.368827	13.15943	0.0122886
cg26347851	0.2286053	0.0653372	0.799856	0.0209187
cg27583690	0.4106516	0.0401137	4.203924	0.4532893
cg00730172	1.4533907	0.5818386	3.630464	0.4234198
cg00184032	2.6917936	0.6077118	11.92301	0.1922158
cg09838217	1.7491862	0.7409947	4.129115	0.201979
cg00520135	4.3557643	0.9178714	20.67031	0.0640114
cg21901643	1.093247	0.5304242	2.25327	0.8090874
cg05350839	1.5996092	0.653354	3.916329	0.3038212
cg06235991	6.0899573	1.23E-08	3.01E+09	0.8595968
cg05969378	0.3124388	0.1153036	0.846618	0.0221757
cg15824864	737.55238	5.9350797	91655.64	0.00728
cg01284709	2.6471852	0.3884769	18.03863	0.3200924
cg02628016	0.4902616	0.2459355	0.977315	0.0428513
cg27206976	2.0560887	0.4020149	10.51578	0.3866989
cg09259308	0.0784409	0.0009125	6.743096	0.2626648
cg22425186	1.154245	0.4664885	2.855979	0.7563095
cg06970451	0.3117408	0.1317679	0.737526	0.0079801
cg13163833	1.8513003	0.7325884	4.678361	0.1928842
cg08161163	1.2322249	0.3415903	4.445027	0.7497166
cg11838299	0.0292003	0.0016824	0.5068	0.0152364
cg00905524	5.2136432	0.9268576	29.32713	0.0609615
cg03543954	1.8703851	0.4541856	7.702448	0.3859134
cg08993690	0.4685173	0.1568268	1.399687	0.1745295
cg04552378	360448.01	1.50E-17	8.64E+27	0.6264993
cg00373616	1.4843123	0.4204901	5.23956	0.5393928
cg08701515	1.25E-25	1.14E-48	0.013697	0.034135
cg27659014	8085967.3	82.32168	7.94E+11	0.0066878
cg01417313	3.7291384	0.9425571	14.75398	0.0607023
cg06746017	4.9346054	0.7291839	33.39395	0.1017917
cg01472113	0.3286935	0.1493849	0.723228	0.005687

cg17795258	2.0259949	0.8941818	4.590404	0.0906564
cg18653372	1.1376842	0.2968127	4.360748	0.8507488
cg20480723	0.3589722	0.1932135	0.666936	0.0011886
cg07665060	2.4133816	0.837858	6.951549	0.1026329
cg09426825	0.4589485	0.1975849	1.066041	0.0701044
cg08772345	1.2614841	0.5626211	2.828444	0.5728527
cg06192497	2.0421528	0.8843915	4.715545	0.0944788
cg26224354	0.3624385	0.1562387	0.840776	0.0180827
cg13304638	0.1999145	0.0744989	0.536462	0.0013911
cg13629388	2.4402572	0.6633779	8.976565	0.1794673
cg08111922	0.3513288	0.1499365	0.823228	0.0160531
cg08909156	0.2042685	0.0508218	0.821018	0.025233
cg19312305	2.3660322	0.8028044	6.973191	0.1183647
cg05361074	0.4327546	0.1381844	1.355265	0.1504237
cg16458021	4018.329	1.95E-14	8.28E+20	0.6832856
cg11841394	1.6472835	0.9345543	2.903569	0.0843625
cg08212862	0.5203608	0.2484335	1.089931	0.0833305
cg00503302	1.5724313	0.6974523	3.545103	0.2751632
cg02870011	0.5283061	0.2570709	1.085721	0.0825318
cg24892571	2.4978127	0.4715481	13.23103	0.2818392
cg24535650	0.7556065	0.3422676	1.668114	0.4879582
cg25449484	2.502112	0.9522938	6.574194	0.0627742
cg27333547	0.0464454	0.0019782	1.090486	0.0566281
cg06380449	0.1571514	0.0324017	0.762199	0.0216172
cg17316718	1.5695289	0.5550827	4.437935	0.3953239
cg02219997	1.3303079	0.5093488	3.474474	0.5601071
cg10569807	19456.123	5.4503727	69452265	0.0179695
cg15032166	6.1768164	1.3817589	27.61195	0.017163
cg14235783	1.14E-09	3.96E-18	0.326588	0.0382112
cg09130728	0.7117308	0.357998	1.414982	0.3320904
cg20086579	2.9547602	0.7286136	11.98249	0.1293361
cg07371747	0.2484826	0.095233	0.648343	0.0044334
cg17648878	0.0650914	0.0003331	12.71986	0.3100793
cg01675241	2.222014	0.4982363	9.909648	0.2952543
cg20130615	2.8921173	0.631184	13.25183	0.1714842
cg03603211	1.3032757	0.5612339	3.026417	0.5377552
cg23935616	1.9069615	1.041369	3.49204	0.036502
cg22507023	1.0657299	0.3911229	2.903896	0.9009406
cg03891318	0.435244	0.1265911	1.496451	0.1867632
cg07092135	0.3867275	0.1592533	0.939121	0.0358417
cg03344384	2.6520711	0.5256203	13.3813	0.2375624
cg03300596	0.587944	0.3215427	1.075062	0.0845445
cg08053370	3.9091016	0.6490908	23.54228	0.1367003
cg10359332	0.1772989	0.0451184	0.69672	0.0132305
cg06996081	0.8726031	0.1794672	4.242759	0.8658854
cg27627524	0.6365243	0.1919067	2.111251	0.4602572
cg01418188	1.4193051	0.4405827	4.572189	0.557417
cg08595989	0.4270696	0.1531749	1.19072	0.1038859
cg19925872	0.4340961	0.2077074	0.907235	0.0264992
cg21310090	0.4261304	0.1547675	1.17329	0.0987991
cg01999051	0.7050839	0.3364612	1.477565	0.3545861
cg23082877	3.9677804	0.921152	17.09086	0.0643518
cg14567085	1.9534745	0.8281953	4.607684	0.126163
cg01754713	3.4685081	0.6261582	19.21327	0.1544561
cg06900514	0.4503232	0.1844086	1.099682	0.0798829
cg00267196	0.2604714	0.0628844	1.07889	0.0635612
cg17643109	3.821E+12	2.1233223	6.88E+24	0.0441922
cg02261543	0.4559053	0.2457791	0.845677	0.0127138

cg22428691	0.1837203	0.0750238	0.449899	0.000209
cg02451443	3.190863	0.7009032	14.52641	0.1335095
cg00919591	1.805022	0.6254776	5.208987	0.2747559
cg21676617	1.8094004	0.716362	4.570217	0.2097098
cg21887111	19.679822	0.00697	55565.81	0.4623559
cg13327513	7.427002	1.539281	35.83515	0.012521
cg25298683	2.302608	0.6824564	7.769	0.1788804
cg27366866	2.4517025	0.3075146	19.54654	0.3971893
cg15460816	1.427288	0.5170666	3.939823	0.4922339
cg06711254	0.3881357	0.1943426	0.775174	0.0073284
cg25801113	0.3859911	0.1284251	1.160124	0.0899933
cg27410449	0.1592662	0.0161066	1.574871	0.1160722
cg24588278	0.7626758	0.3336671	1.743277	0.5206663
cg00788170	0.0959044	0.0236775	0.388455	0.0010203
cg23845609	0.155364	0.0250182	0.964818	0.0456729
cg12356859	0.9450983	0.4667748	1.91358	0.8753368
cg03847705	0.2968771	0.0972261	0.906505	0.0329813
cg02634861	1.4763874	0.3781819	5.763682	0.5750335
cg00851173	0.0094113	0.0002947	0.300553	0.0082858
cg25155774	0.0456354	0.008342	0.249651	0.0003702
cg15313617	147.86327	0.1755259	124560.2	0.1460275
cg06086198	0.2889868	0.117984	0.707836	0.0066083
cg22534585	1.2211411	0.2074713	7.187432	0.8251628
cg23327859	0.7230297	0.316444	1.65202	0.4417509
cg11995282	0.0056332	0.0001552	0.204431	0.0047089
cg25274468	0.9983968	0.4131129	2.41289	0.9971566
cg04769798	1.2752247	0.6155725	2.641766	0.5129467
cg01837879	2.9116873	0.9863891	8.594907	0.0529719
cg23252586	0.1231084	0.0108509	1.396718	0.0909635
cg06784824	0.3256093	0.1016287	1.043223	0.0589273
cg10996070	8.26E+35	3.6E+09	1.89E+62	0.0075739
cg02235289	11418.134	117.97043	1105139	6.21E-05
cg09854515	1.7455164	0.8755299	3.479981	0.1135648
cg11844856	0.3957035	0.1832845	0.854307	0.0182272
cg10403048	39796.636	4.11E-06	3.85E+14	0.3666313
cg16986661	0.4604532	0.2038084	1.040277	0.0621803
cg14586546	8.0205819	1.0283841	62.55419	0.0469589
cg14935626	0.4522145	0.1531381	1.335383	0.150871
cg09287629	3.1504546	0.7218477	13.74994	0.1269072
cg20847746	0.5594634	0.2689279	1.163878	0.120203
cg13392029	0.1561203	0.0344973	0.706534	0.0159115
cg23973272	1.4472877	0.7480205	2.800246	0.2722813
cg08576827	0.5527326	0.1817499	1.680955	0.2961346
cg04526883	0.5080325	0.140873	1.832126	0.3007687
cg21791023	1.6441162	0.3404333	7.940227	0.5360266
cg16302441	1.8211975	0.5532218	5.995353	0.32406
cg24098131	0.508736	0.2612481	0.990676	0.0468656
cg04033857	0.10654	0.0255425	0.444387	0.002119
cg03271893	0.3358527	0.0967523	1.165833	0.085738
cg10286983	1.5221236	0.6253514	3.704893	0.3546375
cg03061677	28.377863	1.0230241	787.179	0.0484511
cg14240647	2.1525044	0.5166369	8.968146	0.2923761
cg12970937	0.6095253	0.2210692	1.680565	0.3386993
cg23690480	591.09595	0.5200014	671910.5	0.0754362
cg27288968	1.8444292	0.1358161	25.04798	0.6455536
cg14996143	1.9615884	0.7366341	5.223528	0.177567
cg05321510	2.18E-12	6.26E-23	0.076026	0.0301576
cg27589368	1.553782	0.5606877	4.305852	0.3967723

cg14483831	0.5881142	0.2279455	1.517373	0.2723355
cg17924021	0.5864043	0.2809999	1.223737	0.1550181
cg16844941	1.7032556	0.7599166	3.817629	0.1959266
cg06285333	1.3020296	0.3453896	4.908315	0.6966761
cg04993257	0.0764596	0.0232582	0.251355	2.29E-05
cg14112754	2.7256756	1.0775437	6.894669	0.034201
cg14331609	0.4125855	0.1555072	1.094655	0.0753545
cg07675169	1.5941072	0.7180617	3.538941	0.2517912
cg17974424	0.5524529	0.2281119	1.337959	0.188564
cg22587293	0.5175116	0.2219543	1.206637	0.1272387
cg01217666	46.361499	0.0020411	1053077	0.4534785
cg26322137	4.4183539	0.5136944	38.00285	0.1759758
cg02456087	3.7369922	1.4626759	9.547645	0.0058777
cg10543450	1.8100303	0.9359027	3.500588	0.0778802
cg26397172	0.0814911	2.43E-17	2.74E+14	0.8906708
cg01831771	4.0580518	1.3536561	12.16541	0.0124004
cg00622702	1.9735916	0.9037336	4.309969	0.0880137
cg04264633	1.1301825	0.2139045	5.971415	0.8854267
cg22931455	0.414297	0.1945295	0.882344	0.0223433
cg03476860	1.4064204	0.7266781	2.722001	0.3113947
cg10603156	3.2063297	0.97198	10.57692	0.0557104
cg02438107	2.8433111	1.0914403	7.407109	0.0324296
cg22068741	7.748E+17	1.64E-08	3.66E+43	0.1720439
cg06961147	0.1477599	0.0301372	0.724453	0.018406
cg17407411	4796.1102	4.59E-29	5.02E+35	0.821736
cg09265876	0.1769515	0.0813334	0.384981	1.26E-05
cg12601963	2.581075	1.0892982	6.115817	0.0312171
cg13298859	1.3362649	0.5309838	3.362822	0.5381495
cg10999689	0.447922	0.1536676	1.305637	0.1411895
cg21161526	0.6657931	0.301906	1.468273	0.3134078
cg22259330	10.208854	0.0003366	309636.1	0.6590431
cg26234900	1.5168659	0.7034174	3.271005	0.2879306
cg04842962	0.0270632	0.0008214	0.891691	0.0429443
cg08908306	0.2523323	0.0900072	0.707405	0.0088421
cg08154107	1.1221763	0.5602964	2.247524	0.7449706
cg08645670	1.5439948	0.6371912	3.741295	0.3360898
cg00491548	0.0878487	0.0239327	0.322463	0.0002466
cg08034440	1.6949063	0.8195597	3.505184	0.1546736
cg18967835	0.891037	0.0961043	8.261301	0.9191236
cg03668470	31.335368	0.0538306	18240.65	0.2889359
cg08856920	27893.936	0.4479246	1.74E+09	0.0691601
cg06576873	0.0409045	0.0062498	0.267715	0.0008536
cg06903451	0.6028532	0.1970892	1.843998	0.374973
cg23662734	0.5334337	0.0810396	3.511267	0.513356
cg13722127	2.7773299	0.9968402	7.738012	0.0507118
cg05894462	1.8319138	0.7830504	4.285686	0.162715
cg05824218	0.2652375	0.1190623	0.590875	0.0011645
cg19972591	2.0779361	1.96E-06	2205550	0.9177152
cg11756387	1.2019571	0.3938783	3.667887	0.7465744
cg12364136	0.1551593	0.0261417	0.920919	0.0403031
cg17157275	16.081924	1.2384421	208.8336	0.0337163
cg04972856	0.167337	0.0138845	2.016758	0.1592416
cg26753208	0.3120573	0.1270898	0.766228	0.0110553
cg11286405	0.2201741	0.0486041	0.997376	0.0496029
cg11542165	4.6325019	1.1743596	18.27385	0.0285604
cg12355586	0.3797254	0.1810305	0.796503	0.0104087
cg14413165	0.0480867	0.0101834	0.227069	0.0001272
cg20625379	0.4487035	0.1987497	1.013007	0.0537484

cg19358520	2.0958153	0.9207725	4.770388	0.0778554
cg27100990	0.0436715	0.0029321	0.650464	0.023084
cg25810455	0.0308694	0.0039153	0.243386	0.0009625
cg00689492	0.4098153	0.1054943	1.592015	0.1976168
cg13550642	0.3666736	0.073064	1.84016	0.2228473
cg09662034	1.250818	0.6370803	2.455806	0.5155886
cg25090051	2.8252735	0.7035753	11.34515	0.1431159
cg18032164	1.705374	0.4485529	6.48374	0.433411
cg14300636	0.3655218	0.1378949	0.968899	0.0430229
cg12175729	0.1428173	0.0266058	0.766628	0.0232122
cg03569277	1.4555109	0.4494436	4.713633	0.5312745
cg00247426	1.7541345	0.6876162	4.474862	0.2395403
cg22742060	0.612593	0.2837544	1.322518	0.2120127
cg08060987	1.6152824	0.4712886	5.536177	0.4454814
cg19751562	0.7285588	0.3649221	1.454552	0.3693154
cg12911122	1.3205001	0.5191983	3.358486	0.5594089
cg27269488	0.2188678	0.0423688	1.130622	0.0697659
cg18397314	5.7646837	1.0452527	31.79287	0.0443501
cg13777798	0.7659818	0.3074799	1.908184	0.5670044
cg16915316	1.1121979	0.4920784	2.513795	0.7982706
cg01256232	0.8729596	0.4484024	1.699497	0.689363
cg20992785	1.6439733	0.6227075	4.340157	0.3155515
cg10673351	1.1366566	0.4742704	2.72416	0.7739403
cg00621197	1.6814467	0.1185581	23.84706	0.7009411
cg25637972	0.8298495	0.3773966	1.824739	0.6426961
cg22271905	0.0511241	0.0086493	0.302182	0.0010378
cg20674577	1.2367795	0.5144975	2.973044	0.6348661
cg10963058	8.7220386	0.6477337	117.4463	0.1025512
cg06315390	1.3067016	0.7018039	2.432972	0.39897
cg17002510	1.0218187	0.0801468	13.02751	0.9867403
cg00938819	1.0389374	0.4267203	2.529504	0.9329472
cg08649707	5.282516	0.7803318	35.7604	0.088052
cg25188398	0.3695643	0.1400966	0.974883	0.044287
cg23842170	0.5416764	0.1125082	2.607929	0.444529
cg17699047	0.4298629	0.2057202	0.89822	0.0247398
cg20521345	1.38E-23	2.33E-40	8.18E-07	0.0075559
cg13306338	2.9913016	1.4865607	6.019186	0.0021317
cg27513097	1.4701591	0.4819897	4.484262	0.4982239
cg03379797	0.5250851	0.2601701	1.059747	0.0721773
cg16553435	0.0766934	0.0103248	0.569683	0.0120756
cg01933329	2.1893713	0.9887682	4.847796	0.0533455
cg08946854	0.3685418	0.1533905	0.885472	0.0256194
cg14483142	0.3874585	0.1894628	0.792367	0.009389
cg02845988	0.0546849	0.0029009	1.030867	0.0524193
cg23543795	1.9801986	0.5680258	6.903184	0.2835965
cg00269553	2.2924502	1.0133059	5.18632	0.0464043
cg21109666	2.7771019	0.3125922	24.67206	0.3593939
cg27608999	0.2070492	0.0359451	1.192633	0.0779388
cg11757894	3.1281469	0.0056356	1736.336	0.7235447
cg13592780	1.6047728	0.6623775	3.887958	0.2948203
cg12517843	4.9215018	0.4132579	58.61032	0.2073737
cg19828063	1.5393839	0.7332562	3.231753	0.2542743
cg06202228	0.0310895	0.0007911	1.221818	0.0638812
cg13347970	2.5383943	1.2403123	5.195018	0.0107922
cg19917083	0.1604966	0.0119252	2.160054	0.167794
cg14862722	0.4039403	0.1964003	0.830792	0.0137468
cg21878650	0.6422904	0.169299	2.436737	0.5152023
cg06775001	0.0675565	0.0036583	1.247536	0.0700938

cg18406792	1.5470739	0.5656763	4.231108	0.3952828
cg02714677	190.93522	0.1796175	202966.1	0.1396527
cg19563248	0.1836625	0.03162	1.06679	0.0590346
cg12580752	1.6088244	0.8544578	3.029191	0.1408074
cg00292513	1.4487457	0.4689632	4.475541	0.5194785
cg16983282	0.6231961	0.0781984	4.966513	0.6552032
cg02077472	2.5628033	0.9014259	7.28619	0.0775133
cg11088471	1.8253513	0.7340013	4.539375	0.1954394
cg26381452	10.354535	1.7493495	61.2893	0.0099841
cg02625944	1.1263771	0.0078434	161.7567	0.9625461
cg07840143	0.7624967	0.1602213	3.628739	0.7333528
cg25699851	0.8832328	0.2199177	3.547238	0.8610497
cg12231246	1.5081285	0.6856272	3.31733	0.3069873
cg02607810	0.7438078	0.3390619	1.631708	0.4602647
cg26282505	0.2119611	0.0749326	0.599572	0.0034537
cg09521719	2.6443906	1.2411934	5.633934	0.0117398
cg21280914	0.3604887	0.1740197	0.746767	0.0060365
cg09320190	2.3401695	0.765631	7.152784	0.1358334
cg25073089	0.2395166	0.070283	0.816246	0.0223403
cg19379175	2578669.3	0.0005867	1.13E+16	0.1925288
cg01917202	13805627	2.02E-06	9.46E+19	0.2756006
cg19776453	0.2691192	0.0933383	0.775942	0.0151199
cg18141622	0.283116	0.0884758	0.90595	0.0334699
cg25423174	0.9835068	0.3505978	2.758961	0.9747905
cg04910877	2.9082835	1.2598257	6.713717	0.0123812
cg17941579	2.1134895	0.7101007	6.290429	0.1787012
cg23229016	4.5743566	1.1502441	18.19156	0.030874
cg19825600	0.3141502	0.1377973	0.716199	0.0058899
cg08914378	2.99E-14	3.53E-26	0.025247	0.0262477
cg22242539	0.1977376	0.0664316	0.588578	0.0035868
cg17368989	399567.27	0.0042477	3.76E+13	0.1685322
cg01465596	2.4979406	0.7886623	7.911761	0.1196267
cg04728863	0.3631933	0.1538046	0.857643	0.0208741
cg02491396	1.2814453	0.6471613	2.537392	0.4767847
cg20125569	1.1017225	0.0195983	61.93357	0.9624144
cg01809941	0.1973812	0.0745315	0.522723	0.0010929
cg15387272	270.62369	4.2238202	17339.09	0.0083209
cg25015585	0.2961885	0.0938838	0.934428	0.0379256
cg11779273	18.031352	0.4225483	769.4498	0.1310057
cg19255608	0.3004283	0.1010891	0.892847	0.030471
cg05725686	0.0136943	0.0002391	0.784446	0.0377543
cg24996315	1.4613248	0.6421356	3.325575	0.3659045
cg02673323	59.659582	0.1403352	25362.6	0.1854882
cg11579999	0.1203315	0.0313438	0.461963	0.0020346
cg19670923	0.0048121	0.0001476	0.15688	0.0026832
cg02634272	0.2254472	0.0277609	1.830865	0.1633142
cg06279128	8.42E-26	3.88E-55	18299.87	0.0938959
cg22305258	1.2299794	0.6804594	2.223276	0.4931328
cg23034827	0.9557229	0.4851314	1.882802	0.8958486
cg04638205	0.5340159	0.2467993	1.155486	0.1111643
cg10363005	0.4264537	0.2127982	0.854625	0.0162667
cg13960493	0.4901135	0.2251986	1.066664	0.0722865
cg17717442	0.8672615	0.4371248	1.720658	0.6837044
cg26433640	0.4205463	0.1811889	0.976104	0.0437725
cg23701776	9.31E-08	1.06E-17	817.9625	0.1657908
cg08258667	35.088912	0.7650402	1609.369	0.0683409
cg26003023	0.5790738	0.2501558	1.340471	0.2020514
cg11693285	1.0783343	0.5785754	2.009772	0.8123346

cg04680494	0.4844869	0.2257434	1.039798	0.0629132
cg06533788	0.2944954	0.0500003	1.734542	0.1766233
cg10178270	0.8379911	0.4626766	1.517753	0.5597465
cg26091131	0.753634	0.1952289	2.909222	0.6814955
cg05349279	1.9327978	0.57295	6.520128	0.2881454
cg03098643	0.0010679	5.19E-09	219.7777	0.2730427
cg00448191	2.1487483	0.7561274	6.106272	0.1511808
cg04046599	1.5453932	0.7834436	3.048388	0.209177
cg04658243	1.1010413	0.3197351	3.79155	0.8787353
cg09437994	2.9849906	0.9564212	9.316156	0.0596686
cg23710748	2.1705231	0.9150217	5.148698	0.0786703
cg07099331	1.3262445	0.6974806	2.521826	0.389159
cg24151352	0.1759996	0.0068073	4.550366	0.2951498
cg18775845	6.637669	1.1178361	39.41423	0.0372944
cg25042430	1.0299981	0.4476878	2.369723	0.9445706
cg07590002	1.7147518	0.834488	3.523566	0.142223
cg18192602	1.2588797	0.6157719	2.573645	0.5280419
cg19867107	1.5303506	0.7417835	3.157219	0.2494992
cg00283535	0.5822013	0.1723963	1.966157	0.383666
cg15922246	0.1884738	0.0524606	0.677125	0.0105427
cg15456144	0.3981684	0.1961329	0.808319	0.0108036
cg21271945	2.0602106	0.5036402	8.427579	0.314578
cg07478245	170.75622	1.719007	16961.94	0.0284614
cg08966208	1.2273612	0.5562415	2.708204	0.6119053
cg24419164	3.16E-26	9.49E-45	1.05E-07	0.0069696
cg01043616	1.203674	0.4071939	3.558086	0.7374529
cg01690062	1.913524	0.9185325	3.986331	0.0830907
cg05709468	1.6075432	0.3298117	7.835366	0.5569342
cg22777513	0.0166224	7.50E-08	3683.194	0.5141489
cg18300770	8.45E-11	1.01E-31	7.09E+10	0.3453959
cg24794107	176.43083	0.0029382	10594223	0.3568089
cg14281087	6.9243943	0.3716825	129.0005	0.1947243
cg16898498	2.2379425	0.4393206	11.4003	0.3321621
cg06303936	0.5651416	0.2967728	1.076194	0.0824715
cg04094829	8.6032039	0.5838086	126.7798	0.116908
cg19956606	1.88E-17	4.61E-30	7.66E-05	0.0093299
cg13418576	1.4894005	0.6559325	3.381924	0.3410411
cg06887224	1.314612	0.5558242	3.109265	0.5334179
cg03320873	0.3667864	0.140497	0.957545	0.040504
cg03870261	0.2521258	0.1003942	0.633178	0.0033604
cg15639842	2.2231779	0.043268	114.2305	0.6909941
cg11057877	0.0152618	1.60E-06	145.4638	0.3709579
cg01197891	124.7812	0.0026561	5862163	0.379196
cg20128456	7.92E-05	2.76E-08	0.227416	0.0200977
cg09259772	0.3237437	0.1128437	0.928807	0.0359664
cg12366968	2.318822	0.4671581	11.50988	0.3035275
cg07296256	4.9337848	1.2635739	19.26459	0.0216429
cg12437197	6.7416974	0.2640813	172.1079	0.2483128
cg10790685	0.2610092	0.0683012	0.997432	0.0495622
cg13605398	0.3834316	0.1525101	0.964	0.0415591
cg07564291	2.4954066	1.0085723	6.174128	0.0478801
cg19832556	3.7640492	1.0805448	13.11197	0.0373773
cg19471574	2.4088045	0.8782931	6.606382	0.0876633
cg24113496	1.522401	0.6031288	3.842802	0.3736469
cg09318283	0.3597543	0.1147458	1.127912	0.0795147
cg11821439	1.3979807	0.6063253	3.223269	0.4318355
cg23283839	0.2799386	0.0683844	1.145957	0.0766428
cg08943374	0.2671621	0.0084044	8.492629	0.4545386

cg13345380	0.1357004	0.0236943	0.777176	0.0248922
cg12669543	0.3736581	0.1857249	0.751759	0.0057809
cg23762633	0.8608372	0.427112	1.735003	0.6751739
cg24323958	0.4200956	0.1420493	1.242388	0.116961
cg20965743	0.3651031	0.1465938	0.909317	0.0304534
cg14808684	0.0356643	0.0015934	0.798281	0.0355511
cg16727416	0.2734394	0.0847303	0.882436	0.0300683
cg02458499	0.2967823	0.0539608	1.63229	0.1625271
cg18822598	0.4232955	0.1827788	0.980306	0.0448151
cg02578846	939.90478	0.811971	1087996	0.0571593
cg21099332	1.889503	0.7177945	4.973877	0.1975607
cg21698294	2.6167714	0.9716938	7.046965	0.0570205
cg19729914	0.2062583	0.0548289	0.775913	0.0195283
cg21635870	2.2076478	1.0281586	4.740231	0.0422358
cg00081919	1.4099011	0.2850032	6.974733	0.6736629
cg05915593	0.4348193	0.1667081	1.134125	0.0886337
cg16758813	1.9184851	0.9087007	4.050382	0.0874774
cg10332816	5.45E-14	8.10E-24	0.000366	0.008163
cg03277515	0.4042794	0.1611898	1.013971	0.0535584
cg22800830	0.1536387	0.0427349	0.552355	0.0041159
cg17313709	0.4698896	0.1699835	1.298927	0.145441
cg14993464	0.3732531	0.0630436	2.209866	0.2774376
cg00460322	0.1658756	0.0380528	0.723067	0.0167738
cg13578134	0.4856954	0.2527669	0.933271	0.0302189
cg06371582	4.0391824	0.751791	21.7015	0.1036546
cg23624957	0.2950655	0.1129521	0.770801	0.0127269
cg00145955	2.4569084	0.8556793	7.054511	0.0948511
cg14150907	1.1842966	0.5340769	2.626136	0.6771911
cg19746897	1.331734	0.6450444	2.749447	0.4385983
cg08330247	0.3486605	0.1524149	0.797587	0.0125728
cg06126721	0.2125502	0.0584971	0.772305	0.0186493
cg05453378	0.948267	0.2675516	3.360885	0.9344235
cg09949713	7.63E-16	1.32E-27	0.000441	0.0117623
cg01165683	0.7693488	0.3722701	1.589968	0.4789718
cg14175593	5090271.7	755.64547	3.43E+10	0.0005958
cg23178714	1.6977955	0.7517669	3.834313	0.2028407
cg11125787	18.615344	0.2717991	1274.953	0.1751344
cg13509147	0.8997676	0.41333	1.958681	0.7901499
cg05955036	1.1593786	0.2600174	5.169496	0.8462604
cg18165186	17.85948	0.3978959	801.6193	0.1375037
cg12479878	2.6343313	1.1692183	5.935334	0.0194297
cg07811198	0.1476482	0.0436137	0.499843	0.0021084
cg09513309	180.42905	0.0077092	4222852	0.311478
cg10788939	0.3187683	0.13746	0.73922	0.0077208
cg00349061	2.1807279	0.6257558	7.599729	0.2209536
cg19543317	1.1846072	0.1479269	9.486402	0.873196
cg23313005	0.3393682	0.1009985	1.140321	0.0805308
cg08716348	1549751.3	2.41E-28	9.95E+39	0.7196896
cg16946445	32.844546	0.3955316	2727.378	0.1214763
cg12950066	0.1100475	0.0194488	0.622684	0.0125715
cg02964356	0.3922829	0.1705293	0.902401	0.0276954
cg19466818	2.4586226	1.0646076	5.677984	0.0351551
cg05160751	0.7537043	0.4189876	1.355816	0.3452462
cg06500074	0.4648526	0.161732	1.336086	0.1550036
cg14093065	1.0073181	0.5109025	1.986073	0.9832047
cg09071093	0.5376418	0.2132442	1.355529	0.1884269
cg21314978	0.3257349	0.0110203	9.627946	0.5162047
cg18305583	0.5480213	0.0007005	428.7077	0.8595567

cg05956518	1.5907799	0.4923784	5.139504	0.4378381
cg04088932	2.8043505	0.7207426	10.9115	0.1368682
cg06270074	0.0356456	0.0051137	0.248473	0.0007641
cg12293132	1.3535474	0.6728017	2.723077	0.3959942
cg15994182	5.4149886	0.4775003	61.40751	0.1727713
cg17807448	0.4069075	0.1688484	0.980606	0.0451125
cg00055341	1.9330406	0.7388193	5.057591	0.1792354
cg25232725	2.1897179	0.7192038	6.666907	0.1676703
cg21073216	1.3471435	0.5412496	3.352974	0.5218507
cg25369980	1.039673	0.4856621	2.225662	0.9201983
cg01909921	1.5435623	0.8371577	2.84604	0.1643528
cg05254518	1.2578157	0.5953122	2.657598	0.547845
cg05147763	0.2285433	0.0577793	0.903992	0.0353933
cg02279471	0.3337902	0.0810643	1.374414	0.1286273
cg06845571	3.08E-19	2.80E-32	3.38E-06	0.0054004
cg13226669	1.1118367	0.4168032	2.965862	0.8322839
cg15173319	2.5888287	0.8119274	8.254475	0.1078782
cg14497940	1.4915586	0.7075818	3.144155	0.2933323
cg10962754	2.3598827	0.7825999	7.116084	0.1273413
cg04717143	0.5072379	0.1900968	1.35347	0.1752505
cg05241134	1.259328	0.4706401	3.369681	0.6461182
cg17687367	0.4832139	0.224389	1.040584	0.0631242
cg23760300	3.2293782	1.2728065	8.193613	0.0135962
cg17709873	4.6740595	0.9726088	22.46209	0.0541934
cg18860329	2.7951591	1.0344205	7.552939	0.0426942
cg05567435	0.1915312	0.0675646	0.54295	0.0018786
cg15585576	7.0117391	1.3652874	36.01036	0.0196515
cg09451572	1.3243876	0.200315	8.75622	0.7706435
cg16738180	1.5374655	0.576283	4.101805	0.390273
cg05726935	0.8391129	0.2365918	2.976055	0.7859604
cg23376470	0.007799	8.49E-05	0.716335	0.0353245
cg16425713	0.0515665	0.007044	0.377497	0.0035102
cg07126525	2.0649825	0.9328472	4.571116	0.073694
cg20337996	1.0852239	0.5464989	2.155011	0.8152428
cg05837855	0.2481718	0.0892589	0.690007	0.007559
cg14275095	15.146221	2.8475015	80.56466	0.0014369
cg18611122	1.8388353	0.7078148	4.777118	0.2111098
cg07571928	0.1898483	0.0443603	0.812491	0.0250977
cg27089226	1.724226	0.652677	4.555018	0.2717138
cg02535223	0.6610651	0.3567027	1.22513	0.1885393
cg17724687	30.828916	0.1210983	7848.35	0.2251227
cg09634031	5.6990563	0.0048151	6745.262	0.6297907
cg26378982	1.2543121	0.5354523	2.938261	0.6018662
cg24476449	1.8461128	0.4822454	7.067217	0.3707134
cg08572611	0.1643918	0.0342532	0.788968	0.0240609
cg13114315	1.5915803	0.1500603	16.88073	0.6997064
cg08331345	1.2013405	0.513451	2.810821	0.6723249
cg09976142	0.2663286	0.0874146	0.811432	0.0199347
cg19854915	2.0402341	0.7172559	5.803445	0.1812544
cg02132307	0.2404577	0.0763516	0.757285	0.0148938
cg15898267	2.6866068	9.44E-08	76461044	0.910148
cg20981615	2.5916616	0.6989629	9.609537	0.1543622
cg19640589	0.4811215	0.1808835	1.279707	0.1426928
cg12361744	1.5635406	0.7598126	3.21745	0.2247768
cg13875844	4.232954	0.9131036	19.62308	0.0652122
cg24293914	3.29E-05	2.88E-10	3.752789	0.0823194
cg27159421	0.1150788	0.0259213	0.510897	0.0044684
cg25386534	3.19E-06	3.54E-16	28735.78	0.2791897

cg01362358	0.3757789	0.1645197	0.858315	0.0202056
cg15578250	0.2982316	0.0542908	1.638255	0.163916
cg22991148	0.6785919	0.1372081	3.35612	0.6344973
cg02986643	0.3225379	0.1289998	0.806441	0.0155179
cg02945266	0.3495899	0.1351488	0.904285	0.0302004
cg04332534	0.5562786	0.1838876	1.682799	0.299067
cg20529344	2.6469429	0.6254865	11.20137	0.1860109
cg02763290	2.5995249	0.7629148	8.857516	0.1266793
cg20019163	8.6713371	1.5683889	47.94225	0.0132935
cg08013143	7.6801665	0.6025445	97.89311	0.1164476
cg04623172	1.7734934	0.902316	3.485784	0.0965481
cg03110901	0.0371429	5.21E-07	2648.049	0.5635522
cg08691567	2.6635987	1.0853364	6.536921	0.0324569
cg01323777	0.4417535	0.1900019	1.027075	0.0577085
cg19100138	0.8636631	0.3999124	1.865193	0.7090612
cg21638712	0.9672954	0.0400272	23.37559	0.9836745
cg00146676	2.024593	0.8139669	5.035803	0.1292112
cg19761480	0.000214	3.03E-07	0.151056	0.0115784
cg15475967	146946.05	0.0606695	3.56E+11	0.1126639
cg15132565	2.4433743	0.9913402	6.02223	0.0522502
cg07043604	0.3071281	0.145537	0.648135	0.0019481
cg26076054	35.904373	3.1495162	409.3086	0.0039275
cg14126777	0.348251	0.1380315	0.878631	0.0254833
cg02747253	1.5088574	0.7426831	3.06544	0.255369
cg18806716	0.368078	0.1533654	0.88339	0.0252508
cg07698102	2.4114416	0.7637823	7.613492	0.1334651
cg01335658	1.7942784	0.4979506	6.46537	0.3713969
cg18352516	0.4503003	0.1485777	1.364743	0.1584541
cg00538642	0.0285034	1.14E-31	7.16E+27	0.9179585
cg26836183	1.42E-12	1.77E-21	0.00113	0.0090876
cg16312163	1.8593207	0.8221278	4.205032	0.1363382
cg27513684	0.0682348	0.0166393	0.279819	0.0001924
cg00965023	0.4195598	0.1596133	1.102855	0.0781677
cg19197652	18.282335	0.1724909	1937.747	0.2219575
cg13540256	0.1637446	0.0370379	0.723915	0.0170333
cg04880737	0.373207	0.0682322	2.041315	0.2555937
cg15113803	1.4200897	0.3087613	6.531437	0.6523605
cg17330278	2.5998413	0.0094889	712.321	0.7386645
cg18364502	0.4113115	0.0686567	2.464103	0.3307352
cg04702572	1.1013305	0.4023715	3.014451	0.8509726
cg26348521	2.4467234	0.5525106	10.835	0.238589
cg07836663	0.1695849	0.0331728	0.866945	0.03305
cg22506302	0.0241822	2.16E-11	27123312	0.7262688
cg08345979	3.24E-23	5.35E-48	196.4075	0.0753043
cg05858126	1.0238117	0.3275237	3.200349	0.9677197
cg20832181	3.1300495	0.8819333	11.10879	0.0774695
cg00320216	0.9135629	0.2112679	3.950421	0.9036826
cg06861115	1.1778188	0.2770643	5.006986	0.8245805
cg10658666	0.3437996	0.1402333	0.842868	0.0196175
cg03610604	4.4080646	1.2505585	15.53788	0.0210099
cg17626301	1.3113644	0.7127154	2.412851	0.3835758
cg07100411	0.2185189	0.0593752	0.804216	0.0221545
cg05168368	0.4287056	0.1699874	1.081189	0.0727224
cg07901199	9.7838347	1.5110771	63.34781	0.0167052
cg02400433	0.8110341	0.3391294	1.939603	0.6377827
cg22685901	0.2657259	0.1178241	0.599286	0.0014036
cg12722998	43492.859	4.67E-05	4.05E+13	0.3107886
cg24998110	1.9342285	0.7608468	4.917205	0.1658049

cg25639084	0.3618619	0.1070295	1.223438	0.1019456
cg14583583	1.6723196	0.7926498	3.528233	0.1770392
cg01391022	2.0836455	0.2330711	18.6277	0.5112783
cg25592021	0.5506109	0.2794416	1.084922	0.0846317
cg03115470	1.6578851	0.4220914	6.51182	0.4689049
cg27246129	0.2959915	0.1306907	0.670369	0.0035139
cg19767800	1.8433414	0.3598273	9.443162	0.4631238
cg09056317	0.049082	0.0035636	0.676019	0.0242871
cg22527280	0.0010594	2.11E-05	0.053112	0.0006045
cg11581305	0.1267799	0.0057835	2.779152	0.1898278
cg00470183	0.7777504	0.3159616	1.914459	0.5844497
cg04242655	0.5687446	0.2341809	1.381285	0.2125859
cg16410115	2.0281603	0.770599	5.33797	0.1520912
cg02616160	0.4552659	0.0060827	34.07476	0.7208081
cg18527583	0.2659788	0.0899449	0.786534	0.0166643
cg21150486	1.98E-34	1.37E-83	2.86E+15	0.1790319
cg14604066	2.6627892	0.5575882	12.71628	0.2195552
cg14527456	2.2392269	0.7771599	6.451873	0.1354285
cg12716367	0.6318766	0.1801786	2.215957	0.4733295
cg23061150	0.5734186	0.2375066	1.38442	0.2162153
cg15405572	0.7951291	0.4031599	1.568187	0.508243
cg15773312	3.0774468	0.594176	15.93918	0.1803788
cg10796899	0.0110171	2.11E-05	5.761521	0.1580586
cg17165580	0.4088944	0.1991004	0.83975	0.0148661
cg16449084	0.1352293	0.0501871	0.364376	7.61E-05
cg21589115	0.0820597	0.0053219	1.265293	0.0732327
cg22933646	0.0162303	8.18E-05	3.218373	0.1267931
cg05775980	6.8096751	1.2509914	37.06794	0.0264869
cg24496978	0.0374927	0.0020797	0.675933	0.0260541
cg13380103	2.4451384	0.2770928	21.57653	0.4209479
cg08682036	0.987683	0.4451337	2.191516	0.9756857
cg26321153	0.4612477	0.1476527	1.440878	0.183029
cg23749029	0.6697124	0.2411033	1.86026	0.4418149
cg05924540	2.4355627	1.0863161	5.460626	0.0306995
cg02334109	2.1753183	0.73062	6.476704	0.1626737
cg01696605	1.5684543	0.7067537	3.480772	0.268456
cg20036770	2.83E-07	1.08E-12	0.074089	0.0178464
cg02121330	0.0001624	5.37E-07	0.049085	0.0027499
cg19274612	0.3928024	0.1631718	0.945591	0.0370893
cg08327690	1.559373	0.7618807	3.191634	0.22408
cg11679069	0.2959912	0.10206	0.858425	0.0250283
cg07710907	3.63E-06	1.20E-10	0.109244	0.0172763
cg20904010	2.1168856	0.6625364	6.763711	0.2057447
cg15038938	4.60E-07	9.89E-17	2138.404	0.1988511
cg17560340	0.4159296	0.1748597	0.98935	0.0472357
cg11696165	0.8657232	0.3055033	2.453252	0.7861456
cg10861953	0.2891839	0.0786257	1.063613	0.0618802
cg16606561	0.2518921	0.0545532	1.16308	0.077326
cg25221196	0.9649986	0.4161435	2.237743	0.9338329
cg09399236	7.85E-14	8.40E-22	7.33E-06	0.00127
cg01967399	1.4084908	0.7099451	2.794366	0.3271295
cg10821226	14.525436	1.0916692	193.2713	0.0427256
cg03045231	0.6268249	0.3205418	1.225767	0.1722379
cg10380221	1.8311337	0.531056	6.31393	0.3381374
cg11711420	0.1368205	0.0306727	0.61031	0.0091287
cg07084627	0.4655211	0.2484274	0.872327	0.0170209
cg08900864	0.1915301	0.079116	0.46367	0.0002485
cg22518433	4.0511593	0.3106971	52.8228	0.2856196

cg10679301	0.1801018	0.0325642	0.996084	0.0494767
cg19488785	1.5216162	0.6790105	3.409838	0.3078986
cg19028997	0.6370007	0.3109436	1.304963	0.2177545
cg13864546	3.1809252	0.3259434	31.04307	0.3194795
cg19600494	1.7815514	0.8109435	3.913868	0.1504041
cg13410614	2.2059771	0.5406624	9.00069	0.2701187
cg22683277	1.0385703	0.535414	2.014568	0.9108615
cg01581326	0.1597451	0.018208	1.401497	0.0978566
cg26869615	0.486752	0.2001651	1.18366	0.1122712
cg17359975	0.3782722	0.1575537	0.908198	0.0295965
cg24086266	1.4101198	0.6091525	3.26427	0.422262
cg21863949	0.5512195	0.1088011	2.792647	0.4718601
cg00435173	0.4577479	0.180823	1.158775	0.0991484
cg21436055	0.5941116	0.1480156	2.384671	0.4627496
cg05751148	1.4701765	0.4403074	4.908887	0.5309942
cg06669276	2.0225081	0.8569765	4.773222	0.1079078
cg08270682	0.3791095	0.1789914	0.802966	0.0113072
cg19285797	0.339321	0.1587471	0.725296	0.005293
cg16530970	1.8458632	0.2222477	15.33069	0.5703721
cg07449091	2.0771894	0.5706205	7.561446	0.2674687
cg04838627	5.0382106	0.9797109	25.90924	0.0529375
cg16274476	1.5406666	0.7410456	3.203114	0.2471004
cg14532541	0.213981	0.0598285	0.765318	0.0177252
cg14514569	1.6793383	0.8090908	3.485613	0.164111
cg01460435	2.0021027	0.9657451	4.150593	0.062005
cg23201907	0.3322921	0.1425459	0.774614	0.0107293
cg00585551	0.6279653	0.2802254	1.407226	0.25841
cg08842907	0.4566211	0.1601399	1.302004	0.1425613
cg09137125	1.7281848	0.8628789	3.46123	0.1226401
cg22533606	0.0263248	0.0017552	0.394827	0.0084738
cg22567613	0.0865727	0.0173264	0.432567	0.0028737
cg02489908	1.4533767	0.5730644	3.685979	0.4310359
cg24766553	6.2166137	1.0018327	38.57559	0.0497706
cg21572957	2.4507308	0.7197678	8.344471	0.1515887
cg27505504	0.5516094	0.2697617	1.127932	0.1030805
cg12691330	2.1658893	0.7669312	6.116685	0.1445643
cg08287175	4.4355254	0.1651937	119.0958	0.3748871
cg00039147	1.3440896	0.3915151	4.614323	0.6384281
cg11076970	0.6847684	0.1830716	2.561335	0.5737049
cg12079548	1.6329665	0.5588568	4.77149	0.3700447
cg02649597	5.3050907	1.9388816	14.51558	0.0011572
cg26676034	2.0116461	0.6748766	5.996237	0.2097318
cg00660096	1.9617462	0.2686377	14.32579	0.5065262
cg07157248	0.5142197	0.2291072	1.154141	0.1068698
cg18559579	2.4518658	0.2692167	22.33014	0.4262009
cg00209893	0.0123606	0.0008226	0.18573	0.001485
cg04115307	0.0654695	0.0144648	0.296324	0.0004019
cg00483030	0.321826	0.1459519	0.709631	0.0049515
cg08711067	4.0054484	1.0578777	15.16585	0.041073
cg17608490	2.5747896	1.1313528	5.859836	0.0241896
cg02366988	0.0035104	0.000137	0.089941	0.0006368
cg20681747	1.19E-05	1.09E-10	1.300087	0.0554136
cg25157604	0.4132217	0.1624843	1.050884	0.0634896
cg10960969	0.6312042	0.2955667	1.347982	0.2345981
cg14181940	2.1160741	1.0697596	4.185772	0.031262
cg06008926	1.9624022	1.0578013	3.640591	0.0325019
cg27160395	7.5114303	1.3100993	43.06665	0.0236289
cg24588162	2.0016005	0.8508333	4.708801	0.1118642

cg11650874	9.5634574	0.5846328	156.4396	0.1133027
cg02492405	0.0683597	0.0108333	0.431361	0.0043099
cg20297791	14.597281	0.009744	21867.96	0.4723892
cg22933133	2.0516011	0.8902919	4.727738	0.0915761
cg00835825	0.3429989	0.150317	0.782668	0.0110177
cg22363224	0.0167809	0.0002006	1.403888	0.0703327
cg12729305	0.8240391	0.3525003	1.926354	0.6550888
cg22007486	2.8956561	1.3651815	6.141912	0.0055822
cg08981626	0.5160461	0.2349231	1.133577	0.0994156
cg10372829	2.1508983	0.1477711	31.30764	0.5751124
cg27191207	0.9643731	0.3709191	2.507327	0.9406813
cg11049042	0.0647418	0.0013287	3.154704	0.1674212
cg13835337	0.210644	0.0762939	0.581579	0.0026472
cg14791054	1.0985764	0.5310048	2.272804	0.7999116
cg08853571	0.4549843	0.199024	1.040129	0.0619437
cg08971667	2.476298	1.0859548	5.646691	0.0310814
cg08065733	2.1978648	0.8828558	5.471573	0.0906029
cg17716724	3.4188084	1.0989552	10.63578	0.0337607
cg12863565	0.4875172	0.2314111	1.02706	0.0587937
cg11528176	0.3974212	0.2102213	0.75132	0.0045122
cg21915935	3.13E-38	4.45E-78	219.7509	0.065071
cg11841288	3.8446208	0.4581539	32.26233	0.2146843
cg12785993	0.2260191	0.0828569	0.616541	0.0036778
cg19682134	0.452098	0.1588861	1.28641	0.1367734
cg27083087	0.4319654	0.1641591	1.136666	0.0890441
cg16964716	0.4673604	0.194173	1.124903	0.0896335
cg10484211	0.525442	0.2518608	1.096198	0.0863154
cg16063747	1.8655025	0.8511143	4.088874	0.1193924
cg15187040	1.9554954	0.8158922	4.686848	0.1326504
cg13065735	0.3514104	0.1215937	1.01559	0.0534342
cg03302088	0.2544621	0.0807381	0.801988	0.019454
cg19201068	0.5477497	0.2514663	1.193121	0.1296642
cg24434800	2.5423393	1.1940374	5.413138	0.0155255
cg07171867	1.48E-33	2.37E-53	9.30E-14	0.0011535
cg12204395	1.9392788	0.7646234	4.918502	0.1630787
cg01422009	0.4357314	0.1659416	1.144149	0.0916859
cg02246725	2.7442284	0.7815873	9.63525	0.1151652
cg13451483	820.1416	9.3950474	71594.34	0.0032571
cg04763558	0.5850043	0.2377359	1.439538	0.2432229
cg11647493	0.1799677	0.0619387	0.52291	0.0016254
cg03333893	3.648529	0.8098439	16.43744	0.0919241
cg25582488	0.1441701	0.0450762	0.461108	0.0010947
cg06521347	0.4304031	0.147191	1.258547	0.1235812
cg10480239	0.2848812	0.0972056	0.834903	0.0220868
cg06439293	0.6265149	0.2987636	1.313818	0.2158755
cg25890074	5.9930217	0.5363186	66.96824	0.1459353
cg06521359	0.2537897	0.0801919	0.803189	0.0196578
cg13904842	0.8595116	0.4555434	1.621712	0.6402351
cg06363485	0.4010196	0.1266308	1.269966	0.120276
cg20626616	0.4793111	0.1890366	1.215316	0.1213394
cg09706929	0.3173942	0.1423769	0.707552	0.00502
cg20062978	0.0546535	0.003721	0.802745	0.0339866
cg08912801	1.5176205	0.6389724	3.604494	0.3445845
cg03255846	1.7855261	0.9093717	3.505831	0.0921823
cg18981979	1.0799878	0.4227221	2.759197	0.8722594
cg02395280	5.447754	1.312306	22.61517	0.0195854
cg22495058	0.0659972	0.0089652	0.485838	0.0076141
cg22372285	4.7841823	0.458728	49.89536	0.1906987

cg00941836	0.3155119	0.1135497	0.876688	0.0269417
cg10852165	0.5598326	0.05868	5.341044	0.6141935
cg03100906	3.8514035	0.5399529	27.47149	0.1785672
cg10822352	0.4385556	0.1673383	1.149355	0.0935834
cg00449941	12033.17	7.79E-15	1.86E+22	0.6601596
cg27242726	0.619516	0.0253613	15.13332	0.7690154
cg19451435	2.70E-05	1.18E-07	0.006192	0.0001485
cg15482928	3.3695415	0.5887297	19.28527	0.1723273
cg25596297	0.0042592	2.42E-09	7503.151	0.4569281
cg22638105	0.2788536	0.0826664	0.94064	0.0395318
cg20704602	1.4559368	0.5522901	3.838113	0.4475212
cg00732251	1.653843	0.556163	4.917976	0.3655645
cg18793086	0.420896	0.2110925	0.839222	0.0139796
cg24516799	1.5343277	0.0661325	35.59765	0.78958
cg23941787	1.1135127	0.3345205	3.706531	0.8608943
cg00268945	0.528819	0.2308036	1.211634	0.1320308
cg22509164	0.4599593	0.1396983	1.514424	0.2014834
cg03573961	0.1469992	0.0337632	0.640009	0.0106323
cg10416294	1.9007645	1.0122016	3.569354	0.0457507
cg03554749	1.7925528	0.9469303	3.393328	0.0730544
cg26632948	2.2931861	1.0683379	4.922321	0.033206
cg23413104	2.2856831	0.840648	6.214666	0.1052681
cg09438605	0.7836824	0.0808011	7.600863	0.8334542
cg05149386	1.1921784	0.6693283	2.123456	0.550622
cg18433784	0.0218007	0.0006812	0.697692	0.0305
cg24718275	0.4537554	3.79E-16	5.43E+14	0.9644179
cg13604553	0.0955204	0.0153763	0.59339	0.0117355
cg02362103	3.1513006	1.3740494	7.22732	0.0067226
cg12609052	1.9175829	0.1836494	20.02252	0.586455
cg25537993	0.6198444	0.3269209	1.17523	0.1428396
cg15651099	8.54E-08	7.89E-19	9250.256	0.2092994
cg20547777	0.3448753	0.1448471	0.821135	0.0161635
cg21723486	0.5281821	0.2346363	1.188973	0.1231073
cg09762021	1.6047981	0.4727471	5.447684	0.4481391
cg09789768	1.7910155	0.854743	3.752866	0.1225625
cg00666696	0.4320924	0.1758645	1.061635	0.0673161
cg00391945	2.7526867	0.8785772	8.624495	0.0822465
cg27319216	1.1366517	0.5126485	2.520201	0.7525459
cg02756849	9.54E-35	7.87E-81	1.16E+12	0.1479199
cg05394800	3.2658477	1.0949579	9.740796	0.033782
cg04340435	0.245511	0.085908	0.70163	0.0087579
cg05540913	1.3363357	0.6223182	2.869582	0.4571425
cg14522298	0.3257069	0.107994	0.982323	0.0464118
cg06822360	1.4490084	0.6441442	3.259558	0.3699154
cg01239708	1.1320995	0.5595034	2.290691	0.730061
cg01701634	2.2749055	0.9906109	5.224246	0.0526568
cg00925953	0.4256255	0.1688942	1.072607	0.0700894
cg17401635	0.0019757	4.18E-06	0.93425	0.0475231
cg13333954	0.246187	0.0842049	0.719769	0.0104463
cg12307314	2.2515212	0.8657548	5.855408	0.0960425
cg25961816	1.509099	0.7624913	2.986762	0.2374232
cg21762523	3.4381611	1.4444657	8.183616	0.005253
cg15929395	2.3549411	0.753089	7.364	0.1408951
cg18617219	0.5124078	0.255887	1.026085	0.0591225
cg14293575	3.5109167	0.4269605	28.87044	0.2426982
cg18127398	0.5128797	0.2045712	1.285839	0.1544903
cg07784042	0.4743608	0.2277784	0.987882	0.0463121
cg03214622	0.4059056	0.1834943	0.897899	0.0260254

cg00917471	0.2672186	0.0733696	0.973233	0.0453811
cg19628148	1.8725102	0.7825144	4.480805	0.1588144
cg18806438	1.351E+15	5.61E-05	3.25E+34	0.126005
cg13826666	1.4523071	0.545146	3.869047	0.4554237
cg13837997	2.6636731	0.7254891	9.779822	0.1398448
cg17443668	0.6325865	0.3132205	1.277584	0.2016387
cg04090347	0.568916	0.2202291	1.469676	0.2441027
cg02604095	3.4764944	0.514424	23.49427	0.2012037
cg08022524	0.4175539	0.1231313	1.415978	0.1610012
cg14681958	1.3483003	0.4297515	4.230151	0.6084626
cg07229027	32.883374	3.48289	310.4652	0.0022934
cg21364111	1.73E-11	2.98E-22	0.998702	0.049988
cg24291203	2.875E+10	1.67E-09	4.94E+29	0.2865677
cg03622431	1.3823564	0.0283423	67.42253	0.8703151
cg09550083	3.1659589	0.6719435	14.91687	0.1450508
cg21217270	0.4118451	0.1289358	1.315511	0.134352
cg09369190	0.5109546	0.2015888	1.295085	0.1570548
cg23698124	487.22533	0.0048082	49371671	0.292634
cg08913523	1.4923492	0.7248897	3.072338	0.2771802
cg25198661	0.2932521	0.1081739	0.794986	0.0159149
cg13418235	2.4375956	1.1392538	5.215582	0.0216815
cg14009588	0.0526909	0.0059904	0.463461	0.0079734
cg08183074	2.2892856	0.8006276	6.545901	0.122313
cg16681083	0.2125166	0.0015364	29.39573	0.5380497
cg10393086	7.2196424	0.9292535	56.09152	0.0587822
cg03762237	0.3995522	0.1631257	0.978644	0.0447288
cg02994974	0.2499867	0.0742301	0.841887	0.0252358
cg21140898	1.0883918	0.6096721	1.943006	0.7745289
cg01151584	4.1014505	0.9994643	16.83091	0.050087
cg19932079	2.7259071	0.8104802	9.168107	0.1051423
cg26019472	0.2257457	0.0862305	0.590987	0.0024365
cg12737660	4.452876	0.6047211	32.78884	0.1425965
cg09484870	0.0373752	7.96E-06	175.5454	0.4460976
cg00510956	0.4226096	0.035602	5.016546	0.4950272
cg14808040	0.0645757	0.0171024	0.243827	5.30E-05
cg17751414	0.475981	0.2252797	1.005674	0.0517582
cg15806038	0.1146382	0.003888	3.380084	0.2096431
cg02005873	1.8832889	0.5890515	6.021166	0.2857532
cg15340270	6924.8014	22.770549	2105916	0.0024343
cg25520960	3.5211362	0.8673653	14.29432	0.078254
cg02432528	0.0018077	1.27E-05	0.257725	0.0125688
cg19721787	0.3480351	0.1299147	0.932369	0.0357955
cg03029127	0.0227924	0.0007474	0.695102	0.0301177
cg00442907	0.5253033	0.2543694	1.084814	0.0818694
cg02705374	63.993209	0.803711	5095.278	0.0625859
cg09754948	471.59378	0.0006457	3.44E+08	0.3714979
cg22107533	2.4229032	0.8342234	7.037036	0.1037846
cg14079333	3.1029268	1.1518607	8.358784	0.0251178
cg24460268	0.2954535	0.0980804	0.890012	0.0302299
cg08317891	1.9391301	0.1383052	27.18788	0.6230343
cg03774957	1.0733183	0.0092946	123.9443	0.9767043
cg26716046	0.0526514	0.0051826	0.534904	0.0128138
cg13167431	1.5580608	0.5213972	4.655862	0.4272218
cg08560387	3.0665102	1.1205837	8.391595	0.0291374
cg00708736	0.1395543	0.0243902	0.798494	0.0269102
cg21871213	6.5291846	0.0146046	2918.967	0.5467796
cg14018153	3.058256	1.1519826	8.118985	0.0248335
cg26155170	1.6137577	0.5093886	5.112431	0.415973

cg10287113	1.872764	0.6050014	5.797086	0.2764634
cg11539674	0.4554093	0.1857957	1.116267	0.0855211
cg26937434	1.2597761	0.5925608	2.678267	0.5484348
cg27659622	1.5162192	0.7071136	3.251133	0.284857
cg08158160	1.4159984	0.3065953	6.539734	0.6559106
cg09017117	0.2979704	0.1104368	0.803956	0.016809
cg00740870	5.4737037	0.7809532	38.36521	0.0870622
cg14693112	0.5558991	0.1256197	2.459995	0.439075
cg23844527	0.3377351	0.136678	0.834553	0.0186823
cg09788586	0.7421587	0.1375102	4.005517	0.728836
cg02094252	2.3963099	1.015758	5.653218	0.0459701
cg22864672	0.9758913	0.4788094	1.989025	0.9464431
cg17323243	8.8418034	8.94E-23	8.74E+23	0.9356981
cg25191332	1.4018081	0.5263664	3.733266	0.4991397
cg18221580	0.181784	0.0520928	0.634357	0.0075012
cg07188104	0.4914825	0.1841073	1.312034	0.1562282
cg12406726	1.5245787	0.5466618	4.25188	0.4203081
cg18630748	0.4115346	0.172419	0.982262	0.0454695
cg10381076	672027835	14113.128	3.2E+13	0.0002168
cg03133378	104.81044	0.0190497	576660.7	0.2897564
cg06110802	1.7252397	0.4831288	6.160783	0.4010344
cg22395807	1.1953046	0.1494207	9.561947	0.8664614
cg20697025	0.1362143	0.0347686	0.533652	0.0042182
cg21196365	0.3570166	0.1783219	0.71478	0.0036375
cg06493612	0.3129557	0.1062079	0.922166	0.035124
cg03801830	2.5975125	0.9025388	7.475658	0.0767541
cg14598387	0.4453343	0.1338796	1.481351	0.1871169
cg22418337	0.5521393	0.1804727	1.689218	0.2978497
cg15460348	0.6978517	0.3908182	1.246096	0.2239184
cg05512157	0.2281857	0.1134766	0.45885	3.39E-05
cg25521254	0.360368	0.0414505	3.133016	0.3549735
cg26269038	1.955755	0.7486602	5.109097	0.1709602
cg19936885	2502935.4	1.28E-10	4.89E+22	0.44142
cg13905606	0.1047314	0.0329113	0.333279	0.0001333
cg23051299	0.0448858	0.0022989	0.876402	0.0406602
cg24933709	3.6328509	0.8483351	15.55707	0.0821536
cg19719902	2.1868687	0.97627	4.898639	0.0572236
cg27244379	2.19E-12	4.92E-22	0.009767	0.0178704
cg05412855	1.4812588	0.0631157	34.76357	0.8072138
cg16201418	0.6057648	0.3047834	1.203973	0.1526314
cg04805577	2.2488153	0.9286559	5.445688	0.0725049
cg03448635	5.042984	1.689193	15.05553	0.0037387
cg18915128	0.1388236	0.0335515	0.574401	0.0064272
cg14166009	0.2479712	0.0874499	0.703142	0.0087345
cg11246580	4.92052	1.1307465	21.41198	0.033692
cg04652903	32.667223	1.2888011	828.0157	0.0345332
cg05141014	0.280812	0.1270775	0.62053	0.0016922
cg13227806	0.2942302	0.1180231	0.733512	0.0086672
cg24493553	7.6600961	1.6713039	35.10856	0.0087627
cg05800983	0.8366684	0.3496233	2.002195	0.6887457
cg01521688	17545895	2.95E-10	1.04E+24	0.3973105
cg11937107	2.4547248	0.8805181	6.843328	0.0860316
cg12974599	1.4768647	0.7034886	3.100447	0.3027833
cg24804782	0.2975386	0.1179854	0.75034	0.0102117
cg07497315	6.19E-18	1.58E-41	2420444	0.152827
cg25643229	0.3038147	0.0941088	0.980816	0.0463317
cg24515136	0.5261466	0.0688402	4.021344	0.5360059
cg26108329	0.3602567	0.1311801	0.989364	0.0476239

cg27573954	0.4696524	0.2123044	1.038949	0.0620914
cg08380391	0.9760085	0.3157269	3.017141	0.9663611
cg23519086	4.8537925	0.2110878	111.609	0.3233628
cg13615516	2.4084653	0.773659	7.497755	0.1292529
cg15012161	1.5419974	0.5985137	3.972768	0.3697688
cg01073788	0.0103315	3.38E-06	31.61789	0.2641705
cg13012115	0.7321697	0.3581113	1.496944	0.3929104
cg11074047	1.4213117	0.6563832	3.077664	0.372439
cg27024932	0.0317654	0.0008864	1.138335	0.0588907
cg19612068	0.0913047	0.0194236	0.429196	0.0024366
cg21686171	0.3216114	0.1621249	0.637989	0.0011706
cg02751808	1.6153659	0.7361126	3.544847	0.2317231
cg17583946	3.3451294	1.0412545	10.74655	0.0425751
cg08843279	9.649E+16	72.84748	1.28E+32	0.0277112
cg06011086	1.5446704	0.6315254	3.778164	0.3406901
cg15331332	2.4222133	1.0061533	5.831236	0.0484216
cg13322290	0.008563	1.45E-06	50.70602	0.2827769
cg02764897	0.9330157	0.3368382	2.584381	0.8938924
cg23517797	0.8757675	0.4007186	1.913983	0.7394769
cg09752496	0.5257889	0.2524122	1.095248	0.085985
cg25268422	1.0370028	0.3591412	2.994295	0.9464545
cg18971054	0.5422669	0.2218254	1.325607	0.1796245
cg06721806	3.0195919	1.0833595	8.416352	0.0345959
cg02265758	2.0376229	0.7990917	5.195783	0.1361298
cg24329783	0.3151485	0.1282202	0.774594	0.0118483
cg03924164	0.1467892	0.0382397	0.563474	0.0051772
cg21513385	1.2231581	0.5456368	2.741962	0.6247819
cg01302668	3.3425914	1.0340601	10.8049	0.0438089
cg02079348	3.2516223	0.6345475	16.66234	0.1572492
cg11186011	6.667872	1.0818592	41.0964	0.0408785
cg05934015	1.9025268	0.2012748	17.98341	0.5746581
cg10535845	0.3152821	0.1364802	0.728331	0.0068922
cg15781397	2.9954638	0.8364542	10.72719	0.0918753
cg08456247	3.7864949	0.4957223	28.92253	0.1993188
cg11075121	3.40E-07	5.68E-21	20366385	0.3574704
cg22116328	2.6691229	0.7227514	9.857078	0.1407905
cg05437823	3.2053497	1.2517757	8.207754	0.0151796
cg15618533	2.8555966	0.6953422	11.72722	0.1454395
cg08318899	0.3432088	0.1007219	1.16948	0.0873264
cg13691684	2.2069169	0.8874276	5.488315	0.0885629
cg25871427	28.794019	1.2476827	664.5083	0.0358928
cg14763352	0.4066355	0.1812054	0.912514	0.0291123
cg00392075	11.655331	0.235127	577.7591	0.2175448
cg07388018	1.760469	0.7951269	3.897807	0.1631207
cg14046477	0.5874604	0.266666	1.294165	0.1868164
cg18528216	4.56E-05	4.63E-14	44965.13	0.3441642
cg27491759	0.2487878	0.0598295	1.034529	0.0557126
cg13824242	66714.467	1.04E-11	4.28E+20	0.5497291
cg11742207	2.0071381	0.6996624	5.757925	0.1950689
cg13680362	1.6230809	0.6495183	4.055916	0.2999777
cg01752189	7.0682674	0.3317819	150.5821	0.2101887
cg24051481	0.4591086	0.0999815	2.108197	0.3168445
cg06784218	0.1517584	0.0453098	0.508292	0.0022341
cg15963552	2.4260826	0.942897	6.242333	0.0660589
cg00476243	0.1387272	0.0267357	0.719832	0.0187087
cg27400447	2.3788438	0.3613469	15.66057	0.3674274
cg02101833	2.4933336	0.8417734	7.385257	0.0991341
cg21493505	0.1352127	0.0318957	0.573196	0.0066246

cg14439529	0.014861	2.27E-06	97.41185	0.3478699
cg12253828	52833.969	0.6456031	4.32E+09	0.0595444
cg17112382	1.5475412	0.5715879	4.189878	0.3901829
cg10715529	2.6949675	0.7360277	9.867631	0.1343603
cg23095383	23.865172	3.1939377	178.3211	0.0019904
cg00292447	0.3307075	0.1175881	0.93009	0.0359641
cg04264726	1.8381515	0.8406635	4.019208	0.127226
cg12904924	2.0474348	0.9468121	4.427477	0.0685962
cg27022615	9.0209449	9.29E-14	8.76E+14	0.8935181
cg09085932	0.1498279	0.0545421	0.411579	0.0002316
cg13736376	2.6796438	1.1939598	6.014014	0.0168592
cg05941025	1.0476751	0.3509282	3.127771	0.9334871
cg20318845	0.892278	0.4461439	1.784537	0.747232
cg24187797	0.0432205	0.0052285	0.357278	0.0035566
cg25371169	2.2697298	0.8510687	6.053182	0.1014742
cg07789940	0.1355555	0.027994	0.656402	0.0130263
cg07632934	0.1836597	0.0568397	0.593439	0.004626
cg16409970	6.40E-06	7.84E-16	52246.53	0.3044098
cg15784646	0.1575663	0.0467537	0.53102	0.0028726
cg16119483	1.68059	0.6614922	4.269714	0.2751526
cg01691160	0.2364638	0.0728946	0.767068	0.0163223
cg22752023	0.0026445	5.29E-05	0.132133	0.0029379
cg04454664	0.3450123	0.1565376	0.760415	0.0083093
cg01014113	0.0558714	0.0001026	30.42986	0.3694903
cg03454607	0.8153235	0.1765688	3.764835	0.7936544
cg15964309	1.6733321	0.8689734	3.222239	0.1235891
cg06841846	1.1237353	0.6213554	2.032301	0.6995754
cg04901053	0.2775982	0.0903197	0.8532	0.0252803
cg13058734	0.0546966	0.0045965	0.650871	0.0214575
cg08299265	3.0472498	1.0858788	8.551352	0.0343057
cg10293804	1.3754284	0.7291673	2.594471	0.3248793
cg16629695	0.7093801	0.2163838	2.325591	0.5708516
cg15310873	2.9090069	1.1878259	7.12421	0.0194594
cg14577716	0.6034027	0.2965208	1.227889	0.1634346
cg10233654	0.1432209	0.0009147	22.42452	0.4510187
cg20702913	0.2258059	0.0992179	0.513902	0.0003902
cg06325088	0.5490991	0.1892589	1.593108	0.2699958
cg21536042	0.7550975	0.2749638	2.073626	0.5857485
cg22803642	1.6432205	0.7242522	3.728223	0.2347686
cg08168400	0.0311716	0.0034494	0.281689	0.002015
cg18003362	1.0104008	0.4114672	2.481145	0.98199
cg11382133	1.7282448	0.6637153	4.500167	0.2625092
cg04251886	0.3310473	0.1597146	0.686176	0.0029518
cg02849750	0.1098757	0.0189444	0.637268	0.0138035
cg14065167	0.0088449	0.0002351	0.332722	0.0106325
cg14579864	1.2360404	0.6553557	2.331247	0.5127206
cg21170796	2.1894789	0.9755966	4.913729	0.057425
cg03517923	0.6160337	0.2869579	1.322485	0.2139154
cg25618573	0.0728	0.0040971	1.293556	0.0743198
cg05702747	1.5301385	0.7541054	3.10477	0.2387088
cg09022422	0.0488867	0.0047859	0.499367	0.0109076
cg01165177	39.137142	2.843051	538.7578	0.006126
cg20522104	0.4585388	0.1918429	1.095989	0.079465
cg27227598	0.4328879	0.1550139	1.208872	0.1100552
cg12955121	7038.3474	7.7637858	6380693	0.0107771
cg13416889	0.2282307	0.0961481	0.541761	0.0008092
cg01172183	1.8388984	0.8419607	4.016277	0.1264209
cg14983135	0.5852025	0.2689214	1.273465	0.1768238

cg11263074	1.7504958	0.7706743	3.976045	0.1810148
cg06365535	3.3182091	0.9834293	11.19604	0.053232
cg16731811	2.6808432	1.1330637	6.342909	0.0248148
cg19609315	0.0252026	0.0002015	3.151799	0.1351728
cg14525310	2.1765988	0.8562481	5.532955	0.1022737
cg19447496	0.1215766	0.0089971	1.642844	0.1126797
cg16321975	1.9235455	0.7111397	5.202954	0.1975651
cg19229344	1.3322738	0.6557128	2.706907	0.4276831
cg08269941	0.1594482	0.0067841	3.747573	0.2543628
cg01944137	1.7242086	0.4069404	7.305481	0.459606
cg20722590	0.2336692	0.08344	0.654378	0.0056559
cg18955629	0.2171228	0.093419	0.504633	0.0003861
cg07596106	1.0501922	0.2267067	4.864893	0.950077
cg24645611	0.7338176	0.3938675	1.367181	0.3296329
cg07791834	1.6348521	0.2577453	10.3697	0.6020037
cg17518215	3.5066613	0.9118888	13.48484	0.0678889
cg08713568	3.2245051	1.1018282	9.436529	0.0326013
cg13049483	3.2007848	1.2696487	8.06918	0.0136626
cg15846744	0.9671825	0.3017807	3.09974	0.9552202
cg11666555	1.6585636	0.7770401	3.540143	0.1909172
cg03645984	0.2528639	0.0687082	0.930605	0.0386261
cg21917349	5.6265441	1.6034786	19.74332	0.0069929
cg02860255	9.8389308	0.1618794	598.0042	0.2752571
cg01148073	1.0969418	0.4401288	2.73393	0.8425897
cg15872672	3.31E-10	9.09E-18	0.012035	0.0139889
cg17102674	0.3840541	0.1860199	0.792913	0.0096723
cg08457158	0.4530638	0.1462778	1.403267	0.1698799
cg14617020	0.4389039	0.1843307	1.04506	0.0628301
cg07979271	0.7324157	0.0810795	6.616131	0.7815386
cg16266358	0.1111904	0.021183	0.583642	0.0094183
cg08078213	119997.09	5.28E-07	2.73E+16	0.3807012
cg20628733	34.124884	1.1649284	999.6389	0.0405056
cg23320454	1.5325947	0.8365791	2.80768	0.1668841
cg04737759	0.4682874	0.2249876	0.97469	0.0425083
cg23794932	92.880215	0.0243375	354462.2	0.281527
cg09163369	2.8678431	0.9093433	9.044466	0.0722087
cg14951038	13726.914	0.0792853	2.38E+09	0.1216002
cg04099095	7.8276027	1.5697347	39.03294	0.0120733
cg05437132	0.417238	0.1718755	1.01287	0.053396
cg14858504	0.369661	0.1393256	0.980791	0.0456166
cg17763743	0.5487776	0.1594428	1.888808	0.3413348
cg13242000	1.9373123	0.8421777	4.456516	0.1197433
cg06028605	0.1318263	0.0257433	0.675055	0.0150358
cg02764006	3.7211167	0.3673842	37.69	0.2659995
cg11753499	0.1386765	0.0412865	0.465799	0.0013942
cg07470489	0.0908953	0.0054854	1.506176	0.0941216
cg13485469	0.3573811	0.1401951	0.911025	0.0311509
cg08295857	0.1533118	0.0354523	0.66299	0.0120702
cg13595415	6.9011412	1.0330116	46.10379	0.0462088
cg13992008	0.8577606	0.2783061	2.643684	0.7893449
cg00336376	0.1656712	0.0251833	1.089889	0.061427
cg26995744	0.0051924	1.07E-05	2.529722	0.0957075
cg21346589	1.8772117	0.8021846	4.392909	0.1465459
cg09103598	0.7540898	0.3878889	1.466016	0.4053406
cg26490758	0.4720233	0.2208203	1.008992	0.0527615
cg21597811	0.5522296	0.2608412	1.169131	0.1207488
cg07556134	1.5453667	0.7852093	3.041429	0.2076731
cg19701540	0.235944	0.0561545	0.991365	0.0486319

cg08274876	2.3603624	0.5541488	10.05382	0.2454179
cg06834637	0.0439572	0.0040106	0.481777	0.0105345
cg26917140	3.4512265	1.4843315	8.024464	0.0040094
cg25184248	1.8904231	0.6765422	5.282301	0.2245073
cg09220088	2.7404739	0.8996741	8.347687	0.0760744
cg16578267	0.0006919	9.82E-09	48.7642	0.2014267
cg24785726	0.5152526	0.2563246	1.035739	0.0626888
cg16229875	5.6390283	0.5086238	62.51897	0.1587787
cg13445854	0.2273597	0.0786206	0.657492	0.0062586
cg10755035	0.1177603	0.0212716	0.651926	0.0142872
cg11940285	3.4821116	0.8613475	14.0769	0.0800246
cg14546712	5130986.2	9.45E-23	2.78E+35	0.6471699
cg05396178	0.4401795	0.0683297	2.835634	0.3879417
cg10217449	2006.0688	0.6628312	6071399	0.0629699
cg23795217	1.0940289	0.5955051	2.009889	0.7721247
cg11496438	9.23E-07	1.41E-14	60.31781	0.1301642
cg21645630	0.159984	0.0304256	0.841228	0.0304548
cg09337897	1.257781	0.4604019	3.436157	0.6546747
cg04937596	0.2496201	0.1046299	0.59553	0.0017584
cg27321311	2.4354195	0.3295962	17.99556	0.3830459
cg16628135	0.4966025	0.0841891	2.929285	0.4395072
cg10625686	0.4022682	0.1934197	0.836625	0.0147926
cg15678744	0.3552062	0.1572213	0.802509	0.012809
cg26927544	5.2699753	1.1816563	23.50315	0.0293481
cg27273054	4.5858597	0.2519141	83.48128	0.3036111
cg03726819	0.0001426	8.53E-12	2384.441	0.2967005
cg16016431	19.526817	0.0766786	4972.659	0.2930811
cg17341170	2.7674055	1.1496347	6.661711	0.0231421
cg14259096	2.4128584	0.9264468	6.284101	0.0713047
cg02763101	0.1206505	0.0151269	0.962295	0.0459075
cg09040942	0.3883829	0.1713609	0.880255	0.0234831
cg15992420	4.052159	0.9612219	17.08242	0.0566388
cg01217815	0.6783819	0.2890993	1.591848	0.3725617
cg11771735	0.0014855	5.01E-09	440.49	0.3110749
cg11161142	0.0021459	0.0001131	0.040707	4.28E-05
cg03302951	1.4362971	0.4377604	4.712508	0.5503308
cg01577114	0.7023791	0.1951557	2.527912	0.5887366
cg12359158	2.5315236	0.7088772	9.04051	0.152668
cg18495710	0.0655234	0.0124649	0.344433	0.0012873
cg01904812	0.2085849	0.0676396	0.643227	0.0063733
cg19795817	2.617174	0.9826158	6.970781	0.0542447
cg26285698	2.0405002	0.8253514	5.044689	0.1225084
cg17747551	6.2593523	1.6541945	23.68494	0.0069079
cg14223293	1.7544208	0.8210644	3.748783	0.1467658
cg24126941	1.1882449	0.467977	3.017084	0.7167644
cg04839207	2.0968675	0.7367295	5.96807	0.1653043
cg05406635	1.9870476	0.5435981	7.263377	0.2991412
cg06490988	1.7050717	0.7953869	3.655164	0.1702045
cg03712270	0.2273841	0.0700441	0.738156	0.0136897
cg02728483	2.43306	0.9664105	6.125535	0.0591016
cg04228709	0.249799	0.119679	0.521391	0.0002202
cg04436554	1.5239558	0.7802046	2.976708	0.217438
cg14255256	0.4926865	0.2061127	1.177705	0.1113654
cg16453794	3.6212049	0.8781955	14.9319	0.0750319
cg15011734	0.4187303	0.200313	0.875305	0.0206688
cg27408345	0.3823743	0.1842758	0.793431	0.0098445
cg17181022	1.6914521	0.6433648	4.446949	0.2865621
cg09804711	2.2979236	0.7092831	7.444775	0.1653707

cg00356916	1.6368677	0.5464442	4.90322	0.3786692
cg17347389	1.3245806	0.5648581	3.106114	0.5180004
cg25124276	0.026942	0.0030059	0.241482	0.0012385
cg24132141	0.8868843	0.4614034	1.70472	0.7188053
cg15325704	0.227264	0.0807766	0.639404	0.0049955
cg11246071	19.947461	2.9713516	133.9125	0.0020635
cg16018995	0.7210412	0.3482072	1.493078	0.3785073
cg04478430	0.0421688	0.004743	0.374909	0.0045115
cg21481662	1.14E+21	0.0637991	2.04E+43	0.0636397
cg21768604	2.2141944	0.7633653	6.422425	0.143469
cg15164702	0.3787663	0.1554226	0.923057	0.0326689
cg08999896	1.7373981	0.6899761	4.374865	0.2410514
cg20145598	0.7103288	0.3257074	1.549142	0.3899343
cg14543104	0.0465013	0.0081446	0.265498	0.0005566
cg16168406	0.256533	0.0558931	1.177411	0.0801355
cg14779797	1.7652399	0.5816635	5.357172	0.3157127
cg08746900	1.3627718	0.7258467	2.558594	0.3355316
cg12104698	2.1336395	0.5836816	7.799488	0.2518463
cg00751770	2.060395	0.6601662	6.430544	0.2131826
cg11617938	3.7053599	1.3392392	10.25186	0.0116515
cg00309313	0.7085866	0.3304462	1.519445	0.3761063
cg21181192	2.4040163	0.6080815	9.504143	0.2110524
cg20893031	1.0222418	0.5654223	1.848138	0.9419591
cg01611017	0.2287557	0.0716994	0.729841	0.0127028
cg23121335	0.0884416	0.015244	0.513114	0.0068549
cg18632102	246628.26	9.24E-09	6.58E+18	0.431205
cg18854441	0.0008498	2.61E-08	27.71903	0.182387
cg06051576	2.49E-05	3.11E-20	2E+10	0.544979
cg02072834	0.1630102	0.0195063	1.362244	0.0940165
cg18180181	0.0138972	5.73E-05	3.367645	0.1268843
cg21402096	0.3259966	0.136885	0.776373	0.0113515
cg25073708	1.4035508	0.5705444	3.452763	0.4604382
cg02300652	2.4613167	0.9814432	6.172624	0.0548536
cg05353869	1.8314191	0.953449	3.517856	0.0692427
cg19630062	0.0083225	4.92E-07	140.8475	0.3350511
cg26436158	7.06E-31	1.27E-53	3.92E-08	0.0093698
cg17434154	8.1542665	1.8734764	35.49127	0.0051646
cg19959519	0.0022135	2.12E-05	0.23073	0.0099219
cg24047802	2.4041425	0.6744172	8.570216	0.176189
cg07623882	130894912	5.6976004	3.01E+15	0.0306822
cg14465207	1.0589077	0.5052445	2.219293	0.8794943
cg11615509	0.7744917	0.3491793	1.717849	0.5295211
cg03855291	1.0016778	0.376244	2.666776	0.9973227
cg13009087	0.407174	0.1970572	0.841333	0.0152434
cg24659419	0.1298852	0.0345115	0.488827	0.0025409
cg19918549	2.0477376	1.030095	4.070721	0.0408996
cg07937631	1.113629	0.4196336	2.955363	0.8288888
cg02407720	2.1464371	0.8660496	5.319779	0.0990643
cg07399299	463990785	47.773036	4.51E+15	0.0150581
cg14314529	31.503871	0.3330665	2979.867	0.1371919
cg03810769	0.2782125	0.1107275	0.699033	0.0064951
cg15846316	0.4194858	0.1742626	1.009788	0.052595
cg17639959	1.4336645	0.6078183	3.381593	0.4106285
cg18512156	1.3926636	0.572075	3.390311	0.4656016
cg01544580	1.6646432	0.5178796	5.350736	0.3923137
cg07990582	2.676018	1.1067173	6.470553	0.0288851
cg15007228	0.4499304	0.1608506	1.258542	0.128059
cg15703632	0.0839002	0.0005113	13.76819	0.3409602

cg02737399	1.9257428	0.8729286	4.248326	0.1045224
cg09381022	0.2782628	0.1230024	0.629501	0.0021324
cg11057897	0.348763	0.0499107	2.437067	0.288269
cg21964481	3.0065632	0.5644156	16.01554	0.1971211
cg10467989	0.9584612	0.4472949	2.053786	0.9131154
cg06101576	0.5678908	0.0841737	3.831361	0.5612959
cg07081887	130.43054	4.56E-07	3.73E+10	0.6239395
cg23942984	1.0101565	0.3374092	3.024269	0.9855895
cg24256745	0.2125677	0.0601461	0.751255	0.0162174
cg24663971	0.2739615	0.0874979	0.857791	0.0261906
cg04452040	1.472074	0.8162615	2.654789	0.198728
cg20616108	3.8941283	1.3002657	11.66241	0.0151352
cg00277334	0.3038987	0.0802884	1.150283	0.0794638
cg06832272	0.6684927	0.2476361	1.804593	0.4267019
cg07015368	1.9898308	0.3008959	13.15879	0.4753
cg11152959	11.771619	1.0712188	129.3583	0.043777
cg01439566	0.0548306	0.0043006	0.699057	0.0253758
cg17852032	1.8937788	0.5199075	6.898147	0.3329403
cg16487097	1.8942476	0.7501219	4.783455	0.1764948
cg13988338	1.973548	0.936975	4.156879	0.0736656
cg27379715	0.2795791	0.0944607	0.827481	0.0213345
cg13634678	8.574E+10	3.73E-17	1.97E+38	0.4335261
cg08203788	2.513254	0.8679431	7.277488	0.0893421
cg15332386	0.2899366	0.1224607	0.686451	0.0048698
cg03609427	1.0966697	0.3757966	3.20036	0.8658957
cg22297966	0.2934976	0.0555442	1.550852	0.1489285
cg03923285	1.3960103	0.6491139	3.002316	0.3931655
cg13194341	3.13E-20	1.50E-38	0.06555	0.0369272
cg06765552	1.5665776	0.7795059	3.14836	0.2074888
cg19190163	3.2573291	1.254659	8.456635	0.0152651
cg16102040	0.2691177	0.0854196	0.847866	0.0249725
cg10651007	0.0648626	0.0103342	0.407109	0.0035128
cg13759905	1.3391512	0.602934	2.974332	0.4731995
cg20336981	2.5075277	1.1396302	5.517312	0.0223237
cg13520893	4.7864894	0.4475299	51.19319	0.1953198
cg13842648	1.6350434	0.6897243	3.875994	0.2642248
cg16330893	0.5402139	0.1183218	2.46642	0.4267388
cg23095729	27.275321	0.9922981	749.7174	0.0505369
cg26874693	1.8708481	0.9524057	3.674981	0.0690026
cg04498349	2.6215748	1.0819708	6.351978	0.0328064
cg25635500	1.3429106	0.4830022	3.733749	0.5719936
cg26646659	0.0843471	0.0180262	0.394671	0.0016848
cg07800320	1.704779	0.812957	3.574939	0.1579855
cg15667299	1.91745	0.6928421	5.306569	0.2100489
cg14251267	0.4780511	0.2204254	1.036781	0.0616903
cg07875786	0.0799427	0.0130071	0.491336	0.0063913
cg20954129	0.3592684	0.1078222	1.197099	0.095513
cg25550629	0.6690354	0.1597962	2.801121	0.5822334
cg11463427	4.7075233	0.9242469	23.97712	0.0621644
cg20926024	0.1362453	0.0187286	0.991144	0.0489818
cg18717423	0.1678008	0.0461513	0.610104	0.0067239
cg12068908	1.0883317	0.4952905	2.391457	0.8330935
cg07540421	2.3255379	0.8544328	6.329493	0.0985308
cg12563269	1.3522519	0.411184	4.447122	0.6193138
cg17075913	1.0506878	0.3586478	3.078076	0.9281592
cg23138772	0.1388754	0.0353691	0.545289	0.0046695
cg22249789	4.4468236	1.5204447	13.00556	0.0064265
cg18465834	0.3858028	0.13304	1.11879	0.0795459

cg13647706	2238.2258	2.14E-25	2.34E+31	0.8147317
cg24759237	24.851569	0.2127784	2902.553	0.1858936
cg20769926	0.1806607	0.0386804	0.843793	0.0295591
cg15346952	1.8652264	0.8839404	3.935864	0.1018039
cg24876969	3.8796121	0.2853698	52.74346	0.3085846
cg19489885	1.7080041	0.6634787	4.396943	0.2671721
cg06403617	2.1878859	0.8127391	5.889768	0.1212408
cg08512167	0.1017349	0.0271962	0.380567	0.0006858
cg14628889	0.0005377	3.34E-08	8.646304	0.1276496
cg10239098	0.0458924	0.0003365	6.258063	0.2191782
cg09442489	1.6561055	0.7502672	3.655612	0.2117625
cg13871695	1.7058529	0.7076926	4.111862	0.2341474
cg13482308	2.7611747	1.0778775	7.07324	0.034326
cg01154355	2.3591997	0.7438539	7.482415	0.1449817
cg13543254	2.7775291	0.4247887	18.16119	0.2862867
cg11488033	0.3456031	0.1497257	0.797736	0.012794
cg09654261	0.784927	0.3200329	1.925148	0.5967805
cg04440551	0.3077943	0.1155917	0.819586	0.0183675
cg12144100	0.0231996	0.0029726	0.181061	0.0003306
cg00907661	0.0002708	5.30E-07	0.138241	0.0098246
cg24700472	0.973038	0.0031131	304.1381	0.9925599
cg10508416	1.6508777	0.6696561	4.069846	0.2761826
cg27270590	0.003017	7.35E-05	0.123882	0.0022004
cg10271819	5.7772377	0.7523829	44.36103	0.0917175
cg12847240	6.5858837	1.022249	42.42984	0.0473546
cg14911766	0.3836685	0.1073816	1.370826	0.1403499
cg21936906	0.3076427	0.0791868	1.195199	0.0886721
cg22227306	1.1898288	0.4515088	3.135471	0.7251611
cg26393977	0.1829309	0.0741366	0.451379	0.0002277
cg03129384	0.3565871	0.1552775	0.818884	0.0150561
cg07776847	1.6164744	0.7933224	3.29373	0.1860261
cg04877287	0.9266349	0.4534769	1.893486	0.8344635
cg11985424	2.6283934	1.1365958	6.078196	0.0238643
cg02173128	4.291564	0.7275287	25.31518	0.1076895
cg20680265	4.9059068	1.6641998	14.46216	0.0039344
cg04105091	0.0187133	0.0008108	0.431916	0.0129859
cg09464061	1.7273824	0.5588227	5.339528	0.3424605
cg00345344	0.2260823	0.0799047	0.639677	0.0050798
cg02551029	0.3370512	0.1358836	0.836036	0.0189588
cg04990420	2.1096749	0.8015931	5.552354	0.1305246
cg24335310	0.7736097	0.3489133	1.715245	0.5274913
cg14007090	0.6361813	0.1204015	3.361476	0.5943759
cg03279443	4.6E+12	1.22E-14	1.73E+39	0.3503726
cg11080643	2.5196213	1.1376521	5.580345	0.0227351
cg10738368	0.1483533	0.0479374	0.459113	0.0009312
cg01002317	0.0087662	0.0002433	0.315791	0.0095896
cg20681184	0.575622	0.3005539	1.102434	0.0957474
cg01767396	0.406002	0.1100423	1.497947	0.175964
cg05564831	0.1389272	0.0411415	0.469131	0.001478
cg01956865	0.5456611	0.1170733	2.543245	0.4404988
cg26525463	0.236509	0.0571839	0.978187	0.0465456
cg26646427	8.7348117	1.4208584	53.69778	0.0193323
cg10860860	6.6937544	1.0488488	42.71955	0.0443892
cg13163930	2.3098868	1.10495	4.828795	0.026066
cg13001274	0.0004406	1.68E-06	0.115481	0.0065339
cg04043150	0.4101949	0.1724671	0.975606	0.0438176
cg13800079	0.02709	0.0029051	0.252616	0.0015361
cg21887345	1.7603656	0.8948811	3.462903	0.1013742

cg01886303	2.1571159	0.9800013	4.748105	0.0561616
cg03627989	0.0991002	0.0074036	1.326492	0.0807243
cg16066219	3.4453843	1.3501302	8.792243	0.009653
cg06809298	0.3171621	0.0100796	9.979716	0.5140218
cg05501584	1.3439781	0.7223349	2.500609	0.3507104
cg00203347	0.2984857	0.1135753	0.784446	0.0141901
cg18985828	0.1139059	0.0078789	1.646747	0.1109425
cg20374928	0.317151	0.082144	1.224493	0.095688
cg18947175	0.4891561	0.2099373	1.139739	0.0975416
cg17037101	0.2433522	0.061989	0.955336	0.0428212
cg22778903	1.8378824	0.7547316	4.475514	0.1801533
cg09150807	2.2111364	0.9220683	5.302345	0.0753795
cg26932690	1.80E-06	2.23E-09	0.001449	0.0001069
cg01715177	2.1221578	0.5018305	8.974252	0.306422
cg01894875	0.2214269	0.0709088	0.691449	0.009458
cg03217880	2.1761084	0.7519769	6.297331	0.1515197
cg26555664	0.2769328	0.0998497	0.768072	0.0136271
cg04774506	3.0342682	0.2346549	39.23543	0.3953613
cg18084554	0.179167	0.0443913	0.723134	0.0157215
cg02383626	0.0788604	0.0179339	0.346772	0.000775
cg23019576	0.3142878	0.0239068	4.131741	0.3785344
cg17949403	7.0636604	0.1151792	433.197	0.3519229
cg13952688	4.0745718	0.8909019	18.6352	0.0701357
cg16329509	35.237408	0.7462875	1663.802	0.0701149
cg11386011	0.2625975	0.0756214	0.911877	0.0352735
cg26708319	1.7130243	0.8702359	3.372019	0.1192986
cg19177744	4.4792356	1.0009747	20.04401	0.0498512
cg27230999	0.5502279	0.2279411	1.328197	0.1839408
cg14080585	0.1069732	0.0321594	0.355829	0.0002674
cg08310008	0.2776748	0.0655491	1.176267	0.081937
cg08923100	1.25E-05	9.03E-12	17.19354	0.1174389
cg25323841	5.7879054	0.9390664	35.67357	0.0584631
cg10550882	1.2036888	0.6554421	2.210518	0.549979
cg14340070	5.01E-05	2.10E-08	0.119613	0.0125929
cg10634728	1.2376602	0.4948887	3.095247	0.6484538
cg15996480	1.753564	0.9219546	3.335291	0.0868528
cg26712159	11.892975	0.8590254	164.655	0.0648007
cg04261408	1.724366	0.2927355	10.15742	0.5470424
cg26190686	2.2428135	0.8569581	5.869847	0.0998683
cg15482000	0.8386785	0.1965055	3.579451	0.8121796
cg00414077	2.4787606	0.1301848	47.1964	0.5459671
cg21610556	2.4539178	0.8518896	7.068654	0.0963116
cg23262020	4.076E+09	1.35E-30	1.24E+49	0.6333051
cg11823178	2.2837482	0.9503308	5.488095	0.0648808
cg17741993	4.2644199	1.142007	15.92396	0.0309667
cg22057874	3.651952	0.9754926	13.67181	0.0544645
cg08162124	3.7094528	1.3556737	10.14996	0.010696
cg01678292	1.5847204	0.6186973	4.059075	0.3373455
cg13538299	0.3136298	0.0808882	1.216044	0.0935307
cg07598385	2.8120278	0.7181288	11.01126	0.1376651
cg08680731	1.97E-09	3.36E-23	115686.5	0.2152697
cg13040418	0.5371481	0.2026238	1.423959	0.211514
cg04899656	1.8086802	0.4793165	6.824977	0.3817883
cg13335271	0.88414	0.470384	1.661841	0.7021294
cg04809274	1.8604234	0.7260618	4.767053	0.1959601
cg11999571	0.420328	0.170319	1.037322	0.0600449
cg05894754	0.2926789	0.1174376	0.729417	0.0083605
cg25449283	0.3170355	0.0884639	1.136187	0.0777468

cg26929894	0.0005473	1.36E-06	0.220932	0.0141618
cg05876496	0.0039473	1.77E-07	87.97921	0.2785847
cg04425201	1074.9034	8.8174534	131037.5	0.004397
cg20445402	0.8333521	0.3949109	1.758563	0.6323346
cg21983151	0.0274771	0.0013334	0.566206	0.0198895
cg08774231	0.3871059	0.0680083	2.203423	0.284797
cg08622757	3.0415737	1.1706458	7.902622	0.022408
cg00199000	3.22E-07	8.33E-11	0.001248	0.0003906
cg19906454	0.2374534	0.0756776	0.745057	0.0137246
cg26495591	23882.291	3.1867521	1.79E+08	0.0267893
cg16405637	0.1046074	0.0151602	0.721804	0.0219771
cg04902265	50.979916	0.2303166	11284.26	0.1535774
cg12236164	1.5541725	0.8212091	2.941336	0.1754923
cg23762915	0.4133557	0.1816117	0.940815	0.0352603
cg07710211	1.0430165	0.331939	3.27736	0.942523
cg20219911	1.3095798	0.3423521	5.009459	0.6935724
cg16519487	5.6349058	1.2714999	24.97221	0.0228352
cg16195245	0.2883147	0.095508	0.87035	0.0273631
cg04662836	0.6119956	0.3038283	1.232731	0.169335
cg13219896	14.510159	0.7306578	288.1577	0.0794019
cg08209473	0.6781698	0.0170702	26.94248	0.8362262
cg04838249	0.3821545	0.1660327	0.879598	0.023723
cg24915511	0.0132765	0.0013867	0.127107	0.0001771
cg14896948	2.4202426	0.9394025	6.235425	0.0671749
cg07755196	1.9611158	0.6980173	5.509857	0.2012984
cg21299542	1.9582158	0.9329107	4.110371	0.0756675
cg07498879	1.5867673	0.5120484	4.917172	0.4236672
cg12125614	0.3179987	0.1638145	0.617303	0.0007109
cg24747537	0.316371	0.086223	1.160834	0.0827215
cg05495029	3.46E-15	1.61E-28	0.074187	0.0334996
cg13655570	0.272724	0.076959	0.966467	0.0441355
cg04774132	0.8570102	0.4657863	1.576831	0.6198821
cg13941830	1.9502698	1.0000901	3.80321	0.0499691
cg24453819	1.0743755	0.4275052	2.700044	0.8787293
cg03190219	1.5961027	0.42482	5.996761	0.488728
cg25927444	0.38882	0.1815681	0.832641	0.0150416
cg05914582	1.0755081	0.2540314	4.553444	0.921245
cg06999762	3.2298809	1.1492566	9.077286	0.0261599
cg20451455	2.9953619	1.2755881	7.03377	0.011775
cg06298346	2.3891507	1.1227123	5.084153	0.0237985
cg15452017	0.588545	0.3201485	1.081952	0.0879313
cg11218954	2.1281695	0.9748809	4.645804	0.0579473
cg11990419	4.4598512	1.3392767	14.8515	0.0148541
cg22130834	1.9615695	0.9211181	4.177265	0.0806516
cg24575067	2.7170903	0.3858852	19.13155	0.315497
cg19056700	0.5652871	0.2374358	1.345836	0.1974462
cg15852446	0.1079883	0.029932	0.3896	0.0006742
cg10472064	1.0686243	0.3923102	2.910854	0.8967107
cg03272310	0.932245	0.350325	2.480785	0.8882665
cg09446910	0.0136765	0.0001727	1.083333	0.0543443
cg16970628	0.748658	0.2732174	2.051439	0.5735398
cg04705318	0.5210634	0.2117791	1.282029	0.1558655
cg06974930	2.0791273	0.819001	5.278101	0.1235865
cg18562989	1.7566141	0.2955396	10.44088	0.5355646
cg11306119	0.8851306	0.1922469	4.075261	0.8755426
cg08653945	1.1735231	0.3285558	4.191546	0.8054134
cg13537276	1206.5069	5.52E-05	2.64E+10	0.4105797
cg01973676	1.4958478	0.7324569	3.05487	0.2690106

cg03866607	4.1542589	1.2770493	13.51386	0.0179666
cg03311232	1.9522708	0.7601331	5.014071	0.1645037
cg00478874	0.0352987	0.000981	1.270191	0.0673789
cg12082681	4.45E-05	5.01E-08	0.039473	0.0038139
cg26100137	2.2105822	0.3088472	15.8223	0.4295566
cg22964598	202584224	0.0011487	3.57E+19	0.1477201
cg10154597	0.1094735	0.0234426	0.511226	0.0049043
cg18833994	1.16031	0.3352782	4.015528	0.814413
cg06155414	0.2541507	0.1169467	0.552325	0.0005424
cg16054907	0.4207487	0.1980364	0.893924	0.024347
cg20942097	5.0762268	1.4447547	17.83561	0.011282
cg19098906	0.9914413	0.4422134	2.222809	0.9833522
cg24033558	0.4103606	0.1890268	0.890857	0.0243104
cg07551199	1754.8255	0.3213256	9583465	0.08887
cg00172803	4.7379179	0.8495187	26.42422	0.0760659
cg14719129	0.4203349	0.2009059	0.879424	0.0213859
cg26112639	0.8723321	0.2919418	2.606558	0.8067953
cg01923816	0.0497531	0.0031406	0.788185	0.0332685
cg03586839	221.07917	3.9229586	12458.96	0.008679
cg23513095	5.0484081	0.6716957	37.94341	0.1156559
cg00414041	1.7720747	0.8491332	3.698182	0.1274396
cg14506032	0.2902534	0.0989129	0.851729	0.0243124
cg21619653	1.2285831	0.6345132	2.378857	0.5414425
cg25198654	1.5716363	0.6808253	3.628009	0.2894858
cg20280672	0.4230656	0.0103612	17.27451	0.6494561
cg07603357	12.386133	1.0186041	150.6143	0.0483333
cg26352401	9.11E-06	5.98E-11	1.387656	0.0566296
cg12890691	7.11E-05	5.72E-09	0.885442	0.0471162
cg13112336	1.73E-21	9.09E-35	3.30E-08	0.0021832
cg04931582	1350.992	9.8303044	185668.6	0.0041068
cg17389813	0.6001915	0.2614909	1.3776	0.2284818
cg25075015	1.0004778	0.48994	2.043017	0.9989537
cg20866121	0.2105602	0.0587975	0.754038	0.0166788
cg01199327	1.0028804	0.1463188	6.873819	0.9976632
cg19949550	4.1697953	0.7214246	24.10119	0.1106729
cg00333020	1.9427662	0.5129996	7.357396	0.3283198
cg16048421	0.945555	0.4058746	2.202834	0.8967718
cg07574686	1.65215	0.6050491	4.511369	0.3272724
cg01247127	0.2453803	0.0941154	0.639762	0.0040594
cg14595003	2.2535198	1.023767	4.960457	0.0435587
cg08830565	3.5991595	1.0079895	12.85127	0.0485847
cg15694146	1.073158	0.42682	2.698253	0.880692
cg04781638	0.4129861	0.1422981	1.198593	0.1037919
cg02524112	0.487513	0.2131861	1.114842	0.088687
cg26161024	0.1895423	0.0534777	0.671799	0.0099913
cg26666292	0.2140948	0.0876525	0.522935	0.0007175
cg00695214	0.0718732	0.0003702	13.95503	0.3273694
cg16585619	3.1855778	1.1408606	8.894957	0.0270013
cg14548509	1.1425765	0.5672809	2.301295	0.7090785
cg01250845	1.4240133	0.2976996	6.811612	0.6580222
cg25730428	2.0034271	0.8955995	4.481601	0.0907327
cg22746426	2.3443545	0.6449512	8.521571	0.1956963
cg11415739	1.6027447	0.3060548	8.393239	0.5765706
cg25403803	0.1806498	0.0003167	103.0552	0.5971768
cg23840338	0.6439036	0.2733595	1.516728	0.3139181
cg01844539	1.6498641	0.8008716	3.398861	0.17453
cg16395366	6.2844495	1.5497601	25.48414	0.0100733
cg02480675	0.2818077	0.1127533	0.70433	0.0067298

cg25566507	0.3538471	0.1589621	0.787658	0.0109402
cg22555392	85468.27	4.36E-25	1.67E+34	0.7414046
cg01772980	0.5363458	0.1969646	1.4605	0.2228929
cg23730781	0.5366039	0.2729389	1.054975	0.0711057
cg00629688	0.9642218	0.5386571	1.726003	0.902388
cg19368016	2.361918	1.0227327	5.454658	0.0441567
cg04348265	1.9315106	0.7992642	4.66771	0.1436699
cg27376392	2.4111392	0.6434125	9.03556	0.1916433
cg11210374	0.727152	0.3753653	1.408628	0.3449559
cg17673237	0.0604593	0.0053854	0.678749	0.0229644
cg27311341	1.4894583	0.7213781	3.075344	0.2814529
cg15169943	0.0354037	0.0034525	0.363045	0.0049064
cg26470937	4.2356129	0.1192011	150.5055	0.428124
cg21166347	0.0009362	4.43E-11	19770.64	0.4177004
cg19781814	0.887622	0.4434147	1.776831	0.7363837
cg02675353	2.14E-05	6.56E-09	0.069644	0.0091733
cg24049888	5.1457256	1.7502421	15.12847	0.0029081
cg05370101	0.4407251	0.1972803	0.984582	0.0457327
cg24418941	0.4056958	0.1295933	1.270043	0.1212844
cg09111484	0.7557263	0.3725084	1.533179	0.4377646
cg02587643	2.29E-05	1.81E-10	2.912027	0.0747986
cg01418261	3.3475669	0.7269605	15.41515	0.1209745
cg04021014	1.9840007	0.8323617	4.729025	0.1221212
cg13042250	0.2605095	0.1218437	0.556986	0.0005217
cg15963463	0.3642689	0.127476	1.040916	0.0594154
cg00457258	26.373328	0.0008791	791224.9	0.5338458
cg21793517	0.0571276	0.00478	0.68276	0.0237314
cg22193247	0.0026146	1.64E-05	0.416912	0.0215586
cg10334247	2.2050758	0.6123026	7.941105	0.2264277
cg04923779	1.7920305	0.6711266	4.785048	0.2443725
cg05960024	0.0002693	1.08E-07	0.670562	0.0393864
cg05244682	0.884338	0.4166342	1.877075	0.7488989
cg16558208	1.3776783	0.289755	6.550353	0.6871149
cg04699227	0.0070236	0.0003611	0.136599	0.0010579
cg14158573	0.0871191	0.0021593	3.514872	0.195786
cg05216056	0.4585624	0.0436674	4.815481	0.5157934
cg21870299	0.5787074	0.2144365	1.561778	0.2802263
cg15339435	0.3186821	0.0972185	1.044639	0.0590437
cg25024920	3.4457346	1.063827	11.16073	0.0390986
cg23072086	1.893488	0.7178114	4.994761	0.197043
cg15989608	2.1494828	0.436018	10.59653	0.3471414
cg27054948	0.0904631	0.0048572	1.684818	0.107321
cg04006061	1.9911759	0.8687042	4.564018	0.1036561
cg22509679	0.5533361	0.185177	1.653449	0.28933
cg00456299	0.9266412	0.2416292	3.553644	0.9115424
cg25230305	0.4098652	0.0388191	4.327501	0.4582642
cg21516044	0.5290229	0.2586626	1.08197	0.0811321
cg26272417	0.0660725	0.0093753	0.465649	0.0063886
cg04487857	1.0833177	0.3972205	2.954473	0.8757667
cg27641522	2.2657527	0.8485834	6.049653	0.1026171
cg11420633	1.1739595	0.5030407	2.7397	0.7106961
cg16918529	0.5149254	0.187525	1.413935	0.1977902
cg16039157	2.1412189	0.8330505	5.50365	0.1139394
cg09864050	2.0535882	1.0532607	4.00397	0.0346621
cg23304605	1.6953668	0.8438413	3.406172	0.1380786
cg18372607	1.3819483	0.6844209	2.790361	0.3668883
cg25107254	0.201489	0.0563861	0.719997	0.0136805
cg23363263	0.5580248	0.258576	1.204256	0.137177

cg14479139	0.294494	0.1047284	0.82811	0.0204758
cg11455690	3.55E-39	2.26E-89	5.58E+11	0.1332773
cg14121020	1.3770444	0.1780851	10.64801	0.7591702
cg10198479	0.3911433	0.1665839	0.918415	0.0311318
cg05762769	0.1474983	0.0268171	0.811265	0.0277761
cg23832862	2.01E-58	3.07E-103	1.32E-13	0.0116284
cg14163484	0.2122702	0.052181	0.863508	0.0303913
cg01942816	0.2408237	0.0895214	0.647846	0.0048062
cg06070263	1.664633	0.6068895	4.56591	0.3222304
cg15361590	2.2857955	0.9208016	5.674253	0.0747329
cg17933765	3.09E+30	6.37E-09	1.50E+69	0.1224099
cg16964569	0.0027502	2.66E-06	2.838511	0.0958533
cg14309307	0.0312177	0.0049169	0.198202	0.0002367
cg21261487	0.3110001	0.1416405	0.682863	0.0036077
cg13958215	0.8212777	0.3673046	1.836343	0.6315248
cg13801475	1.4822765	0.4620808	4.75489	0.5080937
cg12391945	0.516409	0.2478954	1.075769	0.0775787
cg14798020	0.7510212	0.2362525	2.387415	0.6275161
cg05374238	0.6418303	0.288388	1.428444	0.277318
cg20306180	28.151771	2.6236987	302.063	0.0058397
cg25520440	2.8573277	0.9188552	8.885319	0.069714
cg17195706	1.3653134	0.246296	7.568456	0.7215721
cg10591475	60.843935	0.024527	150935.1	0.3029282
cg23878577	0.6781853	0.3241223	1.419018	0.3025819
cg00232827	5.1980657	0.7483242	36.1072	0.0955561
cg06788362	0.1167713	0.0136798	0.996767	0.049655
cg20744362	1.2565241	0.6259071	2.522503	0.5207382
cg24505341	0.3364121	0.132435	0.854556	0.0219977
cg11648730	2.5764207	0.9497299	6.989296	0.0630741
cg23889391	0.9630823	0.4891204	1.896317	0.9133477
cg04357789	0.3742495	0.1516721	0.923457	0.032944
cg19482086	866.38428	13.454656	55788.99	0.0014568
cg18805774	0.3441085	0.1164425	1.016902	0.0536506
cg15864354	3.40E-28	1.12E-67	1.03E+12	0.1727073
cg03707974	3.3048667	0.9624228	11.34859	0.0575494
cg23374863	0.417637	0.1216243	1.434094	0.1653876
cg13641317	1.9627707	0.9089463	4.23839	0.0859973
cg19954341	1.5689401	0.6055424	4.065071	0.353798
cg01873236	3.16E-08	4.64E-14	0.021537	0.0117348
cg10798745	2.8794256	0.843441	9.830079	0.091377
cg05130485	0.8244551	0.4553798	1.492658	0.5238839
cg14232082	0.3796378	0.1571413	0.917167	0.0313903
cg09836921	0.4153312	0.181118	0.952418	0.0379776
cg05189570	0.7262053	0.3192353	1.651992	0.4455188
cg00723907	0.227397	0.1030095	0.501987	0.0002466
cg13682961	2.6862437	0.7436512	9.703347	0.1315623
cg14115936	1.1605735	0.6076538	2.216609	0.6519442
cg17652716	0.1824522	0.0380288	0.875359	0.0334743
cg20426415	0.2992668	0.0794674	1.127011	0.0745493
cg05348746	0.0007401	7.33E-28	7.47E+20	0.7982398
cg25734842	1.6383763	0.4526436	5.930221	0.4519081
cg20187173	1.2189479	0.3221214	4.612652	0.7706017
cg12332902	2.6256581	0.917178	7.516622	0.0720407
cg15605235	4.9871151	1.130671	21.99695	0.0338243
cg00115178	1.2498373	0.5131422	3.044173	0.6234246
cg09624551	0.3956011	0.157072	0.99636	0.0491024
cg11540416	2657.6856	0.3158344	22363912	0.0872622
cg15527230	0.208187	0.0691083	0.627158	0.0052841

cg10476206	0.3335526	0.1287496	0.864137	0.0237829
cg07171687	0.3648106	0.1834912	0.725304	0.0040282
cg05633168	1.329021	0.5697766	3.099981	0.5103851
cg15454374	1.7267638	0.795602	3.747745	0.1670869
cg14832908	0.2014588	0.0288617	1.40621	0.1060723
cg20705392	1.4430941	0.4812964	4.326898	0.5126648
cg11751213	0.7795064	0.3050488	1.991912	0.6027978
cg09200774	0.2144923	0.0685098	0.671538	0.0081988
cg02524941	0.0235517	1.82E-06	304.7634	0.4377616
cg12378878	0.2294749	0.0157731	3.338522	0.2812575
cg04569641	0.39715	0.1600629	0.985413	0.0464088
cg23900905	0.3969623	0.1856308	0.848884	0.017199
cg25006249	0.2717552	0.1126063	0.655833	0.0037501
cg15988887	0.4009723	0.1386874	1.159289	0.091585
cg10419507	0.3781999	0.1455236	0.9829	0.0460036
cg27541317	0.0327103	0.0025112	0.426072	0.0090177
cg00531789	0.8352546	0.1171479	5.955293	0.8574498
cg01030081	0.5023811	0.2499206	1.009867	0.0533101
cg20817257	1.6711706	0.6464409	4.320289	0.2892867
cg03369807	1.603308	0.568394	4.522561	0.3722763
cg00241941	0.1367942	0.0167884	1.114619	0.0630866
cg25900902	0.1913835	0.0893123	0.410108	2.12E-05
cg16782524	2.9531796	0.8125396	10.73335	0.1000366
cg07492924	0.1971626	0.0609183	0.638118	0.0067358
cg21570843	0.43934	0.1692029	1.140758	0.0911324
cg26275470	1.1999565	0.5989044	2.404216	0.6071773
cg20255272	1.1644105	0.3372345	4.020502	0.8097486
cg14204124	0.1817563	0.0406298	0.813081	0.0257029
cg18787420	2.0022219	0.9569252	4.189348	0.065318
cg17265073	8087.541	35.150765	1860794	0.0011835
cg17655451	0.79641	0.4009372	1.581966	0.5156288
cg09372664	8.7068793	0.1828087	414.6944	0.2722565
cg11571790	11.829406	0.230446	607.2349	0.2188757
cg13053608	1.2342703	0.4486069	3.395898	0.683563
cg12495731	0.28851	0.0993196	0.838083	0.0223353
cg10861751	0.6530016	0.3846463	1.10858	0.1145117
cg06238570	154.98318	0.8736783	27492.71	0.0562813
cg20327163	1.3346159	0.4890206	3.642382	0.5731079
cg14564826	6.9500611	0.3047519	158.5006	0.224297
cg27057022	0.0534341	0.0086588	0.329746	0.0016061
cg09080087	1.3176825	0.4832243	3.593129	0.5898821
cg16538359	0.8411317	0.1361359	5.19703	0.852288
cg07925670	0.16537	0.0506601	0.539818	0.0028697
cg25020550	2.570327	0.9834907	6.717482	0.0541043
cg00739155	3.6456554	0.7830253	16.97366	0.0992924
cg23262036	1.1814126	0.6261706	2.229002	0.6067699
cg27209278	6.9455418	0.8425392	57.25615	0.0717392
cg06825478	1.0885779	0.1020406	11.61304	0.943979
cg07181395	1.582112	0.5181817	4.830504	0.4204985
cg24089932	0.9972852	0.0222628	44.67435	0.9988819
cg15426459	0.0253769	0.0021505	0.299453	0.0035285
cg11577329	4.6255777	1.0224548	20.92608	0.0467234
cg13453304	6.3309094	3.26E-07	1.23E+08	0.8293681
cg12348202	0.1293796	0.0405256	0.413049	0.0005547
cg10187932	0.4088587	0.0365009	4.579767	0.4681121
cg25515997	0.459774	0.2068111	1.02215	0.0566221
cg15394626	9.88E-08	8.43E-14	0.115818	0.0236783
cg27436118	0.3455006	0.1755512	0.679976	0.0020946

cg00622988	0.4508646	0.1939127	1.048301	0.0642572
cg04026887	0.3303975	0.163229	0.668769	0.0020824
cg15705203	0.3580568	0.0626707	2.045688	0.2480725
cg18211014	0.6547576	0.3152677	1.35982	0.256078
cg13966575	0.4194701	0.1600818	1.099157	0.0771273
cg01386425	1.2499331	0.5689965	2.745769	0.578478
cg13106577	10.398724	0.0082381	13126.1	0.5203912
cg06140764	15927.085	1.89E-35	1.34E+43	0.8324291
cg03578013	0.604321	0.1877087	1.945588	0.3985172
cg12800200	59.243238	0.0312426	112339.1	0.2891803
cg08767044	1.8584151	0.9808656	3.521081	0.0573401
cg00059930	7.6442669	1.1232889	52.02118	0.0376369
cg08460995	15196.254	136.43354	1692591	6.22E-05
cg26333652	0.4317596	0.1923494	0.969155	0.0417593
cg06770429	0.7683977	0.3348321	1.763376	0.534206
cg10208461	9.66E-09	4.48E-21	20861.69	0.2028057
cg11712482	0.4411804	0.1160836	1.676724	0.229654
cg25629041	2.3356545	0.8087782	6.74509	0.1169422
cg04640972	1.8205733	0.846374	3.916102	0.125237
cg21612617	0.5320852	0.2626977	1.07772	0.079754
cg01638311	0.4513741	0.2140695	0.95174	0.036625
cg06445972	1.7836335	0.8266091	3.848673	0.1402995
cg15944684	0.0694842	8.41E-05	57.40509	0.4364911
cg26296653	1.7808705	0.0514842	61.60142	0.7495774
cg12371933	3.2463898	1.213858	8.682273	0.0189715
cg16603374	0.5384366	0.2464862	1.176188	0.1204454
cg19422030	1.4164973	0.7794148	2.574322	0.2533117
cg16768966	0.4996412	0.1826017	1.367136	0.1766774
cg26649232	3.3089064	0.1742193	62.84527	0.4256654
cg00220113	1.5288223	0.5046249	4.631753	0.4528896
cg27175380	0.8613105	0.1830576	4.05258	0.8501295
cg15575683	0.0549656	0.0099004	0.305162	0.0009096
cg22696167	1.8606572	0.7151635	4.840914	0.2030957
cg05523057	0.4851653	0.2445877	0.962376	0.0384799
cg13832679	2.42E-09	1.09E-23	535911.3	0.2391122
cg06573254	3.8036975	1.0360275	13.96499	0.0440829
cg19802138	0.1560055	0.0555375	0.438221	0.0004225
cg18198734	0.142108	0.0428565	0.471216	0.0014216
cg17738194	0.4463358	0.1455742	1.368482	0.1581902
cg06617136	16.849796	3.16E-08	8.99E+09	0.7829555
cg22598458	0.3545544	0.1626971	0.772656	0.0090829
cg11083457	7.4894369	0.00778	7209.723	0.5656554
cg16789104	1.1234912	0.5128438	2.461242	0.7710408
cg01801101	1.8672424	0.6887752	5.062021	0.2197346
cg20491328	1.9025892	0.8649732	4.184922	0.1097552
cg22632840	0.1177977	0.0269282	0.515308	0.0045048
cg18343292	0.9395372	0.4390666	2.01047	0.8723417
cg16022904	1.3838069	0.3828369	5.001925	0.6202684
cg01903374	1.7931075	0.5998671	5.359911	0.2959178
cg23301140	2.32E+20	1.02E-36	5.24E+76	0.4787718
cg19851487	1.5375585	0.674535	3.504764	0.3061412
cg10309230	0.1923566	0.0748626	0.494253	0.000618
cg25324047	1.9510791	0.8342695	4.562926	0.1230875
cg03837680	0.2950527	0.1315267	0.661889	0.0030663
cg21625570	0.3783202	0.1347939	1.061815	0.0648848
cg15030789	4.1197349	1.4089831	12.04572	0.0097012
cg00114383	791.84321	1.2485632	502189.8	0.0426219
cg09367545	0.1438477	0.0387815	0.533557	0.0037405

cg04689061	1.3430275	0.7672043	2.351033	0.3019058
cg10126324	0.1863012	0.0479682	0.723566	0.0152092
cg11424776	0.4668892	0.1707244	1.276827	0.1378439
cg03538480	2.7121075	0.6548626	11.23217	0.1687924
cg08903465	0.8806141	0.2807394	2.762281	0.8274526
cg25411327	163.10315	0.2082701	127731.4	0.1340093
cg06815976	0.2113508	0.0609972	0.732316	0.014233
cg12608433	2.6301536	0.7532403	9.183932	0.1295716
cg21109038	0.3699246	0.1656511	0.826099	0.0152656
cg09231412	7.23E-20	3.28E-38	0.159519	0.0408409
cg10249734	0.4902848	0.233675	1.02869	0.0594094
cg20687875	0.3841042	0.1720837	0.85735	0.0195092
cg05825855	0.2831492	0.1240318	0.646395	0.002735
cg02374294	0.4385635	0.1347419	1.427455	0.1710298
cg09071889	2.1687962	0.967944	4.859452	0.0599978
cg04397137	0.3027031	0.1394698	0.656982	0.0025068
cg27512176	0.2715877	0.1289323	0.572083	0.0006053
cg15686157	0.1915821	0.0548284	0.669428	0.0096344
cg08188893	0.2296106	0.0504805	1.044384	0.0569395
cg27257408	0.3495203	0.1579027	0.773669	0.0095161
cg07279214	1.741E+10	7.44E-26	4.08E+45	0.5703866
cg07682547	0.9426237	0.4995481	1.778687	0.8552772
cg11598715	0.0123097	9.59E-15	1.58E+10	0.757226
cg00909275	5.03E-08	7.83E-16	3.236163	0.0669608
cg24004327	1.9460128	0.520741	7.272264	0.3222454
cg04907171	0.925487	0.3164082	2.70703	0.8875485
cg10316922	0.3330982	0.121105	0.916183	0.0332097
cg00213142	0.3902794	0.1513778	1.006211	0.0515167
cg24355907	1.7375588	0.5929731	5.09148	0.3138319
cg11801637	0.2935166	0.0252464	3.412453	0.3274126
cg26224652	0.5257631	0.0013787	200.4959	0.8321063
cg07225641	10.156576	1.7084598	60.37955	0.0108073
cg08614441	2.0130365	0.7259148	5.582357	0.1788083
cg27315314	0.2091401	0.0596555	0.733203	0.0144915
cg10420380	1.9594901	0.4312155	8.904137	0.383794
cg07892449	0.2194015	0.0666896	0.721807	0.0125424
cg02101307	0.4217613	0.1711535	1.039316	0.0606335
cg15813266	0.5950774	0.2940213	1.204393	0.1490306
cg27425459	0.249538	0.0863133	0.721432	0.0103839
cg01078434	2.2672858	0.7145563	7.194094	0.1646886
cg23337866	7.0712999	1.892259	26.42518	0.0036354
cg18718102	1.08E-07	1.57E-12	0.007375	0.0047401
cg25843174	7.5560043	1.2488136	45.71795	0.0276735
cg19456451	0.3049505	0.1443029	0.644442	0.0018653
cg07419021	2.1460662	0.8897804	5.176109	0.0891329
cg04489803	55.236999	2.2706754	1343.709	0.0137557
cg19674051	0.5024705	0.2420304	1.043161	0.0648069
cg10052704	0.3943822	0.1099346	1.414817	0.1534176
cg19255477	2.1674413	0.7610932	6.17244	0.1474234
cg06584218	0.5195366	0.2135778	1.263793	0.1488034
cg16553297	0.6527885	0.3297078	1.292456	0.2210187
cg18995042	0.6151954	0.2983	1.268741	0.1883565
cg13590966	2.3163574	0.844876	6.35065	0.1025981
cg08263066	2.48E+27	7074644.6	8.66E+47	0.0089633
cg02538976	1.558425	0.6593704	3.683345	0.3120269
cg02337825	1.229203	0.5279382	2.861964	0.6322359
cg05228717	0.0040041	0.0001045	0.153409	0.0029997
cg09419900	0.1616021	0.0399807	0.653197	0.0105406

cg11516377	0.9615318	0.4094025	2.258275	0.9282498
cg17212000	0.2692915	0.0240864	3.010744	0.2868144
cg05115375	0.0251622	0.0003213	1.970285	0.0978958
cg27239243	2.5251636	0.8422449	7.570781	0.0982293
cg19477361	3.5418783	0.6199454	20.23549	0.1549517
cg01951879	5.5361918	1.0673312	28.71594	0.0415952
cg16265599	1.2267672	0.5518195	2.727265	0.616085
cg16502826	3.804418	0.3525586	41.05302	0.2709184
cg24580526	8.32E-41	5.54E-71	1.25E-10	0.0092352
cg03314977	0.3579537	0.156986	0.816193	0.0145689
cg12302377	6.35E-26	4.99E-45	8.09E-07	0.009739
cg21563737	0.1497698	0.0441695	0.507839	0.0023069
cg13464995	2.3397641	1.0193758	5.37044	0.0449381
cg01309945	1.4204311	0.3472796	5.809799	0.6253085
cg25742458	7.1138882	0.6461439	78.32219	0.1089072
cg20414935	0.427876	0.1350665	1.355465	0.149025
cg01899253	2.0942862	1.1006148	3.985077	0.0243203
cg11493509	0.637988	0.3206222	1.269496	0.2004602
cg10104290	1.7026907	0.6836633	4.240619	0.2529817
cg22855923	0.009964	0.0006081	0.163269	0.001237
cg00877691	0.0978371	0.0117637	0.813699	0.0314983
cg24738650	0.8187246	3.12E-07	2145618	0.9788388
cg08115957	25.596583	1.2168928	538.4082	0.0369543
cg24810735	0.9797379	0.5672485	1.692179	0.9414751
cg03568957	68175.081	0.5178475	8.98E+09	0.0642348
cg26870746	4.3587258	0.9869228	19.25023	0.0520652
cg04500239	0.508518	0.2409021	1.073426	0.0760495
cg17001717	0.5418167	0.2508116	1.170462	0.1188926
cg08209133	0.4244677	0.1284008	1.403206	0.1601203
cg09004241	0.9131513	0.2621969	3.180225	0.8865219
cg18045152	1.6472038	0.5692549	4.766372	0.3572425
cg10660916	0.1008115	0.0261232	0.38904	0.000868
cg11786657	1.326246	0.5915322	2.973513	0.4930804
cg00027570	2.4960602	0.6607503	9.429154	0.1773709
cg24301288	2.1488013	0.6750726	6.839779	0.1953838
cg01516466	2.9973885	1.61E-06	5574066	0.8815216
cg26856607	0.0407078	0.0051144	0.324008	0.0024879
cg25553198	0.0033746	7.57E-06	1.50431	0.0674356
cg25982880	4.8321644	1.3506555	17.28776	0.0154291
cg16102102	1.3131099	0.6843474	2.519565	0.4126477
cg05352754	0.1071179	0.0241997	0.474148	0.0032488
cg25559262	1.7823855	0.4192183	7.578148	0.4338228
cg23417677	2.7046507	0.4823469	15.16571	0.2580078
cg21100712	21.43706	0.14193	3237.847	0.2311878
cg15579696	0.4766742	0.2131383	1.06606	0.071201
cg14860438	2.7626089	0.1467592	52.00362	0.4974167
cg07814763	1.8872564	0.4243299	8.393791	0.404211
cg10215339	1.177171	0.621109	2.23106	0.617057
cg18210160	2.1458286	0.5458572	8.435503	0.2743138
cg22957613	0.6798594	0.3193157	1.447498	0.3169366
cg16366073	1.3203387	0.5730253	3.042264	0.5140784
cg19598514	1.6762165	0.7416487	3.788454	0.2143958
cg15971496	0.0253153	5.27E-10	1216114	0.6837303
cg02390801	2.8772423	0.9546699	8.671608	0.0604429
cg19910201	1.0134981	0.2070957	4.95992	0.9867968
cg21790587	1.0287906	0.4571215	2.31538	0.9453238
cg16032408	2.7621147	0.5517513	13.82739	0.2163322
cg17287155	5.5496754	1.2029741	25.60229	0.0280307

cg06380123	3.595708	1.1946459	10.82255	0.0228274
cg09754549	0.9938879	0.4044093	2.442608	0.989338
cg11104918	186.38021	0.0021634	16057196	0.3672411
cg10492716	34.559829	4.00E-05	29883792	0.6114985
cg09410512	2.1768728	0.8044134	5.89097	0.1256511
cg21107581	1.7480565	0.6522981	4.684517	0.2667991
cg13821077	0.2243938	0.0709601	0.70959	0.0109589
cg10718697	1.3119762	0.3766234	4.570299	0.6697978
cg04304019	852.7673	0.1607899	4522746	0.1230062
cg13635431	2.3978398	0.6925312	8.302349	0.1675362
cg10435235	13.970164	1.5401892	126.7153	0.0190849
cg05438353	2.99E+20	0.9968801	8.95E+40	0.0500152
cg06162185	0.7409454	0.3843502	1.428385	0.3706249
cg23231268	2.8483346	0.7013295	11.56804	0.143243
cg05357919	1.8589971	0.5983407	5.775756	0.2837213
cg07141036	1.9991994	0.7492328	5.334521	0.1665368
cg06533314	1.0074397	0.341773	2.969617	0.9892777
cg00083659	1.2863733	0.6325007	2.616212	0.4868871
cg03194311	1.13E-22	6.82E-43	0.018565	0.0333534
cg23191941	0.1111292	0.0197254	0.62608	0.0127434
cg19911880	0.5839971	0.0411499	8.28805	0.6910692
cg11823034	2.6453287	0.3211581	21.78915	0.3658812
cg08664487	1.1190833	0.5147762	2.4328	0.7764302
cg10072995	0.0002914	1.09E-16	7.79E+08	0.5770982
cg00789416	0.6741839	0.3224833	1.409449	0.294719
cg27201457	2.8675107	1.0593976	7.761597	0.0381222
cg15824100	0.3148837	0.127083	0.780213	0.0125579
cg01152056	1.0607993	0.435006	2.58685	0.8967457
cg27307781	0.0053141	0.0001114	0.253599	0.0079157
cg22859585	0.449154	0.1797555	1.122298	0.0867078
cg13906701	1.0988376	0.5057398	2.387481	0.8118334
cg09380198	41.90883	0.2633678	6668.812	0.1486951
cg27491025	0.2625239	0.0596709	1.154983	0.0768364
cg17409673	1.6974767	0.839312	3.433082	0.1408873
cg23240477	1.4256517	0.3735947	5.440343	0.6037562
cg25154424	1.0487551	0.4071693	2.701302	0.9214446
cg07135408	9.5308577	1.1645195	78.00406	0.0355563
cg15978561	0.5062371	0.2322069	1.103654	0.0869078
cg08421768	0.6903694	0.2759468	1.72718	0.4283966
cg12350309	0.5228156	0.235934	1.158528	0.1101557
cg07871456	0.3557878	0.0745345	1.698341	0.195036
cg08246494	0.3120932	0.027525	3.538677	0.3472656
cg25261377	0.0698237	0.0081052	0.601506	0.0154096
cg01940810	1.5098184	0.6809437	3.347636	0.3105399
cg11784243	0.6054146	0.2883986	1.270904	0.1847192
cg11903057	2.7810128	1.3342186	5.796676	0.0063444
cg10433463	1.9471425	0.6516022	5.818526	0.2328374
cg08388111	0.0046614	1.17E-05	1.863374	0.0790299
cg10454286	0.2750621	0.1321543	0.572506	0.000558
cg26245202	0.8460307	0.4196242	1.705735	0.640248
cg11006442	0.0785153	0.0101628	0.606587	0.0147203
cg25313204	2.7671263	1.0503781	7.289745	0.0394547
cg27249581	5.039E+10	8.92E-14	2.85E+34	0.377164
cg05349952	0.0174388	5.37E-06	56.60006	0.326315
cg12081372	0.584226	0.2924356	1.167163	0.1279634
cg03842258	0.0396204	0.0029181	0.537938	0.0152728
cg12281565	1.4484693	0.6949702	3.018925	0.3227526
cg12572352	7.9535075	1.7334474	36.49276	0.0076378

cg06338958	1.0286332	0.0523861	20.19786	0.9851728
cg19628619	0.2746672	0.0188624	3.999599	0.3443581
cg08901888	13428360	2.38E-07	7.57E+20	0.3096396
cg03370738	0.0936736	0.0090222	0.972574	0.047339
cg04661436	0.3061606	0.1480647	0.633063	0.0014059
cg18712973	10.279692	0.2241621	471.4092	0.2325459
cg23434090	0.7383548	0.3633795	1.500271	0.401718
cg05076755	0.5289335	0.2236979	1.250663	0.1469077
cg23327829	1.9458866	0.6913304	5.477084	0.2073679
cg13385220	2.1167244	0.9665536	4.635565	0.0608055
cg10377144	0.2084042	0.0779197	0.557398	0.0017818
cg03770410	0.4174435	0.1562395	1.115333	0.0814604
cg18862566	2.1265309	0.9441051	4.789862	0.0685868
cg12793826	0.3694382	0.152357	0.895821	0.0275666
cg24041338	1.0360738	0.5503499	1.950485	0.9125753
cg14063008	0.4609568	0.1827221	1.162865	0.1009284
cg00989002	0.3578683	0.1564409	0.818646	0.0149362
cg23228341	8.0393636	1.1835467	54.60821	0.0329772
cg22207584	0.1488671	0.0477772	0.46385	0.0010207
cg00202403	2.91E-16	5.03E-30	0.016781	0.0269121
cg06585708	2.1279294	0.8057032	5.620039	0.1275156
cg01784406	0.007755	3.42E-09	17560.32	0.5151207
cg04464062	5.4936124	0.3529411	85.50938	0.2238464
cg24132481	0.3781758	0.0960594	1.488839	0.1643039
cg20107201	0.0310533	0.0009184	1.049947	0.0532569
cg05918292	0.504069	0.223191	1.138422	0.0993383
cg24748868	1.8473958	0.7023551	4.859182	0.2135312
cg22258713	0.0960473	0.0182305	0.506024	0.0057206
cg25167574	0.4071654	0.1726883	0.960016	0.0400526
cg22680204	0.4584478	0.1946391	1.079816	0.074378
cg22239325	0.3901563	0.1936497	0.786068	0.0084518
cg02067585	11.541584	1.7593894	75.71272	0.010814
cg01267908	8.0470199	1.1512125	56.24898	0.035562
cg00236601	1.4393927	0.4683353	4.423863	0.524913
cg21599100	0.3381785	0.1175321	0.973051	0.0443649
cg23991636	1.7178446	0.782251	3.772434	0.177628
cg23204908	0.4485224	0.1967832	1.022305	0.0564579
cg20633321	0.2833209	0.1060316	0.757045	0.011903
cg09971653	1.1139784	0.3254538	3.812977	0.8634933
cg22675956	0.0706291	0.008721	0.572007	0.013014
cg20381985	0.0097663	0.0002377	0.40121	0.0146176
cg09872934	7.496E+10	3.36E-08	1.67E+29	0.2453783
cg17657322	1.5758155	0.8264454	3.004669	0.1672557
cg00921219	1.6991607	0.7153279	4.036117	0.2297509
cg24247537	1.7132811	0.3444044	8.522922	0.5106971
cg21579294	2.7400328	1.0409371	7.21252	0.0412297
cg14069287	0.295135	0.1356053	0.64234	0.0021013
cg00020175	5.6485541	0.5492167	58.09395	0.1453882
cg09321747	0.0575966	0.0094715	0.350248	0.0019415
cg22390041	0.6774431	0.286747	1.600467	0.3746459
cg16436711	1.4476493	0.6446131	3.25108	0.3701433
cg20435097	0.2442783	0.0814968	0.732199	0.0118529
cg08032665	3.6460656	0.4522649	29.39382	0.2244328
cg15889106	0.3526937	0.0222208	5.598027	0.4600015
cg26708280	1.669E+12	0.0015682	1.78E+27	0.1109
cg17954152	0.1392888	0.0057726	3.360951	0.2248907
cg07458516	0.1220618	0.0063182	2.358119	0.1638804
cg17135423	0.1311907	0.0396959	0.433571	0.000868

cg02642178	6.6142528	0.0013384	32687.71	0.6633139
cg27481559	2.2442773	1.045707	4.816627	0.0380175
cg16266227	1.381322	0.4099301	4.654575	0.6022332
cg10718614	0.1830245	0.0458112	0.731217	0.016264
cg02883595	1.8473918	0.5823525	5.860465	0.2973975
cg02459543	1.8946841	0.2995859	11.98263	0.4970804
cg08529295	0.6532478	0.3132332	1.362348	0.2561954
cg00084338	0.395012	0.1929556	0.808655	0.0110546
cg00985983	0.3125243	0.1105667	0.883371	0.0282439
cg24667575	47.664716	0.4702336	4831.482	0.1010511
cg01268752	4.6030977	0.9489905	22.32742	0.0580952
cg06615667	0.0013527	7.98E-16	2.29E+09	0.6456698
cg22475859	0.4524481	0.1728078	1.184607	0.1063138
cg25279586	0.5069309	0.229825	1.118151	0.092323
cg22239201	0.3642764	0.1118002	1.186915	0.0938106
cg00697129	1.190118	0.6455307	2.194134	0.5770805
cg09324702	0.8240346	0.3698681	1.835879	0.6358281
cg14684716	0.3316985	0.1277936	0.86095	0.0233525
cg04685570	1.446865	0.7369461	2.840667	0.2831909
cg04698020	1.5006252	0.6792809	3.315088	0.3155362
cg03782727	2.6639505	0.5464514	12.98676	0.2254063
cg20798460	0.782434	0.3571197	1.71428	0.5398181
cg12391352	1.4165909	0.7483902	2.681395	0.2847506
cg03543081	0.8633117	0.4294718	1.735404	0.6799112
cg25503888	2.5301537	0.9213823	6.947906	0.0716878
cg26475330	0.3121924	0.1463818	0.665821	0.0025911
cg16150661	0.0930603	0.018646	0.464455	0.0037923
cg05501761	3.1657545	0.8967337	11.17612	0.0733569
cg06724305	1.4699477	0.5551964	3.891859	0.4380702
cg21167135	2.6291853	0.8790339	7.863878	0.083753
cg04276005	0.0678716	0.00962	0.478853	0.006962
cg05261028	0.7573811	0.19462	2.947417	0.688547
cg23526071	24.438018	0.0831248	7184.577	0.2703817
cg04225775	1.0736288	0.4211965	2.736677	0.8817012
cg20574490	3.0750487	1.3200291	7.163421	0.0092285
cg26689643	0.0073964	2.00E-10	273672.7	0.5810398
cg23197007	0.5631436	0.0074227	42.72462	0.7948793
cg12800915	0.668233	0.2087482	2.13911	0.4970968
cg07666840	0.0906035	0.011237	0.730535	0.0241465
cg12097672	16.163432	0.5456252	478.8204	0.1074953
cg05417409	0.3860819	0.1640426	0.908662	0.0293098
cg14887886	7.0347594	0.1286246	384.7463	0.3393277
cg15945769	9.96E-09	1.79E-18	55.6001	0.1076066
cg07168142	1.4719106	0.2883757	7.512841	0.6420756
cg00068412	1.2396462	0.3473564	4.424052	0.7406782
cg02600514	0.2738735	0.0413293	1.814854	0.1795138
cg11692409	0.4717061	0.1785898	1.245909	0.1294469
cg22935317	0.312258	0.0942946	1.034047	0.0567591
cg04783966	2.7552775	0.7991043	9.500079	0.108526
cg04675221	12.003189	1.3490952	106.7949	0.0258493
cg15128555	1.0696453	0.0051084	223.9729	0.9803007
cg24059022	2.3783591	0.5083203	11.12801	0.2711136
cg05529278	1.8595299	0.4724767	7.318565	0.3748661
cg02728342	0.6360954	0.0019943	202.8893	0.8777628
cg10426581	0.0593807	0.004123	0.855225	0.0379979
cg09730170	1.4523088	0.5268295	4.003574	0.4707567
cg24951754	1.1413283	0.4728905	2.754613	0.7687108
cg15127879	0.7738257	0.4073707	1.469929	0.4334799

cg05245515	0.2887786	0.1178674	0.707516	0.006593
cg17791651	0.30456	0.1294647	0.716464	0.0064519
cg06960099	13.483406	0.5458612	333.0558	0.1118436
cg15577595	1.1392103	0.5949632	2.181312	0.6941338
cg14437986	1.3452081	0.5341085	3.388047	0.5291971
cg20367218	2.2173422	0.714199	6.884085	0.1683131
cg11225357	2.2189644	0.6923022	7.112216	0.179862
cg16970604	1.2057661	0.3887821	3.739555	0.7459252
cg14226212	0.4123206	0.1653073	1.028438	0.057455
cg27273602	0.0394438	0.0002467	6.305572	0.2117725
cg16818627	7.26E-05	4.37E-08	0.120539	0.0117609
cg25257900	1.6192191	0.2330456	11.25046	0.6260535
cg07411238	3.7889919	0.7614837	18.85327	0.1037099
cg16098170	1.780601	0.1572911	20.15714	0.6412144
cg10044209	0.6285961	0.260342	1.517746	0.301942
cg09973105	3.046551	1.1094429	8.365886	0.0306586
cg16956686	2.0717024	0.7663905	5.600214	0.1511246
cg06902099	1.7046903	0.6243811	4.654159	0.2979425
cg21573476	0.0921519	0.0108355	0.783714	0.0290278
cg03583812	0.0010278	1.88E-21	5.62E+14	0.7412662
cg18634560	0.9190148	0.4193122	2.014223	0.8329308
cg22020784	1.7212591	0.7410604	3.997964	0.2065877
cg20883195	0.4844351	0.1910772	1.228181	0.1267745
cg19539972	212.70866	3.299364	13713.24	0.011684
cg25533997	0.2043657	0.0610412	0.684216	0.01001
cg18024479	1.7916832	0.8629644	3.719886	0.117688
cg16136466	0.7128773	0.3346131	1.518751	0.3804602
cg25010400	0.95875	0.5066724	1.814193	0.896996
cg06983508	0.5980407	0.2295114	1.558322	0.29275
cg10710412	1.0928294	0.4925036	2.424908	0.8271993
cg13661131	0.6916252	0.2684968	1.781569	0.4450188
cg17965690	0.3024101	0.1057218	0.865023	0.0257227
cg02880444	0.0813875	0.0103598	0.639386	0.0170686
cg06486409	1.4233177	0.6320887	3.204982	0.3940318
cg26460483	0.1419861	0.0358955	0.561632	0.0053986
cg21686213	3.9745758	1.559161	10.13189	0.0038496
cg14626903	2.5184012	0.6775863	9.360202	0.1679276
cg07022728	0.0015586	4.38E-07	5.552005	0.1213458
cg21411674	0.4588645	0.1628278	1.293124	0.1405704
cg07593205	0.5802099	0.2744104	1.226789	0.1541781
cg15770238	0.1017851	0.0156053	0.663888	0.0169352
cg04446345	2.0763556	0.9510839	4.532988	0.0666444
cg11432962	1.4874038	0.7580166	2.91863	0.2483312
cg20121927	0.6771869	0.3524094	1.301277	0.2421121
cg14682846	3.11E-10	9.18E-20	1.056426	0.0505758
cg16365807	0.4599109	0.2214794	0.955023	0.0372146
cg03464765	8.1196787	0.4949563	133.202	0.142309
cg13803976	1.7004615	0.8457414	3.418976	0.1362744
cg22150661	0.2472103	0.0704214	0.867817	0.0291653
cg09769542	1.9000054	0.8743781	4.128672	0.1050288
cg14578009	2.3868398	0.9805741	5.809866	0.0552706
cg07428323	2.2580422	1.0036233	5.080347	0.0489869
cg03529662	0.2462003	0.0448191	1.352429	0.1068288
cg21580007	0.1594459	0.0623559	0.407708	0.0001266
cg03992114	2.6095154	1.1143252	6.110937	0.0271538
cg06588735	5.1564182	0.4735482	56.14771	0.1781793
cg23535596	0.2762492	0.1296187	0.588755	0.000862
cg16965605	1.3182005	0.6280196	2.766876	0.465213

cg21478105	0.5960071	0.3128936	1.135288	0.1154811
cg24311952	2.9541932	1.2017971	7.26184	0.0182486
cg18973457	4.7236039	1.0833967	20.59489	0.0387733
cg11952839	2.4992972	0.6278239	9.949423	0.1937526
cg16058797	1.4711334	0.5861647	3.692194	0.4109428
cg07524919	0.2921642	0.1023262	0.834194	0.0215254
cg12596520	0.2979065	0.1021846	0.86851	0.0265414
cg26180383	3.2076212	0.4739956	21.7066	0.232199
cg04554720	0.4846168	0.2303217	1.019676	0.0563103
cg13799941	4.8695554	1.3353493	17.75758	0.0164823
cg01466976	1.4574441	0.6183974	3.434916	0.3891447
cg00296578	0.2065203	0.0788915	0.540624	0.0013154
cg15741482	122016.45	0.4021584	3.7E+10	0.0689838
cg15827101	0.357932	0.1106044	1.15832	0.0864039
cg23703427	0.0788693	0.0074177	0.838581	0.0352109
cg17636707	0.5548198	0.2275348	1.35287	0.1951838
cg07701443	1.85E-06	2.22E-10	0.015376	0.0041519
cg05765011	0.2211151	0.0619427	0.789308	0.0201045
cg03702677	0.2908369	0.0892031	0.948241	0.04055
cg19936954	0.0053873	0.0003158	0.091914	0.0003073
cg00244642	2.5411114	0.7807528	8.270539	0.1214034
cg13569583	0.1838972	0.0638682	0.529499	0.0016991
cg17468563	2.6048417	0.5844931	11.60869	0.2092445
cg10665488	0.7653886	0.3059872	1.914524	0.5676132
cg25354551	1.4754671	0.7089525	3.070732	0.2982655
cg22735832	5.11E-14	3.37E-35	77511125	0.2187245
cg00268009	5.49E-57	1.44E-98	2.09E-15	0.0080033
cg04134096	0.5805845	0.2307407	1.460853	0.2481322
cg23370170	1.30E-07	1.26E-11	0.001348	0.0007763
cg09129100	1.3618634	0.7514417	2.468151	0.3086588
cg06027620	0.0081908	2.15E-05	3.117534	0.1129895
cg13609861	3.2581645	1.0410667	10.19688	0.0424479
cg18732541	3.3976333	1.2203469	9.459533	0.0192252
cg21108553	4.0119795	1.0314306	15.60549	0.045004
cg12288994	2.0112654	0.9990929	4.048861	0.0502979
cg23090529	1.1761865	0.6580458	2.102308	0.5839255
cg24969672	17256741	2.0273298	1.47E+14	0.0406808
cg12194929	1.4961754	0.6435746	3.478292	0.3492389
cg21659346	1.977511	0.6676473	5.857209	0.21842
cg18849517	1.2086027	0.4620997	3.161051	0.6993205
cg13196806	1.339831	0.5636359	3.184941	0.5078563
cg17465336	1.1153201	0.3201893	3.885011	0.8639037
cg02347652	0.2250684	0.0917775	0.551941	0.00112
cg04370247	1.433073	0.424755	4.835018	0.5619593
cg07571202	0.8018067	0.2692555	2.387673	0.6915547
cg00229809	1.6319008	0.5035634	5.28851	0.4142872
cg18974224	2.5087689	0.5906782	10.65541	0.2125869
cg03433048	1.7049964	0.7584765	3.8327	0.196684
cg19370684	1.3219228	0.7158972	2.440965	0.3724522
cg00493242	0.6053082	0.2965029	1.235732	0.167994
cg06174599	1.5258755	0.7475565	3.114542	0.2457392
cg21069942	1.4671259	0.5091617	4.227455	0.4777773
cg08525922	1.1141241	0.3404505	3.645971	0.8582051
cg10208897	2.0748377	0.747574	5.758562	0.1610979
cg26741530	0.5048978	0.2310607	1.103267	0.0866119
cg27574739	2.8528119	1.0269677	7.924822	0.0443242
cg16195332	3.140227	0.6465868	15.25089	0.1558491
cg06730721	1.27E-26	2.15E-50	0.007452	0.0327276

cg00689534	0.5407287	0.2757635	1.060284	0.0735208
cg10668933	0.622295	0.2798395	1.383833	0.2447159
cg20287434	0.5442521	0.1649013	1.796288	0.3180144
cg09233161	1.453E+12	1140.1358	1.85E+21	0.0088447
cg18057904	2.2509231	0.1458559	34.7374	0.5611653
cg18138426	0.5220824	0.2388786	1.14104	0.1032661
cg24301066	3.953694	0.8787192	17.78918	0.0732178
cg02232704	2.2973726	1.1878958	4.443084	0.0134506
cg13797468	0.3848219	0.1663694	0.890115	0.0256128
cg12317515	264.92463	0.2849835	246277.6	0.1096024
cg00533182	1.6850498	0.4144698	6.850662	0.4658968
cg19661705	1.0496528	0.4902461	2.247384	0.9007155
cg14321522	1.6448606	0.1347023	20.08552	0.6966922
cg13671374	0.4920714	0.1987865	1.218062	0.1251745
cg18052601	6.99E-05	1.89E-15	2592197	0.440949
cg15254881	1.148071	0.5515815	2.389614	0.7119824
cg01089639	2.0719669	0.8820917	4.866894	0.0945212
cg12802819	4.03E-05	8.92E-20	1.82E+10	0.5567558
cg24789467	4.2116574	0.8439121	21.01885	0.0795926
cg14053764	2.7563544	0.4216432	18.01877	0.2898552
cg06573398	0.5423842	0.2084526	1.411259	0.2098745
cg13594630	0.586097	0.0869915	3.948774	0.5830644
cg20685713	2.0373849	0.8392607	4.945945	0.115785
cg26968498	0.6670969	0.3201129	1.390192	0.2798814
cg24432193	2.4077072	0.8823991	6.569651	0.0862214
cg03615568	1.3313473	0.5704946	3.106928	0.5080335
cg03265671	3.5062074	1.3715247	8.963375	0.0088018
cg16634951	1.8501965	0.5584751	6.129596	0.3140439
cg09012411	1.3134851	0.3440071	5.015138	0.6899593
cg20005760	0.4447264	0.2083872	0.949106	0.0361694
cg22930549	1.2202789	0.3068905	4.852156	0.7774261
cg25032745	1.3336428	0.6404907	2.776938	0.4416587
cg17466857	1.6191202	0.6226609	4.210238	0.3229966
cg16961623	5.8847772	0.7629396	45.39101	0.0890598
cg08464402	0.5661623	0.2626164	1.220563	0.1466585
cg10241608	2.104484	0.5985559	7.39923	0.2460888
cg14428080	2.1696714	0.9760529	4.82297	0.0573685
cg21174344	3.3553092	0.601117	18.72863	0.1676405
cg04868219	0.1606244	0.0607446	0.424732	0.0002279
cg20081244	0.2004046	0.0581323	0.690873	0.010909
cg13647528	0.5157037	0.2089145	1.27301	0.1508912
cg03012642	2.6680146	0.3294883	21.60411	0.3577839
cg02304628	2.5869337	0.7159264	9.347645	0.1470262
cg25382214	1.7527022	0.8213745	3.74003	0.1467478
cg03221590	1.158108	0.5510992	2.433707	0.6984558
cg15106082	0.1758468	0.0633759	0.487916	0.0008433
cg19820248	11067874	3.67E-25	3.34E+38	0.6609747
cg03517284	4.8744452	1.4190963	16.7432	0.0118726
cg05253159	1.3004157	0.3922985	4.3107	0.6674797
cg18901104	2.9985096	0.8941205	10.05576	0.0752908
cg05409693	1.732789	0.7655039	3.922328	0.1872124
cg03299990	0.4598347	0.1221136	1.731567	0.250806
cg15396367	0.6200324	0.0588477	6.532797	0.6907514
cg14983362	1.934E+09	1.28E-32	2.92E+50	0.6584903
cg05317396	3.3542192	0.854525	13.16613	0.082806
cg05767421	1.8917922	0.6256967	5.719829	0.2587516
cg00339913	0.1154352	0.0350948	0.379694	0.0003793
cg17066275	0.3218433	0.1486874	0.69665	0.0040095

cg19067392	0.0726654	0.0016796	3.143769	0.1725512
cg23268208	2.1808198	0.8207995	5.79432	0.1178463
cg17306339	2.2905779	0.5602542	9.36494	0.248675
cg22384395	1.5434065	0.741938	3.210651	0.2455322
cg02506984	0.5560296	0.2828887	1.092899	0.0886965
cg26675654	0.4148233	0.137378	1.252591	0.1186326
cg19470459	3.5652308	0.9645128	13.17854	0.0566767
cg03115379	1.4727223	0.7789339	2.784461	0.233574
cg11503661	0.2141397	0.0605641	0.757145	0.0167701
cg21587066	0.9661103	0.4787097	1.94976	0.9233344
cg10493855	3.7849597	0.1575473	90.93091	0.4118669
cg03408354	0.6333223	0.2827459	1.418578	0.2669316
cg10641258	2.6533307	1.0605625	6.638141	0.0370115
cg24759919	1.2452874	0.5961276	2.601357	0.5594608
cg19764540	4.0478059	1.2177005	13.45547	0.0225282
cg24673886	1.5692324	0.6574153	3.745715	0.3100745
cg18366200	0.0608467	4.57E-06	809.6806	0.5634056
cg19244826	562.01683	5.0430526	62633.28	0.0084694
cg26419941	1.01E-10	3.81E-20	0.26608	0.0375647
cg23735602	0.8557137	0.4192615	1.746514	0.6686026
cg27588321	0.4773577	0.1669339	1.365033	0.1677478
cg23147149	127441.18	2.99E-08	5.43E+17	0.4282024
cg22120825	0.4577549	0.2165911	0.967443	0.0406919
cg25142327	3.9061144	0.9114632	16.73982	0.0664897
cg22888848	2.123731	0.9739948	4.630655	0.058263
cg20289913	1.6688761	0.867197	3.211666	0.1251878
cg12938917	147.26797	0.0662944	327144.4	0.2041706
cg02826735	0.1383123	0.0184144	1.03888	0.0544941
cg18689253	0.0265793	1.28E-05	55.23094	0.3519913
cg18763854	1.4766104	0.6090588	3.579914	0.3883669
cg13369206	9.501E+11	0.0028523	3.16E+26	0.1059824
cg03671556	0.8187459	0.3492212	1.919542	0.6455118
cg25691442	0.3832181	0.1634646	0.898397	0.0273534
cg01032851	0.4084334	0.2104044	0.792844	0.0081479
cg08877142	0.4425813	0.0001953	1003.155	0.8361779
cg16476975	1.9245449	0.8530753	4.341789	0.1147601
cg17861836	0.8722968	0.2903634	2.620515	0.807666
cg15578332	0.491383	0.1864995	1.29468	0.1505839
cg11387301	4.8214396	0.5371673	43.27568	0.1600393
cg20054852	0.3497187	0.1458383	0.838622	0.018555
cg19899694	0.2177392	0.0663022	0.715064	0.0119784
cg26818625	1.4128544	0.8040297	2.482691	0.2295131
cg25884399	0.1162047	0.030154	0.447819	0.0017651
cg18477949	2.1323853	0.658305	6.907235	0.206673
cg24341129	0.4944168	0.1382168	1.768584	0.2787357
cg00701514	2.4815412	1.0168118	6.056231	0.0458699
cg25769469	0.6765425	0.3485908	1.313029	0.248091
cg13140167	0.0248223	0.0017341	0.355313	0.006488
cg23917009	0.7105475	0.3596083	1.403966	0.3253801
cg20484832	0.7961154	0.3762592	1.684476	0.5509863
cg16646645	0.3937969	0.1813076	0.85532	0.0185293
cg22705959	2.4812337	0.7116623	8.650901	0.1538252
cg07492746	0.355548	0.1194687	1.058138	0.0631113
cg04585669	0.4023343	0.1845271	0.877231	0.0220609
cg11790922	0.0713962	0.0006511	7.82891	0.2707502
cg09171577	18127.983	23.290627	14109700	0.0038919
cg24849872	0.1756065	0.0475203	0.648936	0.0090974
cg04971534	0.295849	0.1265432	0.691674	0.004943

cg03555502	1.6279048	0.7888164	3.359558	0.1874266
cg12673103	2.3157774	1.1174455	4.799183	0.0239058
cg00743094	1.4166844	0.6924479	2.898405	0.340239
cg27020966	0.4365021	0.1892057	1.007021	0.0519485
cg11479035	3.4569733	0.0061644	1938.666	0.7009022
cg14618583	0.2167005	0.0423132	1.109798	0.0665118
cg27307257	1.4923684	0.7119432	3.128288	0.2890395
cg07180212	0.2705166	0.0931503	0.785604	0.0162355
cg24331049	213.73592	2.584416	17676.35	0.0172444
cg07542475	0.1510513	0.0300484	0.759323	0.0217824
cg01456691	2.4373311	0.6833889	8.692828	0.1696938
cg20977864	0.6868188	0.3058064	1.542545	0.3628022
cg16864999	0.3090578	0.1480252	0.645273	0.00177
cg08799816	0.9618531	0.4904827	1.886226	0.9098803
cg03910767	2.2890937	1.078183	4.859982	0.0310889
cg08498156	3.6614593	1.2405513	10.80671	0.0187574
cg10560317	1.99E-05	5.38E-09	0.073301	0.009779
cg17327115	0.3146236	0.0985059	1.004894	0.050971
cg23193613	0.4145291	0.1438616	1.194442	0.1029114
cg18218112	3.574263	0.8143169	15.68843	0.0914512
cg04939026	2.671727	0.8879137	8.03921	0.0803857
cg07148458	0.5087625	0.2498595	1.035939	0.0625126
cg06915112	0.4974272	0.2237839	1.105682	0.0866286
cg10591385	1.8779025	0.5758175	6.124367	0.2961142
cg03321784	0.2633369	0.0335946	2.064212	0.2040496
cg16634901	0.4415841	0.1740033	1.120648	0.0853889
cg24683185	0.3857663	0.1854934	0.802269	0.010782
cg12446151	0.2390413	0.0163489	3.495092	0.2957213
cg05265020	2.8360194	1.064355	7.556695	0.037097
cg02894139	0.3978945	0.1813405	0.873054	0.0215294
cg11723713	0.1724696	0.0296484	1.003284	0.0504281
cg02053964	3.2899863	1.083791	9.987174	0.0355541
cg13343639	0.9820381	0.4941869	1.951486	0.9587429
cg01518889	1.1516026	0.6641739	1.996749	0.6151893
cg13905199	0.4753416	0.2211312	1.02179	0.0568117
cg22911184	2.8735306	0.8973865	9.201362	0.0754647
cg07395004	3.65E-05	2.90E-16	4583212	0.4332195
cg23635130	0.2859887	0.0917854	0.891095	0.0308647
cg23989119	1.3896271	0.6881927	2.805992	0.3587687
cg04908960	0.4324486	0.1931834	0.968053	0.0414564
cg26238936	1.4308662	0.663377	3.086297	0.3609688
cg23903129	0.0315278	0.0031637	0.314191	0.0032095
cg25741837	3.0034707	0.30013	30.05643	0.3493603
cg26306893	0.2540142	0.081729	0.789477	0.017859
cg25874631	15.103154	1.2592407	181.1451	0.0322084
cg26833120	0.7475518	0.1969695	2.837159	0.6689745
cg10069809	2.9714152	0.9903605	8.915247	0.0520542
cg08189448	0.6665509	0.3135563	1.416939	0.2917759
cg24231804	0.1829074	0.0181099	1.847336	0.1499274
cg11042214	1.9956152	0.8228448	4.839892	0.1263654
cg22473616	13.896745	0.6985714	276.4492	0.0845543
cg09110241	3.0558783	0.6706407	13.92458	0.1488397
cg02898206	0.0453391	0.0002223	9.248232	0.2542255
cg17189985	1.5753141	0.656443	3.780396	0.3089039
cg10595421	0.3726341	0.1680659	0.826201	0.0151025
cg22735324	16.466076	0.0002194	1235877	0.6247822
cg05395523	0.2456335	0.0650996	0.926822	0.0382536
cg11823214	70924769	0.0003332	1.51E+19	0.174358

cg13929065	0.8914901	0.4261454	1.864984	0.7603675
cg00313297	0.7924689	0.322038	1.950102	0.6126643
cg06217504	0.6821797	0.2327617	1.999337	0.4857192
cg26805263	0.5355072	0.2849937	1.006225	0.0522972
cg01739167	2.5098872	0.7089188	8.886115	0.1536836
cg23865980	0.2884553	0.1101701	0.755255	0.0113559
cg16786102	1.6830431	0.7797089	3.632938	0.1848013
cg24719912	0.0870919	0.0079402	0.955265	0.0457799
cg00097384	3.8044338	1.1429347	12.66364	0.0294279
cg17848003	1.5564382	0.3119264	7.766256	0.5895839
cg13051202	1.21E-16	2.35E-37	62559.93	0.1320393
cg04960243	2.83E+27	7427.8025	1.08E+51	0.0225078
cg23695707	1.8843658	0.9433207	3.764186	0.0727036
cg19911493	1.0422771	0.4055438	2.678728	0.9314836
cg00766220	1.5694307	0.7608397	3.237361	0.222441
cg00081935	1.34E-08	9.09E-17	1.986698	0.0589668
cg16938613	2.8018748	1.1056491	7.100356	0.0298815
cg26673975	1.6635861	0.1082091	25.57566	0.7150702
cg08659654	0.9530325	0.3861042	2.352398	0.9168899
cg01379237	0.497183	0.2415844	1.023207	0.0577407
cg25868286	0.0019712	9.07E-07	4.286059	0.1121135
cg05255213	9.71E-11	5.90E-21	1.596271	0.054732
cg14598413	1.739E+09	4.33E-13	6.98E+30	0.4018513
cg07244253	0.0142986	0.0001546	1.322132	0.0659063
cg14756071	80.855287	1.4977559	4364.915	0.0308916
cg03869608	0.8360566	0.000884	790.7539	0.9591517
cg14028684	4.9716929	0.9159224	26.98671	0.0631397
cg23157360	2.2095855	1.083177	4.507359	0.0292853
cg04371413	1.2694328	0.5760598	2.797382	0.553985
cg27137084	0.4717797	0.1800603	1.236119	0.1263556
cg16553354	0.0922756	0.0024057	3.539396	0.2003066
cg16886414	4.1316276	0.9499296	17.97012	0.0585607
cg25930662	3.1433652	0.9910057	9.970421	0.0518203
cg25099065	0.7406361	0.368489	1.488625	0.3992492
cg17349199	2.3051993	0.5025521	10.57392	0.2825415
cg08213590	0.2263803	0.0621729	0.824283	0.0242564
cg09375377	8.9039109	1.7648284	44.92201	0.0080996
cg13375427	0.4869779	0.208324	1.138359	0.0967441
cg22215631	1.7340558	0.2785269	10.7959	0.555208
cg04137323	0.3694093	0.1276784	1.068804	0.0661795
cg16379716	0.1288153	0.036131	0.459256	0.0015793
cg18009690	1.9270928	0.9316249	3.986247	0.0768973
cg06048662	1.9226827	0.6567004	5.629217	0.2329818
cg06825661	2.784946	0.8253959	9.396611	0.0987997
cg08172461	1.45E-07	1.31E-25	1.61E+11	0.4576277
cg02491017	0.1023873	0.0074602	1.405215	0.0881207
cg00185170	1.22E-15	2.12E-27	0.000698	0.0129164
cg12820006	0.5316672	0.2482529	1.138638	0.103986
cg13381844	1.2989277	0.3200039	5.272478	0.7144421
cg26601310	1.5499545	0.6459828	3.718921	0.3264084
cg26067203	1.3479345	0.684237	2.655407	0.3880903
cg17144508	1.67E-10	8.36E-27	3346228	0.2398018
cg16289210	1.2063144	0.5199144	2.798912	0.6622629
cg09477453	0.3018977	0.0497289	1.832781	0.1930633
cg22621867	0.4413275	0.2150759	0.905587	0.0257223
cg02553516	1.878693	0.9668717	3.65042	0.0628062
cg17873910	7.9219337	0.8428926	74.45437	0.0702257
cg19710662	0.3276292	0.1336317	0.803259	0.0147377

cg15149712	1.6370284	0.3470415	7.722022	0.5334376
cg07167594	1.5054976	0.7289131	3.109456	0.2689302
cg08622934	4.0625957	1.0881301	15.16793	0.0370127
cg15066197	1.48479	0.578338	3.81196	0.4112687
cg12527995	3.7154076	1.2734385	10.84014	0.0162871
cg02954903	0.679859	0.3324141	1.390459	0.2905093
cg19311375	1.2750632	0.4501802	3.611411	0.6473405
cg00264419	1.3422777	0.5057731	3.562288	0.5544423
cg26681628	0.3783341	0.1439619	0.994268	0.0486525
cg11049583	0.7385773	0.3857237	1.414215	0.3605642
cg21998339	0.0081849	4.96E-05	1.350081	0.065076
cg05256269	1.9357417	0.6785531	5.522185	0.2168631
cg24431486	0.2740315	0.0689941	1.088402	0.0658292
cg27013630	0.0002549	1.87E-29	3.48E+21	0.7793111
cg05520031	0.2832902	0.1182874	0.67846	0.0046468
cg05807444	0.1934029	0.0421718	0.88696	0.0344868
cg09161043	1.2073298	0.4093541	3.560842	0.7327846
cg13921640	1.2021977	0.6238383	2.316753	0.5821924
cg14483244	0.0517313	0.0028585	0.936201	0.0450075
cg04239798	0.7358277	0.3903802	1.386962	0.3428695
cg21275135	1.3082011	0.6239722	2.742735	0.4769209
cg20079734	2.1276741	0.525814	8.609504	0.2897559
cg00051979	0.2670137	0.0039716	17.95167	0.5385485
cg18918390	0.2852468	0.1350526	0.602475	0.0010082
cg13530377	5.5684569	0.691391	44.8483	0.1066924
cg04720348	3.6518442	0.6208191	21.48124	0.1519534
cg11393040	1.9232464	0.3729013	9.919184	0.4345699
cg08344281	5.7056831	0.519263	62.69428	0.1544283
cg21215550	1.590863	0.8408494	3.009867	0.1535425
cg09790270	1.633129	0.7408741	3.599951	0.2238864
cg01882179	5.05E-16	1.12E-34	2277.003	0.1080081
cg10014563	1.3492993	0.6145521	2.962497	0.4552933
cg24842086	3.555292	1.59635	7.918126	0.0019039
cg26888861	0.0012544	2.13E-11	74030.41	0.4642765
cg26992634	0.4075902	0.1997126	0.831844	0.0136711
cg23352191	0.0018694	6.16E-06	0.567298	0.0312117
cg04903159	0.503429	0.1591459	1.592505	0.2427876
cg02145866	0.3218733	0.1512772	0.684852	0.0032545
cg24134219	1.7386224	0.7194035	4.201826	0.2192669
cg04031344	0.3766791	0.1431015	0.991514	0.0480164
cg22103164	11.270225	1.9196141	66.1685	0.007317
cg20426881	0.215779	0.0813728	0.572188	0.0020562
cg12485386	0.2513126	0.0935349	0.675235	0.0061684
cg04351169	7.521E+12	1.86E-11	3.05E+36	0.2850596
cg15134506	1.4862109	0.6606575	3.343371	0.3381262
cg09846177	1.6314004	0.6101068	4.362297	0.3294032
cg13698778	2.2827588	0.8738325	5.963371	0.0920487
cg09560365	42.455464	2.0810217	866.1449	0.0148392
cg00781749	0.5189356	0.154836	1.739221	0.287751
cg13054212	0.0966377	0.0076125	1.226785	0.0714956
cg08602433	0.0044175	7.03E-06	2.774492	0.0990411
cg02788441	0.1197188	0.002155	6.650922	0.3004056
cg01463828	0.2335385	0.0774651	0.704062	0.0097895
cg27061377	2.5259748	0.5251028	12.15105	0.2475967
cg11374933	4.035222	1.6443473	9.902419	0.0023206
cg08471713	1.9635509	0.681926	5.653887	0.2111239
cg08691479	4.44E+10	0.0002169	9.09E+24	0.1447878
cg04118234	1.7498468	0.8369257	3.658585	0.1370431

cg26311501	0.6846552	0.2423911	1.933869	0.4745595
cg00321478	0.3461763	0.1665829	0.71939	0.0044765
cg26276947	3.8780752	0.5865535	25.6404	0.159611
cg23914904	0.3607216	0.1332186	0.97674	0.0448274
cg00821600	1.2729249	0.6501322	2.492321	0.4814717
cg12726354	0.5898297	0.2698047	1.289447	0.18586
cg26098768	0.2459395	0.1070282	0.565143	0.000952
cg23402920	0.465087	0.0552676	3.913793	0.4811795
cg03625911	2.319711	0.5854275	9.191674	0.230995
cg14515581	0.4049488	0.168504	0.973173	0.0433056
cg27353957	0.6318648	0.3142175	1.270627	0.197748
cg15043384	0.1738211	0.0467442	0.646364	0.0090221
cg13526469	2.148547	0.9689337	4.764262	0.0597965
cg00445548	3.8169311	0.6935237	21.00716	0.123714
cg26079320	0.0298502	0.0018439	0.483239	0.01344
cg25023596	0.4849867	0.1930266	1.218548	0.1236916
cg18645035	0.406002	0.2093363	0.78743	0.0076517
cg07764073	1.2362764	0.2982238	5.124942	0.7700244
cg21186900	2.2959216	1.1174111	4.717383	0.0236904
cg23452289	1.6583229	0.7282878	3.776027	0.2282925
cg05764839	4.2434103	0.9317515	19.32547	0.0616817
cg25316856	0.0155013	0.0009904	0.242626	0.0029866
cg07116947	1.1410198	0.3654293	3.562731	0.8203555
cg24534477	1.0161535	0.4273573	2.41617	0.9710747
cg27537600	1.132832	0.5563466	2.306671	0.7310208
cg17465752	0.544633	0.2295433	1.29224	0.1680824
cg22956116	1.72E-09	5.73E-19	5.157409	0.0698893
cg17064754	2.7320113	1.0636691	7.017113	0.0367788
cg07434407	5.28E-12	8.16E-22	0.034186	0.0242682
cg15002204	0.9434869	0.4105118	2.168433	0.8910229
cg20306842	0.7381114	0.3933629	1.385002	0.3443212
cg12791065	0.4312234	0.2132817	0.871868	0.0191964
cg23903252	0.2235263	0.0481321	1.038061	0.0558396
cg00726029	2.5054887	0.5388794	11.64912	0.2414251
cg11439821	1.1863165	0.4683434	3.004946	0.7186222
cg07957995	0.2394756	0.0710421	0.807248	0.0211484
cg24268698	22.677844	0.0572433	8984.19	0.3064348
cg26184856	7.78E-09	1.35E-27	4.47E+10	0.3968716
cg00918944	0.3110844	0.0982094	0.98538	0.0471444
cg18961546	0.3270751	0.1029142	1.039488	0.0581829
cg16427743	12.239954	0.26316	569.2979	0.201066
cg20345446	0.1094705	0.0227291	0.527246	0.0058153
cg11962774	4.31E-05	7.82E-08	0.023785	0.0018036
cg21197219	1.361227	0.5181287	3.576214	0.5314768
cg05940946	1.605E+13	1.0414554	2.47E+26	0.0496943
cg13923645	6.1000883	0.5070953	73.38084	0.1541895
cg26342114	0.474229	0.1059389	2.122856	0.3292612
cg01556457	1.0136355	0.5424468	1.894116	0.9661343
cg11204152	0.6521979	0.2483813	1.712537	0.3855359
cg03201274	3.36E-11	4.20E-22	2.691799	0.0597463
cg24053165	3.0140986	1.447409	6.276588	0.0031984
cg06572713	0.1373619	0.0231804	0.813976	0.0287655
cg24537512	0.5625645	0.2470451	1.281057	0.1706689
cg01252496	0.1354817	0.030592	0.600002	0.0084693
cg10822665	3.1282442	0.4647895	21.0545	0.2410499
cg07628073	14.207021	0.4932408	409.2107	0.1216809
cg26848718	1.7325821	0.8293722	3.619413	0.143679
cg19803131	3.4571731	0.606978	19.69107	0.1622651

cg20699323	0.1750052	0.0683206	0.448281	0.0002814
cg22625220	0.0681416	0.0058731	0.790607	0.0317272
cg18725375	8.517669	0.9715892	74.67218	0.0531201
cg10220068	2.1465298	0.9722779	4.738964	0.0587061
cg02569778	1.1619245	0.6410495	2.106028	0.6208882
cg14113907	1.8295488	0.7152547	4.6798	0.2074467
cg12428378	0.5510453	0.2343546	1.29569	0.1718976
cg09796640	0.9755568	0.4730981	2.011657	0.9465654
cg26297506	2.29E-06	1.23E-12	4.275852	0.0779461
cg18631996	2.2146192	0.6417882	7.641989	0.2083338
cg15582376	2.7654797	1.2222141	6.257396	0.0146216
cg08892613	0.4546853	0.198798	1.039944	0.0618771
cg18878437	0.1720678	0.0455763	0.649621	0.0094215
cg01767631	1.8659149	0.496266	7.01567	0.3559623
cg06248767	2.3423381	1.2278054	4.468581	0.009803
cg22500140	0.2902765	0.1160583	0.726018	0.0081812
cg03984347	11.986762	1.0702228	134.2547	0.0439023
cg21572000	0.6194594	0.3181677	1.206062	0.1588926
cg05513208	0.7693361	0.311962	1.897276	0.5690929
cg03149245	0.1050411	0.0081248	1.358012	0.0844162
cg13713830	1.5634827	0.7510414	3.254785	0.2322189
cg04590790	0.3579227	0.1513785	0.846281	0.0192782
cg14242680	0.0459077	0.0055628	0.378859	0.0042189
cg12978105	1.6188344	0.8733564	3.000636	0.1260421
cg18349405	8.1980239	0.2303219	291.7985	0.2483544
cg05949203	8.2936907	1.419595	48.45418	0.0188234
cg09933190	1.1522219	0.5691495	2.33263	0.6937677
cg23644855	0.0758947	0.001694	3.400211	0.183813
cg23426958	0.3778904	0.1408143	1.01411	0.0533415
cg26194477	1.4135049	0.5661529	3.529075	0.4584937
cg09008179	1.0000284	0.3545656	2.820513	0.9999571
cg08846592	0.3693135	0.142907	0.954414	0.0397555
cg05091135	0.2301036	0.0798446	0.663134	0.006516
cg26067760	2.8551417	1.0719186	7.6049	0.035825
cg04503600	3.1735383	1.244571	8.092222	0.0156026
cg10831684	3.0609923	0.2169391	43.19034	0.4074408
cg20838323	1.8667714	0.7710703	4.519478	0.1664567
cg09879334	1.9046679	0.8469141	4.283504	0.1191989
cg01082907	2.4251843	1.1359242	5.177739	0.0220618
cg19826026	1.2868154	0.6867165	2.411321	0.4312765
cg27005935	0.4920389	0.1639669	1.476531	0.2059033
cg14852394	0.3973134	0.1616313	0.976654	0.0442789
cg11358114	13.278484	1.4190275	124.2528	0.0234081
cg08787791	1.5526298	0.3141572	7.673417	0.5894267
cg16200736	1.2925175	0.4482526	3.72692	0.6348607
cg03353394	0.1404005	0.036609	0.538456	0.0042019
cg19735151	0.4848912	0.2127123	1.10534	0.0851175
cg01756899	0.5402873	0.290969	1.003235	0.0512077
cg10116432	0.0071066	0.0002416	0.209014	0.0041399
cg18450168	1.75E-07	2.02E-14	1.518901	0.0563026
cg12798338	2.1078621	0.8711417	5.100299	0.0981323
cg01619692	1.49E-06	6.15E-26	3.63E+13	0.55587
cg24892433	2.9847769	1.4087307	6.324057	0.0043103
cg02238051	24.594172	2.72E-07	2.22E+09	0.731885
cg13484813	0.7496475	0.3830605	1.467056	0.4002544
cg00852414	0.4433427	0.2077424	0.946137	0.0354553
cg19111971	0.1746407	0.0196367	1.55318	0.1175673
cg09594635	1.5198474	0.5646992	4.09056	0.4072812

cg12091331	0.3279373	0.1181326	0.910357	0.0323344
cg02360980	4.0218109	1.0874369	14.87439	0.0370169
cg13761780	3558997	0.0004639	2.73E+16	0.1939486
cg16985255	2.7008456	1.0894195	6.695829	0.0319649
cg07639783	0.2128662	0.0442832	1.023234	0.0534469
cg26840889	1.4075519	0.4453513	4.448628	0.5604001
cg20455001	0.3131822	0.1560675	0.628466	0.0010869
cg06288570	0.3892798	0.0789285	1.919951	0.2465431
cg22960649	6.484E+09	3.13E-21	1.34E+40	0.5258636
cg07153588	0.0111328	3.57E-06	34.74976	0.2732314
cg05287409	0.2211635	0.0635033	0.770247	0.0177885
cg08108051	1.0327936	0.5152802	2.070063	0.9275275
cg11549972	1.3198618	0.6185901	2.816138	0.4729084
cg09744966	5.1269919	0.0006882	38196.8	0.7193636
cg08301965	1.956462	0.2501686	15.30065	0.5224632
cg25395619	0.4881085	0.1988033	1.19842	0.117582
cg26584034	0.4169547	0.1971735	0.881717	0.0220549
cg20159415	357.3009	0.0135573	9416638	0.2576878
cg14039968	2.0191591	0.6858316	5.944613	0.2021522
cg14269510	0.2538325	0.0783359	0.822496	0.0222697
cg13616508	1.3668502	0.2939517	6.355736	0.6902271
cg00292135	0.1783175	0.0441693	0.719893	0.0154549
cg12985929	0.7836166	0.3614144	1.699033	0.5368806
cg16656196	345199.87	1.1978983	9.95E+10	0.0467989
cg18666649	4.8947202	0.6865987	34.89416	0.1130203
cg02335376	0.0441831	0.0115631	0.168826	5.09E-06
cg13607316	0.030348	2.29E-12	4.02E+08	0.7688367
cg18330082	1.8492879	0.8077538	4.233797	0.1457319
cg02366262	0.3903695	0.1610296	0.946337	0.0373383
cg00731958	3.2218329	0.0208104	498.7979	0.6492752
cg19202886	0.2279543	0.0912443	0.569495	0.0015501
cg25129837	15.808085	0.0001061	2355620	0.6496736
cg17253810	0.2849416	0.0953803	0.851242	0.0245507
cg06431877	1.7423294	0.6829722	4.444854	0.2452459
cg06843596	0.3962959	3.94E-11	3.99E+09	0.9372189
cg12561217	83387879	0.0113336	6.14E+17	0.1156085
cg10275315	1.7406177	0.7325037	4.136157	0.209456
cg10394898	0.1617368	0.0268886	0.972857	0.0465884
cg26860459	4.3126553	0.0008066	23058.32	0.7386028
cg06295032	4.4000182	0.0939169	206.1414	0.4503348
cg22167515	3.0196467	0.9276587	9.829332	0.0664668
cg12094104	0.6821744	0.2795102	1.664919	0.4008201
cg11398680	1.42E-08	6.24E-15	0.03223	0.0155231
cg11756734	3.0044233	0.833132	10.83449	0.0927636
cg23601905	1.5268565	0.4899865	4.757867	0.4655145
cg00678239	1.72E-12	2.26E-24	1.306479	0.0522812
cg05584078	1.8233832	0.4850599	6.854259	0.3739442
cg19797741	1.6909029	0.6231832	4.58798	0.3023647
cg23430771	0.410003	0.183536	0.91591	0.0296934
cg23285459	0.2553543	0.1322227	0.493151	4.80E-05
cg02978737	1.4716112	0.6554637	3.303981	0.3491214
cg21231400	0.1515269	0.0252684	0.908661	0.0389447
cg05546863	3.5697754	1.0221075	12.46767	0.0461266
cg17388689	1.6426502	0.6242351	4.322569	0.3147104
cg11318342	0.1814874	0.061541	0.535215	0.0019827
cg14196507	0.7441211	0.342508	1.616652	0.4553231
cg18185116	0.6832224	0.3161754	1.476373	0.3325571
cg24595606	3.4588376	1.3269447	9.015868	0.0111274

cg11422861	5016.0862	2.6637888	9445614	0.0267862
cg21385052	5.7765267	0.6113551	54.58081	0.1258852
cg27183113	0.6196584	0.2626505	1.461929	0.274474
cg15259986	0.0464741	0.0024656	0.876007	0.0405284
cg01048962	1.2844361	0.666859	2.47395	0.4541783
cg04918002	0.2220189	0.0783706	0.628965	0.0046156
cg04427437	0.5994648	0.3090853	1.16265	0.1300087
cg01129459	1.3301586	0.6016034	2.94101	0.4809762
cg03738915	2.518356	0.3957678	16.02485	0.327964
cg16359050	31.501287	2.4485666	405.2702	0.00812
cg26064634	1.1604669	0.0983346	13.6949	0.9059268
cg25989745	0.6342785	0.3186777	1.262433	0.1948452
cg21029612	3.0192816	1.1991716	7.601966	0.0190021
cg23282585	1.3097764	0.7097143	2.41719	0.3880421
cg15318546	0.3177171	0.1351806	0.746735	0.0085439
cg12847793	0.3930889	0.1399127	1.104394	0.0764667
cg16016960	7.8734829	0.2318845	267.3388	0.2512418
cg05298088	1.8973209	0.7074942	5.088135	0.2032086
cg15425827	3.1818301	0.95623	10.58746	0.0591607
cg26967100	3.9844553	1.0065601	15.77242	0.0489211
cg23023844	1.6800239	0.6076693	4.644764	0.3173521
cg07595554	1878007.9	0.0256938	1.37E+14	0.1179033
cg09546332	1.07E+10	0.1529796	7.48E+20	0.0698934
cg25588740	0.4082887	0.17479	0.953714	0.0385039
cg04905719	0.1661905	0.0328459	0.840874	0.0300463
cg25371314	1.4192906	0.5304519	3.79749	0.4855995
cg24568579	1.0371777	0.3642496	2.953297	0.9454901
cg25251562	0.3648052	0.1768797	0.752392	0.0063286
cg06237983	1.6573352	0.6529843	4.206472	0.2877317
cg23560214	5.91E-05	2.62E-08	0.133154	0.0134422
cg15462349	1.9551798	0.9770912	3.912355	0.0581612
cg09680007	0.2331999	0.0774545	0.702117	0.00963
cg16680125	1.1122425	0.3995289	3.096355	0.8386339
cg27245348	0.494959	0.1905354	1.285768	0.1487659
cg02513556	0.5031556	0.2585851	0.979041	0.0431424
cg15373592	2.7496408	0.555968	13.59885	0.214909
cg08378220	2.5058442	0.8066261	7.784592	0.1121981
cg22824265	429.49157	3.6064985	51147.4	0.0129208
cg01198994	0.7989382	0.206118	3.096781	0.7453849
cg08746254	2.2303738	0.6415649	7.753802	0.2070202
cg17527435	1.403456	0.0195667	100.6655	0.8764502
cg14871704	3.028E+11	16.292315	5.63E+21	0.0284313
cg11223252	0.0031683	1.72E-06	5.824711	0.1334859
cg14153777	0.2993405	0.0443845	2.018829	0.2155031
cg18119735	0.3651576	0.1086434	1.227318	0.1033565
cg27331665	1.7499892	0.6801117	4.502881	0.245838
cg07816047	0.1702521	0.0688314	0.421113	0.0001273
cg18358020	2.6616125	1.4122399	5.016273	0.0024661
cg09838956	0.7381335	0.1855491	2.936371	0.6664799
cg04748546	0.2589342	0.0869582	0.771024	0.0152221
cg25710753	0.5276873	0.1861231	1.496074	0.229248
cg03515290	3.78E-07	3.75E-10	0.00038	2.76E-05
cg10856045	2.8867042	1.0447894	7.975829	0.0409078
cg25503169	0.2258234	0.08326	0.612494	0.0034678
cg24864097	0.3167312	0.0793633	1.264044	0.1034952
cg27422857	3.409884	0.8273996	14.05283	0.0895571
cg04300115	17.270886	1.4380449	207.4229	0.0246779
cg18050804	0.187444	0.073243	0.479708	0.0004792

cg09169953	2.2462786	1.0372077	4.864761	0.0401091
cg14802591	0.080752	0.0076587	0.851436	0.036279
cg10388017	7.9511066	0.7343736	86.08711	0.0880206
cg02241397	1.3416739	0.6375831	2.823301	0.4387547
cg02257884	6.4689571	0.6351931	65.88139	0.1148634
cg10001715	3.213283	0.9798844	10.53715	0.0540509
cg06103243	0.8758555	0.3813419	2.011641	0.7547008
cg11322687	0.1565393	0.0124748	1.964328	0.1507608
cg20231444	5.1825166	1.2065432	22.26069	0.0269358
cg06480171	1.3679487	0.5265422	3.55391	0.520098
cg24988036	0.2023071	0.0765279	0.534814	0.0012741
cg27580318	1.3724484	0.4417261	4.264214	0.5841337
cg18458352	2.1098767	0.4054616	10.97904	0.3749529
cg03262773	0.0013831	2.69E-10	7102.607	0.4036762
cg00236305	0.6907752	0.3243872	1.47099	0.3374353
cg13621113	0.223609	0.0957394	0.522261	0.0005384
cg27254348	1.0592065	0.0804694	13.94218	0.9651111
cg06564232	1.9397054	1.0439369	3.604104	0.0360826
cg18682430	0.3474565	0.0960656	1.256704	0.1070452
cg22684151	1.7487583	0.7766984	3.937378	0.1771095
cg13178524	4.304516	0.6969052	26.58734	0.116124
cg13565928	1.3622869	0.5710984	3.249572	0.4857963
cg23870587	0.2267202	0.0723504	0.710459	0.0108792
cg25911551	0.4040344	0.1673699	0.975347	0.0438546
cg00941050	2.5730022	0.9989278	6.627446	0.0502603
cg15226275	0.6135088	0.302	1.246334	0.176688
cg18629132	1.6892288	0.8439422	3.381149	0.1386741
cg19512969	0.5014809	0.2229589	1.127935	0.0951438
cg20325850	0.8586088	0.3889441	1.895412	0.705946
cg08481075	0.1951056	0.0621942	0.612054	0.0050851
cg15783848	0.0033534	4.34E-05	0.258966	0.0101944
cg14007706	0.1956256	0.0639717	0.598223	0.0042246
cg04042468	0.18221	0.0642673	0.5166	0.0013639
cg02020296	3.94E-07	1.06E-35	1.47E+22	0.6604106
cg03210866	1.6886158	0.5455603	5.226597	0.3634397
cg19972648	0.4254548	0.1455386	1.243737	0.118421
cg26727032	0.5627084	0.1925366	1.644574	0.2933462
cg06014057	0.0892104	0.0185206	0.42971	0.0025869
cg10509626	1.3629914	0.6371512	2.915706	0.4247621
cg10267872	3.3674016	1.2254162	9.253504	0.0185673
cg20129314	2.1714099	1.0253734	4.598345	0.0428246
cg17297216	1.0030164	0.5743806	1.751525	0.9915514
cg25897251	2.1796454	0.2703535	17.57275	0.4643707
cg08074321	0.4894621	0.2604931	0.919691	0.0264111
cg16482878	1.7497453	0.8321081	3.67934	0.14013
cg26288991	1.3060377	0.4811147	3.54538	0.6002681
cg19184885	0.2758788	0.1335402	0.569934	0.0005038
cg24111025	2.0159474	1.0165208	3.997994	0.0447637
cg05380759	0.5969154	0.2422417	1.470878	0.2621275
cg09731694	0.0565764	0.0082117	0.389795	0.0035374
cg13455634	0.5859805	0.3037824	1.130326	0.1108265
cg03774520	1.6555073	0.7345819	3.730972	0.2240046
cg07186032	3.5987551	0.8045875	16.09649	0.0938382
cg23993425	1.5847632	0.7208804	3.483899	0.2519463
cg15207313	0.4759937	0.2212678	1.023963	0.057515
cg09945896	0.2981866	0.1002388	0.887034	0.0295944
cg06323052	0.3586362	0.1116159	1.152344	0.0850938
cg07632946	0.1521119	0.0290347	0.796909	0.0258387

cg01233392	0.325808	0.1406951	0.754474	0.0088562
cg16546016	0.803379	0.3914468	1.648801	0.5506346
cg01953232	0.5761796	0.2567288	1.293127	0.1813159
cg19280364	0.2515602	0.096812	0.653664	0.004617
cg10080004	0.0049948	0.0003701	0.067401	6.57E-05
cg22491320	0.615114	0.2920442	1.295575	0.2010343
cg14843632	1.1557965	0.6524281	2.047529	0.6197119
cg10547426	0.7991094	0.3814765	1.673958	0.552237
cg04159215	0.9057686	0.4027725	2.036923	0.8108262
cg14755852	0.0015507	1.82E-05	0.131768	0.0043152
cg05641903	1.2425788	0.4978841	3.101127	0.6416151
cg02771362	2.4603562	0.8188935	7.392112	0.108715
cg10476689	1.6274233	0.422511	6.268491	0.4790678
cg21965980	0.3403272	0.1108492	1.044867	0.0596627
cg06483802	1.01E-16	5.54E-28	1.86E-05	0.0053792
cg24750752	4.0690596	1.111336	14.89851	0.0340583
cg05740254	0.333392	0.1528311	0.727275	0.0057771
cg16022081	2.3640829	0.9109092	6.135505	0.0770279
cg16016034	0.3272429	0.1397791	0.766123	0.0100586
cg20024324	0.0157459	0.0002171	1.141822	0.0575271
cg23060272	0.0588268	0.0056474	0.612774	0.0178079
cg08420239	0.0293948	0.0018186	0.47511	0.0129867
cg19772092	6.7055449	0.7079573	63.51277	0.0971388
cg12738718	0.0038081	3.52E-06	4.124232	0.1181618
cg06077405	1.3825849	0.0003644	5246.016	0.9385886
cg13464915	0.0019677	4.14E-05	0.093519	0.0015628
cg04832534	0.1825125	0.0007024	47.42387	0.5487775
cg22488717	0.1000733	0.0139139	0.719762	0.0222178
cg26874367	1.7883507	0.9394299	3.404403	0.0767707
cg06939307	1.3789263	0.5351149	3.553327	0.505867
cg10865498	0.3116206	0.1104027	0.879574	0.0276417
cg22330638	0.8172313	0.3294294	2.027344	0.6632731
cg10899768	3.6566437	1.2597947	10.61367	0.0170907
cg19582946	0.2964441	0.0198463	4.42798	0.3781116
cg08433504	0.30529	0.1087296	0.85719	0.0242902
cg22745369	2.8557085	0.908867	8.972788	0.0724346
cg06097216	3.11E-25	1.44E-45	6.70E-05	0.0181629
cg19753476	4.079953	0.9336407	17.82915	0.0616633
cg07744912	0.1247636	0.0269617	0.577335	0.0077505
cg13758899	0.2919005	0.0580962	1.466633	0.134916
cg15676988	0.2163552	0.0776515	0.602816	0.0034106
cg01906797	0.1295427	0.0013888	12.08309	0.377144
cg02347249	0.7087758	0.2677095	1.876523	0.4883607
cg19913563	0.7534651	0.2505528	2.265829	0.6143242
cg24503407	3.2737663	1.141472	9.389232	0.0273766
cg09475324	2.65E-06	3.79E-34	1.85E+22	0.6946597
cg19248242	2.14E-05	1.25E-11	36.64052	0.1420538
cg22901249	0.3696781	0.152081	0.898612	0.028102
cg05921138	0.8910206	0.4449937	1.78411	0.7446297
cg22484737	1.0144732	0.5188753	1.983436	0.9664936
cg14389122	0.1460912	0.0558879	0.381883	8.73E-05
cg13987674	0.4416329	0.2007538	0.971536	0.0421788
cg21229718	0.4938493	0.2407264	1.01313	0.0543065
cg01451808	0.5763803	0.2768836	1.199834	0.1407674
cg11209832	1.8835999	0.9633161	3.683058	0.0642091
cg14289461	0.247557	0.0902205	0.679275	0.0067102
cg05462339	0.1658352	0.0441164	0.623381	0.0078261
cg21446772	1.8931101	0.9246595	3.875876	0.0808613

cg07026490	0.3081627	0.0929444	1.021731	0.0542532
cg06544310	0.0007536	1.30E-06	0.437562	0.0267933
cg03042113	1448852.9	8.90E-23	2.36E+34	0.6686291
cg18719750	0.8766443	0.2430886	3.161421	0.8405649
cg21133433	0.2898714	0.1027652	0.817645	0.0192588
cg03004598	2.5866139	0.9981674	6.702856	0.050443
cg18458017	2.7764755	1.2777951	6.032905	0.0099066
cg27573549	3.2543413	0.1835247	57.70743	0.4212124
cg23540020	1.4967252	0.7010578	3.195437	0.2973418
cg00717279	2.6091214	0.836427	8.138803	0.0984866
cg02301769	0.0237739	0.0007055	0.801118	0.0372034
cg05889171	0.4269522	0.1628428	1.119412	0.083525
cg01683055	0.0003983	3.37E-09	47.04203	0.1889445
cg22232952	0.1456373	0.0366764	0.578307	0.0061749
cg24401487	0.2851897	0.0830634	0.979169	0.0462164
cg24620905	0.2096576	0.0719994	0.610509	0.0041719
cg10387662	4.3494546	0.3582319	52.80868	0.2484771
cg00384847	0.2655444	0.0742289	0.949951	0.0414584
cg12434747	3.1925206	1.1405918	8.935877	0.0270732
cg21046874	1.0574394	0.4470537	2.501217	0.8988237
cg07286123	1.7756568	0.9151801	3.445176	0.0895332
cg12887033	1.4082297	0.6551307	3.027046	0.3806049
cg08130175	1.2061872	0.5460605	2.664334	0.6429117
cg10996258	0.5812479	0.2797352	1.207746	0.1459182
cg07704300	0.2397128	0.0433069	1.326861	0.1018352
cg04742397	0.6352081	0.1982937	2.034807	0.4448747
cg13646480	0.1732562	0.0625241	0.480099	0.000749
cg03826564	0.5481746	0.2798206	1.073886	0.0797404
cg13994504	3.2532997	0.6023798	17.57024	0.1703993
cg00190162	2.8167368	0.9272378	8.556604	0.067744
cg19139092	0.1458464	0.0637818	0.333499	5.06E-06
cg04680754	0.2678714	0.0871339	0.823504	0.021513
cg16699818	0.2528394	0.1203274	0.531282	0.0002841
cg02966200	1.3459557	0.3422389	5.293369	0.6706558
cg08421587	0.9572163	0.4027985	2.274743	0.9211314
cg10106268	18.936973	0.1788429	2005.162	0.2163151
cg15002904	0.7914285	0.3021496	2.07301	0.6339872
cg21968515	2.2837182	1.0403634	5.013026	0.039532
cg05740157	0.4750952	0.1931177	1.168798	0.1051525
cg00050872	1.1242024	0.3907323	3.234519	0.8281099
cg00758881	1.0326587	0.5667955	1.881426	0.9163784
cg13140981	0.2120314	1.34E-23	3.36E+21	0.9525765
cg01613077	1.3427592	0.4116202	4.380257	0.6251595
cg13497155	4.941E+09	45.998432	5.31E+17	0.0179934
cg16468773	1.6698215	0.7985854	3.491554	0.1730892
cg00649632	3.8506231	0.675947	21.93559	0.1288173
cg23401445	2.1240184	0.7201795	6.264347	0.1722169
cg18264383	4405538.9	1.49E-18	1.31E+31	0.5946387
cg20473155	3.0656138	1.0657134	8.818494	0.0377073
cg16485897	0.9634666	0.4113599	2.256583	0.9316973
cg01737026	7.8728091	2.2294717	27.80081	0.0013483
cg07525804	0.3236057	0.0752823	1.39104	0.1294266
cg00545469	1.9365315	0.9363105	4.005246	0.0746716
cg10692693	0.5129699	0.2586591	1.017317	0.0560277
cg16203262	0.4535962	0.2198129	0.936021	0.0324482
cg00327383	5.554515	0.7908054	39.0142	0.0847117
cg18437319	0.7305044	0.288653	1.848714	0.5074232
cg08309041	1.9087791	0.5460544	6.672298	0.3113366

cg12974394	0.2746625	0.1115349	0.676376	0.004949
cg03082741	0.489981	0.1124799	2.134438	0.3420418
cg17355809	0.2908226	0.1134168	0.745726	0.0101508
cg20904489	3.2065153	1.3856796	7.419999	0.0064893
cg07965774	0.2131475	0.0740621	0.613429	0.0041561
cg03543893	1.8358083	0.8963985	3.759703	0.0967268
cg27331738	5.1499658	0.305588	86.79055	0.2554057
cg17836141	0.4861705	0.201302	1.174165	0.1089178
cg08897188	3.2471957	1.0140309	10.39838	0.0473196
cg07697189	2.649584	0.0876771	80.06988	0.5752722
cg22017797	0.0315916	4.25E-08	23506.41	0.6164782
cg25953688	0.3025687	0.1275097	0.717968	0.0066984
cg11923054	2.524E+17	1037.0826	6.14E+31	0.0177481
cg05654765	0.1032245	0.0072208	1.47565	0.0942763
cg03196689	0.1717762	0.0215717	1.367859	0.0961013
cg26578682	3.2237258	0.9615683	10.80777	0.0578979
cg16707400	5.1694904	0.3921836	68.14062	0.2118271
cg09958192	0.8428137	0.3318112	2.14078	0.7191781
cg22924563	1.6411489	0.6734806	3.999179	0.2756615
cg24293336	0.9378517	0.4297406	2.046737	0.8719804
cg04711162	0.1460385	0.0355335	0.600201	0.0076335
cg01058902	1.9604183	0.1032686	37.21597	0.653996
cg01303723	0.244037	0.053975	1.103363	0.0669234
cg06777194	1.3246762	0.661191	2.653949	0.4277451
cg09774007	1.5116185	0.7916099	2.886511	0.2106027
cg01803258	0.6140317	0.2739881	1.3761	0.236193
cg15928093	0.1865907	0.0261011	1.333894	0.0943503
cg14859724	0.9853552	0.5419765	1.791452	0.9614202
cg14702215	10.481947	0.0002468	445169.5	0.6656319
cg04336905	3.2332075	0.0048871	2139.022	0.7232386
cg02011392	1.8396027	0.9673805	3.498249	0.063051
cg17127816	8.1360086	0.631045	104.8969	0.1080466
cg15520279	0.0391581	0.0002572	5.962202	0.2063564
cg08178035	5259.8666	2.22E-22	1.25E+29	0.7737978
cg25595641	1.5288522	0.3361406	6.953605	0.582804
cg20173277	0.0003741	3.08E-08	4.539817	0.1000422
cg04249522	1.9533572	0.9402244	4.058185	0.0726943
cg10846969	0.4798559	0.0083152	27.69155	0.7226864
cg26489108	1.7672979	0.9212329	3.390393	0.0866856
cg13442689	0.2567722	0.0704934	0.935292	0.0392656
cg16134515	2.3745254	0.6573541	8.577372	0.1869243
cg21716061	0.2852236	0.1023132	0.795132	0.0164749
cg15377111	0.637203	0.2969386	1.367379	0.2473533
cg13999143	0.2438708	0.1075111	0.55318	0.0007334
cg00537820	0.9122208	0.252474	3.29597	0.8885199
cg25141611	4.9138908	1.7761823	13.59451	0.0021664
cg00545796	264438110	2.40E-16	2.92E+32	0.4923368
cg03344570	2.127228	1.0703672	4.227614	0.0312386
cg07543883	1.9519864	0.5006127	7.611174	0.3353648
cg27051686	0.405006	0.1993324	0.822896	0.012459
cg14765818	2.5764011	0.1454676	45.63106	0.5186924
cg02009040	4.8886984	1.4438305	16.55275	0.0107654
cg16158874	3.8710437	0.9519397	15.74152	0.0586047
cg20332679	0.2151958	0.0475487	0.973932	0.0461243
cg19828460	2.9123092	0.9570099	8.862546	0.0597578
cg02385153	1.5147121	0.6555743	3.49976	0.3311653
cg18993457	1.3569202	0.5878764	3.132006	0.4744988
cg07691705	0.3515289	0.1140336	1.08365	0.0687432

cg27182935	0.3582994	0.0557369	2.303294	0.279641
cg14288266	0.2649311	0.0471801	1.487673	0.1313559
cg04428320	0.1975865	0.0853725	0.457295	0.0001522
cg02163215	4.2185104	0.5877531	30.27773	0.1522948
cg05210373	0.1609803	0.0602379	0.430205	0.0002707
cg21807215	1.1944857	0.2520861	5.659955	0.8228376
cg16792800	0.2886127	0.0711657	1.170469	0.081927
cg23534802	1.4619585	0.6727045	3.177209	0.3375922
cg00078245	2.1317236	0.2094302	21.69814	0.5225739
cg16125214	0.5800109	0.2742196	1.2268	0.1541124
cg19307584	0.5535558	0.2833719	1.081349	0.0834446
cg14696926	2.1451137	1.1569915	3.977136	0.0153968
cg03643345	0.2744707	0.1142447	0.65941	0.0038389
cg22705602	0.2766798	0.0885828	0.864182	0.0270243
cg04510815	0.3255854	0.1239384	0.855311	0.022779
cg02285386	0.2646912	0.1239705	0.565146	0.0005936
cg16332813	0.0144773	0.0005337	0.392748	0.0119051
cg19256291	0.3717217	0.1170871	1.180121	0.0931566
cg25009498	0.2945349	0.129242	0.671228	0.0036315
cg03916909	2.1161814	0.79003	5.668422	0.1359252
cg04681554	1.6233327	0.7263246	3.628142	0.2377218
cg18043120	1.6845687	0.7263717	3.906776	0.2243305
cg14747086	0.2760754	0.0753729	1.011208	0.0519995
cg10000250	0.4660548	0.1592503	1.363935	0.1634794
cg15387361	0.023422	2.99E-05	18.34698	0.2695081
cg19204958	0.1815261	0.0789289	0.417486	5.93E-05
cg03666028	2.8743914	0.7245643	11.40289	0.1331691
cg12623101	0.2861485	0.10117	0.80934	0.0183375
cg22168987	0.3076824	0.1417256	0.66797	0.0028805
cg24792289	1.5074151	0.6987305	3.252041	0.2954969
cg15122966	1.8817288	0.6234157	5.679842	0.2620313
cg03626511	0.3685116	0.1464044	0.927573	0.03404
cg26856578	0.2720092	0.0988037	0.748848	0.011745
cg20420651	4.3688032	1.2920449	14.77227	0.0176832
cg19866195	1.1643058	0.6009836	2.255649	0.6520903
cg03311684	1.871199	0.9983669	3.507113	0.050599
cg19522294	347682.68	0.5366116	2.25E+11	0.061652
cg17625535	1198727.7	9.40E-06	1.53E+17	0.2833702
cg07159961	1.4381796	0.5875381	3.520385	0.426269
cg03830329	1.796253	0.824316	3.914184	0.1405328
cg25312372	0.2167734	0.0749279	0.627146	0.0047906
cg09606766	0.9917473	0.3612579	2.722605	0.987168
cg04210100	0.0887481	0.0156275	0.503999	0.0062723
cg17001765	0.5833778	0.2394687	1.421186	0.2355179
cg07050626	3.9434303	1.2397556	12.54331	0.0201262
cg08586892	401.79944	6.16E-06	2.62E+10	0.5136931
cg14023999	0.4976342	0.2537151	0.976055	0.0423079
cg05415131	0.4849098	0.197299	1.191783	0.1146673
cg09002358	1.3156101	0.4772929	3.626348	0.5959488
cg21195277	0.3176272	0.1403624	0.718761	0.0059141
cg07212543	0.2971421	0.1250381	0.706132	0.005999
cg16351318	1.8887046	0.7144778	4.992744	0.1998071
cg12502577	1.2630721	0.6380715	2.50027	0.5026404
cg03948870	1.1540011	0.5834913	2.282328	0.680589
cg06892152	0.0800622	5.69E-15	1.13E+12	0.8701567
cg18338460	4.7260828	1.5238329	14.65768	0.0071587
cg09400316	1.6401739	0.5903598	4.556832	0.3425796
cg14528756	0.0491808	0.0031834	0.759804	0.0310343

cg06573088	0.5091383	0.2426224	1.068417	0.0742659
cg26801613	0.4508059	0.2037312	0.99752	0.0492882
cg00621258	0.1727118	0.0619635	0.481403	0.0007858
cg25406374	0.5308889	0.2544959	1.107456	0.0914317
cg10819420	5.5789006	1.3479784	23.08949	0.0176919
cg06274857	2.1400081	1.0464356	4.376413	0.0371316
cg10773780	4.4824263	0.5519258	36.40371	0.1603783
cg23184556	0.2058992	0.0608206	0.697041	0.0110841
cg16119421	13.165839	0.3952494	438.5568	0.1495763
cg06844526	3.1317515	0.3306528	29.66213	0.3196405
cg04433035	0.8344509	0.3306471	2.105895	0.7015872
cg27087377	0.0855837	0.0063918	1.145932	0.0633026
cg10622644	0.3804132	0.1339681	1.080214	0.0695145
cg03328041	439777849	0.0001934	1.00E+21	0.1703925
cg07458482	4.717842	1.1745133	18.95086	0.0287649
cg13668823	0.5042111	0.0826852	3.07466	0.4578852
cg09515805	2.624E+11	8.11E-07	8.49E+28	0.2011876
cg25953130	0.2878884	0.0926649	0.894402	0.031325
cg19622777	0.4103907	0.2104763	0.800188	0.0089422
cg14601621	0.4226533	0.1979321	0.90251	0.026083
cg10376161	3.5806818	0.7334965	17.47968	0.1148351
cg23244022	1.5693968	0.6650958	3.703236	0.30352
cg10613546	0.4101672	0.1742486	0.9655	0.041316
cg18323768	2.538361	1.2630396	5.101405	0.0089048
cg03522668	1.099027	0.5626145	2.14687	0.7822448
cg17527422	0.3190366	0.1490152	0.683047	0.0032674
cg12854611	0.3002388	0.0545613	1.652148	0.1666975
cg14608424	71.391091	0.8085712	6303.326	0.0618993
cg15109221	0.1512078	8.53E-13	2.68E+10	0.8863276
cg01283300	0.3276344	0.087503	1.22675	0.0976075
cg13769605	1401019.2	3.4702996	5.66E+11	0.0316436
cg04525852	0.2224455	0.0627228	0.7889	0.0199617
cg21771171	1.37E+12	0.0022734	8.26E+26	0.1075207
cg12376422	0.8081769	0.4017873	1.625611	0.5503128
cg16577724	9.5374637	0.5445306	167.0489	0.122622
cg16190718	0.1110462	0.0295893	0.416747	0.0011256
cg00806644	1.5247976	0.8067367	2.881991	0.1940168
cg27113015	0.2239691	0.0641442	0.782022	0.0190082
cg04847825	0.3360022	0.1125726	1.002885	0.0506067
cg21141812	1.5321955	0.4974006	4.719783	0.4572668
cg25240964	0.437323	0.1897185	1.00808	0.0522489
cg10096215	2.4399275	1.0089454	5.900464	0.0477339
cg18330370	0.410053	0.1387714	1.211658	0.1068192
cg10164393	1.2421368	0.4197381	3.675873	0.6952749
cg05870586	0.3312065	0.131531	0.834007	0.0190172
cg07643097	0.5756406	0.2777934	1.192837	0.1373793
cg05919456	1.0357357	0.5096276	2.104965	0.922696
cg14262681	2.2738698	0.9915907	5.214333	0.0523772
cg17293868	0.3374294	0.0916448	1.24239	0.1023413
cg19532307	1.4020951	0.7230387	2.718901	0.3172051
cg02771336	0.7536291	0.1888344	3.007698	0.688745
cg14128641	1.1861547	0.4835895	2.909416	0.7092067
cg22489204	0.1658184	0.0529795	0.518989	0.0020245
cg25292309	0.5354856	0.1897327	1.511309	0.238062
cg03346706	0.3554473	0.1196889	1.055593	0.0625265
cg01776298	1.7389551	0.8271766	3.655767	0.1444358
cg26928531	1.6379166	0.6787609	3.952454	0.2722766
cg10164640	0.1727066	0.0159573	1.869206	0.1483998

cg17704864	0.3586427	0.0100229	12.83311	0.5742546
cg24184689	1.9735911	0.5934539	6.563378	0.2674803
cg25130962	0.3160715	0.1086426	0.919539	0.0345224
cg03020006	0.5270127	0.2631075	1.055623	0.0707258
cg14253677	3.134576	0.110218	89.14668	0.5035763
cg09576074	2.3149423	0.6789015	7.893572	0.1798656
cg19217214	3.1609784	0.7329493	13.6323	0.122748
cg02796420	0.4378526	0.2083968	0.919951	0.0292409
cg01061697	3.6190048	0.7408653	17.67824	0.1119843
cg20099830	1.6703637	0.8746144	3.190109	0.1201536
cg01583940	0.179106	0.0751065	0.427113	0.0001051
cg07148407	0.5459681	0.2795501	1.066289	0.0763895
cg14524579	0.3935518	0.1594487	0.971366	0.0430744
cg16454551	1.5845109	0.8704884	2.884214	0.1320392
cg19366091	0.2382888	0.105897	0.536196	0.0005279
cg08393822	0.4668326	0.2365985	0.921107	0.028023
cg26230851	0.3389423	0.1543347	0.744368	0.0070292
cg26275898	5.9905003	1.5422528	23.26862	0.0097168
cg24478803	0.2194246	0.0482038	0.998825	0.0498225
cg12422704	0.2593481	0.0848091	0.793092	0.0179599
cg04273148	0.7602237	0.0341386	16.92923	0.8625352
cg15409587	2.1201456	0.8966746	5.012986	0.0869766
cg05244766	0.5146097	0.2263543	1.16995	0.1128769
cg22136098	0.4331504	0.188393	0.995893	0.0488782
cg05284927	1.16E-11	8.05E-23	1.657647	0.0546806
cg11119033	0.6897324	0.2986801	1.592777	0.3843643
cg23632656	1.4703338	0.7158954	3.019829	0.2938144
cg00817598	2.1782566	0.9988117	4.750447	0.0503504
cg23656906	0.2303232	0.0052088	10.18447	0.4475676
cg12926300	0.6110648	0.3014528	1.238669	0.1718574
cg06197760	47.850553	0.9143529	2504.149	0.0554134
cg05056545	0.9723625	0.5056863	1.869714	0.9330431
cg23677363	8513.8946	25.192345	2877319	0.0023191
cg13176070	0.408404	0.1659039	1.005364	0.0513761
cg14425564	3.3079736	0.8999645	12.15902	0.0716606
cg09841704	0.5564552	0.2781661	1.113156	0.0975316
cg00405069	4.2821074	1.0122157	18.11515	0.0481023
cg14192813	1.7786251	0.5896338	5.365207	0.3066769
cg11178337	1.7170612	0.5791473	5.090759	0.3295867
cg00042657	0.2759656	0.0677183	1.124615	0.0724746
cg11637718	2.1084491	0.10103	44.00237	0.6303709
cg10838091	0.2156431	0.0559357	0.831347	0.0258644
cg05791506	1.2053758	0.1911082	7.602661	0.8424306
cg07733031	4.05E-14	5.44E-27	0.302196	0.0414405
cg16564940	0.0505737	0.0049716	0.514464	0.0116848
cg22843712	1.2002554	0.5720699	2.518246	0.6292452
cg12742826	1.4987211	0.1661958	13.51517	0.7184007
cg15765502	1.6060275	0.8108827	3.180885	0.1742277
cg26991761	0.1023204	0.0147628	0.709179	0.0210067
cg12077685	1.4555651	0.7181595	2.950138	0.2976545
cg22614142	4.577013	1.0016109	20.91536	0.0497578
cg12592409	0.1622669	0.0376713	0.698955	0.01466
cg01497527	0.2533742	0.0209063	3.070771	0.2807837
cg04038089	1.453212	0.7282904	2.899702	0.2889429
cg10695490	0.7313514	0.267064	2.002797	0.5427307
cg04681489	2.1312917	1.1673206	3.891309	0.013753
cg27314798	16.245566	1.5766793	167.3888	0.0191518
cg10824582	0.7676611	0.3984486	1.478995	0.4293765

cg22416376	2.7392739	1.1766104	6.377321	0.0194301
cg23534245	0.40641	0.1192688	1.384847	0.1500237
cg06739303	0.3193168	0.0919184	1.10928	0.0723783
cg02387510	7.1472363	0.1453394	351.4737	0.3223929
cg07956247	0.4555423	0.178374	1.163392	0.1002578
cg05222958	1.3281522	0.4589471	3.843554	0.6006658
cg19292760	0.4192969	0.1657855	1.060466	0.0663645
cg03339077	0.3945919	0.1228861	1.26705	0.118216
cg14236389	2.2098044	0.7831984	6.234991	0.1340761
cg20344925	0.368645	0.1324028	1.026407	0.0561235
cg00713214	1.2607733	0.512439	3.101929	0.6139316
cg21019522	0.1800963	0.0502485	0.645485	0.008486
cg24962684	0.2921012	0.107754	0.791832	0.0155764
cg04857710	14.626027	2.1672439	98.70632	0.0058885
cg10142237	0.1943363	0.0479615	0.787437	0.0217496
cg06825163	1.2121516	0.4294159	3.421652	0.7163196
cg03417473	0.3348119	0.133471	0.839875	0.0197086
cg10288437	0.2508988	0.068313	0.921497	0.0372389
cg19245980	0.3316449	0.1588646	0.69234	0.003292
cg23772395	0.0302282	0.0001037	8.812875	0.2268954
cg11035271	0.3999131	0.1809766	0.883708	0.0234778
cg24120683	2.6858874	1.4054485	5.132875	0.0027901
cg11507187	7.2819386	0.4306064	123.1441	0.1688176
cg11326968	0.9023468	0.425417	1.913957	0.7888204
cg21578906	3.2500981	0.8437313	12.51955	0.0867101
cg11407507	0.478485	0.1835301	1.247468	0.1316309
cg13984434	1.21E-10	1.38E-17	0.00107	0.0051376
cg02129962	0.4996807	0.2272185	1.098858	0.0844369
cg26723928	4.9113204	1.1017503	21.89341	0.0368853
cg26559808	0.3790241	0.1728915	0.830922	0.0154161
cg06872257	2.6843905	0.7973172	9.037748	0.1108757
cg06111374	0.9495369	0.5133849	1.756227	0.8689155
cg15836711	3413973.4	0.0077809	1.5E+15	0.1384274
cg14683125	0.0090233	0.000281	0.289776	0.0078203
cg11433624	0.0350345	0.0038837	0.316043	0.0028231
cg23682940	0.1567328	0.0444752	0.552334	0.0039314
cg13347577	2.8881822	0.3411824	24.44908	0.3304377
cg23819092	0.3835059	0.1577886	0.932113	0.0344202
cg00434461	2.514681	0.5980681	10.57341	0.2082313
cg02529547	0.1132483	0.0230088	0.557402	0.0073897
cg27234490	4.6729227	1.1279979	19.35838	0.033499
cg09000832	2.5035727	0.8717996	7.189583	0.0881826
cg08695418	0.1517539	0.0526341	0.437534	0.0004831
cg00702417	3.5289964	1.2292027	10.13162	0.019105
cg21228760	2.0470458	0.8852753	4.733439	0.0939254
cg07497520	1.0886765	0.5387585	2.199903	0.8128706
cg00828721	2.3351878	0.9111574	5.984807	0.0773617
cg02609692	1.9519524	0.8438147	4.51535	0.1180332
cg17593958	3.1334577	1.0288338	9.543385	0.0444323
cg26453169	1.5370759	0.7646709	3.089698	0.2275228
cg17003260	0.9779353	0.4405492	2.17083	0.9562662
cg10745499	3.0885776	1.3034801	7.318341	0.0104035
cg04088969	0.0201851	0.0007149	0.569958	0.0220325
cg23681599	0.3220435	0.1487336	0.6973	0.0040444
cg14594696	3.1825388	0.9657048	10.48825	0.0570912
cg23612492	4.7207561	0.5878875	37.90783	0.1442442
cg15480200	0.3642081	0.1677643	0.790678	0.0106553
cg17009842	1.4606017	0.7680743	2.77754	0.2479688

cg22783058	0.2996511	0.0796868	1.126797	0.0745355
cg08390209	0.5903205	0.2518576	1.383632	0.225201
cg14706297	0.1846889	0.0558131	0.611146	0.0056666
cg27001893	0.1252739	0.0361467	0.434163	0.0010543
cg22533317	0.2785192	0.0461273	1.681715	0.1635138
cg17495715	3.0744211	0.9140992	10.34031	0.0695499
cg27262850	0.32569	0.1178084	0.900395	0.0306033
cg04801430	0.1544058	0.0477273	0.499529	0.0018168
cg11613003	2.5549672	0.9297044	7.021434	0.068965
cg21055684	0.8561802	0.4469075	1.64026	0.6397055
cg14185717	0.3200693	0.146024	0.701558	0.0044382
cg04411307	1.3654779	0.0678018	27.4997	0.8388759
cg03572388	53.53787	2.9501448	971.5806	0.0071131
cg24403093	0.271816	0.0537022	1.375807	0.1154022
cg05879527	3.1182712	0.5373203	18.0965	0.2049353
cg08870587	3.3531765	1.3751242	8.176565	0.007805
cg08644023	0.1468865	0.0289048	0.746439	0.0207477
cg07125274	2.3448225	0.3209544	17.13076	0.4009588
cg00463767	3.9381062	1.2726306	12.18632	0.0173943
cg11685186	2.6163065	0.0088192	776.1581	0.7405418
cg15836722	0.7915083	0.3935227	1.591993	0.5119577
cg07274406	2.0744462	0.9469897	4.544217	0.0681782
cg22592722	1.8466769	0.3137945	10.86767	0.497583
cg00142036	0.3782705	0.1609179	0.889203	0.0257976
cg18033335	0.1985946	0.0593712	0.664292	0.0086926
cg19183842	1.2812858	0.3758089	4.368426	0.6920477
cg06686146	5.6086796	1.4276915	22.03367	0.0135112
cg18573327	2.5712413	1.0298174	6.419858	0.0430835
cg15235798	1.2763371	0.6122727	2.660639	0.5150343
cg23411043	2.8922203	0.547579	15.27622	0.2110388
cg25703567	0.2750854	0.0429467	1.761998	0.1731514
cg02632229	0.0021556	2.07E-05	0.22475	0.0096095
cg05399703	0.3488779	0.1489956	0.816909	0.0152735
cg17221864	1.8187212	0.5527931	5.983698	0.3249216
cg08687025	0.2646805	0.0969286	0.722757	0.0095019
cg05457903	1.1209901	0.3883464	3.235819	0.8327567
cg19640821	2.8290747	0.8275725	9.671254	0.0972786
cg18305790	2.7886349	0.3710802	20.95635	0.3189555
cg00089823	1.7107935	0.7474959	3.915492	0.2037073
cg27041619	2.217642	0.4647077	10.58286	0.3178647
cg13345299	0.3693654	0.1015823	1.343057	0.1304951
cg15142214	22.273555	1.9517659	254.1858	0.0124787
cg14122403	0.5773616	0.2726913	1.222432	0.1512312
cg25151376	92871.581	7.89E-22	1.09E+31	0.7087907
cg24244500	2.0927479	1.0157308	4.311767	0.0452542
cg06702880	1.6935704	0.8562756	3.3496	0.130013
cg10734218	0.2684675	0.0861058	0.837049	0.0234184
cg01798157	1.4672283	0.6079191	3.541193	0.3937645
cg00779565	0.46923	0.1740962	1.264684	0.1347142
cg25037165	0.5339133	0.2479735	1.149572	0.1087733
cg22380139	2.1515216	0.7410841	6.246315	0.1588516
cg04040925	0.0409941	0.0044953	0.373834	0.0046195
cg03613003	1.3319266	0.3734132	4.750845	0.6586668
cg12079303	0.3179203	0.1250693	0.808138	0.0160624
cg20637609	2.3985172	0.9425939	6.103249	0.0663725
cg19784198	1.434176	0.6090176	3.377342	0.4092835
cg24155937	0.3172033	0.1206053	0.834274	0.0199542
cg01467266	1.4733564	0.6851995	3.168098	0.3211295

cg25285440	0.5364013	0.2696916	1.066872	0.075823
cg21206217	1.5191156	0.7021888	3.286455	0.288241
cg25011337	2.4412469	0.810993	7.348628	0.1124297
cg26232247	0.1139663	0.0225126	0.576934	0.0086734
cg16102247	0.2182236	0.039191	1.215115	0.0822876
cg04994217	3.4924929	0.7456294	16.35867	0.1124235
cg20295537	1.6023382	0.8216475	3.124804	0.1665107
cg07063911	0.2082384	0.0174361	2.486982	0.2149831
cg26866482	0.0763906	0.0108528	0.537699	0.0097908
cg04277193	9.3708776	0.0082023	10705.94	0.5333668
cg27179353	0.7144712	0.3368465	1.515435	0.3808226
cg04257841	1.842774	0.8303355	4.089691	0.1328768
cg09657354	1.6014942	0.7034432	3.646042	0.2618911
cg19082960	0.4458243	0.1919986	1.035212	0.0601829
cg22331740	0.414446	0.1689779	1.016496	0.054327
cg24973864	0.4511276	0.201218	1.011421	0.0533106
cg22494767	1.2107392	0.4890955	2.997143	0.6792421
cg22098375	8.1370804	0.3635824	182.1103	0.1861773
cg03976543	0.1616176	0.0456859	0.571735	0.0046949
cg14989202	3.7682023	1.3234717	10.72886	0.0129576
cg09230763	9.1855699	1.3208323	63.87995	0.0250144
cg20166931	2.42997	0.9152295	6.451666	0.0747225
cg23923856	0.5888365	0.2444632	1.418326	0.2376878
cg21249754	3.0271669	0.9321775	9.830466	0.0653148
cg14566801	0.409817	0.0156633	10.72252	0.5922419
cg02304879	1085.4319	12.451676	94618.78	0.0021676
cg20335206	2.426252	0.4113426	14.31094	0.3276353
cg14213581	0.5423612	0.2552103	1.152601	0.1116737
cg17306279	0.3875276	0.1142242	1.314762	0.1282818
cg04928693	0.0096421	2.62E-05	3.549719	0.123629
cg20930618	3.278E+11	7.74E-07	1.39E+29	0.2003938
cg27437806	101328595	2.9023357	3.54E+15	0.0375068
cg03074925	2.6825126	0.8196442	8.779266	0.1028503
cg21808287	0.7412628	0.3315928	1.657064	0.4657196
cg14945696	0.5864469	0.3003443	1.145086	0.1180193
cg04603130	0.4692921	0.2265253	0.972232	0.0417762
cg26982544	0.1922857	0.0039083	9.460305	0.4068353
cg18555698	0.5492643	0.2687481	1.12258	0.1004011
cg08090452	1.15E+21	6.58E-10	2.02E+51	0.1722765
cg01150433	1.8973208	0.8245217	4.365957	0.1320202
cg27316798	1.3410726	0.6954209	2.586168	0.3811011
cg10498502	0.2038218	0.0584806	0.710378	0.0125331
cg09214955	253.17486	7.3182054	8758.638	0.0022075
cg21487207	0.001703	1.69E-07	17.20356	0.1753582
cg06777516	2.7106639	0.9761404	7.527297	0.0556684
cg06998422	0.4827525	0.2051336	1.136089	0.095362
cg15724328	1.0044145	0.4673475	2.158669	0.9909969
cg09885951	1.87E-13	5.79E-31	60312.75	0.1542028
cg23571452	0.3850688	0.1727817	0.858181	0.0195952
cg20818265	4.7813526	0.5939656	38.48932	0.1414468
cg12072333	4.08E+36	3.01E-21	5.53E+93	0.209129
cg16185956	5.4080687	1.0239836	28.56218	0.0468253
cg25511335	0.7496908	0.381928	1.471576	0.4024609
cg10421194	0.1493335	0.050867	0.438407	0.0005389
cg17995050	3.9742539	1.250442	12.63129	0.0193465
cg25321556	4.6840497	1.0115481	21.68985	0.0483082
cg13818302	1.9825682	0.8515725	4.615669	0.1124404
cg08535779	0.1974521	0.0746769	0.52208	0.0010752

cg11530678	3.7583546	0.4067123	34.73027	0.2432142
cg12929027	1.4730908	0.5533166	3.921799	0.4381305
cg06940168	0.0206281	0.0035368	0.120312	1.61E-05
cg00451039	267562.6	7.36E-26	9.72E+35	0.7277766
cg27009439	0.4904723	0.2055134	1.170547	0.1084609
cg15268691	3.7599876	0.8640749	16.36144	0.0775233
cg06884470	2.6417226	1.0370659	6.729272	0.0417249
cg14185860	0.523968	0.2507416	1.094922	0.0856498
cg04265964	0.5619481	0.2521087	1.252577	0.1587488
cg20010135	0.5913636	0.2556072	1.368158	0.2196335
cg24310460	7.02E+25	16.573468	2.97E+50	0.039685
cg18196334	2.2410232	0.8296394	6.053455	0.1114766
cg23969311	2.4042301	0.6226755	9.283041	0.2031317
cg23549902	1.0542551	0.5349869	2.077535	0.8786697
cg08346922	2.8470786	0.8550424	9.480064	0.0882321
cg12120741	0.2484336	0.0883046	0.698936	0.008323
cg18791730	0.1903238	0.0327044	1.107593	0.0648569
cg09690632	0.3522494	0.1330803	0.932367	0.0356436
cg00110832	0.2784788	0.0975193	0.795232	0.0169432
cg00180225	0.002277	6.22E-09	834.0932	0.3518973
cg12769506	0.0001339	2.20E-07	0.081447	0.0063968
cg18081863	0.2341817	0.0944092	0.580887	0.0017368
cg08656504	122579.97	8.45E-06	1.78E+15	0.3263803
cg21005830	0.3454108	0.1393703	0.856055	0.021699
cg19875216	5.045E+16	3.0001809	8.48E+32	0.0436329
cg01170591	0.3601705	0.131612	0.985646	0.0467992
cg00656991	0.0159951	0.0008809	0.290441	0.005177
cg24550560	2.4879287	0.7260454	8.525347	0.1469227
cg20116935	1.7810941	0.5353823	5.925292	0.3465932
cg27406975	1.8126244	0.66867	4.913645	0.2424177
cg05834353	31.863059	0.8008464	1267.727	0.0655052
cg05079465	0.444834	0.2140432	0.924473	0.0299787
cg14123992	0.2942557	0.1290851	0.67077	0.0036162
cg24162330	1.14E-07	2.75E-11	0.000475	0.0001699
cg25371672	0.3541498	0.1502487	0.834763	0.0176534
cg07570898	3.8871375	0.3957888	38.17652	0.244109
cg04962865	0.4926622	0.2585147	0.938887	0.031427
cg22652275	3.546219	0.2921549	43.04453	0.3202798
cg10097458	3.3116153	1.1878211	9.2327	0.0220802
cg09477019	2.0284629	0.4205451	9.784116	0.378317
cg09122158	5.101E+10	0.0590643	4.41E+22	0.0787111
cg22716738	0.2590162	0.1058319	0.633924	0.0030951
cg14967066	2.9932142	1.5405075	5.815831	0.0012164
cg02319990	0.2671231	0.1117205	0.63869	0.0029973
cg24182333	34279.287	11.17073	1.05E+08	0.0108009
cg25070637	1.2880745	0.3672883	4.517257	0.6925294
cg13656878	1.1600624	0.5429946	2.478376	0.701469
cg07744618	0.6288542	0.2591678	1.525875	0.3050672
cg13002809	2.6437325	0.6308861	11.07858	0.1835625
cg10759651	0.3122792	0.1205853	0.808708	0.0165168
cg06679209	6.1164477	0.4523999	82.69439	0.1728856
cg10334385	0.0757553	0.001247	4.602073	0.2181611
cg19679327	0.5536159	0.2593651	1.181696	0.1264101
cg10902144	2.4494264	0.9389622	6.389703	0.0670669
cg18764814	0.4110856	0.1640237	1.030287	0.0579183
cg09447007	1.191E+13	0.0005897	2.41E+29	0.1160006
cg09416154	1.1702376	0.5091843	2.689509	0.7111824
cg06620390	0.8015343	0.3764874	1.706451	0.5660942

cg04599342	7.0936031	1.0708439	46.99024	0.0422634
cg18883951	2.4455046	0.5894049	10.14666	0.2180289
cg26101086	187.75909	0.0339697	1037793	0.2337746
cg21477985	1.5451835	0.722268	3.305687	0.2620967
cg01537665	2.68E-11	7.50E-23	9.606051	0.0729274
cg21282452	0.0371336	0.0008192	1.683207	0.0905739
cg25752087	0.3814037	0.1348482	1.078759	0.0692093
cg01362243	4.9627173	1.3522994	18.21236	0.015738
cg03950669	3.726E+19	0.0003601	3.86E+42	0.0955731
cg08437576	0.4208338	0.2126837	0.832697	0.0129264
cg01141636	0.4669889	0.21688	1.005527	0.0516692
cg05533615	0.0812938	0.0142308	0.464392	0.0047631
cg18442685	1.9780265	0.6987843	5.599137	0.1988488
cg24480735	0.4207265	0.1926674	0.918738	0.0298063
cg26514552	1.8890324	0.547523	6.517431	0.314098
cg24163987	11.638316	1.663197	81.43979	0.0134182
cg14161821	0.3900578	0.1856236	0.819643	0.0129585
cg04004158	0.5300657	0.2176927	1.290671	0.1621156
cg12456825	0.4255128	0.1834341	0.987064	0.0465587
cg20725334	0.9779335	0.0782189	12.22664	0.9861862
cg18216587	0.573024	0.2421821	1.355825	0.2050831
cg07401745	0.0394099	0.0050247	0.309098	0.0020893
cg23536473	1.6160564	0.8207444	3.182036	0.1649817
cg16499607	0.1945082	0.0742821	0.509322	0.0008571
cg00989365	2.211753	0.9269072	5.277606	0.0736296
cg02117102	4.8746623	0.8704655	27.29842	0.0715238
cg05512327	2.7870863	0.8219676	9.450311	0.0999145
cg25011475	0.4678156	0.0381236	5.740573	0.5526063
cg03403168	0.3538181	0.1476164	0.848058	0.0198339
cg18310707	11.938807	5.35E-11	2.67E+12	0.8524509
cg09705592	0.7265077	0.2543742	2.074949	0.5506962
cg15984406	1.5636484	0.8078007	3.026732	0.184652
cg18229178	0.5659463	0.2997937	1.068385	0.0791015
cg17277896	0.2997138	0.1371607	0.654914	0.0025176
cg04603976	0.2221592	0.0462568	1.066972	0.0602451
cg12258811	0.1021444	0.0219596	0.475122	0.0036279
cg20659941	0.6902026	0.3358998	1.418219	0.3129464
cg22301854	1.0914595	0.5444226	2.18816	0.8052107
cg02848875	0.2759517	0.101488	0.750329	0.0116428
cg25786525	0.0999625	0.0097388	1.026047	0.052584
cg06419732	0.4512001	0.195952	1.038936	0.0614558
cg07522913	0.2764144	0.1294951	0.590022	0.0008883
cg23258897	3.67E-19	1.98E-43	681074.8	0.1365241
cg09019410	4.6241954	2.072135	10.3194	0.0001848
cg05294577	2.632865	1.1616453	5.967379	0.0204017
cg24950003	0.1582716	0.0329184	0.76097	0.0213965
cg19942256	2.4652827	0.7956356	7.638696	0.1178739
cg26449680	0.1690613	0.0360812	0.792149	0.0240926
cg17213154	0.2366376	0.0842735	0.664472	0.0062203
cg20450689	1.5235023	0.710588	3.266393	0.2792791
cg24246472	0.481637	0.1571328	1.476294	0.2011257
cg21821095	4.1742582	0.8540477	20.40217	0.07755
cg11027330	0.19528	0.066703	0.571702	0.0028809
cg21931938	1.1929208	0.5469982	2.601581	0.6574564
cg27260617	1.2473181	0.6582845	2.363419	0.4979458
cg16658582	2.1445428	0.8456286	5.438633	0.108094
cg04008913	0.2706631	0.0790489	0.926749	0.0374245
cg17608570	0.1750786	0.0491478	0.623681	0.0071808

cg23875509	0.4308289	0.20652	0.898768	0.0248033
cg18016994	0.1058436	0.012244	0.914972	0.0412796
cg11638399	0.3767862	0.1426521	0.995203	0.0488766
cg01134967	1.9018308	0.8267388	4.374974	0.1304496
cg21075593	9.8574129	1.1163829	87.03877	0.0394913
cg02980604	0.292918	0.1057087	0.811673	0.018215
cg25131247	33.751187	1.9788326	575.664	0.0150339
cg02196379	126414.28	0.0138677	1.15E+12	0.1507949
cg27643182	0.3906821	0.1482457	1.029591	0.0573046
cg05960502	4.8751891	0.6418948	37.02704	0.125671
cg04706627	1.3216001	0.514427	3.395286	0.5624389
cg15068428	11.283507	0.098641	1290.716	0.3162851
cg17852224	3.2742006	0.8934274	11.99917	0.0734695
cg24679890	0.0952887	0.0175158	0.518384	0.0065233
cg18915156	0.690279	0.3299573	1.444081	0.3250111
cg07416656	2.8535174	1.1118888	7.32318	0.0292188
cg02225786	0.6070517	0.2631385	1.400448	0.2418772
cg04945652	0.9847719	0.3722487	2.605182	0.9753369
cg11356247	0.120764	0.0317332	0.45958	0.0019346
cg19565738	0.3671367	0.1740956	0.774226	0.0084846
cg08861404	2.4493258	1.1088504	5.410285	0.0267256
cg05580603	1.6295843	0.6359371	4.175798	0.3090917
cg25029689	0.5103055	0.2548505	1.021821	0.0575607
cg04459091	0.5278382	0.2535486	1.098855	0.0876401
cg23446109	1.0967968	0.2645616	4.547007	0.8986703
cg05210497	0.1671541	0.0581441	0.480539	0.0008997
cg24710020	0.1826556	0.0612278	0.544901	0.0022983
cg14051336	3.1001761	0.5355087	17.94759	0.2066312
cg00648573	0.1679556	0.0009573	29.46666	0.4986009
cg25303150	0.0953898	0.0270592	0.33627	0.0002569
cg27473871	0.4368206	0.171302	1.113894	0.0828957
cg11906361	6.4004704	0.6148473	66.62796	0.1204103
cg26192309	0.4495423	0.130642	1.546886	0.2047733
cg23638358	1.7003037	0.3041818	9.504292	0.5454896
cg24337173	0.7879897	0.3912971	1.586845	0.5046907
cg03853793	2.278E+11	1.44E-06	3.59E+28	0.1955403
cg22858630	9.3884493	0.0768642	1146.736	0.3610065
cg08198176	1.366582	0.6760612	2.762393	0.384433
cg24294399	596.88105	0.5227892	681473.6	0.0751734
cg03895540	0.5584849	0.2970293	1.050083	0.0705652
cg19915582	2.0375276	0.712411	5.827421	0.1843457
cg07493596	2.62E+21	1.89E-12	3.64E+54	0.2052977
cg20312962	0.3428159	0.1448227	0.811494	0.0148888
cg12377113	0.8975714	0.4678159	1.722119	0.7451543
cg17690515	0.0073699	0.0002896	0.187572	0.0029454
cg16750275	2.896004	0.9665686	8.67692	0.0575341
cg14846380	0.3067802	0.1211912	0.776575	0.0126463
cg21328643	4.7304504	0.399383	56.02932	0.2178737
cg23254346	7.26E-06	1.22E-09	0.043176	0.0076165
cg01160692	1.7628322	0.6595564	4.711617	0.2583766
cg24187758	1.2221382	0.5113627	2.920866	0.6518025
cg21579147	1.2269511	0.6066799	2.481389	0.5692241
cg25509872	0.0114575	0.000409	0.320947	0.0085802
cg07313319	0.219666	0.056199	0.858612	0.0293219
cg19474047	0.2966376	0.1333883	0.659682	0.0028816
cg21517147	54064.606	4.01E-35	7.29E+43	0.8126053
cg11326758	9.935839	1.0473117	94.26124	0.0454751
cg02684104	0.9444279	0.4129412	2.159978	0.8922479

cg14417861	1.49E-08	8.72E-18	25.37691	0.0965538
cg07959070	0.2914212	0.1237597	0.686219	0.0047765
cg14577706	0.1638661	0.0636934	0.421584	0.0001758
cg11205006	0.2877109	0.0590476	1.401879	0.1231058
cg03874229	0.0114822	0.0003693	0.357006	0.0108549
cg22807585	0.1353002	0.0322474	0.567678	0.0062607
cg06997937	2.5319131	0.8947404	7.164742	0.0800498
cg13520531	0.1601054	0.0222837	1.150334	0.0686426
cg11717507	1.6720778	0.8297178	3.369633	0.1504772
cg09597312	0.0176488	0.0016282	0.191301	0.0008997
cg23183601	1.4976393	0.5514937	4.066997	0.428133
cg16698470	0.6202772	0.309253	1.244107	0.1786588
cg02333730	0.3704955	0.1424693	0.963484	0.0417251
cg05275595	0.2695771	0.1009414	0.719941	0.0089077
cg16329842	0.3607053	0.1692707	0.768641	0.0082506
cg05827546	1.5279305	0.5547025	4.208691	0.4122151
cg11737334	1.1058329	0.546034	2.239542	0.7799324
cg15341760	950.01117	0.4015837	2247405	0.0836672
cg16218705	5.218448	0.1039534	261.9655	0.408279
cg10265690	3.2197871	1.4863553	6.974799	0.003028
cg08832414	2.0550113	0.7656524	5.515651	0.1527535
cg09722315	1.0701926	0.4008677	2.857083	0.8922928
cg01564661	0.3444425	0.1312341	0.904038	0.0303973
cg21410954	2.5600579	0.5229954	12.53146	0.2460236
cg03168749	0.9095191	0.4273516	1.935701	0.8056048
cg08631890	2063.6448	7.3596792	578643.4	0.0079528
cg04187935	0.6450786	0.149899	2.776046	0.556035
cg05168015	0.3997644	0.1879472	0.8503	0.0172609
cg11828251	2.1660186	0.3403977	13.78281	0.4130168
cg06759971	2.8292042	0.7072984	11.31686	0.141465
cg18286474	0.6596967	0.3434325	1.267206	0.2116863
cg16744320	0.218853	0.003619	13.2348	0.4678865
cg21869027	166.39714	2.9437631	9405.652	0.0129751
cg05933789	1.3135751	0.4550489	3.791855	0.614067
cg13026550	1.5995881	0.0524305	48.80138	0.7876505
cg19627869	0.0158648	0.0003246	0.775322	0.036779
cg14431165	1.8444641	0.5837778	5.827642	0.2969579
cg20326928	0.0694871	0.0132148	0.365384	0.0016392
cg00876272	1.0285668	0.0004253	2487.606	0.9943464
cg16002441	0.2200114	0.0088075	5.495892	0.3564528
cg27259408	0.2095387	0.0567412	0.773802	0.0190429
cg03016153	3.346001	0.3460939	32.3488	0.2967835
cg00592007	0.0522855	0.007184	0.380533	0.003568
cg20360395	0.7645153	0.3787486	1.543197	0.4536841
cg10800953	2.5365473	0.6568845	9.79483	0.1769155
cg19962052	3.4576312	1.0048407	11.89762	0.0491114
cg12685539	1.2743615	0.6983784	2.325383	0.4294823
cg26867575	2.1236516	0.9899651	4.555611	0.0531053
cg04781339	0.2941157	0.1222344	0.70769	0.0062998
cg10443148	0.2658623	0.0808661	0.874072	0.0291385
cg20254225	2.9034785	1.0816746	7.793645	0.0343614
cg16189596	0.0509399	2.31E-12	1.12E+09	0.8064538
cg14541011	0.156511	0.0558832	0.438338	0.0004162
cg12815918	1.38294	0.4074308	4.694105	0.6030901
cg23081527	7.5176028	0.6628989	85.25335	0.1034961
cg26911140	8.22E-08	8.26E-17	81.84343	0.1227657
cg24136431	2.6138404	1.1530869	5.925105	0.0213857
cg04379095	0.8941434	0.4311111	1.854493	0.7637081

cg05764403	7.91E+36	1000.2835	6.26E+70	0.0328896
cg14725952	0.9310376	0.4695106	1.846244	0.8379082
cg23520458	0.1504741	0.0245192	0.923457	0.0407577
cg16344968	7.60E-11	6.99E-22	8.269264	0.0723309
cg09604283	2.3827926	0.7954454	7.137762	0.1208708
cg04095826	2.8570584	0.8622531	9.466805	0.085889
cg09419352	1.3962275	0.6253541	3.117356	0.4153803
cg02538337	3.8338135	1.4922699	9.849509	0.005247
cg24459563	1.0180274	0.4664987	2.221614	0.9642075
cg10251070	0.0849508	0.0252243	0.286099	6.89E-05
cg13918518	1.4904241	0.7002882	3.172071	0.3004314
cg18046464	1.4762215	0.7762545	2.807365	0.2349692
cg01476969	0.8621258	0.3536394	2.101748	0.7442015
cg09881253	1.2741409	0.6232103	2.604955	0.5066997
cg20900050	0.1375116	0.0492953	0.383595	0.0001503
cg13599020	0.3679592	0.1055883	1.282282	0.116506
cg00789427	0.4017534	0.1553113	1.039241	0.060028
cg18688305	6.83E-31	2.77E-53	1.69E-08	0.0082844
cg16680159	0.2577796	0.0791145	0.839926	0.0244863
cg10855978	0.3687702	0.0895441	1.51871	0.167171
cg18312113	0.4297982	0.1759627	1.049805	0.0638405
cg02464921	18.137585	0.7643793	430.3779	0.0728671
cg00934299	7.5430888	1.3026009	43.68045	0.0241341
cg24079702	0.217188	0.0579292	0.81428	0.0235332
cg01817965	0.5697214	0.280551	1.156946	0.1195627
cg05822356	2.4776536	1.0612163	5.784652	0.0359664
cg14217558	1.2892842	0.5922928	2.806473	0.5220195
cg19576304	1.419264	0.6508194	3.095037	0.3787505
cg05327967	4.1636823	0.8567772	20.23426	0.0770054
cg18648570	2.3907094	0.7418979	7.703879	0.1443159
cg16643151	1.447358	0.7893979	2.653725	0.2319334
cg21176127	6.8096862	0.3060615	151.5114	0.2255268
cg26127816	0.0954164	0.0042812	2.12656	0.1379292
cg26596964	1.6689714	0.8909752	3.126311	0.1097137
cg26898791	0.4263637	7.45E-09	24388781	0.9254757
cg09711214	2.8754789	0.6394469	12.93052	0.1685102
cg01643090	2.8555411	0.8600951	9.480481	0.0865655
cg23346801	0.66882	0.271859	1.645412	0.3811666
cg07871024	0.2835017	0.1029213	0.780919	0.0147569
cg21551191	3.96E-07	4.46E-13	0.352433	0.0349286
cg27390496	1.3892945	0.6780579	2.84657	0.3689802
cg01021184	0.5583638	0.1626424	1.916906	0.3544556
cg01116067	1.8019933	0.5842964	5.557419	0.3054413
cg20092736	1.4143169	0.5935808	3.369874	0.4339031
cg20219329	1.2630792	0.5711755	2.793133	0.5640749
cg00705004	0.6763001	0.3177975	1.439224	0.3100887
cg03286391	4.5510298	0.2015045	102.7861	0.3407113
cg20212775	4.8334039	0.7171136	32.57753	0.1055765
cg12702615	1.4018977	0.4935727	3.981819	0.5259006
cg19818016	0.0883733	0.0147785	0.528458	0.0078386
cg01550144	124.32306	0.1534829	100703.2	0.1581064
cg09057265	6.55E+32	1.498E+13	2.87E+52	0.0010575
cg03299095	0.4921696	0.2555042	0.94805	0.0340521
cg10444758	0.3986713	0.1355241	1.172771	0.094826
cg08889009	1.475115	0.4454325	4.885059	0.5245951
cg26588061	1.0895636	0.2588055	4.587031	0.9068943
cg25695610	1.2376853	0.6569297	2.331855	0.5093648
cg04009492	1.6580384	0.7844559	3.504456	0.1854387

cg00706441	0.2244833	0.0711481	0.708279	0.0108248
cg05483509	0.7200362	0.2995727	1.730639	0.462893
cg11075561	2.0171869	0.5828125	6.981735	0.267991
cg20378628	113.7622	1.3664078	9471.431	0.0358755
cg03616221	0.9240361	0.4549241	1.876891	0.8270268
cg03119215	2.7981869	0.7092488	11.03964	0.1417311
cg23057232	0.3782271	0.1660858	0.861336	0.0205882
cg03769927	0.4381646	0.1941142	0.989048	0.0469806
cg24126768	0.4495941	0.2007295	1.007001	0.0520154
cg21194785	0.0004263	2.91E-06	0.062425	0.0022867
cg10354764	18103428	1.26E-05	2.61E+19	0.2420075
cg12157364	0.4330212	0.1939765	0.96665	0.0410773
cg05165020	0.1145642	0.0145157	0.904191	0.0398294
cg22376712	2.52E+24	182108870	3.48E+40	0.0030465
cg01671575	1.7640814	0.7488575	4.155641	0.1941427
cg00875382	0.3660014	0.1777412	0.753664	0.0063844
cg01101997	1.7866782	0.4361237	7.319526	0.4198872
cg03447699	0.2824263	0.0970326	0.822039	0.0203694
cg06058262	8.8964698	0.770569	102.7126	0.0799201
cg06174606	0.0717235	0.0081231	0.633287	0.017738
cg05917165	2.7795482	0.954485	8.094301	0.0608553
cg09659734	1.1772733	0.5604293	2.473055	0.6665106
cg02245485	0.7107912	0.3289626	1.53581	0.3851467
cg24174959	0.5170705	0.1889939	1.414658	0.1989872
cg16354207	0.1201495	0.0073573	1.962122	0.1370203
cg24921210	23759.991	3.89E-12	1.45E+20	0.5869239
cg19873420	0.5689855	0.0176281	18.36528	0.7504026
cg27466532	0.2816085	0.0839534	0.944612	0.0401461
cg07029024	0.6605653	0.2980755	1.463879	0.3071011
cg22514764	0.7613547	0.0047548	121.9118	0.9161537
cg05835276	1.4476877	0.6793103	3.085188	0.3378912
cg20671068	0.0066274	2.47E-06	17.75749	0.2128982
cg04768368	1.7153459	0.4422071	6.653922	0.4352763
cg03847230	0.1359473	0.0047608	3.882067	0.2432744
cg11762306	1.9594278	0.6798114	5.64768	0.2129835
cg17251423	0.3645131	0.1449902	0.916405	0.0319083
cg14289826	0.1570942	0.0361885	0.681946	0.0134728
cg03078363	2.651186	1.0433889	6.736498	0.0404392
cg01681367	5.5426174	1.3122893	23.40993	0.0198224
cg21204492	0.284607	0.0824737	0.982145	0.0467594
cg16608652	0.3932828	0.1394109	1.109464	0.0777904
cg09062550	1.7605825	0.6584869	4.707233	0.2596182
cg27019645	0.2172551	0.0873201	0.540537	0.0010278
cg13654085	2.1256895	0.3227396	14.00063	0.4329913
cg13164537	1.7694634	0.5709515	5.48383	0.322741
cg16290520	0.0189069	0.0020934	0.170762	0.0004092
cg10987682	2.03836	0.8190062	5.073114	0.1258237
cg26258770	5.4908377	1.3016819	23.1618	0.0203968
cg04717579	2.473E+16	511693.18	1.19E+27	0.0026362
cg18234542	0.5891932	0.20749	1.673087	0.3204964
cg13237740	0.4573777	0.2056611	1.01718	0.0550864
cg20299414	0.3395098	0.1601364	0.719804	0.0048405
cg04260676	1.4579431	0.1281827	16.58257	0.7611794
cg14072140	0.5140536	0.2709404	0.975311	0.0417032
cg18440230	1.7457584	0.6747658	4.516637	0.2506186
cg24913794	2.6979901	1.4632953	4.974492	0.0014752
cg13396943	0.8713313	0.4497211	1.688198	0.6831597
cg14850026	6.1914399	0.8418317	45.53633	0.0733187

cg01205831	0.2469773	0.0645051	0.945628	0.0411932
cg11974346	1.0802401	0.5735735	2.034471	0.8111337
cg17695462	0.0726955	0.0143633	0.367926	0.0015324
cg02187214	0.9226464	0.3119261	2.729096	0.8843137
cg14462557	0.080553	0.015525	0.417957	0.0027136
cg14508998	3.4517767	1.5076634	7.9028	0.0033742
cg23847109	1.1956533	0.6368196	2.244885	0.5782405
cg17939889	0.5039062	0.2675186	0.949173	0.0338857
cg15758138	0.311364	0.0847919	1.143359	0.0787305
cg27168573	0.3648541	0.1511695	0.880591	0.0249078
cg27060812	43.307363	1.3403342	1399.299	0.0335736
cg06654118	0.3167664	0.0998558	1.004858	0.0509696
cg01176715	0.2758355	0.1190241	0.639242	0.0026694
cg05225996	0.1695962	0.0402286	0.714985	0.0156505
cg17665164	18.627483	6.45E-41	5.38E+42	0.952121
cg16460699	10413077	2.06E-10	5.26E+23	0.4102667
cg13777609	0.271613	0.087862	0.839653	0.0236069
cg09029085	0.2383446	0.0582047	0.976006	0.0461816
cg05243070	0.9667267	0.4978799	1.87708	0.9203821
cg01482860	1.9808837	0.4165448	9.420115	0.3902424
cg19392754	1.8057502	0.880695	3.702455	0.1067065
cg03422911	0.4155665	0.1579915	1.093068	0.0751388
cg09673245	3.19E-05	1.07E-11	95.17271	0.1734856
cg07628771	0.4286096	0.1580804	1.162106	0.0959615
cg27390271	0.2866913	0.0840613	0.977761	0.0459456
cg04392082	1.9810634	0.9341575	4.201232	0.0746864
cg08693738	1.1577388	0.4586945	2.922118	0.756509
cg08131499	0.5095799	0.1813912	1.431556	0.2008192
cg11102782	0.0106252	0.001057	0.106803	0.0001136
cg06795233	2.1540524	0.907996	5.110091	0.0816858
cg02206636	3.99E+35	3.976E+16	4.00E+54	0.0002405
cg13636583	2.0391108	1.0105767	4.114455	0.0466638
cg05813788	0.0062422	0.0001725	0.225885	0.005563
cg01696784	0.2398015	0.1030641	0.557951	0.000919
cg18041277	1.8550477	1.0160887	3.386714	0.0442271
cg00949661	0.1925222	0.0669764	0.553401	0.0022263
cg04724556	0.40102	0.1830563	0.878511	0.02239
cg03699843	2.9198179	1.0406968	8.191951	0.0417754
cg26151467	1.9906477	0.9087765	4.360454	0.0852756
cg02152128	1.0387793	0.3651441	2.955169	0.9431403
cg01095506	0.1430337	0.0257719	0.793835	0.0261479
cg25836061	0.380876	0.1443252	1.005137	0.051222
cg08530414	4.12E-07	4.52E-14	3.74657	0.0721126
cg19652416	0.226413	0.0587343	0.872792	0.0309594
cg08699640	0.0678656	0.0005371	8.57508	0.2758823
cg17604429	0.9848142	0.4729936	2.05047	0.9673788
cg10026473	0.1465188	0.0155601	1.379671	0.0932179
cg01070987	0.4786753	0.245422	0.933616	0.0306574
cg04743758	2.1980993	1.112187	4.34427	0.0234596
cg08489309	0.3292066	0.1168801	0.927249	0.0354722
cg25298189	0.3912973	0.1674238	0.914527	0.0302926
cg10523319	19.776099	0.3447207	1134.525	0.1486007
cg04600000	0.2867246	0.0817586	1.005533	0.051016
cg15848364	0.2947976	0.131204	0.66237	0.0031036
cg15099397	0.4876879	0.2033223	1.169766	0.1076856
cg25120425	0.4507865	0.1837414	1.105948	0.0818521
cg03417884	1.6239678	0.8322032	3.169023	0.1551771
cg25190126	0.2659226	0.0663724	1.065425	0.0614186

cg00369126	7.923E+09	2.54E-06	2.47E+25	0.2104782
cg19931975	0.7863877	0.0350417	17.6477	0.8796607
cg14022542	1.963995	0.7608191	5.069899	0.1630148
cg12980830	8.4253245	0.1394027	509.2159	0.3084813
cg10113191	0.3339013	0.117397	0.949684	0.0397088
cg04944682	1.1640787	0.3782148	3.58283	0.7911067
cg01928068	0.3750281	0.1246848	1.128013	0.0808856
cg19029859	1.6809129	0.6406848	4.410075	0.2912942
cg08076437	5.3796092	1.4454139	20.02208	0.0120946
cg01144086	2.2655086	0.9839674	5.216158	0.0546084
cg06964475	2.767063	0.7574504	10.10843	0.1236307
cg06708634	2.0794113	0.69006	6.266052	0.1933277
cg25311623	0.7783834	0.2214684	2.735744	0.6960452
cg08742424	0.43749	0.1785294	1.072078	0.0706422
cg12234426	1.78E-05	4.42E-40	7.13E+29	0.7878657
cg00055073	3.5903506	0.8535424	15.10249	0.0811741
cg03368046	0.2644841	0.1104641	0.633254	0.0028302
cg00683665	14105.456	9.673903	20567074	0.0101539
cg16466065	57.073474	0.4903977	6642.326	0.095638
cg10844884	0.009247	0.0001531	0.558682	0.0252093
cg12829360	1.0828639	0.548971	2.135986	0.8183343
cg19283806	1.7060966	0.9243378	3.149028	0.0875699
cg23099587	0.1115995	0.002364	5.268281	0.2648427
cg03391684	0.0696733	0.0092765	0.523297	0.0096125
cg02862835	2.31E-19	1.67E-47	3.2E+09	0.1943114
cg22785136	0.5317978	0.2572947	1.099163	0.0882454
cg24515283	6.37E-09	1.66E-28	2.45E+11	0.4121163
cg06975048	0.1726008	0.0541797	0.549856	0.0029617
cg18724430	1.9350974	0.9007184	4.157351	0.0906513
cg26076854	4.5933146	0.2875385	73.3764	0.2808685
cg25679431	0.4573397	0.210701	0.992685	0.0478686
cg23273041	0.4394574	0.2200432	0.877659	0.0198206
cg11225330	21.89302	0.2703779	1772.72	0.1686461
cg00055811	0.4179196	0.1728871	1.010236	0.0527026
cg00259886	0.298902	0.1031202	0.866391	0.0261419
cg21526357	1.2214405	0.4064454	3.670645	0.7216136
cg15644727	0.7260522	0.3416034	1.543169	0.4053001
cg03379552	2.1170301	1.0130546	4.424062	0.0461024
cg17759535	1.458774	0.7462244	2.851718	0.2695697
cg04793096	1.4500222	0.53102	3.959483	0.4684559
cg21171115	0.3295501	0.1586465	0.684561	0.0029202
cg06598091	0.1461452	0.0217662	0.981266	0.0477677
cg03940662	0.0824847	0.0191064	0.356096	0.0008268
cg04831011	0.8618447	0.4660345	1.593823	0.6355169
cg25437411	3.0740563	1.3852297	6.821845	0.0057591
cg20895058	3.2306767	1.2410709	8.409891	0.0162871
cg00468846	2.4773619	0.816901	7.512932	0.1090045
cg14037036	1.11E-22	8.00E-39	1.55E-06	0.0076948
cg11677621	2.3480642	0.3105277	17.75495	0.4082577
cg24110540	0.5237235	0.2016354	1.360308	0.1841407
cg05478779	2.108597	0.7669379	5.797316	0.1482511
cg05237436	0.192094	0.0313056	1.178708	0.0746949
cg08912922	3.5485616	0.9323855	13.50545	0.0632684
cg26550874	2.4425912	1.1946231	4.994254	0.0143939
cg11982577	1.0895331	0.3967876	2.991733	0.8678545
cg02823293	10.37329	1.18E-05	9116984	0.7376325
cg07469700	2.1583119	0.9776759	4.764678	0.056899
cg17228231	7.3926443	0.0154327	3541.265	0.5252365

cg23068717	0.5561481	0.0114178	27.08946	0.7672821
cg25900943	0.3228541	0.1141452	0.913177	0.0330746
cg09396865	45.464374	0.7585747	2724.859	0.0676011
cg12582654	2.4649534	1.04546	5.811791	0.0392504
cg03719128	1.6145993	0.7894162	3.302353	0.1894289
cg03602500	0.2977318	0.1118268	0.792692	0.0153103
cg00737978	0.4656121	0.1548738	1.399815	0.1734876
cg17176016	3.5851067	1.2101969	10.62058	0.021207
cg15129663	0.7090033	0.3283926	1.530746	0.3811655
cg11572080	0.6100427	0.3067067	1.213381	0.1589275
cg18200810	3.8604302	0.8375352	17.79378	0.0831735
cg19882512	1.007015	0.108715	9.327873	0.995089
cg02688927	0.2111403	0.0650135	0.685707	0.0096601
cg01781725	2.4514594	0.9176957	6.548634	0.0736731
cg21232023	1.5233378	0.7590378	3.057237	0.2363147
cg07436152	2.2053878	1.1113757	4.376319	0.023699
cg01953482	1.0258882	0.392336	2.682513	0.9584358
cg12207450	0.002447	5.37E-06	1.114641	0.0542026
cg07782925	7.82E-08	4.19E-47	1.46E+32	0.722825
cg20399842	27.922051	0.5934329	1313.781	0.0901902
cg21183461	1.3623175	0.5681251	3.266726	0.4883831
cg14613546	0.3221059	0.142609	0.727529	0.0064269
cg25655096	1.876086	0.6385406	5.512098	0.252535
cg07398105	1.9574603	0.6158379	6.221849	0.2549759
cg00011605	17.292627	1.4828504	201.6622	0.0229467
cg05684891	0.4053609	0.1069392	1.53655	0.1841234
cg03918306	2.267E+14	1.01E-17	5.10E+45	0.3694881
cg18994606	0.7411759	0.2980628	1.84304	0.5192894
cg07226484	1.0550271	0.4532078	2.456008	0.9011168
cg26349504	3.84E-05	2.59E-08	0.056825	0.0063362
cg01024458	1.4656324	0.0253711	84.66632	0.8534555
cg20633370	1.1334455	0.4857098	2.644993	0.7720315
cg15826897	2.2632233	1.1014878	4.650237	0.0262126
cg10519155	1.74E+27	3.13E-20	9.69E+73	0.2533774
cg16404250	0.5349656	0.1787753	1.600826	0.2633131
cg06092502	0.5175857	0.2415667	1.10899	0.0902864
cg04584700	0.2475359	0.0464498	1.319145	0.1019431
cg09108314	0.4626081	0.2171471	0.985536	0.0457469
cg14251870	2.0762033	1.060038	4.066477	0.0331752
cg03104083	1.9475607	0.9774993	3.880302	0.0580583
cg21429516	0.4279027	0.1748767	1.047027	0.0629838
cg06313241	3.1062606	0.4156442	23.21422	0.2693912
cg15508809	1.3920069	0.6990286	2.771966	0.3466447
cg06779961	0.1129257	0.0259643	0.491145	0.0036379
cg01523474	0.4352044	0.1819119	1.04118	0.0615822
cg04574034	2.4915225	1.1397599	5.446484	0.0221488
cg07011235	0.0010866	2.80E-07	4.217785	0.1055326
cg06878361	0.1643895	0.0586677	0.460627	0.0005936
cg06212607	332.20095	1.694588	65123.48	0.0310981
cg13772742	1.6217599	0.5970205	4.405385	0.3429692
cg11124080	2.4205456	1.0746149	5.452224	0.0328709
cg11101889	4.1483604	1.3873329	12.4043	0.0109035
cg16331565	0.2192083	0.0637949	0.753231	0.0159554
cg09924669	0.3748439	0.1269538	1.106765	0.075679
cg03001176	3.5252709	0.424986	29.24222	0.243115
cg11301670	1.966429	0.6483053	5.96454	0.2323061
cg25362709	0.7905262	0.405815	1.539942	0.4896199
cg15299835	0.0580401	0.0091054	0.369962	0.0025942

cg14713137	4.7855961	0.8981956	25.49771	0.0666275
cg06583576	1.3892686	0.7082108	2.725272	0.3388864
cg19509330	1.505277	0.5843921	3.877292	0.3968876
cg24301866	3.2425003	1.1607279	9.057944	0.0248112
cg20618842	7.09E-05	3.12E-09	1.615724	0.0620076
cg01678339	5.34E-08	6.08E-32	4.68E+16	0.5516152
cg05848640	2.2471356	0.7593848	6.649617	0.143547
cg09881855	4.30E-13	2.49E-26	7.416786	0.0670835
cg25642825	4.714E+12	3496.6919	6.35E+21	0.0065141
cg27273140	1.4992322	0.0001392	16149.1	0.9318763
cg16808455	0.0047343	1.38E-05	1.621093	0.0722213
cg22810875	0.0944785	0.0282887	0.315539	0.0001257
cg01123783	0.2993922	0.1146041	0.782134	0.0138353
cg10087754	0.2752589	0.1187202	0.638202	0.0026412
cg22065894	0.5225992	0.1697286	1.609098	0.2580695
cg25449440	2.4419399	0.8497009	7.017846	0.0974033
cg13435718	1.53381	0.6067117	3.87758	0.3660163
cg10334976	0.2971224	0.009955	8.868104	0.4836729
cg04146228	0.9824118	0.4716252	2.046398	0.9621991
cg08320303	0.2888396	0.0972192	0.858146	0.0253963
cg12018718	0.3115016	0.1168227	0.830602	0.0197595
cg03102500	4.1532788	0.7972287	21.63711	0.090863
cg07328519	0.2050435	0.0383451	1.096432	0.0639766
cg05597836	3.3763649	1.1076782	10.29165	0.0323706
cg12749246	2.7737477	0.9820072	7.834643	0.0541428
cg16334795	0.3286697	0.1589383	0.679658	0.0026847
cg06993512	0.1728143	0.0321726	0.928268	0.0406831
cg10789300	0.7171525	0.3464401	1.48455	0.3704639
cg27266250	3.5973192	0.6649967	19.4598	0.1371984
cg26288806	4.0901154	0.1083817	154.353	0.4470168
cg06489586	0.5154659	0.1963971	1.352898	0.17829
cg14344102	1.0111058	0.3937749	2.596242	0.9816863
cg15848493	6.3809314	1.4878501	27.36585	0.0126017
cg09801924	0.2010033	0.0557391	0.724847	0.0142188
cg05304531	0.4328023	0.2019961	0.927334	0.0312401
cg12934258	0.4063524	0.1934893	0.853392	0.0173723
cg08098176	3.22E-25	1.30E-45	7.98E-05	0.0185828
cg14794445	2.4216045	0.9642552	6.081552	0.0597697
cg10832108	1.7388562	0.7754907	3.898978	0.1793317
cg23517208	1.2918743	0.6064219	2.752109	0.5068852
cg26995656	0.5407703	0.2720747	1.074824	0.0794168
cg18582992	0.4469148	0.2351533	0.849373	0.0139608
cg16983084	0.2192081	0.0693094	0.6933	0.0097815
cg26169081	2.0155774	0.7207647	5.636447	0.1815885
cg20820107	0.0867429	0.0161265	0.466581	0.0043993
cg08994045	1.7174094	0.8175292	3.607816	0.1532927
cg00012148	1.0486855	0.5574446	1.972826	0.8827846
cg06159562	1.5973935	0.2774567	9.196629	0.5999799
cg19511664	1.24E-24	2.84E-48	0.54358	0.0474891
cg13080565	3.0677206	1.297583	7.252645	0.0106689
cg19924120	0.4093864	0.16495	1.016049	0.0541499
cg26420185	248.49424	6.7323428	9172.05	0.002738
cg08012672	0.3240519	0.1552198	0.676522	0.0026949
cg03020485	2.4924549	0.8230424	7.548009	0.1062077
cg16316624	1.6124925	0.7153312	3.634865	0.2492711
cg22467594	0.8867307	0.3255691	2.415129	0.8140903
cg15398976	0.5090139	0.1793822	1.444375	0.2044368
cg06080729	0.3203785	0.0716277	1.432998	0.1364205

cg24892628	0.8021451	0.3293901	1.953418	0.6273318
cg17448336	1.2173032	0.3315323	4.469632	0.766992
cg22445940	2.3814094	0.9133672	6.209015	0.0759589
cg03570232	1.6755269	0.6882567	4.078987	0.2555483
cg06796611	1.5918869	0.4616624	5.489085	0.4616445
cg04711324	0.1404345	0.0406461	0.48521	0.0019146
cg25265360	0.1979539	0.0610527	0.641835	0.0069589
cg24964110	0.4782715	0.2187374	1.045745	0.0646169
cg04207218	1.5258983	0.7786889	2.99011	0.2182555
cg14139311	0.3844592	0.0072099	20.50077	0.637517
cg21404028	22675878	79.986651	6.43E+12	0.0081928
cg04897892	0.8698307	0.0606265	12.47978	0.9182661
cg17339145	2.4242384	0.8458053	6.948327	0.0993009
cg05212349	1.6756589	0.9563283	2.936055	0.0712442
cg00928894	0.0797515	5.54E-06	1147.039	0.6046611
cg16477259	2.7245766	1.197889	6.197	0.0168199
cg05583681	0.0413291	0.0024606	0.694164	0.0268579
cg00779065	2.4547236	0.9019328	6.680839	0.0787621
cg08550394	1.5566724	0.3253736	7.447529	0.5794962
cg11182257	0.770606	0.3936972	1.508351	0.4469776
cg17988842	8.3716526	0.7531227	93.05863	0.0837685
cg16509531	0.288438	0.1097499	0.758055	0.0116749
cg26241806	1.6443078	0.7517207	3.596746	0.2130123
cg11096063	0.4958952	0.2295638	1.071215	0.0742777
cg09163998	17.77813	0.0476774	6629.182	0.3407828
cg20323436	0.6814125	0.2964616	1.566216	0.3663373
cg00822030	0.2831779	0.0883828	0.907301	0.0336943
cg07044282	0.5145038	0.2587968	1.022865	0.0580286
cg09757400	0.0048284	8.43E-06	2.765723	0.0997648
cg11007190	0.5521429	0.0577501	5.278982	0.6061162
cg00222625	0.0221751	0.0014584	0.337169	0.0060901
cg19074292	1.3183309	0.011248	154.5157	0.909474
cg03582285	4.3005763	0.8908102	20.76195	0.069367
cg08838752	0.2619563	0.0608513	1.127685	0.072079
cg20450318	0.7016981	0.3658407	1.345887	0.2864029
cg19726321	0.2888408	0.0773294	1.078879	0.0647406
cg25793931	1.162477	0.5341121	2.530092	0.7043734
cg22061561	3.92516	0.1425977	108.0444	0.4188407
cg08116724	4.66E-06	8.10E-10	0.02681	0.005448
cg22114489	1.0973718	0.4891294	2.461976	0.8216851
cg18395531	1.7722609	0.6892456	4.557024	0.2349845
cg00944001	0.5515638	0.2113996	1.439088	0.2239765
cg01347957	0.2082133	0.0410402	1.056348	0.0582507
cg09121543	0.9532277	0.3750451	2.422757	0.91983
cg26181907	1.2874406	0.5308712	3.122233	0.5761745
cg09173768	4.950367	1.3807673	17.7482	0.0140796
cg19541697	5.2133393	1.1183008	24.30375	0.0355253
cg16403102	2.3574336	1.0323878	5.383145	0.0417881
cg12062324	1.47E+10	1.34E-07	1.61E+27	0.2422194
cg15279476	0.0094454	0.0001962	0.454786	0.0183456
cg20437660	0.3254217	0.1055887	1.002942	0.0506009
cg06570224	3.6818783	0.8834424	15.34478	0.0734875
cg26064460	0.6064062	0.2583435	1.423409	0.2505615
cg02426414	0.5513131	0.2541194	1.196076	0.1318452
cg23557755	0.7946179	0.3782954	1.669112	0.5437822
cg19868934	1.8235088	0.2622891	12.67755	0.5436944
cg05341549	1.8174642	0.4351643	7.590642	0.4126961
cg04947764	1.6056546	0.777121	3.317536	0.2009231

cg22333214	1.2260142	0.6230834	2.412375	0.5551498
cg21690777	0.129359	0.0332289	0.50359	0.0031861
cg22622164	12.59053	0.7315245	216.7001	0.0810484
cg26149682	4.3306509	0.8402669	22.31974	0.0797837
cg19239692	0.6659963	0.2795292	1.586779	0.3588106
cg04594483	6.6379749	1.60452	27.46161	0.0089859
cg21987515	0.6469574	0.2911521	1.437578	0.2850762
cg16635874	0.2909684	0.0154053	5.49568	0.4102628
cg15402095	3.3890953	0.8025523	14.3118	0.0967757
cg03888064	0.3495988	0.1578321	0.774363	0.0095922
cg27402883	1.6009203	0.6984987	3.66922	0.2661259
cg09362300	2.750904	0.5820303	13.00185	0.201612
cg04854089	1.3421795	0.5352936	3.365341	0.5303405
cg16051685	0.3477794	0.1350638	0.895507	0.028621
cg09809672	5.2032588	1.7644483	15.34412	0.0027981
cg08735211	2.2812779	0.9181678	5.668058	0.0757156
cg23098018	4.2435627	1.0361793	17.37906	0.0444978
cg25551699	0.0035582	1.34E-06	9.47984	0.1611903
cg06126809	0.7048541	0.2243311	2.21467	0.5493186
cg19835606	8.00E-11	1.09E-21	5.868567	0.0685559
cg03803789	0.5294509	0.2689556	1.042247	0.065736
cg19028462	0.1684545	0.0770513	0.368286	8.09E-06
cg05018638	0.4256784	0.1988706	0.911155	0.0278364
cg06398643	1.5547867	0.0002395	10094.8	0.9215053
cg13772700	0.5262857	0.1671888	1.65667	0.2725766
cg10439456	2.3101164	0.8040966	6.636811	0.1199393
cg15956008	0.001466	1.31E-35	1.64E+29	0.8624097
cg23767994	5.6921082	1.159921	27.93302	0.0321331
cg12177010	0.2885608	0.0840972	0.990133	0.0481856
cg07009002	1.6275037	0.0010422	2541.402	0.8967117
cg06625244	0.1008618	0.029342	0.346708	0.0002712
cg07766743	0.7220303	0.3571632	1.459635	0.3644646
cg09664216	0.3011858	0.1469622	0.617253	0.0010461
cg05402265	1.15E-10	6.53E-22	20.33516	0.0832775
cg02737384	0.3248896	0.0950452	1.110559	0.073013
cg08276524	1.1055295	0.3530079	3.462233	0.8632455
cg23791482	1.1238694	0.5520638	2.287928	0.7474742
cg04929605	0.6264092	0.2389372	1.642225	0.3414995
cg16592150	1.97E+30	0.3439855	1.13E+61	0.0535534
cg04453552	7.2082646	0.2114782	245.6948	0.2726148
cg26253898	0.3623658	0.1610473	0.815344	0.0141532
cg13182816	3.5101421	0.6913154	17.82269	0.1298588
cg06618097	3.1695099	0.7457912	13.46998	0.1181363
cg00399374	1.9854375	0.985591	3.999592	0.0549405
cg18570553	2.3520753	0.9345242	5.919866	0.0693444
cg24586444	5.5883492	0.9668876	32.29915	0.0545624
cg25836094	1.0871696	0.492911	2.397873	0.8359394
cg00605523	0.2760803	0.0981656	0.776447	0.014705
cg10030684	0.2917307	0.1265179	0.672686	0.0038512
cg04231319	0.3152528	0.0732779	1.356266	0.1209911
cg09095080	0.1009176	0.0168135	0.605724	0.0121332
cg00183067	1.4122648	0.4615346	4.321435	0.5452139
cg08416194	1.4803391	0.6616331	3.312114	0.3397276
cg00347228	0.10525	0.0179565	0.616911	0.0125843
cg12175905	1.3985957	0.5823378	3.358996	0.4529959
cg22157015	439.747	6.90E-20	2.80E+24	0.8121978
cg18906079	0.4050654	0.1444005	1.13627	0.0859402
cg10639412	5024930.6	3.48E-05	7.25E+17	0.2392008

cg16475423	0.3282876	0.1377402	0.782435	0.0119498
cg07436701	1.9775067	0.4047792	9.660904	0.3995224
cg09605636	3.0589417	1.2041273	7.770876	0.0187498
cg00863286	0.3378139	0.0002424	470.7842	0.7689045
cg11462165	0.4105062	0.1769963	0.952084	0.0380458
cg06751243	4.2459717	1.3946731	12.92652	0.0109088
cg11562225	1.18E-07	7.48E-15	1.852606	0.0591552
cg18323589	0.7660622	0.4035812	1.45411	0.4150788
cg06388418	1.5710949	0.3544916	6.96304	0.5520246
cg14852355	7.250644	1.5499988	33.91734	0.0118457
cg06422883	1.4420541	0.7004305	2.968917	0.3204351
cg01413354	1.9465887	0.7791123	4.863494	0.1539528
cg00089821	0.0507282	0.0039967	0.643861	0.0214731
cg16444942	0.0014266	1.95E-05	0.104638	0.00279
cg00527552	2.2645571	0.9021483	5.684452	0.0817417
cg16415340	0.458044	0.1450849	1.446079	0.1831484
cg09561029	0.2360529	0.0385726	1.444573	0.118286
cg22479161	0.3564039	0.0801492	1.584841	0.1753793
cg11212451	0.2702078	0.1396307	0.522896	0.0001024
cg19137606	45.341625	2.8940356	710.3793	0.0065897
cg00811065	0.4838272	0.1597475	1.465367	0.1990963
cg19072957	0.2707282	0.1254688	0.584159	0.0008684
cg14384748	1.1942904	0.5434839	2.624419	0.6584836
cg05710676	0.3521767	0.160692	0.771839	0.0091374
cg27523780	0.4262161	0.1659828	1.094451	0.0763297
cg05526498	0.3614576	0.1688208	0.773907	0.0087979
cg27658068	2.7883028	1.1433134	6.800088	0.0241704
cg24113409	4.154038	1.5070399	11.45028	0.005909
cg03468967	0.4807077	0.2555339	0.904302	0.0230886
cg00851518	0.1772387	0.0699499	0.449086	0.0002647
cg01123449	16.638347	0.9580866	288.9453	0.0535369
cg02876297	1725.039	0.1544754	19263652	0.1170634
cg24735235	1.1356681	0.4516607	2.855555	0.7868293
cg04827692	1.8330017	0.6976575	4.815967	0.2188941
cg00108282	1.1108582	0.27881	4.425974	0.8815049
cg06344195	9.5276367	1.3510715	67.18805	0.0237043
cg26054395	2.93E+32	727583.18	1.18E+59	0.0167655
cg05576262	1.8321914	0.8355602	4.017575	0.1306594
cg26011222	2.3760967	0.9663508	5.842429	0.0593762
cg04784618	0.5570536	0.2644792	1.173282	0.1236852
cg00334863	2.0930803	0.8144182	5.379282	0.1250997
cg00290023	1.3181257	0.492607	3.527062	0.5823032
cg19453154	0.0391852	0.0033213	0.462311	0.0100914
cg11378740	0.1545931	0.0038455	6.214766	0.3218799
cg13528344	2.0897243	0.9450719	4.620757	0.0686941
cg07359633	3.258873	0.9469372	11.21537	0.0609995
cg18642835	3.9062301	0.9762071	15.63053	0.0541134
cg03148184	1.8975791	0.9278452	3.880826	0.0792921
cg13790727	4.0541867	0.0342918	479.311	0.5654025
cg16556111	18.505273	0.9970218	343.4681	0.0502344
cg25372357	2.4849809	1.0537295	5.860261	0.037569
cg02193283	1.4653191	0.2873914	7.471204	0.645729
cg14481604	0.5068726	0.227073	1.131442	0.0972088
cg24109612	0.0020133	3.86E-05	0.104991	0.0020898
cg21747160	0.1682811	0.0319069	0.887536	0.035677
cg10265016	1.8693342	0.5632698	6.203795	0.306722
cg26427534	1.9341829	0.6283685	5.953614	0.2501445
cg06688411	7.618E+16	187.96692	3.09E+31	0.0235075

cg11093356	2.68E-30	1.89E-58	0.037904	0.0395019
cg24376802	2.5263844	0.8851521	7.210759	0.0832773
cg03711129	2.2847084	0.8496306	6.14372	0.1016114
cg25653947	1.6580493	0.4518058	6.084754	0.4459087
cg01928136	1.2477947	0.2814717	5.53161	0.7707617
cg18154417	0.3229785	0.1300319	0.802227	0.0149047
cg18525616	0.6236812	0.3132125	1.241899	0.1791173
cg17900127	6.8176855	1.4516106	32.02018	0.0150087
cg19189965	1.0160632	2.59E-09	3.98E+08	0.9987405
cg14088196	1.300582	0.5838969	2.896939	0.5200946
cg19879075	1.5661473	0.7148925	3.43103	0.2622111
cg06148175	0.6414179	0.261972	1.570462	0.3310517
cg12060123	0.5421902	0.2202854	1.334497	0.1828433
cg00801514	5.0972385	0.1001902	259.3253	0.4165681
cg20348298	0.2883715	0.1196453	0.695039	0.0055977
cg12315363	1.4074819	0.0010617	1865.949	0.9257626
cg12988276	1.8637566	0.8899278	3.903225	0.0987861
cg15155672	101194.02	56.376223	1.82E+08	0.0025726
cg15524224	2.6714406	0.8006085	8.913964	0.1099869
cg25021051	0.4724984	0.1786253	1.249849	0.1308905
cg02310286	0.3951206	0.0870686	1.793071	0.2288683
cg20528350	2.4672817	0.3719963	16.36435	0.3494976
cg21699330	1.625773	0.7696236	3.434325	0.2027746
cg15500259	1.1494624	0.4338615	3.045359	0.7793191
cg05002732	5584951.2	0.0005036	6.19E+16	0.1880155
cg00107187	0.1847935	0.0300648	1.135836	0.0683808
cg05723219	1.2639915	0.2245354	7.11547	0.790452
cg24670566	0.8937264	0.0009653	827.4926	0.9742819
cg00376480	20.295239	0.6568881	627.0425	0.0854553
cg01012595	0.1347441	0.0185749	0.977448	0.0474204
cg23039580	1.0920481	0.5091638	2.342211	0.8210615
cg01864807	0.0049787	7.60E-20	3.26E+14	0.7883851
cg27457284	1.3666479	0.5741359	3.253108	0.4802328
cg11118615	0.3964804	0.1342616	1.170824	0.0940307
cg02672759	3.2432287	1.1586588	9.078196	0.0250665
cg21402832	1.9443188	0.9865028	3.832098	0.0547683
cg07754143	0.2320661	1.08E-07	498617.2	0.8443289
cg19945912	24.168039	0.621626	939.6231	0.0881189
cg11733272	1.562362	0.8038989	3.03642	0.188135
cg11216064	1.757462	1.0105103	3.056548	0.045826
cg09334277	1.7538041	0.9244515	3.327193	0.0855192
cg02218776	0.3058806	0.1222403	0.765401	0.011365
cg03365733	1.4462228	0.5065423	4.129093	0.4906391
cg24335155	0.3923319	0.1446705	1.063965	0.0660395
cg02985541	0.3537739	0.1089401	1.148851	0.0837978
cg19494811	0.5072241	0.2375616	1.082988	0.0794364
cg15893373	707.64415	0.2819005	1776372	0.1003953
cg10092257	3.5715605	1.209748	10.54438	0.0211838
cg08533364	4.8529389	0.1221791	192.7581	0.4004253
cg12710531	0.0023313	1.51E-05	0.359383	0.018369
cg13627197	8.0742195	2.1702106	30.03995	0.0018343
cg10037579	0.3954618	0.1747027	0.895178	0.0260397
cg03109701	0.3801137	0.1256174	1.15021	0.0868522
cg02212491	1.960954	0.3741607	10.27724	0.425567
cg27104604	0.7923452	0.3622905	1.732894	0.5599187
cg09411874	0.1228665	0.0408259	0.36977	0.0001917
cg26417874	0.0839204	0.0004837	14.56053	0.3462495
cg02993352	2.4321325	1.0918387	5.417712	0.0296315

cg16898493	483.37264	7.14E-11	3.27E+15	0.6817679
cg11334165	5.9496257	2.079589	17.02165	0.0008837
cg13380277	0.3464436	0.1275944	0.940662	0.037526
cg06421751	1.9538701	0.8286048	4.607273	0.1259196
cg07898084	2163360.2	5.86E-06	7.99E+17	0.2830771
cg02705573	0.1385518	0.0211267	0.908639	0.0394168
cg25163611	0.3889384	0.1756569	0.861185	0.019888
cg13545297	2.0263174	0.4633725	8.861039	0.348175
cg14345012	0.2296128	0.0833255	0.632724	0.004441
cg25693302	2.0182933	0.9101036	4.475873	0.0839601
cg22812684	1.0076835	0.5147521	1.972651	0.9821821
cg18183247	0.5593315	0.2953427	1.059284	0.0745531
cg16272981	2.9336084	0.791753	10.86962	0.1072815
cg23373626	4.755587	0.4536621	49.85122	0.1933718
cg17831662	349948827	0.0147185	8.32E+18	0.1065515
cg02534744	0.1791141	0.0566367	0.56645	0.0034171
cg19669199	0.965376	0.1654732	5.632036	0.9687639
cg06787912	0.4370037	0.2137879	0.893279	0.0232473
cg10145196	1.5769866	0.7386485	3.366807	0.2391428
cg24282259	2.8097206	0.7903972	9.988054	0.1103841
cg08654915	4.3833462	1.4244226	13.48878	0.0099715
cg16636226	0.4643339	0.2345348	0.919292	0.0277043
cg04157804	7.6114618	0.1678214	345.2144	0.297007
cg19774288	1.518435	0.4574387	5.040337	0.4950389
cg15865175	1.33E-19	1.15E-39	15.47127	0.0652168
cg23305408	1.0757966	0.4759583	2.431596	0.8606098
cg11344744	0.3855474	0.1395798	1.064959	0.0659805
cg12219434	0.479686	0.1836802	1.252713	0.1336326
cg01554231	3.3591583	0.5042964	22.37562	0.2104306
cg10810394	0.4067964	0.1993728	0.83002	0.0134359
cg14939082	0.3540869	0.1560378	0.803507	0.0130201
cg14860524	0.503413	0.1740469	1.456071	0.2053078
cg10606490	1.7327889	0.7961352	3.771416	0.1659286
cg13601472	0.5278264	0.0201804	13.80549	0.7012063
cg25101863	1.3100427	0.6701371	2.560986	0.4297504
cg06913219	0.1689892	0.0603365	0.473301	0.0007157
cg02376426	0.3891877	0.1251542	1.210244	0.1030371
cg23216498	0.0001553	6.36E-12	3791.725	0.3122668
cg21741010	0.3694712	0.1639844	0.832451	0.0162863
cg12504882	4.635262	1.2016173	17.88061	0.0259731
cg07876105	0.3298933	0.1263532	0.861313	0.0235204
cg04433909	0.7377774	0.1123285	4.845748	0.7514901
cg02995295	0.0043382	9.28E-06	2.02728	0.0828056
cg21769820	2.555464	0.6760646	9.659426	0.1666813
cg20424036	0.8660022	0.3970585	1.888789	0.7176522
cg16669650	1.5060376	0.6687533	3.391608	0.3228572
cg23047500	0.1399764	0.0232122	0.844098	0.0319655
cg01493783	7.0387277	0.7786847	63.62484	0.08234
cg04752299	1.5060602	0.3090551	7.339199	0.6123112
cg14373597	0.0226531	0.0018569	0.276361	0.003001
cg20671910	0.1983794	0.0692739	0.568099	0.0025838
cg15188268	0.7589946	0.3828023	1.504883	0.4297455
cg24857294	5.3803204	0.4383275	66.04159	0.1884142
cg20463033	0.5711727	0.2358605	1.383183	0.2145638
cg06376130	0.5099024	0.2178756	1.193343	0.120536
cg07873488	0.3225633	0.1243026	0.837047	0.0200418
cg20610915	1.3450667	0.6369255	2.840528	0.4370204
cg11286163	1170754.3	21.860594	6.27E+10	0.0118959

cg25806347	4.1307698	1.4970997	11.39754	0.0061583
cg06952297	0.9357751	0.294435	2.974085	0.9104153
cg13597544	0.5214451	0.2193365	1.23967	0.1405581
cg08450091	2.4984592	1.0198411	6.120854	0.0451838
cg02654940	1.4180944	0.5245692	3.833606	0.4911791
cg15551096	1.7354771	0.9274303	3.247555	0.08465
cg19016816	1.2535463	0.616776	2.547729	0.5323046
cg06496641	2.78337	0.8207946	9.438596	0.1003829
cg23816049	0.4811769	0.1572215	1.472643	0.1999257
cg07490283	1.7812816	0.4248422	7.468571	0.4298581
cg20343114	2.6173405	1.0228146	6.697667	0.0447476
cg02389180	0.5876004	0.2953928	1.168865	0.1296985
cg20424335	0.4283544	0.1693068	1.083757	0.0734332
cg06978270	0.3786849	0.1790704	0.800815	0.0110449
cg20737382	1.276069	0.7130778	2.283554	0.4116186
cg03102841	3.2232479	0.6017261	17.26587	0.1716949
cg11103390	0.1850799	0.0616572	0.555565	0.0026297
cg07265444	0.2534302	0.1161911	0.552769	0.0005609
cg16489360	0.2320869	0.0880879	0.611484	0.0031259
cg11705208	12.117595	1.1154345	131.6403	0.0403914
cg19637330	1.019386	0.4464736	2.327456	0.9636425
cg20391436	2.47981	0.896397	6.860194	0.0802397
cg11932158	0.2160849	0.069676	0.67014	0.0079755
cg00305193	2.6723662	0.7445885	9.591259	0.1316507
cg17532626	3.0584805	0.4151069	22.53468	0.2725933
cg03590668	6.14E-05	1.97E-08	0.190657	0.0180841
cg15694422	3.4318144	1.0758727	10.94679	0.0372025
cg15478515	3.0788494	1.3316987	7.118212	0.0085419
cg20042612	3.3758799	0.3993174	28.54012	0.2639557
cg27243180	79.834627	0.0003713	17164776	0.4844529
cg13063967	0.0691095	0.0030979	1.541731	0.0916613
cg20264732	2.4549318	0.7202989	8.36693	0.1511334
cg21285383	0.0653113	0.0021162	2.015659	0.1189073
cg13257422	0.4539454	0.1581024	1.303373	0.1422104
cg06336535	0.3697051	0.1198467	1.140472	0.0834037
cg20038493	1.7899021	0.8848289	3.620756	0.1053272
cg24451742	5.5242156	0.0154138	1979.841	0.5689861
cg15756319	2.8902788	1.077705	7.75139	0.0349757
cg11624060	1.1640059	0.5198805	2.606194	0.7119133
cg04071812	2.68E-12	1.19E-28	60306.55	0.1654471
cg23052830	0.9283951	0.453052	1.902469	0.8391582
cg05123447	16.394856	1.2495278	215.1143	0.0332067
cg03010792	1.8071414	0.4666977	6.997592	0.3916173
cg01207974	2.7737406	0.3033377	25.36328	0.3662586
cg26522340	0.5717466	0.2855256	1.144886	0.1145558
cg14210765	1.9868392	0.6387714	6.179879	0.2356969
cg24911827	3.5492307	1.0341275	12.18132	0.0440831
cg00902895	5.3243763	1.4030019	20.20595	0.0139874
cg14829060	120.68349	0.099379	146555.1	0.1859049
cg02576395	0.0337653	0.0046567	0.244831	0.000802
cg25329377	0.8671526	0.4684033	1.605355	0.6501069
cg12136950	0.2594354	0.0789651	0.85236	0.0262036
cg27395391	1.4427321	0.0640252	32.51025	0.8176057
cg18163342	1.0227275	0.3017088	3.466824	0.9712178
cg26745764	1.8176861	0.8575251	3.852928	0.119004
cg09702881	8.29E-05	1.15E-21	5.99E+12	0.6351504
cg01438685	0.2380561	0.0414643	1.366736	0.1074878
cg12499749	1.169E+10	2.32E-10	5.89E+29	0.3165623

cg04568121	3.2494263	0.9847601	10.72218	0.0530208
cg09709592	0.0973278	0.0210559	0.449884	0.0028582
cg15493780	2.6528665	0.9410694	7.478408	0.0650233
cg01641792	0.4772804	0.1799607	1.265812	0.1371987
cg03549705	1.685312	0.5261926	5.397788	0.3794876
cg06710176	761223.89	8.39E-05	6.91E+15	0.2470169
cg22783363	2.1576401	0.8976318	5.186326	0.0856848
cg03287339	0.1947261	0.0726382	0.522016	0.0011459
cg05429895	1.6510477	0.0721234	37.79574	0.753599
cg05229898	1.116E+14	1.33E-16	9.37E+43	0.3575314
cg22141237	0.0497149	0.001707	1.447917	0.0810192
cg12894711	0.1202485	0.0404824	0.357185	0.0001371
cg04852275	5.6241494	0.271531	116.4915	0.264044
cg06687102	13.483419	0.0018087	100513.3	0.5674373
cg09220326	0.4976586	0.2012033	1.230915	0.1309618
cg26038582	0.2639885	0.0902072	0.772553	0.015058
cg15955046	5.0561266	1.1643734	21.95551	0.0305343
cg20102878	2.9821709	1.0648614	8.351644	0.0375648
cg07911664	1.4896317	0.8496775	2.611582	0.1641407
cg02246992	0.5835252	0.2537276	1.341997	0.2049071
cg03542938	1.6833411	0.4453161	6.363204	0.4427275
cg10830649	3.723772	0.7831551	17.70592	0.0983905
cg06657888	0.0691464	0.0070883	0.674523	0.0215177
cg12034488	1.3733602	0.6334036	2.977751	0.4216978
cg19076258	0.6670148	0.3526213	1.261718	0.2130804
cg01568736	0.9058311	0.4632626	1.771199	0.7725202
cg09154615	3.60E-32	5.13E-64	2.532555	0.0529746
cg13930177	0.7228416	0.3055251	1.710171	0.4600903
cg00100948	2.6237392	0.8561507	8.040649	0.0913812
cg14153737	0.2094569	0.0425021	1.032236	0.0547347
cg20370839	2.77E-20	1.33E-36	0.000579	0.0188356
cg18582342	0.2815425	0.0883339	0.897347	0.0321048
cg02540026	0.92394	0.2614535	3.265074	0.9022481
cg24633390	0.2748576	0.0786498	0.960545	0.0430713
cg24908413	1.2657769	0.5028787	3.186039	0.6167776
cg05200628	1.7837265	0.9165023	3.471546	0.0885061
cg10525432	0.5028402	0.2421494	1.044183	0.0651833
cg22540233	0.2858355	5.22E-06	15640.3	0.8219934
cg06908202	0.337764	0.1079384	1.056941	0.0622069
cg07824907	6.0942261	1.0983786	33.81311	0.0387063
cg24171453	0.8924888	0.3534157	2.253823	0.8098279
cg25773515	0.2383964	0.0884131	0.64281	0.0046093
cg18334849	1.3363892	0.4917683	3.631662	0.5697002
cg26638003	3.38E-16	3.79E-51	3.01E+19	0.3856011
cg00076313	1.9724958	0.688618	5.65007	0.2058179
cg22931642	0.559967	0.1453829	2.156808	0.3993331
cg03562952	0.4010183	0.2009406	0.800314	0.0095481
cg22987457	0.0700184	0.0011572	4.236455	0.2039909
cg26617637	0.1574882	0.0566182	0.438066	0.0003982
cg15732218	5.781E+16	2.02E-08	1.66E+41	0.1791873
cg03249573	2.0914626	0.6556152	6.671926	0.2125208
cg13789015	0.4611752	0.1644757	1.293094	0.1412007
cg08893839	3.0096072	1.0194164	8.885217	0.0460669
cg04221833	2.2203973	0.0020553	2398.716	0.8228915
cg07190012	1.4682034	0.563967	3.822247	0.4314637
cg18100702	1.8454929	0.7522472	4.52756	0.1808272
cg13166635	9.7592832	0.0028342	33605.66	0.5835074
cg02297936	0.4473987	0.2052926	0.975026	0.0430117

cg24697925	1.109509	0.445077	2.765836	0.8235518
cg16340136	0.290876	0.0904779	0.935132	0.0382163
cg26417417	1.3088433	0.6042759	2.834915	0.4948987
cg07557342	0.7968747	0.2831051	2.243016	0.6671767
cg09258240	1.3744361	0.5053781	3.737943	0.5332534
cg17634326	694.61121	0.1090739	4423465	0.1431491
cg14741855	0.5956263	0.2583513	1.37321	0.2240657
cg05505872	4.23E-05	7.73E-11	23.19476	0.135271
cg14646893	0.7290408	0.3134181	1.695819	0.4631205
cg19306847	0.3137049	0.0556979	1.766868	0.1886656
cg03079395	1.9252903	1.0701455	3.463774	0.0287996
cg13088253	3.9317929	1.3948746	11.08271	0.0096142
cg10579718	0.561993	0.2205445	1.432075	0.2272492
cg15280185	3.0754941	0.8536441	11.08034	0.0857994
cg10074498	2.5723606	0.4415256	14.98676	0.2933633
cg24405470	1.1544755	0.3678226	3.623523	0.8055697
cg26728390	0.0857063	0.0110104	0.667148	0.0189487
cg23702610	2.1047813	0.7596009	5.832147	0.1523767
cg23047952	1.4631846	0.7938801	2.696766	0.2224415
cg10256726	0.5188106	0.2192791	1.227497	0.1353162
cg23665778	0.290635	0.0007989	105.7314	0.681271
cg03745114	1.4424891	0.299455	6.948538	0.6478564
cg19728345	1.3616013	0.6155712	3.011769	0.4460304
cg10256330	3317.8696	0.0137048	8.03E+08	0.1999413
cg07691531	0.1092665	0.0156093	0.764877	0.0257516
cg10124548	0.2471281	0.1155966	0.528323	0.0003111
cg00575665	1.8363419	0.7015942	4.806413	0.2156988
cg23723818	0.988406	0.5312088	1.839101	0.9706368
cg06718080	0.7083928	0.3716855	1.350121	0.2947801
cg10824802	1.4338262	0.5076143	4.050039	0.4964021
cg12357484	4.2962905	0.8015617	23.02769	0.0888027
cg21932416	2.1616164	0.5489286	8.512192	0.2703343
cg07820996	1.0961555	0.2203949	5.451837	0.9106859
cg13459035	0.2096034	0.0889409	0.493963	0.0003536
cg10202436	0.3192372	0.1278881	0.796887	0.0144285
cg08555552	1.5165643	0.7741191	2.971077	0.2248415
cg17188169	1.2357444	0.5776684	2.643496	0.5853567
cg09920605	1.4348938	0.6383404	3.225427	0.3822477
cg01418527	0.3285158	0.1390508	0.776138	0.0111584
cg00889363	1.4685308	0.5778162	3.732299	0.4194184
cg01580181	2.326928	0.7350267	7.366526	0.150893
cg14941600	4.9634351	1.122269	21.95168	0.0346837
cg06346307	0.3160132	0.0846768	1.179359	0.0864474
cg01143314	0.2762801	0.1051771	0.725735	0.0090399
cg16379513	0.5009937	0.1862143	1.347881	0.1710754
cg07897193	3.29E+23	155.80204	6.93E+44	0.0306574
cg00373455	1.3146734	0.5523403	3.129169	0.5363425
cg16402286	0.6133468	0.2588253	1.453468	0.2668012
cg06008873	0.4614726	0.2159545	0.98612	0.0459294
cg09533708	2.5793907	1.0064401	6.610683	0.0484577
cg08029920	20.677688	0.8947844	477.8434	0.058681
cg00726615	2.0213588	0.8975381	4.552332	0.0893198
cg00922378	1.2262748	0.5365058	2.802859	0.6286499
cg14536184	0.1644755	0.0129808	2.084019	0.1635615
cg19904425	0.5191298	0.2036283	1.323469	0.1697448
cg25662414	1.6443932	0.6482062	4.171557	0.2950207
cg11210160	0.4205587	0.1319476	1.340454	0.1430465
cg02341556	0.0959443	0.0209138	0.440155	0.0025631

cg14544831	0.27957	0.0693795	1.126549	0.0730711
cg07484849	0.3138974	0.1368842	0.719817	0.0062123
cg19638968	0.8866289	0.2699618	2.911934	0.8427891
cg10899301	0.3514141	0.1757921	0.702488	0.0030846
cg21848981	2.0933777	0.8516242	5.145732	0.1074066
cg15686608	1.2959853	0.4255139	3.947175	0.6481951
cg04028450	0.118704	0.0432941	0.325463	3.45E-05
cg11362758	0.4933768	0.2539334	0.958601	0.0370939
cg00433654	0.2734529	0.0555369	1.346429	0.1108835
cg23159337	0.4298239	0.1545742	1.19521	0.1056157
cg18166564	0.2669432	0.1020459	0.6983	0.0071047
cg13048962	0.094926	0.0058084	1.551363	0.0985564
cg03975447	1.5956279	0.5029417	5.062274	0.4276414
cg24849555	2.281242	0.5400822	9.63569	0.2618932
cg24575275	0.1332615	0.0418392	0.424449	0.0006501
cg20536041	2.6110855	1.3220134	5.157109	0.0057123
cg21128568	2.1419027	0.426304	10.76168	0.3550725
cg14170787	0.3460504	0.1389005	0.862134	0.022698
cg11505026	0.2852468	0.1164922	0.698465	0.0060439
cg11019430	0.9165036	0.4483064	1.873671	0.8111254
cg22221831	0.5715034	0.2851172	1.14555	0.114805
cg07363543	0.1693359	0.0264513	1.084054	0.0608255
cg23820828	4.5463198	1.1329273	18.24391	0.0326789
cg16206191	0.5279844	0.2378569	1.171997	0.1164466
cg13318291	3.593772	0.6320773	20.43294	0.1491283
cg16754967	105545339	3722.5374	2.99E+12	0.0004128
cg12032648	0.3998492	0.1802735	0.886871	0.0241116
cg03716936	1.8147059	0.8704821	3.783142	0.111858
cg07133994	2.5180091	0.0877253	72.27524	0.5897769
cg22168321	2.54E-33	1.40E-52	4.63E-14	0.00091
cg21850879	0.2655578	0.0871707	0.808999	0.0196537
cg00077904	0.2041661	0.0629335	0.662347	0.0081435
cg20065005	1.0791198	0.2388098	4.876264	0.9211763
cg04138198	2.8867965	0.8959535	9.301369	0.0757462
cg10466728	5.0327194	1.1316833	22.38105	0.0338006
cg17246140	2.1469968	0.5752104	8.013755	0.2555322
cg00095431	0.5708031	0.2701499	1.206057	0.1418104
cg17292384	0.5457553	0.2261463	1.317063	0.1778955
cg24401187	0.4728272	0.222841	1.003252	0.0509969
cg03465652	0.1443701	0.0291684	0.714566	0.0177
cg08405910	0.0108843	0.0008549	0.138575	0.0004967
cg05057515	2.3933664	0.9580263	5.979171	0.0617382
cg23242944	0.2438174	0.0808212	0.735536	0.012239
cg20967887	1.4730672	0.411973	5.267158	0.5512823
cg11959708	45.730494	0.7455611	2804.972	0.0687344
cg23855715	1.7403984	0.7382997	4.102652	0.2053361
cg17646499	0.4695491	0.2455785	0.897783	0.022253
cg09550945	0.4011147	0.13758	1.169451	0.094279
cg19120064	1.2148393	0.6764874	2.181614	0.5147135
cg03653317	0.5560518	0.2266367	1.36427	0.1999681
cg09439204	2.723012	0.7656035	9.684901	0.1217697
cg15606692	2645.4309	25.156172	278194.3	0.0009075
cg02025583	0.174974	0.0681316	0.449364	0.0002921
cg08317373	0.3225161	0.0464255	2.240505	0.2525209
cg19470652	0.0014708	2.82E-06	0.768359	0.0411035
cg04211275	740.26687	2.6641151	205695	0.021377
cg04874846	4.95E-05	6.39E-09	0.383595	0.0300323
cg17589341	1.0889629	0.4465655	2.655467	0.8513541

cg00064296	0.5328949	0.1592356	1.783376	0.3071148
cg10157558	0.7640095	0.0284079	20.54745	0.8726735
cg00524694	1.4471584	0.6804629	3.07771	0.3370513
cg26106948	2.7512921	0.8431852	8.977397	0.093487
cg08692733	1.532014	0.7965902	2.946392	0.2010995
cg14282221	1.787453	0.8598076	3.715934	0.1198413
cg12422026	0.0591069	0.0141894	0.246214	0.0001023
cg23919549	0.5829611	0.2711061	1.253545	0.1671356
cg16906859	0.6410415	0.3380932	1.215447	0.1731242
cg12459280	0.3737235	0.0469886	2.97241	0.3522178
cg16617774	0.0315883	0.0038277	0.260685	0.0013343
cg05887405	40.088874	0.0625307	25701.27	0.2630013
cg21253335	0.4285935	0.1346783	1.363935	0.1514379
cg06488655	0.4382514	0.1719044	1.117274	0.0840379
cg08080822	6.2631509	0.15887	246.9129	0.3277518
cg23231462	1.1765028	0.5039004	2.74689	0.7071222
cg19978550	1.9796866	0.8935476	4.386066	0.0924437
cg14651616	3.9607423	0.2637317	59.48273	0.3193688
cg13426857	5.9046987	0.0039803	8759.489	0.6336274
cg13354934	0.3310589	0.090518	1.210809	0.094753
cg20256260	3.4533928	0.9555154	12.48114	0.0586844
cg17164063	2.0427089	1.0269448	4.063178	0.0417763
cg14654731	4.4261034	0.7430271	26.36565	0.1023127
cg21880020	4.3645986	1.0091184	18.87759	0.0485968
cg05726758	4.0928288	1.1513503	14.54922	0.0294241
cg00303541	0.6740317	0.3055487	1.486895	0.3284491
cg05436845	0.3043476	0.0893564	1.036608	0.0571104
cg21768294	0.814442	0.1803166	3.678618	0.7896188
cg22964469	0.0356336	0.0037157	0.341729	0.003842
cg17641566	0.1460271	0.0441999	0.482442	0.0016029
cg10913846	1.1164301	0.5880152	2.119701	0.736353
cg08347471	1.7700244	0.3160575	9.91271	0.5159583
cg07901206	3.6822285	1.1467105	11.82409	0.0285274
cg02198044	0.8719176	0.1080766	7.034272	0.8976229
cg00426425	27.78451	1.4345628	538.1284	0.0279053
cg12301814	0.1271313	0.041782	0.386825	0.0002803
cg06528601	0.3111054	0.0959131	1.009107	0.0517915
cg18771553	0.288978	0.1133154	0.736954	0.0093499
cg07653326	2.1312416	1.0107221	4.494006	0.0468136
cg20002177	1.890797	0.6972837	5.127201	0.2107353
cg14822546	0.503762	0.2145185	1.183004	0.1154562
cg07317616	0.3425076	0.1117048	1.050192	0.0608895
cg05412410	0.4845774	0.1776271	1.321956	0.1571065
cg08897388	0.0361156	0.004527	0.288124	0.0017221
cg24146331	2.7173007	0.9948424	7.422003	0.0511907
cg19536631	0.6272556	0.2488395	1.581138	0.3227936
cg10472711	4.9325825	0.2806331	86.69816	0.2752106
cg05389236	0.4298525	0.1546199	1.195015	0.105565
cg18391899	0.2516701	0.0421284	1.503449	0.1303217
cg15713734	0.9456603	0.4747495	1.883674	0.8737369
cg09777416	0.0002638	1.28E-10	543.7868	0.2666264
cg18501409	0.4320751	0.0189047	9.875282	0.5991633
cg18438823	0.7044609	0.3296003	1.505658	0.3660064
cg11741189	1.7474773	0.8215543	3.716951	0.1471915
cg00856775	0.3263677	0.0637428	1.671027	0.1790172
cg18909903	0.1372379	0.0431853	0.436126	0.0007609
cg07951728	2.7677444	1.1196225	6.841957	0.027478
cg00866632	0.389493	0.1424783	1.064757	0.0661103

cg08878352	1.04E-19	1.70E-37	0.063094	0.0364197
cg08777095	1.4853843	0.6658865	3.313427	0.3337482
cg09769410	0.1492653	2.38E-09	9348258	0.8355012
cg00040027	0.0457975	0.0006212	3.3766	0.1599145
cg11640544	854.77084	1.30E-17	5.61E+22	0.7718397
cg12810212	0.1178575	0.0084515	1.643542	0.1117417
cg06294475	0.7935241	0.218641	2.879974	0.7251077
cg10405729	0.63101	0.3202093	1.243479	0.1834056
cg10803767	0.547093	0.2453392	1.219987	0.1404776
cg14091531	1.9361961	0.7964838	4.706756	0.1448732
cg05863637	1.1740397	0.4800965	2.871025	0.7250796
cg27196999	0.3343583	0.1509981	0.740376	0.0069112
cg17477578	1.4130221	0.3585061	5.569309	0.6212656
cg24467387	0.3709873	0.1606895	0.856506	0.0201897
cg19642494	2.5411081	0.7331066	8.808037	0.1414402
cg14091149	14.240117	0.9787943	207.1742	0.0518624
cg10495270	2.3050781	0.8105272	6.555468	0.1173408
cg16183741	0.0155423	0.0014612	0.165318	0.0005564
cg19116924	1.9088385	0.0057972	628.5206	0.8269737
cg09952232	3.442021	1.2375043	9.573711	0.0178724
cg23460943	0.3757471	0.1777205	0.794426	0.0103948
cg22866825	0.001925	4.65E-05	0.079619	0.0009934
cg02147032	3.78E+22	32476.16	4.41E+40	0.0143091
cg00915818	0.5619927	0.3057232	1.033078	0.0635678
cg02632643	0.3282601	0.035548	3.031243	0.3260133
cg19478678	0.8044195	0.3997308	1.618816	0.5418955
cg11476737	0.4823111	0.2102766	1.106276	0.0851581
cg01324261	0.2901735	0.1444477	0.582915	0.0005082
cg05395852	0.412177	0.1643151	1.033927	0.0589108
cg23780731	0.4310904	0.1626204	1.142777	0.0907129
cg26199631	0.1560698	0.0384631	0.633276	0.0093424
cg01923549	0.001252	4.93E-05	0.031818	5.15E-05
cg13891702	1.8166868	0.7248532	4.55313	0.2028274
cg13494951	0.4264922	0.199539	0.911579	0.0278892
cg26751195	28.563323	1.1099516	735.0441	0.0430818
cg04737114	1.611439	0.7954037	3.264676	0.1853314
cg10571987	0.6529875	0.3710181	1.14925	0.139499
cg09486093	0.2583506	0.0540735	1.234339	0.0898628
cg08442052	0.4359081	0.1454085	1.306773	0.1382578
cg17338430	0.9616609	0.5070599	1.823831	0.9047088
cg01016191	1.4231238	0.7132877	2.839361	0.3167105
cg11901248	0.0015427	2.04E-06	1.165874	0.0555462
cg06633438	0.5419615	0.3042832	0.965292	0.0375343
cg03297966	0.0103888	0.0005884	0.183428	0.0018227
cg00941797	3.31E-36	7.68E-54	1.42E-18	8.03E-05
cg17327492	0.7118556	0.3797665	1.334342	0.2890466
cg15876657	21589.918	8.84E-11	5.28E+18	0.5549081
cg01910125	0.1049327	0.0228649	0.481562	0.0037329
cg06733419	0.2946245	0.0797368	1.088626	0.0668589
cg15983698	2.4886798	0.9689897	6.391737	0.0581576
cg04135110	2.5659543	0.876912	7.508303	0.0853976
cg08679238	1.5064963	0.709463	3.198942	0.2861637
cg24135939	0.869986	0.4097143	1.847325	0.716967
cg17827208	1.3007896	0.215244	7.861095	0.7744892
cg21082782	0.1909484	0.0512476	0.711473	0.0136168
cg16868095	0.2667512	0.0882218	0.80656	0.019244
cg26014266	2.1637592	1.1149894	4.199012	0.0225052
cg12557133	8.4403257	0.323865	219.9654	0.1997622

cg20425130	3.0775493	1.1093308	8.537859	0.0308298
cg17490981	2.4676679	0.500679	12.16225	0.2670361
cg16733136	0.0759841	0.0156115	0.36983	0.0014133
cg24439713	15.961805	0.2714158	938.7044	0.1826571
cg25232745	81.44949	0.0154468	429475.7	0.3143006
cg13334135	1.3482188	0.6935763	2.620755	0.3782976
cg13359161	0.732448	0.3623612	1.480512	0.3858578
cg01558212	4.5041241	1.5472428	13.1118	0.0057699
cg04730882	2.3839452	0.7010534	8.106651	0.164164
cg05173058	0.2217378	0.0898298	0.547342	0.001086
cg12785643	13.26835	0.0038193	46094.47	0.5342607
cg09170686	0.0338494	0.0037003	0.30965	0.0027177
cg06306927	1.9424486	0.2264869	16.65927	0.54482
cg10028071	33.373851	1.2771379	872.1172	0.0351269
cg14040633	3.3555403	0.0331063	340.1064	0.6074377
cg15981734	1.7006632	0.771707	3.747867	0.1877857
cg03815917	0.4962468	0.1945207	1.265988	0.1425461
cg00112685	0.084632	0.0174523	0.410409	0.0021727
cg16235995	1.4432945	0.3342866	6.231476	0.6229488
cg04916289	0.3839155	8.80E-05	1674.421	0.8228415
cg22517356	0.8593335	0.2491745	2.963602	0.8103264
cg06630988	0.4623139	0.1101073	1.941144	0.2919254
cg17664182	3.1104947	1.0126495	9.55432	0.0474884
cg02650128	0.2298619	0.09407	0.561672	0.001258
cg13849552	42.897062	4.0664582	452.5211	0.0017665
cg09300089	2.8505478	0.7436009	10.9274	0.1265466
cg09687322	35.615484	1.1997305	1057.29	0.0389018
cg00599564	2.4073702	0.8543151	6.783716	0.096497
cg17334978	0.0321805	0.0014781	0.700629	0.0287923
cg11779900	0.2917694	0.1410535	0.603526	0.0008948
cg08854266	0.5219571	0.2590517	1.051679	0.0689124
cg20249169	420633.84	14.969825	1.18E+10	0.0132221
cg10874992	8.7518771	0.5945455	128.8301	0.1138763
cg19378376	8.39E-29	6.29E-49	1.12E-08	0.0062523
cg08142918	2.0087602	0.8334981	4.841184	0.1201447
cg15128365	0.1897141	0.0724959	0.496462	0.0007075
cg11128457	0.1803178	0.0669221	0.485856	0.0007058
cg27458327	0.306639	0.0719496	1.306851	0.1100101
cg01200177	0.2055171	0.0287017	1.471595	0.1151856
cg18104354	0.2742049	0.1107021	0.679195	0.0051758
cg16845172	2.9574369	0.9625846	9.086404	0.0583076
cg23503775	1.8040229	0.7334402	4.437306	0.1988389
cg24347994	2.8929442	0.763144	10.96664	0.1181952
cg13314167	1.1013968	0.1362094	8.905953	0.927839
cg15707833	0.0923214	0.0043761	1.947687	0.1256586
cg08128007	0.3515209	0.1527489	0.808955	0.013951
cg00752806	83736472	761.32185	9.21E+12	0.0020682
cg15202213	0.7349877	0.2148681	2.514132	0.6236394
cg20043466	0.0115759	6.49E-05	2.064567	0.0918203
cg22379668	2.9427331	0.9601387	9.019195	0.0589212
cg04102161	1.4770735	0.5530952	3.944613	0.4363956
cg03595580	1.7966832	0.7523887	4.290429	0.1870502
cg25915982	3.2401185	0.6966045	15.07077	0.1338788
cg24687970	0.9910688	0.3771901	2.604038	0.9854779
cg16135936	0.4311186	0.1751273	1.061304	0.0671726
cg09356750	0.2034261	0.0451836	0.915866	0.0380376
cg08414888	0.53362	0.258856	1.100033	0.0888201
cg05518803	0.0001254	4.24E-07	0.037111	0.0019714

cg25709789	0.6456996	0.3113658	1.339029	0.2398168
cg26276556	4.008774	0.3214157	49.9984	0.2808494
cg19214408	1.3626227	0.6615528	2.80664	0.4013194
cg11464160	0.3344564	0.1372188	0.815203	0.0159767
cg10317175	0.0958793	0.0098734	0.931072	0.0432238
cg10308445	0.5665255	0.0791656	4.054172	0.571449
cg15355111	1.0017263	0.4496606	2.231584	0.9966325
cg14903689	0.0722777	0.0230031	0.227103	6.87E-06
cg06738242	1.8046543	0.9383395	3.470787	0.0768546
cg17465631	0.6244155	0.1544652	2.524159	0.5087464
cg14417382	1.02E-26	6.88E-62	1.51E+09	0.1474992
cg24581795	3.3531509	0.2522042	44.58143	0.359406
cg15911153	0.4476799	0.1053044	1.903218	0.2764124
cg12591454	0.5570462	0.1972565	1.573082	0.2693098
cg06726390	0.3361563	0.1039116	1.087473	0.0687634
cg23634087	2.0785527	1.0370024	4.166221	0.0391716
cg02351425	0.4556692	0.1535534	1.352197	0.1566952
cg26781437	779492.71	1569.7645	3.87E+08	1.84E-05
cg25210835	2.7062906	1.1469915	6.385408	0.0230211
cg03500965	1.666362	0.7373875	3.765676	0.219598
cg20524040	0.1268058	0.0263018	0.611355	0.0100796
cg07248242	2.1742867	0.3673052	12.87083	0.3919628
cg22217860	4.1606579	1.2359219	14.00661	0.0213365
cg03330490	2.0343302	0.7806223	5.301539	0.1461735
cg24952123	0.4665836	0.2206509	0.986628	0.0460212
cg03393607	3.5511528	0.7809901	16.14705	0.1009934
cg02257550	0.1153438	0.0173969	0.764744	0.0252297
cg09549813	0.4279933	0.1882391	0.973115	0.042868
cg05342945	1.57E-27	2.16E-46	1.14E-08	0.0053459
cg06287951	1.6543532	0.6476405	4.225932	0.2927658
cg03142751	0.2223163	0.0311781	1.585232	0.1335445
cg26922451	0.1851981	0.070788	0.484521	0.000589
cg19355881	0.0343093	1.26E-07	9353.284	0.5974267
cg15877233	0.5381942	0.2471131	1.172147	0.1187582
cg00754063	0.0046466	1.13E-05	1.918911	0.0804837
cg02795691	0.1422418	0.035619	0.568032	0.0057707
cg11905407	0.8539576	0.1409254	5.174679	0.863637
cg07097310	0.2066905	0.0888762	0.48068	0.0002511
cg10347828	3.2986292	0.6511889	16.70937	0.149365
cg25471831	330897.24	5.00E-06	2.19E+16	0.3174206
cg15701085	0.8907235	0.3819417	2.07725	0.7888119
cg02347074	0.0627608	0.0020145	1.955278	0.1146098
cg07638479	0.1839102	0.031188	1.084485	0.0614314
cg16695758	1.4373845	0.7184327	2.875807	0.305174
cg03728674	1.6366016	0.6580837	4.070098	0.2892378
cg25715134	0.6017619	0.2968708	1.219781	0.1588758
cg05945608	0.4664989	0.2236035	0.973246	0.0421293
cg16245345	0.2740143	0.0967418	0.776126	0.0148067
cg22651493	1.561256	0.5944721	4.10031	0.365848
cg04657183	0.4130545	0.1863938	0.915342	0.0294174
cg09631175	1.064556	0.2381776	4.758128	0.9347356
cg24199599	0.1261567	0.0199475	0.797869	0.0278125
cg12889538	2.0323465	0.805578	5.12729	0.1330807
cg27636011	0.3331612	6.82E-05	1628.221	0.7997973
cg25408314	1.6097356	0.8851184	2.927573	0.1187449
cg23685965	2.8366124	1.2658149	6.356672	0.0113245
cg00959616	0.3790788	0.1381113	1.04047	0.0597067
cg03325535	2.7423748	1.122719	6.698577	0.0268288

cg16177481	0.0801871	0.0075061	0.85663	0.0367966
cg04680535	2.9685838	1.4358874	6.137313	0.003322
cg23639196	0.2540191	0.0976386	0.660863	0.0049689
cg03621837	6.4288231	0.0335816	1230.725	0.4876327
cg25784220	0.6559447	0.3131467	1.374	0.2636706
cg06362078	20.464985	8.83E-13	4.74E+14	0.8475404
cg20139336	3.3063787	0.92573	11.80921	0.0655999
cg10942097	2.2105356	0.8031625	6.084034	0.1246318
cg18720698	0.824061	0.4433734	1.531613	0.5406054
cg26133301	1.4474182	0.52324	4.003936	0.4762817
cg08301503	2.21311	0.8746869	5.599553	0.093489
cg13459498	1.4772441	0.6758686	3.228808	0.3280724
cg22816909	0.3454809	0.124842	0.956065	0.0407095
cg11511175	0.2770263	0.1063689	0.721485	0.0085789
cg03658578	1.1892747	0.5518912	2.562777	0.6581097
cg00672558	0.0174301	0.0004157	0.730786	0.0336279
cg06666025	1.9402341	0.8064671	4.667901	0.1389378
cg23172480	2.7007756	0.9950706	7.330323	0.0511447
cg01896119	0.7860203	0.3301898	1.87113	0.586373
cg03526165	0.2977877	0.0883479	1.003731	0.0507062
cg02011723	0.3607786	0.1239534	1.050082	0.0614404
cg03434950	4.9333015	1.09364	22.25363	0.0378552
cg08836729	4.5428546	0.7566268	27.2757	0.0979217
cg24065136	6.10E-14	2.46E-33	1509340	0.1817048
cg23444894	0.6616297	0.3175425	1.378568	0.2701125
cg11122170	0.577374	0.2683931	1.242061	0.1599214
cg15775218	0.3931974	0.0458808	3.369691	0.3944223
cg05997362	1.6966981	0.7901997	3.64311	0.1750952
cg00427260	5.3918553	0.2535368	114.6662	0.2800524
cg26999431	2.3593838	0.3787532	14.69741	0.3577143
cg09263059	2.4948878	0.460769	13.50886	0.2887579
cg15065169	2.0677995	0.8480614	5.041846	0.1101411
cg25750004	0.1643975	0.053459	0.505556	0.0016326
cg01834979	0.2485231	0.080956	0.76293	0.0149827
cg17159699	1.3573344	0.72116	2.554713	0.3437075
cg16333846	0.2589828	0.1233601	0.54371	0.0003566
cg14168983	1.3418658	0.4425293	4.068892	0.6033728
cg06957551	1.4241975	0.5365616	3.780253	0.477723
cg00503840	4.9227626	1.297069	18.68335	0.0191709
cg03905179	0.0707415	0.0021829	2.292551	0.1355751
cg18545424	0.7954719	0.3392506	1.865216	0.5987064
cg18393175	0.7644891	0.3760424	1.554196	0.4581814
cg08095985	0.2364486	0.0742042	0.753434	0.0147374
cg22964621	0.2391671	0.0996646	0.573934	0.0013592
cg13948330	0.8031093	0.255957	2.519895	0.7070461
cg19698137	0.3621071	0.1139569	1.150624	0.0850503
cg01654862	1.5555741	0.795996	3.039979	0.1961751
cg16199277	1.8093119	0.9763025	3.353069	0.0595959
cg07157030	0.4753485	0.1577784	1.432111	0.18627
cg17081645	1993685.1	1.15E-05	3.45E+17	0.2719315
cg14274542	0.3474305	0.126358	0.955286	0.0405005
cg08058408	5.70E-05	4.32E-08	0.075245	0.0076845
cg04940962	1.616E+09	0.0721367	3.62E+19	0.0812039
cg26189983	1.5775176	0.7722229	3.222595	0.2110255
cg27155939	1.1008444	0.3530864	3.432186	0.8684708
cg19253577	2.0197022	0.5234149	7.793428	0.3075812
cg23470939	4.30E-05	1.01E-09	1.827145	0.0644435
cg05616584	19.01796	0.4281805	844.697	0.1280754

cg15602359	0.5026268	0.1941989	1.300902	0.1562506
cg15736127	0.2100326	0.0681668	0.647144	0.006569
cg03970319	1.0112623	0.3327163	3.073644	0.9842464
cg12660466	0.4433223	0.208762	0.94143	0.0342564
cg04927537	0.1096331	0.0147035	0.817451	0.0310365
cg19123462	3.58E-06	5.77E-09	0.002222	0.0001324
cg00998744	1.7052328	0.7368672	3.946191	0.2125104
cg07723983	11.020911	0.7614627	159.5094	0.0783921
cg12113740	0.3642116	0.1618482	0.819595	0.0146583
cg15931168	1.6249374	0.6386277	4.134524	0.3082766
cg03320754	1.8575131	0.6780306	5.088789	0.2284775
cg07937999	0.6645215	0.3507754	1.258893	0.2099519
cg08601959	2.324E+14	1018.3251	5.30E+25	0.0131752
cg04314130	1.7075085	0.8213832	3.549604	0.1518659
cg22664425	11.18547	2.2870294	54.70623	0.0028693
cg16691593	2.1676934	0.911913	5.152788	0.0799047
cg05905030	0.4520147	0.1872175	1.091336	0.0774602
cg21480740	9.044031	2.1850027	37.43451	0.0023782
cg24568905	0.5106723	0.1874444	1.391272	0.1887796
cg08353973	0.4506513	0.1988601	1.021253	0.0561874
cg05723953	0.6668318	0.3535938	1.257558	0.2105948
cg19883017	539.59227	0.0123075	23657104	0.2486766
cg00346392	2.7122446	0.897392	8.197388	0.0770414
cg21830050	1.1272468	0.2316463	5.485455	0.8820548
cg16630085	0.0959261	0.0150557	0.611184	0.0130987
cg03253489	0.4519936	0.1595669	1.280329	0.1349691
cg07448742	0.831021	0.3302276	2.091272	0.6942388
cg08159594	0.2695775	0.0991275	0.733116	0.0102239
cg13732857	1.7909299	0.9532396	3.364768	0.0701213
cg03773789	4.76E-06	8.39E-12	2.696245	0.0697972
cg10960354	1.839589	0.6753039	5.011207	0.233208
cg06728103	0.3351393	0.1275865	0.880331	0.0265115
cg24020215	1.2856494	0.6933303	2.383993	0.4251588
cg01812401	167.51746	1.1794982	23791.56	0.0428415
cg14612133	1.1257551	0.3054207	4.149439	0.8587463
cg22512438	2.9058897	1.0508804	8.035353	0.0398214
cg09354348	0.5162573	0.2146919	1.241414	0.1397036
cg25607920	0.2547746	0.0826749	0.785125	0.0172541
cg04053485	0.140449	0.0213802	0.922625	0.0409718
cg15164708	1.97435	0.8461071	4.60705	0.1156191
cg15705175	0.1297881	0.0385024	0.437504	0.0009902
cg24124703	0.3827102	0.1524868	0.960523	0.0407807
cg23514016	3.1345255	0.9489144	10.3542	0.0609367
cg04602992	4.4090359	0.9933403	19.56993	0.0510361
cg06341779	3.2786023	0.9365162	11.47789	0.0632585
cg12970155	6.282931	0.6376717	61.90524	0.1153717
cg05126581	1.7313357	0.7842525	3.822141	0.1743087
cg04897621	25.993607	0.1860114	3632.398	0.1961433
cg15566506	0.0850341	0.0063472	1.139209	0.06267
cg22704788	0.340045	0.1157269	0.999168	0.0498234
cg25941985	0.9834435	0.4496035	2.151142	0.9666528
cg01926813	1.2472744	0.4854453	3.204673	0.6462799
cg04024840	0.2059446	2.25E-07	188182.3	0.8214784
cg12215340	0.1836069	0.0409421	0.823393	0.026845
cg25036707	0.2906822	0.0752037	1.123564	0.0732817
cg19492423	0.2442234	0.0947359	0.629593	0.0035278
cg05278454	0.0204414	0.0005319	0.785605	0.0366558
cg03421440	23.180106	2.1046098	255.3049	0.0102325

cg13543273	0.0981608	0.0212629	0.453162	0.0029381
cg09412654	0.6101618	0.2955337	1.259746	0.1816569
cg23686029	5.1293308	0.5747089	45.77976	0.143195
cg05280698	0.1556651	0.0338976	0.714849	0.0167763
cg07179195	1.3615993	1.34E-07	13804248	0.9700854
cg03012726	1.1129085	0.5778477	2.143412	0.7490426
cg10933774	0.2904318	0.1032406	0.81703	0.0191347
cg16540590	0.7356712	0.363593	1.488511	0.3932629
cg10078335	0.0971396	0.0006542	14.42436	0.3607815
cg17397592	2.5613078	1.2121256	5.412226	0.0137418
cg03001863	0.3043313	0.1202567	0.770165	0.0120311
cg12753009	1.9854115	0.5353928	7.362554	0.3050575
cg25198545	1.84E-08	6.36E-18	53.37983	0.1091205
cg22004422	2.0866474	0.8464319	5.144061	0.1100878
cg20847580	0.639868	0.3155842	1.297375	0.2156912
cg22027897	1.7051784	0.8745424	3.324748	0.1172374
cg19256125	4.17E-07	4.63E-12	0.037531	0.0116049
cg19257762	0.183604	0.0513785	0.656119	0.0090939
cg00188321	0.976033	0.1743787	5.463054	0.9779756
cg15633416	5.8546916	0.1210804	283.0963	0.3718304
cg09597192	0.6884257	0.3051434	1.553139	0.368457
cg17231999	1.7192459	0.9807977	3.013676	0.0584567
cg03679305	1.2676706	0.6314556	2.544896	0.5047462
cg16976547	50788566	0.0001233	2.09E+19	0.193486
cg04425617	1.5982974	0.689205	3.706524	0.2745397
cg09121047	1.6080209	0.6217081	4.159076	0.3272378
cg15153770	0.1532651	0.000204	115.1605	0.5787997
cg06895759	5.105556	0.7194643	36.23071	0.102964
cg05847060	0.8829972	0.4233738	1.841597	0.740051
cg10714509	0.3900987	0.0717298	2.121531	0.2759433
cg03106288	9.71E-11	9.22E-20	0.102198	0.0296192
cg01553866	0.0002899	1.24E-10	677.9449	0.2762859
cg26104932	0.2642284	0.0700565	0.996576	0.0494111
cg11349093	2.794749	0.2383652	32.76745	0.4132012
cg27154418	0.1819064	0.0805848	0.410623	4.08E-05
cg13372658	1.8435352	0.2357936	14.41355	0.5599094
cg15152331	2.3940822	0.8217105	6.975242	0.1095859
cg14224786	0.1310077	0.0363568	0.472071	0.0018858
cg13976496	0.4543295	0.1787959	1.154474	0.0973033
cg23364226	0.6197039	0.3016785	1.272988	0.1926385
cg21185662	0.2807639	0.119251	0.661029	0.0036435
cg11241151	0.5109489	0.2370657	1.101251	0.0865642
cg12583138	0.0010817	3.68E-07	3.177408	0.0936971
cg07212940	0.4326389	0.1756014	1.065917	0.0685753
cg15930041	0.531183	0.2035066	1.386468	0.1962081
cg04116155	0.4495466	0.1740861	1.160875	0.0985804
cg13562102	0.2256173	0.0888726	0.572766	0.0017341
cg15249357	2.7656121	1.0213895	7.488437	0.045327
cg11782387	0.3202513	0.0732923	1.399342	0.1301825
cg15582789	0.058302	0.0076448	0.444631	0.0061086
cg14343924	28.78741	0.3128651	2648.793	0.1453046
cg15557906	0.0022286	2.01E-15	2.47E+09	0.6660899
cg09424595	3.8589518	1.614674	9.22261	0.0023831
cg26330518	1.3390296	0.6779899	2.644582	0.4004761
cg00554993	1.4151246	0.3711595	5.395463	0.6111092
cg16294425	2.4152791	0.6482775	8.998574	0.1888244
cg21221545	97.815141	0.2284353	41884.08	0.1382362
cg17336714	2807839.4	223.84858	3.52E+10	0.002044

cg10666081	6.105652	0.8031013	46.41879	0.0804472
cg04760448	1.4195743	0.6780661	2.971969	0.3526933
cg13606025	1.8736546	0.1877528	18.69789	0.5926903
cg19226872	1.2730514	0.6152366	2.634206	0.5152387
cg13687885	1.8827533	0.6587913	5.380703	0.2376068
cg03876340	0.3360247	0.141151	0.799942	0.0137256
cg08981777	0.2297222	0.0850979	0.620136	0.003696
cg06688763	0.4996783	0.2047507	1.219426	0.1274707
cg10100811	0.5317338	0.2343614	1.206431	0.1307863
cg02637304	2.29E-06	3.39E-10	0.015429	0.0038864
cg08636203	3.6620734	1.1600037	11.56098	0.0268969
cg23591125	268.69276	0.0018032	40036752	0.3573801
cg22921027	0.4001603	0.1273405	1.257482	0.1169321
cg04545963	2.1130324	0.7711344	5.790049	0.1457703
cg06937051	0.0711307	0.0170027	0.297576	0.0002947
cg14703002	0.5232817	0.2150533	1.273283	0.1534486
cg02110782	3.66E-44	8.08E-82	1.66E-06	0.0237673
cg23999422	0.0070295	0.000174	0.283947	0.0086115
cg07214164	2.8626505	0.6911541	11.85664	0.1469144
cg22513396	2.7500631	1.0142441	7.456634	0.0468388
cg08911391	0.2847015	0.0454794	1.782232	0.1794441
cg19745515	0.2861899	0.0788258	1.039059	0.0572073
cg11115431	4.0375386	0.9549405	17.07093	0.0577903
cg21215818	0.3160401	0.1223383	0.816436	0.0173698
cg26405913	7.2176567	0.6568463	79.31013	0.1060364
cg16200116	3.10859	0.1448557	66.71004	0.4684629
cg16163558	0.8986676	0.340498	2.37183	0.8291663
cg22218316	0.2703276	0.1255574	0.582021	0.0008279
cg26086468	0.5261246	0.2728821	1.014384	0.0551963
cg04206389	0.8744186	0.3549128	2.154354	0.7705183
cg10117719	0.3906875	0.0875172	1.744077	0.2182225
cg06436905	0.2155806	0.0509091	0.912902	0.0371864
cg06763161	0.4868058	0.2009645	1.179212	0.1107619
cg06641548	36.43677	0.6675308	1988.879	0.0780842
cg24599065	1.6518779	0.4432271	6.156438	0.4546102
cg00049156	0.465832	0.2334475	0.929543	0.0302167
cg15245581	0.5753249	0.3031369	1.091912	0.090837
cg03025473	0.7043844	0.327521	1.514887	0.3697644
cg03717364	0.2357238	0.0671526	0.827455	0.0240962
cg00748494	0.2537037	0.0672079	0.957709	0.0429989
cg22170602	21035661	3.60E-09	1.23E+23	0.362649
cg19205031	1.1362535	0.4924355	2.621809	0.7646147
cg24182521	0.3092756	0.1103965	0.866435	0.0255665
cg15832822	1.228138	0.594319	2.537901	0.5789599
cg13985767	4.442E+13	1.49E-23	1.32E+50	0.4633332
cg26864036	10.373723	1.1002586	97.80804	0.0410103
cg03714110	2.508359	1.0074534	6.245316	0.048164
cg15627072	2.2254786	0.7064591	7.010675	0.1718068
cg26886277	1.1914438	0.4945052	2.870623	0.696228
cg14468055	24.985227	0.173647	3595.003	0.2042945
cg15248035	3.0741447	0.5602679	16.86758	0.196025
cg05079794	0.0644435	0.0026104	1.590925	0.0937121
cg10203922	2.4238148	0.9145522	6.423775	0.0750191
cg06553033	6.8236686	0.7314201	63.66034	0.0918999
cg17677490	2.3204539	0.995143	5.410787	0.0513322
cg05799962	0.739431	0.2307354	2.369633	0.6114285
cg23694882	0.9818641	0.4632019	2.081289	0.9619177
cg01467882	1.1431578	0.5332613	2.450599	0.730926

cg23549179	0.4606592	0.2196491	0.966118	0.0402492
cg00116838	1.2986411	0.7108974	2.37231	0.395315
cg20671920	2.5536118	1.0855122	6.007241	0.0317173
cg18461635	2.4357024	0.4461968	13.29603	0.3039287
cg01084435	2.0873295	0.5478337	7.953041	0.280933
cg13432682	0.415686	0.1374249	1.257376	0.120086
cg01161204	1.5159561	0.7628787	3.012436	0.235044
cg27284331	1.3425854	0.5549065	3.248359	0.5134354
cg10451940	0.1443161	0.0405425	0.513711	0.0028061
cg07408740	0.3046348	0.1080247	0.859085	0.0246334
cg05099576	4.5042825	1.1058182	18.3471	0.0356993
cg03529598	2.225509	0.7048304	7.027067	0.1726667
cg26916780	0.8369712	0.3521617	1.989202	0.6870082
cg17011134	0.6427381	0.3101763	1.331863	0.2344191
cg21006913	0.5372921	0.2436598	1.184778	0.1236308
cg00524967	0.7219393	0.3579547	1.45604	0.3626815
cg27620436	1.5655935	0.6208165	3.94816	0.3421959
cg26428191	0.5218258	0.2126666	1.280418	0.155543
cg03761513	1.6537857	0.8413733	3.250647	0.1445569
cg15928680	1.2967458	0.6608979	2.544341	0.4498647
cg01132443	0.3792426	0.1673185	0.859588	0.0202131
cg14219543	1.699394	0.72801	3.966896	0.2201906
cg21153102	4.2168245	0.5003427	35.53886	0.1857548
cg13954457	0.2102286	0.0686492	0.643796	0.0063111
cg20026651	2.48E-14	2.25E-41	2.73E+13	0.3240594
cg18220030	27703580	25.28223	3.04E+13	0.0157268
cg02490989	1.9926184	1.0255561	3.871585	0.0419085
cg05103957	2.73E-23	2.37E-39	3.14E-07	0.0058963
cg20256153	6.5015361	1.7740101	23.82736	0.0047277
cg08317694	4.9528464	1.1741589	20.89214	0.029363
cg21089930	0.3331003	0.1527517	0.72638	0.0057162
cg09731946	1.8972038	0.7687936	4.681858	0.1646911
cg17315219	1.5610636	0.6407825	3.803037	0.3269309
cg18476604	0.283974	0.0704279	1.145019	0.0767935
cg18853287	2.0222418	0.1499865	27.26553	0.5957205
cg07668501	2.2024995	1.0167011	4.771318	0.0452892
cg27287745	1.9095777	0.9081861	4.015132	0.0880114
cg14441787	0.5719707	0.2605273	1.255724	0.1637965
cg16629523	0.2189926	0.0546862	0.876963	0.0319183
cg22157494	0.2915996	0.1229253	0.691723	0.00517
cg26272614	1.50E+20	7.79E-27	2.88E+66	0.3929039
cg11478481	0.4840497	0.2076949	1.128117	0.0928172
cg27058931	9.5247079	1.0707981	84.72191	0.0432477
cg13678243	91.241142	0.5124957	16243.93	0.0877974
cg03487637	5.5766086	0.0001822	170679.6	0.7443427
cg02298862	1.1595053	0.5958833	2.256235	0.6630384
cg16126754	0.17274	0.0450772	0.661955	0.0104113
cg04217539	0.6721746	0.3547937	1.273469	0.2230509
cg18164611	2.3739506	1.0214045	5.517541	0.0445178
cg14011077	3.3302009	0.507794	21.84004	0.209941
cg09424566	0.2734869	0.1270897	0.588522	0.0009138
cg08603449	0.6722296	0.0687711	6.570962	0.7327757
cg15713809	0.0477007	0.0006751	3.37053	0.1613189
cg03336500	1.4629127	0.6988688	3.062254	0.3128073
cg25690958	0.306666	0.1149265	0.818297	0.0182544
cg17038444	0.0005521	8.04E-09	37.92963	0.1867825
cg07839742	0.4254866	0.2079911	0.870416	0.0192834
cg07582204	1.6294367	0.4609799	5.759609	0.4485248

cg17945323	1.9686752	0.9921669	3.90628	0.0526879
cg25392692	3.0871166	1.1828784	8.056863	0.0212725
cg02862761	3.7110034	0.9860249	13.96673	0.0524829
cg05916707	1.2157337	0.6948158	2.127195	0.4937421
cg16105510	7.6418274	0.7067845	82.62423	0.0940795
cg05431171	0.5318134	0.1028023	2.751158	0.4514133
cg03354707	1.0566479	0.435482	2.563837	0.9030278
cg08842287	0.096309	0.0241676	0.383795	0.0009081
cg00393837	0.683699	0.0389529	12.00025	0.7947806
cg01473249	2.0523341	0.4947636	8.513308	0.3219185
cg21801223	0.7724995	0.4111492	1.451433	0.4224502
cg10711778	0.3555985	0.1015839	1.244787	0.1057848
cg16486653	2.3752406	0.4968701	11.35461	0.2784734
cg06579875	1.561792	0.7127925	3.422026	0.2652803
cg22778014	0.2788548	0.1057861	0.735068	0.009813
cg08287551	1.7668078	0.6763772	4.615191	0.2453059
cg10052782	0.4510034	0.1816379	1.119832	0.0861522
cg26171469	2285.2387	0.0338441	1.54E+08	0.1728273
cg20403938	2.6655015	0.9859863	7.205879	0.0533409
cg04142864	1.5534424	0.9092102	2.654153	0.1070268
cg13011928	0.5175196	0.2475655	1.081841	0.0799681
cg01319892	0.2757011	0.0794625	0.956565	0.0423641
cg09528265	0.5748325	0.1647637	2.005493	0.385148
cg02859129	2.6923213	0.8175094	8.86668	0.1033919
cg04267691	1.8593511	0.6365583	5.43106	0.2567627
cg25498731	0.7754672	0.3671269	1.637988	0.5050765
cg20545941	2.0972612	0.9176064	4.793455	0.079074
cg11336860	3.5533191	1.6430847	7.684374	0.0012738
cg08764927	0.5311279	0.2355169	1.197777	0.1272556
cg13902110	1.7921486	0.8854243	3.62741	0.1048657
cg25756241	2.37E-08	1.10E-15	0.510709	0.041555
cg25756635	0.0002261	1.09E-06	0.046967	0.0020472
cg07206053	0.423689	0.2163126	0.829875	0.0122925
cg23178550	3.1446577	1.0520864	9.399296	0.0402812
cg22083684	0.449637	0.078897	2.562499	0.3680102
cg06567596	3.21884	0.3303427	31.36419	0.3142184
cg03223580	0.0634389	0.0072833	0.552565	0.0125215
cg01982240	0.6418633	0.2833137	1.454177	0.2879674
cg04176122	0.2435104	0.0732418	0.80961	0.0211934
cg05225187	0.0027463	2.42E-05	0.311976	0.0145916
cg01248445	0.4125458	0.1641582	1.036768	0.059678
cg16434372	45287.984	24.090316	85138006	0.0053172
cg16415457	2.1670477	0.5043881	9.31048	0.2984409
cg01238234	0.86674	0.419802	1.789506	0.699013
cg03101580	0.8222844	0.2819046	2.398512	0.7201624
cg01786466	1.3760313	0.5337072	3.547755	0.5088922
cg21586203	0.0361166	0.0038474	0.339032	0.0036529
cg01405040	1.9899452	0.7600207	5.210229	0.1611584
cg00945507	0.3118291	0.0605123	1.606902	0.1636249
cg03060590	3.3995375	1.035217	11.1637	0.0436938
cg07531248	0.1184273	0.0219771	0.638164	0.0130414
cg12726784	8.29E-20	4.52E-41	151.9616	0.0786015
cg06768385	338.96538	0.0165019	6962673	0.2501917
cg10728060	45541461	1.90E-11	1.09E+26	0.414137
cg19951871	335.64491	12.374498	9104.007	0.0005526
cg01404163	2.8215698	0.8729003	9.120464	0.0831177
cg09695652	1528.2396	1.71E-05	1.37E+11	0.4325554
cg13261306	0.6497471	0.297549	1.41883	0.2792326

cg18374322	3.67E-15	2.04E-30	6.606278	0.0636513
cg16935061	8.037745	2.3221741	27.82106	0.0010023
cg27521476	2.1872563	0.8825144	5.420977	0.0910132
cg14323210	0.002686	1.58E-05	0.457685	0.0239395
cg06076931	1.735286	0.6308655	4.773153	0.2856819
cg20673829	1.4904744	0.6738857	3.296574	0.3244209
cg23043937	0.5106166	0.2316909	1.125332	0.0954955
cg03716838	7.36E-24	3.62E-43	0.00015	0.0188638
cg16145211	1.6614735	0.7775771	3.550123	0.1900044
cg11393310	3.46E-06	1.16E-13	103.5552	0.1522452
cg06777813	8.9128444	1.6708398	47.54423	0.0104395
cg05711023	0.4586515	0.2334764	0.900995	0.023661
cg06811360	563128583	0.0072713	4.36E+19	0.1152414
cg06144361	0.2510219	0.0945265	0.666607	0.00554
cg02478731	1.5026559	0.2121939	10.64109	0.6834579
cg09424398	0.4128365	0.1386785	1.228986	0.111945
cg06807926	0.3208375	0.1076776	0.955971	0.041271
cg14502847	1.8088907	0.7976197	4.102313	0.1559817
cg10022788	1.6937155	0.6275438	4.57127	0.2982593
cg12835048	0.2247303	0.0421763	1.197443	0.0803129
cg25256924	2.6182121	0.741113	9.249649	0.1349933
cg27147718	404.6163	2.0951345	78140.26	0.0253921
cg02387403	0.3738857	0.1549549	0.902137	0.0285872
cg15363333	2.033003	0.625189	6.610963	0.2382877
cg17379860	2.243548	0.7782479	6.467743	0.1346911
cg00151676	35213.873	0.3053811	4.06E+09	0.0783241
cg27302539	1.4675527	0.6493254	3.316844	0.3565159
cg12768523	0.444317	0.2011012	0.981683	0.0448913
cg08913530	0.5074654	0.1645627	1.564882	0.2377692
cg09469682	1.7143391	0.8578078	3.426127	0.1270573
cg15400629	3.0178414	0.9451474	9.635922	0.0622202
cg13583230	1.3580363	0.6345461	2.906428	0.430505
cg22938275	3.717E+18	2454.3038	5.63E+33	0.0165007
cg21584979	0.3185259	0.1417494	0.715761	0.0056144
cg02053678	2.2037738	1.101749	4.408099	0.0254891
cg24831725	1.3667747	0.5109378	3.656165	0.5336918
cg25336874	3.3561804	1.104428	10.1989	0.0327515
cg03673694	1.3479658	0.5877111	3.091675	0.4808053
cg20183802	0.0480092	0.0012036	1.91498	0.1064189
cg20734996	0.168834	0.0397495	0.717114	0.0159273
cg15504747	1.9549218	0.7619272	5.015859	0.1632024
cg01092213	0.222102	0.0642635	0.76761	0.0174094
cg06028917	0.245162	0.0673409	0.892539	0.0329737
cg22697864	2.5029303	0.8170272	7.667628	0.108234
cg11873372	0.4767502	0.199089	1.141654	0.0963883
cg00421089	0.8509934	0.4407565	1.643061	0.6307473
cg03879160	2.4319192	0.9061917	6.526468	0.0776659
cg12614633	2200.2358	1.5570968	3109015	0.0375605
cg21742463	0.2644901	0.0908329	0.770151	0.0147319
cg21913897	1.5643632	0.8155679	3.000648	0.1781411
cg04013733	3.0131452	0.1888532	48.07461	0.4350957
cg03626208	0.4831102	0.1758114	1.327533	0.1583587
cg22980697	0.3128964	0.1020042	0.959805	0.0421839
cg19255554	4.8377781	0.8080046	28.9653	0.0842598
cg04281344	0.4997665	0.2427941	1.028718	0.0596872
cg22697108	0.5667027	0.1640546	1.957591	0.3692238
cg16980245	0.2053827	0.0461584	0.913854	0.0376873
cg03903268	334.22156	0.0133748	8351852	0.2606324

cg25677548	40.1413	0.5251687	3068.202	0.095142
cg21747818	0.1133702	0.002443	5.261112	0.2661607
cg15951545	0.2193301	0.0288555	1.667126	0.1426272
cg08297686	0.3461716	0.1053247	1.137765	0.0805735
cg14704669	1.1140792	0.4643437	2.672961	0.8088306
cg16410171	1.9867029	0.4869348	8.105783	0.3386283
cg23615483	1.4325071	0.645205	3.180503	0.3771213
cg08370996	0.0100593	1.43E-05	7.061176	0.1689986
cg05875017	1.2139503	0.5494993	2.681851	0.6316436
cg24686236	1.3649388	0.2034917	9.155447	0.7486788
cg07318284	0.2510827	0.0891842	0.70688	0.0088752
cg25980539	0.5017151	0.2545718	0.98879	0.0463127
cg21937867	7.8877357	0.0685662	907.391	0.3936335
cg02886589	1.4848459	0.696375	3.166063	0.3061816
cg07745674	0.1468209	0.0441743	0.487985	0.0017435
cg18731327	2.0022194	0.7493789	5.349607	0.1661816
cg26185340	0.4390902	0.2045694	0.942468	0.0346855
cg27128761	0.5663456	0.214532	1.495102	0.2510011
cg09143673	0.9885985	0.440786	2.217237	0.977802
cg27012424	0.5209577	0.2369867	1.145199	0.1046745
cg09618974	0.5580515	0.1889453	1.64821	0.2911313
cg00040986	8.7892167	0.330828	233.506	0.1939728
cg05825073	2.5800452	1.2739428	5.225221	0.0084781
cg16996788	4.3256264	0.9050563	20.6739	0.0665094
cg26802786	0.3931936	0.1583506	0.976323	0.0442618
cg14539658	0.2565967	0.0885475	0.743577	0.012219
cg15345477	0.3463576	0.1354548	0.885635	0.0268625
cg12192691	2.3592314	0.882898	6.304208	0.086969
cg02642949	13.234856	0.1336353	1310.742	0.2706448
cg18875629	0.2760405	0.0991637	0.76841	0.0137285
cg04962528	0.609768	0.2544648	1.461173	0.2672448
cg23111830	0.1139667	0.0187042	0.694409	0.018498
cg04567517	0.159182	0.0403067	0.628653	0.0087333
cg03873826	1.142641	0.4669735	2.795937	0.7702378
cg21644914	0.3174224	0.1247968	0.807368	0.0159875
cg03687765	1.5325917	0.3549791	6.616833	0.5672353
cg24402667	0.437616	0.1864761	1.026982	0.0575921
cg19439022	2.1215846	0.4031562	11.16471	0.3746687
cg21229570	1.6869848	0.8404042	3.386368	0.1413167
cg06639267	0.3749919	0.1809965	0.776915	0.0083113
cg05251389	0.3809127	0.0661418	2.193687	0.2799139
cg26057752	0.2453616	0.0905763	0.664658	0.0057211
cg09628216	0.316501	0.0893813	1.120737	0.0745416
cg22522509	0.013503	3.99E-05	4.566704	0.1473907
cg15595627	1.0482356	0.568969	1.931209	0.8798931
cg13990065	0.0663915	0.010158	0.433925	0.0046314
cg01940743	1.20E-19	1.28E-31	1.13E-07	0.0019537
cg19807420	0.2310777	0.0602789	0.885831	0.0326156
cg01794853	0.3557981	0.1185901	1.067478	0.0652594
cg04316624	2.6658599	0.8315971	8.545976	0.0990033
cg03746015	0.5030387	0.2688733	0.941142	0.0315742
cg26859016	1.4003305	0.4425765	4.430704	0.5666883
cg24808105	0.8352148	0.291843	2.39027	0.7371372
cg06942159	0.5779806	0.1925724	1.734732	0.3282575
cg24502192	0.3069028	0.0482644	1.951528	0.2107352
cg26221111	0.3868119	0.1682584	0.889248	0.02533
cg27097542	2.4177887	0.4576019	12.77464	0.2985723
cg20588193	0.2755113	0.0604995	1.254662	0.0955825

cg23708624	1.2358442	0.4950629	3.085085	0.6500646
cg21964466	2.1630607	0.5998979	7.79938	0.2383769
cg23051248	0.4763859	0.2043879	1.110357	0.0858873
cg03012544	1.7382815	0.8215404	3.677997	0.1482061
cg25180342	1.9347689	0.8281683	4.520012	0.1273917
cg04945350	1.7321958	0.6245415	4.804328	0.2911788
cg14670315	0.6398776	0.2905476	1.409213	0.267695
cg23411932	1.9676611	0.7549227	5.128591	0.1661217
cg03140421	2.2400974	0.7353288	6.824208	0.1558875
cg07904567	0.4279605	0.000198	924.9395	0.828488
cg19863740	1.783853	0.6984505	4.555988	0.2263596
cg05078611	2.9158069	0.7925063	10.7279	0.1073812
cg12595026	4.1054088	0.509328	33.09141	0.1847211
cg17066932	0.6082945	0.2388264	1.549335	0.2973613
cg12262564	0.0380092	0.0031546	0.457961	0.0100255
cg01140247	1.1630853	0.4590062	2.947166	0.7501278
cg21108691	0.0010191	2.51E-06	0.414104	0.0246008
cg15113766	0.7345816	0.3282782	1.643759	0.4528963
cg17173369	2.0070761	0.8109388	4.96752	0.1318787
cg12737110	0.0605246	0.0105115	0.348496	0.0016885
cg26931050	0.6889814	0.3499813	1.356345	0.2810335
cg06635133	0.4139986	0.1968364	0.870747	0.0200817
cg18571412	0.6520584	0.3280958	1.295902	0.2223592
cg06293872	0.3901152	0.1714824	0.887495	0.0247965
cg22023531	0.3005254	0.1387322	0.651006	0.0023012
cg10500474	0.5952739	0.0381005	9.30044	0.7114788
cg21899743	2.8174513	1.2502529	6.349141	0.0124635
cg18540674	1.8368844	0.922317	3.658335	0.0836469
cg14693521	0.5905977	0.1407068	2.478953	0.471805
cg14528319	0.363386	0.1402691	0.9414	0.0371329
cg02675173	0.3699785	0.1615595	0.847267	0.018672
cg00099519	8.0008504	0.9148957	69.9682	0.0601664
cg04016730	0.5612227	0.2763503	1.139753	0.110028
cg25574111	0.1408768	0.05411	0.366777	5.96E-05
cg10012595	0.4105456	0.0956827	1.761529	0.2308993
cg18090004	1.2562082	0.639928	2.465995	0.5074524
cg23038824	11.565898	0.3646388	366.8562	0.1651439
cg17848828	0.1351219	0.0060413	3.022184	0.2068003
cg04880052	1.3097505	0.0473576	36.22324	0.8734288
cg14153637	1.7866494	0.9461596	3.373761	0.0735623
cg12549906	0.4750375	0.1553376	1.452712	0.1918315
cg08612539	3.6986478	1.2741129	10.73688	0.0161509
cg24185055	1.6931519	0.6401481	4.478281	0.2886329
cg00503017	0.574164	0.2265485	1.45516	0.2422528
cg03565868	8.6013944	1.0163656	72.79269	0.0482839
cg02527030	0.8332801	0.2354368	2.949223	0.7773118
cg13400077	1.2785968	0.3993201	4.093983	0.6789418
cg21568145	0.9943259	0.4072142	2.427921	0.9900325
cg13661740	1.2924666	0.7030092	2.37617	0.4089429
cg14790153	0.1046555	0.0002759	39.69654	0.4562992
cg08744878	9.24E-15	4.18E-46	2.05E+17	0.3801905
cg14993900	3.7722746	0.8626476	16.49579	0.0777836
cg19721478	0.4119487	0.13972	1.214584	0.1079277
cg12193289	0.6006448	0.2402827	1.501457	0.2754968
cg03034101	0.8371419	0.42375	1.653821	0.6088449
cg02668822	26.278456	0.4225988	1634.073	0.1208509
cg23305678	0.2125657	0.0556375	0.812117	0.0235575
cg20256494	0.3224195	0.0995584	1.044154	0.0590396

cg03722295	2.3493765	0.8757479	6.302693	0.0898006
cg10806639	0.1421007	0.0200633	1.006447	0.0507568
cg22680075	15.008889	1.4966545	150.5135	0.0212915
cg27629384	0.2314352	0.0812084	0.659565	0.0061659
cg05813032	52.514738	0.4149907	6645.445	0.1087464
cg10887937	0.6417946	0.2756977	1.494029	0.3036181
cg27638913	0.1378174	0.0582811	0.325897	6.39E-06
cg00424169	7.8418247	1.3382934	45.94972	0.0224313
cg03493211	0.2373967	0.1050772	0.536341	0.000544
cg13428921	0.2523134	0.0620024	1.026767	0.0544706
cg25922105	1.7300155	0.3331507	8.983782	0.5142906
cg11521979	0.2926253	0.0741521	1.154784	0.0793459
cg00364304	1.5976912	0.7263821	3.514152	0.2439872
cg01865937	1.8329479	0.8301595	4.047051	0.1337787
cg22360318	1.1743289	0.4924514	2.800375	0.7170411
cg17019053	4.0004774	1.2160874	13.16009	0.0224907
cg01319701	1.3694329	0.5009649	3.743469	0.5400318
cg21829783	1.5155137	0.8094717	2.837384	0.1938211
cg07462863	3.5386983	0.2723755	45.97472	0.3340878
cg07114422	2.8959331	0.4366644	19.20566	0.2706518
cg24446326	4.8763361	0.3612269	65.82748	0.2328089
cg15680288	2.2904687	1.0330591	5.078361	0.0413479
cg19869037	0.3007395	0.1243985	0.727053	0.0076374
cg06306198	1.400459	0.6308821	3.108799	0.4077851
cg22970173	0.2672008	0.0780204	0.915098	0.0356212
cg24688837	2.866637	0.3161125	25.99583	0.3491737
cg26620710	4.0936062	1.6083424	10.41918	0.0031072
cg16695253	2.0470139	0.149193	28.0862	0.5918649
cg14124310	6.2148722	1.3214913	29.22807	0.0207299
cg07994435	0.0965014	0.0198618	0.468866	0.0037423
cg04473302	2.4628943	1.01589	5.97097	0.0460586
cg07010633	0.8653578	0.1722696	4.346932	0.8606073
cg11040181	0.3878341	0.1746796	0.861093	0.0199411
cg11506205	4.4245729	1.315526	14.88138	0.0162568
cg13641082	0.2268873	0.0883859	0.582422	0.0020438
cg16420801	0.3811416	0.1057785	1.373331	0.1402418
cg22488081	0.4621344	0.2181854	0.978838	0.0438187
cg01258201	1.458259	0.5487727	3.875045	0.4493219
cg14092276	0.4733441	0.2330696	0.961321	0.0385376
cg18522715	0.1566915	0.042739	0.574469	0.0051705
cg03948781	0.3518027	0.1265082	0.978317	0.0452876
cg07536847	1.6204658	0.7601231	3.454584	0.2113644
cg02319187	0.4783684	0.0070935	32.25992	0.7314582
cg00218620	3.6934396	1.0536173	12.9473	0.0411937
cg01747263	0.0006218	7.17E-12	53921.78	0.4285539
cg02787967	0.2444996	0.049645	1.20415	0.0833476
cg07830534	5.9194745	0.9142957	38.32478	0.0620492
cg25074751	1.4730825	0.7399034	2.932777	0.2702238
cg08380411	0.0438608	0.0070513	0.272823	0.0007999
cg00474500	2.2506006	0.9493408	5.335495	0.0654868
cg03295928	1.4994815	0.4125854	5.449648	0.538348
cg16539272	1.5562253	0.8613804	2.811577	0.1427833
cg05862431	8.18E-08	9.72E-15	0.688308	0.0448696
cg05409601	0.0009887	9.81E-23	9.96E+15	0.7566215
cg06184463	0.0020608	4.48E-07	9.478334	0.1506328
cg21756647	0.2343937	0.0976906	0.562392	0.0011585
cg21461745	0.2393108	0.0674388	0.849209	0.0269045
cg00633740	0.3526458	0.1645869	0.755583	0.0073442

cg05155840	1.7111534	0.8965833	3.265782	0.1033278
cg08999081	0.328938	0.11832	0.914471	0.0330602
cg16653620	2.1959969	1.0961138	4.399545	0.0264992
cg10762126	3.1839776	0.5407422	18.74778	0.2004405
cg08058560	3.3066401	1.302222	8.396317	0.0118904
cg01439408	1.7269573	0.5596706	5.328816	0.3419236
cg02569236	1.2718464	0.5986433	2.702099	0.5316771
cg01200344	0.3575086	0.1703145	0.750449	0.0065523
cg06748476	6.8177682	1.527706	30.42599	0.011895
cg21503148	1.8476478	0.8296646	4.114678	0.1328792
cg10639981	1.3944673	0.6428724	3.024767	0.3999804
cg14155831	2.4273038	0.4254592	13.84811	0.3182308
cg17911882	3.937E+09	7643.5517	2.03E+15	0.0009931
cg08129759	0.1331687	0.0424468	0.417791	0.0005481
cg18388910	14.739018	1.7158554	126.6066	0.0142057
cg23058405	0.8502123	0.3588664	2.01429	0.7123299
cg18331061	101.25817	7.85E-05	1.31E+08	0.5200616
cg03038245	0.3756231	0.089291	1.580145	0.1816121
cg24134845	1.4453014	0.637695	3.275698	0.3776276
cg21751623	0.2862869	0.1142758	0.717214	0.0076006
cg05741530	2.96E-13	2.70E-22	0.000325	0.0066027
cg03534410	1.2293435	0.3597851	4.200522	0.7418837
cg11893552	1.2068499	0.3856592	3.776615	0.7466842
cg06327227	0.7467275	0.4229816	1.318265	0.313878
cg04545296	0.0503906	0.0072524	0.350118	0.0025187
cg04643397	0.2427082	0.1047014	0.562622	0.0009642
cg25865120	0.8914197	0.3452356	2.301701	0.8122784
cg20177385	0.6329884	0.1553108	2.579822	0.5235235
cg06833110	1.9469617	0.7166846	5.289161	0.1913272
cg05793323	0.2246068	0.0725803	0.695068	0.0095679
cg04249142	0.0035208	3.94E-05	0.314313	0.013702
cg00819788	0.0574775	0.0062893	0.525282	0.0113969
cg02605601	0.594545	0.2467829	1.432367	0.2464531
cg07660838	1.3008231	0.5934578	2.851325	0.5112953
cg19627093	4.2932245	0.9111047	20.23014	0.0654381
cg08839081	139.24468	4.5485965	4262.651	0.0046879
cg03267139	0.4374661	0.095187	2.010532	0.2880282
cg04722215	2.0703758	0.6543409	6.550799	0.2156111
cg24365867	0.2325645	0.0844181	0.640695	0.0047872
cg04126095	10.516289	0.9771847	113.1745	0.0522673
cg26033520	0.2695509	0.1073949	0.676547	0.0052351
cg14162417	1.3564922	0.3817849	4.819654	0.6373797
cg26539818	5.3274123	0.7555218	37.56519	0.0932206
cg10372920	1.4262217	0.4744333	4.287449	0.5272536
cg18009376	6.127E+12	29.578109	1.27E+24	0.0267783
cg13988209	2.4345042	0.8585993	6.902883	0.0942762
cg03884018	0.5219631	0.215611	1.263598	0.149499
cg02625942	1713410.1	2.6678499	1.1E+12	0.0353973
cg08793792	1.4454429	0.659345	3.168759	0.3576051
cg01607849	0.0698097	0.0026145	1.863995	0.1121983
cg24448870	0.4349489	0.0138082	13.70059	0.6362362
cg18835300	0.5242636	0.1689722	1.626613	0.2636424
cg16639713	6.541096	0.0063314	6757.73	0.5958484
cg02649698	3.0404431	1.3599103	6.797724	0.006752
cg08185223	0.459957	0.2328818	0.908446	0.0253205
cg16796959	1.7644661	0.4612387	6.749956	0.4068081
cg06256596	0.36894	0.1282236	1.061557	0.0644316
cg25080973	4.3681399	0.3172209	60.1494	0.2705182

cg23558601	0.5201853	0.1765402	1.532755	0.235864
cg23261097	3.4749447	0.0099999	1207.535	0.676488
cg23912878	35691.909	0.0181692	7.01E+10	0.156234
cg09057885	3.245479	0.1250207	84.25112	0.478609
cg00521255	0.1465707	0.0422518	0.508451	0.0024802
cg01454752	1.6859346	0.8133228	3.494769	0.1602018
cg02242989	0.613809	0.193823	1.943843	0.4066223
cg00247631	1.8432653	0.613214	5.540687	0.276129
cg13232249	0.7060338	0.3613867	1.379364	0.3083386
cg20704442	2.4520019	1.029137	5.842092	0.0428877
cg27503398	0.7699356	0.2887845	2.052744	0.6012851
cg23124325	1.2028161	0.4391507	3.294465	0.7194338
cg07488327	0.2600165	0.0876231	0.771584	0.0152149
cg08657492	0.8805041	0.4044738	1.91678	0.7484851
cg06802630	2.131045	0.9090128	4.995917	0.0817687
cg24473594	0.1608596	0.0668913	0.386834	4.48E-05
cg08473764	0.239111	0.0059452	9.616889	0.4477943
cg07159397	0.9212112	0.450794	1.882523	0.8219318
cg08632081	3.4003729	0.724586	15.95744	0.120768
cg05643286	1.0874927	0.4992118	2.369015	0.8327801
cg05075562	1.8105168	0.7009896	4.676205	0.2201438
cg14032033	0.5338486	0.2152334	1.324117	0.1756665
cg13725172	0.323693	0.1080656	0.96957	0.0438864
cg08259462	0.1919876	0.0569408	0.647326	0.0077843
cg21191241	0.3702596	0.1601445	0.856053	0.0201565
cg25851152	1.5721915	0.6575277	3.759212	0.3090075
cg06721393	1.8330304	0.9624303	3.491162	0.0652607
cg00306578	0.4535484	0.1504101	1.367635	0.1603172
cg13947999	0.180996	0.0507543	0.645454	0.008418
cg25515801	0.0002552	4.68E-12	13904.45	0.3626598
cg22286906	0.0787849	0.0203299	0.305317	0.0002364
cg18563413	0.1727319	0.0642665	0.464259	0.0004994
cg12392429	2.816709	1.1070559	7.16662	0.0297489
cg03862290	0.589922	0.3158754	1.101726	0.0977244
cg00776960	1.3996485	0.3489431	5.614141	0.6352108
cg13519696	0.3669962	0.1431032	0.941182	0.036968
cg03901958	0.0005659	1.48E-06	0.216228	0.0137094
cg03907390	1.9020875	0.6467451	5.594069	0.2427406
cg06948492	1.0269107	0.5430809	1.941784	0.9348857
cg24692177	0.108132	0.0157184	0.743877	0.0237798
cg09354556	0.1885896	0.0555676	0.640049	0.0074583
cg17170758	2.0604047	1.1188856	3.794193	0.0203106
cg20321319	2.7040049	0.7538338	9.699277	0.1269209
cg17658266	0.3899817	0.1390913	1.093423	0.0734264
cg11938718	1.8713725	0.6446622	5.432357	0.2491024
cg25259754	0.8845554	0.3729628	2.097899	0.7807045
cg02178898	63.699815	0.0069249	585957	0.3723387
cg00559635	0.1695002	0.0652133	0.44056	0.0002706
cg14892570	0.0574222	0.0091831	0.359063	0.0022497
cg13619044	0.3862024	0.1461925	1.020246	0.054918
cg18175606	0.2145675	0.0393418	1.170237	0.0753502
cg12052265	1.051355	0.6123111	1.805206	0.8559218
cg23270841	0.447952	0.1903375	1.054237	0.0659133
cg24421410	3.1238938	1.1440741	8.529791	0.0262435
cg19645788	0.2425069	0.0656411	0.895926	0.0336043
cg14305239	0.0854987	4.16E-17	1.76E+14	0.8912617
cg00435526	0.0032846	3.62E-06	2.983283	0.099876
cg15750500	0.6808843	0.3574921	1.296821	0.2422941

cg07314530	0.4570572	0.2027298	1.030442	0.059071
cg02704158	0.9505842	0.4275535	2.113444	0.9010652
cg07781701	0.7805425	0.1686981	3.611461	0.7512401
cg13502540	2.0229389	0.649982	6.295992	0.2238859
cg08760829	0.0226714	7.09E-05	7.246526	0.1981338
cg24746594	0.1654514	0.0309401	0.884746	0.0354559
cg17848848	0.0355435	0.0046408	0.272224	0.0013155
cg06814287	0.5017354	0.1953915	1.28838	0.1517559
cg10330885	93.086218	7.176521	1207.416	0.0005258
cg08372212	0.4939429	0.2150876	1.134327	0.0963471
cg19237113	1.5294617	0.6328287	3.696503	0.3453047
cg09689137	0.5185785	0.254639	1.056097	0.0703644
cg08739841	0.934761	0.0429935	20.32351	0.965748
cg02501882	0.1480879	0.0358178	0.612266	0.0083543
cg03431524	3.6562655	0.9519229	14.04345	0.0589987
cg07113653	1.7537851	0.775021	3.968618	0.1775684
cg19734216	0.6490285	0.3423901	1.230287	0.185234
cg10511850	0.4933498	0.219689	1.107902	0.0869492
cg06385583	1.03864	0.5694272	1.894488	0.9016078
cg20382867	0.2652395	0.0430912	1.632628	0.1523457
cg07802350	1.7647933	0.781238	3.986615	0.1718775
cg17811919	3.9934428	0.9853804	16.18419	0.0524604
cg26365545	1.9059645	0.6223565	5.837009	0.2586934
cg04586579	1.7994551	0.9630393	3.362312	0.0654915
cg09230996	1.4957053	0.7341689	3.047166	0.2674924
cg04609841	3.5437626	1.7953771	6.994772	0.0002655
cg21182407	0.0024921	3.44E-05	0.180666	0.00609
cg24603464	0.1458087	0.0172741	1.230757	0.0768628
cg20758929	0.1974038	0.0034988	11.13748	0.4303795
cg17645823	2.0705914	0.9100604	4.71106	0.0826929
cg15805568	0.017157	0.0011451	0.257073	0.0032452
cg27087809	0.3412485	0.1464119	0.795363	0.0127642
cg16945312	2.1615613	1.0757542	4.343322	0.0303828
cg08261525	1.0925446	0.5235493	2.279926	0.8135742
cg23926866	0.0845194	0.0170589	0.418757	0.0024777
cg16732939	0.5644487	0.2611594	1.219953	0.145842
cg14855519	1.3451484	0.6661051	2.716425	0.4083079
cg07990111	0.1850486	0.0562391	0.608882	0.0054962
cg02348751	1.6427893	0.8952199	3.01463	0.1090198
cg00404641	1.8072525	0.5844706	5.58824	0.304176
cg20068274	0.2564218	0.1156739	0.568427	0.0008059
cg01170045	2.5673508	0.897926	7.340572	0.0785619
cg00139242	4.2635691	1.3465273	13.49993	0.0136664
cg17834768	1.568078	0.7771026	3.164149	0.2091485
cg09555544	1.2920008	0.5506144	3.031643	0.5560488
cg06588802	3.2687493	1.0830131	9.865737	0.0356009
cg14143954	0.4829629	0.2010461	1.160198	0.1035967
cg01329005	1.0358903	0.494332	2.170745	0.9255722
cg00692780	1.2063672	0.4784982	3.041437	0.6908864
cg14518261	1.5220091	0.4355775	5.318253	0.5105313
cg07938743	2.8590501	0.8384173	9.749521	0.0932722
cg02200642	0.5474313	0.2780142	1.077934	0.0813545
cg20955836	1.9184914	0.9553704	3.852547	0.0670088
cg11261667	1.1565353	0.548871	2.436955	0.7021404
cg18432145	0.1975986	0.0193734	2.015404	0.171156
cg00105102	0.3948722	0.1676572	0.930017	0.0335065
cg05919661	122677121	3.65E-15	4.12E+30	0.4815583
cg23979832	0.3549334	0.1567423	0.803725	0.0129943

cg01768814	1.4448972	0.6367396	3.278778	0.3787001
cg18756771	1.5359111	0.6353327	3.713051	0.3406904
cg08372947	1559.2902	0.0230741	1.05E+08	0.1950756
cg02297541	0.1105505	0.0332352	0.367725	0.0003289
cg00930618	1.869341	0.3301307	10.58501	0.4794618
cg19195724	0.5258597	0.1588802	1.740484	0.2925745
cg21506790	0.7152781	0.3580133	1.429061	0.3426586
cg15121304	8.2858041	0.6837836	100.4039	0.0966488
cg08509270	0.3418497	0.1064554	1.097748	0.0713434
cg02800571	0.4509388	0.0661371	3.074609	0.4161208
cg11496593	0.1997615	0.0868574	0.459427	0.0001505
cg03408433	1.383224	0.6745582	2.836388	0.3759204
cg04730794	0.3962077	0.1634587	0.960369	0.040415
cg03625260	2.1637015	1.0862904	4.309717	0.0281353
cg12513284	6.54E-08	1.49E-19	28600.2	0.2264084
cg26942124	0.478078	0.1476427	1.548052	0.218317
cg14285533	0.1626262	0.0530099	0.498913	0.0014948
cg20945738	1.4139801	0.8117035	2.46314	0.2212289
cg00963675	2.2899483	0.5524771	9.491549	0.2534221
cg26188543	2.0916162	0.3599929	12.15262	0.4110988
cg15239904	1.8643746	0.7761313	4.478486	0.1635703
cg11584284	0.5838623	0.2816602	1.210307	0.1479636
cg15669092	0.000141	5.20E-10	38.21389	0.1647804
cg04005938	8.0076345	1.1814353	54.27484	0.0331116
cg09860601	8.3434667	1.0237046	68.00149	0.047496
cg14486857	0.2525113	0.1104512	0.577286	0.0011053
cg01558931	1.4971865	0.7048161	3.180358	0.2937541
cg00770693	1.3075112	0.4667336	3.662872	0.6099468
cg02473606	0.1354281	0.0233152	0.786646	0.0259267
cg00411097	1.3747667	0.4667424	4.049308	0.563618
cg02134353	1.601993	0.8229635	3.118463	0.1655514
cg17822955	0.212803	0.0670159	0.675737	0.0086692
cg01709766	0.4582172	0.2038154	1.030162	0.0590166
cg11599830	0.2492824	0.0613796	1.012416	0.0520516
cg05971474	6.38E-09	2.93E-17	1.389153	0.0540535
cg16639880	1.6728691	0.7820536	3.578388	0.1847409
cg04885832	0.8880939	0.4575614	1.723727	0.7257774
cg03747342	0.4001418	0.1623533	0.986204	0.0465746
cg21617357	0.4321082	0.2035198	0.917441	0.0289423
cg14276730	0.5796122	0.2641813	1.271666	0.17368
cg24469980	1.5596811	0.7054224	3.448438	0.2722207
cg21244086	0.3946187	0.1659322	0.938479	0.0354124
cg16576213	4.394828	1.1492432	16.80629	0.0305239
cg22042546	0.3186561	0.1026926	0.988793	0.0477629
cg26878709	0.3956969	0.1960348	0.798715	0.0096775
cg19216211	0.1970687	0.0737012	0.52694	0.0012093
cg07519816	1.5389411	0.7615915	3.109725	0.2296966
cg11006288	1.88E-06	1.23E-13	28.87487	0.1183861
cg12854428	0.5242251	0.2281701	1.204417	0.1280795
cg14397309	0.3116032	0.1427229	0.680315	0.003424
cg01866431	1.5926668	0.7802058	3.251178	0.2011525
cg13732865	3.0779299	0.9225114	10.26942	0.0674346
cg14151426	117.64285	1.41E-16	9.81E+19	0.8208506
cg07968906	2.1211311	0.1390228	32.36303	0.5886258
cg03688699	3.2107691	1.3048062	7.900819	0.0111148
cg10148841	2.1437515	0.4054108	11.33584	0.3694913
cg27539046	0.780491	0.3897704	1.562885	0.4842096
cg08036502	0.1019042	0.0120375	0.862675	0.0361259

cg12531601	5.34E-08	3.47E-16	8.239077	0.0817311
cg19647567	3.4894646	0.731864	16.63747	0.1168231
cg21534623	2.28E+15	7.68E-10	6.76E+39	0.2186988
cg25425667	1.9026369	0.5185514	6.981038	0.3321345
cg04561261	0.4922923	0.2317561	1.045719	0.0652322
cg03600259	1.6496263	0.6307812	4.314122	0.3074889
cg02925222	0.1111218	0.0146971	0.84017	0.0332799
cg05272807	0.250283	0.0781323	0.801738	0.0197016
cg02282640	0.0817321	0.0019787	3.375995	0.187137
cg02582057	2.072769	0.9030716	4.757509	0.0855317
cg00066805	2.0882734	0.8378484	5.204862	0.1140439
cg04977602	0.149631	0.0550612	0.406628	0.000196
cg01109219	3.1903206	0.4015447	25.34748	0.2725992
cg10244594	0.5141226	0.1857482	1.423013	0.2002614
cg23588605	0.328236	0.1243937	0.866112	0.0244286
cg05166490	0.268701	0.0586518	1.230999	0.0905827
cg01046070	0.5471924	0.1905133	1.571646	0.2626807
cg22276371	2.323243	0.9756118	5.532383	0.0568854
cg06002157	4.3270563	0.9832399	19.04257	0.0526711
cg02458152	6.6955244	0.6808294	65.84623	0.1030309
cg20537886	0.3522173	0.0865238	1.433791	0.1451444
cg15892963	0.0969965	0.0125234	0.751257	0.0254962
cg05753993	1.9930248	0.5123713	7.752479	0.3196902
cg08130814	0.5462633	0.1716795	1.738144	0.3058969
cg12635919	0.4467215	0.1344881	1.483849	0.1882933
cg22467129	0.0053792	0.0001001	0.289038	0.0101529
cg07786675	0.4992704	0.101013	2.467711	0.3942154
cg16545105	7.7143279	0.4666673	127.5231	0.1534447
cg06957622	0.6679165	8.57E-19	5.21E+17	0.984681
cg08181212	0.8097938	0.3670168	1.786746	0.601311
cg21407899	0.9888871	0.4308285	2.269807	0.9789691
cg13200838	1.4926863	0.7321908	3.043076	0.2703568
cg08360229	3.8287911	0.9791232	14.97221	0.0536514
cg05698078	1.5195563	0.7264266	3.178644	0.2664944
cg11201532	1.3491263	0.5926833	3.071019	0.4755106
cg03399199	0.0222013	0.0006308	0.781332	0.0361014
cg08969855	0.2730012	0.1258334	0.592289	0.0010185
cg16126760	0.5165169	0.2135477	1.249321	0.1426472
cg10189695	0.1644041	0.0535767	0.504486	0.0015994
cg06887454	0.3369919	0.1623581	0.699463	0.003508
cg04080041	2.3388577	0.8596102	6.363646	0.0961632
cg06468920	4.0921381	0.7663048	21.8524	0.0992399
cg04876582	1.2226712	0.4834067	3.092479	0.6711078
cg24109477	7.3313791	0.0429006	1252.874	0.4475587
cg05681560	5.3987702	0.9740574	29.923	0.0536217
cg26042300	0.2657833	0.0739651	0.955055	0.0423129
cg06245229	0.0427955	0.0013445	1.362213	0.0742798
cg25445931	2.9177797	0.5726438	14.8669	0.1974243
cg22721765	3.14E+13	8.14E-08	1.21E+34	0.1987942
cg26856527	2.6524157	1.1012636	6.388397	0.0296271
cg12595697	0.4042876	0.0610845	2.675778	0.3476186
cg09185884	0.5046754	0.2718111	0.937038	0.0303157
cg06737912	0.1672094	0.0630758	0.44326	0.0003236
cg10138494	0.3501127	0.0680841	1.800403	0.2090564
cg07977202	1.6529911	0.4166751	6.557578	0.4747183
cg22481960	0.3738662	0.0427932	3.266311	0.3736558
cg05188158	0.0002519	7.76E-11	817.1593	0.2786708
cg12963560	0.2363357	0.100015	0.558462	0.0010099

cg23647157	1.3798709	0.456561	4.170404	0.5682757
cg23527418	0.3642565	0.1831089	0.724611	0.0040032
cg03434384	0.3765731	0.1174942	1.20693	0.1002861
cg01402409	0.8506647	0.3024067	2.392905	0.7592218
cg02122920	1.7149639	0.129773	22.66342	0.6821381
cg16753167	2.3138706	0.8634616	6.20062	0.0953031
cg08138366	5.6494683	0.0021332	14962.13	0.6667656
cg02452840	0.0489012	0.0001657	14.43585	0.2983477
cg11877647	0.4820634	0.2350232	0.988776	0.0465072
cg03894068	0.2495522	0.0813217	0.765801	0.0152499
cg23125506	0.2991878	0.1000551	0.89464	0.0308363
cg26997028	3.5068381	1.6090297	7.643062	0.0015966
cg17742359	16.313309	0.4822311	551.86	0.1201802
cg25955565	0.2205647	0.01904	2.555088	0.2265087
cg19098437	0.3141938	0.1548342	0.63757	0.0013433
cg05142982	0.1978182	0.0373815	1.04683	0.0566336
cg04193065	0.2222713	0.0569156	0.868032	0.0304968
cg17843418	0.3708229	0.1752108	0.784824	0.0095039
cg26130864	2.1021568	0.4754166	9.295139	0.3272903
cg23275914	0.8726793	0.3137642	2.427202	0.7941392
cg00307818	2.4535812	0.6346729	9.485296	0.193269
cg12426802	0.4532525	0.1965232	1.045362	0.0634655
cg16327282	2.7830098	1.2104184	6.398733	0.0159735
cg18776287	0.0588897	0.0107945	0.321273	0.0010692
cg16148365	1064.5269	0.0144406	78474191	0.2228793
cg01474424	0.4619154	0.1252645	1.703323	0.2460248
cg00746386	1.9829245	0.5096046	7.715766	0.3233872
cg03436208	1.5724884	0.6168229	4.0088	0.343115
cg06935438	0.9013405	0.3830568	2.120873	0.8119452
cg25560443	0.6924517	0.3132246	1.530816	0.3638869
cg01089395	2.0805394	0.3261284	13.27282	0.4384107
cg03032512	0.142278	0.0313086	0.646564	0.011585
cg17339488	1.845676	0.9349507	3.643529	0.0773746
cg13556639	0.2327753	0.073186	0.740365	0.0135426
cg05428864	0.020009	0.0017848	0.224319	0.0015135
cg19215199	2.4529781	0.2910439	20.6742	0.4093384
cg01385669	0.2944202	0.0925554	0.936555	0.0383605
cg08897854	1.423E+15	9.47E-16	2.14E+45	0.3250224
cg16283385	0.5336157	0.11348	2.509215	0.4264956
cg06394058	2.1108647	0.684021	6.514054	0.1937954
cg06291350	1.7127683	0.649653	4.515603	0.2766221
cg02566627	1.403139	0.4894325	4.022616	0.5284871
cg04209035	0.1410523	0.0123806	1.607015	0.1146076
cg16204357	1.3388202	0.6544842	2.738705	0.4242468
cg02147465	0.1444208	0.0102964	2.025693	0.1509815
cg02277819	0.0027941	3.77E-18	2.07E+12	0.7364153
cg06555468	0.1385564	0.0264129	0.726837	0.0194259
cg10228162	0.3999101	0.196534	0.813743	0.0114514
cg13680246	0.5057818	0.2615484	0.97808	0.0427817
cg25189074	0.3100442	0.1153102	0.833642	0.020313
cg05629931	2.7841884	1.0746979	7.212916	0.0350058
cg06973615	2.3651208	0.865564	6.462603	0.0932572
cg05096777	0.3505177	0.1348013	0.911435	0.0315423
cg26175729	0.3960471	0.1329053	1.180189	0.0963972
cg04873577	0.634242	0.1650958	2.436542	0.50729
cg17101296	0.2109934	0.0575744	0.773229	0.0188705
cg12494515	2.9172912	0.8538449	9.967369	0.0876525
cg16503006	1.1684924	0.4001891	3.411823	0.7757815

cg00047338	14.44908	0.3791333	550.6663	0.1504883
cg06915826	3.050164	0.6115269	15.21356	0.1737838
cg18681287	0.0617134	0.0015789	2.412139	0.1364393
cg21063722	1.9484309	1.0797292	3.516051	0.0267836
cg20025086	0.9894621	0.3138213	3.119722	0.9855739
cg03374976	0.0777712	0.021095	0.286721	0.0001248
cg01074659	3.2935977	1.3453433	8.06321	0.0090713
cg00386007	0.7666866	0.3457209	1.700239	0.5132397
cg21786191	0.4229243	0.1591259	1.124047	0.0844377
cg21633052	1.3035151	0.3634737	4.674758	0.6841621
cg14329989	1.0029442	0.4245654	2.369239	0.9946519
cg00552318	2.6079992	0.8036107	8.463874	0.1105002
cg14304073	0.2153147	0.0009647	48.05445	0.5778335
cg24251193	0.3782511	0.1807038	0.79176	0.0098945
cg13291570	1.426604	0.639488	3.182545	0.385463
cg16279541	0.075782	0.0014589	3.936407	0.2005191
cg06103394	4.1412807	0.0812964	210.9591	0.4785958
cg08830105	0.2385954	0.1039371	0.547714	0.0007252
cg20451050	3.7477731	1.1179689	12.56368	0.0323025
cg02994246	0.348165	0.1394746	0.869111	0.0237886
cg06121323	0.091241	2.03E-11	4.1E+08	0.8327774
cg12555306	0.019855	8.71E-05	4.525721	0.1570932
cg06971773	0.2612802	0.0732303	0.932228	0.0386307
cg04352304	2.4475929	1.1834757	5.061964	0.0157641
cg08325885	0.1480395	0.0184782	1.186032	0.0719768
cg14190534	0.6662919	0.3023615	1.468259	0.3138351
cg22161562	0.2546454	0.0660149	0.982267	0.04704
cg11305134	0.7226213	0.3189507	1.637186	0.4362468
cg16573136	1.4829665	0.5516039	3.986901	0.4348455
cg21774827	11.610898	1.6739554	80.53557	0.0130894
cg09091766	0.154298	0.0403298	0.59033	0.0063361
cg17498476	0.0833119	0.0016322	4.252468	0.2155092
cg00330170	0.1442201	0.0175099	1.187868	0.0718705
cg07931190	911414480	0.0012784	6.50E+20	0.1384641
cg12700145	1.7311025	0.5885013	5.092114	0.3188325
cg21834604	0.5117385	0.2185227	1.198394	0.1228064
cg26082838	0.8751709	0.2937695	2.607228	0.8107959
cg18144710	0.6621482	0.3439449	1.27474	0.2173473
cg24343913	0.4807584	0.2464407	0.937867	0.0317052
cg19418525	0.4209126	0.1853286	0.955964	0.0386806
cg04006327	0.2019018	0.0706149	0.577277	0.0028355
cg16771652	2.7326951	0.8973449	8.321909	0.0768392
cg21217070	1.4183138	0.3919529	5.132286	0.594322
cg27327357	16453.001	5.10E-22	5.31E+29	0.7459717
cg12565681	2001.8126	8.5387317	469303.2	0.0063295
cg16659728	0.5991694	0.2659483	1.349901	0.216466
cg18642271	1.3461119	0.5269133	3.438929	0.534543
cg14074042	122.70971	1.0706054	14064.63	0.0467935
cg23189739	0.0242893	0.0007612	0.775016	0.0353592
cg09907219	0.1687707	0.0512495	0.555782	0.0034346
cg20311631	1.7491182	0.6292856	4.861727	0.2837408
cg13715631	1.5947084	0.8506886	2.989454	0.145504
cg07723558	3.37E-14	2.30E-28	4.946413	0.0623345
cg25350306	4.6756637	0.8990625	24.31625	0.0667314
cg24256946	3.27E-12	1.51E-23	0.70622	0.0470239
cg09802339	1.4992666	0.0002702	8320.248	0.9266461
cg00829136	1.4897858	0.3399793	6.528227	0.5969435
cg22459052	2.5315374	0.7301806	8.776844	0.1431302

cg04557197	1.5288071	0.7056016	3.312423	0.2819123
cg09657673	0.4137419	0.1932423	0.885843	0.0230845
cg00696323	0.3919359	0.1558593	0.985593	0.0465019
cg03412153	1.5635582	0.5237415	4.667788	0.4231513
cg08256939	1.9980672	0.5930368	6.731913	0.2640464
cg06705004	4.04E-05	3.85E-07	0.004243	2.04E-05
cg07324054	0.3880597	0.1392427	1.081495	0.0702728
cg27471989	2.8199959	0.9476897	8.39133	0.0624068
cg17054060	1.5687484	0.8275711	2.973728	0.167603
cg06120492	0.3003399	0.018985	4.751336	0.3932249
cg10055501	1.9531931	1.0224502	3.731197	0.0426427
cg14397690	0.9962943	0.432342	2.295873	0.9930455
cg03652676	2.0696096	0.9610255	4.456993	0.0631133
cg13400003	3.703E+09	361.76383	3.79E+16	0.0074669
cg15069471	1.5530056	0.3855485	6.255573	0.535765
cg23249399	14.334687	0.0722466	2844.193	0.3239037
cg03479289	0.8725605	0.1385506	5.495188	0.8845563
cg07630255	2.1557579	0.4973933	9.343294	0.3046071
cg24565274	0.4402648	0.1214163	1.596434	0.2119458
cg14633820	1.52E-06	3.51E-11	0.066127	0.0139512
cg21047367	33.46209	1.2329065	908.1885	0.0371349
cg25453664	1.0524944	0.5390227	2.055098	0.8808785
cg18673377	0.25996	0.1024039	0.659928	0.0045915
cg18594332	1.2814487	0.4683469	3.506184	0.6291688
cg22306579	0.046366	0.010115	0.212536	7.70E-05
cg21584691	1.7140219	0.0981128	29.94381	0.7119727
cg21448513	0.0958415	0.0171971	0.534136	0.0074638
cg11801524	0.6477672	0.3429878	1.223374	0.1807354
cg21012737	1.7262272	0.7461877	3.993446	0.2020326
cg12065272	2062635.5	27.488334	1.55E+11	0.0111319
cg13762060	1.5449576	0.3063094	7.792428	0.5982755
cg14377416	1.5351683	0.4122761	5.716416	0.5228108
cg08035555	0.1750225	0.0335207	0.913849	0.0387531
cg00409816	2.4949443	0.6253896	9.95339	0.1952937
cg09591072	0.6881175	0.3602158	1.314506	0.2576786
cg07406498	1.5597445	0.5596011	4.347388	0.3953511
cg11076902	2.2596199	0.7398045	6.901664	0.152443
cg17215278	0.2735464	0.1011367	0.739867	0.0106664
cg17977409	0.0013591	1.19E-05	0.15572	0.0063577
cg08631819	1.9014954	0.554028	6.526177	0.307072
cg01922437	3.2540579	1.1640771	9.096385	0.0244721
cg24009722	1.5042892	0.7725411	2.929147	0.2297754
cg27016579	1.0236659	0.0456376	22.96118	0.9882405
cg08677210	0.4540712	0.2243373	0.919066	0.0281949
cg05349042	0.0158887	0.0001309	1.927872	0.0906742
cg17179195	6.9187919	1.5449051	30.98552	0.0114526
cg01620672	0.3466434	0.1041072	1.154211	0.084297
cg01156844	1.8400665	0.2376398	14.2478	0.5592671
cg00044871	4.0668786	1.3341597	12.39694	0.0136274
cg08605016	24.319649	0.7829273	755.4281	0.0687022
cg14552508	2.8615863	1.2217327	6.70251	0.0154713
cg04706044	0.4159551	0.1703496	1.015668	0.0541254
cg26998693	6673.1323	0.0025577	1.74E+10	0.2427374
cg13730600	2.6523876	0.9891904	7.112038	0.0525785
cg23119977	0.5941308	0.161209	2.189651	0.4340221
cg11130630	0.5309689	0.2498112	1.128564	0.0998518
cg12354382	0.3978235	0.165583	0.955796	0.0392976
cg08857479	2.0770157	0.9854426	4.377723	0.0546794

cg03394722	0.4127953	0.1636642	1.041156	0.060859
cg20669834	0.4653701	0.2134449	1.014638	0.0544269
cg15160263	2.5416828	1.0907731	5.922544	0.0306743
cg00279755	0.4623359	0.2377059	0.899239	0.0230342
cg10761558	1.1225159	0.3288916	3.831177	0.8536047
cg12862231	2.0946957	0.7392309	5.935561	0.1641052
cg10518282	0.4482158	0.2085485	0.963312	0.0398101
cg15698795	1.7625754	0.5036137	6.16876	0.3752094
cg17707274	1.3875454	0.6928001	2.778987	0.3553401
cg23168425	0.2710071	0.0898986	0.816974	0.020394
cg18159860	2.5127521	0.8991786	7.021879	0.0788707
cg04944527	1.8599588	0.6465987	5.350222	0.2496792
cg18128914	0.2584138	0.0853466	0.782429	0.0166642
cg15739717	1.1827895	0.6152709	2.273781	0.6146574
cg10704263	2.0027325	0.7243536	5.537265	0.1807396
cg09420761	2.64E-08	3.74E-17	18.55719	0.0931609
cg17124430	14.248036	0.0182779	11106.69	0.4342334
cg10453419	2.3526503	1.1971947	4.623278	0.0130599
cg27247510	3.7389674	0.0046647	2996.951	0.6990743
cg23141851	1.2079173	0.505994	2.88356	0.6704781
cg22392857	1.2569323	0.4395309	3.594466	0.6697018
cg06965803	0.4591943	0.1115743	1.889857	0.2809491
cg00180762	0.2370691	0.103281	0.544163	0.0006854
cg22035980	15040.503	1.473517	1.54E+08	0.0411244
cg12226009	1.1288607	0.5831069	2.185408	0.7191286
cg20693854	0.2570467	0.0793718	0.832449	0.0234619
cg08799766	7.0333518	0.0060813	8134.411	0.5877796
cg22563937	0.4459856	0.1806001	1.101346	0.0800025
cg23415756	0.4008924	0.0754406	2.130348	0.2834736
cg22781236	2.027E+09	6.7008662	6.13E+17	0.0314849
cg16742873	3.4682412	0.868552	13.84914	0.078328
cg02885464	2.5953852	0.9155226	7.357573	0.0728213
cg03153765	0.5853817	0.3105077	1.103585	0.0978663
cg17032646	1.2749445	0.4218045	3.853642	0.6668993
cg06704122	0.2890221	0.0610086	1.369214	0.1178138
cg23715667	0.0898843	0.0140853	0.573591	0.0108416
cg17062171	0.3996579	0.1563142	1.021829	0.0555081
cg00356131	2.4482094	1.1189768	5.356437	0.0250018
cg03991297	0.2466283	0.1009611	0.602465	0.0021267
cg27519145	0.7028049	0.2394798	2.062532	0.520844
cg07787634	2.3923729	1.0015121	5.714807	0.0496038
cg00310139	0.4034797	0.164833	0.987641	0.0469012
cg12126038	0.3133833	0.0884397	1.110463	0.0722344
cg17711365	1.6255967	0.787355	3.356256	0.18898
cg08813545	28.573848	2.7790918	293.7884	0.004808
cg10192713	0.6948427	0.3408546	1.416458	0.3164047
cg26779406	0.1597272	0.0543994	0.46899	0.0008446
cg10440939	251.59412	3.85E-14	1.64E+18	0.7660721
cg04251368	1.8038176	0.925381	3.516128	0.0832306
cg24783785	0.1665946	0.0567489	0.489063	0.0011074
cg18722086	0.531698	0.2480411	1.139742	0.1044319
cg23109606	0.1586948	0.043496	0.578996	0.0053123
cg01612366	0.6018247	0.2494142	1.452175	0.2585307
cg00008544	11.193384	0.4675304	267.9866	0.1360355
cg18476993	0.0574461	0.0085896	0.384191	0.0032127
cg11897120	0.493903	0.1065647	2.289128	0.3673013
cg14652773	1.6556786	0.8136667	3.369035	0.1642052
cg25397029	4.5524147	0.6475791	32.00301	0.1276917

cg24202668	0.7268231	0.3383326	1.561398	0.4134443
cg18570853	0.2013043	0.0691613	0.585926	0.0032754
cg03989068	0.7298918	0.2825103	1.885744	0.5155939
cg15393297	0.9201204	0.4287569	1.974596	0.8307974
cg24990327	0.7214359	0.3821284	1.362028	0.3139226
cg26040060	0.3323885	0.1116907	0.989179	0.0477579
cg11888381	0.0018763	1.00E-05	0.351274	0.01868
cg27024950	0.2559628	0.0928125	0.705906	0.0084674
cg14868466	1.9729469	0.5717262	6.808363	0.2822552
cg18304052	0.9549958	0.5169768	1.764135	0.8830833
cg24911545	1.6335275	0.0565061	47.22342	0.7749487
cg17173498	0.1410399	0.0207182	0.960134	0.0453347
cg10941445	0.3250018	0.1142729	0.924333	0.0350738
cg13104274	1.7785827	0.6837407	4.626544	0.2377889
cg25303831	2.5673728	0.9348309	7.050904	0.0673658
cg04876454	2.11E-19	2.89E-42	15477.42	0.1094166
cg25232942	0.6626888	0.3151091	1.393665	0.2780097
cg19466180	1.6976215	0.2733755	10.54198	0.5700267
cg12088314	3.1988863	1.3115931	7.801866	0.0105807
cg00428709	0.9640281	0.5247996	1.770867	0.9060068
cg11287888	11.115156	2.4093671	51.27765	0.0020203
cg07464974	2.9626874	0.7820348	11.22395	0.1100012
cg20237595	1.1630062	0.5530344	2.445749	0.6905113
cg20426713	0.4950048	0.240384	1.019327	0.0563882
cg25956344	0.34521	0.155104	0.768323	0.0091716
cg02430430	0.20545	0.0551654	0.765149	0.0183252
cg08264335	5.2138365	0.0001931	140753.4	0.7510929
cg18686206	1.5868424	0.8669928	2.904371	0.1343446
cg00332961	1.9233442	0.6830098	5.416105	0.215634
cg20490015	0.2885019	0.0738441	1.12715	0.0738056
cg10135708	0.5435833	0.2700272	1.09427	0.0877106
cg25889711	3.6865665	1.5541464	8.744847	0.0030718
cg16322276	0.6557175	0.2012061	2.136941	0.4838354
cg14707834	2.1330327	0.8066914	5.64011	0.1267691
cg08894744	1.9765991	0.7494553	5.213045	0.1684867
cg14608020	1.7522313	0.8659507	3.5456	0.1188239
cg12580870	2612112.5	2.17E-08	3.14E+20	0.3717255
cg07708487	0.274931	0.06807	1.110432	0.0698478
cg00814898	0.4591735	0.2386304	0.883543	0.0197674
cg00919971	1.2358894	0.6004443	2.543821	0.5652692
cg04967982	3.7801348	1.4066759	10.15829	0.0083761
cg12245706	0.3150641	0.1430626	0.69386	0.0041399
cg25065716	1.6131624	0.8249613	3.154442	0.162235
cg24637974	0.679357	0.3481848	1.32552	0.2569463
cg20396299	1.7720238	0.6710471	4.679356	0.248179
cg19426128	0.413584	0.0730227	2.342446	0.3183298
cg20071227	0.8550767	0.4424565	1.652492	0.6413931
cg07239293	1.1420334	0.533764	2.443477	0.7321784
cg10780890	1.4021634	0.7202378	2.729741	0.3199987
cg08415434	0.2743689	0.0025032	30.07258	0.5894229
cg18921771	1.231304	0.7019229	2.159937	0.4680558
cg06343669	0.2771175	0.0781638	0.982477	0.0468847
cg11800379	1.6114323	0.6329052	4.102848	0.3170068
cg10097651	0.9784399	0.353221	2.710327	0.9665559
cg20806712	5.3168808	6.56E-09	4.31E+09	0.8731561
cg12011522	1.1522915	0.5182617	2.561979	0.7280579
cg00725635	0.1988187	0.021607	1.829449	0.1537097
cg00275126	0.8480713	0.3893523	1.847234	0.6782224

cg20281678	0.0048212	6.81E-12	3413287	0.6078827
cg21812476	0.3270427	0.1442262	0.741592	0.0074583
cg01978937	1.6113894	0.6408653	4.051672	0.3105044
cg23213951	2.463528	0.7793915	7.786806	0.1246645
cg00876678	0.1306988	0.0235301	0.72597	0.0200167
cg19416795	2.5835919	1.1759143	5.676389	0.0181053
cg14120112	80.955297	0.7286097	8994.885	0.0675158
cg05324273	8.8360696	0.5502767	141.8852	0.1239876
cg19577312	0.1273108	0.0377511	0.42934	0.0008899
cg12524168	2.2746139	0.6826806	7.578754	0.1807924
cg24996440	0.2031644	0.0532096	0.775721	0.0197277
cg11871064	0.3599079	0.1660939	0.779882	0.0095951
cg25463688	0.2255606	0.0855922	0.594418	0.0025944
cg14342707	2.8954152	0.4806779	17.44085	0.2458907
cg09444060	0.1967759	0.0107383	3.605853	0.2732513
cg24757533	0.1006879	0.0322433	0.314424	7.77E-05
cg03739354	1.2810489	0.7315096	2.243424	0.3862928
cg15407213	0.4866918	0.2149725	1.101857	0.0841128
cg24625984	0.3611012	0.1321526	0.986693	0.0470239
cg08378342	0.0127337	0.0002974	0.545163	0.022818
cg23916679	0.752781	0.3815139	1.485343	0.412805
cg09384159	3.01E+15	3.29E-19	2.76E+49	0.3717056
cg08735550	0.3507842	0.1454172	0.846183	0.0197156
cg25962699	1.1140455	0.4844937	2.561638	0.7993292
cg27638428	1.5851442	0.5127367	4.900531	0.4237249
cg13618111	7.4904197	0.8963831	62.59198	0.0630303
cg11753100	0.2327898	0.0963835	0.562245	0.001196
cg12910087	1.2724889	0.5672695	2.854425	0.5588117
cg05232479	1.40E-30	4.13E-58	0.004727	0.0335432
cg03004350	0.1523892	0.0411493	0.564347	0.0048566
cg22740783	0.4022567	0.1587999	1.018958	0.0548124
cg01577850	0.9599695	0.500187	1.842394	0.9022458
cg18460422	0.4494877	0.2000425	1.009981	0.0528776
cg07760269	10.337876	1.1009732	97.07019	0.040939
cg17538768	0.6825977	0.3437841	1.355326	0.2752065
cg25309292	0.3315644	0.1342381	0.818955	0.0167162
cg10495931	0.4413167	0.2180901	0.893027	0.0229322
cg11201931	3.9094746	0.0009617	15892.46	0.7477866
cg20438687	0.3205689	0.119299	0.861402	0.0240838
cg22658758	1.2983634	0.4895335	3.44358	0.5998207
cg23757056	0.0002607	3.05E-36	2.23E+28	0.8258942
cg10285525	0.0614284	0.0136903	0.27563	0.00027
cg25344845	0.0431553	0.005549	0.335622	0.0026717
cg03481509	1.4812668	0.4783734	4.586692	0.4956715
cg19092721	5.2594925	0.0022166	12479.53	0.6754776
cg23621729	1.4096561	0.6631316	2.996585	0.3722057
cg00274815	1.1819669	0.5889695	2.372017	0.6380647
cg09061216	0.0533907	0.0007585	3.758092	0.177017
cg10576245	0.3415628	0.1377318	0.847046	0.0204387
cg22857085	2.705948	1.2057922	6.072485	0.0157904
cg03099611	9.48E-16	4.17E-36	215859.9	0.1480661
cg18924331	0.2640813	0.1149224	0.606835	0.0017089
cg15448975	0.184416	0.0482047	0.705518	0.0135297
cg17332091	0.4594193	0.1693268	1.246501	0.1266874
cg03322633	2.4014246	0.4990356	11.55597	0.2744513
cg18248869	6.1508563	1.9061989	19.84737	0.0023715
cg09644722	2.2576339	0.9653633	5.279785	0.0602937
cg27316369	0.2416131	0.0609471	0.957829	0.0432511

cg12298445	0.1446008	0.0433336	0.482521	0.0016596
cg13452386	0.2205199	0.0866962	0.560913	0.0015045
cg14579051	0.6102851	0.3018482	1.233891	0.1691823
cg21172944	1.6844194	0.5864725	4.837855	0.3327238
cg01947906	0.5288744	0.2297087	1.217664	0.1343623
cg06058073	0.5554851	0.2278745	1.354095	0.1959467
cg04645160	2.345476	1.0358719	5.310751	0.0409055
cg19083774	1.7724055	0.8299543	3.785054	0.1392774
cg09273516	1.70E-06	1.87E-11	0.153788	0.0225234
cg19936847	0.3710531	0.1756176	0.783978	0.0093867
cg23042230	0.0496443	0.0017611	1.399436	0.0779527
cg16818768	0.8481738	0.0006666	1079.183	0.9639893
cg03982568	0.1231821	0.0174141	0.871353	0.0359119
cg09644974	2.7169776	0.7736944	9.541193	0.1188521
cg27102141	0.4645356	0.1784231	1.209447	0.1163093
cg15466684	0.5141116	0.1662639	1.589706	0.2480342
cg18293662	3.8068231	1.1034824	13.13288	0.0343602
cg18542098	0.2932948	0.0626728	1.372555	0.1192852
cg15085603	1.1925162	0.6054562	2.348799	0.6106878
cg22598373	5.159E+11	1.15E-10	2.31E+33	0.2890286
cg04543319	1.9851249	0.6955634	5.665509	0.2000236
cg16949674	0.5687541	0.2328599	1.389167	0.2155194
cg01119912	1.7762527	0.0376846	83.72311	0.7701016
cg05565052	0.2377206	0.0739177	0.764514	0.0159311
cg04210361	2.916E+09	5828.9716	1.46E+15	0.001134
cg14383078	2.9923509	0.7279982	12.29971	0.1285657
cg20191922	2.9423803	0.588805	14.70368	0.1886031
cg11560403	0.2884242	0.0089453	9.299707	0.4829292
cg02285155	0.415812	0.1785703	0.968244	0.0418716
cg20091689	0.2883797	0.1300568	0.639435	0.002209
cg04806887	1.0364773	0.3071926	3.497106	0.9539538
cg21945930	3.245154	1.2148168	8.668817	0.0188682
cg10459425	4.0552433	1.5719659	10.46142	0.0037862
cg16194233	3.9747483	1.2107097	13.04906	0.0228929
cg00036788	2.8473781	1.1821283	6.858445	0.0196479
cg02439789	0.1062285	0.0280266	0.402636	0.0009733
cg22488745	1.0537636	0.43538	2.550456	0.9075567
cg24575705	0.4830507	0.2109966	1.105885	0.0851049
cg23332586	1.4577545	0.5419585	3.921053	0.4553222
cg01609275	2.6832362	1.1957838	6.020952	0.0166856
cg21223521	2.2349833	0.7196867	6.940729	0.1642186
cg21877930	1.05E+11	4364.204	2.52E+18	0.0034281
cg25691899	3.0875637	0.5078344	18.77196	0.2208831
cg13337662	1.716636	0.736951	3.99869	0.2103944
cg25279613	0.4638153	0.1903653	1.130062	0.090864
cg22862767	0.7261331	0.2969451	1.775646	0.483019
cg13338137	2.2734386	1.0549123	4.899481	0.036045
cg17151604	0.0310261	0.0002976	3.23457	0.142967
cg12028740	0.4818413	0.1459769	1.590464	0.2307744
cg19704238	0.0078898	5.17E-05	1.203706	0.0590682
cg19455965	7.4545626	0.9116545	60.95566	0.0609729
cg21566771	0.743129	0.3095095	1.784245	0.506471
cg12013443	1.1420736	0.4625148	2.820087	0.7733104
cg10073571	6.7715492	0.3983969	115.096	0.1857445
cg25478712	0.5055822	0.0074203	34.44781	0.7515006
cg01214900	0.7506755	0.2312485	2.436832	0.6331058
cg26388509	1.2555894	0.7226761	2.181482	0.4193415
cg08123444	1.5840505	0.6603703	3.79971	0.3028135

cg16604566	0.6746413	0.3293474	1.381948	0.2820359
cg09059880	5.8119455	1.1235835	30.06337	0.0358228
cg08206308	0.0198133	0.0013013	0.301674	0.0047642
cg09750084	0.2573071	0.0875011	0.756642	0.0136368
cg17178900	1.9747889	0.7929278	4.918217	0.1438531
cg01777121	4.3235654	1.5458736	12.09233	0.0052699
cg14762024	1.4417115	0.5540047	3.751831	0.45344
cg13208054	0.6976676	0.2478357	1.963962	0.4953871
cg26889654	8.445E+11	125672.66	5.67E+18	0.0006175
cg24038454	1.21E-06	5.86E-13	2.48873	0.0662026
cg05998930	2.4798236	0.9276956	6.628818	0.0702403
cg25314706	5.2149546	0.8802594	30.89515	0.0688417
cg15562399	0.0697727	0.0020137	2.417559	0.1410369
cg14298457	2.9655316	1.0619683	8.281206	0.038013
cg27492749	1.2785372	0.3397309	4.811624	0.7163201
cg15042142	3.1756463	0.1513442	66.63441	0.4568287
cg03077671	2.2307599	0.8444526	5.892918	0.1054803
cg24663236	4.0315006	0.8398098	19.35319	0.0815359
cg24986840	0.103054	0.0239956	0.442587	0.0022418
cg20986996	1.579378	0.8003873	3.116535	0.1875371
cg13995774	0.2240759	0.0805752	0.623145	0.0041527
cg04127303	1.225E+11	8.83E-25	1.70E+46	0.5363014
cg14577373	0.3709456	0.1865896	0.737451	0.0046743
cg14156441	1.5103246	0.0697958	32.68219	0.7926643
cg17482424	0.6032714	0.3196138	1.138675	0.1189286
cg05475386	2.2731428	0.6972477	7.410821	0.1732328
cg21668832	0.7643213	0.1393536	4.192121	0.7569355
cg12833765	1.5187322	0.466795	4.941243	0.4875326
cg20386316	1.4005485	0.763774	2.568215	0.2762055
cg03085637	7.00E-20	1.49E-50	3.29E+11	0.2209551
cg02814805	0.4948979	0.2214775	1.105863	0.0864058
cg25745600	3.1323026	0.3511657	27.93929	0.3064744
cg20583073	1.5529681	0.5497101	4.387239	0.4061405
cg04517274	8.0943009	0.255199	256.7319	0.2357653
cg05170342	0.53282	0.2236785	1.26922	0.1551337
cg04311961	0.8097653	0.2826753	2.319692	0.6943457
cg14205321	1.7528079	0.7904253	3.88694	0.1672263
cg20612486	123.44491	0.0927324	164329.2	0.1894978
cg17249170	2.7888732	0.9351318	8.317345	0.0658173
cg17039369	1.5470918	0.6130563	3.904198	0.3555094
cg12284235	4.8411744	1.4291742	16.39896	0.0112891
cg12598635	0.1200703	0.0274862	0.524512	0.0048359
cg14712262	5.0822711	1.3087023	19.73671	0.018843
cg26059202	2.3349663	0.6542394	8.333444	0.1914329
cg19047265	1.7223219	0.4156565	7.136644	0.4535076
cg00465319	0.427974	0.1898101	0.964973	0.0407647
cg15934191	0.3039904	0.1014863	0.910568	0.033392
cg24469784	1.3409978	0.4366914	4.117954	0.6082479
cg08789739	0.0101454	1.15E-07	894.8656	0.4294448
cg20279561	4.7644143	1.2902125	17.59373	0.0191676
cg18632637	0.4919187	0.215522	1.122781	0.0920043
cg03725652	0.3951721	0.1355445	1.152102	0.089015
cg16046951	18794.998	6.92E-05	5.1E+12	0.3205885
cg18574274	0.3035441	0.1222588	0.753639	0.0101827
cg02118635	0.97913	0.0044759	214.189	0.9938785
cg06528737	0.5636388	0.2645144	1.201026	0.1374389
cg18559249	1.4903306	0.7007731	3.169479	0.3000241
cg13711238	1.6654501	0.7333095	3.782474	0.2229165

cg22252999	0.2620273	0.1064899	0.64474	0.0035527
cg05529874	0.1429773	0.0274639	0.744342	0.0208482
cg04546573	0.3926445	0.1337016	1.153088	0.0889785
cg18794622	2.4000579	0.7782749	7.401341	0.1275855
cg15571705	0.6111988	0.2779911	1.343798	0.2206425
cg17471102	0.3082957	0.0903439	1.05205	0.0602524
cg00940280	0.0005721	7.99E-19	4.1E+11	0.6687794
cg02048416	2.0388075	0.7291195	5.701036	0.1745248
cg12188526	0.2922454	0.1001226	0.853027	0.024397
cg12093220	0.1082532	0.0044726	2.620143	0.1714687
cg20634726	0.6551241	0.3230456	1.328567	0.2410336
cg10747531	2.8593404	0.6781749	12.05563	0.152431
cg14461181	0.6881444	0.3477111	1.361885	0.2832113
cg16831361	0.5826499	0.2403093	1.412683	0.2319335
cg18153816	6.4588242	1.2327347	33.84054	0.0272741
cg10186400	1.0274785	0.5147458	2.050939	0.9387287
cg12592691	1.2227717	0.4723747	3.165222	0.6785417
cg00215224	0.085431	0.0153447	0.475636	0.0049812
cg12416067	30.044957	0.1129042	7995.269	0.23234
cg16724196	1.03E-07	5.84E-15	1.814923	0.0587649
cg03503642	0.4890722	0.1970962	1.213578	0.1229514
cg05142765	1.7627998	0.8849891	3.511301	0.1068647
cg08794928	4.45E-05	1.30E-13	15234.01	0.3176093
cg09378743	2.8495029	0.8893207	9.13019	0.0779795
cg27178401	0.1849028	0.0828539	0.412642	3.77E-05
cg15019617	3.34E-08	9.74E-18	114.3749	0.1243276
cg24776736	3.1438452	0.8390847	11.77922	0.0891992
cg19391006	0.571982	0.3080764	1.061955	0.0768013
cg04292672	0.3574394	0.1571868	0.81281	0.0141108
cg19791630	1.7464347	0.6067607	5.02675	0.3012743
cg14787477	0.2199846	0.0819238	0.590711	0.00266
cg27361370	2.6884429	1.0061867	7.183285	0.0485795
cg06626750	0.2877599	0.1039314	0.796735	0.0165169
cg06575626	3.4752556	1.1457991	10.54059	0.0277801
cg11143193	0.8019706	0.3703062	1.736824	0.5756594
cg01925156	1.8240593	0.7665491	4.340481	0.1741762
cg06525670	2.1396343	0.6698531	6.834387	0.1992423
cg00464773	379.57434	0.661334	217857.7	0.0668938
cg26025891	1.0745877	0.3240266	3.563715	0.9063798
cg19576843	1.3534703	0.6576245	2.785605	0.4111461
cg24894783	2.2027132	0.7844477	6.185174	0.1338489
cg23690350	2.4394704	0.697629	8.530345	0.1626477
cg20366832	0.1986882	0.072497	0.544533	0.0016802
cg21233902	0.5187427	0.2348082	1.146016	0.1045992
cg22643294	0.483111	0.2104012	1.109292	0.0862723
cg19193384	4.1299038	0.6561556	25.994	0.1307782
cg23018236	5.404535	0.5320025	54.90387	0.1537481
cg21069965	0.4423752	0.2042844	0.957958	0.0385537
cg05483875	4.55E-06	8.58E-10	0.024138	0.0049385
cg01635736	1.4120716	0.4758363	4.190404	0.5341069
cg05405594	0.0531681	3.51E-05	80.5933	0.4322934
cg05709578	1.6882474	0.8131307	3.505192	0.1600268
cg18158419	0.1736707	0.0278963	1.0812	0.0606159
cg06879508	2.73E-12	3.54E-26	209.7903	0.1026249
cg10421247	0.0339065	0.0001774	6.48085	0.2067076
cg14179950	0.6556944	0.3298393	1.303469	0.2286078
cg18936283	604.17542	2.3170291	157541.4	0.0240716
cg03648483	5.23E-05	4.06E-09	0.673503	0.0411685

cg06890028	0.4205595	0.1892582	0.934545	0.0334922
cg07418452	1.2812038	0.6932722	2.367732	0.4290387
cg11076737	0.5220415	0.2077822	1.3116	0.1666997
cg06411434	0.4128122	0.0591473	2.881177	0.3721224
cg00827382	0.8215226	0.3242116	2.081663	0.6785599
cg19099433	0.4256302	0.1839697	0.984733	0.0459435
cg10501976	2.5163531	1.0540033	6.007603	0.0376704
cg13707794	1.8934011	0.6468002	5.54262	0.2440666
cg03442425	0.1139309	0.0141973	0.914277	0.0409229
cg14474520	1.2599099	0.6166279	2.57428	0.5262465
cg10495843	1.3047106	0.6406198	2.657223	0.463618
cg12990337	1.0861046	0.6361331	1.854365	0.762175
cg10539700	0.4818502	0.2016163	1.151592	0.1004967
cg10280412	0.0861379	0.0122417	0.606103	0.0137801
cg08095700	0.3396783	0.1118608	1.031472	0.056743
cg21827153	0.0646544	0.004503	0.928314	0.0439377
cg05299847	1.2119145	0.1914023	7.673558	0.8382648
cg16766249	0.0744353	0.0106474	0.520373	0.0088361
cg14165142	2.1228697	0.8645938	5.212362	0.1004863
cg14419424	0.1740453	0.037256	0.813072	0.0262107
cg25897951	0.4547614	0.1963422	1.053304	0.0659476
cg18553657	0.456023	0.2036441	1.021178	0.0562604
cg12580687	0.2383422	0.0840789	0.67564	0.0069859
cg10970392	2.6340448	0.8885841	7.808144	0.0806543
cg12909523	0.0735674	0.0178612	0.303012	0.0003025
cg09042026	7.9823451	1.2284874	51.8669	0.0295941
cg13435649	1.7100407	0.7581409	3.857118	0.1960859
cg02445664	1.6184334	0.617686	4.240547	0.3272539
cg09815356	13.082712	1.0995688	155.6586	0.041842
cg16990557	1.0256422	0.4293534	2.450061	0.9545552
cg26581165	0.4581159	0.2024117	1.036848	0.0610496
cg12752647	1.4421382	0.1425249	14.59228	0.7565142
cg08906015	0.3457609	0.1522541	0.785204	0.0111551
cg03763873	1.1592968	0.6469295	2.077458	0.6194387
cg03732295	0.0148564	0.0001153	1.914138	0.0894986
cg27572120	4.7161989	1.0027713	22.18106	0.0495919
cg06934523	0.0924329	0.0052025	1.64225	0.1047912
cg17567562	0.3080479	0.0291293	3.257669	0.3278158
cg24647506	0.4035283	0.178149	0.914039	0.0295983
cg15817635	1.39E-06	4.30E-12	0.450194	0.0372286
cg14161165	0.4724734	0.2220945	1.005118	0.0515695
cg08462030	2.3922538	1.0786894	5.3054	0.0318444
cg12099658	0.0992642	0.0123794	0.795948	0.0296426
cg25546651	0.5714571	0.2329179	1.402053	0.2217162
cg16725305	0.9124507	0.4033147	2.064309	0.8259069
cg15415716	1.1683193	0.4583905	2.977745	0.7445064
cg26616156	2.6719949	1.0028311	7.119401	0.0493427
cg26132452	3.73E-10	3.81E-19	0.365303	0.0398496
cg00474798	0.4156475	0.1661313	1.039918	0.0606128
cg02404507	0.4917057	0.2269376	1.065379	0.0719506
cg23087931	0.3493493	0.0656923	1.857828	0.217396
cg13806095	0.0475846	0.0014596	1.551292	0.0867174
cg17013340	5.6946126	0.0563789	575.1905	0.4600676
cg13263383	292.91334	1.36832	62703.33	0.0380333
cg19960114	0.1960941	0.0492334	0.781033	0.0208632
cg09665338	0.5211066	0.2500931	1.085804	0.0818268
cg26379339	3.479807	1.1577656	10.45899	0.02636
cg27619217	0.5295394	0.2362997	1.186679	0.1225347

cg01904169	0.0031828	2.82E-05	0.358952	0.0170827
cg08246366	0.3334008	0.125108	0.888481	0.0280631
cg21322654	2.7554906	0.9507679	7.985891	0.0619053
cg01936839	2.8063036	0.4736471	16.62702	0.2556514
cg02315926	1.4582779	0.5826952	3.649549	0.420226
cg26019250	2.7587876	1.3015144	5.847733	0.0081094
cg07997596	1.7455602	0.7269828	4.191269	0.2125793
cg02744705	0.1523118	0.0581931	0.398654	0.0001264
cg06332339	1.4098878	0.0372447	53.37093	0.8530083
cg04389950	2.4865026	0.2145858	28.81223	0.4661783
cg01896807	0.0871526	0.016584	0.458007	0.0039469
cg21976687	9.984E+17	4.29E-06	2.32E+41	0.1311082
cg18711553	0.5961414	0.3154281	1.126674	0.1112217
cg07980518	1.545202	0.8763755	2.724459	0.1326059
cg17155978	0.3109275	0.0176814	5.467669	0.4245231
cg16379671	1.9253123	0.6561546	5.64932	0.2329611
cg20175390	2.0428448	0.8565542	4.872097	0.1072207
cg10858327	2.403369	0.87009	6.638604	0.0907373
cg16700364	2.727827	0.8065825	9.225393	0.1064831
cg19692192	0.7090315	0.1375956	3.653646	0.6810384
cg02532824	3.0913532	1.1306424	8.452244	0.0278618
cg20704530	0.8871127	0.4344211	1.811535	0.742284
cg01708202	0.4838325	0.1812388	1.291632	0.1472923
cg00753286	0.6345568	0.1588577	2.534737	0.5197815
cg13378628	2.599608	1.0181062	6.637777	0.0457729
cg25644556	2.257702	0.7192085	7.087261	0.1629422
cg09287356	0.2513797	0.0887164	0.712289	0.0093655
cg19526425	0.2510093	0.1022711	0.616065	0.0025497
cg10250660	0.343473	0.1126848	1.046935	0.0602032
cg20810198	0.3373925	0.1144469	0.994642	0.0488724
cg01201932	0.628079	0.291028	1.355482	0.2360166
cg21486532	1.2619963	0.5179361	3.074964	0.6085825
cg01885859	7.27E-08	2.01E-25	2.63E+10	0.4255521
cg17396707	0.3063942	0.1469337	0.63891	0.0016063
cg16766828	0.2271184	0.0759227	0.679411	0.0080172
cg02102807	1.9379532	0.7695179	4.880539	0.1603161
cg25052456	2.6380334	1.1950127	5.823553	0.016354
cg09842118	1.2879968	0.6267879	2.646726	0.4909966
cg05031931	1.7088518	0.3263964	8.946713	0.5258338
cg01310473	1.2166537	0.6168247	2.399784	0.5715061
cg07842386	2.8184288	0.7036511	11.28903	0.1433216
cg05393567	2.715839	0.5861473	12.5835	0.201557
cg09814975	2.0804909	0.6762264	6.400878	0.2013684
cg20636382	0.0090205	8.57E-05	0.949837	0.0475213
cg21854560	1.5906622	0.7063954	3.581855	0.2624094
cg09155219	0.283718	0.0002601	309.4311	0.724083
cg22681186	0.5431282	0.2761912	1.068058	0.0768714
cg01022087	2.5908842	1.0951216	6.129622	0.0302519
cg03480346	0.4137575	0.2295426	0.74581	0.0033292
cg05642789	4.8787062	0.9778293	24.34144	0.0532825
cg13838528	2.45E+20	1.04E-16	5.76E+56	0.2719093
cg01842807	0.2887769	0.1230107	0.677926	0.0043345
cg13260278	0.3322135	0.1390506	0.79371	0.0131422
cg00934355	1.3599301	0.7517252	2.460221	0.3094243
cg04457114	4.3728795	1.5252547	12.53697	0.006041
cg19708554	0.1319213	0.0168477	1.032974	0.0537225
cg25595388	0.3166832	0.0832272	1.204993	0.0917059
cg23058901	1.7974041	0.6868585	4.703533	0.2322262

cg02072322	0.8646091	0.185402	4.032045	0.8530879
cg14423692	0.2933142	0.066857	1.286825	0.1040117
cg11360522	3.2167643	0.9929419	10.42113	0.0513966
cg25923345	0.1710249	0.0573954	0.509614	0.0015242
cg17652741	3.83E+11	3.22E-16	4.56E+38	0.4017489
cg03889542	1.6124032	0.7497575	3.46758	0.2214106
cg01654446	0.9795949	0.2966521	3.234786	0.9730165
cg06422467	0.6064195	0.2192198	1.677516	0.3353053
cg15456293	1.8695488	0.834153	4.190134	0.1286201
cg14653225	2.6930552	1.0332662	7.019049	0.0426707
cg02435734	1.37E-07	1.19E-18	15837.26	0.224034
cg13201342	0.5597197	0.2869184	1.0919	0.0887376
cg17241283	1.993699	0.7967088	4.989069	0.1403874
cg05360477	0.4041264	0.1675442	0.974777	0.0437131
cg08327151	2.4281097	0.76607	7.696055	0.1317568
cg17870484	0.3756828	0.1697814	0.83129	0.0156944
cg05146756	0.9548214	0.4752498	1.918326	0.8966662
cg12984636	0.4094028	0.19026	0.880956	0.0223632
cg23513930	1.7796771	0.3643118	8.69379	0.4762978
cg18400182	3.2103398	0.787434	13.08844	0.1038054
cg27631256	1.0856679	0.5796952	2.033266	0.7973685
cg08449049	1.1630705	0.5339519	2.533436	0.7037128
cg14621784	2.3032044	1.0618536	4.995746	0.0346964
cg04013650	0.5102552	0.243408	1.069646	0.0748013
cg05724065	0.345055	0.0963905	1.235215	0.1019845
cg02481697	2.0950269	0.7704259	5.697027	0.1473444
cg11795276	3.2026453	0.8678057	11.81939	0.0806131
cg25338577	2.22E-17	1.77E-34	2.779233	0.0562523
cg27143688	1.5775453	0.8113894	3.067145	0.179
cg12073779	2.166398	0.9416828	4.983929	0.0689707
cg24931520	1.9067975	0.7153641	5.082554	0.1969414
cg01950810	0.1792868	0.0269962	1.190678	0.0751914
cg20969675	3.2822613	0.3357761	32.08459	0.3068886
cg08552189	0.6196796	0.2522306	1.522428	0.2967235
cg10112270	0.0459416	0.0090211	0.233967	0.0002081
cg07665134	0.315282	0.1133602	0.876875	0.026986
cg19358877	0.3689197	0.130482	1.043069	0.0600474
cg15068641	0.3458006	0.1126301	1.061688	0.0635428
cg16452067	1.1905841	0.3181321	4.455667	0.7955807
cg10446968	257.17412	0.8176947	80884.14	0.0585747
cg06551919	2.79E-19	7.10E-46	1.09E+08	0.1714632
cg02773945	1.2740168	0.6378829	2.544541	0.4926257
cg06595212	1.5684037	0.6247424	3.937447	0.3379073
cg09799633	0.8080228	0.237511	2.748929	0.7329294
cg21070172	0.2721662	0.0970605	0.763178	0.013372
cg11039361	1.0319645	0.5476956	1.944421	0.9224514
cg24103044	1.8231627	0.6480008	5.129503	0.2551557
cg18769818	2.2632534	1.0109603	5.066782	0.0469807
cg01802532	1.3736385	0.6568529	2.872611	0.3990117
cg02860199	0.6462335	0.1943042	2.149298	0.4764269
cg11393994	9419.6253	0.0860434	1.03E+09	0.1221918
cg22893838	10994397	5.83E-10	2.07E+23	0.3964845
cg07435282	0.2836816	0.0953232	0.844236	0.0235576
cg00124095	0.3613571	0.1461443	0.893493	0.0275395
cg06228507	0.3399675	0.0942468	1.226333	0.0992982
cg13362627	1.7997373	0.6620961	4.892122	0.249415
cg13218831	8.6558348	0.4065128	184.3078	0.1666318
cg06745083	2.4689607	1.1791149	5.169782	0.0165331

cg10261621	0.6745423	0.3446481	1.320208	0.2504863
cg08734931	0.1570273	0.0426316	0.578388	0.0053858
cg09827249	0.1227947	0.030585	0.493005	0.0031045
cg18128164	2.2936093	0.8722313	6.031249	0.0924055
cg07646999	0.0698001	0.0168163	0.289722	0.0002464
cg05506209	0.9026228	0.4705918	1.731283	0.7578547
cg24425316	1.9707996	0.813462	4.774717	0.132921
cg05714673	3.5744805	1.6186187	7.893712	0.0016252
cg26990587	0.21385	0.0444166	1.029612	0.0544083
cg19474888	0.696947	0.2829524	1.716667	0.4324444
cg00718883	0.3480053	0.1603443	0.755298	0.0075896
cg25432738	0.4240663	0.2080819	0.864238	0.0181945
cg24045378	0.3984261	0.1548289	1.025282	0.0563666
cg02753187	1.4352989	0.6996857	2.944298	0.3242424
cg11185653	3.85E-13	5.08E-22	0.000291	0.0061372
cg26651303	0.4758756	0.2132005	1.062181	0.0698766
cg24688926	2801814	38.987546	2.01E+11	0.0092676
cg05329976	1.9843703	1.0336967	3.809363	0.0394401
cg26376025	0.6564934	0.283995	1.517575	0.3249468
cg06164804	0.1806117	0.0565158	0.577194	0.0038883
cg26307926	2.3739008	0.7912538	7.12212	0.123006
cg03709663	0.3849612	0.1280297	1.157505	0.0892146
cg14011416	2.4032466	0.8291621	6.965579	0.1063274
cg02873315	1.9095383	0.3145343	11.59281	0.4820737
cg18008467	4.2317028	0.0008937	20037.66	0.7383002
cg13178372	24.009712	0.0082272	70067.99	0.4349315
cg01909242	0.1222915	0.0215852	0.692846	0.0175664
cg01334682	2.3375709	0.7493694	7.291781	0.1434989
cg23521603	2.1077442	0.1225163	36.26119	0.6075006
cg26131286	3.3005921	1.2229569	8.907843	0.0184085
cg14542554	1.5100935	0.5418731	4.208333	0.4305688
cg05240350	0.3485531	0.0970282	1.252102	0.1062278
cg16494192	0.0822127	0.0142382	0.474705	0.0052253
cg03287046	0.3208413	0.1676498	0.614013	0.0005974
cg10850215	0.3985252	0.1782416	0.891051	0.0250294
cg16806891	1.4997639	0.68965	3.261498	0.3065261
cg08262088	3.1716486	0.2170766	46.34011	0.3989009
cg09510954	6.0196394	0.1170555	309.563	0.3719049
cg18034294	0.0271467	0.0014505	0.508077	0.0158215
cg23094961	0.135509	0.0226909	0.809255	0.0283737
cg25736830	0.0997487	0.0265606	0.374607	0.0006394
cg06893560	0.8702726	0.2449981	3.091348	0.8298842
cg11832020	1.841322	0.8603201	3.940936	0.1158478
cg21342586	0.310673	0.1352632	0.713555	0.0058608
cg06405317	0.0877037	0.013033	0.590188	0.0123469
cg12910851	2.3486851	1.0349695	5.329937	0.0411345
cg18145810	0.4771825	0.2477896	0.918938	0.0269113
cg04831806	2.7604503	1.2240618	6.225246	0.0143958
cg03420013	0.6803272	0.3454759	1.339732	0.2652548
cg07119829	2.1587067	0.7333724	6.354227	0.1624152
cg12226028	2.8058321	0.8912985	8.832836	0.0778521
cg12817782	0.2933628	0.1344775	0.639971	0.0020598
cg14505733	17.24343	1.9077757	155.8547	0.0112436
cg20866810	1.4811795	0.0854419	25.677	0.7872397
cg08565632	1.7562173	0.515461	5.983574	0.3679
cg02423610	50.335143	3.8075314	665.425	0.0029303
cg02101812	1.2074511	0.604916	2.41015	0.5929539
cg12552261	3.5598317	0.6660816	19.0253	0.1375994

cg03519145	2.266926	0.7593498	6.767571	0.1424757
cg17950348	0.7148179	0.3558905	1.435736	0.3454163
cg09701682	1.1585407	0.5664954	2.369334	0.68684
cg07349899	2.99253	0.9210023	9.723359	0.0682894
cg10666916	4.0197055	1.3275697	12.17114	0.0138453
cg03401266	0.0767985	0.0056857	1.037339	0.0533146
cg14754581	1.6782861	0.8755918	3.216847	0.1188194
cg18344930	1.96595	0.7466709	5.176255	0.1711445
cg12588041	0.0946872	0.0204709	0.437971	0.0025572
cg19291576	1.3227187	0.7126302	2.455109	0.3754382
cg14435498	2.8368279	1.0896674	7.385366	0.0326902
cg07717559	0.8731258	0.4414729	1.72683	0.6965865
cg14109335	2.49E-07	7.57E-16	82.1087	0.1286472
cg20584555	0.4611605	0.1663161	1.278704	0.1368843
cg14314533	0.2563228	0.0111108	5.913265	0.3952551
cg16995668	1.32459	0.4883946	3.592461	0.5808095
cg21382392	1.4998895	0.2676085	8.406568	0.644814
cg08527797	1.7282347	0.6883707	4.338934	0.2440709
cg23355637	0.263387	0.0428227	1.619997	0.1500219
cg22510442	1.292539	0.4168082	4.008216	0.6567539
cg14140673	0.0819215	0.0091355	0.734624	0.0253836
cg24001514	0.9313801	0.2230867	3.888484	0.922334
cg18044044	0.4196502	0.1861364	0.946114	0.0363035
cg08936307	1.4733713	0.7444066	2.916179	0.2658835
cg06265809	0.537461	0.1990971	1.450871	0.2204093
cg07373578	0.4271303	0.1782237	1.023659	0.0564522
cg00458754	1.1357088	0.3992039	3.231017	0.8114505
cg27418402	0.3575213	0.1440935	0.887073	0.0265269
cg02734661	3.1698214	6.45E-15	1.56E+15	0.9467082
cg06982885	1.0544638	0.4397877	2.528252	0.9053871
cg07398767	0.2800307	0.0659117	1.189732	0.0846029
cg09729182	2.6634518	0.8449329	8.395904	0.0944612
cg18094781	1.1779142	0.5955809	2.329628	0.6379234
cg05486035	1.9149978	0.871218	4.209298	0.1059044
cg02154186	0.0756552	0.0143763	0.398136	0.0023118
cg17156633	0.5201759	0.1302187	2.077911	0.3549924
cg13629586	0.3941815	0.1171165	1.326705	0.1327302
cg04839764	0.0806336	0.0117078	0.555337	0.0105464
cg25451456	0.8236059	0.3953241	1.715875	0.6043139
cg07811074	3.1735395	0.9826007	10.24969	0.05353
cg07671622	0.3334039	0.1043566	1.065175	0.0638217
cg10132273	2.1587681	0.52401	8.893493	0.2867295
cg06571387	2.0723952	0.6712856	6.397905	0.2051584
cg25830379	34.421457	1.2389371	956.3332	0.0369532
cg16100845	3.5905143	1.0407284	12.38728	0.0430581
cg23258173	0.511945	0.249349	1.051088	0.0681204
cg26756479	3.55E-22	7.02E-37	1.79E-07	0.0042472
cg12623735	0.5665598	0.1905067	1.684928	0.3069004
cg17707140	2.1916548	1.1436777	4.199917	0.0180537
cg02963641	0.7070026	0.0371526	13.45403	0.8175694
cg14144201	0.0586043	0.0095102	0.361134	0.0022302
cg08864645	2.1444703	1.0761964	4.273154	0.0301043
cg23606718	0.0541079	0.0080896	0.361903	0.002628
cg07213264	118.98162	0.0002015	70256012	0.4809
cg07985359	277606.5	0.0372791	2.07E+12	0.1205356
cg18420599	0.3851044	0.1734487	0.855039	0.019038
cg12894126	0.056494	0.0058006	0.550218	0.0133458
cg16171484	3.1926955	0.9124356	11.17153	0.0692836

cg16306978	2.9099439	1.2211995	6.933981	0.0159072
cg13615337	2.2384746	0.8332881	6.013248	0.1099907
cg18175809	2.2046781	1.099837	4.419387	0.0258693
cg14851390	0.37761	0.1260553	1.131165	0.0818957
cg24319836	0.8494289	0.2937458	2.456305	0.763248
cg10378325	77.022585	3.3957863	1747.012	0.0063803
cg14322697	0.3635493	0.1617794	0.816965	0.0143125
cg14822257	4.1741933	0.8159351	21.35451	0.0862141
cg21616243	0.0675672	0.0134678	0.338982	0.0010581
cg17778434	2.05E-06	1.11E-16	37728.54	0.2774212
cg10459187	0.9969726	0.2281848	4.355919	0.9967845
cg03846249	1.5569635	0.5584756	4.340629	0.3973563
cg04072597	1.1727155	0.6119317	2.247411	0.6311769
cg26764244	0.0528561	0.0031689	0.881627	0.0405886
cg12063847	0.4515477	0.1232756	1.653979	0.2300176
cg10997248	2.1709994	0.7428169	6.345088	0.1565874
cg11641102	1.136215	0.4883302	2.643671	0.7669312
cg05078075	0.6286018	0.2453727	1.610368	0.3334104
cg10971134	0.6321849	0.2665939	1.499125	0.297913
cg03582827	9.0634632	0.1152197	712.9541	0.3223153
cg02735446	0.0549069	0.0063952	0.471408	0.0081571
cg18861189	0.0802442	0.0056919	1.131271	0.0616782
cg22130808	1.4486326	0.7443791	2.819177	0.2752815
cg13555278	0.2141737	0.0579878	0.791035	0.0207993
cg20210376	16.268925	0.1189043	2225.974	0.2663781
cg01124546	2.9456963	0.9155818	9.477173	0.0699807
cg06303875	1.9981185	1.0293019	3.878821	0.0408253
cg13115952	0.4926037	0.2255475	1.075864	0.0756511
cg01859191	0.2994426	0.0974364	0.920251	0.035287
cg00096603	0.4906957	0.1113978	2.161462	0.3466622
cg01243513	0.2551286	0.0738099	0.881868	0.0308793
cg09888012	1.1074788	0.2858524	4.290709	0.8825532
cg10534507	0.1271556	0.028571	0.565906	0.0067823
cg14063129	0.3136031	0.1472449	0.667914	0.0026448
cg04894027	10.705041	0.7302818	156.9228	0.0835374
cg14681511	0.821877	0.3651071	1.850092	0.6356125
cg25389328	0.1747044	0.050604	0.603146	0.0057851
cg01508045	1.1426135	0.4885328	2.672421	0.7584393
cg08142172	2.6572293	1.1305043	6.245768	0.0250081
cg26754170	0.3952757	0.1620724	0.964031	0.041301
cg10482532	0.7557541	0.2596489	2.199756	0.6074376
cg27316811	9.0115203	0.2362558	343.727	0.23667
cg24753061	2.7497813	0.5999141	12.60397	0.1928573
cg01990572	0.3750713	0.1680335	0.837205	0.01668
cg01046703	3.2087686	0.8550723	12.04132	0.0840028
cg21072795	0.1777493	0.0394705	0.800468	0.0244592
cg19950556	0.3362431	0.0641686	1.761911	0.1971444
cg14984160	3.0846008	0.7718014	12.32799	0.1110429
cg13890451	1.2946477	0.5987716	2.799252	0.511584
cg11010178	0.046184	0.003279	0.6505	0.0226912
cg18612627	0.6300345	0.192415	2.062954	0.4452341
cg00930194	1.467066	0.790543	2.722537	0.2243966
cg00331027	0.9929762	0.2027045	4.864233	0.993063
cg26885488	1.7571448	0.5278467	5.849346	0.3582753
cg05975710	2.1409165	0.5158113	8.886046	0.2945004
cg22030890	6.43E-05	4.90E-08	0.084315	0.0084099
cg04890480	0.2716859	0.0858034	0.86026	0.0266968
cg02691035	2.6943231	0.7276358	9.976663	0.137828

cg21726844	0.1963461	0.060903	0.633003	0.0064185
cg06204638	0.2693364	0.0124283	5.836864	0.4032384
cg18322321	1.1209627	0.4195983	2.994667	0.8198353
cg11694510	2.4134999	0.7683497	7.58116	0.1313664
cg15380508	1.6396123	0.8542835	3.146881	0.1371482
cg05650559	0.7359837	0.3983975	1.359627	0.327618
cg01851632	0.2236522	0.0541774	0.923269	0.0384219
cg09405661	12.871976	2.1815357	75.95006	0.0047835
cg14999934	0.1512572	0.0339553	0.673789	0.0132132
cg17928651	4.8576583	1.2109331	19.4865	0.0257477
cg05945401	0.3819665	0.1389804	1.049777	0.0620708
cg19879189	1.9640734	0.545553	7.070961	0.3016887
cg19477706	0.285861	0.0856529	0.954043	0.0417028
cg05927789	1.5215189	0.5040253	4.593063	0.4565396
cg23936070	2.2656169	0.90128	5.695256	0.0820416
cg23549641	2.0157112	0.4610983	8.811769	0.3516618
cg04321396	0.2238158	0.0391198	1.280515	0.0925468
cg12054869	1.1387321	0.6116139	2.120146	0.6820584
cg26242531	0.2023126	0.0652151	0.627621	0.0056679
cg23968676	2946741.7	8.58E-06	1.01E+18	0.2717113
cg19178081	92.58831	2.2498085	3810.367	0.0169641
cg13518232	0.1645921	0.0514994	0.526036	0.0023378
cg20988861	2.1533034	0.9965032	4.652986	0.0510507
cg24307102	1.2342	0.4465385	3.411239	0.684988
cg21698185	0.2307941	0.0733507	0.726182	0.0121747
cg00820718	2.0577785	0.2937702	14.41417	0.467479
cg16945633	0.259865	0.1126601	0.599412	0.0015767
cg03994820	4.9164285	1.1509014	21.00203	0.0315811
cg12444411	0.2531497	0.1288886	0.497211	6.64E-05
cg13706582	0.3050397	0.133227	0.698426	0.0049668
cg16593917	0.5332492	0.1874161	1.517238	0.2385775
cg16376770	0.2858251	0.1116391	0.731786	0.0090282
cg14041921	4.1378888	1.5625233	10.958	0.0042611
cg23448505	0.1690392	0.0470726	0.607025	0.0064251
cg07263322	2.4368421	0.9898745	5.998942	0.0526449
cg22124564	0.891463	0.3247475	2.447151	0.8235402
cg23654971	3.52E+26	1.52E-07	8.15E+59	0.1188848
cg01960280	0.3483316	0.1430368	0.848277	0.0202162
cg09873284	0.3971632	0.148481	1.062348	0.0658445
cg02550691	0.0094599	0.000268	0.333904	0.0103705
cg23377551	0.3392465	0.1422688	0.808949	0.0147625
cg06394229	3.2716139	0.1672265	64.00576	0.4346717
cg25404233	0.1851014	0.0005171	66.26316	0.5739614
cg11326429	0.2466501	0.0817654	0.744034	0.0129618
cg05324982	0.0525976	0.0035662	0.775765	0.0319623
cg11426726	0.3149628	0.0453347	2.188204	0.2427406
cg04658129	0.8371545	0.0870349	8.052259	0.8776908
cg21177778	2.605251	1.0664184	6.364605	0.035635
cg18419358	1.7762219	0.5085594	6.203728	0.367958
cg14074944	0.2907864	0.0994138	0.850553	0.0240986
cg00582628	0.07034	0.008419	0.587685	0.0142562
cg11148483	0.0008191	2.47E-07	2.71359	0.0856916
cg08273635	2.6268157	0.231837	29.76298	0.435528
cg18135555	3.8078446	0.6129665	23.65493	0.1513563
cg04678936	0.028402	0.0009016	0.894721	0.0430565
cg04504095	2.03E-08	1.01E-16	4.062745	0.0693355
cg11585357	2.8550154	0.0185705	438.9276	0.6830158
cg27136547	4.6891652	0.6081483	36.1561	0.138142

cg13393433	1.9328548	0.4186169	8.924455	0.3985
cg22907952	3.0122601	0.500767	18.11963	0.2283978
cg00599770	2.6046047	0.8637381	7.854193	0.0891594
cg07935568	0.127759	0.0359207	0.4544	0.001481
cg00688962	0.0665617	0.0120081	0.368957	0.0019281
cg13431152	3.2710227	0.621269	17.22215	0.1620137
cg20969242	5034.6172	54.401542	465931.1	0.0002243
cg18817990	5.0008698	0.8539564	29.28569	0.0742781
cg04910970	3.6334028	1.1244733	11.74027	0.0310829
cg10961099	1.8052678	0.8933414	3.648092	0.099818
cg07221526	0.0667009	0.0055025	0.808536	0.0334269
cg02286809	0.3191237	0.1456671	0.699128	0.004311
cg26781150	0.1604954	0.0250864	1.0268	0.0533553
cg05265611	0.9519024	0.4848017	1.869049	0.8861419
cg27055236	0.4564421	0.1979374	1.052552	0.0657953
cg24834436	1.9682147	0.7034573	5.5069	0.1970859
cg20746815	0.0983002	0.0062299	1.551057	0.09933
cg04359250	0.0265374	0.0018778	0.375026	0.0072363
cg15864571	0.3284544	0.1411794	0.76415	0.0097562
cg17830244	0.0836674	0.0102793	0.681001	0.0203897
cg02770843	2.0828864	0.8631133	5.026473	0.102584
cg09245378	2.39E-21	5.70E-45	1001.115	0.0870791
cg11199751	0.4954549	0.2417772	1.015296	0.0550484
cg03620376	0.0634132	0.0072333	0.555933	0.0127741
cg10626305	1.296719	0.2561649	6.564054	0.7535044
cg25030694	0.4768769	0.2293287	0.99164	0.0474302
cg27584713	0.6653801	0.2869275	1.543005	0.3424684
cg16420641	0.1216221	0.0140376	1.05374	0.0558188
cg23013029	4.2708863	1.336788	13.645	0.0142953
cg02402423	0.2692142	0.1020014	0.710542	0.0080473
cg23337031	0.17579	0.044385	0.696228	0.0133028
cg03765423	0.1566613	0.0574409	0.42727	0.0002934
cg11393173	0.8371832	0.2953916	2.3727	0.7381127
cg23008083	0.384847	0.1183705	1.251217	0.1124217
cg15028514	0.3135016	0.029646	3.315224	0.3350691
cg08257257	1.2773786	0.3049906	5.349989	0.7376232
cg07636653	0.8500269	0.4370493	1.653236	0.6321227
cg00844074	0.7167264	0.3288497	1.562102	0.4020975
cg00041575	1.7904838	0.9167828	3.496828	0.0880901
cg01663016	0.1609871	0.0318606	0.813445	0.0271206
cg25137711	3.418688	1.0494079	11.13716	0.0413506
cg09372617	4.2401857	0.2740751	65.59944	0.3012568
cg23463715	0.0105388	1.39E-10	801575.5	0.6229231
cg22566355	2.6548475	0.5150496	13.68454	0.243224
cg21075560	83178072	2.77E-05	2.50E+20	0.2134859
cg12935170	1.3213782	0.6813874	2.562478	0.4095461
cg24140939	0.1954755	0.0652497	0.585607	0.0035474
cg08944940	0.1926472	0.075268	0.493078	0.0005934
cg23181900	5.8378519	0.9950066	34.25155	0.0506517
cg08680771	1.7356158	0.2647429	11.37844	0.5654913
cg02499614	0.8724732	0.1872867	4.064406	0.8620429
cg18149394	0.3042207	0.1017175	0.909875	0.0332598
cg04399632	1.0347364	0.4340993	2.466439	0.9385852
cg00574742	0.4010831	0.1622995	0.991178	0.0477979
cg25461905	5.0427629	0.0985975	257.9119	0.4202732
cg09761230	0.9910686	0.510471	1.924139	0.9788556
cg00813378	0.4172368	0.2216807	0.785303	0.0067489
cg22188703	0.0026193	8.39E-06	0.817425	0.0424826

cg13220900	0.1592697	0.0478613	0.530007	0.0027452
cg07377519	0.5331847	0.1982491	1.433984	0.2128111
cg27100140	0.2438991	0.0933146	0.637486	0.0039969
cg13734061	0.3732876	0.1787929	0.779358	0.008698
cg04436971	0.247432	0.0977378	0.626397	0.0032086
cg09990086	7.0014906	0.0155531	3151.838	0.5324202
cg07878514	1.6566817	0.4990095	5.500083	0.4096241
cg08492173	1.0003748	0.4825488	2.073883	0.9991961
cg00440043	1.4814468	0.8291981	2.646756	0.1843805
cg07574896	0.3407897	0.1221301	0.950934	0.0397771
cg05330472	2.1269366	0.8366018	5.407423	0.1129168
cg08771706	2.7770968	1.2827246	6.01241	0.0095489
cg04465784	0.558803	0.1750749	1.783584	0.3257075
cg14817490	2.870235	0.8677059	9.494287	0.0840821
cg25575845	1.8957773	0.7262849	4.948433	0.1913334
cg13909612	0.3630658	0.1797457	0.733351	0.0047346
cg17329648	1.3074195	0.6689601	2.555228	0.4330127
cg24376776	2.0824048	1.0277139	4.219471	0.0417669
cg14685355	5.67E+21	7.00E-05	4.58E+47	0.0998349
cg09372486	1.9452889	0.9468423	3.996599	0.0700978
cg18568990	1.27E-06	3.01E-19	5338850	0.3599278
cg08129017	0.3487784	0.1334599	0.911482	0.0316294
cg14334794	1.5574186	0.0440769	55.02998	0.8075569
cg11581627	2.691469	0.9219068	7.857633	0.0701072
cg23358740	1.4286041	0.6096178	3.347851	0.4116903
cg12119029	0.2730378	0.050855	1.465926	0.1300499
cg02043994	0.289816	0.1153167	0.728371	0.0084377
cg16126286	1.7034422	0.5500885	5.274997	0.3556915
cg05897809	1.2526936	0.624783	2.511658	0.5255818
cg21174841	1.9991674	1.0737859	3.722036	0.0289287
cg14556323	0.3237429	0.1128514	0.928739	0.0359536
cg24811352	0.2463889	0.0648814	0.935668	0.0396253
cg10363885	4.2848076	0.9149121	20.06704	0.0647356
cg16338313	0.6264342	0.2857145	1.373468	0.2429308
cg22840650	1.9088691	0.3242219	11.23854	0.4747627
cg03468349	0.1723797	0.0414215	0.717376	0.0156694
cg10665321	0.4447632	0.2135583	0.926278	0.0304216
cg25340966	2.0976783	1.1155408	3.944503	0.0214867
cg16606238	4.7247239	0.4078965	54.72716	0.2140698
cg21306949	0.4048076	0.1680965	0.974852	0.0437202
cg17734698	0.2369259	0.0586097	0.957758	0.0433291
cg18817970	0.377876	0.1587066	0.899712	0.0278973
cg25119946	2.1332518	0.7274312	6.255936	0.1675172
cg25350986	0.5328507	0.2667372	1.064455	0.0745795
cg09114153	2.1686527	0.6901561	6.81448	0.185122
cg04201675	0.5934329	0.2997517	1.174848	0.1342543
cg16314254	0.2204458	0.1096146	0.443338	2.22E-05
cg07069514	0.1175312	0.0156403	0.883203	0.0374649
cg18652285	0.4347316	0.2153802	0.877479	0.0200873
cg01373896	0.200154	0.0838265	0.477911	0.0002916
cg08583625	0.4160735	0.0683068	2.534406	0.3415025
cg07151830	783435.08	6.7417586	9.1E+10	0.022569
cg15379412	0.0790013	0.0021825	2.859674	0.1656953
cg22262757	1.9811106	0.8793954	4.463065	0.0989807
cg15195814	0.3186702	0.1244155	0.816222	0.0171658
cg05347613	22.744818	0.4470588	1157.178	0.119138
cg26426334	2.2423129	0.6486681	7.751216	0.2019527
cg05410910	0.3800222	0.177597	0.813172	0.0126735

cg07402639	3.2977461	0.6217831	17.49023	0.160986
cg07832354	0.6156026	0.258606	1.465421	0.2729138
cg09415272	1.9739613	0.8105327	4.807361	0.1342851
cg02109162	1.3465065	0.6605264	2.744901	0.4129483
cg04420138	0.0440062	0.0020416	0.948565	0.0461882
cg00306909	2.6619159	1.1395223	6.218216	0.023717
cg17886959	0.447561	0.1678111	1.193669	0.108217
cg09856467	1.9554665	0.4492831	8.511002	0.3714766
cg00113321	0.4458294	0.1504556	1.32108	0.1449633
cg11139044	2.6952697	1.0433702	6.962513	0.0405949
cg06470558	0.3134643	0.0575184	1.708321	0.1799356
cg08382072	0.6328303	0.3014243	1.328606	0.2266151
cg24398258	2.0369934	0.6302072	6.584092	0.2345899
cg21179618	0.9157583	0.2163768	3.875707	0.9048375
cg13952899	0.321252	0.131886	0.782516	0.0124242
cg08406071	14.186882	8.62E-15	2.34E+16	0.8820519
cg11702455	1.2022513	1.62E-08	89294739	0.9841071
cg07905444	1.9095735	0.9881087	3.690354	0.0543074
cg12929809	0.5584659	0.2579277	1.209192	0.1393994
cg07833554	0.6599512	0.244041	1.784682	0.4129157
cg18782774	0.3411783	0.1326115	0.877772	0.0257236
cg20910875	0.2018353	0.0290309	1.403245	0.1057639
cg09654300	0.4965727	0.2510465	0.982226	0.044273
cg04190037	1.8640482	0.8901983	3.90326	0.0986342
cg06997226	0.0293626	0.001741	0.495202	0.0143847
cg19679544	2.895481	1.2131591	6.910726	0.0166062
cg00985117	0.1125028	1.11E-06	11396.39	0.7102497
cg19702802	15.106816	2.7500959	82.98471	0.0017847
cg15994554	64.53207	1.1304519	3683.826	0.0434474
cg02074956	0.3679808	0.1730663	0.782416	0.0093911
cg04961582	1.1074775	0.5562146	2.205096	0.7714121
cg01758106	0.351858	0.1196461	1.034752	0.0577103
cg08002791	2.6592776	1.0126519	6.983404	0.0470904
cg00011482	2.5594449	1.0999006	5.955773	0.0291881
cg23087306	1.6802094	0.7274409	3.88087	0.2243957
cg14634842	1.575E+13	17.556725	1.41E+25	0.0304627
cg06577710	1.3594604	0.5411728	3.415051	0.5134764
cg14230378	2.4499719	0.8618073	6.964854	0.0927687
cg18998321	0.262051	0.1005427	0.683	0.0061436
cg04256238	0.3883326	0.1530174	0.985524	0.0465191
cg18989081	4.05E-06	7.54E-11	0.217988	0.0254786
cg04170999	1.09E-08	4.95E-21	24097.88	0.2061685
cg24685134	3.1584881	1.3747904	7.256413	0.0067287
cg06538426	1.756797	0.5812884	5.309474	0.3180006
cg22094607	4.9353324	0.0490767	496.315	0.4973854
cg17139436	0.4581025	0.1801026	1.165213	0.1012234
cg10531167	9.66E-06	9.40E-09	0.009933	0.0011011
cg23676956	5.4056586	0.5465324	53.46645	0.1489537
cg25541653	1.3645094	0.6710319	2.774661	0.3907403
cg21521453	0.0476386	8.73E-05	25.98589	0.3437462
cg10575367	1.1189432	0.5484539	2.282843	0.7573839
cg14790396	0.0678768	0.0112152	0.410805	0.0034068
cg12000131	0.2789888	0.0932744	0.834471	0.0223904
cg05299836	2.7697797	0.6747257	11.37007	0.1573882
cg17676119	0.0350353	0.0007238	1.695858	0.090432
cg16351246	2.79016	0.5165	15.07259	0.2331505
cg21595709	1.8938387	0.7132451	5.028601	0.1999413
cg02013846	21.934975	1.0033101	479.5558	0.0497551

cg00329411	0.2822843	0.1248309	0.638339	0.0023799
cg14325123	0.0193437	0.0014214	0.263255	0.0030574
cg12186317	0.2077962	0.0704153	0.613209	0.0044311
cg16172278	6.8070542	0.7754852	59.75096	0.0835332
cg02545106	0.0171655	1.83E-08	16105.66	0.5623594
cg11147919	0.082324	0.016439	0.412266	0.0023817
cg21017610	1.664E+15	6.71E-20	4.13E+49	0.3857384
cg17186355	0.3134049	0.0768202	1.278604	0.1057986
cg03346041	167.06595	0.002992	9328665	0.3587185
cg06937409	0.4054825	0.1018304	1.614607	0.200405
cg00255919	1.5128503	0.4401847	5.199445	0.5110181
cg08981421	1.4624601	0.4739361	4.512823	0.5084957
cg10762533	0.0001971	5.87E-08	0.661972	0.0394416
cg22031848	0.143652	0.0440173	0.468813	0.0013033
cg24376214	1.1139469	0.5554863	2.233858	0.7611617
cg26442210	1.5840947	0.027944	89.79942	0.823298
cg07568203	0.7471124	0.360664	1.547637	0.4326823
cg11757130	0.3561813	0.0692105	1.833032	0.2168267
cg22051763	1246483.3	0.0984717	1.58E+13	0.092538
cg09419176	0.2518145	0.1197235	0.529642	0.0002776
cg02399048	0.2759668	0.0972682	0.782966	0.0155284
cg02179341	0.4458764	0.1895863	1.048629	0.0641495
cg08734125	58879768	0.001092	3.17E+18	0.1558854
cg10150592	0.0329463	0.0017637	0.615454	0.0223164
cg26691533	1.2709412	0.572052	2.82368	0.5560901
cg02366421	4.7132289	0.168364	131.9435	0.3617859
cg25675673	0.5264923	0.2718068	1.01982	0.0571997
cg03518058	0.0010024	1.22E-05	0.082229	0.0021334
cg10713839	1.5362752	0.5576793	4.232076	0.4062787
cg11552023	2.4112767	0.4609977	12.61233	0.2971129
cg09080522	1.1073251	0.3797122	3.229206	0.8519032
cg16639998	0.9848959	0.0096152	100.884	0.9948587
cg13912702	5.132766	1.447603	18.19925	0.0113168
cg24411946	0.7890848	0.3919179	1.588738	0.5070583
cg16625109	1.4199193	0.7592996	2.655304	0.2723017
cg06691520	1.6266345	0.6837256	3.869885	0.271249
cg02255871	0.5046702	0.060457	4.212782	0.5276227
cg22158929	0.1701549	0.0384243	0.753499	0.0196609
cg06850548	375.70312	0.0022769	61993370	0.3334214
cg06276653	0.5851195	0.2706552	1.264949	0.1730508
cg26298967	3.9096967	1.3123431	11.64766	0.0143658
cg19149314	1.7581327	0.5792308	5.336441	0.3192288
cg02909991	2.1482213	0.8462675	5.453186	0.1076663
cg11231958	0.3908966	0.1714553	0.891195	0.0254884
cg18895336	1.0394411	0.4451406	2.427183	0.9287621
cg13430807	9.9647595	0.6984989	142.1569	0.0900066
cg05837036	6.3540295	0.9109266	44.32156	0.0620656
cg02833108	0.4912458	0.2304082	1.047369	0.0657462
cg02528400	0.8422224	0.4400529	1.611939	0.6041484
cg01182310	1.9443092	0.2846887	13.27885	0.4975819
cg07714085	8.382493	1.3481315	52.12117	0.022587
cg20919799	0.0045804	9.43E-05	0.222484	0.006557
cg07407787	0.38601	0.123467	1.20683	0.10169
cg10859329	4.416482	1.3629706	14.31088	0.0132786
cg08616702	0.234396	0.0476385	1.153299	0.0743388
cg06344544	0.0031896	2.87E-05	0.354253	0.0167665
cg00516515	1.0919086	0.0080481	148.1415	0.9720025
cg06872313	0.2463572	0.092639	0.655144	0.004994

cg11753157	1.390649	0.6338946	3.05083	0.410686
cg12565335	0.551129	0.2401361	1.26488	0.1598433
cg08685085	2.0556984	0.5976449	7.070914	0.2529216
cg14333542	0.4701578	0.1777187	1.243811	0.1284072
cg24593832	0.2273459	0.0876486	0.589698	0.0023191
cg10484107	0.0327437	0.0029764	0.360223	0.0051983
cg18533397	1.4930108	0.7891224	2.82476	0.217958
cg05578480	0.450863	0.2439929	0.833128	0.0109994
cg04404381	0.2077778	0.062148	0.694659	0.0107228
cg05397490	1.4796336	0.5448447	4.018238	0.4421106
cg06346081	0.3088583	0.0883486	1.079739	0.0657942
cg15114651	1.3930318	0.3686443	5.263984	0.625047
cg27180974	0.4023707	0.0957537	1.69082	0.2139011
cg11923320	3.2288173	0.9520551	10.95027	0.0599567
cg17842670	0.6691058	0.1784933	2.508232	0.5511804
cg15000232	2.74E-07	5.82E-12	0.012918	0.0059208
cg25149155	0.0398078	0.0020859	0.759696	0.0321424
cg10521599	3.7592349	0.86349	16.36597	0.0776644
cg04199779	0.0003509	4.56E-07	0.269985	0.0189688
cg22209431	0.2580797	0.0689757	0.965632	0.0442294
cg02313495	1.8497036	0.6663091	5.134859	0.2377604
cg19224787	0.3981581	0.1871683	0.846991	0.0167954
cg03935736	0.5549577	0.1826851	1.685841	0.2989349
cg03444184	7.0116963	1.2876624	38.18073	0.0242997
cg22902161	1.3812315	0.2136834	8.928166	0.7344618
cg19371526	0.3612293	0.1005545	1.29767	0.1186176
cg15825116	2.3937619	0.9739128	5.883582	0.0571249
cg19621753	1.3768196	0.7248027	2.615377	0.3286653
cg09318921	0.5971833	0.2692967	1.324293	0.2045405
cg07777349	0.0173575	0.001009	0.298604	0.0052289
cg05785768	1.26E+21	6241.0605	2.55E+38	0.0168576
cg23813755	5.76E-08	4.53E-17	73.17664	0.1190941
cg18851332	0.193487	0.0665433	0.562599	0.0025599
cg18524934	0.2519542	0.0892143	0.711556	0.0092575
cg23896685	0.2878558	0.0691538	1.198212	0.0869992
cg15402627	1.0872433	0.5500365	2.149126	0.8098728
cg10393744	0.2894328	0.1180468	0.709646	0.0067378
cg10343364	2.8432599	0.4880361	16.56461	0.2451769
cg05204566	0.2723744	0.1231267	0.602532	0.0013247
cg24924779	0.0536694	0.0052138	0.552459	0.0139412
cg09685096	0.1701871	0.0339828	0.852303	0.0312096
cg15819333	0.2034353	0.0647089	0.639571	0.006435
cg04548002	0.1192653	0.0268191	0.530376	0.0052236
cg05618222	0.3603698	0.156628	0.829139	0.0163645
cg16077086	1.3390371	0.6146466	2.917157	0.4624186
cg17892601	0.3822361	0.1837671	0.795052	0.0100605
cg22672431	0.3257338	0.154904	0.684956	0.0030986
cg21381065	0.0495272	0.006176	0.397177	0.0046654
cg10975897	0.1421554	0.0424632	0.475898	0.0015537
cg19269749	0.1866542	0.0692164	0.503346	0.0009123
cg01284289	0.4229882	0.1519763	1.177283	0.0994632
cg07078095	0.2047122	0.0437246	0.958432	0.0440233
cg14130595	0.3160574	0.1027833	0.971873	0.0444582
cg01726775	1.0592586	0.4232227	2.651155	0.9021155
cg06931464	8.9840739	1.4617007	55.21895	0.017803
cg04431629	1.4259233	0.7523365	2.70259	0.2767499
cg21475610	0.214644	0.101068	0.455852	6.22E-05
cg19728226	1.9394997	0.8600821	4.373604	0.1103407

cg15742858	0.448274	0.2068538	0.971457	0.042017
cg21896394	0.1446478	7.68E-05	272.4134	0.6152916
cg19977977	0.6786431	0.3504828	1.314063	0.2502074
cg03644238	0.4430039	0.2207833	0.888892	0.0219376
cg10731256	0.3033366	0.1229692	0.748261	0.0096121
cg04623371	1.730949	0.9125366	3.283358	0.0930052
cg09837088	2.1256199	0.8638604	5.230313	0.1007125
cg21649258	0.0630767	0.0096267	0.413296	0.0039613
cg08746496	0.140391	0.0380469	0.518036	0.0032055
cg09694722	0.099424	0.0133671	0.73951	0.024151
cg06951450	6948.0769	0.0073252	6.59E+09	0.2077393
cg08482167	0.4149424	0.2024033	0.850664	0.016326
cg13380109	10.134139	0.9293815	110.5044	0.0574483
cg00059225	0.2538043	0.089316	0.721222	0.0100741
cg02932355	0.284719	0.1288063	0.629355	0.0019081
cg14847983	28726.288	14.662621	56279135	0.0079478
cg04367503	3.524311	0.890298	13.95125	0.0727434
cg01312445	1.9734326	0.8247155	4.722157	0.1267498
cg19672751	0.7491865	0.1121887	5.003005	0.7656511
cg07210840	4.1358967	1.4853478	11.51625	0.0065836
cg07699978	0.9718746	0.0915829	10.3135	0.9811136
cg18650219	0.979195	0.4982324	1.924449	0.9513689
cg26423471	0.0008847	3.54E-06	0.221341	0.0125884
cg15672853	2.1893077	0.8051714	5.952854	0.1246952
cg18268988	0.0244782	0.0003287	1.822665	0.0916041
cg15336686	2.9940538	0.9355803	9.581602	0.0646362
cg17124700	0.4310796	0.1974539	0.941129	0.034663
cg13606629	1.3508851	0.4665088	3.911803	0.5792926
cg02467602	3.3841406	0.8419824	13.60172	0.0858636
cg04677410	0.9527147	0.4269361	2.125998	0.9058466
cg10301588	0.1184089	0.0353789	0.396301	0.0005368
cg03545246	0.0897209	0.0145134	0.554648	0.0094826
cg14318477	0.4035402	0.1602911	1.015931	0.0540528
cg24324628	0.4157977	0.2117399	0.81651	0.0108119
cg06370069	0.9378286	0.3778273	2.327843	0.8899393
cg01449674	0.3403266	0.1561267	0.741847	0.0067072
cg22810710	3.7493015	0.942729	14.91124	0.060623
cg19923326	0.1835245	0.0525553	0.640872	0.0078764
cg03479529	4.87E-07	8.67E-20	2739741	0.331879
cg11556846	0.4580927	0.1964149	1.068396	0.0707866
cg00130393	1.5878764	0.6535646	3.857846	0.3072923
cg16742874	0.2104283	0.0781641	0.566501	0.002038
cg25834632	1.0994792	0.0006991	1729.092	0.979853
cg00227665	1.2657683	0.0559682	28.62643	0.882251
cg26162616	0.9702009	0.139303	6.757141	0.9756284
cg02927252	2.0907433	1.1494408	3.802899	0.015681
cg16387436	2.7421363	1.0199851	7.371982	0.0455888
cg18470038	0.2702962	0.0232881	3.137221	0.295608
cg15906799	1404783.6	0.0659406	2.99E+13	0.1001449
cg14519350	4.1708442	1.0349432	16.8086	0.0446153
cg02822745	0.6055961	0.2734945	1.340965	0.2162395
cg10869879	0.0299191	0.002214	0.404316	0.0082507
cg26858144	0.252627	0.043814	1.456622	0.1237582
cg14438609	0.002432	6.99E-06	0.845542	0.0437829
cg22277567	1.2626524	0.4297983	3.709393	0.6714528
cg05042743	1.1512353	0.4275299	3.1	0.7805047
cg01582937	0.2331428	0.0800479	0.679038	0.0075933
cg10223989	0.6423711	0.3503864	1.177673	0.1523891

cg22424782	1.6026499	0.3816451	6.730039	0.5194202
cg13401963	107871.15	0.0178146	6.53E+11	0.1458197
cg07782808	1.2958623	0.371219	4.523634	0.6844959
cg27237671	0.3930691	0.1213095	1.273629	0.1195344
cg07254706	0.629982	0.3292839	1.205274	0.1627402
cg05267963	173.13113	9.04E-10	3.32E+13	0.6973819
cg25212763	5.5633417	1.4369338	21.53945	0.0129612
cg01424263	3.6179956	0.340733	38.41686	0.2860696
cg08591091	1.62E-06	1.41E-11	0.186316	0.0249249
cg10089647	1.52E-41	3.17E-92	7.28E+09	0.1144382
cg05852888	0.3117623	0.1159679	0.838126	0.0208913
cg09975576	0.2141046	0.0833538	0.549954	0.0013638
cg12903911	4.3575711	1.4946746	12.70405	0.0070146
cg13921605	1.19619	0.4555523	3.140958	0.7160821
cg02499214	1.7402898	0.6997345	4.328225	0.2333116
cg15236484	12.095723	1.8447738	79.30865	0.0093714
cg14059339	0.3737084	0.1757823	0.794494	0.0105343
cg25518707	1.5461936	0.7538577	3.171308	0.2344237
cg11123440	1.4141056	0.6915035	2.891807	0.3424625
cg25665622	0.1517831	0.0109765	2.098859	0.1594993
cg03840504	2.5990972	1.0489619	6.439992	0.039092
cg21963436	1.5908818	0.6961426	3.635613	0.270884
cg07003632	0.3483892	0.1187592	1.022026	0.0548217
cg02545261	1.7453405	0.7243434	4.205482	0.2145146
cg16837769	11257810	80.064475	1.58E+12	0.0072607
cg20099806	0.0555087	0.0073313	0.420282	0.0051228
cg24902932	0.0205554	5.02E-06	84.24219	0.3600359
cg07349094	3.4741589	1.404041	8.596458	0.007058
cg12536279	0.0968845	0.0028488	3.294892	0.194533
cg14467816	2.639017	0.3534713	19.7029	0.3441075
cg08071700	9.8236717	0.0967612	997.3477	0.3324328
cg13095450	5.082865	1.4190218	18.20657	0.0125051
cg09107315	1.8947812	0.7780702	4.614231	0.1593173
cg19610519	6.6155208	0.3453941	126.7107	0.2097469
cg21506220	6670.5399	5.76E-25	7.72E+31	0.7894082
cg05285352	1.7970015	0.6322701	5.107334	0.2714326
cg18850728	0.3155785	0.1383148	0.720022	0.0061357
cg14382976	4.201891	1.3492644	13.08557	0.0132562
cg09422614	1.3838736	0.4206223	4.553031	0.5928642
cg00215611	1.293944	0.4653803	3.597684	0.6213678
cg20856330	0.1148962	0.0134603	0.980747	0.0479588
cg08220122	0.1682949	0.0024182	11.71229	0.4103731
cg15772924	0.4618157	0.2017807	1.056958	0.0674242
cg16212053	0.4901794	0.2055587	1.168891	0.1078335
cg22994198	0.1644805	0.0347855	0.777734	0.0227811
cg17928895	0.409715	0.1763237	0.952035	0.0380583
cg17936564	0.1054895	0.0049543	2.246134	0.1494783
cg24767368	0.3387751	0.1486388	0.772131	0.010018
cg13516654	0.5246811	0.2077884	1.324858	0.1723384
cg04525464	1.0848381	0.0800523	14.70131	0.9511745
cg16583884	0.2490751	0.1083166	0.572751	0.0010689
cg25112877	1.0953689	0.3880462	3.091985	0.8634017
cg20935368	0.2488888	0.0778443	0.795764	0.0190168
cg11448015	4.54E-11	8.75E-18	0.000236	0.0025388
cg03755522	3.6719496	0.766313	17.59492	0.1037311
cg11872776	1.5819048	0.537756	4.653454	0.4047886
cg07201017	4.2678007	1.4404842	12.64444	0.0088294
cg02920600	1.7047473	0.8197487	3.545188	0.1533183

cg14913946	2.51E-21	8.32E-34	7.56E-09	0.0012141
cg16598679	2.3933228	1.026446	5.580414	0.0433424
cg01501135	0.0003164	2.55E-09	39.25233	0.1780875
cg03191045	0.8783321	0.3659673	2.108023	0.7714863
cg12665414	0.778916	0.3945793	1.537613	0.4714879
cg04936619	0.523864	0.2558325	1.072708	0.0770562
cg08176081	2.9653707	0.9572441	9.186187	0.0595353
cg20242781	0.5048734	0.2131394	1.195917	0.1203439
cg02098752	3.1539857	1.1597998	8.57702	0.0244235
cg19500415	5.0437272	0.5680794	44.78103	0.1463918
cg22614759	2.1075256	0.7835556	5.668601	0.1397307
cg04640675	2.4370033	0.9731761	6.102683	0.057183
cg27609554	1708644.9	0.0070939	4.12E+14	0.1449999
cg06791328	5.5964854	1.5477293	20.23652	0.0086395
cg14338916	2.3029913	0.9778621	5.423841	0.0562955
cg15215077	0.8163117	0.3932693	1.694424	0.5859626
cg22190721	0.7875958	0.101558	6.107907	0.8192833
cg09627567	1.8012853	0.9441872	3.436425	0.0741478
cg14184130	0.3543968	0.1383655	0.90772	0.0306391
cg17554875	2.0948015	0.6164101	7.118951	0.2361144
cg27237508	1.1415326	0.5719263	2.278435	0.7073646
cg23634401	0.1172145	0.0239824	0.572888	0.008095
cg25179758	0.2917069	0.0901376	0.944034	0.0397748
cg05289897	0.408239	0.0144507	11.53291	0.5991972
cg20095851	2.4671704	0.2538425	23.97916	0.4363802
cg07888205	1.5037942	0.5790808	3.905149	0.4020654
cg26463106	0.605403	3.21E-25	1.14E+24	0.9859604
cg09912512	0.4018687	0.1657795	0.974176	0.0436042
cg19290455	0.3807764	0.1537961	0.942746	0.0368496
cg08363278	6.333E+10	0.4178206	9.60E+21	0.0582883
cg18954051	920543.59	0.118337	7.16E+12	0.0898225
cg00256374	470187.01	0.0131674	1.68E+13	0.1410291
cg21428990	1.6097232	0.849731	3.049446	0.1441721
cg04699519	0.1667453	0.0688914	0.403592	7.13E-05
cg19856593	1.8745492	0.8268194	4.249942	0.1324244
cg09364988	3.7563527	0.6440677	21.90792	0.1412988
cg01946191	1.3691628	0.4781757	3.92033	0.5582835
cg15463966	2.072746	0.9096827	4.722829	0.0827975
cg26604799	8.8166585	0.4798722	161.9879	0.1427612
cg12357053	0.2252968	0.0853995	0.594367	0.002603
cg23647768	0.1941277	0.0533826	0.705952	0.012825
cg16344081	1.7943124	0.5616493	5.732326	0.32388
cg06828431	0.5017385	0.2059894	1.22211	0.1289199
cg02884053	2.0690599	1.001201	4.275873	0.0496224
cg19993359	7.295E+10	2.15E-25	2.47E+46	0.5490069
cg16761390	2.2816168	1.1993565	4.340473	0.0119374
cg07712198	0.6370705	0.3084218	1.315921	0.2231471
cg05108467	1.2522185	0.7439547	2.107724	0.3972063
cg02516845	2.679655	0.8619991	8.330114	0.0885039
cg26311666	0.1626755	0.0112368	2.355052	0.1829291
cg13506670	0.6083968	0.318163	1.163387	0.1329898
cg25848875	0.2070184	0.0011328	37.83104	0.5533803
cg21549434	1.8641847	0.6093854	5.702769	0.2749447
cg02991558	0.2776691	0.0967915	0.796558	0.0171732
cg26866949	2.9011485	0.5284494	15.92709	0.2202435
cg24497361	0.9973338	0.3558981	2.79483	0.9959483
cg20053493	0.0056561	1.56E-05	2.049471	0.0851989
cg08188886	0.3508211	0.1299927	0.946788	0.0386482

cg24799451	8.33E-26	3.80E-48	0.001826	0.0277917
cg18334977	2.1399023	0.9912184	4.619751	0.0526842
cg10253371	1.7278364	0.6237309	4.786389	0.2928193
cg27565555	1.6956158	0.9351876	3.07437	0.0819898
cg03539850	154786887	0.0270149	8.87E+17	0.0999812
cg20097219	0.2354675	0.0746061	0.743169	0.0136576
cg15611018	0.2397048	0.0551209	1.042406	0.0568341
cg13990926	1.0803323	0.6004255	1.943818	0.7965403
cg02794695	3.2349148	1.4016047	7.466209	0.005939
cg27586243	0.4374922	0.2068347	0.925374	0.0305507
cg15935791	0.0124554	9.35E-05	1.658643	0.0788803
cg12734852	0.3599246	0.1414535	0.915819	0.0319919
cg26645401	0.3659516	0.0976763	1.371065	0.1357854
cg10956096	2.0882452	0.8436293	5.169058	0.1113268
cg10695105	2.3418186	0.4476109	12.25197	0.3135151
cg21552290	5.7143603	0.696906	46.85555	0.1044625
cg11153172	0.8010533	0.3021754	2.123556	0.6556267
cg00130165	0.403203	0.0740766	2.194657	0.2933908
cg12808359	2.6832021	0.9895392	7.275683	0.0524646
cg04104132	0.4057568	0.1591442	1.034525	0.0589071
cg20587236	0.2591946	0.0900267	0.746244	0.0123329
cg15153412	785985274	0.2482251	2.49E+18	0.0664878
cg17285883	0.2359319	0.0398327	1.397441	0.1115532
cg23169957	0.0983111	0.0155663	0.620899	0.0136331
cg05432996	1.9492077	0.5479698	6.933613	0.3026039
cg09158035	0.7970517	0.3956068	1.605866	0.5256397
cg05734456	1.2749422	0.5493952	2.958667	0.5717196
cg23254918	6.37E-18	6.79E-35	0.59755	0.0470568
cg23676302	2.1730296	0.8322931	5.673551	0.1129525
cg26132511	0.20331	0.0592585	0.697536	0.0113214
cg27534828	0.1022793	0.0113158	0.924464	0.042368
cg05275605	6.4662844	0.2878362	145.2661	0.2397489
cg09565670	2.1773313	0.6634298	7.145852	0.1994068
cg24192505	1.8335919	0.7616076	4.414425	0.1762251
cg07338205	0.3934504	0.192975	0.802193	0.0102774
cg02071243	1.8520213	0.7613877	4.50491	0.1741893
cg08291342	1.3222493	0.4843368	3.609767	0.5856598
cg05616999	0.0231506	0.0011708	0.457755	0.0133922
cg06592170	6.23E-19	3.72E-30	1.04E-07	0.001478
cg13675859	0.369497	0.1551517	0.879965	0.0245257
cg25642974	0.0028285	2.50E-05	0.320143	0.0150147
cg06298530	0.4221173	0.1655321	1.076426	0.0709542
cg24013213	1.6054376	0.5469445	4.712415	0.3888743
cg00188627	1.1562028	0.5074704	2.634252	0.7297488
cg26509573	1.37E-22	1.33E-46	141.1727	0.0743419
cg10317681	0.1736254	0.0013908	21.67527	0.4771359
cg21300403	2.6666825	0.0210325	338.1044	0.6913794
cg02181369	0.0581158	0.0085288	0.396002	0.0036599
cg26621943	0.1256769	0.0305003	0.517853	0.0040938
cg14932133	0.2735706	0.1103032	0.678502	0.0051596
cg03232933	0.2532282	0.1116208	0.574485	0.0010158
cg16046375	0.4197411	0.1261526	1.396583	0.1569606
cg24794857	0.0973356	0.0038032	2.491134	0.1590658
cg09786383	6.10E-06	1.24E-11	3.011059	0.072629
cg19092620	49.161466	3.99E-05	60601705	0.5862047
cg06391412	0.6157227	0.2737085	1.385103	0.2410375
cg16148593	0.0757753	0.0062258	0.922271	0.0430293
cg07304315	1.984653	0.7504384	5.248729	0.1671634

cg09506473	1.93095	1.0410571	3.581521	0.0368322
cg11320084	0.1777953	0.0447307	0.7067	0.0141663
cg11095099	5.2144089	0.341549	79.60808	0.2350336
cg12658906	0.0793286	0.011553	0.544707	0.0099381
cg17849542	3.1850066	0.7499049	13.5274	0.1164319
cg08950745	0.195292	0.0068012	5.60768	0.3403592
cg16311685	0.0038103	2.43E-06	5.977946	0.1378941
cg09624565	1.8378454	0.6842135	4.936581	0.2273501
cg03549506	1.2460266	0.5075148	3.059186	0.6312413
cg06507244	0.2858058	0.1214306	0.672688	0.0041335
cg10395330	0.4505146	2.34E-05	8678.676	0.8741389
cg06459327	141207.38	1.4077373	1.42E+10	0.0435738
cg20746552	0.2892319	0.0596569	1.402271	0.123512
cg25370441	0.4340179	0.2193801	0.858653	0.016497
cg00360798	54.507777	1.179779	2518.351	0.040904
cg12502297	0.1974759	0.0628675	0.6203	0.0054743
cg04463386	0.3098105	0.1093645	0.877639	0.0274093
cg10812016	2.3178283	0.9207692	5.834609	0.0743078
cg21072567	3.6642896	1.18229	11.35679	0.0244423
cg14348967	0.0711507	0.0028879	1.752969	0.1059592
cg05547200	0.7182159	0.1969564	2.619026	0.6160822
cg10791260	0.1861514	0.0426976	0.811576	0.0252295
cg15234492	0.5836868	0.2005417	1.69885	0.3232882
cg14020052	0.5222527	0.2861903	0.95303	0.034283
cg10796568	3.6035883	1.1220513	11.57331	0.0312865
cg11901456	143.08939	0.0255103	802601.6	0.2597525
cg04658841	33.047241	0.9151905	1193.325	0.055936
cg05779081	8.186E+10	3.29E-22	2.04E+43	0.5090925
cg27002522	0.2663789	0.0928231	0.76444	0.0139187
cg11201571	2.8529077	0.6840623	11.89816	0.1502003
cg02768671	0.2164652	0.0575404	0.814335	0.0235874
cg09649347	3.0973501	0.5910554	16.23127	0.1809795
cg26945715	0.0001909	1.18E-07	0.307923	0.0230539
cg22996742	0.4870534	0.1930642	1.228716	0.1275826
cg16511708	3.89E-17	2.35E-30	0.000642	0.0149619
cg25816739	3.0368231	0.8524821	10.81817	0.0865779
cg09504612	1.6548653	0.6375696	4.295341	0.300631
cg06911238	0.5695379	0.2933757	1.105659	0.0962713
cg06640279	1.171625	0.5041061	2.723048	0.7127977
cg27405988	0.2670878	0.1224763	0.582446	0.0009042
cg00902104	1.8095441	0.9587404	3.415366	0.0672566
cg21540035	0.4181539	0.1946689	0.898206	0.0254057
cg21565017	0.7751527	0.3111968	1.930809	0.584392
cg23889772	1.7355114	0.584687	5.151473	0.3206349
cg12174341	2.0307519	0.7697214	5.357722	0.1523739
cg00215432	3.2428867	0.9161187	11.4792	0.0681333
cg02358862	3.1644535	1.0027187	9.986616	0.0494612
cg05794813	2.1567276	0.1429625	32.53631	0.5788258
cg10773972	0.4334026	0.2153908	0.872079	0.0190967
cg05575639	0.3914401	0.1877169	0.816258	0.0123693
cg01107136	2.0190902	0.8428085	4.837072	0.1149544
cg23302603	1.2073879	0.6554797	2.223998	0.5453848
cg22727932	0.7420853	0.3982237	1.382867	0.3475995
cg24801210	1.47E+19	6.97E-06	3.10E+43	0.122475
cg24826236	0.0458612	0.0021955	0.95799	0.0468514
cg24495667	1.4669688	0.5351645	4.021189	0.4563844
cg02731042	1.0394375	0.4136881	2.611703	0.9344203
cg19553910	0.2314886	0.0605069	0.885634	0.0325678

cg03668763	0.5375714	0.246802	1.17091	0.1181189
cg20146573	0.4226611	0.1848167	0.966592	0.0413034
cg26549860	0.3905755	0.1903916	0.801239	0.0103351
cg03113916	0.4263541	0.0828096	2.195129	0.3079195
cg15574642	0.1572927	0.029338	0.843309	0.0308596
cg15929771	0.3697991	0.1679876	0.814056	0.0134749
cg12981137	0.2587213	0.0789696	0.847627	0.0255488
cg12404590	1.0133882	0.3784082	2.713883	0.9788895
cg19952095	24.64316	8.76E-26	6.94E+27	0.9178613
cg09830037	3.186E+19	0.0004474	2.27E+42	0.094385
cg04414860	0.888954	0.4070226	1.941512	0.76774
cg27629673	1.0987845	0.4950281	2.438907	0.8168756
cg21942218	5.4085402	1.291499	22.64989	0.0208861
cg08395899	931988.57	233.30599	3.72E+09	0.0011597
cg17697016	1.3920614	0.6027046	3.215232	0.4386469
cg12233487	3.625567	0.7670039	17.13777	0.1041099
cg08275638	2.3675845	0.6876632	8.151456	0.1718342
cg22608160	1.8538269	0.3174741	10.82506	0.4929745
cg07061269	0.0063224	2.14E-08	1866.354	0.4307238
cg09258804	3.3348376	1.1818716	9.409771	0.0228647
cg18211918	0.243357	0.0898815	0.658897	0.005421
cg18090390	0.6622144	0.3302172	1.327998	0.2456642
cg15473473	12.006703	0.1565011	921.1499	0.2616896
cg11941630	0.3521084	0.1742396	0.711551	0.0036368
cg05459971	1.3300123	0.6125026	2.888041	0.4709871
cg02331808	1.4026591	0.6862123	2.867119	0.3536034
cg24221738	1.265488	0.5711066	2.804135	0.5618984
cg15489541	0.0804134	0.0088378	0.731667	0.0252682
cg17757079	2.0664813	0.4466726	9.560346	0.3530197
cg02320355	0.0238161	0.0030672	0.184923	0.0003516
cg14812743	0.9368359	0.3884636	2.259314	0.884498
cg20199836	0.2183632	0.0741769	0.642821	0.0057429
cg14524975	1.0006477	0.4008068	2.498201	0.9988933
cg00469602	1.5723112	0.5833138	4.238135	0.3710491
cg09152259	0.0934739	0.0062132	1.406263	0.0866244
cg27365295	1.7648502	0.1860193	16.74394	0.620709
cg19977966	0.1417818	0.004751	4.231091	0.259554
cg08362283	0.2201263	0.0558687	0.867312	0.030507
cg18138036	4.36E-09	9.35E-15	0.00203	0.0038406
cg15744128	0.3814895	0.1372098	1.06067	0.064737
cg03254067	1.3455443	0.5396105	3.355179	0.5243504
cg25221207	1.7767948	0.5603407	5.634072	0.3289425
cg02918581	1.72E+16	2.08E-07	1.42E+39	0.1649825
cg11662098	8.54373	0.4098981	178.0816	0.1662334
cg01904985	4.0682938	1.0697845	15.47135	0.0394996
cg18393747	2.167091	0.9957474	4.71634	0.0512688
cg15377871	1.2735481	0.2859115	5.672821	0.7510543
cg01361068	0.4497181	0.1920106	1.053308	0.0657156
cg26741380	0.4034739	0.1754353	0.927927	0.0326795
cg10696085	1.3775737	0.651012	2.915014	0.402256
cg01764198	1.6713224	0.9116032	3.064183	0.0967711
cg18471488	0.3484822	0.1221346	0.994311	0.0487664
cg07305719	1.3643831	0.211075	8.819334	0.7441933
cg02604730	0.4182305	0.1825316	0.958282	0.0393318
cg11237751	0.0487978	0.0031079	0.766197	0.0315938
cg07636870	5.713E+13	0.2936308	1.11E+28	0.0591651
cg18066515	1.7498447	0.6510019	4.703452	0.2673834
cg05848175	1.1368337	0.4655968	2.775773	0.7782673

cg05908442	0.1037722	0.021235	0.507118	0.0051294
cg26651775	0.5182457	0.2393642	1.12205	0.0953602
cg03059876	0.2579569	0.0631832	1.053155	0.059052
cg00325661	13.540183	1.0355028	177.0508	0.046971
cg04571522	1.4642043	0.6962695	3.079116	0.3146957
cg01958256	27.024793	0.11453	6376.837	0.2369546
cg09822018	1.28E-07	6.98E-16	23.30646	0.1019329
cg22955140	0.5004572	0.2437366	1.027574	0.0593136
cg13913015	0.1846492	0.038799	0.878769	0.0338106
cg05305099	0.2675883	0.0919754	0.778507	0.0155427
cg24893721	44314.961	1.52E-20	1.29E+29	0.7097032
cg18055467	1.6351787	0.8502722	3.144651	0.1405257
cg23429794	2.4553527	0.9067003	6.649118	0.0771832
cg22571664	2.0735164	1.1280228	3.81151	0.0188844
cg18504989	0.2339987	0.0792938	0.690538	0.0085232
cg25076459	2.8431584	1.0857213	7.445327	0.033386
cg19062099	1.0910686	0.4979212	2.390801	0.8276168
cg06631625	0.392932	0.1317136	1.172207	0.0939247
cg07348311	1.6448125	0.097651	27.70488	0.7298133
cg26500804	6.0974979	0.1735174	214.2695	0.319488
cg11363444	0.4914369	0.2393619	1.008975	0.0529143
cg07462521	2.05077	0.6190352	6.793891	0.2399108
cg06901094	0.1543414	0.0336869	0.707137	0.0161197
cg10159215	0.0853539	0.0117322	0.620968	0.0150757
cg03573443	0.0001493	3.20E-37	6.96E+28	0.8184566
cg15731317	0.6702534	0.3074143	1.461349	0.3143884
cg06692929	1.6450365	0.9033817	2.995572	0.1035893
cg23862389	3.883E+09	0.0001685	8.95E+22	0.1595762
cg22274133	1.2334526	0.5090399	2.988774	0.6421841
cg07892597	3.3700488	1.2550311	9.04936	0.0159218
cg12120457	6.69E-11	8.61E-20	0.051934	0.0248866
cg25426807	2.0154822	0.8350352	4.864668	0.1190071
cg07937271	2.5912525	0.9341575	7.187856	0.0673816
cg02481307	0.1200626	0.0263818	0.5464	0.006112
cg25096264	0.5944835	0.2501353	1.412878	0.239018
cg06736785	2.3308827	0.9664696	5.621506	0.0595604
cg25044990	0.9494962	0.47118	1.913373	0.884742
cg20777829	0.3780237	0.1468923	0.972834	0.0436881
cg24112692	0.3679921	0.1550634	0.873308	0.0233789
cg17308545	0.4784416	0.2153819	1.062793	0.0702323
cg05168977	0.1038877	0.0116967	0.922708	0.042138
cg14020824	0.4019216	0.197311	0.818713	0.0120396
cg23351327	0.1306748	0.0312708	0.546066	0.0052841
cg00376910	0.1680959	0.0486182	0.581186	0.0048419
cg15581429	5.0823105	0.7525069	34.32511	0.0952765
cg07416733	2.4723861	0.8133201	7.515729	0.1105547
cg14277260	2.632E+12	5.49E-08	1.26E+32	0.2161287
cg15558098	1.2850446	0.4982627	3.314195	0.6038826
cg02783918	2.3014049	0.8908283	5.94555	0.0852074
cg22033586	0.2398762	0.0746754	0.770543	0.0164961
cg04473763	0.8115235	0.3866013	1.703487	0.5809447
cg14311811	2.0259364	1.0732018	3.824461	0.0294145
cg22704520	95.332033	1.1537888	7876.828	0.0430238
cg06842954	1.9554504	0.9730812	3.929565	0.0596556
cg19348272	0.7236685	0.0907883	5.768322	0.7600818
cg12776171	2.5923623	1.0172157	6.606605	0.0459638
cg09290694	1.6568714	0.0633908	43.30632	0.7616922
cg23525243	1.3008538	0.1242977	13.61426	0.826226

cg19256400	1.9212044	0.7807789	4.727364	0.1552288
cg03352428	0.0327462	0.0039432	0.271943	0.0015474
cg16935039	1.1022442	0.4063237	2.990085	0.8483765
cg03289872	0.4366928	0.1855056	1.028005	0.0578629
cg09716613	1.4251147	0.7517333	2.701692	0.2776949
cg13023621	0.2417741	0.1027822	0.568724	0.0011416
cg08175413	3.1117077	0.5919892	16.35625	0.180001
cg03029292	0.9629617	0.2776883	3.339339	0.9525646
cg06767314	1.1287817	0.2206153	5.775429	0.884363
cg01205019	1.6163706	0.078638	33.22382	0.7555585
cg12873454	1.9809645	0.82588	4.751562	0.1256723
cg21038682	0.1995464	0.0549138	0.725114	0.0143565
cg03374559	1.67E-21	1.74E-32	1.60E-10	0.0002089
cg21146272	473493.95	11.794782	1.9E+10	0.0156822
cg11461298	1.3010922	0.5681085	2.979785	0.5335832
cg24428600	1.8242417	0.4970187	6.695639	0.3648561
cg18489755	1.6667779	0.131577	21.11424	0.6933069
cg02525108	0.1037323	0.0195912	0.549245	0.0077081
cg03209871	0.401968	0.1305543	1.237632	0.112198
cg17860962	10.69553	0.2503848	456.8742	0.2160519
cg05583398	0.0240833	0.0014677	0.395176	0.0090448
cg18560936	1.0534441	0.4771965	2.325551	0.8974662
cg05941027	0.6680536	0.1936365	2.304811	0.5231931
cg00989160	1.9702909	0.0439269	88.37523	0.72673
cg01726295	0.5850341	0.3026599	1.130856	0.1108798
cg25662857	1.9433321	0.7768201	4.861537	0.1555638
cg13775636	0.2895735	0.1182535	0.709094	0.0066819
cg21504793	1.306282	9.47E-10	1.8E+09	0.9801478
cg10218510	3.5181931	1.1593474	10.67642	0.0263497
cg03431846	2.110682	0.708033	6.292049	0.1801068
cg01670758	0.0254462	2.57E-06	251.9712	0.434176
cg10035607	513192.83	9.62E-05	2.74E+15	0.2498913
cg16319578	0.5638963	0.2126278	1.495473	0.2496345
cg07338584	2.3578316	0.6811746	8.161446	0.1757593
cg14153654	1.6891146	0.5388303	5.295003	0.3685308
cg20070090	0.2987908	0.1016977	0.877856	0.0280292
cg11527988	2.8259374	0.7327919	10.89794	0.1314236
cg26724949	1.6060055	0.8780596	2.937447	0.124088
cg22909609	2.0181317	0.9121211	4.465258	0.0831042
cg04610450	4.0139385	0.7932892	20.31	0.0929509
cg24747763	0.4442647	0.1998901	0.987398	0.0464714
cg07660627	0.0019548	1.23E-05	0.309818	0.0158075
cg23105419	1.7660418	0.4769799	6.538858	0.394458
cg20600729	2.0892716	0.755628	5.776726	0.1556188
cg16111448	0.1931418	0.0638766	0.583997	0.0035831
cg27096807	0.7079918	0.1781311	2.813952	0.623794
cg13621882	1.0850278	0.215225	5.47002	0.9212394
cg10850197	52.126514	3.09E-05	87905040	0.588885
cg20375836	0.2529297	0.104763	0.610649	0.0022375
cg02389590	1.5077208	0.5932153	3.832035	0.3882815
cg15773261	1.912339	0.6859442	5.331396	0.2152125
cg16355271	2.700549	0.8983602	8.118085	0.0768779
cg07163991	1.2535167	0.3512824	4.473051	0.7277441
cg10547761	0.4823028	0.2120306	1.097086	0.0820367
cg02399645	1.5767193	0.0089145	278.8755	0.8630896
cg15944457	8084.5272	3.39E-07	1.93E+14	0.4604749
cg14263779	2.0865481	0.8915588	4.883226	0.0900029
cg25369184	0.257048	0.1050429	0.629016	0.0029268

cg23723410	1.6462416	0.7604845	3.563664	0.2058344
cg06127801	1.6648885	0.8529469	3.249737	0.1352161
cg26377281	0.1146279	0.0256137	0.512989	0.0046116
cg24065451	0.1007624	0.0221739	0.457883	0.0029654
cg20823695	0.2542618	0.0388266	1.665074	0.1532347
cg04590959	1171.6471	1.61E-23	8.50E+28	0.8160863
cg07224931	0.2468646	0.0865671	0.703988	0.0088852
cg27653384	0.4194898	0.1053126	1.670947	0.2179769
cg26504229	0.0740917	0.0051423	1.067538	0.0558827
cg08473330	3.0645548	0.9975128	9.414912	0.0505105
cg16318688	1.1847398	0.4993828	2.810686	0.7005327
cg13104257	0.3553439	0.1532399	0.823997	0.0159054
cg09165842	1.8467869	0.8233147	4.142549	0.1366749
cg01836137	0.2217337	0.0769328	0.639075	0.0052876
cg16596716	21.585432	0.0564782	8249.748	0.3112345
cg03379706	0.1743484	0.0521239	0.583176	0.0045779
cg00828709	0.8523835	0.3154359	2.303345	0.7528324
cg27478020	0.1769433	0.0383632	0.816119	0.0263864
cg14926097	2.9588907	1.1089622	7.894799	0.0302717
cg18126557	0.2404409	0.0897364	0.64424	0.0045923
cg15949553	0.9740484	0.2933034	3.234774	0.9657514
cg23967461	0.0799078	0.0070532	0.905304	0.0413214
cg09923769	0.3954996	0.1559543	1.002985	0.0507384
cg13644197	1.9983277	0.6365542	6.273328	0.2355794
cg15672022	0.9368971	0.3783311	2.320127	0.8879616
cg17634067	801.81888	0.0171159	37562358	0.2229799
cg16434331	0.3798652	0.1705603	0.846021	0.0178241
cg05278074	1.7820224	0.4192584	7.574335	0.4338897
cg27401784	3.89138	0.4949469	30.59487	0.196536
cg17774634	1329.8287	13.191976	134054.5	0.0022436
cg17627559	2.1321432	0.8500237	5.348127	0.1066033
cg08426733	0.0598151	0.00756	0.473263	0.0076111
cg16369400	0.3965795	0.1265675	1.24262	0.11247
cg06306219	0.139119	0.0369221	0.524187	0.0035648
cg06739333	0.6693919	1.24E-06	360958.6	0.9524678
cg08121478	1.4002905	0.6537056	2.999536	0.3863603
cg12542490	126.14345	0.7383259	21551.69	0.0651391
cg23904377	1.029156	0.3872223	2.735282	0.9540479
cg13520525	0.5352914	0.2205191	1.299375	0.1672236
cg26601039	1.8649627	0.6137918	5.666557	0.2717026
cg09546172	0.1100693	0.0190959	0.634443	0.0135459
cg14336501	10.2232	0.0469389	2226.593	0.3973714
cg18377660	0.8581914	0.4400241	1.673755	0.6536448
cg05429448	0.525504	0.220481	1.25251	0.1465317
cg05095428	0.2392444	0.0825125	0.693688	0.0084552
cg24710309	0.4634319	0.2300404	0.933615	0.0313827
cg11732134	2.0826004	0.7541209	5.751364	0.1569305
cg20073050	0.4390493	0.1694777	1.137402	0.0900998
cg21189224	0.6493942	0.2609568	1.616026	0.3533493
cg16865953	1.7083749	0.8791053	3.319904	0.1141403
cg03491459	10.917384	1.3446302	88.64092	0.02528
cg04177091	1.7294287	0.7759909	3.854328	0.1803397
cg07436562	0.3587615	0.1206789	1.066548	0.0651738
cg03126058	0.2261499	0.0859654	0.594935	0.0025933
cg13767223	4.1185924	1.1762937	14.42055	0.0268346
cg17118478	1.5033702	0.6305682	3.584262	0.3577172
cg00490406	1.1288488	0.6314042	2.0182	0.6826503
cg01427630	0.4906988	0.2012597	1.196391	0.1174343

cg02258482	0.3040745	0.1045985	0.883964	0.0287795
cg09640960	0.3026916	0.0393411	2.328916	0.2510075
cg09560599	337190.76	1.01E-09	1.13E+20	0.455733
cg23112188	0.2886521	0.1202009	0.693174	0.0054382
cg11141463	0.0179635	0.0006026	0.535525	0.0203134
cg11299537	2.3315875	0.8157393	6.664262	0.1141342
cg23152216	0.1206621	0.047802	0.304576	7.59E-06
cg13885156	2.0377446	0.6175239	6.724279	0.2425587
cg08663298	5.1583962	0.3314634	80.27751	0.2414038
cg27222162	4.2702184	0.307399	59.31954	0.2795612
cg22637834	0.2474204	0.0919187	0.665989	0.0057001
cg17478203	1.0883733	0.2005149	5.907572	0.9218356
cg03240966	15796.039	0.1440648	1.73E+09	0.1025233
cg00473416	1.2142118	0.6046941	2.438109	0.5852752
cg13349346	1.6229619	0.6503077	4.050399	0.2993707
cg23114771	0.84671	6.41E-16	1.12E+15	0.9925262
cg16284684	0.441712	0.1492114	1.307604	0.1400459
cg26337070	0.4171152	0.1655362	1.051039	0.063683
cg26174807	0.5008725	0.2540965	0.987315	0.0458433
cg24947456	1.4570965	0.5197409	4.084978	0.474161
cg05982345	0.4012831	0.1899967	0.847531	0.0166827
cg25310430	6.38E-42	1.33E-86	3051.748	0.0707455
cg08826345	0.9290899	0.0590873	14.60904	0.9582728
cg19464804	0.0101038	0.0001675	0.60938	0.0280369
cg23548612	0.2567252	0.0763568	0.863156	0.0279615
cg12226095	4.1236769	0.7545479	22.53629	0.1020595
cg11404544	1.5789842	0.6162965	4.045441	0.3412988
cg01617071	1.7159779	0.730133	4.032937	0.2155157
cg18090197	2.8652709	1.0553838	7.778949	0.0388524
cg24875484	0.0009324	5.32E-06	0.163448	0.0081188
cg11521470	0.7769277	0.3120708	1.93423	0.5875594
cg19946699	0.000357	8.97E-07	0.142059	0.0093524
cg21574271	0.585314	0.2766153	1.238516	0.1613372
cg21048669	0.4726916	0.1570669	1.422561	0.1825425
cg07685557	0.3705564	0.116095	1.182756	0.0936381
cg04187121	1.9198737	0.8558305	4.306828	0.1135817
cg27405731	0.3136395	0.1432679	0.686614	0.003726
cg09537259	1.4433076	0.8703134	2.393548	0.1550948
cg16915653	2.1796548	0.7656883	6.204738	0.144352
cg09408902	2.1775979	1.0027759	4.728806	0.0491866
cg19618297	2.0327073	1.0443708	3.956353	0.0368216
cg06996956	0.5016002	0.1856414	1.355316	0.1736837
cg26381263	7.88E-08	3.96E-18	1568.136	0.176427
cg24580146	0.3870168	0.1444567	1.036864	0.0590305
cg20272648	0.2076303	0.0631258	0.682927	0.0096604
cg11756029	0.9024767	0.3491809	2.332499	0.8322619
cg00049664	2.2917007	0.8827846	5.949234	0.0884154
cg18684142	0.5823661	0.2771459	1.223724	0.1535643
cg00629382	0.5380778	0.155176	1.865802	0.3286298
cg26512148	1.77E-05	9.66E-15	32459.99	0.3146903
cg11562513	7.3047769	0.9735029	54.81213	0.0531318
cg13865934	0.3714276	0.12547	1.099533	0.0736784
cg06663615	0.326733	0.164907	0.647362	0.0013439
cg09273683	0.1499382	0.0458986	0.489808	0.0016798
cg02011953	0.2597557	0.0852442	0.791526	0.0177297
cg12072757	7.58E-06	2.87E-12	19.99678	0.1180841
cg27268513	65919765	1.3985886	3.11E+15	0.045806
cg06296570	17.354199	0.0268708	11208.01	0.3873453

cg21512773	1.8148878	0.4706817	6.997973	0.3867202
cg00188055	0.0016202	4.98E-05	0.052744	0.0002995
cg00988396	2.9172833	0.7175146	11.86114	0.1346296
cg00425678	0.7551181	0.2896063	1.968891	0.5656691
cg06763375	1.4831807	0.4921232	4.470069	0.4837317
cg25456477	0.75056	0.3958971	1.422946	0.3793014
cg02934500	0.7809216	0.3568718	1.708845	0.535981
cg12129209	11187.1	11.694249	10701944	0.0077632
cg27370185	6025.4122	5.8388438	6217942	0.0139576
cg27492942	0.2542248	0.0688231	0.939078	0.0399519
cg02213822	1.5323028	0.6856886	3.424225	0.2982303
cg19309616	2.317709	0.9233239	5.817866	0.0734416
cg03803541	0.4935481	0.162278	1.501064	0.2134049
cg02878486	0.2690827	0.0227874	3.177438	0.2973338
cg04398171	8.2029262	0.8168972	82.37021	0.0737559
cg25055744	0.2988481	0.1174578	0.76036	0.0112462
cg23169762	0.5865372	0.2399549	1.43371	0.2420235
cg26361436	0.6961515	0.2972431	1.630406	0.4041967
cg21543670	1.2357134	0.4184155	3.649453	0.7016775
cg05392265	5.5606938	1.2477714	24.78123	0.0244304
cg08902940	0.6765462	0.3082768	1.484753	0.3298684
cg07936672	0.235983	0.0775434	0.718153	0.01099
cg08423682	0.3272886	0.1017849	1.052394	0.0608935
cg03773198	0.2431746	0.0461825	1.280438	0.0952564
cg06990194	0.1160409	0.0248525	0.541817	0.0061549
cg27404824	2.0291808	0.8339484	4.937446	0.1188245
cg23596244	3.4989634	1.3049987	9.381423	0.0128111
cg13534791	0.4614466	0.1544432	1.378714	0.166087
cg17151150	103194765	1.16E-08	9.14E+23	0.3246774
cg26322711	0.87518	0.1918361	3.99268	0.863306
cg00601648	0.720796	0.3739863	1.389214	0.328083
cg22938837	3.6467825	0.9346111	14.22947	0.0625169
cg16055410	3.1440606	0.7447138	13.27371	0.1190314
cg15612947	5.554864	0.6207167	49.71111	0.1251583
cg16708938	0.1532026	0.0369016	0.636044	0.0097952
cg08543242	6.28E-05	3.86E-11	101.9681	0.1847907
cg19763108	0.4716887	0.2090351	1.064368	0.0703379
cg23067535	7.4403656	1.1596573	47.73741	0.034332
cg24163242	0.3460371	0.1753747	0.682776	0.0022102
cg01123946	2.8097145	0.2741059	28.8009	0.3842925
cg11757651	0.0475801	0.002794	0.810251	0.0352535
cg22077313	0.1007087	0.0216586	0.468278	0.0034165
cg13433250	4.1433275	1.3307485	12.90038	0.0141645
cg01188314	1.6189682	0.8267793	3.170203	0.1599682
cg25490633	1.2065969	0.4687704	3.105734	0.6970333
cg14438812	2.5110414	0.5052359	12.47997	0.2604099
cg22726349	0.9432795	0.3866596	2.301187	0.8978874
cg00456593	1.4019138	0.276882	7.098196	0.6831044
cg21609526	2.0542467	1.0627521	3.970756	0.032277
cg24990131	1.9197385	0.940016	3.920567	0.0734264
cg00783104	1.8423707	0.7984205	4.251306	0.1520609
cg04132891	0.3541617	0.1328683	0.944021	0.0379746
cg02229135	2.2751776	0.9267585	5.585526	0.0728174
cg26378073	1.8569607	0.5122302	6.731941	0.3462405
cg07780669	18.178263	0.2915641	1133.367	0.1689929
cg24304309	0.6278028	0.1032254	3.818211	0.6132716
cg00270764	0.133372	0.0422473	0.421047	0.0005932
cg16709734	2.0683338	0.786222	5.441217	0.1408576

cg15840462	1.5197669	0.544655	4.24065	0.4240334
cg06419219	1.3191416	0.5352069	3.25133	0.5473075
cg25020570	0.2866961	0.096901	0.848233	0.0239847
cg10623219	0.1519576	0.0409101	0.564436	0.0048898
cg25046720	0.2794857	0.1087144	0.718509	0.0081413
cg06839417	152.62237	0.2973329	78341.78	0.114325
cg01534391	3.0815016	1.1082697	8.567997	0.0310063
cg09601923	4.1396578	1.5028955	11.4025	0.0059956
cg25746499	0.2483673	0.0726756	0.84879	0.0263214
cg15129052	0.5750881	0.251934	1.31275	0.188929
cg25481999	0.5753225	0.2605938	1.270161	0.1712711
cg00202190	9.9584859	1.1161074	88.85475	0.0395578
cg24890054	0.1001202	0.0067096	1.493994	0.0951471
cg10779336	0.830121	0.3354508	2.054253	0.6871456
cg19133023	0.0825944	0.0150517	0.453227	0.0040914
cg13019491	3.1102501	1.2555014	7.705014	0.0142238
cg01204305	0.4980235	0.1631173	1.520546	0.2209167
cg25722465	0.2848961	0.0786902	1.031459	0.0557764
cg22757399	2.0620108	1.0483829	4.055664	0.0360053
cg07457626	0.502883	0.2094504	1.207404	0.1239967
cg10740965	6.9721664	1.64E-07	2.97E+08	0.828484
cg26971710	1.3856794	0.5403876	3.553204	0.4971815
cg02023469	0.0005141	1.40E-06	0.188404	0.011934
cg10204320	0.440397	0.1344758	1.442263	0.1754448
cg23410129	1.8511281	0.3404376	10.0655	0.4759918
cg00718681	5.1399737	0.0858054	307.8983	0.43306
cg08266286	1.0994365	0.3242668	3.727673	0.8790511
cg15889260	0.2447757	0.0602331	0.994721	0.0491414
cg03466428	0.6244632	0.321207	1.214028	0.165081
cg09127314	0.6056635	0.149858	2.447839	0.4816317
cg19366753	0.8943851	0.4380496	1.826105	0.7592368
cg17992177	0.7332033	0.3514502	1.529625	0.4081588
cg08494871	0.656145	0.1418142	3.035846	0.5897958
cg22276332	1.5385105	0.7115666	3.326483	0.2735019
cg07523753	0.3283482	0.1157975	0.931044	0.0362308
cg06372919	3.049197	1.2825064	7.249556	0.0116341
cg27647054	1.6430526	0.6243017	4.324227	0.3145408
cg00752045	0.3222953	0.0609988	1.702889	0.1824702
cg14475966	1.5950609	0.6163413	4.127939	0.3358398
cg19517718	0.7021054	0.2197162	2.243585	0.5507252
cg03540622	2.3807019	0.303805	18.65585	0.4089352
cg10516993	0.4705346	0.2371318	0.93367	0.0310629
cg08694699	1.9264707	0.7087495	5.236391	0.1987218
cg06722639	0.6050461	0.2320652	1.577491	0.3041133
cg16862791	0.4755205	0.0817332	2.766558	0.408036
cg18757405	4.9412171	1.1547918	21.14288	0.0312397
cg12107018	1.6945871	0.8661483	3.315397	0.1234846
cg25894551	3.1199268	1.0942419	8.895604	0.0333008
cg16531955	0.772346	0.3454139	1.726967	0.5292241
cg03439662	2.5878785	1.1713647	5.717361	0.0187202
cg09215582	0.583288	0.2221862	1.53126	0.2736483
cg23405212	1.9087172	0.9967431	3.655106	0.0511615
cg11261412	0.3869936	0.1482753	1.01004	0.0524339
cg05887092	2.1111454	0.2795044	15.94585	0.4688713
cg27608032	0.2497274	0.0700872	0.889803	0.0323498
cg23971069	1.5730651	0.6128635	4.037659	0.3462205
cg14215459	5.826E+13	5.716457	5.94E+26	0.0380755
cg02948125	0.2535176	0.1152302	0.557763	0.0006469

cg20104535	1248.7661	0.1325669	11763245	0.126722
cg23021796	2.0259995	0.8059893	5.092715	0.1332673
cg09331409	1.4966234	0.7126735	3.142928	0.2868087
cg10389454	0.4323039	0.1889785	0.988931	0.0469969
cg12187999	0.4153136	0.1508926	1.1431	0.0889321
cg21146912	145.56407	3.073987	6892.969	0.0113894
cg21773633	3.0806423	0.5898194	16.09028	0.1821988
cg02377941	9.0291544	0.9421267	86.53362	0.056357
cg12133451	0.9817343	0.4138191	2.329043	0.9666392
cg15859850	747169.53	30.552122	1.83E+10	0.0087102
cg25574761	0.0861916	0.0055589	1.33641	0.0796672
cg18674643	1.4324457	0.6459882	3.176375	0.3764263
cg02287004	0.9325994	0.4521409	1.923607	0.8501656
cg25804065	6.69E-09	2.61E-17	1.713867	0.0567248
cg04377908	0.7872075	0.2730295	2.269702	0.6578684
cg02393479	0.7590468	0.4159673	1.38509	0.3689746
cg04786391	0.574777	0.2474088	1.335315	0.1978828
cg00800383	0.0621519	0.0081292	0.475185	0.0074309
cg14606858	1.1731663	0.4521194	3.04415	0.7427011
cg11191302	2.7911025	0.8589468	9.069541	0.0878055
cg05374982	1.2134996	0.4370313	3.36951	0.7103586
cg12171875	0.2128924	0.0267737	1.692824	0.1436434
cg21510194	0.1888552	0.0459853	0.775602	0.0207484
cg04747824	2.4678474	1.0698619	5.692577	0.0341481
cg15810415	0.4871018	0.2288521	1.036775	0.062005
cg12810523	1.4706992	0.6601793	3.276316	0.3452298
cg01938825	0.974911	0.2949598	3.222309	0.9667724
cg23281012	504.80378	2.5944872	98218.58	0.0206411
cg11271157	199.20848	0.0040767	9734244	0.3365053
cg08543849	49.49275	0.57514	4259.019	0.0860506
cg14323834	0.4775189	0.1710431	1.333139	0.1582291
cg09894631	16.227957	1.1286861	233.3214	0.040465
cg15844154	2.91E+22	0.7312599	1.16E+45	0.0513939
cg03530006	0.4617921	0.1956951	1.089715	0.0777608
cg06800840	1.4861654	0.6003693	3.678881	0.3916014
cg06741653	0.5075031	0.182776	1.409153	0.1930188
cg11018604	0.4813179	0.2427134	0.954487	0.0363212
cg20417024	5.3583303	1.284026	22.36069	0.0212823
cg13567360	1.186059	0.051877	27.11674	0.9148948
cg01257145	0.0952389	0.0125905	0.720419	0.0227505
cg01606885	0.9853326	0.4571897	2.123583	0.969915
cg21964793	0.1689218	0.018645	1.530414	0.1137595
cg07025157	2.6510432	0.8885999	7.909105	0.0804321
cg00156230	0.4314084	0.2089475	0.890718	0.0230362
cg11121994	8.29E-08	4.36E-16	15.7743	0.0936646
cg03660115	1.1847777	0.6065817	2.314113	0.6196158
cg12291408	0.3077668	0.0402218	2.354954	0.2563761
cg22480742	0.0463366	0.002883	0.74474	0.0301613
cg13403724	0.3399925	0.1319317	0.876172	0.0255054
cg07238439	0.4029716	0.1935124	0.839151	0.0151599
cg23248729	0.4552603	0.1262089	1.642214	0.2293077
cg02769206	0.3253997	0.1122552	0.943252	0.0386821
cg16485474	0.3877526	0.147399	1.020034	0.0548871
cg19768079	0.5612259	0.2744269	1.147754	0.1135495
cg11965634	5.64E-07	1.05E-10	0.003035	0.0010283
cg22544144	4.68E+28	165945.01	1.32E+52	0.0165641
cg22531668	2.7901776	1.2730269	6.115418	0.0103801
cg03211636	1.1955282	0.5614952	2.545503	0.643252

cg22776369	80.347314	3.684477	1752.132	0.0052831
cg26492686	4.1478711	0.8779633	19.5963	0.0725453
cg09852209	0.358982	0.106782	1.206833	0.0977086
cg00018606	2.2148134	0.9386885	5.2258	0.0694465
cg26091142	0.083984	0.0062228	1.133461	0.0620954
cg19838154	0.0354342	0.0032553	0.385708	0.0061053
cg11086312	2.9420825	1.0859513	7.970753	0.0338279
cg25671438	2.8530776	1.0371532	7.848457	0.0422935
cg07841173	2.0103599	0.8550328	4.726774	0.1093955
cg13725062	23.192643	0.7782407	691.1727	0.0694928
cg26649096	0.6337187	0.2444869	1.642621	0.347896
cg16784366	0.551026	0.2734712	1.11028	0.0954548
cg02611812	0.5782515	0.2531625	1.320791	0.1936871
cg25577023	1.4090606	0.7124853	2.786657	0.3243167
cg07504763	0.4637202	0.2190349	0.981745	0.0446319
cg18061656	5.03E-07	1.24E-40	2.03E+27	0.7133706
cg12622519	0.5258294	0.058489	4.727323	0.5662021
cg27083787	1.28762	0.5086778	3.259363	0.5936953
cg01242400	0.0466135	0.0032556	0.667405	0.023962
cg14301212	2.1974633	0.5776785	8.359053	0.248103
cg10602135	3.1075805	0.6702241	14.4087	0.1474202
cg14906110	0.2713024	0.05831	1.262305	0.0963101
cg02672936	0.2153562	0.0511536	0.906648	0.0362967
cg21239317	0.3607258	0.1407557	0.924461	0.0337082
cg21839504	1.3949278	0.6329485	3.074221	0.4090575
cg03371662	2.0927715	1.0002504	4.378596	0.0499223
cg17907648	2.3293731	0.1726458	31.42839	0.5241753
cg25945961	460.90142	0.7796267	272476.7	0.0596307
cg15463484	3.4620481	0.0207178	578.5266	0.6344173
cg23989821	0.99029	0.5338965	1.836825	0.975305
cg08032641	1.5363845	0.2980987	7.918443	0.6077496
cg04042861	0.5406305	0.2246224	1.301212	0.1699334
cg24158936	0.8390065	0.4381812	1.606486	0.5963626
cg13667021	0.9297205	0.4261687	2.028258	0.8547215
cg19593878	2.4784967	0.759459	8.088581	0.1325742
cg12944192	0.553043	0.262859	1.163577	0.1185793
cg18184092	0.2572304	0.100499	0.658389	0.0046316
cg06884401	0.5495278	0.2183501	1.383012	0.2035973
cg22018086	4.672418	1.391265	15.69183	0.0126242
cg16570414	0.3352293	0.1047578	1.072748	0.0655284
cg01062942	0.376164	0.1789119	0.790888	0.0099172
cg22851420	2.5163325	0.6649173	9.522882	0.174153
cg17526952	146.47372	9.7774566	2194.287	0.0003051
cg10474881	0.5476079	0.2939827	1.020041	0.0577696
cg27519828	1.8707961	0.5320723	6.577823	0.3288725
cg07039975	0.0822482	0.0151499	0.446523	0.003803
cg03486485	1.7322785	0.6046814	4.962595	0.3062273
cg23485738	0.3507071	0.1254487	0.980445	0.0457584
cg10658438	0.5576508	0.2671698	1.163958	0.1198109
cg04003264	0.8891923	0.4548762	1.738194	0.7312923
cg10307548	2.4415406	0.7987869	7.462717	0.1173812
cg23663129	0.0664962	0.0106261	0.416123	0.003767
cg15311822	0.0404669	0.0007858	2.083836	0.1107422
cg07451520	0.9062001	0.331847	2.474631	0.8476126
cg02161503	0.2768816	0.097276	0.788102	0.0161219
cg08823975	2.46E-25	2.41E-41	2.51E-09	0.0025881
cg07054247	0.0652549	0.0007509	5.670466	0.2308394
cg11955344	4.396E+11	452.55464	4.27E+20	0.0111135

cg20556639	1.6482587	0.5335594	5.09176	0.3851948
cg05687227	0.2714932	0.1140724	0.646156	0.0032077
cg17826834	1.2487759	0.7180771	2.171691	0.4313318
cg27278086	1.3592853	0.6404169	2.885084	0.4240552
cg15323828	2.6610159	0.7447565	9.507813	0.1319698
cg12145907	2.190744	0.9094506	5.277207	0.080401
cg23280720	2.7281841	0.9711108	7.664407	0.0568658
cg17611074	1.9981326	0.7470414	5.344461	0.1678989
cg01007305	3.7771438	1.2872884	11.08284	0.0155297
cg24132991	4.3369358	1.232437	15.26164	0.0222817
cg25298161	0.4874528	0.2225948	1.067456	0.0723773
cg00095396	1.1115185	0.4385535	2.817156	0.8236768
cg27594073	0.3831328	0.1442934	1.017308	0.0541639
cg26227465	1.6161827	0.7679978	3.401112	0.2060114
cg11034978	0.7854034	0.3136669	1.966603	0.6059868
cg25570913	1.2956305	0.7337933	2.287645	0.3719207
cg08716584	0.2276186	0.0618167	0.838126	0.0260488
cg07223632	27.486489	1.1238901	672.2251	0.0421972
cg11210878	0.346539	0.1254593	0.957197	0.0409187
cg05364141	2.05E-07	3.48E-12	0.012035	0.005981
cg04850731	1.457589	0.6044158	3.515073	0.4015137
cg02483128	1.77E-14	8.87E-25	0.000352	0.0088671
cg25723459	0.3206216	0.1275743	0.805791	0.0155546
cg00396865	0.1834013	0.0464717	0.723797	0.0154586
cg03997039	3.9180441	0.691782	22.19062	0.1227145
cg21712830	0.3237436	0.0840453	1.247065	0.1011971
cg04848686	0.7875017	0.198515	3.12399	0.7340232
cg19461519	6.400768	0.4674525	87.6449	0.1644064
cg09024435	1.4649417	0.7290049	2.943813	0.2835874
cg02611282	0.4598936	0.1931254	1.095155	0.0793213
cg01031101	1.7346105	0.7595182	3.961556	0.1911618
cg26464482	2144.1483	2.2316307	2060095	0.028593
cg06146977	0.3215458	0.1522402	0.679135	0.0029368
cg08060857	0.1666586	0.0465477	0.596701	0.0058979
cg12159215	0.2239442	0.0371238	1.350913	0.1026938
cg00934232	2.0881566	0.5433095	8.025625	0.2837901
cg13673023	0.2835402	0.0717109	1.1211	0.0723374
cg26955491	0.5409445	0.2382052	1.228441	0.1420225
cg02184413	1.9942359	0.9182725	4.330933	0.0810746
cg03186440	1.9012253	0.7882561	4.585639	0.152633
cg15587771	4.1435891	1.7520844	9.799374	0.0012082
cg00605777	0.3878147	0.151547	0.992433	0.0481766
cg02734259	0.5020694	0.1804472	1.396939	0.1869355
cg13630239	0.4309207	0.1661621	1.117539	0.0833799
cg00992048	0.3671845	0.1515755	0.889487	0.0264604
cg00082833	0.359907	0.0258928	5.002666	0.4466469
cg01174264	1.3457947	0.6921263	2.61681	0.3813864
cg06216883	0.0771308	0.0034597	1.719546	0.105722
cg06242719	3.8278475	0.8985289	16.30712	0.0694829
cg17504164	1.6015912	0.7089077	3.618375	0.2573626
cg08354681	1.0523317	0.0832531	13.30163	0.9685647
cg06477444	1.5475341	0.0420627	56.93546	0.812356
cg14413700	0.2947947	0.1078486	0.805795	0.0172735
cg25960038	1.4457737	0.4852759	4.307368	0.5080672
cg23768117	1.5992946	0.6304209	4.057199	0.3228546
cg08340277	0.4767458	0.1872523	1.213799	0.1202784
cg15605744	3.3561894	1.096162	10.27586	0.0339401
cg25730237	3.5895414	1.2880981	10.00297	0.0145203

cg09589565	1.8420967	0.758573	4.473295	0.1771597
cg25027367	0.7636979	0.2976361	1.959556	0.5749841
cg22260762	4.2255843	1.2005351	14.873	0.0247921
cg02066621	1.8662248	0.7921253	4.396773	0.1535854
cg23366050	0.2311061	0.0813622	0.656448	0.005956
cg18560014	8.2021199	1.0001142	67.26709	0.0499876
cg19937499	0.2650486	0.1020145	0.688635	0.0064159
cg06873218	1.6557943	0.6236763	4.395958	0.3114152
cg13587915	2.9927992	0.3795683	23.59746	0.2981142
cg13442388	2.7916136	1.0287792	7.575101	0.0438343
cg21371809	0.2837106	0.0657846	1.223565	0.0911443
cg08248176	0.0347749	3.35E-15	3.61E+11	0.8261474
cg06486618	0.4009778	0.2085798	0.770847	0.0061356
cg10623198	1.5510934	0.4123916	5.833996	0.5160513
cg15769343	2.8221315	0.8523261	9.344341	0.0894339
cg07684775	0.3097308	0.1358261	0.706294	0.0053243
cg27455606	0.1363932	0.0459386	0.404956	0.0003331
cg06732241	1.1272366	0.5697268	2.230301	0.7308366
cg24235581	0.0009531	3.88E-06	0.234292	0.0132613
cg09795809	3.9297775	1.504393	10.26537	0.0052129
cg01964170	4.3356303	0.9288738	20.23708	0.0620273
cg14609139	4.8700541	0.3594325	65.98576	0.2338517
cg07234388	0.1453743	0.034973	0.604286	0.0079803
cg26403171	0.2542002	0.1056945	0.611363	0.0022212
cg03470073	0.8684442	0.3906648	1.930543	0.7292924
cg23657782	4.7055768	0.9406331	23.53995	0.0593683
cg18587504	0.030875	0.0037921	0.251385	0.0011521
cg15065535	0.961564	0.4243373	2.17894	0.9251828
cg24718722	0.253464	0.0796816	0.806259	0.0200875
cg12443285	0.3628707	0.1531129	0.859987	0.0213023
cg07959864	0.5437979	0.2840055	1.041234	0.0660555
cg15379025	0.5250318	0.2586321	1.065832	0.0745081
cg05329785	1244.8111	3.2726419	473487.4	0.0187186
cg03640756	2.4317058	1.0135755	5.833994	0.0465729
cg05828515	8.72E-08	3.21E-20	236767	0.2657979
cg04938381	0.0580449	0.0136595	0.246658	0.0001152
cg16487621	2.1454347	0.7694051	5.982401	0.144579
cg16163981	1.6112874	0.619365	4.191788	0.3281217
cg16616765	0.4676248	0.2268651	0.96389	0.0394347
cg09180520	0.3546032	0.1474178	0.852973	0.020609
cg24159335	0.0648864	0.0096985	0.434115	0.0047958
cg24275315	3.2863178	1.314894	8.213502	0.0109055
cg20060396	0.5358362	0.1981302	1.44915	0.2190203
cg09571376	0.4331359	0.1582483	1.185521	0.1033777
cg20675703	0.0017126	1.38E-05	0.212535	0.0096102
cg00369314	2.4509766	0.4762249	12.61439	0.2835094
cg08970050	3.26E-21	9.51E-37	1.12E-05	0.0097475
cg10945385	0.1659656	0.0214998	1.281153	0.0850045
cg04857672	0.0171895	0.002143	0.137879	0.0001307
cg22988146	5.44E-05	2.06E-08	0.143938	0.014604
cg11350504	0.4857301	0.2128915	1.108234	0.0862027
cg05617030	7.0620412	0.693611	71.90258	0.0987444
cg00973737	0.8296742	0.3078885	2.235742	0.7119919
cg13344961	23373.225	24.961578	21885942	0.0039566
cg12401152	0.0808495	0.0126996	0.514712	0.0077401
cg26832915	1.6549605	0.6580128	4.162373	0.2843678
cg06824394	1.0124094	0.5408202	1.895219	0.9692475
cg20519577	2.2613493	0.1562814	32.72111	0.5494993

cg07232003	0.5804504	0.3147193	1.07055	0.0815639
cg17760405	1.7695772	1.0002324	3.130676	0.0499067
cg16989719	0.3808433	0.1416031	1.024282	0.0558205
cg12754495	0.3885025	0.1822614	0.828119	0.0143508
cg08871356	0.6817616	0.341609	1.360616	0.27724
cg08629043	0.5294035	0.2380825	1.177189	0.1187903
cg01471232	1.8559728	0.6852773	5.02663	0.2237904
cg20002051	0.0870321	0.0005441	13.92199	0.3457279
cg13784557	1.6179178	0.8577856	3.051646	0.137242
cg12102320	0.1572492	0.0249324	0.991775	0.0489814
cg18915379	7.49E-25	3.49E-45	0.000161	0.0200358
cg01535619	0.6475284	0.3189127	1.314758	0.2291036
cg24931993	0.7531953	0.3514919	1.613986	0.4660694
cg06868562	0.2994839	0.0821378	1.091952	0.0677465
cg11862081	0.303906	0.1169763	0.789552	0.0144838
cg10786098	0.1163351	0.0076024	1.780202	0.1221991
cg27478863	0.123691	0.0334515	0.457363	0.0017336
cg20288565	1.3270687	0.6846077	2.572439	0.4020655
cg25728206	0.7865438	0.3998572	1.54718	0.4866803
cg02929780	2.168903	0.6181474	7.610062	0.226709
cg01806261	0.4837055	0.1851482	1.263695	0.1382606
cg18010348	0.6772535	0.3338378	1.373938	0.280246
cg01065679	0.4844576	0.1698886	1.381489	0.1752508
cg06937978	2.8228185	0.5512033	14.4562	0.2130513
cg01995938	1.8937739	0.7520212	4.768987	0.1753657
cg09388414	2.7433568	0.8880583	8.474676	0.0794876
cg17797121	7.8735617	0.1351319	458.7589	0.3197701
cg02596089	0.3507779	0.1355646	0.90765	0.030794
cg04077417	1.9978687	1.0060943	3.967301	0.0480051
cg07413480	1.0731834	0.4844124	2.377566	0.8618426
cg09364426	0.0860039	0.0058088	1.273346	0.0743873
cg13774369	1.21E-25	1.44E-50	1.012843	0.050051
cg01924952	2.9327452	1.3151077	6.540145	0.0085545
cg17276002	0.9858741	0.4976145	1.953214	0.9674688
cg05404233	0.0671155	0.0174326	0.258394	8.58E-05
cg26298737	0.439376	0.1803057	1.070689	0.0703474
cg02143496	1.9739906	0.7502143	5.194034	0.1682874
cg27144709	136.3477	0.0004069	45685590	0.448908
cg01109462	5.4572463	0.3106482	95.86903	0.2458578
cg18031675	2.0239742	0.8217602	4.984996	0.1252489
cg10230591	0.2985024	0.088519	1.006605	0.0512537
cg23297413	1.7748372	0.7994561	3.940237	0.1585664
cg04436818	1.5683642	0.8522773	2.88611	0.1481002
cg18474072	0.244216	0.0964523	0.618352	0.0029384
cg01106338	0.5030971	0.1293648	1.956534	0.3214996
cg22079684	0.0001747	4.19E-19	7.29E+10	0.6144364
cg01449136	3.6327373	1.2381844	10.65817	0.0188237
cg21171335	2.2928648	0.6741335	7.798498	0.1839789
cg04928513	1127.5352	0.1343577	9462321	0.1273757
cg07719523	9.2172005	0.8522938	99.68016	0.0674901
cg08739188	3.0057841	1.0443336	8.651199	0.0413117
cg21708354	3.47E+26	175868633	6.84E+44	0.0044652
cg07563723	0.1408035	6.29E-25	3.15E+22	0.9430279
cg09558414	1.81E+10	1.5116377	2.17E+20	0.046058
cg24987440	0.7128709	0.3616374	1.405233	0.3283427
cg08594379	0.5388473	0.2242789	1.294622	0.1667929
cg27049201	0.0001956	2.38E-09	16.04414	0.1390813
cg08655664	7.9894487	0.74612	85.55097	0.0858211

cg05779272	1.4707349	0.4237756	5.104261	0.5434341
cg12560447	2.2399922	0.4968023	10.09972	0.2939259
cg02352252	0.504326	0.211215	1.204198	0.1231895
cg27237745	1.7620124	0.4787639	6.484799	0.3941822
cg06794263	6242193.2	6.23E-06	6.26E+18	0.2670851
cg00332802	0.9517447	0.4994111	1.813772	0.8805118
cg22111043	1.3795594	0.4731258	4.022575	0.5556588
cg03481445	2.5262651	0.1629989	39.15374	0.5075033
cg05944967	2.2201863	0.4884998	10.09054	0.3018264
cg13651908	1.6240095	0.3572537	7.382448	0.5302373
cg25363445	1.6395716	0.8625466	3.11658	0.1313615
cg04487205	2.3788695	0.6850255	8.261036	0.1724469
cg12125484	0.7346625	0.2604138	2.072582	0.5600939
cg08862499	2.91E-11	1.64E-26	51675.47	0.1756738
cg08942342	0.3479345	0.117297	1.032067	0.0570318
cg11342452	0.1785884	0.0424035	0.75215	0.018864
cg04516518	2.0307294	0.6120604	6.737672	0.2469944
cg01916632	0.4675688	0.1928018	1.133914	0.0925852
cg03704073	0.356787	0.1608115	0.791591	0.0112523
cg01548463	0.2506029	0.0932235	0.673669	0.00609
cg16655240	0.669689	0.3368205	1.33152	0.2528637
cg21869055	1.7042247	0.7722171	3.761095	0.1868497
cg23261171	1.1616248	0.4746952	2.842608	0.7428158
cg02262786	5394.2059	5.7811431	5033167	0.0137839
cg26827987	1.7126377	0.773369	3.792663	0.1847089
cg26422861	0.4274041	0.1431132	1.276432	0.1278242
cg26064794	0.2644484	0.1067631	0.65503	0.004051
cg26135388	0.4338219	0.1891736	0.994861	0.0485946
cg06585893	9.1134868	1.5256521	54.43944	0.0153851
cg24149455	0.5701289	0.2303437	1.411139	0.2243041
cg06414073	0.0125133	0.0013723	0.114107	0.0001025
cg18230796	1749.1714	4.96E-19	6.17E+24	0.7680194
cg10010249	1.1744921	0.4501956	3.064072	0.7423517
cg18425087	131.91653	0.1481394	117470.3	0.1588669
cg07055879	0.4043735	0.1364947	1.19798	0.1022642
cg16752876	1.839182	0.5427506	6.23231	0.3278028
cg05190718	1.4136112	0.4504261	4.436459	0.553054
cg20998435	0.0081652	0.0001102	0.60487	0.0286082
cg05664581	1.7071198	0.6652865	4.380456	0.265994
cg19554569	0.7574862	0.3373078	1.701074	0.501013
cg06383709	0.5686555	0.2828507	1.14325	0.1131397
cg20191307	2.31E-12	5.70E-33	9.38E+08	0.268437
cg24689177	0.7315472	0.3272326	1.635416	0.4463195
cg03551062	0.3971589	0.1994339	0.790915	0.008605
cg03363653	0.0335898	0.0025643	0.439995	0.0097249
cg09696411	5.7032693	1.1702976	27.79403	0.0311952
cg02294687	0.5902537	0.2586726	1.346874	0.2103881
cg07734975	0.3051383	0.1479618	0.62928	0.0013082
cg25832824	1.1831412	0.6249201	2.240003	0.6055833
cg00372692	0.8088997	0.1270015	5.152055	0.8223626
cg26607337	3.1404291	1.1593015	8.507101	0.0244054
cg00170003	2.4858422	1.0469238	5.902447	0.0390275
cg24442740	0.3455817	0.1756103	0.680067	0.0020961
cg22338446	2.3184195	0.6790367	7.915727	0.1795497
cg06786773	0.0882641	0.0170902	0.45585	0.0037582
cg09176893	19.141284	0.5597796	654.5232	0.1014216
cg01940181	0.3036471	0.1045269	0.882085	0.0284838
cg03833511	0.0179132	2.74E-05	11.71653	0.2239961

cg00301762	1.6309448	0.8079786	3.292143	0.1722587
cg11204212	3.6323798	0.7315826	18.03512	0.1146381
cg17393635	0.183555	0.0600098	0.561449	0.0029596
cg07398561	12.418949	3.56E-21	4.33E+22	0.9207098
cg06657240	0.548299	0.2391631	1.257016	0.155723
cg19300923	0.2039983	0.0821683	0.506464	0.000612
cg26495303	2.1223043	1.096767	4.106775	0.0254697
cg04042914	0.0161801	0.0014345	0.1825	0.0008502
cg22377814	0.1793758	0.0708883	0.453892	0.0002861
cg01434694	0.3626529	0.1094315	1.201821	0.0970681
cg09319828	2.089299	0.7610496	5.735724	0.1527109
cg23400607	1.0238266	0.5180316	2.023469	0.9459895
cg00476149	0.0098624	8.21E-05	1.184763	0.0586818
cg13170705	0.3016607	0.0760384	1.196753	0.088286
cg17984666	2.6994639	0.4843522	15.04505	0.2572488
cg04106630	4.2331772	0.1544676	116.01	0.3929748
cg12804677	0.2701507	0.0693165	1.052873	0.0593315
cg01049205	2.7389586	1.2232835	6.132588	0.0142846
cg20239081	0.141722	0.0144419	1.390754	0.0935657
cg03309932	0.6349725	0.0102427	39.36375	0.829228
cg14304515	3.0768195	0.7516897	12.59405	0.1180498
cg14314674	2.168855	0.9806941	4.796533	0.0558989
cg22282941	0.4341776	0.182049	1.035492	0.0599285
cg18693345	1.0385228	0.5281466	2.042103	0.912755
cg00149716	3.1268908	0.9865705	9.91054	0.0527463
cg10421739	0.615203	0.3017149	1.254412	0.1814133
cg05025612	0.7431688	0.2376016	2.324478	0.6099205
cg08241401	0.3211361	0.1289624	0.799678	0.014679
cg16203594	2.8255596	0.9388084	8.504171	0.0646538
cg26404511	0.348183	0.1459622	0.830568	0.0173837
cg18887969	3.6608678	1.0186334	13.1568	0.046784
cg03053579	1.7537725	0.8097661	3.798279	0.1542181
cg24603922	1.9943116	0.7722718	5.150102	0.153841
cg13142152	2.3342252	0.4465056	12.20278	0.3151389
cg04329454	3.91566	0.9816728	15.61864	0.0531428
cg19801921	2.8885096	0.8859795	9.417247	0.0785454
cg11328253	1.2965422	0.4841119	3.472383	0.605378
cg27110374	1.2865563	0.1915272	8.642259	0.7954187
cg01133856	63.589723	0.1481029	27303	0.179433
cg19628497	0.296313	0.0744593	1.179187	0.0843355
cg19381814	23.103798	0.407523	1309.829	0.1274534
cg10502121	0.2147202	0.0425765	1.08287	0.0623885
cg15937073	0.1376044	0.0334248	0.566495	0.006013
cg04544033	0.1988544	0.0709176	0.557592	0.0021381
cg01010517	0.8650234	0.3620105	2.066971	0.744234
cg16890093	1.5499266	0.8592304	2.795842	0.1454212
cg08856772	2589.4391	0.8031615	8348501	0.0565488
cg00301192	0.3393788	0.0001315	875.6198	0.7874532
cg07014973	2.273483	0.9451988	5.4684	0.0666385
cg17876514	0.3352863	0.1110305	1.012486	0.0526284
cg17454592	0.0889862	0.0049124	1.611945	0.1016473
cg11737710	0.016537	0.0004081	0.670039	0.0298576
cg04310104	1.830275	1.0554074	3.174041	0.0314015
cg13540171	0.2752224	0.0976168	0.775966	0.0147043
cg03207151	2.4169111	0.4936178	11.83397	0.2762122
cg22636722	1.2458737	0.4515274	3.437668	0.6711841
cg00278547	2.769992	0.8276682	9.270449	0.0983148
cg23436703	2.5328315	0.0232705	275.6816	0.6977345

cg07938847	0.3241735	0.119227	0.881415	0.0272929
cg24540114	2.6316749	1.063585	6.511668	0.0363201
cg04235075	1.9465828	0.6381603	5.937669	0.2417659
cg20953388	0.3509437	0.1612352	0.763862	0.0083207
cg09736286	3.9253175	0.2453668	62.79625	0.3336901
cg23793599	2.2228799	0.997184	4.955149	0.0508114
cg23554497	2.0455079	0.9236323	4.530052	0.0777094
cg00797651	0.4245508	0.1894455	0.951426	0.0374424
cg08564375	0.2230739	0.066293	0.750637	0.0153816
cg16480368	0.0742796	0.001739	3.172719	0.1747067
cg25450333	4.56E-17	3.06E-34	6.808507	0.0621967
cg14963406	0.1440141	0.0480457	0.431674	0.0005404
cg18813170	44598857	3.03E-09	6.57E+23	0.3537766
cg05348871	1.7422748	0.7199443	4.216329	0.2182252
cg20661290	0.5837884	0.2677029	1.273086	0.1760539
cg12993791	0.0652877	0.0042254	1.00877	0.0507352
cg20360285	1.093297	0.2700003	4.427026	0.9005188
cg08155109	1.4497209	0.8153439	2.577674	0.2059674
cg19020339	1.7881669	0.504695	6.335591	0.3678592
cg09548241	1.4624184	0.1281404	16.69003	0.759623
cg11194299	9.5753567	0.3665427	250.1413	0.1747547
cg05881566	45.140778	0.1236367	16481.27	0.205671
cg05279149	1.0748857	0.1677978	6.885546	0.9392523
cg27215578	2.12E-17	4.10E-35	10.94876	0.0650437
cg10812247	0.4054639	0.1778107	0.924584	0.0318409
cg24618169	2.3157538	1.1147121	4.810852	0.0243806
cg22908922	4.6597103	0.2094538	103.6644	0.3308983
cg06654697	2.2182103	1.0305919	4.774399	0.0416491
cg26263675	0.1286759	0.042727	0.387519	0.0002671
cg03424478	1.17E-06	7.61E-15	178.7027	0.1554031
cg08164191	2.1065539	0.5630465	7.881355	0.2684084
cg10811485	0.314219	0.1395185	0.707674	0.0051951
cg24030680	0.5433935	0.1605099	1.839616	0.3269504
cg05100419	2.4357085	0.8337006	7.116075	0.1036387
cg20431565	0.0012425	1.56E-05	0.09886	0.002733
cg16922039	3.9738897	0.8149526	19.37757	0.0878537
cg23629166	0.3760631	0.1089643	1.297888	0.121763
cg06588466	0.4367967	0.1495658	1.275634	0.1298336
cg06995967	0.4089716	0.1907232	0.876966	0.0216024
cg15043318	0.5236852	0.2034772	1.347798	0.1798741
cg14669524	6.389163	1.24926	32.67647	0.0259319
cg13384816	3.2775955	0.0496864	216.2088	0.5786133
cg03052030	0.3372583	0.1152421	0.986993	0.0472714
cg14087715	0.3782745	0.169327	0.845061	0.0177659
cg13811240	0.3638416	0.1447092	0.914805	0.0316143
cg19653282	0.2761591	0.1113959	0.68462	0.0054708
cg05626664	2.8092254	1.149911	6.862921	0.0234228
cg06535156	0.165325	0.0625363	0.437064	0.0002849
cg20677171	0.0630474	0.0055835	0.711918	0.0254371
cg00161225	2.2163217	0.8932001	5.49942	0.0860925
cg06707663	1.2248028	0.5090154	2.947144	0.65081
cg09046168	2.1726253	0.3678444	12.83233	0.3918346
cg27223158	0.5082901	0.1717382	1.504376	0.2215878
cg18645150	0.2400941	0.081968	0.703264	0.0092693
cg06798887	0.5497417	0.2336283	1.293576	0.1705674
cg15455643	1.6445174	0.6776834	3.99071	0.2714285
cg27553419	2.4917657	0.820634	7.565975	0.1071519
cg13493071	0.6585781	0.2284987	1.898151	0.4393205

cg00333800	0.3829989	0.1215712	1.206603	0.1011737
cg19671650	0.0150091	4.57E-09	49274.78	0.5833374
cg23750445	3.8770811	1.3647241	11.0145	0.0109698
cg15111638	2.1065622	0.6981997	6.355781	0.1860503
cg08493590	1.4972677	0.4629919	4.842008	0.5002798
cg10395252	0.4716615	0.0028851	77.10765	0.7725881
cg14554846	2.0488751	0.9458357	4.438286	0.0689473
cg05873857	9.51E-06	3.78E-13	238.963	0.1834989
cg14351952	0.3419694	0.1069211	1.093732	0.070463
cg08595782	0.2169566	0.0942505	0.499416	0.0003279
cg21816491	62.979367	0.001905	2082129	0.4352212
cg10761978	0.3473315	0.0986654	1.22271	0.0995924
cg01804183	0.932695	0.4094726	2.124489	0.8682411
cg26012061	5.8228738	0.9209906	36.81456	0.0611392
cg14186816	3.0660831	1.0235928	9.184185	0.0453252
cg23326607	3.5815619	0.9797039	13.09333	0.0537357
cg18765405	1.9503396	0.5058148	7.520192	0.3319865
cg15267318	0.4647351	0.1930679	1.118667	0.0873105
cg07728874	5.3644334	1.7432332	16.50792	0.0034006
cg23983710	2.8509106	0.1279923	63.50141	0.5082052
cg21301224	1.6055909	0.7698057	3.348796	0.2067925
cg09990194	0.5403382	0.2025642	1.441347	0.218821
cg24586554	0.7295385	0.342251	1.555076	0.4141548
cg14989968	2.5223237	0.6938778	9.16893	0.1600279
cg17723710	1.4514366	0.5852144	3.599823	0.4214643
cg08195983	2.2388396	0.6032832	8.30854	0.2283505
cg26987551	1.09E-10	7.02E-27	1691265	0.2278089
cg14792350	0.439818	0.203051	0.952667	0.0372579
cg25951288	0.2698585	0.0979759	0.743281	0.0112805
cg07134541	0.4819494	0.179186	1.29628	0.1482013
cg26446929	0.1251691	0.0001781	87.97916	0.534378
cg16306115	2.7506003	0.8851778	8.547211	0.0802701
cg21819468	1.1143542	0.6047041	2.053542	0.7284718
cg09766360	0.7775604	0.4003629	1.51013	0.4575553
cg07028733	0.2369522	0.07656	0.733364	0.0124913
cg25597117	0.4702862	0.2069879	1.068512	0.071592
cg11319265	0.1859416	0.0044051	7.848633	0.3783169
cg07505457	1.6525259	0.8571158	3.186083	0.1337057
cg08181251	0.6217695	0.2543795	1.519766	0.2973765
cg15143788	2.5475151	0.961025	6.753033	0.0601029
cg13976799	0.0788034	0.0156599	0.396553	0.0020569
cg22192999	9.0749193	0.7188641	114.5615	0.0882286
cg23366376	0.7646499	0.3497849	1.671569	0.5012905
cg21165064	2.5544533	0.9780228	6.67186	0.0555439
cg20620783	0.5321576	0.2712926	1.043861	0.0664926
cg13553997	0.3157064	0.1264218	0.788397	0.013544
cg25812683	0.535776	0.2288792	1.254181	0.1504195
cg03261347	0.636618	0.3064339	1.322577	0.2260805
cg14929208	0.1263672	0.0182737	0.873862	0.0360267
cg16619576	4.5215489	0.9349181	21.86759	0.0606168
cg00214791	0.2951575	0.1210885	0.719457	0.0072691
cg25138715	1.3495635	0.6927028	2.629297	0.3783257
cg24136690	0.8820939	0.3922553	1.983631	0.7615665
cg02482346	0.159969	0.0417835	0.612445	0.0074557
cg23219282	2.2977495	0.7886806	6.694285	0.1272978
cg11381539	0.0047805	0.0001683	0.135809	0.0017529
cg10686758	1.4811072	0.6541702	3.353376	0.3461471
cg18576206	3.5135393	0.9957206	12.39801	0.0507843

cg08101036	0.782877	0.4152445	1.475989	0.4492959
cg23480697	1.9531684	0.6692327	5.700359	0.2205629
cg06354527	1.1533888	0.4982094	2.670174	0.7389884
cg02185052	1.5505087	0.7192976	3.342256	0.26306
cg15020801	0.5490318	0.1798444	1.676093	0.2923506
cg09318453	0.3935066	0.1917672	0.807476	0.0109893
cg18772838	0.1348352	0.0437742	0.415325	0.0004816
cg20286882	2.4123285	0.9782881	5.948482	0.0558384
cg19381766	1.9401549	0.5891575	6.389125	0.2757476
cg14350114	0.0622086	0.0123128	0.3143	0.0007783
cg16758970	0.7848003	0.3227494	1.908328	0.5929812
cg21416692	0.2895234	0.1151365	0.728038	0.0084236
cg16953297	1.0004344	0.5229068	1.914049	0.9989531
cg26726490	0.0705552	0.0085979	0.578985	0.013556
cg23643151	11.142628	1.2555428	98.88804	0.0304443
cg25327343	1.2571273	0.4759572	3.320402	0.6442463
cg11100481	0.6130966	0.2509843	1.497653	0.282996
cg03695591	0.4841517	0.2320801	1.010008	0.0531845
cg23173586	2.2194335	0.6389292	7.709594	0.2095248
cg13419083	1.658E+11	101.42678	2.71E+20	0.0169987
cg25955126	1.258122	0.4072818	3.886426	0.6898756
cg13350140	1.6231003	0.6585641	4.000301	0.2926225
cg13992911	0.1454115	0.0278002	0.76059	0.022363
cg13830932	0.327038	0.1265389	0.845225	0.0210517
cg09897970	2.6607451	1.1458565	6.178404	0.0228028
cg17348388	0.437318	0.1806417	1.058709	0.0667289
cg25705486	5.1896179	1.0009701	26.90603	0.0498652
cg16980637	0.0930565	0.0071739	1.207079	0.069366
cg01153451	0.2663132	0.1026757	0.690745	0.0065123
cg13790288	1.0645283	0.4799633	2.361056	0.8777214
cg22233974	1.0221821	0.3509787	2.97698	0.9679125
cg03547355	0.2405601	0.0589095	0.98234	0.0471685
cg21794222	2.4333002	1.0129211	5.845421	0.0467371
cg02196694	0.2020661	0.0172462	2.367525	0.2028109
cg05032934	0.1450266	0.0251943	0.83482	0.0306082
cg18371750	1.5538455	0.3936512	6.133439	0.5292589
cg05616959	1.1249804	0.6073685	2.083712	0.7080564
cg18224556	2.60E-14	1.81E-23	3.73E-05	0.0036403
cg10809507	0.2097378	0.0677355	0.649437	0.0067591
cg18608055	2.7551613	1.0033744	7.565385	0.0492408
cg01767202	0.4666113	2.17E-06	100358.6	0.9031576
cg09432154	1.4210498	0.5306858	3.805232	0.4844115
cg19797376	2.4050808	0.9084837	6.367108	0.0772715
cg08701621	0.3672954	0.160195	0.842136	0.0179913
cg25597625	0.1978233	0.064383	0.607832	0.0046659
cg14793844	2.6517083	1.0857569	6.47618	0.0323094
cg01614716	90917.155	4.75E-06	1.74E+15	0.3445353
cg13627062	1.1346781	0.5276814	2.439908	0.7463521
cg23959518	0.6423327	0.0219241	18.8191	0.797281
cg08238215	2.863741	0.9121356	8.991001	0.0714802
cg22120446	6.6076309	1.8573758	23.50671	0.0035431
cg05688084	1274.2151	2.2322783	727339.4	0.0272487
cg09384937	1.9381798	0.6150607	6.107593	0.2584728
cg08539991	1.0306208	0.377828	2.811277	0.9530236
cg12632315	2.3770864	0.0793338	71.22488	0.6176755
cg16880424	1.1593286	0.3331669	4.034142	0.8162452
cg00301483	1.2717616	0.471826	3.427911	0.6346461
cg12427941	0.3640939	0.1221074	1.085638	0.0699004

cg04136748	4.2154995	0.4400703	40.3809	0.2120364
cg15404019	0.3308023	0.1416196	0.772705	0.0105981
cg22671299	1.71E+11	6.26E-06	4.68E+27	0.1804226
cg22353755	0.3357634	0.1448087	0.778524	0.0109772
cg02073304	2.3691258	0.5002615	11.21965	0.2770177
cg09756373	2.25E-08	5.31E-14	0.009547	0.0077314
cg04398983	0.4175309	0.12041	1.447821	0.168615
cg06947839	1.7259879	0.7062536	4.21808	0.2312491
cg00806900	0.7390188	0.0745743	7.323556	0.7960618
cg24997231	0.3785166	0.1560234	0.91829	0.0316758
cg03297901	2.4325688	0.9485794	6.238161	0.0642996
cg05625526	0.2008335	0.0763641	0.528181	0.0011388
cg15679892	1.2042791	0.4543501	3.192006	0.7085898
cg13921220	0.3328247	0.0654615	1.692174	0.184849
cg04184179	0.3289983	0.06555	1.651257	0.1768125
cg00088007	0.8233712	0.3618742	1.873414	0.6431219
cg13674558	0.3534595	0.1268454	0.984928	0.0467001
cg22045977	0.4559515	0.2239152	0.92844	0.0304177
cg13856674	0.4083	0.165813	1.005403	0.0513858
cg17198051	0.2911796	0.0975951	0.868749	0.0269499
cg23079252	3.4813672	1.2156111	9.970226	0.0201435
cg10724490	58321893	4.11E-12	8.27E+26	0.4267662
cg00910297	1.35E-16	3.47E-29	0.000523	0.0134777
cg15893285	0.0005039	4.31E-30	5.89E+22	0.8041803
cg13662628	2695.535	0.0303329	2.4E+08	0.1742353
cg17250929	2.578626	0.4964055	13.39492	0.2598137
cg13916298	0.3747293	0.136467	1.028982	0.0568413
cg15571353	0.3921242	0.1660125	0.926204	0.0327794
cg11360546	0.3766455	0.1899458	0.746854	0.0051795
cg14449524	0.6726572	0.3041331	1.487729	0.3275409
cg04966682	2.5183138	1.161148	5.461753	0.019376
cg05224126	0.3561228	0.1202934	1.054284	0.0622511
cg25945732	0.0173262	0.0007352	0.408294	0.0118831
cg16733705	2.0646337	0.6813485	6.256287	0.1999653
cg10996058	0.0700985	0.009084	0.540927	0.0107924
cg04825872	2.0355093	0.5964666	6.946404	0.2564269
cg03514404	0.5765146	0.2493371	1.333011	0.1978024
cg27273675	0.2524155	0.0951201	0.669823	0.0056963
cg17961327	0.3353861	0.142478	0.789482	0.0123796
cg09196248	0.5186187	0.2509348	1.071854	0.0762903
cg03241909	0.3146126	0.131654	0.751827	0.0092757
cg13728106	0.4517166	0.2225695	0.916783	0.0277679
cg24902842	1.4534856	0.3376847	6.256192	0.6155546
cg14871870	1.3590581	0.523729	3.526707	0.5283172
cg10011623	3.3917785	1.1700879	9.831878	0.0244973
cg13757263	0.3967645	0.142645	1.103593	0.0765427
cg02916272	1.1743316	0.5066619	2.721844	0.7078944
cg00572212	7.99E-09	2.58E-24	24767146	0.3056062
cg05304366	0.3466642	0.1291094	0.930808	0.0355315
cg01918001	0.9358065	0.4367334	2.005191	0.8645125
cg16753400	0.5323413	0.2577174	1.099605	0.0884889
cg25581330	1.5549793	0.7731575	3.127384	0.2156012
cg06719334	0.3041855	0.1169822	0.790965	0.0146497
cg05264578	4.5699578	0.6052237	34.5071	0.1407155
cg21095344	4.0185864	1.0269646	15.72502	0.0456963
cg15152824	35.116369	0.0007799	1581252	0.5150853
cg18098806	6.8825789	0.7069285	67.00804	0.0966586
cg03803177	0.2480604	0.0677676	0.908014	0.035229

cg26058820	0.600577	0.2838408	1.270757	0.1824167
cg17918544	0.0644096	0.0091633	0.452739	0.0058436
cg25953930	0.2580196	0.1034743	0.643388	0.0036614
cg25432336	0.5354518	0.2436275	1.176832	0.1200175
cg02593403	0.4629478	0.2394425	0.895082	0.0220522
cg18450254	0.4151712	0.1516803	1.136384	0.0870624
cg04040975	0.4280901	0.1626022	1.127052	0.0858332
cg00877212	0.6133193	0.3036432	1.238824	0.1729122
cg05999049	1.0925641	0.5063878	2.357277	0.8214838
cg00513205	1.9320326	0.9439467	3.954407	0.0715275
cg10946573	0.8357933	0.3975465	1.757154	0.6361224
cg03814316	0.1896452	9.37E-05	384.0068	0.6686357
cg02235497	0.5956695	0.3033126	1.169823	0.1324615
cg09926380	0.0728444	0.0082103	0.6463	0.0186792
cg18036068	1.1621243	0.3821451	3.534084	0.7911829
cg01923089	1.9605526	0.3875147	9.91902	0.4157086
cg03128101	40877.437	24.225297	68976031	0.0050998
cg25413843	1.3484148	0.5142316	3.535805	0.543344
cg01363170	1.7546234	0.8650471	3.559001	0.1191861
cg25846153	0.0529048	0.0027008	1.036327	0.0528119
cg01061843	9.256E+15	0.0003146	2.72E+35	0.1079706
cg05183646	0.4171825	0.1675663	1.038641	0.060313
cg13252307	0.2613925	0.0616287	1.108672	0.068755
cg21000999	0.6045315	0.2620044	1.394856	0.238065
cg25270844	0.197246	0.0769446	0.505636	0.0007254
cg26132462	2.72749	0.981567	7.578904	0.0543191
cg18887230	0.0943703	0.0212715	0.418671	0.0019005
cg16837898	1.4858685	0.4447153	4.964536	0.5199656
cg02511809	1.4447155	0.6279752	3.323703	0.3867716
cg17533563	0.3626477	0.1259373	1.044276	0.0601519
cg02573468	1.6512078	0.6029493	4.521918	0.3292195
cg09174690	3.7737455	0.9638579	14.77516	0.0565073
cg20459687	0.5513916	0.215481	1.410949	0.2143006
cg15519474	0.9312082	0.3806258	2.278219	0.8759242
cg01564268	0.4136664	0.1340709	1.276339	0.1246573
cg03401875	1.367732	0.7118316	2.627996	0.3473066
cg08500200	0.6749495	0.3331933	1.367245	0.2750594
cg16237204	79.721859	0.1723708	36871.52	0.1619785
cg05075579	0.7058612	0.2615062	1.905271	0.4917252
cg20467114	15.227307	0.0032418	71524.89	0.5278676
cg03229061	0.3272956	0.1299032	0.824632	0.0178396
cg01559901	1.1280427	0.3586789	3.547686	0.8367179
cg21971285	3.2049208	1.0093956	10.17591	0.0481742
cg19736660	0.6801079	0.1166824	3.964153	0.6681982
cg07749074	2.2891535	0.9397037	5.576464	0.0682936
cg02962944	3.3371457	1.3010181	8.559866	0.012159
cg09317772	0.2037324	0.0820442	0.505909	0.0006074
cg04955116	0.2054301	0.0286721	1.47187	0.115201
cg26526719	0.1863984	0.0503741	0.689727	0.011856
cg08865350	0.114937	0.0180396	0.732308	0.0220379
cg20862669	1.18E-08	7.20E-14	0.001924	0.0028736
cg02190400	35130.03	3.32E-10	3.72E+18	0.5252493
cg11313335	2.0317764	0.0560331	73.67276	0.6987914
cg21579556	0.2731619	0.1050985	0.709976	0.0077491
cg24941241	0.6296522	0.2796444	1.417736	0.2639716
cg25860912	83.05739	0.4349116	15861.91	0.0990955
cg24378559	1.1188966	0.3928738	3.186595	0.8333693
cg03589296	1.3431529	0.505427	3.569377	0.5541081

cg27305735	2.386022	1.262012	4.511131	0.0074493
cg15866542	1.4454437	0.4987693	4.188926	0.4973715
cg08109624	3.0671552	0.7790939	12.07485	0.1089474
cg19851563	0.2878396	0.1093836	0.757441	0.0116449
cg18248284	1.3690691	0.4946814	3.789005	0.5453016
cg10356260	0.6039206	0.2788804	1.307801	0.2008048
cg24730688	0.2920962	0.1007229	0.847078	0.0234836
cg12572827	4.4635424	0.003789	5258.238	0.6784239
cg00424967	0.401243	0.1807921	0.890503	0.0247635
cg01885839	0.6456294	0.3202546	1.301581	0.2212834
cg25412641	0.5061918	0.2577722	0.994018	0.0479975
cg23417171	2.2792943	0.3159523	16.44293	0.4138327
cg10597661	0.3937676	0.151631	1.022568	0.0556035
cg24158594	1.2774303	0.7162957	2.278149	0.4068002
cg00819310	11.954027	0.0588099	2429.842	0.3601892
cg27310090	2.6720664	1.1882871	6.008597	0.0174437
cg19207486	0.3798865	0.1486182	0.971037	0.0432442
cg16843718	0.0567539	0.0102543	0.314114	0.0010146
cg02713162	0.5956066	0.2813649	1.260808	0.1756505
cg21359468	2.3658434	1.1689576	4.78821	0.0166677
cg14855841	0.913311	0.3518499	2.370718	0.8521922
cg16926310	0.2931749	0.1155485	0.743857	0.0097985
cg08748969	0.8791145	0.2932541	2.635401	0.8180842
cg00112588	0.7976609	0.3133332	2.030628	0.6353634
cg03934150	6.77E-12	1.16E-21	0.039337	0.0249597
cg11789534	0.537466	0.2386365	1.210501	0.1339224
cg15544004	0.9346911	0.4792295	1.823025	0.8429244
cg02619408	1.6085426	0.7619427	3.395806	0.2124697
cg05433111	1.9002948	0.5756606	6.273003	0.2920443
cg04407215	0.0569774	0.008183	0.396727	0.0038073
cg14922279	158968.91	0.4296753	5.88E+10	0.0671257
cg05885410	0.0045888	0.0001766	0.119236	0.0011973
cg13835894	2.1350616	0.5348659	8.522674	0.282837
cg23340017	2.0989903	1.0463725	4.210509	0.0368348
cg18857768	0.921575	0.2302039	3.68934	0.9081287
cg05027713	0.4879374	0.2145391	1.109742	0.0869717
cg13294753	0.9892779	0.0185212	52.84045	0.9957623
cg17345994	0.3013476	0.1164096	0.780094	0.0134471
cg07312641	0.3267386	0.1213093	0.880049	0.0269166
cg00675229	1.8558695	0.9662278	3.564637	0.0633396
cg01953615	1.8236418	0.751772	4.423774	0.1838806
cg09703679	1.2221497	0.5136321	2.908015	0.6501303
cg13977235	3.9057656	0.35946	42.43867	0.2629851
cg17259096	0.6518976	0.1911926	2.222735	0.4941776
cg14718247	0.0723338	0.0046596	1.122888	0.0605006
cg10220206	4.1237122	0.3995521	42.56016	0.2341929
cg14820497	9.5662655	1.1812973	77.46859	0.0343372
cg00709979	1.4138065	0.5978251	3.343534	0.4303963
cg00002449	115.66652	0.715151	18707.58	0.0671356
cg16974893	0.1927546	0.0274383	1.354106	0.0978855
cg23033014	7.3149246	0.4275421	125.1529	0.1696027
cg04560832	0.5461815	0.2712096	1.09994	0.0904029
cg03307776	1.7324201	0.5203225	5.768114	0.3705609
cg14109579	2.2880691	0.8177569	6.401976	0.1148614
cg16348820	0.397766	0.1771406	0.893177	0.0255038
cg13525231	5.1679857	0.8313639	32.12562	0.078094
cg05501868	0.4596063	0.1473269	1.433804	0.1805007
cg00155846	2.3523034	0.8672604	6.380242	0.0929142

cg19484848	0.0044858	7.64E-06	2.632187	0.096433
cg09797337	1.1972478	0.3886319	3.688329	0.753827
cg14149172	0.1185928	0.0185535	0.758038	0.0242809
cg05754148	3.58E-05	6.13E-11	20.93769	0.1307864
cg08090557	0.5494088	0.2339985	1.289966	0.1690421
cg22512531	2.9565559	0.7219376	12.108	0.1318073
cg18472160	85.797752	1.2909379	5702.253	0.0375962
cg25456368	4.6451361	1.5619086	13.81469	0.0057478
cg00628697	1.6743084	0.8113843	3.45497	0.1631788
cg24670219	1.4255967	0.7016266	2.896592	0.3269336
cg25450450	0.3439522	0.0653595	1.810037	0.2077953
cg07959469	1.9077609	0.5989341	6.076715	0.274499
cg20370678	1.6783326	0.8131789	3.463937	0.1613382
cg19540471	0.4597855	0.2313485	0.913785	0.0266062
cg09176256	1.5637677	0.651777	3.75185	0.3166776
cg27105619	0.8078236	0.3211612	2.031936	0.6502119
cg12398330	1.3294794	0.6531652	2.706077	0.4322361
cg20303331	0.2271704	0.0631576	0.817104	0.0232539
cg23071962	0.0019681	8.25E-12	469667.1	0.5266971
cg06264984	3.2906875	0.911058	11.88577	0.0690941
cg04020211	9.315706	0.4400622	197.2048	0.15188
cg13803727	3.711732	1.0487125	13.13702	0.0419799
cg27528660	0.31732	0.1337423	0.752881	0.0092178
cg17092519	1.1475332	0.5036862	2.614391	0.7432431
cg07728307	2.0464155	0.9413125	4.448912	0.0707124
cg06175025	12830459	0.1243065	1.32E+15	0.0821228
cg21157725	1.3474817	0.3269281	5.553841	0.679802
cg14611402	3.308671	0.831349	13.16812	0.0895321
cg11726792	2.796331	0.9575125	8.166438	0.0600312
cg17468569	1.2418794	0.0979088	15.75206	0.8672643
cg16992960	0.4409852	0.1985788	0.979299	0.0442882
cg13230208	0.5878465	0.1729038	1.998589	0.3948087
cg27188860	1.0214237	0.5066099	2.059388	0.9527536
cg08301525	0.4485321	0.2307068	0.87202	0.0180945
cg17884026	0.4442331	0.1676814	1.176893	0.1026157
cg00584683	0.4542185	0.1754338	1.176024	0.1039679
cg03095814	0.0276276	0.0023279	0.327879	0.0044629
cg09403392	27.523658	0.6517836	1162.275	0.0825937
cg03020424	2.3997591	0.6469342	8.901746	0.190599
cg04167844	1.496759	0.6930372	3.232565	0.3046073
cg00952789	0.3900873	0.2004292	0.759211	0.0055924
cg24301724	0.0534161	0.0067886	0.420302	0.0053774
cg14689532	0.2311189	0.0493435	1.082533	0.0629841
cg26391277	1.5699365	0.798859	3.085276	0.1907127
cg20728514	1.2828396	0.2886959	5.700385	0.7434278
cg14508696	0.0841746	0.0141392	0.501115	0.0065468
cg18256640	0.2604531	0.1122628	0.604259	0.0017294
cg07092145	0.4930071	0.2499784	0.972308	0.0412501
cg04012082	0.1041617	0.0252219	0.430168	0.0017734
cg19849211	0.5019732	0.2212431	1.138915	0.0991917
cg23549534	0.2670919	0.0747225	0.954707	0.0422263
cg10230442	13.555849	1.9074484	96.33866	0.0091774
cg04470984	0.3486979	0.1402689	0.866836	0.0233575
cg21618333	1.8278858	0.7830243	4.267002	0.163173
cg02689863	1.5088463	0.6924164	3.287931	0.3006408
cg01023808	0.0010584	3.48E-06	0.322186	0.0188658
cg04036593	3.8205887	1.0089913	14.46682	0.0484796
cg11274962	0.2978139	0.0626381	1.415962	0.1278269

cg13508841	1.1239585	0.5224044	2.418208	0.7649896
cg10177032	8.74E-05	7.11E-10	10.73676	0.1180583
cg16029659	0.3458647	0.1460852	0.818854	0.0157592
cg02890435	6.1518969	0.8680618	43.59809	0.069011
cg24866203	0.2150283	0.1043899	0.442928	3.06E-05
cg01416891	2.2258435	0.9205696	5.381863	0.0756938
cg11617816	0.4115444	0.1729255	0.979432	0.0447559
cg26328438	0.0799904	0.0099696	0.6418	0.0174364
cg26520739	3.0448574	0.9292189	9.977365	0.0659542
cg05211868	1.4562984	0.6421508	3.302659	0.3682507
cg07568117	0.3218397	0.0339907	3.047325	0.3229296
cg19826697	2.73E-17	2.51E-31	0.002965	0.0207256
cg11484553	4.384E+16	0.0040409	4.76E+35	0.0866174
cg07561747	0.9910452	0.4439041	2.212574	0.9824869
cg13587621	0.2107311	0.0591255	0.751073	0.0163319
cg15754109	2.797E+09	0.0136755	5.72E+20	0.1016402
cg16363586	1.6459195	0.908911	2.980546	0.100027
cg20382344	5.9184198	0.6412815	54.6214	0.1168499
cg04756223	0.2833316	0.1208186	0.66444	0.0037311
cg08446989	0.0011248	4.82E-17	2.63E+10	0.6654847
cg13492223	1.9212481	0.6657108	5.544741	0.2272365
cg17239057	0.4434054	0.1797138	1.094008	0.077568
cg11562401	1.3315991	0.5998187	2.956154	0.4815502
cg22887911	0.2030209	0.0481526	0.855977	0.0298718
cg23460707	0.6887621	0.1887829	2.512903	0.5723299
cg09714458	1.7710878	0.7067963	4.437986	0.2226286
cg26203383	1.5085607	0.7789025	2.921746	0.2228091
cg23280754	0.4528619	0.2192408	0.935428	0.0323292
cg10155853	1.1736124	0.3261375	4.223267	0.8064348
cg12878183	0.0244537	0.0032871	0.18192	0.0002896
cg03686964	1.8752476	0.8175737	4.301207	0.1376941
cg10915509	1.02129	0.2569112	4.059898	0.9761324
cg02473600	1.8247538	0.6768021	4.919793	0.2346242
cg25875953	1.4539153	0.6119211	3.454481	0.3966523
cg19058685	8.4754837	1.4315681	50.17842	0.0185047
cg17379558	1.826E+10	11541.729	2.89E+16	0.0011775
cg18222590	0.1655239	0.049745	0.550773	0.0033643
cg06489615	0.4912964	0.2237186	1.07891	0.0766054
cg09182189	0.0151301	0.0002568	0.891517	0.043886
cg09740450	0.1713816	0.0405548	0.724245	0.016453
cg04365123	2.011771	0.8644016	4.682109	0.1048318
cg08901157	0.3283201	0.1445505	0.745719	0.0077919
cg05011258	0.3282766	0.1411595	0.763431	0.0096862
cg20103124	1.9889679	0.5332997	7.417955	0.3058985
cg24935810	1.144416	0.1253075	10.4518	0.9048546
cg09955084	0.1676996	0.0623412	0.451117	0.0004053
cg24537836	0.4107411	0.1608689	1.048731	0.0628182
cg17284168	0.4238971	0.2032655	0.88401	0.0220949
cg11099262	6.0981079	0.9214913	40.35515	0.0607702
cg26029221	9.1025192	0.9494597	87.26632	0.0554931
cg03335835	1.7695743	0.157945	19.82585	0.6433925
cg23610041	1.1974845	0.5743993	2.496467	0.6306496
cg08949656	1.3876237	0.5413247	3.557014	0.4951827
cg18702012	0.2080829	0.0647747	0.668448	0.008378
cg01627483	0.1926711	0.0205019	1.810665	0.149698
cg06895640	0.3928074	0.1969684	0.783362	0.0079726
cg04395431	0.4606822	0.1625077	1.305957	0.1448795
cg04899225	0.3166466	0.1261026	0.795107	0.0143632

cg09559196	0.571611	0.2942446	1.110434	0.098782
cg08252585	0.5231948	0.266894	1.025624	0.0592556
cg03844894	6.3704294	2.0790231	19.51992	0.001191
cg17588293	1.2576035	0.3980193	3.973593	0.6961774
cg15487251	0.4294735	0.1906082	0.967679	0.0414266
cg13546414	2.2403147	0.738504	6.796185	0.1542739
cg13731761	2.6752628	1.1824264	6.052834	0.0181662
cg19355325	3.1482706	0.9898273	10.01347	0.0520592
cg05786809	0.0885297	0.009778	0.801548	0.031025
cg13343818	2.1662127	0.7666869	6.120461	0.1446677
cg07895124	0.4527829	0.196753	1.041978	0.0624252
cg15176829	1.5059767	0.7410872	3.060323	0.2577448
cg09672187	0.7732085	0.2906605	2.056872	0.6063793
cg12792011	0.1543331	0.015158	1.571362	0.1145074
cg21620953	2.8485516	0.8748235	9.275295	0.0822218
cg18481342	0.306759	0.1260307	0.746652	0.0092227
cg23466418	1.3061436	0.4836105	3.527655	0.59829
cg26797158	2.7568487	0.9187152	8.272656	0.0704898
cg00931520	0.1567367	0.0400446	0.613476	0.0077731
cg11670000	0.6997217	0.187003	2.618195	0.5958577
cg14022022	0.2014446	0.0511339	0.793601	0.0219963
cg20452961	2.5831235	0.9144044	7.297129	0.0732803
cg14549951	1.1638861	0.4128943	3.280817	0.7740926
cg20332645	0.2212257	0.0574314	0.852161	0.0283457
cg16664233	101129400	2.10E-18	4.86E+33	0.5412645
cg04652943	12.496162	1.8567794	84.09941	0.0094279
cg20689661	0.3864359	0.2017469	0.740198	0.0041418
cg20218614	3.643173	0.923908	14.36583	0.0647609
cg03582793	0.0014651	0.0001013	0.021192	1.69E-06
cg11611341	10.541209	1.6902077	65.74167	0.0116705
cg10226036	2.0864472	0.7702847	5.651497	0.1480077
cg19998533	2.4979671	1.224437	5.096089	0.0118504
cg27278787	2.1042538	0.8071557	5.485787	0.1280727
cg00806239	0.424493	0.1624193	1.109439	0.0804486
cg05526487	2.9037615	0.6676704	12.62873	0.1552157
cg27576755	2.08E-17	4.81E-45	8.98E+10	0.2367525
cg17765291	0.0286901	2.00E-05	41.16989	0.3382977
cg08729719	7589.8542	0.1142536	5.04E+08	0.1147836
cg21828319	0.4528011	0.1958599	1.046814	0.0638873
cg08820231	2.7251867	0.8652146	8.583585	0.0867789
cg18029761	0.0208619	0.0009314	0.467267	0.0147026
cg14178347	0.1498115	0.0372843	0.601956	0.0074674
cg06640997	1.5049852	0.5764623	3.929105	0.4037703
cg08430972	2.7314457	0.1249421	59.71402	0.5231844
cg20056212	1.6504173	0.2986114	9.121812	0.5657052
cg07001918	0.5746281	0.2598525	1.270711	0.1712227
cg01380607	0.1340084	0.0305255	0.588302	0.0077485
cg17139392	0.1197702	0.027372	0.524072	0.0048337
cg09731211	4.8288571	0.9681742	24.08437	0.0547928
cg15161050	0.4387938	0.2224057	0.865715	0.0175072
cg27590105	1.0435513	0.4298666	2.533342	0.9249454
cg26433975	4.0757167	1.0014833	16.58686	0.0497585
cg00909156	1.1229371	0.4746814	2.656493	0.7918393
cg05782444	0.9197264	0.4053758	2.086698	0.8413321
cg14800351	6.5130591	1.2731057	33.32005	0.0244563
cg04973493	1.7853381	0.8846598	3.603003	0.1056885
cg17804401	2.1119172	0.6877213	6.485468	0.1915612
cg18091275	1.2394662	0.4201137	3.656811	0.6973422

cg13421247	0.1994312	0.0451353	0.88119	0.0334359
cg01456938	17935.688	4.61E-07	6.98E+14	0.431124
cg10832186	0.4333115	0.1758633	1.067641	0.0691094
cg24129356	3.1101996	1.5396093	6.282985	0.0015626
cg18606843	1.7102502	0.7527109	3.885895	0.1999968
cg26228577	0.0869488	0.0110321	0.68528	0.0204084
cg08146733	1.2627242	0.5367286	2.970724	0.5930597
cg12126869	0.0523487	0.0110059	0.248991	0.0002094
cg01989157	79988819	0.4119435	1.55E+16	0.0616392
cg00310940	1.9678359	0.556678	6.956227	0.2933799
cg18374914	0.3322448	0.1267787	0.870703	0.0249852
cg05361406	0.0674097	0.0154994	0.293178	0.0003233
cg17482033	1.6969696	0.8783936	3.278377	0.1154772
cg16581347	1.7019866	0.7250337	3.995343	0.2219168
cg22175814	1.8122974	0.9043378	3.631853	0.0936486
cg27308504	1.2475998	0.4187025	3.717449	0.6912761
cg14135887	0.9577775	0.2491659	3.681635	0.9499302
cg19965868	3.876E+19	4.58E-12	3.28E+50	0.214475
cg06588876	0.2629548	0.0788497	0.876924	0.029729
cg10495572	0.9485831	0.3998427	2.25041	0.9046751
cg12486169	2.4968281	1.2913691	4.827551	0.0065263
cg06548292	0.3612317	0.1711659	0.76235	0.0075393
cg26335760	0.4296665	0.167697	1.100874	0.0784485
cg15048534	0.0505254	0.0025947	0.983864	0.0487579
cg01013737	11236.46	6.38E-06	1.98E+13	0.3905385
cg05969808	1.6301788	0.7673019	3.463412	0.2037128
cg05434863	0.0001338	1.85E-25	9.66E+16	0.7158781
cg22796704	0.6069296	0.2384813	1.544623	0.2947702
cg12130797	0.0226714	0.0027831	0.184685	0.0004027
cg10199770	1.2776922	0.6470485	2.522991	0.4802384
cg19883905	2.0439254	0.8392612	4.977748	0.1154621
cg12315391	2.6554461	0.7693648	9.165214	0.122312
cg06620117	0.4356202	0.1541112	1.231351	0.1170166
cg14703454	0.6234371	0.3170954	1.225731	0.1707258
cg13053653	1.4683991	0.6150748	3.505583	0.3868777
cg08551532	0.3629383	0.1162045	1.133555	0.0811194
cg13843611	0.5031231	0.2275678	1.11234	0.0897057
cg22092811	4.3672964	1.0344765	18.43761	0.0448466
cg09225701	2.7319305	0.2504152	29.80428	0.4097698
cg22699004	1.804622	0.6860097	4.747252	0.2315843
cg23655970	1.8581566	1.0838386	3.185664	0.0242795
cg16667275	7.63E+28	3.506E+13	1.66E+44	0.0002235
cg00580805	4.77E+28	73634.925	3.09E+52	0.0182463
cg24782378	0.3698016	0.1386135	0.986579	0.0469278
cg18030409	2.1357583	1.0800302	4.223459	0.0291632
cg11119340	0.5458815	0.2734501	1.08973	0.0861014
cg03074016	0.2153221	0.0320387	1.447114	0.11416
cg08768218	0.9612748	0.4702841	1.964875	0.9137772
cg03040848	1.5600002	0.6508222	3.739271	0.3187721
cg26827033	6.3785318	1.3699585	29.69847	0.0182225
cg06613765	0.6763551	0.3278527	1.39531	0.2898894
cg08183663	0.5752359	0.2666496	1.24094	0.1586394
cg19734013	2.5463077	1.4142472	4.584547	0.0018384
cg27054655	2.4644915	0.7693971	7.894128	0.128862
cg05949020	0.0574483	0.0054775	0.602517	0.0171968
cg20674490	0.347325	0.1248962	0.965879	0.0427145
cg13508904	0.7945884	0.3423223	1.844375	0.5925282
cg14044057	0.2539087	0.0510703	1.262369	0.0938897

cg12499316	0.9817438	0.1256435	7.67108	0.9859857
cg19466160	0.60655	0.3067822	1.199232	0.1505557
cg01526748	0.4567847	0.2291221	0.91066	0.0260266
cg09546679	3.6847432	0.8030537	16.90713	0.0933861
cg17352109	1.2784469	0.5575453	2.931468	0.5618009
cg20161190	3.3352706	1.3551642	8.208621	0.0087579
cg09573795	4.3468854	1.3356941	14.14651	0.0146574
cg08894532	1.5181426	0.5996732	3.843355	0.3783547
cg16211174	1.5100848	0.6836822	3.335403	0.3079957
cg13083637	216610316	0.0015326	3.06E+19	0.1428598
cg27205902	0.0333513	8.75E-07	1271.136	0.527472
cg03339956	0.234637	0.0735945	0.748079	0.014262
cg03182782	5.1522407	1.3267518	20.00795	0.0178644
cg26470798	2.4294074	0.9111433	6.477598	0.0760647
cg14081950	19.435093	0.5289094	714.1541	0.1066186
cg26344606	2.9856053	0.6989768	12.7527	0.1398053
cg10369896	1.8684567	0.7139021	4.890209	0.2028643
cg06826278	0.7550904	0.287387	1.98395	0.5687028
cg26320946	2.4361193	1.077452	5.508067	0.0324205
cg05812430	0.3199009	0.0928877	1.101724	0.0708525
cg07782112	0.253911	0.1257218	0.512805	0.0001323
cg13393580	1.0070042	0.2325798	4.360042	0.992552
cg17172308	1.9829607	1.1024851	3.566609	0.0222706
cg23346622	1.9870928	0.912814	4.325676	0.0836087
cg19640754	0.0062222	0.0002622	0.147669	0.0016678
cg00661673	0.9610291	0.2031688	4.54586	0.960014
cg02841651	0.9147615	0.3573075	2.341929	0.8526412
cg20389822	0.0273592	0.0035302	0.212036	0.0005721
cg01834010	57134806	2.4903965	1.31E+15	0.0388775
cg27016929	0.0267205	0.0016165	0.441674	0.0113759
cg09329930	0.3587624	0.1335217	0.963967	0.0420793
cg10168894	0.012227	0.0006199	0.241164	0.0037936
cg22998749	0.8123365	0.4031061	1.637015	0.5610049
cg02128191	0.2623349	0.0926933	0.742444	0.0117011
cg04804726	0.2960072	0.1214885	0.721222	0.0073797
cg05289022	20.334704	1.105829	373.9278	0.0425932
cg17525495	0.5212855	0.281716	0.964583	0.0380043
cg14647640	0.1502662	0.0334041	0.675964	0.0134964
cg14181271	1.2217747	0.4798425	3.110882	0.674441
cg07273342	1.7063423	0.549391	5.299694	0.35542
cg04013024	1.9372927	0.6645874	5.647268	0.2257227
cg16721582	0.0146583	0.0002924	0.734716	0.0344885
cg25396728	1.7408451	0.8143741	3.721314	0.152654
cg09871133	23.930113	1.3486261	424.6176	0.0304809
cg07471156	8.7101999	0.4297163	176.5527	0.1585921
cg01314743	0.3103038	0.1130676	0.851601	0.0230971
cg05740808	0.1309753	0.0538338	0.318657	7.43E-06
cg03830049	2.2241717	1.0706997	4.620287	0.0321046
cg13905899	0.3718787	0.0890431	1.55311	0.1750007
cg19309549	1.7035711	0.0615809	47.12748	0.753154
cg22416916	0.3814306	0.1809977	0.803819	0.0112722
cg13420985	1.2696791	0.5564879	2.896892	0.5704954
cg13016916	0.4492879	0.2055902	0.981854	0.0448697
cg02550110	1.627E+09	0.0066453	3.98E+20	0.1129136
cg20443278	0.116004	0.0223887	0.60106	0.010274
cg11697194	1.5642686	0.5889391	4.15482	0.3693428
cg20980587	1.7621943	0.5672158	5.474687	0.3272895
cg06605803	3.18E-06	2.62E-10	0.038549	0.0083251

cg09704054	1.1844979	0.4116656	3.408192	0.7535181
cg06174078	0.546017	0.2425841	1.228995	0.1437881
cg03242698	3.5314683	1.3964133	8.930929	0.0076912
cg17102984	3.1539796	0.9024368	11.02303	0.0719916
cg05677402	0.6575385	0.1478519	2.924257	0.5818792
cg11925907	1.8423052	0.9159606	3.705496	0.0865734
cg24327307	0.0508854	0.0039289	0.659042	0.0226642
cg18082362	0.4230982	0.1445994	1.237986	0.1163599
cg16914151	0.0680839	0.0094462	0.490718	0.0076672
cg13945265	1.9224113	0.9366598	3.945579	0.0748154
cg24130774	0.282178	0.1068384	0.745279	0.0106719
cg25653802	8.7800041	1.5602981	49.40625	0.0137137
cg26642224	0.2077252	0.0543573	0.793818	0.0215884
cg21759048	0.387676	0.1510673	0.994872	0.0487633
cg02719154	1.7580651	0.8469597	3.64928	0.1299771
cg27040700	4.1135914	1.0352997	16.34467	0.0445108
cg07084746	2.816425	0.6380902	12.43124	0.17166
cg16604035	0.1909294	0.0745331	0.489098	0.0005603
cg02034447	0.0003312	1.96E-43	5.61E+35	0.8619741
cg05576845	0.2309777	0.0842273	0.633413	0.0044114
cg08367223	0.548256	0.2729138	1.10139	0.0912908
cg05072547	10.071113	1.804445	56.20971	0.0084686
cg03852570	0.0866651	0.0209757	0.358074	0.000728
cg06961098	0.3502485	0.1678335	0.730927	0.0051895
cg24741609	0.1569273	0.0160522	1.534135	0.1113713
cg22736483	1.6363937	0.6295726	4.253337	0.312239
cg12144728	2.0926137	0.9966685	4.39367	0.0510396
cg00491021	0.408972	0.1648229	1.014775	0.0538139
cg20239174	2.7854131	0.7964617	9.741242	0.1087817
cg20100910	2.9965462	1.472079	6.099733	0.0024764
cg07880943	5.2479462	0.9451489	29.13926	0.0580308
cg20022223	1.3644315	0.5217438	3.568175	0.5263794
cg18368019	0.0009354	1.76E-06	0.496775	0.0293696
cg25587091	0.9812112	0.500672	1.922966	0.9559375
cg22829821	1.3299578	0.71529	2.472826	0.3675325
cg01647936	1.2857757	0.4970561	3.326022	0.6042042
cg03645522	3.4192944	0.6129401	19.07458	0.1609643
cg01004382	2.0685978	0.71368	5.99582	0.180666
cg26244661	1.3450793	0.6267869	2.886529	0.4467069
cg05260466	2.68185	1.0389978	6.922362	0.0414463
cg04413153	1.2450545	0.6858658	2.260152	0.4712342
cg15054274	0.9414344	0.4593239	1.929572	0.8690834
cg06955484	3.6658194	1.0514405	12.78078	0.0414815
cg18614025	70.738444	4.21E-13	1.19E+16	0.7988474
cg07291005	2.42E-05	1.28E-11	45.83182	0.1495053
cg09646197	0.7289244	0.338005	1.571961	0.4200229
cg10437265	0.3030718	0.1169422	0.785452	0.0140102
cg04008252	1.8487098	0.9561034	3.574642	0.06777
cg03706154	1.6764649	0.6842797	4.107289	0.2584185
cg09321959	0.8686989	0.0823145	9.16774	0.9068004
cg20644626	0.5406938	0.262036	1.115686	0.0961587
cg02455723	7.97E-19	9.62E-32	6.60E-06	0.0060332
cg14856738	3.1235737	1.1661349	8.36671	0.0234698
cg16011371	0.8051142	0.4034355	1.606723	0.5386326
cg10855825	1.82632	0.5352651	6.231388	0.3361178
cg25026237	1.3459044	0.6614135	2.738769	0.4124759
cg02813863	0.2319444	0.1174962	0.457872	2.54E-05
cg20783697	2.4580279	0.7323127	8.250439	0.1454768

cg06165761	3.5427824	0.1252758	100.1894	0.4582129
cg03797115	0.2969229	0.1209341	0.729019	0.0080585
cg17650057	0.2978544	0.1217141	0.728899	0.0079895
cg18448949	1.7304242	0.8849668	3.383594	0.1089833
cg23193870	1.995697	0.7004179	5.686329	0.1958596
cg13393476	0.1630068	0.0381363	0.696744	0.0143851
cg06294856	21.321643	1.2588864	361.1227	0.0340533
cg05081629	39.681969	3.14E-07	5.02E+09	0.6989693
cg18214661	0.2871156	0.109526	0.752656	0.0111537
cg05330360	1.1831462	0.5703263	2.454446	0.6514803
cg27470087	1.3667131	0.4295353	4.348665	0.5967975
cg02246190	1.2096854	0.6792816	2.154245	0.5179363
cg16261823	4.0465489	1.125077	14.55417	0.0323209
cg10227358	1.6052369	0.8043709	3.203479	0.1794467
cg16339286	3.5258706	1.8106318	6.865981	0.0002106
cg19516105	2.0012822	0.7990976	5.012066	0.1385623
cg15416250	0.938028	0.0785761	11.19802	0.9596712
cg25356468	0.4382211	0.2045797	0.938694	0.0337755
cg01206723	3.0392024	1.3458631	6.863069	0.0074801
cg09297288	1.7458787	0.8179525	3.726491	0.149724
cg21584234	2.7214489	1.2861537	5.758475	0.0088435
cg24038762	2.2043047	0.2223133	21.85636	0.4994887
cg10725623	0.3765577	0.1799288	0.788066	0.0095402
cg18278638	2.882986	1.1182175	7.432908	0.0284376
cg21074190	0.5168609	0.2591514	1.030846	0.0609698
cg13384150	0.2006551	0.033204	1.212578	0.0801254
cg04270867	0.2256219	0.0644097	0.790335	0.0199202
cg23706615	0.8400142	0.4595546	1.535452	0.5710511
cg14377059	3.0871869	0.8147309	11.698	0.0972157
cg10331100	1.5589011	0.6492666	3.74295	0.3204734
cg25771797	0.9506525	0.3835807	2.356063	0.9129761
cg22282038	3.3049308	1.1680898	9.350794	0.0242744
cg05674475	0.0089452	0.0002806	0.28519	0.0075799
cg26673396	0.4356566	0.204275	0.929123	0.0315392
cg08614290	2.0678121	1.0962748	3.900342	0.0248411
cg21097698	4.948E+11	1.15E-29	2.13E+52	0.5727018
cg08419921	3.1848284	0.8529081	11.89241	0.0848386
cg00450617	0.4644762	0.2300388	0.937834	0.0324364
cg18831899	0.5984153	0.2486654	1.440091	0.2517985
cg16604086	0.0366285	0.0022712	0.590722	0.019752
cg08079387	0.2535933	0.0834046	0.771056	0.0155972
cg18801906	1.4537722	0.7659786	2.759155	0.2524225
cg01252526	0.2208008	0.0837988	0.581786	0.0022452
cg18063120	0.7384014	0.343083	1.589227	0.4380741
cg14806867	0.0020896	6.79E-07	6.429142	0.1321024
cg19978669	1.25E-07	6.88E-17	226.9411	0.1439416
cg20090162	0.4243498	0.1881542	0.957049	0.0388511
cg10120897	0.9569333	0.4744832	1.929934	0.9021124
cg16767968	5.48E-08	4.12E-28	7.3E+12	0.4794481
cg19353947	0.4862732	0.2194056	1.077737	0.0757998
cg03244494	0.2044862	0.0784137	0.533257	0.0011718
cg23861078	8.599E+12	8.71E-06	8.49E+30	0.1588856
cg06706029	0.4233251	0.1954249	0.916998	0.0292813
cg13569051	0.2775626	0.1019414	0.755738	0.0121427
cg06398474	0.1443594	0.0378246	0.550955	0.0046217
cg05635036	2.2405224	0.71248	7.045728	0.1675766
cg15194222	8.03E-17	1.87E-28	3.45E-05	0.0066922
cg07156733	4.6087771	0.3240239	65.55326	0.2593161

cg12135457	4.2944172	1.6290476	11.32074	0.0032119
cg26217318	1.1282336	0.5488587	2.319196	0.7427755
cg10311048	0.4122624	0.2280512	0.745273	0.0033549
cg05025332	1.0852293	0.2729968	4.314054	0.9075274
cg18541254	1.3518422	0.4207629	4.343247	0.6126841
cg15253444	1.2144583	0.5459403	2.701594	0.6338654
cg03693105	1.373877	0.6312963	2.98994	0.4233658
cg23515330	0.3222843	0.1432623	0.725014	0.006194
cg09863248	7.8208931	1.74433	35.06582	0.0072154
cg07217293	0.7200113	0.3479783	1.489795	0.3759206
cg03298716	1.1414083	0.3836628	3.395724	0.812059
cg22962982	1.5399846	0.7076298	3.351403	0.2764689
cg17199018	3.2250353	0.8353636	12.45069	0.089327
cg20702295	5.07E-26	9.63E-45	2.66E-07	0.0080902
cg18839504	0.9140476	0.4330972	1.929089	0.8135636
cg10168635	0.1985358	0.0604337	0.652226	0.0077175
cg26706652	954782.87	4.29E-23	2.12E+34	0.6792703
cg14195115	0.3018315	0.1355371	0.672158	0.0033627
cg19551589	0.3817722	0.1647854	0.884484	0.0246838
cg03460756	0.2094241	0.0762351	0.575305	0.0024275
cg09889479	1.6873172	0.6580529	4.326459	0.2761903
cg14311559	4.2465892	1.2259059	14.71036	0.0225323
cg13912887	1.061473	0.23385	4.818152	0.9383888
cg23174201	3.4339568	0.9126471	12.92072	0.0680362
cg02749804	0.6145924	0.2935531	1.286731	0.1966185
cg04420349	0.1894311	0.0494455	0.725731	0.0151925
cg22967016	0.3112606	0.1128399	0.85859	0.0241666
cg26192503	3.1435965	1.2356483	7.997582	0.0162124
cg12610207	1.6664398	0.8155911	3.404919	0.1612668
cg06833431	4.1837206	0.1258091	139.1276	0.4234216
cg16253809	1.8578169	0.6580513	5.245006	0.2421201
cg07533422	1.4632405	0.5987902	3.575664	0.4037197
cg01557411	0.0984027	0.0137644	0.703489	0.0208654
cg15697257	0.1328904	0.0277674	0.635992	0.0115202
cg10278394	2.4895791	0.8671325	7.147702	0.0900697
cg07004999	0.1393216	0.0425988	0.455659	0.0011139
cg21669226	0.6121695	0.2830247	1.324095	0.2124854
cg06417962	0.4466924	0.1947644	1.024489	0.0570618
cg04446762	1.0235153	0.4344505	2.411284	0.9576026
cg25764105	1.496062	0.6114434	3.660522	0.3775604
cg05248321	2.0431214	0.6079481	6.866285	0.2479804
cg06198312	5.192061	0.6324915	42.62112	0.125157
cg03931660	2.6119142	1.0775205	6.331291	0.0335669
cg01250699	12967426	0.0003906	4.3E+17	0.1851564
cg20912205	0.3118818	0.0120911	8.044811	0.4822947
cg19244380	0.4002691	0.2024055	0.791556	0.0084916
cg05937603	0.5596893	0.2848319	1.099779	0.0921822
cg23124641	0.2006033	0.0604256	0.665971	0.0086914
cg25674938	0.2636205	0.1120421	0.620265	0.0022581
cg11638298	1.7022394	0.8100023	3.577297	0.1603615
cg14741804	66118.715	6.80E-07	6.43E+15	0.3898712
cg10109841	0.0577778	0.0077856	0.428774	0.0053027
cg23272978	2.1201376	0.2817131	15.95589	0.465547
cg27406678	1.836168	0.9428138	3.576011	0.0739674
cg25473596	0.9462311	0.3848163	2.326703	0.9041683
cg24737570	3.9611158	1.0722469	14.63323	0.038962
cg25146557	0.1009431	0.0167913	0.606832	0.0122186
cg20072118	0.8868272	0.344206	2.28486	0.8035687

cg11714341	0.5947648	0.2563904	1.379713	0.226186
cg11761622	3.2086759	0.9145659	11.25736	0.0686812
cg21877355	0.2340263	0.0518707	1.055862	0.058858
cg14306709	2.7687786	0.708028	10.82745	0.1432711
cg00727673	1.4556039	0.3751591	5.64769	0.587334
cg11869770	0.0896345	0.0175105	0.458829	0.0037908
cg12362369	0.0015907	1.71E-16	1.48E+10	0.6723615
cg18011672	1.6166692	0.6195997	4.218239	0.3262451
cg17555102	0.0528502	0.0010185	2.742467	0.1444914
cg09396704	3.5544913	0.7972471	15.84754	0.0963401
cg10948657	2.3960417	1.0558737	5.437219	0.0366175
cg13931752	0.1615291	0.0372079	0.70124	0.0149432
cg18976044	2.1000709	0.6266594	7.037791	0.2291617
cg00036723	0.3255633	0.0857222	1.236453	0.0993061
cg14255644	0.1629217	0.0339322	0.782252	0.0234052
cg07095330	0.4755564	0.2383435	0.948857	0.0349518
cg18358723	2.8748674	1.0733315	7.700196	0.0356635
cg09968470	3.3127311	1.0494649	10.45694	0.0411232
cg11072645	0.1643726	0.0427692	0.631725	0.0085734
cg09683730	0.1044762	0.0043869	2.488124	0.162583
cg22358580	0.2279266	0.076542	0.67872	0.0079057
cg26566358	0.7875655	0.389578	1.592132	0.5060735
cg07834510	2.562135	0.919607	7.138414	0.0719154
cg02100543	0.230025	0.0405046	1.306308	0.0972325
cg19021985	1.6793025	0.6478094	4.35322	0.2861397
cg24686551	0.6991439	0.3556049	1.374565	0.299447
cg12209693	0.1157227	0.0356519	0.375624	0.0003307
cg18603698	0.0826857	0.0057238	1.194467	0.0673188
cg08320879	2.6554502	0.0338222	208.4849	0.6608839
cg02204442	0.3145864	0.1193583	0.829138	0.0193408
cg02099878	2.4311291	1.0477836	5.640849	0.0385782
cg21602160	0.0832316	0.0128829	0.537728	0.0090092
cg25628989	0.1497619	0.0154347	1.45313	0.1014986
cg26038649	3.829612	0.7086111	20.69672	0.1187994
cg11362935	7.9309985	1.8237137	34.49047	0.0057595
cg20933005	3.869E+14	3.20E-15	4.67E+43	0.3255447
cg26387064	2.1717141	0.932417	5.05819	0.0722163
cg14188508	1.927522	0.6260408	5.934662	0.2527403
cg03431918	1.890996	0.8087852	4.42128	0.1415007
cg12848457	0.8595258	0.2661087	2.776251	0.8002339
cg10107725	1.8562825	0.6642306	5.187633	0.238117
cg08791882	0.3143624	0.092143	1.072504	0.0645773
cg09235788	0.6520555	0.3148008	1.35062	0.249741
cg05327844	3.0177542	0.3952211	23.0424	0.2869095
cg25306480	0.3276756	0.1380655	0.777684	0.0114016
cg11088380	29999.656	19.519012	46107834	0.0058932
cg03585734	0.5726696	0.2116447	1.549534	0.2723689
cg12470219	2.1541014	0.6209438	7.472742	0.2266123
cg20780944	173.05171	0.6321306	47374.53	0.0718943
cg01624504	1.6899747	0.6716282	4.252374	0.2650651
cg06894134	2.4825527	0.9137784	6.744598	0.074563
cg16107628	1.4562471	0.7487129	2.832402	0.2681433
cg16574737	1.168544	0.5248191	2.601839	0.7029198
cg23731908	0.2910469	0.1254337	0.675323	0.0040522
cg06796381	0.4595502	0.2285305	0.924106	0.0291532
cg06359086	0.0002689	6.35E-08	1.138523	0.0536669
cg20363586	3.1766508	0.8819931	11.44126	0.0770782
cg18552334	0.2723489	0.0845077	0.877718	0.0293752

cg24882324	0.1444421	0.0385043	0.541849	0.004126
cg03762242	0.3086245	0.0779808	1.221442	0.0939402
cg24708861	0.0889196	0.0133621	0.591724	0.0123292
cg22645961	0.3571423	0.1201602	1.061505	0.063944
cg02434554	1.6975054	0.6684625	4.310675	0.2657588
cg27411982	8.0364754	1.5559025	41.50963	0.0128593
cg07729842	0.536907	0.2514375	1.146484	0.1080999
cg17383329	0.3653011	0.1627147	0.820116	0.014664
cg17411031	6.5998859	1.8492828	23.55426	0.0036481
cg14864148	0.5914739	0.2812423	1.243915	0.1662004
cg17882322	0.1443387	0.0270695	0.769635	0.0234161
cg16870867	0.3143846	0.1214599	0.813748	0.0170921
cg15808008	0.2285435	0.0857841	0.608879	0.0031539
cg07042164	0.0094176	1.72E-05	5.156809	0.1470316
cg10532384	0.061697	0.013003	0.292743	0.0004544
cg11749828	0.9967435	0.4623672	2.14872	0.9933594
cg01176433	0.1890689	0.0500247	0.714588	0.0140752
cg23511072	1.6900005	0.2601891	10.97702	0.5825547
cg13514955	2.08E-09	1.71E-48	2.52E+30	0.6632648
cg14438885	1.5209645	0.6633741	3.487222	0.3219167
cg24424889	2.1761865	0.6465053	7.32521	0.2092505
cg13576552	0.0051006	0.0001206	0.215688	0.0057297
cg10494537	1.6914549	0.814559	3.512354	0.1585994
cg00805880	3.4974777	0.6556883	18.65574	0.1426956
cg06988336	13.723365	1.0268033	183.4146	0.0477081
cg02077256	0.8926088	0.1877834	4.242924	0.8864176
cg06059147	1.8838784	0.6840056	5.188551	0.2204879
cg07169003	8.01E-06	4.68E-13	136.9891	0.1672796
cg17153727	2.4265686	1.2042864	4.889398	0.0131383
cg16155923	0.3982775	0.1776192	0.893062	0.0254518
cg02414499	1.6669471	0.7967808	3.487424	0.1748529
cg08465311	0.0028826	2.75E-09	3024.318	0.4082841
cg11478249	0.1166398	0.019412	0.700846	0.0188497
cg27151651	2.12E-21	5.18E-35	8.64E-08	0.0029102
cg07960067	1.63E-23	6.35E-38	4.21E-09	0.0019404
cg04945735	0.5396519	0.1186502	2.454476	0.4247937
cg06144260	3.8986294	0.6025411	25.22535	0.1532334
cg16562730	1.1291122	0.4536958	2.81002	0.7940651
cg21822187	0.3260295	0.0460905	2.306231	0.2615142
cg16136916	0.6411559	0.3004133	1.368384	0.2505029
cg15483084	0.5482944	0.2682548	1.120676	0.0994353
cg26842815	3.0659592	1.0728921	8.761464	0.0365015
cg06179152	0.405744	0.1545991	1.064872	0.0669084
cg12611527	1.5148897	0.2462808	9.318189	0.6540704
cg03752259	3.1834212	1.1872642	8.535733	0.0213879
cg13399816	0.0180212	0.0013744	0.236288	0.0022228
cg23204113	1.7937534	0.5464475	5.888125	0.3353031
cg06622573	1.5711696	0.5743615	4.297945	0.3788631
cg21125143	0.1319121	0.0042901	4.056054	0.2465033
cg03752885	0.4644207	0.2207526	0.977051	0.0432647
cg08602528	0.239799	0.0537562	1.06971	0.0612567
cg21432062	0.3426539	0.1572779	0.746524	0.0070233
cg13010797	0.0004224	9.68E-07	0.184296	0.0122352
cg00619978	0.0557947	0.0089365	0.34835	0.002012
cg01813071	1.217067	0.5746508	2.577656	0.6079057
cg13060154	0.6511669	0.3028891	1.399913	0.2719791
cg07449499	2.2115531	0.8176789	5.981526	0.1179441
cg11787789	7.2684536	0.0942011	560.8258	0.371018

cg00501919	3.789206	0.8466175	16.95935	0.0814725
cg22147598	0.2248252	0.0603847	0.837073	0.0260733
cg03383886	1.3731601	0.671226	2.809141	0.385203
cg12833018	0.2225561	0.0414105	1.196102	0.0799012
cg02599912	10.418323	1.0755891	100.9135	0.0430875
cg23941354	0.4409134	0.1482432	1.31139	0.1408831
cg23682641	0.000107	4.13E-15	2773093	0.4548751
cg02798576	2.5212154	1.0383299	6.121876	0.041046
cg02571636	1.1603812	0.6093705	2.209632	0.6508006
cg17033333	0.3403325	0.1605886	0.72126	0.0049136
cg22389121	0.5644886	0.1192154	2.672871	0.471056
cg14506667	0.3016917	0.1080396	0.84245	0.0221854
cg20980494	0.3521807	0.1571624	0.789191	0.0112432
cg16146501	3.4927751	0.9960017	12.24845	0.050736
cg04085571	1.859882	0.7413756	4.665869	0.1860743
cg12433575	0.2519544	0.1201365	0.528408	0.0002642
cg08084860	0.1760707	0.0379449	0.816998	0.0265494
cg18814174	1.4206726	0.6334344	3.186298	0.394202
cg14036495	0.0006897	1.30E-05	0.036629	0.0003286
cg23101551	0.4393282	0.2086991	0.924821	0.0303299
cg03804985	0.0002961	1.82E-09	48.25885	0.1845492
cg21101465	3.146995	1.2774603	7.752552	0.0126917
cg07582955	0.13837	0.0395544	0.484049	0.0019642
cg20198393	3.1472264	0.0652763	151.7402	0.5620425
cg01525839	10.16649	0.6067209	170.3543	0.1068487
cg15305112	6.0818165	0.0416888	887.2531	0.4776391
cg11007406	0.0004881	3.58E-07	0.666093	0.0384255
cg20598389	40969569	1.45E-10	1.15E+25	0.3925361
cg22258538	0.2234907	0.0692395	0.721382	0.0122031
cg23727321	0.0001032	1.10E-18	9.73E+09	0.576098
cg12049088	107528.1	37.117195	3.12E+08	0.0043916
cg24039042	3.1602171	1.1296036	8.84113	0.0283696
cg07573727	1.1976592	0.6334811	2.264294	0.5788511
cg02869708	0.2446828	0.0844298	0.709105	0.00951
cg12487792	0.4942106	0.2206788	1.106786	0.0866537
cg06182329	0.0138829	0.0015874	0.121417	0.0001108
cg09491120	2.3300368	0.9927817	5.468545	0.0519774
cg20498962	1.6666829	0.5298995	5.242187	0.3822611
cg02610425	0.1045349	0.0011194	9.762166	0.329262
cg01563465	0.0351013	0.0038149	0.322968	0.0030955
cg22061628	2.4812113	0.7900702	7.792231	0.1196135
cg02078943	0.9445288	0.427078	2.088927	0.8879306
cg12139707	0.5328821	0.2679305	1.059839	0.0727658
cg22997113	0.9003317	0.4316302	1.87799	0.7795544
cg00174573	1.1481316	0.4866465	2.708755	0.752443
cg14707948	0.3326267	0.1400695	0.789897	0.0126155
cg03436967	0.6901146	0.3430495	1.388308	0.2983375
cg23429042	1.1789545	0.5958388	2.332734	0.6363349
cg14773619	4.3518774	0.8468121	22.36486	0.0782606
cg09749788	0.2573859	0.0913535	0.725177	0.0102291
cg21055576	65.976205	0.8353533	5210.801	0.0602086
cg18373158	0.3788673	0.1710599	0.839123	0.0167436
cg21852117	2.1660729	0.7683697	6.106269	0.1438284
cg16429725	0.876903	0.3059917	2.513006	0.8068144
cg11405475	0.2889295	0.1226906	0.680413	0.004496
cg25123225	0.0736015	0.0008337	6.497706	0.2537374
cg03510349	0.7865406	0.2867013	2.157807	0.6409886
cg17300051	0.2781586	0.1024682	0.755085	0.0120281

cg25645491	0.5875115	0.2703363	1.276816	0.179293
cg20564259	0.2383664	0.1039613	0.546535	0.0007067
cg12630243	2.1641649	0.5249436	8.922121	0.2854126
cg27215641	0.2475006	0.0493308	1.241751	0.0897261
cg07795325	0.628241	0.1011336	3.902627	0.6179188
cg02362336	0.6443463	0.3343311	1.241829	0.1891953
cg18656132	2.5309377	0.5087243	12.59159	0.2566458
cg01289343	6.0130337	1.3832375	26.13909	0.0167262
cg01129566	6.002E+10	2.24E-13	1.61E+34	0.3672132
cg05333968	1.821334	0.1772539	18.71472	0.6139766
cg02251850	2.7656484	0.857219	8.922821	0.0887222
cg17938383	1.2828145	0.6388376	2.575949	0.483812
cg06794020	0.0055236	0.0003677	0.082978	0.0001696
cg09024606	0.5125929	0.1962835	1.338632	0.1724172
cg20146030	3.6507954	1.0099203	13.19738	0.0482659
cg23655242	0.1695413	0.0461594	0.622717	0.0075055
cg03007309	8.7454229	0.7886788	96.97538	0.0773
cg25975165	13.793987	1.94E-26	9.80E+27	0.9337013
cg13077316	14.738943	1.2099046	179.5484	0.0349145
cg07551230	2.2522577	0.6420319	7.900954	0.2048105
cg01997529	1531312.4	1.94E-07	1.21E+19	0.3472716
cg14128584	4.07E-23	1.77E-42	0.000937	0.0234206
cg10385290	0.352957	0.1863649	0.668467	0.0013932
cg16684958	0.3245936	0.1269441	0.82998	0.0188234
cg12582607	0.1073969	0.0026278	4.389262	0.2385511
cg14507310	1.7809894	0.7553675	4.199179	0.1872089
cg13691622	1.8387156	0.3151759	10.72695	0.4985029
cg04151826	0.6953193	0.1735981	2.78499	0.6077666
cg21077069	1044673.5	15.047946	7.25E+10	0.0148246
cg14022501	0.1756153	0.0279466	1.103561	0.0636129
cg16603578	2.9834102	0.9785267	9.096058	0.0546311
cg14089032	17.873183	0.2181171	1464.584	0.1996323
cg18591107	0.6243894	0.1544411	2.524342	0.5087435
cg27541863	0.6719783	0.1378236	3.276324	0.6228571
cg25168494	3.1032289	0.9945423	9.682876	0.0511121
cg23243400	1.7049358	0.63936	4.546431	0.2863564
cg05785488	1218893.6	5.97E-06	2.49E+17	0.2915624
cg17456304	0.2028615	0.0479409	0.858406	0.0302041
cg12158389	3.56E-05	1.07E-07	0.011897	0.0005513
cg21879571	0.1180059	0.0126216	1.1033	0.0609624
cg19544946	3800.3394	4.33E-08	3.34E+14	0.5214339
cg04244849	2.2052223	0.5578003	8.718184	0.2594841
cg05163325	0.0908182	0.0111851	0.737407	0.0247654
cg18794364	0.4494185	0.0819166	2.465641	0.3571099
cg03631656	0.3494202	0.159899	0.763572	0.0083822
cg01678799	0.3898417	0.1952016	0.778562	0.007603
cg06588605	0.8335155	0.3970167	1.749922	0.6303535
cg19754622	3.1120077	0.5808882	16.67204	0.1849503
cg00290158	0.4923793	0.2337255	1.037274	0.062364
cg09642951	0.3299089	0.0017025	63.9304	0.6798405
cg06132620	1.9947436	0.5523674	7.203542	0.2918868
cg07312880	1.6879567	0.8986533	3.170519	0.1035839
cg01384111	1.6969751	0.6543762	4.400717	0.2767134
cg24549236	3.5504715	0.8887515	14.18377	0.072962
cg00980058	0.3682614	0.0834064	1.625972	0.1873661
cg14995803	0.0003419	1.23E-06	0.094992	0.0054382
cg15862841	0.2732647	0.1248346	0.59818	0.0011724
cg25546248	1.3084825	0.649746	2.635071	0.4515871

cg21656522	5.1845021	1.0348981	25.97266	0.0453183
cg00165323	1.0446256	0.5483401	1.990084	0.8943796
cg13934553	1.6086228	0.5410471	4.782702	0.3925038
cg20956390	2.5688567	1.1071387	5.960432	0.0280226
cg03070741	0.3154004	0.1044206	0.95266	0.0407612
cg04695513	0.4009456	0.1362943	1.179487	0.0968932
cg09850012	0.2682674	0.0828935	0.868191	0.0281029
cg01353448	2.8692573	1.2041097	6.837116	0.0173494
cg02898493	1.4214519	0.586593	3.44451	0.4361243
cg11628739	1.9341209	0.5862727	6.380689	0.2787333
cg25925578	0.3585376	0.1749286	0.734867	0.0050895
cg08594148	4.74E-07	1.16E-17	19394.42	0.2427996
cg07123170	0.2276441	0.0686857	0.754478	0.015487
cg17884674	4.5872859	0.8575506	24.53872	0.0750179
cg11471666	4.1E+15	4.31E-07	3.90E+37	0.1638391
cg20686828	0.3506435	0.0889395	1.38241	0.1343159
cg01914278	1.7486163	0.7637906	4.003269	0.1860549
cg24653728	0.7949148	0.363119	1.740172	0.5658641
cg13364558	0.509994	0.2575717	1.009792	0.0533589
cg11930926	1.8552729	0.8085316	4.257147	0.1447235
cg03754708	0.1812989	0.0466813	0.70412	0.0136357
cg08858245	0.3215181	0.1443967	0.715902	0.005465
cg13721881	2.3530351	1.1182856	4.951127	0.0241639
cg06993714	0.3889889	0.1400846	1.080149	0.0699856
cg00152322	1.7308554	0.7543981	3.971193	0.1953895
cg10120555	0.4181548	0.1759968	0.993503	0.0482993
cg07215236	2.3192378	1.1690072	4.601224	0.016097
cg03593280	0.0181981	0.0007458	0.444057	0.0139706
cg25919202	6.438E+15	1.35E-12	3.06E+43	0.262933
cg24055029	0.1490602	0.0441467	0.503298	0.0021706
cg05303901	0.4538277	0.204439	1.007438	0.0521672
cg25657713	1.6784034	0.6285642	4.481702	0.3014218
cg03485672	0.8554925	0.4436223	1.649754	0.6413429
cg07929642	4.9731213	1.2221006	20.23723	0.0250868
cg00203913	2.4095266	0.8065911	7.19797	0.115252
cg01009145	0.0057289	9.38E-05	0.349773	0.0138669
cg27170816	0.4784397	0.2096744	1.091714	0.0798615
cg06268398	0.5516758	0.2093174	1.453994	0.2290009
cg22612003	1.995372	0.4626181	8.606472	0.3542741
cg08312355	0.2985658	0.1116503	0.7984	0.0160143
cg24027477	1.4308608	0.3773515	5.425612	0.5983008
cg16700924	2.8499027	1.3577175	5.982059	0.0056349
cg19838087	0.1043363	0.0195437	0.55701	0.0081762
cg04844534	1.0523994	0.3369804	3.286673	0.9299562
cg16705627	0.3706952	0.1509382	0.910405	0.0304093
cg08134678	2.3691597	0.8647773	6.490593	0.0934598
cg22009464	0.5860734	0.2821389	1.217422	0.1519985
cg00346985	3.5343835	1.187508	10.5194	0.023281
cg04645123	0.1902925	0.0518723	0.698084	0.0123517
cg01652190	0.2282656	0.0847777	0.614609	0.0034647
cg02429622	5.152E+16	1.03E-23	2.56E+56	0.4093033
cg00253182	1.9030982	0.2431439	14.89563	0.5399071
cg11725852	2.8583854	0.306958	26.61722	0.3562467
cg18192417	1.8716917	0.8714745	4.019888	0.1080027
cg25373553	1.8202643	0.8764731	3.780335	0.1081934
cg06071824	2.3844418	0.6386467	8.902517	0.1960683
cg08732300	0.3094542	0.1101941	0.869029	0.0259859
cg22422264	0.5180163	0.1957382	1.370917	0.1852953

cg23304165	0.3525176	0.1161902	1.069528	0.0655832
cg22960907	5.77E-31	2.43E-50	1.37E-11	0.0022224
cg12486913	0.2995669	0.1242616	0.722189	0.0072552
cg14077106	92.105436	0.1374698	61711.11	0.1731074
cg16772023	1.3867865	0.6291765	3.056657	0.4174185
cg00100564	0.3865871	0.0047149	31.69711	0.6725032
cg04916523	1.1143844	0.4842889	2.564281	0.7989478
cg02370877	2.2959188	1.0094665	5.221811	0.0474303
cg02043679	1.3745246	0.6302257	2.997843	0.4239695
cg24042452	0.3664772	0.1684979	0.797075	0.0113392
cg16564033	1.5330313	0.8815097	2.666091	0.1302129
cg19687075	2.1221943	0.6449566	6.982964	0.2156259
cg24795748	4.7191614	0.628657	35.4255	0.1313878
cg26137915	1.5760437	0.5677242	4.375212	0.382526
cg12991093	3.2542193	0.5932872	17.84961	0.1742198
cg04577441	6109.8031	46.490774	802948.4	0.000461
cg21525330	1.241288	0.4281052	3.599106	0.6906579
cg08269389	0.003117	8.69E-06	1.118245	0.0545142
cg18482892	1.3014859	0.5800781	2.920065	0.522752
cg24908603	1.5656688	0.8355839	2.93366	0.1617209
cg22675604	0.7604304	0.2699307	2.142233	0.6042737
cg11738446	0.3516149	0.1131713	1.092441	0.0707465
cg01614915	2.2106365	0.7382362	6.619716	0.156303
cg25668416	0.0695753	0.0064077	0.755452	0.0284928
cg11465442	1.2808202	0.5792789	2.83197	0.5409651
cg26672375	0.0823601	0.01856	0.365473	0.0010237
cg12750884	0.128406	0.0214689	0.768001	0.0244985
cg03299781	0.5022545	0.2209977	1.141458	0.1001568
cg13797205	2.2854521	0.9691445	5.38959	0.0589774
cg19963589	2.2512338	0.7445456	6.806908	0.1505932
cg27498434	0.2468328	0.0743656	0.819283	0.0222776
cg02392395	0.8074922	0.3383935	1.92688	0.6299071
cg23622322	13.324461	1.87E-05	9517057	0.7065075
cg23489627	0.2008483	0.0203607	1.981274	0.169288
cg00334274	2.2885928	0.8532727	6.138315	0.1000224
cg25916307	1.4261571	0.6725946	3.023997	0.3546002
cg07080224	0.4690133	0.2284162	0.963038	0.039154
cg02932167	7.0443946	1.3045863	38.03772	0.0232689
cg23939865	27.363164	0.873844	856.838	0.0596705
cg20181209	1.6070377	0.7855112	3.287757	0.1939674
cg22530586	0.8095808	0.3367684	1.946207	0.6369112
cg27264018	0.2396429	0.0126147	4.552524	0.3416047
cg16175599	0.2263223	0.0570266	0.898209	0.0346345
cg12162100	0.3519195	0.0150403	8.234353	0.5161718
cg23733123	0.4054858	0.1858333	0.884765	0.0233586
cg05307141	0.5204714	0.2325384	1.164928	0.1121525
cg04210471	2.3273849	0.8509046	6.365838	0.0998734
cg02472646	4.0740024	0.8851517	18.75102	0.0713351
cg03045067	0.0263267	0.0013761	0.503656	0.015716
cg05557991	1.4289626	0.6089168	3.353388	0.4121303
cg14542839	3.0327645	1.2413954	7.409131	0.0149151
cg08966619	6.7160029	0.7992385	56.43459	0.0794955
cg00187889	2.2339049	0.959598	5.200439	0.0622788
cg15763984	0.7977071	0.2558484	2.487163	0.6968696
cg16232674	1.2872123	0.6309244	2.626171	0.4876874
cg07263235	1.6804423	0.8390266	3.365669	0.1430041
cg01021631	0.6088595	0.2764132	1.341144	0.2181516
cg17105041	0.3570667	0.110213	1.15682	0.0859654

cg06315884	0.5308182	0.0337274	8.354275	0.6524316
cg00067600	0.4835936	0.1951178	1.198572	0.1166878
cg16252933	0.7889344	0.4003139	1.554824	0.4934134
cg07252758	0.0204992	3.60E-07	1167.584	0.4865512
cg10096929	0.172822	0.0509306	0.586434	0.0048611
cg06904616	2.3933496	0.5297592	10.81269	0.2566982
cg04350645	1.1705727	0.3737067	3.66662	0.7868901
cg09897131	2.7891053	0.7136643	10.90023	0.1402407
cg10119075	1.2410045	0.6271464	2.455714	0.5352096
cg08611567	3.52E-08	3.61E-15	0.342865	0.0365881
cg11819305	0.9080911	0.2620345	3.147026	0.8791582
cg16060761	15.512705	0.0019475	123564.5	0.549706
cg19539667	4.0275411	1.080819	15.00814	0.0379158
cg22708914	1.4731685	0.6593015	3.291704	0.3449446
cg09615821	0.3490337	0.1599992	0.761407	0.0081711
cg03492827	0.055982	0.0083577	0.374981	0.0029701
cg10949862	0.7282045	0.1044821	5.075335	0.7488328
cg13564061	0.3761536	0.0945952	1.495757	0.1650514
cg10541813	41975.715	156.35026	11269317	0.0001911
cg03893692	5.5273891	0.2908822	105.0323	0.2551091
cg23632849	0.0932816	0.0248322	0.35041	0.0004432
cg01248421	45942.732	11.242409	1.88E+08	0.0113969
cg06561886	0.2195629	0.0639019	0.754404	0.0160628
cg01826254	2.074561	0.8519445	5.051741	0.1080348
cg22480875	1.8403087	0.1509834	22.43118	0.6325935
cg11113589	1.6251729	0.5218186	5.061504	0.402141
cg16901082	233.38296	0.9802888	55562.81	0.0508393
cg24542751	4.0397906	1.1304683	14.43641	0.031659
cg02086349	0.0522115	0.0070538	0.386466	0.0038422
cg05011265	2.0909032	0.3491757	12.52056	0.4192441
cg22344830	1.3407352	0.635849	2.82704	0.4410872
cg18997124	1.9284882	0.7674326	4.846115	0.1624375
cg14131038	0.2010764	0.0663826	0.609071	0.0045563
cg13866767	0.5532434	0.2992707	1.022747	0.0589968
cg05077231	0.7245185	0.2702931	1.942066	0.5218072
cg04001138	0.7204638	0.3228106	1.607965	0.4234724
cg09246754	33074418	11.745524	9.31E+13	0.0223078
cg13485564	2.6344721	1.2011245	5.778288	0.0156374
cg21633105	2.1992725	0.8848637	5.466153	0.0897654
cg20252111	0.3693896	0.0358457	3.806553	0.4027069
cg00197993	3.4396019	0.6781694	17.44529	0.1359138
cg25522046	15.486392	1.3127963	182.6851	0.0295465
cg17317391	0.2461429	0.104464	0.579973	0.001347
cg23263911	2.4235799	1.0891853	5.392783	0.030059
cg08991742	1.2174376	0.5416798	2.736218	0.6339501
cg27089736	4.3080437	1.2336995	15.04357	0.0220711
cg12406027	0.0411925	0.0009491	1.787841	0.0973281
cg03129645	0.5250044	0.2228425	1.236881	0.1405544
cg06836948	0.5591865	0.2494879	1.253325	0.1580645
cg24196240	0.531513	0.1824112	1.548732	0.246745
cg16544463	1.8738342	0.5531908	6.347276	0.3130486
cg04528477	3.0184385	0.9979703	9.129501	0.0504221
cg25098479	0.3464919	0.1125282	1.066903	0.0647318
cg15312943	0.5241641	0.2211943	1.242112	0.1422607
cg06067208	0.0795399	0.0077184	0.819679	0.033417
cg04865113	0.3345068	0.1023288	1.093482	0.069973
cg15374924	0.3253024	0.10497	1.008113	0.0516593
cg04357965	0.1540722	0.0604063	0.392976	9.04E-05

cg02806675	0.6102635	0.3018248	1.2339	0.1691773
cg14870136	3.8294576	1.4204199	10.32423	0.0079656
cg11471772	2.6145088	1.3060081	5.234007	0.0066509
cg01574233	0.4039352	0.2014774	0.809836	0.0106403
cg25268435	0.4434899	0.2147246	0.915979	0.0280123
cg11694987	0.0891873	0.0063641	1.249887	0.072754
cg00713113	89219323	1.4737785	5.4E+15	0.0452433
cg26707398	11.755357	0.5419911	254.9644	0.1164637
cg17852507	0.4182547	0.2038659	0.858098	0.0174375
cg03933341	0.0288616	0.0008872	0.938871	0.0459919
cg22891862	1.5971715	0.7042887	3.622033	0.2623678
cg00529371	1.0919585	0.3421091	3.48536	0.8818974
cg15408020	0.3419921	0.1767782	0.661612	0.0014383
cg12267883	3.7475813	0.000486	28895.61	0.772352
cg26163153	3.5882199	0.9115571	14.12454	0.067623
cg27375378	0.0108689	0.0012774	0.092478	3.48E-05
cg25466154	1.4608601	0.3933012	5.426152	0.5713067
cg20674658	0.3442193	0.1377855	0.859938	0.0224314
cg14696064	1.7296454	0.7061256	4.236744	0.2306417
cg07738955	0.3325852	0.1113099	0.993738	0.0486996
cg22864414	2.8742403	2.36E-08	3.51E+08	0.9115081
cg18698884	1.8292473	0.582428	5.745166	0.3010278
cg08415508	4.5456485	1.3795048	14.97851	0.0128187
cg21203569	0.4612474	0.1474777	1.442585	0.1834842
cg10580715	0.2093137	0.0656223	0.667642	0.0082266
cg01094351	1.0519956	0.4709968	2.349686	0.901608
cg05928806	3.2759038	1.177257	9.11572	0.0230569
cg00027586	0.3813484	0.1436405	1.012434	0.0529709
cg01946760	0.0139929	0.0002659	0.736304	0.034742
cg01933248	0.4710359	0.1950686	1.13742	0.0941904
cg21617494	42.258858	0.2720752	6563.667	0.1458586
cg13803688	4.3934999	0.8222816	23.47473	0.0834319
cg00049868	1.365575	0.5920056	3.149962	0.465001
cg01213134	2.0634856	0.5135154	8.291811	0.3073528
cg11357013	2.0032667	0.7756385	5.173902	0.1512434
cg09697978	0.505878	0.2621958	0.976036	0.0421231
cg08015503	0.1834875	0.0457206	0.736378	0.0167761
cg19358442	2.5690295	1.1365686	5.806876	0.0233521
cg27318810	4.1789166	0.8695673	20.0828	0.0741852
cg07036914	0.3316838	0.1433483	0.76746	0.0099283
cg01084837	0.7136135	0.0021355	238.4663	0.9094026
cg05006892	0.0007334	1.17E-06	0.459062	0.0280246
cg07623116	1.8669166	0.4507378	7.732605	0.3892514
cg03223659	109.11131	4.3558786	2733.152	0.0042979
cg01167556	1.9482192	0.8494642	4.468179	0.1153182
cg27224809	2.7397424	0.967804	7.755897	0.0576533
cg20450123	1.5813164	0.4548953	5.497005	0.4709877
cg12757570	0.279355	0.0859333	0.908138	0.0339923
cg01445411	0.6705333	0.3582291	1.255104	0.2114532
cg24310133	0.3577236	0.138967	0.920838	0.033096
cg19206146	1.6219491	0.9063367	2.902585	0.1033638
cg26578274	0.6056157	0.2863265	1.280952	0.189473
cg20963696	29.009469	8.71E-06	96645482	0.6603188
cg26104143	1.9461437	1.0498621	3.607593	0.0344738
cg18068521	0.8543966	0.3736478	1.953694	0.7092222
cg10587183	1.6548328	0.676299	4.049202	0.2699071
cg09750210	0.5555907	0.2295944	1.344463	0.1924078
cg06064964	3.8333832	1.0941504	13.43035	0.0356741

cg03930965	129.6153	1.1835364	14194.85	0.0423268
cg09420988	9.835E+09	1.64E-46	5.90E+65	0.7254903
cg24008577	1.0772463	0.4472095	2.594891	0.868246
cg02304465	3.9526542	1.0026268	15.58254	0.0495635
cg11566975	0.0473484	0.0041757	0.536884	0.0138168
cg03938015	0.8089806	0.3116896	2.099684	0.6631172
cg24092340	1.0525726	0.4369933	2.5353	0.9090497
cg04315923	30036.181	3.79E-06	2.38E+14	0.3753322
cg19501982	1.9815429	1.0714896	3.664536	0.0292513
cg04957757	0.2165653	0.0955992	0.490595	0.0002456
cg23115907	2.4256821	1.1194276	5.256199	0.02471
cg07750130	0.8023948	0.0598269	10.76168	0.8679949
cg15570293	0.5330848	0.1539624	1.845772	0.3208333
cg10997500	0.1985747	0.0611499	0.64484	0.0071438
cg01720920	0.298143	0.1201049	0.740097	0.0090869
cg02758236	0.2914632	0.1185763	0.716423	0.0072156
cg26588076	0.2153753	0.072067	0.643658	0.0059826
cg09783253	3.1554084	0.6046739	16.46607	0.1728247
cg18124162	2.4620031	0.2399442	25.26196	0.4481915
cg07922933	2.2475568	0.7870759	6.418074	0.1303493
cg17661642	2.4273351	0.7241175	8.13674	0.1507426
cg08625842	0.2624998	0.1084856	0.635164	0.0030103
cg03264156	4.39E-05	9.63E-10	2.004163	0.0668099
cg01966334	2.1480591	0.9749848	4.732543	0.0578141
cg18117669	0.4597883	0.1547809	1.365835	0.1618951
cg15515095	0.0027755	0.0001409	0.054684	0.0001084
cg21331510	0.355252	0.1168378	1.080164	0.0681441
cg09160123	0.0983785	0.0232971	0.415431	0.001604
cg03617700	32.617307	1.7788513	598.0763	0.0188723
cg18282041	0.170982	0.0219716	1.330573	0.0915767
cg03596167	3.5680741	1.2045709	10.56904	0.0216815
cg04058563	0.2485576	0.1247848	0.4951	7.51E-05
cg11755201	1.217314	0.5803968	2.553173	0.6028173
cg27582546	1.5586575	0.5080232	4.782091	0.4377787
cg20321153	2.8738233	0.7655705	10.78785	0.1177822
cg05209306	1.6497102	0.7388527	3.683473	0.2219064
cg07548982	0.5491812	0.2832257	1.064875	0.0760771
cg02995791	0.4719781	0.2577613	0.864223	0.0149834
cg16587707	0.2510458	0.0828052	0.761112	0.0145925
cg27568360	1.5450325	0.5517329	4.326596	0.4076425
cg13466694	1.9499902	1.0072668	3.77503	0.0475412
cg04235768	0.0427919	0.0048672	0.376224	0.0044922
cg16176188	1.978E+09	1.23E-06	3.18E+24	0.2308419
cg16243646	0.9666784	0.0481294	19.41571	0.9823356
cg09910120	0.4472265	0.2039814	0.980538	0.0445336
cg18149119	673.68736	5.72E-05	7.93E+09	0.4330438
cg22188917	0.2197434	0.0012792	37.74893	0.5638691
cg13399741	0.8920592	0.2996663	2.655519	0.8373962
cg02788266	2.1162403	1.0679795	4.193407	0.0316776
cg03878567	0.5630099	0.2653177	1.194719	0.1345245
cg11726857	0.5525742	0.2376645	1.284745	0.1682287
cg12206103	1.851713	0.7065103	4.853207	0.2101097
cg12730087	1.8564932	0.0921341	37.40817	0.6863802
cg15751116	2.1081921	0.660449	6.729473	0.2078677
cg14698932	1.2414436	0.0971958	15.85647	0.867836
cg16966992	0.929758	0.4083909	2.116722	0.862251
cg14862981	0.6693795	0.0295399	15.16828	0.8009559
cg14501985	0.6941549	0.277041	1.739277	0.4359988

cg17202909	3.0718983	0.8454983	11.16094	0.088195
cg06721231	0.712898	0.3228511	1.574173	0.4024096
cg09222791	0.5032744	1.86E-11	1.36E+10	0.9553223
cg05922961	5.31E-06	6.64E-10	0.04252	0.0080823
cg11127561	2.04E-08	1.80E-21	230938.6	0.2482165
cg02505454	1.5061941	0.6234443	3.638851	0.3627751
cg08969925	2.5053942	0.6814718	9.210945	0.1667757
cg08532834	0.0996502	0.0013756	7.218539	0.2912589
cg06740995	0.5607657	0.2563658	1.2266	0.1474747
cg06328724	0.1206282	0.0276019	0.527179	0.0049422
cg07834682	3.0597535	1.1118922	8.419963	0.0303629
cg09447006	72553.737	0.0054537	9.65E+11	0.1811325
cg22804362	0.2909682	0.1326155	0.638406	0.0020743
cg11214757	3.9659304	0.9490618	16.57279	0.0589846
cg03529184	1.3064075	0.587844	2.903322	0.5118272
cg06787669	2.3289501	0.613259	8.844564	0.2143247
cg15440026	0.6163537	0.2105309	1.804447	0.3772435
cg07010359	40398085	8.0369958	2.03E+14	0.0261025
cg24332783	2.3739208	0.8957841	6.291136	0.0820987
cg01117549	0.3471956	0.0757463	1.591428	0.1732522
cg04875128	0.6921668	0.2778488	1.724301	0.4294934
cg11255443	0.0521193	0.0046727	0.581336	0.0163605
cg14564229	0.3825773	0.1768459	0.827644	0.0146688
cg06768010	0.1626498	0.0529845	0.499296	0.0015052
cg01334209	0.4008341	0.1675768	0.958772	0.0399193
cg09492169	0.4268556	0.1737339	1.048763	0.0634315
cg07434271	2.7368393	1.0652163	7.031708	0.0365112
cg20649847	125.25977	4.64E-10	3.38E+13	0.719084
cg06529453	8.813E+16	74.19403	1.05E+32	0.0275847
cg02952849	1.6671642	0.6918938	4.017143	0.2546582
cg16619858	1.6734403	0.5641142	4.964247	0.3533787
cg09886931	0.0466436	0.0020961	1.037933	0.0528134
cg01027405	0.7004369	0.3136875	1.564015	0.3850014
cg08314996	5.5831498	0.5951435	52.37655	0.1321632
cg22511293	9.92E-14	5.95E-33	1656726	0.1848932
cg24599270	0.2617384	0.0010294	66.55	0.6352462
cg09950208	1.8302004	0.9118702	3.673367	0.0890524
cg09313931	1.1245471	0.5313674	2.379909	0.7589359
cg27580375	1.1588378	0.5735775	2.341279	0.6811922
cg13547725	1.4906044	0.7040159	3.156039	0.2969545
cg06896770	0.2078623	0.0144799	2.983912	0.2478116
cg14094639	0.6192527	0.2460995	1.558206	0.3087246
cg26292575	2.4382689	0.9451723	6.290023	0.0652789
cg17304084	113.16532	0.005093	2514522	0.3544315
cg13518488	0.6605548	0.3220765	1.354749	0.2578435
cg18137099	0.0330008	0.0026415	0.41229	0.0081049
cg18257485	3.6481911	1.0196553	13.05274	0.0466029
cg23987134	2.5180221	0.4008512	15.81743	0.3246505
cg16274376	0.2130823	0.0737735	0.615452	0.004278
cg12738008	3.2270205	1.0104627	10.30583	0.0479814
cg02555393	0.0428744	0.0001313	13.99971	0.2862434
cg07637243	1.6498012	0.438772	6.203322	0.4587563
cg23723775	0.2429671	0.108429	0.544439	0.0005884
cg26886259	1.8769339	0.5640568	6.245614	0.3046671
cg13717434	0.4488767	0.2110437	0.954732	0.0375007
cg07984358	0.4127406	0.1313945	1.296514	0.1296948
cg05046722	0.6680011	0.2322662	1.921181	0.4541255
cg27340749	0.0221017	0.0003204	1.524715	0.0776141

cg15330298	1.4795002	0.5042743	4.340734	0.4756753
cg02329670	1.9976035	0.3512921	11.35927	0.435226
cg06008531	0.4702897	0.190887	1.158656	0.101034
cg14741143	0.1468431	0.0296655	0.726867	0.0187282
cg19934294	0.5822138	0.1361998	2.488791	0.4655176
cg14242995	0.3445024	0.1184764	1.001734	0.0503731
cg04034290	0.6444267	0.3243197	1.280483	0.2097567
cg18096388	1.4927211	0.5753311	3.872929	0.4102061
cg20047349	0.4846768	0.2324943	1.010397	0.0533142
cg03159396	1.7118778	0.9510123	3.08148	0.0730548
cg14554519	1.9440752	0.8193004	4.612995	0.1315817
cg14511375	4.6588158	1.0170397	21.34092	0.0475101
cg04025049	0.3305197	0.1544876	0.707133	0.0043309
cg26957602	0.1413942	0.0267935	0.746164	0.0211677
cg27582527	2.2930249	0.6545588	8.032836	0.19449
cg13430960	1.5323564	0.0362611	64.75571	0.8231915
cg26532627	3.4877361	1.1218021	10.84354	0.0308843
cg27570249	2.5418889	0.6072818	10.63954	0.2015473
cg05143386	0.1360976	0.0343416	0.539362	0.0045299
cg12018521	0.1776703	0.0851395	0.370765	4.16E-06
cg14899408	2.8920899	1.1988489	6.976846	0.0180976
cg19539568	2.31746	0.3650623	14.71152	0.3727595
cg25497530	0.2927994	0.1056126	0.811754	0.0182341
cg23411426	1.5086334	0.6009348	3.787391	0.3812598
cg18687753	0.0534819	0.0003023	9.460688	0.2674394
cg03783663	2.4476682	1.0761856	5.566958	0.0327534
cg01388757	1.9550798	0.9792448	3.903352	0.0573664
cg17016175	1.7801392	0.001948	1626.754	0.8683226
cg03764259	1.634663	0.889626	3.003647	0.1133782
cg19182423	0.3223442	0.1337869	0.776651	0.0116249
cg06282247	1.9930072	0.5560893	7.142877	0.2896369
cg17497052	0.0244005	0.0026354	0.225914	0.0010753
cg09727277	2.8085742	1.1762742	6.705995	0.020041
cg00519829	2.2282768	0.8040415	6.175324	0.1234154
cg23704362	0.3261116	0.138636	0.767108	0.0102448
cg23502378	0.2777902	0.0912793	0.845398	0.0240875
cg26052586	0.3723018	0.1455281	0.952453	0.039245
cg05415936	2.3911322	0.0312739	182.8207	0.6935884
cg14688342	2.5772583	0.8894426	7.467891	0.0811376
cg05296818	1.2385404	0.5052144	3.036299	0.6400688
cg18063332	2.2837714	0.5179197	10.07031	0.2753299
cg10989175	5.7468974	0.0398783	828.191	0.4904968
cg04276765	2.6943241	1.0320955	7.033634	0.0429196
cg01129320	2.0934873	0.7837979	5.591606	0.140489
cg23005196	4.37E-27	2.25E-52	0.084571	0.0410412
cg14091103	0.2302564	0.0833867	0.635809	0.0045994
cg21113446	0.3581123	0.1403244	0.913914	0.0316914
cg24498760	4.430364	0.0743058	264.1532	0.4754534
cg21282907	0.508506	0.13061	1.979775	0.3294869
cg22246785	0.2496418	0.0890093	0.700163	0.0083548
cg20596640	0.0439471	0.0025115	0.768988	0.032367
cg24171992	0.1767419	0.0591131	0.528439	0.0019262
cg21983491	0.5303374	0.1976635	1.422912	0.2078383
cg02787917	2.1206329	1.0310695	4.361572	0.0410401
cg03484438	28684.416	6.07E-06	1.35E+14	0.3664694
cg02627405	0.3442253	0.1448559	0.817993	0.0157402
cg03750587	4.280664	1.15072	15.92402	0.0300515
cg09647110	0.2900985	0.1367724	0.615308	0.001256

cg18261246	2.2849664	0.9592354	5.442951	0.0620435
cg22719241	0.4196342	0.1952662	0.901809	0.026098
cg07784959	6.6576742	1.4392902	30.79617	0.0152682
cg11893127	0.3793702	0.1496262	0.961875	0.0411664
cg09512004	4.5946689	1.8090059	11.66994	0.0013441
cg01434302	0.4851658	0.2224136	1.058325	0.0691386
cg12646642	7.10E-12	4.47E-24	11.28086	0.0733063
cg16058485	0.1299605	0.0486159	0.347412	4.75E-05
cg12699756	1.7445474	0.4239582	7.178645	0.4406894
cg04105511	2.4203519	0.8598098	6.813255	0.0941459
cg13282195	0.1292979	0.0264876	0.631162	0.0114432
cg07374928	0.0176339	0.0011247	0.276482	0.0040343
cg11668923	0.207095	0.0383118	1.119454	0.067415
cg01712737	1.753725	0.721815	4.260858	0.2148875
cg12004430	0.1326551	0.0428749	0.410436	0.0004561
cg26490274	11.300633	0.0300061	4255.941	0.4229624
cg13371767	3.5141323	1.448632	8.524681	0.0054414
cg12707346	2.0415051	0.4175672	9.981012	0.3780938
cg01631226	0.4477994	0.1501053	1.335891	0.1496806
cg21868134	0.3026221	0.083606	1.095378	0.0685823
cg27109056	5.5971156	1.6971527	18.45898	0.004673
cg14145194	2.8820118	0.8938092	9.292803	0.0763906
cg10359143	0.6839261	0.3506427	1.333993	0.2650497
cg06762457	1.195063	0.499798	2.857506	0.6886806
cg01079599	1.2254299	0.4371837	3.434891	0.699068
cg14183540	0.4711774	0.2136259	1.039238	0.0622375
cg12100721	0.3015314	0.1355624	0.670696	0.00329
cg06254440	1.8846785	0.9083076	3.910584	0.0888064
cg08161802	1.777456	0.5372665	5.880415	0.3460695
cg19187185	0.1125188	1.30E-05	975.7361	0.6367857
cg14975632	3.1019332	0.147399	65.27851	0.4664574
cg07565540	0.012853	8.91E-06	18.54181	0.2407194
cg24957695	0.066557	0.0084455	0.524522	0.0100944
cg05618401	0.2898079	0.108074	0.77714	0.0138569
cg15615645	0.2681559	0.132053	0.544536	0.0002708
cg20841134	0.0663833	0.0075449	0.584067	0.0144999
cg20146177	0.7670084	0.3707305	1.586872	0.474546
cg11026987	0.3803159	0.0206184	7.015092	0.5156545
cg12063490	0.3394308	0.1431424	0.804886	0.01418
cg04876424	11.974988	2.1225364	67.56084	0.0049156
cg14557552	78.745281	1.8797303	3298.781	0.0219551
cg27299776	0.5012247	0.0060042	41.84173	0.7596352
cg16327381	0.0680438	0.0126264	0.36669	0.0017639
cg21042248	2.0808344	0.8293275	5.220943	0.1184667
cg19224645	2.092845	0.8308498	5.271711	0.1171555
cg14053318	17.810029	0.0038471	82451.85	0.5036671
cg11837377	149.71081	1.40E-19	1.60E+23	0.8393324
cg03953513	1.6793654	0.2924235	9.644464	0.5610449
cg17777432	1.6723041	0.8047151	3.475268	0.1682652
cg08244750	0.3255885	0.1045729	1.013722	0.0528133
cg00993677	1.7059089	0.7373059	3.946971	0.2120627
cg18230627	8.717415	2.2643535	33.56072	0.0016425
cg03401357	1.2559242	0.6595148	2.391676	0.4880725
cg24701780	0.198532	0.049483	0.796535	0.0225553
cg01629435	1.1424663	0.0332941	39.20307	0.9411422
cg02575078	1.6941262	0.0717667	39.99156	0.7438072
cg03258475	2.08603	0.3336267	13.04309	0.4317543
cg01876531	1.2637542	0.5896809	2.708371	0.5472433

cg15526825	0.1840421	0.060536	0.559527	0.0028499
cg08470180	0.0376348	0.0018865	0.750779	0.0317407
cg18220168	0.0693385	0.0011923	4.032314	0.1979691
cg13293266	1.0585192	0.2599119	4.310933	0.9367344
cg08964780	3.044995	0.9384209	9.880423	0.0637201
cg16937735	0.2571378	0.0504105	1.311629	0.1023296
cg01281231	0.4840667	0.2284032	1.025907	0.0583284
cg20371142	0.3058035	0.1176415	0.794922	0.0150636
cg13149245	0.3391499	0.144973	0.793408	0.012644
cg03176434	1.862666	0.5676897	6.111657	0.3048786
cg02825511	0.0959552	0.0217463	0.423401	0.00197
cg06039161	0.114674	0.0206415	0.637073	0.0133125
cg16871519	0.066549	0.0056577	0.782781	0.0311855
cg23274824	1.5733622	0.5438613	4.551654	0.4030373
cg14900154	0.1860925	0.0544932	0.6355	0.0072872
cg18932726	0.2732125	0.0071749	10.40368	0.4847344
cg22512256	0.457258	0.1650822	1.26655	0.1322265
cg11534680	2.0327248	0.9225239	4.478984	0.078425
cg12575771	2.0747063	0.7985359	5.390373	0.1340959
cg04387010	0.0864739	0.0036061	2.073633	0.1310253
cg03416085	1.6422826	0.5407543	4.987648	0.3814294
cg27168131	1.4940306	0.4002555	5.576757	0.550226
cg22732126	0.224082	0.0664601	0.755532	0.0158641
cg03155301	11.361972	0.238451	541.3875	0.2176625
cg16235962	2.2619277	0.7864931	6.505228	0.1299336
cg27576576	1.499832	0.6829572	3.293758	0.3125352
cg09917979	0.0883434	0.014171	0.550743	0.009355
cg04025127	0.2902898	0.1168211	0.721344	0.0077378
cg12827405	0.4168677	0.1810983	0.959582	0.0396906
cg24174996	0.7265799	0.350593	1.505787	0.3903002
cg18213545	0.1690446	0.0371333	0.769554	0.0215222
cg14911393	1.8397708	0.3102607	10.9094	0.5020397
cg23837191	1.7204401	0.6578236	4.499556	0.2686677
cg27569107	0.1270569	0.0123257	1.309737	0.0830466
cg20558646	1.27E-12	1.11E-32	1.45E+08	0.2449925
cg02054776	2.039038	0.6519986	6.376818	0.2206752
cg24512093	2.219166	0.8218432	5.992259	0.1157585
cg13239041	0.4949975	0.2064318	1.186942	0.1150496
cg24030735	1.350491	0.3534379	5.160245	0.6604343
cg24338748	0.0717545	0.0145877	0.352949	0.0011901
cg03943135	5.1783741	0.2102242	127.557	0.3144385
cg21593409	0.4328952	0.1564864	1.197537	0.1068025
cg18207201	0.5821585	0.2676674	1.266155	0.1723488
cg10336790	0.3484728	0.164963	0.736124	0.0057295
cg17587456	2.2396345	0.9412014	5.329319	0.0683094
cg01689404	3.910178	0.9323611	16.39868	0.0622914
cg20482274	0.6583356	0.2535385	1.709428	0.3905213
cg00537210	0.301412	0.122658	0.740671	0.0089388
cg18411108	1.0408148	0.3539816	3.060316	0.9420461
cg08272151	0.2085469	0.0707132	0.615045	0.0044999
cg22539359	7.725E+13	2.09E-12	2.86E+39	0.2870609
cg00133970	13.493149	0.0791878	2299.155	0.3208964
cg08124910	3.2045698	1.1340205	9.055628	0.028002
cg00259549	2.5120767	0.7301941	8.642263	0.1439718
cg25680014	0.2571319	0.0944802	0.699795	0.0078427
cg15109118	0.0206493	0.0010106	0.421921	0.0117176
cg21503330	0.1000384	0.021595	0.463427	0.0032482
cg17347386	0.4866461	0.222288	1.065394	0.0716216

cg02921623	2.183969	1.0307998	4.627204	0.0414348
cg09375756	0.5193726	0.1648556	1.636268	0.2631669
cg18146246	0.6215473	0.2445961	1.579425	0.3175992
cg01538821	2.2659486	0.9708841	5.288503	0.0585399
cg02079551	0.7422211	0.3595244	1.53228	0.420211
cg23315892	0.4061856	0.1599281	1.031631	0.0581612
cg03840289	0.4624753	0.1572799	1.359891	0.1611091
cg14426525	0.2239573	0.0082863	6.052986	0.3737109
cg11323828	0.3836055	0.1419648	1.036547	0.0588665
cg13770573	0.2282498	0.0587921	0.886139	0.0327912
cg00719165	0.0687468	0.0081581	0.579314	0.0138179
cg03536375	2.0706275	1.0104894	4.242992	0.0467596
cg04301682	10.640165	0.0005159	219466.9	0.6408403
cg27373591	1.2669551	0.6857336	2.340815	0.4499769
cg27628784	0.0863184	0.0136085	0.547513	0.0093481
cg15901199	0.0006802	5.80E-15	79731984	0.5749068
cg15096085	0.2130221	0.0444043	1.021938	0.053256
cg12412089	0.0553063	0.0056483	0.541546	0.0128882
cg02560510	0.931569	0.4780409	1.815369	0.8350417
cg24904818	0.7477434	0.2302837	2.427962	0.6285531
cg20725013	1.8623515	0.7574957	4.57871	0.1754684
cg05774756	0.4475907	0.1101674	1.818481	0.2610561
cg26171114	0.4124385	0.1326308	1.282549	0.1260023
cg21506819	0.7575884	0.3924324	1.46252	0.4081211
cg02489685	5.4166609	1.63E-11	1.8E+12	0.90067
cg10740660	0.193419	0.0410513	0.911322	0.0377664
cg07506081	0.3030891	0.1529212	0.600721	0.0006261
cg09452082	1.2134215	0.4725989	3.115521	0.6876243
cg17925329	0.0027734	2.61E-07	29.44843	0.2132079
cg25349276	0.6659804	0.2909351	1.524498	0.3360342
cg05367028	4.4407242	1.2824338	15.37704	0.0186474
cg06094745	0.410111	0.1565727	1.074204	0.0696368
cg12473916	0.158823	0.0217232	1.161192	0.0698736
cg15843217	0.126879	0.0320377	0.502479	0.0032821
cg13117272	0.2768636	0.090715	0.844992	0.0240821
cg00395931	1.35E-28	9.48E-44	1.91E-13	0.0003122
cg06989610	1.1134685	0.3579966	3.463195	0.8527209
cg22511852	4.2015319	1.4778132	11.94527	0.0070904
cg27639104	2.6408119	0.5681093	12.27561	0.2154572
cg10547908	0.1609622	0.0431464	0.600486	0.0065436
cg01961525	20263734	9.80E-11	4.19E+24	0.4082077
cg01804934	2.0160375	1.0283907	3.952201	0.0412035
cg26458072	0.2435864	0.1015809	0.584109	0.0015516
cg00735599	0.9092656	0.473476	1.746158	0.7751096
cg07723948	0.4037657	0.1560229	1.04489	0.0615604
cg03937361	3.0938366	0.9658771	9.909983	0.0572352
cg09145900	6.1021957	0.8450356	44.06535	0.0729664
cg26118759	1.9887387	0.7334503	5.392433	0.1767406
cg16282892	1.449838	0.7874092	2.669552	0.2330269
cg00201819	0.2386077	0.0644657	0.883161	0.0318695
cg04153130	0.4743224	0.2043034	1.101214	0.0826322
cg26620370	5.2193618	1.091753	24.95229	0.0384587
cg26593241	0.9137343	0.3074736	2.715389	0.8710335
cg06581356	12.197897	1.0847419	137.1651	0.0427806
cg02020945	2.5497961	1.2897361	5.040923	0.0071103
cg13849647	1.5658335	0.6624225	3.701316	0.3069527
cg23475045	0.5482193	0.2654183	1.132342	0.1043473
cg16415104	0.0849639	0.0242991	0.297083	0.0001132

cg00406319	0.4152813	0.0965871	1.785523	0.2376263
cg05886811	3.2402207	1.3535929	7.756416	0.0082958
cg09791673	0.6475693	0.2516779	1.666202	0.3675047
cg14462402	0.3976166	0.1318484	1.199097	0.101511
cg12331004	0.5137185	0.2371739	1.112714	0.0911959
cg09163779	44.840585	0.0387934	51830.4	0.2905538
cg12032027	2.4686939	0.615196	9.906517	0.2024159
cg00973118	0.1964667	0.0804021	0.480076	0.0003574
cg09445097	2.209E+10	0.0024281	2.01E+23	0.1177002
cg05228110	1.0488017	0.1480585	7.429396	0.9619545
cg03290530	2.2645403	1.098479	4.668403	0.0267992
cg09917805	3.3090981	0.8614728	12.71094	0.0813682
cg06941093	14.192475	1.1646196	172.9546	0.0375784
cg20408104	0.4729093	0.2528761	0.884398	0.0190481
cg03757552	1.9757034	0.8322986	4.689908	0.1226396
cg09019635	0.39157	0.1601376	0.957471	0.0398569
cg01067936	2.97E-24	3.97E-46	0.022159	0.0350127
cg27326306	0.4893006	0.1253368	1.910174	0.3036625
cg01920011	0.2606612	0.0703895	0.965261	0.0441255
cg20629587	0.2531234	0.093423	0.685821	0.0069015
cg00328376	0.4760879	0.2152577	1.052969	0.066874
cg12271981	1.56439	0.8064536	3.034665	0.1856098
cg17242351	2.4965655	1.3955849	4.466113	0.0020478
cg10841124	1.2930142	0.4257071	3.927315	0.650296
cg13141791	3.71328	0.9956716	13.84839	0.0507598
cg22804000	0.3291002	0.1474384	0.734591	0.0066707
cg03535066	2551.6554	8.43E-07	7.72E+12	0.4812488
cg15244101	2.0597753	0.5188316	8.177363	0.3043302
cg09692695	0.2036125	0.0722045	0.574175	0.0026221
cg14481336	0.4608568	0.2070104	1.025983	0.0578087
cg20776169	1.88E+16	0.0204478	1.73E+34	0.075792
cg18845797	0.9545824	0.4178272	2.180872	0.9121992
cg00175150	0.1526311	0.0351826	0.662152	0.0120533
cg01549977	0.4393339	0.2005546	0.962403	0.0398069
cg16396223	3.8067543	1.3165748	11.00688	0.0135995
cg08368875	0.2301593	0.0813572	0.65112	0.0056294
cg13253642	0.3047282	0.0699737	1.32706	0.1134177
cg11473876	2.3490006	1.2670622	4.354801	0.0066976
cg04334496	1.467379	0.7333285	2.936203	0.2785581
cg16574871	1.6899828	0.7068927	4.040277	0.2380243
cg03178541	1.3207167	0.5735517	3.041212	0.5133261
cg23273364	2.127E+09	9.27E-28	4.88E+45	0.6151097
cg18303360	1.7923493	0.6915219	4.645574	0.2298023
cg17684572	0.5966072	1.69E-05	21033.1	0.9229774
cg00319312	0.6555788	0.3220331	1.334594	0.2443539
cg03022263	0.3299794	0.1284502	0.847694	0.0212666
cg22242614	0.4317173	0.1882021	0.990318	0.047375
cg26543150	0.441851	0.220565	0.885146	0.0212153
cg16219322	2.6608586	0.9520825	7.436507	0.0619964
cg16949120	0.0844861	0.01407	0.507314	0.006893
cg03036452	6.26E-05	1.94E-08	0.202433	0.0189065
cg26955996	0.1328114	0.0415364	0.424661	0.0006638
cg13490403	1.7496283	0.76174	4.018693	0.1873333
cg20779964	0.2990333	0.1123827	0.795682	0.015619
cg20508508	0.017069	0.0001656	1.759557	0.0852419
cg18090331	0.336796	0.1460315	0.776761	0.0106962
cg11147215	2.4391506	0.620852	9.582727	0.2015335
cg15355713	0.4326315	0.1025509	1.825142	0.2539586

cg20598720	2.0496691	0.4640257	9.053687	0.3436864
cg03437479	0.7231881	0.3912865	1.336619	0.3010725
cg07496902	0.073782	2.22E-06	2451.728	0.6236283
cg08172445	1.1992939	0.454714	3.1631	0.7134142
cg09505809	0.3414093	0.0971661	1.199599	0.0937136
cg00362657	1.4140802	0.7661499	2.609963	0.2678324
cg21320768	1.9218896	0.9363837	3.944601	0.0749457
cg13776918	0.173093	0.0139332	2.150349	0.172448
cg11223573	1.6175931	0.5765657	4.538264	0.3608511
cg06448070	225.62537	0.0044675	11394987	0.3267403
cg22673542	0.7811375	0.4110447	1.484451	0.4508361
cg06462065	1.294824	0.3892063	4.307662	0.6735397
cg12509524	6.01E-63	6.00E-101	6.03E-25	0.0013313
cg20290367	0.3955427	0.1680005	0.931271	0.0337584
cg19216561	0.3244996	0.0922099	1.141959	0.0795708
cg12872686	1.2107094	0.481774	3.042541	0.6842369
cg03052078	0.6135248	0.2797398	1.345581	0.2227692
cg08529852	2.9408236	0.7638384	11.32235	0.1168137
cg14428815	0.3668653	0.1451711	0.927114	0.0340095
cg17082938	0.4798621	0.1781516	1.292538	0.1463946
cg07281647	0.0606821	0.0039776	0.925765	0.0438581
cg07101926	0.3253098	0.1107413	0.955619	0.0410986
cg05356496	7.2257555	1.7809307	29.317	0.0056463
cg06575692	7.6573142	0.6141973	95.46518	0.1138054
cg04994708	0.931818	0.4188095	2.073221	0.8625959
cg23329372	1.5480989	0.6288537	3.811078	0.3417072
cg23484380	1.1074586	0.4888286	2.508987	0.8067542
cg23434459	0.2276389	2.41E-16	2.15E+14	0.9329594
cg10883266	0.9300631	0.0008316	1040.146	0.983849
cg26561413	2.7593618	1.0321339	7.377025	0.0430729
cg03270710	0.2068328	0.0690823	0.619259	0.0048551
cg08432204	0.3392792	0.1497699	0.768582	0.0095743
cg18178197	0.9172861	0.3800075	2.214203	0.847725
cg08069931	0.1776309	0.0004048	77.95589	0.5777502
cg11051095	2.7234957	1.0590418	7.003906	0.0376177
cg16548262	0.8769315	0.4515683	1.702973	0.6981514
cg23944804	0.0010628	6.40E-07	1.765011	0.0703289
cg22062539	3.2367769	0.1483175	70.63714	0.4552308
cg07302910	2.5668232	1.1684935	5.638526	0.0188855
cg01073479	0.3464761	0.0970731	1.236653	0.1025186
cg10536276	0.1449939	0.0504084	0.417058	0.0003406
cg05875463	0.2135677	0.0677611	0.673117	0.0083942
cg05525416	2.0169487	0.4641992	8.763657	0.3492475
cg13615963	1.2478436	0.6213033	2.506205	0.5337384
cg10067737	8.5968115	2.3173602	31.89196	0.0012979
cg10573476	0.1080225	0.0270706	0.431052	0.0016227
cg02042649	6.5108683	0.7833891	54.11284	0.0829161
cg03034759	0.000599	1.15E-06	0.313275	0.0201583
cg01353646	0.1602275	0.0509935	0.503453	0.0017198
cg10231116	0.2733561	0.1007415	0.741736	0.0108786
cg16361168	7.35E-05	3.01E-08	0.179529	0.0167821
cg01420088	17340.689	1.09E-05	2.75E+13	0.3664882
cg00995253	0.3002573	0.0790144	1.140988	0.0773412
cg20577572	0.5337329	0.2635579	1.080866	0.0811648
cg00044107	0.487265	0.2482845	0.956271	0.0366226
cg14621350	5.8665713	1.0938804	31.46291	0.0389535
cg01868791	2.6777135	1.1107066	6.455485	0.0282481
cg07439157	0.3048587	0.1325752	0.701027	0.0051733

cg22792735	0.3760948	0.125955	1.122999	0.0797527
cg19332452	0.1184617	0.0264037	0.531484	0.0053482
cg24216966	2.6631456	0.405713	17.48119	0.3075897
cg14871736	0.1684147	0.0460284	0.616217	0.0071131
cg02616489	0.2835734	0.1235265	0.650985	0.0029548
cg05192440	2.40E+40	24471068	2.35E+73	0.0164437
cg27563968	2.3306188	0.0319986	169.7506	0.6989531
cg24092179	0.4077874	0.1736822	0.957442	0.0394146
cg12086340	2.7098812	0.9801711	7.492015	0.0546859
cg25154630	0.4279944	0.1763189	1.038908	0.0607099
cg14933266	1.283E+12	1.98E-24	8.31E+47	0.5075291
cg18296189	0.5257851	0.1719108	1.608101	0.2597062
cg14620593	0.7720433	0.3626445	1.643623	0.5021767
cg01881549	0.3032257	0.1090919	0.842829	0.0221495
cg25125317	9.26E-10	5.82E-20	14.73195	0.0826492
cg23122650	1.8903011	1.065091	3.354867	0.0295995
cg07560587	0.2154562	0.0526637	0.881468	0.0327212
cg24762962	0.5647071	0.2252166	1.415944	0.2230677
cg02621571	1.25E-13	1.12E-25	0.139308	0.0357965
cg17212502	3.7819985	1.112132	12.86134	0.0331593
cg15600915	0.2638686	0.1204441	0.578083	0.0008698
cg01562897	0.3968616	0.1794417	0.877718	0.0224873
cg16811771	2.1476083	0.3753888	12.28652	0.3903769
cg26090107	1.5733682	0.6347642	3.899854	0.3277792
cg06437740	0.3875632	0.1853319	0.810466	0.0117933
cg09934782	5.2149931	1.2724186	21.37359	0.02175
cg10350263	0.220878	0.0937216	0.520553	0.0005553
cg09279169	0.114967	0.0184233	0.71743	0.0205895
cg20949700	0.3578513	0.1594351	0.803195	0.0127292
cg03463411	0.2295099	0.0807021	0.652707	0.0057803
cg21961852	1.4357522	0.2221473	9.279358	0.7040338
cg12792264	0.4950036	0.2133618	1.148418	0.1014894
cg14822303	1.22E-05	7.46E-09	0.019816	0.0027083
cg23476898	1.5593014	0.5286306	4.599471	0.4208629
cg19930116	0.2450755	0.0767958	0.782101	0.0175453
cg21163617	0.4933512	0.1746384	1.39371	0.1823878
cg00448707	5.3894897	0.2715495	106.9661	0.2692097
cg25885803	0.2372852	0.0476933	1.180549	0.0788822
cg19375810	0.7635142	0.2524735	2.308971	0.632729
cg04992638	2.7610612	0.9010217	8.460905	0.0754785
cg15031983	1.6535066	0.4600171	5.943441	0.4410532
cg20193872	0.4740106	0.117321	1.91514	0.2946967
cg04492927	1.7127739	0.6998877	4.191522	0.2386034
cg06786219	0.0677631	0.0081763	0.561606	0.012607
cg07383757	3.592717	1.2706303	10.15843	0.0158823
cg20080624	2.3988547	0.868927	6.62254	0.0912585
cg09430518	0.6386207	0.3304355	1.234239	0.182222
cg01744822	2.2989504	0.689135	7.669285	0.1756523
cg16166559	2.6334061	1.2823008	5.408113	0.0083593
cg15926557	2.86E-08	5.82E-15	0.140093	0.0271041
cg16155858	0.277948	0.0232261	3.326214	0.3120311
cg07602073	0.4376297	0.1767175	1.083762	0.0740818
cg27517282	0.7232893	0.3649768	1.433372	0.3532611
cg21732915	2.0395588	0.9395947	4.427228	0.0714826
cg15261861	2.0424014	0.8835693	4.721083	0.094837
cg26466801	0.472672	0.2043688	1.093214	0.079836
cg20114739	308267377	956.84666	9.93E+13	0.0025223
cg13054640	1.3584469	0.62163	2.968612	0.4424616

cg27364741	2.6789525	0.9297997	7.718637	0.0679779
cg15820868	0.1238886	0.0204204	0.751622	0.0231852
cg01152073	0.0906427	0.0068257	1.203698	0.0688413
cg11811212	2.290202	1.0481965	5.003857	0.0377089
cg23682934	0.6053727	0.125985	2.908887	0.5308525
cg03554158	0.2427518	3.34E-06	17634.94	0.8042173
cg06200824	0.64133	0.0412631	9.967852	0.750988
cg07429804	1.8532529	0.7812571	4.396179	0.161557
cg21342994	1.6574957	0.0925643	29.67983	0.7313963
cg07869405	1.9857427	0.8629535	4.569393	0.1066748
cg16485048	1.179128	0.6529158	2.129437	0.5848073
cg15703970	0.3733134	0.1867017	0.746447	0.0053176
cg21756326	2.4032794	0.5223259	11.05776	0.2601788
cg11106652	0.3271914	0.1391633	0.76927	0.0104266
cg23429510	2.0699965	0.8291027	5.1681	0.1191139
cg01981760	0.0003336	1.46E-07	0.764383	0.0425573
cg14825976	0.6258989	0.230165	1.702037	0.3586119
cg03512250	13.498036	0.2724993	668.6146	0.191203
cg08492912	0.0583272	0.0060717	0.560311	0.0138247
cg25249068	0.4290244	0.1733825	1.061595	0.0671514
cg04625873	2.5352507	0.7159858	8.977128	0.1492816
cg10082354	0.6716471	0.3152302	1.431049	0.3023982
cg23521069	380321.01	310.26631	4.66E+08	0.0003982
cg11279838	1.0095093	0.4647563	2.192782	0.9809216
cg00206779	2.1754917	0.3464376	13.66123	0.4070232
cg11187806	0.0051843	6.69E-05	0.401757	0.0177487
cg17158414	0.3631266	0.1544096	0.853969	0.020245
cg01699425	6048.1186	0.2977192	1.23E+08	0.0853312
cg05956058	0.3826047	0.1745669	0.838569	0.0164079
cg03448202	2.6728375	0.6932517	10.30515	0.1533281
cg18825531	0.601848	0.2622843	1.381025	0.2308503
cg23588161	3.172E+09	1.83E-18	5.49E+36	0.4941754
cg15900058	1.136028	0.4596778	2.807531	0.7823325
cg14431361	0.1731487	0.03865	0.775691	0.0219091
cg27620176	5895.9206	2.89E-11	1.2E+18	0.6055626
cg19373545	0.6284737	0.3167857	1.246834	0.1839103
cg01902758	1.4208817	0.283561	7.119824	0.6692275
cg00689580	2.816773	0.9556615	8.302323	0.0604177
cg11802899	1.8392592	0.9440465	3.583377	0.0733331
cg16807061	0.2027624	0.0336963	1.220091	0.0813836
cg11093762	0.0433865	0.0014517	1.296714	0.0702853
cg14720207	0.0002333	4.50E-07	0.120956	0.008734
cg15600430	0.162447	0.0374578	0.7045	0.015187
cg18076399	0.2779394	0.062877	1.228595	0.091321
cg24928110	4.9545923	0.8526238	28.79111	0.0746861
cg17786697	3.24776	1.401325	7.527123	0.006019
cg00260883	0.0231307	0.0002645	2.022943	0.0987146
cg22545121	0.5480362	0.2837624	1.058434	0.0733162
cg13327134	0.1319472	0.0300173	0.580001	0.0073394
cg24896460	0.5082958	0.2107418	1.225977	0.1319611
cg09545984	1.3004816	0.5679095	2.978031	0.5342545
cg27015057	0.0091506	5.04E-10	166065.1	0.5820235
cg12176783	0.3990001	0.1488035	1.069875	0.0678878
cg01760983	1.7159934	0.5923171	4.97138	0.3197462
cg26931308	1.51E+22	1.43E-08	1.61E+52	0.1476634
cg01994902	4.2969531	0.9607935	19.21725	0.056439
cg03429569	2.3473316	0.9099712	6.055099	0.0775913
cg19850406	0.1914529	0.0432658	0.847186	0.0293685

cg26002713	7.9163113	1.193849	52.49239	0.0320701
cg00849713	2.1796868	0.7609051	6.243925	0.1467549
cg07233230	0.3092533	0.1440527	0.663907	0.0026055
cg08770761	1.0408057	0.3473347	3.118826	0.9430575
cg18594033	0.5729423	0.2313056	1.419174	0.2287773
cg09983885	0.4532951	0.1588847	1.293242	0.1390852
cg13961449	1.28E-20	3.93E-51	4.2E+10	0.201389
cg12068734	1.3843281	0.5879433	3.259437	0.4566697
cg19788186	0.5067258	0.1545231	1.6617	0.2619206
cg06218523	0.7474511	0.129442	4.31609	0.7448997
cg01442005	1.1815169	0.5544776	2.517653	0.6656451
cg04761077	3.5560373	1.2656882	9.990929	0.0160843
cg17351862	0.4262178	0.1161103	1.56456	0.198674
cg11525285	2.4106916	1.0931696	5.316132	0.0292027
cg22753376	2.79E+32	2.413E+09	3.23E+55	0.0058276
cg23519572	0.3333834	0.1512719	0.734733	0.0064397
cg21759685	0.3854948	0.1363379	1.089985	0.0722586
cg14462779	2.2390923	0.3680933	13.62028	0.381554
cg06260527	0.0925711	0.0191609	0.447235	0.003064
cg08984500	1.8860638	0.5417177	6.566588	0.3188339
cg00210994	2.0582964	0.9345425	4.533324	0.0731462
cg17505469	2.4910155	1.0382513	5.976548	0.0409503
cg14462265	0.6284782	0.3061025	1.290368	0.2057241
cg20665464	0.4299442	0.218048	0.847758	0.0148201
cg11577716	2.7088115	1.1838374	6.198199	0.0182966
cg13630043	1.5137119	0.6861424	3.339429	0.3044598
cg00792022	3.384296	0.3690758	31.03281	0.2808842
cg15864906	1189.4468	0.2190065	6460009	0.106559
cg06482783	0.6901734	0.3644323	1.307072	0.255088
cg07257824	1.7398887	0.6771659	4.470415	0.2500302
cg21055554	0.6153413	0.0802934	4.715767	0.6402627
cg13541788	1.8402849	0.3995975	8.475149	0.4337769
cg07883762	67.311174	2.2468672	2016.494	0.0152386
cg07450086	2.6871749	0.5897018	12.24502	0.2014461
cg18896156	3.1143978	0.9435549	10.27971	0.0622372
cg06993703	0.8971243	0.4003489	2.010326	0.7920044
cg09294084	1.8189885	0.8191927	4.039	0.1415736
cg05903736	0.6439869	0.2867923	1.446061	0.2862972
cg21477717	0.1168214	0.0251603	0.542412	0.0061279
cg20276114	0.2208187	3.56E-05	1368.256	0.7345832
cg05249393	1.2918386	0.7209762	2.314705	0.3894909
cg26986731	1.8722964	0.7241873	4.84059	0.195633
cg14846981	2.366672	1.0145843	5.520622	0.0462102
cg00364016	2.740884	0.9816224	7.653091	0.0542845
cg10182321	4.51E-12	5.58E-21	0.003648	0.012547
cg18943866	0.4422201	0.1993656	0.980904	0.0447073
cg21606287	0.2640248	0.113525	0.614042	0.001985
cg07051718	3.005E+13	7.84E-16	1.15E+42	0.3553953
cg14452720	1.216E+10	1257.8741	1.18E+17	0.0046597
cg05293646	0.7553464	0.379808	1.502202	0.4237817
cg22900681	1.1844875	0.5354157	2.620414	0.6760017
cg14005025	0.7240977	0.3633801	1.44289	0.3587758
cg16276348	2.3646924	0.7718612	7.244528	0.1319013
cg23171102	1.61386	0.4463218	5.835574	0.465488
cg24725201	0.1376712	0.036139	0.524458	0.0036641
cg02475416	2.68E-20	1.83E-35	3.92E-05	0.0114226
cg16005939	2.7291474	0.9994348	7.452458	0.0501291
cg16535035	4.3577503	1.1931231	15.9162	0.0259387

cg12180703	0.1742931	0.0335943	0.904263	0.037547
cg07805952	0.1489639	0.0369968	0.599789	0.0073785
cg03110921	1.5455105	0.3924865	6.085822	0.5335769
cg01062504	0.7207224	0.2747522	1.890579	0.5056693
cg13734410	4.52E-07	2.93E-13	0.699629	0.0445276
cg12458866	20847.125	2.82E-17	1.54E+25	0.6850088
cg00113663	1.2690205	0.5024934	3.204844	0.6142329
cg12091542	0.0059255	1.18E-05	2.985655	0.1062193
cg06962748	1.3644358	0.4787543	3.888602	0.5608835
cg17284334	2.6897982	1.1573935	6.251127	0.0214658
cg18542639	0.3198111	0.1435409	0.712543	0.0052849
cg11787791	1.4981672	0.6184087	3.629485	0.3705686
cg15921271	73.134337	1.924416	2779.353	0.0207407
cg25928986	1.0661233	0.5721653	1.986522	0.8401951
cg15726813	2.7150391	1.0574561	6.970916	0.037886
cg11378575	0.149376	0.047657	0.468204	0.0011069
cg19258425	1.523247	0.7664143	3.027451	0.2298073
cg03861703	0.1399101	0.0348159	0.562238	0.0055821
cg16884847	27.156303	0.0583448	12639.76	0.2921573
cg09233651	1.5457279	0.8084804	2.955266	0.1878309
cg16033723	0.4173977	0.2079276	0.837892	0.0139939
cg10548978	2.8618325	1.1555072	7.087871	0.0230666
cg23962380	66.716632	0.0306316	145310.9	0.2841201
cg01318265	0.0554319	0.000795	3.865077	0.1816547
cg26017506	0.8004146	0.3703457	1.729907	0.571283
cg09217748	641.4167	4.12E-06	9.99E+10	0.5018556
cg08090407	0.5912175	0.2166718	1.613215	0.3047968
cg19360930	0.3919375	0.1782231	0.861925	0.0198322
cg12383699	2.3425721	0.6852407	8.008345	0.1746915
cg01378667	2.0848757	0.5497028	7.907376	0.280052
cg12238015	1.0517734	0.4488935	2.464342	0.9074975
cg18464364	1.4002042	0.2038589	9.617296	0.7320603
cg07694985	6.5978633	0.9834521	44.26428	0.0520425
cg18181201	1.8918726	0.860847	4.157745	0.1125138
cg18382389	2.0053837	0.7864773	5.113388	0.1451101
cg03147470	0.2001308	0.0834881	0.479737	0.0003102
cg14259556	4.0579817	0.8683843	18.96305	0.0749828
cg26600461	0.3236238	0.1290519	0.811552	0.0161676
cg15571330	0.3607798	0.1120778	1.161354	0.0874176
cg16441238	2.4035251	0.9340867	6.184579	0.0689783
cg20994083	7.00E-07	2.52E-15	194.1478	0.1530605
cg00584450	1.620624	0.819998	3.202962	0.1648255
cg13913666	0.5288979	0.2335865	1.197556	0.1266125
cg15994861	1.0428986	0.3319196	3.27681	0.9426743
cg26373134	0.0707623	0.0093261	0.536913	0.0104233
cg05687834	2.5589656	1.099402	5.956242	0.0292711
cg00981928	0.1891432	0.0344417	1.038714	0.0553327
cg13318129	0.9908842	0.2502194	3.923962	0.9895946
cg05191891	0.7772323	0.2860271	2.112003	0.6212257
cg26248114	0.5664529	0.2667351	1.20295	0.1391131
cg09540676	0.0999624	0.0135445	0.737752	0.0239332
cg06603923	0.5827643	0.2604737	1.303833	0.188768
cg10501093	0.8949709	0.4446193	1.80148	0.7558892
cg07517739	0.3611037	0.1472385	0.88561	0.0260568
cg16696462	2.3071949	0.9168151	5.806131	0.0758124
cg02914427	1.4023932	0.5828749	3.374149	0.450277
cg18613421	3.1400208	1.4638518	6.73547	0.0032965
cg26079571	2.48E-29	3.47E-60	177.0124	0.0691924

cg02382016	2.3792223	0.7866718	7.195757	0.1247764
cg17178922	0.6544122	0.3314899	1.29191	0.2217472
cg00020474	0.0975717	0.0249556	0.381486	0.0008222
cg13104187	0.8646198	0.4250732	1.758679	0.6880203
cg15803845	1.3677206	0.5772592	3.240589	0.4767691
cg05154234	3.4143739	0.4984437	23.3887	0.2110147
cg19332832	4.7664994	0.4795688	47.37489	0.1826039
cg24699005	1.6825469	0.6718726	4.213543	0.2666183
cg00770316	0.1843039	0.062227	0.545871	0.0022678
cg11812625	2.3459538	0.5425135	10.14445	0.2537124
cg25805189	2980.6778	3.54E-12	2.51E+18	0.6482163
cg22595920	2.5336577	0.5190836	12.36683	0.2504166
cg12502929	0.1914566	0.0596292	0.614727	0.0054778
cg01798341	7.17396	0.3118064	165.0566	0.218106
cg11156286	1.746E+15	21.87297	1.39E+29	0.0316449
cg06568724	4.2570288	1.356342	13.36115	0.0130556
cg03455255	6.5438082	0.0553941	773.0322	0.4403628
cg04880412	0.1433696	0.0462153	0.444763	0.000772
cg09614653	2.7419625	1.0454235	7.191687	0.0403393
cg00994629	2.0643642	0.8459745	5.037503	0.1112787
cg17390301	3.3867656	1.6466607	6.965722	0.0009147
cg01896341	1.4689103	0.6927607	3.114636	0.3159888
cg15477040	0.4529921	0.2363441	0.868234	0.0170499
cg02943578	0.2004995	0.0517656	0.776578	0.0200206
cg16379462	0.8767002	0.2358488	3.258881	0.8442709
cg13700073	0.1758376	0.0553784	0.558319	0.0031915
cg05982017	1.5934632	0.1829827	13.87631	0.673078
cg00251405	0.2048938	0.0508769	0.825158	0.0257244
cg10717691	3.941616	1.1562439	13.4369	0.0283813
cg23559636	0.8779525	0.3302743	2.33382	0.7941377
cg24594658	0.3185256	0.1448667	0.700358	0.0044278
cg22165105	0.8125048	0.4247205	1.554349	0.5304317
cg12937183	6.5436871	1.5046618	28.45812	0.0122543
cg03905144	0.9781949	0.2265062	4.224456	0.9764367
cg26461417	1.2901243	0.7049697	2.360982	0.4087155
cg24950894	0.1175359	0.0146363	0.943864	0.0439761
cg02616947	0.976941	0.5304347	1.799305	0.9403197
cg02068356	0.8149007	0.3759845	1.766198	0.6040067
cg08313393	0.6401483	0.2912374	1.407065	0.2669664
cg14811319	3.5751779	1.0698636	11.94722	0.0384833
cg11742472	5.0800147	0.5814844	44.38046	0.1416425
cg01998750	1.1579158	0.587958	2.280382	0.6715451
cg12619509	0.3825334	0.1790322	0.817349	0.0131157
cg25216910	0.1715199	0.0087249	3.371851	0.2459879
cg18714224	1.0305874	0.5409066	1.963574	0.9270125
cg07313589	2.05E-07	1.19E-13	0.352182	0.035515
cg11838898	2.2010702	0.9425233	5.140149	0.0682775
cg07373877	0.5890816	0.256041	1.355318	0.2132082
cg15172772	7.86E-07	9.49E-13	0.650773	0.043202
cg14873958	2.3118604	0.8506911	6.282772	0.1003934
cg14735704	2.2469538	0.6367808	7.928634	0.2082427
cg14811105	1.4312338	0.4447733	4.60556	0.547661
cg11733057	58.427171	0.7372314	4630.479	0.0682552
cg08447254	3.10753	0.9008895	10.71912	0.0726934
cg08732750	1.3861944	0.2984507	6.438367	0.6768414
cg06690609	0.0311319	0.0061572	0.157408	2.72E-05
cg01602001	0.4426413	0.1625081	1.205671	0.1109086
cg04637506	14.088208	0.8107292	244.8137	0.0693812

cg26059839	2.6152427	0.8219153	8.321411	0.1035513
cg20979233	1.8217433	0.9553977	3.473683	0.0685452
cg06850312	3.40E+23	3.275E+09	3.53E+37	0.0010001
cg20281962	1.618953	0.7106938	3.687958	0.251405
cg16501033	0.2122152	0.0719335	0.626068	0.0049795
cg09827833	1.4245445	0.6613221	3.068591	0.3661072
cg07513723	1.088774	0.4671093	2.537798	0.8438373
cg11238366	181.15238	4.8587566	6754.03	0.0048598
cg27256565	7.0997061	0.9647233	52.249	0.0542672
cg00440980	4.4321563	0.675978	29.06013	0.1207059
cg23541926	0.297343	0.1115047	0.792907	0.0153648
cg00415971	0.1514461	0.0176367	1.300461	0.0853428
cg05205528	168.16612	1.1556077	24471.84	0.043708
cg08770034	1.3826564	0.6160325	3.103308	0.4321646
cg11410718	0.759277	0.289309	1.992684	0.5758869
cg06375761	0.9661778	0.426988	2.186243	0.9341824
cg10004780	2.3592986	1.0045358	5.541156	0.048798
cg23627948	2.76193	0.8416537	9.063416	0.0938095
cg08264906	0.5212669	0.2119181	1.282189	0.1559915
cg06468780	1.2363194	0.325315	4.698479	0.7554772
cg07777793	2.3533681	0.9230737	5.999891	0.0730801
cg08732611	9589425.5	2.10E-07	4.38E+20	0.3164563
cg14972625	0.6909406	0.2910582	1.640218	0.4019497
cg16988194	0.3149148	0.1282789	0.773092	0.0116819
cg02378847	0.0802834	0.0086602	0.744256	0.026423
cg13937449	8.237E+18	3095300.8	2.19E+31	0.0028467
cg07739826	0.1199495	0.0139043	1.034777	0.0537469
cg25438801	0.4822301	0.1914422	1.214706	0.1217864
cg26175815	1.6643736	0.2947187	9.399264	0.5640921
cg20991819	1.8527546	0.6016818	5.705174	0.2825319
cg22488367	0.2474692	0.0966663	0.63353	0.0035951
cg16143578	1.5216696	0.5940258	3.897942	0.3817195
cg16848937	0.7226168	0.3653893	1.429092	0.3504276
cg21482122	0.0008643	3.86E-06	0.193715	0.0106381
cg11111460	2.0440139	1.0805325	3.866605	0.0279415
cg16238918	0.1769085	0.0293626	1.065867	0.0587114
cg16356516	0.2613113	0.0361836	1.887144	0.1833845
cg03812676	1.4422982	0.3775708	5.509494	0.5922437
cg17947172	1.6766701	0.4476854	6.279461	0.4430254
cg11143063	1.7857729	0.572066	5.574505	0.3181038
cg01141269	6.8177886	0.9847593	47.20163	0.0518464
cg06628473	5.9820342	0.7172566	49.89112	0.0983541
cg26918305	319.1312	1.3054353	78015.91	0.0398826
cg08683249	0.6522398	0.3434816	1.238543	0.1915171
cg11762213	0.9127969	0.2839299	2.934521	0.8782906
cg26711184	2.6856522	0.8993295	8.020117	0.076748
cg06896281	0.2315158	0.0170743	3.139187	0.2713556
cg25283841	0.5752667	0.2741112	1.207291	0.1437688
cg25772723	1.5467855	0.6698816	3.571594	0.3069781
cg27072218	1.6398743	0.7578299	3.548538	0.2091582
cg13382703	12.083052	1.180421	123.6848	0.035752
cg11024728	0.2291805	0.0694487	0.756295	0.0155843
cg19307543	1.7090835	0.7483732	3.903088	0.2033621
cg07657658	0.4577151	0.2258059	0.927802	0.0301714
cg22264616	0.627087	0.3157347	1.24547	0.1825439
cg09395718	2.3610684	0.3074123	18.1341	0.4088365
cg08241477	3.3456711	1.1084738	10.09813	0.0321387
cg25717917	0.3860146	0.1905784	0.781868	0.0082109

cg00961640	0.4711769	0.1861921	1.192358	0.1121575
cg02767242	2.5148599	1.0350067	6.110608	0.041758
cg01611622	2.2921866	0.9078894	5.78718	0.0791802
cg04811502	2259.3375	69.553218	73391.37	1.37E-05
cg11229610	52.136763	0.5517449	4926.628	0.0884332
cg09209669	0.8204732	0.3465087	1.94274	0.6527624
cg22698272	2.1048415	1.0635053	4.165807	0.0326196
cg24713204	0.1338219	0.0397718	0.450276	0.0011588
cg04803153	0.2038111	0.0896673	0.463256	0.0001466
cg12584394	0.4580101	0.2058476	1.019071	0.0556627
cg24360909	0.3514508	0.1056279	1.169365	0.0882184
cg14783074	0.2684516	0.0071021	10.14714	0.4779428
cg17494838	1.4735017	0.5977835	3.632096	0.3997024
cg23400942	2.0821015	0.9339006	4.641979	0.0730063
cg15230164	0.5609753	0.304115	1.034784	0.0642403
cg10005098	6.994E+09	8.10E-13	6.04E+31	0.3790676
cg04667919	3954.7965	1.76E-24	8.91E+30	0.7965967
cg16745033	0.1949893	0.0307981	1.23452	0.0825261
cg04712361	0.0294639	0.0007186	1.207991	0.0628524
cg20468939	1.0944581	0.4832535	2.478696	0.8286722
cg14467066	0.7380101	0.0282128	19.30537	0.8552577
cg20701531	0.4659628	0.1203342	1.804319	0.2689228
cg23008973	1.969175	0.2757954	14.05988	0.4992732
cg26147554	0.033464	0.000886	1.263881	0.0667179
cg18878351	0.9454156	0.331401	2.697067	0.916419
cg04947084	0.2179823	0.0039945	11.89539	0.4553544
cg25601889	2.6181869	0.8138847	8.422449	0.1064156
cg16134686	1.7473342	0.8406235	3.632038	0.1349347
cg04071964	1.5967814	0.7287206	3.498887	0.2422928
cg10648960	0.2617448	0.0958847	0.714507	0.0088951
cg12477370	2.1206126	1.07578	4.18022	0.0299372
cg20027133	2.2184817	0.8088041	6.085109	0.121675
cg00906147	8509337.4	1.42E-05	5.11E+18	0.2488561
cg14684749	0.5975863	0.3087104	1.156778	0.1265637
cg19940077	0.8425149	0.2786816	2.547106	0.761441
cg06847670	1.9033729	0.1767805	20.49337	0.5955415
cg01912298	0.3077984	0.1414656	0.669702	0.0029705
cg03619352	0.9157224	0.4262208	1.967402	0.8214818
cg13410390	0.5981745	0.2405539	1.487453	0.2688812
cg04343045	0.6741967	0.3017268	1.506466	0.3365277
cg14817049	1.8758872	0.4709321	7.472315	0.3723449
cg03122453	0.7835482	0.0431039	14.24344	0.8690679
cg03417975	40.01247	0.1568663	10206.13	0.191957
cg24282516	0.2665883	0.055052	1.290949	0.1004543
cg17284854	7.8174404	0.3881113	157.461	0.1795311
cg04015912	0.5636732	0.2595985	1.223919	0.1472857
cg01660473	1.0168769	0.4766415	2.169426	0.9654703
cg00816240	0.1786329	0.0569173	0.560633	0.003161
cg05318142	0.1666902	0.0121465	2.287535	0.1800068
cg16797275	3.4492881	1.0067484	11.81784	0.0487618
cg26070333	0.5289483	0.2175752	1.285929	0.159986
cg07279638	0.1891692	0.0241209	1.483569	0.1130598
cg23184518	0.6528067	0.3162003	1.347742	0.2488773
cg24504307	0.0158991	0.002337	0.108164	2.30E-05
cg13114145	1.1439771	0.438516	2.984346	0.7833584
cg02524863	1.0603553	0.5493036	2.04687	0.861363
cg16646726	1.3655331	0.4043236	4.611853	0.6158754
cg14009912	0.4027535	0.1992446	0.814127	0.0113207

cg04962479	2.1060183	0.8443702	5.252806	0.1102218
cg05238276	0.3083835	0.1339235	0.71011	0.0057026
cg04947578	359.8749	3.87E-20	3.35E+24	0.8196064
cg26275647	5.7756669	0.9353477	35.66409	0.0590251
cg04422019	1.5549848	0.7488988	3.228711	0.2363013
cg08722383	0.2016127	0.0672701	0.604246	0.0042429
cg04955791	0.5038947	0.248096	1.023434	0.0579747
cg18377680	0.0032956	3.41E-06	3.183346	0.1031516
cg00466425	0.2385432	0.0492327	1.155795	0.075055
cg10452974	0.8697458	0.278877	2.712514	0.8099635
cg04303033	0.2069826	0.0605709	0.7073	0.0119943
cg13319197	6.05E-08	3.21E-14	0.113942	0.0241578
cg00781519	0.5767604	0.1734126	1.918272	0.3694299
cg14346208	1.9901321	0.8509294	4.65447	0.1123818
cg27183818	1.3791428	0.6979112	2.725325	0.3549559
cg10500461	1317570.1	1.67E-05	1.04E+17	0.2710647
cg09943401	0.1243768	0.0231108	0.669367	0.0152057
cg04862622	1.0300606	0.0295604	35.89351	0.986957
cg06088282	0.0743875	0.0110378	0.501323	0.0076012
cg24179027	0.1091628	0.0367187	0.324535	6.77E-05
cg16089796	3.4307427	1.0655474	11.04596	0.0387922
cg19476426	0.4909266	0.2269947	1.061738	0.0706461
cg02282200	77.658629	1.0788211	5590.234	0.0460717
cg22998476	0.2198979	0.025546	1.892865	0.1678955
cg12597983	2.3345384	0.9644417	5.65101	0.0601501
cg09234164	0.359556	0.1485383	0.870351	0.0233399
cg18345406	0.6005293	0.2714005	1.328794	0.2082334
cg27475923	0.4537689	0.2140106	0.962131	0.0393375
cg18206040	7.35E-06	2.45E-26	2.21E+15	0.6231842
cg02183671	1.5426193	0.7280723	3.268459	0.257824
cg08907257	0.089759	0.0079989	1.00722	0.0506856
cg01186919	1334.2917	0.0110194	1.62E+08	0.2281842
cg25320780	0.4187602	0.1507665	1.163123	0.0949099
cg00291366	0.0733615	0.0117237	0.459061	0.0052366
cg10663078	3.3617913	1.0267639	11.00705	0.0451119
cg04343794	1.2870559	0.6488628	2.552948	0.470187
cg04148285	0.1088357	0.0208228	0.568858	0.0085755
cg17563773	0.2395773	0.0758135	0.757085	0.0149333
cg22517740	0.2281357	0.0614816	0.846528	0.0271737
cg25656762	0.2775684	0.1005306	0.766376	0.013381
cg00060956	0.3818117	0.0476145	3.061676	0.3646802
cg05003890	2.2723079	0.9348642	5.523137	0.0700901
cg07653363	417.68654	0.04527	3853813	0.1951423
cg16017089	0.6232927	0.3493904	1.111919	0.109434
cg18037834	0.1788272	0.0498181	0.641919	0.0082959
cg07983955	1.43E-08	1.62E-15	0.12534	0.0267927
cg06563229	1.4440822	0.6685773	3.119121	0.3496454
cg00146612	5.3403158	0.5086549	56.06742	0.1625705
cg18766054	1.9676084	0.7750594	4.995079	0.1544797
cg06993761	0.9782621	0.373775	2.560355	0.9642898
cg08083988	3.2743124	1.0818556	9.909938	0.0357984
cg18739166	0.229107	0.0867139	0.605324	0.0029526
cg27318774	0.3185061	0.123219	0.8233	0.0182138
cg06191034	0.6900155	0.3304474	1.440839	0.3232884
cg07755752	0.674489	0.2910481	1.563094	0.3584413
cg20743744	0.3103201	0.10557	0.912178	0.0334157
cg10553672	0.4299692	0.0003537	522.6822	0.8158392
cg12124478	0.0026847	1.35E-05	0.532029	0.028249

cg05620814	5.15E+36	3829871.6	6.92E+66	0.0169301
cg07224067	0.2160661	0.0587187	0.795054	0.0211672
cg11811131	95.838388	0.403594	22758.01	0.1020801
cg15026998	1.6961331	0.6777669	4.244626	0.2589374
cg23881204	9.08E-19	2.05E-28	4.03E-09	0.000247
cg12242345	0.9001976	0.2491245	3.252814	0.8725581
cg05404236	0.1599555	0.0368594	0.694144	0.0143868
cg02075590	0.3786185	0.186589	0.768277	0.0071431
cg19935945	1.7512889	0.9450122	3.245474	0.0750306
cg02456804	0.7230394	0.209769	2.492198	0.6075079
cg01422797	3.7883122	1.6470495	8.713344	0.0017237
cg26504021	1.1276948	0.557687	2.280304	0.7379945
cg02860797	0.049705	0.0078844	0.313353	0.0013972
cg09676376	4.1027026	0.5287203	31.83568	0.1769058
cg25543316	5.971E+09	0.0004274	8.34E+22	0.1449448
cg17272224	1.582652	0.5495344	4.558018	0.3949545
cg02949067	0.951106	0.3230997	2.799763	0.9274904
cg24942919	0.3759828	0.161847	0.873436	0.0229286
cg01815567	2.5978369	0.9000543	7.498166	0.0775204
cg04086391	0.8311944	0.3462237	1.995485	0.6790342
cg27220097	13.105351	0.933287	184.0272	0.0562948
cg02287007	2.1913903	0.9528398	5.039873	0.0648519
cg10498429	0.0022089	4.79E-06	1.018706	0.0506963
cg26135172	0.3626147	0.1286311	1.022221	0.0550602
cg22906574	2.7452764	0.1673688	45.02954	0.4792236
cg09232225	0.3003105	0.1164761	0.774291	0.0127986
cg18832407	1.0461046	0.4492848	2.435727	0.9167521
cg10634669	10.968664	0.047117	2553.466	0.3890758
cg11897736	1.8880533	0.8502151	4.192757	0.118446
cg05111155	11.828124	0.1223109	1143.843	0.2895346
cg11939075	53.008437	1.534871	1830.704	0.0280173
cg26231241	0.092174	9.04E-11	93936022	0.8217651
cg00613284	0.2724231	0.073288	1.012639	0.0522323
cg25954914	2.1430832	0.8806598	5.215187	0.0929791
cg13572289	4.345E+09	0.0006588	2.87E+22	0.1405952
cg27341128	0.0447848	0.0025882	0.774929	0.0327396
cg01437411	1.7206421	0.422994	6.999177	0.4483991
cg01913259	1.908861	0.8450686	4.31178	0.1199323
cg00512404	1.1303992	0.529293	2.414168	0.7515437
cg25112312	0.9729917	0.4649743	2.036054	0.9420642
cg07177174	0.6303564	0.2863274	1.387744	0.2517433
cg15568892	2.3724093	0.4488122	12.54049	0.3091929
cg12741639	1.5826576	0.6465955	3.873836	0.3147814
cg16264966	0.0942824	0.0183723	0.483837	0.0046544
cg14218343	0.0141975	0.0010959	0.183937	0.001132
cg08369368	1.2443677	0.5279235	2.933097	0.6172505
cg18744021	2.2216843	0.9260555	5.330005	0.0737913
cg01298102	0.211482	0.0630559	0.709285	0.0118591
cg16215817	0.2698903	0.089516	0.813718	0.020015
cg01860370	1.6736573	0.6980679	4.012688	0.2483652
cg09383424	1.1133217	0.4220351	2.936924	0.8282846
cg00520380	0.3034554	0.0842893	1.09249	0.0680588
cg26170014	0.4490873	0.1897196	1.063039	0.0686202
cg00381755	3.3043723	0.9032104	12.08896	0.0708973
cg04296178	2.0523132	0.6876269	6.1254	0.1975064
cg01399477	9.252E+17	4.40E-26	1.95E+61	0.4163291
cg18998590	0.7549898	0.2382389	2.392596	0.6329524
cg18950940	2.8361737	0.3271024	24.59133	0.3441782

cg05872614	4.1036996	1.3292718	12.66885	0.0140942
cg11835619	0.2631147	0.0962748	0.719081	0.0092449
cg07140797	0.4261039	0.2196142	0.826743	0.0116499
cg14112958	0.1705648	0.0363286	0.800812	0.0249951
cg00510991	1.1932888	0.1213641	11.73278	0.8795565
cg10072237	2.0176304	0.9360838	4.34879	0.0732298
cg13588354	0.0252374	0.0014474	0.440055	0.0116431
cg16291276	1.4888964	0.5797541	3.823711	0.4081653
cg25140783	2.1558902	0.8494798	5.471422	0.1059517
cg12292060	1.6503473	0.3561138	7.648247	0.5219691
cg21814801	3.5547619	1.1003756	11.48365	0.0340197
cg04652957	2.2292341	0.8740822	5.685374	0.0933029
cg23055735	1.3834486	0.6520834	2.9351	0.3976754
cg21278103	0.4407647	0.1546934	1.255861	0.1251495
cg13299325	6.7213916	1.9026442	23.74438	0.0030872
cg17208360	0.3869282	0.1317836	1.136055	0.084017
cg13924974	0.4083119	0.1027113	1.623177	0.2033505
cg01208318	0.3615785	0.1239521	1.054754	0.0625508
cg00737841	0.4404509	0.1823779	1.063709	0.0683527
cg10755077	0.177795	0.0375623	0.841563	0.0294483
cg15844596	2.3208837	0.8566971	6.287521	0.0977651
cg10111831	0.1663459	0.0387512	0.714066	0.0158205
cg19436567	4.692E+11	3.35E-05	6.58E+27	0.1565647
cg21634842	3.68E-14	1.96E-24	0.00069	0.0103753
cg20131013	0.2422161	0.0123082	4.766616	0.3509672
cg26581503	1.6818533	0.4960986	5.701751	0.4039272
cg20554353	0.1750147	0.055835	0.548583	0.0027897
cg00415993	1.0159081	0.438698	2.352573	0.9706142
cg10997479	3.3886398	0.9863857	11.64137	0.0525995
cg20710709	2.8788492	1.1818997	7.012247	0.0199178
cg11065271	2.0896567	0.7680716	5.685232	0.1489544
cg17370718	3.4302355	1.2388225	9.498145	0.0176872
cg02100674	1.0671783	0.438068	2.599755	0.8861962
cg23671626	22.130122	0.2571486	1904.511	0.1730474
cg13594244	321.04151	5.581662	18465.41	0.0052437
cg15087147	0.534848	0.2154802	1.327558	0.1773024
cg06527050	4.4714022	0.7731401	25.86004	0.0944022
cg24414127	3.473835	0.7978005	15.126	0.0971137
cg17050097	0.2861481	0.0948965	0.862843	0.0262879
cg09874127	3.711422	1.0102409	13.63502	0.0482329
cg03570045	1.5807314	0.6550683	3.81443	0.3083093
cg05406749	0.5748999	0.2991661	1.104771	0.0967147
cg25660566	2.6908653	1.2931524	5.599306	0.0081069
cg05730301	1620.3258	3.2847125	799295.4	0.0194988
cg01451057	0.0122071	2.74E-07	544.0496	0.4198637
cg15224291	0.3093577	0.1381495	0.692744	0.0043383
cg19455642	1.6645916	0.6760308	4.098726	0.2676973
cg12149606	0.6545417	0.2614777	1.638476	0.3653174
cg09824558	1.104147	0.3448056	3.535734	0.8674936
cg10996596	3.096241	1.121806	8.545781	0.02912
cg19460508	0.8427976	0.3802642	1.867932	0.6736152
cg06201207	0.2322006	0.047216	1.141925	0.0723892
cg15174951	1.0577594	0.4987333	2.243393	0.8836174
cg14313868	249.62781	2.4986778	24938.81	0.0187838
cg06442489	0.4429457	0.1977264	0.992285	0.0478402
cg00593773	1.2885543	0.5128677	3.237428	0.589637
cg00861411	10.814616	5.77E-07	2.03E+08	0.7805004
cg23462956	2.6860516	1.2678678	5.690557	0.0098919

cg00604935	2.0216175	0.52921	7.722714	0.3033118
cg25325209	2.5415122	0.9819259	6.578179	0.0545588
cg26146855	2.2922119	0.7516822	6.989969	0.1447877
cg18468354	1.7434674	0.7611143	3.993722	0.1886875
cg25613180	2.8092347	0.8587974	9.189361	0.0875955
cg24290574	1.8426702	0.8638595	3.930539	0.1138004
cg27074174	2.1672913	0.9852656	4.767396	0.0544728
cg18268547	3.2423053	0.4959445	21.19702	0.2194845
cg06705997	1.6659721	0.5062584	5.482305	0.4009815
cg15785898	0.2949989	0.0726637	1.197632	0.0876943
cg02930239	6.2099291	0.985079	39.14733	0.0519002
cg20543645	1.3584189	0.4787165	3.854687	0.5648554
cg16606638	0.0852178	0.0119236	0.609051	0.0141229
cg02399098	0.1860334	0.0574026	0.602907	0.0050569
cg06525453	0.2591111	0.1257189	0.534037	0.0002522
cg13851989	0.4972278	0.2171039	1.138789	0.0984179
cg12408494	0.5856988	0.2453479	1.39819	0.2282133
cg09686635	137.69336	2.0346669	9318.214	0.0220045
cg14508832	2.6801388	0.9838257	7.301237	0.053846
cg11863058	0.2705756	0.1305612	0.560742	0.0004383
cg03812107	1.3826796	0.6784076	2.818074	0.3724358
cg03699904	2.9686353	0.9504898	9.271846	0.0611268
cg18101022	0.366278	0.1108263	1.21054	0.0996194
cg05035248	0.2401672	0.0641972	0.898486	0.034092
cg25673241	1.1165476	0.3541954	3.519749	0.8507282
cg01297806	2.5965379	0.3811259	17.68972	0.3297356
cg16223546	0.232318	0.0689698	0.78254	0.0184876
cg07316943	0.4021165	0.1668903	0.968886	0.0423148
cg03368634	0.3727905	0.1886386	0.736714	0.0045236
cg17095147	2.4872904	0.6594621	9.381303	0.1785312
cg06458171	3.8968096	0.9683298	15.68177	0.0555357
cg20147515	14.379846	0.9531591	216.9417	0.0541898
cg11819369	0.6438683	0.2931011	1.414414	0.2728734
cg09219734	0.4256789	0.2065746	0.877177	0.0206018
cg26956007	0.4093884	0.1646776	1.017739	0.0545906
cg08892899	1.6050022	0.6292704	4.09368	0.3219903
cg07949165	1.35E-12	5.29E-29	34669.59	0.1562867
cg13539545	2.0042083	1.0204135	3.936494	0.043525
cg10228500	2.232825	0.9615389	5.184925	0.0616615
cg12176793	0.3976751	0.1238779	1.276624	0.1212451
cg02680159	0.473726	0.1380776	1.625292	0.2349108
cg04285549	1.4435884	0.5857582	3.557693	0.4250087
cg00851732	0.0781336	0.0192813	0.31662	0.0003558
cg25133533	0.1015037	0.0140545	0.733075	0.0233432
cg24371990	0.2479666	0.0665531	0.923885	0.0377155
cg05884711	1.4946206	0.325362	6.865862	0.605435
cg00190674	171.00389	0.0755411	387104.8	0.1920382
cg11610350	2.224573	0.7319835	6.760706	0.1585886
cg24753760	2.9583166	1.3519213	6.473481	0.0066348
cg22575379	3.5879835	0.6092873	21.12899	0.1578708
cg02257953	2.4786783	1.2078181	5.086731	0.0133336
cg06934774	2.2669842	0.9689259	5.304036	0.0591366
cg26078407	4.6215547	0.5114608	41.76033	0.1728938
cg12599765	3.7715203	1.09634	12.97441	0.0352153
cg12962078	1.0288054	0.5585233	1.895069	0.9273995
cg11097968	0.3063613	0.0984173	0.953666	0.0411666
cg17850055	8.1385922	1.6237739	40.79182	0.0107907
cg18127481	0.7835461	0.3965066	1.548384	0.4827461

cg13232118	0.9281616	0.4380348	1.966702	0.8457191
cg10318678	1.0879344	0.1381783	8.565754	0.9361956
cg12255501	0.3711771	0.1490993	0.924031	0.0331922
cg02281208	0.0656298	0.0099518	0.432814	0.004653
cg04496042	0.3681787	0.1430508	0.947604	0.0383082
cg01577646	6.496604	2.0251391	20.84097	0.0016526
cg27492839	0.0138262	0.00034	0.562237	0.0235402
cg08928958	0.2819685	0.0610964	1.301325	0.1047136
cg17886546	2.2501426	0.6943285	7.292142	0.1764211
cg17468841	5.845E+15	1.41E-09	2.43E+40	0.2093973
cg09853238	4.8541884	1.502244	15.6853	0.0082901
cg12243007	0.0485545	0.0089637	0.263009	0.0004492
cg03448301	2.2043878	0.6007007	8.089429	0.2334048
cg27529871	1.0256538	0.5543928	1.897509	0.9356822
cg18085517	0.3367806	0.1359876	0.834055	0.0186661
cg17071957	0.390848	0.16823	0.908056	0.0289467
cg02058624	2.2979754	1.1271655	4.68493	0.0220598
cg06001716	0.5248443	0.24268	1.135081	0.101418
cg16429927	1.2270889	0.5844872	2.576185	0.588642
cg03250019	0.2931323	0.0662153	1.297684	0.1059506
cg07688931	1.5575654	0.6097976	3.978385	0.3543637
cg25541666	0.3902908	0.1133389	1.343994	0.1358718
cg15527643	0.1310874	0.0301219	0.570479	0.0067688
cg01010923	0.1778996	0.0394124	0.803003	0.0247502
cg16384355	0.4492367	0.1393774	1.447965	0.1802218
cg13220123	0.5796871	0.2149855	1.563069	0.2812966
cg22981961	4.6207149	1.5552868	13.72802	0.0058703
cg16472834	1.5040826	0.6405819	3.531577	0.3486156
cg04404489	1.3252169	0.6866852	2.557504	0.4012355
cg22296149	0.149446	0.0444695	0.502235	0.0021153
cg07201717	0.3139255	0.1217946	0.809143	0.0164687
cg13022763	3.3454309	0.8758215	12.77875	0.077387
cg11220502	1.760898	0.7589756	4.085456	0.1876019
cg02330214	0.2736392	0.1194698	0.626756	0.0021776
cg18446641	0.403258	0.1846279	0.880782	0.0227002
cg11146023	0.3423775	0.1237179	0.947497	0.0390359
cg04936178	0.1858133	0.0733562	0.47067	0.0003865
cg23949574	1.3670824	0.6159966	3.033968	0.4420436
cg00433296	8.0835359	0.2650881	246.4975	0.2307122
cg25366586	1.3572801	0.5827508	3.16123	0.478845
cg05793120	1.4181662	0.6519596	3.084847	0.3782592
cg08525461	3.1866821	0.8242416	12.32035	0.0929943
cg16049391	1.7892019	0.9970393	3.210749	0.0511731
cg05914723	6.8689034	0.5507677	85.66558	0.1344696
cg23036947	1.0120959	0.4354091	2.352588	0.9777118
cg27107685	0.1855765	0.0572481	0.601568	0.0050017
cg22131825	176.04325	2.28E-11	1.36E+15	0.732714
cg03545404	1.5171134	0.63091	3.648116	0.3518119
cg05739379	2.3414471	0.9240321	5.9331	0.0729066
cg26581206	0.4465518	0.1751742	1.138344	0.0913016
cg14431257	1.1588883	0.3623253	3.706675	0.8036849
cg14611399	1.0762559	0.5470819	2.117282	0.8314319
cg23030863	2.1275449	1.0419495	4.34421	0.0381921
cg03753191	2.2211422	1.0517335	4.690801	0.0364203
cg27451550	3.0741346	0.7438589	12.70443	0.1208461
cg11331988	977220.12	0.0008557	1.12E+15	0.1949223
cg15345907	3.6795055	0.4735923	28.58737	0.2129675
cg24074477	0.6921213	0.3069261	1.56074	0.3750874

cg05088898	0.9786721	0.395891	2.41935	0.962763
cg13036546	1.7532477	0.3719169	8.264958	0.4778774
cg04457354	0.3782227	0.1792943	0.797864	0.0106832
cg07065453	1.2048575	0.6361622	2.281936	0.5673791
cg04722901	0.552458	0.0358727	8.508143	0.6706025
cg11464806	2.7622322	0.9620614	7.93081	0.0590137
cg00866476	2.3281865	1.0201488	5.313394	0.0447126
cg20198768	1.5135556	0.671069	3.413733	0.3179149
cg11781976	0.0495463	0.0024458	1.003684	0.0502807
cg24720571	2.0523091	1.0413686	4.044651	0.037795
cg26947834	31791.334	0.0006208	1.63E+12	0.2523626
cg01454519	1.7432238	0.748278	4.061097	0.1977708
cg22745102	0.2581408	0.1049547	0.634908	0.0031851
cg08057985	0.2581429	0.0843307	0.790196	0.0176686
cg01518723	0.3037471	0.1425751	0.647113	0.0020162
cg09856470	0.0010594	8.50E-06	0.132107	0.005402
cg11315141	29519.114	0.6118498	1.42E+09	0.0613898
cg08073133	1.3802046	0.437991	4.349323	0.5821527
cg13808183	0.2670899	0.0781081	0.913312	0.0353335
cg12777588	3.4505378	1.4177585	8.397912	0.0063493
cg04513422	0.2287133	0.0693427	0.754366	0.0153972
cg22892437	2.9747674	1.2027397	7.357569	0.0182991
cg13480197	0.1784011	0.0617743	0.515213	0.0014448
cg23735214	0.0484424	0.0048314	0.485712	0.0100547
cg16578291	0.0019597	9.84E-08	39.02481	0.2170244
cg20071561	2.0999535	0.6628493	6.652801	0.2072966
cg01926804	1.31E-12	1.96E-24	0.880847	0.0489421
cg04987608	2.17E-07	7.43E-14	0.633428	0.0433753
cg26847805	2.31E-20	6.66E-36	8.04E-05	0.0132714
cg02531479	1.1211887	0.5428725	2.315579	0.7572264
cg11951143	1.3354021	0.3716665	4.798116	0.6576005
cg15768886	6.6093468	0.6631639	65.87129	0.1074338
cg09648727	0.1597872	0.0366221	0.697173	0.0146923
cg14306330	1.4436121	0.4061747	5.130837	0.570407
cg06150919	3.4376034	0.6072743	19.45927	0.1627008
cg00080081	1.3795165	0.6516857	2.92022	0.4004245
cg07954489	0.6037919	0.3091109	1.179398	0.1396929
cg26512283	0.297018	0.1065089	0.828285	0.0203402
cg08667024	1.5218033	0.3679325	6.294321	0.5621402
cg25482146	0.2327163	0.0655919	0.825664	0.0240422
cg09980085	0.5981021	0.2799483	1.277829	0.1845049
cg27387923	0.2405127	0.0831157	0.695974	0.0085755
cg14536906	0.0549717	0.0105526	0.286365	0.0005711
cg26281310	2.8813821	1.2243637	6.780961	0.0153707
cg01371631	7.9866083	0.2491267	256.038	0.2402297
cg23101632	0.7489562	0.129892	4.318474	0.746398
cg08946601	2.3177015	0.9299313	5.776491	0.0712229
cg08110693	0.4386009	0.2180892	0.882074	0.0207798
cg05004705	0.7996476	0.4071867	1.570376	0.5161399
cg01328833	0.1438889	0.049613	0.41731	0.0003589
cg27115973	3.8000582	1.3251139	10.89751	0.0130039
cg11735308	3.1649754	0.0147944	677.0831	0.6738605
cg12374123	0.9205772	0.388657	2.180489	0.8508016
cg05704183	0.4155446	0.1871488	0.922674	0.0309507
cg06626102	0.4041661	0.1395609	1.170458	0.0949498
cg21406402	1.3884734	0.5458799	3.531653	0.4907923
cg09561125	2.0711015	0.6066055	7.071254	0.245194
cg14051161	1.9138029	0.8402874	4.358796	0.1221987

cg10239563	0.094189	0.026813	0.330868	0.0002284
cg03883295	2.0524723	1.0622526	3.965763	0.0323812
cg12407685	0.2079662	0.0829974	0.5211	0.0008059
cg08934785	2.1419864	0.8823161	5.200071	0.0923199
cg05088892	2.2947372	1.1189233	4.706148	0.0234152
cg14302471	1.9769873	0.9584329	4.077989	0.0650324
cg00530015	6.5081041	0.5726421	73.9649	0.1309398
cg19903766	0.3803849	0.116563	1.241326	0.109216
cg16426479	1.7820633	0.9221096	3.444005	0.0856624
cg25758828	0.3595697	0.1682721	0.768341	0.0082866
cg06532546	1.5869725	0.6690926	3.764026	0.294612
cg15185106	4010.2693	18.847134	853300	0.0024162
cg18012383	14344.189	0.9064394	2.27E+08	0.0523733
cg13941760	0.3587753	0.1407677	0.914412	0.0317612
cg12389490	12.768093	0.9579823	170.1745	0.05392
cg15271054	1.3283167	0.3517106	5.016696	0.6753996
cg23015138	1.7818872	0.8846148	3.589271	0.1059178
cg17224613	1.5630055	0.3682356	6.634301	0.5448451
cg12485556	0.5056614	0.2586842	0.988439	0.0461555
cg02572713	5.3476938	1.498526	19.08397	0.0097911
cg06133910	0.1972526	0.0520856	0.747013	0.0168817
cg11218175	0.3653021	0.0725998	1.838099	0.2218757
cg21424703	1.8117602	0.9117123	3.600341	0.0898553
cg10233454	0.3747396	0.1610714	0.871848	0.0227092
cg23355725	1.73E-22	1.37E-37	2.19E-07	0.0047388
cg24435209	0.4007394	0.1401031	1.146242	0.088117
cg16619049	0.1835362	0.060186	0.55969	0.0028808
cg12676702	1.6176341	0.5298905	4.938266	0.3983052
cg25654677	0.838046	0.3172188	2.213996	0.7214986
cg05646575	0.3691661	0.1697033	0.80307	0.0119696
cg14767950	1.9538863	0.8044903	4.745454	0.1390173
cg23092956	0.4944565	0.2332333	1.048252	0.0662027
cg17836612	0.2648979	0.0822999	0.852624	0.0259283
cg26562592	13.636108	0.0011225	165651.2	0.5861076
cg02613937	0.0267339	0.0010371	0.689161	0.0289252
cg18650670	2.9164373	1.02212	8.321534	0.0454072
cg04221650	1.7262269	0.8828716	3.37519	0.110529
cg21505886	1.5458351	0.5453303	4.381943	0.412594
cg06014251	4.376928	1.1115774	17.23452	0.0347522
cg19965031	0.2200594	0.0580309	0.834489	0.026013
cg17553080	1.428E+11	3.60E-08	5.66E+29	0.2397908
cg16819028	18.099713	1.4251692	229.8672	0.0255374
cg04556008	1.8667019	0.8347429	4.17443	0.1284942
cg18855674	1.4207884	0.6588847	3.063722	0.3703503
cg03841065	1.8707421	0.8633948	4.05339	0.1123672
cg02771673	0.4131818	0.1712294	0.99702	0.0492288
cg05399210	0.4693071	0.2136515	1.030881	0.0595361
cg25763864	0.942836	0.2879401	3.087238	0.9225164
cg03717367	0.1649926	0.0351969	0.773436	0.0222606
cg00862290	2.4020847	0.2044679	28.21965	0.4857004
cg07689920	0.4138581	0.1428661	1.198875	0.1040087
cg02568911	1.39E-08	4.36E-20	4416.982	0.1806108
cg18210026	2.549733	0.8493752	7.654024	0.0951412
cg22863838	0.5138413	0.2414686	1.093446	0.0839689
cg24019851	51.353983	3.4158177	772.0645	0.0043955
cg03719475	14.828537	6.37E-09	3.45E+10	0.8064249
cg21861151	4.130834	1.6117208	10.58731	0.0031376
cg13654873	0.4940752	0.2476829	0.985576	0.0453706

cg24574014	0.0493073	0.0012845	1.892674	0.1058438
cg21045388	1.9926393	0.4673755	8.495549	0.3513935
cg25549459	0.0350309	0.0001131	10.84933	0.2520955
cg20426710	0.4676719	0.2002492	1.092224	0.0790678
cg12871687	0.0058304	1.43E-05	2.376145	0.0934011
cg15111351	2.6714527	0.753339	9.473371	0.1281552
cg15817440	0.3045955	0.1279956	0.724856	0.007201
cg13000039	0.7714426	0.1484442	4.009073	0.7576222
cg03719070	3.4227893	0.8554352	13.69535	0.0819903
cg00164997	2.4451725	0.8507828	7.027491	0.0969241
cg21415450	0.9019774	0.3748933	2.17012	0.8178499
cg14726583	1.8138663	0.891821	3.689206	0.1001987
cg07820868	2.2444773	1.1408423	4.415753	0.0192011
cg09586183	1.2336054	0.5490683	2.771572	0.6112243
cg05241571	1.4409721	0.5895164	3.52221	0.4230671
cg10453602	2.9856688	0.9752046	9.14087	0.0553671
cg19910327	0.4719251	0.1523469	1.461883	0.1930114
cg08369013	7.7074745	0.4865643	122.0911	0.1473734
cg19182008	0.4707315	0.1505149	1.472201	0.1952668
cg04323925	1.8166948	0.5727745	5.762093	0.3107095
cg12633998	2.6761975	0.4998943	14.3271	0.2501528
cg00509007	0.4005251	0.1652377	0.970846	0.04282
cg01364826	0.3901982	0.1802117	0.844865	0.0169559
cg06629702	0.6048491	0.2559718	1.429229	0.2518122
cg20148127	1.6059546	0.6693444	3.853159	0.2887365
cg12630461	0.6806602	0.3165775	1.46346	0.3246435
cg08615070	1.5597903	0.5987294	4.063514	0.3628321
cg19876672	2.7145539	1.222528	6.027512	0.0141424
cg03048225	1.954081	0.347263	10.9958	0.4472378
cg03839714	0.6330711	0.2918191	1.373382	0.2472699
cg03748352	0.0002085	4.99E-09	8.714386	0.118483
cg23697400	98.035033	1.9123893	5025.581	0.0224458
cg19740491	283.41723	0.0827456	970750.2	0.1738746
cg13204568	2.3578787	1.0524619	5.282464	0.0371415
cg00966405	0.2053248	0.0713727	0.590678	0.0033193
cg27324117	2.7426349	0.8883954	8.467003	0.0793947
cg24685778	0.3799948	0.178207	0.810272	0.0122616
cg16936421	0.3417451	0.1650821	0.707464	0.0038261
cg15078329	0.0531317	0.0089244	0.316322	0.0012619
cg09012538	2.2290982	0.3162191	15.7134	0.4211143
cg13051263	1.97686	0.8894496	4.393701	0.0944327
cg12354861	1.4897847	0.7417392	2.992235	0.2625746
cg16740427	0.4399658	0.1844327	1.049542	0.0641757
cg14823851	9.9777295	1.6522139	60.25557	0.0121679
cg14634687	0.1110297	0.0178252	0.691584	0.0185177
cg08794544	0.1756271	0.0596154	0.517398	0.0016033
cg04100843	1.3566657	0.564749	3.259044	0.4951377
cg22851683	1.6400968	0.464924	5.785715	0.4417648
cg10767420	0.368903	0.1483888	0.917114	0.0318589
cg22116670	0.0379696	0.0013607	1.059492	0.0541122
cg01159294	0.6457618	0.325969	1.279288	0.2099111
cg05297739	0.378231	0.164846	0.867833	0.0217613
cg07160163	1.5158855	0.5063566	4.538123	0.4571313
cg07375367	0.0252254	0.0008709	0.730684	0.0321402
cg13716443	1.2105295	0.4190074	3.497269	0.7241166
cg26128018	1.324887	0.4653124	3.772359	0.5982253
cg02137583	3.3702602	0.6610615	17.18245	0.1437602
cg19670883	1.6195109	0.8170031	3.210289	0.1672721

cg01279538	0.1354078	0.0371752	0.493212	0.0024321
cg13311902	4.6280075	0.6726007	31.84423	0.1194852
cg14571710	0.4029874	0.0834693	1.945611	0.2578847
cg01199888	0.5881105	0.2783957	1.242383	0.1641691
cg26289880	4472.7147	2.59E-15	7.72E+21	0.6948138
cg14887099	0.4670368	0.2235964	0.975523	0.0427743
cg08494738	6.0589106	1.5878983	23.11886	0.0083702
cg08633665	1.8921626	0.4975687	7.195548	0.3494064
cg01569339	8.6416539	0.3723756	200.5453	0.1788753
cg09915444	2.0765711	0.8408869	5.128095	0.1131385
cg26432256	0.1040865	0.0105295	1.028923	0.0529202
cg12580943	0.3567547	0.1694946	0.750902	0.0066391
cg13158481	0.8642351	0.4631374	1.612701	0.6466424
cg15773251	0.4555328	0.2191552	0.946864	0.0351852
cg16332577	0.021123	0.0004797	0.93015	0.0457753
cg22444124	0.2795663	0.1238853	0.630885	0.002146
cg23709476	3.0251276	0.8505102	10.75989	0.0872925
cg27498364	1.7433426	0.2171259	13.99761	0.6010062
cg24559044	2.5220554	0.7258508	8.763182	0.1454623
cg01550307	1.4570258	0.7029822	3.019883	0.3114342
cg16044595	0.2435788	0.0895323	0.662673	0.005679
cg08402246	0.3085029	0.1368143	0.695644	0.004586
cg20156545	2.4992099	0.6350586	9.835392	0.1900574
cg03476007	1.5806905	0.6058613	4.124018	0.3493803
cg08294136	0.5369148	0.1988288	1.449878	0.2198087
cg01961547	1.5321013	0.6541409	3.588423	0.3258417
cg09197279	1.6704251	0.5634896	4.951857	0.3547594
cg20252202	3.6898005	1.3117341	10.37911	0.0133534
cg03841977	0.0010268	1.10E-05	0.095786	0.0029436
cg00297741	2.5240703	1.0478376	6.080075	0.0390044
cg16122707	2.8286645	0.8819719	9.072106	0.0803362
cg08558340	0.2200369	0.0632197	0.765841	0.0173495
cg25537724	2.05E-22	2.84E-36	1.48E-08	0.0021582
cg03589751	1.2757	0.4164194	3.908105	0.6699061
cg01727969	2.8630421	1.1655412	7.032793	0.0217879
cg25736663	0.8016168	0.3153184	2.037907	0.6422929
cg07005778	1.2766436	0.6608878	2.466105	0.4671982
cg13153834	0.0428054	0.0063356	0.289208	0.0012262
cg26146569	1.3247948	0.3373903	5.201931	0.6869254
cg26652484	72.076077	4.4542164	1166.302	0.0025979
cg12149692	1.769E+12	632.20683	4.95E+21	0.0110514
cg10963543	0.007521	0.0001906	0.296842	0.0091172
cg26607620	1.9027082	0.8795323	4.116163	0.1022759
cg23620184	1.2640657	0.3475464	4.59755	0.7220604
cg23371883	52.905959	0.3683599	7598.657	0.1173724
cg23391368	1.1360574	0.3860166	3.343448	0.8168324
cg16978168	5.202081	0.493378	54.84972	0.1700242
cg12377026	0.4189059	0.0379992	4.618051	0.4773621
cg10151367	6.5670348	1.6000202	26.95338	0.0089919
cg10830905	1.9906528	0.0051506	769.3618	0.8208022
cg03144922	2.6704305	1.0644436	6.699462	0.0363453
cg22694931	3.9810055	1.32918	11.92344	0.0135721
cg08293777	0.5010111	0.2445264	1.026523	0.0589674
cg03454425	1.1819711	0.3044373	4.588977	0.8091189
cg02139586	1.7363367	0.7726854	3.901802	0.1816473
cg07493097	1.7932212	0.6412994	5.014261	0.265634
cg07782221	1.7981347	0.5840756	5.535737	0.3064462
cg06848199	0.3095616	0.0826169	1.159913	0.0818843

cg13036855	0.2941875	0.1183394	0.731339	0.0084546
cg05764628	0.127322	0.0306259	0.52932	0.0045822
cg04922154	2.263441	0.9400239	5.450037	0.0684532
cg11338426	2.15E-20	7.02E-34	6.62E-07	0.0042638
cg10437931	0.4164664	0.1596272	1.086558	0.0734065
cg14992253	6.95E-05	4.19E-08	0.115269	0.0113703
cg10583736	1.03E-09	2.62E-19	4.041325	0.066349
cg04057288	0.6590771	0.2346653	1.851073	0.4287814
cg23934700	1.5154024	0.8074767	2.843976	0.1956006
cg13828701	0.3858547	0.1905512	0.781333	0.0081585
cg09909671	0.0460222	3.87E-05	54.66436	0.3940588
cg04723401	1.8661791	0.968551	3.595706	0.0622556
cg24815934	2.3278606	0.8045015	6.735767	0.1190719
cg01536615	0.3779824	0.0021187	67.43413	0.7129983
cg01362383	0.0030948	3.72E-06	2.576432	0.0921591
cg10421011	0.6323587	0.081437	4.910269	0.6612053
cg08885114	1.0126838	0.1661674	6.171658	0.9890947
cg26237681	1.4619789	0.7182636	2.975763	0.2949286
cg00362285	1.881056	0.4615388	7.666467	0.3781076
cg00846769	1.72E-05	5.29E-12	55.67017	0.1514346
cg14269191	0.6936338	0.3188977	1.508722	0.3561833
cg15574301	2.9706498	0.7969654	11.07295	0.1048252
cg16905265	1.5556359	0.8227209	2.941463	0.1739658
cg15147534	0.6453299	0.3207716	1.298278	0.2194255
cg18813650	14.035925	0.7873274	250.2227	0.0722912
cg20779334	0.2685715	0.0679769	1.061104	0.0607437
cg13564742	1.2132232	0.6201339	2.373537	0.5724273
cg21929781	1.9183562	0.4867832	7.560019	0.3518245
cg21184369	0.328038	0.1641341	0.655616	0.0016053
cg07993367	0.6210824	0.261192	1.476858	0.2811668
cg16874494	4.9685551	0.8050718	30.66377	0.0842647
cg04028464	2.6896483	0.3460264	20.90652	0.3443244
cg10625723	2.735097	1.1185945	6.687639	0.0274091
cg06678137	0.4322293	0.1193644	1.565141	0.2013817
cg27120405	0.7748625	0.278987	2.152114	0.6245618
cg18469326	4.67E-40	3.95E-63	5.51E-17	0.0008342
cg10628098	9.99E-13	2.80E-23	0.035622	0.025816
cg11283429	2.2455031	0.997284	5.056016	0.0507726
cg02448222	1.34E-06	1.18E-10	0.015136	0.0045112
cg02673986	3.1090361	0.808944	11.94904	0.0986768
cg05956452	1.0003484	0.0256902	38.95253	0.9998512
cg02648589	0.5024356	0.2286718	1.103947	0.086577
cg14430943	1.8409807	0.6310902	5.370405	0.2638756
cg13880779	1.3810647	0.7650802	2.492993	0.2840023
cg05275231	379124.2	0.0015715	9.15E+13	0.1920926
cg07420827	2.2612358	0.4399684	11.62171	0.3286165
cg26622320	0.0327061	0.0009091	1.176696	0.0613512
cg04899753	0.4728852	0.1930868	1.158134	0.1012718
cg24287175	0.3285308	0.1275152	0.846429	0.0211522
cg18899758	0.4935768	0.1819786	1.338718	0.1654566
cg09569347	3.9847902	0.9300508	17.07278	0.0625635
cg06889086	1.7835388	0.9584744	3.318827	0.0678342
cg19175364	0.4793624	0.1907395	1.204723	0.117854
cg13783653	1.4363512	0.6322614	3.263057	0.3870845
cg06414161	1.38E-05	1.89E-10	1.006738	0.0501376
cg22579504	1.1450565	0.4313212	3.039856	0.7856882
cg06241689	2.8829713	0.7467949	11.12959	0.1244577
cg02216667	0.5406952	0.2147943	1.361076	0.1917314

cg08104568	0.247578	0.0661495	0.926612	0.0381579
cg02970077	379960.39	2.95E-08	4.9E+18	0.4042012
cg12363584	2.1721625	0.8607919	5.481336	0.1004754
cg24077001	0.1004389	0.0138845	0.726561	0.0228245
cg02536620	0.0021856	2.20E-05	0.217589	0.0090623
cg12600417	0.356044	0.0091975	13.78277	0.5798472
cg06410158	0.1584851	0.0521572	0.481573	0.0011599
cg18560638	2.7180358	0.2832825	26.07898	0.3861096
cg11801411	0.2712368	0.1008583	0.729433	0.0097376
cg11269533	1.4314411	0.4403647	4.653015	0.5509382
cg18959207	2.0632533	0.9876756	4.310134	0.0539832
cg25658272	0.1997881	0.081007	0.492739	0.0004711
cg11553116	49.237875	0.8457374	2866.573	0.060222
cg17427986	0.8864201	0.3545076	2.216428	0.7965292
cg02118886	0.6439668	0.2993439	1.385341	0.260155
cg01918604	0.382985	0.1716869	0.854331	0.0190496
cg08856033	1.5915646	0.7024335	3.606146	0.2654552
cg26267430	1.5328702	0.5702019	4.120805	0.3972325
cg26654775	0.5480109	0.2823369	1.063679	0.0754829
cg24792365	0.7865368	0.3616372	1.710665	0.5447219
cg13552867	2.0860973	0.9304623	4.677032	0.0742616
cg16288318	1.7560855	0.895788	3.442596	0.1011031
cg06419846	1.0849167	0.2882415	4.083535	0.9040719
cg27547053	0.6223749	0.3010553	1.286642	0.2006219
cg26550194	2.2903582	0.8914674	5.884389	0.0851909
cg18881764	40.24167	0.006001	269854.8	0.4111123
cg03721175	0.3265297	0.146064	0.729965	0.0063949
cg14135025	6.31E-12	1.97E-22	0.201901	0.0366535
cg13727085	8.9661344	1.0089183	79.68095	0.0490761
cg20553394	0.1865641	0.0373907	0.930877	0.0406281
cg07979652	0.2552191	0.0832901	0.782047	0.0168366
cg02000253	0.4518625	0.1581077	1.291397	0.138163
cg27356342	0.6953368	0.2439559	1.981888	0.4965453
cg26897150	0.1938197	0.0567071	0.662457	0.0088792
cg15367698	1.8671633	0.5241429	6.651428	0.3353756
cg07215749	0.0033184	1.03E-07	106.758	0.2810503
cg12538674	0.5535097	0.2217549	1.381584	0.2050229
cg14921903	2.0021735	0.8405794	4.768971	0.1169324
cg23829102	0.7042744	0.334881	1.481131	0.355316
cg26060489	0.3160238	0.1040018	0.960282	0.0422109
cg21995038	7.5176973	0.7558441	74.77173	0.0852263
cg24865132	4.44E-05	1.43E-09	1.375996	0.0575022
cg13909949	0.6804011	0.3759675	1.231345	0.2032508
cg12574408	0.6349795	0.048916	8.242675	0.7284119
cg14481454	2.0313741	0.9906585	4.165392	0.0530704
cg26884320	2.02E-09	8.39E-24	486475.5	0.236051
cg11819799	0.0481961	0.0046167	0.503143	0.0112795
cg10981541	0.0036038	3.51E-06	3.704673	0.1118649
cg17226286	1.6624978	0.6988576	3.954881	0.250302
cg04353053	56.136577	0.4097984	7689.915	0.1085871
cg03987221	15.228651	0.274799	843.9325	0.1837216
cg01702009	3.5094794	0.0786011	156.6955	0.5171509
cg14653576	1.906795	0.0299198	121.5204	0.7607628
cg24892473	1.1228627	0.551732	2.285205	0.7492462
cg11866674	1.2781964	0.6331792	2.58029	0.4934394
cg24020222	0.3264818	0.1156298	0.921825	0.0345437
cg08954025	0.2502296	0.0834226	0.750574	0.0134395
cg20658779	0.885369	0.2311235	3.391599	0.858978

cg12336693	9.90E-09	1.23E-21	79835.95	0.2241678
cg16241204	0.2455501	0.0190651	3.162579	0.2815045
cg20490386	8.46E-07	7.38E-26	9.69E+12	0.5322955
cg13393809	0.0001283	1.57E-06	0.010474	6.61E-05
cg11165035	0.0452799	0.0002168	9.455412	0.2561168
cg10951214	0.0762401	0.0185295	0.313692	0.000362
cg27104695	2.4952502	0.6679009	9.322151	0.1739059
cg07647164	0.6902571	0.2956546	1.611525	0.3915004
cg08221350	1.3190299	0.6583134	2.642875	0.4348574
cg12363375	1.6853769	0.7097298	4.002221	0.2368319
cg17865555	0.3569744	0.1908607	0.667663	0.0012618
cg07502829	1.4172697	0.6220748	3.228958	0.4064989
cg04407776	2.7033414	0.2995317	24.39827	0.375632
cg24836242	3.954817	0.8703271	17.97092	0.075052
cg06257052	0.635205	0.2642912	1.52667	0.3104338
cg16928066	1.4633762	0.6786925	3.155287	0.3314205
cg02829706	0.000664	1.16E-07	3.797416	0.0973825
cg27395066	0.237631	0.1029292	0.548615	0.0007617
cg02103647	1.8555639	0.754551	4.563134	0.1781348
cg04560810	0.0126968	4.16E-05	3.87789	0.1347293
cg20626907	1.1213816	0.5725904	2.196154	0.7383351
cg20001829	1.0869633	0.5846616	2.020808	0.7921177
cg23349301	1.4684317	0.6028567	3.57679	0.3976534
cg22187843	6.48E-17	1.83E-36	2290.43	0.1045693
cg11319300	1.7108762	0.7563528	3.870016	0.1972441
cg18876602	0.290528	0.1031199	0.818528	0.0193419
cg17633592	2.7408352	1.2109103	6.203744	0.015558
cg23024484	0.4824687	0.2180314	1.067626	0.0720994
cg01744056	9.4071804	0.7857455	112.6256	0.0767934
cg02849507	0.4486888	0.1254381	1.604948	0.2177849
cg11788486	0.7236907	0.3415208	1.533518	0.3986481
cg14099718	2.3054664	0.8369493	6.350654	0.1061642
cg22108469	1.8357995	0.8790899	3.833692	0.1058888
cg14671000	0.3629365	0.10381	1.268885	0.1124975
cg05617039	6.12E-09	2.39E-17	1.569185	0.0555746
cg02389877	0.2847181	0.1159323	0.699239	0.0061368
cg00426720	1.2005566	0.3189975	4.51833	0.7869246
cg25121437	0.3719987	0.1611651	0.858641	0.0204998
cg23709172	0.0189071	0.0025068	0.142605	0.0001185
cg00972420	0.2545897	0.0862278	0.751682	0.0132601
cg15912040	0.0451638	0.0102316	0.199359	4.34E-05
cg16024274	0.7751749	0.3578772	1.679057	0.5184094
cg10729496	1.1205105	0.0507579	24.73591	0.9425475
cg19240233	0.3799352	0.1559149	0.925831	0.0332093
cg11525052	1.9668096	0.7598146	5.091164	0.163343
cg14940165	1.4514588	0.714894	2.946916	0.3024891
cg21485555	315.38721	3.6321626	27385.64	0.0115278
cg04733196	0.3434634	0.0080588	14.63829	0.5767039
cg05715828	0.2399469	0.0224458	2.565046	0.2377083
cg13630574	534.91069	0.7998776	357716.5	0.0583993
cg26668837	1.1574025	0.5624483	2.381696	0.6913518
cg08143038	4.5302881	1.4159973	14.49403	0.0108909
cg24017912	0.0193373	0.0003423	1.092412	0.0552353
cg09373727	1.1830861	0.5310821	2.635548	0.680775
cg13369337	0.61362	0.2797606	1.345899	0.2229636
cg01030404	0.3521481	0.1665447	0.744595	0.0062968
cg08234689	5.7539849	1.1667728	28.376	0.031601
cg00989315	0.6888289	0.3340255	1.420506	0.3127684

cg04978408	0.1629825	0.0077968	3.406961	0.2421501
cg17796043	1.3590523	0.482618	3.827091	0.5613881
cg13020518	3.0701856	1.4373584	6.557891	0.0037684
cg03471122	2.4036493	1.1161605	5.176254	0.0250422
cg20231694	0.9233387	0.4022069	2.119691	0.8507904
cg19152840	0.0079255	2.99E-05	2.102064	0.0893112
cg02224980	2.2644286	0.8167809	6.277861	0.116191
cg10862923	0.0626251	0.0013182	2.975105	0.15958
cg01655781	0.0033721	1.39E-09	8203.995	0.4480256
cg13821785	0.2008789	0.0926673	0.435454	4.78E-05
cg23141096	1.13E-25	5.39E-55	23691.62	0.095405
cg04080595	0.253163	0.0422972	1.515264	0.1323914
cg18842300	22.710075	0.690667	746.7383	0.0797236
cg08898868	13.230774	0.0642969	2722.579	0.3419934
cg08219814	1.1200571	0.4866119	2.578087	0.7898102
cg20708856	0.528501	0.2295725	1.216667	0.1338785
cg21553700	0.1350229	0.0128677	1.416817	0.0950248
cg22472154	0.0079143	2.91E-07	215.3878	0.3529947
cg05272349	1.5094987	0.6178835	3.687728	0.3662408
cg24480379	0.2644796	0.1284633	0.544509	0.0003064
cg15104158	0.361876	0.0865593	1.512885	0.1637119
cg03702990	0.2326043	0.0469344	1.152774	0.07412
cg16758800	4.4351743	1.4280429	13.77464	0.0099896
cg12936121	1.3932477	0.5834958	3.326741	0.4551732
cg12482260	1939.3275	1.46022	2575633	0.039099
cg22594201	0.8740639	0.4504033	1.69623	0.6907007
cg16536740	0.3559525	0.109631	1.155714	0.0855936
cg06494185	0.4945878	0.2034252	1.202492	0.1203832
cg15021031	0.4999893	0.2393267	1.044553	0.0651821
cg16708174	0.4662997	0.2322495	0.936215	0.0319289
cg08736680	1.671697	0.5787786	4.828393	0.3423683
cg20951642	1.6025418	0.7926125	3.240096	0.1892146
cg02160608	0.0838355	0.0206815	0.33984	0.0005179
cg13436155	2.5997624	0.980253	6.894919	0.0548723
cg11410473	2.2787467	0.8145316	6.375058	0.1166162
cg24034459	0.4583475	0.2255115	0.931582	0.0310985
cg09354317	0.1767353	0.0498478	0.626615	0.0072793
cg23644992	1.608631	0.5299221	4.883159	0.4014179
cg26058289	0.054863	1.62E-05	185.8185	0.4839095
cg05308504	1.9841604	0.8805301	4.471048	0.0983265
cg22713958	0.284492	0.1013184	0.798825	0.0170158
cg03182958	1.6111411	0.7576645	3.426022	0.2153367
cg02680909	3.8692612	1.0407347	14.38521	0.0434292
cg06998361	0.37387	0.158	0.884676	0.0251691
cg10307345	0.1795479	0.0389141	0.828426	0.0277196
cg06198069	0.9648927	0.1578772	5.897104	0.9691335
cg13683864	8.0674544	0.9641662	67.5027	0.0540674
cg13857119	0.1610279	0.056268	0.46083	0.0006638
cg26878655	2.6433248	0.5280511	13.23199	0.2368537
cg14397696	1.2132715	0.5575886	2.639989	0.6260024
cg07514503	4.5059638	1.0578385	19.19358	0.0417493
cg26420566	5.7008807	1.06E-10	3.06E+11	0.8901725
cg12056000	0.0524032	0.0020977	1.309123	0.0725079
cg11630404	1.3371356	0.6479297	2.759453	0.4318929
cg20690922	0.2501374	0.0892639	0.700941	0.0083928
cg03344767	1.4834531	0.4635054	4.747804	0.5064052
cg21020871	0.3316951	0.1107877	0.993085	0.0485679
cg16686429	0.4921339	0.1965714	1.232101	0.1299741

cg15669196	0.9502256	0.4210103	2.144671	0.9021651
cg15618525	1.2913355	0.471739	3.534894	0.6187441
cg13100190	0.4012511	0.183524	0.877283	0.022137
cg20675389	1.7735978	0.7336791	4.2875	0.2032552
cg13692105	0.8676041	0.3893825	1.933156	0.7282665
cg24404878	1.8209731	0.9820757	3.376464	0.0570991
cg20825110	1.165309	0.1541722	8.807973	0.8821507
cg18513970	1.0524424	0.478963	2.312569	0.8987389
cg17656763	0.4045575	0.1777846	0.92059	0.0309905
cg25822369	2.542236	1.0680277	6.051307	0.0349701
cg20328456	0.2596881	0.0640557	1.052801	0.0590373
cg24478470	6.5476707	0.1021223	419.8104	0.3760565
cg01310330	0.18041	0.0614219	0.529905	0.0018384
cg16539957	1.2846767	0.6695611	2.46489	0.4511733
cg02380813	0.0061031	8.59E-05	0.433798	0.0190845
cg04670568	2.8887852	1.2165307	6.859736	0.0162095
cg24791188	0.3396349	0.1253113	0.920523	0.0337746
cg23080538	3.4967301	0.2876631	42.505	0.3259608
cg27258486	0.5275881	0.2186316	1.273142	0.1548289
cg20248866	0.1466594	0.0435348	0.494064	0.0019497
cg05991902	1.375398	0.3007764	6.289456	0.6810961
cg15771763	6941.147	12.13166	3971388	0.006326
cg15816503	1.9357347	0.9033188	4.148113	0.0894158
cg18680107	0.0037109	3.78E-09	3642.696	0.4266
cg07810039	0.1880772	0.0295503	1.197043	0.0768109
cg18866792	0.6127545	0.2800269	1.340829	0.2202381
cg12817436	2.0698419	0.524341	8.170724	0.2990813
cg23369985	4.0350023	0.7299471	22.30469	0.1097935
cg09076339	1.6972963	0.7010354	4.109371	0.2409379
cg22505086	3.0878446	1.1308194	8.431748	0.0278183
cg11724366	1.8962346	0.3408745	10.54847	0.4649054
cg12178913	14.957118	1.69E-17	1.32E+19	0.8979089
cg10486879	6.23E+32	1.872E+14	2.07E+51	0.00052
cg25787940	3.559E+09	7.28E-13	1.74E+31	0.3880724
cg07367144	0.383176	0.1637024	0.896895	0.0270534
cg05454389	1.2602197	0.2987831	5.315407	0.752801
cg21148404	2.6387819	0.7920513	8.791312	0.1140419
cg18696027	1.7219778	0.936906	3.164893	0.0801018
cg01681847	3.9667699	1.3737951	11.45387	0.0108665
cg25894806	3721041.1	2.46E-11	5.64E+23	0.4535025
cg25017304	2.18E-09	1.12E-18	4.250757	0.067643
cg19376461	3.3673777	0.4049688	28.00026	0.2612258
cg14213297	0.0757769	0.0158806	0.361583	0.0012129
cg04890406	0.1144548	0.0150364	0.871213	0.0363407
cg03008204	29.686794	0.0003257	2705753	0.5606195
cg15545021	0.119697	0.0021655	6.616094	0.299754
cg16100063	2.66E-20	6.80E-44	10369.24	0.1038773
cg04307702	3.7519078	0.9575145	14.70141	0.0577412
cg12479444	0.3707026	0.1038449	1.323323	0.1263959
cg06985664	2.8849898	0.4280971	19.44224	0.2764101
cg08056778	2.3536864	0.2311815	23.96316	0.4696935
cg27014354	0.4846614	0.1889713	1.243028	0.1317465
cg03782584	0.1045866	0.016933	0.645978	0.0150836
cg02479744	0.3509522	0.0922543	1.335087	0.1245316
cg00331237	0.1888902	0.0471197	0.75721	0.0186452
cg01500115	8.1654457	1.0409749	64.05006	0.0456976
cg06226630	3.5757065	0.9982713	12.80782	0.0503115
cg09089913	0.4683329	0.186744	1.174526	0.1058675

cg21616626	0.3904263	0.1405585	1.084479	0.0711726
cg13331542	0.4513914	0.1916973	1.062896	0.0687028
cg06394460	0.437999	0.2071343	0.926178	0.0307199
cg13649020	6.179733	1.4087871	27.10779	0.0157661
cg02455453	26.241382	0.9347798	736.6549	0.0548169
cg00252442	0.0919486	0.0102004	0.828842	0.0333953
cg05237360	0.3346444	0.1551662	0.721722	0.0052448
cg11039476	1.4291928	0.5895967	3.464388	0.4292405
cg02363950	0.3741897	0.170047	0.823407	0.0145727
cg27484512	0.3325488	0.163933	0.674597	0.002283
cg18087902	2.29E-09	5.49E-20	95.35109	0.1107881
cg15067907	1.2032666	0.3651849	3.964705	0.7610099
cg00826767	0.2748785	0.1256505	0.601337	0.0012235
cg01883777	1.1065476	0.5848955	2.093447	0.7556182
cg16211507	1.3510637	0.7342187	2.486144	0.333526
cg09827071	0.2351602	0.0670069	0.825293	0.0238381
cg09001777	2.2397395	0.608662	8.241739	0.2251074
cg11555067	3.4258351	0.8972258	13.0807	0.0716529
cg10280342	0.1545413	0.0435167	0.548824	0.0038788
cg08440418	1.5184973	0.7288925	3.163476	0.2646379
cg04175027	2.2452367	0.7210965	6.990864	0.1628012
cg06487369	2.2170369	0.3685392	13.33712	0.3844962
cg13930261	0.9986207	0.4780966	2.085861	0.9970694
cg14057241	0.2223437	0.0538528	0.917996	0.0376881
cg27057027	2.4048243	0.7837758	7.378615	0.1250205
cg19125584	0.7217988	0.2998463	1.737535	0.4670076
cg17834443	3.967E+11	327.1031	4.81E+20	0.0123305
cg16850150	0.2793106	0.0800999	0.973964	0.0453535
cg25058261	1.6675288	0.8410464	3.306182	0.1431234
cg09987620	3.0481488	1.1772953	7.891997	0.0216621
cg04289585	1.5009145	0.582584	3.866814	0.4003445
cg04171052	0.1752677	0.0503	0.610711	0.0062527
cg18636558	0.4234801	0.1778377	1.008422	0.052256
cg19673881	0.1063748	0.0148964	0.759622	0.0254781
cg20853771	1.0739391	0.5476379	2.106036	0.8355443
cg25144149	0.343419	0.1243287	0.948587	0.0392284
cg17416644	1.521128	0.6489807	3.56533	0.3344743
cg20770512	1.6045091	0.5318617	4.840449	0.4013215
cg05162032	0.0959773	0.0020125	4.577224	0.2346143
cg23218363	0.4505187	0.2047996	0.991052	0.0474446
cg03256310	0.1971163	0.0362659	1.071388	0.0600902
cg17121140	1.816207	0.6414314	5.142573	0.2611165
cg02004337	0.4651922	0.2071364	1.04474	0.0637483
cg16021126	0.2212517	0.0711578	0.687941	0.0091544
cg27144670	0.5143837	0.2208226	1.198204	0.1233538
cg12483561	0.0015282	7.35E-06	0.317886	0.0172753
cg19500236	0.561151	0.2340071	1.345645	0.195422
cg13554213	1.12E-07	2.38E-10	5.24E-05	3.38E-07
cg02645852	0.277794	0.0991136	0.778596	0.0148548
cg10818794	2.1263647	0.0072245	625.8441	0.7947817
cg13357602	0.0028553	2.51E-06	3.246043	0.1026849
cg22896873	8.8931572	1.5036641	52.59702	0.0159625
cg15689991	0.5165534	0.1313979	2.030683	0.3442676
cg16504019	0.0503648	0.0014555	1.742834	0.0983832
cg10498083	1.0087145	0.4410485	2.307013	0.9835993
cg05887270	0.8679789	0.4158482	1.811688	0.7060798
cg06800231	0.2386462	0.0407433	1.397826	0.1121469
cg04578149	2.1794443	0.4105322	11.57029	0.3603568

cg26746573	3.3037059	0.9740855	11.20484	0.0551329
cg14398883	1.08562	0.4192825	2.810923	0.8656037
cg06078469	0.3043168	0.1012527	0.914629	0.034099
cg02788731	0.4320293	0.2011362	0.927975	0.0314288
cg04388989	1.6763869	0.8518069	3.29919	0.1347495
cg25766801	0.9968855	0.459116	2.164553	0.9937084
cg02750850	8.8502881	0.642414	121.927	0.10325
cg17512474	4.6891062	0.5974408	36.80317	0.1415728
cg13476072	0.2429847	0.0749608	0.787632	0.0183827
cg21191737	0.9862172	0.462679	2.102158	0.9713293
cg24803517	0.586136	0.2711122	1.267208	0.1744738
cg00228796	4.4823405	0.9807619	20.48548	0.0530015
cg04359828	0.4727177	0.2093965	1.067171	0.0713129
cg08578491	0.3859242	0.1730893	0.860466	0.0199491
cg14326196	0.208827	0.0632312	0.689671	0.0101848
cg27444437	0.3374298	0.0827604	1.375765	0.129753
cg06319713	0.0285771	0.0006748	1.210189	0.062865
cg15540496	0.3025992	0.1040036	0.880414	0.028257
cg04710764	7.56E-05	1.21E-09	4.721348	0.0920901
cg05441768	1.6016881	0.737669	3.477718	0.233728
cg20492912	0.7427426	0.251846	2.190492	0.5899132
cg17393321	1.7812479	0.4150454	7.644571	0.437291
cg03462130	0.0847112	0.0112302	0.638991	0.0166485
cg12164955	1.9512758	0.667962	5.700141	0.2216334
cg22108567	0.4677584	0.2018347	1.084046	0.076431
cg11166893	2.1392065	0.6693093	6.837204	0.1995973
cg20697044	1.0913656	0.4533829	2.627092	0.8453379
cg14584092	0.1805033	0.0570029	0.571575	0.0036016
cg06712013	0.7486152	0.3928494	1.426564	0.3788209
cg10561989	0.5492411	0.0771593	3.909648	0.5495777
cg04136445	0.000131	2.75E-10	62.29198	0.1800935
cg06882058	1.8619062	0.4663415	7.433812	0.3788555
cg17683110	2.0829077	1.0031459	4.324899	0.0490232
cg24576868	0.5842126	0.3142303	1.08616	0.0893661
cg15540602	3.466323	1.1759276	10.2178	0.02421
cg05967404	2.1588172	1.0992715	4.239618	0.0254289
cg08963712	1.05E-11	2.20E-23	5.04287	0.0654671
cg07387931	3.8814105	0.7837495	19.22215	0.0966224
cg01016119	2.0194246	0.8707863	4.683211	0.1015088
cg24722950	3.2209641	1.0493299	9.886891	0.0409424
cg01210550	1.5393892	0.529342	4.476726	0.4283413
cg07757535	0.0803539	0.007386	0.874187	0.0384171
cg07847664	1.9164536	0.2710639	13.54955	0.5145072
cg20399871	1.9571299	0.7636062	5.016143	0.1620167
cg17344040	2.3788991	0.828389	6.831526	0.107362
cg26535925	0.0691529	0.0115515	0.413982	0.0034346
cg13071386	1.2257101	0.6183376	2.429684	0.5599129
cg13057055	1.9447018	0.8025718	4.712183	0.140775
cg22214854	1.1644481	0.4130998	3.282353	0.7733905
cg25233534	1.5244855	0.0001977	11753.31	0.9264308
cg20704450	2.1799322	0.7981515	5.953887	0.1284692
cg05249010	0.344129	0.1562707	0.757818	0.0080858
cg17346647	0.3659438	0.1872141	0.715303	0.0032847
cg01963618	2.4432506	0.7978659	7.4818	0.1177018
cg00350503	0.3740006	0.1544157	0.905844	0.0293269
cg23547429	0.3378118	0.0702632	1.624133	0.175538
cg12858469	2.3943014	0.7649168	7.494513	0.1337033
cg26879339	0.4698716	0.2321552	0.950999	0.0357612

cg18802021	0.4133117	0.1212389	1.409008	0.1579499
cg08799967	2.2344214	0.901537	5.537919	0.0825406
cg03321020	1.982292	0.7235104	5.431133	0.1833178
cg07915730	1.4320049	0.2585589	7.931029	0.6809597
cg20457891	37.564175	0.0097476	144761	0.3893825
cg10291238	0.3905484	0.2009118	0.759179	0.0055648
cg06930095	5.18E-19	1.66E-31	1.62E-06	0.0041281
cg06112894	0.4843347	0.2135132	1.098668	0.0827769
cg13859433	0.0625773	0.0098853	0.396134	0.0032454
cg12990614	0.2256409	0.0657981	0.773789	0.0178924
cg09559352	3.22E-05	4.41E-10	2.350444	0.0702367
cg00964997	2.3150013	0.7472336	7.172096	0.1456894
cg23895262	0.091698	0.0231488	0.363238	0.0006693
cg03330678	0.2817935	0.0620318	1.280111	0.1009683
cg07164047	0.1980356	0.0571126	0.686681	0.0106965
cg07330481	3.3003167	0.5173101	21.05524	0.2066418
cg10443500	3.0027382	0.40674	22.16757	0.2810354
cg09273716	1.2978471	0.217326	7.750603	0.7749314
cg06459293	12.283079	1.2955617	116.4545	0.0288448
cg21348975	1.3773541	0.6620262	2.865603	0.3917015
cg26483472	6050.7958	0.460823	79449436	0.0718869
cg07847030	1.679628	0.7459011	3.782204	0.2105289
cg01217071	0.4721578	0.251226	0.88738	0.0197474
cg15500907	1.8525619	0.7521056	4.56317	0.1800602
cg04174070	0.0263152	2.62E-06	264.7996	0.4391908
cg02678688	0.2383969	1.98E-23	2.87E+21	0.9559198
cg10327980	0.3676878	0.1076676	1.255664	0.1103433
cg17755964	1.3441728	0.4137365	4.367031	0.6227254
cg02492873	0.5661152	0.2708369	1.183319	0.1304068
cg24841244	4.355259	1.1842802	16.01672	0.0267928
cg11224582	0.2204469	0.0725697	0.669657	0.0076464
cg16406212	9.619E+16	54043.894	1.71E+29	0.0065844
cg13518670	0.2765503	0.0845541	0.90451	0.0335065
cg07703277	2.41E-20	2.33E-50	2.49E+10	0.200179
cg09781650	0.3634682	0.0722413	1.828722	0.2195511
cg03280235	3.2659517	0.9459341	11.27609	0.0611999
cg20141229	0.4784697	0.236484	0.968071	0.0403434
cg19827787	7.5921318	0.9284111	62.08507	0.0586667
cg19835839	915.60081	0.0046361	1.81E+08	0.2730037
cg26414521	1.2430003	0.5370307	2.877023	0.6114373
cg08421051	0.3290653	0.1287517	0.84103	0.0202558
cg21438252	0.3361169	0.1438828	0.785185	0.0117819
cg15952370	0.7196085	0.3072296	1.685503	0.4486062
cg25676335	11.764594	0.5975725	231.6132	0.1049487
cg23461361	5.77658	0.247582	134.7791	0.2751404
cg01297180	0.5294987	0.2431992	1.152836	0.1092246
cg02271227	4.311243	1.9001513	9.781756	0.0004729
cg15674813	0.3047293	0.1010731	0.91874	0.0348166
cg22880770	0.4622143	0.1812923	1.178439	0.1060681
cg06840837	0.2694353	0.0803488	0.903503	0.0336416
cg19751990	0.4964659	0.2450635	1.005773	0.0518976
cg25375764	1.7561412	0.6746184	4.571521	0.2486588
cg26770014	0.0669662	0.0108042	0.415069	0.0036762
cg16012536	5.497E+10	0.0034998	8.63E+23	0.1106696
cg15998609	0.3635706	0.1369256	0.965368	0.042285
cg05277881	0.3211609	0.1447055	0.712788	0.0052332
cg09736959	3.1387633	0.5176055	19.03348	0.213558
cg18598861	0.5177558	0.0813538	3.295125	0.4857302

cg25615081	0.5934336	0.2381679	1.478635	0.2625902
cg11430077	1.9767756	0.7920677	4.93347	0.1441787
cg09548390	0.3547914	0.1600916	0.786281	0.0107059
cg17696847	1.8296841	0.5107978	6.553952	0.3533909
cg08037710	0.5826834	0.2904558	1.168921	0.1283718
cg01310205	2.6823073	0.7767324	9.262872	0.1186671
cg04146962	1.1001891	0.4406915	2.746629	0.8379236
cg16563470	0.418858	0.1529602	1.146978	0.090426
cg02825887	1.5639272	0.851405	2.872743	0.1494589
cg21492137	0.6172843	0.3106885	1.226437	0.1684345
cg13280041	0.4123018	0.1965015	0.865097	0.0191181
cg11740878	1.47E-13	9.04E-22	2.39E-05	0.002189
cg02964087	0.9632036	0.4567781	2.031098	0.9215435
cg06990298	0.2177422	0.0483143	0.981318	0.0471985
cg15947940	0.1542231	0.0432381	0.550088	0.0039625
cg21993290	0.7021086	0.1861823	2.647708	0.6015171
cg00415057	0.4301484	0.2156369	0.858052	0.0166436
cg24009439	4.71E-24	2.85E-34	7.77E-14	7.65E-06
cg24735307	2.2418468	0.9839888	5.107657	0.054663
cg04583285	0.4917045	0.1331482	1.815822	0.2868754
cg26339162	0.4225332	0.1974439	0.904228	0.0264654
cg10727935	0.3785951	0.1582636	0.905667	0.029064
cg15587745	0.0794936	0.0099043	0.638029	0.0171791
cg26275986	0.418315	0.162075	1.07967	0.0716224
cg12970084	0.1975087	0.0705686	0.552791	0.0020095
cg10227678	4.8534316	0.4018502	58.61836	0.2139618
cg00567298	0.1141196	0.0136375	0.954959	0.0452328
cg02621993	2.5224325	0.8562536	7.430819	0.0932616
cg14196225	2.1857848	1.0592755	4.510305	0.0343643
cg05624623	6.461957	0.7612776	54.85107	0.0872658
cg07275439	0.4069848	0.1527159	1.084606	0.072246
cg12915834	0.9942051	0.1132867	8.725156	0.9958156
cg06741568	0.5076704	0.1882237	1.369271	0.1805226
cg15694585	2.8332516	0.5077743	15.80882	0.2351055
cg00525496	0.3871328	0.1501523	0.998132	0.0495494
cg17847044	4.592228	0.7466554	28.24403	0.1000231
cg22164577	0.5218633	0.2513338	1.083584	0.0810508
cg18232497	1.5338763	0.5297298	4.441465	0.4303238
cg06248560	3.2420776	1.3631639	7.710788	0.0077954
cg09753064	3.381223	0.7346426	15.56222	0.1178044
cg03294619	1.3873082	0.7162043	2.687256	0.3318175
cg20516712	2.6111458	0.7280503	9.364851	0.1407776
cg10439725	0.4550263	0.1172642	1.765662	0.255049
cg12799677	7.93E-05	6.88E-07	0.009137	9.67E-05
cg10425624	0.0394533	0.0002769	5.622016	0.2014036
cg15513620	0.1380244	0.0253113	0.752656	0.02212
cg01221157	0.3729221	0.1617005	0.860052	0.0206909
cg05164350	8633.3167	0.1494449	4.99E+08	0.1051954
cg22816913	1.7721492	0.7701042	4.078036	0.1784221
cg10418524	1.3626903	0.6244788	2.97356	0.4369773
cg14566624	0.1187145	0.0281673	0.500338	0.0036911
cg02861260	1.594605	0.7144445	3.55908	0.2546541
cg26112797	1.5462799	0.5017409	4.765371	0.4478622
cg05605921	9.13E-17	2.79E-27	2.99E-06	0.0027918
cg02038492	0.8780485	0.2974334	2.592073	0.8138432
cg05949181	1.6514183	0.2236737	12.19268	0.6228684
cg11052081	7.29E-13	2.38E-28	2232.561	0.1245068
cg11878452	1.9115726	0.7822152	4.671489	0.1552597

cg15453278	0.1583351	0.0444692	0.563762	0.004448
cg11079129	1.428381	0.6600933	3.090885	0.365311
cg24359048	0.6427592	0.2292275	1.802312	0.400806
cg26909217	1.9601855	0.2526326	15.20915	0.5196806
cg09524946	2.5724236	1.0340093	6.399713	0.0421654
cg08363067	4.3303364	0.8202162	22.86204	0.084257
cg02610360	0.4564728	0.2267046	0.919114	0.0280801
cg02352716	4.4361076	0.8400243	23.42676	0.0793198
cg03719152	1.7513044	0.5593785	5.482991	0.3358884
cg06547017	8.745773	0.1784422	428.6461	0.2748117
cg16991765	0.9576685	0.4552308	2.014646	0.9092442
cg12656815	2.9780734	0.4748399	18.67771	0.2440499
cg16632096	4.562336	0.0802244	259.4586	0.4615951
cg14191134	0.0453808	0.0006597	3.121835	0.1519682
cg05963085	0.2422062	0.0677473	0.865922	0.0291512
cg05551825	1.2096118	0.5148584	2.84187	0.6623567
cg27518976	0.3342283	0.1497872	0.745781	0.007445
cg26767897	0.7042221	0.3603959	1.376067	0.3049084
cg18974966	4.8902087	0.6217325	38.46372	0.1314673
cg23279538	0.0177572	0.000425	0.741946	0.0342855
cg01791634	3.2191784	1.2244527	8.463463	0.0177619
cg16352928	1.9826907	0.683554	5.750917	0.2077618
cg27303421	0.4149822	0.1471798	1.170067	0.0963138
cg07433773	1.7603442	0.6488528	4.775832	0.266769
cg19428573	1.3837197	0.0821531	23.30625	0.8216597
cg14695378	0.7507077	0.3517824	1.602019	0.4584396
cg23954922	2.2542916	1.0813308	4.699608	0.030115
cg15914239	4.7585944	0.1099001	206.0437	0.4171383
cg08703055	0.5043294	0.2421762	1.050261	0.0674083
cg17690322	1.3293974	0.2476057	7.137548	0.7398539
cg11060194	0.4113011	0.1342635	1.259974	0.1198535
cg24638668	0.8862599	0.3099487	2.534151	0.8217793
cg03367679	0.1055456	0.0057262	1.945407	0.1304375
cg27067214	0.0133473	9.27E-07	192.2683	0.3769514
cg20126496	0.0049559	1.15E-05	2.138402	0.086449
cg21359303	1.8566876	0.5565221	6.194342	0.3141192
cg10850838	1.5721445	0.5148833	4.800386	0.4269554
cg12409547	1.3993539	0.2398606	8.163872	0.7088507
cg02470874	1.8686456	0.3561007	9.805754	0.4597915
cg10817441	1.8230267	0.8515865	3.902629	0.1220369
cg26562772	0.428184	0.222433	0.824255	0.0111371
cg22510337	0.5003216	0.2105238	1.189043	0.1168974
cg11947245	4.1973085	1.0807412	16.30122	0.0382534
cg03411644	0.6264296	0.191685	2.047182	0.4388536
cg13401703	0.2686253	0.0767962	0.939624	0.0396438
cg01424997	0.400318	0.1628873	0.983836	0.0459902
cg03734784	1.15E-09	8.09E-16	0.001621	0.0043853
cg11574130	3.14E-07	3.11E-11	0.003167	0.0014561
cg23881653	1.0994938	0.5380185	2.246923	0.7947805
cg00835857	1.6704317	0.3828938	7.287509	0.4948174
cg05205813	4.3999692	1.5329776	12.62884	0.0058855
cg27172769	0.7971644	0.4415063	1.439325	0.4520719
cg23850205	0.4431182	0.0810159	2.423643	0.3478179
cg06223834	0.469972	0.1895511	1.165246	0.1031315
cg23447239	0.223306	0.0556849	0.895496	0.0343676
cg05914981	0.3357489	0.1099192	1.025547	0.0554052
cg25217313	1445285.4	1.80E-08	1.16E+20	0.3852261
cg23246320	0.0159851	0.0014992	0.170437	0.0006142

cg11671400	1.47418	0.7848734	2.768863	0.2275228
cg27454883	1.2314121	0.6684285	2.268568	0.5042907
cg17515110	0.2517178	0.106268	0.596246	0.0017171
cg00094735	0.2484011	0.0664838	0.928092	0.0383655
cg02294420	1.9686521	0.8371724	4.629382	0.1205211
cg02603756	2.0527047	0.6829947	6.169296	0.2002326
cg01092932	0.3497784	0.1707289	0.716604	0.004097
cg21938541	0.2213323	0.0671279	0.729771	0.0132311
cg06557376	0.668068	0.043648	10.22531	0.7719861
cg14147498	1.8744661	1.0552511	3.329656	0.0320793
cg07004443	1.680357	0.7341294	3.846188	0.2192856
cg00740547	0.2203373	0.0747882	0.649147	0.0060739
cg18374948	0.1655281	0.0503495	0.544188	0.0030566
cg08631650	2.012519	0.6034495	6.7118	0.2550943
cg07835154	7.1747535	0.5491922	93.73236	0.1328671
cg16430963	1.586158	0.4190031	6.004483	0.4970041
cg25740565	2.2457702	0.9619548	5.242953	0.0614426
cg17445812	2.4830871	0.7223466	8.535683	0.148828
cg21063282	1.6505157	0.7770326	3.505905	0.1923551
cg05608541	2.2742245	1.1144433	4.640969	0.0239642
cg16977035	4.8245897	1.1912225	19.54015	0.0274435
cg18628371	2.3331979	0.7909306	6.882794	0.1247793
cg24757553	2.917896	1.073647	7.930089	0.0357932
cg26405020	0.2664637	0.1148849	0.618035	0.0020629
cg24663419	0.7004378	0.3212045	1.527416	0.3707335
cg27076552	0.5519527	0.1034988	2.943528	0.4865197
cg08688548	0.2512559	0.1073088	0.588297	0.0014618
cg02339083	0.5186988	0.2653606	1.013897	0.054908
cg19374305	0.1591868	0.0535528	0.473186	0.0009458
cg11940973	0.3736515	0.1546854	0.902577	0.0286872
cg11702639	0.3049799	0.1105963	0.841011	0.0217602
cg25548986	0.9111086	0.4608496	1.80128	0.7889332
cg11611553	0.7077733	0.0215556	23.23959	0.8461588
cg08135379	1.9904142	1.0152639	3.902186	0.0450628
cg06307913	0.3209042	0.1508192	0.682801	0.0031738
cg12439899	0.249514	0.0068804	9.048434	0.4486093
cg02183676	0.0045626	9.56E-05	0.2178	0.0062808
cg12078211	0.6993711	0.2765656	1.768549	0.4499949
cg19560648	2.0249056	0.638487	6.421811	0.2308848
cg26899718	1.2403005	0.4475001	3.437642	0.678845
cg06811732	1.308544	0.2720656	6.293657	0.7371911
cg12302402	3.613291	1.0259434	12.72572	0.0455179
cg07672076	0.0287928	0.0001821	4.552071	0.1696641
cg26786407	2.084278	0.838923	5.178324	0.1137177
cg11746396	0.3663747	0.168962	0.794442	0.0110003
cg18574813	0.2739896	0.1152229	0.651522	0.0033963
cg01253107	5.82E+24	1.03E-08	3.30E+57	0.138358
cg23099439	0.3994566	0.1726561	0.924182	0.032017
cg18355120	5.9262119	1.9274988	18.2205	0.0019021
cg04011671	0.3402519	0.0954064	1.213455	0.0965639
cg06048193	0.4746332	0.213354	1.055882	0.0677487
cg09754197	0.3544208	0.0696123	1.804481	0.2116178
cg27177997	16234114	1.50E-06	1.75E+20	0.2782334
cg25487404	0.6188124	0.3090356	1.239109	0.1754853
cg02583740	0.6680651	0.3500999	1.27481	0.2211383
cg02766539	0.8222415	0.3566079	1.895867	0.6460974
cg21411962	0.357559	0.0439667	2.90785	0.3361674
cg07184986	0.2113095	0.0796746	0.560426	0.0017868

cg14425054	0.3784099	0.1349482	1.061104	0.0647148
cg06858185	0.221516	0.066831	0.73423	0.0136918
cg10548163	1.7959249	0.615284	5.242045	0.2840216
cg01111006	0.5170139	0.2322836	1.150763	0.1060994
cg13930136	145.8363	0.5276138	40310.22	0.082378
cg01207684	0.255511	0.0739098	0.883318	0.0310833
cg14563754	0.0029606	8.60E-35	1.02E+29	0.8751278
cg00291213	0.811963	0.2493931	2.643553	0.729448
cg12988424	1.5857395	0.8870927	2.834619	0.1197777
cg22991232	2.0557668	0.8466615	4.991578	0.1113397
cg24343287	1.2225979	0.6327812	2.362184	0.5497774
cg01942127	0.1252632	0.0186447	0.841572	0.0325624
cg15643000	8.0456169	1.5765182	41.06007	0.0121635
cg16484473	1.6031047	0.5787641	4.440401	0.3639213
cg24141516	1.7252027	0.3860105	7.710474	0.4752975
cg13965224	0.0157735	0.0013493	0.184388	0.0009406
cg25035101	1.4295313	0.435587	4.691508	0.5556273
cg25635864	0.0419048	0.0076125	0.230675	0.0002669
cg10334741	0.273959	0.1064596	0.704996	0.0072572
cg27323975	2.9161005	1.1747157	7.238894	0.0210503
cg13649028	2.0016283	0.7205505	5.560354	0.183107
cg26402169	0.3782862	0.1420592	1.00733	0.0517329
cg25930229	3.3630308	1.4072981	8.03666	0.0063593
cg13298199	2.2167815	0.8113545	6.056687	0.1205869
cg24342864	1979.612	2.3609823	1659845	0.0270987
cg19708901	0.6659055	0.2772242	1.599536	0.3631332
cg04535578	1.7013402	0.8308472	3.483864	0.1461639
cg03424436	1.08E-13	2.12E-26	0.55187	0.0455237
cg19288979	8.75E-16	3.96E-28	0.001934	0.0168124
cg00853068	3.1099552	0.8192419	11.80582	0.0955085
cg11712990	1.1326427	0.6367397	2.014763	0.6716689
cg22334684	1.9192029	0.0026153	1408.358	0.8464541
cg09935271	0.1341695	0.0321	0.560793	0.0059126
cg08425229	0.2829361	0.0853682	0.937736	0.0389118
cg22165507	6.48E-05	1.77E-20	2.38E+11	0.5979044
cg03352106	4.324999	1.2317667	15.18601	0.0222979
cg05604819	5.4158711	1.8640256	15.73565	0.0019073
cg12028152	0.025887	0.0001495	4.481339	0.1646607
cg11693986	0.4327205	0.1706143	1.097488	0.0777214
cg19439071	0.2153349	0.058127	0.79772	0.0215501
cg17976588	5.487886	1.2226741	24.63199	0.026257
cg03583494	0.3437858	0.1433966	0.824209	0.016697
cg17044311	0.4642867	0.2460751	0.876001	0.017852
cg09556138	1.9025909	0.9739062	3.716839	0.0597576
cg09024126	3.5122752	1.0269495	12.01235	0.0452471
cg17298989	1.0093307	0.3887572	2.620526	0.984778
cg16032518	3.2084154	0.9277652	11.0954	0.0655444
cg07589202	0.4499068	0.1738428	1.164363	0.0997013
cg09292377	20.69329	0.6572945	651.477	0.0851539
cg26623837	1.5384981	0.5971498	3.96379	0.3722898
cg12936671	0.8597205	0.2043968	3.6161	0.8366188
cg18326819	1.676536	0.3516283	7.993591	0.5167142
cg14831085	2.0334663	0.8830818	4.682449	0.0953576
cg11721913	0.1571679	0.0477195	0.517645	0.0023448
cg05885676	12.14179	0.0232757	6333.784	0.4341792
cg16450654	1.2098402	0.4022593	3.63873	0.7345677
cg11024603	2.3673683	0.7930916	7.066564	0.1224678
cg19171919	2.3101865	0.2453288	21.75432	0.4642679

cg02722633	1.3330362	0.4966146	3.578199	0.5682714
cg27026786	3.4364156	0.6724053	17.56225	0.1380444
cg03374317	0.2955376	0.0559952	1.55982	0.1509531
cg03829194	0.1567019	0.0403128	0.609123	0.0074592
cg06578342	0.3230892	0.1416043	0.737172	0.0072639
cg17194154	1.9278518	0.8735947	4.25439	0.1040894
cg15790820	2.3695038	1.1425363	4.914109	0.020449
cg10538146	0.1156753	0.0239041	0.559769	0.0073354
cg04589828	0.1489911	0.040211	0.552046	0.0043851
cg11165752	2.4888317	0.8378267	7.393275	0.1007077
cg23168192	0.7357527	0.3813051	1.419682	0.3601814
cg08344524	0.3445403	0.1294551	0.916982	0.0328842
cg17578639	1.2060409	0.5863441	2.480684	0.610656
cg17306686	0.1824285	0.0581461	0.572355	0.0035403
cg07018260	0.0494803	0.0012784	1.915183	0.1070487
cg05136724	0.4247352	0.2315598	0.779064	0.0056644
cg08325124	0.8190452	0.3797843	1.766358	0.6107017
cg23613429	0.4988865	0.2463622	1.010251	0.0534049
cg16120244	7.3676222	0.3824375	141.9365	0.1857878
cg09103979	10.855895	0.2468146	477.4857	0.2167403
cg14717906	1.6198009	0.3481552	7.536165	0.538645
cg13459910	0.0097938	6.64E-06	14.43562	0.2139564
cg09663343	1.6869779	0.6770423	4.203421	0.2615833
cg20742385	2.098994	0.8241434	5.345885	0.1200709
cg00571935	4.45E+32	108426164	1.82E+57	0.0093274
cg05694921	2.8998862	0.9601228	8.758609	0.0590518
cg05134426	0.6944413	0.3606193	1.33728	0.2754205
cg24009656	0.410779	0.1806408	0.934115	0.0337905
cg20703614	1.4426642	0.362562	5.740481	0.6029811
cg03928154	0.2777061	0.0792927	0.972608	0.0451349
cg11918450	0.6046202	0.2697729	1.355086	0.2217137
cg04287259	2.798889	0.915144	8.560161	0.0711537
cg27314048	0.1548013	0.0470181	0.509665	0.0021509
cg04009446	3.6598362	1.2949659	10.34344	0.0143812
cg05552569	0.4216839	0.1258103	1.413377	0.161723
cg01014262	0.4361178	0.1224753	1.552955	0.2003074
cg02367723	0.6155002	0.2382021	1.590416	0.316346
cg03850117	0.0057749	9.26E-05	0.36003	0.0145072
cg12958778	0.2656687	0.0817151	0.863731	0.0275601
cg20745684	1.9263427	0.5459257	6.797255	0.3081483
cg18144247	0.2520972	0.080581	0.788685	0.0178893
cg02875787	1.914528	0.7512544	4.879063	0.1735985
cg07747241	83883.12	41.282959	1.7E+08	0.0035305
cg04175604	1.5685364	0.260448	9.446442	0.6231602
cg22159341	0.0905094	0.0075478	1.085349	0.0580466
cg27010159	1.9849255	0.937633	4.201995	0.0731855
cg25862072	0.5741505	0.2491841	1.322913	0.192616
cg00622318	0.3563336	0.1394494	0.910535	0.0311021
cg11278204	0.4468945	0.1472079	1.356685	0.1551514
cg07161062	0.0686516	0.0116785	0.403567	0.0030364
cg00082310	0.1278042	0.0236797	0.689784	0.0167697
cg26336265	3.5852199	1.0352599	12.41601	0.0439423
cg08314408	0.3510547	0.0260144	4.737349	0.430447
cg06918467	1.5364947	0.7230555	3.265055	0.2640809
cg27119904	2.476046	0.7123886	8.605982	0.1537471
cg14804706	0.3159617	0.0861748	1.15848	0.0822031
cg03388789	2.0665666	0.7912863	5.397159	0.138335
cg20618695	0.2780648	0.0742864	1.040837	0.0573636

cg24870340	1.153769	0.34077	3.906397	0.8181952
cg25707669	0.003386	2.25E-10	51042.49	0.4999938
cg11308649	0.3454037	0.1344257	0.887507	0.0272569
cg26717066	0.3633907	0.1566872	0.84278	0.0183495
cg01484075	3.4988395	1.3453804	9.099194	0.0102181
cg25828207	0.2754502	0.1085523	0.698951	0.0066504
cg11959694	1.2262353	0.6079163	2.473454	0.5688883
cg04724540	0.1035229	0.0200663	0.53408	0.0067446
cg22488849	1.8128804	0.7681193	4.278678	0.1745138
cg17297362	0.4866224	0.2447792	0.967408	0.0399294
cg16290689	7.0205713	1.4241555	34.60888	0.0166487
cg16531222	0.3293615	0.1021344	1.06212	0.0630157
cg27282281	5.4657515	1.4990278	19.92921	0.0100742
cg10516832	7.80E-30	4.01E-50	1.52E-09	0.0049239
cg05614028	0.4556967	0.1579902	1.314382	0.1459001
cg11168004	1.1423643	0.4841765	2.69529	0.7612025
cg27471008	4.2159022	0.8926766	19.91072	0.0692743
cg01758575	9.8092322	1.0210481	94.23751	0.0479278
cg08221781	1.2574289	0.2514203	6.288782	0.780311
cg20124188	0.1032357	0.0088779	1.200464	0.0696769
cg06796869	2.6062572	0.5289314	12.84208	0.239099
cg03403991	1.8832346	0.5922987	5.987811	0.2834797
cg03076790	12.036211	1.659943	87.27431	0.0138423
cg22895969	0.2884296	0.076961	1.080959	0.0651141
cg20805475	2.4368183	0.7603083	7.8101	0.1339172
cg01755342	2.5305979	0.788629	8.120327	0.1185751
cg22542373	0.3483668	0.1222165	0.992987	0.0484805
cg18786593	1.9753481	0.7792554	5.007344	0.1514545
cg08374799	1.2079828	0.5229395	2.790423	0.6582488
cg10542975	2.5766772	0.6743854	9.844913	0.166377
cg11511795	4.0689697	1.394999	11.86848	0.0101858
cg02482497	0.2897684	0.1361162	0.616868	0.001313
cg07561338	0.5395488	0.0255288	11.40331	0.6918211
cg01166399	0.4020039	0.1613115	1.001832	0.0504611
cg19188612	0.1826186	0.0674349	0.494545	0.0008222
cg18552482	0.3926998	0.1335668	1.154577	0.0893678
cg02272429	1.4271908	0.6006835	3.390927	0.4204651
cg11982564	1.8402429	0.6755544	5.012911	0.2329283
cg00406392	1.1404453	0.5359817	2.426604	0.733008
cg21771251	292.45568	7.65E-18	1.12E+22	0.8050456
cg07691146	0.3881214	0.157687	0.955299	0.0394487
cg24000437	0.4665769	0.1441928	1.509743	0.2032307
cg13162749	0.0001837	8.65E-08	0.389895	0.0277398
cg17579753	1.2645871	0.5141769	3.110175	0.6091745
cg08226752	1.5840678	0.5875527	4.270716	0.3633274
cg26796873	0.1634397	0.0532782	0.501378	0.0015394
cg10840704	0.3540418	0.1475038	0.849779	0.0201068
cg06623151	1.8658643	0.6426675	5.417186	0.251403
cg14494620	1.5480089	0.6286924	3.811612	0.3418777
cg16009558	2.3533963	1.1615546	4.768157	0.0175176
cg00634714	0.2187548	0.0316573	1.511618	0.1233127
cg03827705	0.7092881	0.3801792	1.323296	0.2803378
cg25652701	6.034778	0.9412429	38.69197	0.0579481
cg13662173	1.4084134	0.6578661	3.015246	0.3779027
cg05305434	1.6053888	0.7178768	3.590133	0.2490027
cg14805932	2.7887641	0.5026195	15.47335	0.2407545
cg20313963	0.2268587	0.0276639	1.860361	0.1670501
cg11599721	2.0715411	0.8211841	5.225725	0.1229125

cg09039698	26956.737	4.31E-10	1.69E+18	0.5290583
cg05492170	2.1256717	0.9401598	4.806077	0.0700302
cg20414749	1.422767	0.4880001	4.148085	0.5183747
cg12436568	4.4755238	0.9725307	20.59607	0.0543295
cg19248557	1.2276133	0.4145363	3.63547	0.71122
cg19771372	0.7560484	0.2343443	2.439186	0.6398282
cg02977524	0.147236	0.0305379	0.709885	0.0169915
cg00866690	0.2360532	0.0738392	0.754628	0.0149018
cg05419798	1.927531	0.6858081	5.417515	0.2132645
cg13014803	1.4658976	0.5557171	3.866816	0.4396194
cg08850264	0.8923955	0.4381647	1.817512	0.7537549
cg23434592	0.4626179	0.218302	0.980364	0.0442496
cg21363377	0.2991495	0.1320936	0.677477	0.0038087
cg21604972	0.9446526	0.2283049	3.90867	0.9373654
cg16856833	1.1831506	0.5777355	2.422987	0.6456251
cg10914467	0.1398385	0.0276751	0.706584	0.0173045
cg02339850	0.2335923	0.0724906	0.752723	0.0148604
cg05708497	0.0969499	0.0294128	0.319564	0.0001258
cg06806638	1.0847333	0.4432564	2.65455	0.8586243
cg22533099	7.3863893	0.3946825	138.2345	0.1809187
cg23234811	17.290794	3.1433517	95.11234	0.0010506
cg11313862	0.5364303	0.2638391	1.090655	0.0853825
cg16837973	2.6034859	0.5420219	12.50529	0.232067
cg14787267	0.4080753	0.1634352	1.018908	0.0548778
cg16135989	1.1002466	0.5900279	2.05167	0.7638002
cg27587095	0.1972336	0.0654055	0.594768	0.0039444
cg13579960	5.79E+26	1.76E-19	1.90E+72	0.2491499
cg20378070	0.1836587	0.0646224	0.521963	0.001473
cg02023345	1.4455019	0.7305294	2.860221	0.2899635
cg19421371	1.4863193	0.3462083	6.38097	0.5939628
cg15724945	2.5284894	1.1102566	5.758361	0.0271721
cg24725640	1.9418969	0.8832685	4.269329	0.0987084
cg14719722	1.8649808	0.6656566	5.225147	0.2357397
cg22464570	0.2636221	0.0389522	1.784153	0.1717652
cg17891421	0.9235332	0.0007381	1155.546	0.9825586
cg25940946	0.1672428	0.0565156	0.49491	0.0012351
cg10134963	0.8623305	0.1263868	5.883634	0.8798363
cg10501049	0.0929097	0.0046163	1.869952	0.1208251
cg20980321	0.6356199	0.157244	2.569335	0.5248694
cg16578636	1.6130853	0.7318653	3.555359	0.2356975
cg22103601	1.4504632	0.6406457	3.283942	0.3724133
cg20255933	3.8105558	1.06679	13.61124	0.0394465
cg07061355	0.5844503	0.2969115	1.150451	0.1201008
cg26985447	0.3850056	0.0447639	3.311362	0.3846395
cg08790581	0.750785	0.1173137	4.804878	0.7621579
cg21880392	0.3206777	0.1558945	0.65964	0.0019977
cg03363904	2.0950446	0.2423156	18.11362	0.5015907
cg21287936	0.2296376	0.0497785	1.059362	0.0592896
cg00090521	2.8324399	1.1508888	6.970887	0.0234633
cg17727597	3.3068091	1.1113134	9.839696	0.0315816
cg11203827	0.4656552	0.2259072	0.95984	0.0383559
cg02009766	0.2981215	0.0893316	0.994904	0.0490367
cg00547727	101779.47	0.002864	3.62E+12	0.1936488
cg22505898	1.5975432	0.6386999	3.995843	0.3165769
cg09747169	2.1773743	0.9469485	5.006565	0.0670037
cg14014796	1.6700288	0.8776191	3.177912	0.1182204
cg06108323	0.2800289	0.145636	0.53844	0.0001357
cg10099813	0.4502934	0.2243984	0.90359	0.0247516

cg16640061	167.36624	2.97E-09	9.44E+12	0.6851971
cg23211791	0.062506	0.009898	0.394727	0.0031926
cg16731318	0.2672527	0.034739	2.05602	0.2049469
cg00883831	2.6061286	0.8060664	8.425989	0.1096261
cg06778853	0.3767578	0.1428835	0.993441	0.0484653
cg24971846	1.4652499	0.1672959	12.83329	0.7300594
cg24309739	0.5095259	0.2780843	0.93359	0.0290815
cg26250228	0.1650338	0.0521282	0.522483	0.002184
cg09556952	0.6436122	0.3048565	1.358792	0.247764
cg14774117	2.7499621	0.9927831	7.617265	0.0516511
cg14826683	0.6800809	0.289877	1.595539	0.3755479
cg12989104	0.977368	0.3370021	2.834547	0.9663886
cg10442735	0.457201	0.197386	1.059005	0.0678215
cg12973168	1.861E+18	1.18E-09	2.93E+45	0.1879667
cg11779118	0.5159135	0.2781957	0.956761	0.0357082
cg19095187	2.4190803	1.1353457	5.154333	0.0220876
cg21046148	2.139603	1.0723637	4.268982	0.0309122
cg11908155	2.5862063	0.4551458	14.69521	0.2837394
cg06576532	1.2135894	0.3870547	3.805145	0.7398798
cg14648534	0.3802132	0.073353	1.970773	0.2493777
cg13281814	0.951425	0.0084063	107.6817	0.9835347
cg01044662	0.1095234	0.0182242	0.658213	0.0156474
cg25685510	0.6709178	0.1981859	2.271256	0.5212156
cg25250998	1.4399003	0.7105879	2.917743	0.3116462
cg25151834	0.0032725	1.77E-05	0.605154	0.0316696
cg14414203	0.7920678	0.410005	1.530156	0.4877773
cg11682724	0.56921	0.3156611	1.026417	0.0610292
cg21245652	1.8329312	0.6598111	5.091816	0.2451011
cg27495908	3.0741541	1.1426388	8.2707	0.0261465
cg13502592	22.320638	2.0463106	243.4679	0.0108562
cg11737757	0.240175	0.0830857	0.694272	0.0084458
cg22424284	0.6351297	0.2629839	1.533895	0.312971
cg05363871	0.157635	0.0359991	0.690262	0.0142093
cg09690219	0.3037542	3.63E-06	25396.48	0.8367506
cg15665276	2.0566591	0.9011018	4.694083	0.0867806
cg01880908	0.2464685	0.1048594	0.579316	0.0013184
cg22987011	0.3510035	0.1263775	0.974884	0.0445617
cg27144224	0.0150106	0.0006699	0.336347	0.0081261
cg14269716	0.4134674	0.1772429	0.964526	0.0409992
cg05705223	17.083271	0.4461753	654.0886	0.1270041
cg08610201	1.793E+18	0.0008637	3.72E+39	0.0932917
cg19172447	2.7417299	0.0039499	1903.105	0.7625452
cg08498987	2.0051165	0.6485358	6.199337	0.227036
cg20485191	0.3701002	0.1290829	1.061133	0.0643782
cg26228460	0.3709645	0.1095393	1.256303	0.1110832
cg24620761	1.7864471	0.7258188	4.396956	0.2067233
cg18649319	2.0666992	0.5864028	7.283808	0.258684
cg23808165	0.4252816	0.1781389	1.0153	0.054134
cg05563385	2.7592375	1.1176926	6.811704	0.0277159
cg26244109	0.6154496	0.0468829	8.079239	0.7117495
cg15813951	9.23E-05	1.72E-25	4.96E+16	0.7028533
cg24594293	0.2645955	0.1241607	0.563872	0.000573
cg15042866	2.8364638	1.1427206	7.040677	0.0246032
cg07521193	5.3935248	1.5779522	18.43536	0.0072024
cg22546168	0.2435951	0.0638328	0.929593	0.038752
cg13228421	3.3618158	0.603252	18.7348	0.1665646
cg11600721	21218.94	5.68E-09	7.93E+16	0.4999835
cg15434599	2138.2499	9.6485436	473865.6	0.005393

cg00300627	0.279377	0.0951741	0.820092	0.0202891
cg14068657	1.8191011	0.8597943	3.848745	0.1176106
cg20600704	152.91112	2.2344253	10464.35	0.0196559
cg26298599	0.7326873	0.2615344	2.05262	0.5540007
cg07233933	0.5273851	0.2408529	1.154792	0.1095871
cg14545570	0.9072856	0.3185001	2.584512	0.8554503
cg15133564	0.1540913	0.0632504	0.375399	3.85E-05
cg23564664	0.3019413	0.0781478	1.166616	0.0824761
cg26347746	1.2621898	0.3911713	4.072699	0.6968487
cg05846798	0.3301265	0.1132159	0.962616	0.0423827
cg02886263	0.699192	0.3606394	1.355563	0.2894444
cg16083439	0.1672242	0.0229811	1.216826	0.0773689
cg08651674	8.7825569	1.79E-10	4.31E+11	0.8626577
cg03577157	0.3464356	0.0525003	2.286038	0.2708452
cg00958381	0.0005791	1.06E-14	31657316	0.5545863
cg09679945	1.3600239	0.6202094	2.982323	0.4427448
cg04318704	0.0876293	0.008355	0.919076	0.0423223
cg18071162	0.5609058	0.2307315	1.363556	0.2020403
cg27100370	0.413964	0.2170106	0.789667	0.0074371
cg03641062	6.0906112	0.5317431	69.76215	0.1464232
cg02389949	1.00901	0.4465981	2.279681	0.9827917
cg13733654	6.7099596	1.5627843	28.80983	0.010452
cg06073041	4.8628039	0.0179069	1320.542	0.5801662
cg11310863	0.4656434	0.2338888	0.927038	0.0295844
cg07950002	0.5951793	0.2818902	1.256654	0.1735671
cg10779126	1.737739	0.0957542	31.53632	0.7086644
cg22590522	0.2045446	0.0684889	0.61088	0.0044712
cg27104671	0.4683986	0.1931756	1.13574	0.093288
cg04031383	5.11E-12	2.84E-26	919.5964	0.1205465
cg14126884	0.2656103	0.1224728	0.576037	0.0007895
cg09805271	0.5817787	0.2667476	1.268864	0.1733702
cg09076720	0.4735915	0.1870053	1.199372	0.1149107
cg26688527	1.8293542	1.0154808	3.29552	0.044313
cg24279243	0.1899127	0.0732597	0.492315	0.0006307
cg17215154	0.1429315	0.0388557	0.525776	0.0034187
cg02736280	0.1143019	0.0446726	0.29246	6.04E-06
cg17468855	0.1495762	0.0456584	0.490009	0.0017
cg15961901	0.7373318	0.248395	2.188684	0.5830602
cg13193239	2.192977	0.8173464	5.883855	0.118895
cg16466652	0.3479482	0.141783	0.853896	0.0211784
cg03982544	0.3142977	0.0556923	1.773729	0.1898955
cg16680704	1.2084557	0.4515178	3.234347	0.7062071
cg17875320	5.2341702	1.075213	25.4801	0.0403869
cg10867349	0.7220158	0.36036	1.446628	0.358304
cg08763617	0.3236672	0.1447116	0.723926	0.0060222
cg24666355	0.4648075	0.1422192	1.519105	0.2048107
cg27629782	1.8469242	0.1500125	22.73896	0.6319605
cg07953015	0.4909489	0.2548214	0.945881	0.0334822
cg02278959	0.2329732	0.0281392	1.928859	0.1767489
cg15867125	1.9544313	0.8592986	4.445255	0.1099799
cg24005158	25.03069	1.7907014	349.8827	0.0167154
cg11650479	0.0212348	0.0008994	0.501368	0.0169423
cg12126344	0.2608163	0.0721109	0.943341	0.0404732
cg07798980	4.2474416	0.790178	22.83126	0.0918885
cg07565551	1.8838459	0.6870079	5.165698	0.2184953
cg13904562	1.5521199	0.6621326	3.638359	0.3118139
cg27571196	9.7037671	1.6856138	55.86279	0.01094
cg06246094	2.5698364	0.7638208	8.646085	0.1273272

cg24507760	0.3734382	0.1783372	0.78198	0.0089978
cg11422337	1.183E+12	2.72E-27	5.16E+50	0.5402692
cg03183800	1.3051134	0.4861948	3.503372	0.5971111
cg13001868	2.997919	0.9164581	9.806796	0.0694176
cg14403090	0.5113303	0.2351716	1.111778	0.090536
cg03625515	0.437293	0.154662	1.236407	0.1188091
cg00097038	0.6168808	0.3029307	1.256201	0.1830739
cg00528572	1.5644614	0.8566357	2.857153	0.1452826
cg12381575	3566223.1	301.0884	4.22E+10	0.0016183
cg17189494	0.1949584	0.019916	1.908452	0.1601115
cg11104088	2.9699036	0.8332576	10.58535	0.0932184
cg19196826	1.319413	0.4883121	3.565037	0.5846792
cg09977376	0.0048635	0.000164	0.144224	0.0020725
cg03177666	5.9999173	1.3416979	26.83093	0.0190478
cg20389678	0.7053764	0.1969368	2.526475	0.5918399
cg01538731	0.2134114	0.0889536	0.512002	0.0005416
cg05836043	4.4040127	1.078117	17.99	0.0389497
cg10753836	1.84566	0.7739456	4.401421	0.1669522
cg27623214	1.9398438	0.6854265	5.490004	0.2119024
cg22598426	2.2587528	0.9919841	5.143192	0.0522833
cg19481686	0.6111467	0.2245761	1.663134	0.3350258
cg02173030	2.5195633	0.4228476	15.01297	0.3102199
cg10512886	4.7885187	0.6943259	33.02471	0.1119059
cg11012046	1.8180624	1.046385	3.158829	0.0339361
cg20649665	45.131812	5.96E-06	3.42E+08	0.6373575
cg00242597	1844291.8	991.93077	3.43E+09	0.0001724
cg06922635	0.3186488	0.1451975	0.699303	0.0043465
cg09125300	0.376042	0.1548879	0.912967	0.0306825
cg19699170	0.3252515	0.1301588	0.812765	0.0162333
cg01196842	1227301.5	3.29E-07	4.57E+18	0.3424648
cg08415417	3.04E+20	11044.472	8.34E+36	0.0146056
cg09480515	0.5418969	0.0084641	34.69401	0.7728008
cg09309024	3.1695077	0.4475142	22.44796	0.2481086
cg00541718	0.2088643	0.0952089	0.458195	9.34E-05
cg17675088	0.2918945	0.1226248	0.694822	0.0053891
cg10149455	1.8526779	0.4197558	8.177173	0.4156375
cg07759042	2.1412986	0.4140925	11.07279	0.3637413
cg15604241	0.4563234	0.2163128	0.962639	0.0394043
cg06742021	0.3833513	0.1109506	1.324537	0.1296045
cg05866021	1.0028812	0.4764711	2.110874	0.9939545
cg06389703	0.2218321	0.0194642	2.528203	0.2251713
cg10020892	1.4838175	0.8428134	2.612339	0.1715011
cg24268161	0.401721	0.1324417	1.218497	0.1072009
cg03300649	3.0186343	0.8254376	11.03918	0.0949227
cg16600996	0.0186117	0.0017022	0.203502	0.0010964
cg08011328	116712477	3.64E-12	3.74E+27	0.4175938
cg16434190	0.2348261	0.054082	1.019624	0.0531102
cg22392150	8.4246053	1.4918615	47.57411	0.0158276
cg04978078	1.1537754	0.517641	2.571662	0.7265046
cg10091487	11.0972	0.3586104	343.4029	0.1693356
cg18142353	0.020578	0.0012984	0.326141	0.0058742
cg15439078	5.8999096	1.045231	33.30262	0.0444246
cg12430007	1.8724146	0.845901	4.144618	0.1218244
cg20723705	0.7988514	0.3997411	1.596442	0.5249368
cg09435090	0.0083886	0.0001117	0.629695	0.0300155
cg05169988	1.4592211	0.4302515	4.949026	0.544202
cg17831440	0.4405919	0.1972241	0.984267	0.0456482
cg06906952	0.6614654	0.3109456	1.407116	0.2832085

cg20478514	2.4805224	0.6769589	9.089165	0.1703348
cg25622125	0.2532064	0.1119367	0.572765	0.0009736
cg19764143	2.4166633	1.2116796	4.819972	0.0122428
cg02504690	2.236062	0.7595607	6.582718	0.1440847
cg15531450	0.071321	0.0175804	0.289338	0.0002193
cg19186806	0.491531	0.218254	1.10698	0.0864184
cg08869160	0.3051465	0.0765114	1.217	0.092624
cg15071854	0.3813847	0.1190973	1.221306	0.1045263
cg16944405	6.897164	0.0481788	987.3813	0.4457739
cg20982052	0.3750821	0.1530977	0.918933	0.0319623
cg25214158	1.5712255	0.7398361	3.336887	0.2396592
cg24793228	1.9504566	0.0260103	146.2605	0.7616726
cg03767565	2.63E-07	4.46E-13	0.154674	0.0253975
cg15173428	2.9299546	0.7981395	10.75581	0.1052005
cg27245646	0.2361468	0.0752276	0.741288	0.0134028
cg13046257	0.6945201	0.3528215	1.367145	0.2914483
cg14535717	0.0150895	9.10E-05	2.502282	0.1077843
cg07974367	12.958316	1.872614	89.67035	0.0094429
cg08302003	0.0675347	1.22E-05	373.0388	0.5398588
cg00035969	1.6059746	0.6404178	4.027299	0.3125284
cg19256314	0.4129078	0.1725697	0.987965	0.0469041
cg27663938	2.399306	0.9638492	5.972583	0.0599945
cg05839365	0.8048014	0.2181438	2.969166	0.7443944
cg06426831	0.6264273	0.2321703	1.690187	0.3557001
cg12116939	63603.26	1.68E-24	2.40E+33	0.7418197
cg10138970	3.4563005	1.0987353	10.87251	0.0339221
cg14011734	3.74E-07	9.70E-23	1.44E+09	0.4189949
cg08815110	2.2194706	0.7180447	6.860366	0.1661456
cg21123740	1.3220502	0.656634	2.661782	0.4342676
cg22289643	2.0768257	0.7307046	5.902803	0.1702875
cg09848445	4.2637769	0.0130501	1393.073	0.6234511
cg00506540	0.4781735	0.1361101	1.679889	0.2498032
cg12161887	0.4472829	0.2113144	0.946751	0.0354665
cg02350790	0.3093596	0.1180875	0.810445	0.0169548
cg13353733	0.2680065	0.0825458	0.870153	0.0284197
cg08934799	3.3144782	0.8100097	13.56251	0.0955417
cg08830157	2.5048888	0.8837154	7.100101	0.0840943
cg17206748	4.2315567	1.0142014	17.65534	0.0477808
cg09244312	0.8668961	0.3666658	2.049574	0.7449158
cg26385618	2.472278	0.6122252	9.983513	0.2037321
cg01117213	30.829058	0.0017601	539989.8	0.4916261
cg27376339	2.1846166	0.9205396	5.184513	0.076362
cg25874953	0.167107	0.0594585	0.469651	0.0006902
cg08367801	0.5715194	0.2977845	1.096882	0.0925772
cg08882707	5.2772007	1.2666129	21.98687	0.022338
cg00956142	0.2199286	0.0664196	0.728228	0.0131708
cg06559878	2.0495892	0.7963083	5.275364	0.1368123
cg09626867	0.3906464	0.1519532	1.004287	0.051047
cg16506172	1.513E+11	0.0210785	1.09E+24	0.0883014
cg25858249	1.0893228	0.481821	2.46279	0.837131
cg09059548	9.59E-19	6.36E-38	14.45771	0.0655622
cg11614513	0.0947695	0.0090635	0.990928	0.049117
cg13776560	1.1384426	0.4900375	2.644801	0.7630454
cg15114474	0.3921275	0.1610332	0.954859	0.0392372
cg06699669	2.4709202	0.6924292	8.817431	0.1634121
cg07011163	1.1473237	0.5504368	2.391467	0.7138125
cg14898611	3.2420092	0.5191105	20.24737	0.2082236
cg24996278	889553.63	8.44E-10	9.38E+20	0.4376571

cg18385440	2.8582109	0.8834819	9.246787	0.0795752
cg08071609	1.8139737	0.9193985	3.578971	0.0858715
cg05384957	2.2249504	0.8125821	6.092189	0.1196764
cg01816304	0.0016431	8.97E-06	0.301036	0.0158854
cg10522125	2.1323838	0.9639747	4.716992	0.0615689
cg24268004	3.05E-06	5.67E-19	16454191	0.3958574
cg17862947	45.33097	0.4456497	4611.014	0.1058238
cg18714469	0.0090944	6.27E-06	13.18142	0.2056641
cg09093150	2.243188	1.1795801	4.265833	0.0137551
cg26796245	1.9769128	0.8494369	4.600912	0.1138002
cg18489675	2.7224172	1.2275226	6.037816	0.0137246
cg24757562	1.1509198	0.5582347	2.372866	0.70338
cg04364311	0.2830172	0.1192405	0.671741	0.0042075
cg03641497	0.2586184	0.0393602	1.699267	0.1591374
cg06713671	0.2770151	0.1057594	0.725585	0.0089778
cg04916802	2.123275	0.8602963	5.2404	0.1023621
cg10090116	0.1042726	0.0136174	0.798448	0.0295045
cg06402675	1.5182076	0.6952331	3.315369	0.294747
cg27086874	0.098323	0.0224741	0.430159	0.0020683
cg25909854	0.0066756	0.000261	0.170723	0.0024554
cg18590092	2.3127222	0.414517	12.90341	0.3391147
cg26746037	1.4031675	0.5172694	3.806293	0.5058687
cg08923054	3.5284557	0.4333081	28.73244	0.2386487
cg07613391	2.3945662	0.7020895	8.166975	0.1630345
cg08555556	1.5433672	0.7077441	3.365598	0.2752885
cg16551520	1.8564324	0.9490062	3.631526	0.0707498
cg13967908	0.2738993	0.1239464	0.605268	0.0013693
cg03585912	2.1347813	0.6946475	6.56058	0.185535
cg23530268	2.5694934	0.9976391	6.61792	0.0505751
cg12673429	1.08046	0.5420683	2.153592	0.8259497
cg26296969	0.0035558	0.0001194	0.10592	0.0011283
cg03636488	2.5854298	0.6936923	9.63604	0.1570343
cg20613400	0.0222191	0.0009188	0.537349	0.0191759
cg08163578	2.10E-05	2.03E-09	0.216879	0.0223709
cg00352195	9.50E-09	2.46E-40	3.67E+23	0.6186332
cg09214398	0.2378718	0.1158081	0.488593	9.22E-05
cg16862319	0.7737056	0.4115285	1.454626	0.4257275
cg08892078	3.7131501	0.6479168	21.27971	0.1408179
cg14532755	0.1505529	0.0537171	0.421955	0.0003171
cg07700234	9.44E-09	1.06E-19	842.5186	0.1509034
cg19443920	0.1968406	0.0751656	0.515478	0.000936
cg08347042	1.2025679	0.5840608	2.47606	0.6166572
cg13028471	0.3833868	0.1302494	1.128492	0.0817702
cg13927756	1.208099	0.2737139	5.332221	0.8029273
cg25181381	3.1341329	0.9794752	10.02862	0.0542272
cg01891736	0.4776101	0.2078442	1.097511	0.0817227
cg03188580	3.3331468	0.4291769	25.88646	0.24967
cg09223940	0.1798383	0.0723385	0.44709	0.0002221
cg00267142	3.0397957	0.9098189	10.15626	0.0708548
cg01109643	2.8827839	1.2960452	6.412155	0.0094391
cg10126903	3.0549001	1.1139833	8.377518	0.0300311
cg09632271	1.0069296	0.4015938	2.524708	0.9882522
cg25814969	2.6520116	1.2005899	5.858092	0.0158617
cg00684075	0.0683646	0.0081715	0.571957	0.0133066
cg22514682	2.0362105	0.9511187	4.359238	0.0671119
cg11852218	11.27218	2.7242963	46.64032	0.0008283
cg12597169	3.0496581	1.0703265	8.689324	0.0368716
cg06240450	1.4280815	0.7583176	2.689397	0.2698794

cg06091535	3.1294856	0.9831819	9.961208	0.0534519
cg27485730	3.7296803	0.7772969	17.89601	0.0999482
cg13600622	1.3705814	0.4832798	3.886969	0.5533677
cg27235955	3.5081932	1.3681184	8.995873	0.0089924
cg23316449	1.5610952	0.7557562	3.224609	0.2288384
cg19921389	18.2044	0.4518189	733.4801	0.1238835
cg04329870	1.898591	0.808014	4.46112	0.1413231
cg26886066	0.1832022	0.0730544	0.459425	0.0002968
cg03184290	1.4600118	0.7991373	2.66742	0.2184132
cg13823169	1.8986222	0.548116	6.576648	0.311814
cg07013787	0.5673483	0.252815	1.2732	0.169347
cg18663307	2.4881611	0.7425948	8.336909	0.1395248
cg21948027	3.1190659	0.7009471	13.87918	0.1353162
cg04305082	0.4587431	0.0895492	2.35005	0.349845
cg19770671	0.6103268	0.2721458	1.368747	0.23083
cg09511126	1.8535922	0.8171268	4.204738	0.1397565
cg11367159	1.3588359	0.4646961	3.973425	0.5754155
cg23777302	0.3899739	0.1270377	1.197122	0.0998549
cg06958535	2.6506389	0.7729872	9.089266	0.1210404
cg07253384	2.1803098	1.0186001	4.666945	0.0447043
cg16832958	6.93E-06	1.54E-11	3.113855	0.0736265
cg12278179	1.7490318	0.6433168	4.755219	0.2732781
cg00853940	1.9672363	0.8650326	4.473841	0.1065067
cg11853320	1.8246473	0.8908893	3.737095	0.1001541
cg02646021	2.3602314	0.7812029	7.130916	0.1279427
cg13023584	19.927528	0.7818745	507.8902	0.0701361
cg13560436	0.3499767	0.1046116	1.170842	0.0883853
cg27553955	0.9509819	0.2879559	3.140642	0.9342847
cg23929682	3.6496403	1.2214992	10.90453	0.0204364
cg13827361	0.3466155	0.172563	0.696223	0.0029062
cg20903900	0.2997245	0.1127356	0.796863	0.0157302
cg10181820	0.9993151	0.2057982	4.852476	0.999322
cg10950111	0.2182736	0.0974784	0.488758	0.0002151
cg11040363	1.9761905	0.7966222	4.90236	0.1417084
cg11461670	0.2433034	0.0548134	1.079964	0.0630558
cg01680573	0.5881349	0.1385686	2.496256	0.4717289
cg26926138	3.1781782	1.0548721	9.575395	0.039889
cg19529577	1.0315243	0.5014675	2.121857	0.9327838
cg01569709	1.7769783	0.742414	4.253222	0.1966739
cg14032572	1.6430611	0.8780254	3.074683	0.120397
cg06837799	1.3701828	0.6375513	2.944705	0.4197628
cg00420526	0.3610234	0.1115004	1.168945	0.089214
cg04368939	0.2147827	0.0732872	0.629463	0.0050516
cg09028651	0.215095	0.0420452	1.100383	0.0650221
cg20647257	0.4685451	0.18756	1.170476	0.1045935
cg05296180	83.064325	2.1859849	3156.327	0.0172492
cg10758466	0.1904291	0.063599	0.570186	0.0030368
cg03032497	3.5107448	1.3321805	9.251996	0.0110823
cg23320862	1.8650768	0.7663613	4.538997	0.1695782
cg06430061	0.4333223	0.1338803	1.402508	0.1628646
cg21641573	15.895928	0.3071618	822.63	0.1695227
cg12363472	3.253535	0.9392977	11.26958	0.0627202
cg09763644	2.4120183	0.7854416	7.407084	0.1240305
cg02067431	0.0001024	4.56E-13	22989.06	0.3490805
cg21096718	0.3625317	0.158287	0.830323	0.016407
cg23489038	2.4140261	1.1121773	5.239742	0.0258237
cg24897392	0.476375	0.1700402	1.334586	0.1582904
cg14178364	1.1483955	0.5747252	2.294683	0.695231

cg09068993	0.3956139	0.0046991	33.30678	0.681815
cg04550950	0.5217267	0.2280891	1.193388	0.1232757
cg18410444	320884554	5.14E-09	2.00E+25	0.320872
cg00959635	1.8033873	0.8580484	3.790236	0.1197117
cg14763173	8.28E-07	1.97E-13	3.479842	0.0719055
cg12762862	0.4669762	0.2306291	0.94553	0.0343812
cg17209188	0.5979993	0.2078285	1.720665	0.3403298
cg07431888	0.2894614	0.1220517	0.686495	0.0048978
cg01644285	0.6259981	0.2034324	1.926308	0.4140587
cg13446199	1.471326	0.5942947	3.642638	0.4037777
cg01448132	1.2002574	0.5085173	2.832977	0.6769784
cg04247568	0.4114433	0.2025695	0.835692	0.0140317
cg23743114	4.4925099	1.0958805	18.41683	0.0368739
cg21356998	1.8901942	0.5116913	6.982401	0.339594
cg11429292	0.079741	0.0144775	0.439206	0.003671
cg10666224	10.743565	0.4448155	259.4878	0.1439169
cg07306331	0.3020181	0.1179954	0.773038	0.0125317
cg01970923	0.4846726	0.2420996	0.970293	0.0408432
cg00164949	1.4479928	0.7318113	2.86506	0.2876933
cg05628049	0.1435578	0.0389493	0.52912	0.0035414
cg10711528	0.5579232	0.2634267	1.18165	0.1275002
cg15476479	1336.7143	1.81E-07	9.89E+12	0.5347293
cg04265492	0.3457577	0.1216367	0.982831	0.0463212
cg08239858	1.4296945	0.6648278	3.07452	0.3601877
cg00757413	0.1343437	0.0110702	1.630338	0.1149868
cg03146993	1.3415383	0.7448244	2.416308	0.3277448
cg01427976	575.97297	1.20E-07	2.77E+12	0.5763263
cg18808929	0.2930698	0.099944	0.859381	0.0253481
cg07514158	0.0668782	0.0097606	0.458241	0.0058747
cg10978753	0.1769148	0.0441763	0.708499	0.0144147
cg11930400	1.8997316	0.8854724	4.075768	0.099423
cg21214293	3.1931619	1.2576722	8.107266	0.014597
cg19997861	3.926444	0.2934003	52.54583	0.3013951
cg16440058	0.4680083	0.1961262	1.11679	0.0870734
cg11463271	0.3923698	0.1772079	0.868776	0.021065
cg05784847	0.4106053	0.1823297	0.92468	0.0316328
cg15047134	1.941742	0.6775772	5.564476	0.2166982
cg05437692	0.9581962	0.411187	2.232901	0.9211933
cg26549601	0.6809328	0.0873551	5.307872	0.7137754
cg16325174	0.5075369	0.2146324	1.200162	0.1224793
cg15490784	0.2119539	0.0712475	0.63054	0.0052861
cg00161024	1.432E+09	8.68E-06	2.37E+23	0.2068804
cg27188056	1.1802328	0.4784441	2.911415	0.7190666
cg01761968	2.638798	1.1133711	6.254209	0.0275326
cg24457649	2.3388123	0.7804082	7.009208	0.1292117
cg10442355	0.1741757	0.0623512	0.486554	0.0008547
cg09561280	0.4547549	0.0010558	195.8789	0.7990104
cg15834592	3.278443	0.4106106	26.17611	0.2626268
cg26912278	1.579E+11	3.12E-19	7.98E+40	0.459957
cg17782713	0.4884556	0.2305385	1.03492	0.0614332
cg20026673	1.5392946	0.4853244	4.882153	0.4639253
cg08160706	0.4202761	0.2034425	0.868216	0.0191953
cg17237962	188.55039	0.1009398	352202.5	0.1727972
cg04074481	0.7262352	0.3512519	1.501537	0.388063
cg04774469	0.2928476	0.108577	0.789851	0.0152672
cg22364465	0.6122326	0.2318334	1.616802	0.3220431
cg20178075	0.5219497	0.2181489	1.248833	0.1440876
cg12044828	1.6286394	0.6370324	4.163786	0.3084823

cg21575861	290.23497	1.64E-26	5.13E+30	0.8643173
cg09287328	1.4215863	0.3552343	5.688942	0.619063
cg24531489	0.5706119	0.2537053	1.283371	0.1748861
cg19280014	359.37871	9.35E-23	1.38E+27	0.8385608
cg00689225	1.4342	0.6674044	3.081984	0.3555215
cg12043732	0.3451352	0.1425566	0.835586	0.0183675
cg06069100	0.2138904	0.0913865	0.500611	0.0003783
cg09861992	1.8768182	0.5646018	6.238816	0.304301
cg14326671	1.8512551	1.0335449	3.315913	0.038367
cg18246056	0.8588288	0.0077281	95.44161	0.9495122
cg16403860	1.8556135	0.3159748	10.89739	0.4936938
cg26165286	0.3663001	0.1601909	0.837599	0.0173166
cg05359028	0.0167975	0.0001844	1.529804	0.0758526
cg07774964	0.278747	0.0841404	0.923456	0.036594
cg02786012	0.3695884	0.1494715	0.913857	0.0311625
cg07440376	0.1558947	0.0001764	137.7473	0.5912947
cg18267506	2.06E-07	7.72E-16	54.94852	0.1198891
cg14510504	80.647596	4.3415567	1498.088	0.0032312
cg05827190	1.6489575	0.6383631	4.259426	0.3016262
cg03977657	0.7460381	0.2459253	2.263178	0.6048495
cg19789466	1.2048498	0.5856975	2.47852	0.6125962
cg24603969	1.0071498	0.0772444	13.1317	0.9956614
cg00718242	14.439936	0.8738664	238.6083	0.0620765
cg16727774	1.0696878	0.4500508	2.542451	0.8787854
cg12074182	2.2418029	0.8220087	6.113902	0.1147816
cg21946374	3.1308219	0.737192	13.29646	0.1219261
cg06531800	25284.894	0.0012906	4.95E+11	0.2366506
cg27369242	0.0373929	0.0076707	0.182282	4.78E-05
cg16606773	0.2454021	0.1027771	0.58595	0.0015578
cg06808305	0.1963967	0.0732298	0.526721	0.0012223
cg24480453	1.6196577	0.6517904	4.024747	0.2991232
cg07091412	2.1970558	0.9918281	4.866825	0.052411
cg11980819	1.9295165	0.7785907	4.78176	0.1557621
cg11774314	9.52E-10	6.81E-22	1331.544	0.1454532
cg18380490	4.56E-08	1.51E-24	1.38E+09	0.3826437
cg24220279	2.4012742	1.1216073	5.140941	0.0241054
cg20029881	0.6366327	0.2799829	1.447593	0.2813014
cg00127894	0.099738	0.0042663	2.331712	0.1517146
cg22831256	748.12578	0.0147605	37918205	0.231211
cg01797106	1.2183502	0.5996556	2.475383	0.5850366
cg15171791	0.2465186	0.1252154	0.485335	5.09E-05
cg15065854	0.0992687	0.0273589	0.360186	0.0004433
cg20403557	2.5615168	0.9879578	6.641345	0.0529854
cg14103123	3.1095337	0.1510203	64.02581	0.4622815
cg20300500	0.2709412	0.0373758	1.964081	0.1963341
cg25549619	0.8020931	0.4038435	1.593076	0.5287636
cg18276155	0.7446104	0.3821209	1.450966	0.3862826
cg23601416	0.1892722	0.062066	0.577191	0.0034331
cg05376601	821.10317	0.0049054	1.37E+08	0.2741765
cg16549711	1.4204376	0.611145	3.301415	0.4147201
cg01042465	0.1785736	0.0585683	0.544468	0.0024552
cg17745697	2.5459766	0.5878901	11.02586	0.211436
cg09907439	0.2096606	0.0852052	0.515902	0.0006724
cg18016254	2.3827802	0.9411899	6.032408	0.0669404
cg13420075	0.1993068	0.066298	0.599161	0.0040779
cg25253677	0.5019269	0.1419466	1.774826	0.2847649
cg13540795	0.0427455	0.0048804	0.374393	0.0044091
cg07825294	0.2924535	0.1329869	0.643139	0.00223

cg05957749	0.5427659	0.224649	1.311356	0.1745555
cg12678421	0.8990009	0.4692068	1.722487	0.7482657
cg01839492	2.7938321	1.1482742	6.79759	0.0235292
cg01871631	0.4634956	0.1926938	1.114868	0.0859523
cg12366974	0.2653826	0.0705443	0.99835	0.0497152
cg01277511	1.7650877	0.5425731	5.742147	0.3451355
cg08383063	4.06E-05	5.47E-16	3012768	0.428473
cg00604356	1.5472875	0.6640739	3.605169	0.3118121
cg17387870	0.26903	0.0805431	0.898615	0.032868
cg10043427	4.7453049	1.8663326	12.06533	0.0010736
cg04330371	0.1566978	0.0150237	1.634367	0.1213058
cg01576496	0.2086932	0.0939635	0.463508	0.0001188
cg14305763	1.78E-36	7.23E-62	4.40E-11	0.005792
cg17565490	4.52E-08	1.37E-17	148.6136	0.1303669
cg09931909	0.8049764	0.3725983	1.739103	0.5809598
cg08537737	1.8835955	0.8082758	4.389507	0.1424139
cg07427438	0.3343655	0.125447	0.891215	0.0285085
cg10520405	1.1868092	0.2527528	5.572703	0.8281756
cg12734107	0.3343391	0.1552044	0.720228	0.0051396
cg07465627	1.5096584	0.457703	4.979361	0.4987609
cg12900649	0.0588741	0.0156115	0.222026	2.89E-05
cg05741611	1.47E-06	3.92E-20	55235391	0.3997384
cg27514017	0.3086868	0.1271903	0.749173	0.0093677
cg16703220	0.4946487	0.2219904	1.102198	0.0850824
cg26130669	0.5103129	0.2084442	1.249347	0.1408491
cg19141411	0.3142206	0.1196139	0.825444	0.0188111
cg09529783	4.7833665	1.5999359	14.30094	0.0050941
cg17176005	3.47E-06	6.19E-09	0.001948	9.92E-05
cg15568107	0.7269864	0.328433	1.609185	0.4315773
cg08966293	5.11E-18	1.21E-34	0.215298	0.0414906
cg18414987	2.3159346	0.1805451	29.70754	0.5188688
cg25261361	0.3791588	0.1300322	1.105583	0.0757104
cg19374303	63.102092	0.1915614	20786.41	0.1611341
cg02741158	2.0931345	1.0080699	4.346139	0.0475324
cg10541466	0.2920295	0.0911067	0.936059	0.0383452
cg05005367	1.0376197	0.1613116	6.674378	0.9689814
cg25492213	0.0834562	0.0016657	4.181337	0.2136553
cg00602245	0.2141687	0.0639099	0.717702	0.0125047
cg00468395	0.5146348	0.1874535	1.412878	0.1973279
cg23903588	0.2277441	0.0837072	0.619629	0.0037647
cg19879111	0.7167351	0.1288593	3.98659	0.7036471
cg10145246	2.4061982	1.2146826	4.766504	0.0118158
cg05350315	2.9138967	0.9309123	9.120939	0.0662102
cg02141498	2.0795958	0.8552566	5.056633	0.1062955
cg16956153	0.2259887	0.035102	1.454926	0.1175056
cg06602723	1.8604903	0.8687948	3.984168	0.110053
cg18568843	0.0142218	1.65E-07	1227.967	0.4633257
cg21690050	0.440021	0.1441999	1.342708	0.1492335
cg04337358	0.7710082	0.3536055	1.681121	0.5131973
cg10438391	2.485294	0.8231976	7.503285	0.1063422
cg21646032	0.3882911	0.1619121	0.931184	0.0340294
cg25934863	0.1541307	0.0412481	0.575937	0.0054301
cg18752527	1.0650114	0.4357454	2.603009	0.8901334
cg20023712	0.3905477	0.1733509	0.879877	0.0232822
cg06532379	3.2376321	0.6994896	14.98559	0.1328926
cg19736009	0.0056975	0.0002269	0.143037	0.0016751
cg02735682	2.157766	0.569009	8.182567	0.2581152
cg11700258	2.8527936	1.1196669	7.268619	0.0280323

cg11607648	0.6712252	0.3501905	1.286566	0.2297885
cg11239749	0.5760884	0.256219	1.29529	0.1821782
cg08250135	0.7629137	0.3761167	1.547491	0.4532953
cg07584558	3.034112	0.8816418	10.4417	0.0783752
cg07567256	1.8677383	0.5098173	6.842543	0.3456713
cg16635578	0.5507917	0.2484847	1.220886	0.1419582
cg15262954	2.3801814	1.0099427	5.60949	0.047414
cg02741359	8.27E-07	4.01E-12	0.170673	0.0248877
cg05128201	5.4215584	0.6235791	47.13643	0.1255349
cg27093361	4.2251849	1.1947321	14.94242	0.0253493
cg18475128	7.4777377	1.2365392	45.22021	0.0284374
cg12228123	1.845286	0.8018227	4.246675	0.1496984
cg05211267	1.5002098	0.6783428	3.317835	0.3165407
cg09457752	0.4109525	0.1847839	0.913943	0.0292112
cg12649727	0.3951707	0.0625354	2.497143	0.3236198
cg00095918	0.29707	0.0972391	0.907563	0.0331563
cg14521421	0.2822068	0.0832942	0.956137	0.0421529
cg11661914	0.3888749	0.1976275	0.765196	0.00624
cg16694489	0.1117085	0.0111092	1.123283	0.0627097
cg01098812	0.1889569	0.0584252	0.611118	0.0053978
cg02988727	0.1846605	0.0510469	0.668003	0.0100246
cg13659914	0.1781219	0.0525739	0.603482	0.0055857
cg24154699	0.4118207	0.145196	1.168051	0.095331
cg04400047	0.011326	0.0007281	0.176184	0.0013747
cg19052355	0.0143593	0.0004144	0.497569	0.0189838
cg14055970	1.1275094	0.057196	22.22667	0.9371137
cg21918755	2.6178437	1.0289621	6.660212	0.0433949
cg06799321	0.2743264	0.0542562	1.387029	0.1177495
cg26439759	19208.04	1.310003	2.82E+08	0.0438906
cg00934735	1.9173056	0.7715594	4.764456	0.1610487
cg07757611	0.3159893	0.1087849	0.917859	0.034217
cg01333884	2.9981818	0.9906368	9.074056	0.0519782
cg07125635	1.1394649	0.3117007	4.165472	0.8435105
cg20707409	0.5267141	0.2002581	1.385351	0.1938272
cg06952534	0.8613697	0.3464679	2.14149	0.7480915
cg25388528	1.0695188	0.5044709	2.267466	0.860847
cg01445827	0.6182842	0.2992314	1.277524	0.1941143
cg18741439	1.0974216	0.4104315	2.934312	0.8530251
cg10094489	4.4076982	1.4375647	13.51439	0.0094622
cg16786712	3.26E-14	1.84E-31	5761.324	0.125365
cg02190383	14.217297	0.1247241	1620.629	0.2719837
cg25119155	0.4278666	0.1821324	1.005147	0.0513931
cg10613224	0.3021929	0.163423	0.558799	0.0001359
cg16276063	1.9080992	0.5997224	6.07088	0.2738957
cg15453836	0.1028997	0.0014582	7.261461	0.2950651
cg01894064	0.5739797	0.3406651	0.967087	0.0370061
cg22904255	154842.6	17.511575	1.37E+09	0.0099538
cg20291049	0.1933861	0.028229	1.324816	0.0942322
cg07047570	1.3690833	0.5518145	3.396774	0.4980386
cg03382549	0.4968277	0.1743057	1.41612	0.1905573
cg01040654	1.2340111	0.4610147	3.303113	0.6755308
cg09336228	5.693E+14	68063242	4.76E+21	2.94E-05
cg14746387	1.3274411	0.4945965	3.562702	0.5738936
cg11795628	3.730656	1.2620799	11.02766	0.0172711
cg08765317	1.5879767	0.7784895	3.239183	0.2035487
cg08306955	6.475934	1.6711416	25.09525	0.0068725
cg19017185	0.2784711	0.0822327	0.943009	0.0399513
cg05505961	0.7605016	0.356303	1.623232	0.4791173

cg03788239	2.4687447	0.7420657	8.213154	0.1406047
cg08526705	0.6319838	0.3271347	1.220914	0.1719813
cg05335277	1.623994	0.5461653	4.828861	0.3831451
cg26455386	0.3057138	0.1304096	0.716672	0.0064039
cg09207762	2.9826769	0.9056646	9.82302	0.0723313
cg27583671	12.442575	0.541443	285.9353	0.1149437
cg09465703	0.0018989	1.23E-05	0.292932	0.0147865
cg20930114	0.0010917	1.31E-05	0.090931	0.0025062
cg02219026	0.6899514	0.3207491	1.484129	0.3422811
cg11096000	6.5583423	0.8885319	48.40778	0.0651711
cg11549417	2.5702824	0.9409331	7.021064	0.0655898
cg12729518	1.4068948	0.4094521	4.834151	0.5877624
cg14463412	1.7242114	0.8138406	3.652933	0.1549692
cg14083015	2.7219997	0.8186709	9.050379	0.102348
cg06904403	2.4115615	0.9919025	5.863105	0.0521338
cg12929364	0.5726181	0.1629869	2.011766	0.3844947
cg11799650	0.2574594	0.0959706	0.690684	0.0070392
cg11887996	1.4414663	0.3880859	5.354033	0.5849472
cg23062357	0.39355	0.1628195	0.951247	0.0383625
cg01269798	3.5053991	0.4050195	30.33884	0.2546476
cg21550141	0.6883886	0.2999736	1.579735	0.3782895
cg24088496	1.6067353	0.7233579	3.568908	0.2441769
cg01295600	7.5990715	8.57E-06	6736562	0.771632
cg10502206	0.4166533	0.1791865	0.968823	0.0419987
cg01159576	1.8004838	0.7753543	4.180981	0.1712971
cg16716975	0.0011863	4.67E-06	0.301677	0.0171232
cg08727202	0.3448866	0.107658	1.104857	0.0731175
cg06270993	0.5891278	0.2924886	1.186616	0.1385994
cg15390560	419.00174	2.3055819	76146.7	0.0229263
cg11119419	1.3936859	0.7190658	2.701227	0.3255262
cg16419526	0.4637685	0.1465159	1.467972	0.1912173
cg07725970	3.57E-13	7.54E-30	16895.29	0.143456
cg02874908	2.4995014	0.5110445	12.22498	0.2580106
cg06441398	1.5322313	0.6537465	3.591197	0.3261365
cg02491557	3.6742168	1.8006212	7.49734	0.0003486
cg02333588	5.2342904	1.3441352	20.38321	0.0170166
cg07270872	0.4331351	0.1601759	1.17125	0.0992454
cg08345082	0.0811873	0.0099543	0.662167	0.0190301
cg25558099	2.4006656	0.7839891	7.351117	0.1250908
cg24536529	0.3130462	0.118619	0.826157	0.0189932
cg07251194	4.82E+23	0.0169782	1.37E+49	0.068203
cg18760587	4.07E-05	7.19E-17	23064280	0.4641014
cg07824663	0.3823188	0.1330558	1.098544	0.0741901
cg04337007	1.5150548	0.6818308	3.366511	0.3078019
cg06328674	3.7272366	1.4189153	9.790783	0.0075841
cg04917258	0.8241249	0.0639372	10.62264	0.8821045
cg09579623	0.4254181	0.1772472	1.021063	0.0557095
cg00654888	4.4918921	1.5647466	12.8948	0.0052368
cg12832856	30.383406	1.0823962	852.8775	0.0448029
cg11127874	0.2670962	0.1202484	0.593275	0.0011861
cg05224015	1.6962612	0.8174763	3.519738	0.1559454
cg08382220	0.2940493	0.0743765	1.162532	0.0809443
cg10330187	0.0071383	0.0002104	0.242152	0.0059831
cg01514538	0.7678689	0.3129748	1.88393	0.5640584
cg13660126	0.4103585	0.1867614	0.901654	0.026574
cg24166966	0.1098881	0.0080446	1.501058	0.0978284
cg00320597	0.6131122	0.2825396	1.330456	0.215853
cg05327596	0.1485131	0.0478928	0.460532	0.0009572

cg02579047	7.7650332	1.53074	39.38993	0.0133673
cg16886259	9.3762466	0.3183947	276.1164	0.1946858
cg21618730	1.4957402	0.736153	3.039095	0.2656633
cg17065262	0.4268643	0.2066655	0.881681	0.0214359
cg02164225	2.7727305	1.1176974	6.878458	0.027807
cg26221941	0.9986874	0.5252435	1.898884	0.9968034
cg23375431	4.3923534	7.03E-26	2.75E+26	0.9610533
cg09839592	8.070503	1.169226	55.7061	0.034126
cg25558761	7.2161665	0.3052606	170.5856	0.2206997
cg20024259	0.0813819	0.0018041	3.671178	0.1967762
cg17472736	0.3174075	0.1514842	0.66507	0.0023606
cg12555233	0.0001224	7.66E-08	0.195634	0.0166901
cg23259607	0.4462033	0.221424	0.899168	0.0239917
cg21910709	0.1405498	0.0179534	1.100309	0.0616343
cg15650694	1.15E-10	5.14E-23	258.1805	0.1147495
cg12708802	0.2998477	0.0624793	1.439015	0.1322852
cg05840833	0.0028812	1.90E-06	4.37848	0.1176045
cg03110993	0.0193659	0.0001445	2.595866	0.114506
cg12393651	0.1242281	0.0357523	0.431653	0.0010306
cg12845215	2.4909549	0.8993913	6.898951	0.0790968
cg21608600	1.3762663	0.6365699	2.975492	0.4168799
cg16199239	1411.8248	0.1699137	11730953	0.1152469
cg19519964	2.3236248	0.9204802	5.865669	0.0743292
cg18397073	0.6854162	0.1660185	2.829776	0.6015831
cg17109042	1.0060252	0.4401209	2.299566	0.9886372
cg12947103	489.17202	0.6394443	374214.4	0.0675537
cg00234370	0.4907558	0.2242769	1.073857	0.0748123
cg00552087	0.4597445	0.1698555	1.244381	0.1261157
cg22507069	0.383126	0.120959	1.213515	0.1028954
cg02026204	2.575092	0.9201004	7.20693	0.0716435
cg19110902	1.2781766	0.4450234	3.671123	0.6484349
cg07745344	4.5281335	1.1307594	18.13294	0.0328783
cg16076328	1.2517355	0.4388307	3.570492	0.6745968
cg10215032	0.1247009	0.0148116	1.049876	0.0554685
cg00504902	2.4259804	0.9408937	6.255096	0.0666701
cg10534923	0.150417	0.031603	0.715922	0.0173227
cg03872623	3.4472832	0.9720555	12.22539	0.0553545
cg04038680	0.1721797	0.0250578	1.183096	0.0736174
cg06508867	3.3890341	0.8871348	12.94679	0.0742876
cg24919344	0.8756647	0.4490286	1.707661	0.6968147
cg11606463	325.62724	0.0188112	5636691	0.2452418
cg02625804	3.2905348	1.1596301	9.337132	0.0252026
cg13435392	0.890989	0.3516178	2.25774	0.8077663
cg11180921	0.3802144	0.1414047	1.022335	0.0553405
cg01630690	1.7414243	0.9379235	3.233269	0.0789222
cg20830867	0.7137053	0.3061742	1.663678	0.434737
cg13763516	6.57E-13	3.30E-22	0.001306	0.0102327
cg27634050	0.1417722	0.0120217	1.671923	0.1207318
cg03809403	19.498849	0.2387661	1592.375	0.1860534
cg07044016	4.5592103	0.1688639	123.0956	0.3669383
cg20558230	3.6296057	1.0766006	12.2367	0.0376177
cg11261261	3.7485643	0.5241515	26.80853	0.1880352
cg15162330	2.1850014	1.0908611	4.376571	0.02743
cg13861407	0.2105728	0.0152774	2.902382	0.2444592
cg21798544	0.3392809	0.1222512	0.941598	0.0379393
cg26014401	2.6915614	1.2317455	5.881493	0.0130436
cg03519180	0.3670699	0.1206162	1.1171	0.0775721
cg02696253	1.5505859	0.6710984	3.582659	0.3046339

cg01080927	0.7978823	0.2894418	2.199462	0.662521
cg13303654	1.1471416	0.5319586	2.473752	0.7262522
cg25134859	0.8725438	0.3294842	2.31068	0.7837832
cg21078322	0.4316134	0.1973672	0.943876	0.035322
cg00197322	0.365911	0.1387125	0.96524	0.0422093
cg06942701	3.5911619	0.8500733	15.17098	0.0820315
cg21787280	0.0884925	0.0047952	1.633072	0.103054
cg19169932	0.4720057	0.2422961	0.919492	0.0273376
cg01814364	6.4472763	0.2169342	191.6128	0.281518
cg18308535	0.1981381	0.0485748	0.808211	0.0240192
cg00787856	0.134566	0.0346459	0.52266	0.0037655
cg02678305	4.5358594	0.8926755	23.04759	0.068292
cg10380019	0.5085415	0.2500528	1.03424	0.0619
cg23671279	0.3211327	0.1441558	0.71538	0.0054431
cg22823081	1.8902883	0.6930194	5.155974	0.2136082
cg26985149	2.1304118	0.6464331	7.021074	0.213884
cg05969445	8.672462	1.572474	47.83011	0.0131552
cg00969288	0.8172191	0.4089681	1.633005	0.567678
cg20079766	2.1998421	0.9304627	5.200967	0.0725272
cg19015264	0.0273669	0.0034419	0.217597	0.0006697
cg01779806	0.6112624	0.317389	1.177236	0.141019
cg11007962	1.9954778	0.469481	8.48156	0.3493783
cg10522535	0.6288313	0.3252893	1.215622	0.1677792
cg13266327	0.4264432	0.1143236	1.590694	0.204479
cg10768734	0.4185219	0.200072	0.875488	0.0207178
cg23541923	0.6591749	0.3343672	1.299504	0.2287984
cg23270617	1.03E-06	2.58E-20	40868433	0.3881034
cg18422058	0.4038594	0.096254	1.694501	0.2152795
cg10004897	0.2747845	0.0827662	0.912287	0.0348664
cg10426951	1.0918882	0.4506563	2.645519	0.8456314
cg18064663	0.2094033	0.0721565	0.607703	0.0040248
cg18993949	0.2412807	0.0395184	1.473146	0.1234923
cg01179256	0.3884325	0.1535098	0.982868	0.0458853
cg15493607	0.4353154	0.1978543	0.957773	0.0387152
cg22931002	0.3458487	0.1167429	1.024571	0.0553452
cg21774377	2.435E+10	434.80882	1.36E+18	0.0086055
cg02260386	4943762.3	5.31E-29	4.61E+41	0.7075199
cg02283238	2.3956706	0.6202551	9.253028	0.2050847
cg23708211	0.2466669	0.08994	0.676502	0.0065439
cg23616126	3.3090132	0.0885567	123.6447	0.5171392
cg11752894	0.4172938	0.1712346	1.016933	0.0544779
cg16320910	5.0639574	0.1112324	230.5413	0.4050333
cg02666042	2.5722792	0.4114003	16.08317	0.3123781
cg12390011	2.2189685	0.7146116	6.890206	0.16798
cg10820736	0.509187	0.2814468	0.921209	0.0256632
cg06739462	5.4499058	0.2834865	104.7721	0.2609333
cg09731306	0.5708586	0.1962536	1.660502	0.3034424
cg09962974	0.4661168	0.1098126	1.978505	0.3007273
cg08343671	0.516848	0.2434717	1.097178	0.0857075
cg04371818	6.2667186	0.0153459	2559.103	0.5496448
cg00927256	0.5506216	0.1784175	1.699296	0.2993608
cg18483404	0.5662341	0.2609825	1.228516	0.1500988
cg16748816	0.5555216	0.2764057	1.11649	0.0988264
cg20504791	2.1070582	1.0485277	4.234217	0.0363453
cg02222844	0.8908957	0.2695837	2.944151	0.8497586
cg22796051	0.3490878	0.1389856	0.876798	0.0251055
cg08456334	0.3061027	0.1230815	0.761275	0.0108733
cg02061173	2.6341979	0.892529	7.774536	0.0794196

cg02646779	0.2450591	0.0716782	0.837827	0.0249565
cg13877916	4.6084336	1.0398923	20.42294	0.0442767
cg24114371	0.721818	0.2541157	2.050331	0.5405405
cg23500204	0.0910114	5.10E-05	162.4589	0.5303868
cg24256211	0.1242963	0.0187865	0.822375	0.0305556
cg26901352	6.6798281	1.4860756	30.02546	0.0132656
cg15259060	0.8785305	0.2706737	2.851463	0.8293057
cg23929194	3.1746668	0.8006888	12.5873	0.1002408
cg24601480	0.5203357	0.2611795	1.036641	0.0632204
cg26392521	3.3262979	0.9964499	11.10368	0.0506798
cg22501604	0.0138482	0.0001874	1.023415	0.0512452
cg01228685	1.7819468	0.3393404	9.357373	0.4947756
cg08720365	2.8042031	0.4378216	17.96064	0.2764828
cg09360458	79.35789	6.7536075	932.4905	0.0005026
cg06345736	36.562418	0.252356	5297.32	0.1563038
cg21560142	0.0017058	2.74E-07	10.62506	0.152768
cg23350385	1.580852	0.7332784	3.408109	0.2426269
cg01545109	2.1986011	0.8004924	6.038592	0.126442
cg26412358	4.9141409	0.4456431	54.18862	0.1935964
cg07529210	0.2912996	0.0747372	1.135384	0.0755632
cg15856454	0.0133403	0.000243	0.732303	0.0346504
cg13879523	2.3645007	0.6456317	8.659525	0.193823
cg03990732	2.407846	0.8331756	6.958584	0.1046124
cg02243630	0.2532162	0.1035176	0.619396	0.0026165
cg09380135	0.3557555	0.1365087	0.927135	0.0344494
cg25141417	0.9040923	0.2327548	3.511776	0.884214
cg15459774	0.6607803	0.3314485	1.317341	0.23919
cg07255664	1.9399683	0.6769493	5.559467	0.2173376
cg17535702	1.671269	0.4614296	6.053231	0.4341399
cg12009856	0.3979168	0.196461	0.80595	0.0104955
cg17530411	0.3589069	0.0631273	2.040546	0.2478374
cg22135566	1.6357246	0.4971521	5.381843	0.4180342
cg07628580	2.9683039	0.6833524	12.89354	0.1465359
cg21645554	0.4186188	0.1929536	0.908207	0.0275513
cg16565002	0.2724459	0.1278917	0.580388	0.0007517
cg19748684	1.3450132	0.640973	2.822366	0.4331493
cg15788231	2.779824	1.0268289	7.525521	0.0442128
cg21888214	0.867574	0.3793456	1.984166	0.7364475
cg01660999	0.9151868	0.4989132	1.678783	0.7746371
cg19413392	0.1438711	0.0201847	1.025473	0.0530073
cg04735908	0.0722315	0.0039972	1.305249	0.0751471
cg01699430	3.548882	0.9491436	13.2694	0.0597825
cg04469219	1.4692216	0.652555	3.307939	0.3528299
cg17066470	1.9217439	0.9133894	4.043291	0.0852048
cg04860563	0.5912232	0.2978414	1.173594	0.1329984
cg19418318	0.316991	0.1323153	0.759423	0.009957
cg18570800	0.1496564	0.0369966	0.605381	0.0077251
cg00076774	1.9626198	0.6452088	5.96997	0.2348474
cg10389771	4.0989806	1.209792	13.88804	0.0234604
cg05031202	19282.641	0.0076496	4.86E+10	0.189522
cg21530308	1.5931107	0.8310923	3.053814	0.1607101
cg06546806	2.1211713	0.9671553	4.652167	0.0605702
cg06768213	0.0042639	3.12E-05	0.583643	0.0296669
cg00474328	1.9730447	0.9368563	4.155285	0.073724
cg02551743	3.0268714	0.9351342	9.797471	0.0645941
cg26036626	0.6797848	0.3111354	1.485229	0.3330659
cg02571055	0.3943413	0.1572468	0.988924	0.0472885
cg20952167	1.7706187	0.5806078	5.39967	0.3152422

cg10959672	2.5967739	0.7064297	9.545514	0.1507953
cg13712023	0.2975012	0.0895702	0.988129	0.047764
cg07779313	0.2857105	0.1086832	0.751087	0.0110728
cg22056218	1.0460596	0.305932	3.576744	0.9427706
cg15578140	0.1871267	0.0304305	1.150699	0.0705296
cg23745444	1.9323806	0.8952968	4.17079	0.0933071
cg01305981	2.51867	1.218139	5.207697	0.0126891
cg25822709	0.1258197	0.0063329	2.499754	0.1740792
cg12839363	56145.25	6.78E-05	4.65E+13	0.2965863
cg07020001	0.5563637	0.1203859	2.571236	0.4528016
cg24994593	0.5221872	0.19396	1.405854	0.198505
cg03860466	1.8830022	0.4582881	7.73683	0.3800689
cg05161019	0.9581668	0.3901223	2.353323	0.9257358
cg26461267	3.069339	1.2479789	7.548879	0.0145891
cg01263877	0.0824229	0.0188505	0.36039	0.0009139
cg10361005	4.2608183	0.4749814	38.22165	0.1953614
cg06796682	0.7159065	0.3532549	1.450856	0.3537532
cg00162969	0.0388185	0.0032121	0.469131	0.0106112
cg01616225	0.7173116	0.0905678	5.681225	0.7530099
cg15322932	0.2132302	0.0150433	3.022413	0.2533048
cg15232971	1.5457113	0.6288487	3.799362	0.3425916
cg23892924	0.4101029	0.1447411	1.161967	0.0934529
cg11282657	3.7338219	0.6365593	21.90122	0.1444114
cg11890641	0.0019462	9.74E-06	0.38908	0.0209332
cg09234872	5.86E-14	3.05E-25	0.011246	0.0215321
cg16704958	3.071968	1.0214253	9.239038	0.0457494
cg01167274	1.8285098	0.7592456	4.403645	0.1783762
cg15551687	1.8262524	0.6094807	5.472196	0.2820894
cg03344526	1.4688901	0.2908679	7.41793	0.6416651
cg19922825	2.1759683	1.0734529	4.410848	0.0310389
cg00840694	1.1737793	0.4834911	2.849603	0.7232866
cg02671333	6.3435605	0.5010663	80.31025	0.153746
cg26181818	3.1546733	0.9759526	10.19718	0.0549464
cg00640479	1.8519044	0.6658428	5.15069	0.2377215
cg10644890	21619251	1.6434073	2.84E+14	0.0434497
cg15801019	2.0018562	0.9055109	4.425599	0.0863916
cg09391093	2.288E+10	0.0051161	1.02E+23	0.1084924
cg08690459	0.2784773	0.0573034	1.353315	0.112994
cg13390867	1.4224659	0.7986555	2.533519	0.2314781
cg21778268	2.8210636	0.0021273	3741.122	0.7773977
cg00902815	2.0755556	0.7178322	6.001307	0.1776628
cg26219070	0.361038	0.1862552	0.699838	0.0025541
cg05903289	1.2150327	0.5797738	2.546345	0.6058905
cg27560781	0.7630329	0.3649904	1.595163	0.472251
cg26992415	8.5792846	0.2065927	356.2765	0.2582652
cg05138082	1.5459752	0.587996	4.06472	0.3770794
cg02039370	11.971899	1.6599243	86.34512	0.0137904
cg19328475	1.9214149	0.9675052	3.81583	0.0620981
cg02507889	1.4448785	0.5915523	3.529145	0.4192533
cg22944823	1735.4537	5.63E-05	5.35E+10	0.3965419
cg05309399	0.4308991	0.1844236	1.00678	0.0518524
cg23927124	2.4580437	0.314783	19.19411	0.3910723
cg07280097	2.0826627	0.5091865	8.518458	0.3073373
cg26082001	1.9315323	0.577443	6.460927	0.2852574
cg15723874	1.9865696	0.3612478	10.92452	0.4299722
cg12800105	3.7596653	0.7492564	18.86548	0.1075736
cg14574047	0.2595093	0.0987942	0.68167	0.0061875
cg06513814	1.3411322	0.6355857	2.829887	0.4410609

cg00908766	0.2261699	0.0514611	0.994009	0.0490773
cg01135315	2.1866598	0.3409041	14.02588	0.4093287
cg20244273	0.2147733	0.0465024	0.991941	0.0488006
cg21535655	0.8991691	0.4341642	1.86221	0.774782
cg22029856	6.6505247	0.7914168	55.88646	0.0810599
cg07755653	0.2546607	0.0538087	1.205233	0.0845996
cg15148064	2.1185534	0.1710492	26.23964	0.5587519
cg18444589	0.3128271	0.1123892	0.870732	0.026082
cg00338801	1.3317886	0.720346	2.462235	0.3608204
cg24003749	1.7345574	0.6735843	4.466686	0.2537867
cg15499368	2.234728	0.9079334	5.500414	0.080154
cg16899892	11.378539	1.6275805	79.54823	0.01425
cg03774463	4.1242796	1.2796804	13.29213	0.0176451
cg21202178	0.2105326	0.0724723	0.611598	0.0041885
cg20114528	0.920859	0.2093857	4.049854	0.9131208
cg13023619	0.3022258	0.124244	0.73517	0.0083323
cg15150034	1.3363761	0.3483017	5.127455	0.6725513
cg05835346	7.9314708	1.7349046	36.26034	0.0075751
cg09262446	0.8833356	0.4390192	1.777329	0.7280278
cg22417566	1.9039733	0.8886741	4.079239	0.0976457
cg03707634	2.1456685	0.9124486	5.045646	0.0801272
cg05940231	2.5807691	1.195914	5.569271	0.0156984
cg08755703	1.7070366	0.69626	4.185181	0.2425117
cg16305333	1.9135917	0.3973094	9.216577	0.4184363
cg09112760	1.142308	0.5136279	2.540492	0.7442344
cg06574296	0.7981608	0.4115845	1.547825	0.5046629
cg20219379	5.746715	0.9055063	36.47102	0.0636415
cg00952054	0.3402169	0.1801652	0.642452	0.000887
cg22956483	0.0496979	0.0064718	0.381635	0.0039
cg25960090	0.2377805	0.0860362	0.65716	0.0056161
cg21588305	8.4743652	0.0216372	3319.044	0.482959
cg24284497	1.2970312	0.5085175	3.308224	0.5861637
cg16817237	1.7028673	0.5327207	5.443296	0.3692887
cg04268950	1.0793924	0.5210484	2.236046	0.8371063
cg04789392	0.806616	0.4245928	1.532361	0.5115788
cg19860752	0.393344	0.1804378	0.857467	0.0189401
cg06802488	0.1103122	0.0312745	0.389096	0.0006088
cg02069772	0.5527505	0.2153785	1.418587	0.2176362
cg08260286	9.02E-21	2.04E-36	3.98E-05	0.0120328
cg03420389	2.3734838	0.7031564	8.011625	0.1637493
cg17377658	0.3407778	0.1398177	0.830578	0.0178673
cg12813482	95.147142	0.7463933	12128.97	0.0655178
cg03462348	0.3836646	0.141693	1.038855	0.0594354
cg15227982	0.2739938	0.1138345	0.659489	0.0038663
cg05449607	0.2127701	0.0003895	116.2366	0.6303695
cg01067603	0.8883864	0.45722	1.72615	0.7269329
cg07963563	0.6099515	0.2939021	1.265866	0.1844757
cg20930514	2.631E+17	16670.908	4.15E+30	0.0096834
cg16082058	3.4927212	1.0725171	11.37427	0.0378773
cg11987802	0.9564124	0.1555908	5.879043	0.9616365
cg01808968	3.9451891	1.5670119	9.932609	0.0035748
cg12160741	1.5707007	0.379738	6.496849	0.5330837
cg04650789	1.6816708	0.8388161	3.371438	0.1430063
cg25158622	0.0004555	9.23E-10	224.6742	0.2499812
cg14202850	2.5614406	1.0622647	6.176406	0.0362181
cg01601628	1.8180099	0.9395079	3.517969	0.0759473
cg11065575	0.5722524	0.2781787	1.177203	0.1293485
cg11414540	1100.9535	20.51883	59072.5	0.0005671

cg03534847	0.3882973	0.1871402	0.805678	0.0110802
cg21658616	64.463068	0.4640393	8955.033	0.0979318
cg01286665	1.4067346	0.4194754	4.717564	0.5804121
cg07751641	1.8707324	0.7248181	4.828301	0.1954253
cg23440636	2.249787	1.0773734	4.698039	0.0309012
cg21598067	0.314145	0.1100512	0.896737	0.0304932
cg03630413	3.0367066	1.0425664	8.845083	0.0417116
cg18177275	1.2083445	0.6169539	2.366621	0.5810865
cg00763594	0.3928903	0.1907719	0.809148	0.0112611
cg12323063	0.4342556	0.2177208	0.866146	0.0178891
cg09974063	0.0643131	0.0028507	1.450913	0.0843711
cg03537872	0.0837955	0.0129163	0.543628	0.0093547
cg25859099	1.8810632	0.7790929	4.54169	0.160047
cg15018704	0.2418282	0.0940706	0.62167	0.0032118
cg00513564	0.120357	0.0397915	0.364043	0.0001773
cg11742103	0.1220199	0.0402941	0.369505	0.0001983
cg03441171	1.8981906	0.9800285	3.676554	0.0574131
cg12929160	1.7494045	0.6486614	4.718049	0.2692175
cg04458368	1.3759573	0.6907096	2.741034	0.3640764
cg03233332	0.6157204	0.2748814	1.379182	0.2385461
cg14539990	3.57E-09	1.05E-22	121601.9	0.2211422
cg24391931	8277.9036	6.52E-18	1.05E+25	0.7159575
cg21514857	0.2134928	0.0220715	2.065071	0.1823174
cg10579705	0.0011747	1.38E-06	1.000884	0.05003
cg22958090	2.7272433	0.8307255	8.953446	0.0980887
cg21721867	0.6150385	0.3071884	1.231402	0.1699711
cg05401670	2.5240072	1.0378001	6.138573	0.0411725
cg16014906	1.4582645	0.4674254	4.549465	0.5157803
cg18898693	106.70529	0.0246606	461709.5	0.2742944
cg22764193	0.9856576	0.123805	7.847186	0.9891109
cg20663403	2.42E-06	8.12E-19	7213375	0.3775522
cg03035167	1.4114269	0.438889	4.53902	0.5631263
cg16214492	2.0123763	0.6928126	5.845243	0.1986534
cg16238618	2.85E-13	9.08E-29	896.9097	0.1126198
cg01111842	3.37E-07	3.33E-13	0.342152	0.0346897
cg09736083	0.4013915	0.1377304	1.169786	0.0944037
cg10008953	0.5548105	0.1730075	1.779198	0.3217413
cg14445366	3.003276	0.6844443	13.17809	0.1449871
cg03512414	0.3732321	0.1791866	0.777414	0.0084761
cg19776877	6.4665594	1.932081	21.64319	0.0024578
cg20853370	0.5420362	0.2506999	1.171932	0.1195444
cg24298423	0.4915919	0.2544216	0.949851	0.0345952
cg24717029	0.0220382	4.38E-14	1.11E+10	0.7813932
cg04108298	1.0616865	0.3294481	3.421413	0.9201399
cg12498395	0.6734425	0.2949152	1.537814	0.3480226
cg21577626	0.7944653	0.2704978	2.333384	0.6755368
cg20219074	1.926E+13	159.43675	2.33E+24	0.0187982
cg02393727	0.1185308	0.0230617	0.609217	0.0106702
cg21853021	12.10108	0.3631161	403.2764	0.1634083
cg24861272	1.9580344	0.9263982	4.1385	0.0784509
cg23922433	4.1672209	0.4905879	35.39779	0.1910284
cg13468041	1.5398111	0.5011356	4.731291	0.4510398
cg08373003	2.766477	0.9568834	7.998252	0.0602995
cg09933726	3.69E+21	49586980	2.75E+35	0.0023095
cg10362335	2.4383569	1.1950506	4.975173	0.0142978
cg14242936	0.2070378	0.0575295	0.74509	0.0159388
cg15472658	3.4979209	0.8163363	14.98825	0.0916753
cg17940113	1.7426449	0.8643549	3.513385	0.1205439

cg04184297	1.7355161	0.6320279	4.765638	0.284752
cg14020320	1.7570047	0.8656686	3.566106	0.1186301
cg00816620	0.345913	0.1333892	0.897043	0.0290035
cg20464175	0.8456332	0.3530866	2.025269	0.706714
cg27169559	0.4148287	0.1838	0.936251	0.034127
cg23295886	2.5593525	0.9123139	7.179859	0.0741652
cg06501988	0.514785	0.2453602	1.08006	0.079044
cg07927098	0.3054652	0.0778314	1.19886	0.0891356
cg05649724	0.117781	0.00622	2.230298	0.1540386
cg12580156	1.3919116	0.3427303	5.652893	0.6437595
cg18039789	0.3560132	0.0886312	1.43003	0.1454557
cg11920943	4.1336305	0.7702366	22.18396	0.097835
cg05384135	5.51E-38	3.85E-63	7.89E-13	0.0036964
cg08463848	0.1731307	0.0537424	0.557739	0.0033015
cg17940464	2.1408933	0.4283347	10.70057	0.3538107
cg00349948	2.2454463	0.8468845	5.95362	0.1039674
cg15552853	1.2905581	0.4988314	3.338884	0.5989308
cg10801328	0.2982313	0.0583903	1.523231	0.1459011
cg24889744	8.00E-05	1.35E-07	0.047327	0.0037698
cg18334681	1.5259557	0.6365459	3.658088	0.3434401
cg21902966	0.2584601	0.1283084	0.520633	0.0001526
cg16444607	1.106995	0.5567832	2.200925	0.7718915
cg22519545	2.0586137	0.904158	4.687113	0.0854391
cg18660409	2.5716969	0.7354227	8.992957	0.1391849
cg06914048	0.0269205	0.0008609	0.841788	0.0395886
cg15310583	9.6764967	1.7551243	53.34926	0.0091658
cg12858693	1.0042722	0.5283109	1.909032	0.9896214
cg02771886	4.8731412	0.2771374	85.68855	0.278943
cg00850193	5.7488627	0.9614023	34.37627	0.0552602
cg24751773	0.3481968	0.1072764	1.130174	0.0790457
cg16347256	1.0795094	0.4774808	2.440602	0.8541533
cg10220544	2.1954892	0.8354204	5.769757	0.1106668
cg17873998	5.678197	0.4735202	68.08986	0.1706375
cg24700485	1.1726205	0.5424271	2.534974	0.6855968
cg01919941	0.4235092	0.1953583	0.918108	0.0295259
cg19363889	0.7397172	0.3101503	1.764246	0.4966204
cg18459489	2.758149	1.3258798	5.737614	0.0066328
cg23631759	0.365098	0.1503483	0.886585	0.0260212
cg25322008	0.7916964	0.3677505	1.704371	0.550473
cg10018632	2.272879	0.87254	5.920621	0.0927946
cg05271255	0.1583759	0.0235088	1.066957	0.0583082
cg05628771	1.6154728	0.7464747	3.496103	0.2233564
cg14202757	1.8108031	0.6966285	4.706968	0.2231263
cg22115305	2.1572274	0.6208473	7.495612	0.2263346
cg21567371	4.5505051	0.581538	35.60747	0.1488694
cg07962128	1.1511667	0.5564697	2.381414	0.7042653
cg22519102	0.5079094	0.2021773	1.275969	0.1494641
cg09307412	3.6671854	0.4512215	29.8041	0.2241603
cg11862491	6.068E+12	5.31E-07	6.94E+31	0.1886407
cg20331595	2.94E-18	1.40E-30	6.17E-06	0.0052933
cg22185879	0.3315582	0.1500749	0.732507	0.0063402
cg14492872	9.765798	1.0964199	86.98383	0.0411055
cg26814635	10.895023	2.21E-05	5366859	0.7209984
cg26778754	1.30E-08	4.34E-18	38.7393	0.102795
cg02620054	1.9288842	0.8416854	4.42041	0.1205114
cg02863073	2.3816939	1.0757309	5.273128	0.0323565
cg11537406	1.1341022	0.5329678	2.413256	0.7439532
cg03472888	0.488833	0.2047367	1.167146	0.1069879

cg14590902	1.2681451	0.6386584	2.518079	0.4972801
cg23143104	2.4281754	0.9946746	5.927603	0.0513865
cg17009069	2.5872333	0.4535708	14.75795	0.284608
cg25223634	0.258294	0.0855643	0.779716	0.0163334
cg06758644	1.6976776	0.5595379	5.150874	0.3499873
cg13796319	0.0014698	7.23E-17	2.99E+10	0.6765393
cg15095917	0.3593118	0.057039	2.263449	0.2757001
cg04713531	0.2278113	0.0579139	0.896124	0.0342666
cg20821187	1.44588	0.6080437	3.438189	0.4041241
cg04625871	1.7482707	0.5910487	5.171233	0.3126896
cg23983671	2.1808548	0.9486751	5.013442	0.0663719
cg14899065	0.5749741	0.1648746	2.005131	0.385197
cg08985885	0.0221058	0.0018231	0.268045	0.0027526
cg23503176	1.9707606	0.981358	3.957676	0.056512
cg12423278	0.3725016	0.1676831	0.827498	0.0153115
cg03213216	1.7445164	0.7367621	4.130692	0.2057522
cg26659853	0.0366558	0.0033338	0.403036	0.0068744
cg02156071	1612733.4	0.0033506	7.76E+14	0.1611279
cg26487259	0.1792323	0.0681456	0.471406	0.0004937
cg14339466	1.4814297	0.6672408	3.289118	0.3341774
cg04447981	2.09E-05	1.31E-08	0.033331	0.0041814
cg01078772	2.1159201	0.7805164	5.736097	0.1407603
cg27400746	0.0541644	0.0082702	0.35474	0.0023597
cg09731087	4.3631838	1.2355499	15.40802	0.0221059
cg23871640	1.8158113	0.6787496	4.857713	0.2347742
cg07341624	2.4934127	0.9917019	6.269129	0.0521068
cg04429285	4.41E-16	1.75E-41	1.11E+10	0.2360882
cg00000714	0.0183738	0.0002618	1.289447	0.0653644
cg00399510	2.57E-12	2.35E-38	2.82E+14	0.3830439
cg01532168	0.0910263	0.0116742	0.709751	0.0221873
cg19611002	0.3824979	0.1435561	1.019146	0.0546015
cg09802066	2.1358866	1.0924901	4.175792	0.0265158
cg27419119	0.0269113	0.0005342	1.355639	0.0706361
cg08229468	0.4182177	0.1681895	1.039935	0.0606951
cg25467652	0.1530683	0.026257	0.892329	0.0369229
cg01228193	0.8482798	0.0028377	253.5755	0.954882
cg14202477	2.1147469	0.8623507	5.186004	0.1017585
cg27007060	0.4457032	0.162044	1.22591	0.1174887
cg17206968	2.2066263	0.6174488	7.885997	0.2232333
cg04675542	0.3333341	0.1134723	0.979196	0.0456948
cg15531249	3.1847367	0.7322186	13.8518	0.1224875
cg16779839	0.3014509	0.0603059	1.506861	0.1441383
cg05293365	3.2594885	1.1676682	9.098702	0.024076
cg11214047	0.5750179	0.2994642	1.104124	0.0964344
cg02790458	0.0966006	0.027554	0.338669	0.0002606
cg03578041	1.4031977	0.5638844	3.491787	0.4664412
cg11272244	2.0404328	0.5629979	7.394994	0.2776875
cg06639320	0.1557992	0.0425224	0.570838	0.0050132
cg18043267	0.0278683	0.0027176	0.285783	0.0025733
cg12932675	0.006646	6.43E-09	6865.673	0.4779437
cg02930033	1.3558585	0.5815255	3.161258	0.4809029
cg15366684	1.2994811	0.6436709	2.62347	0.4648743
cg03726357	1.7442751	0.5993136	5.076634	0.3074049
cg20980960	0.3285147	0.1259869	0.856612	0.0228177
cg26820037	2.3787287	0.9801191	5.773125	0.0554194
cg23663774	1.7554152	1.0237753	3.009921	0.0408181
cg23728189	6.1842577	1.0616913	36.02275	0.0427087
cg26143053	2.2165725	0.7917087	6.20581	0.1296916

cg05365670	0.3227074	0.111103	0.93733	0.037624
cg15553814	0.8344265	0.4222082	1.64911	0.6025251
cg08401657	0.6178247	0.3113052	1.226151	0.1685197
cg08690859	5.2240306	1.5758292	17.31818	0.0068573
cg26204079	0.2851165	0.1063275	0.764538	0.0126508
cg00170213	0.0014978	2.49E-07	9.010567	0.1429691
cg21169267	0.1075994	0.0296643	0.390289	0.000696
cg08944086	2.1404537	0.9904339	4.625793	0.0529269
cg08352774	1.6656654	0.5495486	5.048582	0.367148
cg00935015	0.0001191	4.22E-15	3357519	0.4617315
cg27343216	7.339E+11	1.2959888	4.16E+23	0.0478451
cg13613815	0.2627803	0.1063819	0.64911	0.003772
cg04043382	2.3667827	0.9652929	5.803068	0.0597317
cg14269083	1.1318983	0.499823	2.563295	0.7664059
cg19303898	0.4189562	0.197815	0.887316	0.0230736
cg23645885	0.1155867	0.0082438	1.620636	0.1092465
cg23389215	0.5134789	0.2106819	1.251463	0.1425238
cg13536703	2.8265294	1.0273579	7.776519	0.0441953
cg11525409	0.3721985	0.1359955	1.018649	0.0543554
cg21501241	0.7239364	0.35733	1.466666	0.3698335
cg12686260	3.0055772	0.661803	13.64982	0.1540637
cg00348802	0.362714	0.1327257	0.991228	0.0480257
cg00686132	1.1343625	0.4762896	2.701672	0.7758461
cg15377933	0.40196	0.1895079	0.852586	0.0175172
cg18175672	0.0654664	0.0006081	7.047727	0.2534577
cg14488957	2.98E-20	2.56E-30	3.47E-10	0.0001437
cg20116138	2.7990549	0.3799186	20.62207	0.3124222
cg00636675	0.3365229	0.1248103	0.907358	0.0313919
cg10702818	1.2544461	0.4866019	3.233927	0.638943
cg04260633	0.2073542	0.0404114	1.063952	0.0593398
cg03065165	0.1853427	0.0693473	0.49536	0.0007781
cg15679064	0.2907338	0.1224036	0.690553	0.0051286
cg03461851	3.390507	1.3066393	8.79779	0.0120825
cg23600311	0.5603824	0.2796653	1.122872	0.1024362
cg16834187	0.1187108	0.0185541	0.759523	0.0244213
cg21481950	0.4068175	0.1538805	1.075513	0.0698008
cg09726240	0.1643145	0.0331739	0.813871	0.0269494
cg18237616	0.3331334	0.1603285	0.69219	0.0032198
cg08385211	1.138805	0.5276917	2.457641	0.7405046
cg09536336	0.6617713	0.3337797	1.312067	0.2371273
cg26393983	14725.873	1.91E-08	1.14E+16	0.4919627
cg19523082	1.6601533	0.8624181	3.195792	0.1292651
cg03598074	0.719041	0.3741893	1.381707	0.3222915
cg08208317	0.5263381	0.2390862	1.158711	0.1109161
cg07056967	0.605437	0.0695152	5.273001	0.6495364
cg01043370	4.447E+12	7.60E-05	2.60E+29	0.1392819
cg05591769	1.5848168	0.5845373	4.296808	0.365544
cg14405603	0.4262142	0.2007241	0.905016	0.0264368
cg07340145	4.1250595	1.4148293	12.02697	0.0094439
cg05226008	1.228081	0.6461845	2.333982	0.5305888
cg15880348	0.3760249	0.1501859	0.941465	0.0367279
cg11748354	2.4441309	0.8778801	6.804775	0.0871448
cg04097639	1.4577767	0.8367553	2.539706	0.1832787
cg09645336	0.2071162	0.0801539	0.535184	0.0011515
cg16295133	0.6257048	0.277645	1.410098	0.2580533
cg15187175	0.7914796	0.431433	1.451998	0.4500393
cg12810837	0.8732247	0.3379811	2.256106	0.7795417
cg06003429	2.4099631	0.8247987	7.041624	0.1078627

cg09539058	1.0130883	0.1200734	8.54767	0.9904651
cg06491415	3.2735836	1.1776389	9.09986	0.0230004
cg20981127	0.086991	0.0118134	0.64058	0.0165218
cg26665224	0.0986435	0.0018544	5.247354	0.253299
cg22162694	0.3116542	0.1075459	0.903134	0.0317418
cg12290486	0.7702314	0.2051725	2.8915	0.698903
cg17270257	0.4846004	0.1557018	1.508252	0.2110969
cg00155314	0.9993745	0.0996272	10.02486	0.9995756
cg13434411	1.6005617	0.8411064	3.045747	0.1519032
cg07331478	0.4648651	0.2306889	0.936758	0.0321364
cg11348188	1.7992985	0.9724154	3.329313	0.0613628
cg03311134	1.2477295	0.5210609	2.987806	0.6193484
cg16368146	3.6553034	0.6729845	19.85372	0.1332862
cg04075973	0.003901	3.35E-05	0.454789	0.0223426
cg08815340	0.5170553	0.2780301	0.961573	0.0371826
cg20786876	0.7161296	0.3518687	1.45748	0.3570835
cg05038288	0.2221032	0.0846692	0.582618	0.0022291
cg13152690	2.1733341	0.7043186	6.706313	0.1769351
cg10397070	2.87E-07	4.35E-14	1.889082	0.0600315
cg22507406	4.4432415	0.0263106	750.3601	0.5687527
cg04998498	0.0803919	0.0069632	0.928139	0.0434132
cg22414192	1.952107	0.6054045	6.294505	0.2627944
cg12487088	0.1824127	0.0417833	0.796356	0.0236485
cg01119072	2.8211637	0.6932361	11.48089	0.1475258
cg13031551	0.6744472	0.1012367	4.493222	0.6839664
cg08711674	0.259536	0.0636978	1.057478	0.0598376
cg23682913	2.1073986	0.5120574	8.673107	0.3017351
cg15246511	2.1451344	0.8128988	5.660731	0.1231819
cg25605313	0.4251916	0.1947397	0.928357	0.031829
cg26358590	0.4645495	0.2046486	1.054521	0.0667965
cg05910443	0.4928099	0.2305596	1.053357	0.0678745
cg06919693	0.1325522	0.0314691	0.558328	0.0058811
cg07452164	0.034338	0.0020665	0.570578	0.018709
cg08937102	0.3231435	0.1221834	0.854631	0.0228144
cg18254850	1.086972	0.6277464	1.882142	0.7659167
cg03327263	1.338847	0.7057268	2.539951	0.3717611
cg00340855	2.2593839	1.1466819	4.451815	0.0184974
cg01792592	1.5459357	0.3988099	5.992622	0.528583
cg16719404	2.0889046	0.9406907	4.638637	0.0703338
cg01134296	0.1667861	0.0287168	0.968688	0.0459985
cg15836660	0.000207	4.67E-12	9174.305	0.3450211
cg04284993	0.7282622	0.2445797	2.168479	0.5689546
cg08087849	4.9854881	1.0459212	23.76383	0.0437671
cg21144890	0.3408593	0.1231499	0.943445	0.0382614
cg24077501	3.91E-11	1.91E-18	0.000801	0.0052698
cg15618336	1.3466542	0.5674502	3.195836	0.4996916
cg14230696	1.4521742	0.7382953	2.856323	0.2797496
cg09002049	1.020474	0.5760843	1.807665	0.9446124
cg03560256	1.6359602	0.6005066	4.456847	0.3357363
cg07612655	2.6284884	0.8540343	8.089782	0.0920123
cg00561322	0.3191532	0.1080591	0.942621	0.0387431
cg26316946	3.0082434	1.3574471	6.666579	0.0066741
cg22016094	0.7239262	0.3361804	1.558893	0.4090844
cg01730148	0.4796587	0.138227	1.664453	0.2471298
cg05753328	0.4360142	0.2092825	0.908382	0.0266536
cg17611674	1.6296861	0.6822232	3.892973	0.2716538
cg13520520	0.0818783	0.0092192	0.727184	0.0247125
cg05211068	0.1755346	0.0506979	0.607766	0.006036

cg00455747	14.484623	1.3960199	150.2875	0.0251254
cg10836173	0.0407865	0.0064152	0.259311	0.0006985
cg18120576	0.566891	0.1622524	1.980651	0.3738737
cg09317502	0.3720476	0.1030364	1.343402	0.1312156
cg12359001	0.1258615	0.0363138	0.436229	0.0010828
cg11064537	0.3833901	0.1817357	0.808801	0.0118322
cg03376712	7.7699762	0.5625762	107.3144	0.1258809
cg05257772	2.9297607	1.1050037	7.767846	0.0307211
cg15715969	0.3083715	0.0249859	3.805867	0.358854
cg08478871	0.0230892	0.0012137	0.439248	0.0121639
cg11738921	2.0794602	0.935105	4.624245	0.0725873
cg24315209	0.7525379	0.1902833	2.976159	0.6852766
cg10682560	1.8433918	0.9235257	3.679479	0.0828539
cg03340119	1.3985345	0.5944388	3.290328	0.4422451
cg24752836	0.3899594	0.1889038	0.805004	0.0108806
cg02134839	3.9676728	0.5216075	30.1806	0.1830986
cg21522289	2.165331	0.6675612	7.023563	0.1981517
cg23120518	0.5420883	0.2678402	1.097146	0.0887123
cg16897264	1.8302302	0.8713881	3.844145	0.1104054
cg05514299	0.3827304	0.1133298	1.292533	0.1219318
cg05565697	1.8055216	0.1136787	28.67651	0.6753713
cg12051614	0.1960754	0.0461997	0.83216	0.027169
cg07757702	0.5870682	0.1739703	1.98108	0.3907304
cg11417323	1.5531403	0.5486221	4.396916	0.4069668
cg21971919	1.46E-18	2.42E-37	8.85834	0.0627296
cg19642230	0.0008402	2.02E-08	34.97087	0.1919026
cg18200270	8.247E+09	3.31E-08	2.05E+27	0.2638881
cg19494252	0.8219635	0.4316688	1.565144	0.5507367
cg18867653	0.0112589	6.14E-05	2.062995	0.0914919
cg24311416	2.0280538	0.7619563	5.39795	0.1568766
cg23308950	149.14484	0.727144	30591.17	0.0653795
cg00660163	7.56E-40	1.87E-80	30.47559	0.0589809
cg27066326	8.47E-29	3.87E-56	0.185345	0.0441734
cg08705942	1.8636028	0.8569955	4.052548	0.1162742
cg24716558	1.1732711	0.3955248	3.480351	0.7733182
cg27227250	1.8067116	0.6532759	4.996674	0.254428
cg20707230	0.4158707	0.1974006	0.876129	0.02101
cg16794061	1.8208323	0.6350844	5.220457	0.2647794
cg21229179	28.814221	0.3886145	2136.46	0.1260773
cg05200313	2.876582	0.6857422	12.06681	0.1486576
cg13947534	2.8426724	1.0691178	7.558368	0.0362676
cg13331179	5.7096689	1.4366843	22.69136	0.0133369
cg24214699	1.3924867	0.4762747	4.07122	0.545271
cg17812951	323.95234	5.5587496	18879.27	0.0053199
cg04047827	0.716345	4.22E-16	1.22E+15	0.9851246
cg17865752	7.46E+23	0.0001321	4.22E+51	0.0917937
cg25076325	1.3418274	0.5024046	3.583767	0.5574534
cg08229905	3.0446573	1.0038116	9.234739	0.0492197
cg02084087	1.3579612	0.7171669	2.57131	0.3475445
cg11832408	0.1211353	0.0137513	1.067079	0.0572393
cg22538959	0.1918497	0.0428908	0.85814	0.0307631
cg17295573	0.6908062	0.3736993	1.276998	0.2380124
cg17330983	2.2574044	3.33E-18	1.53E+18	0.9689959
cg07179075	0.4947463	0.2415401	1.013388	0.0544036
cg20547015	1.0753181	0.447002	2.58681	0.8711975
cg03604892	1.5254342	0.6747864	3.448424	0.310235
cg15576338	0.8613644	0.4476723	1.657348	0.654921
cg05865451	4.75E-05	1.07E-23	2.1E+14	0.6494601

cg08496086	0.4066133	0.1159185	1.426298	0.1598988
cg25859141	1.3916928	0.5869368	3.299859	0.4530527
cg20705390	0.2865619	0.1029772	0.797436	0.0166909
cg24046159	0.1227837	0.0127889	1.178821	0.0691549
cg18735136	1.3156679	0.1893042	9.143919	0.7815136
cg25988603	1.266207	0.0827332	19.37891	0.8653518
cg00543684	2.0277018	0.5974718	6.881621	0.2568591
cg15473502	0.6000262	0.2895585	1.243381	0.1694435
cg14483667	6.2582137	1.169778	33.48092	0.0320947
cg03223072	0.3978929	0.1299981	1.217855	0.1063867
cg08692130	9.445E+10	0.1460678	6.11E+22	0.0685578
cg00075975	1.7180074	0.7718873	3.823809	0.1849409
cg06745704	1.4330269	0.6216711	3.3033	0.3984547
cg01833057	0.2514302	0.1141112	0.553996	0.0006143
cg15409746	7.066E+10	1.95E-11	2.56E+32	0.3239777
cg05214546	5966.9826	0.1709416	2.08E+08	0.1033154
cg13502403	3.3694217	0.8780357	12.93	0.0766607
cg00439318	1.99E+11	101.51244	3.90E+20	0.0171636
cg03123608	6.95E-05	2.67E-08	0.180598	0.0170061
cg14764956	0.1537479	0.0155883	1.516425	0.1088405
cg19848684	2.1713075	0.0020039	2352.696	0.8278487
cg04384626	2.190609	1.0981602	4.369825	0.0260322
cg10354845	8.773E+11	0.0397263	1.94E+25	0.0793966
cg03354590	1.3589221	0.6195493	2.980665	0.4440957
cg27137003	589690.26	3.61E-08	9.64E+18	0.3920132
cg01907194	0.21295	0.0791478	0.572949	0.0021921
cg23631930	0.3437105	0.1347972	0.876405	0.0253382
cg15023686	0.2981389	0.0852293	1.042914	0.058199
cg16055869	1.6878212	0.7379551	3.860317	0.2149507
cg23085023	0.1112195	4.90E-05	252.2427	0.5774539
cg01438467	4.1516276	0.3995864	43.13463	0.2333039
cg22813542	0.4325518	0.1924255	0.97233	0.0425744
cg05657800	1.1949832	0.4243878	3.364811	0.73593
cg25191598	0.0281008	0.0018088	0.436565	0.010706
cg06894464	3.908187	1.1888403	12.84775	0.0247783
cg19857541	0.4928227	0.2316453	1.048474	0.0661994
cg03541909	0.3348194	0.100652	1.113779	0.0743842
cg04619381	2.7199801	1.0430511	7.092934	0.040741
cg21516291	0.244035	0.0970072	0.613904	0.0027303
cg21112391	3.2206692	0.2469065	42.01068	0.3721012
cg23914535	0.633571	0.3361994	1.193971	0.1580633
cg26767974	0.6277685	0.2948659	1.336517	0.2271994
cg00569276	1.6880233	0.6990208	4.076306	0.2444541
cg01788025	21.169099	0.0044305	101146.6	0.4800564
cg24224304	0.0703394	0.0140397	0.352404	0.0012444
cg12409821	1.7969491	0.7035166	4.589836	0.2205885
cg14557202	0.2978634	0.1184278	0.749171	0.0100634
cg13569146	1.577563	0.657745	3.783693	0.307081
cg05002166	2.7029772	0.7469181	9.781642	0.1296986
cg09115026	0.8419393	0.4124033	1.718856	0.6365894
cg14637111	2.3510257	1.0679223	5.175771	0.0337388
cg15851696	0.4077599	0.2003177	0.830022	0.0133723
cg27038197	0.2830522	0.0007991	100.2654	0.6734477
cg03470331	0.6165001	0.2853799	1.331812	0.2183878
cg17568707	1.5370088	0.8235106	2.868689	0.1769931
cg08791424	0.2743765	0.032688	2.303063	0.2334902
cg12268236	0.4008889	0.1883607	0.853213	0.0176975
cg03651613	13.587794	0.9665286	191.0219	0.0530247

cg12308746	1.9987243	0.7757898	5.14946	0.151518
cg02982734	2.7976962	0.862001	9.080156	0.0867599
cg08776908	1.0593844	0.3859896	2.907579	0.9108332
cg09624942	8.2161896	1.2220966	55.23767	0.0302913
cg07027613	1.1716668	0.5619346	2.442995	0.6726004
cg25500028	2.594676	1.0440682	6.448184	0.0400908
cg23466916	9406.0269	33.725408	2623344	0.0014496
cg04986555	3.6594638	1.0262971	13.04854	0.0455024
cg14255337	1.0847661	0.4043416	2.910206	0.8716251
cg06311355	0.0647339	0.0156476	0.267803	0.0001578
cg12414301	0.3246195	0.0959109	1.098705	0.0705066
cg03895593	0.3308233	0.1478121	0.740427	0.0071219
cg22110655	3.5352287	0.7605191	16.4333	0.1072299
cg04375036	0.4050236	0.1781081	0.921037	0.0310681
cg02477931	5.1188441	1.5177775	17.26377	0.0084718
cg13455410	1.2886397	0.6477754	2.563531	0.4699091
cg20944283	0.7444866	0.2963823	1.870086	0.5300809
cg19942640	3.22E+24	1835.6309	5.65E+45	0.0237545
cg08284443	1.5948226	0.577579	4.403656	0.3677366
cg19563270	0.0280521	5.72E-05	13.76391	0.2582642
cg01357958	0.2058306	0.0638364	0.663669	0.0081374
cg05866214	3.8259381	1.3689138	10.69301	0.0105038
cg08750547	7.9809971	0.153263	415.6014	0.3030425
cg06288684	2.7448991	0.9011451	8.360996	0.0756006
cg14500486	1.4781396	0.7900958	2.765357	0.2214179
cg10317778	0.0045582	2.19E-07	94.80695	0.2879291
cg27165033	0.16731	0.0527483	0.530684	0.0023993
cg06551905	1.7372744	0.8117265	3.718152	0.1548321
cg04181038	2.2937583	0.9878615	5.325976	0.0534151
cg00513316	3.5410457	1.1856247	10.57586	0.0235145
cg04380681	2.5680629	0.7330354	8.996764	0.1403591
cg24651824	0.3754772	0.0183296	7.69157	0.5249104
cg09169779	5.3642544	0.9524705	30.21114	0.0568132
cg17827477	2.5437858	1.0290578	6.288127	0.0431767
cg09533845	1.56527	0.5995383	4.086595	0.3601397
cg05897169	2.4887524	0.3568387	17.35767	0.3575227
cg23696808	4.41E-07	1.55E-11	0.012562	0.0051675
cg14016257	0.1497769	0.0404245	0.554939	0.0044938
cg11412466	1.7830389	0.8714985	3.648001	0.113333
cg00983482	0.3647949	0.1443753	0.921732	0.0329825
cg01376763	3.2529684	1.0592366	9.990027	0.0393516
cg13206063	0.1180625	0.0439746	0.316973	2.23E-05
cg18493113	0.5020483	0.2425636	1.039119	0.0633719
cg07968520	1.3321367	0.4011407	4.423855	0.639558
cg02788857	11.840123	0.4915897	285.1738	0.1278802
cg02768694	5.7468532	1.0913893	30.26081	0.039099
cg25545323	0.3331327	0.1322187	0.839347	0.0197316
cg16964748	0.2250887	0.0680341	0.744699	0.0145724
cg03094935	3.73E+20	38304012	3.63E+33	0.0019073
cg10397082	1.2631593	0.5413484	2.947402	0.5889265
cg15504041	1.3327828	0.4350189	4.083294	0.6150517
cg25258233	1.7517718	0.8830172	3.475249	0.1087113
cg03963198	0.1918093	0.0509217	0.722497	0.0146737
cg15872079	1.5172579	0.3942899	5.838526	0.544274
cg03957884	0.6865828	0.2295025	2.05399	0.5012259
cg11669284	1.8981963	0.9404551	3.831283	0.0736739
cg24041556	0.3204178	0.1131662	0.907228	0.0320879
cg24838349	0.3278145	0.0826843	1.29967	0.1125126

cg06642945	11.296586	0.6053913	210.794	0.1044129
cg21508023	0.0228994	0.0003209	1.634249	0.0828485
cg23230554	0.4353871	0.1614695	1.17398	0.1003777
cg10175795	0.1608179	0.0360102	0.718197	0.0166886
cg17348667	86.417549	1.0961436	6812.97	0.0453751
cg14094027	0.5284519	0.1689661	1.652766	0.272943
cg19612574	0.4692014	0.2089204	1.05375	0.0667826
cg18272633	2.7412454	1.1330412	6.632086	0.0252829
cg24319508	0.2722668	0.1185083	0.625519	0.0021733
cg09391949	0.2296888	0.042935	1.228763	0.0855777
cg01571842	0.3582399	0.1432832	0.89568	0.0281205
cg13212186	5.78992	1.0930939	30.66815	0.0389597
cg22539420	2.8679303	1.1361619	7.239306	0.0257356
cg04521333	7.153186	1.6207398	31.57081	0.0093924
cg18256471	0.3751567	0.1440154	0.977274	0.0447472
cg07440264	0.4017563	0.1356814	1.189611	0.0996656
cg20187011	0.3301981	0.1563277	0.69745	0.0036791
cg04064550	0.1658545	0.059432	0.462843	0.0006009
cg13670057	0.3764695	0.1676013	0.845633	0.0179789
cg07905673	7.85E-05	2.93E-09	2.103487	0.0692125
cg16606256	0.699075	0.3389838	1.441679	0.3323426
cg24313597	0.3643903	0.1371147	0.968388	0.0429313
cg13735469	3.5007532	0.9264343	13.22843	0.0647027
cg13928759	1.5684578	0.6130348	4.012921	0.3477065
cg18302890	0.9511415	0.4192576	2.157791	0.9046011
cg00203124	2.8740004	0.9482377	8.710768	0.0620388
cg05049335	0.0661771	0.0101264	0.432476	0.0045804
cg27470066	1.0603437	0.3104584	3.621512	0.92551
cg13710842	517336183	0.0111746	2.4E+19	0.1093115
cg19502936	0.2275478	0.0892565	0.580103	0.0019324
cg20652042	7.1706768	0.9524907	53.98332	0.0557859
cg01044293	1.7993076	0.7295225	4.437845	0.2022078
cg18767964	0.1492774	0.045842	0.486099	0.0015913
cg12869334	0.6298876	0.2620627	1.513982	0.3015903
cg04557423	0.4733203	0.1193964	1.876372	0.2871485
cg12220788	1.5414361	0.7789952	3.050115	0.2139753
cg07635271	1786.2419	3.40E-05	9.39E+10	0.4090826
cg14727512	0.5164211	0.0689238	3.869355	0.5201411
cg04855216	1.2025056	0.4133461	3.498327	0.7350179
cg11998619	4.0169812	0.4928207	32.74241	0.1939594
cg24637035	2.2536807	1.0447971	4.861304	0.0382941
cg27506254	2.2318659	1.0183618	4.89141	0.0449188
cg04275404	1.6871763	0.6682309	4.259851	0.268344
cg14523734	2.0044932	0.3214479	12.49967	0.4564831
cg11894422	4.0977053	0.9885454	16.98575	0.0518853
cg03904220	0.4074231	0.1582563	1.048891	0.06274
cg18794404	1.5421794	0.770862	3.08527	0.2208021
cg01781963	3.6297463	0.8233563	16.00165	0.0885349
cg21241862	5.1648261	0.0503142	530.1773	0.4871598
cg25894160	0.3827304	0.1391585	1.052631	0.0628012
cg25903143	0.2079234	0.0647801	0.667367	0.008299
cg05944661	1.4464887	0.450579	4.643647	0.5350572
cg13944468	1.0920025	0.557232	2.139987	0.7976419
cg21847800	0.4248048	0.2138188	0.843982	0.0145157
cg07731535	2.3509325	0.4100615	13.47818	0.3373461
cg25690589	3.4668507	0.9656825	12.44618	0.0565962
cg02804819	0.6882433	0.2989885	1.584271	0.3797834
cg25214684	621872.26	9.32E-28	4.15E+38	0.7293826

cg13488011	1.1474413	0.383108	3.436685	0.8058887
cg19049964	1.3562108	0.6849324	2.685386	0.3820106
cg26229618	0.7197143	0.3354371	1.54422	0.3984441
cg05036314	0.3789237	0.1577183	0.910377	0.0300127
cg13470069	0.0765958	0.007658	0.766119	0.0287631
cg17311587	0.0265482	0.0017353	0.406168	0.009125
cg00414292	5.4687296	1.3346035	22.4089	0.0182227
cg26014570	4.6619864	1.1584347	18.76163	0.0302359
cg03847373	1.4980521	0.7806184	2.874849	0.2242659
cg05851542	0.2568117	0.0934146	0.706017	0.0084227
cg03986562	0.5512051	0.2228883	1.363136	0.1972669
cg10861135	1.6757524	0.8767609	3.202864	0.1182815
cg10205928	1.9683702	0.6945172	5.578669	0.2026236
cg10086328	0.356747	0.043393	2.932929	0.3375978
cg22152082	0.4324115	0.1734353	1.078095	0.0720754
cg07020846	89.974197	0.4038766	20044.13	0.1028341
cg08820025	1.7882542	0.5894822	5.424851	0.3046347
cg10051814	0.9852612	0.5491885	1.767589	0.9602871
cg01737592	1.3066524	0.0069714	244.9074	0.9202097
cg17810878	4.2700837	0.6236027	29.23915	0.1391767
cg07679370	0.48755	0.1733319	1.371386	0.1733795
cg09228327	0.5060103	0.2540711	1.007775	0.0526316
cg17837330	0.2828507	0.0859327	0.931014	0.0377498
cg15646359	4.0954063	0.8618711	19.46039	0.0762246
cg17226042	4.2977581	0.6063011	30.46461	0.1445089
cg00959883	3.1208498	0.9708125	10.03253	0.0561016
cg20177310	89.280666	2.4422207	3263.848	0.0144353
cg18664336	1311243.5	6.8328758	2.52E+11	0.0232323
cg23393242	2.9279252	1.2624972	6.790309	0.0123126
cg05445291	0.3168167	0.130801	0.767371	0.0108776
cg00057791	4981.8748	1.1284338	21994268	0.0467916
cg08610986	38.765835	0.0377383	39821.36	0.3012542
cg22454769	0.3037559	0.1150557	0.801939	0.0161468
cg18315638	0.0006561	2.64E-06	0.163174	0.0092112
cg15563382	0.0017325	1.92E-05	0.155955	0.0056178
cg21571166	2.0509103	0.8703232	4.832955	0.1005103
cg14106308	1.2104038	0.648323	2.259796	0.5488576
cg02054697	2.2881062	0.6669428	7.849894	0.1881801
cg14392966	71.559642	4.58E-35	1.12E+38	0.9200002
cg13060114	0.240954	0.0844337	0.687627	0.0078154
cg00866215	3.3289052	0.4440314	24.95682	0.2419672
cg15722293	1.3842767	0.5441373	3.521578	0.4948788
cg07725206	0.4730273	0.196561	1.138348	0.094768
cg05495790	1.6401095	0.445641	6.036156	0.4567454
cg11469587	1.9958313	0.4963427	8.025388	0.3303832
cg12038298	1.6935538	0.648533	4.422481	0.2820464
cg18766754	0.0003048	4.36E-11	2129.812	0.3140074
cg00122254	5357.6478	0.2516903	1.14E+08	0.0912867
cg24496586	1.78E-06	1.78E-41	1.78E+29	0.7474832
cg15130788	1.721753	0.4295242	6.901668	0.4430754
cg06154159	6.0876351	0.6291981	58.89926	0.1187936
cg15097584	0.0530656	0.0073702	0.382073	0.0035543
cg16774528	1.3356578	0.0175962	101.3844	0.8957577
cg14347219	2.1115249	0.6788971	6.567324	0.1967022
cg13058581	12.070061	3.57E-36	4.09E+37	0.9537182
cg16283753	1.73E-23	4.74E-42	6.29E-05	0.0162361
cg09654471	0.4956058	0.2195097	1.118971	0.0911372
cg18109369	0.2248149	0.085635	0.590199	0.0024397

cg17877566	0.0113202	3.56E-05	3.599878	0.1274422
cg24177611	1.5862492	0.6591522	3.817307	0.3031411
cg22475867	1.6833825	0.7058127	4.014914	0.240254
cg00219855	0.87572	0.3416059	2.244943	0.7823187
cg08717880	4.8575922	1.2816807	18.41036	0.0200701
cg27292079	2.3754688	0.8491784	6.645073	0.099256
cg20959676	1.08E-09	4.95E-23	23590.92	0.187682
cg01327474	532811.32	2.8939248	9.81E+10	0.0330269
cg03335938	1.942807	0.8769751	4.303998	0.1017379
cg18368845	1.7390355	0.1963573	15.40174	0.6190349
cg02564134	1.7038043	0.8631656	3.363143	0.1245764
cg05820859	0.1514474	0.0445955	0.514319	0.002479
cg26248878	1.2938468	0.3020346	5.542542	0.7285406
cg08149581	0.0820724	0.0108679	0.619797	0.0153633
cg16857641	1.2250256	0.2426075	6.185662	0.805942
cg23270523	0.1969871	0.066732	0.581489	0.0032648
cg00644416	0.0705412	1.32E-08	375853.4	0.7372206
cg03270332	0.1585938	0.0333256	0.754735	0.0206956
cg08380477	2.0816794	0.7131381	6.076508	0.1797861
cg23599056	3.17E-06	4.97E-15	2017.794	0.22086
cg24013620	1.0505916	0.5520434	1.999377	0.8805089
cg15717225	1.2691772	0.5572082	2.89086	0.5703443
cg00061769	3.83E+18	0.0045986	3.19E+39	0.0816862
cg00519537	0.3526304	0.1272073	0.977524	0.0451063
cg09981361	0.2785433	0.1026694	0.755691	0.012071
cg00730694	0.6538427	0.3706405	1.153437	0.1423537
cg27228578	0.0279265	0.0020776	0.375386	0.0069542
cg19657351	0.0248083	0.0010198	0.603482	0.0232006
cg07054208	2.1227087	0.684789	6.579972	0.1922375
cg04466898	1.9382317	0.6335134	5.930013	0.24609
cg10923838	0.2265054	0.0828736	0.619072	0.0037948
cg00090431	1.5141684	0.5201967	4.407382	0.4466237
cg10538433	0.1647948	0.0436383	0.622327	0.0078245
cg25396488	1.3200123	0.4120756	4.22843	0.6401988
cg05043311	0.1081756	0.0281083	0.416317	0.001219
cg18599110	4.23E-10	3.01E-21	59.5393	0.0993653
cg16061528	1.6102977	0.6920958	3.746676	0.2688277
cg04724477	0.173817	0.0389408	0.775854	0.0218783
cg08496016	1.552E+11	0.0174511	1.38E+24	0.0902942
cg14235811	2.0716803	0.5391658	7.960184	0.2889075
cg04342999	195903269	3376.3313	1.14E+13	0.0006455
cg10365562	1.5581629	0.7203813	3.370259	0.259853
cg06191357	2.3654794	0.543048	10.30386	0.2514836
cg11003012	2.8753246	0.9910447	8.342198	0.0519665
cg13935577	0.1699902	0.0415733	0.695078	0.0136562
cg07580762	0.1276447	0.020162	0.808113	0.0287982
cg10575261	0.0991966	0.0212238	0.463629	0.0033141
cg03147002	0.005303	0.0001232	0.228305	0.0063446
cg01819512	4.4098409	1.0636783	18.2825	0.0408504
cg14633329	2.3670047	0.739895	7.572306	0.1464385
cg25456593	0.1019558	0.0131736	0.789076	0.0287528
cg12612296	2.4722433	0.9551399	6.399049	0.062129
cg11898212	2865.4956	1.62E-06	5.08E+12	0.4637711
cg12744031	3.2529142	1.3270776	7.973498	0.0099209
cg11904435	0.3315183	0.1512115	0.726825	0.0058405
cg04290826	0.4005937	0.1320885	1.214907	0.1060792
cg24274272	1203.4342	0.5348806	2707621	0.0716903
cg00010954	3.0359064	1.2945653	7.119554	0.0106602

cg24928161	6.6154878	0.0238608	1834.165	0.5103122
cg09016968	2.2213783	0.7321775	6.739515	0.1586996
cg25457927	3.3172568	1.0724642	10.26066	0.0373978
cg02078039	0.0084619	0.0001076	0.665289	0.0321156
cg04058685	1.789708	0.6674509	4.798937	0.2474363
cg14667585	1.7761339	0.7986037	3.950209	0.1589741
cg09443360	2.1062464	0.7726993	5.741268	0.1454051
cg16710604	0.514298	0.1811724	1.459949	0.2116181
cg23742930	3.3198951	1.1788324	9.349678	0.0231229
cg08807892	2.0432976	1.0766298	3.877902	0.0288285
cg26082320	1.4576392	7.54E-11	2.82E+10	0.975124
cg07153168	1.4034452	0.3845463	5.122032	0.6078703
cg27523577	1.3495127	0.3873055	4.702191	0.6379014
cg17840469	3.1911857	0.7417403	13.72942	0.1190751
cg03524147	0.2617506	0.1098299	0.623814	0.0024866
cg10043090	0.3984192	0.1771342	0.896145	0.0260747
cg26967619	0.0515847	0.0087295	0.304826	0.001073
cg25644174	0.0849864	0.0079487	0.908659	0.0414306
cg07340870	5.97E-05	8.00E-16	4457590	0.4464195
cg08269742	1.0490722	0.4569058	2.408707	0.9100587
cg22110973	0.7410766	0.3668584	1.49702	0.4035626
cg19029181	2.6355659	0.8638107	8.041355	0.0886182
cg04752871	1.572282	0.6781878	3.645112	0.2915165
cg24955196	10.117364	0.9536051	107.3411	0.0547897
cg10262747	0.690194	0.3280919	1.451934	0.3284718
cg16808587	3.9793263	1.0318332	15.34651	0.0449131
cg25744957	1.9270581	0.4496921	8.257991	0.3769409
cg21527411	0.8704461	0.4645184	1.631101	0.664994
cg26577320	0.410165	0.1788163	0.940828	0.0353816
cg02060732	0.2194131	0.0675834	0.712336	0.0115853
cg09106903	1.5634711	0.8401413	2.909561	0.158453
cg03077077	2.5680069	0.5099566	12.93181	0.2528401
cg05221664	2.1924663	1.1128516	4.319452	0.0232672
cg10623913	1.6542006	0.482008	5.677042	0.4237132
cg13462622	2.7024843	0.7892289	9.25387	0.1134081
cg09816018	0.2947847	0.0810031	1.072774	0.0638281
cg00015373	8.06E-05	6.23E-08	0.104181	0.0099192
cg22108175	1.2581208	0.6855923	2.30876	0.4585031
cg18621299	2.627137	1.265085	5.45564	0.00958
cg14530143	0.9463182	0.5020933	1.783569	0.8645151
cg26043322	0.7863771	0.4014825	1.540264	0.4835322
cg03515587	7.7914507	1.7036734	35.63283	0.0081244
cg18049167	0.1260751	0.016106	0.986894	0.0485492
cg07465387	1.5644287	0.792099	3.089812	0.1974775
cg15366353	1.6211958	0.6546921	4.014522	0.2963157
cg05506480	1.6048451	0.7502538	3.432875	0.2227324
cg03381216	4.40E-08	1.40E-36	1.38E+21	0.6128499
cg04842553	0.2635687	0.092956	0.747327	0.0121519
cg14392677	4.1015367	1.331596	12.63341	0.0139365
cg20464804	0.0315942	0.0006121	1.63089	0.0859996
cg02792560	0.5195018	0.1194161	2.260015	0.3826567
cg04603391	2.8166475	1.0141331	7.822941	0.0469345
cg01621390	0.4891653	0.1983024	1.206655	0.1206173
cg03638795	1.4126367	0.5805674	3.437228	0.4463896
cg02903104	0.5785012	0.2848074	1.175052	0.1300777
cg08263708	1.8888373	0.815901	4.372719	0.1375699
cg19304093	3.1874114	0.4606629	22.05429	0.2401585
cg12152384	0.3784661	0.1722957	0.831342	0.0155192

cg14784922	0.5452689	0.2206723	1.347329	0.1888363
cg13603914	4.0723421	1.0351565	16.02074	0.0444939
cg08594554	0.2962321	0.1224037	0.716918	0.0069764
cg10836392	0.2330763	0.0696546	0.779913	0.0181115
cg14236976	2.8175211	0.6500713	12.21162	0.1662406
cg24688655	2.2709268	0.8233006	6.263943	0.113109
cg22027399	0.1400574	0.041655	0.470918	0.0014873
cg02492143	1.5544218	0.535186	4.514742	0.417461
cg14692377	0.3007143	0.0967966	0.934218	0.0377446
cg04999435	0.6816741	0.2784745	1.668661	0.4014873
cg15144216	0.0161166	0.0003447	0.753523	0.035359
cg25110734	1.2784638	0.6026981	2.711921	0.5219959
cg08079376	0.8268529	0.3629344	1.883772	0.6508613
cg10879958	0.0956236	0.0147647	0.619307	0.0137911
cg25164589	1.5542362	0.6030427	4.00577	0.361282
cg13908523	537791	2.48E-08	1.17E+19	0.3996804
cg05992079	2.3939986	0.8631232	6.640105	0.0935105
cg01961783	5.20E-08	3.04E-17	89.05909	0.1220767
cg25876227	1.5429763	0.6969206	3.416136	0.2848292
cg11328313	0.7864959	0.3579301	1.728203	0.5498867
cg00949670	1.93E-12	1.33E-33	2.82E+09	0.2779966
cg05100067	0.4853514	0.1962853	1.20012	0.117577
cg04718145	1.6440811	0.5898191	4.582766	0.3418184
cg16922937	0.3102962	0.1546154	0.62273	0.0009925
cg05504117	2.4023307	1.1807658	4.887669	0.0155855
cg08841098	17.695715	0.3173567	986.7079	0.1613543
cg10570241	1.7958711	1.0001804	3.224571	0.0499294
cg11369736	1.9427374	0.8346322	4.522026	0.1234103
cg05113410	1.1423952	0.4794698	2.721896	0.7637696
cg10792727	3.02E-21	2.43E-39	0.003736	0.0262203
cg20281882	2.8650021	0.3140448	26.13716	0.3507426
cg01536011	1.1240466	0.5046947	2.503455	0.7747078
cg13632520	0.2010007	0.0867931	0.46549	0.0001807
cg00176496	1.14E-05	7.41E-09	0.017504	0.0023615
cg02390209	0.0624969	0.0087597	0.44589	0.0056819
cg19694010	1.9711534	0.9768036	3.977714	0.0581659
cg09761080	1.4691784	0.532827	4.051006	0.4572381
cg18278424	0.0910288	0.0246907	0.335602	0.0003181
cg01807768	3.731469	1.2278896	11.33967	0.0202344
cg03512997	0.6347725	0.2349712	1.714832	0.3700738
cg14654495	3.6898399	0.7132134	19.08954	0.1194883
cg16504798	0.4515673	0.1353268	1.506819	0.195975
cg21746573	0.0374172	0.0025117	0.557403	0.0171231
cg01928553	0.3765198	0.1026858	1.380592	0.140627
cg17047829	0.5730226	0.2376823	1.381486	0.2149001
cg02806012	0.0955157	0.0258169	0.353383	0.0004343
cg15772366	0.2009153	0.0375951	1.073729	0.060549
cg03922381	0.542483	0.2399741	1.226332	0.1416478
cg25801976	0.3252079	0.1746396	0.605591	0.0003985
cg15802848	38736.336	0.0042317	3.55E+11	0.1964488
cg00098819	2.2859458	0.9817495	5.32269	0.055206
cg03440556	0.5838646	0.1178993	2.891433	0.5097609
cg03529261	2.8332393	0.738744	10.86607	0.1288992
cg20733108	2.501922	0.2444234	25.60972	0.4396566
cg21092808	1.6255482	0.6443539	4.100863	0.3034536
cg02573743	6.2894286	0.1775081	222.8457	0.3123833
cg04374719	1.1559313	0.3153653	4.236919	0.8269226
cg03277819	0.313623	0.1303512	0.754572	0.0096361

cg11072570	0.3766919	0.1617824	0.877084	0.0235676
cg02565891	1.4588322	0.3962041	5.371452	0.5701459
cg23819330	5.36231	0.3871971	74.26286	0.2104275
cg06627916	0.5586142	0.2238894	1.393768	0.2119412
cg19962304	76.027209	0.3760179	15371.97	0.1098475
cg10796749	2.3171997	0.8035857	6.681819	0.1198828
cg23416439	1.63E-08	6.44E-15	0.041186	0.017126
cg08886651	1.9241203	0.7716133	4.798049	0.1603698
cg10343233	2.48E+12	0.1936069	3.18E+25	0.0638341
cg22501294	81.458549	1.1665863	5687.959	0.042246
cg06187750	1.2030024	0.4962887	2.916075	0.6824523
cg13298167	0.6529341	0.2360089	1.806385	0.4116259
cg06367607	5.23E-05	1.49E-10	18.30553	0.1301243
cg11936560	0.2823568	0.1219267	0.653879	0.0031622
cg05290481	4.73E-06	2.07E-13	108.1554	0.1561185
cg16648632	2.1781798	0.8678194	5.467114	0.0973134
cg10432569	1.4567624	0.6652759	3.18989	0.3468073
cg08867439	3.44E-18	1.89E-30	6.25E-06	0.0052385
cg19340889	2.3227971	0.8222762	6.561526	0.1116904
cg04240109	1.3476735	0.6289955	2.887499	0.4428076
cg20644754	0.407585	0.1820739	0.912408	0.0290416
cg00157012	1.006242	0.2970893	3.408143	0.9920235
cg04833713	0.4513286	0.2049541	0.993869	0.0482417
cg11902408	1.0959555	0.4375958	2.744813	0.8449171
cg17882580	0.0314459	0.0031822	0.310743	0.0030764
cg05372170	0.0612161	0.0041077	0.912286	0.0427069
cg14909198	0.4024556	0.1918989	0.844041	0.0160107
cg16461345	25476.357	0.0314209	2.07E+10	0.143879
cg01309645	1.5613233	0.6976937	3.493984	0.2783326
cg19546232	1.3597069	0.7339599	2.518943	0.3286916
cg05899471	0.5336447	0.2477799	1.149313	0.1086181
cg06520258	1257.2669	12.048857	131192.5	0.0026162
cg13832201	0.1775518	0.0529627	0.595223	0.0051012
cg00260634	1.8809012	0.7878439	4.49047	0.1547676
cg20368124	2.1853505	0.9742347	4.90206	0.0578759
cg02683714	1.7423541	0.7487159	4.054673	0.1975986
cg08722104	24.747556	9.03E-07	6.78E+08	0.7134513
cg27330193	0.5349735	0.2646221	1.081529	0.0815548
cg00331343	1.08E-14	5.38E-24	2.18E-05	0.0032592
cg08844365	0.3800545	0.1621921	0.890557	0.0259642
cg02266500	0.0917906	0.0280092	0.300812	8.03E-05
cg18876826	3.7369811	1.1570815	12.06918	0.0275328
cg04907151	0.3082819	0.1366797	0.695332	0.0045746
cg25949338	0.2011791	0.0459685	0.88045	0.0332538
cg18362509	4.1893516	0.8290279	21.17018	0.0830741
cg25908635	4.35E-07	1.85E-15	102.3701	0.1363983
cg04456029	1.3358769	0.2773792	6.433673	0.7180494
cg13327952	0.0776415	0.0008052	7.486849	0.2729273
cg03338754	0.5271107	0.2498484	1.112057	0.092739
cg04623837	2.316421	0.8826775	6.079011	0.0879242
cg09996240	0.4133572	0.2185511	0.781804	0.0065878
cg11579905	0.380766	0.1616195	0.897062	0.0272151
cg06121450	0.0087813	0.0001906	0.404558	0.0153909
cg07499182	1.6816654	0.9144007	3.092734	0.0945056
cg19040483	0.0257631	0.0003953	1.679231	0.0860239
cg03698765	142.42292	1.3409511	15126.79	0.0372317
cg04434244	1.2887852	0.4577206	3.628779	0.6309879
cg17220632	0.3191256	0.1549921	0.657073	0.0019374

cg22462856	0.0444378	0.0035401	0.55781	0.015857
cg18342026	2.5871178	0.8141219	8.221346	0.1071022
cg19460214	1.8401342	0.7710396	4.391595	0.1694124
cg11963658	0.3626679	0.1932205	0.680714	0.0015931
cg01803928	2.4903829	0.7036123	8.814524	0.1571072
cg15287353	5.6638344	1.2082561	26.54985	0.0278094
cg07243519	0.9166857	4.99E-10	1.68E+09	0.9936225
cg24740195	7.249E+10	8.04E-12	6.54E+32	0.3322872
cg16681436	0.3709117	0.1674346	0.821667	0.0145261
cg22525715	0.5888901	0.2541449	1.364543	0.2168225
cg13948456	0.1477397	0.0321983	0.677894	0.0138901
cg18069493	0.3535913	0.1174754	1.064281	0.0644358
cg14013103	5.6208901	1.1164249	28.29962	0.0363039
cg22544485	1.8597536	0.9193078	3.762269	0.0843613
cg21341520	1.4292608	0.6188144	3.301129	0.4030246
cg16320208	0.5389421	0.2728506	1.064533	0.0750929
cg15990451	0.9956852	0.4055703	2.444432	0.9924709
cg14316231	0.8193346	0.2895187	2.318708	0.7073421
cg03665360	0.4182285	0.1809526	0.966635	0.0414149
cg02570888	3.6290243	0.6226034	21.15282	0.1518227
cg24924449	0.0290199	0.0005123	1.643833	0.0856793
cg01844321	0.2434997	0.0747188	0.793537	0.0190973
cg02097120	1.9540142	0.7033164	5.428811	0.1988282
cg24781699	1.1377302	0.5220022	2.479741	0.7454814
cg17871993	1.4803152	0.5111119	4.287384	0.4697078
cg02048377	0.4438731	0.1630847	1.208105	0.111858
cg01727434	2.0132983	0.4480171	9.047355	0.3613934
cg04255201	2.3171929	0.7695556	6.977252	0.1351201
cg07225255	0.5047198	0.2266405	1.123992	0.094165
cg12366960	0.403412	0.1785111	0.911659	0.029087
cg16723488	3.7186152	1.4263324	9.694864	0.0072251
cg22949738	1.4795904	0.5429138	4.032293	0.4437491
cg26635219	1.9615144	1.0501044	3.663958	0.0345733
cg02284273	0.2854607	0.0983491	0.828556	0.0211168
cg00649377	0.0271161	0.0034096	0.21565	0.0006495
cg14030719	1.9069799	0.5314171	6.84316	0.32208
cg01709045	1.7072476	0.7193538	4.051823	0.2251416
cg00283857	0.600016	0.0060109	59.89439	0.8278336
cg12414526	0.3589225	0.0912155	1.412318	0.1426425
cg16399393	1.5208371	0.6801199	3.400791	0.3072017
cg15316716	3.5115487	1.1170775	11.0386	0.0316006
cg09766856	0.3584577	0.0624354	2.057999	0.2499121
cg00791854	0.5383	0.2123341	1.364674	0.1919296
cg14155397	0.0883906	0.0001125	69.47499	0.475724
cg19097880	1.8661841	0.8274511	4.208881	0.1327054
cg15575498	0.1812839	0.0457954	0.717623	0.0149895
cg15506609	4.216815	0.872625	20.37706	0.073382
cg02528159	0.2066637	0.0506754	0.842812	0.0279198
cg01767200	0.3764004	0.1654914	0.856101	0.0197777
cg16734913	0.4190368	0.1887981	0.930051	0.032498
cg26522684	0.4216296	0.1404158	1.266036	0.1236888
cg16642281	1.147594	0.6155702	2.139434	0.6648759
cg16685388	0.1079918	0.0179683	0.649043	0.015001
cg00465247	3.0771636	1.0077598	9.396024	0.0484345
cg14335159	0.4257934	0.1707042	1.062071	0.0671259
cg04597765	3.9331726	0.0947188	163.324	0.4713371
cg00179026	2.315649	0.6591161	8.135486	0.19028
cg13669060	0.0309198	0.001523	0.627732	0.0236299

cg13572309	1.796575	0.9058302	3.56323	0.0935646
cg20453969	0.1568446	0.0448744	0.548201	0.0037144
cg06853894	0.2356102	0.0811075	0.684427	0.0078874
cg16175245	1.2852607	0.3975215	4.155486	0.6750961
cg21389456	0.3020658	0.1168408	0.780923	0.013503
cg13842295	1.609E+10	4.68E-06	5.53E+25	0.1978793
cg16991298	175158.52	0.0041934	7.32E+12	0.1774891
cg10802936	1.5412952	0.3870315	6.137979	0.5394755
cg00461578	0.2216597	0.1002621	0.490046	0.0001976
cg25384043	0.5953943	0.2964398	1.19584	0.1450292
cg01485177	0.25279	0.0653628	0.977663	0.0462945
cg23903244	2.3568505	0.959679	5.788128	0.0614582
cg09322555	3.2349116	0.4358361	24.01053	0.2510004
cg20807852	0.1093598	0.0081008	1.476345	0.0955947
cg06417752	0.113415	0.0158043	0.813889	0.0304056
cg21164369	3.7941025	0.9668158	14.8893	0.0559294
cg20276377	9.9680608	1.5191388	65.40695	0.016593
cg27055156	0.3088301	0.1233157	0.77343	0.0121255
cg07939567	5.1737648	1.7015568	15.73138	0.00377
cg03109101	1.6118945	0.6991471	3.716248	0.2626291
cg10995755	0.4224918	0.1307282	1.365424	0.1499918
cg05433637	7021165	519.48461	9.49E+10	0.0011605
cg12751644	1.2591226	0.5393278	2.939566	0.5942761
cg05117638	2.1130888	0.9112895	4.899809	0.0812493
cg12257233	0.2074089	0.0388146	1.108307	0.0658123
cg11283819	0.0818197	0.0132714	0.504428	0.0069891
cg25010508	0.2267836	0.0523842	0.981799	0.0471966
cg11821200	2.355299	0.9625884	5.763038	0.0605942
cg02057833	2.1324525	1.0960222	4.148961	0.0257502
cg13396201	3.8772287	0.6488088	23.17	0.1373666
cg16079347	6.3080607	1.6292861	24.42274	0.0076593
cg02060584	1.5229873	0.622637	3.725269	0.3566403
cg05117324	0.5177228	0.2155293	1.243621	0.1409291
cg26280578	0.5539893	0.2016986	1.521598	0.2519224
cg16278661	0.0568285	0.0130504	0.247462	0.0001332
cg18555299	5.4308569	1.263856	23.33668	0.02292
cg07047830	413039971	8.63E-05	1.98E+21	0.1829252
cg14516100	4.0226245	1.1974212	13.51363	0.0243615
cg14014041	1.9386719	0.6803915	5.52395	0.2152897
cg24585346	0.0419772	4.45E-05	39.60183	0.3642664
cg25005374	2.000926	0.4707387	8.505154	0.3474969
cg13607311	0.2748598	0.0784072	0.963533	0.0435902
cg24704396	1.4067395	0.6093941	3.24735	0.4239642
cg03763950	2.97409	0.6431005	13.75401	0.1630263
cg09835740	2.6149806	1.0554322	6.47898	0.037847
cg18218257	1.154E+09	0.2023668	6.58E+18	0.0686723
cg05255275	1.3757319	0.7786534	2.430656	0.2720154
cg24029819	0.2428162	0.0562144	1.048837	0.0579481
cg07913124	0.7166064	0.3650775	1.406618	0.3328368
cg13293488	0.2349664	0.0655791	0.841872	0.0261271
cg21355619	0.3520265	0.0001065	1163.829	0.8006394
cg00354125	1.3674353	0.3699417	5.054524	0.6389605
cg19697512	0.4592138	0.1832856	1.150539	0.0967699
cg10316834	247.97052	0.0200087	3073131	0.2515776
cg26715042	0.3101819	0.0927394	1.037453	0.0573969
cg03405473	0.2525843	0.1017635	0.626933	0.003011
cg23964386	0.0483112	0.0100027	0.233334	0.0001625
cg05613447	3.1396882	0.8263711	11.92883	0.0929692

cg05700129	1.6077328	0.7324622	3.528926	0.2365055
cg06541550	0.0013676	2.11E-45	8.86E+38	0.8931996
cg15543284	2.2969229	0.8933882	5.905445	0.0843515
cg01750654	0.2497932	0.1087388	0.573821	0.0010796
cg03169059	0.3807369	0.1535504	0.944059	0.0371407
cg07854959	0.398825	0.1800555	0.883402	0.0234816
cg21951594	7.7014822	0.5224903	113.5195	0.1369922
cg13961587	6.2146367	1.7762088	21.7439	0.0042499
cg17372223	0.0017022	5.10E-06	0.567708	0.0314794
cg09088834	0.2476608	0.077453	0.791911	0.0186049
cg10225408	4.0210467	0.9445891	17.1173	0.059723
cg07695475	1.345721	0.4835589	3.745076	0.5696256
cg20789993	16216.895	2.14E-10	1.23E+18	0.552201
cg00628382	0.2834762	0.1129315	0.711571	0.0072612
cg13443844	10.230229	1.3109934	79.83075	0.0265357
cg11938080	103.77936	0.3341611	32230.43	0.1128352
cg12081643	2.2240579	0.9417579	5.252341	0.068288
cg01341487	0.1128454	0.0305946	0.41622	0.001052
cg02593884	0.2122715	0.0813172	0.554117	0.0015459
cg21726840	0.7076616	0.0024806	201.877	0.9045783
cg21752660	0.8440893	0.2359653	3.019456	0.7943683
cg25569396	1.2842317	0.5219557	3.159753	0.5860406
cg23387287	0.4119483	0.1973981	0.859691	0.0181405
cg23893997	0.9536069	0.281666	3.228526	0.9391441
cg03964696	0.3620302	0.1277408	1.02603	0.0559254
cg16875420	0.667271	0.3078017	1.44655	0.3054626
cg03855973	0.1156931	0.0127722	1.04797	0.0550742
cg08698718	0.0037434	2.93E-05	0.47833	0.023948
cg04520793	0.0021045	8.10E-05	0.054674	0.0002083
cg15742758	2.5672195	1.0818097	6.092214	0.0324918
cg06550629	2.863087	0.8949963	9.158996	0.0762318
cg26850469	1.028443	0.0779908	13.5618	0.9829964
cg26814987	0.2896199	0.1181323	0.710049	0.0067617
cg03363743	0.9549621	0.3700445	2.464441	0.924099
cg23652026	0.6390978	3.28E-05	12446.82	0.9292086
cg05960850	0.2943285	0.0973134	0.890209	0.0303174
cg03204427	0.2182964	0.0650415	0.732661	0.0137589
cg09169739	3.0419391	0.8903376	10.39313	0.075952
cg24556026	1.2855561	0.4862686	3.398645	0.6125677
cg12584960	3.1444977	0.8619248	11.47184	0.0827501
cg02845569	1.2719225	0.4137743	3.909829	0.6746258
cg00589914	0.8728878	3.71E-16	2.05E+15	0.9939935
cg01382153	0.0568464	0.0005417	5.965269	0.2271505
cg24089887	0.3693165	0.1699662	0.802481	0.0118795
cg05670193	0.118792	0.0273993	0.515033	0.0044197
cg06822067	0.3691715	0.1348155	1.010919	0.0525216
cg27106909	2.3743709	0.8966959	6.287123	0.0817719
cg00911289	129107.23	93.851265	1.78E+08	0.0014142
cg23042612	0.0155214	3.14E-05	7.664187	0.1880473
cg08724493	2.2575923	0.5684664	8.965741	0.2471646
cg18746357	0.3681701	0.113952	1.189529	0.0949373
cg00718175	4.0595854	1.1910228	13.83704	0.0251319
cg12965426	66.746256	0.6660216	6689.066	0.0739267
cg03947203	0.1999624	0.0880771	0.453977	0.0001192
cg19246654	101.84894	1.5453205	6712.657	0.0304922
cg21696393	1.3544689	0.5024635	3.651182	0.5487169
cg14429128	1.493768	0.7002	3.186722	0.2992371
cg18120111	0.8785421	0.2139083	3.608257	0.857425

cg02628823	2.1292596	0.9322329	4.863319	0.0729018
cg18363035	2.1739974	0.7863771	6.010176	0.1344532
cg13415078	0.5393655	0.2450809	1.187016	0.1250358
cg04663692	0.5651254	0.292555	1.091647	0.0893326
cg03016446	1.451E+12	0.0030887	6.82E+26	0.1042409
cg18243846	0.4715736	0.2285222	0.97313	0.0419863
cg14644204	3.3553061	0.5135398	21.92251	0.2062053
cg06373648	0.2671257	0.100685	0.708707	0.0080112
cg21805138	0.2394998	0.1137019	0.504478	0.0001698
cg00066153	65.186439	0.0004318	9841888	0.4923548
cg24718827	1.9863166	0.7123754	5.538447	0.189612
cg02642891	0.4059415	0.1847546	0.891932	0.0247863
cg07198150	1.1655118	0.2464152	5.512719	0.8468146
cg08133486	2.2440408	1.1061639	4.552417	0.0251217
cg25341313	1.4464465	0.4995953	4.187805	0.496173
cg07048832	0.390081	0.1856773	0.819503	0.0129362
cg14042099	0.5497181	0.2584003	1.169465	0.1202996
cg27552493	5.1978932	0.7267203	37.17812	0.1005967
cg17388996	2.1555231	0.947802	4.902163	0.0669391
cg12161959	2.2884854	0.8249587	6.348398	0.11176
cg03234732	0.147706	0.0223534	0.976005	0.0471252
cg00067824	2.3828602	1.0127957	5.606286	0.0466913
cg25546588	0.9056913	0.4526499	1.812166	0.7795378
cg16405166	0.8190394	0.3816984	1.757475	0.6083382
cg20238105	0.5337681	0.202803	1.404853	0.2035548
cg02396982	0.5336799	0.2398509	1.187464	0.1238294
cg13209192	0.9782194	0.4871511	1.964305	0.9506347
cg15736553	0.198248	0.0446102	0.881016	0.0334678
cg11258102	0.1410045	0.0268717	0.739895	0.0205508
cg02426611	1.5529541	0.7304757	3.301501	0.2526951
cg12718582	0.468386	0.2040116	1.075357	0.0736743
cg07612923	1.2467661	0.6884153	2.257977	0.4667112
cg01411786	1.8697167	0.7092225	4.929117	0.2057741
cg14699728	0.4421027	0.1729726	1.129976	0.088242
cg22912095	0.678398	0.294249	1.564063	0.3625843
cg24209528	2.8884176	1.0366003	8.048383	0.0424873
cg01138020	1.3598656	0.6546156	2.824917	0.4099048
cg04861263	0.9899982	0.4209471	2.328312	0.98162
cg03090448	0.3083323	0.1197237	0.794068	0.0147808
cg07109923	0.0076036	6.89E-05	0.838692	0.0420251
cg01635742	0.373199	0.1708217	0.815338	0.013437
cg02340378	3.54E-08	1.12E-18	1119.585	0.1642762
cg02604018	2.0833285	0.7093485	6.118654	0.1817996
cg05005659	11.820053	1.1634158	120.0892	0.0368049
cg19872222	0.8767879	0.3747288	2.051502	0.761758
cg12784386	0.2862043	0.1303328	0.62849	0.001826
cg24462247	3.4850803	1.08517	11.19252	0.0359698
cg16694702	0.139822	0.0193759	1.008996	0.0510472
cg11849561	0.1567358	0.0304593	0.806523	0.0266078
cg14271231	0.0105877	1.58E-09	71008.97	0.5706459
cg14407987	0.3564742	0.1281585	0.991536	0.0481266
cg05604112	1.1397292	0.582056	2.231714	0.7028489
cg11712187	0.9377387	0.3325416	2.644343	0.9032688
cg13735819	3.1511625	1.0107481	9.824233	0.0478846
cg21355793	2.3646627	1.1645826	4.801402	0.0172381
cg18568460	3.827887	0.3048034	48.07268	0.2984743
cg00190835	0.0358628	0.0020944	0.614086	0.0216511
cg12520996	0.1067856	0.0123169	0.925817	0.0423658

cg05143420	3.4385586	0.1795556	65.84972	0.4122637
cg23963802	4.2787391	1.3620856	13.44086	0.012807
cg14580747	1.2073435	0.571606	2.550145	0.6213783
cg07860213	2.4947902	1.2942257	4.809036	0.0063296
cg27409761	1.43E-09	4.30E-19	4.785529	0.0687544
cg04627266	0.1626924	0.0386586	0.684681	0.0132644
cg12670963	3.4109495	0.092806	125.3645	0.5046246
cg00686926	0.5285139	0.2064638	1.35291	0.1836175
cg26880963	3.1918876	0.0792139	128.6156	0.5382714
cg21356630	0.861769	0.3709704	2.0019	0.7293884
cg23626122	0.3644547	0.1197678	1.10904	0.0754552
cg25320665	0.8261802	0.2186093	3.122345	0.7783393
cg06677890	1.9627722	0.5352279	7.197821	0.3090778
cg17145370	3.4984394	1.0818216	11.3134	0.0365013
cg04007303	1.4487687	0.6090794	3.446071	0.4017444
cg03173367	0.4852738	0.2050072	1.148695	0.1000435
cg05475091	0.3596113	0.1762766	0.733622	0.004931
cg14475035	0.0942158	0.0089687	0.989727	0.0490025
cg03780851	1.8055328	0.866105	3.763918	0.1149258
cg09077126	0.000915	2.27E-05	0.036965	0.0002094
cg17939295	2.1614524	1.0854665	4.304026	0.0282835
cg17522897	0.4180396	0.1448379	1.20657	0.1068014
cg06567920	1.401243	0.3310106	5.93178	0.6467856
cg08289525	1.2149007	0.5886978	2.507201	0.5984646
cg02247838	0.13491	0.0362918	0.50151	0.0027885
cg07787614	0.5152874	0.1842183	1.441339	0.2064529
cg09184378	0.5082353	0.2466442	1.04727	0.0665417
cg22620363	0.0376086	0.0020973	0.674395	0.0259175
cg04215256	0.1021109	0.0001749	59.63108	0.4826409
cg11256607	4.0344539	1.6399444	9.925226	0.0023898
cg12005760	0.0134488	0.000823	0.219768	0.002503
cg03887471	0.0103093	0.0001668	0.637073	0.0296862
cg05650628	25.157551	2.6225552	241.3304	0.0051781
cg10347032	0.0975965	0.0225253	0.422862	0.0018676
cg05894910	0.0448134	0.0012273	1.636338	0.0907069
cg03754605	3.360171	1.4230791	7.934028	0.0056952
cg26207239	6.0144136	1.0450812	34.61279	0.0445003
cg09194778	1.9924574	0.6247531	6.354329	0.2440159
cg08819022	0.5482142	0.3127471	0.960964	0.035816
cg21257950	1.5218821	0.7298472	3.173438	0.2626961
cg07175848	2.2110636	0.1001877	48.79641	0.6152357
cg02345060	0.2194172	0.0886125	0.543309	0.0010427
cg03024931	710227.12	4.558102	1.11E+11	0.0272008
cg02240030	5.1918016	0.3264385	82.57238	0.2432683
cg14415160	1.5974659	0.7659206	3.331804	0.2116902
cg22564433	1.1425475	0.5580231	2.339356	0.7155063
cg11469098	6.5454749	1.2353574	34.68085	0.0272159
cg06456154	1.7303061	0.7457881	4.014491	0.2016415
cg20165520	1.5361798	0.6237504	3.783322	0.3505372
cg07345734	1.4182942	0.8271537	2.431904	0.204012
cg07630229	0.4570667	0.1881205	1.110511	0.0838912
cg07837209	5.5171237	0.763875	39.84769	0.0904627
cg23105471	0.0515603	0.0073007	0.364139	0.0029504
cg03702975	0.0932266	0.0118237	0.735065	0.024315
cg11059800	0.0981369	0.0126438	0.761707	0.0263981
cg18520851	0.2496596	0.1184236	0.52633	0.0002657
cg03457400	3.3045248	1.257949	8.680705	0.0152804
cg15848350	0.0928965	0.025007	0.345094	0.0003868

cg04852148	3.2587693	0.6490654	16.36134	0.1512999
cg20053454	1.4924602	0.7615972	2.924692	0.2433872
cg08042538	3.1080506	0.9418107	10.25682	0.0626666
cg11652691	1.6246393	0.0660462	39.96375	0.7664797
cg14871588	0.4181715	0.1603089	1.090815	0.0747051
cg00937681	2.7998528	0.3256012	24.076	0.3483238
cg19344315	1.8714454	0.8231229	4.254903	0.1347884
cg09030672	0.3922221	0.1638654	0.938808	0.035574
cg00141447	2.6324125	1.0510967	6.592729	0.0387951
cg15470365	224.22604	1.7457007	28800.65	0.0288988
cg01716380	3.61E-07	6.90E-36	1.88E+22	0.6601329
cg06032735	0.6530903	0.3121969	1.366211	0.2579109
cg00965985	0.3391315	0.0877673	1.3104	0.1168852
cg15703035	0.3149647	0.1185182	0.837025	0.0205197
cg23937608	0.2371082	0.0819894	0.685702	0.007899
cg10631515	0.5780875	0.1690487	1.976858	0.3823387
cg14637470	0.4083091	0.1627814	1.024173	0.0562547
cg22260478	0.2347933	0.0858084	0.642453	0.00478
cg27048142	0.1787105	0.0787651	0.405477	3.80E-05
cg14616345	1.8985333	0.6518714	5.529356	0.2398323
cg18424091	0.0637103	0.0050221	0.808227	0.0336515
cg14971586	1.4736304	0.5173533	4.197493	0.4678456
cg21845794	0.7489601	0.088457	6.341399	0.7908359
cg16678545	9.1598918	0.742944	112.934	0.0839659
cg26889953	4.1639285	0.5439258	31.87622	0.1695688
cg16002963	0.4013209	0.1266416	1.271766	0.1207955
cg05949660	0.1429531	0.050244	0.406727	0.0002661
cg18096987	0.4034056	0.1395615	1.166053	0.0936808
cg11967721	0.4049496	0.1614417	1.015749	0.054022
cg08751265	1.9313097	0.9120682	4.08956	0.0855211
cg05583632	3.5927792	0.8040336	16.05413	0.0940514
cg05803237	2.2372238	0.7865084	6.363785	0.1311172
cg23820933	0.199276	0.0119306	3.328503	0.2614912
cg13681081	2.0622582	0.8651921	4.915566	0.1024224
cg01994290	3.0014333	0.6155516	14.63501	0.1739313
cg17820591	0.0001328	2.89E-08	0.609918	0.0379965
cg06814792	0.0064881	0.0001974	0.213265	0.004697
cg00449767	0.634713	0.2717748	1.482332	0.2935252
cg15929495	0.0041753	9.44E-14	1.85E+08	0.6613456
cg26783404	0.7915651	0.3365677	1.861662	0.5921733
cg14558673	1644974.8	0.4536753	5.96E+12	0.0632544
cg24000937	1.2491637	0.4981579	3.13236	0.6352769
cg17287605	0.2849625	0.0869776	0.933615	0.0381338
cg04356090	1.5197791	0.0396122	58.30856	0.8220313
cg26756396	0.7391565	0.3478526	1.570643	0.4319001
cg22460896	2.1500848	0.5982931	7.726755	0.2408314
cg16439198	1.9838695	0.9294545	4.23446	0.0765853
cg06225581	3.8570677	0.0260212	571.7247	0.5966069
cg05877109	0.0314611	0.0012187	0.812189	0.0370351
cg17109517	3.02E-05	1.42E-08	0.06414	0.0077542
cg22696358	8.2335858	3.23E-05	2100422	0.7399603
cg17967261	0.6905281	0.2521435	1.891102	0.4712793
cg21374048	0.142455	0.0185029	1.09677	0.0613082
cg24709033	0.3737822	0.1735252	0.805146	0.0119526
cg02207155	0.5539149	0.1692164	1.813192	0.3288704
cg14066471	1.7396053	0.8301873	3.645234	0.1424058
cg21238619	0.2480118	0.0921621	0.66741	0.0057706
cg08738300	1.562952	0.8769627	2.785545	0.129858

cg06555661	0.413124	0.1781302	0.958128	0.0394347
cg09247084	0.4829987	0.2534492	0.920452	0.0269734
cg22981628	0.0265064	0.0005521	1.272499	0.0660682
cg00520933	1.8916422	0.9503052	3.765433	0.069548
cg20713700	0.5847707	0.2065242	1.655771	0.3123206
cg13266292	0.1460397	0.0367221	0.580784	0.0063062
cg18876728	0.1882482	0.0453178	0.781975	0.0215365
cg10236857	0.2178106	0.0786354	0.603309	0.0033667
cg00467202	0.7312788	0.0070634	75.70946	0.8948259
cg17646721	2.3710375	0.9073476	6.195883	0.0781416
cg23000942	0.2897638	0.1259694	0.666535	0.0035636
cg04063216	0.0566442	0.0064513	0.497353	0.0095952
cg05677712	0.3403294	0.1299758	0.891121	0.0281861
cg07034362	1.3590199	0.7257794	2.544761	0.3378068
cg01814149	0.0158282	0.0001958	1.279849	0.0643322
cg10812708	1.6383984	0.7057124	3.803744	0.2506013
cg05938437	0.0259572	1.51E-15	4.47E+11	0.8143573
cg01249187	1.6941031	0.8129847	3.530184	0.1593517
cg11916054	1.0237075	0.4406692	2.378149	0.95655
cg04395377	0.2604156	0.1087397	0.623657	0.0025311
cg17202735	1.1203988	0.1487937	8.43647	0.9121182
cg06776146	2.1666017	0.9434664	4.975443	0.0683387
cg11122451	0.3898945	0.1468164	1.035427	0.0587443
cg14754555	0.3733064	0.1703336	0.818145	0.0138422
cg03312984	0.6934918	0.3320141	1.448525	0.3300789
cg07583088	2.5558058	0.8687525	7.518992	0.0883048
cg02629070	1.7167923	0.6617021	4.454234	0.2665445
cg00587301	1.5764913	0.8138797	3.053676	0.1771936
cg17378273	1635.4324	16.159672	165513.2	0.0016829
cg25159610	0.1115891	1.49E-05	837.6416	0.6300504
cg27318506	64.582113	0.4365328	9554.493	0.102083
cg21850668	3.037E+16	300.10783	3.07E+30	0.0210745
cg25671374	7.51E-07	1.30E-13	4.332258	0.0758347
cg15915835	0.8455318	0.452511	1.579904	0.5988544
cg02654763	8.18E-05	1.86E-07	0.036026	0.0024462
cg10241039	1.1740525	0.4813845	2.863406	0.7242724
cg21581038	1.79E-10	1.51E-20	2.129438	0.0579461
cg07996838	0.0418516	0.0030449	0.575234	0.0176189
cg25674883	0.4017583	0.1985577	0.812911	0.0112127
cg19516404	3.5930493	0.9970629	12.94803	0.050528
cg20635393	1.22E-16	2.53E-41	5.92E+08	0.2064303
cg11799589	0.3615246	0.1671534	0.781917	0.0097379
cg23336266	3.0383429	1.1452621	8.060624	0.0255867
cg03611868	1.6111353	0.7738902	3.354167	0.2023713
cg09101902	0.4919808	0.1354591	1.78685	0.2810818
cg14769556	0.0149918	0.0015125	0.148602	0.0003319
cg13066546	1.2226172	0.5711999	2.616935	0.6046989
cg26056577	3.4374725	0.3627715	32.57206	0.2818442
cg06148736	1.8698598	0.8268951	4.228318	0.1327409
cg03037974	0.2632449	0.0961773	0.720522	0.0093768
cg24204951	0.3447354	0.1596357	0.744461	0.0067037
cg09215152	0.4041086	0.0024096	67.77302	0.7288184
cg03823901	0.4825291	0.2263713	1.02855	0.0591514
cg14495399	2.5147646	0.4675326	13.52642	0.2826974
cg27089200	1.0622556	0.4343674	2.597771	0.8946933
cg13243638	2.4086728	0.9750139	5.950382	0.0567636
cg00988841	0.2560304	0.0774508	0.846364	0.0255226
cg20371573	0.3170658	0.0204996	4.904034	0.4110582

cg02907662	0.2931636	0.0822245	1.045246	0.0585258
cg05976481	1.8940653	0.7886775	4.548733	0.1530374
cg07190752	0.1069719	0.0103897	1.101377	0.060272
cg20627988	2.0209111	0.8453016	4.831508	0.1136385
cg12486710	0.0970362	0.0202076	0.465966	0.0035697
cg05598477	12.224019	0.370253	403.5799	0.1605889
cg15422708	0.1563982	0.0115834	2.111683	0.1623837
cg25935831	0.1677479	0.0021446	13.12121	0.4221851
cg02428402	2.4034293	0.889481	6.494205	0.0838026
cg25670583	0.6555855	0.347855	1.23555	0.1916166
cg15705536	1.1397228	0.3994671	3.251752	0.806845
cg11989407	0.1307223	0.0293233	0.582755	0.0076295
cg06094523	2.3904598	0.5325033	10.73101	0.2553428
cg13628057	2.7112705	0.0187434	392.1903	0.6943209
cg26035689	0.6009364	0.3054354	1.182327	0.1402381
cg10596412	0.453157	0.1525675	1.34597	0.1541455
cg04609090	3.1662045	0.8143628	12.31006	0.0961997
cg03528393	0.2587324	0.0001375	486.7086	0.7252515
cg00754357	0.3121606	0.1388103	0.701995	0.0048672
cg11092486	5.6211018	0.6831172	46.25383	0.1083678
cg12556802	0.4156041	0.2049701	0.842692	0.0149112
cg08969344	0.3021436	0.1216727	0.750298	0.0099082
cg19676227	1.2399681	0.5501339	2.794812	0.6039498
cg06071058	1.5457734	0.7735347	3.088957	0.2175785
cg06358250	3.5914984	1.6420966	7.855117	0.0013643
cg09765014	6.1701022	0.4071899	93.49486	0.1894817
cg27364162	6.5796956	1.90146	22.76798	0.0029339
cg26796341	2.7212369	0.8174501	9.058816	0.1027906
cg08546406	0.2265272	0.0415615	1.234666	0.0861053
cg26352652	0.7325332	0.2739513	1.95876	0.5351065
cg12252297	0.3772969	0.0529819	2.686823	0.3304665
cg25654705	1.8608347	0.7109015	4.870866	0.2058918
cg01812894	1.2029651	0.4988898	2.900691	0.6807101
cg14713118	0.001097	1.18E-05	0.10191	0.0032015
cg05013728	3.6792595	1.5257832	8.872132	0.0037225
cg19481531	1.5283512	0.5433972	4.298618	0.4214102
cg19440720	0.0224746	0.0010895	0.463618	0.0139813
cg09599971	0.5154838	0.2453025	1.083248	0.0803056
cg24838825	5.3996101	1.406571	20.72827	0.0140088
cg02593352	7.81E-35	9.14E-69	0.667399	0.048826
cg24154937	1.9909801	0.8338198	4.754027	0.120971
cg11149194	0.7252869	0.3500254	1.502865	0.387558
cg17168235	15.452842	2.0496683	116.5019	0.007901
cg26789064	2.5245685	0.955364	6.671223	0.0617812
cg26057751	2.1008385	0.7170678	6.154958	0.1758821
cg12526473	0.6952703	0.3377941	1.431051	0.3237254
cg17584288	28263213	2.92E-06	2.74E+20	0.2607744
cg22437221	6.2561837	1.3514239	28.96192	0.0190195
cg08154219	0.6470831	0.3091976	1.354204	0.2479942
cg17053060	0.8206558	0.3945309	1.70703	0.5968558
cg12041387	2.012446	0.9292493	4.358291	0.0760886
cg27526098	2.139777	0.7100646	6.44821	0.1765043
cg06200397	1.3508378	0.6135795	2.973963	0.4551402
cg27635859	0.3920758	0.1206323	1.274314	0.1194975
cg15022400	1.7292501	0.6310244	4.738812	0.2869552
cg05089225	0.1800273	0.0434566	0.745798	0.0180588
cg03481077	3.2375126	0.7611179	13.77118	0.1117386
cg11323585	1.945874	0.6894437	5.492001	0.2085687

cg27506210	0.3376458	0.1398815	0.815009	0.0157379
cg03269363	0.4241395	0.1513321	1.188738	0.1028571
cg12073436	1.3491717	0.5898464	3.085998	0.4780425
cg02892755	1.3800021	0.0629948	30.23115	0.837956
cg07016184	0.0015192	9.96E-08	23.17544	0.1866885
cg21467935	0.3825223	0.1681444	0.870224	0.0219392
cg04649468	57.860406	8.77E-19	3.82E+21	0.8616418
cg00350942	0.1747095	0.048173	0.633621	0.0079508
cg03418002	0.5222781	0.252357	1.080907	0.0800632
cg24759599	0.4457675	0.0210285	9.449481	0.6040839
cg14509196	0.1650836	0.0430714	0.632731	0.0085978
cg16068263	2.4664688	0.8744719	6.956733	0.0879289
cg01945624	3.6101242	0.9630036	13.53369	0.0569037
cg03423916	1.191618	0.5286395	2.686053	0.672468
cg03693911	0.5318533	0.1297525	2.180057	0.3803792
cg25906537	0.4710337	0.2220338	0.999274	0.0497793
cg11746851	0.0966935	0.0163111	0.573207	0.0100868
cg04820773	0.536705	0.2340581	1.230687	0.1416348
cg14841628	0.401066	0.0004708	341.6407	0.7907101
cg03018256	1.3708288	0.3454512	5.43976	0.6537784
cg04670874	0.301586	0.1199523	0.758252	0.0108257
cg21569398	0.4428403	0.1954404	1.003414	0.0509622
cg00475193	0.1574032	0.0291976	0.848555	0.0314751
cg05469118	0.6338529	0.3169622	1.267563	0.1972469
cg26476925	0.2370434	0.0585338	0.959952	0.0436705
cg12904569	3.553821	1.7051487	7.40677	0.0007138
cg24104938	0.3339985	0.0591613	1.885609	0.2143232
cg25822376	0.7571779	0.4006826	1.430854	0.3916546
cg05776004	1.5005312	0.1287685	17.48559	0.7460027
cg07180307	2.2213756	1.0907344	4.524025	0.0278575
cg04208863	0.3274285	0.0671374	1.596866	0.1672716
cg00057900	1.8385765	0.7154261	4.724966	0.2060203
cg08129331	0.3618243	0.1766409	0.741147	0.0054564
cg26409748	0.4118843	0.1475122	1.150065	0.0904393
cg12824782	0.9783268	0.5405367	1.770691	0.9422939
cg17166802	192.67144	4.5336231	8188.216	0.005958
cg07318204	2.0877088	0.8699667	5.009994	0.0993387
cg24150986	2.7312664	0.8724799	8.55013	0.0844065
cg02698900	1.2943778	0.4915368	3.408522	0.6014517
cg07385490	0.0391036	0.0054708	0.279498	0.0012366
cg04595479	0.5435988	0.2405629	1.228367	0.1427964
cg23851558	0.2657209	0.0877682	0.804479	0.0190322
cg12788159	10.761801	0.8200636	141.2285	0.0704611
cg07800670	0.978322	0.1303962	7.340047	0.9829943
cg21530026	0.320483	0.1230926	0.834407	0.0197655
cg27506783	0.1104991	0.0100006	1.220927	0.0723179
cg25001003	0.0263546	0.0018963	0.36628	0.0067701
cg15637765	0.4337123	0.2058765	0.913686	0.0279911
cg16227748	3.0675193	0.7308708	12.87461	0.1256289
cg19476368	0.6013867	0.1770856	2.042323	0.4149544
cg24570064	0.4146896	0.1696838	1.013458	0.0535274
cg10251347	5.077E+09	4.24E-07	6.08E+25	0.2367569
cg02850689	0.0585793	0.0093211	0.368145	0.0024823
cg24517989	0.188023	0.0542531	0.651625	0.0084054
cg27441689	1.692E+14	1.15E-05	2.49E+33	0.1456899
cg01059609	0.1149578	0.0157292	0.840179	0.0330432
cg26668151	0.4202162	0.159235	1.108937	0.0799261
cg24488229	0.1943802	0.0559332	0.675514	0.0099606

cg13689681	0.0067366	1.31E-06	34.71333	0.251554
cg11328127	1.71772	0.492781	5.987573	0.3957922
cg22495801	0.0114643	0.0006142	0.214002	0.0027675
cg25166437	0.9985684	0.2543988	3.91959	0.9983616
cg13069441	0.049952	0.0060773	0.410574	0.0052995
cg07295586	1.7354688	0.8481012	3.551288	0.1313021
cg19857564	1.1337967	0.3901241	3.295092	0.8175542
cg18325834	0.9701169	0.4662653	2.018436	0.9353147
cg04687439	0.3541862	0.0993909	1.262166	0.1094081
cg26268565	2.0929984	0.3215418	13.62386	0.4396419
cg03811411	0.6248877	0.2714652	1.438433	0.2690235
cg25556330	2.9579723	1.0338101	8.46345	0.0431807
cg04516112	0.3253003	0.1180306	0.896549	0.029925
cg12603671	0.8336614	0.4003698	1.735873	0.62685
cg07386061	1.8302847	0.4453477	7.522082	0.4018968
cg26140749	0.143193	0.0245557	0.835009	0.0307423
cg03187444	1.6618993	0.6948955	3.974568	0.2535432
cg23596671	3.76E-29	4.07E-63	347737.6	0.1009617
cg08068240	0.1223563	0.0151897	0.985603	0.0484287
cg21828951	0.0770766	0.0122875	0.483483	0.0062251
cg17242937	1.7466748	0.9521826	3.204084	0.0715964
cg22452230	0.1685885	0.0477674	0.59501	0.0056602
cg22259797	0.36762	0.1934937	0.698444	0.0022432
cg06003986	2.2295129	1.0405432	4.777051	0.0391904
cg08258494	0.3329257	0.1615727	0.686004	0.0028668
cg16167809	3.107681	0.4237397	22.79154	0.2646986
cg23869743	3.2156299	0.7370131	14.02998	0.1201888
cg00243313	0.0359852	0.0023565	0.549509	0.0168274
cg02065717	0.0006448	7.61E-06	0.054661	0.0011825
cg09996325	5.5962095	0.8682662	36.06908	0.0700816
cg13635968	3.23E-05	6.33E-13	1652.306	0.2535748
cg13426079	1.9534412	0.5687569	6.70925	0.2875075
cg05007992	1.8570304	0.7670378	4.495948	0.1700436
cg13949259	0.576915	0.2070073	1.607822	0.2928619
cg14740417	0.3832611	0.1802598	0.814874	0.0127063
cg04759767	2.4720866	0.4605373	13.26974	0.2911417
cg10096948	2.3313259	0.5792681	9.382669	0.2334829
cg12368542	0.006455	0.0002705	0.154032	0.0018352
cg15786280	1.6892	0.8410301	3.392741	0.1406444
cg07552068	3.09E-11	4.63E-25	2066.991	0.1362369
cg08491964	1.1160513	0.5264561	2.365954	0.7745703
cg13131034	3.48E-24	7.94E-45	0.001524	0.0259178
cg16121744	0.0989229	0.0357505	0.273723	8.39E-06
cg05677025	2.7972146	0.7669541	10.20193	0.1192171
cg24720630	3.74E-19	1.18E-34	0.001189	0.0198197
cg17034390	2.907017	1.0247946	8.246285	0.0448557
cg05265596	1.2658604	0.4520536	3.544718	0.6536227
cg11344005	1.3246707	0.4048526	4.3343	0.6420148
cg00061185	0.287261	0.0070497	11.70536	0.5096179
cg02364970	0.3371553	0.1792031	0.634329	0.0007475
cg02854554	2.0034253	0.8362964	4.79939	0.1190215
cg11495377	0.6592889	0.2954348	1.471262	0.3090657
cg22142254	1.9588375	0.5469606	7.015212	0.3016203
cg11122767	0.37107	0.0275154	5.004216	0.4551541
cg04788993	2.7149957	0.3101123	23.76946	0.366909
cg19792316	0.0003828	1.75E-12	83878.99	0.4219993
cg26069615	0.9192076	0.4377237	1.93031	0.8238873
cg26866474	0.2345082	0.0979291	0.56157	0.0011337

cg04506728	1.4247625	0.4943813	4.106038	0.5121331
cg00372774	0.4139094	0.1451514	1.180291	0.0989593
cg24830524	5.8238822	0.6343697	53.46662	0.1193225
cg07205823	1.3912037	0.5541329	3.49275	0.4820595
cg02068351	0.6045239	0.2842603	1.285614	0.1910869
cg09927842	2.76E-11	4.73E-21	0.161308	0.0340928
cg11481720	5.72E-13	3.10E-42	1.06E+17	0.4122776
cg00570269	2.0596849	0.7330978	5.786816	0.1704067
cg08319974	0.2712585	0.0384073	1.915814	0.1908352
cg08866557	0.071045	0.0008282	6.09432	0.2443223
cg08460621	2.3679302	0.9978141	5.619376	0.0505829
cg19455189	3.2113886	0.9940442	10.37481	0.0511785
cg24086869	881.06171	9.20E-13	8.44E+17	0.7000223
cg04557294	3.3122655	0.0224376	488.9599	0.6383791
cg00400104	0.002668	1.84E-05	0.38746	0.019635
cg00666746	0.0607954	0.0161199	0.229287	3.56E-05
cg14032261	0.5209722	0.215442	1.259792	0.1478008
cg07941927	1.805927	0.8565615	3.807517	0.1203924
cg08100159	0.2269967	0.1058751	0.486682	0.0001386
cg02503970	22123801	2.99E-13	1.64E+27	0.46875
cg16306870	3.247457	0.8798656	11.98589	0.0770827
cg06817264	0.0066781	0.0002844	0.156828	0.0018685
cg07512258	17.188355	1.4028584	210.5983	0.0260983
cg21134737	2.4843845	0.8646413	7.138413	0.0910498
cg19009781	5.57E-10	7.96E-28	3.9E+08	0.3094522
cg08065963	3.4775554	0.6327029	19.11385	0.1517222
cg06535102	3.5423768	0.0184893	678.6853	0.6371402
cg07294263	0.6220383	0.2335646	1.656636	0.3421463
cg09710580	8.55E-06	7.62E-10	0.09605	0.0141931
cg00092400	3.0303253	1.0492723	8.751657	0.040477
cg18072147	0.4421081	0.1927785	1.013907	0.053936
cg22143698	0.4895923	0.2237349	1.07136	0.073865
cg09247981	0.0035983	2.62E-05	0.494702	0.0250821
cg01609688	0.6608017	0.2942846	1.483798	0.3154537
cg05960677	1.3218912	0.6456816	2.706282	0.4452508
cg09041756	2.11E-15	1.73E-30	2.57513	0.056573
cg07304015	0.3863157	0.1873249	0.79669	0.0100116
cg18178840	0.2769029	0.0986774	0.777029	0.0147208
cg03651021	4.3079249	0.7313719	25.37453	0.1064843
cg07538190	0.129617	0.0384667	0.436756	0.000979
cg06929414	0.2685884	0.1026222	0.702964	0.0074076
cg07986058	0.0973302	0.0231904	0.408495	0.0014561
cg05969659	0.2836579	0.0815198	0.987021	0.0476475
cg22966302	0.8214769	0.1698952	3.972002	0.8067861
cg00977903	1.9891453	0.0072069	549.0122	0.8104713
cg09097839	1.3797972	0.7495066	2.540125	0.3011684
cg01267709	0.2825122	0.1015129	0.786236	0.0154995
cg07241146	1924.397	4.6245978	800783.9	0.0139854
cg07376547	5.0419714	1.3150943	19.33053	0.0183026
cg21518151	3.346459	1.189302	9.41627	0.0221135
cg06748434	2.0048107	0.7826543	5.13543	0.1472477
cg08578317	1.9229282	0.9176227	4.029601	0.0832361
cg19309463	3.5167809	0.5826846	21.22546	0.1703473
cg12000587	0.1081537	0.0257189	0.454811	0.0024048
cg04066400	2.1002634	0.8726222	5.055001	0.0977388
cg15854333	0.227315	0.0249056	2.07472	0.1891582
cg07341290	1.9524774	0.9473887	4.023869	0.0697576
cg11251012	0.6026972	0.0999529	3.634151	0.5807113

cg19426625	2.3371232	0.6064011	9.007478	0.217473
cg11785425	2.3244542	0.9938076	5.436754	0.0516985
cg14750543	1.5669949	0.646992	3.795214	0.319638
cg18437193	2.1705649	0.8015231	5.877999	0.1273346
cg21501525	3.302347	1.3529248	8.060681	0.008694
cg11042866	249333	56.034881	1.11E+09	0.0037402
cg09915769	0.0178916	0.0003754	0.852699	0.0412717
cg16402452	0.3790423	0.1201787	1.195495	0.0978662
cg19037884	0.7915654	0.3732155	1.678858	0.5423069
cg09677741	1.0946073	0.5737933	2.088147	0.7838459
cg13228596	249.01161	0.0798192	776840.5	0.1789098
cg26767214	0.1213148	0.0118229	1.244807	0.0757939
cg01874152	2.093302	1.0972213	3.993646	0.0249951
cg13500819	3.5667711	0.9346088	13.61196	0.0627448
cg05080966	0.1872555	0.0264414	1.326127	0.0934736
cg26006870	1.6680154	0.8295884	3.353802	0.1510853
cg12288941	1.1997249	0.6780419	2.122789	0.5316891
cg20295353	0.3703279	0.1528988	0.896951	0.0277416
cg01200965	0.4068399	0.0980928	1.687368	0.2152984
cg06870618	21.474134	0.1006823	4580.136	0.2623355
cg01748572	0.0003952	3.36E-08	4.640848	0.1012256
cg12034869	0.1451258	0.0472776	0.445486	0.0007435
cg07942920	0.6570431	0.1118829	3.85855	0.6419282
cg10700718	1.5998626	0.6145298	4.165072	0.335753
cg17894318	3.3214454	1.1351092	9.718888	0.0284297
cg18046394	4.4184481	0.674166	28.95827	0.1213986
cg18968920	0.3549863	0.1349099	0.93407	0.0358931
cg20223728	0.7402408	0.2447548	2.238797	0.5942609
cg09786278	0.5443248	0.2540309	1.166352	0.1177686
cg11870037	8.3068345	2.62E-11	2.64E+12	0.8754978
cg23819016	1.0210173	0.556186	1.87433	0.946494
cg02413187	0.3365308	0.162741	0.695909	0.0033036
cg16345647	2.1700893	0.7997751	5.888265	0.1281929
cg27035251	1.4417921	0.726106	2.862894	0.2958133
cg02911432	0.2034834	0.0611337	0.677295	0.0094579
cg16908257	1.5964275	0.6273792	4.062265	0.3262855
cg10922915	0.1795955	0.0460787	0.699988	0.0133656
cg02665650	0.6866613	0.3512258	1.342452	0.2717709
cg19001809	1.64E+20	4.25E-07	6.34E+46	0.1361551
cg26085328	0.1358582	0.0326045	0.566102	0.0061185
cg20864214	0.5070019	0.2184833	1.176525	0.1137714
cg22759096	0.0927453	0.018785	0.457903	0.0035148
cg00938547	0.6976669	0.3307441	1.471649	0.3444754
cg07762181	2.4640902	0.7435402	8.165988	0.1401539
cg09178261	0.0995328	0.0237259	0.417551	0.0016121
cg18955367	0.3389405	0.1313045	0.874918	0.0253424
cg08862033	0.8293513	0.3125207	2.20089	0.7070952
cg16116203	0.1888677	0.0409109	0.871919	0.0327137
cg21513610	3.6083108	1.1653782	11.17226	0.0260562
cg26257822	1.9270792	0.7916414	4.691056	0.1483949
cg14530834	0.8272689	0.3281713	2.085416	0.687706
cg03790422	2.6181336	0.8619915	7.952078	0.0895145
cg00602811	0.6575496	0.3154069	1.370837	0.2633699
cg27325314	4.06E-21	2.18E-49	75464485	0.157423
cg13844500	0.5785661	0.2471432	1.354432	0.2073472
cg23751110	0.7273783	0.3749097	1.411218	0.3465388
cg20413742	0.2443289	0.0976609	0.611265	0.0025952
cg00033915	0.0008578	1.28E-06	0.575837	0.0334905

cg14382750	2.0811627	0.8390831	5.161871	0.1137841
cg27406823	0.9672727	0.4265549	2.193425	0.9365109
cg11269166	1.3849564	0.5674984	3.379929	0.4743419
cg11980897	7.6241882	1.4915675	38.97125	0.0146756
cg02963613	0.3996166	0.1605711	0.994534	0.0486386
cg06057398	0.5762819	0.063061	5.266347	0.6253729
cg26502461	1.972518	0.0154389	252.0141	0.7836921
cg03512692	0.441679	0.1783412	1.09386	0.077383
cg02468320	1.7231938	0.847835	3.502328	0.1326314
cg08663425	4.7035966	0.6863207	32.2354	0.1148727
cg25902939	0.3283815	0.1494664	0.721463	0.0055557
cg01478155	0.2726361	0.111374	0.667395	0.0044375
cg04499029	5.7382599	1.0674376	30.84735	0.0417489
cg21217911	1.9600177	0.931857	4.122596	0.0760757
cg21127362	6.1633748	0.0076621	4957.805	0.5941765
cg13679817	3.2436596	0.1485671	70.81867	0.4544806
cg02576753	3.4191816	0.7954946	14.69627	0.098444
cg03952484	18.19687	1.08E-09	3.08E+11	0.809212
cg09784072	5.4037685	1.3838139	21.10162	0.0152101
cg13621836	310.25326	0.0011109	86648558	0.3698589
cg00068160	1.1773535	0.6241766	2.220784	0.6140756
cg22102996	0.7342553	0.1064013	5.066956	0.7539554
cg26768532	0.3771598	0.033813	4.206942	0.428126
cg15797101	0.2399808	0.010511	5.479114	0.3712028
cg24907814	1.5135915	0.670205	3.418296	0.3186672
cg25575961	2.3837141	0.847672	6.703174	0.099623
cg07154944	0.6022358	0.2906378	1.247904	0.1725077
cg01045374	2.7010186	0.4125	17.68606	0.3000334
cg05531134	2.24E-31	2.83E-54	1.78E-08	0.0087084
cg06481122	1.4534976	0.7553595	2.796887	0.2627823
cg11111372	0.4385141	0.1975884	0.973208	0.0426898
cg05233899	0.0492277	0.0045899	0.527973	0.0128611
cg21629394	0.0280402	0.0025785	0.304922	0.003331
cg14015502	0.2301153	0.0971463	0.545086	0.0008404
cg19735590	2.3043019	0.9756209	5.44249	0.0569519
cg08179564	1.3019043	0.4249643	3.988465	0.6441785
cg01816936	5.0631442	0.9803473	26.14933	0.0528347
cg12690066	19.504193	0.1301218	2923.52	0.2451701
cg13035218	6.4435851	0.6164494	67.35312	0.1197224
cg06793974	1.1044994	0.4109085	2.968834	0.8438148
cg22439792	0.3743544	0.1583677	0.88491	0.0251868
cg19804859	0.4108476	0.1306956	1.291519	0.1279593
cg04692403	1.4487606	0.5370492	3.908222	0.4640718
cg12636435	0.5187001	0.2053514	1.310193	0.1649885
cg09063936	0.8242524	0.2951194	2.302092	0.7122583
cg04333575	0.2040709	0.0593978	0.701119	0.0116081
cg04139895	2.4671421	1.0430045	5.835823	0.0398002
cg07333545	1.3590028	0.6600628	2.79805	0.4051162
cg12572295	3.6416186	0.6816907	19.45367	0.1305955
cg15130541	1.9979341	0.9757054	4.091133	0.058397
cg25742037	0.3545386	0.0761077	1.651575	0.1865487
cg13514230	0.0038695	1.49E-07	100.256	0.2840376
cg24974365	2.4472172	0.8965593	6.679839	0.0806656
cg16204205	2.3011546	0.5273214	10.04191	0.2675755
cg24659858	4.1225394	0.9296372	18.28168	0.0623282
cg06300308	3.7421031	0.5305064	26.39617	0.1855143
cg18659867	0.5402873	0.1943301	1.502137	0.237977
cg26757472	1.616441	0.6884905	3.795087	0.2701095

cg22335261	0.7721371	0.3589814	1.660798	0.5081277
cg07344338	1.386306	0.5259617	3.653963	0.5088858
cg10539274	1.5493172	0.8436087	2.845376	0.1580602
cg19687985	0.0830942	0.0206157	0.334922	0.0004687
cg13853156	100.95683	0.0020608	4945869	0.4023033
cg12719233	0.4065421	0.1909539	0.865531	0.019568
cg08718459	14.743455	2.0612081	105.4573	0.0073516
cg02081001	0.7786792	0.1413295	4.290268	0.7738746
cg00444341	2.0915176	0.8598286	5.087579	0.1037425
cg01427575	0.0881909	0.0166222	0.467907	0.0043448
cg04918708	2.413744	0.6257338	9.310924	0.2007882
cg09072870	0.245767	0.089458	0.675193	0.0064953
cg15555217	0.1549697	0.0577746	0.415677	0.0002124
cg08489349	0.0858363	0.0143999	0.511663	0.0070253
cg21209859	1.0018676	0.2688978	3.732789	0.9977816
cg01377523	0.1973205	0.0765268	0.508781	0.0007844
cg07664454	2.05E-07	3.26E-12	0.012843	0.0062836
cg06773295	2.2634727	0.6514578	7.864376	0.1985957
cg04457317	2.7681584	0.996828	7.687084	0.0507169
cg21759430	0.0104061	0.0005575	0.19425	0.0022334
cg16059978	1.519616	0.7234536	3.191957	0.2691263
cg15493618	2.2414237	0.8146478	6.167058	0.1180573
cg16396948	0.6163108	0.2497467	1.520897	0.2936364
cg26382679	0.2077874	0.0921883	0.468342	0.000151
cg13710556	1.674939	0.6606007	4.246772	0.2772372
cg11419235	35.632658	0.4269899	2973.575	0.1134284
cg17037282	0.2268122	0.0500191	1.028483	0.0544108
cg14256102	0.0804654	0.0037619	1.721103	0.1068494
cg19034038	1.4997288	0.7990809	2.814717	0.207054
cg11029191	0.3931667	0.15932	0.970249	0.0428164
cg10773982	0.9486148	0.0420319	21.40921	0.9735349
cg02047803	4.0829222	0.404435	41.21863	0.2330397
cg07316978	0.0002129	4.62E-07	0.09808	0.0068909
cg00564465	1.362166	0.6424091	2.88834	0.4202555
cg05531309	0.1550453	0.0532051	0.451819	0.0006359
cg05271336	0.1531613	0.0151035	1.553171	0.1124125
cg00334177	1.5115816	0.664577	3.438095	0.3244225
cg24499516	1.4174794	0.5105344	3.935578	0.5031052
cg20341998	2.6382139	0.8129121	8.562024	0.1062865
cg20973735	1.3637765	0.5150433	3.611127	0.5323131
cg03501539	0.8689405	0.4569381	1.652429	0.6683682
cg02205193	1.7297063	0.841229	3.556563	0.1362573
cg12968732	0.3834881	0.1623654	0.905754	0.0288375
cg01675596	0.4686978	0.153639	1.42983	0.1829765
cg12962308	0.7741282	0.2831069	2.116778	0.6178949
cg07594804	4.7501426	0.1762864	127.9954	0.3538327
cg23791611	1.5417337	0.8283568	2.869467	0.1719904
cg03965138	0.2078697	0.0594478	0.726853	0.0139142
cg24807850	0.3509547	0.1238345	0.994627	0.048827
cg08155249	1.1730256	0.162168	8.484962	0.874398
cg08855903	0.3491154	0.0578791	2.105794	0.2510685
cg25219329	1.9671532	0.9419293	4.108261	0.071744
cg26996890	0.3664634	0.1457787	0.921228	0.0328088
cg13753946	2.1542516	0.7273573	6.380358	0.1659526
cg26673070	0.1255119	0.0237265	0.663951	0.014613
cg19543867	0.4844953	0.220434	1.06488	0.0713078
cg23817893	2.1607254	0.8303818	5.622394	0.1143305
cg04196298	0.1821368	0.0469602	0.706423	0.0137972

cg11251470	3.1789194	0.6742624	14.98753	0.1437961
cg16913064	1.7541158	0.683595	4.50109	0.2424812
cg22387781	2.2833604	0.7354235	7.089431	0.1531958
cg18959827	0.7261428	0.3683097	1.43163	0.3555068
cg20371046	0.228654	0.0361941	1.444507	0.116666
cg11435872	0.2087282	0.0719231	0.60575	0.00395
cg16833340	0.5043235	0.2015901	1.26168	0.1434302
cg14001518	5.0557705	0.0001118	228625.5	0.7669975
cg20247380	7.34E-05	5.28E-14	101941	0.3754561
cg12379383	2.2465831	0.5856117	8.61857	0.2380313
cg12732864	0.2578678	0.0169434	3.924588	0.3292233
cg02586830	1.9334408	0.0027695	1349.791	0.8435681
cg01594538	1.0007775	0.554111	1.8075	0.9979441
cg12312338	0.5405944	0.2318741	1.260349	0.1543894
cg27452939	0.2639442	0.099135	0.702744	0.0076757
cg06036459	0.2635359	0.0791651	0.877295	0.0297569
cg14733637	1.9778086	0.138652	28.21255	0.6150138
cg23973538	1.53E-06	1.16E-11	0.200264	0.02592
cg01430302	0.2012259	0.0697687	0.580373	0.0030101
cg23954908	1.0272541	0.2850011	3.70262	0.9672127
cg22522688	0.4171132	0.1625522	1.070323	0.0689701
cg23089272	1.4921244	0.4707568	4.729481	0.4965476
cg15696634	3.4395148	1.612029	7.33874	0.0013989
cg16558659	3.5257486	0.6090644	20.40983	0.1595708
cg23423163	1.9017159	0.6826339	5.297896	0.2188509
cg19978859	2.9007784	0.0084079	1000.788	0.7209421
cg11584690	1.660415	0.4556366	6.050826	0.4421599
cg11379350	4.73E-08	4.65E-24	4.82E+08	0.3698238
cg04338863	0.5579537	0.2357358	1.320599	0.1843926
cg04254159	0.2064073	0.0673628	0.632455	0.005747
cg15651727	0.2148741	0.0613227	0.752917	0.0162359
cg26364091	3.3460379	0.3884473	28.82236	0.2716383
cg26682580	0.18976	0.0331293	1.08692	0.0619901
cg09648933	3.9944121	1.0212863	15.62278	0.0465652
cg17700835	2.567921	1.0111891	6.521251	0.0473267
cg12472351	0.3114904	0.1531849	0.633394	0.0012771
cg01786715	0.5953458	0.1629258	2.175448	0.4328056
cg26287152	0.291817	0.1212755	0.70218	0.0059745
cg21543434	2.0596239	0.6964569	6.090902	0.1915339
cg22535729	0.3397765	0.1459561	0.790978	0.0122847
cg25237894	2.1823769	0.5888301	8.088528	0.2429709
cg19639560	1.201709	0.4430392	3.259541	0.7181653
cg13896204	2.7070749	0.881265	8.315608	0.0819961
cg27312458	1.6651781	0.6988369	3.967761	0.2496991
cg02301079	0.042553	0.0066806	0.271046	0.0008321
cg08204783	2.3179428	0.9287158	5.785256	0.0716255
cg17801546	0.2720586	0.0994807	0.744023	0.0112125
cg10416206	0.0173137	0.0001106	2.710171	0.1156581
cg00427296	0.1691942	0.0044641	6.412588	0.3380653
cg10909185	1.9280436	1.0873065	3.418863	0.0246801
cg00289125	107743377	100.02507	1.16E+14	0.0090589
cg00207352	0.3383626	0.0957449	1.195773	0.0924948
cg04515533	1.9031156	0.810007	4.471379	0.1398133
cg02938320	3.8269836	0.7164289	20.44279	0.1164423
cg03235923	0.1582403	0.034892	0.717643	0.0168445
cg04268643	0.3569938	0.1256644	1.014166	0.0531674
cg15185804	9.30E-08	1.09E-14	0.792949	0.0467612
cg13605988	1.9382702	0.9423064	3.986911	0.0721021

cg00290033	0.4170708	0.024024	7.240605	0.5481643
cg27271738	0.5594512	0.2813615	1.112397	0.0976776
cg06821460	1.6081774	0.0920761	28.08802	0.7447569
cg00792966	8.875885	2.77E-16	2.85E+17	0.9103533
cg07258998	1.2654369	0.5812377	2.755036	0.5531398
cg10407488	0.4189325	0.1893037	0.927105	0.0318161
cg04917276	2.2781317	1.0327093	5.025503	0.0413805
cg17993073	8.72E-11	6.44E-19	0.011804	0.0153223
cg24406162	0.3580853	0.1340364	0.956644	0.0405236
cg07273992	0.3644551	0.1059519	1.253659	0.1093072
cg15973098	1.9377134	0.5145133	7.297641	0.3281998
cg07478100	10.853599	5.56E-08	2.12E+09	0.8066003
cg27381418	9.7085804	2.2678465	41.56213	0.0021869
cg18179039	1.8238374	0.8225271	4.044101	0.1391155
cg03736795	1.4685654	0.7731849	2.789351	0.2403709
cg02903822	0.1925852	0.0690193	0.537372	0.001654
cg26070379	0.1249302	0.0308994	0.505109	0.003521
cg26325723	1.1428481	0.5725698	2.281123	0.7049483
cg04552206	1.4422818	0.5586439	3.723619	0.4491767
cg03206179	1761.5892	25.970192	119490.7	0.0005133
cg01292793	1.1116103	0.5613377	2.201309	0.7614875
cg08769189	0.660234	0.2760524	1.579081	0.3507484
cg04938549	1.5258777	0.8495256	2.740709	0.1573034
cg27231912	1.6283579	0.6831505	3.881355	0.2712567
cg22927302	2.5296815	0.6875435	9.307467	0.1626157
cg05123976	1.6676907	0.6041294	4.603637	0.3235474
cg26815454	1.9791009	0.8830167	4.435749	0.0973525
cg13641778	0.1288088	0.0371357	0.446785	0.0012397
cg09305096	0.3602726	0.1684155	0.770691	0.0085058
cg06023487	4.84E-30	4.29E-80	5.46E+20	0.2510006
cg21075077	0.1934916	0.0256476	1.459747	0.1111411
cg01337391	4.012296	1.0974411	14.66914	0.0356814
cg11291773	3.2299617	0.3950026	26.41161	0.2741343
cg00141688	10.919182	0.0248667	4794.706	0.4412921
cg00612202	0.7813724	0.0010821	564.1996	0.9414391
cg01462744	38973058	4.51E-20	3.37E+34	0.5807293
cg20404150	0.3993812	0.1917417	0.831876	0.0142207
cg09479241	0.0263571	0.0019749	0.35176	0.0059551
cg02618579	6.01E-16	5.73E-32	6.314386	0.0625954
cg15935770	1.6738951	0.4862702	5.762074	0.414043
cg16123366	8.6593756	0.7915584	94.73058	0.0769835
cg06949933	0.6410076	0.2286343	1.797153	0.3978412
cg12788684	0.6236113	0.2498606	1.556432	0.3115641
cg03127886	2.1188469	0.9440303	4.755686	0.0687085
cg02223905	1.9225	0.6981397	5.294079	0.2059822
cg14314896	2.655965	0.6278382	11.23562	0.1843707
cg04996251	22.128691	0.8476985	577.657	0.0627881
cg25215295	0.6177955	0.2676942	1.425773	0.2590401
cg17804635	2.43E-09	1.03E-18	5.749939	0.0716835
cg03446636	3.1652202	0.499744	20.0475	0.2211658
cg26034150	2.10E+21	0.0478893	9.20E+43	0.0649329
cg01649623	3.1788331	0.8295054	12.18193	0.0915543
cg27103348	0.1454683	0.0525313	0.402827	0.0002076
cg26606256	2.6198689	1.3027656	5.268571	0.006893
cg23501567	1.666502	0.3608566	7.696213	0.5129496
cg18517222	2.4001397	0.4436764	12.98395	0.3094028
cg09643139	0.5965191	0.2934485	1.212598	0.1534673
cg09174855	61497.392	1.7763436	2.13E+09	0.0386679

cg22470426	14641.237	38.030824	5636633	0.0015895
cg07638500	0.1328198	0.0257812	0.684262	0.0157965
cg17658568	0.6486656	0.2455183	1.713791	0.3825581
cg09886009	1.1163698	0.3080905	4.04518	0.8669093
cg21632964	0.2099658	0.0564701	0.78069	0.0198343
cg11196544	3.2929433	1.1518262	9.414159	0.0261685
cg26672452	0.2070807	0.0730429	0.587086	0.0030597
cg12871285	0.3111129	0.101144	0.956964	0.0416805
cg19626303	0.1385076	0.0003604	53.22499	0.5150268
cg09414264	2.2967021	0.1153132	45.74358	0.585926
cg08470031	0.4680729	0.2128396	1.029377	0.0590319
cg17444090	2.1210327	0.5728244	7.85368	0.260269
cg09027601	1.6067621	0.8637658	2.988871	0.134265
cg14189808	1.2680419	0.4419615	3.638168	0.6587848
cg24041541	2.444086	1.1005737	5.427675	0.0281362
cg04330057	0.1704777	0.0655979	0.443042	0.0002827
cg11201772	2.351E+12	1489.2466	3.71E+21	0.0083876
cg01204844	0.2860369	0.0819525	0.998348	0.0496978
cg14851544	0.9392874	0.4468352	1.974466	0.8687572
cg05581469	0.3236258	0.1332924	0.785743	0.0126761
cg17088185	1.0769988	0.4181111	2.774206	0.8778813
cg16819272	0.428293	0.1895031	0.967978	0.0415306
cg12038696	2.4946078	0.9855161	6.314527	0.0537093
cg19791727	8.1310513	0.7907697	83.60714	0.0779791
cg18485596	0.0063306	0.0001216	0.329553	0.0120587
cg19717595	1.5348692	0.8177971	2.880694	0.182272
cg10897045	0.0062432	7.53E-05	0.517924	0.0243337
cg13792714	0.2970423	0.09872	0.893781	0.0307913
cg12041266	0.4824867	0.2206996	1.054798	0.0678083
cg18863119	0.5976459	0.2879215	1.240549	0.1671333
cg19394047	0.5244152	0.2584483	1.064086	0.0737912
cg22436229	0.005645	6.49E-05	0.490646	0.023055
cg13167730	1.9227163	0.7164453	5.159972	0.194312
cg05044706	0.3412182	0.122149	0.953179	0.0402227
cg08511772	0.1598318	0.0510161	0.500748	0.0016494
cg17786642	1.5489139	0.7668741	3.128459	0.2224938
cg16727538	5.29E-11	3.48E-23	80.51246	0.0982623
cg04058799	2.5195306	0.7557234	8.399944	0.1325592
cg06929449	0.0704402	0.0147162	0.337169	0.0008976
cg15992932	0.2408071	0.0836087	0.693565	0.0083417
cg01629329	11457.249	5.5401411	23694083	0.0164179
cg04129469	1.5845681	0.813138	3.08786	0.1762862
cg14698025	3.6026118	0.704954	18.41086	0.1235855
cg16733866	0.0124083	0.0002061	0.746966	0.0357724
cg07995570	2.337122	1.0326255	5.289565	0.0416501
cg15708696	1.3870435	0.7254679	2.651929	0.322462
cg23170988	0.1120917	0.0356241	0.352698	0.0001827
cg18315680	2.2995236	0.6733081	7.853476	0.1839243
cg18284427	2.2468945	0.8083941	6.245141	0.1206277
cg11394588	0.1315352	0.0009206	18.79419	0.4229958
cg08155312	0.2880059	0.0834031	0.994536	0.0489957
cg11775521	0.4390576	0.2211865	0.871534	0.0186211
cg11402363	1.9740732	0.931338	4.184265	0.0760007
cg14151290	1.17E+28	1.981E+10	6.87E+45	0.0019636
cg08781728	1.7351817	0.41958	7.175879	0.4467268
cg04001941	2.5299183	0.4538645	14.1022	0.2896796
cg12628196	0.5663553	0.297214	1.079217	0.0839481
cg04186487	2.5007377	0.5332087	11.72841	0.2450548

cg03116016	0.7299117	0.2236299	2.382379	0.6019243
cg09567013	1.8387982	0.918639	3.68064	0.0853795
cg21468929	2.1334478	0.9714702	4.685269	0.059046
cg08270630	0.1098014	0.0273104	0.441456	0.0018598
cg10494981	0.3239717	0.1563124	0.671461	0.0024365
cg10124773	0.0009317	1.36E-29	6.39E+22	0.8181572
cg25954235	2.7338362	1.2098379	6.177572	0.0156089
cg11830605	1.7957202	0.7897033	4.08332	0.1625111
cg05596199	0.5498495	0.2468065	1.224986	0.1433466
cg05099708	2.3075643	0.8858897	6.01074	0.0869131
cg17761815	2.5115356	0.9447361	6.676797	0.0648916
cg22875527	11.371795	2.0648369	62.62854	0.0052236
cg04600795	0.534319	0.2426102	1.176772	0.1197347
cg13426876	0.2091463	0.0571342	0.765604	0.018109
cg19761211	0.1526818	0.0291012	0.801058	0.0262663
cg25216580	0.1011209	0.0224711	0.455048	0.0028269
cg26435670	0.0216811	2.05E-05	22.98434	0.2810495
cg00230271	5.44E-18	1.87E-38	1584.977	0.0982331
cg21250356	2.4280391	1.1436575	5.154842	0.0209203
cg05900530	1.5427893	0.3889758	6.119143	0.5373767
cg24513276	0.2584491	0.1038874	0.642965	0.0036169
cg02282041	0.8129077	0.4061514	1.627026	0.5584934
cg14038339	1.4382286	0.1032824	20.02763	0.7868168
cg12217971	0.2524768	0.0857642	0.743253	0.012469
cg21202452	2.2642376	1.1152078	4.597145	0.0237136
cg21626848	0.230019	0.0844231	0.626709	0.0040572
cg27312709	2.0484406	0.7069549	5.935469	0.1864763
cg08190298	2.6363454	1.1445035	6.072779	0.0227865
cg10877084	1.0818413	0.6408543	1.826282	0.7684135
cg24852442	0.198499	0.0740947	0.531777	0.0012998
cg20774166	0.1254133	0.0130978	1.200849	0.0716752
cg08357787	2.7296099	1.2337304	6.039221	0.0131984
cg13308350	0.4352226	0.0536122	3.533125	0.4362046
cg05249026	1.3400139	0.5706913	3.146425	0.5015599
cg11426590	2.37E-23	7.12E-48	78.83228	0.0705494
cg14698297	7.45E+23	3.63E-21	1.53E+68	0.2910154
cg01787382	0.6709589	0.3390543	1.327769	0.2518443
cg15222091	2.7071649	1.0501123	6.979008	0.0392874
cg23064516	0.1518672	0.0141887	1.625499	0.1191631
cg22681784	2.2356719	0.8053086	6.2066	0.122508
cg03934069	0.1738577	0.0329427	0.917548	0.0392695
cg13496270	1.5596844	0.6602428	3.684426	0.3108572
cg16966520	0.3887279	0.1134125	1.332388	0.132744
cg03528287	0.7261775	0.3448854	1.529011	0.3996576
cg04236664	0.1366437	0.0276566	0.675119	0.0146075
cg12151942	0.5456387	0.2423969	1.22824	0.1433685
cg00403955	32.558978	2.3692305	447.4394	0.0091845
cg15146462	4.9196419	0.8382053	28.87464	0.0776479
cg25433316	0.1345933	0.0496888	0.364576	7.99E-05
cg07529654	0.707892	0.2797527	1.791264	0.4658017
cg10635443	3.5461796	1.7122407	7.344405	0.000655
cg03532673	0.4752172	0.1980785	1.14011	0.0956566
cg19529471	1.0733555	0.5364595	2.147584	0.8414416
cg22291359	0.1005161	0.0212734	0.474935	0.0037347
cg04951051	8.3472111	0.1084314	642.5805	0.3383221
cg25042258	0.2768679	0.0759841	1.008841	0.05158
cg10063260	4.08E-07	4.83E-17	3447.705	0.207128
cg26230076	0.5868239	0.0527126	6.532827	0.664639

cg26891598	0.1916639	0.0110011	3.339228	0.2572067
cg15044573	0.4074698	0.1781133	0.932169	0.0334763
cg13260133	0.0790961	0.0096174	0.65051	0.018278
cg10775050	6190609.3	0.1510294	2.54E+14	0.0803594
cg02451122	2.10E-31	2.78E-52	1.59E-10	0.0039777
cg01215339	0.0174615	9.67E-06	31.52898	0.2900634
cg02470008	0.5489135	0.3072371	0.980695	0.0427852
cg20733500	35.431343	1.1656192	1077.007	0.0405662
cg01697487	1.2428526	0.5649603	2.734144	0.5888705
cg20426108	1.5191101	0.5013053	4.603373	0.4597941
cg10848640	2.0452653	0.8783009	4.76273	0.0971
cg17428519	0.1913114	0.068186	0.536768	0.001678
cg05737153	2.005036	0.7793195	5.158564	0.1490673
cg13430439	0.0521275	9.07E-05	29.97382	0.3622117
cg26950369	0.4081111	0.1916553	0.869033	0.0201272
cg03961551	2.5328274	0.9269996	6.920408	0.0699624
cg15062189	15210.865	29.442607	7858354	0.0025183
cg19568844	0.4204803	0.1624497	1.08836	0.0741859
cg10522845	1.129997	0.3164656	4.034856	0.8507172
cg05289698	0.5176071	0.2395032	1.118637	0.0939664
cg08726298	1.1449029	0.6313285	2.076261	0.6559115
cg00806535	0.5160709	0.2136232	1.246724	0.141576
cg02230593	1.3072403	0.7207037	2.371123	0.3778429
cg09253696	0.075766	0.007569	0.758421	0.0281469
cg09930017	213968.97	0.4395895	1.04E+11	0.0662172
cg19399220	0.2447234	0.0674651	0.887711	0.0322627
cg13794993	0.6253378	0.1994017	1.961104	0.4207996
cg09473613	2.5339548	0.7067132	9.085619	0.1535381
cg24913868	2.5784671	1.2359761	5.379143	0.011581
cg01297808	2.3403724	0.969249	5.651121	0.0586883
cg16224163	0.361449	0.1663586	0.785324	0.0101597
cg17951588	2.0270873	0.8138005	5.04925	0.1291462
cg07002832	0.4573009	0.1761998	1.186857	0.1078543
cg01585703	220.5584	0.0006761	71954638	0.404799
cg10714061	0.1817931	0.0591411	0.558811	0.0029234
cg25132772	9.5854029	1.2665201	72.5452	0.0286139
cg25665590	5.779E+13	6.67E-09	5.01E+35	0.2188763
cg17897626	0.5048345	0.2418582	1.053749	0.0686796
cg03997321	23.863139	2.3614727	241.1417	0.0071864
cg01943221	4.4358014	0.801049	24.56321	0.0880206
cg13895650	0.1218559	0.033865	0.438472	0.0012732
cg06913865	0.5127249	0.198145	1.326739	0.1684739
cg10727416	0.0694513	0.0073474	0.656485	0.0199558
cg08993878	0.6592358	0.3320942	1.30864	0.2336295
cg11479156	0.2986251	0.0899632	0.99126	0.0483472
cg23975646	0.4465972	0.2143307	0.930567	0.0313908
cg11410436	1.7443786	0.9379637	3.244109	0.078807
cg08687105	1.1541475	0.3363319	3.960541	0.819737
cg15165122	0.5332489	0.2823924	1.006948	0.0525481
cg13290001	0.9596	0.2772096	3.321791	0.9481014
cg23894086	1.7842654	0.82773	3.846185	0.1395411
cg24935154	0.3198207	0.1274185	0.80275	0.0151869
cg12005186	1.3163486	0.5382334	3.219372	0.5469244
cg09163021	0.3747263	0.1215847	1.154913	0.0874185
cg11688093	1.7548237	0.5990074	5.140848	0.3051434
cg05657656	0.3047067	0.0804204	1.154509	0.080366
cg20459022	1.7465308	0.6194289	4.924488	0.2917183
cg17555825	3.2633946	1.1268835	9.450616	0.0292462

cg26643142	0.9028513	0.5025914	1.621875	0.7323941
cg20571513	0.1864326	0.0546363	0.636154	0.0073127
cg05524246	0.1178958	0.0110203	1.261258	0.0770581
cg03599357	2.1763154	0.7353644	6.440819	0.1601099
cg24808442	1.1706833	0.5101913	2.686246	0.7099835
cg01820754	0.0027222	1.38E-08	537.0983	0.3423913
cg04292442	0.4025729	0.1802313	0.899205	0.0264813
cg08147001	0.9139135	0.3248189	2.571396	0.8645729
cg14230938	0.0006804	2.48E-07	1.868168	0.0710336
cg14742148	0.0008441	2.45E-05	0.029114	8.94E-05
cg12935178	2.5741019	0.6409927	10.33709	0.1825412
cg25408008	0.5274373	0.1862892	1.493324	0.2282926
cg16010370	440.28115	13.577315	14277.31	0.0006048
cg02922094	2.3353986	0.15013	36.32909	0.5446893
cg05343088	1.9997542	0.883844	4.524573	0.0961986
cg04242559	0.3949132	0.0906512	1.720402	0.2159473
cg05366156	0.0222071	0.0009568	0.515442	0.0176429
cg13881637	1.4272427	0.5611484	3.630094	0.4551211
cg00422461	2.0854979	0.9226079	4.714138	0.0773317
cg14375046	3.5994984	0.0063282	2047.417	0.6923063
cg25510164	0.7085818	0.3732371	1.345226	0.2922266
cg00896370	1.6843569	0.6652708	4.264516	0.2713067
cg22549545	3.4328593	1.2096815	9.74184	0.0204676
cg06638568	1.4220296	0.5034847	4.016345	0.5062888
cg03428864	1.2282026	0.5970174	2.526696	0.5765086
cg05705492	15.62918	0.8244674	296.2776	0.0670435
cg13640200	1.543289	0.7445887	3.198733	0.2432635
cg20699463	36435.857	0.0004728	2.81E+12	0.2569679
cg10607939	25.7951	2.77E-18	2.40E+20	0.8840433
cg09679862	5.6834563	0.4441641	72.72465	0.1815584
cg02423014	0.356335	0.145848	0.870596	0.0235734
cg23479458	0.5936483	0.1879868	1.874697	0.3741039
cg04703197	9.0289985	1.4145632	57.63109	0.0199822
cg27287944	0.5496121	0.2551976	1.183685	0.1262284
cg11010395	0.2595012	0.106493	0.63235	0.0029927
cg04554690	3.0888853	1.0203253	9.351147	0.045981
cg22145559	0.4412305	0.1943656	1.00164	0.0504597
cg25054705	0.1027005	0.0332481	0.317233	7.65E-05
cg20987938	2.8021321	1.2391307	6.336655	0.0133245
cg24866923	2.4543631	1.2906875	4.667201	0.0061786
cg08781655	0.5550784	0.2252484	1.367876	0.2008237
cg21935742	2.7345038	0.9386754	7.966024	0.0651892
cg16900671	7.1448676	1.4755433	34.59684	0.0145516
cg11597131	1.0512766	0.5166876	2.138976	0.8902582
cg03327221	0.2134548	0.0822096	0.554229	0.0015125
cg19863411	0.2888026	0.070233	1.187576	0.0851313
cg06783197	1.9596193	0.6697567	5.733586	0.2193783
cg13891189	0.1251947	0.0249558	0.628057	0.0115625
cg02909329	3.2851823	1.7086383	6.316388	0.0003624
cg01206912	1.3156097	0.3571935	4.845634	0.6800797
cg02643433	1.5992033	0.6437491	3.972745	0.3118835
cg03863514	0.6608887	6.28E-09	69517100	0.9649466
cg22935319	0.118721	0.0131684	1.070344	0.0575155
cg22022881	2.5751113	1.0123967	6.55	0.0470522
cg08696165	3.3427005	1.5787596	7.077484	0.0016156
cg04509074	0.9150836	0.4492712	1.863859	0.8068519
cg01484915	1.1472098	0.3543062	3.714556	0.8187968
cg24856264	2.0571177	0.8888627	4.76084	0.0920302

cg23639374	0.2367662	0.0624567	0.897553	0.0340963
cg07762761	51.48361	1.4480119	1830.484	0.0305303
cg19683821	2.9765473	1.3962004	6.345675	0.0047416
cg02330706	0.2472896	0.0834405	0.732883	0.0117153
cg00014118	1.5102991	0.628793	3.62759	0.3564121
cg15694987	0.3451292	0.1401059	0.850172	0.0207309
cg16006732	0.4360412	0.1301979	1.46033	0.1783235
cg17237460	175.53838	4.6859458	6575.775	0.0051824
cg05353571	0.4722342	0.2182	1.022022	0.0568234
cg16368763	2.3339105	0.6772618	8.042884	0.1793921
cg05165940	2.9302603	1.1114767	7.725241	0.0297314
cg11784830	635.67858	0.0139458	28975563	0.2382678
cg07408989	0.7036779	0.322289	1.536393	0.3777285
cg09354331	2.063291	0.2091472	20.3549	0.5351386
cg03899215	0.3912292	0.1390469	1.100781	0.0753972
cg24696151	0.3556557	0.1700952	0.743648	0.0060144
cg16413445	6.16E-08	4.09E-16	9.267322	0.083951
cg11960719	0.4831281	0.0654716	3.565102	0.4756066
cg22921096	2.7250782	0.4788274	15.50883	0.2585035
cg13737221	0.3442783	0.1591878	0.744577	0.006741
cg12424548	9.7869316	0.4151265	230.7346	0.1571557
cg08155625	1.7570791	0.6164389	5.008326	0.2915647
cg16119522	3.1328458	1.1530383	8.512053	0.0251434
cg02216380	0.0871603	0.003807	1.9955	0.1266468
cg13277385	7.609E+17	0.0001321	4.38E+39	0.1072721
cg18610205	3.1817732	0.9460141	10.70141	0.0614441
cg11237792	0.3008182	0.1019896	0.887263	0.0295024
cg05869750	0.1619722	0.0338984	0.773931	0.0225422
cg12749890	0.6103483	0.2341107	1.591235	0.3125623
cg16874805	0.5683351	0.1669963	1.934204	0.365865
cg20207911	0.4195336	0.1641142	1.072475	0.0697005
cg21376733	4.5624804	1.564863	13.30227	0.0054332
cg19033615	2.11E-08	2.34E-15	0.189841	0.0305163
cg18050233	0.4250622	0.2020616	0.894172	0.0241478
cg21912060	1.4925589	0.6414689	3.47286	0.3526302
cg13720362	6.6374013	1.4724581	29.91942	0.0137548
cg08583780	3.9405474	0.2058064	75.44914	0.362592
cg10264188	1.4856841	0.4558405	4.84217	0.5113638
cg02956542	0.2281149	0.0630263	0.82563	0.0243272
cg03087334	3.875E+09	0.0005724	2.62E+22	0.1430081
cg01513978	0.2112423	0.0524024	0.851551	0.0288234
cg15194163	9.2294358	1.1947337	71.2983	0.0331277
cg18145414	1.4390998	0.638833	3.241862	0.3796688
cg09859764	2.8595177	0.5704641	14.33366	0.2014333
cg00391696	9.398E+09	1.32E-08	6.71E+27	0.2735886
cg16510128	0.6071049	0.308395	1.195144	0.1487071
cg13452214	0.3401347	0.1454551	0.795376	0.0128395
cg09234582	0.4794719	0.2632677	0.87323	0.0162553
cg13169132	1.3234228	0.5473793	3.199697	0.5338669
cg08562099	0.3258049	0.1396947	0.759863	0.009444
cg07310525	401152599	0.0724069	2.22E+18	0.0835227
cg16311536	1.607902	0.8014989	3.225642	0.1812113
cg24961406	0.0417868	0.0071725	0.243449	0.0004136
cg10361585	6.1879737	0.613929	62.37043	0.1220797
cg26735598	1.1237025	0.5747296	2.197046	0.7331559
cg08148891	1.4884487	0.633455	3.497453	0.3615082
cg24317955	0.0850273	0.0187994	0.384567	0.0013692
cg17407166	0.5302122	0.224357	1.253026	0.1481976

cg01733783	0.710127	6.24E-08	8079371	0.9670611
cg01219347	3.2502135	1.2797366	8.254736	0.0131887
cg21672450	1.2136115	0.5545646	2.655872	0.6280282
cg17811545	1.159054	0.5665225	2.37132	0.6861116
cg05958050	2.682086	0.9947042	7.231884	0.0512391
cg14184780	2.0736572	0.267161	16.09536	0.4854595
cg21806238	3.7853908	0.393974	36.37088	0.2488734
cg20454518	1.7566525	0.5557876	5.552171	0.3372672
cg16721418	0.5651585	0.2003779	1.594009	0.2807445
cg01898246	1.12E-21	5.27E-37	2.38E-06	0.0073851
cg20956366	3.27E-08	9.64E-14	0.011112	0.0079905
cg14584215	1.2579192	0.6356945	2.489184	0.5099275
cg20764656	1.56186	0.2851644	8.554386	0.6073291
cg25487008	1.0620004	0.2732559	4.127431	0.9307899
cg18765542	1.6154609	0.6503311	4.012901	0.3015432
cg13728003	2.0033077	1.0413329	3.853947	0.0374076
cg02065637	0.0637172	0.0055708	0.728774	0.0267991
cg16821992	1.9855959	1.0882934	3.622728	0.025368
cg04002885	3.002E+18	15220840	5.92E+29	0.0013446
cg00490052	3.593733	0.9779527	13.20608	0.0540556
cg03392837	1.4664908	0.396155	5.428672	0.5664062
cg19758134	0.2727351	0.0940387	0.790998	0.0167782
cg14029393	25.269201	0.0004018	1589046	0.5667205
cg13641470	8.011E+10	7.75E-19	8.28E+39	0.4613868
cg09031522	1.7256304	0.8245286	3.611518	0.1476392
cg18112235	0.4955614	0.2411335	1.018444	0.0561029
cg27635422	4.93E-05	3.76E-12	647.8505	0.2356832
cg26367197	1.5612504	0.7378263	3.303627	0.2440559
cg13767625	0.1219477	0.0217796	0.682807	0.0166623
cg26478036	2.8852469	0.5018718	16.5872	0.2350674
cg20229493	2.0610593	0.0535915	79.26569	0.6977228
cg26635363	1138.8355	0.0013385	9.69E+08	0.312382
cg26048025	2.0061751	0.3446151	11.67894	0.4385481
cg24718382	6.7160629	1.306916	34.51293	0.0225795
cg19500766	2.9386009	0.8095484	10.6669	0.101263
cg10077746	0.0605356	0.0052838	0.693547	0.0241913
cg19001871	0.2984933	0.0473584	1.88136	0.1980486
cg17256260	3.02E-19	2.10E-37	0.43595	0.0456212
cg01987417	1.2031739	0.5617095	2.577181	0.6341346
cg22185100	0.9638496	0.4454985	2.085318	0.9254981
cg13982601	0.354015	0.1743217	0.718938	0.0040674
cg08304059	0.3610284	0.1467581	0.888138	0.0265375
cg03817621	0.8580021	0.3676742	2.002228	0.723177
cg15873112	0.8797636	0.1815754	4.262603	0.8735808
cg03462322	0.7619425	0.3281562	1.769147	0.5270006
cg10660854	0.4025495	0.1531529	1.058067	0.0649667
cg04094640	2.7710692	0.6263842	12.25897	0.1791443
cg25647845	3.01E-11	2.09E-24	433.8432	0.1170841
cg16452224	0.2504189	0.086658	0.723645	0.0105464
cg01951459	0.4738361	0.16328	1.375065	0.1694321
cg02115302	3.5741628	0.7374922	17.32173	0.1136921
cg04059197	0.2056889	0.0266646	1.586671	0.1292427
cg09864076	0.415904	0.1782832	0.970232	0.0423682
cg05945030	3.15E-05	3.19E-08	0.031102	0.0032142
cg20293352	1.4606152	0.6913791	3.085712	0.3208023
cg10064907	4.71E-25	1.32E-43	1.68E-06	0.0101668
cg01320433	0.992987	0.4766817	2.068515	0.985004
cg09799938	2.8983865	0.3390791	24.77488	0.3310258

cg15684772	1.9664921	0.5988373	6.457666	0.2649677
cg15800776	19.253932	0.2391033	1550.434	0.1865235
cg00762029	0.5425402	0.1100861	2.673815	0.4524036
cg16595458	0.5861676	0.2116512	1.62339	0.3040767
cg22216491	7.072E+14	952694.99	5.25E+23	0.0010343
cg03245159	4.4777499	0.8915023	22.4904	0.0686835
cg12020444	1.6341019	0.45906	5.816863	0.4483957
cg02443847	3.725153	0.9909324	14.00375	0.0515969
cg09831620	0.5491949	0.2505245	1.203934	0.1345192
cg06506470	4.3130275	2.1321593	8.724585	4.78E-05
cg00056541	4.3964502	0.892045	21.66794	0.0688217
cg08158778	5.7738235	1.4576808	22.86992	0.0125408
cg00808170	4.2902061	0.6748743	27.27303	0.1227669
cg17772171	0.0166749	0.0018535	0.150016	0.0002598
cg02289903	2.7057308	1.1677576	6.269263	0.0202491
cg20731469	3.1230221	1.3249778	7.36108	0.0092354
cg00499277	0.4858556	0.1424063	1.657621	0.2489789
cg21291449	0.1572858	0.0448713	0.551328	0.0038475
cg08915632	1.9249765	0.8567551	4.32508	0.1128196
cg02298949	0.0015886	1.16E-05	0.218353	0.0102957
cg16537483	0.4316047	0.1935796	0.962305	0.0399867
cg08249988	0.0667083	0.0060768	0.73229	0.0267699
cg03473046	3.2901175	0.1310101	82.62625	0.4689853
cg04035553	0.4393552	0.1061344	1.818761	0.2564984
cg15733882	1.5203555	0.7124267	3.244517	0.2787047
cg22166446	0.8346293	0.3918328	1.777815	0.6393888
cg07499032	2.0404625	0.7551623	5.513367	0.1596532
cg02558120	0.3232931	0.0864269	1.209327	0.0934264
cg26904049	0.3256378	0.1426255	0.743485	0.0077295
cg09593033	0.4453649	0.1943361	1.020654	0.0559213
cg19524362	0.3287594	0.0813577	1.328489	0.1184512
cg00077281	8871.238	4.84E-13	1.63E+20	0.6342205
cg26336059	0.0591387	0.0088938	0.393239	0.0034386
cg04023641	0.2297726	0.0801442	0.658756	0.0062061
cg11877061	1.3752233	0.3389427	5.57982	0.6556823
cg09985802	1.6347213	0.5693255	4.693824	0.3611138
cg24659501	10.078026	0.4599367	220.8273	0.1424154
cg15442434	0.3684452	0.137817	0.985015	0.0465848
cg05668996	4.3157547	1.5352848	12.13178	0.0055551
cg13457961	7.874E+12	19.588353	3.16E+24	0.0293923
cg15214448	1.4701855	0.6885006	3.139351	0.3194073
cg14126601	1.0030261	0.4037036	2.49208	0.9948081
cg23497611	1.6464071	0.381717	7.10122	0.50377
cg19143950	0.4920744	0.0431669	5.609322	0.5679161
cg03264693	1.727085	0.6611983	4.511237	0.2646538
cg21904074	20.272237	0.6622715	620.5365	0.0847263
cg03810301	3.356453	0.5896277	19.10659	0.1723701
cg25136124	0.3137651	0.1001472	0.983039	0.0466655
cg18769909	0.4958457	0.2405445	1.02211	0.0573402
cg23347835	0.527468	0.2235351	1.244648	0.1441988
cg26785896	3.7686809	1.0971678	12.94511	0.0350958
cg24719138	1.3391556	0.512861	3.496733	0.5509305
cg10147479	2.6974863	0.7020303	10.36484	0.1484998
cg01450870	0.7453692	0.3494972	1.589641	0.4469597
cg08061755	0.7169975	0.3383421	1.519425	0.3852737
cg21749951	1.7664233	0.6478029	4.816668	0.2662843
cg09106999	0.2196773	0.0486607	0.991728	0.0487507
cg12445208	2.2844123	0.703246	7.420646	0.1693481

cg05398095	0.3558066	0.0908359	1.393705	0.1379626
cg13208413	2.0057148	0.6267812	6.418335	0.2408816
cg08983133	160112386	206.40973	1.24E+14	0.0063285
cg12593303	2.1621713	0.9904731	4.719951	0.0528761
cg19056833	0.4574128	7.90E-05	2648.058	0.8595502
cg12440105	1.6004814	0.7080581	3.617698	0.258359
cg06568438	0.4106518	0.2171532	0.776571	0.0061846
cg16583193	3.9628264	3.88E-07	40444665	0.8671916
cg19357499	0.1459565	0.0160309	1.32889	0.0877009
cg21887193	0.3119956	0.1019432	0.954858	0.0412605
cg11739758	1.4546611	0.6526956	3.242	0.359377
cg18368599	0.3255714	0.1655684	0.640199	0.0011434
cg09819656	3.2838865	0.9456496	11.40371	0.061209
cg00417288	2.1931267	0.8627294	5.575102	0.0989881
cg13378687	0.8765846	0.4235802	1.814062	0.7226077
cg07281938	0.0094943	5.27E-07	170.9722	0.3515784
cg27370471	1.870288	0.7435512	4.70442	0.1834064
cg26821498	116279.86	0.510494	2.65E+10	0.0638627
cg10564075	67.176698	1.4140486	3191.339	0.0326923
cg00033877	3.8919323	1.068481	14.17633	0.0393608
cg07077277	2.8647841	0.980573	8.369584	0.0543421
cg00767000	0.4681475	0.2437744	0.899037	0.022629
cg20909795	0.9313426	0.4515519	1.920929	0.8472959
cg18077636	1.4879185	0.5354791	4.134431	0.4459989
cg02197387	0.2365702	0.0584964	0.956733	0.0431756
cg19125370	3.3839706	0.6967836	16.43445	0.1305605
cg18194738	0.004994	2.29E-05	1.08756	0.0536806
cg12273284	0.5398959	0.0952919	3.058892	0.4860981
cg10064248	199.75742	0.2641551	151059	0.1172717
cg00383136	0.4138695	0.1636016	1.046982	0.062461
cg04250991	0.0013769	2.21E-06	0.859209	0.0448378
cg07333922	0.7570002	0.3804591	1.506205	0.427722
cg25205727	15.384032	0.8689381	272.3651	0.0623002
cg00733567	0.2627802	0.0830179	0.831789	0.0230116
cg12135769	0.1874053	0.0714305	0.491678	0.0006676
cg01589157	1.4678719	0.0063361	340.0587	0.890123
cg11774346	0.9206817	0.175553	4.828485	0.9221383
cg02487453	0.0967057	0.010598	0.882434	0.0383743
cg13779610	0.8264951	0.4527965	1.508612	0.5348122
cg23213217	3.3083448	0.6152043	17.79108	0.1633284
cg18729350	4.3577641	1.5965832	11.89422	0.004063
cg19433037	1616.5518	3.0776282	849108.4	0.0207936
cg22993706	1.6848964	0.5379775	5.276941	0.3704355
cg18301703	0.1953869	0.0376388	1.014273	0.0520043
cg15517965	2.38E-17	5.64E-36	100.3557	0.0802324
cg09554443	1.7113255	0.6314175	4.638191	0.2909064
cg02796405	11118849	1.25E-06	9.87E+19	0.2861746
cg26052200	1.0455268	0.5410624	2.020333	0.8946176
cg27281030	1.3997758	0.6961187	2.81471	0.3453664
cg17470674	1.5390702	0.7723664	3.066856	0.2203088
cg11800192	8.0487632	0.2839271	228.1663	0.2216519
cg27229100	0.814583	0.4179229	1.587722	0.5469897
cg04254167	0.3414291	0.1624037	0.717803	0.0045894
cg19403377	0.367057	0.1075294	1.252967	0.1096082
cg13375571	0.3120199	0.1554459	0.626304	0.0010522
cg15945287	0.0725774	0.0120735	0.436283	0.0041524
cg01022974	0.5447041	0.3161517	0.938482	0.028618
cg16009877	0.43265	0.2012862	0.929949	0.0318743

cg08722675	0.2457994	0.1053016	0.573755	0.0011767
cg01875459	1.5026929	0.7330284	3.080489	0.2661466
cg00391031	2.8744261	0.5989274	13.7952	0.187036
cg13688329	0.5368003	0.0636563	4.526726	0.567394
cg23048215	2.1735503	0.7084466	6.668563	0.1746716
cg04889069	1.7555914	0.7771141	3.966086	0.1758913
cg01172419	0.1290374	0.0294612	0.565171	0.0065844
cg24709285	1.3812245	0.2241686	8.510476	0.7277442
cg14600484	0.2878702	0.0698803	1.185874	0.0847174
cg23501005	0.409416	0.1417941	1.182147	0.0988068
cg22396280	1.2477382	0.5702376	2.730179	0.5795767
cg16974832	1.5794428	0.768972	3.244122	0.2132708
cg12394800	5.8297826	1.5167144	22.40789	0.0102784
cg16248187	1.8427756	0.3085139	11.00703	0.502641
cg23015341	2.8893972	1.1619505	7.185001	0.0224354
cg08578323	0.2428884	0.0049242	11.98068	0.4767898
cg25974380	1.3823479	0.5412913	3.530236	0.4984997
cg07296447	1.5123144	0.8140665	2.809469	0.1905415
cg24219974	2.2448107	0.5081564	9.916584	0.2860488
cg01599633	0.561079	0.2688501	1.170949	0.1236729
cg19913537	0.6444249	0.0050302	82.55735	0.8591457
cg10668614	2.2422337	0.4337495	11.59105	0.3353524
cg25119261	0.3914767	0.1437074	1.06643	0.0666281
cg01894846	0.3662798	0.1489482	0.900722	0.0286903
cg09684245	0.8589605	0.4272106	1.727048	0.6696488
cg10682299	1.5162742	0.8157734	2.818292	0.1881243
cg11527279	0.2009107	0.0286492	1.408942	0.1063167
cg25873462	2.4384017	0.7821881	7.6015	0.1244169
cg24216566	0.4056202	0.1773827	0.92753	0.0324978
cg10425754	1.6980146	0.7667471	3.760371	0.1918195
cg06642626	0.5339986	0.2059396	1.384651	0.1968752
cg17813891	2.3998523	1.0826917	5.319419	0.0311147
cg22732770	0.505252	0.168896	1.51146	0.2220434
cg23288563	2.0954057	0.3978232	11.03687	0.3828622
cg06813419	1.2179236	0.1970865	7.526328	0.8319808
cg07287698	0.4685128	0.1849409	1.186889	0.1098889
cg21146833	0.5686731	0.1927386	1.677864	0.3065506
cg19530886	2.5697706	1.0222284	6.460122	0.0447808
cg13909582	1.6279095	0.5965211	4.442575	0.3414327
cg11033273	0.0446187	0.0050962	0.39065	0.0049686
cg05204171	0.0043358	0.0002637	0.071295	0.0001397
cg00542775	1.4028811	0.6244799	3.151543	0.41234
cg13948082	0.6566299	0.3277688	1.315448	0.2354053
cg11776534	1.9107025	0.5459818	6.68664	0.311024
cg24255032	0.3510908	0.1444142	0.85355	0.0209253
cg22970629	541397.78	4.3769338	6.7E+10	0.0273322
cg16492615	0.2893994	0.0594437	1.40893	0.1246771
cg04974899	0.0003875	7.17E-08	2.0941	0.0732269
cg24356321	140.01491	0.0616568	317956.4	0.2100849
cg12180551	0.7446774	0.4056748	1.366968	0.3414633
cg03893150	1.6319132	0.7575066	3.515667	0.2110363
cg00210271	2.525884	0.9733783	6.554585	0.0568445
cg02751532	2.1111293	0.8535141	5.221785	0.1058428
cg04705429	12.245541	0.3807408	393.8461	0.1571659
cg16156617	1.7160862	0.7596691	3.876625	0.1939895
cg20709370	0.1534183	0.0494503	0.475976	0.001174
cg08706575	0.9901565	0.3215419	3.049089	0.9862466
cg21654383	1.9987271	0.7867383	5.077813	0.1454624

cg12475182	0.0102456	0.0001682	0.624097	0.0289023
cg10123421	0.3581322	0.1249417	1.026548	0.0559793
cg03450104	0.5649461	0.0019111	167.0053	0.8440414
cg13348293	0.0001658	1.48E-40	1.85E+32	0.8371454
cg08357850	0.8325376	0.3227346	2.147644	0.7046429
cg19954234	0.3746532	0.1502033	0.9345	0.0352713
cg27394566	2.4260926	0.162765	36.16211	0.5202557
cg11824827	20.559395	2.0434513	206.8504	0.0102683
cg21307484	1.1163646	0.4616151	2.699803	0.8069929
cg22792176	0.5567891	0.2564227	1.208996	0.1388174
cg10876928	0.451264	0.1738768	1.171169	0.1019962
cg20383624	3.7621082	0.0121283	1166.981	0.6508045
cg25596215	8.1302613	1.2905485	51.21942	0.0256425
cg03402235	0.4159834	0.1688369	1.024907	0.0565869
cg02086046	0.3040054	0.1256082	0.735774	0.008282
cg14191654	0.0985341	0.017677	0.549242	0.0082049
cg11708690	0.2107939	0.0941215	0.472093	0.000154
cg14189750	1.6318476	0.6223299	4.278963	0.3194126
cg00303606	1.2933757	0.4890125	3.420814	0.6041763
cg08496775	3.4667231	1.3191657	9.110432	0.0116736
cg16894306	6.3532734	0.2679568	150.6366	0.2523453
cg08355301	2.1529227	0.789533	5.870656	0.134069
cg08814947	0.2087152	0.0531373	0.819802	0.0247928
cg22794078	0.0078053	0.0001431	0.425723	0.017383
cg08759041	0.4927937	0.2176685	1.115668	0.089616
cg09710586	0.0219414	0.0007661	0.628373	0.0256539
cg04003582	0.116608	0.0332708	0.40869	0.0007841
cg13071569	0.6094728	0.2587584	1.435537	0.2572844
cg18385299	0.5508212	0.1686931	1.798556	0.3232829
cg05148180	0.7117638	0.3510767	1.443011	0.3457182
cg04440885	0.4390309	0.1826861	1.055078	0.0657512
cg02473540	0.2509604	0.0918288	0.685853	0.0070367
cg27476810	1744330.6	1.10E-13	2.77E+25	0.5240522
cg16327477	4.922307	0.8073518	30.01059	0.0839967
cg04885037	0.1690444	0.041628	0.68646	0.0129144
cg02956255	1.6339757	0.8373826	3.188359	0.1499742
cg04124740	0.4655778	0.2291145	0.946089	0.0345878
cg27496526	0.445139	0.189703	1.044521	0.0629039
cg25277573	0.1500571	0.0328587	0.685272	0.0143778
cg08900426	0.2785358	0.1213188	0.63949	0.0025759
cg06108383	0.2100006	0.0671203	0.657033	0.007325
cg22235407	0.3028979	0.1250855	0.733476	0.0081237
cg07149641	7.6916307	1.0250181	57.71721	0.0472565
cg02029841	1.136906	0.5072365	2.54823	0.755349
cg26254169	0.0345892	0.0008063	1.483802	0.0793956
cg05355757	2.9540432	1.3107768	6.657404	0.0089823
cg04071866	0.3515202	0.1313229	0.940936	0.03742
cg26633223	1.2089189	0.2756214	5.302509	0.801414
cg04404310	1.5451836	0.6002893	3.977403	0.3670369
cg00934037	1.7383147	0.4919961	6.141793	0.3905743
cg26828017	0.3956512	0.1842539	0.849588	0.0174061
cg24031355	1.1107307	0.5465097	2.257458	0.7716463
cg20294319	1.1872979	0.6752458	2.087649	0.5510224
cg07756434	0.2687712	0.12874	0.561115	0.0004677
cg15171226	0.1076982	0.0190059	0.61028	0.0118034
cg05967403	0.3223994	0.1135423	0.915442	0.0335126
cg21368161	1.2193404	0.5650589	2.631214	0.6133149
cg20478261	0.2356443	0.0843626	0.658209	0.005816

cg07148370	0.0715933	0.0138749	0.369416	0.001636
cg14485744	0.3753732	0.1759901	0.800642	0.0112365
cg00586420	2.006161	0.5763728	6.982775	0.2739165
cg11589932	1.8355828	0.6692263	5.034715	0.2380816
cg12577411	1.2306146	0.1073768	14.10372	0.8675575
cg27376924	1.3922459	0.7206366	2.689773	0.3246783
cg01889899	2.1235423	0.854613	5.276578	0.1048765
cg13729116	0.357477	0.1178166	1.08465	0.0692972
cg12415975	0.0218763	0.0005618	0.851903	0.0407811
cg08331313	0.2295346	0.0104657	5.03417	0.3502477
cg13496793	0.2670555	0.1407658	0.506647	5.32E-05
cg04520047	0.0212441	5.45E-06	82.85991	0.3612611
cg06434480	2.427E+13	9.77E-20	6.03E+45	0.4180417
cg07050504	1.1991327	0.540873	2.658516	0.6548398
cg01363734	0.3339684	0.1392911	0.800732	0.0139697
cg17738841	0.4248124	0.1428163	1.26362	0.1237384
cg09741070	0.6838609	0.108141	4.324591	0.6863391
cg20333014	0.8161483	0.3165478	2.104258	0.674182
cg21041701	0.656195	0.3208	1.342244	0.2485697
cg01085765	2.4082705	0.184838	31.37756	0.5022083
cg13207250	2.5355358	0.3716213	17.29971	0.3423003
cg08582840	0.5450955	0.2397046	1.239564	0.1477243
cg13492692	2.3365091	0.9747576	5.600648	0.0570868
cg02593082	3.35E-05	6.14E-13	1829.511	0.2569842
cg23273257	0.0004237	3.31E-07	0.542762	0.0333911
cg16272420	2.6835477	0.9541165	7.547745	0.0613531
cg25127444	1.0343138	0.4345901	2.461642	0.939211
cg02592271	0.4074021	0.1833577	0.905206	0.0274918
cg12748890	0.1550361	0.0607571	0.395612	9.61E-05
cg12065138	1.4214821	0.6886791	2.934039	0.3415001
cg20267775	0.122676	0.0389731	0.386148	0.0003353
cg02191336	0.4056083	0.0168248	9.778329	0.5784004
cg14641625	0.6906723	0.3350404	1.423793	0.316009
cg04557677	1.8203929	0.8045243	4.118993	0.1504644
cg05167973	0.4600042	0.1484355	1.425561	0.1784423
cg18613324	0.5332739	0.271805	1.046269	0.0674857
cg17910478	0.3188325	0.1496379	0.679334	0.0030589
cg06200092	1.3156634	0.5874024	2.946822	0.5048997
cg06769296	0.4272848	0.1872155	0.975199	0.0434235
cg06244417	0.322937	0.1194131	0.87334	0.0259626
cg17177006	1.4081394	0.6002158	3.303573	0.4314654
cg26605774	0.2558754	0.1232306	0.531298	0.0002557
cg25446191	0.1317165	0.039697	0.437042	0.0009243
cg25736517	2.6040959	1.0290712	6.589744	0.0433357
cg12004671	12.934267	1.3868298	120.6314	0.024639
cg05262316	103.19931	0.6460929	16483.85	0.0732585
cg09315367	0.252642	0.0838854	0.760895	0.0144555
cg09298147	3.8856189	0.7532009	20.04516	0.1049339
cg15117891	0.6373082	0.2394929	1.695924	0.3669738
cg16855352	2.9928819	0.7730468	11.58706	0.1124561
cg23557906	0.5670406	0.2562424	1.254808	0.1615489
cg03510310	1.8334121	0.2309756	14.55305	0.5663018
cg03035538	0.6939369	0.3583302	1.343868	0.2785817
cg05567511	1.554936	0.6348142	3.808714	0.3341574
cg22471112	0.2382995	0.0786076	0.722407	0.0112573
cg10366062	0.5848149	0.3028796	1.129189	0.1100355
cg05771369	4.8684415	1.2161691	19.48884	0.0253189
cg08575894	0.5148957	0.2411784	1.099259	0.0862719

cg04762815	2.787695	0.1041496	74.61614	0.5410101
cg04288108	2.1415503	0.8621732	5.319393	0.100902
cg14653877	5.7702583	1.0860706	30.6572	0.0396999
cg05708536	1.9155146	0.6746443	5.438712	0.2221702
cg05727199	0.3469588	0.1638471	0.734712	0.0056873
cg08713365	0.0004768	1.71E-06	0.132871	0.0077538
cg09773238	0.5440275	0.2344146	1.262574	0.1564292
cg06021750	0.7816307	0.3016007	2.02568	0.6120982
cg04773333	3.4245469	0.9414001	12.45753	0.0617187
cg27316939	2.9185333	0.5810525	14.65932	0.1933703
cg19592277	4.2187526	0.165605	107.4718	0.383515
cg07599225	1.3320463	0.6027354	2.943825	0.4785425
cg08887114	0.333693	0.1623978	0.685668	0.0028177
cg06087349	2.2871255	0.5285953	9.895932	0.2683218
cg13088568	2.4659091	0.5741167	10.59141	0.2248521
cg02187822	1.0167141	0.3590135	2.8793	0.9751024
cg23459424	1.1036269	0.2755878	4.419616	0.889222
cg13677149	1.8046242	0.805958	4.040742	0.1511633
cg10126923	2.6993694	1.0041959	7.256149	0.0490378
cg11788465	1.0464497	0.4812652	2.275371	0.9087884
cg13328400	2.1344605	0.758229	6.008636	0.1510476
cg03456560	1.6759735	0.7868416	3.569826	0.1807146
cg25652742	0.4686154	0.1825014	1.203281	0.1151743
cg16817180	0.066803	0.0132176	0.33763	0.0010624
cg05955412	52.364631	0.2178537	12586.68	0.1570294
cg24272907	0.5153365	0.2595242	1.023302	0.0582054
cg12284171	3.61E-08	4.23E-14	0.03085	0.0139256
cg05004518	0.4718864	0.2272443	0.9799	0.0439649
cg26839356	0.222385	0.0653842	0.756376	0.0160833
cg22446709	1.0774716	0.5294578	2.192706	0.8369227
cg15673547	0.1034815	0.0277948	0.385267	0.0007194
cg07960450	0.0377877	0.0018157	0.786425	0.034422
cg19649083	0.0253902	0.0013169	0.489513	0.0149694
cg02666265	0.3882168	0.1293463	1.165184	0.0915387
cg01842890	0.0938531	0.008929	0.986497	0.0486906
cg16389209	0.1943433	0.036176	1.044043	0.0561696
cg06538549	1.263803	0.5122402	3.118064	0.6113685
cg16693212	2943.7795	30.707745	282203.7	0.0006015
cg25565082	1.9789615	0.5940693	6.59231	0.266241
cg13572782	0.4444271	0.1992682	0.991204	0.0475294
cg26289436	0.8540303	0.2439702	2.989577	0.8050388
cg11853817	7.55E-09	5.54E-16	0.102944	0.0256665
cg19034132	2.2574316	0.9615594	5.299722	0.0614921
cg12136632	0.5058367	0.2504225	1.021756	0.057438
cg16871520	0.2628598	0.1077704	0.641134	0.0033129
cg18080509	0.2807575	0.1063561	0.74114	0.0103228
cg16025451	1.6818601	0.8783942	3.220255	0.1167103
cg14091208	3.4212347	1.0404934	11.24932	0.0428339
cg07469647	2.9629115	0.4789688	18.32864	0.2427135
cg26342147	2.2825782	1.0460169	4.980955	0.0381754
cg23401912	0.8766925	0.4179013	1.839166	0.7277467
cg06892635	0.0206493	0.0001343	3.175228	0.130979
cg09644662	0.2374934	0.0544646	1.035592	0.0556948
cg01360605	0.0477135	0.0037013	0.61508	0.0196714
cg08996483	0.3142724	0.124935	0.790548	0.0139197
cg07820280	2.142185	0.7092292	6.470344	0.176767
cg19951443	7637374.8	273.72628	2.13E+11	0.0024092
cg05252264	0.5044478	0.2171938	1.171615	0.1114785

cg14005153	5.870922	1.2911579	26.6952	0.0219824
cg24890446	1.78E-07	5.24E-16	60.47157	0.1209833
cg20385901	1.1354656	0.4988543	2.584486	0.7620876
cg17901382	1.8132107	0.0735952	44.67319	0.715854
cg23886218	5.2094815	0.786442	34.5082	0.0870942
cg26062174	2.12E-16	9.86E-48	4.54E+15	0.3268327
cg24745816	0.8467317	0.1088006	6.589621	0.8737322
cg19689322	1.7492316	0.8643091	3.540181	0.1200512
cg14300812	1.528594	0.0250213	93.3845	0.8397252
cg13518968	2.0216664	0.1673778	24.41862	0.57974
cg10269431	0.2992847	0.1250056	0.716538	0.0067634
cg02798848	5.1726632	0.8756014	30.55779	0.0697735
cg01806956	2.7882484	0.7770867	10.00446	0.1157039
cg16536918	4.3116978	1.1407098	16.29752	0.0312387
cg04460668	511.09723	0.057791	4520088	0.1785979
cg12492496	0.3176332	0.1380051	0.731066	0.0070077
cg02747280	0.0400925	0.0038625	0.416155	0.0070532
cg19046189	2.1027546	0.0915924	48.2745	0.6420252
cg02740606	1.7382443	0.3571258	8.460585	0.4935136
cg11399254	7.1716291	1.3528849	38.01673	0.0206058
cg27117720	0.0004978	1.11E-09	222.3497	0.2518827
cg12917790	0.4318747	0.007865	23.71465	0.681205
cg13990351	0.9867026	0.3300018	2.950233	0.9808885
cg11363972	0.2447301	0.0895848	0.66856	0.0060474
cg25652454	0.2686689	0.1211351	0.595888	0.0012217
cg23518439	0.1550573	0.0345359	0.696168	0.0149901
cg17931409	0.4474596	0.1355298	1.477314	0.1869631
cg21182196	0.4355848	0.2132005	0.889933	0.0226161
cg15414042	83.764843	0.7099226	9883.541	0.0688794
cg22361106	0.9571785	0.3680037	2.489624	0.9284968
cg08815970	1.0738279	0.4670257	2.469043	0.8668377
cg04593538	0.2320943	0.0905683	0.594776	0.0023493
cg26184741	0.2737121	0.0755972	0.991019	0.0484152
cg08662757	0.200163	0.0205793	1.946865	0.165759
cg04593550	1.23E-10	1.37E-24	11136	0.1640375
cg04553559	0.0674584	0.0073974	0.615163	0.0168123
cg03578193	6.8954215	1.0300245	46.16088	0.0465403
cg16056164	0.6293506	0.339524	1.166581	0.1413894
cg15094605	1.4841018	0.6855187	3.21298	0.3164194
cg03360907	0.5833944	0.2322984	1.465137	0.2513797
cg05714563	0.0277002	1.64E-30	4.69E+26	0.9138827
cg25178784	1.13E-09	5.27E-19	2.430885	0.0602522
cg05114606	0.2361505	0.0800252	0.696869	0.0089463
cg05934660	0.1962422	0.038246	1.006929	0.0509752
cg24338833	2.0357835	0.9875869	4.196506	0.0540894
cg04671742	4.2419942	0.7019262	25.63591	0.115404
cg09676684	0.9890259	0.3959985	2.470141	0.9811487
cg22999989	0.1604316	0.0436462	0.589703	0.0058666
cg09767076	1.2036452	0.5924862	2.445224	0.6082642
cg07353895	3.1366523	0.0769041	127.9332	0.5457182
cg24197303	1.7348499	0.6509921	4.623258	0.2706266
cg08884752	0.6263022	0.1786138	2.196104	0.4647826
cg09890374	0.7881795	0.3246949	1.913263	0.598848
cg17018096	0.2684825	0.0757556	0.951518	0.0416554
cg03897241	1.415242	0.6009311	3.333011	0.4268044
cg09398082	5.0563665	1.8326584	13.95069	0.001749
cg23589174	2.1544011	0.7352126	6.313064	0.1617514
cg22134650	0.8366003	0.4540168	1.541573	0.5672537

cg20747380	1.0119141	0.3237379	3.162961	0.9837494
cg14028015	2.0174873	0.8794522	4.628171	0.097572
cg06769546	0.4429266	0.1425646	1.376105	0.1591367
cg12775181	1.1170222	0.452589	2.756891	0.810264
cg05148592	52.176513	0.03876	70237.01	0.2820291
cg19753675	3.2038542	1.1203252	9.162234	0.0298632
cg11684572	3.7390497	1.2932348	10.81048	0.014905
cg22828989	1.9947344	0.9400575	4.232683	0.0720305
cg11869510	0.2686151	0.0603274	1.19604	0.0845213
cg26724194	2.2663121	0.6598968	7.783293	0.193718
cg13639536	4.6064675	1.0994065	19.30091	0.0366526
cg25296374	0.3702369	0.0813432	1.685148	0.1987759
cg24283921	2.3570653	0.9013905	6.16354	0.0804154
cg13730341	0.4713838	0.2036883	1.090896	0.0789608
cg01800744	6.474761	0.2842897	147.4641	0.2414861
cg09730349	0.3499153	0.0760218	1.610601	0.1776301
cg00493554	2.6948048	0.668655	10.86057	0.1633203
cg14628274	0.2023403	0.0583977	0.701083	0.0117328
cg10204692	2.7899521	0.5653491	13.76819	0.2077627
cg20976787	0.359867	0.1480224	0.874896	0.0241441
cg16805189	0.4661279	0.2175654	0.998666	0.0496001
cg01021743	1.5146811	0.7603069	3.017543	0.23772
cg24350395	0.0754331	0.0140481	0.405049	0.0025797
cg14660328	0.5464298	0.2105824	1.417904	0.2141514
cg08407014	0.3967043	0.1964201	0.801213	0.0099396
cg13617837	0.4267853	0.1719934	1.059027	0.066316
cg25110932	1.3424367	0.3249485	5.545914	0.6840998
cg09304803	0.0929631	0.0086254	1.001946	0.0501877
cg07488381	0.4775566	0.2005829	1.136988	0.0949408
cg20157281	1.7745231	0.8795333	3.580231	0.1092613
cg08214847	1.0958338	0.4427484	2.712266	0.8431091
cg04353769	0.7937592	0.1652152	3.813534	0.7730162
cg19537243	0.6428308	0.2917991	1.416151	0.2728472
cg08747717	44.242813	0.0084174	232545.4	0.3859455
cg16313640	11.285374	0.4693215	271.3698	0.1352492
cg00961517	2.8461383	1.0643955	7.610426	0.0371307
cg24301620	3.1013341	1.0981746	8.758419	0.0326173
cg24524546	0.0699732	0.0122352	0.400176	0.0027957
cg10281846	0.5152133	0.2352328	1.128434	0.0973388
cg15126201	0.4219825	0.0326501	5.453866	0.5087465
cg07512676	11873.194	9.9091912	14226462	0.009484
cg02773192	1.0379193	0.2722481	3.956966	0.9565303
cg21836586	2.2407934	0.9547629	5.25906	0.0637947
cg02768721	1.7360975	0.7710102	3.909202	0.1828527
cg22086322	0.3677964	0.1647966	0.820856	0.0146099
cg25077733	0.9059051	0.3248888	2.525984	0.8501885
cg13876315	6.0387165	1.1728014	31.09316	0.031508
cg16365421	0.2863	0.1141246	0.71823	0.0076933
cg08980355	0.4329072	0.1868335	1.003078	0.0508439
cg22759800	2.5241337	0.9386277	6.787836	0.0665834
cg27510001	2.9204364	0.5723139	14.90257	0.1974525
cg07189204	0.0103886	0.0007845	0.137575	0.0005306
cg24375215	1.2130077	0.5066655	2.904061	0.6646297
cg14823201	2.9946518	0.6682255	13.42053	0.1517999
cg02019574	1.1484959	0.6057722	2.177457	0.6714192
cg23021638	0.3185431	0.1235166	0.821507	0.0179462
cg11526347	0.9813572	0.4551808	2.115779	0.9617074
cg01692088	0.1134676	0.0311847	0.412859	0.0009586

cg00579807	0.3526618	0.0954734	1.30267	0.1179713
cg07616374	1.880877	0.7869563	4.495419	0.1553043
cg09047881	0.1461038	9.15E-12	2.33E+09	0.8725153
cg14411375	0.3181819	0.1395871	0.72528	0.0064492
cg07382842	0.9653406	0.3359481	2.773888	0.9477763
cg14002425	0.0669076	0.0049847	0.898083	0.0412418
cg04603812	0.3308551	0.1202182	0.910553	0.0322439
cg22583148	2.4842521	0.767582	8.040194	0.1288752
cg19217535	0.3022816	0.0591813	1.54397	0.1504566
cg16713321	0.2990433	0.1276937	0.700324	0.005429
cg16882373	0.7059795	0.2811565	1.772703	0.4585756
cg15940508	0.0065365	0.0001331	0.320952	0.0113415
cg11109374	5.4410209	1.1242006	26.33401	0.0352499
cg02765403	0.1697122	0.0442124	0.651451	0.0097544
cg18614734	0.0288591	0.0003	2.77655	0.1280944
cg26743024	0.5711698	0.1993737	1.636299	0.2969697
cg07734359	2160.481	2.30E-17	2.03E+23	0.743509
cg11173822	0.4056614	0.1875455	0.877447	0.0218997
cg16258854	0.4699279	0.2117299	1.04299	0.0633841
cg05992395	0.6576393	0.359589	1.202733	0.1736239
cg11001780	0.0159209	0.0016679	0.151974	0.0003222
cg10378459	0.0353922	0.0010694	1.171321	0.0612902
cg16530959	3.9267438	0.1833799	84.08401	0.3815998
cg18021942	0.1500471	0.0186643	1.206269	0.0744851
cg17274215	1.5754656	0.2983934	8.318185	0.5923505
cg09119349	2.6175902	0.7013713	9.769117	0.1521265
cg03315869	0.1470873	0.0211645	1.022214	0.0526535
cg11340537	1.6104016	0.4990145	5.19703	0.4253903
cg13981425	1.0496178	0.3121078	3.529863	0.9376231
cg07542476	0.3120519	0.1205713	0.807625	0.01638
cg01476062	6.96E-05	2.62E-10	18.52849	0.1331187
cg14394939	0.160604	0.0468488	0.550572	0.0036214
cg21176130	0.9737636	0.3707109	2.55783	0.9569692
cg23798668	0.4818164	0.1935678	1.199306	0.1165652
cg15451100	4.9350926	4.61E-05	527838.8	0.7870152
cg08339023	1.2138193	0.4996083	2.949024	0.6687749
cg20316440	0.8894086	0.3627158	2.180902	0.7978743
cg07870914	3.2813777	0.9756155	11.03656	0.0548486
cg15150621	0.5595049	0.2768145	1.130886	0.1057965
cg07893738	0.4208752	0.1907738	0.928513	0.0320578
cg14369273	0.0230591	0.0001267	4.195493	0.1556522
cg26688911	0.2973757	0.1147701	0.770517	0.0125377
cg06951265	0.6038449	0.2241912	1.626418	0.3183558
cg23618218	0.3235123	0.1409618	0.742472	0.0077566
cg11067179	1.4360587	0.4123797	5.000888	0.5697005
cg10319870	0.5494037	0.2096977	1.439426	0.2229367
cg04346701	8.6656642	1.5318912	49.02028	0.0145915
cg15405515	1.6930508	0.779953	3.67512	0.1830246
cg01692757	116.34155	3.0830854	4390.198	0.0102348
cg02590088	0.3034784	0.1201535	0.766512	0.0116541
cg05929767	0.5357977	0.1971416	1.456208	0.2212477
cg22060735	0.930051	0.4719673	1.832743	0.8340368
cg13941555	1.5971522	0.5343973	4.773406	0.4019156
cg07881405	1.2975422	0.680151	2.475356	0.4293065
cg09797202	0.3205268	0.1226726	0.837493	0.0202403
cg25628898	8.6623704	0.3831568	195.838	0.1747805
cg07999732	0.302398	0.0917768	0.99638	0.0493072
cg02981003	1.2729296	0.5656697	2.86448	0.5597862

cg26951340	0.4756303	0.2588385	0.873997	0.0166747
cg27039312	0.2471313	0.0830426	0.735452	0.0119985
cg22289831	1.6425032	0.8727485	3.091173	0.1240278
cg22590032	2.5581035	0.8734164	7.492295	0.0866915
cg11030811	4.7394264	1.4952806	15.02204	0.0082055
cg01691710	2.4726002	0.7755765	7.882848	0.1259343
cg01595997	0.6902757	0.0788146	6.045586	0.7377847
cg09948076	0.1264466	0.0189405	0.844158	0.0327722
cg23627149	0.3358898	0.1537491	0.733806	0.0062145
cg21003380	0.1408252	0.0393617	0.503833	0.0025785
cg13655082	0.2505966	0.1025158	0.612575	0.0024085
cg26557834	0.1644842	0.0569503	0.475064	0.0008518
cg19790294	0.4241261	0.1477961	1.217102	0.1107834
cg24476497	0.2551953	0.0629047	1.035291	0.0559502
cg01461199	1.653033	0.9011666	3.032201	0.1044259
cg07135540	3.4816658	1.0574939	11.46295	0.0401782
cg25395653	0.2082744	0.0021709	19.98169	0.5004449
cg21964649	1.6176365	0.6629194	3.947309	0.2906341
cg23088510	0.3989348	0.2032654	0.782961	0.007559
cg26717009	1.3776633	0.635545	2.986345	0.4169862
cg17568996	0.9512707	0.438067	2.065702	0.8995181
cg14557487	3.0543296	1.1381608	8.196495	0.0266291
cg00682367	0.1799273	0.028705	1.127813	0.067022
cg03916421	0.295018	0.1046273	0.831863	0.0209981
cg09701102	0.2695248	0.093801	0.774444	0.0149077
cg17065850	0.4098374	0.1891263	0.888119	0.0237798
cg24336808	1.3058337	0.6394912	2.666497	0.4638202
cg10037068	4.2773621	0.9167847	19.95651	0.0643995
cg00483217	0.2726503	0.0779505	0.953659	0.0419277
cg09554898	2.9335659	1.2349206	6.968715	0.0147705
cg23013853	0.4653462	0.2024782	1.069483	0.0715856
cg01465177	0.3168051	0.0847744	1.183912	0.0874574
cg10360121	0.0312913	8.17E-43	1.20E+39	0.9420743
cg20311863	0.4850944	0.1521122	1.546994	0.221487
cg18300285	19.370808	0.500613	749.5375	0.1120612
cg01766941	7.2604279	0.8801622	59.89102	0.0655635
cg04396251	1.6748712	0.7871663	3.56366	0.1806526
cg24362661	0.0937362	0.0134914	0.651266	0.0166856
cg04349839	0.7495342	0.3241175	1.733327	0.5002971
cg03573679	3.1106884	1.0189133	9.496767	0.046276
cg08944723	0.6871702	0.3638407	1.297828	0.2475105
cg25009842	0.6201648	0.2754367	1.396344	0.2486044
cg05923681	0.3351043	0.1516326	0.740572	0.0068866
cg09076123	2.6587148	0.8324728	8.491286	0.0988442
cg00049502	3.77E-06	6.85E-15	2069.819	0.2238477
cg03318695	0.8272037	0.3294239	2.07716	0.6863338
cg13163269	19068.973	2.377948	1.53E+08	0.0316482
cg25007683	1.0860933	0.4940363	2.387676	0.8371929
cg01733928	0.3062068	0.0763702	1.227738	0.0948437
cg14152912	1.5947232	0.7561749	3.363167	0.2202514
cg22136038	0.2115275	0.0741808	0.603173	0.0036657
cg24286765	2.0044261	0.948469	4.23601	0.0685485
cg16982073	0.4828576	0.2238812	1.041407	0.063382
cg00653615	1.0555357	0.4131477	2.696749	0.9100819
cg24728542	0.1364031	0.0332225	0.560037	0.0057013
cg00397849	2.3583512	0.7219753	7.703616	0.1554396
cg15575339	12903914	0.0004718	3.53E+17	0.1817684
cg23403750	7.1294861	0.2011702	252.6695	0.2805708

cg12544243	0.2381187	0.0958032	0.591844	0.0020078
cg00350931	2.2541622	0.7124861	7.131714	0.1666345
cg09915889	0.0038705	0.0003078	0.048666	1.71E-05
cg02124724	0.1174843	0.0422057	0.327031	4.14E-05
cg00446472	1.8605369	0.7327289	4.724254	0.1915948
cg17732038	2.6168402	1.0190902	6.719574	0.0455796
cg26876101	0.1819367	0.0483862	0.684099	0.011676
cg10582556	0.2514153	0.1013334	0.623779	0.002902
cg09437909	0.2752306	0.0102237	7.409442	0.4425428
cg16046740	8.92E-07	6.83E-25	1.17E+12	0.5127943
cg19527799	0.8453006	0.4068751	1.756149	0.652352
cg05280133	0.4769056	0.2391509	0.951027	0.0355053
cg19921353	0.9073643	0.2624731	3.136741	0.8779215
cg00837434	939.7832	9.2878896	95090.76	0.0036598
cg13021301	0.0112816	0.0002019	0.630369	0.0289057
cg19451168	0.5470664	0.2419196	1.237112	0.1473759
cg02352281	1.6415792	0.7784275	3.461828	0.1929157
cg21341008	84.212182	1.3869509	5113.152	0.0343365
cg09929187	0.3908587	0.1693829	0.901924	0.0276712
cg06673826	3.67E+27	34216808	3.94E+47	0.0069929
cg04512603	2.5279507	0.8497469	7.520515	0.095463
cg03536664	0.5402917	0.237046	1.23147	0.1430214
cg11890453	0.4472958	0.2475184	0.808318	0.0077033
cg16933440	1.2321591	0.6250879	2.428804	0.5465447
cg15981475	9.07E-08	1.32E-15	6.241206	0.0782224
cg11015038	0.1095604	0.0155807	0.770405	0.0262781
cg18442362	1.2150932	0.6558701	2.251134	0.5357475
cg21858108	1.1108182	0.4817202	2.561481	0.8052599
cg23921459	0.3570265	0.032989	3.863953	0.3966652
cg08942800	0.2933055	0.1111876	0.77372	0.0131997
cg07814565	1.8965149	0.6754294	5.325159	0.2243601
cg06251764	2.508248	1.120153	5.616472	0.0253623
cg22221730	0.3518445	0.1457214	0.849529	0.0202033
cg14001871	4.2557886	0.989763	18.29906	0.0516383
cg22094633	1.1467393	0.085277	15.42047	0.9177524
cg20739354	0.0133314	0.0017837	0.099636	2.59E-05
cg13750219	2.6393797	1.0383034	6.709335	0.0414571
cg04606397	0.4022889	0.1714505	0.943925	0.0363864
cg21526019	0.1902773	0.0454312	0.796929	0.0231723
cg12186771	1.819238	0.9171326	3.608668	0.086818
cg25370941	3.83282	1.1908135	12.33653	0.0242735
cg06817669	0.2915351	0.1509999	0.562866	0.0002405
cg10103580	119146.67	1.85E-34	7.68E+43	0.7976769
cg07478122	0.5200373	0.1807122	1.496517	0.2253481
cg01891818	0.2267089	0.0327027	1.571643	0.1330208
cg09029959	0.7807099	0.334855	1.820214	0.5665292
cg22372968	0.5924482	0.2760178	1.271639	0.1791684
cg11062056	0.0175526	0.0016084	0.191549	0.0009156
cg22842048	3.9929284	1.0266774	15.5292	0.0457214
cg14870958	0.6815764	0.257707	1.802615	0.4398037
cg00497472	0.0544216	0.0024984	1.185454	0.0640627
cg18794809	0.5271628	0.2581802	1.076382	0.0787695
cg03142956	1.4187593	0.4851694	4.148814	0.5228902
cg16744314	1.8337754	0.7272656	4.623802	0.1987708
cg13427827	1.3550788	0.527524	3.480863	0.5278634
cg04206342	3.3054965	0.5756599	18.98049	0.1800183
cg27582173	0.5830478	8.51E-14	4E+12	0.9714612
cg04494750	0.1486619	0.042383	0.521444	0.0029114

cg07737503	1.3706459	0.6116469	3.071495	0.4437724
cg07845392	0.248197	0.0945379	0.651609	0.0046594
cg17435479	0.3934059	0.1631418	0.948673	0.0377748
cg16809542	5.1139849	0.8733772	29.9445	0.0703233
cg19276952	1.43E-11	5.91E-29	3442598	0.2213681
cg23314364	0.3042063	0.0973698	0.950413	0.0406124
cg12879013	0.2708371	0.0651902	1.125211	0.0722379
cg05768403	0.192384	0.0618691	0.598224	0.0044049
cg12517191	0.2075536	0.067846	0.634945	0.0058487
cg08032924	3.3695344	0.8858541	12.81674	0.0747245
cg06517940	13.093613	1.9077391	89.86695	0.0088654
cg24643429	3.5207133	0.0066064	1876.281	0.6943747
cg26922546	2.1298831	0.7294233	6.219162	0.1666976
cg16173067	0.3392096	0.008445	13.62504	0.5661179
cg12938068	2.0093623	1.0773459	3.74767	0.0282193
cg19472656	1.0532048	0.4955287	2.238499	0.8928066
cg26805905	4.1443884	0.8892606	19.31487	0.0702171
cg14851471	1.6734069	0.5778237	4.846272	0.342624
cg16923709	0.1499242	0.037017	0.607215	0.0078374
cg00934322	0.2568773	0.0782504	0.843266	0.025023
cg18032217	183.54897	0.4442199	75841.32	0.0898949
cg02193883	1.6398004	0.6947766	3.87023	0.2589807
cg04460847	4.091512	1.007478	16.61621	0.0487945
cg05905531	0.1295052	0.0313035	0.535773	0.0047828
cg18519988	0.0792449	0.0050441	1.244982	0.0712252
cg25678088	3.9700167	1.2452203	12.65722	0.0197696
cg04588079	5.41E-06	1.24E-11	2.355021	0.0671494
cg21610111	460148.14	0.9907082	2.14E+11	0.0501641
cg21500126	0.4623861	0.203029	1.053056	0.0662309
cg20464884	0.7299557	0.320331	1.66339	0.4538261
cg10133777	12.495826	0.1687846	925.1179	0.2501939
cg01300096	0.8416683	0.2131918	3.322856	0.8056643
cg03025880	3.4816504	0.8109453	14.94785	0.0933311
cg02497758	0.3918405	0.1550302	0.990381	0.0476592
cg27517345	0.3217168	0.1465584	0.706215	0.0046979
cg20607287	1.2714492	0.617297	2.618809	0.5147681
cg03459415	0.5157641	0.2326123	1.143588	0.1031617
cg01447495	0.4533554	0.19969	1.029251	0.0586186
cg06201393	1.2746559	0.6761199	2.403047	0.4531684
cg08170950	0.5732925	0.1932372	1.700833	0.3159921
cg17565113	1.9113673	0.8580981	4.257468	0.1128684
cg14535274	1.5373066	0.6349662	3.721949	0.3404813
cg20163288	0.8036928	0.2484346	2.599969	0.7152365
cg14213590	0.1996405	0.0762272	0.522862	0.0010381
cg17854066	1.3269098	0.547835	3.213905	0.5308699
cg08823110	2.8565782	1.2091086	6.748806	0.0167184
cg27424995	0.3307849	0.166856	0.655767	0.0015326
cg00814752	1.95885	0.8116343	4.727613	0.1347346
cg18189994	1.0202094	0.4462141	2.332574	0.9621783
cg11407681	0.7994181	0.2006711	3.184661	0.7509051
cg19167793	3.1950181	1.2463241	8.190599	0.0155884
cg15428620	0.5479353	0.1252793	2.39651	0.4242524
cg00155310	1.4827105	0.8392625	2.619479	0.1749482
cg11520030	0.2642528	0.0563199	1.239873	0.0915345
cg01137401	1.7195368	0.527189	5.608627	0.3688489
cg23631538	0.9097362	0.4496411	1.840624	0.7924671
cg11334733	0.7988644	0.4079497	1.56437	0.5125192
cg14373068	0.2765398	0.1048572	0.729318	0.0093792

cg22136363	1.4636044	0.6799774	3.150308	0.330129
cg13757640	2.7928965	1.2512557	6.233954	0.0121723
cg17122157	1.746384	0.6226764	4.897981	0.289312
cg21302989	2.2351427	0.7867389	6.35009	0.1311115
cg24943998	2.7062013	1.0329411	7.089974	0.0427732
cg24775300	12.965604	0.2265246	742.1133	0.214657
cg20702419	0.0414617	0.0048142	0.357083	0.0037635
cg02326931	435956636	5.59E-05	3.40E+21	0.1890261
cg04177132	0.0180458	0.0022976	0.141737	0.0001346
cg26777456	3.0600834	1.0585248	8.846377	0.0389254
cg19421125	2.4979279	0.8530681	7.314356	0.0949081
cg00541544	1.3313517	0.6549906	2.706142	0.4290657
cg11324467	1.7947781	0.4823524	6.678164	0.382971
cg08475996	0.5940301	0.3204465	1.101188	0.0981519
cg00109764	0.1062798	0.0307256	0.367621	0.0003995
cg19236431	1.7314544	0.668969	4.481425	0.2578836
cg23205454	1.7000666	0.6475273	4.463482	0.2812479
cg19746719	31.175459	0.7321367	1327.497	0.0723249
cg19003803	0.4464517	0.1138099	1.751334	0.2475199
cg19857761	0.1000696	0.0189628	0.528083	0.0066816
cg27263068	0.4407134	0.0013903	139.7028	0.7803528
cg02076869	1.8025498	0.9790763	3.318623	0.0584829
cg27499820	2.4922309	0.9224902	6.733096	0.071725
cg22242842	0.9511613	0.4445762	2.034989	0.8973305
cg09762242	3.3674215	0.9711357	11.67656	0.0556461
cg09406795	0.200438	0.0227515	1.765833	0.1476827
cg23658684	0.3654189	0.1494325	0.893587	0.0273438
cg23599820	0.5175616	0.2153884	1.24366	0.1408966
cg17358883	1.7310584	0.852843	3.513616	0.1286994
cg16121929	23858.9	2.83E-33	2.01E+41	0.8162631
cg15953892	1.3954862	0.4379119	4.446971	0.57306
cg23368159	1.2306124	0.138783	10.91205	0.8521581
cg05044185	0.0644953	0.0111449	0.373235	0.0022117
cg22845912	3.4016308	1.369629	8.448341	0.0083487
cg24326223	0.2938919	0.0038846	22.2348	0.5790498
cg23572751	0.0001504	9.90E-10	22.84614	0.14818
cg26976744	1.56E-16	3.08E-39	7921887	0.1724309
cg27520536	0.2729657	0.1274822	0.584476	0.0008304
cg13212803	1.4039957	0.6654484	2.962219	0.3730556
cg03293765	1.5151699	0.4492619	5.110026	0.5029021
cg04415014	0.1111511	0.0091756	1.346454	0.084308
cg12915406	0.0094461	7.12E-05	1.253174	0.0615576
cg11189107	1.6227875	0.6967233	3.779749	0.2617405
cg01710432	5.2350573	1.2818937	21.37917	0.0211167
cg11440344	0.4863536	0.1777047	1.331084	0.1605512
cg24212621	4.9538791	0.9380793	26.16082	0.0594733
cg18055585	0.557378	0.1764822	1.760349	0.3191664
cg23064797	8.93801	0.9055364	88.22177	0.06079
cg07779777	0.2740028	0.0363059	2.067918	0.2093265
cg23268102	1.423352	0.3525967	5.745746	0.6200182
cg08286551	0.3942233	0.151526	1.025646	0.0563839
cg18902057	805.2148	4.4780907	144787.3	0.0115397
cg07867367	0.355533	0.1293964	0.976872	0.0449267
cg17286695	7.3438298	0.9209916	58.55844	0.0597999
cg02683363	7.12E-06	5.35E-13	94.85958	0.1567557
cg09434175	16.2555	0.1315663	2008.426	0.2565234
cg17090968	2.1440496	1.0378075	4.429481	0.0393788
cg16654732	0.2705832	0.0494868	1.479492	0.1315372

cg25808094	1.2683911	0.2563757	6.275228	0.770711
cg14374866	0.9072992	0.4745734	1.734593	0.7685896
cg25558027	2.3468761	0.4692561	11.73736	0.2989358
cg07447769	0.6004819	0.2247718	1.604198	0.3090207
cg16447311	3.9510254	0.8681883	17.98066	0.0755449
cg23817981	0.9985554	0.5098599	1.955661	0.9966368
cg26296364	1.4817409	0.6569303	3.342144	0.3433836
cg17993313	0.3745217	0.13182	1.064076	0.065272
cg02579509	1.9331227	0.4721958	7.914013	0.3593753
cg07941983	1.1482601	0.5712159	2.308237	0.697968
cg11111859	0.0868714	0.0171735	0.439435	0.0031355
cg00381974	2.0749053	0.9232926	4.662912	0.0772645
cg11721464	2.6388866	0.9521722	7.313512	0.0620788
cg16565154	1.0132602	0.4193189	2.448485	0.9766548
cg24092960	0.1864364	0.0666866	0.521222	0.0013641
cg09577907	0.5344903	0.2799773	1.020368	0.0575848
cg08871244	1.966803	0.7349915	5.263073	0.1780201
cg19867914	2.2102993	0.5047397	9.679093	0.2925305
cg25374359	3.3588694	0.8629494	13.07377	0.0805708
cg19343611	0.2751537	0.0684086	1.106727	0.069192
cg03302546	2.2427112	0.4141371	12.14514	0.3486937
cg19416290	0.7812148	0.3665126	1.665145	0.5225487
cg04461228	2.2005332	0.9095284	5.324019	0.0801871
cg11339587	0.2318435	0.0793607	0.677305	0.0075333
cg02540307	1.1185842	0.0706292	17.71548	0.9366257
cg20541870	0.4329387	0.2024805	0.925698	0.0308436
cg04212699	0.0048499	1.36E-08	1727.175	0.4139054
cg04342425	2.3233876	0.6830755	7.902684	0.1771042
cg24844387	1.8499362	0.0677304	50.52772	0.7154537
cg18274320	16.666313	1.387703	200.1624	0.0265337
cg03997398	1.3540354	0.6332316	2.895326	0.434433
cg09009506	1.3100883	0.0079833	214.9896	0.9173367
cg01898433	3.0055345	0.8743343	10.33156	0.0806727
cg20942223	0.0765337	0.0011863	4.937712	0.2267236
cg19408149	0.0952904	0.0100968	0.899322	0.0401097
cg10802927	3.4368156	1.1368846	10.38953	0.0287235
cg05676251	0.5258252	0.2606084	1.060949	0.0726905
cg04664643	0.4191704	0.0670867	2.619056	0.3523389
cg22799420	3.7717922	1.5387273	9.245573	0.0037074
cg03533979	0.3456648	0.0218654	5.464526	0.4507237
cg04140212	0.1780565	0.0372574	0.850949	0.030603
cg07916323	3.43E-07	1.79E-13	0.655222	0.0436687
cg27637873	1.9846676	0.4463243	8.825209	0.3679365
cg16926213	0.2734527	0.1101814	0.678666	0.0051779
cg11342287	3.6304548	1.1649739	11.31373	0.0261979
cg21658267	5.5526084	0.7759137	39.73568	0.0877693
cg17934277	0.3109395	0.1214432	0.79612	0.0148797
cg18063312	2.5429255	1.0987232	5.885441	0.0292679
cg27198632	1.9442981	0.6257413	6.041307	0.2503601
cg11746952	0.65921	0.3200672	1.357708	0.2582983
cg20577477	0.5348943	0.2501506	1.143759	0.106621
cg02596779	0.0783099	0.0166495	0.368325	0.0012627
cg12550344	1.002537	0.5078263	1.979181	0.9941744
cg23013977	0.5750046	0.268853	1.229781	0.1536651
cg25024579	0.4553532	0.1862221	1.113437	0.0846307
cg25507226	0.34194	0.1583291	0.738481	0.0063014
cg18362448	0.3505775	0.1528724	0.803968	0.0133152
cg27066052	0.1533218	0.0313533	0.749764	0.0205804

cg26555136	1.5414702	0.5638418	4.214179	0.3990462
cg07159802	0.6388246	0.2886284	1.413918	0.2689425
cg14958041	8.6363906	0.935575	79.72342	0.0572708
cg01604412	1.5047064	0.4557353	4.968106	0.5025581
cg25204742	1.903411	7.07E-07	5125563	0.9320999
cg11240722	2.0187706	0.6695771	6.086581	0.2121762
cg17014647	0.1966329	0.0174631	2.214075	0.1879868
cg03927812	1.3814227	0.6894449	2.767921	0.362172
cg13148921	1.9103338	0.5398087	6.760497	0.3154671
cg09980114	11.986074	0.020643	6959.565	0.4443188
cg17436460	0.5327691	0.2445431	1.160707	0.1129981
cg25247402	1.0079549	0.2683185	3.786444	0.9906381
cg21614201	0.3852506	0.1724711	0.860538	0.0200046
cg24724630	0.328008	0.138659	0.775927	0.011166
cg21368354	1.41E-09	7.22E-21	274.8195	0.1244033
cg11087503	0.3393237	0.1625429	0.70837	0.0040005
cg12209313	4.8619921	0.3342266	70.72737	0.2469905
cg18875357	3.63767	0.6555415	20.18582	0.1396843
cg08358803	0.2940441	0.1419745	0.608996	0.0009841
cg04521004	3.8291801	1.1448891	12.80702	0.0292857
cg27030482	1.4357206	0.7211999	2.858145	0.3032194
cg14694038	0.2264301	0.0690033	0.743017	0.0142897
cg08781257	0.6657828	0.3484397	1.272148	0.2181918
cg08834383	3.53E-15	7.39E-25	1.69E-05	0.0034302
cg17339440	0.6420911	0.2002948	2.058371	0.456047
cg01658277	1.8565716	0.8757634	3.935833	0.1065428
cg16931969	1.3655592	0.2410893	7.734693	0.7247376
cg01148731	2.4704324	0.9101711	6.705373	0.0758628
cg12198334	1.3809245	0.418904	4.552242	0.5958996
cg18444689	0.4493637	0.2194955	0.919963	0.0286578
cg14116632	0.2183677	0.0778079	0.612848	0.0038532
cg17520215	0.2640248	0.1128474	0.617729	0.0021356
cg02091526	1.9939952	0.9841782	4.039936	0.055404
cg26403416	0.3159968	0.1008739	0.989889	0.0479956
cg05396156	0.3946916	0.1878755	0.829174	0.0141058
cg24037629	0.4109879	4.93E-17	3.42E+15	0.962082
cg18004175	0.0023994	5.26E-12	1094591	0.5531792
cg04062218	0.0838207	0.0172492	0.407318	0.0021158
cg15371806	0.3783871	0.1567053	0.913669	0.0307185
cg16269733	2.6503626	0.7977301	8.805512	0.1115935
cg17031543	1.4263531	0.6511853	3.124277	0.3747058
cg23677657	4.2258862	1.2372358	14.43388	0.0214688
cg17388024	1.07289	0.534389	2.154035	0.8431668
cg23756474	1.8296958	0.9296187	3.601247	0.0803394
cg01739831	287.58035	6.0370608	13699.13	0.0040784
cg15734516	54656034	2.15E-05	1.39E+20	0.2215119
cg07703530	0.5629355	0.2953093	1.0731	0.0808761
cg16590552	0.1502284	4.79E-07	47087.86	0.7690824
cg10186768	6.7888246	0.8676402	53.11895	0.0680463
cg25478299	4.915758	0.5894244	40.99708	0.1411554
cg18978531	0.0771843	0.0140821	0.423048	0.0031671
cg06853489	0.5585882	0.2488865	1.253667	0.1579908
cg18098567	2.5216945	1.1071263	5.743647	0.0276459
cg19434066	0.0089131	4.58E-18	1.73E+13	0.7927052
cg07902983	1.8337081	0.9639244	3.488329	0.064605
cg02578327	0.2720311	0.0891469	0.830101	0.0221901
cg22381454	1.3411731	0.7015506	2.563957	0.3746181
cg18845377	1.6613907	0.9073129	3.042191	0.1000082

cg19068510	1.3675537	0.6671299	2.803357	0.3927047
cg11969493	2.3861922	1.0436008	5.456026	0.0392931
cg02505588	4.2139928	0.061206	290.1304	0.5052942
cg10699171	1.7184074	0.7762106	3.804282	0.1818118
cg09226888	0.3382452	0.0751263	1.5229	0.1579355
cg00279406	0.5084673	0.2249898	1.149114	0.1039814
cg18818531	1.2206951	0.479083	3.11031	0.6760247
cg14654926	2.110322	0.9984626	4.460316	0.0504729
cg02068160	0.0789056	0.0085283	0.730052	0.0252771
cg03317505	0.0044311	1.68E-05	1.166676	0.0566825
cg23013137	3.3017266	0.707473	15.40893	0.1285912
cg02558625	1.1228319	0.5621802	2.242611	0.7427329
cg05394500	0.3532688	0.0309783	4.028592	0.4020886
cg08567760	0.2609556	0.0952276	0.715105	0.0090035
cg12363722	0.0967101	0.0235538	0.397083	0.0011885
cg07993507	0.9766381	0.4575213	2.08476	0.9512795
cg07536910	2.0189119	0.8097074	5.033924	0.1317734
cg16121641	0.8108226	0.4128319	1.592496	0.5425875
cg22251148	14.409301	0.2425243	856.1119	0.2004813
cg26719062	4.6617991	1.2938122	16.79716	0.0185806
cg10907257	0.6270843	0.3398937	1.156934	0.1353176
cg21199406	1.4769682	0.7482531	2.91537	0.2609861
cg09922452	0.7551681	0.2719464	2.097027	0.589963
cg14259755	0.3927577	0.1998598	0.771834	0.0067015
cg04574346	0.0943579	0.020227	0.440175	0.0026622
cg07516307	0.2620512	0.1131164	0.607081	0.0017821
cg08748615	0.1458485	0.0358716	0.592997	0.0071414
cg24689583	1.4886974	0.607097	3.65052	0.3845988
cg15063946	0.1479034	0.0314352	0.69589	0.0155706
cg27401846	0.2259538	0.07954	0.64188	0.0052344
cg03577433	1.6829606	0.8311519	3.407748	0.1481298
cg26253500	1.0021083	0.551706	1.820211	0.9944818
cg12536527	0.5378897	0.2644116	1.094223	0.0869988
cg11217934	3.0765326	0.6424685	14.73232	0.1596333
cg09662616	0.6843262	0.3416083	1.370875	0.2845864
cg03345454	1.5440697	0.7752367	3.075385	0.2165469
cg06534568	1.2174707	0.4776062	3.103466	0.6802248
cg18369972	0.4545955	0.0732038	2.823039	0.3974918
cg03697308	1.8508652	0.7431639	4.609619	0.1860427
cg01305539	0.9287686	0.1785472	4.831277	0.9300115
cg21533216	0.3495914	0.1642968	0.743862	0.0063715
cg12013713	0.6090713	0.1217082	3.04801	0.5461893
cg05036608	0.0016853	5.12E-12	554461.7	0.5233472
cg03669663	1.56E-05	8.78E-10	0.277897	0.0266631
cg05142677	0.4398647	0.1885685	1.026052	0.0573738
cg09587880	0.4080527	0.2091511	0.796108	0.0085724
cg16915733	1.0516193	0.3681241	3.004159	0.9251253
cg22437405	0.5654511	0.1984866	1.610865	0.2858027
cg23012192	0.4156508	0.187703	0.92042	0.030433
cg26718878	1.6441562	0.7875573	3.432448	0.1854931
cg04131583	1.3825412	0.6672887	2.864458	0.3834597
cg05760722	1.2861123	0.7286694	2.270008	0.385383
cg11285834	0.2665895	0.0850762	0.835368	0.0232899
cg17967319	1.16E-15	3.24E-26	4.14E-05	0.0055389
cg07297068	1.5367451	0.6698181	3.525712	0.3105312
cg03509039	0.5227381	0.2260884	1.20862	0.129297
cg14479136	0.4133314	0.1092891	1.56322	0.1930066
cg14054376	3.7387441	0.8159215	17.1318	0.0895042

cg12685200	0.4147948	5.08E-10	3.39E+08	0.9330187
cg15934804	0.0433523	2.31E-05	81.37043	0.4144532
cg17063371	0.67379	0.321062	1.414035	0.2965073
cg03156547	0.1917595	0.0572076	0.642776	0.0074481
cg04865008	1.5239765	0.2431221	9.552831	0.6527904
cg16553500	1.0132321	0.4369159	2.349741	0.975565
cg00071012	0.4030596	0.1555163	1.04463	0.0614695
cg19444609	1.7162895	0.8105675	3.634059	0.1581693
cg01985965	1.5635061	0.7082895	3.451345	0.2686168
cg07194846	0.9908386	0.2192573	4.477668	0.9904578
cg02436796	0.0038003	1.01E-05	1.432879	0.0656026
cg26864905	0.4946226	0.0614114	3.983812	0.5083791
cg00476944	0.3709368	0.0930093	1.479359	0.1599868
cg15246590	0.2877119	0.1063936	0.778037	0.0141103
cg05606039	8.40E-08	3.20E-16	22.06398	0.0995248
cg00394658	0.1619819	0.057535	0.456038	0.0005674
cg22047766	4.1049153	0.9990292	16.8667	0.0501577
cg03857640	0.2369967	0.0919018	0.611168	0.0028949
cg20191740	1.9026572	0.4553507	7.950146	0.3779494
cg04689178	0.6090569	0.2791284	1.32896	0.212926
cg25029529	0.0024326	9.00E-07	6.573016	0.1354612
cg05400196	0.3403576	0.1463613	0.791489	0.0123129
cg11937849	2.3261146	0.9460533	5.719349	0.0658934
cg23254031	0.0558112	0.000121	25.74841	0.3564999
cg07855465	0.4478591	0.241409	0.830863	0.0108461
cg15842722	2.9688527	1.0991944	8.018678	0.031831
cg18161025	0.3103921	0.1170869	0.822835	0.0186734
cg12840719	1.2459451	0.6756722	2.297533	0.4812514
cg25142170	2.235508	1.1424891	4.374218	0.0188283
cg27171580	0.4450869	0.1323004	1.497368	0.1909561
cg18009710	0.9559485	0.1914703	4.772738	0.9562077
cg14759482	0.3225423	0.1247111	0.834196	0.0196016
cg05948411	0.1383836	0.0367003	0.521795	0.0034943
cg18219160	0.2210304	0.0847278	0.576605	0.0020326
cg18123677	0.3382892	0.1390603	0.822949	0.016868
cg20799165	0.4721782	0.2317225	0.962152	0.0388095
cg22485289	2.495661	0.9936636	6.268041	0.0516025
cg09383860	2.5996885	0.5588695	12.09295	0.223178
cg10898212	0.2324692	0.0842308	0.641593	0.0048508
cg16004706	0.1233006	0.0046876	3.243264	0.2095928
cg14916175	1.5870123	0.4541605	5.545634	0.4693713
cg12242062	30.731967	1.3627506	693.0496	0.0311888
cg27595100	0.0003124	5.88E-06	0.016606	6.85E-05
cg24495643	0.2286295	0.0376911	1.38684	0.1086253
cg15644324	0.3144249	0.1327865	0.744526	0.0085201
cg14829814	2.5600297	1.0744477	6.099647	0.0338322
cg06389444	0.2550368	0.0588538	1.105174	0.0678055
cg12216448	1.5057289	0.1846804	12.27645	0.7022573
cg05886453	0.699944	0.1502219	3.261318	0.6495611
cg00260802	3.0563732	0.941229	9.924701	0.0630028
cg01486665	0.0290985	0.0014726	0.574983	0.0201522
cg04201526	0.8287495	0.4332405	1.585322	0.570312
cg19464016	0.7916204	0.3716558	1.686138	0.5447025
cg07512814	1.1931015	0.5639712	2.524049	0.644212
cg12915878	1.5629571	0.7412155	3.295715	0.2407039
cg13643138	1.6668748	0.7014104	3.961264	0.2473051
cg02130329	0.7512695	0.3913836	1.442078	0.3900037
cg20261917	1.1817954	0.0213195	65.50998	0.9350154

cg15613567	0.0066181	0.0002292	0.191063	0.0034484
cg25061610	0.1820474	0.0576644	0.574726	0.0036816
cg17173891	0.2997924	0.05958	1.508483	0.1439372
cg03598412	1.7168244	0.6055451	4.867492	0.3093837
cg00916640	0.3159019	0.1190104	0.838532	0.0206937
cg08220136	0.9965743	0.4688355	2.118356	0.9928835
cg11051429	0.5498759	0.2558087	1.181991	0.1255873
cg04882349	0.9496551	0.2427328	3.71538	0.9408363
cg06901238	3.967247	0.1130072	139.2747	0.4478248
cg24860819	7.1979487	1.1774218	44.00332	0.0326161
cg20513447	1.2603023	0.6453495	2.461243	0.4981078
cg08855742	2.6691932	1.0058198	7.083368	0.0486533
cg21549497	22.324377	0.9637032	517.1487	0.0527569
cg14604470	0.4069335	0.1605937	1.031142	0.0580502
cg12835684	0.0005322	2.03E-13	1396160	0.4957011
cg07669403	2.3920536	0.9160201	6.246501	0.0749363
cg18000029	2.7442547	0.9840063	7.653339	0.0537118
cg24682986	2.5049583	0.8732644	7.185471	0.0876524
cg06757399	0.1530708	0.0069854	3.354228	0.2334172
cg07556261	0.1409596	0.0357497	0.555799	0.0051252
cg22558061	3.0213973	1.3652901	6.686375	0.0063677
cg04620298	0.1379606	0.0369384	0.515267	0.0032169
cg03353885	4.5492332	0.9377258	22.06991	0.0600854
cg11644585	4.1492735	1.2170079	14.14656	0.0229782
cg27640302	0.3698878	0.1099967	1.243828	0.1079812
cg18087491	2.5066555	0.6911624	9.090949	0.1621078
cg24330598	0.8319246	0.4056618	1.706098	0.6155576
cg00816177	9.26E-05	9.19E-09	0.932554	0.0482895
cg07273304	5.6417061	0.3775528	84.30303	0.2098424
cg06803614	1.9506756	0.5689877	6.687553	0.2878159
cg09179646	3.1616834	1.2076378	8.277517	0.0190692
cg06544111	2.3714656	1.1615397	4.841719	0.0177322
cg24312283	1.8143708	0.9832768	3.34793	0.0566493
cg06913958	1.6662733	0.2632613	10.54643	0.5875796
cg07902673	0.5916688	0.264219	1.324931	0.2019843
cg17918280	2.0171784	0.7756063	5.24623	0.1501815
cg22707529	0.2757369	0.0695563	1.093083	0.0667564
cg04684114	2.0078116	0.8289111	4.863377	0.1225265
cg01705934	7.7818582	1.1674672	51.87068	0.0340108
cg06341195	0.2058711	0.0751718	0.563814	0.0021068
cg13089859	0.0018829	3.09E-07	11.4675	0.1581579
cg03716405	0.8645456	0.3160595	2.364868	0.7767961
cg08610862	1.9802995	0.9651253	4.063292	0.062439
cg08182826	0.217966	0.1016551	0.467356	9.06E-05
cg10664971	0.5063614	0.2277721	1.125695	0.0950201
cg26591391	2.3591638	1.1119543	5.00529	0.0253206
[truncated: 1,954,628 more chars]
